# Supplementary figures and images for: Delayed delivery of antibiotics by ultrasound-mediated rupture of polylactic acid pockets: In vitro and in vivo studies
Source: PLoS One. 2025 Dec 11;20(12):e0337717. doi: 10.1371/journal.pone.0337717 (PMC12698016; doi:10.1371/journal.pone.0337717)

A.

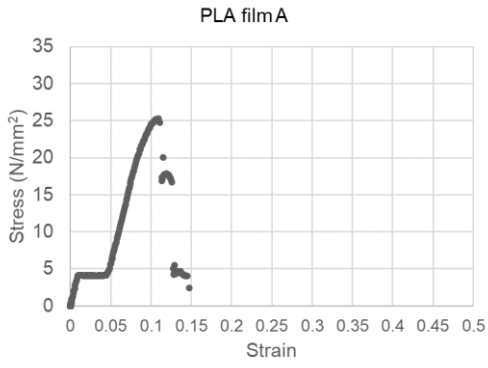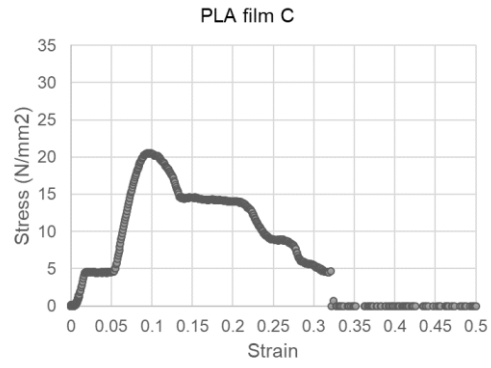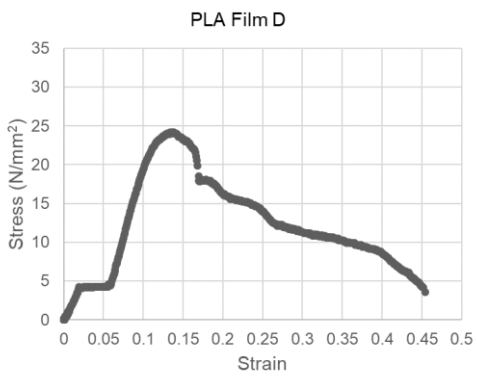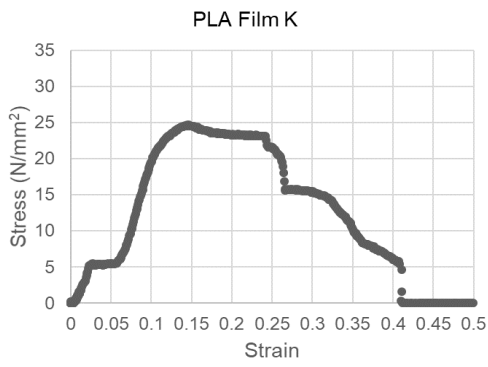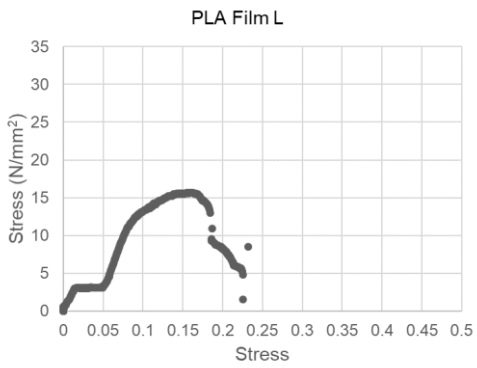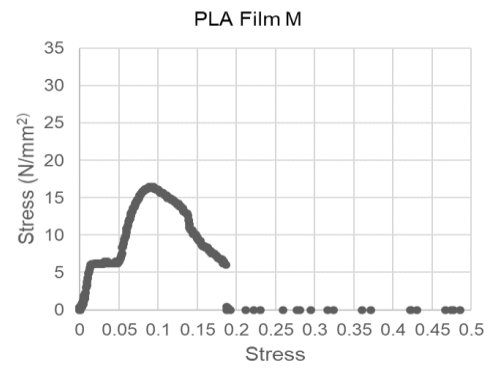

B.

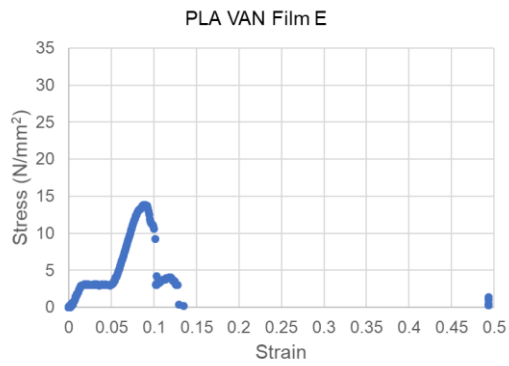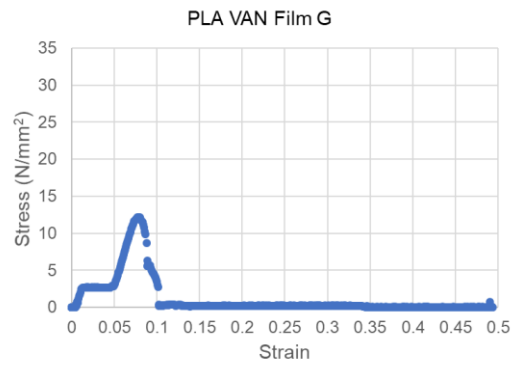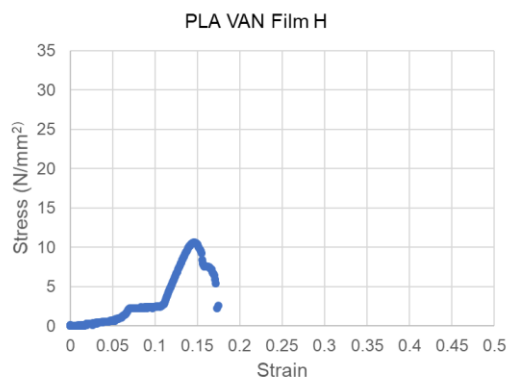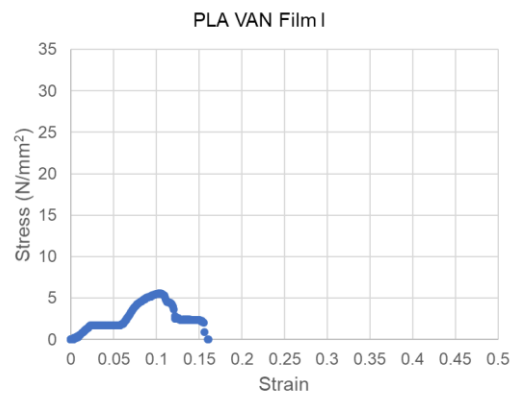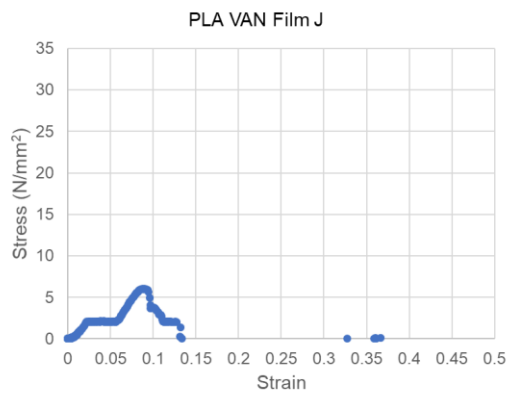

Supplement: S1 Fig — (A) curves from neat PLA films, (B) curves from PLA-VAN films. (PDF) [file pone.0337717.s001.pdf]
