# Supplementary material for: Delayed delivery of antibiotics by ultrasound-mediated rupture of polylactic acid pockets: In vitro and in vivo studies
Source: PLoS One. 2025 Dec 11;20(12):e0337717. doi: 10.1371/journal.pone.0337717 (PMC12698016; doi:10.1371/journal.pone.0337717)
Supplement: S1 Data — (PDF) [file pone.0337717.s002.pdf]

PLA Amounts

	Ruptured	Unruptured
<=0.5 g	21	18
>0.5 g	1	8

Contingency analysis

Table Analyzed	PLA amount		
P value and statistical significance			
Test	Fisher's exact test		
P value	0.0276		
P value summary	*		
One- or two-sided	Two-sided		
Statistically significant (P < 0.05)?	Yes		
Data analyzed	Ruptured	Unruptured	Total
<=0.5 g	21	18	39
>0.5 g	1	8	9
Total	22	26	48
Percentage of row total	Ruptured	Unruptured	
<=0.5 g	53.85%	46.15%	
>0.5 g	11.11%	88.89%	
Percentage of column total	Ruptured	Unruptured	
<=0.5 g	95.45%	69.23%	
>0.5 g	4.55%	30.77%	
Percentage of grand total	Ruptured	Unruptured	
<=0.5 g	43.75%	37.50%	
>0.5 g	2.08%	16.67%	

Clinical vs HIFU

	Ruptured	Unruptured
Clinical US	18	22
HIFU	4	4

Table Analyzed	type of US 12.20.22		
P value and statistical significance			
Test	Fisher's exact test		
P value	>0.9999		
P value summary	ns		
One- or two-sided	Two-sided		
Statistically significant (P < 0.05)?	No		
Data analyzed	Yes Rupture	No Rupture	Total
S50	18	22	40
HIFU	4	4	8
Total	22	26	48
Percentage of row total	Yes Rupture	No Rupture	
S50	45.00%	55.00%	
HIFU	50.00%	50.00%	
Percentage of column total	Yes Rupture	No Rupture	
S50	81.82%	84.62%	
HIFU	18.18%	15.38%	
Percentage of grand total	Yes Rupture	No Rupture	
S50	37.50%	45.83%	
HIFU	8.33%	8.33%	

Definity droplets vs Sonazoid

	Ruptured	Unruptured
Sonazoid	18	19
Definity droplets	4	4

Table Analyzed	Son v Def 12.20.22		
P value and statistical significance			
Test	Fisher's exact test		
P value	>0.9999		
P value summary	ns		
One- or two-sided	Two-sided		
Statistically significant (P < 0.05)?	No		
Data analyzed	Yes Rupture	No Rupture	Total
Def	4	4	8
Son	18	19	37
Total	22	23	45
Percentage of row total	Yes Rupture	No Rupture	
Def	50.00%	50.00%	
Son	48.65%	51.35%	
Percentage of column total	Yes Rupture	No Rupture	
Def	18.18%	17.39%	
Son	81.82%	82.61%	
Percentage of grand total	Yes Rupture	No Rupture	
Def	8.89%	8.89%	
Son	40.00%	42.22%	

Days	PLA				PLA-VAN			
0	0.022293	0.018076	0.0136	0.038584	0.0304	0.039225	0.023644	0.02432
1	0.04256	0.031221	0.0232	0.031569	0.638395	0.071587	0.19028	0.664741
2	0.047626	0.034508	0.0224	0.194091	1.127831	0.081393	0.655284	1.387242
3	0.041546	0.047654	0.0248	0.342582	1.625374	0.114735	1.095517	2.10265
4	0.067893	0.051762	0.024	0.413904	2.372194	0.152979	1.808223	2.709632
5	0.031413	0.059978	0.0352	0.454827	2.701525	0.157883	2.354292	3.375386
6	0.032426	0.056691	0.0296	0.498088	3.277094	0.21476	2.749489	3.686477
7	0.041546	0.061621	0.0384	0.620857	3.664184	0.248101	3.426165	4.1445

Table Analyzed	Day 7
Column B	VAN-PLA
vs.	vs.
Column A	neat PLA
Mann Whitney test	
P value	0.0571
Exact or approximate P value?	Exact
P value summary	ns
Significantly different (P < 0.05)?	No
One- or two-tailed P value?	Two-tailed
Sum of ranks in column A,B	11 , 25
Mann-Whitney U	1
Difference between medians	
Median of column A	0.05158, n=4
Median of column B	3.545, n=4
Difference: Actual	3.494
Difference: Hodges-Lehmann	3.386

neat PLAvPLA-VAN Ult Strength

neat PLA	PLA-VAN
25.3	13.83
30.76	16.84
20.54	12.18
24.17	10.65
24.73	5.59
15.7	6.04
16.42	8.14
7.38	13
18.82	10.58
14.33	6.98
	9.6

Table Analyzed	neat PLAvPLA-VAN Ult Strength
Column B	PLA-VAN
vs.	vs.
Column A	neat PLA
Mann Whitney test	
P value	0.0011
Exact or approximate P value?	Exact
P value summary	**
Significantly different (P < 0.05)?	Yes
One- or two-tailed P value?	Two-tailed
Sum of ranks in column A,B	154 , 77
Mann-Whitney U	11
Difference between medians	
Median of column A	19.68, n=10
Median of column B	10.58, n=11
Difference: Actual	-9.100
Difference: Hodges-Lehmann	-9.775

Toughness--Combined 3_12 and 12_12 toughness

neat PLA	PLA-VAN
1.72	0.92
4.48	0.91
3.52	0.68
5.88	0.86
6.92	0.45
2.17	0.38
2.3	0.9326
1.8604	0.5168
3.0543	0.7833
2.812	0.4476
	0.5785

Unpaired t test

	neat PLA	PLA-VAN
Test for normal distribution		
Shapiro-Wilk test		
W	0.8771	0.8895
P value	0.1210	0.1370
Passed normality test (alpha=0.05)?	Yes	Yes
P value summary	ns	ns
Number of values	10	11

Table Analyzed	Combined 3_12 and 12_12 toughness
Column B	PLA-VAN
vs.	vs.
Column A	neat PLA
Unpaired t test	
P value	<0.0001
P value summary	****
Significantly different (P < 0.05)?	Yes
One- or two-tailed P value?	Two-tailed
t, df	t=5.220, df=19
How big is the difference?	
Mean of column A	3.472
Mean of column B	0.6781
Difference between means (B - A) ± SEM	-2.794 ± 0.5352
95% confidence interval	-3.914 to -1.674
R squared (eta squared)	0.5892
F test to compare variances	
F, DFn, Dfd	69.08, 9, 10
P value	<0.0001
P value summary	****
Significantly different (P < 0.05)?	Yes
Data analyzed	
Sample size, column A	10
Sample size, column B	11

Primary Data cut into 4 quadrants

AFM of Neat PLA (No Van) Height Retrace (Image 0020)

Quadrant 1 Data (16384 values):

5.76E-08	5.84E-08	5.74E-08	5.65E-08	5.60E-08	5.63E-08	5.56E-08	5.80E-08	5.77E-08	5.81E-08	5.82E-08	5.72E-08	5.77E-08	5.69E-08	5.60E-08
5.66E-08	5.67E-08	5.64E-08	5.52E-08	5.47E-08	5.48E-08	5.44E-08	5.69E-08	5.69E-08	5.76E-08	5.69E-08	5.61E-08	5.62E-08	5.45E-08	5.58E-08
5.51E-08	5.50E-08	5.45E-08	5.38E-08	5.34E-08	5.37E-08	5.34E-08	5.38E-08	5.48E-08	5.62E-08	5.51E-08	5.50E-08	5.40E-08	5.37E-08	5.50E-08
5.32E-08	5.35E-08	5.29E-08	5.26E-08	5.21E-08	5.26E-08	5.23E-08	5.25E-08	5.27E-08	5.37E-08	5.32E-08	5.39E-08	5.23E-08	5.22E-08	5.27E-08
5.19E-08	5.17E-08	5.13E-08	5.12E-08	5.10E-08	5.12E-08	5.14E-08	5.16E-08	5.16E-08	5.12E-08	5.12E-08	5.24E-08	5.05E-08	5.06E-08	5.08E-08
5.10E-08	5.08E-08	4.99E-08	4.98E-08	4.96E-08	5.00E-08	5.09E-08	5.04E-08	5.06E-08	5.00E-08	4.93E-08	5.04E-08	4.91E-08	4.94E-08	4.93E-08
4.96E-08	4.94E-08	4.83E-08	4.89E-08	4.83E-08	4.82E-08	5.03E-08	4.92E-08	4.91E-08	4.87E-08	4.81E-08	4.85E-08	4.79E-08	4.81E-08	4.74E-08
4.85E-08	4.78E-08	4.61E-08	4.67E-08	4.65E-08	4.69E-08	4.74E-08	4.75E-08	4.79E-08	4.77E-08	4.69E-08	4.67E-08	4.68E-08	4.66E-08	4.56E-08
4.77E-08	4.68E-08	4.51E-08	4.59E-08	4.51E-08	4.59E-08	4.57E-08	4.58E-08	4.63E-08	4.67E-08	4.54E-08	4.56E-08	4.53E-08	4.56E-08	4.39E-08
4.68E-08	4.58E-08	4.45E-08	4.55E-08	4.43E-08	4.43E-08	4.41E-08	4.47E-08	4.48E-08	4.62E-08	4.43E-08	4.44E-08	4.36E-08	4.40E-08	4.27E-08
4.52E-08	4.46E-08	4.40E-08	4.51E-08	4.37E-08	4.30E-08	4.29E-08	4.36E-08	4.38E-08	4.49E-08	4.29E-08	4.37E-08	4.27E-08	4.28E-08	4.19E-08
4.35E-08	4.30E-08	4.32E-08	4.35E-08	4.31E-08	4.21E-08	4.22E-08	4.23E-08	4.22E-08	4.30E-08	4.16E-08	4.25E-08	4.22E-08	4.17E-08	4.09E-08
4.24E-08	4.18E-08	4.16E-08	4.17E-08	4.25E-08	4.11E-08	4.09E-08	4.12E-08	4.06E-08	4.11E-08	4.03E-08	4.13E-08	4.19E-08	4.01E-08	3.95E-08
4.14E-08	4.09E-08	4.05E-08	3.99E-08	4.11E-08	3.99E-08	4.01E-08	4.02E-08	3.96E-08	3.98E-08	3.95E-08	4.06E-08	4.14E-08	3.82E-08	3.80E-08
4.05E-08	4.03E-08	3.97E-08	3.89E-08	3.99E-08	3.87E-08	3.96E-08	3.92E-08	3.86E-08	3.84E-08	3.85E-08	3.95E-08	3.71E-08	3.70E-08	3.69E-08
3.92E-08	3.91E-08	3.85E-08	3.81E-08	3.83E-08	3.79E-08	3.86E-08	3.82E-08	3.73E-08	3.69E-08	3.70E-08	3.79E-08	3.57E-08	3.60E-08	3.62E-08
3.82E-08	3.75E-08	3.68E-08	3.62E-08	3.68E-08	3.70E-08	3.72E-08	3.72E-08	3.64E-08	3.56E-08	3.53E-08	3.64E-08	3.52E-08	3.49E-08	3.54E-08
3.73E-08	3.65E-08	3.59E-08	3.57E-08	3.54E-08	3.58E-08	3.58E-08	3.60E-08	3.55E-08	3.42E-08	3.40E-08	3.51E-08	3.46E-08	3.40E-08	3.43E-08
3.69E-08	3.59E-08	3.50E-08	3.51E-08	3.45E-08	3.50E-08	3.41E-08	3.41E-08	3.41E-08	3.31E-08	3.28E-08	3.37E-08	3.37E-08	3.38E-08	3.40E-08
3.64E-08	3.50E-08	3.46E-08	3.44E-08	3.42E-08	3.49E-08	3.29E-08	3.25E-08	3.24E-08	3.23E-08	3.21E-08	3.32E-08	3.29E-08	3.33E-08	3.32E-08
3.55E-08	3.42E-08	3.38E-08	3.38E-08	3.36E-08	3.39E-08	3.17E-08	3.10E-08	3.10E-08	3.12E-08	3.17E-08	3.27E-08	3.19E-08	3.24E-08	3.21E-08
3.42E-08	3.28E-08	3.28E-08	3.33E-08	3.25E-08	3.18E-08	3.05E-08	3.00E-08	3.00E-08	3.04E-08	3.10E-08	3.15E-08	3.04E-08	3.07E-08	3.06E-08
3.23E-08	3.15E-08	3.16E-08	3.23E-08	3.18E-08	2.97E-08	2.98E-08	2.93E-08	2.91E-08	2.96E-08	2.98E-08	2.98E-08	2.91E-08	2.95E-08	2.98E-08
3.11E-08	3.07E-08	3.04E-08	3.11E-08	3.04E-08	2.90E-08	2.96E-08	2.88E-08	2.84E-08	2.85E-08	2.87E-08	2.88E-08	2.87E-08	2.87E-08	2.86E-08
3.02E-08	3.02E-08	2.92E-08	3.01E-08	2.89E-08	2.89E-08	2.95E-08	2.85E-08	2.84E-08	2.80E-08	2.81E-08	2.79E-08	2.89E-08	2.89E-08	2.75E-08
2.93E-08	2.97E-08	2.81E-08	2.89E-08	2.77E-08	2.88E-08	2.89E-08	2.83E-08	2.84E-08	2.74E-08	2.75E-08	2.74E-08	2.96E-08	2.92E-08	2.72E-08
2.78E-08	2.85E-08	2.72E-08	2.81E-08	2.71E-08	2.82E-08	2.80E-08	2.80E-08	2.72E-08	2.65E-08	2.67E-08	2.68E-08	2.93E-08	2.83E-08	2.65E-08
2.65E-08	2.72E-08	2.65E-08	2.74E-08	2.65E-08	2.70E-08	2.72E-08	2.72E-08	2.58E-08	2.56E-08	2.59E-08	2.54E-08	2.73E-08	2.63E-08	2.54E-08
2.57E-08	2.64E-08	2.62E-08	2.65E-08	2.52E-08	2.57E-08	2.64E-08	2.61E-08	2.49E-08	2.51E-08	2.49E-08	2.45E-08	2.52E-08	2.45E-08	2.52E-08
2.52E-08	2.61E-08	2.60E-08	2.49E-08	2.41E-08	2.44E-08	2.52E-08	2.58E-08	2.44E-08	2.41E-08	2.41E-08	2.37E-08	2.34E-08	2.32E-08	2.43E-08
2.48E-08	2.60E-08	2.54E-08	2.35E-08	2.30E-08	2.31E-08	2.44E-08	2.48E-08	2.39E-08	2.37E-08	2.34E-08	2.31E-08	2.21E-08	2.30E-08	2.32E-08
2.36E-08	2.49E-08	2.45E-08	2.22E-08	2.23E-08	2.27E-08	2.33E-08	2.37E-08	2.34E-08	2.33E-08	2.27E-08	2.23E-08	2.13E-08	2.24E-08	2.13E-08
2.21E-08	2.29E-08	2.39E-08	2.13E-08	2.20E-08	2.24E-08	2.23E-08	2.28E-08	2.23E-08	2.17E-08	2.18E-08	2.13E-08	2.06E-08	2.10E-08	2.04E-08
2.13E-08	2.14E-08	2.21E-08	2.11E-08	2.17E-08	2.13E-08	2.15E-08	2.21E-08	2.11E-08	2.05E-08	2.06E-08	2.01E-08	1.96E-08	2.00E-08	1.95E-08
2.07E-08	2.08E-08	2.09E-08	2.04E-08	2.10E-08	2.09E-08	2.07E-08	2.08E-08	1.98E-08	1.93E-08	1.95E-08	1.86E-08	1.82E-08	1.84E-08	1.85E-08
1.93E-08	2.00E-08	2.05E-08	1.99E-08	2.01E-08	2.02E-08	1.93E-08	1.90E-08	1.87E-08	1.85E-08	1.88E-08	1.78E-08	1.73E-08	1.69E-08	1.77E-08
1.84E-08	1.89E-08	1.98E-08	1.92E-08	1.95E-08	1.89E-08	1.80E-08	1.79E-08	1.78E-08	1.73E-08	1.82E-08	1.70E-08	1.69E-08	1.64E-08	1.71E-08
1.76E-08	1.79E-08	1.89E-08	1.82E-08	1.91E-08	1.78E-08	1.71E-08	1.65E-08	1.66E-08	1.63E-08	1.74E-08	1.65E-08	1.62E-08	1.57E-08	1.65E-08
1.73E-08	1.68E-08	1.77E-08	1.70E-08	1.80E-08	1.67E-08	1.60E-08	1.57E-08	1.58E-08	1.56E-08	1.68E-08	1.59E-08	1.57E-08	1.48E-08	1.58E-08
1.68E-08	1.65E-08	1.66E-08	1.63E-08	1.61E-08	1.63E-08	1.54E-08	1.46E-08	1.54E-08	1.51E-08	1.61E-08	1.49E-08	1.51E-08	1.42E-08	1.49E-08
1.62E-08	1.57E-08	1.60E-08	1.57E-08	1.55E-08	1.67E-08	1.54E-08	1.39E-08	1.48E-08	1.46E-08	1.51E-08	1.42E-08	1.49E-08	1.37E-08	1.45E-08
1.56E-08	1.51E-08	1.56E-08	1.59E-08	1.63E-08	1.71E-08	1.55E-08	1.37E-08	1.44E-08	1.48E-08	1.49E-08	1.43E-08	1.48E-08	1.38E-08	1.47E-08
1.39E-08	1.36E-08	1.46E-08	1.56E-08	1.62E-08	1.58E-08	1.46E-08	1.30E-08	1.30E-08	1.37E-08	1.35E-08	1.28E-08	1.31E-08	1.25E-08	1.35E-08
1.29E-08	1.21E-08	1.32E-08	1.42E-08	1.63E-08	1.53E-08	1.42E-08	1.25E-08	1.20E-08	1.30E-08	1.26E-08	1.15E-08	1.21E-08	1.12E-08	1.18E-08
1.19E-08	1.11E-08	1.19E-08	1.29E-08	1.51E-08	1.47E-08	1.38E-08	1.19E-08	1.15E-08	1.28E-08	1.17E-08	1.06E-08	1.12E-08	1.06E-08	1.02E-08
1.17E-08	1.08E-08	1.13E-08	1.14E-08	1.22E-08	1.35E-08	1.24E-08	1.10E-08	1.08E-08	1.20E-08	1.09E-08	9.65E-09	1.04E-08	9.60E-09	9.14E-09
1.17E-08	1.04E-08	1.09E-08	1.02E-08	1.08E-08	1.13E-08	1.07E-08	9.91E-09	9.74E-09	1.04E-08	9.63E-09	8.99E-09	9.67E-09	8.66E-09	7.90E-09
1.02E-08	9.13E-09	1.00E-08	9.63E-09	9.52E-09	9.52E-09	8.97E-09	9.01E-09	8.89E-09	8.95E-09	8.33E-09	8.14E-09	9.09E-09	7.88E-09	7.29E-09
8.51E-09	7.61E-09	8.55E-09	8.72E-09	8.31E-09	8.44E-09	8.06E-09	8.18E-09	8.17E-09	7.76E-09	7.22E-09	7.17E-09	8.00E-09	7.03E-09	6.63E-09
7.17E-09	6.72E-09	7.22E-09	7.70E-09	7.19E-09	7.41E-09	7.74E-09	7.35E-09	7.83E-09	6.69E-09	6.44E-09	6.21E-09	6.87E-09	6.31E-09	5.87E-09
6.87E-09	6.05E-09	6.17E-09	6.62E-09	6.38E-09	6.52E-09	7.12E-09	6.91E-09	7.31E-09	5.41E-09	5.49E-09	5.58E-09	5.81E-09	5.58E-09	5.51E-09
6.42E-09	5.28E-09	5.32E-09	5.43E-09	5.38E-09	5.54E-09	6.08E-09	6.37E-09	6.46E-09	4.77E-09	4.97E-09	4.73E-09	5.12E-09	6.06E-09	5.43E-09
5.69E-09	4.37E-09	4.45E-09	4.19E-09	4.79E-09	5.41E-09	5.64E-09	5.73E-09	5.39E-09	5.15E-09	4.96E-09	4.04E-09	4.45E-09	6.20E-09	4.63E-09
5.02E-09	3.98E-09	4.00E-09	3.79E-09	4.54E-09	4.68E-09	5.00E-09	5.09E-09	4.43E-09	5.10E-09	4.74E-09	3.27E-09	3.53E-09	5.18E-09	3.62E-09
3.80E-09	3.68E-09	4.15E-09	4.05E-09	3.94E-09	4.13E-09	4.40E-09	3.79E-09	3.43E-09	4.04E-09	3.42E-09	2.66E-09	2.49E-09	3.43E-09	2.69E-09
2.78E-09	3.22E-09	4.02E-09	3.48E-09	3.21E-09	3.63E-09	3.65E-09	2.42E-09	2.34E-09	2.58E-09	2.33E-09	2.19E-09	2.17E-09	2.45E-09	1.89E-09
2.05E-09	2.52E-09	3.61E-09	2.64E-09	2.43E-09	2.92E-09	2.84E-09	1.70E-09	1.52E-09	1.78E-09	1.83E-09	1.56E-09	1.52E-09	2.01E-09	9.95E-10
1.44E-09	1.28E-09	2.48E-09	1.89E-09	2.04E-09	2.14E-09	2.07E-09	9.49E-10	6.53E-10	1.35E-09	1.62E-09	8.50E-10	3.68E-10	1.24E-09	2.97E-10
1.66E-10	5.44E-11	1.20E-09	7.74E-10	1.04E-09	5.68E-10	5.56E-10	-3.46E-10	-2.66E-10	2.89E-10	6.59E-10	-1.68E-10	-8.16E-10	-2.17E-10	-1.09E-09
-7.80E-10	-5.07E-10	3.64E-10	2.00E-10	-1.64E-10	-3.62E-10	-1.68E-10	-8.09E-10	-9.68E-10	-1.20E-10	-2.69E-10	-8.64E-10	-1.30E-09	-6.16E-10	-1.71E-09
-1.56E-09	-9.24E-10	-3.55E-10	-5.54E-10	-1.18E-09	-1.30E-09	-8.09E-10	-1.25E-09	-1.28E-09	-2.96E-10	-8.79E-10	-1.29E-09	-1.56E-09	-1.30E-09	-2.69E-09
-2.27E-09	-1.25E-09	-1.09E-09	-1.33E-09	-1.77E-09	-2.41E-09	-1.83E-09	-1.95E-09	-1.32E-09	-9.98E-10	-1.52E-09	-1.62E-09	-2.06E-09	-2.24E-09	-3.46E-09
-2.47E-09	-1.54E-09	-1.95E-09	-2.03E-09	-2.37E-09	-2.91E-09	-2.87E-09	-2.73E-09	-1.92E-09	-1.52E-09	-1.87E-09	-2.09E-09	-2.99E-09	-3.30E-09	-3.52E-09
-3.61E-09	-2.35E-09	-3.07E-09	-3.09E-09	-2.85E-09	-3.44E-09	-3.80E-09	-3.22E-09	-2.54E-09	-2.09E-09	-2.28E-09	-2.89E-09	-3.76E-09	-3.78E-09	-3.13E-09
-4.73E-09	-3.46E-09	-3.77E-09	-3.75E-09	-3.08E-09	-3.66E-09	-4.45E-09	-3.27E-09	-2.87E-09	-2.70E-09	-3.40E-09	-4.26E-09	-4.67E-09	-4.46E-09	-2.98E-09
-5.29E-09	-4.38E-09	-4.66E-09	-4.08E-09	-4.19E-09	-4.54E-09	-4.81E-09	-3.19E-09	-3.54E-09	-3.91E-09	-4.77E-09	-5.45E-09	-5.51E-09	-4.96E-09	-3.68E-09
-5.90E-09	-5.30E-09	-5.71E-09	-4.98E-09	-5.04E-09	-5.56E-09	-5.79E-09	-4.46E-09	-4.50E-09	-5.44E-09	-5.89E-09	-6.44E-09	-5.97E-09	-5.72E-09	-4.64E-09
-6.64E-09	-6.30E-09	-6.74E-09	-6.27E-09	-6.17E-09	-6.81E-09	-6.85E-09	-5.93E-09	-6.05E-09	-6.90E-09	-6.89E-09	-7.07E-09	-6.10E-09	-5.76E-09	-4.89E-09
-7.57E-09	-7.21E-09	-7.33E-09	-7.83E-09	-7.68E-09	-7.89E-09	-7.76E-09	-7.18E-09	-7.09E-09	-7.78E-09	-7.63E-09	-7.75E-09	-7.03E-09	-5.89E-09	-5.50E-09
-8.79E-09	-8.08E-09	-8.78E-09	-9.04E-09	-9.25E-09	-8.86E-09	-8.45E-09	-8.43E-09	-8.14E-09	-8.71E-09	-8.46E-09	-8.55E-09	-7.89E-09	-6.32E-09	-6.05E-09
-9.56E-09	-8.89E-09	-9.69E-09	-9.80E-09	-1.00E-08	-1.00E-08	-9.55E-09	-9.89E-09	-8.93E-09	-9.35E-09	-9.60E-09	-9.24E-09	-8.16E-09	-6.99E-09	-6.91E-09
-1.00E-08	-1.01E-08	-1.00E-08	-1.04E-08	-1.04E-08	-1.13E-08	-1.09E-08	-1.06E-08	-9.86E-09	-1.02E-08	-1.04E-08	-9.97E-09	-8.77E-09	-7.95E-09	-8.19E-09
-1.10E-08	-1.08E-08	-1.10E-08	-1.10E-08	-1.07E-08	-1.19E-08	-1.17E-08	-1.12E-08	-1.07E-08	-1.10E-08	-1.13E-08	-1.09E-08	-9.78E-09	-8.88E-09	-9.45E-09
-1.19E-08	-1.18E-08	-1.20E-08	-1.12E-08	-1.15E-08	-1.27E-08	-1.24E-08	-1.15E-08	-1.13E-08	-1.24E-08	-1.18E-08	-1.18E-08	-1.06E-08	-9.97E-09	-1.09E-08
-1.28E-08	-1.25E-08	-1.29E-08	-1.23E-08	-1.23E-08	-1.33E-08	-1.27E-08	-1.17E-08	-1.22E-08	-1.29E-08	-1.25E-08	-1.28E-08	-1.20E-08	-1.20E-08	-1.19E-08
-1.40E-08	-1.35E-08	-1.41E-08	-1.33E-08	-1.31E-08	-1.36E-08	-1.29E-08	-1.22E-08	-1.28E-08	-1.34E-08	-1.33E-08	-1.35E-08	-1.30E-08	-1.34E-08	-1.30E-08
-1.53E-08	-1.46E-08	-1.52E-08	-1.48E-08	-1.45E-08	-1.46E-08	-1.36E-08	-1.29E-08	-1.36E-08	-1.43E-08	-1.40E-08	-1.44E-08	-1.40E-08	-1.41E-08	-1.42E-08
-1.61E-08	-1.55E-08	-1.59E-08	-1.59E-08	-1.58E-08	-1.57E-08	-1.53E-08	-1.42E-08	-1.50E-08	-1.53E-08	-1.46E-08	-1.50E-08	-1.46E-08	-1.50E-08	-1.50E-08

-1.63E-08	-1.60E-08	-1.66E-08	-1.68E-08	-1.70E-08	-1.72E-08	-1.65E-08	-1.60E-08	-1.59E-08	-1.62E-08	-1.51E-08	-1.57E-08	-1.53E-08	-1.60E-08	-1.60E-08
-1.69E-08	-1.68E-08	-1.72E-08	-1.76E-08	-1.83E-08	-1.79E-08	-1.68E-08	-1.71E-08	-1.70E-08	-1.69E-08	-1.59E-08	-1.68E-08	-1.56E-08	-1.64E-08	-1.63E-08
-1.78E-08	-1.77E-08	-1.80E-08	-1.82E-08	-1.84E-08	-1.84E-08	-1.71E-08	-1.80E-08	-1.74E-08	-1.73E-08	-1.71E-08	-1.81E-08	-1.65E-08	-1.67E-08	-1.64E-08
-1.80E-08	-1.84E-08	-1.84E-08	-1.86E-08	-1.85E-08	-1.88E-08	-1.86E-08	-1.90E-08	-1.81E-08	-1.83E-08	-1.84E-08	-1.89E-08	-1.73E-08	-1.71E-08	-1.69E-08
-1.87E-08	-1.89E-08	-1.90E-08	-1.92E-08	-1.89E-08	-1.92E-08	-1.96E-08	-1.96E-08	-1.88E-08	-1.95E-08	-1.91E-08	-1.92E-08	-1.79E-08	-1.76E-08	-1.78E-08
-1.97E-08	-1.98E-08	-2.00E-08	-1.96E-08	-1.92E-08	-1.95E-08	-1.99E-08	-2.02E-08	-1.97E-08	-2.04E-08	-1.94E-08	-1.98E-08	-1.91E-08	-1.84E-08	-1.88E-08
-2.04E-08	-2.10E-08	-2.12E-08	-2.02E-08	-1.94E-08	-1.99E-08	-2.02E-08	-2.09E-08	-2.06E-08	-2.10E-08	-2.00E-08	-2.07E-08	-2.05E-08	-1.96E-08	-1.96E-08
-2.16E-08	-2.19E-08	-2.21E-08	-2.09E-08	-2.07E-08	-2.09E-08	-2.08E-08	-2.18E-08	-2.16E-08	-2.18E-08	-2.14E-08	-2.16E-08	-2.13E-08	-2.04E-08	-2.00E-08
-2.25E-08	-2.21E-08	-2.22E-08	-2.19E-08	-2.19E-08	-2.14E-08	-2.19E-08	-2.28E-08	-2.26E-08	-2.23E-08	-2.19E-08	-2.21E-08	-2.12E-08	-2.03E-08	-2.05E-08
-2.30E-08	-2.24E-08	-2.27E-08	-2.28E-08	-2.29E-08	-2.23E-08	-2.28E-08	-2.33E-08	-2.32E-08	-2.27E-08	-2.23E-08	-2.27E-08	-2.11E-08	-2.03E-08	-2.11E-08
-2.37E-08	-2.33E-08	-2.37E-08	-2.38E-08	-2.37E-08	-2.33E-08	-2.34E-08	-2.35E-08	-2.35E-08	-2.33E-08	-2.32E-08	-2.31E-08	-2.18E-08	-2.15E-08	-2.19E-08
-2.43E-08	-2.44E-08	-2.45E-08	-2.48E-08	-2.41E-08	-2.38E-08	-2.35E-08	-2.33E-08	-2.38E-08	-2.37E-08	-2.36E-08	-2.36E-08	-2.30E-08	-2.25E-08	-2.28E-08
-2.47E-08	-2.49E-08	-2.51E-08	-2.53E-08	-2.46E-08	-2.42E-08	-2.38E-08	-2.35E-08	-2.39E-08	-2.38E-08	-2.39E-08	-2.42E-08	-2.41E-08	-2.33E-08	-2.30E-08
-2.50E-08	-2.55E-08	-2.56E-08	-2.59E-08	-2.51E-08	-2.48E-08	-2.45E-08	-2.47E-08	-2.46E-08	-2.42E-08	-2.43E-08	-2.44E-08	-2.49E-08	-2.38E-08	-2.36E-08
-2.55E-08	-2.61E-08	-2.64E-08	-2.66E-08	-2.59E-08	-2.56E-08	-2.56E-08	-2.58E-08	-2.54E-08	-2.50E-08	-2.48E-08	-2.48E-08	-2.52E-08	-2.44E-08	-2.46E-08
-2.63E-08	-2.65E-08	-2.67E-08	-2.68E-08	-2.67E-08	-2.66E-08	-2.66E-08	-2.68E-08	-2.60E-08	-2.57E-08	-2.52E-08	-2.53E-08	-2.56E-08	-2.48E-08	-2.51E-08
-2.72E-08	-2.70E-08	-2.67E-08	-2.67E-08	-2.72E-08	-2.73E-08	-2.75E-08	-2.77E-08	-2.69E-08	-2.64E-08	-2.59E-08	-2.56E-08	-2.62E-08	-2.52E-08	-2.58E-08
-2.81E-08	-2.79E-08	-2.72E-08	-2.69E-08	-2.75E-08	-2.77E-08	-2.81E-08	-2.84E-08	-2.77E-08	-2.76E-08	-2.72E-08	-2.66E-08	-2.71E-08	-2.58E-08	-2.64E-08
-2.88E-08	-2.88E-08	-2.81E-08	-2.77E-08	-2.83E-08	-2.82E-08	-2.87E-08	-2.89E-08	-2.85E-08	-2.83E-08	-2.79E-08	-2.72E-08	-2.75E-08	-2.63E-08	-2.62E-08
-2.92E-08	-2.92E-08	-2.89E-08	-2.87E-08	-2.93E-08	-2.90E-08	-2.95E-08	-2.94E-08	-2.90E-08	-2.85E-08	-2.81E-08	-2.74E-08	-2.74E-08	-2.64E-08	-2.63E-08
-2.96E-08	-2.97E-08	-2.98E-08	-2.95E-08	-3.03E-08	-2.99E-08	-2.99E-08	-2.97E-08	-2.91E-08	-2.86E-08	-2.83E-08	-2.76E-08	-2.75E-08	-2.66E-08	-2.70E-08
-2.99E-08	-3.00E-08	-3.05E-08	-3.02E-08	-3.08E-08	-3.03E-08	-2.99E-08	-2.95E-08	-2.93E-08	-2.93E-08	-2.88E-08	-2.78E-08	-2.75E-08	-2.69E-08	-2.77E-08
-3.06E-08	-3.06E-08	-3.08E-08	-3.05E-08	-3.08E-08	-3.04E-08	-2.98E-08	-2.94E-08	-2.92E-08	-2.95E-08	-2.90E-08	-2.79E-08	-2.73E-08	-2.74E-08	-2.85E-08
-3.14E-08	-3.10E-08	-3.10E-08	-3.08E-08	-3.08E-08	-3.05E-08	-2.98E-08	-2.95E-08	-2.92E-08	-2.96E-08	-2.91E-08	-2.77E-08	-2.72E-08	-2.74E-08	-2.88E-08
-3.18E-08	-3.12E-08	-3.10E-08	-3.09E-08	-3.09E-08	-3.09E-08	-3.02E-08	-2.97E-08	-2.94E-08	-2.98E-08	-2.91E-08	-2.80E-08	-2.76E-08	-2.74E-08	-2.89E-08
-3.24E-08	-3.15E-08	-3.12E-08	-3.10E-08	-3.11E-08	-3.15E-08	-3.12E-08	-3.06E-08	-3.03E-08	-3.02E-08	-2.93E-08	-2.86E-08	-2.86E-08	-2.83E-08	-2.91E-08
-3.29E-08	-3.17E-08	-3.16E-08	-3.14E-08	-3.13E-08	-3.21E-08	-3.18E-08	-3.15E-08	-3.12E-08	-3.11E-08	-3.00E-08	-2.95E-08	-2.97E-08	-2.93E-08	-2.94E-08
-3.32E-08	-3.19E-08	-3.18E-08	-3.19E-08	-3.17E-08	-3.26E-08	-3.21E-08	-3.21E-08	-3.17E-08	-3.16E-08	-3.05E-08	-3.00E-08	-3.07E-08	-3.04E-08	-2.97E-08
-3.37E-08	-3.21E-08	-3.21E-08	-3.24E-08	-3.25E-08	-3.29E-08	-3.27E-08	-3.29E-08	-3.22E-08	-3.21E-08	-3.08E-08	-3.01E-08	-3.08E-08	-3.11E-08	-3.03E-08
-3.40E-08	-3.31E-08	-3.28E-08	-3.32E-08	-3.33E-08	-3.34E-08	-3.32E-08	-3.32E-08	-3.27E-08	-3.24E-08	-3.11E-08	-3.06E-08	-3.08E-08	-3.14E-08	-3.12E-08
-3.45E-08	-3.39E-08	-3.34E-08	-3.37E-08	-3.41E-08	-3.38E-08	-3.36E-08	-3.34E-08	-3.30E-08	-3.28E-08	-3.13E-08	-3.15E-08	-3.12E-08	-3.21E-08	-3.22E-08
-3.47E-08	-3.42E-08	-3.37E-08	-3.41E-08	-3.48E-08	-3.41E-08	-3.39E-08	-3.33E-08	-3.27E-08	-3.28E-08	-3.21E-08	-3.20E-08	-3.18E-08	-3.25E-08	-3.29E-08
-3.51E-08	-3.44E-08	-3.43E-08	-3.47E-08	-3.46E-08	-3.40E-08	-3.41E-08	-3.34E-08	-3.29E-08	-3.29E-08	-3.24E-08	-3.27E-08	-3.20E-08	-3.32E-08	-3.29E-08
-3.54E-08	-3.50E-08	-3.50E-08	-3.51E-08	-3.45E-08	-3.36E-08	-3.42E-08	-3.37E-08	-3.39E-08	-3.36E-08	-3.29E-08	-3.34E-08	-3.27E-08	-3.36E-08	-3.30E-08
-3.60E-08	-3.55E-08	-3.50E-08	-3.54E-08	-3.48E-08	-3.40E-08	-3.42E-08	-3.37E-08	-3.45E-08	-3.44E-08	-3.36E-08	-3.40E-08	-3.36E-08	-3.42E-08	-3.29E-08
-3.69E-08	-3.65E-08	-3.55E-08	-3.57E-08	-3.50E-08	-3.47E-08	-3.47E-08	-3.45E-08	-3.50E-08	-3.51E-08	-3.40E-08	-3.45E-08	-3.42E-08	-3.45E-08	-3.33E-08
-3.70E-08	-3.67E-08	-3.61E-08	-3.58E-08	-3.52E-08	-3.53E-08	-3.56E-08	-3.54E-08	-3.55E-08	-3.53E-08	-3.41E-08	-3.45E-08	-3.44E-08	-3.49E-08	-3.35E-08
-3.68E-08	-3.69E-08	-3.62E-08	-3.61E-08	-3.51E-08	-3.56E-08	-3.59E-08	-3.56E-08	-3.58E-08	-3.54E-08	-3.40E-08	-3.48E-08	-3.47E-08	-3.50E-08	-3.33E-08
-3.68E-08	-3.73E-08	-3.65E-08	-3.66E-08	-3.55E-08	-3.60E-08	-3.57E-08	-3.54E-08	-3.57E-08	-3.56E-08	-3.43E-08	-3.46E-08	-3.47E-08	-3.52E-08	-3.36E-08
-3.69E-08	-3.74E-08	-3.66E-08	-3.71E-08	-3.59E-08	-3.60E-08	-3.57E-08	-3.55E-08	-3.54E-08	-3.54E-08	-3.44E-08	-3.47E-08	-3.47E-08	-3.57E-08	-3.39E-08
-3.73E-08	-3.75E-08	-3.66E-08	-3.70E-08	-3.57E-08	-3.57E-08	-3.58E-08	-3.58E-08	-3.51E-08	-3.52E-08	-3.47E-08	-3.49E-08	-3.50E-08	-3.61E-08	-3.42E-08
-3.74E-08	-3.76E-08	-3.67E-08	-3.66E-08	-3.62E-08	-3.57E-08	-3.60E-08	-3.56E-08	-3.46E-08	-3.44E-08	-3.42E-08	-3.42E-08	-3.50E-08	-3.56E-08	-3.39E-08
-3.74E-08	-3.76E-08	-3.66E-08	-3.68E-08	-3.64E-08	-3.61E-08	-3.65E-08	-3.50E-08	-3.38E-08	-3.36E-08	-3.37E-08	-3.37E-08	-3.48E-08	-3.51E-08	-3.40E-08
-3.76E-08	-3.80E-08	-3.65E-08	-3.66E-08	-3.65E-08	-3.62E-08	-3.67E-08	-3.48E-08	-3.37E-08	-3.32E-08	-3.37E-08	-3.37E-08	-3.52E-08	-3.48E-08	-3.40E-08
-3.73E-08	-3.83E-08	-3.66E-08	-3.65E-08	-3.66E-08	-3.69E-08	-3.68E-08	-3.52E-08	-3.40E-08	-3.31E-08	-3.42E-08	-3.45E-08	-3.55E-08	-3.51E-08	-3.41E-08
-3.76E-08	-3.86E-08	-3.68E-08	-3.66E-08	-3.68E-08	-3.74E-08	-3.70E-08	-3.56E-08	-3.50E-08	-3.43E-08	-3.49E-08	-3.50E-08	-3.52E-08	-3.55E-08	-3.41E-08
-3.76E-08	-3.85E-08	-3.71E-08	-3.71E-08	-3.64E-08	-3.71E-08	-3.65E-08	-3.57E-08	-3.54E-08	-3.54E-08	-3.57E-08	-3.56E-08	-3.46E-08	-3.52E-08	-3.44E-08
-3.73E-08	-3.79E-08	-3.68E-08	-3.69E-08	-3.64E-08	-3.66E-08	-3.60E-08	-3.54E-08	-3.54E-08	-3.55E-08	-3.59E-08	-3.57E-08	-3.40E-08	-3.45E-08	-3.42E-08
-3.74E-08	-3.74E-08	-3.66E-08	-3.64E-08	-3.62E-08	-3.59E-08	-3.57E-08	-3.49E-08	-3.52E-08	-3.59E-08	-3.54E-08	-3.54E-08	-3.39E-08	-3.42E-08	-3.37E-08
-3.70E-08	-3.70E-08	-3.61E-08	-3.58E-08	-3.56E-08	-3.53E-08	-3.56E-08	-3.52E-08	-3.50E-08	-3.56E-08	-3.53E-08	-3.53E-08	-3.43E-08	-3.38E-08	-3.33E-08

Quadrant 1 Data Squared (16384 values):

3.32E-15	3.41E-15	3.29E-15	3.19E-15	3.14E-15	3.16E-15	3.09E-15	3.36E-15	3.33E-15	3.37E-15	3.39E-15	3.28E-15	3.33E-15	3.24E-15	3.13E-15
3.21E-15	3.22E-15	3.18E-15	3.04E-15	3.00E-15	3.00E-15	2.96E-15	3.23E-15	3.24E-15	3.32E-15	3.24E-15	3.15E-15	3.16E-15	2.97E-15	3.11E-15
3.03E-15	3.03E-15	2.97E-15	2.90E-15	2.85E-15	2.88E-15	2.85E-15	2.89E-15	3.00E-15	3.15E-15	3.03E-15	3.03E-15	2.91E-15	2.88E-15	3.03E-15
2.83E-15	2.86E-15	2.80E-15	2.77E-15	2.72E-15	2.77E-15	2.74E-15	2.76E-15	2.78E-15	2.88E-15	2.83E-15	2.91E-15	2.73E-15	2.72E-15	2.78E-15
2.70E-15	2.68E-15	2.64E-15	2.62E-15	2.60E-15	2.62E-15	2.65E-15	2.67E-15	2.67E-15	2.62E-15	2.62E-15	2.74E-15	2.55E-15	2.56E-15	2.58E-15
2.60E-15	2.58E-15	2.49E-15	2.48E-15	2.46E-15	2.50E-15	2.59E-15	2.54E-15	2.56E-15	2.50E-15	2.43E-15	2.54E-15	2.41E-15	2.44E-15	2.43E-15
2.46E-15	2.44E-15	2.33E-15	2.39E-15	2.33E-15	2.32E-15	2.53E-15	2.42E-15	2.42E-15	2.37E-15	2.31E-15	2.36E-15	2.30E-15	2.31E-15	2.24E-15
2.35E-15	2.28E-15	2.12E-15	2.18E-15	2.17E-15	2.20E-15	2.25E-15	2.25E-15	2.29E-15	2.27E-15	2.20E-15	2.18E-15	2.19E-15	2.17E-15	2.08E-15
2.27E-15	2.19E-15	2.03E-15	2.11E-15	2.03E-15	2.11E-15	2.09E-15	2.10E-15	2.14E-15	2.18E-15	2.06E-15	2.08E-15	2.05E-15	2.08E-15	1.93E-15
2.19E-15	2.10E-15	1.98E-15	2.07E-15	1.96E-15	1.97E-15	1.95E-15	2.00E-15	2.01E-15	2.13E-15	1.96E-15	1.97E-15	1.90E-15	1.94E-15	1.83E-15
2.05E-15	1.99E-15	1.93E-15	2.03E-15	1.91E-15	1.85E-15	1.84E-15	1.90E-15	1.92E-15	2.01E-15	1.84E-15	1.91E-15	1.82E-15	1.83E-15	1.76E-15
1.90E-15	1.85E-15	1.87E-15	1.89E-15	1.86E-15	1.78E-15	1.78E-15	1.79E-15	1.78E-15	1.85E-15	1.73E-15	1.80E-15	1.78E-15	1.74E-15	1.67E-15
1.80E-15	1.75E-15	1.73E-15	1.74E-15	1.80E-15	1.69E-15	1.67E-15	1.69E-15	1.65E-15	1.69E-15	1.62E-15	1.70E-15	1.75E-15	1.61E-15	1.56E-15
1.72E-15	1.68E-15	1.64E-15	1.59E-15	1.69E-15	1.59E-15	1.61E-15	1.62E-15	1.57E-15	1.58E-15	1.56E-15	1.65E-15	1.71E-15	1.46E-15	1.44E-15
1.64E-15	1.62E-15	1.57E-15	1.52E-15	1.59E-15	1.50E-15	1.57E-15	1.53E-15	1.49E-15	1.47E-15	1.48E-15	1.56E-15	1.38E-15	1.37E-15	1.36E-15
1.54E-15	1.53E-15	1.48E-15	1.45E-15	1.47E-15	1.43E-15	1.49E-15	1.46E-15	1.39E-15	1.36E-15	1.37E-15	1.44E-15	1.27E-15	1.30E-15	1.31E-15
1.46E-15	1.41E-15	1.35E-15	1.31E-15	1.36E-15	1.37E-15	1.39E-15	1.39E-15	1.32E-15	1.27E-15	1.24E-15	1.32E-15	1.24E-15	1.22E-15	1.25E-15
1.39E-15	1.33E-15	1.29E-15	1.27E-15	1.25E-15	1.28E-15	1.28E-15	1.29E-15	1.26E-15	1.17E-15	1.15E-15	1.23E-15	1.20E-15	1.16E-15	1.18E-15
1.36E-15	1.29E-15	1.23E-15	1.23E-15	1.19E-15	1.23E-15	1.16E-15	1.17E-15	1.16E-15	1.10E-15	1.07E-15	1.13E-15	1.14E-15	1.14E-15	1.15E-15
1.32E-15	1.23E-15	1.20E-15	1.18E-15	1.17E-15	1.22E-15	1.08E-15	1.06E-15	1.05E-15	1.04E-15	1.03E-15	1.10E-15	1.08E-15	1.11E-15	1.10E-15
1.26E-15	1.17E-15	1.14E-15	1.14E-15	1.13E-15	1.15E-15	1.01E-15	9.58E-16	9.61E-16	9.72E-16	1.01E-15	1.07E-15	1.02E-15	1.05E-15	1.03E-15
1.17E-15	1.08E-15	1.08E-15	1.11E-15	1.06E-15	1.01E-15	9.30E-16	9.01E-16	9.00E-16	9.23E-16	9.62E-16	9.94E-16	9.21E-16	9.45E-16	9.36E-16
1.04E-15	9.95E-16	9.96E-16	1.05E-15	1.01E-15	8.83E-16	8.86E-16	8.59E-16	8.45E-16	8.77E-16	8.89E-16	8.90E-16	8.45E-16	8.68E-16	8.89E-16
9.66E-16	9.41E-16	9.24E-16	9.64E-16	9.23E-16	8.43E-16	8.78E-16	8.27E-16	8.04E-16	8.13E-16	8.26E-16	8.27E-16	8.21E-16	8.25E-16	8.15E-16
9.12E-16	9.12E-16	8.53E-16	9.04E-16	8.37E-16	8.35E-16	8.70E-16	8.13E-16	8.06E-16	7.84E-16	7.89E-16	7.80E-16	8.33E-16	8.36E-16	7.59E-16
8.59E-16	8.84E-16	7.90E-16	8.34E-16	7.67E-16	8.28E-16	8.38E-16	8.02E-16	8.05E-16	7.50E-16	7.59E-16	7.53E-16	8.78E-16	8.51E-16	7.42E-16
7.71E-16	8.12E-16	7.39E-16	7.89E-16	7.34E-16	7.94E-16	7.84E-16	7.82E-16	7.40E-16	7.00E-16	7.12E-16	7.16E-16	8.56E-16	8.01E-16	7.05E-16
7.01E-16	7.39E-16	7.02E-16	7.50E-16	7.02E-16	7.31E-16	7.41E-16	7.40E-16	6.68E-16	6.56E-16	6.72E-16	6.45E-16	7.47E-16	6.93E-16	6.46E-16
6.59E-16	6.98E-16	6.85E-16	7.00E-16	6.34E-16	6.61E-16	6.98E-16	6.81E-16	6.19E-16	6.31E-16	6.21E-16	6.02E-16	6.34E-16	5.99E-16	6.34E-16
6.34E-16	6.81E-16	6.77E-16	6.22E-16	5.82E-16	5.96E-16	6.37E-16	6.64E-16	5.94E-16	5.81E-16	5.83E-16	5.63E-16	5.49E-16	5.40E-16	5.92E-16
6.17E-16	6.75E-16	6.48E-16	5.52E-16	5.31E-16	5.34E-16	5.93E-16	6.16E-16	5.72E-16	5.62E-16	5.47E-16	5.34E-16	4.89E-16	5.30E-16	5.37E-16

5.55E-16	6.20E-16	6.02E-16	4.93E-16	4.99E-16	5.13E-16	5.43E-16	5.64E-16	5.49E-16	5.44E-16	5.17E-16	4.98E-16	4.55E-16	5.01E-16	4.53E-16
4.87E-16	5.25E-16	5.70E-16	4.55E-16	4.84E-16	5.03E-16	4.99E-16	5.22E-16	4.96E-16	4.71E-16	4.77E-16	4.55E-16	4.23E-16	4.43E-16	4.15E-16
4.55E-16	4.57E-16	4.89E-16	4.45E-16	4.71E-16	4.55E-16	4.64E-16	4.88E-16	4.47E-16	4.20E-16	4.24E-16	4.06E-16	3.83E-16	4.01E-16	3.81E-16
4.27E-16	4.34E-16	4.38E-16	4.18E-16	4.39E-16	4.36E-16	4.29E-16	4.32E-16	3.92E-16	3.72E-16	3.78E-16	3.46E-16	3.33E-16	3.38E-16	3.41E-16
3.74E-16	4.00E-16	4.21E-16	3.95E-16	4.04E-16	4.10E-16	3.74E-16	3.63E-16	3.50E-16	3.42E-16	3.53E-16	3.17E-16	3.00E-16	2.87E-16	3.14E-16
3.39E-16	3.58E-16	3.92E-16	3.70E-16	3.79E-16	3.55E-16	3.23E-16	3.19E-16	3.16E-16	3.01E-16	3.33E-16	2.88E-16	2.86E-16	2.70E-16	2.93E-16
3.10E-16	3.19E-16	3.58E-16	3.32E-16	3.66E-16	3.16E-16	2.91E-16	2.73E-16	2.75E-16	2.65E-16	3.03E-16	2.72E-16	2.63E-16	2.46E-16	2.71E-16
2.98E-16	2.84E-16	3.15E-16	2.90E-16	3.24E-16	2.79E-16	2.56E-16	2.46E-16	2.51E-16	2.43E-16	2.83E-16	2.53E-16	2.45E-16	2.18E-16	2.50E-16
2.82E-16	2.74E-16	2.76E-16	2.64E-16	2.60E-16	2.65E-16	2.37E-16	2.12E-16	2.39E-16	2.28E-16	2.58E-16	2.23E-16	2.28E-16	2.00E-16	2.21E-16
2.64E-16	2.47E-16	2.56E-16	2.47E-16	2.42E-16	2.78E-16	2.37E-16	1.94E-16	2.19E-16	2.13E-16	2.29E-16	2.02E-16	2.22E-16	1.88E-16	2.10E-16
2.42E-16	2.27E-16	2.44E-16	2.52E-16	2.65E-16	2.91E-16	2.41E-16	1.89E-16	2.08E-16	2.18E-16	2.22E-16	2.06E-16	2.20E-16	1.90E-16	2.17E-16
1.93E-16	1.84E-16	2.13E-16	2.43E-16	2.63E-16	2.49E-16	2.13E-16	1.69E-16	1.70E-16	1.88E-16	1.84E-16	1.64E-16	1.72E-16	1.57E-16	1.83E-16
1.66E-16	1.46E-16	1.75E-16	2.00E-16	2.67E-16	2.34E-16	2.02E-16	1.56E-16	1.45E-16	1.69E-16	1.58E-16	1.33E-16	1.46E-16	1.27E-16	1.39E-16
1.41E-16	1.23E-16	1.43E-16	1.66E-16	2.28E-16	2.16E-16	1.90E-16	1.43E-16	1.31E-16	1.64E-16	1.36E-16	1.13E-16	1.26E-16	1.12E-16	1.05E-16
1.36E-16	1.17E-16	1.28E-16	1.30E-16	1.48E-16	1.83E-16	1.54E-16	1.22E-16	1.16E-16	1.44E-16	1.18E-16	9.31E-17	1.09E-16	9.21E-17	8.34E-17
1.37E-16	1.09E-16	1.20E-16	1.04E-16	1.17E-16	1.27E-16	1.14E-16	9.82E-17	9.49E-17	1.09E-16	9.27E-17	8.09E-17	9.34E-17	7.50E-17	6.23E-17
1.04E-16	8.33E-17	1.01E-16	9.28E-17	9.06E-17	9.05E-17	8.05E-17	8.11E-17	7.91E-17	8.01E-17	6.93E-17	6.62E-17	8.27E-17	6.21E-17	5.32E-17
7.25E-17	5.79E-17	7.31E-17	7.60E-17	6.91E-17	7.13E-17	6.50E-17	6.68E-17	6.68E-17	6.02E-17	5.21E-17	5.14E-17	6.40E-17	4.95E-17	4.39E-17
5.14E-17	4.52E-17	5.22E-17	5.92E-17	5.17E-17	5.49E-17	5.99E-17	5.40E-17	6.13E-17	4.48E-17	4.15E-17	3.86E-17	4.72E-17	3.99E-17	3.45E-17
4.73E-17	3.66E-17	3.81E-17	4.38E-17	4.07E-17	4.25E-17	5.08E-17	4.78E-17	5.35E-17	2.92E-17	3.01E-17	3.11E-17	3.38E-17	3.12E-17	3.04E-17
4.13E-17	2.79E-17	2.84E-17	2.95E-17	2.89E-17	3.07E-17	3.69E-17	4.06E-17	4.17E-17	2.27E-17	2.47E-17	2.23E-17	2.63E-17	3.68E-17	2.94E-17
3.23E-17	1.91E-17	1.98E-17	1.76E-17	2.30E-17	2.93E-17	3.18E-17	3.28E-17	2.91E-17	2.65E-17	2.46E-17	1.63E-17	1.98E-17	3.85E-17	2.14E-17
2.52E-17	1.58E-17	1.60E-17	1.44E-17	2.06E-17	2.19E-17	2.50E-17	2.60E-17	1.96E-17	2.60E-17	2.25E-17	1.07E-17	1.25E-17	2.68E-17	1.31E-17
1.44E-17	1.36E-17	1.72E-17	1.64E-17	1.55E-17	1.71E-17	1.94E-17	1.44E-17	1.18E-17	1.63E-17	1.17E-17	7.05E-18	6.22E-18	1.17E-17	7.26E-18
7.71E-18	1.04E-17	1.62E-17	1.21E-17	1.03E-17	1.32E-17	1.33E-17	5.85E-18	5.47E-18	6.66E-18	5.44E-18	4.78E-18	4.70E-18	6.01E-18	3.58E-18
4.19E-18	6.34E-18	1.31E-17	6.99E-18	5.91E-18	8.53E-18	8.09E-18	2.88E-18	2.32E-18	3.18E-18	3.34E-18	2.43E-18	2.31E-18	4.04E-18	9.91E-19
2.06E-18	1.64E-18	6.13E-18	3.59E-18	4.17E-18	4.57E-18	4.30E-18	9.01E-19	4.26E-19	1.82E-18	2.63E-18	7.23E-19	1.35E-19	1.55E-18	8.82E-20
2.76E-20	2.96E-21	1.44E-18	5.99E-19	1.09E-18	3.23E-19	3.09E-19	1.20E-19	7.10E-20	8.37E-20	4.35E-19	2.83E-20	6.65E-19	4.70E-20	1.19E-18
6.08E-19	2.57E-19	1.33E-19	3.98E-20	2.70E-20	1.31E-19	2.84E-20	6.55E-19	9.36E-19	1.44E-20	7.25E-20	7.46E-19	1.69E-18	3.80E-19	2.94E-18
2.43E-18	8.53E-19	1.26E-19	3.07E-19	1.40E-18	1.69E-18	6.54E-19	1.56E-18	1.63E-18	8.78E-20	7.73E-19	1.67E-18	2.44E-18	1.69E-18	7.26E-18
5.16E-18	1.56E-18	1.19E-18	1.77E-18	3.14E-18	5.83E-18	3.36E-18	3.81E-18	1.75E-18	9.96E-19	2.32E-18	2.64E-18	4.26E-18	5.02E-18	1.20E-17
6.09E-18	2.36E-18	3.79E-18	4.14E-18	5.63E-18	8.48E-18	8.23E-18	7.47E-18	3.70E-18	2.32E-18	3.48E-18	4.35E-18	8.95E-18	1.09E-17	1.24E-17
1.30E-17	5.50E-18	9.43E-18	9.55E-18	8.14E-18	1.19E-17	1.44E-17	1.03E-17	6.47E-18	4.39E-18	5.22E-18	8.35E-18	1.41E-17	1.43E-17	9.80E-18
2.23E-17	1.20E-17	1.42E-17	1.41E-17	9.47E-18	1.34E-17	1.98E-17	1.07E-17	8.22E-18	7.29E-18	1.16E-17	1.81E-17	2.18E-17	1.98E-17	8.87E-18
2.80E-17	1.92E-17	2.17E-17	1.66E-17	1.76E-17	2.06E-17	2.31E-17	1.02E-17	1.25E-17	1.53E-17	2.28E-17	2.97E-17	3.04E-17	2.46E-17	1.35E-17
3.48E-17	2.81E-17	3.26E-17	2.48E-17	2.54E-17	3.09E-17	3.35E-17	1.99E-17	2.02E-17	2.96E-17	3.47E-17	4.14E-17	3.56E-17	3.27E-17	2.15E-17
4.42E-17	3.97E-17	4.54E-17	3.93E-17	3.81E-17	4.64E-17	4.69E-17	3.52E-17	3.65E-17	4.76E-17	4.75E-17	5.00E-17	3.72E-17	3.32E-17	2.39E-17
5.73E-17	5.20E-17	5.38E-17	6.13E-17	5.90E-17	6.22E-17	6.02E-17	5.15E-17	5.02E-17	6.05E-17	5.83E-17	6.01E-17	4.94E-17	3.47E-17	3.02E-17
7.72E-17	6.52E-17	7.71E-17	8.17E-17	8.56E-17	7.84E-17	7.15E-17	7.10E-17	6.62E-17	7.58E-17	7.16E-17	7.32E-17	6.23E-17	3.99E-17	3.67E-17
9.14E-17	7.90E-17	9.38E-17	9.60E-17	1.00E-16	1.01E-16	9.13E-17	9.79E-17	7.98E-17	8.74E-17	9.21E-17	8.54E-17	6.66E-17	4.89E-17	4.77E-17
1.01E-16	1.02E-16	1.01E-16	1.09E-16	1.09E-16	1.27E-16	1.19E-16	1.13E-16	9.73E-17	1.04E-16	1.09E-16	9.94E-17	7.69E-17	6.32E-17	6.70E-17
1.21E-16	1.16E-16	1.22E-16	1.21E-16	1.14E-16	1.43E-16	1.37E-16	1.25E-16	1.16E-16	1.21E-16	1.28E-16	1.19E-16	9.57E-17	7.88E-17	8.94E-17
1.42E-16	1.39E-16	1.43E-16	1.26E-16	1.32E-16	1.61E-16	1.54E-16	1.33E-16	1.27E-16	1.53E-16	1.39E-16	1.39E-16	1.11E-16	9.95E-17	1.18E-16
1.64E-16	1.56E-16	1.66E-16	1.50E-16	1.52E-16	1.76E-16	1.62E-16	1.38E-16	1.49E-16	1.66E-16	1.57E-16	1.65E-16	1.43E-16	1.45E-16	1.41E-16
1.97E-16	1.82E-16	1.99E-16	1.77E-16	1.71E-16	1.84E-16	1.66E-16	1.48E-16	1.63E-16	1.78E-16	1.77E-16	1.83E-16	1.70E-16	1.80E-16	1.69E-16
2.34E-16	2.12E-16	2.32E-16	2.19E-16	2.11E-16	2.12E-16	1.86E-16	1.66E-16	1.85E-16	2.06E-16	1.97E-16	2.06E-16	1.97E-16	2.00E-16	2.01E-16
2.58E-16	2.40E-16	2.53E-16	2.51E-16	2.51E-16	2.46E-16	2.33E-16	2.02E-16	2.26E-16	2.33E-16	2.14E-16	2.26E-16	2.14E-16	2.26E-16	2.25E-16
2.66E-16	2.57E-16	2.77E-16	2.82E-16	2.90E-16	2.97E-16	2.73E-16	2.55E-16	2.53E-16	2.63E-16	2.28E-16	2.46E-16	2.34E-16	2.56E-16	2.56E-16
2.86E-16	2.81E-16	2.96E-16	3.09E-16	3.35E-16	3.21E-16	2.82E-16	2.93E-16	2.88E-16	2.85E-16	2.54E-16	2.81E-16	2.43E-16	2.70E-16	2.66E-16
3.16E-16	3.13E-16	3.25E-16	3.30E-16	3.39E-16	3.38E-16	2.93E-16	3.24E-16	3.04E-16	3.00E-16	2.91E-16	3.29E-16	2.71E-16	2.79E-16	2.70E-16
3.24E-16	3.39E-16	3.37E-16	3.46E-16	3.41E-16	3.55E-16	3.45E-16	3.63E-16	3.27E-16	3.34E-16	3.37E-16	3.56E-16	3.00E-16	2.92E-16	2.87E-16
3.51E-16	3.58E-16	3.61E-16	3.67E-16	3.58E-16	3.70E-16	3.83E-16	3.83E-16	3.52E-16	3.81E-16	3.63E-16	3.69E-16	3.22E-16	3.09E-16	3.17E-16
3.87E-16	3.92E-16	4.02E-16	3.84E-16	3.68E-16	3.79E-16	3.95E-16	4.09E-16	3.87E-16	4.16E-16	3.77E-16	3.91E-16	3.65E-16	3.40E-16	3.53E-16
4.17E-16	4.39E-16	4.50E-16	4.08E-16	3.77E-16	3.97E-16	4.10E-16	4.37E-16	4.26E-16	4.40E-16	4.00E-16	4.30E-16	4.21E-16	3.86E-16	3.84E-16
4.65E-16	4.78E-16	4.86E-16	4.39E-16	4.29E-16	4.36E-16	4.31E-16	4.76E-16	4.65E-16	4.74E-16	4.59E-16	4.66E-16	4.54E-16	4.15E-16	4.02E-16
5.07E-16	4.88E-16	4.91E-16	4.80E-16	4.81E-16	4.59E-16	4.79E-16	5.21E-16	5.12E-16	4.96E-16	4.79E-16	4.89E-16	4.49E-16	4.14E-16	4.22E-16
5.30E-16	5.00E-16	5.14E-16	5.20E-16	5.23E-16	4.97E-16	5.20E-16	5.45E-16	5.39E-16	5.17E-16	4.97E-16	5.13E-16	4.46E-16	4.13E-16	4.43E-16
5.61E-16	5.43E-16	5.59E-16	5.68E-16	5.62E-16	5.45E-16	5.47E-16	5.54E-16	5.52E-16	5.44E-16	5.37E-16	5.34E-16	4.74E-16	4.62E-16	4.81E-16
5.92E-16	5.97E-16	6.02E-16	6.16E-16	5.82E-16	5.69E-16	5.53E-16	5.45E-16	5.64E-16	5.63E-16	5.55E-16	5.59E-16	5.28E-16	5.07E-16	5.21E-16
6.11E-16	6.19E-16	6.30E-16	6.41E-16	6.05E-16	5.87E-16	5.65E-16	5.50E-16	5.74E-16	5.67E-16	5.70E-16	5.88E-16	5.82E-16	5.43E-16	5.30E-16
6.25E-16	6.48E-16	6.58E-16	6.72E-16	6.31E-16	6.16E-16	5.99E-16	6.09E-16	6.07E-16	5.86E-16	5.91E-16	5.97E-16	6.21E-16	5.69E-16	5.57E-16
6.51E-16	6.80E-16	6.95E-16	7.07E-16	6.72E-16	6.56E-16	6.55E-16	6.66E-16	6.45E-16	6.27E-16	6.17E-16	6.17E-16	6.35E-16	5.95E-16	6.07E-16
6.94E-16	7.01E-16	7.13E-16	7.19E-16	7.11E-16	7.09E-16	7.08E-16	7.17E-16	6.78E-16	6.62E-16	6.37E-16	6.38E-16	6.58E-16	6.14E-16	6.32E-16
7.39E-16	7.31E-16	7.11E-16	7.13E-16	7.37E-16	7.44E-16	7.55E-16	7.68E-16	7.26E-16	6.99E-16	6.73E-16	6.57E-16	6.84E-16	6.34E-16	6.63E-16
7.89E-16	7.80E-16	7.39E-16	7.25E-16	7.59E-16	7.65E-16	7.90E-16	8.04E-16	7.69E-16	7.60E-16	7.42E-16	7.09E-16	7.34E-16	6.65E-16	6.95E-16
8.31E-16	8.28E-16	7.89E-16	7.68E-16	8.02E-16	7.98E-16	8.23E-16	8.37E-16	8.12E-16	8.02E-16	7.77E-16	7.42E-16	7.54E-16	6.92E-16	6.84E-16
8.55E-16	8.55E-16	8.37E-16	8.24E-16	8.58E-16	8.39E-16	8.69E-16	8.66E-16	8.40E-16	8.12E-16	7.89E-16	7.48E-16	7.48E-16	6.99E-16	6.94E-16
8.78E-16	8.83E-16	8.85E-16	8.70E-16	9.17E-16	8.95E-16	8.95E-16	8.81E-16	8.49E-16	8.19E-16	8.03E-16	7.60E-16	7.55E-16	7.06E-16	7.27E-16
8.95E-16	9.02E-16	9.31E-16	9.15E-16	9.46E-16	9.17E-16	8.92E-16	8.70E-16	8.60E-16	8.57E-16	8.29E-16	7.71E-16	7.54E-16	7.25E-16	7.67E-16
9.38E-16	9.35E-16	9.50E-16	9.32E-16	9.49E-16	9.24E-16	8.88E-16	8.66E-16	8.52E-16	8.72E-16	8.41E-16	7.77E-16	7.48E-16	7.50E-16	8.11E-16
9.83E-16	9.59E-16	9.64E-16	9.49E-16	9.46E-16	9.30E-16	8.88E-16	8.73E-16	8.51E-16	8.76E-16	8.44E-16	7.70E-16	7.38E-16	7.52E-16	8.28E-16
1.01E-15	9.73E-16	9.62E-16	9.57E-16	9.57E-16	9.53E-16	9.11E-16	8.83E-16	8.63E-16	8.86E-16	8.48E-16	7.85E-16	7.62E-16	7.51E-16	8.34E-16
1.05E-15	9.93E-16	9.75E-16	9.63E-16	9.66E-16	9.93E-16	9.72E-16	9.36E-16	9.15E-16	9.13E-16	8.58E-16	8.16E-16	8.18E-16	8.01E-16	8.46E-16
1.08E-15	1.00E-15	9.97E-16	9.83E-16	9.78E-16	1.03E-15	1.01E-15	9.90E-16	9.73E-16	9.68E-16	9.01E-16	8.68E-16	8.82E-16	8.60E-16	8.63E-16
1.10E-15	1.02E-15	1.01E-15	1.02E-15	1.00E-15	1.06E-15	1.03E-15	1.03E-15	1.00E-15	1.00E-15	9.32E-16	9.01E-16	9.42E-16	9.27E-16	8.83E-16
1.13E-15	1.03E-15	1.03E-15	1.05E-15	1.06E-15	1.08E-15	1.07E-15	1.08E-15	1.04E-15	1.03E-15	9.52E-16	9.06E-16	9.51E-16	9.65E-16	9.16E-16
1.16E-15	1.09E-15	1.08E-15	1.10E-15	1.11E-15	1.12E-15	1.10E-15	1.11E-15	1.07E-15	1.05E-15	9.66E-16	9.34E-16	9.49E-16	9.87E-16	9.72E-16
1.19E-15	1.15E-15	1.12E-15	1.14E-15	1.16E-15	1.14E-15	1.13E-15	1.11E-15	1.09E-15	1.08E-15	9.80E-16	9.90E-16	9.74E-16	1.03E-15	1.03E-15
1.21E-15	1.17E-15	1.14E-15	1.16E-15	1.21E-15	1.16E-15	1.15E-15	1.11E-15	1.07E-15	1.08E-15	1.03E-15	1.02E-15	1.01E-15	1.06E-15	1.08E-15
1.23E-15	1.18E-15	1.18E-15	1.21E-15	1.20E-15	1.15E-15	1.16E-15	1.11E-15	1.08E-15	1.08E-15	1.05E-15	1.07E-15	1.03E-15	1.11E-15	1.08E-15
1.25E-15	1.22E-15	1.22E-15	1.24E-15	1.19E-15	1.13E-15	1.17E-15	1.13E-15	1.15E-15	1.13E-15	1.09E-15	1.12E-15	1.07E-15	1.13E-15	1.09E-15
1.30E-15	1.26E-15	1.23E-15	1.25E-15	1.21E-15	1.16E-15	1.17E-15	1.13E-15	1.19E-15	1.18E-15	1.13E-15	1.16E-15	1.13E-15	1.17E-15	1.09E-15
1.36E-15	1.33E-15	1.26E-15	1.28E-15	1.23E-15	1.21E-15	1.20E-15	1.19E-15	1.23E-15	1.23E-15	1.16E-15	1.19E-15	1.17E-15	1.19E-15	1.11E-15
1.37E-15	1.35E-15	1.31E-15	1.28E-15	1.24E-15	1.24E-15	1.27E-15	1.25E-15	1.26E-15	1.25E-15	1.16E-15	1.19E-15	1.19E-15	1.22E-15	1.13E-15
1.36E-15	1.36E-15	1.31E-15	1.30E-15	1.23E-15	1.27E-15	1.29E-15	1.27E-15	1.28E-15	1.25E-15	1.16E-15	1.21E-15	1.20E-15	1.23E-15	1.11E-15
1.35E-15	1.39E-15	1.33E-15	1.34E-15	1.26E-15	1.30E-15	1.28E-15	1.26E-15	1.27E-15	1.27E-15	1.18E-15	1.20E-15	1.21E-15	1.24E-15	1.13E-15

1.36E-15	1.40E-15	1.34E-15	1.38E-15	1.29E-15	1.30E-15	1.28E-15	1.26E-15	1.25E-15	1.25E-15	1.19E-15	1.20E-15	1.20E-15	1.27E-15	1.15E-15
1.39E-15	1.41E-15	1.34E-15	1.37E-15	1.27E-15	1.27E-15	1.28E-15	1.28E-15	1.24E-15	1.24E-15	1.20E-15	1.22E-15	1.22E-15	1.30E-15	1.17E-15
1.40E-15	1.41E-15	1.34E-15	1.34E-15	1.31E-15	1.27E-15	1.30E-15	1.26E-15	1.19E-15	1.18E-15	1.17E-15	1.17E-15	1.22E-15	1.27E-15	1.15E-15
1.40E-15	1.42E-15	1.34E-15	1.35E-15	1.33E-15	1.30E-15	1.33E-15	1.23E-15	1.14E-15	1.13E-15	1.14E-15	1.14E-15	1.21E-15	1.23E-15	1.16E-15
1.41E-15	1.45E-15	1.33E-15	1.34E-15	1.33E-15	1.31E-15	1.35E-15	1.21E-15	1.14E-15	1.10E-15	1.14E-15	1.14E-15	1.24E-15	1.21E-15	1.16E-15
1.39E-15	1.47E-15	1.34E-15	1.33E-15	1.34E-15	1.36E-15	1.36E-15	1.24E-15	1.16E-15	1.09E-15	1.17E-15	1.19E-15	1.26E-15	1.23E-15	1.16E-15
1.42E-15	1.49E-15	1.35E-15	1.34E-15	1.36E-15	1.40E-15	1.37E-15	1.27E-15	1.23E-15	1.18E-15	1.22E-15	1.22E-15	1.24E-15	1.26E-15	1.17E-15
1.42E-15	1.48E-15	1.38E-15	1.38E-15	1.32E-15	1.38E-15	1.33E-15	1.27E-15	1.25E-15	1.25E-15	1.28E-15	1.27E-15	1.20E-15	1.24E-15	1.18E-15
1.39E-15	1.43E-15	1.36E-15	1.36E-15	1.33E-15	1.34E-15	1.29E-15	1.25E-15	1.25E-15	1.26E-15	1.29E-15	1.28E-15	1.16E-15	1.19E-15	1.17E-15
1.40E-15	1.40E-15	1.34E-15	1.32E-15	1.31E-15	1.29E-15	1.28E-15	1.22E-15	1.24E-15	1.29E-15	1.25E-15	1.25E-15	1.15E-15	1.17E-15	1.13E-15
1.37E-15	1.37E-15	1.30E-15	1.28E-15	1.27E-15	1.25E-15	1.27E-15	1.24E-15	1.23E-15	1.27E-15	1.25E-15	1.25E-15	1.18E-15	1.14E-15	1.11E-15
1.08E-13	1.06E-13	1.04E-13	1.04E-13	1.02E-13	1.02E-13	1.02E-13	1.01E-13	9.93E-14	9.94E-14	9.69E-14	9.73E-14	9.56E-14	9.46E-14	9.31E-14

Total Values:

RMS Quadrant 1	RMS Quadrant 2	RMS Quadrant 3	RMS Quadrant 4
2.2164E-08	9.4455E-09	1.9207E-08	1.32E-08

**RMS AVG**	1.600E-08
**STDEV**	5.747E-09

5.57E-08	5.59E-08	5.63E-08	5.64E-08	5.68E-08	5.83E-08	5.55E-08	5.43E-08	5.28E-08	5.40E-08	5.37E-08	5.49E-08	5.60E-08	5.48E-08	5.36E-08
5.45E-08	5.47E-08	5.57E-08	5.56E-08	5.51E-08	5.65E-08	5.42E-08	5.37E-08	5.17E-08	5.25E-08	5.27E-08	5.39E-08	5.45E-08	5.33E-08	5.27E-08
5.38E-08	5.42E-08	5.54E-08	5.46E-08	5.38E-08	5.42E-08	5.34E-08	5.30E-08	5.03E-08	5.15E-08	5.13E-08	5.26E-08	5.25E-08	5.15E-08	5.14E-08
5.30E-08	5.32E-08	5.42E-08	5.29E-08	5.25E-08	5.17E-08	5.20E-08	5.19E-08	4.90E-08	5.06E-08	4.98E-08	5.01E-08	5.03E-08	4.98E-08	4.99E-08
5.22E-08	5.28E-08	5.18E-08	5.08E-08	5.06E-08	4.99E-08	4.96E-08	5.04E-08	4.85E-08	4.94E-08	4.89E-08	4.86E-08	4.89E-08	4.85E-08	4.86E-08
5.09E-08	5.16E-08	4.97E-08	4.88E-08	4.91E-08	4.86E-08	4.79E-08	4.85E-08	4.81E-08	4.86E-08	4.86E-08	4.74E-08	4.80E-08	4.72E-08	4.73E-08
4.82E-08	4.90E-08	4.77E-08	4.73E-08	4.79E-08	4.73E-08	4.63E-08	4.68E-08	4.69E-08	4.79E-08	4.85E-08	4.62E-08	4.65E-08	4.57E-08	4.59E-08
4.58E-08	4.62E-08	4.60E-08	4.60E-08	4.67E-08	4.56E-08	4.49E-08	4.51E-08	4.55E-08	4.68E-08	4.75E-08	4.50E-08	4.47E-08	4.44E-08	4.44E-08
4.41E-08	4.47E-08	4.48E-08	4.45E-08	4.53E-08	4.38E-08	4.35E-08	4.37E-08	4.41E-08	4.50E-08	4.52E-08	4.37E-08	4.32E-08	4.28E-08	4.27E-08
4.27E-08	4.37E-08	4.35E-08	4.30E-08	4.39E-08	4.30E-08	4.23E-08	4.21E-08	4.20E-08	4.34E-08	4.33E-08	4.23E-08	4.21E-08	4.14E-08	4.16E-08
4.22E-08	4.26E-08	4.25E-08	4.22E-08	4.25E-08	4.28E-08	4.13E-08	4.10E-08	4.07E-08	4.13E-08	4.15E-08	4.11E-08	4.13E-08	4.02E-08	4.05E-08
4.14E-08	4.10E-08	4.14E-08	4.10E-08	4.13E-08	4.12E-08	4.06E-08	4.01E-08	3.97E-08	4.00E-08	4.03E-08	4.01E-08	4.07E-08	3.89E-08	3.91E-08
3.99E-08	4.00E-08	4.05E-08	4.00E-08	3.98E-08	3.96E-08	3.97E-08	3.92E-08	3.90E-08	3.93E-08	3.98E-08	3.92E-08	3.93E-08	3.78E-08	3.73E-08
3.86E-08	3.90E-08	3.89E-08	3.92E-08	3.82E-08	3.84E-08	3.87E-08	3.81E-08	3.84E-08	3.86E-08	3.88E-08	3.79E-08	3.77E-08	3.66E-08	3.59E-08
3.75E-08	3.78E-08	3.73E-08	3.80E-08	3.68E-08	3.67E-08	3.74E-08	3.71E-08	3.77E-08	3.73E-08	3.78E-08	3.70E-08	3.67E-08	3.56E-08	3.45E-08
3.66E-08	3.59E-08	3.58E-08	3.67E-08	3.58E-08	3.55E-08	3.64E-08	3.62E-08	3.63E-08	3.59E-08	3.68E-08	3.62E-08	3.53E-08	3.50E-08	3.38E-08
3.58E-08	3.43E-08	3.45E-08	3.56E-08	3.48E-08	3.45E-08	3.51E-08	3.49E-08	3.53E-08	3.50E-08	3.59E-08	3.48E-08	3.40E-08	3.43E-08	3.31E-08
3.40E-08	3.27E-08	3.30E-08	3.42E-08	3.36E-08	3.36E-08	3.40E-08	3.32E-08	3.32E-08	3.39E-08	3.43E-08	3.39E-08	3.32E-08	3.35E-08	3.30E-08
3.28E-08	3.17E-08	3.23E-08	3.28E-08	3.32E-08	3.31E-08	3.33E-08	3.23E-08	3.22E-08	3.30E-08	3.34E-08	3.34E-08	3.27E-08	3.28E-08	3.25E-08
3.25E-08	3.12E-08	3.21E-08	3.23E-08	3.28E-08	3.26E-08	3.25E-08	3.17E-08	3.21E-08	3.33E-08	3.29E-08	3.30E-08	3.19E-08	3.21E-08	3.15E-08
3.19E-08	3.05E-08	3.14E-08	3.15E-08	3.19E-08	3.20E-08	3.15E-08	3.13E-08	3.14E-08	3.26E-08	3.14E-08	3.15E-08	3.07E-08	3.07E-08	3.00E-08
3.09E-08	2.95E-08	3.03E-08	3.00E-08	3.07E-08	3.05E-08	3.05E-08	3.05E-08	3.02E-08	3.01E-08	2.95E-08	2.93E-08	2.91E-08	2.88E-08	2.87E-08
2.95E-08	2.90E-08	2.91E-08	2.86E-08	2.95E-08	2.87E-08	2.95E-08	2.88E-08	2.84E-08	2.83E-08	2.82E-08	2.81E-08	2.77E-08	2.72E-08	2.76E-08
2.82E-08	2.84E-08	2.77E-08	2.76E-08	2.81E-08	2.75E-08	2.83E-08	2.73E-08	2.71E-08	2.69E-08	2.71E-08	2.71E-08	2.65E-08	2.63E-08	2.66E-08
2.71E-08	2.72E-08	2.68E-08	2.69E-08	2.74E-08	2.68E-08	2.69E-08	2.64E-08	2.66E-08	2.61E-08	2.60E-08	2.60E-08	2.55E-08	2.54E-08	2.52E-08
2.63E-08	2.66E-08	2.62E-08	2.62E-08	2.68E-08	2.63E-08	2.58E-08	2.56E-08	2.63E-08	2.54E-08	2.53E-08	2.50E-08	2.46E-08	2.42E-08	2.41E-08
2.56E-08	2.57E-08	2.54E-08	2.56E-08	2.60E-08	2.58E-08	2.50E-08	2.50E-08	2.63E-08	2.43E-08	2.49E-08	2.44E-08	2.41E-08	2.36E-08	2.36E-08
2.49E-08	2.44E-08	2.47E-08	2.44E-08	2.48E-08	2.41E-08	2.41E-08	2.43E-08	2.55E-08	2.39E-08	2.36E-08	2.39E-08	2.35E-08	2.32E-08	2.33E-08
2.42E-08	2.36E-08	2.35E-08	2.32E-08	2.31E-08	2.25E-08	2.30E-08	2.36E-08	2.45E-08	2.33E-08	2.28E-08	2.30E-08	2.23E-08	2.23E-08	2.25E-08
2.29E-08	2.28E-08	2.23E-08	2.17E-08	2.18E-08	2.17E-08	2.22E-08	2.30E-08	2.37E-08	2.25E-08	2.22E-08	2.24E-08	2.14E-08	2.14E-08	2.20E-08
2.24E-08	2.22E-08	2.17E-08	2.04E-08	2.12E-08	2.11E-08	2.14E-08	2.19E-08	2.20E-08	2.13E-08	2.10E-08	2.14E-08	2.09E-08	2.07E-08	2.10E-08
2.13E-08	2.16E-08	2.08E-08	1.95E-08	2.04E-08	2.01E-08	2.09E-08	2.05E-08	2.07E-08	2.01E-08	1.98E-08	2.07E-08	2.04E-08	2.05E-08	2.04E-08
1.96E-08	2.06E-08	1.97E-08	1.91E-08	1.92E-08	1.93E-08	2.00E-08	1.95E-08	1.95E-08	1.91E-08	1.92E-08	2.03E-08	1.95E-08	1.97E-08	1.89E-08
1.87E-08	2.03E-08	1.93E-08	1.90E-08	1.84E-08	1.89E-08	1.88E-08	1.82E-08	1.84E-08	1.84E-08	1.89E-08	1.92E-08	1.92E-08	1.90E-08	1.80E-08
1.79E-08	1.94E-08	1.88E-08	1.88E-08	1.73E-08	1.79E-08	1.76E-08	1.75E-08	1.75E-08	1.79E-08	1.84E-08	1.78E-08	1.88E-08	1.82E-08	1.70E-08
1.73E-08	1.80E-08	1.79E-08	1.76E-08	1.65E-08	1.70E-08	1.67E-08	1.70E-08	1.67E-08	1.70E-08	1.71E-08	1.74E-08	1.86E-08	1.69E-08	1.59E-08
1.66E-08	1.66E-08	1.72E-08	1.69E-08	1.60E-08	1.65E-08	1.58E-08	1.64E-08	1.57E-08	1.60E-08	1.58E-08	1.68E-08	1.73E-08	1.61E-08	1.51E-08
1.62E-08	1.57E-08	1.60E-08	1.62E-08	1.54E-08	1.57E-08	1.52E-08	1.54E-08	1.47E-08	1.51E-08	1.46E-08	1.54E-08	1.60E-08	1.57E-08	1.47E-08
1.55E-08	1.42E-08	1.46E-08	1.53E-08	1.46E-08	1.45E-08	1.38E-08	1.43E-08	1.38E-08	1.39E-08	1.32E-08	1.40E-08	1.48E-08	1.53E-08	1.44E-08
1.45E-08	1.35E-08	1.30E-08	1.39E-08	1.38E-08	1.34E-08	1.30E-08	1.28E-08	1.31E-08	1.32E-08	1.25E-08	1.27E-08	1.42E-08	1.47E-08	1.39E-08
1.34E-08	1.30E-08	1.21E-08	1.32E-08	1.31E-08	1.29E-08	1.24E-08	1.17E-08	1.24E-08	1.28E-08	1.22E-08	1.14E-08	1.28E-08	1.36E-08	1.32E-08
1.36E-08	1.33E-08	1.24E-08	1.32E-08	1.29E-08	1.25E-08	1.21E-08	1.13E-08	1.23E-08	1.26E-08	1.20E-08	1.13E-08	1.18E-08	1.22E-08	1.22E-08
1.27E-08	1.27E-08	1.16E-08	1.24E-08	1.16E-08	1.13E-08	1.09E-08	1.02E-08	1.15E-08	1.08E-08	1.07E-08	1.01E-08	1.00E-08	1.06E-08	1.04E-08
1.18E-08	1.19E-08	1.11E-08	1.16E-08	1.04E-08	1.02E-08	9.91E-09	9.48E-09	9.62E-09	9.28E-09	8.71E-09	8.99E-09	9.27E-09	9.67E-09	9.01E-09
1.09E-08	1.17E-08	1.15E-08	1.09E-08	9.10E-09	8.93E-09	8.91E-09	9.14E-09	8.32E-09	7.77E-09	7.39E-09	8.23E-09	9.20E-09	9.07E-09	8.10E-09
1.00E-08	1.10E-08	1.08E-08	9.94E-09	8.19E-09	8.27E-09	8.78E-09	8.28E-09	6.86E-09	6.87E-09	6.91E-09	7.68E-09	9.01E-09	8.70E-09	7.55E-09
9.36E-09	1.01E-08	9.64E-09	9.33E-09	7.67E-09	7.77E-09	7.90E-09	7.61E-09	6.09E-09	6.47E-09	6.69E-09	7.77E-09	8.74E-09	8.29E-09	7.39E-09
8.38E-09	9.26E-09	9.57E-09	8.69E-09	7.12E-09	7.57E-09	6.96E-09	7.00E-09	5.81E-09	6.00E-09	6.54E-09	7.47E-09	8.16E-09	7.56E-09	7.11E-09
7.70E-09	8.21E-09	8.80E-09	7.45E-09	6.75E-09	6.75E-09	6.02E-09	6.31E-09	5.53E-09	5.28E-09	6.27E-09	6.74E-09	6.68E-09	6.35E-09	6.15E-09
6.99E-09	6.81E-09	7.72E-09	6.05E-09	5.71E-09	5.91E-09	5.05E-09	5.44E-09	5.07E-09	5.02E-09	5.90E-09	6.23E-09	5.37E-09	4.94E-09	5.26E-09
5.86E-09	5.48E-09	6.21E-09	4.64E-09	4.17E-09	5.06E-09	4.37E-09	4.68E-09	4.94E-09	4.88E-09	5.46E-09	5.33E-09	4.19E-09	3.87E-09	5.02E-09
4.80E-09	4.49E-09	4.78E-09	3.82E-09	3.42E-09	4.07E-09	4.24E-09	4.18E-09	4.88E-09	4.38E-09	4.38E-09	4.81E-09	3.81E-09	3.45E-09	4.65E-09
4.17E-09	3.21E-09	3.52E-09	3.00E-09	3.16E-09	3.10E-09	3.69E-09	3.70E-09	4.38E-09	3.66E-09	3.45E-09	4.04E-09	3.67E-09	3.40E-09	4.29E-09
3.09E-09	1.78E-09	2.94E-09	2.77E-09	3.05E-09	2.43E-09	2.15E-09	2.23E-09	3.23E-09	2.59E-09	3.03E-09	3.54E-09	3.16E-09	2.94E-09	3.50E-09
2.46E-09	1.08E-09	2.33E-09	2.48E-09	2.95E-09	2.10E-09	1.21E-09	9.92E-10	2.10E-09	1.42E-09	2.05E-09	2.63E-09	2.75E-09	1.24E-09	2.38E-09
1.48E-09	5.13E-10	1.32E-09	2.27E-09	3.05E-09	1.57E-09	5.77E-10	3.77E-11	5.31E-10	7.46E-10	1.11E-09	1.47E-09	1.52E-09	5.92E-10	1.23E-09
3.38E-10	1.83E-10	5.95E-10	1.42E-09	2.60E-09	1.06E-09	5.47E-10	-1.50E-10	-1.49E-10	5.65E-11	2.49E-11	-2.29E-10	4.80E-10	2.67E-10	1.68E-10
-5.17E-10	-5.74E-10	7.82E-11	1.27E-10	1.37E-09	1.53E-10	-3.74E-10	-7.22E-10	-6.09E-10	-9.42E-10	-1.39E-09	-2.01E-09	-1.20E-09	-1.45E-09	-1.05E-09
-9.77E-10	-1.02E-09	-7.09E-10	-9.74E-10	-2.40E-10	-1.07E-09	-1.41E-09	-1.19E-09	-1.10E-09	-1.73E-09	-2.65E-09	-2.70E-09	-2.32E-09	-2.62E-09	-1.81E-09
-1.49E-09	-1.90E-09	-1.46E-09	-1.73E-09	-1.27E-09	-1.63E-09	-1.78E-09	-1.98E-09	-2.08E-09	-2.66E-09	-3.46E-09	-3.05E-09	-3.47E-09	-3.46E-09	-2.37E-09
-2.30E-09	-2.69E-09	-2.01E-09	-2.55E-09	-2.28E-09	-2.58E-09	-2.08E-09	-2.71E-09	-3.02E-09	-2.91E-09	-4.11E-09	-3.66E-09	-4.53E-09	-4.11E-09	-3.19E-09
-2.85E-09	-3.11E-09	-3.25E-09	-3.59E-09	-3.31E-09	-3.59E-09	-2.94E-09	-3.71E-09	-3.57E-09	-3.60E-09	-4.75E-09	-4.24E-09	-4.92E-09	-4.29E-09	-2.76E-09
-3.17E-09	-3.46E-09	-4.00E-09	-4.68E-09	-4.36E-09	-4.27E-09	-4.08E-09	-4.32E-09	-3.33E-09	-4.26E-09	-5.46E-09	-5.38E-09	-5.46E-09	-4.67E-09	-3.07E-09
-3.41E-09	-3.75E-09	-4.66E-09	-5.60E-09	-4.77E-09	-5.08E-09	-5.16E-09	-5.04E-09	-3.54E-09	-4.79E-09	-6.22E-09	-6.23E-09	-6.50E-09	-5.17E-09	-4.61E-09
-3.86E-09	-4.51E-09	-5.38E-09	-5.68E-09	-5.16E-09	-5.93E-09	-5.59E-09	-5.45E-09	-4.41E-09	-5.41E-09	-6.68E-09	-6.53E-09	-6.82E-09	-5.89E-09	-5.92E-09
-4.56E-09	-5.10E-09	-5.54E-09	-5.87E-09	-5.60E-09	-6.08E-09	-6.05E-09	-5.85E-09	-5.83E-09	-5.92E-09	-6.78E-09	-6.55E-09	-6.84E-09	-6.31E-09	-7.00E-09
-5.34E-09	-5.62E-09	-5.73E-09	-6.03E-09	-5.94E-09	-5.94E-09	-6.53E-09	-6.52E-09	-7.02E-09	-6.71E-09	-6.86E-09	-6.86E-09	-7.49E-09	-7.29E-09	-6.89E-09
-6.04E-09	-6.01E-09	-5.92E-09	-6.33E-09	-6.20E-09	-5.93E-09	-6.59E-09	-7.04E-09	-7.69E-09	-7.50E-09	-7.43E-09	-7.50E-09	-8.39E-09	-7.72E-09	-7.11E-09
-6.32E-09	-6.14E-09	-5.42E-09	-6.39E-09	-6.81E-09	-6.56E-09	-6.37E-09	-8.02E-09	-8.20E-09	-8.28E-09	-8.12E-09	-8.32E-09	-8.80E-09	-7.80E-09	-7.39E-09
-6.70E-09	-6.72E-09	-6.71E-09	-7.05E-09	-7.09E-09	-7.23E-09	-6.33E-09	-8.79E-09	-8.85E-09	-8.86E-09	-8.77E-09	-8.67E-09	-8.82E-09	-7.72E-09	-7.61E-09
-7.17E-09	-7.04E-09	-7.82E-09	-7.75E-09	-7.74E-09	-8.45E-09	-8.33E-09	-9.49E-09	-9.60E-09	-9.87E-09	-9.53E-09	-9.85E-09	-9.55E-09	-8.23E-09	-7.83E-09
-7.88E-09	-7.67E-09	-7.97E-09	-8.52E-09	-9.22E-09	-9.58E-09	-9.74E-09	-1.07E-08	-1.05E-08	-1.06E-08	-1.03E-08	-1.07E-08	-1.00E-08	-8.29E-09	-7.85E-09
-9.24E-09	-9.10E-09	-8.83E-09	-9.85E-09	-1.07E-08	-1.14E-08	-1.07E-08	-1.16E-08	-1.08E-08	-1.11E-08	-1.08E-08	-1.08E-08	-1.01E-08	-8.63E-09	-8.49E-09
-1.10E-08	-1.09E-08	-1.09E-08	-1.12E-08	-1.18E-08	-1.25E-08	-1.18E-08	-1.20E-08	-1.15E-08	-1.15E-08	-1.10E-08	-1.09E-08	-1.07E-08	-9.67E-09	-1.02E-08
-1.25E-08	-1.21E-08	-1.19E-08	-1.27E-08	-1.23E-08	-1.28E-08	-1.23E-08	-1.20E-08	-1.26E-08	-1.20E-08	-1.13E-08	-1.19E-08	-1.20E-08	-1.10E-08	-1.10E-08
-1.33E-08	-1.27E-08	-1.25E-08	-1.34E-08	-1.27E-08	-1.29E-08	-1.29E-08	-1.23E-08	-1.34E-08	-1.31E-08	-1.26E-08	-1.26E-08	-1.31E-08	-1.21E-08	-1.13E-08
-1.38E-08	-1.34E-08	-1.36E-08	-1.38E-08	-1.34E-08	-1.30E-08	-1.36E-08	-1.28E-08	-1.39E-08	-1.43E-08	-1.40E-08	-1.32E-08	-1.38E-08	-1.27E-08	-1.24E-08
-1.45E-08	-1.45E-08	-1.45E-08	-1.41E-08	-1.39E-08	-1.34E-08	-1.35E-08	-1.34E-08	-1.45E-08	-1.55E-08	-1.54E-08	-1.39E-08	-1.42E-08	-1.33E-08	-1.36E-08

-1.55E-08	-1.56E-08	-1.55E-08	-1.52E-08	-1.54E-08	-1.42E-08	-1.36E-08	-1.39E-08	-1.50E-08	-1.62E-08	-1.63E-08	-1.50E-08	-1.51E-08	-1.41E-08	-1.46E-08
-1.63E-08	-1.64E-08	-1.67E-08	-1.66E-08	-1.63E-08	-1.51E-08	-1.45E-08	-1.51E-08	-1.60E-08	-1.66E-08	-1.66E-08	-1.57E-08	-1.57E-08	-1.46E-08	-1.53E-08
-1.67E-08	-1.69E-08	-1.76E-08	-1.73E-08	-1.67E-08	-1.65E-08	-1.56E-08	-1.66E-08	-1.70E-08	-1.69E-08	-1.70E-08	-1.60E-08	-1.59E-08	-1.54E-08	-1.62E-08
-1.72E-08	-1.74E-08	-1.84E-08	-1.80E-08	-1.72E-08	-1.72E-08	-1.70E-08	-1.73E-08	-1.76E-08	-1.67E-08	-1.70E-08	-1.61E-08	-1.65E-08	-1.64E-08	-1.63E-08
-1.77E-08	-1.80E-08	-1.90E-08	-1.86E-08	-1.74E-08	-1.75E-08	-1.78E-08	-1.78E-08	-1.81E-08	-1.71E-08	-1.67E-08	-1.64E-08	-1.73E-08	-1.68E-08	-1.65E-08
-1.82E-08	-1.89E-08	-1.94E-08	-1.89E-08	-1.80E-08	-1.76E-08	-1.79E-08	-1.81E-08	-1.84E-08	-1.76E-08	-1.65E-08	-1.69E-08	-1.72E-08	-1.69E-08	-1.69E-08
-1.90E-08	-1.98E-08	-1.98E-08	-1.92E-08	-1.91E-08	-1.86E-08	-1.92E-08	-1.85E-08	-1.89E-08	-1.80E-08	-1.66E-08	-1.72E-08	-1.75E-08	-1.75E-08	-1.76E-08
-1.96E-08	-2.04E-08	-2.03E-08	-1.94E-08	-1.99E-08	-1.95E-08	-2.02E-08	-1.91E-08	-1.91E-08	-1.81E-08	-1.71E-08	-1.80E-08	-1.85E-08	-1.84E-08	-1.87E-08
-1.97E-08	-2.07E-08	-2.06E-08	-1.96E-08	-2.01E-08	-1.99E-08	-2.03E-08	-1.95E-08	-1.97E-08	-1.88E-08	-1.78E-08	-1.88E-08	-1.93E-08	-1.87E-08	-1.92E-08
-2.03E-08	-2.12E-08	-2.17E-08	-2.02E-08	-2.07E-08	-2.01E-08	-2.01E-08	-1.96E-08	-1.99E-08	-1.91E-08	-1.85E-08	-1.97E-08	-1.99E-08	-1.86E-08	-1.96E-08
-2.14E-08	-2.21E-08	-2.27E-08	-2.12E-08	-2.14E-08	-2.02E-08	-2.00E-08	-1.95E-08	-2.00E-08	-1.96E-08	-1.96E-08	-2.03E-08	-2.00E-08	-1.91E-08	-1.99E-08
-2.26E-08	-2.28E-08	-2.37E-08	-2.22E-08	-2.23E-08	-2.06E-08	-2.08E-08	-2.07E-08	-1.98E-08	-2.06E-08	-2.10E-08	-2.10E-08	-2.03E-08	-1.98E-08	-2.04E-08
-2.31E-08	-2.36E-08	-2.42E-08	-2.26E-08	-2.31E-08	-2.17E-08	-2.14E-08	-2.12E-08	-2.04E-08	-2.18E-08	-2.20E-08	-2.22E-08	-2.14E-08	-2.15E-08	-2.08E-08
-2.37E-08	-2.39E-08	-2.41E-08	-2.29E-08	-2.35E-08	-2.26E-08	-2.18E-08	-2.18E-08	-2.15E-08	-2.29E-08	-2.25E-08	-2.31E-08	-2.28E-08	-2.29E-08	-2.17E-08
-2.41E-08	-2.42E-08	-2.41E-08	-2.29E-08	-2.36E-08	-2.27E-08	-2.19E-08	-2.25E-08	-2.21E-08	-2.30E-08	-2.26E-08	-2.37E-08	-2.33E-08	-2.35E-08	-2.24E-08
-2.46E-08	-2.46E-08	-2.44E-08	-2.31E-08	-2.40E-08	-2.33E-08	-2.24E-08	-2.32E-08	-2.24E-08	-2.28E-08	-2.23E-08	-2.37E-08	-2.36E-08	-2.35E-08	-2.27E-08
-2.54E-08	-2.53E-08	-2.46E-08	-2.38E-08	-2.43E-08	-2.39E-08	-2.33E-08	-2.36E-08	-2.26E-08	-2.25E-08	-2.22E-08	-2.39E-08	-2.41E-08	-2.37E-08	-2.26E-08
-2.60E-08	-2.59E-08	-2.52E-08	-2.43E-08	-2.46E-08	-2.47E-08	-2.43E-08	-2.43E-08	-2.32E-08	-2.25E-08	-2.26E-08	-2.41E-08	-2.47E-08	-2.44E-08	-2.28E-08
-2.62E-08	-2.61E-08	-2.59E-08	-2.52E-08	-2.51E-08	-2.52E-08	-2.50E-08	-2.52E-08	-2.40E-08	-2.32E-08	-2.36E-08	-2.47E-08	-2.48E-08	-2.48E-08	-2.31E-08
-2.64E-08	-2.62E-08	-2.63E-08	-2.64E-08	-2.58E-08	-2.52E-08	-2.56E-08	-2.56E-08	-2.48E-08	-2.41E-08	-2.46E-08	-2.59E-08	-2.56E-08	-2.48E-08	-2.35E-08
-2.69E-08	-2.65E-08	-2.66E-08	-2.68E-08	-2.61E-08	-2.56E-08	-2.60E-08	-2.63E-08	-2.52E-08	-2.47E-08	-2.56E-08	-2.68E-08	-2.61E-08	-2.44E-08	-2.39E-08
-2.76E-08	-2.72E-08	-2.71E-08	-2.72E-08	-2.63E-08	-2.63E-08	-2.64E-08	-2.69E-08	-2.53E-08	-2.56E-08	-2.66E-08	-2.68E-08	-2.61E-08	-2.43E-08	-2.40E-08
-2.87E-08	-2.81E-08	-2.75E-08	-2.75E-08	-2.67E-08	-2.69E-08	-2.69E-08	-2.76E-08	-2.64E-08	-2.66E-08	-2.68E-08	-2.66E-08	-2.61E-08	-2.50E-08	-2.44E-08
-2.95E-08	-2.87E-08	-2.80E-08	-2.82E-08	-2.72E-08	-2.77E-08	-2.77E-08	-2.81E-08	-2.74E-08	-2.72E-08	-2.67E-08	-2.64E-08	-2.60E-08	-2.55E-08	-2.48E-08
-2.97E-08	-2.88E-08	-2.84E-08	-2.87E-08	-2.78E-08	-2.87E-08	-2.88E-08	-2.87E-08	-2.82E-08	-2.75E-08	-2.65E-08	-2.65E-08	-2.65E-08	-2.62E-08	-2.54E-08
-2.97E-08	-2.87E-08	-2.85E-08	-2.90E-08	-2.84E-08	-2.95E-08	-2.94E-08	-2.92E-08	-2.91E-08	-2.81E-08	-2.68E-08	-2.65E-08	-2.70E-08	-2.65E-08	-2.61E-08
-2.97E-08	-2.90E-08	-2.86E-08	-2.93E-08	-2.86E-08	-2.94E-08	-2.94E-08	-2.97E-08	-2.95E-08	-2.85E-08	-2.76E-08	-2.68E-08	-2.74E-08	-2.72E-08	-2.71E-08
-2.99E-08	-2.93E-08	-2.89E-08	-2.95E-08	-2.84E-08	-2.95E-08	-2.96E-08	-3.00E-08	-2.96E-08	-2.89E-08	-2.85E-08	-2.73E-08	-2.79E-08	-2.78E-08	-2.78E-08
-3.06E-08	-2.99E-08	-2.93E-08	-2.97E-08	-2.94E-08	-2.96E-08	-2.97E-08	-3.03E-08	-3.00E-08	-2.94E-08	-2.92E-08	-2.86E-08	-2.84E-08	-2.83E-08	-2.85E-08
-3.12E-08	-3.06E-08	-2.98E-08	-3.02E-08	-3.01E-08	-3.01E-08	-2.98E-08	-3.02E-08	-3.01E-08	-2.97E-08	-2.99E-08	-2.95E-08	-2.90E-08	-2.85E-08	-2.88E-08
-3.21E-08	-3.12E-08	-3.05E-08	-3.04E-08	-3.03E-08	-3.01E-08	-2.98E-08	-3.03E-08	-3.03E-08	-2.98E-08	-3.01E-08	-3.01E-08	-2.96E-08	-2.87E-08	-2.90E-08
-3.31E-08	-3.21E-08	-3.10E-08	-3.01E-08	-3.03E-08	-2.98E-08	-2.97E-08	-3.04E-08	-3.03E-08	-3.00E-08	-3.03E-08	-3.03E-08	-2.98E-08	-2.90E-08	-2.95E-08
-3.37E-08	-3.29E-08	-3.14E-08	-2.99E-08	-3.02E-08	-2.97E-08	-2.99E-08	-3.02E-08	-3.04E-08	-3.00E-08	-3.05E-08	-3.03E-08	-3.01E-08	-2.97E-08	-2.97E-08
-3.35E-08	-3.31E-08	-3.16E-08	-2.97E-08	-3.02E-08	-3.00E-08	-3.03E-08	-3.01E-08	-3.08E-08	-3.06E-08	-3.08E-08	-3.05E-08	-3.04E-08	-2.98E-08	-2.99E-08
-3.33E-08	-3.30E-08	-3.21E-08	-3.07E-08	-3.11E-08	-3.08E-08	-3.05E-08	-3.04E-08	-3.15E-08	-3.12E-08	-3.11E-08	-3.08E-08	-3.07E-08	-3.01E-08	-3.01E-08
-3.30E-08	-3.31E-08	-3.29E-08	-3.21E-08	-3.23E-08	-3.18E-08	-3.10E-08	-3.10E-08	-3.24E-08	-3.17E-08	-3.16E-08	-3.11E-08	-3.08E-08	-3.05E-08	-3.03E-08
-3.30E-08	-3.33E-08	-3.32E-08	-3.31E-08	-3.30E-08	-3.21E-08	-3.17E-08	-3.15E-08	-3.27E-08	-3.22E-08	-3.21E-08	-3.11E-08	-3.11E-08	-3.04E-08	-3.06E-08
-3.29E-08	-3.33E-08	-3.31E-08	-3.32E-08	-3.34E-08	-3.25E-08	-3.21E-08	-3.11E-08	-3.24E-08	-3.21E-08	-3.22E-08	-3.16E-08	-3.14E-08	-3.05E-08	-3.07E-08
-3.29E-08	-3.33E-08	-3.24E-08	-3.29E-08	-3.34E-08	-3.29E-08	-3.24E-08	-3.07E-08	-3.25E-08	-3.20E-08	-3.20E-08	-3.22E-08	-3.16E-08	-3.08E-08	-3.10E-08
-3.33E-08	-3.35E-08	-3.23E-08	-3.30E-08	-3.32E-08	-3.33E-08	-3.23E-08	-3.04E-08	-3.19E-08	-3.16E-08	-3.18E-08	-3.22E-08	-3.19E-08	-3.19E-08	-3.14E-08
-3.37E-08	-3.39E-08	-3.29E-08	-3.35E-08	-3.35E-08	-3.30E-08	-3.22E-08	-2.96E-08	-3.07E-08	-3.05E-08	-3.12E-08	-3.19E-08	-3.23E-08	-3.23E-08	-3.10E-08
-3.39E-08	-3.40E-08	-3.39E-08	-3.38E-08	-3.39E-08	-3.27E-08	-3.20E-08	-2.81E-08	-2.84E-08	-2.89E-08	-3.04E-08	-3.19E-08	-3.21E-08	-3.25E-08	-3.14E-08
-3.46E-08	-3.45E-08	-3.45E-08	-3.38E-08	-3.42E-08	-3.28E-08	-3.14E-08	-2.66E-08	-2.56E-08	-2.75E-08	-3.01E-08	-3.20E-08	-3.18E-08	-3.25E-08	-3.16E-08
-3.48E-08	-3.51E-08	-3.46E-08	-3.38E-08	-3.39E-08	-3.27E-08	-3.02E-08	-2.48E-08	-2.28E-08	-2.62E-08	-2.99E-08	-3.20E-08	-3.20E-08	-3.27E-08	-3.18E-08
-3.45E-08	-3.51E-08	-3.46E-08	-3.40E-08	-3.37E-08	-3.27E-08	-2.97E-08	-2.43E-08	-2.11E-08	-2.61E-08	-2.98E-08	-3.18E-08	-3.23E-08	-3.24E-08	-3.21E-08
-3.43E-08	-3.48E-08	-3.45E-08	-3.39E-08	-3.41E-08	-3.30E-08	-3.08E-08	-2.64E-08	-2.37E-08	-2.78E-08	-3.03E-08	-3.24E-08	-3.24E-08	-3.22E-08	-3.18E-08
-3.42E-08	-3.49E-08	-3.47E-08	-3.40E-08	-3.40E-08	-3.32E-08	-3.19E-08	-3.00E-08	-2.84E-08	-3.09E-08	-3.16E-08	-3.29E-08	-3.29E-08	-3.24E-08	-3.12E-08
-3.36E-08	-3.47E-08	-3.50E-08	-3.44E-08	-3.42E-08	-3.36E-08	-3.28E-08	-3.24E-08	-3.18E-08	-3.25E-08	-3.27E-08	-3.33E-08	-3.30E-08	-3.24E-08	-3.16E-08
-3.28E-08	-3.41E-08	-3.47E-08	-3.43E-08	-3.43E-08	-3.34E-08	-3.32E-08	-3.34E-08	-3.26E-08	-3.28E-08	-3.28E-08	-3.26E-08	-3.26E-08	-3.19E-08	-3.15E-08
-3.29E-08	-3.34E-08	-3.37E-08	-3.37E-08	-3.45E-08	-3.32E-08	-3.34E-08	-3.31E-08	-3.28E-08	-3.24E-08	-3.19E-08	-3.23E-08	-3.15E-08	-3.14E-08	-3.13E-08

3.10E-15	3.13E-15	3.17E-15	3.18E-15	3.23E-15	3.40E-15	3.08E-15	2.94E-15	2.79E-15	2.91E-15	2.88E-15	3.02E-15	3.13E-15	3.01E-15	2.88E-15
2.97E-15	3.00E-15	3.10E-15	3.09E-15	3.04E-15	3.20E-15	2.94E-15	2.88E-15	2.67E-15	2.76E-15	2.78E-15	2.91E-15	2.97E-15	2.84E-15	2.77E-15
2.89E-15	2.94E-15	3.07E-15	2.99E-15	2.90E-15	2.94E-15	2.86E-15	2.81E-15	2.54E-15	2.65E-15	2.63E-15	2.76E-15	2.76E-15	2.65E-15	2.64E-15
2.81E-15	2.83E-15	2.94E-15	2.80E-15	2.75E-15	2.68E-15	2.71E-15	2.70E-15	2.41E-15	2.56E-15	2.48E-15	2.51E-15	2.53E-15	2.48E-15	2.49E-15
2.72E-15	2.79E-15	2.69E-15	2.58E-15	2.56E-15	2.49E-15	2.47E-15	2.54E-15	2.35E-15	2.44E-15	2.39E-15	2.37E-15	2.39E-15	2.35E-15	2.36E-15
2.59E-15	2.67E-15	2.47E-15	2.38E-15	2.42E-15	2.36E-15	2.30E-15	2.35E-15	2.31E-15	2.36E-15	2.36E-15	2.25E-15	2.31E-15	2.23E-15	2.24E-15
2.33E-15	2.40E-15	2.28E-15	2.24E-15	2.29E-15	2.24E-15	2.14E-15	2.19E-15	2.20E-15	2.29E-15	2.35E-15	2.14E-15	2.16E-15	2.09E-15	2.11E-15
2.10E-15	2.13E-15	2.12E-15	2.11E-15	2.18E-15	2.08E-15	2.01E-15	2.04E-15	2.07E-15	2.19E-15	2.26E-15	2.03E-15	1.99E-15	1.97E-15	1.98E-15
1.94E-15	1.99E-15	2.01E-15	1.98E-15	2.05E-15	1.92E-15	1.89E-15	1.91E-15	1.94E-15	2.02E-15	2.05E-15	1.91E-15	1.86E-15	1.84E-15	1.82E-15
1.82E-15	1.91E-15	1.90E-15	1.85E-15	1.93E-15	1.85E-15	1.79E-15	1.77E-15	1.76E-15	1.88E-15	1.88E-15	1.79E-15	1.77E-15	1.71E-15	1.73E-15
1.78E-15	1.81E-15	1.80E-15	1.78E-15	1.80E-15	1.83E-15	1.70E-15	1.68E-15	1.66E-15	1.71E-15	1.73E-15	1.69E-15	1.71E-15	1.61E-15	1.64E-15
1.71E-15	1.68E-15	1.72E-15	1.68E-15	1.71E-15	1.70E-15	1.65E-15	1.61E-15	1.58E-15	1.60E-15	1.62E-15	1.61E-15	1.65E-15	1.52E-15	1.53E-15
1.59E-15	1.60E-15	1.64E-15	1.60E-15	1.58E-15	1.57E-15	1.58E-15	1.54E-15	1.52E-15	1.54E-15	1.58E-15	1.53E-15	1.54E-15	1.43E-15	1.39E-15
1.49E-15	1.52E-15	1.52E-15	1.54E-15	1.46E-15	1.47E-15	1.49E-15	1.45E-15	1.48E-15	1.49E-15	1.50E-15	1.43E-15	1.42E-15	1.34E-15	1.29E-15
1.40E-15	1.43E-15	1.39E-15	1.44E-15	1.35E-15	1.35E-15	1.40E-15	1.38E-15	1.42E-15	1.39E-15	1.43E-15	1.37E-15	1.35E-15	1.27E-15	1.19E-15
1.34E-15	1.29E-15	1.28E-15	1.34E-15	1.28E-15	1.26E-15	1.32E-15	1.31E-15	1.32E-15	1.29E-15	1.36E-15	1.31E-15	1.24E-15	1.23E-15	1.14E-15
1.29E-15	1.18E-15	1.19E-15	1.27E-15	1.21E-15	1.19E-15	1.24E-15	1.22E-15	1.24E-15	1.23E-15	1.29E-15	1.21E-15	1.16E-15	1.17E-15	1.10E-15
1.16E-15	1.07E-15	1.09E-15	1.17E-15	1.13E-15	1.13E-15	1.15E-15	1.10E-15	1.10E-15	1.15E-15	1.18E-15	1.15E-15	1.10E-15	1.13E-15	1.09E-15
1.08E-15	1.01E-15	1.05E-15	1.08E-15	1.10E-15	1.10E-15	1.11E-15	1.04E-15	1.04E-15	1.09E-15	1.12E-15	1.12E-15	1.07E-15	1.08E-15	1.06E-15
1.05E-15	9.72E-16	1.03E-15	1.04E-15	1.08E-15	1.06E-15	1.06E-15	1.00E-15	1.03E-15	1.11E-15	1.08E-15	1.09E-15	1.02E-15	1.03E-15	9.94E-16
1.02E-15	9.28E-16	9.86E-16	9.91E-16	1.02E-15	1.02E-15	9.93E-16	9.78E-16	9.86E-16	1.06E-15	9.84E-16	9.91E-16	9.41E-16	9.44E-16	8.97E-16
9.53E-16	8.73E-16	9.18E-16	9.02E-16	9.45E-16	9.31E-16	9.31E-16	9.32E-16	9.12E-16	9.06E-16	8.70E-16	8.56E-16	8.45E-16	8.31E-16	8.21E-16
8.68E-16	8.43E-16	8.45E-16	8.18E-16	8.71E-16	8.22E-16	8.73E-16	8.31E-16	8.06E-16	8.01E-16	7.92E-16	7.92E-16	7.69E-16	7.42E-16	7.62E-16
7.93E-16	8.05E-16	7.69E-16	7.63E-16	7.91E-16	7.54E-16	7.99E-16	7.45E-16	7.36E-16	7.26E-16	7.32E-16	7.37E-16	7.03E-16	6.92E-16	7.07E-16
7.34E-16	7.41E-16	7.20E-16	7.24E-16	7.51E-16	7.20E-16	7.21E-16	6.95E-16	7.06E-16	6.81E-16	6.77E-16	6.78E-16	6.48E-16	6.44E-16	6.36E-16
6.91E-16	7.06E-16	6.84E-16	6.84E-16	7.18E-16	6.92E-16	6.65E-16	6.56E-16	6.94E-16	6.43E-16	6.39E-16	6.23E-16	6.04E-16	5.88E-16	5.82E-16
6.55E-16	6.59E-16	6.46E-16	6.53E-16	6.76E-16	6.63E-16	6.24E-16	6.24E-16	6.92E-16	5.89E-16	6.20E-16	5.97E-16	5.81E-16	5.56E-16	5.57E-16
6.19E-16	5.97E-16	6.08E-16	5.98E-16	6.17E-16	5.80E-16	5.79E-16	5.91E-16	6.50E-16	5.69E-16	5.55E-16	5.70E-16	5.51E-16	5.36E-16	5.41E-16
5.88E-16	5.58E-16	5.52E-16	5.36E-16	5.36E-16	5.07E-16	5.29E-16	5.58E-16	5.99E-16	5.43E-16	5.20E-16	5.28E-16	4.96E-16	4.95E-16	5.08E-16
5.23E-16	5.18E-16	4.98E-16	4.72E-16	4.73E-16	4.72E-16	4.95E-16	5.27E-16	5.60E-16	5.07E-16	4.92E-16	5.01E-16	4.59E-16	4.57E-16	4.86E-16
5.01E-16	4.94E-16	4.73E-16	4.17E-16	4.51E-16	4.46E-16	4.57E-16	4.79E-16	4.84E-16	4.56E-16	4.40E-16	4.57E-16	4.36E-16	4.29E-16	4.41E-16

4.54E-16	4.67E-16	4.34E-16	3.82E-16	4.18E-16	4.04E-16	4.39E-16	4.19E-16	4.28E-16	4.05E-16	3.91E-16	4.28E-16	4.14E-16	4.21E-16	4.15E-16
3.83E-16	4.26E-16	3.87E-16	3.63E-16	3.67E-16	3.74E-16	4.01E-16	3.82E-16	3.82E-16	3.63E-16	3.68E-16	4.13E-16	3.82E-16	3.89E-16	3.57E-16
3.50E-16	4.13E-16	3.73E-16	3.61E-16	3.37E-16	3.57E-16	3.52E-16	3.33E-16	3.38E-16	3.40E-16	3.57E-16	3.69E-16	3.68E-16	3.59E-16	3.23E-16
3.22E-16	3.78E-16	3.53E-16	3.53E-16	3.01E-16	3.20E-16	3.10E-16	3.08E-16	3.07E-16	3.22E-16	3.39E-16	3.17E-16	3.55E-16	3.32E-16	2.88E-16
2.99E-16	3.26E-16	3.19E-16	3.11E-16	2.74E-16	2.90E-16	2.77E-16	2.91E-16	2.78E-16	2.89E-16	2.91E-16	3.02E-16	3.46E-16	2.84E-16	2.52E-16
2.75E-16	2.76E-16	2.95E-16	2.87E-16	2.55E-16	2.73E-16	2.50E-16	2.68E-16	2.46E-16	2.54E-16	2.50E-16	2.83E-16	2.99E-16	2.60E-16	2.27E-16
2.62E-16	2.45E-16	2.56E-16	2.62E-16	2.36E-16	2.47E-16	2.30E-16	2.38E-16	2.15E-16	2.27E-16	2.12E-16	2.39E-16	2.55E-16	2.46E-16	2.15E-16
2.40E-16	2.03E-16	2.13E-16	2.33E-16	2.14E-16	2.11E-16	1.90E-16	2.05E-16	1.91E-16	1.94E-16	1.75E-16	1.97E-16	2.19E-16	2.33E-16	2.06E-16
2.12E-16	1.82E-16	1.69E-16	1.94E-16	1.92E-16	1.80E-16	1.69E-16	1.64E-16	1.71E-16	1.74E-16	1.57E-16	1.62E-16	2.00E-16	2.17E-16	1.93E-16
1.81E-16	1.69E-16	1.46E-16	1.75E-16	1.73E-16	1.66E-16	1.54E-16	1.37E-16	1.54E-16	1.65E-16	1.49E-16	1.31E-16	1.63E-16	1.84E-16	1.74E-16
1.84E-16	1.76E-16	1.53E-16	1.74E-16	1.66E-16	1.56E-16	1.47E-16	1.28E-16	1.51E-16	1.59E-16	1.45E-16	1.27E-16	1.40E-16	1.49E-16	1.48E-16
1.61E-16	1.61E-16	1.34E-16	1.54E-16	1.35E-16	1.29E-16	1.20E-16	1.04E-16	1.33E-16	1.16E-16	1.14E-16	1.01E-16	1.00E-16	1.12E-16	1.09E-16
1.39E-16	1.43E-16	1.23E-16	1.35E-16	1.09E-16	1.04E-16	9.82E-17	8.99E-17	9.25E-17	8.61E-17	7.59E-17	8.09E-17	8.59E-17	9.36E-17	8.11E-17
1.19E-16	1.37E-16	1.33E-16	1.19E-16	8.29E-17	7.97E-17	7.93E-17	8.35E-17	6.92E-17	6.04E-17	5.47E-17	6.77E-17	8.46E-17	8.23E-17	6.57E-17
1.01E-16	1.21E-16	1.17E-16	9.88E-17	6.71E-17	6.83E-17	7.71E-17	6.86E-17	4.70E-17	4.71E-17	4.77E-17	5.90E-17	8.11E-17	7.56E-17	5.69E-17
8.76E-17	1.03E-16	9.29E-17	8.71E-17	5.89E-17	6.03E-17	6.24E-17	5.80E-17	3.71E-17	4.19E-17	4.47E-17	6.03E-17	7.64E-17	6.88E-17	5.46E-17
7.03E-17	8.57E-17	9.15E-17	7.55E-17	5.07E-17	5.73E-17	4.84E-17	4.90E-17	3.38E-17	3.60E-17	4.27E-17	5.58E-17	6.66E-17	5.72E-17	5.05E-17
5.93E-17	6.74E-17	7.74E-17	5.56E-17	4.55E-17	4.56E-17	3.62E-17	3.98E-17	3.06E-17	2.78E-17	3.93E-17	4.54E-17	4.46E-17	4.04E-17	3.78E-17
4.88E-17	4.63E-17	5.96E-17	3.66E-17	3.26E-17	3.49E-17	2.55E-17	2.96E-17	2.57E-17	2.52E-17	3.48E-17	3.88E-17	2.89E-17	2.44E-17	2.77E-17
3.43E-17	3.01E-17	3.86E-17	2.16E-17	1.74E-17	2.56E-17	1.91E-17	2.19E-17	2.44E-17	2.38E-17	2.98E-17	2.84E-17	1.75E-17	1.50E-17	2.52E-17
2.30E-17	2.01E-17	2.28E-17	1.46E-17	1.17E-17	1.66E-17	1.79E-17	1.74E-17	2.39E-17	1.92E-17	1.92E-17	2.31E-17	1.45E-17	1.19E-17	2.16E-17
1.73E-17	1.03E-17	1.24E-17	9.02E-18	9.97E-18	9.61E-18	1.36E-17	1.37E-17	1.92E-17	1.34E-17	1.19E-17	1.63E-17	1.34E-17	1.16E-17	1.84E-17
9.52E-18	3.16E-18	8.62E-18	7.66E-18	9.30E-18	5.91E-18	4.61E-18	4.96E-18	1.05E-17	6.69E-18	9.17E-18	1.25E-17	9.97E-18	8.65E-18	1.23E-17
6.07E-18	1.16E-18	5.44E-18	6.17E-18	8.71E-18	4.40E-18	1.46E-18	9.85E-19	4.40E-18	2.00E-18	4.21E-18	6.94E-18	7.59E-18	1.54E-18	5.65E-18
2.19E-18	2.64E-19	1.75E-18	5.14E-18	9.31E-18	2.47E-18	3.33E-19	1.42E-21	2.82E-19	5.57E-19	1.24E-18	2.15E-18	2.30E-18	3.51E-19	1.52E-18
1.15E-19	3.35E-20	3.55E-19	2.01E-18	6.74E-18	1.13E-18	2.99E-19	2.24E-20	2.23E-20	3.19E-21	6.20E-22	5.27E-20	2.30E-19	7.13E-20	2.81E-20
2.68E-19	3.30E-19	6.12E-21	1.61E-20	1.89E-18	2.34E-20	1.40E-19	5.22E-19	3.71E-19	8.87E-19	1.94E-18	4.04E-18	1.45E-18	2.10E-18	1.11E-18
9.54E-19	1.05E-18	5.03E-19	9.50E-19	5.76E-20	1.14E-18	2.00E-18	1.42E-18	1.21E-18	3.00E-18	7.03E-18	7.27E-18	5.38E-18	6.85E-18	3.27E-18
2.21E-18	3.62E-18	2.14E-18	2.98E-18	1.62E-18	2.64E-18	3.17E-18	3.91E-18	4.31E-18	7.09E-18	1.20E-17	9.28E-18	1.20E-17	1.20E-17	5.62E-18
5.27E-18	7.22E-18	4.05E-18	6.51E-18	5.20E-18	6.68E-18	4.31E-18	7.35E-18	9.09E-18	8.44E-18	1.69E-17	1.34E-17	2.05E-17	1.69E-17	1.02E-17
8.10E-18	9.68E-18	1.05E-17	1.29E-17	1.10E-17	1.29E-17	8.63E-18	1.37E-17	1.28E-17	1.30E-17	2.26E-17	1.80E-17	2.42E-17	1.84E-17	7.63E-18
1.00E-17	1.20E-17	1.60E-17	2.19E-17	1.90E-17	1.83E-17	1.66E-17	1.86E-17	1.11E-17	1.81E-17	2.98E-17	2.89E-17	2.98E-17	2.18E-17	9.43E-18
1.17E-17	1.41E-17	2.18E-17	3.14E-17	2.28E-17	2.59E-17	2.66E-17	2.54E-17	1.26E-17	2.29E-17	3.87E-17	3.88E-17	4.23E-17	2.67E-17	2.13E-17
1.49E-17	2.03E-17	2.90E-17	3.23E-17	2.66E-17	3.52E-17	3.13E-17	2.98E-17	1.95E-17	2.93E-17	4.47E-17	4.26E-17	4.66E-17	3.47E-17	3.50E-17
2.08E-17	2.60E-17	3.07E-17	3.45E-17	3.14E-17	3.69E-17	3.66E-17	3.42E-17	3.40E-17	3.51E-17	4.60E-17	4.28E-17	4.68E-17	3.98E-17	4.91E-17
2.85E-17	3.16E-17	3.29E-17	3.63E-17	3.53E-17	3.52E-17	4.26E-17	4.25E-17	4.92E-17	4.51E-17	4.70E-17	4.71E-17	5.60E-17	5.31E-17	4.75E-17
3.65E-17	3.61E-17	3.50E-17	4.00E-17	3.84E-17	3.52E-17	4.35E-17	4.95E-17	5.91E-17	5.62E-17	5.51E-17	5.63E-17	7.04E-17	5.96E-17	5.05E-17
3.99E-17	3.78E-17	2.94E-17	4.08E-17	4.64E-17	4.30E-17	4.06E-17	6.43E-17	6.72E-17	6.85E-17	6.59E-17	6.93E-17	7.74E-17	6.08E-17	5.46E-17
4.49E-17	4.51E-17	4.50E-17	4.97E-17	5.02E-17	5.22E-17	4.01E-17	7.72E-17	7.83E-17	7.85E-17	7.69E-17	7.52E-17	7.78E-17	5.96E-17	5.79E-17
5.15E-17	4.95E-17	6.12E-17	6.01E-17	5.99E-17	7.15E-17	6.94E-17	9.00E-17	9.21E-17	9.74E-17	9.08E-17	9.71E-17	9.11E-17	6.77E-17	6.13E-17
6.21E-17	5.89E-17	6.36E-17	7.26E-17	8.50E-17	9.18E-17	9.48E-17	1.15E-16	1.10E-16	1.13E-16	1.06E-16	1.15E-16	1.00E-16	6.87E-17	6.17E-17
8.53E-17	8.27E-17	7.79E-17	9.71E-17	1.14E-16	1.29E-16	1.13E-16	1.34E-16	1.16E-16	1.24E-16	1.16E-16	1.17E-16	1.01E-16	7.45E-17	7.21E-17
1.22E-16	1.19E-16	1.19E-16	1.26E-16	1.39E-16	1.55E-16	1.40E-16	1.43E-16	1.32E-16	1.31E-16	1.22E-16	1.20E-16	1.15E-16	9.34E-17	1.04E-16
1.57E-16	1.46E-16	1.42E-16	1.62E-16	1.50E-16	1.63E-16	1.52E-16	1.45E-16	1.58E-16	1.44E-16	1.28E-16	1.41E-16	1.45E-16	1.21E-16	1.21E-16
1.77E-16	1.61E-16	1.55E-16	1.80E-16	1.62E-16	1.65E-16	1.66E-16	1.50E-16	1.81E-16	1.73E-16	1.60E-16	1.59E-16	1.71E-16	1.46E-16	1.27E-16
1.91E-16	1.80E-16	1.84E-16	1.89E-16	1.81E-16	1.70E-16	1.84E-16	1.64E-16	1.95E-16	2.04E-16	1.95E-16	1.73E-16	1.90E-16	1.62E-16	1.54E-16
2.10E-16	2.09E-16	2.09E-16	2.00E-16	1.94E-16	1.79E-16	1.83E-16	1.78E-16	2.10E-16	2.40E-16	2.36E-16	1.94E-16	2.01E-16	1.77E-16	1.85E-16
2.39E-16	2.44E-16	2.41E-16	2.31E-16	2.37E-16	2.02E-16	1.84E-16	1.93E-16	2.25E-16	2.63E-16	2.64E-16	2.24E-16	2.28E-16	1.98E-16	2.14E-16
2.66E-16	2.69E-16	2.78E-16	2.75E-16	2.66E-16	2.29E-16	2.09E-16	2.27E-16	2.56E-16	2.77E-16	2.74E-16	2.47E-16	2.48E-16	2.12E-16	2.34E-16
2.79E-16	2.86E-16	3.08E-16	3.01E-16	2.78E-16	2.73E-16	2.43E-16	2.77E-16	2.88E-16	2.85E-16	2.91E-16	2.58E-16	2.52E-16	2.37E-16	2.61E-16
2.96E-16	3.04E-16	3.37E-16	3.25E-16	2.96E-16	2.95E-16	2.89E-16	2.99E-16	3.09E-16	2.81E-16	2.89E-16	2.58E-16	2.72E-16	2.68E-16	2.65E-16
3.15E-16	3.24E-16	3.61E-16	3.48E-16	3.03E-16	3.05E-16	3.17E-16	3.18E-16	3.28E-16	2.93E-16	2.80E-16	2.70E-16	2.99E-16	2.84E-16	2.72E-16
3.30E-16	3.58E-16	3.77E-16	3.57E-16	3.24E-16	3.09E-16	3.22E-16	3.29E-16	3.39E-16	3.10E-16	2.72E-16	2.85E-16	2.97E-16	2.84E-16	2.87E-16
3.61E-16	3.90E-16	3.94E-16	3.68E-16	3.65E-16	3.46E-16	3.68E-16	3.43E-16	3.56E-16	3.25E-16	2.77E-16	2.94E-16	3.08E-16	3.05E-16	3.11E-16
3.85E-16	4.17E-16	4.11E-16	3.76E-16	3.94E-16	3.79E-16	4.08E-16	3.63E-16	3.67E-16	3.28E-16	2.92E-16	3.26E-16	3.41E-16	3.38E-16	3.49E-16
3.90E-16	4.27E-16	4.26E-16	3.83E-16	4.04E-16	3.94E-16	4.12E-16	3.80E-16	3.88E-16	3.52E-16	3.17E-16	3.54E-16	3.73E-16	3.50E-16	3.68E-16
4.12E-16	4.49E-16	4.70E-16	4.07E-16	4.28E-16	4.03E-16	4.06E-16	3.84E-16	3.96E-16	3.64E-16	3.43E-16	3.89E-16	3.96E-16	3.47E-16	3.85E-16
4.57E-16	4.89E-16	5.16E-16	4.48E-16	4.56E-16	4.06E-16	4.01E-16	3.80E-16	3.98E-16	3.86E-16	3.85E-16	4.13E-16	4.01E-16	3.66E-16	3.96E-16
5.10E-16	5.18E-16	5.62E-16	4.92E-16	4.98E-16	4.25E-16	4.32E-16	4.27E-16	3.92E-16	4.24E-16	4.42E-16	4.43E-16	4.11E-16	3.92E-16	4.16E-16
5.36E-16	5.57E-16	5.88E-16	5.12E-16	5.35E-16	4.73E-16	4.59E-16	4.50E-16	4.16E-16	4.76E-16	4.83E-16	4.92E-16	4.60E-16	4.60E-16	4.32E-16
5.61E-16	5.70E-16	5.81E-16	5.24E-16	5.54E-16	5.09E-16	4.74E-16	4.76E-16	4.61E-16	5.25E-16	5.08E-16	5.34E-16	5.20E-16	5.23E-16	4.72E-16
5.81E-16	5.83E-16	5.81E-16	5.23E-16	5.59E-16	5.15E-16	4.80E-16	5.08E-16	4.87E-16	5.31E-16	5.10E-16	5.64E-16	5.44E-16	5.50E-16	5.02E-16
6.06E-16	6.03E-16	5.94E-16	5.32E-16	5.74E-16	5.41E-16	5.04E-16	5.37E-16	5.02E-16	5.20E-16	4.99E-16	5.62E-16	5.55E-16	5.51E-16	5.13E-16
6.43E-16	6.40E-16	6.06E-16	5.67E-16	5.91E-16	5.71E-16	5.43E-16	5.55E-16	5.11E-16	5.05E-16	4.92E-16	5.69E-16	5.80E-16	5.64E-16	5.11E-16
6.75E-16	6.71E-16	6.37E-16	5.90E-16	6.04E-16	6.12E-16	5.89E-16	5.89E-16	5.39E-16	5.08E-16	5.11E-16	5.82E-16	6.08E-16	5.95E-16	5.20E-16
6.86E-16	6.82E-16	6.73E-16	6.35E-16	6.32E-16	6.36E-16	6.27E-16	6.37E-16	5.78E-16	5.40E-16	5.56E-16	6.08E-16	6.16E-16	6.16E-16	5.33E-16
6.98E-16	6.87E-16	6.91E-16	6.95E-16	6.63E-16	6.34E-16	6.54E-16	6.58E-16	6.17E-16	5.79E-16	6.04E-16	6.70E-16	6.54E-16	6.15E-16	5.54E-16
7.24E-16	7.03E-16	7.10E-16	7.20E-16	6.80E-16	6.56E-16	6.78E-16	6.89E-16	6.35E-16	6.10E-16	6.55E-16	7.18E-16	6.80E-16	5.96E-16	5.69E-16
7.64E-16	7.40E-16	7.33E-16	7.38E-16	6.94E-16	6.94E-16	6.98E-16	7.25E-16	6.38E-16	6.54E-16	7.07E-16	7.18E-16	6.83E-16	5.91E-16	5.77E-16
8.25E-16	7.92E-16	7.59E-16	7.57E-16	7.15E-16	7.26E-16	7.25E-16	7.61E-16	6.95E-16	7.06E-16	7.16E-16	7.07E-16	6.84E-16	6.25E-16	5.94E-16
8.70E-16	8.23E-16	7.82E-16	7.95E-16	7.38E-16	7.66E-16	7.69E-16	7.90E-16	7.52E-16	7.38E-16	7.10E-16	6.95E-16	6.77E-16	6.52E-16	6.14E-16
8.84E-16	8.28E-16	8.05E-16	8.23E-16	7.73E-16	8.24E-16	8.30E-16	8.23E-16	7.94E-16	7.59E-16	7.05E-16	7.01E-16	7.04E-16	6.86E-16	6.45E-16
8.84E-16	8.24E-16	8.12E-16	8.41E-16	8.08E-16	8.68E-16	8.65E-16	8.55E-16	8.47E-16	7.89E-16	7.18E-16	7.02E-16	7.31E-16	7.03E-16	6.83E-16
8.84E-16	8.38E-16	8.20E-16	8.56E-16	8.19E-16	8.67E-16	8.66E-16	8.83E-16	8.68E-16	8.14E-16	7.59E-16	7.17E-16	7.52E-16	7.41E-16	7.34E-16
8.97E-16	8.58E-16	8.37E-16	8.70E-16	8.08E-16	8.72E-16	8.74E-16	9.02E-16	8.75E-16	8.36E-16	8.10E-16	7.47E-16	7.79E-16	7.70E-16	7.73E-16
9.34E-16	8.94E-16	8.59E-16	8.82E-16	8.64E-16	8.75E-16	8.80E-16	9.15E-16	9.01E-16	8.62E-16	8.55E-16	8.15E-16	8.06E-16	8.01E-16	8.13E-16
9.74E-16	9.34E-16	8.89E-16	9.15E-16	9.06E-16	9.04E-16	8.87E-16	9.10E-16	9.03E-16	8.82E-16	8.91E-16	8.69E-16	8.42E-16	8.11E-16	8.28E-16
1.03E-15	9.74E-16	9.29E-16	9.24E-16	9.18E-16	9.04E-16	8.86E-16	9.20E-16	9.19E-16	8.87E-16	9.06E-16	9.05E-16	8.77E-16	8.25E-16	8.41E-16
1.10E-15	1.03E-15	9.61E-16	9.09E-16	9.17E-16	8.86E-16	8.83E-16	9.21E-16	9.19E-16	8.99E-16	9.20E-16	9.17E-16	8.87E-16	8.39E-16	8.69E-16
1.13E-15	1.08E-15	9.87E-16	8.96E-16	9.10E-16	8.83E-16	8.92E-16	9.10E-16	9.24E-16	9.03E-16	9.30E-16	9.18E-16	9.06E-16	8.79E-16	8.83E-16
1.12E-15	1.10E-15	1.00E-15	8.83E-16	9.13E-16	8.99E-16	9.19E-16	9.03E-16	9.49E-16	9.36E-16	9.46E-16	9.33E-16	9.23E-16	8.91E-16	8.95E-16
1.11E-15	1.09E-15	1.03E-15	9.45E-16	9.69E-16	9.47E-16	9.32E-16	9.22E-16	9.95E-16	9.75E-16	9.69E-16	9.51E-16	9.42E-16	9.06E-16	9.08E-16
1.09E-15	1.10E-15	1.08E-15	1.03E-15	1.05E-15	1.01E-15	9.64E-16	9.61E-16	1.05E-15	1.01E-15	9.99E-16	9.68E-16	9.51E-16	9.28E-16	9.19E-16
1.09E-15	1.11E-15	1.10E-15	1.10E-15	1.09E-15	1.03E-15	1.00E-15	9.90E-16	1.07E-15	1.04E-15	1.03E-15	9.65E-16	9.66E-16	9.23E-16	9.37E-16
1.08E-15	1.11E-15	1.09E-15	1.10E-15	1.11E-15	1.05E-15	1.03E-15	9.65E-16	1.05E-15	1.03E-15	1.04E-15	9.96E-16	9.84E-16	9.31E-16	9.43E-16
1.08E-15	1.11E-15	1.05E-15	1.08E-15	1.11E-15	1.08E-15	1.05E-15	9.40E-16	1.05E-15	1.03E-15	1.02E-15	1.04E-15	9.96E-16	9.47E-16	9.61E-16

1.11E-15	1.12E-15	1.04E-15	1.09E-15	1.10E-15	1.11E-15	1.04E-15	9.24E-16	1.01E-15	1.00E-15	1.01E-15	1.03E-15	1.02E-15	1.02E-15	9.84E-16
1.13E-15	1.15E-15	1.08E-15	1.12E-15	1.12E-15	1.09E-15	1.04E-15	8.75E-16	9.41E-16	9.28E-16	9.70E-16	1.02E-15	1.04E-15	1.04E-15	9.62E-16
1.15E-15	1.15E-15	1.15E-15	1.15E-15	1.15E-15	1.07E-15	1.03E-15	7.90E-16	8.04E-16	8.32E-16	9.22E-16	1.02E-15	1.03E-15	1.06E-15	9.83E-16
1.20E-15	1.19E-15	1.19E-15	1.15E-15	1.17E-15	1.07E-15	9.84E-16	7.05E-16	6.56E-16	7.54E-16	9.03E-16	1.03E-15	1.01E-15	1.06E-15	1.00E-15
1.21E-15	1.23E-15	1.20E-15	1.14E-15	1.15E-15	1.07E-15	9.15E-16	6.16E-16	5.19E-16	6.84E-16	8.91E-16	1.02E-15	1.02E-15	1.07E-15	1.01E-15
1.19E-15	1.23E-15	1.20E-15	1.16E-15	1.14E-15	1.07E-15	8.84E-16	5.93E-16	4.47E-16	6.81E-16	8.86E-16	1.01E-15	1.04E-15	1.05E-15	1.03E-15
1.17E-15	1.21E-15	1.19E-15	1.15E-15	1.16E-15	1.09E-15	9.48E-16	6.99E-16	5.62E-16	7.75E-16	9.20E-16	1.05E-15	1.05E-15	1.04E-15	1.01E-15
1.17E-15	1.22E-15	1.21E-15	1.16E-15	1.15E-15	1.11E-15	1.02E-15	9.01E-16	8.07E-16	9.57E-16	9.96E-16	1.08E-15	1.08E-15	1.05E-15	9.74E-16
1.13E-15	1.20E-15	1.23E-15	1.18E-15	1.17E-15	1.13E-15	1.07E-15	1.05E-15	1.01E-15	1.05E-15	1.07E-15	1.11E-15	1.09E-15	1.05E-15	9.97E-16
1.08E-15	1.16E-15	1.20E-15	1.17E-15	1.17E-15	1.11E-15	1.10E-15	1.11E-15	1.07E-15	1.07E-15	1.07E-15	1.06E-15	1.06E-15	1.02E-15	9.93E-16
1.08E-15	1.12E-15	1.14E-15	1.14E-15	1.19E-15	1.11E-15	1.12E-15	1.09E-15	1.07E-15	1.05E-15	1.02E-15	1.05E-15	9.95E-16	9.89E-16	9.79E-16
9.28E-14	9.29E-14	9.21E-14	9.06E-14	9.03E-14	8.87E-14	8.66E-14	8.44E-14	8.31E-14	8.44E-14	8.51E-14	8.54E-14	8.52E-14	8.24E-14	8.07E-14

5.37E-08	5.35E-08	5.38E-08	5.25E-08	5.21E-08	5.15E-08	5.32E-08	5.29E-08	5.34E-08	5.21E-08	5.17E-08	5.28E-08	5.23E-08	5.15E-08	5.05E-08	4.97E-08
5.26E-08	5.21E-08	5.20E-08	5.16E-08	5.07E-08	5.01E-08	5.18E-08	5.10E-08	5.18E-08	5.01E-08	5.02E-08	5.06E-08	5.12E-08	5.03E-08	4.92E-08	4.88E-08
5.14E-08	5.10E-08	5.10E-08	5.09E-08	4.95E-08	4.89E-08	5.02E-08	4.97E-08	5.11E-08	4.85E-08	4.91E-08	4.89E-08	4.96E-08	4.91E-08	4.79E-08	4.79E-08
5.02E-08	5.01E-08	5.01E-08	4.95E-08	4.84E-08	4.77E-08	4.88E-08	4.89E-08	4.90E-08	4.75E-08	4.78E-08	4.74E-08	4.73E-08	4.77E-08	4.65E-08	4.62E-08
4.90E-08	4.86E-08	4.91E-08	4.82E-08	4.75E-08	4.69E-08	4.75E-08	4.86E-08	4.74E-08	4.67E-08	4.65E-08	4.56E-08	4.59E-08	4.60E-08	4.50E-08	4.47E-08
4.73E-08	4.71E-08	4.74E-08	4.71E-08	4.61E-08	4.56E-08	4.66E-08	4.82E-08	4.70E-08	4.59E-08	4.51E-08	4.36E-08	4.46E-08	4.47E-08	4.37E-08	4.34E-08
4.59E-08	4.62E-08	4.56E-08	4.59E-08	4.52E-08	4.47E-08	4.61E-08	4.70E-08	4.63E-08	4.49E-08	4.36E-08	4.22E-08	4.34E-08	4.36E-08	4.23E-08	4.24E-08
4.45E-08	4.51E-08	4.45E-08	4.53E-08	4.43E-08	4.38E-08	4.53E-08	4.56E-08	4.49E-08	4.36E-08	4.17E-08	4.10E-08	4.20E-08	4.16E-08	4.12E-08	4.12E-08
4.31E-08	4.38E-08	4.33E-08	4.29E-08	4.32E-08	4.25E-08	4.39E-08	4.33E-08	4.23E-08	4.21E-08	4.07E-08	4.01E-08	4.09E-08	4.00E-08	4.00E-08	4.00E-08
4.18E-08	4.20E-08	4.20E-08	4.11E-08	4.22E-08	4.15E-08	4.18E-08	4.09E-08	4.04E-08	3.97E-08	3.97E-08	3.90E-08	3.98E-08	3.90E-08	3.91E-08	3.92E-08
4.06E-08	4.08E-08	4.02E-08	3.97E-08	4.13E-08	3.99E-08	3.97E-08	3.90E-08	3.86E-08	3.80E-08	3.89E-08	3.77E-08	3.91E-08	3.81E-08	3.83E-08	3.82E-08
3.94E-08	3.91E-08	3.89E-08	3.86E-08	3.96E-08	3.85E-08	3.83E-08	3.75E-08	3.69E-08	3.70E-08	3.78E-08	3.67E-08	3.80E-08	3.71E-08	3.74E-08	3.70E-08
3.77E-08	3.77E-08	3.70E-08	3.76E-08	3.85E-08	3.73E-08	3.73E-08	3.65E-08	3.58E-08	3.59E-08	3.64E-08	3.58E-08	3.70E-08	3.62E-08	3.62E-08	3.61E-08
3.63E-08	3.68E-08	3.58E-08	3.69E-08	3.69E-08	3.61E-08	3.68E-08	3.62E-08	3.53E-08	3.46E-08	3.47E-08	3.47E-08	3.58E-08	3.46E-08	3.50E-08	3.52E-08
3.47E-08	3.52E-08	3.52E-08	3.65E-08	3.55E-08	3.50E-08	3.58E-08	3.63E-08	3.54E-08	3.40E-08	3.36E-08	3.37E-08	3.49E-08	3.37E-08	3.38E-08	3.45E-08
3.38E-08	3.39E-08	3.43E-08	3.57E-08	3.46E-08	3.43E-08	3.45E-08	3.59E-08	3.51E-08	3.40E-08	3.32E-08	3.31E-08	3.42E-08	3.33E-08	3.30E-08	3.36E-08
3.28E-08	3.31E-08	3.36E-08	3.51E-08	3.43E-08	3.34E-08	3.34E-08	3.44E-08	3.35E-08	3.34E-08	3.27E-08	3.26E-08	3.36E-08	3.28E-08	3.22E-08	3.26E-08
3.22E-08	3.31E-08	3.30E-08	3.50E-08	3.37E-08	3.28E-08	3.20E-08	3.20E-08	3.16E-08	3.23E-08	3.18E-08	3.21E-08	3.29E-08	3.21E-08	3.14E-08	3.19E-08
3.15E-08	3.29E-08	3.26E-08	3.54E-08	3.29E-08	3.27E-08	3.11E-08	3.04E-08	3.00E-08	3.12E-08	3.09E-08	3.15E-08	3.15E-08	3.12E-08	3.04E-08	3.06E-08
3.04E-08	3.16E-08	3.12E-08	3.30E-08	3.20E-08	3.23E-08	3.02E-08	2.90E-08	2.91E-08	3.02E-08	3.03E-08	3.05E-08	2.96E-08	3.00E-08	2.91E-08	2.84E-08
2.88E-08	2.94E-08	2.93E-08	3.06E-08	3.06E-08	3.11E-08	2.90E-08	2.76E-08	2.81E-08	2.93E-08	2.96E-08	2.96E-08	2.88E-08	2.85E-08	2.77E-08	2.73E-08
2.80E-08	2.79E-08	2.78E-08	2.89E-08	2.91E-08	2.97E-08	2.81E-08	2.64E-08	2.72E-08	2.82E-08	2.83E-08	2.86E-08	2.80E-08	2.73E-08	2.64E-08	2.61E-08
2.71E-08	2.73E-08	2.73E-08	2.80E-08	2.77E-08	2.84E-08	2.68E-08	2.54E-08	2.65E-08	2.71E-08	2.67E-08	2.73E-08	2.69E-08	2.61E-08	2.56E-08	2.55E-08
2.61E-08	2.64E-08	2.66E-08	2.65E-08	2.59E-08	2.66E-08	2.54E-08	2.44E-08	2.55E-08	2.61E-08	2.57E-08	2.61E-08	2.58E-08	2.49E-08	2.48E-08	2.49E-08
2.48E-08	2.51E-08	2.55E-08	2.58E-08	2.45E-08	2.47E-08	2.38E-08	2.35E-08	2.43E-08	2.48E-08	2.45E-08	2.48E-08	2.46E-08	2.40E-08	2.37E-08	2.44E-08
2.43E-08	2.45E-08	2.43E-08	2.45E-08	2.33E-08	2.33E-08	2.31E-08	2.28E-08	2.29E-08	2.36E-08	2.35E-08	2.37E-08	2.34E-08	2.37E-08	2.27E-08	2.27E-08
2.39E-08	2.38E-08	2.33E-08	2.33E-08	2.26E-08	2.21E-08	2.25E-08	2.19E-08	2.21E-08	2.25E-08	2.21E-08	2.30E-08	2.32E-08	2.33E-08	2.20E-08	2.16E-08
2.34E-08	2.30E-08	2.28E-08	2.22E-08	2.18E-08	2.13E-08	2.17E-08	2.08E-08	2.06E-08	2.10E-08	2.11E-08	2.21E-08	2.27E-08	2.20E-08	2.11E-08	2.08E-08
2.23E-08	2.17E-08	2.16E-08	2.09E-08	2.11E-08	2.02E-08	2.05E-08	2.00E-08	1.99E-08	1.99E-08	2.04E-08	2.15E-08	2.23E-08	2.10E-08	2.04E-08	2.03E-08
2.12E-08	2.07E-08	2.05E-08	2.02E-08	2.07E-08	1.99E-08	1.97E-08	1.94E-08	1.94E-08	1.92E-08	2.00E-08	2.14E-08	2.17E-08	2.03E-08	2.00E-08	1.96E-08
2.02E-08	2.00E-08	1.99E-08	1.94E-08	1.97E-08	1.93E-08	1.92E-08	1.85E-08	1.91E-08	1.88E-08	1.91E-08	2.06E-08	2.08E-08	1.98E-08	1.92E-08	1.86E-08
1.95E-08	1.93E-08	1.93E-08	1.83E-08	1.86E-08	1.89E-08	1.83E-08	1.76E-08	1.87E-08	1.82E-08	1.80E-08	1.90E-08	1.95E-08	1.90E-08	1.81E-08	1.74E-08
1.90E-08	1.84E-08	1.82E-08	1.73E-08	1.81E-08	1.85E-08	1.77E-08	1.71E-08	1.80E-08	1.74E-08	1.76E-08	1.76E-08	1.83E-08	1.76E-08	1.71E-08	1.62E-08
1.83E-08	1.76E-08	1.71E-08	1.63E-08	1.78E-08	1.82E-08	1.69E-08	1.67E-08	1.74E-08	1.67E-08	1.75E-08	1.68E-08	1.69E-08	1.64E-08	1.64E-08	1.51E-08
1.75E-08	1.67E-08	1.65E-08	1.56E-08	1.70E-08	1.75E-08	1.67E-08	1.65E-08	1.67E-08	1.57E-08	1.70E-08	1.64E-08	1.57E-08	1.55E-08	1.53E-08	1.47E-08
1.60E-08	1.57E-08	1.53E-08	1.46E-08	1.59E-08	1.62E-08	1.57E-08	1.58E-08	1.58E-08	1.44E-08	1.54E-08	1.54E-08	1.44E-08	1.45E-08	1.42E-08	1.43E-08
1.49E-08	1.41E-08	1.42E-08	1.38E-08	1.48E-08	1.45E-08	1.45E-08	1.51E-08	1.51E-08	1.36E-08	1.38E-08	1.36E-08	1.34E-08	1.29E-08	1.29E-08	1.35E-08
1.42E-08	1.31E-08	1.33E-08	1.31E-08	1.41E-08	1.29E-08	1.28E-08	1.43E-08	1.40E-08	1.27E-08	1.31E-08	1.24E-08	1.24E-08	1.20E-08	1.19E-08	1.24E-08
1.40E-08	1.29E-08	1.29E-08	1.29E-08	1.27E-08	1.17E-08	1.13E-08	1.26E-08	1.25E-08	1.22E-08	1.26E-08	1.22E-08	1.15E-08	1.11E-08	1.16E-08	1.21E-08
1.33E-08	1.31E-08	1.30E-08	1.22E-08	1.20E-08	1.09E-08	1.03E-08	1.11E-08	1.11E-08	1.22E-08	1.25E-08	1.19E-08	1.09E-08	1.03E-08	1.10E-08	1.10E-08
1.29E-08	1.25E-08	1.27E-08	1.14E-08	1.12E-08	1.02E-08	9.39E-09	1.03E-08	1.01E-08	1.14E-08	1.21E-08	1.07E-08	1.06E-08	9.59E-09	1.04E-08	9.98E-09
1.24E-08	1.14E-08	1.20E-08	1.09E-08	1.06E-08	9.94E-09	9.06E-09	9.94E-09	9.63E-09	1.00E-08	1.10E-08	1.04E-08	1.00E-08	9.37E-09	9.90E-09	9.35E-09
1.05E-08	9.64E-09	1.09E-08	9.17E-09	8.99E-09	8.63E-09	8.46E-09	9.33E-09	8.69E-09	8.36E-09	9.01E-09	9.29E-09	8.87E-09	8.74E-09	8.66E-09	8.11E-09
8.86E-09	8.87E-09	9.31E-09	7.77E-09	7.69E-09	7.87E-09	7.81E-09	9.55E-09	8.31E-09	7.56E-09	8.05E-09	8.54E-09	7.82E-09	7.92E-09	8.22E-09	7.79E-09
8.38E-09	8.32E-09	7.92E-09	6.56E-09	6.79E-09	7.41E-09	6.82E-09	8.36E-09	7.93E-09	7.04E-09	7.06E-09	7.56E-09	6.86E-09	7.70E-09	7.54E-09	6.84E-09
8.22E-09	8.10E-09	7.11E-09	5.75E-09	6.12E-09	6.87E-09	6.15E-09	7.03E-09	6.83E-09	6.31E-09	6.40E-09	6.40E-09	6.42E-09	7.31E-09	6.47E-09	5.74E-09
7.97E-09	7.57E-09	6.33E-09	5.22E-09	5.83E-09	6.61E-09	6.00E-09	6.58E-09	6.04E-09	6.32E-09	6.25E-09	5.57E-09	6.73E-09	6.91E-09	5.84E-09	5.22E-09
6.99E-09	6.76E-09	5.56E-09	4.73E-09	5.40E-09	5.89E-09	5.75E-09	5.97E-09	5.30E-09	6.26E-09	6.04E-09	5.39E-09	6.30E-09	6.31E-09	5.23E-09	4.79E-09
6.04E-09	5.46E-09	4.74E-09	4.34E-09	4.80E-09	4.70E-09	5.13E-09	4.97E-09	4.95E-09	5.83E-09	5.38E-09	4.84E-09	5.07E-09	5.77E-09	4.72E-09	4.60E-09
5.25E-09	4.79E-09	4.18E-09	4.08E-09	4.63E-09	4.07E-09	4.49E-09	4.28E-09	4.71E-09	5.40E-09	4.50E-09	3.83E-09	3.76E-09	4.59E-09	4.41E-09	4.29E-09
4.38E-09	4.05E-09	3.63E-09	3.86E-09	4.11E-09	3.95E-09	4.12E-09	3.91E-09	3.90E-09	4.48E-09	3.37E-09	2.69E-09	3.30E-09	3.25E-09	3.66E-09	3.57E-09
3.42E-09	3.25E-09	3.25E-09	3.20E-09	3.48E-09	3.75E-09	3.38E-09	3.04E-09	2.48E-09	3.03E-09	2.50E-09	1.86E-09	2.47E-09	2.28E-09	2.61E-09	3.00E-09
2.61E-09	2.90E-09	3.16E-09	2.53E-09	2.72E-09	3.52E-09	3.01E-09	2.62E-09	2.05E-09	2.18E-09	1.59E-09	1.32E-09	1.48E-09	1.69E-09	1.98E-09	2.78E-09
2.29E-09	3.12E-09	3.62E-09	2.18E-09	2.29E-09	2.97E-09	2.98E-09	2.26E-09	1.63E-09	1.86E-09	1.17E-09	7.65E-10	8.70E-10	9.36E-10	1.18E-09	2.39E-09
2.05E-09	3.18E-09	3.91E-09	2.67E-09	2.31E-09	2.53E-09	2.57E-09	1.77E-09	1.47E-09	1.96E-09	7.56E-10	-9.15E-11	5.04E-10	5.41E-10	4.21E-10	1.62E-09
1.61E-09	2.84E-09	3.84E-09	2.92E-09	2.03E-09	2.15E-09	1.57E-09	7.35E-10	1.54E-09	1.43E-09	5.00E-10	9.43E-11	2.45E-10	7.96E-10	-6.73E-11	5.00E-10
9.99E-10	2.15E-09	2.13E-09	2.25E-09	1.62E-09	1.67E-09	6.05E-10	-4.47E-10	6.50E-10	7.03E-10	2.52E-10	-3.64E-11	-7.23E-11	1.13E-09	-6.28E-10	-5.87E-10
-3.49E-10	2.29E-10	-9.17E-10	1.75E-10	-9.38E-11	-3.31E-10	-1.09E-09	-1.93E-09	-8.73E-10	2.61E-12	-3.27E-10	-7.72E-10	-1.15E-09	2.64E-10	-1.20E-09	-1.04E-09
-1.36E-09	-8.11E-10	-2.54E-09	-1.29E-09	-1.63E-09	-1.33E-09	-1.59E-09	-2.50E-09	-2.17E-09	-6.96E-10	-2.46E-10	-1.44E-09	-2.34E-09	-6.13E-10	-1.68E-09	-9.65E-10
-2.42E-09	-1.60E-09	-3.38E-09	-2.23E-09	-2.65E-09	-2.12E-09	-1.45E-09	-2.51E-09	-2.83E-09	-1.52E-09	-5.25E-10	-1.49E-09	-3.26E-09	-1.69E-09	-2.27E-09	-2.02E-09
-3.20E-09	-1.81E-09	-3.30E-09	-3.06E-09	-3.40E-09	-2.33E-09	-1.52E-09	-2.10E-09	-3.07E-09	-2.56E-09	-2.84E-09	-2.30E-09	-4.03E-09	-2.77E-09	-2.96E-09	-2.91E-09
-2.98E-09	-2.10E-09	-2.92E-09	-3.21E-09	-4.00E-09	-2.80E-09	-1.78E-09	-1.95E-09	-3.70E-09	-3.57E-09	-4.32E-09	-3.96E-09	-4.88E-09	-4.65E-09	-3.62E-09	-3.86E-09
-3.82E-09	-2.72E-09	-2.83E-09	-3.62E-09	-4.42E-09	-3.70E-09	-2.45E-09	-2.19E-09	-4.50E-09	-3.81E-09	-5.28E-09	-5.27E-09	-5.32E-09	-5.61E-09	-4.79E-09	-5.58E-09
-4.92E-09	-3.66E-09	-3.11E-09	-4.38E-09	-5.23E-09	-4.91E-09	-3.84E-09	-3.78E-09	-5.46E-09	-3.76E-09	-5.53E-09	-6.19E-09	-5.43E-09	-5.96E-09	-6.27E-09	-6.88E-09
-5.87E-09	-4.78E-09	-4.09E-09	-4.77E-09	-5.66E-09	-6.39E-09	-5.52E-09	-4.83E-09	-5.56E-09	-4.74E-09	-6.80E-09	-6.91E-09	-5.81E-09	-6.39E-09	-6.96E-09	-7.58E-09
-6.78E-09	-5.71E-09	-4.68E-09	-4.40E-09	-6.26E-09	-6.99E-09	-6.32E-09	-5.59E-09	-5.82E-09	-6.24E-09	-6.76E-09	-7.05E-09	-7.17E-09	-7.19E-09	-7.54E-09	-8.11E-09
-7.41E-09	-6.72E-09	-5.53E-09	-4.92E-09	-6.57E-09	-6.92E-09	-7.52E-09	-6.88E-09	-6.67E-09	-7.34E-09	-7.15E-09	-7.71E-09	-8.42E-09	-8.21E-09	-8.01E-09	-8.05E-09
-7.88E-09	-7.92E-09	-7.23E-09	-6.50E-09	-7.15E-09	-6.93E-09	-8.44E-09	-8.33E-09	-7.51E-09	-8.15E-09	-7.74E-09	-8.53E-09	-9.25E-09	-8.81E-09	-8.33E-09	-8.13E-09
-8.35E-09	-9.44E-09	-8.85E-09	-8.53E-09	-8.23E-09	-7.57E-09	-8.72E-09	-8.94E-09	-8.49E-09	-8.99E-09	-8.85E-09	-9.48E-09	-1.04E-08	-9.56E-09	-8.50E-09	-9.06E-09
-8.27E-09	-9.92E-09	-9.79E-09	-9.83E-09	-9.15E-09	-8.85E-09	-9.43E-09	-9.44E-09	-9.19E-09	-8.95E-09	-9.60E-09	-1.00E-08	-1.09E-08	-1.04E-08	-8.75E-09	-9.84E-09
-7.55E-09	-9.89E-09	-1.03E-08	-1.03E-08	-1.01E-08	-9.15E-09	-1.00E-08	-1.02E-08	-9.55E-09	-9.28E-09	-1.06E-08	-1.04E-08	-1.12E-08	-1.11E-08	-9.78E-09	-1.06E-08
-7.07E-09	-9.98E-09	-1.07E-08	-1.05E-08	-1.00E-08	-9.26E-09	-1.02E-08	-1.05E-08	-9.63E-09	-1.00E-08	-1.12E-08	-1.13E-08	-1.16E-08	-1.12E-08	-1.02E-08	-1.12E-08
-8.17E-09	-1.08E-08	-1.10E-08	-1.06E-08	-9.64E-09	-9.88E-09	-1.04E-08	-9.96E-09	-9.41E-09	-1.09E-08	-1.20E-08	-1.23E-08	-1.26E-08	-1.16E-08	-1.07E-08	-1.16E-08
-9.80E-09	-1.16E-08	-1.13E-08	-1.12E-08	-9.74E-09	-9.55E-09	-1.07E-08	-9.42E-09	-9.42E-09	-1.15E-08	-1.26E-08	-1.25E-08	-1.32E-08	-1.20E-08	-1.06E-08	-1.16E-08
-1.10E-08	-1.25E-08	-1.20E-08	-1.23E-08	-1.10E-08	-9.61E-09	-1.08E-08	-1.00E-08	-1.08E-08	-1.24E-08	-1.30E-08	-1.28E-08	-1.34E-08	-1.20E-08	-1.01E-08	-1.09E-08
-1.15E-08	-1.32E-08	-1.29E-08	-1.33E-08	-1.24E-08	-1.07E-08	-1.13E-08	-1.15E-08	-1.20E-08	-1.29E-08	-1.31E-08	-1.29E-08	-1.36E-08	-1.24E-08	-1.09E-08	-1.10E-08
-1.27E-08	-1.32E-08	-1.34E-08	-1.39E-08	-1.36E-08	-1.25E-08	-1.24E-08	-1.29E-08	-1.25E-08	-1.36E-08	-1.38E-08	-1.33E-08	-1.34E-08	-1.29E-08	-1.20E-08	-1.19E-08
-1.34E-08	-1.40E-08	-1.40E-08	-1.44E-08	-1.45E-08	-1.40E-08	-1.39E-08	-1.38E-08	-1.36E-08	-1.45E-08	-1.49E-08	-1.40E-08	-1.34E-08	-1.33E-08	-1.26E-08	-1.28E-08

-1.45E-08	-1.47E-08	-1.47E-08	-1.46E-08	-1.51E-08	-1.46E-08	-1.48E-08	-1.41E-08	-1.40E-08	-1.54E-08	-1.59E-08	-1.46E-08	-1.44E-08	-1.33E-08	-1.25E-08	-1.27E-08
-1.53E-08	-1.55E-08	-1.53E-08	-1.54E-08	-1.55E-08	-1.51E-08	-1.52E-08	-1.43E-08	-1.42E-08	-1.55E-08	-1.57E-08	-1.48E-08	-1.50E-08	-1.37E-08	-1.25E-08	-1.24E-08
-1.57E-08	-1.62E-08	-1.59E-08	-1.63E-08	-1.60E-08	-1.58E-08	-1.55E-08	-1.47E-08	-1.46E-08	-1.57E-08	-1.55E-08	-1.50E-08	-1.56E-08	-1.44E-08	-1.30E-08	-1.21E-08
-1.61E-08	-1.63E-08	-1.64E-08	-1.67E-08	-1.65E-08	-1.63E-08	-1.59E-08	-1.53E-08	-1.49E-08	-1.60E-08	-1.51E-08	-1.50E-08	-1.57E-08	-1.52E-08	-1.39E-08	-1.31E-08
-1.63E-08	-1.63E-08	-1.65E-08	-1.69E-08	-1.70E-08	-1.70E-08	-1.67E-08	-1.59E-08	-1.51E-08	-1.61E-08	-1.53E-08	-1.48E-08	-1.56E-08	-1.55E-08	-1.50E-08	-1.49E-08
-1.66E-08	-1.66E-08	-1.66E-08	-1.73E-08	-1.75E-08	-1.71E-08	-1.67E-08	-1.64E-08	-1.57E-08	-1.66E-08	-1.63E-08	-1.50E-08	-1.53E-08	-1.54E-08	-1.55E-08	-1.60E-08
-1.74E-08	-1.76E-08	-1.75E-08	-1.81E-08	-1.78E-08	-1.73E-08	-1.68E-08	-1.69E-08	-1.65E-08	-1.71E-08	-1.70E-08	-1.60E-08	-1.56E-08	-1.50E-08	-1.54E-08	-1.61E-08
-1.76E-08	-1.79E-08	-1.82E-08	-1.86E-08	-1.80E-08	-1.74E-08	-1.75E-08	-1.73E-08	-1.76E-08	-1.75E-08	-1.72E-08	-1.65E-08	-1.61E-08	-1.54E-08	-1.57E-08	-1.63E-08
-1.75E-08	-1.80E-08	-1.87E-08	-1.88E-08	-1.83E-08	-1.79E-08	-1.82E-08	-1.78E-08	-1.83E-08	-1.79E-08	-1.74E-08	-1.70E-08	-1.68E-08	-1.69E-08	-1.66E-08	-1.69E-08
-1.78E-08	-1.83E-08	-1.94E-08	-1.91E-08	-1.83E-08	-1.81E-08	-1.84E-08	-1.84E-08	-1.88E-08	-1.82E-08	-1.76E-08	-1.79E-08	-1.72E-08	-1.81E-08	-1.74E-08	-1.79E-08
-1.92E-08	-1.91E-08	-1.98E-08	-1.97E-08	-1.85E-08	-1.83E-08	-1.85E-08	-1.87E-08	-1.92E-08	-1.82E-08	-1.76E-08	-1.82E-08	-1.76E-08	-1.84E-08	-1.81E-08	-1.86E-08
-2.04E-08	-2.00E-08	-2.01E-08	-2.00E-08	-1.86E-08	-1.87E-08	-1.89E-08	-1.91E-08	-1.97E-08	-1.84E-08	-1.81E-08	-1.87E-08	-1.77E-08	-1.86E-08	-1.83E-08	-1.87E-08
-2.12E-08	-2.06E-08	-2.05E-08	-2.02E-08	-1.88E-08	-1.92E-08	-1.92E-08	-1.93E-08	-2.00E-08	-1.86E-08	-1.90E-08	-1.92E-08	-1.82E-08	-1.84E-08	-1.86E-08	-1.85E-08
-2.12E-08	-2.07E-08	-2.08E-08	-2.04E-08	-1.92E-08	-1.97E-08	-1.95E-08	-1.96E-08	-2.02E-08	-1.89E-08	-1.95E-08	-1.94E-08	-1.84E-08	-1.81E-08	-1.88E-08	-1.86E-08
-2.14E-08	-2.07E-08	-2.09E-08	-2.02E-08	-1.91E-08	-1.99E-08	-1.96E-08	-1.99E-08	-2.05E-08	-1.94E-08	-1.96E-08	-1.95E-08	-1.90E-08	-1.85E-08	-1.92E-08	-1.91E-08
-2.15E-08	-2.03E-08	-2.09E-08	-2.00E-08	-1.89E-08	-2.05E-08	-2.02E-08	-2.02E-08	-2.08E-08	-1.99E-08	-1.97E-08	-1.96E-08	-2.00E-08	-1.95E-08	-2.00E-08	-1.94E-08
-2.15E-08	-2.02E-08	-2.10E-08	-2.03E-08	-1.95E-08	-2.13E-08	-2.06E-08	-2.07E-08	-2.09E-08	-2.04E-08	-1.97E-08	-1.99E-08	-2.09E-08	-2.03E-08	-2.10E-08	-1.98E-08
-2.19E-08	-2.09E-08	-2.17E-08	-2.10E-08	-2.05E-08	-2.18E-08	-2.13E-08	-2.13E-08	-2.15E-08	-2.09E-08	-2.08E-08	-2.09E-08	-2.19E-08	-2.14E-08	-2.13E-08	-1.98E-08
-2.28E-08	-2.18E-08	-2.23E-08	-2.21E-08	-2.18E-08	-2.23E-08	-2.22E-08	-2.18E-08	-2.20E-08	-2.15E-08	-2.15E-08	-2.13E-08	-2.24E-08	-2.19E-08	-2.13E-08	-1.99E-08
-2.32E-08	-2.26E-08	-2.30E-08	-2.30E-08	-2.30E-08	-2.31E-08	-2.28E-08	-2.27E-08	-2.24E-08	-2.18E-08	-2.17E-08	-2.14E-08	-2.23E-08	-2.23E-08	-2.20E-08	-2.05E-08
-2.32E-08	-2.29E-08	-2.34E-08	-2.36E-08	-2.36E-08	-2.36E-08	-2.31E-08	-2.35E-08	-2.23E-08	-2.18E-08	-2.15E-08	-2.14E-08	-2.23E-08	-2.27E-08	-2.31E-08	-2.17E-08
-2.34E-08	-2.31E-08	-2.40E-08	-2.37E-08	-2.35E-08	-2.34E-08	-2.35E-08	-2.39E-08	-2.26E-08	-2.17E-08	-2.13E-08	-2.14E-08	-2.27E-08	-2.32E-08	-2.36E-08	-2.26E-08
-2.38E-08	-2.36E-08	-2.47E-08	-2.35E-08	-2.37E-08	-2.36E-08	-2.40E-08	-2.43E-08	-2.36E-08	-2.23E-08	-2.18E-08	-2.22E-08	-2.32E-08	-2.34E-08	-2.40E-08	-2.32E-08
-2.43E-08	-2.43E-08	-2.54E-08	-2.39E-08	-2.45E-08	-2.41E-08	-2.43E-08	-2.48E-08	-2.46E-08	-2.34E-08	-2.35E-08	-2.36E-08	-2.37E-08	-2.35E-08	-2.40E-08	-2.34E-08
-2.49E-08	-2.50E-08	-2.55E-08	-2.49E-08	-2.54E-08	-2.48E-08	-2.46E-08	-2.51E-08	-2.51E-08	-2.48E-08	-2.47E-08	-2.42E-08	-2.45E-08	-2.37E-08	-2.38E-08	-2.35E-08
-2.59E-08	-2.58E-08	-2.59E-08	-2.56E-08	-2.61E-08	-2.56E-08	-2.52E-08	-2.55E-08	-2.53E-08	-2.58E-08	-2.56E-08	-2.47E-08	-2.52E-08	-2.41E-08	-2.39E-08	-2.33E-08
-2.69E-08	-2.69E-08	-2.60E-08	-2.57E-08	-2.68E-08	-2.61E-08	-2.54E-08	-2.59E-08	-2.56E-08	-2.65E-08	-2.63E-08	-2.51E-08	-2.60E-08	-2.50E-08	-2.42E-08	-2.38E-08
-2.76E-08	-2.75E-08	-2.61E-08	-2.57E-08	-2.70E-08	-2.61E-08	-2.54E-08	-2.61E-08	-2.67E-08	-2.68E-08	-2.68E-08	-2.56E-08	-2.63E-08	-2.56E-08	-2.48E-08	-2.45E-08
-2.82E-08	-2.80E-08	-2.65E-08	-2.63E-08	-2.73E-08	-2.61E-08	-2.53E-08	-2.65E-08	-2.73E-08	-2.70E-08	-2.63E-08	-2.55E-08	-2.68E-08	-2.58E-08	-2.56E-08	-2.61E-08
-2.86E-08	-2.84E-08	-2.71E-08	-2.72E-08	-2.76E-08	-2.67E-08	-2.57E-08	-2.64E-08	-2.72E-08	-2.74E-08	-2.68E-08	-2.60E-08	-2.72E-08	-2.59E-08	-2.60E-08	-2.69E-08
-2.90E-08	-2.87E-08	-2.82E-08	-2.80E-08	-2.77E-08	-2.69E-08	-2.61E-08	-2.63E-08	-2.75E-08	-2.79E-08	-2.70E-08	-2.66E-08	-2.77E-08	-2.69E-08	-2.56E-08	-2.48E-08
-2.95E-08	-2.92E-08	-2.90E-08	-2.86E-08	-2.84E-08	-2.78E-08	-2.70E-08	-2.70E-08	-2.78E-08	-2.83E-08	-2.72E-08	-2.69E-08	-2.78E-08	-2.78E-08	-2.63E-08	-2.51E-08
-2.95E-08	-2.96E-08	-2.96E-08	-2.93E-08	-2.91E-08	-2.87E-08	-2.82E-08	-2.82E-08	-2.83E-08	-2.85E-08	-2.75E-08	-2.73E-08	-2.80E-08	-2.85E-08	-2.72E-08	-2.62E-08
-2.97E-08	-2.98E-08	-3.00E-08	-2.92E-08	-2.92E-08	-2.91E-08	-2.91E-08	-2.85E-08	-2.85E-08	-2.89E-08	-2.80E-08	-2.77E-08	-2.81E-08	-2.86E-08	-2.78E-08	-2.68E-08
-3.01E-08	-3.01E-08	-3.00E-08	-2.94E-08	-2.93E-08	-2.92E-08	-2.94E-08	-2.86E-08	-2.85E-08	-2.90E-08	-2.84E-08	-2.81E-08	-2.83E-08	-2.87E-08	-2.76E-08	-2.67E-08
-3.05E-08	-3.04E-08	-3.04E-08	-2.99E-08	-3.01E-08	-2.99E-08	-2.95E-08	-2.89E-08	-2.83E-08	-2.90E-08	-2.85E-08	-2.82E-08	-2.82E-08	-2.87E-08	-2.75E-08	-2.68E-08
-3.08E-08	-3.09E-08	-3.10E-08	-3.02E-08	-3.06E-08	-3.02E-08	-2.96E-08	-2.91E-08	-2.82E-08	-2.87E-08	-2.80E-08	-2.80E-08	-2.80E-08	-2.86E-08	-2.75E-08	-2.72E-08
-3.09E-08	-3.13E-08	-3.17E-08	-3.05E-08	-3.07E-08	-3.00E-08	-2.96E-08	-2.91E-08	-2.81E-08	-2.87E-08	-2.79E-08	-2.80E-08	-2.78E-08	-2.84E-08	-2.77E-08	-2.75E-08
-3.08E-08	-3.11E-08	-3.17E-08	-3.10E-08	-3.09E-08	-2.99E-08	-2.97E-08	-2.91E-08	-2.78E-08	-2.86E-08	-2.79E-08	-2.81E-08	-2.81E-08	-2.84E-08	-2.78E-08	-2.75E-08
-3.10E-08	-3.10E-08	-3.13E-08	-3.10E-08	-3.09E-08	-3.00E-08	-3.01E-08	-2.92E-08	-2.76E-08	-2.84E-08	-2.79E-08	-2.82E-08	-2.83E-08	-2.87E-08	-2.81E-08	-2.78E-08
-3.10E-08	-3.10E-08	-3.07E-08	-3.08E-08	-3.07E-08	-3.04E-08	-3.02E-08	-2.93E-08	-2.82E-08	-2.85E-08	-2.78E-08	-2.81E-08	-2.85E-08	-2.87E-08	-2.81E-08	-2.83E-08
-3.15E-08	-3.17E-08	-3.02E-08	-3.04E-08	-3.07E-08	-3.07E-08	-3.05E-08	-2.94E-08	-2.92E-08	-2.88E-08	-2.79E-08	-2.83E-08	-2.87E-08	-2.85E-08	-2.85E-08	-2.85E-08
-3.19E-08	-3.22E-08	-2.98E-08	-3.04E-08	-3.09E-08	-3.10E-08	-3.05E-08	-2.98E-08	-2.96E-08	-2.87E-08	-2.79E-08	-2.87E-08	-2.90E-08	-2.85E-08	-2.87E-08	-2.83E-08
-3.24E-08	-3.24E-08	-3.05E-08	-3.08E-08	-3.09E-08	-3.09E-08	-3.06E-08	-2.99E-08	-2.96E-08	-2.87E-08	-2.82E-08	-2.90E-08	-2.90E-08	-2.83E-08	-2.87E-08	-2.84E-08
-3.28E-08	-3.26E-08	-3.09E-08	-3.08E-08	-3.08E-08	-3.08E-08	-3.11E-08	-3.05E-08	-3.01E-08	-2.91E-08	-2.83E-08	-2.93E-08	-2.87E-08	-2.82E-08	-2.87E-08	-2.87E-08
-3.28E-08	-3.31E-08	-3.12E-08	-3.09E-08	-3.09E-08	-3.05E-08	-3.10E-08	-3.10E-08	-3.01E-08	-2.99E-08	-2.83E-08	-2.91E-08	-2.83E-08	-2.84E-08	-2.88E-08	-2.89E-08
-3.20E-08	-3.31E-08	-3.19E-08	-3.13E-08	-3.11E-08	-3.10E-08	-3.07E-08	-3.08E-08	-2.97E-08	-2.98E-08	-2.84E-08	-2.91E-08	-2.85E-08	-2.87E-08	-2.91E-08	-2.92E-08
-3.26E-08	-3.30E-08	-3.29E-08	-3.16E-08	-3.13E-08	-3.14E-08	-3.06E-08	-3.03E-08	-2.93E-08	-2.92E-08	-2.84E-08	-2.92E-08	-2.95E-08	-2.91E-08	-2.92E-08	-2.92E-08
-3.25E-08	-3.27E-08	-3.24E-08	-3.14E-08	-3.11E-08	-3.13E-08	-3.07E-08	-2.98E-08	-2.89E-08	-2.85E-08	-2.86E-08	-2.96E-08	-3.00E-08	-2.91E-08	-2.85E-08	-2.89E-08
-3.18E-08	-3.20E-08	-3.19E-08	-3.11E-08	-3.11E-08	-3.12E-08	-3.13E-08	-3.03E-08	-2.92E-08	-2.86E-08	-2.90E-08	-3.00E-08	-3.00E-08	-2.93E-08	-2.79E-08	-2.86E-08

2.88E-15	2.87E-15	2.89E-15	2.76E-15	2.71E-15	2.66E-15	2.83E-15	2.80E-15	2.85E-15	2.71E-15	2.67E-15	2.79E-15	2.73E-15	2.65E-15	2.55E-15	2.47E-15
2.77E-15	2.71E-15	2.71E-15	2.66E-15	2.57E-15	2.51E-15	2.69E-15	2.61E-15	2.69E-15	2.51E-15	2.52E-15	2.56E-15	2.63E-15	2.53E-15	2.43E-15	2.38E-15
2.65E-15	2.60E-15	2.60E-15	2.59E-15	2.45E-15	2.39E-15	2.52E-15	2.47E-15	2.61E-15	2.36E-15	2.41E-15	2.40E-15	2.46E-15	2.41E-15	2.30E-15	2.29E-15
2.52E-15	2.51E-15	2.51E-15	2.45E-15	2.34E-15	2.28E-15	2.39E-15	2.40E-15	2.41E-15	2.25E-15	2.28E-15	2.24E-15	2.24E-15	2.27E-15	2.16E-15	2.14E-15
2.41E-15	2.36E-15	2.41E-15	2.32E-15	2.25E-15	2.20E-15	2.25E-15	2.36E-15	2.25E-15	2.18E-15	2.16E-15	2.08E-15	2.10E-15	2.11E-15	2.02E-15	1.99E-15
2.24E-15	2.22E-15	2.24E-15	2.22E-15	2.13E-15	2.08E-15	2.17E-15	2.32E-15	2.21E-15	2.11E-15	2.03E-15	1.90E-15	1.99E-15	2.00E-15	1.91E-15	1.88E-15
2.11E-15	2.14E-15	2.08E-15	2.11E-15	2.04E-15	2.00E-15	2.12E-15	2.21E-15	2.14E-15	2.02E-15	1.90E-15	1.78E-15	1.88E-15	1.90E-15	1.79E-15	1.79E-15
1.98E-15	2.03E-15	1.98E-15	2.06E-15	1.97E-15	1.92E-15	2.05E-15	2.08E-15	2.02E-15	1.90E-15	1.74E-15	1.68E-15	1.76E-15	1.73E-15	1.70E-15	1.70E-15
1.86E-15	1.92E-15	1.88E-15	1.84E-15	1.87E-15	1.81E-15	1.93E-15	1.87E-15	1.79E-15	1.77E-15	1.65E-15	1.60E-15	1.67E-15	1.60E-15	1.60E-15	1.60E-15
1.75E-15	1.76E-15	1.76E-15	1.69E-15	1.78E-15	1.72E-15	1.75E-15	1.67E-15	1.63E-15	1.58E-15	1.57E-15	1.52E-15	1.59E-15	1.52E-15	1.53E-15	1.54E-15
1.65E-15	1.66E-15	1.62E-15	1.58E-15	1.70E-15	1.59E-15	1.58E-15	1.52E-15	1.49E-15	1.44E-15	1.51E-15	1.42E-15	1.53E-15	1.45E-15	1.47E-15	1.46E-15
1.55E-15	1.53E-15	1.51E-15	1.49E-15	1.57E-15	1.48E-15	1.47E-15	1.41E-15	1.36E-15	1.37E-15	1.43E-15	1.35E-15	1.45E-15	1.37E-15	1.40E-15	1.37E-15
1.42E-15	1.42E-15	1.37E-15	1.41E-15	1.48E-15	1.39E-15	1.39E-15	1.34E-15	1.28E-15	1.29E-15	1.32E-15	1.28E-15	1.37E-15	1.31E-15	1.31E-15	1.30E-15
1.32E-15	1.35E-15	1.28E-15	1.36E-15	1.36E-15	1.30E-15	1.35E-15	1.31E-15	1.24E-15	1.19E-15	1.20E-15	1.20E-15	1.28E-15	1.20E-15	1.22E-15	1.24E-15
1.21E-15	1.24E-15	1.24E-15	1.34E-15	1.26E-15	1.23E-15	1.28E-15	1.31E-15	1.26E-15	1.16E-15	1.13E-15	1.13E-15	1.22E-15	1.13E-15	1.14E-15	1.19E-15
1.14E-15	1.15E-15	1.18E-15	1.27E-15	1.20E-15	1.18E-15	1.19E-15	1.29E-15	1.23E-15	1.16E-15	1.11E-15	1.10E-15	1.17E-15	1.11E-15	1.09E-15	1.13E-15
1.08E-15	1.09E-15	1.13E-15	1.23E-15	1.17E-15	1.11E-15	1.11E-15	1.18E-15	1.12E-15	1.11E-15	1.07E-15	1.06E-15	1.13E-15	1.07E-15	1.04E-15	1.06E-15
1.04E-15	1.09E-15	1.09E-15	1.23E-15	1.14E-15	1.08E-15	1.03E-15	1.03E-15	1.00E-15	1.04E-15	1.01E-15	1.03E-15	1.08E-15	1.03E-15	9.85E-16	1.02E-15
9.91E-16	1.08E-15	1.07E-15	1.25E-15	1.08E-15	1.07E-15	9.66E-16	9.25E-16	8.98E-16	9.73E-16	9.56E-16	9.95E-16	9.91E-16	9.75E-16	9.23E-16	9.34E-16
9.25E-16	9.99E-16	9.75E-16	1.09E-15	1.02E-15	1.04E-15	9.11E-16	8.42E-16	8.46E-16	9.12E-16	9.15E-16	9.33E-16	8.79E-16	9.02E-16	8.46E-16	8.09E-16
8.27E-16	8.67E-16	8.59E-16	9.36E-16	9.35E-16	9.68E-16	8.41E-16	7.63E-16	7.88E-16	8.61E-16	8.79E-16	8.76E-16	8.29E-16	8.10E-16	7.67E-16	7.44E-16
7.84E-16	7.76E-16	7.71E-16	8.34E-16	8.45E-16	8.85E-16	7.88E-16	6.99E-16	7.40E-16	7.98E-16	7.99E-16	8.21E-16	7.87E-16	7.47E-16	6.96E-16	6.83E-16
7.35E-16	7.47E-16	7.44E-16	7.85E-16	7.66E-16	8.05E-16	7.20E-16	6.47E-16	7.03E-16	7.33E-16	7.15E-16	7.43E-16	7.23E-16	6.80E-16	6.54E-16	6.50E-16
6.83E-16	6.95E-16	7.05E-16	7.05E-16	6.70E-16	7.09E-16	6.43E-16	5.95E-16	6.48E-16	6.83E-16	6.61E-16	6.82E-16	6.64E-16	6.21E-16	6.14E-16	6.19E-16
6.17E-16	6.32E-16	6.52E-16	6.65E-16	5.98E-16	6.12E-16	5.66E-16	5.54E-16	5.90E-16	6.13E-16	6.02E-16	6.18E-16	6.07E-16	5.77E-16	5.61E-16	5.93E-16
5.91E-16	6.02E-16	5.90E-16	6.01E-16	5.42E-16	5.43E-16	5.32E-16	5.20E-16	5.26E-16	5.56E-16	5.52E-16	5.61E-16	5.49E-16	5.61E-16	5.15E-16	5.16E-16
5.72E-16	5.67E-16	5.44E-16	5.44E-16	5.09E-16	4.90E-16	5.08E-16	4.82E-16	4.87E-16	5.07E-16	4.90E-16	5.29E-16	5.37E-16	5.43E-16	4.83E-16	4.66E-16
5.50E-16	5.30E-16	5.18E-16	4.92E-16	4.77E-16	4.53E-16	4.71E-16	4.32E-16	4.25E-16	4.43E-16	4.47E-16	4.87E-16	5.14E-16	4.85E-16	4.46E-16	4.33E-16
4.99E-16	4.73E-16	4.68E-16	4.36E-16	4.45E-16	4.10E-16	4.22E-16	3.99E-16	3.96E-16	3.95E-16	4.16E-16	4.61E-16	4.97E-16	4.41E-16	4.16E-16	4.10E-16
4.52E-16	4.26E-16	4.20E-16	4.10E-16	4.30E-16	3.97E-16	3.90E-16	3.75E-16	3.77E-16	3.70E-16	4.02E-16	4.57E-16	4.70E-16	4.11E-16	3.98E-16	3.84E-16
4.10E-16	4.01E-16	3.97E-16	3.77E-16	3.87E-16	3.71E-16	3.68E-16	3.41E-16	3.66E-16	3.55E-16	3.64E-16	4.25E-16	4.32E-16	3.91E-16	3.68E-16	3.47E-16

3.79E-16	3.73E-16	3.73E-16	3.34E-16	3.48E-16	3.56E-16	3.36E-16	3.09E-16	3.49E-16	3.31E-16	3.24E-16	3.62E-16	3.80E-16	3.62E-16	3.29E-16	3.04E-16
3.60E-16	3.40E-16	3.32E-16	3.00E-16	3.26E-16	3.43E-16	3.12E-16	2.92E-16	3.25E-16	3.02E-16	3.11E-16	3.11E-16	3.34E-16	3.10E-16	2.92E-16	2.61E-16
3.36E-16	3.11E-16	2.93E-16	2.67E-16	3.16E-16	3.31E-16	2.87E-16	2.79E-16	3.04E-16	2.79E-16	3.07E-16	2.83E-16	2.85E-16	2.70E-16	2.67E-16	2.29E-16
3.05E-16	2.79E-16	2.71E-16	2.44E-16	2.89E-16	3.05E-16	2.79E-16	2.74E-16	2.78E-16	2.47E-16	2.89E-16	2.68E-16	2.46E-16	2.41E-16	2.33E-16	2.17E-16
2.57E-16	2.46E-16	2.35E-16	2.13E-16	2.53E-16	2.61E-16	2.45E-16	2.50E-16	2.51E-16	2.08E-16	2.36E-16	2.36E-16	2.07E-16	2.10E-16	2.01E-16	2.04E-16
2.23E-16	2.00E-16	2.00E-16	1.89E-16	2.20E-16	2.10E-16	2.09E-16	2.27E-16	2.27E-16	1.85E-16	1.89E-16	1.86E-16	1.79E-16	1.67E-16	1.67E-16	1.82E-16
2.03E-16	1.73E-16	1.76E-16	1.72E-16	1.98E-16	1.66E-16	1.64E-16	2.05E-16	1.95E-16	1.62E-16	1.72E-16	1.54E-16	1.53E-16	1.43E-16	1.42E-16	1.55E-16
1.97E-16	1.68E-16	1.66E-16	1.66E-16	1.60E-16	1.37E-16	1.28E-16	1.59E-16	1.55E-16	1.50E-16	1.59E-16	1.50E-16	1.33E-16	1.24E-16	1.36E-16	1.46E-16
1.76E-16	1.71E-16	1.70E-16	1.49E-16	1.44E-16	1.20E-16	1.06E-16	1.23E-16	1.23E-16	1.48E-16	1.56E-16	1.41E-16	1.18E-16	1.05E-16	1.20E-16	1.20E-16
1.67E-16	1.57E-16	1.62E-16	1.30E-16	1.26E-16	1.04E-16	8.82E-17	1.06E-16	1.03E-16	1.30E-16	1.47E-16	1.15E-16	1.13E-16	9.21E-17	1.09E-16	9.97E-17
1.53E-16	1.29E-16	1.43E-16	1.19E-16	1.12E-16	9.88E-17	8.21E-17	9.88E-17	9.28E-17	1.00E-16	1.21E-16	1.07E-16	1.01E-16	8.79E-17	9.80E-17	8.75E-17
1.11E-16	9.29E-17	1.19E-16	8.40E-17	8.07E-17	7.45E-17	7.15E-17	8.70E-17	7.56E-17	6.98E-17	8.11E-17	8.62E-17	7.87E-17	7.63E-17	7.49E-17	6.58E-17
7.85E-17	7.88E-17	8.66E-17	6.03E-17	5.92E-17	6.20E-17	6.11E-17	9.13E-17	6.90E-17	5.72E-17	6.48E-17	7.29E-17	6.12E-17	6.26E-17	6.76E-17	6.07E-17
7.03E-17	6.93E-17	6.28E-17	4.31E-17	4.61E-17	5.50E-17	4.65E-17	6.98E-17	6.29E-17	4.95E-17	4.99E-17	5.71E-17	4.71E-17	5.92E-17	5.69E-17	4.68E-17
6.75E-17	6.57E-17	5.05E-17	3.31E-17	3.74E-17	4.72E-17	3.78E-17	4.95E-17	4.66E-17	3.98E-17	4.10E-17	4.10E-17	4.13E-17	5.34E-17	4.19E-17	3.29E-17
6.35E-17	5.73E-17	4.01E-17	2.73E-17	3.40E-17	4.37E-17	3.61E-17	4.33E-17	3.65E-17	3.99E-17	3.91E-17	3.11E-17	4.52E-17	4.77E-17	3.41E-17	2.72E-17
4.89E-17	4.57E-17	3.09E-17	2.24E-17	2.92E-17	3.47E-17	3.31E-17	3.56E-17	2.81E-17	3.91E-17	3.65E-17	2.91E-17	3.96E-17	3.98E-17	2.73E-17	2.30E-17
3.65E-17	2.98E-17	2.24E-17	1.89E-17	2.30E-17	2.21E-17	2.63E-17	2.47E-17	2.45E-17	3.40E-17	2.90E-17	2.34E-17	2.57E-17	3.33E-17	2.23E-17	2.12E-17
2.75E-17	2.29E-17	1.75E-17	1.66E-17	2.14E-17	1.66E-17	2.02E-17	1.84E-17	2.22E-17	2.92E-17	2.02E-17	1.47E-17	1.42E-17	2.11E-17	1.94E-17	1.84E-17
1.92E-17	1.64E-17	1.32E-17	1.49E-17	1.69E-17	1.56E-17	1.70E-17	1.53E-17	1.52E-17	2.01E-17	1.13E-17	7.25E-18	1.09E-17	1.06E-17	1.34E-17	1.27E-17
1.17E-17	1.05E-17	1.06E-17	1.03E-17	1.21E-17	1.40E-17	1.14E-17	9.22E-18	6.14E-18	9.16E-18	6.25E-18	3.46E-18	6.11E-18	5.19E-18	6.80E-18	9.02E-18
6.83E-18	8.40E-18	9.99E-18	6.39E-18	7.42E-18	1.24E-17	9.04E-18	6.86E-18	4.22E-18	4.73E-18	2.54E-18	1.74E-18	2.19E-18	2.87E-18	3.92E-18	7.75E-18
5.25E-18	9.75E-18	1.31E-17	4.75E-18	5.24E-18	8.82E-18	8.86E-18	5.10E-18	2.67E-18	3.48E-18	1.37E-18	5.86E-19	7.57E-19	8.76E-19	1.39E-18	5.73E-18
4.21E-18	1.01E-17	1.53E-17	7.10E-18	5.34E-18	6.41E-18	6.62E-18	3.12E-18	2.17E-18	3.86E-18	5.72E-19	8.37E-21	2.54E-19	2.93E-19	1.77E-19	2.62E-18
2.61E-18	8.08E-18	1.48E-17	8.54E-18	4.10E-18	4.61E-18	2.47E-18	5.40E-19	2.38E-18	2.06E-18	2.50E-19	8.89E-21	5.98E-20	6.33E-19	4.53E-21	2.50E-19
9.98E-19	4.64E-18	4.52E-18	5.04E-18	2.61E-18	2.78E-18	3.66E-19	2.00E-19	4.23E-19	4.94E-19	6.35E-20	1.32E-21	5.22E-21	1.28E-18	3.95E-19	3.45E-19
1.21E-19	5.26E-20	8.40E-19	3.05E-20	8.79E-21	1.10E-19	1.18E-18	3.71E-18	7.61E-19	6.84E-24	1.07E-19	5.96E-19	1.32E-18	6.99E-20	1.43E-18	1.09E-18
1.84E-18	6.58E-19	6.43E-18	1.66E-18	2.65E-18	1.77E-18	2.52E-18	6.26E-18	4.70E-18	4.85E-19	6.04E-20	2.09E-18	5.47E-18	3.76E-19	2.82E-18	9.31E-19
5.88E-18	2.58E-18	1.14E-17	4.96E-18	7.04E-18	4.50E-18	2.11E-18	6.31E-18	8.03E-18	2.31E-18	2.76E-19	2.22E-18	1.06E-17	2.84E-18	5.17E-18	4.09E-18
1.03E-17	3.28E-18	1.09E-17	9.38E-18	1.16E-17	5.43E-18	2.32E-18	4.42E-18	9.44E-18	6.55E-18	8.04E-18	5.28E-18	1.63E-17	7.69E-18	8.75E-18	8.47E-18
8.89E-18	4.43E-18	8.52E-18	1.03E-17	1.60E-17	7.82E-18	3.17E-18	3.80E-18	1.37E-17	1.28E-17	1.86E-17	1.57E-17	2.38E-17	2.16E-17	1.31E-17	1.49E-17
1.46E-17	7.41E-18	8.01E-18	1.31E-17	1.95E-17	1.37E-17	6.01E-18	4.78E-18	2.03E-17	1.45E-17	2.79E-17	2.77E-17	2.83E-17	3.14E-17	2.30E-17	3.12E-17
2.42E-17	1.34E-17	9.69E-18	1.92E-17	2.73E-17	2.41E-17	1.48E-17	1.43E-17	2.98E-17	1.41E-17	3.05E-17	3.83E-17	2.95E-17	3.55E-17	3.93E-17	4.74E-17
3.44E-17	2.28E-17	1.67E-17	2.28E-17	3.20E-17	4.08E-17	3.05E-17	2.33E-17	3.09E-17	2.25E-17	4.62E-17	4.77E-17	3.37E-17	4.08E-17	4.84E-17	5.74E-17
4.59E-17	3.26E-17	2.19E-17	1.94E-17	3.92E-17	4.89E-17	3.99E-17	3.13E-17	3.39E-17	3.89E-17	4.57E-17	4.97E-17	5.14E-17	5.16E-17	5.68E-17	6.58E-17
5.49E-17	4.52E-17	3.06E-17	2.42E-17	4.32E-17	4.79E-17	5.66E-17	4.74E-17	4.45E-17	5.39E-17	5.11E-17	5.94E-17	7.09E-17	6.75E-17	6.42E-17	6.48E-17
6.20E-17	6.28E-17	5.22E-17	4.22E-17	5.11E-17	4.80E-17	7.12E-17	6.94E-17	5.64E-17	6.63E-17	5.99E-17	7.28E-17	8.56E-17	7.76E-17	6.94E-17	6.61E-17
6.97E-17	8.91E-17	7.83E-17	7.27E-17	6.78E-17	5.73E-17	7.61E-17	8.00E-17	7.20E-17	8.08E-17	7.83E-17	8.99E-17	1.08E-16	9.14E-17	7.22E-17	8.22E-17
6.84E-17	9.83E-17	9.59E-17	9.67E-17	8.37E-17	7.83E-17	8.89E-17	8.91E-17	8.44E-17	8.02E-17	9.22E-17	1.00E-16	1.19E-16	1.09E-16	7.66E-17	9.69E-17
5.70E-17	9.79E-17	1.07E-16	1.05E-16	1.02E-16	8.37E-17	1.01E-16	1.03E-16	9.12E-17	8.62E-17	1.13E-16	1.08E-16	1.26E-16	1.23E-16	9.57E-17	1.13E-16
5.00E-17	9.97E-17	1.14E-16	1.10E-16	1.00E-16	8.57E-17	1.05E-16	1.10E-16	9.27E-17	1.01E-16	1.26E-16	1.28E-16	1.34E-16	1.26E-16	1.04E-16	1.26E-16
6.67E-17	1.16E-16	1.20E-16	1.12E-16	9.30E-17	9.76E-17	1.08E-16	9.93E-17	8.85E-17	1.19E-16	1.44E-16	1.51E-16	1.58E-16	1.35E-16	1.15E-16	1.35E-16
9.61E-17	1.34E-16	1.29E-16	1.26E-16	9.49E-17	9.13E-17	1.14E-16	8.88E-17	8.87E-17	1.32E-16	1.59E-16	1.57E-16	1.74E-16	1.44E-16	1.12E-16	1.33E-16
1.22E-16	1.56E-16	1.43E-16	1.52E-16	1.21E-16	9.24E-17	1.16E-16	1.00E-16	1.17E-16	1.54E-16	1.70E-16	1.63E-16	1.79E-16	1.45E-16	1.02E-16	1.20E-16
1.32E-16	1.74E-16	1.65E-16	1.76E-16	1.55E-16	1.14E-16	1.27E-16	1.32E-16	1.43E-16	1.67E-16	1.71E-16	1.66E-16	1.86E-16	1.53E-16	1.18E-16	1.22E-16
1.61E-16	1.74E-16	1.81E-16	1.94E-16	1.86E-16	1.57E-16	1.54E-16	1.65E-16	1.56E-16	1.84E-16	1.91E-16	1.76E-16	1.80E-16	1.66E-16	1.44E-16	1.43E-16
1.81E-16	1.95E-16	1.97E-16	2.07E-16	2.11E-16	1.95E-16	1.95E-16	1.90E-16	1.85E-16	2.09E-16	2.22E-16	1.95E-16	1.80E-16	1.78E-16	1.59E-16	1.64E-16
2.12E-16	2.17E-16	2.16E-16	2.14E-16	2.27E-16	2.12E-16	2.20E-16	1.98E-16	1.96E-16	2.37E-16	2.54E-16	2.12E-16	2.07E-16	1.76E-16	1.55E-16	1.61E-16
2.33E-16	2.40E-16	2.35E-16	2.38E-16	2.41E-16	2.27E-16	2.30E-16	2.03E-16	2.03E-16	2.41E-16	2.46E-16	2.18E-16	2.26E-16	1.89E-16	1.55E-16	1.53E-16
2.47E-16	2.63E-16	2.53E-16	2.66E-16	2.56E-16	2.49E-16	2.42E-16	2.17E-16	2.13E-16	2.47E-16	2.39E-16	2.26E-16	2.42E-16	2.07E-16	1.69E-16	1.47E-16
2.58E-16	2.65E-16	2.70E-16	2.79E-16	2.72E-16	2.67E-16	2.53E-16	2.34E-16	2.22E-16	2.56E-16	2.29E-16	2.25E-16	2.46E-16	2.30E-16	1.93E-16	1.72E-16
2.66E-16	2.66E-16	2.72E-16	2.87E-16	2.89E-16	2.88E-16	2.77E-16	2.53E-16	2.29E-16	2.61E-16	2.35E-16	2.20E-16	2.43E-16	2.40E-16	2.24E-16	2.23E-16
2.77E-16	2.77E-16	2.75E-16	2.99E-16	3.05E-16	2.93E-16	2.80E-16	2.69E-16	2.47E-16	2.75E-16	2.66E-16	2.26E-16	2.34E-16	2.37E-16	2.40E-16	2.56E-16
3.04E-16	3.08E-16	3.07E-16	3.28E-16	3.16E-16	2.99E-16	2.83E-16	2.85E-16	2.73E-16	2.92E-16	2.90E-16	2.55E-16	2.42E-16	2.26E-16	2.39E-16	2.59E-16
3.11E-16	3.21E-16	3.32E-16	3.45E-16	3.22E-16	3.03E-16	3.05E-16	2.99E-16	3.09E-16	3.08E-16	2.94E-16	2.72E-16	2.60E-16	2.37E-16	2.48E-16	2.65E-16
3.05E-16	3.25E-16	3.49E-16	3.54E-16	3.36E-16	3.19E-16	3.29E-16	3.17E-16	3.35E-16	3.19E-16	3.03E-16	2.90E-16	2.82E-16	2.86E-16	2.76E-16	2.86E-16
3.17E-16	3.33E-16	3.75E-16	3.66E-16	3.35E-16	3.28E-16	3.37E-16	3.38E-16	3.52E-16	3.29E-16	3.09E-16	3.21E-16	2.97E-16	3.26E-16	3.03E-16	3.22E-16
3.70E-16	3.65E-16	3.94E-16	3.87E-16	3.43E-16	3.36E-16	3.41E-16	3.49E-16	3.68E-16	3.32E-16	3.10E-16	3.30E-16	3.09E-16	3.37E-16	3.29E-16	3.48E-16
4.14E-16	4.01E-16	4.04E-16	3.98E-16	3.45E-16	3.50E-16	3.56E-16	3.64E-16	3.88E-16	3.40E-16	3.27E-16	3.49E-16	3.13E-16	3.46E-16	3.36E-16	3.50E-16
4.51E-16	4.25E-16	4.19E-16	4.10E-16	3.55E-16	3.69E-16	3.67E-16	3.72E-16	4.02E-16	3.47E-16	3.59E-16	3.67E-16	3.32E-16	3.38E-16	3.45E-16	3.42E-16
4.50E-16	4.27E-16	4.34E-16	4.15E-16	3.69E-16	3.90E-16	3.81E-16	3.84E-16	4.10E-16	3.59E-16	3.81E-16	3.76E-16	3.38E-16	3.29E-16	3.54E-16	3.45E-16
4.57E-16	4.29E-16	4.37E-16	4.07E-16	3.63E-16	3.97E-16	3.85E-16	3.97E-16	4.21E-16	3.77E-16	3.83E-16	3.82E-16	3.61E-16	3.44E-16	3.68E-16	3.65E-16
4.63E-16	4.10E-16	4.37E-16	4.01E-16	3.55E-16	4.20E-16	4.07E-16	4.08E-16	4.31E-16	3.94E-16	3.87E-16	3.83E-16	4.01E-16	3.78E-16	3.99E-16	3.78E-16
4.63E-16	4.06E-16	4.40E-16	4.12E-16	3.82E-16	4.54E-16	4.26E-16	4.28E-16	4.37E-16	4.15E-16	3.89E-16	3.95E-16	4.36E-16	4.14E-16	4.41E-16	3.91E-16
4.79E-16	4.35E-16	4.69E-16	4.43E-16	4.20E-16	4.74E-16	4.56E-16	4.52E-16	4.61E-16	4.35E-16	4.34E-16	4.37E-16	4.79E-16	4.57E-16	4.53E-16	3.93E-16
5.21E-16	4.75E-16	4.98E-16	4.89E-16	4.76E-16	4.98E-16	4.93E-16	4.76E-16	4.84E-16	4.63E-16	4.61E-16	4.52E-16	5.04E-16	4.78E-16	4.54E-16	3.95E-16
5.39E-16	5.13E-16	5.29E-16	5.31E-16	5.28E-16	5.35E-16	5.18E-16	5.14E-16	5.03E-16	4.75E-16	4.71E-16	4.57E-16	4.97E-16	4.96E-16	4.83E-16	4.20E-16
5.40E-16	5.24E-16	5.49E-16	5.57E-16	5.55E-16	5.58E-16	5.35E-16	5.51E-16	4.97E-16	4.76E-16	4.62E-16	4.59E-16	4.97E-16	5.17E-16	5.34E-16	4.71E-16
5.45E-16	5.32E-16	5.78E-16	5.63E-16	5.53E-16	5.47E-16	5.51E-16	5.73E-16	5.12E-16	4.73E-16	4.53E-16	4.59E-16	5.13E-16	5.38E-16	5.57E-16	5.10E-16
5.67E-16	5.57E-16	6.10E-16	5.51E-16	5.63E-16	5.59E-16	5.75E-16	5.90E-16	5.57E-16	4.95E-16	4.74E-16	4.95E-16	5.37E-16	5.49E-16	5.76E-16	5.39E-16
5.91E-16	5.91E-16	6.44E-16	5.72E-16	5.98E-16	5.81E-16	5.93E-16	6.15E-16	6.06E-16	5.49E-16	5.52E-16	5.55E-16	5.63E-16	5.53E-16	5.76E-16	5.48E-16
6.18E-16	6.25E-16	6.51E-16	6.18E-16	6.43E-16	6.15E-16	6.05E-16	6.30E-16	6.31E-16	6.16E-16	6.12E-16	5.86E-16	6.02E-16	5.61E-16	5.64E-16	5.51E-16
6.71E-16	6.64E-16	6.71E-16	6.54E-16	6.82E-16	6.58E-16	6.33E-16	6.52E-16	6.40E-16	6.67E-16	6.57E-16	6.09E-16	6.33E-16	5.80E-16	5.70E-16	5.45E-16
7.22E-16	7.23E-16	6.76E-16	6.58E-16	7.16E-16	6.83E-16	6.47E-16	6.71E-16	6.53E-16	7.00E-16	6.93E-16	6.32E-16	6.76E-16	6.26E-16	5.84E-16	5.68E-16
7.62E-16	7.59E-16	6.80E-16	6.63E-16	7.27E-16	6.82E-16	6.45E-16	6.82E-16	7.12E-16	7.17E-16	7.19E-16	6.54E-16	6.91E-16	6.58E-16	6.13E-16	6.02E-16
7.94E-16	7.85E-16	7.02E-16	6.92E-16	7.45E-16	6.82E-16	6.39E-16	7.00E-16	7.48E-16	7.27E-16	6.93E-16	6.52E-16	7.17E-16	6.67E-16	6.57E-16	6.79E-16
8.17E-16	8.08E-16	7.34E-16	7.39E-16	7.63E-16	7.13E-16	6.59E-16	6.97E-16	7.41E-16	7.48E-16	7.18E-16	6.78E-16	7.41E-16	6.73E-16	6.75E-16	7.21E-16
8.40E-16	8.27E-16	7.98E-16	7.85E-16	7.68E-16	7.25E-16	6.84E-16	6.89E-16	7.56E-16	7.78E-16	7.28E-16	7.07E-16	7.70E-16	7.23E-16	6.54E-16	6.16E-16
8.68E-16	8.52E-16	8.41E-16	8.19E-16	8.09E-16	7.73E-16	7.28E-16	7.27E-16	7.73E-16	7.99E-16	7.42E-16	7.26E-16	7.74E-16	7.73E-16	6.90E-16	6.31E-16
8.69E-16	8.77E-16	8.73E-16	8.56E-16	8.46E-16	8.21E-16	7.94E-16	7.93E-16	7.98E-16	8.15E-16	7.54E-16	7.43E-16	7.81E-16	8.11E-16	7.41E-16	6.89E-16
8.81E-16	8.88E-16	9.00E-16	8.54E-16	8.55E-16	8.48E-16	8.49E-16	8.13E-16	8.14E-16	8.34E-16	7.84E-16	7.70E-16	7.91E-16	8.21E-16	7.73E-16	7.20E-16
9.04E-16	9.08E-16	9.03E-16	8.62E-16	8.58E-16	8.54E-16	8.64E-16	8.19E-16	8.14E-16	8.42E-16	8.06E-16	7.92E-16	7.98E-16	8.22E-16	7.61E-16	7.15E-16
9.31E-16	9.27E-16	9.22E-16	8.94E-16	9.06E-16	8.95E-16	8.73E-16	8.33E-16	7.99E-16	8.39E-16	8.11E-16	7.97E-16	7.93E-16	8.26E-16	7.57E-16	7.17E-16
9.46E-16	9.55E-16	9.63E-16	9.14E-16	9.37E-16	9.10E-16	8.79E-16	8.45E-16	7.97E-16	8.23E-16	7.86E-16	7.84E-16	7.82E-16	8.20E-16	7.56E-16	7.38E-16
9.54E-16	9.80E-16	1.00E-15	9.32E-16	9.40E-16	9.02E-16	8.75E-16	8.48E-16	7.89E-16	8.26E-16	7.81E-16	7.86E-16	7.74E-16	8.09E-16	7.65E-16	7.54E-16
9.51E-16	9.69E-16	1.01E-15	9.64E-16	9.54E-16	8.93E-16	8.82E-16	8.47E-16	7.70E-16	8.18E-16	7.79E-16	7.91E-16	7.91E-16	8.09E-16	7.70E-16	7.58E-16

9.59E-16	9.60E-16	9.82E-16	9.59E-16	9.54E-16	9.00E-16	9.08E-16	8.50E-16	7.59E-16	8.07E-16	7.79E-16	7.93E-16	8.01E-16	8.22E-16	7.87E-16	7.72E-16
9.63E-16	9.64E-16	9.41E-16	9.46E-16	9.45E-16	9.24E-16	9.15E-16	8.58E-16	7.96E-16	8.13E-16	7.73E-16	7.91E-16	8.11E-16	8.26E-16	7.90E-16	8.02E-16
9.94E-16	1.00E-15	9.14E-16	9.22E-16	9.44E-16	9.43E-16	9.29E-16	8.66E-16	8.50E-16	8.28E-16	7.79E-16	8.04E-16	8.24E-16	8.13E-16	8.12E-16	8.13E-16
1.02E-15	1.04E-15	8.90E-16	9.27E-16	9.52E-16	9.58E-16	9.32E-16	8.85E-16	8.78E-16	8.24E-16	7.80E-16	8.23E-16	8.40E-16	8.10E-16	8.21E-16	8.03E-16
1.05E-15	1.05E-15	9.30E-16	9.50E-16	9.58E-16	9.57E-16	9.36E-16	8.92E-16	8.79E-16	8.23E-16	7.93E-16	8.43E-16	8.42E-16	7.99E-16	8.24E-16	8.08E-16
1.07E-15	1.06E-15	9.53E-16	9.50E-16	9.47E-16	9.50E-16	9.64E-16	9.29E-16	9.05E-16	8.48E-16	8.00E-16	8.57E-16	8.26E-16	7.94E-16	8.24E-16	8.23E-16
1.07E-15	1.09E-15	9.71E-16	9.52E-16	9.53E-16	9.33E-16	9.59E-16	9.59E-16	9.04E-16	8.94E-16	8.03E-16	8.48E-16	8.02E-16	8.07E-16	8.30E-16	8.36E-16
1.02E-15	1.09E-15	1.02E-15	9.78E-16	9.65E-16	9.64E-16	9.41E-16	9.47E-16	8.84E-16	8.90E-16	8.06E-16	8.47E-16	8.12E-16	8.23E-16	8.49E-16	8.51E-16
1.06E-15	1.09E-15	1.08E-15	9.96E-16	9.80E-16	9.87E-16	9.38E-16	9.18E-16	8.56E-16	8.54E-16	8.08E-16	8.53E-16	8.68E-16	8.45E-16	8.53E-16	8.53E-16
1.05E-15	1.07E-15	1.05E-15	9.84E-16	9.65E-16	9.78E-16	9.45E-16	8.89E-16	8.34E-16	8.14E-16	8.16E-16	8.78E-16	8.98E-16	8.50E-16	8.15E-16	8.33E-16
1.01E-15	1.02E-15	1.02E-15	9.66E-16	9.64E-16	9.76E-16	9.80E-16	9.18E-16	8.55E-16	8.17E-16	8.41E-16	8.97E-16	8.98E-16	8.61E-16	7.76E-16	8.16E-16
8.02E-14	8.04E-14	7.95E-14	7.88E-14	7.78E-14	7.62E-14	7.60E-14	7.51E-14	7.42E-14	7.30E-14	7.18E-14	7.15E-14	7.32E-14	7.12E-14	6.89E-14	6.81E-14

5.05E-08	4.97E-08	5.07E-08	5.02E-08	4.93E-08	4.82E-08	4.73E-08	4.78E-08	4.81E-08	4.72E-08	4.69E-08	4.79E-08	4.73E-08	4.72E-08	4.71E-08
4.92E-08	4.82E-08	4.93E-08	4.89E-08	4.82E-08	4.77E-08	4.61E-08	4.70E-08	4.76E-08	4.65E-08	4.60E-08	4.66E-08	4.59E-08	4.57E-08	4.56E-08
4.75E-08	4.70E-08	4.79E-08	4.70E-08	4.68E-08	4.68E-08	4.49E-08	4.64E-08	4.73E-08	4.59E-08	4.49E-08	4.45E-08	4.44E-08	4.47E-08	4.42E-08
4.59E-08	4.63E-08	4.62E-08	4.55E-08	4.51E-08	4.47E-08	4.45E-08	4.55E-08	4.61E-08	4.44E-08	4.36E-08	4.31E-08	4.34E-08	4.36E-08	4.30E-08
4.44E-08	4.55E-08	4.46E-08	4.41E-08	4.35E-08	4.31E-08	4.38E-08	4.43E-08	4.42E-08	4.29E-08	4.28E-08	4.28E-08	4.23E-08	4.21E-08	4.16E-08
4.32E-08	4.35E-08	4.27E-08	4.26E-08	4.22E-08	4.17E-08	4.22E-08	4.31E-08	4.24E-08	4.14E-08	4.17E-08	4.20E-08	4.09E-08	4.08E-08	4.06E-08
4.25E-08	4.20E-08	4.12E-08	4.10E-08	4.07E-08	4.06E-08	4.13E-08	4.25E-08	4.23E-08	4.04E-08	4.06E-08	4.05E-08	3.93E-08	4.01E-08	4.00E-08
4.11E-08	4.07E-08	4.02E-08	3.98E-08	3.90E-08	4.02E-08	4.11E-08	4.29E-08	4.16E-08	3.97E-08	3.94E-08	3.90E-08	3.86E-08	3.94E-08	3.93E-08
3.99E-08	3.97E-08	3.93E-08	3.84E-08	3.81E-08	3.99E-08	4.05E-08	4.29E-08	4.10E-08	3.88E-08	3.81E-08	3.84E-08	3.84E-08	3.85E-08	3.89E-08
3.88E-08	3.92E-08	3.87E-08	3.78E-08	3.76E-08	3.88E-08	3.88E-08	4.12E-08	3.93E-08	3.81E-08	3.72E-08	3.77E-08	3.78E-08	3.77E-08	3.84E-08
3.81E-08	3.90E-08	3.81E-08	3.76E-08	3.65E-08	3.72E-08	3.73E-08	3.89E-08	3.77E-08	3.77E-08	3.64E-08	3.70E-08	3.70E-08	3.65E-08	3.74E-08
3.76E-08	3.84E-08	3.78E-08	3.74E-08	3.55E-08	3.58E-08	3.61E-08	3.72E-08	3.65E-08	3.73E-08	3.57E-08	3.63E-08	3.59E-08	3.49E-08	3.64E-08
3.64E-08	3.76E-08	3.72E-08	3.75E-08	3.50E-08	3.54E-08	3.50E-08	3.60E-08	3.52E-08	3.52E-08	3.46E-08	3.51E-08	3.50E-08	3.41E-08	3.45E-08
3.48E-08	3.57E-08	3.60E-08	3.66E-08	3.50E-08	3.54E-08	3.44E-08	3.42E-08	3.33E-08	3.34E-08	3.33E-08	3.40E-08	3.41E-08	3.26E-08	3.31E-08
3.41E-08	3.39E-08	3.43E-08	3.44E-08	3.45E-08	3.44E-08	3.31E-08	3.27E-08	3.20E-08	3.24E-08	3.22E-08	3.29E-08	3.23E-08	3.15E-08	3.18E-08
3.33E-08	3.26E-08	3.23E-08	3.29E-08	3.36E-08	3.26E-08	3.19E-08	3.13E-08	3.10E-08	3.15E-08	3.09E-08	3.21E-08	3.13E-08	3.05E-08	3.08E-08
3.24E-08	3.15E-08	3.06E-08	3.17E-08	3.18E-08	3.06E-08	3.03E-08	2.92E-08	2.94E-08	3.03E-08	2.94E-08	3.11E-08	3.02E-08	2.93E-08	3.02E-08
3.10E-08	3.08E-08	2.95E-08	3.01E-08	3.00E-08	2.92E-08	2.92E-08	2.79E-08	2.83E-08	2.99E-08	2.81E-08	2.85E-08	2.95E-08	2.82E-08	2.88E-08
2.97E-08	3.01E-08	2.88E-08	2.86E-08	2.86E-08	2.86E-08	2.81E-08	2.70E-08	2.72E-08	2.99E-08	2.71E-08	2.77E-08	2.84E-08	2.72E-08	2.63E-08
2.83E-08	2.89E-08	2.78E-08	2.74E-08	2.74E-08	2.78E-08	2.71E-08	2.65E-08	2.65E-08	2.73E-08	2.61E-08	2.63E-08	2.72E-08	2.63E-08	2.54E-08
2.70E-08	2.72E-08	2.65E-08	2.65E-08	2.64E-08	2.67E-08	2.64E-08	2.60E-08	2.57E-08	2.58E-08	2.54E-08	2.49E-08	2.61E-08	2.56E-08	2.47E-08
2.61E-08	2.60E-08	2.53E-08	2.57E-08	2.55E-08	2.60E-08	2.57E-08	2.48E-08	2.51E-08	2.51E-08	2.48E-08	2.43E-08	2.52E-08	2.44E-08	2.39E-08
2.56E-08	2.47E-08	2.47E-08	2.45E-08	2.48E-08	2.56E-08	2.50E-08	2.35E-08	2.42E-08	2.44E-08	2.41E-08	2.35E-08	2.42E-08	2.30E-08	2.28E-08
2.52E-08	2.40E-08	2.44E-08	2.34E-08	2.39E-08	2.47E-08	2.46E-08	2.26E-08	2.31E-08	2.35E-08	2.31E-08	2.24E-08	2.30E-08	2.20E-08	2.20E-08
2.39E-08	2.36E-08	2.41E-08	2.27E-08	2.31E-08	2.36E-08	2.44E-08	2.22E-08	2.20E-08	2.28E-08	2.25E-08	2.19E-08	2.18E-08	2.12E-08	2.08E-08
2.23E-08	2.28E-08	2.30E-08	2.20E-08	2.20E-08	2.26E-08	2.32E-08	2.19E-08	2.18E-08	2.24E-08	2.18E-08	2.14E-08	2.08E-08	2.01E-08	2.01E-08
2.12E-08	2.19E-08	2.17E-08	2.09E-08	2.11E-08	2.16E-08	2.29E-08	2.14E-08	2.14E-08	2.13E-08	2.10E-08	2.10E-08	2.01E-08	1.95E-08	1.89E-08
2.08E-08	2.12E-08	2.07E-08	1.97E-08	2.02E-08	2.02E-08	2.16E-08	1.97E-08	2.01E-08	1.93E-08	2.03E-08	1.99E-08	1.90E-08	1.84E-08	1.76E-08
2.06E-08	2.06E-08	2.04E-08	1.93E-08	1.97E-08	1.94E-08	1.99E-08	1.78E-08	1.83E-08	1.76E-08	1.93E-08	1.87E-08	1.83E-08	1.76E-08	1.68E-08
1.97E-08	1.99E-08	2.03E-08	1.92E-08	1.89E-08	1.84E-08	1.84E-08	1.66E-08	1.74E-08	1.63E-08	1.74E-08	1.75E-08	1.74E-08	1.71E-08	1.60E-08
1.88E-08	1.88E-08	1.96E-08	1.88E-08	1.84E-08	1.73E-08	1.70E-08	1.59E-08	1.69E-08	1.60E-08	1.61E-08	1.63E-08	1.63E-08	1.65E-08	1.53E-08
1.78E-08	1.72E-08	1.83E-08	1.76E-08	1.77E-08	1.63E-08	1.64E-08	1.52E-08	1.60E-08	1.52E-08	1.50E-08	1.48E-08	1.51E-08	1.62E-08	1.44E-08
1.60E-08	1.59E-08	1.67E-08	1.62E-08	1.66E-08	1.57E-08	1.54E-08	1.47E-08	1.47E-08	1.41E-08	1.39E-08	1.36E-08	1.37E-08	1.43E-08	1.31E-08
1.44E-08	1.47E-08	1.54E-08	1.51E-08	1.55E-08	1.52E-08	1.41E-08	1.38E-08	1.43E-08	1.37E-08	1.26E-08	1.29E-08	1.25E-08	1.28E-08	1.23E-08
1.39E-08	1.42E-08	1.40E-08	1.42E-08	1.42E-08	1.46E-08	1.35E-08	1.32E-08	1.38E-08	1.33E-08	1.18E-08	1.24E-08	1.18E-08	1.22E-08	1.20E-08
1.35E-08	1.34E-08	1.35E-08	1.33E-08	1.35E-08	1.33E-08	1.30E-08	1.31E-08	1.29E-08	1.23E-08	1.16E-08	1.17E-08	1.14E-08	1.20E-08	1.20E-08
1.32E-08	1.26E-08	1.27E-08	1.31E-08	1.32E-08	1.27E-08	1.28E-08	1.34E-08	1.23E-08	1.17E-08	1.17E-08	1.11E-08	1.13E-08	1.18E-08	1.12E-08
1.32E-08	1.28E-08	1.20E-08	1.24E-08	1.23E-08	1.23E-08	1.23E-08	1.29E-08	1.15E-08	1.13E-08	1.19E-08	1.12E-08	1.10E-08	1.11E-08	1.05E-08
1.26E-08	1.25E-08	1.14E-08	1.15E-08	1.12E-08	1.14E-08	1.15E-08	1.21E-08	1.10E-08	1.14E-08	1.11E-08	1.14E-08	1.05E-08	1.03E-08	9.74E-09
1.09E-08	1.12E-08	1.07E-08	1.06E-08	1.05E-08	1.03E-08	1.00E-08	1.11E-08	1.04E-08	1.06E-08	9.95E-09	1.08E-08	1.03E-08	9.85E-09	9.07E-09
1.02E-08	1.04E-08	9.85E-09	9.85E-09	1.04E-08	9.65E-09	9.11E-09	1.00E-08	9.57E-09	9.39E-09	9.26E-09	9.96E-09	9.67E-09	9.29E-09	8.60E-09
9.80E-09	9.75E-09	9.11E-09	9.13E-09	1.05E-08	9.87E-09	8.97E-09	9.43E-09	8.42E-09	8.78E-09	8.93E-09	9.28E-09	8.34E-09	8.55E-09	7.97E-09
8.54E-09	8.62E-09	7.96E-09	8.62E-09	1.01E-08	9.35E-09	8.81E-09	8.75E-09	7.63E-09	7.58E-09	8.32E-09	8.47E-09	7.12E-09	7.48E-09	7.40E-09
7.65E-09	7.65E-09	7.33E-09	8.57E-09	9.21E-09	7.67E-09	8.24E-09	8.19E-09	6.94E-09	6.79E-09	7.91E-09	7.98E-09	6.33E-09	6.47E-09	6.65E-09
6.65E-09	6.77E-09	7.00E-09	8.02E-09	8.54E-09	6.63E-09	6.91E-09	7.54E-09	6.18E-09	5.64E-09	6.83E-09	6.91E-09	5.62E-09	6.31E-09	5.87E-09
5.86E-09	5.79E-09	6.84E-09	7.39E-09	7.31E-09	5.72E-09	5.70E-09	6.66E-09	5.37E-09	4.82E-09	5.69E-09	5.81E-09	4.68E-09	5.76E-09	4.97E-09
6.00E-09	5.60E-09	6.98E-09	7.18E-09	6.00E-09	4.37E-09	5.07E-09	5.33E-09	4.81E-09	4.66E-09	5.26E-09	5.30E-09	3.95E-09	4.60E-09	4.42E-09
5.65E-09	5.39E-09	6.24E-09	6.55E-09	5.17E-09	3.80E-09	4.74E-09	4.66E-09	4.65E-09	4.40E-09	4.86E-09	4.86E-09	3.49E-09	3.92E-09	4.16E-09
4.67E-09	4.89E-09	4.72E-09	5.47E-09	4.31E-09	3.37E-09	4.41E-09	4.37E-09	4.04E-09	3.69E-09	4.53E-09	4.02E-09	3.31E-09	3.84E-09	3.93E-09
4.05E-09	4.23E-09	4.14E-09	4.90E-09	3.97E-09	2.48E-09	3.99E-09	3.48E-09	3.10E-09	3.15E-09	4.08E-09	3.21E-09	2.73E-09	3.04E-09	3.51E-09
3.59E-09	3.47E-09	3.14E-09	3.68E-09	2.85E-09	1.58E-09	2.03E-09	2.73E-09	2.72E-09	3.13E-09	3.57E-09	2.44E-09	2.02E-09	2.06E-09	2.03E-09
2.93E-09	2.89E-09	1.86E-09	2.53E-09	1.73E-09	7.16E-10	1.38E-09	2.21E-09	2.33E-09	2.83E-09	2.34E-09	1.91E-09	1.45E-09	1.03E-09	7.71E-10
2.55E-09	2.29E-09	1.36E-09	1.44E-09	7.85E-10	2.37E-10	7.25E-10	1.75E-09	2.02E-09	1.73E-09	9.63E-10	1.12E-09	8.35E-10	3.86E-10	-9.79E-11
1.80E-09	1.97E-09	1.16E-09	1.08E-09	2.72E-10	-4.55E-11	2.08E-10	7.88E-10	6.56E-10	4.25E-11	5.69E-11	6.87E-11	-1.53E-11	-1.72E-10	-3.26E-10
6.54E-10	1.14E-09	8.06E-10	1.17E-09	-2.20E-11	-1.03E-10	1.36E-10	-8.57E-11	-2.80E-10	-9.22E-10	-4.83E-10	-1.82E-10	-4.75E-10	-8.06E-10	-3.00E-11
-4.86E-10	3.94E-11	-2.80E-10	6.11E-10	-3.83E-10	4.29E-10	4.87E-10	-5.76E-10	-8.02E-10	-1.06E-09	-1.18E-09	-6.41E-10	-9.99E-10	-1.24E-09	-9.71E-11
-1.77E-09	-1.51E-09	-1.53E-09	-9.72E-10	-1.11E-09	4.66E-10	-1.14E-10	-1.15E-09	-1.71E-09	-1.75E-09	-2.11E-09	-1.70E-09	-1.23E-09	-1.33E-09	-6.93E-10
-2.27E-09	-2.10E-09	-2.13E-09	-1.93E-09	-1.96E-09	-6.27E-10	-6.79E-10	-1.23E-09	-2.08E-09	-2.11E-09	-2.52E-09	-2.25E-09	-1.60E-09	-1.61E-09	-1.53E-09
-2.52E-09	-2.69E-09	-2.92E-09	-3.31E-09	-3.01E-09	-1.78E-09	-1.26E-09	-1.73E-09	-3.17E-09	-2.74E-09	-2.86E-09	-2.90E-09	-2.48E-09	-2.23E-09	-2.33E-09
-3.00E-09	-3.81E-09	-3.74E-09	-3.73E-09	-3.37E-09	-2.24E-09	-1.93E-09	-2.29E-09	-3.91E-09	-3.15E-09	-3.15E-09	-3.79E-09	-3.14E-09	-3.50E-09	-3.12E-09
-3.33E-09	-4.68E-09	-4.20E-09	-3.83E-09	-3.23E-09	-2.32E-09	-2.89E-09	-3.05E-09	-4.23E-09	-3.27E-09	-3.57E-09	-4.75E-09	-3.80E-09	-4.36E-09	-4.08E-09
-4.27E-09	-5.29E-09	-4.37E-09	-4.09E-09	-2.73E-09	-1.87E-09	-3.30E-09	-3.77E-09	-4.74E-09	-3.65E-09	-4.32E-09	-5.47E-09	-4.71E-09	-5.31E-09	-4.70E-09
-5.16E-09	-5.39E-09	-4.89E-09	-4.61E-09	-2.80E-09	-1.65E-09	-3.51E-09	-4.44E-09	-4.72E-09	-4.39E-09	-4.99E-09	-5.92E-09	-5.59E-09	-5.72E-09	-4.89E-09
-5.62E-09	-5.96E-09	-5.65E-09	-5.38E-09	-4.01E-09	-2.83E-09	-4.22E-09	-5.56E-09	-4.96E-09	-4.99E-09	-5.88E-09	-6.92E-09	-6.44E-09	-5.91E-09	-4.93E-09
-6.26E-09	-6.57E-09	-6.00E-09	-6.30E-09	-5.84E-09	-5.12E-09	-5.17E-09	-5.92E-09	-5.25E-09	-6.02E-09	-6.59E-09	-7.29E-09	-6.60E-09	-6.12E-09	-5.31E-09
-7.48E-09	-7.12E-09	-6.63E-09	-7.08E-09	-7.09E-09	-6.31E-09	-5.91E-09	-6.51E-09	-5.28E-09	-6.13E-09	-6.72E-09	-7.42E-09	-6.56E-09	-6.24E-09	-5.34E-09
-8.32E-09	-7.67E-09	-7.01E-09	-7.40E-09	-7.69E-09	-6.97E-09	-6.61E-09	-7.46E-09	-5.44E-09	-5.96E-09	-6.87E-09	-7.65E-09	-6.74E-09	-6.93E-09	-5.54E-09
-8.38E-09	-8.13E-09	-7.53E-09	-7.75E-09	-8.31E-09	-8.05E-09	-7.87E-09	-8.57E-09	-5.81E-09	-6.16E-09	-6.98E-09	-8.48E-09	-7.64E-09	-7.71E-09	-6.44E-09
-8.22E-09	-8.75E-09	-8.08E-09	-8.63E-09	-8.89E-09	-8.93E-09	-9.13E-09	-9.28E-09	-6.81E-09	-6.48E-09	-7.89E-09	-8.94E-09	-8.27E-09	-8.26E-09	-7.45E-09
-8.81E-09	-9.04E-09	-8.47E-09	-8.83E-09	-9.31E-09	-9.59E-09	-9.85E-09	-9.77E-09	-7.66E-09	-7.05E-09	-8.46E-09	-9.23E-09	-8.39E-09	-8.39E-09	-8.01E-09
-1.01E-08	-9.71E-09	-9.05E-09	-8.32E-09	-9.37E-09	-9.85E-09	-9.77E-09	-1.01E-08	-8.61E-09	-7.88E-09	-8.46E-09	-9.42E-09	-8.71E-09	-8.18E-09	-8.05E-09
-1.10E-08	-1.06E-08	-9.70E-09	-8.31E-09	-9.34E-09	-1.03E-08	-1.00E-08	-1.04E-08	-9.76E-09	-8.67E-09	-8.81E-09	-9.32E-09	-8.83E-09	-7.49E-09	-7.90E-09
-1.16E-08	-1.12E-08	-1.01E-08	-9.74E-09	-9.38E-09	-1.04E-08	-1.04E-08	-1.09E-08	-1.07E-08	-9.42E-09	-9.30E-09	-9.48E-09	-8.83E-09	-7.68E-09	-7.48E-09
-1.17E-08	-1.16E-08	-1.08E-08	-1.08E-08	-9.87E-09	-1.02E-08	-1.10E-08	-1.14E-08	-1.11E-08	-1.07E-08	-9.79E-09	-9.94E-09	-8.71E-09	-8.50E-09	-8.24E-09
-1.19E-08	-1.14E-08	-1.10E-08	-1.14E-08	-1.02E-08	-1.04E-08	-1.18E-08	-1.23E-08	-1.17E-08	-1.21E-08	-1.04E-08	-1.08E-08	-9.37E-09	-9.61E-09	-9.35E-09
-1.25E-08	-1.20E-08	-1.11E-08	-1.17E-08	-1.04E-08	-1.16E-08	-1.27E-08	-1.26E-08	-1.23E-08	-1.28E-08	-1.13E-08	-1.14E-08	-9.95E-09	-1.08E-08	-1.07E-08
-1.29E-08	-1.35E-08	-1.20E-08	-1.21E-08	-1.09E-08	-1.26E-08	-1.33E-08	-1.27E-08	-1.30E-08	-1.30E-08	-1.24E-08	-1.20E-08	-1.12E-08	-1.17E-08	-1.12E-08
-1.31E-08	-1.43E-08	-1.27E-08	-1.27E-08	-1.22E-08	-1.34E-08	-1.36E-08	-1.29E-08	-1.35E-08	-1.31E-08	-1.26E-08	-1.27E-08	-1.24E-08	-1.24E-08	-1.15E-08

-1.35E-08	-1.43E-08	-1.32E-08	-1.32E-08	-1.33E-08	-1.39E-08	-1.41E-08	-1.35E-08	-1.38E-08	-1.29E-08	-1.24E-08	-1.28E-08	-1.35E-08	-1.32E-08	-1.21E-08
-1.34E-08	-1.38E-08	-1.36E-08	-1.36E-08	-1.36E-08	-1.40E-08	-1.42E-08	-1.39E-08	-1.42E-08	-1.31E-08	-1.30E-08	-1.31E-08	-1.40E-08	-1.34E-08	-1.24E-08
-1.38E-08	-1.38E-08	-1.38E-08	-1.42E-08	-1.40E-08	-1.39E-08	-1.40E-08	-1.41E-08	-1.50E-08	-1.40E-08	-1.42E-08	-1.37E-08	-1.39E-08	-1.37E-08	-1.23E-08
-1.43E-08	-1.38E-08	-1.43E-08	-1.50E-08	-1.47E-08	-1.44E-08	-1.45E-08	-1.46E-08	-1.55E-08	-1.45E-08	-1.51E-08	-1.43E-08	-1.44E-08	-1.40E-08	-1.32E-08
-1.50E-08	-1.39E-08	-1.51E-08	-1.56E-08	-1.55E-08	-1.56E-08	-1.53E-08	-1.55E-08	-1.60E-08	-1.53E-08	-1.55E-08	-1.52E-08	-1.56E-08	-1.46E-08	-1.46E-08
-1.53E-08	-1.45E-08	-1.58E-08	-1.64E-08	-1.62E-08	-1.64E-08	-1.60E-08	-1.61E-08	-1.64E-08	-1.60E-08	-1.60E-08	-1.57E-08	-1.62E-08	-1.52E-08	-1.54E-08
-1.59E-08	-1.58E-08	-1.68E-08	-1.72E-08	-1.67E-08	-1.71E-08	-1.70E-08	-1.67E-08	-1.70E-08	-1.64E-08	-1.62E-08	-1.56E-08	-1.63E-08	-1.60E-08	-1.62E-08
-1.59E-08	-1.66E-08	-1.73E-08	-1.72E-08	-1.67E-08	-1.72E-08	-1.74E-08	-1.70E-08	-1.73E-08	-1.65E-08	-1.66E-08	-1.58E-08	-1.63E-08	-1.69E-08	-1.69E-08
-1.66E-08	-1.75E-08	-1.79E-08	-1.75E-08	-1.70E-08	-1.74E-08	-1.78E-08	-1.74E-08	-1.76E-08	-1.67E-08	-1.69E-08	-1.63E-08	-1.68E-08	-1.78E-08	-1.76E-08
-1.80E-08	-1.87E-08	-1.87E-08	-1.77E-08	-1.73E-08	-1.77E-08	-1.80E-08	-1.80E-08	-1.80E-08	-1.72E-08	-1.73E-08	-1.71E-08	-1.77E-08	-1.84E-08	-1.80E-08
-1.87E-08	-1.93E-08	-1.90E-08	-1.80E-08	-1.78E-08	-1.83E-08	-1.83E-08	-1.84E-08	-1.86E-08	-1.81E-08	-1.77E-08	-1.79E-08	-1.83E-08	-1.85E-08	-1.82E-08
-1.90E-08	-1.92E-08	-1.91E-08	-1.86E-08	-1.82E-08	-1.86E-08	-1.89E-08	-1.87E-08	-1.87E-08	-1.87E-08	-1.83E-08	-1.85E-08	-1.87E-08	-1.86E-08	-1.85E-08
-1.85E-08	-1.90E-08	-1.89E-08	-1.90E-08	-1.87E-08	-1.88E-08	-1.91E-08	-1.93E-08	-1.90E-08	-1.91E-08	-1.86E-08	-1.87E-08	-1.90E-08	-1.91E-08	-1.88E-08
-1.85E-08	-1.88E-08	-1.89E-08	-1.92E-08	-1.91E-08	-1.91E-08	-1.90E-08	-1.94E-08	-1.97E-08	-1.94E-08	-1.88E-08	-1.92E-08	-1.93E-08	-1.91E-08	-1.88E-08
-1.88E-08	-1.87E-08	-1.93E-08	-1.96E-08	-1.94E-08	-1.91E-08	-1.94E-08	-1.96E-08	-2.01E-08	-1.99E-08	-1.91E-08	-2.01E-08	-1.97E-08	-1.87E-08	-1.86E-08
-1.92E-08	-1.94E-08	-1.98E-08	-1.96E-08	-1.95E-08	-1.90E-08	-2.00E-08	-1.97E-08	-2.03E-08	-2.00E-08	-1.94E-08	-2.00E-08	-1.97E-08	-1.89E-08	-1.86E-08
-2.00E-08	-1.99E-08	-2.01E-08	-1.97E-08	-1.99E-08	-1.95E-08	-2.03E-08	-2.00E-08	-2.02E-08	-2.01E-08	-1.95E-08	-1.96E-08	-1.96E-08	-1.91E-08	-1.86E-08
-2.08E-08	-2.02E-08	-2.05E-08	-1.96E-08	-2.03E-08	-2.02E-08	-2.02E-08	-2.00E-08	-2.05E-08	-2.06E-08	-1.99E-08	-1.97E-08	-1.96E-08	-1.93E-08	-1.91E-08
-2.12E-08	-2.06E-08	-2.07E-08	-2.00E-08	-2.09E-08	-2.10E-08	-2.07E-08	-2.06E-08	-2.07E-08	-2.04E-08	-2.05E-08	-1.99E-08	-1.95E-08	-1.98E-08	-1.98E-08
-2.18E-08	-2.09E-08	-2.17E-08	-2.15E-08	-2.18E-08	-2.19E-08	-2.14E-08	-2.15E-08	-2.16E-08	-2.11E-08	-2.07E-08	-2.03E-08	-2.01E-08	-2.05E-08	-2.09E-08
-2.24E-08	-2.16E-08	-2.20E-08	-2.24E-08	-2.27E-08	-2.24E-08	-2.16E-08	-2.23E-08	-2.22E-08	-2.17E-08	-2.10E-08	-2.11E-08	-2.10E-08	-2.10E-08	-2.17E-08
-2.29E-08	-2.15E-08	-2.19E-08	-2.26E-08	-2.29E-08	-2.25E-08	-2.25E-08	-2.34E-08	-2.29E-08	-2.23E-08	-2.17E-08	-2.18E-08	-2.14E-08	-2.13E-08	-2.19E-08
-2.30E-08	-2.23E-08	-2.20E-08	-2.29E-08	-2.29E-08	-2.30E-08	-2.33E-08	-2.36E-08	-2.33E-08	-2.27E-08	-2.18E-08	-2.22E-08	-2.16E-08	-2.19E-08	-2.20E-08
-2.32E-08	-2.31E-08	-2.27E-08	-2.36E-08	-2.33E-08	-2.38E-08	-2.39E-08	-2.33E-08	-2.32E-08	-2.27E-08	-2.15E-08	-2.26E-08	-2.19E-08	-2.24E-08	-2.20E-08
-2.33E-08	-2.37E-08	-2.36E-08	-2.43E-08	-2.37E-08	-2.43E-08	-2.38E-08	-2.33E-08	-2.28E-08	-2.31E-08	-2.18E-08	-2.29E-08	-2.25E-08	-2.23E-08	-2.18E-08
-2.32E-08	-2.40E-08	-2.46E-08	-2.48E-08	-2.41E-08	-2.45E-08	-2.42E-08	-2.39E-08	-2.27E-08	-2.32E-08	-2.22E-08	-2.29E-08	-2.25E-08	-2.22E-08	-2.20E-08
-2.31E-08	-2.42E-08	-2.53E-08	-2.53E-08	-2.44E-08	-2.42E-08	-2.47E-08	-2.46E-08	-2.33E-08	-2.36E-08	-2.22E-08	-2.29E-08	-2.21E-08	-2.22E-08	-2.27E-08
-2.30E-08	-2.42E-08	-2.53E-08	-2.55E-08	-2.50E-08	-2.45E-08	-2.48E-08	-2.53E-08	-2.42E-08	-2.41E-08	-2.25E-08	-2.34E-08	-2.20E-08	-2.22E-08	-2.35E-08
-2.38E-08	-2.46E-08	-2.53E-08	-2.60E-08	-2.56E-08	-2.52E-08	-2.46E-08	-2.59E-08	-2.49E-08	-2.49E-08	-2.34E-08	-2.39E-08	-2.31E-08	-2.25E-08	-2.40E-08
-2.45E-08	-2.56E-08	-2.55E-08	-2.64E-08	-2.61E-08	-2.56E-08	-2.48E-08	-2.59E-08	-2.56E-08	-2.54E-08	-2.47E-08	-2.49E-08	-2.42E-08	-2.34E-08	-2.39E-08
-2.49E-08	-2.65E-08	-2.62E-08	-2.69E-08	-2.62E-08	-2.59E-08	-2.50E-08	-2.58E-08	-2.62E-08	-2.61E-08	-2.57E-08	-2.54E-08	-2.52E-08	-2.46E-08	-2.46E-08
-2.57E-08	-2.70E-08	-2.69E-08	-2.70E-08	-2.60E-08	-2.60E-08	-2.50E-08	-2.61E-08	-2.63E-08	-2.66E-08	-2.60E-08	-2.52E-08	-2.57E-08	-2.55E-08	-2.54E-08
-2.63E-08	-2.73E-08	-2.69E-08	-2.71E-08	-2.60E-08	-2.60E-08	-2.54E-08	-2.65E-08	-2.68E-08	-2.69E-08	-2.61E-08	-2.51E-08	-2.62E-08	-2.64E-08	-2.65E-08
-2.65E-08	-2.76E-08	-2.74E-08	-2.75E-08	-2.63E-08	-2.58E-08	-2.56E-08	-2.64E-08	-2.70E-08	-2.71E-08	-2.65E-08	-2.55E-08	-2.66E-08	-2.71E-08	-2.72E-08
-2.65E-08	-2.77E-08	-2.78E-08	-2.77E-08	-2.69E-08	-2.58E-08	-2.61E-08	-2.60E-08	-2.70E-08	-2.70E-08	-2.63E-08	-2.56E-08	-2.70E-08	-2.71E-08	-2.70E-08
-2.69E-08	-2.82E-08	-2.79E-08	-2.80E-08	-2.75E-08	-2.66E-08	-2.68E-08	-2.66E-08	-2.71E-08	-2.66E-08	-2.58E-08	-2.58E-08	-2.67E-08	-2.67E-08	-2.66E-08
-2.76E-08	-2.84E-08	-2.80E-08	-2.84E-08	-2.82E-08	-2.74E-08	-2.76E-08	-2.74E-08	-2.78E-08	-2.73E-08	-2.60E-08	-2.60E-08	-2.59E-08	-2.57E-08	-2.61E-08
-2.76E-08	-2.81E-08	-2.79E-08	-2.85E-08	-2.87E-08	-2.79E-08	-2.78E-08	-2.78E-08	-2.80E-08	-2.76E-08	-2.64E-08	-2.59E-08	-2.52E-08	-2.46E-08	-2.54E-08
-2.73E-08	-2.75E-08	-2.77E-08	-2.83E-08	-2.85E-08	-2.80E-08	-2.76E-08	-2.78E-08	-2.77E-08	-2.74E-08	-2.68E-08	-2.59E-08	-2.52E-08	-2.43E-08	-2.48E-08
-2.73E-08	-2.69E-08	-2.74E-08	-2.78E-08	-2.79E-08	-2.77E-08	-2.75E-08	-2.76E-08	-2.74E-08	-2.74E-08	-2.70E-08	-2.60E-08	-2.54E-08	-2.45E-08	-2.46E-08
-2.79E-08	-2.70E-08	-2.74E-08	-2.75E-08	-2.74E-08	-2.71E-08	-2.72E-08	-2.71E-08	-2.74E-08	-2.74E-08	-2.67E-08	-2.61E-08	-2.51E-08	-2.43E-08	-2.45E-08
-2.79E-08	-2.71E-08	-2.79E-08	-2.78E-08	-2.79E-08	-2.68E-08	-2.67E-08	-2.66E-08	-2.71E-08	-2.71E-08	-2.63E-08	-2.60E-08	-2.51E-08	-2.40E-08	-2.45E-08
-2.79E-08	-2.75E-08	-2.81E-08	-2.80E-08	-2.85E-08	-2.72E-08	-2.68E-08	-2.62E-08	-2.64E-08	-2.61E-08	-2.57E-08	-2.56E-08	-2.53E-08	-2.46E-08	-2.46E-08
-2.82E-08	-2.80E-08	-2.83E-08	-2.83E-08	-2.87E-08	-2.76E-08	-2.70E-08	-2.56E-08	-2.55E-08	-2.52E-08	-2.44E-08	-2.45E-08	-2.51E-08	-2.51E-08	-2.47E-08
-2.83E-08	-2.83E-08	-2.86E-08	-2.85E-08	-2.87E-08	-2.73E-08	-2.64E-08	-2.49E-08	-2.48E-08	-2.47E-08	-2.38E-08	-2.36E-08	-2.49E-08	-2.48E-08	-2.47E-08
-2.83E-08	-2.83E-08	-2.86E-08	-2.81E-08	-2.85E-08	-2.70E-08	-2.59E-08	-2.46E-08	-2.45E-08	-2.45E-08	-2.40E-08	-2.36E-08	-2.51E-08	-2.48E-08	-2.45E-08
-2.83E-08	-2.86E-08	-2.86E-08	-2.79E-08	-2.78E-08	-2.67E-08	-2.59E-08	-2.46E-08	-2.41E-08	-2.46E-08	-2.51E-08	-2.44E-08	-2.49E-08	-2.50E-08	-2.48E-08
-2.89E-08	-2.89E-08	-2.85E-08	-2.78E-08	-2.72E-08	-2.65E-08	-2.63E-08	-2.52E-08	-2.42E-08	-2.48E-08	-2.53E-08	-2.45E-08	-2.45E-08	-2.47E-08	-2.48E-08
-2.92E-08	-2.89E-08	-2.84E-08	-2.74E-08	-2.66E-08	-2.63E-08	-2.62E-08	-2.56E-08	-2.48E-08	-2.45E-08	-2.51E-08	-2.48E-08	-2.44E-08	-2.45E-08	-2.43E-08
-2.88E-08	-2.87E-08	-2.85E-08	-2.74E-08	-2.67E-08	-2.67E-08	-2.59E-08	-2.58E-08	-2.50E-08	-2.46E-08	-2.46E-08	-2.49E-08	-2.45E-08	-2.45E-08	-2.44E-08

2.55E-15	2.47E-15	2.57E-15	2.52E-15	2.43E-15	2.32E-15	2.24E-15	2.29E-15	2.31E-15	2.23E-15	2.20E-15	2.29E-15	2.23E-15	2.23E-15	2.22E-15
2.42E-15	2.32E-15	2.43E-15	2.40E-15	2.32E-15	2.28E-15	2.13E-15	2.21E-15	2.26E-15	2.16E-15	2.12E-15	2.17E-15	2.10E-15	2.09E-15	2.08E-15
2.26E-15	2.21E-15	2.29E-15	2.21E-15	2.19E-15	2.19E-15	2.02E-15	2.15E-15	2.24E-15	2.11E-15	2.02E-15	1.98E-15	1.97E-15	1.99E-15	1.95E-15
2.10E-15	2.14E-15	2.14E-15	2.07E-15	2.04E-15	2.00E-15	1.98E-15	2.07E-15	2.13E-15	1.97E-15	1.90E-15	1.86E-15	1.89E-15	1.90E-15	1.85E-15
1.97E-15	2.07E-15	1.99E-15	1.94E-15	1.89E-15	1.86E-15	1.91E-15	1.97E-15	1.95E-15	1.84E-15	1.83E-15	1.83E-15	1.79E-15	1.77E-15	1.73E-15
1.87E-15	1.89E-15	1.83E-15	1.81E-15	1.78E-15	1.74E-15	1.78E-15	1.86E-15	1.80E-15	1.71E-15	1.74E-15	1.77E-15	1.67E-15	1.67E-15	1.65E-15
1.80E-15	1.76E-15	1.70E-15	1.68E-15	1.66E-15	1.65E-15	1.71E-15	1.81E-15	1.79E-15	1.63E-15	1.64E-15	1.64E-15	1.55E-15	1.60E-15	1.60E-15
1.69E-15	1.66E-15	1.61E-15	1.58E-15	1.52E-15	1.62E-15	1.69E-15	1.84E-15	1.73E-15	1.58E-15	1.55E-15	1.52E-15	1.49E-15	1.55E-15	1.55E-15
1.59E-15	1.58E-15	1.54E-15	1.47E-15	1.45E-15	1.59E-15	1.64E-15	1.84E-15	1.68E-15	1.50E-15	1.45E-15	1.48E-15	1.47E-15	1.48E-15	1.51E-15
1.51E-15	1.53E-15	1.50E-15	1.43E-15	1.41E-15	1.50E-15	1.50E-15	1.70E-15	1.55E-15	1.45E-15	1.39E-15	1.42E-15	1.43E-15	1.42E-15	1.47E-15
1.45E-15	1.52E-15	1.46E-15	1.42E-15	1.33E-15	1.38E-15	1.39E-15	1.51E-15	1.42E-15	1.42E-15	1.33E-15	1.37E-15	1.37E-15	1.33E-15	1.40E-15
1.42E-15	1.47E-15	1.43E-15	1.40E-15	1.26E-15	1.28E-15	1.30E-15	1.38E-15	1.33E-15	1.39E-15	1.28E-15	1.32E-15	1.29E-15	1.22E-15	1.32E-15
1.33E-15	1.41E-15	1.38E-15	1.41E-15	1.23E-15	1.26E-15	1.23E-15	1.30E-15	1.24E-15	1.24E-15	1.20E-15	1.23E-15	1.23E-15	1.17E-15	1.19E-15
1.21E-15	1.28E-15	1.30E-15	1.34E-15	1.22E-15	1.26E-15	1.19E-15	1.17E-15	1.11E-15	1.11E-15	1.11E-15	1.15E-15	1.16E-15	1.07E-15	1.09E-15
1.16E-15	1.15E-15	1.18E-15	1.18E-15	1.19E-15	1.18E-15	1.09E-15	1.07E-15	1.02E-15	1.05E-15	1.04E-15	1.08E-15	1.04E-15	9.94E-16	1.01E-15
1.11E-15	1.06E-15	1.04E-15	1.08E-15	1.13E-15	1.06E-15	1.02E-15	9.79E-16	9.59E-16	9.91E-16	9.52E-16	1.03E-15	9.79E-16	9.30E-16	9.51E-16
1.05E-15	9.95E-16	9.36E-16	1.00E-15	1.01E-15	9.37E-16	9.15E-16	8.53E-16	8.65E-16	9.18E-16	8.65E-16	9.66E-16	9.15E-16	8.56E-16	9.10E-16
9.60E-16	9.50E-16	8.69E-16	9.07E-16	9.01E-16	8.53E-16	8.54E-16	7.76E-16	8.03E-16	8.91E-16	7.92E-16	8.10E-16	8.70E-16	7.92E-16	8.29E-16
8.81E-16	9.09E-16	8.27E-16	8.18E-16	8.16E-16	8.19E-16	7.92E-16	7.27E-16	7.40E-16	8.91E-16	7.33E-16	7.66E-16	8.05E-16	7.38E-16	6.91E-16
7.98E-16	8.35E-16	7.70E-16	7.50E-16	7.52E-16	7.74E-16	7.35E-16	7.01E-16	7.02E-16	7.45E-16	6.84E-16	6.94E-16	7.41E-16	6.91E-16	6.44E-16
7.32E-16	7.42E-16	7.05E-16	7.00E-16	6.97E-16	7.11E-16	6.96E-16	6.77E-16	6.59E-16	6.65E-16	6.44E-16	6.22E-16	6.79E-16	6.54E-16	6.10E-16
6.82E-16	6.76E-16	6.42E-16	6.59E-16	6.52E-16	6.77E-16	6.62E-16	6.13E-16	6.30E-16	6.30E-16	6.13E-16	5.88E-16	6.33E-16	5.97E-16	5.72E-16
6.53E-16	6.09E-16	6.09E-16	6.02E-16	6.17E-16	6.56E-16	6.27E-16	5.51E-16	5.84E-16	5.94E-16	5.81E-16	5.53E-16	5.87E-16	5.29E-16	5.19E-16
6.36E-16	5.75E-16	5.95E-16	5.49E-16	5.71E-16	6.08E-16	6.06E-16	5.11E-16	5.35E-16	5.51E-16	5.32E-16	5.02E-16	5.31E-16	4.83E-16	4.84E-16
5.70E-16	5.55E-16	5.79E-16	5.16E-16	5.34E-16	5.58E-16	5.94E-16	4.92E-16	4.83E-16	5.19E-16	5.06E-16	4.78E-16	4.76E-16	4.48E-16	4.34E-16
4.95E-16	5.21E-16	5.30E-16	4.85E-16	4.86E-16	5.11E-16	5.37E-16	4.81E-16	4.77E-16	5.01E-16	4.75E-16	4.57E-16	4.34E-16	4.04E-16	4.05E-16
4.50E-16	4.81E-16	4.70E-16	4.38E-16	4.45E-16	4.66E-16	5.26E-16	4.59E-16	4.58E-16	4.52E-16	4.42E-16	4.40E-16	4.04E-16	3.78E-16	3.59E-16
4.32E-16	4.48E-16	4.27E-16	3.89E-16	4.07E-16	4.09E-16	4.68E-16	3.86E-16	4.05E-16	3.74E-16	4.12E-16	3.95E-16	3.62E-16	3.40E-16	3.10E-16
4.25E-16	4.25E-16	4.18E-16	3.74E-16	3.88E-16	3.75E-16	3.96E-16	3.18E-16	3.34E-16	3.08E-16	3.74E-16	3.50E-16	3.34E-16	3.10E-16	2.82E-16
3.89E-16	3.97E-16	4.11E-16	3.68E-16	3.57E-16	3.39E-16	3.37E-16	2.77E-16	3.03E-16	2.67E-16	3.04E-16	3.08E-16	3.03E-16	2.91E-16	2.54E-16
3.53E-16	3.53E-16	3.83E-16	3.54E-16	3.39E-16	2.98E-16	2.89E-16	2.52E-16	2.85E-16	2.56E-16	2.60E-16	2.64E-16	2.67E-16	2.73E-16	2.33E-16

3.16E-16	2.94E-16	3.36E-16	3.10E-16	3.13E-16	2.66E-16	2.70E-16	2.31E-16	2.56E-16	2.33E-16	2.26E-16	2.18E-16	2.28E-16	2.62E-16	2.07E-16
2.57E-16	2.53E-16	2.80E-16	2.62E-16	2.75E-16	2.45E-16	2.37E-16	2.16E-16	2.15E-16	2.00E-16	1.92E-16	1.85E-16	1.89E-16	2.03E-16	1.72E-16
2.08E-16	2.17E-16	2.36E-16	2.29E-16	2.39E-16	2.31E-16	1.99E-16	1.90E-16	2.05E-16	1.86E-16	1.59E-16	1.65E-16	1.55E-16	1.64E-16	1.52E-16
1.92E-16	2.02E-16	1.97E-16	2.01E-16	2.02E-16	2.13E-16	1.81E-16	1.75E-16	1.90E-16	1.76E-16	1.38E-16	1.54E-16	1.40E-16	1.48E-16	1.45E-16
1.83E-16	1.79E-16	1.82E-16	1.77E-16	1.82E-16	1.77E-16	1.69E-16	1.73E-16	1.68E-16	1.51E-16	1.34E-16	1.38E-16	1.30E-16	1.43E-16	1.43E-16
1.74E-16	1.59E-16	1.61E-16	1.71E-16	1.73E-16	1.62E-16	1.63E-16	1.79E-16	1.51E-16	1.38E-16	1.37E-16	1.24E-16	1.28E-16	1.39E-16	1.26E-16
1.74E-16	1.65E-16	1.44E-16	1.55E-16	1.51E-16	1.52E-16	1.51E-16	1.66E-16	1.33E-16	1.28E-16	1.41E-16	1.26E-16	1.22E-16	1.22E-16	1.10E-16
1.59E-16	1.57E-16	1.31E-16	1.32E-16	1.25E-16	1.29E-16	1.31E-16	1.46E-16	1.22E-16	1.31E-16	1.24E-16	1.30E-16	1.09E-16	1.07E-16	9.48E-17
1.19E-16	1.26E-16	1.15E-16	1.13E-16	1.10E-16	1.06E-16	1.01E-16	1.24E-16	1.08E-16	1.13E-16	9.91E-17	1.17E-16	1.06E-16	9.70E-17	8.23E-17
1.04E-16	1.07E-16	9.70E-17	9.71E-17	1.08E-16	9.31E-17	8.29E-17	1.01E-16	9.16E-17	8.83E-17	8.57E-17	9.93E-17	9.34E-17	8.63E-17	7.40E-17
9.60E-17	9.51E-17	8.29E-17	8.34E-17	1.10E-16	9.75E-17	8.04E-17	8.89E-17	7.09E-17	7.71E-17	7.98E-17	8.62E-17	6.95E-17	7.31E-17	6.35E-17
7.29E-17	7.44E-17	6.34E-17	7.42E-17	1.02E-16	8.75E-17	7.76E-17	7.66E-17	5.82E-17	5.75E-17	6.93E-17	7.18E-17	5.06E-17	5.59E-17	5.47E-17
5.85E-17	5.86E-17	5.37E-17	7.34E-17	8.48E-17	5.88E-17	6.79E-17	6.70E-17	4.82E-17	4.61E-17	6.25E-17	6.36E-17	4.01E-17	4.19E-17	4.42E-17
4.42E-17	4.58E-17	4.89E-17	6.43E-17	7.29E-17	4.40E-17	4.78E-17	5.68E-17	3.82E-17	3.18E-17	4.66E-17	4.77E-17	3.16E-17	3.98E-17	3.45E-17
3.43E-17	3.35E-17	4.68E-17	5.47E-17	5.35E-17	3.27E-17	3.25E-17	4.44E-17	2.88E-17	2.33E-17	3.24E-17	3.38E-17	2.19E-17	3.32E-17	2.47E-17
3.60E-17	3.14E-17	4.88E-17	5.16E-17	3.60E-17	1.91E-17	2.57E-17	2.84E-17	2.31E-17	2.17E-17	2.77E-17	2.81E-17	1.56E-17	2.12E-17	1.95E-17
3.19E-17	2.91E-17	3.89E-17	4.29E-17	2.67E-17	1.44E-17	2.24E-17	2.17E-17	2.16E-17	1.94E-17	2.36E-17	2.36E-17	1.22E-17	1.54E-17	1.73E-17
2.19E-17	2.40E-17	2.23E-17	3.00E-17	1.86E-17	1.14E-17	1.94E-17	1.91E-17	1.64E-17	1.36E-17	2.05E-17	1.62E-17	1.10E-17	1.48E-17	1.54E-17
1.64E-17	1.79E-17	1.71E-17	2.40E-17	1.58E-17	6.15E-18	1.60E-17	1.21E-17	9.62E-18	9.93E-18	1.66E-17	1.03E-17	7.44E-18	9.23E-18	1.23E-17
1.29E-17	1.20E-17	9.84E-18	1.35E-17	8.15E-18	2.51E-18	4.11E-18	7.45E-18	7.40E-18	9.80E-18	1.27E-17	5.97E-18	4.09E-18	4.26E-18	4.13E-18
8.60E-18	8.35E-18	3.44E-18	6.38E-18	3.00E-18	5.12E-19	1.89E-18	4.88E-18	5.44E-18	8.01E-18	5.47E-18	3.65E-18	2.11E-18	1.05E-18	5.95E-19
6.50E-18	5.25E-18	1.86E-18	2.09E-18	6.16E-19	5.61E-20	5.25E-19	3.08E-18	4.08E-18	2.98E-18	9.28E-19	1.26E-18	6.98E-19	1.49E-19	9.59E-21
3.23E-18	3.89E-18	1.35E-18	1.17E-18	7.38E-20	2.07E-21	4.34E-20	6.20E-19	4.30E-19	1.80E-21	3.23E-21	4.72E-21	2.34E-22	2.96E-20	1.06E-19
4.28E-19	1.30E-18	6.49E-19	1.37E-18	4.83E-22	1.06E-20	1.86E-20	7.35E-21	7.85E-20	8.50E-19	2.33E-19	3.30E-20	2.26E-19	6.49E-19	8.99E-22
2.37E-19	1.55E-21	7.84E-20	3.74E-19	1.47E-19	1.84E-19	2.37E-19	3.32E-19	6.43E-19	1.12E-18	1.38E-18	4.11E-19	9.97E-19	1.54E-18	9.42E-21
3.15E-18	2.28E-18	2.35E-18	9.45E-19	1.23E-18	2.17E-19	1.31E-20	1.33E-18	2.93E-18	3.06E-18	4.45E-18	2.88E-18	1.51E-18	1.77E-18	4.81E-19
5.15E-18	4.42E-18	4.54E-18	3.73E-18	3.85E-18	3.93E-19	4.60E-19	1.51E-18	4.32E-18	4.47E-18	6.35E-18	5.07E-18	2.57E-18	2.61E-18	2.34E-18
6.33E-18	7.23E-18	8.53E-18	1.10E-17	9.06E-18	3.15E-18	1.58E-18	2.98E-18	1.01E-17	7.51E-18	8.16E-18	8.39E-18	6.17E-18	4.98E-18	5.43E-18
9.01E-18	1.45E-17	1.40E-17	1.39E-17	1.14E-17	5.04E-18	3.72E-18	5.25E-18	1.53E-17	9.94E-18	9.92E-18	1.43E-17	9.86E-18	1.23E-17	9.74E-18
1.11E-17	2.19E-17	1.76E-17	1.47E-17	1.04E-17	5.40E-18	8.33E-18	9.28E-18	1.79E-17	1.07E-17	1.28E-17	2.25E-17	1.44E-17	1.90E-17	1.66E-17
1.82E-17	2.80E-17	1.91E-17	1.67E-17	7.47E-18	3.49E-18	1.09E-17	1.42E-17	2.25E-17	1.33E-17	1.86E-17	3.00E-17	2.22E-17	2.82E-17	2.21E-17
2.66E-17	2.91E-17	2.39E-17	2.13E-17	7.86E-18	2.71E-18	1.23E-17	1.97E-17	2.23E-17	1.93E-17	2.49E-17	3.51E-17	3.13E-17	3.27E-17	2.39E-17
3.15E-17	3.55E-17	3.19E-17	2.89E-17	1.61E-17	8.03E-18	1.78E-17	3.09E-17	2.46E-17	2.49E-17	3.46E-17	4.78E-17	4.15E-17	3.50E-17	2.43E-17
3.92E-17	4.31E-17	3.60E-17	3.97E-17	3.41E-17	2.62E-17	2.68E-17	3.50E-17	2.76E-17	3.63E-17	4.34E-17	5.31E-17	4.36E-17	3.75E-17	2.82E-17
5.59E-17	5.07E-17	4.39E-17	5.01E-17	5.03E-17	3.99E-17	3.49E-17	4.24E-17	2.78E-17	3.76E-17	4.51E-17	5.51E-17	4.30E-17	3.89E-17	2.85E-17
6.93E-17	5.88E-17	4.91E-17	5.48E-17	5.91E-17	4.85E-17	4.37E-17	5.57E-17	2.96E-17	3.56E-17	4.72E-17	5.86E-17	4.54E-17	4.81E-17	3.07E-17
7.03E-17	6.61E-17	5.67E-17	6.01E-17	6.91E-17	6.48E-17	6.20E-17	7.34E-17	3.38E-17	3.79E-17	4.88E-17	7.19E-17	5.83E-17	5.94E-17	4.15E-17
6.76E-17	7.66E-17	6.52E-17	7.45E-17	7.91E-17	7.98E-17	8.34E-17	8.61E-17	4.64E-17	4.20E-17	6.23E-17	7.99E-17	6.84E-17	6.83E-17	5.55E-17
7.77E-17	8.17E-17	7.17E-17	7.80E-17	8.67E-17	9.20E-17	9.70E-17	9.54E-17	5.86E-17	4.98E-17	7.16E-17	8.52E-17	7.05E-17	7.04E-17	6.42E-17
1.01E-16	9.43E-17	8.20E-17	6.92E-17	8.78E-17	9.71E-17	9.55E-17	1.02E-16	7.41E-17	6.20E-17	7.16E-17	8.88E-17	7.58E-17	6.70E-17	6.49E-17
1.22E-16	1.12E-16	9.41E-17	6.90E-17	8.72E-17	1.06E-16	1.01E-16	1.09E-16	9.53E-17	7.52E-17	7.76E-17	8.69E-17	7.79E-17	5.61E-17	6.24E-17
1.35E-16	1.25E-16	1.03E-16	9.49E-17	8.79E-17	1.07E-16	1.09E-16	1.20E-16	1.14E-16	8.88E-17	8.66E-17	8.98E-17	7.79E-17	5.90E-17	5.59E-17
1.36E-16	1.36E-16	1.16E-16	1.17E-16	9.73E-17	1.05E-16	1.22E-16	1.31E-16	1.23E-16	1.15E-16	9.59E-17	9.89E-17	7.59E-17	7.22E-17	6.80E-17
1.42E-16	1.30E-16	1.22E-16	1.30E-16	1.05E-16	1.08E-16	1.40E-16	1.52E-16	1.36E-16	1.46E-16	1.07E-16	1.17E-16	8.78E-17	9.23E-17	8.73E-17
1.55E-16	1.45E-16	1.23E-16	1.37E-16	1.08E-16	1.34E-16	1.61E-16	1.58E-16	1.51E-16	1.63E-16	1.27E-16	1.30E-16	9.90E-17	1.16E-16	1.15E-16
1.68E-16	1.81E-16	1.45E-16	1.46E-16	1.19E-16	1.59E-16	1.77E-16	1.62E-16	1.70E-16	1.70E-16	1.53E-16	1.44E-16	1.26E-16	1.37E-16	1.25E-16
1.71E-16	2.05E-16	1.62E-16	1.61E-16	1.49E-16	1.79E-16	1.85E-16	1.66E-16	1.83E-16	1.70E-16	1.59E-16	1.60E-16	1.53E-16	1.55E-16	1.32E-16
1.82E-16	2.05E-16	1.74E-16	1.75E-16	1.76E-16	1.93E-16	1.98E-16	1.82E-16	1.91E-16	1.66E-16	1.53E-16	1.64E-16	1.81E-16	1.75E-16	1.46E-16
1.81E-16	1.89E-16	1.84E-16	1.84E-16	1.84E-16	1.95E-16	2.03E-16	1.94E-16	2.03E-16	1.71E-16	1.69E-16	1.71E-16	1.95E-16	1.81E-16	1.55E-16
1.90E-16	1.89E-16	1.90E-16	2.01E-16	1.97E-16	1.93E-16	1.97E-16	1.98E-16	2.24E-16	1.95E-16	2.01E-16	1.87E-16	1.94E-16	1.88E-16	1.51E-16
2.05E-16	1.90E-16	2.04E-16	2.24E-16	2.16E-16	2.06E-16	2.09E-16	2.14E-16	2.40E-16	2.10E-16	2.28E-16	2.06E-16	2.07E-16	1.97E-16	1.73E-16
2.25E-16	1.94E-16	2.27E-16	2.44E-16	2.40E-16	2.44E-16	2.33E-16	2.39E-16	2.56E-16	2.36E-16	2.41E-16	2.31E-16	2.42E-16	2.12E-16	2.14E-16
2.35E-16	2.10E-16	2.51E-16	2.68E-16	2.62E-16	2.69E-16	2.56E-16	2.58E-16	2.69E-16	2.57E-16	2.56E-16	2.46E-16	2.63E-16	2.32E-16	2.38E-16
2.52E-16	2.50E-16	2.81E-16	2.95E-16	2.78E-16	2.91E-16	2.90E-16	2.78E-16	2.88E-16	2.69E-16	2.62E-16	2.44E-16	2.67E-16	2.57E-16	2.61E-16
2.53E-16	2.74E-16	2.99E-16	2.98E-16	2.79E-16	2.97E-16	3.02E-16	2.89E-16	2.99E-16	2.73E-16	2.75E-16	2.49E-16	2.67E-16	2.86E-16	2.87E-16
2.76E-16	3.06E-16	3.20E-16	3.05E-16	2.87E-16	3.04E-16	3.17E-16	3.03E-16	3.09E-16	2.79E-16	2.87E-16	2.67E-16	2.82E-16	3.16E-16	3.08E-16
3.25E-16	3.51E-16	3.49E-16	3.13E-16	3.01E-16	3.12E-16	3.26E-16	3.23E-16	3.25E-16	2.95E-16	2.98E-16	2.91E-16	3.14E-16	3.37E-16	3.25E-16
3.50E-16	3.71E-16	3.60E-16	3.24E-16	3.17E-16	3.34E-16	3.35E-16	3.39E-16	3.47E-16	3.28E-16	3.12E-16	3.19E-16	3.33E-16	3.41E-16	3.31E-16
3.60E-16	3.69E-16	3.64E-16	3.45E-16	3.30E-16	3.46E-16	3.58E-16	3.49E-16	3.49E-16	3.50E-16	3.36E-16	3.43E-16	3.50E-16	3.46E-16	3.42E-16
3.44E-16	3.62E-16	3.57E-16	3.63E-16	3.49E-16	3.52E-16	3.66E-16	3.71E-16	3.62E-16	3.66E-16	3.48E-16	3.49E-16	3.59E-16	3.64E-16	3.55E-16
3.43E-16	3.52E-16	3.58E-16	3.68E-16	3.66E-16	3.66E-16	3.63E-16	3.76E-16	3.88E-16	3.77E-16	3.52E-16	3.70E-16	3.73E-16	3.64E-16	3.54E-16
3.52E-16	3.49E-16	3.73E-16	3.85E-16	3.76E-16	3.64E-16	3.75E-16	3.85E-16	4.02E-16	3.95E-16	3.65E-16	4.03E-16	3.89E-16	3.51E-16	3.46E-16
3.68E-16	3.78E-16	3.92E-16	3.84E-16	3.82E-16	3.61E-16	3.98E-16	3.87E-16	4.11E-16	3.99E-16	3.75E-16	3.99E-16	3.90E-16	3.55E-16	3.45E-16
4.01E-16	3.95E-16	4.06E-16	3.87E-16	3.95E-16	3.79E-16	4.12E-16	4.01E-16	4.09E-16	4.05E-16	3.82E-16	3.83E-16	3.84E-16	3.65E-16	3.47E-16
4.32E-16	4.09E-16	4.18E-16	3.85E-16	4.11E-16	4.09E-16	4.09E-16	4.01E-16	4.21E-16	4.24E-16	3.97E-16	3.88E-16	3.84E-16	3.72E-16	3.64E-16
4.49E-16	4.25E-16	4.30E-16	3.99E-16	4.37E-16	4.43E-16	4.27E-16	4.25E-16	4.30E-16	4.17E-16	4.19E-16	3.94E-16	3.81E-16	3.92E-16	3.93E-16
4.76E-16	4.38E-16	4.71E-16	4.62E-16	4.77E-16	4.81E-16	4.59E-16	4.61E-16	4.66E-16	4.43E-16	4.28E-16	4.13E-16	4.03E-16	4.19E-16	4.35E-16
5.02E-16	4.66E-16	4.85E-16	5.00E-16	5.14E-16	5.00E-16	4.68E-16	4.98E-16	4.92E-16	4.70E-16	4.42E-16	4.46E-16	4.39E-16	4.42E-16	4.70E-16
5.25E-16	4.61E-16	4.80E-16	5.09E-16	5.24E-16	5.08E-16	5.07E-16	5.47E-16	5.25E-16	4.99E-16	4.70E-16	4.73E-16	4.60E-16	4.52E-16	4.81E-16
5.29E-16	4.99E-16	4.82E-16	5.27E-16	5.25E-16	5.28E-16	5.43E-16	5.56E-16	5.45E-16	5.17E-16	4.76E-16	4.92E-16	4.65E-16	4.80E-16	4.84E-16
5.40E-16	5.36E-16	5.13E-16	5.57E-16	5.44E-16	5.68E-16	5.72E-16	5.44E-16	5.39E-16	5.18E-16	4.63E-16	5.12E-16	4.82E-16	5.02E-16	4.83E-16
5.43E-16	5.62E-16	5.58E-16	5.89E-16	5.60E-16	5.91E-16	5.67E-16	5.41E-16	5.21E-16	5.36E-16	4.77E-16	5.23E-16	5.07E-16	4.97E-16	4.77E-16
5.39E-16	5.75E-16	6.05E-16	6.15E-16	5.83E-16	5.99E-16	5.84E-16	5.72E-16	5.14E-16	5.37E-16	4.93E-16	5.24E-16	5.06E-16	4.92E-16	4.86E-16
5.34E-16	5.84E-16	6.39E-16	6.39E-16	5.97E-16	5.84E-16	6.08E-16	6.07E-16	5.43E-16	5.55E-16	4.92E-16	5.25E-16	4.90E-16	4.95E-16	5.14E-16
5.28E-16	5.84E-16	6.42E-16	6.48E-16	6.23E-16	5.99E-16	6.13E-16	6.38E-16	5.84E-16	5.79E-16	5.04E-16	5.47E-16	4.86E-16	4.92E-16	5.53E-16
5.65E-16	6.06E-16	6.41E-16	6.78E-16	6.55E-16	6.33E-16	6.03E-16	6.69E-16	6.22E-16	6.21E-16	5.47E-16	5.73E-16	5.32E-16	5.08E-16	5.75E-16
6.00E-16	6.53E-16	6.53E-16	6.99E-16	6.82E-16	6.53E-16	6.16E-16	6.70E-16	6.54E-16	6.47E-16	6.10E-16	6.22E-16	5.84E-16	5.49E-16	5.73E-16
6.22E-16	7.01E-16	6.89E-16	7.21E-16	6.84E-16	6.68E-16	6.26E-16	6.68E-16	6.85E-16	6.82E-16	6.60E-16	6.47E-16	6.33E-16	6.07E-16	6.04E-16
6.59E-16	7.29E-16	7.22E-16	7.31E-16	6.75E-16	6.78E-16	6.26E-16	6.80E-16	6.94E-16	7.06E-16	6.75E-16	6.37E-16	6.59E-16	6.53E-16	6.45E-16
6.90E-16	7.43E-16	7.25E-16	7.36E-16	6.73E-16	6.78E-16	6.47E-16	7.03E-16	7.19E-16	7.22E-16	6.84E-16	6.29E-16	6.87E-16	6.99E-16	7.03E-16
7.01E-16	7.62E-16	7.53E-16	7.55E-16	6.90E-16	6.65E-16	6.56E-16	6.99E-16	7.29E-16	7.35E-16	7.01E-16	6.49E-16	7.09E-16	7.34E-16	7.37E-16
7.04E-16	7.65E-16	7.73E-16	7.67E-16	7.23E-16	6.67E-16	6.81E-16	6.74E-16	7.31E-16	7.27E-16	6.93E-16	6.57E-16	7.28E-16	7.34E-16	7.27E-16
7.24E-16	7.94E-16	7.81E-16	7.85E-16	7.56E-16	7.10E-16	7.19E-16	7.07E-16	7.36E-16	7.08E-16	6.64E-16	6.66E-16	7.15E-16	7.11E-16	7.07E-16
7.62E-16	8.04E-16	7.83E-16	8.09E-16	7.95E-16	7.51E-16	7.61E-16	7.52E-16	7.73E-16	7.45E-16	6.78E-16	6.78E-16	6.72E-16	6.60E-16	6.80E-16
7.63E-16	7.88E-16	7.76E-16	8.10E-16	8.22E-16	7.81E-16	7.72E-16	7.70E-16	7.81E-16	7.60E-16	6.99E-16	6.71E-16	6.33E-16	6.06E-16	6.46E-16
7.47E-16	7.56E-16	7.69E-16	8.02E-16	8.10E-16	7.84E-16	7.62E-16	7.73E-16	7.68E-16	7.49E-16	7.16E-16	6.70E-16	6.34E-16	5.92E-16	6.16E-16

7.46E-16	7.24E-16	7.53E-16	7.74E-16	7.80E-16	7.65E-16	7.59E-16	7.62E-16	7.49E-16	7.50E-16	7.28E-16	6.78E-16	6.45E-16	6.01E-16	6.06E-16
7.77E-16	7.31E-16	7.53E-16	7.56E-16	7.53E-16	7.33E-16	7.39E-16	7.34E-16	7.49E-16	7.53E-16	7.14E-16	6.83E-16	6.28E-16	5.89E-16	6.01E-16
7.77E-16	7.34E-16	7.76E-16	7.74E-16	7.78E-16	7.16E-16	7.13E-16	7.05E-16	7.34E-16	7.35E-16	6.92E-16	6.77E-16	6.28E-16	5.75E-16	6.03E-16
7.79E-16	7.59E-16	7.90E-16	7.86E-16	8.12E-16	7.38E-16	7.19E-16	6.86E-16	6.99E-16	6.84E-16	6.63E-16	6.53E-16	6.38E-16	6.05E-16	6.03E-16
7.96E-16	7.83E-16	8.01E-16	7.99E-16	8.26E-16	7.61E-16	7.28E-16	6.58E-16	6.50E-16	6.37E-16	5.97E-16	6.00E-16	6.32E-16	6.31E-16	6.09E-16
8.01E-16	8.00E-16	8.17E-16	8.13E-16	8.21E-16	7.46E-16	6.95E-16	6.22E-16	6.13E-16	6.09E-16	5.64E-16	5.58E-16	6.21E-16	6.17E-16	6.10E-16
8.02E-16	8.03E-16	8.20E-16	7.90E-16	8.14E-16	7.27E-16	6.71E-16	6.04E-16	6.01E-16	6.00E-16	5.74E-16	5.58E-16	6.28E-16	6.16E-16	6.01E-16
8.01E-16	8.19E-16	8.19E-16	7.78E-16	7.73E-16	7.11E-16	6.73E-16	6.07E-16	5.80E-16	6.05E-16	6.28E-16	5.95E-16	6.19E-16	6.25E-16	6.14E-16
8.33E-16	8.34E-16	8.15E-16	7.72E-16	7.40E-16	7.01E-16	6.91E-16	6.36E-16	5.88E-16	6.17E-16	6.40E-16	6.02E-16	6.01E-16	6.08E-16	6.15E-16
8.54E-16	8.36E-16	8.09E-16	7.49E-16	7.10E-16	6.91E-16	6.86E-16	6.56E-16	6.15E-16	6.02E-16	6.28E-16	6.17E-16	5.96E-16	5.98E-16	5.89E-16
8.30E-16	8.23E-16	8.09E-16	7.53E-16	7.16E-16	7.12E-16	6.73E-16	6.67E-16	6.24E-16	6.04E-16	6.04E-16	6.21E-16	6.01E-16	6.00E-16	5.93E-16
6.77E-14	6.82E-14	6.79E-14	6.73E-14	6.59E-14	6.52E-14	6.45E-14	6.49E-14	6.41E-14	6.26E-14	6.04E-14	6.07E-14	5.99E-14	5.88E-14	5.85E-14

4.71E-08	4.68E-08	4.73E-08	4.74E-08	4.89E-08	4.93E-08	4.87E-08	4.92E-08	4.78E-08	4.64E-08	4.48E-08	4.45E-08	4.46E-08	4.46E-08	4.40E-08
4.58E-08	4.55E-08	4.63E-08	4.64E-08	4.84E-08	4.88E-08	4.84E-08	4.93E-08	4.70E-08	4.50E-08	4.40E-08	4.37E-08	4.36E-08	4.35E-08	4.31E-08
4.45E-08	4.44E-08	4.45E-08	4.53E-08	4.77E-08	4.80E-08	4.75E-08	4.84E-08	4.61E-08	4.35E-08	4.30E-08	4.26E-08	4.26E-08	4.26E-08	4.25E-08
4.30E-08	4.31E-08	4.34E-08	4.43E-08	4.66E-08	4.60E-08	4.52E-08	4.62E-08	4.42E-08	4.25E-08	4.15E-08	4.18E-08	4.20E-08	4.15E-08	4.16E-08
4.19E-08	4.22E-08	4.30E-08	4.27E-08	4.43E-08	4.31E-08	4.30E-08	4.39E-08	4.27E-08	4.14E-08	4.03E-08	4.06E-08	4.11E-08	4.03E-08	4.08E-08
4.13E-08	4.09E-08	4.19E-08	4.14E-08	4.22E-08	4.13E-08	4.16E-08	4.19E-08	4.17E-08	4.01E-08	3.94E-08	3.99E-08	4.01E-08	3.94E-08	4.04E-08
4.03E-08	4.01E-08	4.05E-08	4.04E-08	4.06E-08	4.05E-08	4.05E-08	4.12E-08	4.08E-08	3.94E-08	3.91E-08	3.91E-08	3.90E-08	3.82E-08	3.94E-08
4.02E-08	4.02E-08	3.97E-08	3.96E-08	3.95E-08	3.98E-08	3.99E-08	4.04E-08	3.96E-08	3.85E-08	3.81E-08	3.81E-08	3.77E-08	3.75E-08	3.63E-08
4.03E-08	4.00E-08	3.94E-08	3.92E-08	3.87E-08	3.88E-08	3.90E-08	3.91E-08	3.86E-08	3.77E-08	3.72E-08	3.74E-08	3.69E-08	3.65E-08	3.52E-08
3.94E-08	3.94E-08	3.90E-08	3.84E-08	3.75E-08	3.79E-08	3.81E-08	3.75E-08	3.82E-08	3.67E-08	3.64E-08	3.75E-08	3.67E-08	3.59E-08	3.45E-08
3.76E-08	3.76E-08	3.74E-08	3.74E-08	3.64E-08	3.74E-08	3.72E-08	3.67E-08	3.72E-08	3.51E-08	3.56E-08	3.76E-08	3.64E-08	3.46E-08	3.36E-08
3.59E-08	3.56E-08	3.51E-08	3.57E-08	3.53E-08	3.76E-08	3.64E-08	3.59E-08	3.51E-08	3.35E-08	3.45E-08	3.79E-08	3.57E-08	3.38E-08	3.34E-08
3.43E-08	3.43E-08	3.37E-08	3.41E-08	3.39E-08	3.65E-08	3.51E-08	3.43E-08	3.31E-08	3.24E-08	3.33E-08	3.64E-08	3.47E-08	3.30E-08	3.25E-08
3.30E-08	3.32E-08	3.24E-08	3.23E-08	3.28E-08	3.47E-08	3.37E-08	3.25E-08	3.21E-08	3.20E-08	3.23E-08	3.49E-08	3.38E-08	3.21E-08	3.13E-08
3.12E-08	3.15E-08	3.21E-08	3.12E-08	3.18E-08	3.27E-08	3.19E-08	3.15E-08	3.23E-08	3.21E-08	3.15E-08	3.29E-08	3.26E-08	3.16E-08	3.04E-08
2.97E-08	2.95E-08	3.30E-08	3.02E-08	3.10E-08	3.03E-08	3.01E-08	3.08E-08	3.18E-08	3.14E-08	3.11E-08	3.16E-08	3.08E-08	3.02E-08	2.94E-08
2.85E-08	2.79E-08	3.40E-08	2.90E-08	2.99E-08	2.91E-08	2.88E-08	2.98E-08	3.03E-08	2.97E-08	3.01E-08	2.99E-08	2.93E-08	2.90E-08	2.86E-08
2.74E-08	2.69E-08	2.78E-08	2.79E-08	2.93E-08	2.85E-08	2.83E-08	2.80E-08	2.86E-08	2.77E-08	2.84E-08	2.86E-08	2.79E-08	2.80E-08	2.71E-08
2.64E-08	2.61E-08	2.60E-08	2.68E-08	2.82E-08	2.77E-08	2.75E-08	2.67E-08	2.72E-08	2.67E-08	2.68E-08	2.72E-08	2.65E-08	2.64E-08	2.54E-08
2.52E-08	2.51E-08	2.47E-08	2.52E-08	2.57E-08	2.67E-08	2.66E-08	2.61E-08	2.67E-08	2.66E-08	2.63E-08	2.55E-08	2.47E-08	2.47E-08	2.35E-08
2.43E-08	2.40E-08	2.35E-08	2.44E-08	2.43E-08	2.56E-08	2.58E-08	2.51E-08	2.58E-08	2.62E-08	2.52E-08	2.39E-08	2.26E-08	2.26E-08	2.17E-08
2.36E-08	2.30E-08	2.24E-08	2.36E-08	2.32E-08	2.41E-08	2.43E-08	2.29E-08	2.40E-08	2.52E-08	2.37E-08	2.20E-08	2.14E-08	2.14E-08	2.04E-08
2.27E-08	2.16E-08	2.17E-08	2.17E-08	2.18E-08	2.24E-08	2.33E-08	2.22E-08	2.23E-08	2.34E-08	2.23E-08	2.09E-08	2.05E-08	2.06E-08	1.97E-08
2.20E-08	2.06E-08	2.02E-08	2.07E-08	2.04E-08	2.07E-08	2.20E-08	2.24E-08	2.16E-08	2.15E-08	2.04E-08	1.99E-08	1.94E-08	1.90E-08	1.88E-08
2.10E-08	1.97E-08	1.92E-08	2.00E-08	1.92E-08	2.00E-08	2.16E-08	2.23E-08	2.16E-08	2.02E-08	1.91E-08	1.88E-08	1.85E-08	1.78E-08	1.80E-08
2.02E-08	1.90E-08	1.85E-08	1.91E-08	1.87E-08	1.95E-08	2.04E-08	2.05E-08	2.00E-08	1.86E-08	1.79E-08	1.76E-08	1.72E-08	1.72E-08	1.77E-08
1.93E-08	1.84E-08	1.78E-08	1.77E-08	1.82E-08	1.91E-08	1.82E-08	1.79E-08	1.79E-08	1.70E-08	1.68E-08	1.69E-08	1.62E-08	1.63E-08	1.68E-08
1.86E-08	1.77E-08	1.65E-08	1.68E-08	1.81E-08	1.93E-08	1.74E-08	1.66E-08	1.59E-08	1.57E-08	1.62E-08	1.62E-08	1.52E-08	1.54E-08	1.54E-08
1.77E-08	1.74E-08	1.59E-08	1.65E-08	1.76E-08	1.95E-08	1.74E-08	1.58E-08	1.47E-08	1.50E-08	1.57E-08	1.55E-08	1.48E-08	1.49E-08	1.46E-08
1.66E-08	1.69E-08	1.57E-08	1.59E-08	1.71E-08	1.81E-08	1.63E-08	1.46E-08	1.44E-08	1.46E-08	1.46E-08	1.43E-08	1.46E-08	1.47E-08	1.41E-08
1.62E-08	1.58E-08	1.50E-08	1.48E-08	1.55E-08	1.51E-08	1.44E-08	1.38E-08	1.42E-08	1.42E-08	1.35E-08	1.38E-08	1.39E-08	1.38E-08	1.35E-08
1.54E-08	1.54E-08	1.48E-08	1.38E-08	1.35E-08	1.26E-08	1.30E-08	1.26E-08	1.33E-08	1.34E-08	1.27E-08	1.27E-08	1.28E-08	1.26E-08	1.22E-08
1.48E-08	1.51E-08	1.45E-08	1.33E-08	1.27E-08	1.18E-08	1.23E-08	1.19E-08	1.21E-08	1.30E-08	1.25E-08	1.19E-08	1.21E-08	1.15E-08	1.09E-08
1.36E-08	1.38E-08	1.29E-08	1.29E-08	1.26E-08	1.14E-08	1.16E-08	1.17E-08	1.15E-08	1.20E-08	1.17E-08	1.11E-08	1.15E-08	1.03E-08	9.89E-09
1.23E-08	1.29E-08	1.20E-08	1.29E-08	1.28E-08	1.18E-08	1.20E-08	1.15E-08	1.08E-08	1.06E-08	1.05E-08	1.01E-08	1.04E-08	9.38E-09	9.12E-09
1.15E-08	1.22E-08	1.20E-08	1.29E-08	1.28E-08	1.26E-08	1.32E-08	1.11E-08	9.82E-09	9.05E-09	9.25E-09	8.99E-09	8.58E-09	8.34E-09	7.91E-09
1.08E-08	1.11E-08	1.14E-08	1.26E-08	1.28E-08	1.29E-08	1.39E-08	1.07E-08	8.69E-09	8.03E-09	8.09E-09	7.78E-09	7.45E-09	7.27E-09	7.25E-09
1.00E-08	1.01E-08	1.09E-08	1.19E-08	1.16E-08	1.14E-08	1.24E-08	9.60E-09	7.74E-09	7.33E-09	7.40E-09	7.04E-09	6.94E-09	6.65E-09	6.85E-09
9.67E-09	9.68E-09	1.00E-08	1.04E-08	9.57E-09	9.06E-09	1.00E-08	8.25E-09	7.02E-09	6.68E-09	6.78E-09	6.36E-09	6.49E-09	6.25E-09	6.85E-09
9.14E-09	9.17E-09	9.19E-09	9.34E-09	8.48E-09	7.28E-09	8.55E-09	7.64E-09	6.76E-09	5.86E-09	6.13E-09	5.68E-09	5.87E-09	5.41E-09	6.65E-09
8.45E-09	8.25E-09	8.17E-09	8.14E-09	8.13E-09	6.78E-09	7.56E-09	7.44E-09	6.97E-09	5.71E-09	6.00E-09	5.38E-09	5.40E-09	4.58E-09	6.39E-09
7.41E-09	7.59E-09	7.48E-09	6.93E-09	7.11E-09	6.32E-09	6.98E-09	6.69E-09	6.47E-09	5.56E-09	6.23E-09	5.34E-09	4.67E-09	4.02E-09	5.41E-09
6.49E-09	6.55E-09	6.71E-09	5.95E-09	6.17E-09	5.51E-09	5.71E-09	5.52E-09	5.39E-09	5.75E-09	6.38E-09	5.21E-09	4.37E-09	4.13E-09	4.60E-09
5.85E-09	5.47E-09	5.80E-09	5.62E-09	5.20E-09	4.91E-09	4.79E-09	4.83E-09	4.61E-09	5.71E-09	5.62E-09	4.59E-09	4.03E-09	3.92E-09	3.37E-09
5.28E-09	4.67E-09	4.86E-09	5.02E-09	4.76E-09	4.74E-09	4.32E-09	4.04E-09	4.42E-09	5.11E-09	4.63E-09	3.76E-09	4.14E-09	3.47E-09	2.83E-09
4.62E-09	3.90E-09	3.64E-09	4.21E-09	4.40E-09	4.17E-09	3.10E-09	3.38E-09	4.12E-09	3.53E-09	3.10E-09	2.66E-09	3.65E-09	2.94E-09	2.32E-09
3.61E-09	3.20E-09	3.01E-09	3.54E-09	3.65E-09	3.08E-09	2.72E-09	3.28E-09	3.87E-09	2.83E-09	2.37E-09	1.88E-09	3.04E-09	2.73E-09	2.35E-09
3.14E-09	2.48E-09	2.15E-09	3.04E-09	2.36E-09	2.49E-09	2.33E-09	2.96E-09	3.18E-09	1.90E-09	1.91E-09	1.02E-09	2.71E-09	2.72E-09	1.84E-09
2.88E-09	2.22E-09	1.74E-09	2.46E-09	1.46E-09	1.88E-09	1.71E-09	2.26E-09	2.25E-09	1.63E-09	1.22E-09	2.72E-10	2.12E-09	2.10E-09	1.12E-09
2.01E-09	1.92E-09	1.35E-09	1.94E-09	1.28E-09	1.41E-09	9.57E-10	1.21E-09	1.46E-09	1.21E-09	5.49E-10	-1.46E-10	7.62E-10	1.20E-09	5.20E-10
1.08E-09	1.47E-09	1.35E-09	1.53E-09	6.72E-10	7.89E-10	-3.13E-10	3.36E-10	7.02E-10	6.49E-10	-2.85E-10	-3.09E-11	-9.55E-11	2.15E-10	-2.22E-10
2.02E-10	6.96E-10	1.07E-09	1.06E-09	-1.17E-10	1.26E-11	-8.55E-10	-4.42E-10	-9.68E-11	-3.54E-10	-9.46E-10	-6.92E-11	-6.40E-10	-8.16E-10	-7.83E-10
-2.09E-10	1.74E-10	7.93E-10	3.30E-10	-7.30E-10	-5.34E-10	-1.27E-09	-1.44E-09	-9.15E-10	-1.11E-09	-1.29E-09	-1.82E-10	-4.91E-10	-1.21E-09	-9.47E-10
-2.32E-10	-3.22E-10	-9.22E-11	3.50E-12	-1.36E-09	-8.57E-10	-2.10E-09	-1.65E-09	-1.33E-09	-1.49E-09	-9.23E-10	-2.44E-10	-8.26E-10	-1.38E-09	-1.11E-09
8.37E-11	-5.77E-10	-7.69E-10	-2.87E-10	-2.10E-09	-1.98E-09	-3.12E-09	-2.19E-09	-1.84E-09	-1.67E-09	-7.54E-10	-1.42E-10	-8.04E-10	-8.93E-10	-1.82E-09
-1.96E-10	-1.09E-09	-1.68E-09	-1.15E-09	-2.78E-09	-3.17E-09	-3.77E-09	-2.92E-09	-2.75E-09	-2.32E-09	-1.60E-09	-1.52E-09	-1.40E-09	-1.88E-09	-3.22E-09
-1.19E-09	-2.08E-09	-2.74E-09	-2.86E-09	-3.52E-09	-3.85E-09	-4.20E-09	-3.87E-09	-3.55E-09	-3.09E-09	-3.18E-09	-2.82E-09	-2.52E-09	-3.21E-09	-3.87E-09
-1.50E-09	-2.71E-09	-3.69E-09	-3.95E-09	-3.95E-09	-4.07E-09	-4.47E-09	-4.36E-09	-4.17E-09	-3.59E-09	-4.20E-09	-3.46E-09	-4.03E-09	-4.22E-09	-4.67E-09
-2.30E-09	-2.99E-09	-3.99E-09	-4.38E-09	-4.19E-09	-3.98E-09	-5.30E-09	-4.45E-09	-4.53E-09	-3.73E-09	-4.74E-09	-4.88E-09	-4.77E-09	-4.87E-09	-5.38E-09
-3.32E-09	-2.89E-09	-4.49E-09	-4.28E-09	-3.97E-09	-4.14E-09	-5.45E-09	-4.77E-09	-5.23E-09	-4.36E-09	-5.12E-09	-5.80E-09	-5.28E-09	-5.35E-09	-5.80E-09
-4.01E-09	-3.18E-09	-4.91E-09	-4.80E-09	-3.89E-09	-3.94E-09	-5.20E-09	-5.20E-09	-5.65E-09	-5.30E-09	-5.61E-09	-6.00E-09	-5.87E-09	-5.41E-09	-6.17E-09
-4.66E-09	-4.30E-09	-5.15E-09	-5.24E-09	-4.52E-09	-4.59E-09	-5.22E-09	-5.70E-09	-5.81E-09	-5.63E-09	-6.08E-09	-6.40E-09	-6.17E-09	-5.40E-09	-6.34E-09
-5.49E-09	-5.25E-09	-5.20E-09	-5.58E-09	-5.37E-09	-5.15E-09	-5.97E-09	-6.15E-09	-6.13E-09	-5.87E-09	-5.96E-09	-6.73E-09	-6.14E-09	-5.51E-09	-6.69E-09
-5.98E-09	-5.78E-09	-5.39E-09	-6.44E-09	-6.49E-09	-5.83E-09	-6.85E-09	-6.93E-09	-6.25E-09	-5.88E-09	-5.88E-09	-7.11E-09	-6.09E-09	-5.59E-09	-7.07E-09
-6.49E-09	-6.31E-09	-6.02E-09	-7.17E-09	-7.00E-09	-6.52E-09	-7.80E-09	-8.52E-09	-7.08E-09	-6.00E-09	-7.20E-09	-7.27E-09	-6.42E-09	-5.75E-09	-7.40E-09
-6.50E-09	-6.96E-09	-6.11E-09	-7.62E-09	-7.41E-09	-7.37E-09	-8.30E-09	-9.23E-09	-8.19E-09	-6.94E-09	-7.71E-09	-7.27E-09	-6.04E-09	-5.64E-09	-7.60E-09
-6.43E-09	-7.40E-09	-7.05E-09	-7.59E-09	-7.67E-09	-7.89E-09	-8.96E-09	-9.98E-09	-9.14E-09	-7.73E-09	-7.80E-09	-7.25E-09	-6.00E-09	-6.06E-09	-7.84E-09
-7.30E-09	-8.11E-09	-8.34E-09	-7.85E-09	-7.59E-09	-8.33E-09	-9.62E-09	-1.02E-08	-9.60E-09	-7.98E-09	-8.02E-09	-7.42E-09	-6.61E-09	-7.24E-09	-8.63E-09
-8.43E-09	-8.75E-09	-8.81E-09	-8.32E-09	-7.97E-09	-9.12E-09	-9.50E-09	-1.03E-08	-1.00E-08	-8.93E-09	-8.43E-09	-8.24E-09	-8.71E-09	-8.51E-09	-9.73E-09
-9.18E-09	-9.14E-09	-9.30E-09	-8.88E-09	-8.85E-09	-9.74E-09	-9.93E-09	-1.01E-08	-1.03E-08	-9.82E-09	-9.50E-09	-9.26E-09	-9.77E-09	-9.32E-09	-1.04E-08
-9.05E-09	-9.32E-09	-9.53E-09	-9.81E-09	-9.13E-09	-1.00E-08	-1.01E-08	-9.75E-09	-1.02E-08	-1.05E-08	-1.00E-08	-1.05E-08	-1.05E-08	-1.02E-08	-1.13E-08
-8.83E-09	-9.08E-09	-9.81E-09	-1.02E-08	-9.78E-09	-1.03E-08	-1.04E-08	-9.91E-09	-9.66E-09	-1.12E-08	-1.08E-08	-1.11E-08	-1.09E-08	-1.03E-08	-1.14E-08
-8.45E-09	-8.67E-09	-9.11E-09	-9.97E-09	-9.59E-09	-1.01E-08	-1.07E-08	-1.04E-08	-1.02E-08	-1.14E-08	-1.07E-08	-1.13E-08	-1.16E-08	-1.07E-08	-1.11E-08
-7.95E-09	-8.28E-09	-8.17E-09	-8.97E-09	-9.16E-09	-9.77E-09	-1.11E-08	-1.07E-08	-1.14E-08	-1.18E-08	-9.52E-09	-1.18E-08	-1.24E-08	-1.17E-08	-1.14E-08
-8.78E-09	-9.05E-09	-8.28E-09	-8.72E-09	-8.79E-09	-9.79E-09	-1.18E-08	-1.13E-08	-1.20E-08	-1.21E-08	-8.37E-09	-1.23E-08	-1.28E-08	-1.19E-08	-1.17E-08
-1.13E-08	-1.04E-08	-9.09E-09	-9.50E-09	-9.52E-09	-1.11E-08	-1.22E-08	-1.21E-08	-1.24E-08	-1.23E-08	-1.17E-08	-1.26E-08	-1.31E-08	-1.20E-08	-1.11E-08
-1.24E-08	-1.15E-08	-1.02E-08	-1.01E-08	-1.03E-08	-1.18E-08	-1.32E-08	-1.26E-08	-1.28E-08	-1.27E-08	-1.33E-08	-1.30E-08	-1.32E-08	-1.24E-08	-1.10E-08
-1.25E-08	-1.12E-08	-1.15E-08	-1.09E-08	-1.19E-08	-1.32E-08	-1.39E-08	-1.32E-08	-1.32E-08	-1.33E-08	-1.37E-08	-1.32E-08	-1.33E-08	-1.27E-08	-1.16E-08

-1.25E-08	-1.13E-08	-1.20E-08	-1.25E-08	-1.29E-08	-1.45E-08	-1.43E-08	-1.36E-08	-1.39E-08	-1.38E-08	-1.41E-08	-1.32E-08	-1.33E-08	-1.31E-08	-1.23E-08
-1.23E-08	-1.22E-08	-1.31E-08	-1.33E-08	-1.40E-08	-1.55E-08	-1.49E-08	-1.41E-08	-1.43E-08	-1.44E-08	-1.42E-08	-1.36E-08	-1.30E-08	-1.32E-08	-1.28E-08
-1.27E-08	-1.35E-08	-1.37E-08	-1.39E-08	-1.51E-08	-1.59E-08	-1.51E-08	-1.47E-08	-1.49E-08	-1.42E-08	-1.41E-08	-1.38E-08	-1.28E-08	-1.34E-08	-1.34E-08
-1.34E-08	-1.42E-08	-1.44E-08	-1.43E-08	-1.55E-08	-1.64E-08	-1.59E-08	-1.55E-08	-1.54E-08	-1.36E-08	-1.41E-08	-1.43E-08	-1.33E-08	-1.36E-08	-1.36E-08
-1.50E-08	-1.51E-08	-1.48E-08	-1.49E-08	-1.56E-08	-1.63E-08	-1.60E-08	-1.61E-08	-1.55E-08	-1.39E-08	-1.43E-08	-1.48E-08	-1.36E-08	-1.38E-08	-1.32E-08
-1.61E-08	-1.51E-08	-1.56E-08	-1.58E-08	-1.63E-08	-1.63E-08	-1.63E-08	-1.60E-08	-1.55E-08	-1.46E-08	-1.47E-08	-1.50E-08	-1.40E-08	-1.39E-08	-1.31E-08
-1.68E-08	-1.46E-08	-1.60E-08	-1.60E-08	-1.64E-08	-1.65E-08	-1.65E-08	-1.62E-08	-1.56E-08	-1.49E-08	-1.48E-08	-1.52E-08	-1.41E-08	-1.38E-08	-1.27E-08
-1.72E-08	-1.50E-08	-1.62E-08	-1.63E-08	-1.67E-08	-1.69E-08	-1.65E-08	-1.65E-08	-1.59E-08	-1.59E-08	-1.49E-08	-1.51E-08	-1.41E-08	-1.36E-08	-1.27E-08
-1.73E-08	-1.64E-08	-1.61E-08	-1.68E-08	-1.74E-08	-1.74E-08	-1.61E-08	-1.61E-08	-1.57E-08	-1.58E-08	-1.45E-08	-1.44E-08	-1.30E-08	-1.27E-08	-1.24E-08
-1.76E-08	-1.74E-08	-1.67E-08	-1.70E-08	-1.76E-08	-1.74E-08	-1.54E-08	-1.52E-08	-1.49E-08	-1.52E-08	-1.39E-08	-1.39E-08	-1.27E-08	-1.20E-08	-1.19E-08
-1.84E-08	-1.79E-08	-1.65E-08	-1.70E-08	-1.75E-08	-1.73E-08	-1.56E-08	-1.48E-08	-1.51E-08	-1.51E-08	-1.33E-08	-1.32E-08	-1.29E-08	-1.19E-08	-1.12E-08
-1.83E-08	-1.81E-08	-1.68E-08	-1.70E-08	-1.74E-08	-1.75E-08	-1.64E-08	-1.56E-08	-1.52E-08	-1.54E-08	-1.36E-08	-1.33E-08	-1.29E-08	-1.24E-08	-1.10E-08
-1.82E-08	-1.81E-08	-1.71E-08	-1.73E-08	-1.74E-08	-1.74E-08	-1.64E-08	-1.60E-08	-1.53E-08	-1.56E-08	-1.43E-08	-1.39E-08	-1.29E-08	-1.26E-08	-1.07E-08
-1.82E-08	-1.82E-08	-1.76E-08	-1.79E-08	-1.71E-08	-1.71E-08	-1.61E-08	-1.63E-08	-1.52E-08	-1.57E-08	-1.52E-08	-1.45E-08	-1.35E-08	-1.34E-08	-1.10E-08
-1.85E-08	-1.83E-08	-1.82E-08	-1.79E-08	-1.73E-08	-1.70E-08	-1.64E-08	-1.63E-08	-1.53E-08	-1.56E-08	-1.54E-08	-1.46E-08	-1.38E-08	-1.41E-08	-1.19E-08
-1.86E-08	-1.93E-08	-1.87E-08	-1.77E-08	-1.80E-08	-1.76E-08	-1.72E-08	-1.68E-08	-1.55E-08	-1.57E-08	-1.56E-08	-1.45E-08	-1.42E-08	-1.40E-08	-1.24E-08
-1.84E-08	-1.96E-08	-1.90E-08	-1.79E-08	-1.85E-08	-1.85E-08	-1.77E-08	-1.74E-08	-1.60E-08	-1.64E-08	-1.61E-08	-1.54E-08	-1.53E-08	-1.42E-08	-1.27E-08
-1.89E-08	-1.97E-08	-1.99E-08	-1.84E-08	-1.94E-08	-1.93E-08	-1.81E-08	-1.77E-08	-1.70E-08	-1.71E-08	-1.67E-08	-1.66E-08	-1.61E-08	-1.47E-08	-1.32E-08
-1.95E-08	-1.98E-08	-2.03E-08	-1.90E-08	-1.98E-08	-1.97E-08	-1.85E-08	-1.83E-08	-1.86E-08	-1.79E-08	-1.74E-08	-1.74E-08	-1.72E-08	-1.57E-08	-1.45E-08
-2.04E-08	-2.00E-08	-2.05E-08	-1.96E-08	-2.05E-08	-2.06E-08	-1.97E-08	-1.93E-08	-1.94E-08	-1.86E-08	-1.79E-08	-1.74E-08	-1.79E-08	-1.61E-08	-1.53E-08
-2.10E-08	-2.06E-08	-2.07E-08	-2.03E-08	-2.13E-08	-2.10E-08	-2.04E-08	-1.99E-08	-1.99E-08	-1.90E-08	-1.83E-08	-1.80E-08	-1.83E-08	-1.63E-08	-1.59E-08
-2.09E-08	-2.16E-08	-2.09E-08	-2.10E-08	-2.16E-08	-2.11E-08	-2.10E-08	-2.04E-08	-2.03E-08	-1.93E-08	-1.91E-08	-1.87E-08	-1.85E-08	-1.71E-08	-1.63E-08
-2.12E-08	-2.22E-08	-2.16E-08	-2.10E-08	-2.16E-08	-2.11E-08	-2.13E-08	-2.07E-08	-2.10E-08	-1.95E-08	-1.91E-08	-1.93E-08	-1.92E-08	-1.80E-08	-1.69E-08
-2.17E-08	-2.21E-08	-2.19E-08	-2.12E-08	-2.16E-08	-2.16E-08	-2.17E-08	-2.14E-08	-2.12E-08	-1.98E-08	-1.92E-08	-2.00E-08	-1.96E-08	-1.83E-08	-1.79E-08
-2.16E-08	-2.22E-08	-2.27E-08	-2.19E-08	-2.22E-08	-2.18E-08	-2.19E-08	-2.18E-08	-2.14E-08	-1.98E-08	-1.93E-08	-2.05E-08	-2.01E-08	-1.91E-08	-1.92E-08
-2.17E-08	-2.24E-08	-2.30E-08	-2.30E-08	-2.34E-08	-2.26E-08	-2.23E-08	-2.21E-08	-2.16E-08	-2.00E-08	-1.97E-08	-2.11E-08	-2.05E-08	-1.95E-08	-2.00E-08
-2.20E-08	-2.22E-08	-2.27E-08	-2.41E-08	-2.47E-08	-2.37E-08	-2.30E-08	-2.28E-08	-2.22E-08	-2.09E-08	-2.08E-08	-2.18E-08	-2.10E-08	-2.01E-08	-2.06E-08
-2.24E-08	-2.18E-08	-2.31E-08	-2.54E-08	-2.67E-08	-2.47E-08	-2.38E-08	-2.31E-08	-2.27E-08	-2.14E-08	-2.19E-08	-2.22E-08	-2.18E-08	-2.11E-08	-2.10E-08
-2.25E-08	-2.24E-08	-2.38E-08	-2.70E-08	-2.84E-08	-2.57E-08	-2.40E-08	-2.34E-08	-2.29E-08	-2.16E-08	-2.23E-08	-2.26E-08	-2.22E-08	-2.20E-08	-2.11E-08
-2.26E-08	-2.28E-08	-2.43E-08	-2.73E-08	-2.78E-08	-2.60E-08	-2.39E-08	-2.39E-08	-2.36E-08	-2.21E-08	-2.27E-08	-2.26E-08	-2.22E-08	-2.19E-08	-2.15E-08
-2.39E-08	-2.38E-08	-2.50E-08	-2.62E-08	-2.55E-08	-2.55E-08	-2.38E-08	-2.38E-08	-2.40E-08	-2.26E-08	-2.31E-08	-2.31E-08	-2.26E-08	-2.25E-08	-2.17E-08
-2.51E-08	-2.50E-08	-2.56E-08	-2.58E-08	-2.48E-08	-2.55E-08	-2.38E-08	-2.38E-08	-2.48E-08	-2.37E-08	-2.38E-08	-2.30E-08	-2.31E-08	-2.29E-08	-2.19E-08
-2.60E-08	-2.55E-08	-2.58E-08	-2.61E-08	-2.55E-08	-2.57E-08	-2.47E-08	-2.45E-08	-2.51E-08	-2.42E-08	-2.44E-08	-2.33E-08	-2.33E-08	-2.29E-08	-2.20E-08
-2.65E-08	-2.59E-08	-2.60E-08	-2.64E-08	-2.59E-08	-2.58E-08	-2.56E-08	-2.50E-08	-2.54E-08	-2.45E-08	-2.41E-08	-2.33E-08	-2.32E-08	-2.31E-08	-2.16E-08
-2.62E-08	-2.58E-08	-2.57E-08	-2.67E-08	-2.60E-08	-2.59E-08	-2.57E-08	-2.55E-08	-2.57E-08	-2.47E-08	-2.36E-08	-2.30E-08	-2.29E-08	-2.33E-08	-2.17E-08
-2.63E-08	-2.54E-08	-2.51E-08	-2.64E-08	-2.57E-08	-2.60E-08	-2.58E-08	-2.61E-08	-2.55E-08	-2.45E-08	-2.35E-08	-2.31E-08	-2.31E-08	-2.35E-08	-2.21E-08
-2.62E-08	-2.52E-08	-2.46E-08	-2.58E-08	-2.55E-08	-2.63E-08	-2.60E-08	-2.59E-08	-2.50E-08	-2.42E-08	-2.44E-08	-2.39E-08	-2.40E-08	-2.35E-08	-2.24E-08
-2.56E-08	-2.49E-08	-2.46E-08	-2.55E-08	-2.55E-08	-2.63E-08	-2.59E-08	-2.57E-08	-2.45E-08	-2.42E-08	-2.46E-08	-2.48E-08	-2.48E-08	-2.40E-08	-2.31E-08
-2.47E-08	-2.40E-08	-2.44E-08	-2.52E-08	-2.54E-08	-2.61E-08	-2.57E-08	-2.54E-08	-2.46E-08	-2.43E-08	-2.46E-08	-2.49E-08	-2.52E-08	-2.45E-08	-2.37E-08
-2.46E-08	-2.33E-08	-2.40E-08	-2.51E-08	-2.52E-08	-2.56E-08	-2.55E-08	-2.49E-08	-2.45E-08	-2.45E-08	-2.44E-08	-2.47E-08	-2.49E-08	-2.45E-08	-2.39E-08
-2.50E-08	-2.38E-08	-2.41E-08	-2.50E-08	-2.51E-08	-2.52E-08	-2.49E-08	-2.49E-08	-2.48E-08	-2.51E-08	-2.45E-08	-2.50E-08	-2.45E-08	-2.39E-08	-2.35E-08
-2.51E-08	-2.42E-08	-2.42E-08	-2.49E-08	-2.49E-08	-2.44E-08	-2.45E-08	-2.44E-08	-2.47E-08	-2.52E-08	-2.43E-08	-2.45E-08	-2.40E-08	-2.37E-08	-2.34E-08
-2.49E-08	-2.45E-08	-2.46E-08	-2.48E-08	-2.47E-08	-2.41E-08	-2.40E-08	-2.36E-08	-2.43E-08	-2.47E-08	-2.41E-08	-2.39E-08	-2.37E-08	-2.39E-08	-2.32E-08
-2.51E-08	-2.44E-08	-2.50E-08	-2.47E-08	-2.45E-08	-2.42E-08	-2.41E-08	-2.34E-08	-2.42E-08	-2.43E-08	-2.38E-08	-2.36E-08	-2.35E-08	-2.38E-08	-2.33E-08
-2.50E-08	-2.45E-08	-2.52E-08	-2.49E-08	-2.46E-08	-2.43E-08	-2.41E-08	-2.36E-08	-2.45E-08	-2.41E-08	-2.31E-08	-2.30E-08	-2.28E-08	-2.32E-08	-2.34E-08
-2.45E-08	-2.42E-08	-2.50E-08	-2.47E-08	-2.46E-08	-2.42E-08	-2.38E-08	-2.37E-08	-2.42E-08	-2.39E-08	-2.29E-08	-2.23E-08	-2.23E-08	-2.24E-08	-2.34E-08
-2.47E-08	-2.41E-08	-2.46E-08	-2.43E-08	-2.44E-08	-2.41E-08	-2.35E-08	-2.33E-08	-2.37E-08	-2.37E-08	-2.25E-08	-2.21E-08	-2.23E-08	-2.24E-08	-2.33E-08
-2.46E-08	-2.41E-08	-2.44E-08	-2.38E-08	-2.37E-08	-2.36E-08	-2.30E-08	-2.30E-08	-2.35E-08	-2.35E-08	-2.26E-08	-2.25E-08	-2.25E-08	-2.29E-08	-2.35E-08
-2.40E-08	-2.37E-08	-2.40E-08	-2.35E-08	-2.33E-08	-2.32E-08	-2.25E-08	-2.29E-08	-2.34E-08	-2.33E-08	-2.24E-08	-2.22E-08	-2.25E-08	-2.31E-08	-2.36E-08
-2.40E-08	-2.33E-08	-2.36E-08	-2.29E-08	-2.32E-08	-2.36E-08	-2.28E-08	-2.31E-08	-2.32E-08	-2.27E-08	-2.19E-08	-2.20E-08	-2.25E-08	-2.31E-08	-2.32E-08

2.22E-15	2.19E-15	2.24E-15	2.25E-15	2.39E-15	2.43E-15	2.38E-15	2.42E-15	2.29E-15	2.15E-15	2.01E-15	1.98E-15	1.99E-15	1.99E-15	1.94E-15
2.10E-15	2.07E-15	2.14E-15	2.15E-15	2.34E-15	2.38E-15	2.34E-15	2.43E-15	2.20E-15	2.03E-15	1.93E-15	1.91E-15	1.90E-15	1.89E-15	1.86E-15
1.98E-15	1.97E-15	1.98E-15	2.06E-15	2.27E-15	2.31E-15	2.25E-15	2.34E-15	2.13E-15	1.89E-15	1.85E-15	1.82E-15	1.82E-15	1.82E-15	1.81E-15
1.85E-15	1.85E-15	1.88E-15	1.96E-15	2.17E-15	2.12E-15	2.04E-15	2.13E-15	1.95E-15	1.81E-15	1.72E-15	1.75E-15	1.76E-15	1.73E-15	1.73E-15
1.76E-15	1.78E-15	1.85E-15	1.82E-15	1.96E-15	1.86E-15	1.85E-15	1.92E-15	1.82E-15	1.71E-15	1.63E-15	1.65E-15	1.69E-15	1.63E-15	1.66E-15
1.70E-15	1.67E-15	1.75E-15	1.72E-15	1.78E-15	1.71E-15	1.73E-15	1.75E-15	1.74E-15	1.61E-15	1.56E-15	1.59E-15	1.61E-15	1.55E-15	1.63E-15
1.63E-15	1.61E-15	1.64E-15	1.63E-15	1.65E-15	1.64E-15	1.64E-15	1.69E-15	1.67E-15	1.55E-15	1.53E-15	1.53E-15	1.52E-15	1.46E-15	1.55E-15
1.61E-15	1.61E-15	1.57E-15	1.57E-15	1.56E-15	1.59E-15	1.59E-15	1.63E-15	1.57E-15	1.48E-15	1.45E-15	1.45E-15	1.42E-15	1.41E-15	1.32E-15
1.63E-15	1.60E-15	1.55E-15	1.53E-15	1.50E-15	1.50E-15	1.52E-15	1.53E-15	1.49E-15	1.42E-15	1.38E-15	1.40E-15	1.36E-15	1.34E-15	1.24E-15
1.55E-15	1.55E-15	1.52E-15	1.48E-15	1.41E-15	1.43E-15	1.45E-15	1.41E-15	1.46E-15	1.35E-15	1.32E-15	1.40E-15	1.34E-15	1.29E-15	1.19E-15
1.41E-15	1.41E-15	1.40E-15	1.40E-15	1.33E-15	1.40E-15	1.38E-15	1.35E-15	1.39E-15	1.23E-15	1.27E-15	1.41E-15	1.33E-15	1.20E-15	1.13E-15
1.29E-15	1.27E-15	1.23E-15	1.27E-15	1.24E-15	1.41E-15	1.32E-15	1.29E-15	1.24E-15	1.12E-15	1.19E-15	1.43E-15	1.28E-15	1.14E-15	1.11E-15
1.18E-15	1.17E-15	1.14E-15	1.16E-15	1.15E-15	1.33E-15	1.23E-15	1.17E-15	1.09E-15	1.05E-15	1.11E-15	1.33E-15	1.20E-15	1.09E-15	1.06E-15
1.09E-15	1.10E-15	1.05E-15	1.05E-15	1.08E-15	1.20E-15	1.14E-15	1.05E-15	1.03E-15	1.02E-15	1.05E-15	1.22E-15	1.14E-15	1.03E-15	9.81E-16
9.73E-16	9.90E-16	1.03E-15	9.75E-16	1.01E-15	1.07E-15	1.02E-15	9.95E-16	1.04E-15	1.03E-15	9.90E-16	1.08E-15	1.06E-15	1.00E-15	9.23E-16
8.80E-16	8.69E-16	1.09E-15	9.10E-16	9.59E-16	9.19E-16	9.09E-16	9.48E-16	1.01E-15	9.86E-16	9.68E-16	9.98E-16	9.51E-16	9.14E-16	8.64E-16
8.10E-16	7.79E-16	1.16E-15	8.40E-16	8.91E-16	8.45E-16	8.29E-16	8.86E-16	9.21E-16	8.83E-16	9.06E-16	8.94E-16	8.59E-16	8.39E-16	8.18E-16
7.51E-16	7.24E-16	7.70E-16	7.77E-16	8.58E-16	8.14E-16	8.02E-16	7.83E-16	8.20E-16	7.70E-16	8.05E-16	8.19E-16	7.80E-16	7.86E-16	7.34E-16
6.95E-16	6.83E-16	6.76E-16	7.17E-16	7.94E-16	7.69E-16	7.54E-16	7.13E-16	7.37E-16	7.13E-16	7.18E-16	7.41E-16	7.04E-16	6.98E-16	6.44E-16
6.33E-16	6.32E-16	6.12E-16	6.37E-16	6.58E-16	7.11E-16	7.06E-16	6.80E-16	7.15E-16	7.09E-16	6.94E-16	6.52E-16	6.09E-16	6.10E-16	5.50E-16
5.89E-16	5.76E-16	5.53E-16	5.95E-16	5.92E-16	6.57E-16	6.68E-16	6.30E-16	6.63E-16	6.88E-16	6.34E-16	5.74E-16	5.10E-16	5.09E-16	4.69E-16
5.57E-16	5.28E-16	5.02E-16	5.55E-16	5.38E-16	5.83E-16	5.91E-16	5.24E-16	5.77E-16	6.33E-16	5.61E-16	4.82E-16	4.58E-16	4.59E-16	4.17E-16
5.17E-16	4.69E-16	4.69E-16	4.73E-16	4.75E-16	5.00E-16	5.42E-16	4.93E-16	4.96E-16	5.47E-16	4.98E-16	4.38E-16	4.18E-16	4.24E-16	3.88E-16
4.86E-16	4.24E-16	4.10E-16	4.30E-16	4.14E-16	4.29E-16	4.85E-16	5.01E-16	4.69E-16	4.60E-16	4.15E-16	3.94E-16	3.78E-16	3.62E-16	3.52E-16
4.40E-16	3.89E-16	3.67E-16	3.99E-16	3.68E-16	4.00E-16	4.68E-16	4.95E-16	4.65E-16	4.09E-16	3.65E-16	3.53E-16	3.42E-16	3.18E-16	3.22E-16
4.07E-16	3.62E-16	3.42E-16	3.64E-16	3.48E-16	3.79E-16	4.16E-16	4.20E-16	4.00E-16	3.47E-16	3.21E-16	3.09E-16	2.96E-16	2.95E-16	3.14E-16
3.73E-16	3.40E-16	3.17E-16	3.14E-16	3.30E-16	3.65E-16	3.31E-16	3.22E-16	3.22E-16	2.90E-16	2.82E-16	2.86E-16	2.61E-16	2.64E-16	2.83E-16
3.48E-16	3.14E-16	2.73E-16	2.83E-16	3.26E-16	3.73E-16	3.04E-16	2.75E-16	2.52E-16	2.47E-16	2.61E-16	2.62E-16	2.32E-16	2.36E-16	2.37E-16
3.13E-16	3.02E-16	2.52E-16	2.72E-16	3.10E-16	3.81E-16	3.03E-16	2.50E-16	2.18E-16	2.24E-16	2.47E-16	2.39E-16	2.19E-16	2.23E-16	2.13E-16
2.76E-16	2.86E-16	2.46E-16	2.52E-16	2.92E-16	3.27E-16	2.67E-16	2.13E-16	2.08E-16	2.12E-16	2.14E-16	2.04E-16	2.14E-16	2.16E-16	1.98E-16
2.63E-16	2.51E-16	2.26E-16	2.18E-16	2.41E-16	2.27E-16	2.06E-16	1.90E-16	2.01E-16	2.02E-16	1.82E-16	1.90E-16	1.95E-16	1.90E-16	1.83E-16

2.37E-16	2.38E-16	2.19E-16	1.91E-16	1.82E-16	1.60E-16	1.68E-16	1.58E-16	1.77E-16	1.80E-16	1.61E-16	1.61E-16	1.64E-16	1.58E-16	1.50E-16
2.18E-16	2.27E-16	2.09E-16	1.77E-16	1.61E-16	1.39E-16	1.52E-16	1.42E-16	1.47E-16	1.70E-16	1.56E-16	1.41E-16	1.47E-16	1.31E-16	1.18E-16
1.84E-16	1.90E-16	1.67E-16	1.67E-16	1.60E-16	1.30E-16	1.35E-16	1.36E-16	1.31E-16	1.45E-16	1.36E-16	1.24E-16	1.31E-16	1.07E-16	9.77E-17
1.52E-16	1.66E-16	1.44E-16	1.67E-16	1.64E-16	1.40E-16	1.45E-16	1.32E-16	1.18E-16	1.12E-16	1.11E-16	1.03E-16	1.08E-16	8.80E-17	8.31E-17
1.33E-16	1.48E-16	1.43E-16	1.66E-16	1.64E-16	1.58E-16	1.74E-16	1.23E-16	9.64E-17	8.20E-17	8.56E-17	8.08E-17	7.36E-17	6.96E-17	6.25E-17
1.16E-16	1.22E-16	1.29E-16	1.59E-16	1.65E-16	1.65E-16	1.94E-16	1.15E-16	7.55E-17	6.45E-17	6.54E-17	6.06E-17	5.55E-17	5.28E-17	5.26E-17
1.01E-16	1.01E-16	1.19E-16	1.43E-16	1.34E-16	1.29E-16	1.53E-16	9.21E-17	5.98E-17	5.38E-17	5.48E-17	4.96E-17	4.81E-17	4.43E-17	4.69E-17
9.35E-17	9.38E-17	1.00E-16	1.08E-16	9.16E-17	8.21E-17	1.00E-16	6.81E-17	4.92E-17	4.46E-17	4.60E-17	4.05E-17	4.21E-17	3.90E-17	4.69E-17
8.35E-17	8.40E-17	8.44E-17	8.72E-17	7.18E-17	5.29E-17	7.30E-17	5.84E-17	4.57E-17	3.43E-17	3.76E-17	3.22E-17	3.44E-17	2.93E-17	4.42E-17
7.14E-17	6.81E-17	6.68E-17	6.63E-17	6.60E-17	4.60E-17	5.72E-17	5.53E-17	4.86E-17	3.26E-17	3.59E-17	2.89E-17	2.91E-17	2.09E-17	4.08E-17
5.49E-17	5.75E-17	5.60E-17	4.81E-17	5.05E-17	4.00E-17	4.88E-17	4.47E-17	4.18E-17	3.09E-17	3.88E-17	2.86E-17	2.18E-17	1.62E-17	2.93E-17
4.21E-17	4.30E-17	4.50E-17	3.54E-17	3.81E-17	3.04E-17	3.26E-17	3.05E-17	2.91E-17	3.31E-17	4.07E-17	2.71E-17	1.91E-17	1.71E-17	2.12E-17
3.42E-17	2.99E-17	3.36E-17	3.16E-17	2.71E-17	2.41E-17	2.30E-17	2.33E-17	2.12E-17	3.26E-17	3.15E-17	2.11E-17	1.62E-17	1.54E-17	1.13E-17
2.78E-17	2.18E-17	2.37E-17	2.52E-17	2.27E-17	2.25E-17	1.87E-17	1.63E-17	1.95E-17	2.61E-17	2.14E-17	1.42E-17	1.72E-17	1.21E-17	8.00E-18
2.14E-17	1.52E-17	1.33E-17	1.77E-17	1.94E-17	1.74E-17	9.59E-18	1.14E-17	1.70E-17	1.25E-17	9.60E-18	7.06E-18	1.33E-17	8.62E-18	5.38E-18
1.30E-17	1.03E-17	9.05E-18	1.26E-17	1.33E-17	9.51E-18	7.41E-18	1.08E-17	1.50E-17	8.02E-18	5.63E-18	3.54E-18	9.21E-18	7.44E-18	5.53E-18
9.86E-18	6.16E-18	4.62E-18	9.22E-18	5.56E-18	6.20E-18	5.45E-18	8.75E-18	1.01E-17	3.62E-18	3.66E-18	1.05E-18	7.35E-18	7.42E-18	3.39E-18
8.31E-18	4.92E-18	3.01E-18	6.03E-18	2.14E-18	3.52E-18	2.93E-18	5.10E-18	5.08E-18	2.67E-18	1.49E-18	7.39E-20	4.48E-18	4.41E-18	1.25E-18
4.05E-18	3.70E-18	1.82E-18	3.75E-18	1.63E-18	1.99E-18	9.15E-19	1.47E-18	2.13E-18	1.46E-18	3.01E-19	2.12E-20	5.81E-19	1.43E-18	2.71E-19
1.16E-18	2.17E-18	1.82E-18	2.34E-18	4.51E-19	6.23E-19	9.79E-20	1.13E-19	4.92E-19	4.21E-19	8.13E-20	9.54E-22	9.11E-21	4.60E-20	4.94E-20
4.08E-20	4.84E-19	1.14E-18	1.13E-18	1.38E-20	1.59E-22	7.31E-19	1.96E-19	9.38E-21	1.25E-19	8.95E-19	4.79E-21	4.10E-19	6.67E-19	6.13E-19
4.37E-20	3.01E-20	6.29E-19	1.09E-19	5.32E-19	2.85E-19	1.60E-18	2.06E-18	8.37E-19	1.22E-18	1.68E-18	3.31E-20	2.41E-19	1.46E-18	8.96E-19
5.36E-20	1.04E-19	8.49E-21	1.22E-23	1.85E-18	7.34E-19	4.42E-18	2.71E-18	1.77E-18	2.21E-18	8.52E-19	5.96E-20	6.82E-19	1.91E-18	1.24E-18
7.00E-21	3.33E-19	5.91E-19	8.24E-20	4.42E-18	3.91E-18	9.76E-18	4.78E-18	3.37E-18	2.78E-18	5.69E-19	2.00E-20	6.47E-19	7.98E-19	3.31E-18
3.82E-20	1.19E-18	2.83E-18	1.32E-18	7.73E-18	1.01E-17	1.42E-17	8.53E-18	7.59E-18	5.40E-18	2.55E-18	2.30E-18	1.95E-18	3.54E-18	1.04E-17
1.41E-18	4.32E-18	7.50E-18	8.18E-18	1.24E-17	1.49E-17	1.76E-17	1.50E-17	1.26E-17	9.54E-18	1.01E-17	7.93E-18	6.33E-18	1.03E-17	1.50E-17
2.24E-18	7.37E-18	1.36E-17	1.56E-17	1.56E-17	1.66E-17	2.00E-17	1.90E-17	1.74E-17	1.29E-17	1.76E-17	1.20E-17	1.62E-17	1.78E-17	2.18E-17
5.28E-18	8.95E-18	1.59E-17	1.92E-17	1.76E-17	1.58E-17	2.81E-17	1.98E-17	2.05E-17	1.39E-17	2.24E-17	2.38E-17	2.27E-17	2.37E-17	2.89E-17
1.10E-17	8.35E-18	2.02E-17	1.83E-17	1.58E-17	1.72E-17	2.97E-17	2.28E-17	2.74E-17	1.90E-17	2.62E-17	3.36E-17	2.79E-17	2.86E-17	3.37E-17
1.61E-17	1.01E-17	2.42E-17	2.31E-17	1.51E-17	1.56E-17	2.71E-17	2.70E-17	3.19E-17	2.81E-17	3.14E-17	3.60E-17	3.45E-17	2.93E-17	3.81E-17
2.17E-17	1.85E-17	2.65E-17	2.74E-17	2.05E-17	2.10E-17	2.73E-17	3.25E-17	3.37E-17	3.17E-17	3.70E-17	4.10E-17	3.81E-17	2.92E-17	4.01E-17
3.02E-17	2.75E-17	2.70E-17	3.11E-17	2.88E-17	2.65E-17	3.56E-17	3.78E-17	3.75E-17	3.44E-17	3.55E-17	4.53E-17	3.77E-17	3.04E-17	4.47E-17
3.57E-17	3.34E-17	2.90E-17	4.14E-17	4.21E-17	3.40E-17	4.69E-17	4.81E-17	3.91E-17	3.46E-17	3.46E-17	5.06E-17	3.71E-17	3.12E-17	5.00E-17
4.21E-17	3.98E-17	3.63E-17	5.15E-17	4.90E-17	4.25E-17	6.08E-17	7.26E-17	5.02E-17	3.59E-17	5.18E-17	5.28E-17	4.12E-17	3.30E-17	5.48E-17
4.23E-17	4.85E-17	3.73E-17	5.81E-17	5.49E-17	5.43E-17	6.90E-17	8.52E-17	6.71E-17	4.81E-17	5.94E-17	5.28E-17	3.65E-17	3.19E-17	5.78E-17
4.13E-17	5.48E-17	4.97E-17	5.76E-17	5.89E-17	6.23E-17	8.03E-17	9.95E-17	8.36E-17	5.97E-17	6.09E-17	5.26E-17	3.60E-17	3.67E-17	6.14E-17
5.33E-17	6.58E-17	6.95E-17	6.16E-17	5.77E-17	6.95E-17	9.25E-17	1.04E-16	9.21E-17	6.37E-17	6.43E-17	5.51E-17	4.37E-17	5.25E-17	7.45E-17
7.11E-17	7.66E-17	7.76E-17	6.93E-17	6.35E-17	8.32E-17	9.03E-17	1.06E-16	9.99E-17	7.98E-17	7.11E-17	6.79E-17	7.59E-17	7.24E-17	9.46E-17
8.44E-17	8.35E-17	8.65E-17	7.89E-17	7.82E-17	9.48E-17	9.86E-17	1.02E-16	1.06E-16	9.65E-17	9.02E-17	8.57E-17	9.54E-17	8.68E-17	1.07E-16
8.18E-17	8.69E-17	9.09E-17	9.61E-17	8.33E-17	1.00E-16	1.03E-16	9.51E-17	1.04E-16	1.11E-16	1.01E-16	1.10E-16	1.09E-16	1.04E-16	1.27E-16
7.79E-17	8.25E-17	9.62E-17	1.03E-16	9.56E-17	1.06E-16	1.07E-16	9.83E-17	9.33E-17	1.26E-16	1.17E-16	1.24E-16	1.19E-16	1.06E-16	1.29E-16
7.14E-17	7.51E-17	8.29E-17	9.94E-17	9.20E-17	1.02E-16	1.14E-16	1.08E-16	1.03E-16	1.29E-16	1.15E-16	1.29E-16	1.34E-16	1.15E-16	1.24E-16
6.32E-17	6.85E-17	6.68E-17	8.04E-17	8.39E-17	9.55E-17	1.24E-16	1.15E-16	1.30E-16	1.38E-16	9.06E-17	1.39E-16	1.54E-16	1.36E-16	1.29E-16
7.71E-17	8.19E-17	6.86E-17	7.61E-17	7.72E-17	9.58E-17	1.38E-16	1.28E-16	1.45E-16	1.47E-16	7.00E-17	1.52E-16	1.64E-16	1.41E-16	1.36E-16
1.27E-16	1.07E-16	8.26E-17	9.03E-17	9.07E-17	1.23E-16	1.49E-16	1.46E-16	1.55E-16	1.52E-16	1.38E-16	1.60E-16	1.71E-16	1.44E-16	1.23E-16
1.54E-16	1.32E-16	1.03E-16	1.03E-16	1.06E-16	1.40E-16	1.74E-16	1.59E-16	1.63E-16	1.62E-16	1.78E-16	1.70E-16	1.73E-16	1.53E-16	1.22E-16
1.56E-16	1.26E-16	1.32E-16	1.19E-16	1.41E-16	1.73E-16	1.92E-16	1.75E-16	1.76E-16	1.76E-16	1.88E-16	1.73E-16	1.78E-16	1.60E-16	1.36E-16
1.57E-16	1.28E-16	1.45E-16	1.57E-16	1.68E-16	2.09E-16	2.04E-16	1.86E-16	1.94E-16	1.90E-16	1.97E-16	1.74E-16	1.77E-16	1.71E-16	1.52E-16
1.52E-16	1.48E-16	1.70E-16	1.77E-16	1.96E-16	2.40E-16	2.21E-16	1.98E-16	2.06E-16	2.06E-16	2.01E-16	1.85E-16	1.68E-16	1.75E-16	1.63E-16
1.62E-16	1.82E-16	1.89E-16	1.93E-16	2.27E-16	2.53E-16	2.29E-16	2.16E-16	2.22E-16	2.01E-16	1.98E-16	1.91E-16	1.63E-16	1.78E-16	1.80E-16
1.79E-16	2.02E-16	2.08E-16	2.06E-16	2.39E-16	2.70E-16	2.53E-16	2.41E-16	2.38E-16	1.85E-16	1.99E-16	2.04E-16	1.76E-16	1.84E-16	1.85E-16
2.24E-16	2.27E-16	2.18E-16	2.21E-16	2.42E-16	2.66E-16	2.55E-16	2.58E-16	2.42E-16	1.94E-16	2.06E-16	2.18E-16	1.86E-16	1.90E-16	1.75E-16
2.58E-16	2.27E-16	2.42E-16	2.50E-16	2.64E-16	2.67E-16	2.66E-16	2.57E-16	2.42E-16	2.12E-16	2.17E-16	2.24E-16	1.95E-16	1.93E-16	1.71E-16
2.81E-16	2.14E-16	2.57E-16	2.56E-16	2.70E-16	2.73E-16	2.72E-16	2.62E-16	2.44E-16	2.21E-16	2.19E-16	2.31E-16	1.99E-16	1.89E-16	1.62E-16
2.96E-16	2.26E-16	2.61E-16	2.66E-16	2.79E-16	2.87E-16	2.71E-16	2.72E-16	2.51E-16	2.53E-16	2.22E-16	2.27E-16	1.98E-16	1.84E-16	1.61E-16
3.01E-16	2.70E-16	2.61E-16	2.82E-16	3.01E-16	3.02E-16	2.59E-16	2.58E-16	2.46E-16	2.50E-16	2.09E-16	2.07E-16	1.69E-16	1.60E-16	1.55E-16
3.09E-16	3.02E-16	2.78E-16	2.90E-16	3.08E-16	3.01E-16	2.37E-16	2.32E-16	2.23E-16	2.32E-16	1.92E-16	1.93E-16	1.61E-16	1.45E-16	1.42E-16
3.38E-16	3.19E-16	2.74E-16	2.89E-16	3.07E-16	3.00E-16	2.44E-16	2.20E-16	2.27E-16	2.29E-16	1.76E-16	1.75E-16	1.67E-16	1.42E-16	1.27E-16
3.37E-16	3.28E-16	2.81E-16	2.90E-16	3.03E-16	3.05E-16	2.69E-16	2.43E-16	2.32E-16	2.37E-16	1.84E-16	1.77E-16	1.65E-16	1.54E-16	1.22E-16
3.31E-16	3.28E-16	2.94E-16	2.99E-16	3.02E-16	3.04E-16	2.68E-16	2.57E-16	2.33E-16	2.43E-16	2.05E-16	1.92E-16	1.68E-16	1.59E-16	1.15E-16
3.32E-16	3.32E-16	3.11E-16	3.19E-16	2.92E-16	2.91E-16	2.61E-16	2.66E-16	2.31E-16	2.46E-16	2.32E-16	2.11E-16	1.83E-16	1.79E-16	1.21E-16
3.43E-16	3.36E-16	3.32E-16	3.20E-16	2.98E-16	2.88E-16	2.70E-16	2.65E-16	2.34E-16	2.44E-16	2.37E-16	2.13E-16	1.90E-16	1.98E-16	1.41E-16
3.45E-16	3.72E-16	3.50E-16	3.12E-16	3.23E-16	3.10E-16	2.95E-16	2.81E-16	2.39E-16	2.46E-16	2.45E-16	2.10E-16	2.03E-16	1.96E-16	1.53E-16
3.40E-16	3.85E-16	3.61E-16	3.22E-16	3.42E-16	3.42E-16	3.13E-16	3.02E-16	2.55E-16	2.67E-16	2.61E-16	2.37E-16	2.34E-16	2.03E-16	1.61E-16
3.56E-16	3.89E-16	3.95E-16	3.39E-16	3.78E-16	3.71E-16	3.27E-16	3.12E-16	2.89E-16	2.91E-16	2.80E-16	2.76E-16	2.60E-16	2.16E-16	1.74E-16
3.82E-16	3.91E-16	4.11E-16	3.60E-16	3.93E-16	3.90E-16	3.44E-16	3.34E-16	3.46E-16	3.21E-16	3.02E-16	3.03E-16	2.96E-16	2.46E-16	2.09E-16
4.16E-16	4.00E-16	4.22E-16	3.83E-16	4.19E-16	4.22E-16	3.88E-16	3.73E-16	3.77E-16	3.46E-16	3.20E-16	3.04E-16	3.22E-16	2.60E-16	2.35E-16
4.42E-16	4.25E-16	4.27E-16	4.10E-16	4.54E-16	4.40E-16	4.17E-16	3.98E-16	3.94E-16	3.60E-16	3.37E-16	3.23E-16	3.34E-16	2.67E-16	2.52E-16
4.35E-16	4.65E-16	4.38E-16	4.40E-16	4.65E-16	4.46E-16	4.39E-16	4.16E-16	4.14E-16	3.73E-16	3.64E-16	3.48E-16	3.43E-16	2.92E-16	2.66E-16
4.50E-16	4.91E-16	4.66E-16	4.41E-16	4.65E-16	4.44E-16	4.54E-16	4.29E-16	4.40E-16	3.79E-16	3.63E-16	3.73E-16	3.67E-16	3.23E-16	2.85E-16
4.71E-16	4.90E-16	4.79E-16	4.51E-16	4.68E-16	4.66E-16	4.72E-16	4.56E-16	4.51E-16	3.92E-16	3.69E-16	3.99E-16	3.84E-16	3.35E-16	3.21E-16
4.67E-16	4.91E-16	5.14E-16	4.81E-16	4.92E-16	4.74E-16	4.81E-16	4.73E-16	4.60E-16	3.93E-16	3.71E-16	4.21E-16	4.04E-16	3.65E-16	3.70E-16
4.72E-16	5.04E-16	5.27E-16	5.29E-16	5.48E-16	5.12E-16	4.99E-16	4.86E-16	4.68E-16	4.01E-16	3.90E-16	4.45E-16	4.21E-16	3.81E-16	3.99E-16
4.84E-16	4.94E-16	5.16E-16	5.81E-16	6.11E-16	5.63E-16	5.28E-16	5.20E-16	4.92E-16	4.35E-16	4.35E-16	4.77E-16	4.41E-16	4.04E-16	4.24E-16
5.00E-16	4.75E-16	5.34E-16	6.47E-16	7.11E-16	6.09E-16	5.65E-16	5.32E-16	5.13E-16	4.59E-16	4.77E-16	4.95E-16	4.77E-16	4.43E-16	4.40E-16
5.05E-16	5.03E-16	5.64E-16	7.27E-16	8.09E-16	6.59E-16	5.76E-16	5.48E-16	5.25E-16	4.66E-16	4.97E-16	5.11E-16	4.91E-16	4.82E-16	4.46E-16
5.10E-16	5.20E-16	5.91E-16	7.44E-16	7.72E-16	6.76E-16	5.69E-16	5.70E-16	5.56E-16	4.91E-16	5.17E-16	5.10E-16	4.92E-16	4.81E-16	4.62E-16
5.69E-16	5.68E-16	6.27E-16	6.87E-16	6.48E-16	6.52E-16	5.66E-16	5.67E-16	5.78E-16	5.10E-16	5.32E-16	5.36E-16	5.11E-16	5.06E-16	4.69E-16
6.29E-16	6.25E-16	6.53E-16	6.67E-16	6.15E-16	6.49E-16	5.64E-16	5.66E-16	6.13E-16	5.63E-16	5.66E-16	5.31E-16	5.32E-16	5.23E-16	4.81E-16
6.77E-16	6.52E-16	6.66E-16	6.81E-16	6.48E-16	6.63E-16	6.08E-16	5.99E-16	6.29E-16	5.85E-16	5.96E-16	5.45E-16	5.42E-16	5.23E-16	4.83E-16
7.01E-16	6.72E-16	6.74E-16	6.97E-16	6.69E-16	6.68E-16	6.53E-16	6.25E-16	6.43E-16	6.02E-16	5.81E-16	5.43E-16	5.37E-16	5.34E-16	4.68E-16
6.86E-16	6.65E-16	6.61E-16	7.13E-16	6.74E-16	6.71E-16	6.63E-16	6.49E-16	6.59E-16	6.09E-16	5.55E-16	5.28E-16	5.23E-16	5.43E-16	4.69E-16
6.94E-16	6.44E-16	6.29E-16	6.97E-16	6.59E-16	6.76E-16	6.66E-16	6.79E-16	6.53E-16	6.00E-16	5.54E-16	5.33E-16	5.34E-16	5.52E-16	4.89E-16
6.87E-16	6.34E-16	6.05E-16	6.68E-16	6.51E-16	6.92E-16	6.77E-16	6.73E-16	6.23E-16	5.83E-16	5.93E-16	5.72E-16	5.76E-16	5.50E-16	5.03E-16
6.53E-16	6.22E-16	6.05E-16	6.52E-16	6.51E-16	6.93E-16	6.73E-16	6.62E-16	6.00E-16	5.85E-16	6.07E-16	6.13E-16	6.17E-16	5.78E-16	5.35E-16
6.10E-16	5.75E-16	5.94E-16	6.37E-16	6.46E-16	6.79E-16	6.62E-16	6.44E-16	6.05E-16	5.91E-16	6.04E-16	6.20E-16	6.35E-16	5.98E-16	5.63E-16

6.03E-16	5.43E-16	5.75E-16	6.30E-16	6.37E-16	6.56E-16	6.48E-16	6.20E-16	6.02E-16	6.01E-16	5.95E-16	6.11E-16	6.18E-16	6.02E-16	5.73E-16
6.27E-16	5.69E-16	5.83E-16	6.23E-16	6.28E-16	6.33E-16	6.21E-16	6.20E-16	6.13E-16	6.28E-16	6.02E-16	6.23E-16	6.00E-16	5.73E-16	5.51E-16
6.28E-16	5.86E-16	5.86E-16	6.19E-16	6.19E-16	5.96E-16	6.00E-16	5.94E-16	6.09E-16	6.36E-16	5.90E-16	6.01E-16	5.76E-16	5.62E-16	5.45E-16
6.22E-16	5.99E-16	6.07E-16	6.17E-16	6.11E-16	5.81E-16	5.75E-16	5.57E-16	5.89E-16	6.12E-16	5.79E-16	5.70E-16	5.64E-16	5.72E-16	5.38E-16
6.29E-16	5.97E-16	6.25E-16	6.12E-16	6.02E-16	5.88E-16	5.79E-16	5.48E-16	5.85E-16	5.90E-16	5.68E-16	5.56E-16	5.52E-16	5.66E-16	5.41E-16
6.23E-16	5.99E-16	6.35E-16	6.22E-16	6.06E-16	5.91E-16	5.80E-16	5.56E-16	6.01E-16	5.81E-16	5.36E-16	5.27E-16	5.19E-16	5.40E-16	5.49E-16
6.02E-16	5.85E-16	6.25E-16	6.11E-16	6.06E-16	5.85E-16	5.69E-16	5.62E-16	5.88E-16	5.71E-16	5.24E-16	4.98E-16	4.96E-16	5.03E-16	5.47E-16
6.08E-16	5.79E-16	6.07E-16	5.90E-16	5.96E-16	5.79E-16	5.55E-16	5.44E-16	5.62E-16	5.61E-16	5.07E-16	4.89E-16	4.96E-16	5.00E-16	5.44E-16
6.05E-16	5.79E-16	5.97E-16	5.67E-16	5.62E-16	5.56E-16	5.27E-16	5.29E-16	5.54E-16	5.52E-16	5.12E-16	5.05E-16	5.05E-16	5.25E-16	5.50E-16
5.78E-16	5.60E-16	5.78E-16	5.53E-16	5.45E-16	5.40E-16	5.08E-16	5.22E-16	5.47E-16	5.45E-16	5.03E-16	4.93E-16	5.05E-16	5.36E-16	5.55E-16
5.75E-16	5.44E-16	5.57E-16	5.24E-16	5.37E-16	5.55E-16	5.19E-16	5.34E-16	5.39E-16	5.14E-16	4.81E-16	4.83E-16	5.05E-16	5.33E-16	5.39E-16
5.84E-14	5.72E-14	5.81E-14	5.87E-14	6.01E-14	6.07E-14	5.93E-14	5.84E-14	5.72E-14	5.41E-14	5.25E-14	5.31E-14	5.16E-14	4.97E-14	4.81E-14

4.38E-08	4.36E-08	4.32E-08	4.33E-08	4.26E-08	4.31E-08	4.25E-08	4.27E-08	4.18E-08	4.21E-08	4.20E-08	4.24E-08	4.32E-08	4.35E-08	4.48E-08
4.27E-08	4.26E-08	4.29E-08	4.28E-08	4.22E-08	4.20E-08	4.10E-08	4.15E-08	4.12E-08	4.12E-08	4.09E-08	4.12E-08	4.18E-08	4.23E-08	4.37E-08
4.17E-08	4.18E-08	4.21E-08	4.21E-08	4.14E-08	4.11E-08	3.95E-08	4.05E-08	4.00E-08	4.01E-08	3.98E-08	3.91E-08	4.03E-08	4.05E-08	4.18E-08
4.11E-08	4.12E-08	4.10E-08	4.11E-08	4.06E-08	4.02E-08	3.82E-08	3.89E-08	3.84E-08	3.86E-08	3.81E-08	3.78E-08	3.87E-08	3.90E-08	4.00E-08
4.03E-08	3.97E-08	3.92E-08	3.98E-08	3.93E-08	3.89E-08	3.75E-08	3.76E-08	3.69E-08	3.75E-08	3.70E-08	3.67E-08	3.71E-08	3.75E-08	3.86E-08
3.93E-08	3.86E-08	3.78E-08	3.83E-08	3.83E-08	3.76E-08	3.66E-08	3.63E-08	3.57E-08	3.62E-08	3.56E-08	3.57E-08	3.58E-08	3.69E-08	3.73E-08
3.83E-08	3.79E-08	3.67E-08	3.74E-08	3.73E-08	3.68E-08	3.55E-08	3.49E-08	3.44E-08	3.46E-08	3.42E-08	3.47E-08	3.51E-08	3.60E-08	3.60E-08
3.70E-08	3.64E-08	3.54E-08	3.68E-08	3.68E-08	3.58E-08	3.43E-08	3.34E-08	3.30E-08	3.34E-08	3.33E-08	3.37E-08	3.42E-08	3.50E-08	3.52E-08
3.52E-08	3.48E-08	3.45E-08	3.61E-08	3.57E-08	3.40E-08	3.27E-08	3.23E-08	3.20E-08	3.28E-08	3.25E-08	3.35E-08	3.35E-08	3.40E-08	3.46E-08
3.39E-08	3.38E-08	3.39E-08	3.50E-08	3.55E-08	3.26E-08	3.18E-08	3.13E-08	3.10E-08	3.21E-08	3.15E-08	3.27E-08	3.28E-08	3.27E-08	3.31E-08
3.25E-08	3.33E-08	3.32E-08	3.31E-08	3.56E-08	3.16E-08	3.07E-08	3.05E-08	2.99E-08	3.13E-08	3.10E-08	3.12E-08	3.16E-08	3.16E-08	3.15E-08
3.20E-08	3.28E-08	3.27E-08	3.15E-08	3.45E-08	3.05E-08	2.98E-08	2.93E-08	2.91E-08	3.05E-08	3.03E-08	3.00E-08	3.06E-08	3.07E-08	3.01E-08
3.13E-08	3.18E-08	3.03E-08	2.98E-08	2.91E-08	2.91E-08	2.91E-08	2.79E-08	2.83E-08	2.94E-08	2.93E-08	2.89E-08	2.97E-08	2.98E-08	2.93E-08
3.00E-08	2.97E-08	2.87E-08	2.84E-08	2.79E-08	2.77E-08	2.81E-08	2.69E-08	2.69E-08	2.82E-08	2.85E-08	2.81E-08	2.88E-08	2.87E-08	2.81E-08
2.93E-08	2.89E-08	2.76E-08	2.72E-08	2.67E-08	2.64E-08	2.69E-08	2.59E-08	2.58E-08	2.77E-08	2.77E-08	2.68E-08	2.70E-08	2.70E-08	2.70E-08
2.89E-08	2.83E-08	2.67E-08	2.63E-08	2.54E-08	2.55E-08	2.58E-08	2.56E-08	2.50E-08	2.64E-08	2.60E-08	2.51E-08	2.53E-08	2.58E-08	2.55E-08
2.83E-08	2.77E-08	2.60E-08	2.56E-08	2.40E-08	2.47E-08	2.54E-08	2.51E-08	2.44E-08	2.49E-08	2.44E-08	2.39E-08	2.43E-08	2.51E-08	2.47E-08
2.67E-08	2.68E-08	2.53E-08	2.46E-08	2.28E-08	2.34E-08	2.45E-08	2.35E-08	2.33E-08	2.39E-08	2.28E-08	2.27E-08	2.34E-08	2.39E-08	2.40E-08
2.52E-08	2.53E-08	2.45E-08	2.29E-08	2.15E-08	2.19E-08	2.31E-08	2.25E-08	2.21E-08	2.15E-08	2.14E-08	2.18E-08	2.22E-08	2.28E-08	2.31E-08
2.33E-08	2.29E-08	2.29E-08	2.16E-08	2.06E-08	2.06E-08	2.17E-08	2.15E-08	2.13E-08	1.95E-08	2.05E-08	2.08E-08	2.09E-08	2.17E-08	2.19E-08
2.15E-08	2.11E-08	2.08E-08	2.05E-08	2.00E-08	1.96E-08	2.06E-08	2.04E-08	2.00E-08	1.85E-08	1.94E-08	1.96E-08	1.99E-08	2.08E-08	2.08E-08
2.02E-08	2.01E-08	1.99E-08	1.93E-08	1.91E-08	1.84E-08	1.96E-08	1.91E-08	1.88E-08	1.80E-08	1.87E-08	1.88E-08	1.94E-08	1.99E-08	2.02E-08
1.93E-08	1.94E-08	1.93E-08	1.84E-08	1.77E-08	1.72E-08	1.88E-08	1.75E-08	1.79E-08	1.74E-08	1.83E-08	1.85E-08	1.89E-08	1.95E-08	1.98E-08
1.85E-08	1.86E-08	1.89E-08	1.76E-08	1.67E-08	1.63E-08	1.87E-08	1.64E-08	1.71E-08	1.68E-08	1.74E-08	1.83E-08	1.82E-08	1.84E-08	1.90E-08
1.79E-08	1.80E-08	1.79E-08	1.70E-08	1.58E-08	1.58E-08	1.75E-08	1.56E-08	1.62E-08	1.62E-08	1.65E-08	1.81E-08	1.74E-08	1.72E-08	1.75E-08
1.70E-08	1.75E-08	1.66E-08	1.64E-08	1.52E-08	1.49E-08	1.58E-08	1.49E-08	1.53E-08	1.47E-08	1.57E-08	1.68E-08	1.66E-08	1.66E-08	1.65E-08
1.61E-08	1.64E-08	1.55E-08	1.49E-08	1.46E-08	1.39E-08	1.54E-08	1.44E-08	1.44E-08	1.38E-08	1.44E-08	1.53E-08	1.55E-08	1.56E-08	1.54E-08
1.57E-08	1.55E-08	1.42E-08	1.43E-08	1.39E-08	1.31E-08	1.53E-08	1.44E-08	1.43E-08	1.36E-08	1.35E-08	1.43E-08	1.45E-08	1.44E-08	1.49E-08
1.52E-08	1.49E-08	1.37E-08	1.36E-08	1.31E-08	1.23E-08	1.33E-08	1.41E-08	1.40E-08	1.28E-08	1.30E-08	1.38E-08	1.34E-08	1.35E-08	1.39E-08
1.39E-08	1.40E-08	1.33E-08	1.29E-08	1.24E-08	1.17E-08	1.21E-08	1.35E-08	1.26E-08	1.17E-08	1.25E-08	1.31E-08	1.27E-08	1.30E-08	1.24E-08
1.31E-08	1.32E-08	1.28E-08	1.23E-08	1.20E-08	1.10E-08	1.10E-08	1.23E-08	1.14E-08	1.08E-08	1.16E-08	1.26E-08	1.20E-08	1.27E-08	1.16E-08
1.20E-08	1.22E-08	1.15E-08	1.15E-08	1.12E-08	1.03E-08	1.02E-08	1.06E-08	1.02E-08	1.04E-08	1.04E-08	1.15E-08	1.10E-08	1.18E-08	1.11E-08
1.13E-08	1.11E-08	9.99E-09	1.03E-08	9.91E-09	9.17E-09	9.33E-09	9.21E-09	9.14E-09	9.51E-09	9.46E-09	1.03E-08	9.92E-09	1.08E-08	1.05E-08
1.04E-08	9.92E-09	9.23E-09	9.46E-09	8.93E-09	8.14E-09	8.21E-09	8.08E-09	7.66E-09	8.12E-09	8.30E-09	9.35E-09	9.08E-09	9.32E-09	9.60E-09
1.02E-08	9.35E-09	8.55E-09	8.71E-09	8.41E-09	7.72E-09	6.96E-09	7.18E-09	6.64E-09	7.55E-09	7.68E-09	8.93E-09	8.63E-09	8.30E-09	8.25E-09
9.23E-09	8.60E-09	8.27E-09	8.11E-09	7.88E-09	7.25E-09	5.72E-09	6.39E-09	6.49E-09	6.91E-09	7.40E-09	8.29E-09	8.29E-09	7.71E-09	7.74E-09
8.15E-09	8.46E-09	8.35E-09	7.94E-09	7.45E-09	6.71E-09	4.97E-09	6.04E-09	6.34E-09	6.40E-09	6.42E-09	7.24E-09	7.36E-09	7.23E-09	7.67E-09
7.17E-09	8.21E-09	8.33E-09	7.52E-09	7.16E-09	6.26E-09	4.64E-09	5.88E-09	5.73E-09	5.30E-09	4.99E-09	5.73E-09	6.00E-09	5.92E-09	6.40E-09
6.65E-09	7.24E-09	7.61E-09	6.92E-09	7.42E-09	6.40E-09	4.84E-09	6.41E-09	5.27E-09	4.66E-09	4.04E-09	4.29E-09	5.24E-09	4.81E-09	5.39E-09
6.01E-09	6.27E-09	7.03E-09	6.74E-09	8.28E-09	7.21E-09	5.37E-09	6.88E-09	4.85E-09	3.94E-09	3.16E-09	3.51E-09	5.18E-09	4.12E-09	4.68E-09
5.75E-09	5.48E-09	5.61E-09	5.97E-09	8.39E-09	7.65E-09	5.71E-09	7.20E-09	4.42E-09	3.68E-09	2.58E-09	3.09E-09	4.42E-09	3.83E-09	4.09E-09
5.72E-09	5.12E-09	4.10E-09	4.78E-09	6.39E-09	7.18E-09	5.58E-09	6.85E-09	4.10E-09	3.63E-09	2.46E-09	2.38E-09	3.52E-09	3.18E-09	3.89E-09
5.00E-09	4.51E-09	3.16E-09	3.96E-09	4.60E-09	5.99E-09	5.76E-09	6.42E-09	3.71E-09	3.69E-09	2.25E-09	2.12E-09	2.81E-09	2.57E-09	3.21E-09
4.15E-09	3.50E-09	2.81E-09	3.32E-09	3.67E-09	5.18E-09	5.24E-09	5.78E-09	3.48E-09	3.23E-09	1.93E-09	2.12E-09	2.38E-09	2.02E-09	2.64E-09
3.66E-09	3.27E-09	2.95E-09	3.11E-09	2.59E-09	3.90E-09	4.31E-09	4.18E-09	2.60E-09	3.15E-09	2.20E-09	2.13E-09	1.60E-09	1.17E-09	1.42E-09
2.89E-09	2.42E-09	2.30E-09	2.78E-09	1.45E-09	1.62E-09	2.00E-09	1.65E-09	4.38E-10	1.86E-09	2.09E-09	7.39E-10	5.33E-10	1.60E-10	2.72E-10
2.29E-09	1.63E-09	1.48E-09	2.62E-09	9.17E-10	1.86E-10	-3.62E-11	1.56E-11	-1.05E-09	3.00E-10	1.17E-09	-1.39E-11	6.76E-11	-1.69E-10	-4.41E-10
1.16E-09	1.19E-09	5.42E-10	2.19E-09	4.28E-10	-3.70E-10	-7.54E-10	-8.63E-10	-1.96E-09	-5.17E-10	2.57E-10	-4.63E-10	-1.12E-10	1.57E-11	-1.48E-09
5.61E-10	6.63E-10	-2.21E-10	1.65E-09	-1.27E-10	-3.84E-10	-1.26E-09	-1.27E-09	-2.48E-09	-1.37E-09	-8.96E-10	-1.15E-09	-5.77E-10	-4.15E-10	-2.45E-09
4.05E-11	-4.97E-10	-1.11E-09	-3.15E-10	-7.10E-10	1.31E-11	-1.64E-09	-1.58E-09	-3.03E-09	-2.30E-09	-2.41E-09	-2.57E-09	-2.22E-09	-1.88E-09	-3.21E-09
-4.62E-10	-1.47E-09	-1.89E-09	-1.61E-09	-1.81E-09	-2.31E-10	-1.50E-09	-2.38E-09	-3.44E-09	-3.45E-09	-3.86E-09	-3.58E-09	-3.67E-09	-3.16E-09	-3.84E-09
-1.30E-09	-2.10E-09	-2.36E-09	-2.21E-09	-2.82E-09	-1.84E-09	-2.03E-09	-2.94E-09	-3.80E-09	-3.69E-09	-4.32E-09	-4.23E-09	-4.77E-09	-3.96E-09	-4.54E-09
-1.53E-09	-2.11E-09	-2.78E-09	-2.71E-09	-3.29E-09	-2.91E-09	-2.68E-09	-3.71E-09	-3.95E-09	-4.35E-09	-4.66E-09	-4.53E-09	-5.32E-09	-4.56E-09	-4.92E-09
-1.74E-09	-2.24E-09	-3.04E-09	-3.10E-09	-3.67E-09	-3.51E-09	-3.56E-09	-4.59E-09	-4.18E-09	-5.03E-09	-4.94E-09	-5.34E-09	-5.37E-09	-4.88E-09	-5.24E-09
-2.52E-09	-2.74E-09	-3.17E-09	-3.39E-09	-4.27E-09	-4.07E-09	-3.89E-09	-5.55E-09	-4.96E-09	-5.27E-09	-5.04E-09	-6.00E-09	-5.63E-09	-5.44E-09	-5.54E-09
-3.87E-09	-4.25E-09	-3.82E-09	-4.34E-09	-5.61E-09	-5.56E-09	-5.42E-09	-6.24E-09	-5.91E-09	-5.47E-09	-5.22E-09	-6.40E-09	-6.56E-09	-6.12E-09	-6.06E-09
-4.31E-09	-4.72E-09	-3.72E-09	-4.68E-09	-5.71E-09	-5.91E-09	-5.94E-09	-6.29E-09	-5.84E-09	-5.52E-09	-5.32E-09	-6.91E-09	-7.17E-09	-6.74E-09	-6.46E-09
-4.94E-09	-5.60E-09	-3.58E-09	-4.91E-09	-5.77E-09	-6.02E-09	-6.26E-09	-5.88E-09	-5.25E-09	-5.77E-09	-5.77E-09	-7.50E-09	-7.60E-09	-7.47E-09	-6.63E-09
-5.43E-09	-6.27E-09	-4.26E-09	-5.30E-09	-6.51E-09	-6.32E-09	-5.94E-09	-5.59E-09	-5.72E-09	-6.27E-09	-6.61E-09	-7.65E-09	-7.88E-09	-8.32E-09	-7.02E-09
-5.69E-09	-6.41E-09	-5.31E-09	-5.67E-09	-6.47E-09	-7.33E-09	-6.25E-09	-6.16E-09	-6.56E-09	-7.09E-09	-7.24E-09	-7.60E-09	-8.00E-09	-8.88E-09	-7.97E-09
-5.92E-09	-6.85E-09	-5.82E-09	-6.57E-09	-7.13E-09	-7.98E-09	-6.89E-09	-7.39E-09	-7.15E-09	-7.76E-09	-8.60E-09	-7.67E-09	-8.55E-09	-8.97E-09	-8.71E-09
-6.80E-09	-7.23E-09	-6.19E-09	-7.39E-09	-7.92E-09	-7.93E-09	-7.44E-09	-7.91E-09	-8.17E-09	-8.77E-09	-9.29E-09	-8.00E-09	-8.84E-09	-9.01E-09	-9.05E-09
-7.60E-09	-7.83E-09	-7.69E-09	-8.56E-09	-8.48E-09	-7.92E-09	-7.62E-09	-8.23E-09	-9.04E-09	-9.65E-09	-9.70E-09	-8.87E-09	-9.03E-09	-9.43E-09	-9.64E-09
-7.98E-09	-8.40E-09	-8.02E-09	-8.99E-09	-8.88E-09	-8.45E-09	-7.84E-09	-8.68E-09	-9.44E-09	-1.03E-08	-9.85E-09	-9.17E-09	-9.61E-09	-1.03E-08	-1.02E-08
-8.02E-09	-8.26E-09	-7.76E-09	-8.80E-09	-8.84E-09	-8.54E-09	-7.68E-09	-9.48E-09	-9.48E-09	-1.04E-08	-9.60E-09	-9.54E-09	-9.83E-09	-1.06E-08	-1.03E-08
-7.92E-09	-8.29E-09	-8.18E-09	-8.37E-09	-8.77E-09	-9.03E-09	-8.02E-09	-9.50E-09	-9.49E-09	-1.01E-08	-8.97E-09	-9.50E-09	-1.01E-08	-1.06E-08	-1.05E-08
-8.36E-09	-8.77E-09	-8.56E-09	-8.59E-09	-8.82E-09	-9.37E-09	-7.83E-09	-9.87E-09	-9.74E-09	-9.97E-09	-8.48E-09	-9.19E-09	-1.02E-08	-1.04E-08	-1.09E-08
-9.15E-09	-9.15E-09	-8.93E-09	-9.32E-09	-9.02E-09	-9.71E-09	-8.08E-09	-9.77E-09	-9.87E-09	-9.62E-09	-8.51E-09	-9.38E-09	-9.91E-09	-1.01E-08	-1.07E-08
-9.72E-09	-8.72E-09	-9.23E-09	-9.56E-09	-9.34E-09	-9.59E-09	-8.91E-09	-9.40E-09	-9.43E-09	-9.56E-09	-9.18E-09	-1.01E-08	-9.59E-09	-1.01E-08	-1.06E-08
-1.07E-08	-8.38E-09	-9.15E-09	-9.73E-09	-9.82E-09	-9.64E-09	-9.98E-09	-1.01E-08	-9.61E-09	-1.01E-08	-9.56E-09	-1.06E-08	-9.67E-09	-9.99E-09	-1.05E-08
-1.13E-08	-9.39E-09	-9.37E-09	-1.05E-08	-1.01E-08	-9.99E-09	-1.09E-08	-1.10E-08	-9.84E-09	-1.07E-08	-1.01E-08	-1.12E-08	-1.06E-08	-1.06E-08	-1.07E-08
-1.13E-08	-1.08E-08	-9.85E-09	-1.06E-08	-1.04E-08	-1.06E-08	-1.12E-08	-1.12E-08	-1.03E-08	-1.11E-08	-1.03E-08	-1.10E-08	-1.14E-08	-1.10E-08	-1.07E-08
-1.14E-08	-1.14E-08	-1.01E-08	-1.08E-08	-9.65E-09	-1.08E-08	-1.15E-08	-1.15E-08	-1.05E-08	-1.10E-08	-9.46E-09	-9.87E-09	-1.11E-08	-1.06E-08	-1.04E-08
-1.16E-08	-1.15E-08	-1.09E-08	-1.15E-08	-8.23E-09	-1.06E-08	-1.13E-08	-1.12E-08	-1.04E-08	-1.11E-08	-9.01E-09	-9.35E-09	-1.04E-08	-1.00E-08	-9.97E-09
-1.20E-08	-1.14E-08	-1.16E-08	-1.21E-08	-8.14E-09	-1.08E-08	-1.07E-08	-1.09E-08	-1.04E-08	-1.10E-08	-9.06E-09	-8.84E-09	-9.36E-09	-9.36E-09	-9.83E-09
-1.17E-08	-1.13E-08	-1.19E-08	-1.20E-08	-1.21E-08	-1.11E-08	-1.05E-08	-1.10E-08	-1.04E-08	-1.11E-08	-9.79E-09	-9.16E-09	-8.60E-09	-9.50E-09	-9.68E-09
-1.17E-08	-1.13E-08	-1.18E-08	-1.15E-08	-1.13E-08	-1.09E-08	-1.02E-08	-1.17E-08	-1.04E-08	-1.09E-08	-9.53E-09	-9.68E-09	-8.59E-09	-9.70E-09	-9.88E-09
-1.18E-08	-1.13E-08	-1.16E-08	-1.09E-08	-1.02E-08	-1.02E-08	-1.01E-08	-1.21E-08	-1.05E-08	-1.06E-08	-9.90E-09	-9.88E-09	-9.81E-09	-1.02E-08	-1.00E-08

-1.21E-08	-1.10E-08	-1.11E-08	-1.03E-08	-9.29E-09	-9.53E-09	-1.03E-08	-1.16E-08	-1.06E-08	-1.09E-08	-1.06E-08	-1.02E-08	-9.95E-09	-1.05E-08	-1.03E-08
-1.29E-08	-1.12E-08	-1.10E-08	-9.86E-09	-9.00E-09	-9.59E-09	-1.04E-08	-1.10E-08	-1.03E-08	-1.15E-08	-1.10E-08	-9.88E-09	-1.01E-08	-1.05E-08	-1.08E-08
-1.34E-08	-1.15E-08	-1.10E-08	-9.79E-09	-9.44E-09	-9.34E-09	-9.47E-09	-1.03E-08	-9.81E-09	-1.12E-08	-1.10E-08	-1.03E-08	-9.99E-09	-1.04E-08	-1.07E-08
-1.32E-08	-1.14E-08	-1.08E-08	-9.37E-09	-9.60E-09	-8.83E-09	-8.39E-09	-9.65E-09	-9.21E-09	-1.05E-08	-1.08E-08	-1.03E-08	-9.99E-09	-9.78E-09	-1.07E-08
-1.25E-08	-1.12E-08	-1.07E-08	-9.21E-09	-9.78E-09	-8.45E-09	-7.83E-09	-9.12E-09	-8.50E-09	-9.75E-09	-1.02E-08	-9.45E-09	-9.73E-09	-9.51E-09	-1.06E-08
-1.18E-08	-1.10E-08	-1.06E-08	-9.28E-09	-9.56E-09	-8.36E-09	-8.21E-09	-9.03E-09	-8.25E-09	-8.87E-09	-8.97E-09	-8.49E-09	-9.04E-09	-9.60E-09	-1.07E-08
-1.17E-08	-1.12E-08	-1.06E-08	-9.23E-09	-9.35E-09	-8.18E-09	-8.07E-09	-9.22E-09	-8.49E-09	-8.75E-09	-8.42E-09	-8.00E-09	-8.25E-09	-9.88E-09	-1.11E-08
-1.19E-08	-1.11E-08	-1.01E-08	-9.43E-09	-8.70E-09	-7.75E-09	-7.22E-09	-9.27E-09	-7.73E-09	-8.69E-09	-8.49E-09	-8.19E-09	-7.78E-09	-9.20E-09	-1.07E-08
-1.14E-08	-1.09E-08	-1.00E-08	-9.53E-09	-8.20E-09	-7.00E-09	-6.43E-09	-8.60E-09	-7.17E-09	-7.98E-09	-8.21E-09	-7.62E-09	-7.96E-09	-8.74E-09	-9.97E-09
-1.12E-08	-1.08E-08	-1.03E-08	-9.54E-09	-8.13E-09	-6.78E-09	-6.77E-09	-7.90E-09	-6.64E-09	-6.98E-09	-7.18E-09	-6.64E-09	-7.60E-09	-8.05E-09	-9.25E-09
-1.04E-08	-1.05E-08	-1.04E-08	-9.37E-09	-8.68E-09	-7.22E-09	-6.92E-09	-6.93E-09	-5.69E-09	-5.51E-09	-5.72E-09	-6.31E-09	-7.45E-09	-7.80E-09	-8.87E-09
-9.63E-09	-1.08E-08	-1.01E-08	-9.32E-09	-8.61E-09	-7.65E-09	-6.63E-09	-5.69E-09	-3.61E-09	-3.41E-09	-4.67E-09	-5.77E-09	-6.90E-09	-7.74E-09	-9.32E-09
-9.97E-09	-1.10E-08	-9.66E-09	-9.01E-09	-8.66E-09	-7.41E-09	-5.71E-09	-3.65E-09	-1.07E-09	-9.66E-10	-3.09E-09	-5.17E-09	-6.56E-09	-7.56E-09	-9.60E-09
-1.02E-08	-1.11E-08	-9.20E-09	-8.57E-09	-8.70E-09	-6.96E-09	-5.04E-09	-1.04E-09	1.45E-09	1.73E-09	-1.37E-09	-4.46E-09	-6.63E-09	-7.24E-09	-9.59E-09
-1.06E-08	-1.13E-08	-9.54E-09	-8.69E-09	-8.56E-09	-6.21E-09	-3.88E-09	8.20E-10	2.96E-09	3.80E-09	1.02E-10	-4.18E-09	-6.67E-09	-7.30E-09	-9.67E-09
-1.18E-08	-1.21E-08	-1.04E-08	-8.88E-09	-8.37E-09	-5.62E-09	-2.90E-09	1.98E-09	3.13E-09	3.62E-09	3.71E-11	-4.99E-09	-7.12E-09	-8.20E-09	-9.74E-09
-1.21E-08	-1.25E-08	-1.10E-08	-9.50E-09	-8.17E-09	-5.53E-09	-3.21E-09	2.25E-09	2.11E-09	1.92E-09	-1.46E-09	-6.04E-09	-7.53E-09	-8.74E-09	-9.69E-09
-1.27E-08	-1.31E-08	-1.15E-08	-1.02E-08	-8.59E-09	-5.74E-09	-4.86E-09	-9.39E-10	-1.86E-10	-7.97E-10	-3.55E-09	-7.38E-09	-8.17E-09	-8.59E-09	-9.90E-09
-1.39E-08	-1.36E-08	-1.26E-08	-1.15E-08	-9.32E-09	-7.28E-09	-7.22E-09	-4.64E-09	-3.71E-09	-3.45E-09	-5.48E-09	-8.96E-09	-1.02E-08	-9.57E-09	-1.12E-08
-1.48E-08	-1.46E-08	-1.39E-08	-1.22E-08	-1.04E-08	-9.16E-09	-8.83E-09	-7.33E-09	-6.41E-09	-7.07E-09	-7.50E-09	-1.03E-08	-1.20E-08	-1.13E-08	-1.20E-08
-1.52E-08	-1.54E-08	-1.47E-08	-1.32E-08	-1.12E-08	-1.06E-08	-9.86E-09	-9.48E-09	-8.17E-09	-9.33E-09	-9.86E-09	-1.16E-08	-1.29E-08	-1.23E-08	-1.33E-08
-1.54E-08	-1.60E-08	-1.56E-08	-1.50E-08	-1.27E-08	-1.14E-08	-1.09E-08	-1.13E-08	-1.00E-08	-1.21E-08	-1.24E-08	-1.27E-08	-1.34E-08	-1.29E-08	-1.47E-08
-1.58E-08	-1.60E-08	-1.66E-08	-1.62E-08	-1.47E-08	-1.32E-08	-1.29E-08	-1.31E-08	-1.22E-08	-1.41E-08	-1.45E-08	-1.35E-08	-1.39E-08	-1.45E-08	-1.59E-08
-1.69E-08	-1.67E-08	-1.73E-08	-1.74E-08	-1.64E-08	-1.56E-08	-1.53E-08	-1.50E-08	-1.46E-08	-1.52E-08	-1.53E-08	-1.45E-08	-1.49E-08	-1.60E-08	-1.63E-08
-1.87E-08	-1.77E-08	-1.78E-08	-1.83E-08	-1.80E-08	-1.79E-08	-1.73E-08	-1.67E-08	-1.61E-08	-1.61E-08	-1.59E-08	-1.60E-08	-1.58E-08	-1.64E-08	-1.63E-08
-1.97E-08	-1.87E-08	-1.85E-08	-1.89E-08	-1.87E-08	-1.86E-08	-1.84E-08	-1.75E-08	-1.64E-08	-1.69E-08	-1.74E-08	-1.72E-08	-1.67E-08	-1.70E-08	-1.64E-08
-2.05E-08	-1.97E-08	-1.94E-08	-1.97E-08	-1.93E-08	-1.91E-08	-1.92E-08	-1.84E-08	-1.72E-08	-1.78E-08	-1.85E-08	-1.84E-08	-1.81E-08	-1.76E-08	-1.70E-08
-2.12E-08	-2.06E-08	-2.02E-08	-2.00E-08	-2.01E-08	-2.00E-08	-2.02E-08	-1.93E-08	-1.82E-08	-1.90E-08	-1.89E-08	-1.93E-08	-1.94E-08	-1.84E-08	-1.80E-08
-2.17E-08	-2.11E-08	-2.11E-08	-2.07E-08	-2.08E-08	-2.05E-08	-2.08E-08	-2.01E-08	-1.90E-08	-2.01E-08	-1.91E-08	-1.94E-08	-1.99E-08	-1.96E-08	-1.95E-08
-2.22E-08	-2.13E-08	-2.18E-08	-2.18E-08	-2.13E-08	-2.06E-08	-2.13E-08	-2.12E-08	-2.01E-08	-2.07E-08	-1.99E-08	-1.96E-08	-2.01E-08	-1.99E-08	-2.04E-08
-2.22E-08	-2.16E-08	-2.21E-08	-2.22E-08	-2.14E-08	-2.06E-08	-2.20E-08	-2.17E-08	-2.10E-08	-2.13E-08	-2.11E-08	-1.96E-08	-2.04E-08	-2.03E-08	-2.07E-08
-2.18E-08	-2.17E-08	-2.26E-08	-2.26E-08	-2.20E-08	-2.12E-08	-2.20E-08	-2.20E-08	-2.10E-08	-2.15E-08	-2.17E-08	-2.03E-08	-2.09E-08	-2.08E-08	-2.08E-08
-2.15E-08	-2.17E-08	-2.21E-08	-2.28E-08	-2.25E-08	-2.14E-08	-2.22E-08	-2.17E-08	-2.05E-08	-2.16E-08	-2.18E-08	-2.04E-08	-2.15E-08	-2.14E-08	-2.10E-08
-2.14E-08	-2.17E-08	-2.18E-08	-2.29E-08	-2.25E-08	-2.16E-08	-2.29E-08	-2.18E-08	-2.08E-08	-2.12E-08	-2.17E-08	-2.06E-08	-2.16E-08	-2.17E-08	-2.13E-08
-2.13E-08	-2.18E-08	-2.13E-08	-2.29E-08	-2.24E-08	-2.19E-08	-2.33E-08	-2.26E-08	-2.10E-08	-2.13E-08	-2.19E-08	-2.04E-08	-2.16E-08	-2.17E-08	-2.12E-08
-2.16E-08	-2.20E-08	-2.08E-08	-2.22E-08	-2.25E-08	-2.25E-08	-2.31E-08	-2.29E-08	-2.15E-08	-2.10E-08	-2.15E-08	-2.05E-08	-2.22E-08	-2.22E-08	-2.13E-08
-2.24E-08	-2.24E-08	-2.15E-08	-2.20E-08	-2.24E-08	-2.25E-08	-2.32E-08	-2.27E-08	-2.22E-08	-2.12E-08	-2.15E-08	-2.14E-08	-2.23E-08	-2.22E-08	-2.12E-08
-2.28E-08	-2.33E-08	-2.25E-08	-2.24E-08	-2.27E-08	-2.27E-08	-2.33E-08	-2.26E-08	-2.26E-08	-2.17E-08	-2.13E-08	-2.17E-08	-2.23E-08	-2.21E-08	-2.12E-08
-2.30E-08	-2.36E-08	-2.26E-08	-2.23E-08	-2.25E-08	-2.29E-08	-2.35E-08	-2.24E-08	-2.27E-08	-2.19E-08	-2.18E-08	-2.16E-08	-2.21E-08	-2.19E-08	-2.15E-08
-2.35E-08	-2.36E-08	-2.26E-08	-2.21E-08	-2.23E-08	-2.30E-08	-2.34E-08	-2.21E-08	-2.28E-08	-2.22E-08	-2.19E-08	-2.18E-08	-2.19E-08	-2.21E-08	-2.15E-08
-2.33E-08	-2.31E-08	-2.23E-08	-2.17E-08	-2.21E-08	-2.25E-08	-2.33E-08	-2.26E-08	-2.29E-08	-2.27E-08	-2.20E-08	-2.23E-08	-2.19E-08	-2.19E-08	-2.10E-08
-2.29E-08	-2.28E-08	-2.22E-08	-2.21E-08	-2.22E-08	-2.16E-08	-2.31E-08	-2.28E-08	-2.27E-08	-2.26E-08	-2.25E-08	-2.28E-08	-2.21E-08	-2.16E-08	-2.10E-08
-2.22E-08	-2.30E-08	-2.19E-08	-2.25E-08	-2.22E-08	-2.14E-08	-2.24E-08	-2.29E-08	-2.24E-08	-2.22E-08	-2.24E-08	-2.26E-08	-2.18E-08	-2.17E-08	-2.10E-08
-2.24E-08	-2.31E-08	-2.21E-08	-2.29E-08	-2.29E-08	-2.18E-08	-2.20E-08	-2.28E-08	-2.21E-08	-2.16E-08	-2.17E-08	-2.19E-08	-2.12E-08	-2.14E-08	-2.06E-08
-2.31E-08	-2.30E-08	-2.24E-08	-2.30E-08	-2.30E-08	-2.18E-08	-2.16E-08	-2.28E-08	-2.18E-08	-2.12E-08	-2.10E-08	-2.05E-08	-2.02E-08	-2.08E-08	-2.02E-08
-2.30E-08	-2.32E-08	-2.25E-08	-2.29E-08	-2.24E-08	-2.17E-08	-2.17E-08	-2.23E-08	-2.17E-08	-2.06E-08	-1.98E-08	-1.95E-08	-1.93E-08	-2.04E-08	-2.02E-08
-2.26E-08	-2.28E-08	-2.24E-08	-2.25E-08	-2.20E-08	-2.14E-08	-2.17E-08	-2.16E-08	-2.12E-08	-1.99E-08	-1.89E-08	-1.84E-08	-1.84E-08	-1.98E-08	-2.02E-08
-2.23E-08	-2.22E-08	-2.17E-08	-2.18E-08	-2.17E-08	-2.10E-08	-2.13E-08	-2.13E-08	-2.08E-08	-1.97E-08	-1.85E-08	-1.80E-08	-1.83E-08	-1.94E-08	-2.02E-08
-2.24E-08	-2.21E-08	-2.12E-08	-2.13E-08	-2.12E-08	-2.10E-08	-2.09E-08	-2.14E-08	-2.05E-08	-1.95E-08	-1.88E-08	-1.85E-08	-1.83E-08	-1.89E-08	-1.99E-08
-2.21E-08	-2.18E-08	-2.13E-08	-2.10E-08	-2.09E-08	-2.06E-08	-2.05E-08	-2.11E-08	-2.05E-08	-1.97E-08	-1.90E-08	-1.94E-08	-1.91E-08	-1.91E-08	-1.94E-08

1.92E-15	1.90E-15	1.87E-15	1.88E-15	1.81E-15	1.86E-15	1.81E-15	1.82E-15	1.74E-15	1.77E-15	1.76E-15	1.80E-15	1.86E-15	1.89E-15	2.00E-15
1.83E-15	1.81E-15	1.84E-15	1.83E-15	1.78E-15	1.77E-15	1.68E-15	1.73E-15	1.70E-15	1.70E-15	1.68E-15	1.70E-15	1.74E-15	1.79E-15	1.91E-15
1.74E-15	1.75E-15	1.78E-15	1.77E-15	1.71E-15	1.69E-15	1.56E-15	1.64E-15	1.60E-15	1.61E-15	1.58E-15	1.53E-15	1.62E-15	1.64E-15	1.74E-15
1.69E-15	1.69E-15	1.68E-15	1.69E-15	1.65E-15	1.62E-15	1.46E-15	1.51E-15	1.48E-15	1.49E-15	1.45E-15	1.43E-15	1.50E-15	1.52E-15	1.60E-15
1.62E-15	1.57E-15	1.54E-15	1.58E-15	1.54E-15	1.51E-15	1.40E-15	1.42E-15	1.36E-15	1.40E-15	1.37E-15	1.35E-15	1.38E-15	1.41E-15	1.49E-15
1.55E-15	1.49E-15	1.43E-15	1.47E-15	1.46E-15	1.42E-15	1.34E-15	1.32E-15	1.27E-15	1.31E-15	1.27E-15	1.28E-15	1.28E-15	1.36E-15	1.39E-15
1.47E-15	1.44E-15	1.34E-15	1.40E-15	1.39E-15	1.35E-15	1.26E-15	1.22E-15	1.18E-15	1.20E-15	1.17E-15	1.20E-15	1.23E-15	1.30E-15	1.30E-15
1.37E-15	1.32E-15	1.25E-15	1.35E-15	1.35E-15	1.28E-15	1.17E-15	1.12E-15	1.09E-15	1.12E-15	1.11E-15	1.14E-15	1.17E-15	1.22E-15	1.24E-15
1.24E-15	1.21E-15	1.19E-15	1.30E-15	1.27E-15	1.15E-15	1.07E-15	1.04E-15	1.03E-15	1.08E-15	1.06E-15	1.12E-15	1.12E-15	1.15E-15	1.19E-15
1.15E-15	1.14E-15	1.15E-15	1.22E-15	1.26E-15	1.06E-15	1.01E-15	9.80E-16	9.62E-16	1.03E-15	9.92E-16	1.07E-15	1.07E-15	1.07E-15	1.10E-15
1.06E-15	1.11E-15	1.10E-15	1.10E-15	1.27E-15	9.97E-16	9.42E-16	9.29E-16	8.94E-16	9.78E-16	9.60E-16	9.71E-16	1.00E-15	9.96E-16	9.90E-16
1.02E-15	1.08E-15	1.07E-15	9.92E-16	1.19E-15	9.28E-16	8.88E-16	8.59E-16	8.47E-16	9.33E-16	9.19E-16	9.00E-16	9.39E-16	9.44E-16	9.07E-16
9.78E-16	1.01E-15	9.17E-16	8.91E-16	8.49E-16	8.48E-16	8.49E-16	7.80E-16	7.99E-16	8.62E-16	8.56E-16	8.36E-16	8.84E-16	8.86E-16	8.59E-16
9.02E-16	8.79E-16	8.23E-16	8.06E-16	7.76E-16	7.65E-16	7.87E-16	7.23E-16	7.26E-16	7.97E-16	8.10E-16	7.92E-16	8.27E-16	8.21E-16	7.88E-16
8.59E-16	8.33E-16	7.61E-16	7.39E-16	7.12E-16	6.95E-16	7.22E-16	6.70E-16	6.65E-16	7.66E-16	7.65E-16	7.20E-16	7.30E-16	7.29E-16	7.27E-16
8.35E-16	7.99E-16	7.14E-16	6.91E-16	6.43E-16	6.50E-16	6.67E-16	6.53E-16	6.23E-16	6.97E-16	6.76E-16	6.30E-16	6.42E-16	6.67E-16	6.52E-16
8.02E-16	7.66E-16	6.76E-16	6.57E-16	5.78E-16	6.09E-16	6.47E-16	6.31E-16	5.95E-16	6.21E-16	5.97E-16	5.69E-16	5.92E-16	6.30E-16	6.12E-16
7.11E-16	7.20E-16	6.42E-16	6.04E-16	5.20E-16	5.46E-16	5.98E-16	5.53E-16	5.45E-16	5.70E-16	5.20E-16	5.17E-16	5.49E-16	5.72E-16	5.74E-16
6.33E-16	6.39E-16	5.99E-16	5.25E-16	4.62E-16	4.81E-16	5.35E-16	5.05E-16	4.90E-16	4.62E-16	4.56E-16	4.75E-16	4.93E-16	5.22E-16	5.32E-16
5.43E-16	5.23E-16	5.26E-16	4.67E-16	4.24E-16	4.23E-16	4.72E-16	4.61E-16	4.53E-16	3.80E-16	4.21E-16	4.32E-16	4.37E-16	4.72E-16	4.79E-16
4.61E-16	4.45E-16	4.34E-16	4.21E-16	4.01E-16	3.83E-16	4.23E-16	4.16E-16	4.01E-16	3.42E-16	3.76E-16	3.82E-16	3.96E-16	4.31E-16	4.33E-16
4.09E-16	4.05E-16	3.95E-16	3.71E-16	3.64E-16	3.40E-16	3.83E-16	3.63E-16	3.54E-16	3.26E-16	3.49E-16	3.54E-16	3.76E-16	3.97E-16	4.10E-16
3.72E-16	3.76E-16	3.71E-16	3.38E-16	3.12E-16	2.95E-16	3.53E-16	3.05E-16	3.21E-16	3.04E-16	3.33E-16	3.44E-16	3.56E-16	3.79E-16	3.91E-16
3.43E-16	3.47E-16	3.56E-16	3.11E-16	2.78E-16	2.65E-16	3.48E-16	2.69E-16	2.91E-16	2.82E-16	3.02E-16	3.34E-16	3.32E-16	3.39E-16	3.60E-16
3.19E-16	3.24E-16	3.19E-16	2.90E-16	2.50E-16	2.50E-16	3.05E-16	2.42E-16	2.61E-16	2.62E-16	2.72E-16	3.29E-16	3.02E-16	2.97E-16	3.06E-16
2.89E-16	3.07E-16	2.74E-16	2.70E-16	2.32E-16	2.21E-16	2.48E-16	2.23E-16	2.33E-16	2.16E-16	2.46E-16	2.83E-16	2.74E-16	2.74E-16	2.72E-16
2.60E-16	2.69E-16	2.39E-16	2.22E-16	2.13E-16	1.92E-16	2.36E-16	2.08E-16	2.08E-16	1.90E-16	2.06E-16	2.35E-16	2.41E-16	2.44E-16	2.38E-16
2.48E-16	2.42E-16	2.01E-16	2.04E-16	1.94E-16	1.73E-16	2.33E-16	2.07E-16	2.05E-16	1.84E-16	1.84E-16	2.06E-16	2.09E-16	2.07E-16	2.21E-16
2.30E-16	2.22E-16	1.87E-16	1.86E-16	1.70E-16	1.53E-16	1.78E-16	1.98E-16	1.96E-16	1.64E-16	1.69E-16	1.89E-16	1.80E-16	1.83E-16	1.93E-16
1.94E-16	1.97E-16	1.78E-16	1.66E-16	1.54E-16	1.36E-16	1.48E-16	1.83E-16	1.59E-16	1.36E-16	1.57E-16	1.71E-16	1.62E-16	1.70E-16	1.54E-16
1.70E-16	1.74E-16	1.64E-16	1.51E-16	1.45E-16	1.20E-16	1.22E-16	1.50E-16	1.29E-16	1.17E-16	1.35E-16	1.60E-16	1.44E-16	1.61E-16	1.34E-16

1.44E-16	1.49E-16	1.33E-16	1.32E-16	1.26E-16	1.06E-16	1.03E-16	1.12E-16	1.04E-16	1.09E-16	1.08E-16	1.33E-16	1.21E-16	1.38E-16	1.24E-16
1.27E-16	1.23E-16	9.98E-17	1.06E-16	9.82E-17	8.41E-17	8.71E-17	8.49E-17	8.35E-17	9.04E-17	8.95E-17	1.07E-16	9.83E-17	1.16E-16	1.09E-16
1.08E-16	9.85E-17	8.51E-17	8.95E-17	7.97E-17	6.62E-17	6.73E-17	6.53E-17	5.87E-17	6.59E-17	6.89E-17	8.73E-17	8.25E-17	8.68E-17	9.22E-17
1.04E-16	8.75E-17	7.30E-17	7.58E-17	7.08E-17	5.96E-17	4.85E-17	5.15E-17	4.41E-17	5.70E-17	5.90E-17	7.97E-17	7.45E-17	6.90E-17	6.81E-17
8.52E-17	7.39E-17	6.83E-17	6.58E-17	6.20E-17	5.26E-17	3.27E-17	4.08E-17	4.21E-17	4.77E-17	5.47E-17	6.87E-17	6.86E-17	5.95E-17	5.99E-17
6.64E-17	7.15E-17	6.97E-17	6.30E-17	5.54E-17	4.50E-17	2.47E-17	3.64E-17	4.02E-17	4.09E-17	4.13E-17	5.24E-17	5.42E-17	5.23E-17	5.88E-17
5.14E-17	6.73E-17	6.94E-17	5.66E-17	5.12E-17	3.92E-17	2.15E-17	3.46E-17	3.28E-17	2.81E-17	2.49E-17	3.28E-17	3.60E-17	3.50E-17	4.09E-17
4.43E-17	5.23E-17	5.80E-17	4.79E-17	5.50E-17	4.09E-17	2.34E-17	4.11E-17	2.77E-17	2.17E-17	1.63E-17	1.84E-17	2.74E-17	2.31E-17	2.90E-17
3.61E-17	3.93E-17	4.95E-17	4.55E-17	6.86E-17	5.20E-17	2.88E-17	4.73E-17	2.35E-17	1.55E-17	9.97E-18	1.23E-17	2.68E-17	1.70E-17	2.19E-17
3.31E-17	3.00E-17	3.15E-17	3.56E-17	7.04E-17	5.85E-17	3.26E-17	5.18E-17	1.96E-17	1.35E-17	6.64E-18	9.53E-18	1.95E-17	1.47E-17	1.67E-17
3.28E-17	2.62E-17	1.68E-17	2.28E-17	4.08E-17	5.15E-17	3.12E-17	4.69E-17	1.68E-17	1.32E-17	6.07E-18	5.66E-18	1.24E-17	1.01E-17	1.51E-17
2.50E-17	2.03E-17	9.96E-18	1.57E-17	2.11E-17	3.59E-17	3.32E-17	4.12E-17	1.38E-17	1.36E-17	5.05E-18	4.52E-18	7.92E-18	6.61E-18	1.03E-17
1.72E-17	1.23E-17	7.90E-18	1.10E-17	1.35E-17	2.68E-17	2.74E-17	3.34E-17	1.21E-17	1.05E-17	3.73E-18	4.50E-18	5.66E-18	4.08E-18	6.99E-18
1.34E-17	1.07E-17	8.72E-18	9.68E-18	6.69E-18	1.52E-17	1.86E-17	1.75E-17	6.78E-18	9.91E-18	4.82E-18	4.54E-18	2.56E-18	1.36E-18	2.02E-18
8.37E-18	5.85E-18	5.31E-18	7.73E-18	2.09E-18	2.64E-18	4.01E-18	2.74E-18	1.92E-19	3.47E-18	4.37E-18	5.46E-19	2.84E-19	2.56E-20	7.42E-20
5.24E-18	2.66E-18	2.18E-18	6.85E-18	8.42E-19	3.45E-20	1.31E-21	2.43E-22	1.10E-18	9.01E-20	1.36E-18	1.93E-22	4.57E-21	2.85E-20	1.94E-19
1.36E-18	1.41E-18	2.94E-19	4.78E-18	1.83E-19	1.37E-19	5.69E-19	7.44E-19	3.84E-18	2.67E-19	6.60E-20	2.15E-19	1.26E-20	2.46E-22	2.19E-18
3.14E-19	4.40E-19	4.89E-20	2.72E-18	1.62E-20	1.48E-19	1.58E-18	1.62E-18	6.14E-18	1.89E-18	8.03E-19	1.32E-18	3.32E-19	1.73E-19	6.00E-18
1.64E-21	2.47E-19	1.23E-18	9.94E-20	5.03E-19	1.71E-22	2.70E-18	2.51E-18	9.18E-18	5.29E-18	5.81E-18	6.63E-18	4.93E-18	3.55E-18	1.03E-17
2.14E-19	2.15E-18	3.57E-18	2.60E-18	3.27E-18	5.32E-20	2.26E-18	5.65E-18	1.19E-17	1.19E-17	1.49E-17	1.28E-17	1.35E-17	9.96E-18	1.47E-17
1.70E-18	4.39E-18	5.56E-18	4.88E-18	7.96E-18	3.38E-18	4.11E-18	8.65E-18	1.44E-17	1.36E-17	1.86E-17	1.79E-17	2.28E-17	1.57E-17	2.06E-17
2.34E-18	4.47E-18	7.73E-18	7.37E-18	1.08E-17	8.46E-18	7.17E-18	1.38E-17	1.56E-17	1.90E-17	2.17E-17	2.06E-17	2.83E-17	2.08E-17	2.42E-17
3.02E-18	5.01E-18	9.24E-18	9.61E-18	1.34E-17	1.23E-17	1.27E-17	2.11E-17	1.75E-17	2.53E-17	2.44E-17	2.85E-17	2.88E-17	2.38E-17	2.75E-17
6.36E-18	7.50E-18	1.00E-17	1.15E-17	1.82E-17	1.65E-17	1.51E-17	3.08E-17	2.46E-17	2.78E-17	2.54E-17	3.60E-17	3.17E-17	2.96E-17	3.07E-17
1.50E-17	1.81E-17	1.46E-17	1.88E-17	3.14E-17	3.09E-17	2.93E-17	3.90E-17	3.49E-17	2.99E-17	2.72E-17	4.10E-17	4.30E-17	3.75E-17	3.67E-17
1.86E-17	2.23E-17	1.38E-17	2.19E-17	3.26E-17	3.49E-17	3.53E-17	3.96E-17	3.41E-17	3.05E-17	2.83E-17	4.77E-17	5.14E-17	4.54E-17	4.18E-17
2.44E-17	3.14E-17	1.28E-17	2.41E-17	3.33E-17	3.62E-17	3.92E-17	3.46E-17	2.75E-17	3.33E-17	3.32E-17	5.63E-17	5.77E-17	5.58E-17	4.40E-17
2.95E-17	3.93E-17	1.81E-17	2.81E-17	4.24E-17	4.00E-17	3.53E-17	3.12E-17	3.27E-17	3.94E-17	4.37E-17	5.85E-17	6.21E-17	6.92E-17	4.93E-17
3.23E-17	4.11E-17	2.82E-17	3.21E-17	4.18E-17	5.37E-17	3.90E-17	3.80E-17	4.31E-17	5.03E-17	5.24E-17	5.77E-17	6.41E-17	7.88E-17	6.36E-17
3.51E-17	4.70E-17	3.39E-17	4.32E-17	5.08E-17	6.36E-17	4.75E-17	5.46E-17	5.12E-17	6.02E-17	7.39E-17	5.89E-17	7.31E-17	8.04E-17	7.58E-17
4.62E-17	5.22E-17	3.83E-17	5.47E-17	6.28E-17	6.28E-17	5.53E-17	6.26E-17	6.67E-17	7.69E-17	8.64E-17	6.40E-17	7.81E-17	8.12E-17	8.20E-17
5.78E-17	6.14E-17	5.91E-17	7.33E-17	7.18E-17	6.27E-17	5.80E-17	6.78E-17	8.17E-17	9.31E-17	9.42E-17	7.87E-17	8.16E-17	8.88E-17	9.30E-17
6.36E-17	7.06E-17	6.43E-17	8.08E-17	7.89E-17	7.14E-17	6.15E-17	7.54E-17	8.92E-17	1.05E-16	9.70E-17	8.41E-17	9.23E-17	1.07E-16	1.03E-16
6.44E-17	6.82E-17	6.02E-17	7.74E-17	7.82E-17	7.30E-17	5.90E-17	8.99E-17	8.99E-17	1.08E-16	9.22E-17	9.10E-17	9.66E-17	1.13E-16	1.07E-16
6.27E-17	6.87E-17	6.69E-17	7.01E-17	7.69E-17	8.15E-17	6.44E-17	9.02E-17	9.00E-17	1.01E-16	8.05E-17	9.02E-17	1.02E-16	1.12E-16	1.10E-16
6.99E-17	7.69E-17	7.32E-17	7.37E-17	7.78E-17	8.77E-17	6.13E-17	9.75E-17	9.49E-17	9.94E-17	7.19E-17	8.45E-17	1.04E-16	1.08E-16	1.19E-16
8.37E-17	8.37E-17	7.98E-17	8.68E-17	8.14E-17	9.42E-17	6.53E-17	9.54E-17	9.75E-17	9.25E-17	7.25E-17	8.79E-17	9.82E-17	1.02E-16	1.15E-16
9.45E-17	7.61E-17	8.52E-17	9.14E-17	8.72E-17	9.20E-17	7.93E-17	8.84E-17	8.90E-17	9.14E-17	8.43E-17	1.01E-16	9.19E-17	1.02E-16	1.13E-16
1.14E-16	7.02E-17	8.37E-17	9.47E-17	9.65E-17	9.28E-17	9.97E-17	1.02E-16	9.24E-17	1.01E-16	9.13E-17	1.13E-16	9.35E-17	9.98E-17	1.11E-16
1.28E-16	8.82E-17	8.78E-17	1.10E-16	1.02E-16	9.97E-17	1.20E-16	1.20E-16	9.68E-17	1.15E-16	1.01E-16	1.26E-16	1.13E-16	1.13E-16	1.15E-16
1.28E-16	1.17E-16	9.71E-17	1.12E-16	1.08E-16	1.13E-16	1.26E-16	1.25E-16	1.07E-16	1.24E-16	1.06E-16	1.20E-16	1.29E-16	1.21E-16	1.15E-16
1.30E-16	1.30E-16	1.01E-16	1.17E-16	9.32E-17	1.16E-16	1.33E-16	1.32E-16	1.10E-16	1.22E-16	8.94E-17	9.75E-17	1.23E-16	1.11E-16	1.08E-16
1.35E-16	1.32E-16	1.19E-16	1.33E-16	6.77E-17	1.12E-16	1.28E-16	1.26E-16	1.08E-16	1.24E-16	8.12E-17	8.75E-17	1.09E-16	1.00E-16	9.93E-17
1.43E-16	1.31E-16	1.34E-16	1.45E-16	6.62E-17	1.16E-16	1.15E-16	1.20E-16	1.09E-16	1.22E-16	8.20E-17	7.81E-17	8.76E-17	8.77E-17	9.67E-17
1.38E-16	1.27E-16	1.41E-16	1.43E-16	1.46E-16	1.24E-16	1.11E-16	1.21E-16	1.09E-16	1.23E-16	9.58E-17	8.38E-17	7.40E-17	9.02E-17	9.37E-17
1.37E-16	1.27E-16	1.40E-16	1.32E-16	1.28E-16	1.19E-16	1.05E-16	1.37E-16	1.07E-16	1.18E-16	9.08E-17	9.37E-17	7.39E-17	9.41E-17	9.76E-17
1.38E-16	1.28E-16	1.35E-16	1.20E-16	1.04E-16	1.05E-16	1.02E-16	1.46E-16	1.10E-16	1.12E-16	9.80E-17	9.76E-17	9.62E-17	1.04E-16	1.00E-16
1.47E-16	1.20E-16	1.23E-16	1.07E-16	8.63E-17	9.08E-17	1.07E-16	1.35E-16	1.12E-16	1.19E-16	1.12E-16	1.04E-16	9.91E-17	1.10E-16	1.06E-16
1.67E-16	1.25E-16	1.22E-16	9.73E-17	8.09E-17	9.19E-17	1.07E-16	1.20E-16	1.06E-16	1.33E-16	1.21E-16	9.75E-17	1.03E-16	1.11E-16	1.17E-16
1.79E-16	1.33E-16	1.21E-16	9.58E-17	8.91E-17	8.72E-17	8.98E-17	1.07E-16	9.61E-17	1.27E-16	1.21E-16	1.07E-16	9.99E-17	1.07E-16	1.15E-16
1.75E-16	1.29E-16	1.17E-16	8.78E-17	9.22E-17	7.79E-17	7.03E-17	9.31E-17	8.49E-17	1.11E-16	1.17E-16	1.05E-16	9.98E-17	9.57E-17	1.15E-16
1.56E-16	1.25E-16	1.14E-16	8.49E-17	9.57E-17	7.14E-17	6.13E-17	8.31E-17	7.23E-17	9.50E-17	1.04E-16	8.93E-17	9.47E-17	9.03E-17	1.11E-16
1.39E-16	1.22E-16	1.12E-16	8.61E-17	9.13E-17	6.98E-17	6.74E-17	8.15E-17	6.80E-17	7.86E-17	8.04E-17	7.20E-17	8.16E-17	9.22E-17	1.16E-16
1.37E-16	1.26E-16	1.11E-16	8.52E-17	8.74E-17	6.69E-17	6.51E-17	8.49E-17	7.21E-17	7.66E-17	7.10E-17	6.41E-17	6.81E-17	9.77E-17	1.23E-16
1.40E-16	1.22E-16	1.03E-16	8.90E-17	7.57E-17	6.01E-17	5.21E-17	8.60E-17	5.97E-17	7.55E-17	7.21E-17	6.71E-17	6.05E-17	8.46E-17	1.15E-16
1.31E-16	1.18E-16	1.01E-16	9.08E-17	6.72E-17	4.91E-17	4.14E-17	7.39E-17	5.15E-17	6.37E-17	6.75E-17	5.81E-17	6.34E-17	7.64E-17	9.95E-17
1.26E-16	1.16E-16	1.05E-16	9.10E-17	6.62E-17	4.60E-17	4.58E-17	6.24E-17	4.40E-17	4.87E-17	5.15E-17	4.41E-17	5.78E-17	6.47E-17	8.56E-17
1.08E-16	1.10E-16	1.08E-16	8.79E-17	7.54E-17	5.21E-17	4.79E-17	4.81E-17	3.23E-17	3.03E-17	3.27E-17	3.98E-17	5.56E-17	6.09E-17	7.87E-17
9.26E-17	1.17E-16	1.02E-16	8.68E-17	7.41E-17	5.85E-17	4.40E-17	3.24E-17	1.30E-17	1.16E-17	2.18E-17	3.33E-17	4.76E-17	5.99E-17	8.68E-17
9.94E-17	1.22E-16	9.33E-17	8.12E-17	7.50E-17	5.49E-17	3.26E-17	1.33E-17	1.15E-18	9.34E-19	9.56E-18	2.68E-17	4.30E-17	5.71E-17	9.22E-17
1.03E-16	1.24E-16	8.46E-17	7.35E-17	7.56E-17	4.85E-17	2.54E-17	1.08E-18	2.11E-18	3.00E-18	1.87E-18	1.99E-17	4.39E-17	5.24E-17	9.19E-17
1.13E-16	1.27E-16	9.10E-17	7.56E-17	7.32E-17	3.85E-17	1.51E-17	6.73E-19	8.79E-18	1.44E-17	1.05E-20	1.75E-17	4.45E-17	5.33E-17	9.35E-17
1.38E-16	1.45E-16	1.07E-16	7.88E-17	7.00E-17	3.16E-17	8.42E-18	3.91E-18	9.77E-18	1.31E-17	1.38E-21	2.49E-17	5.07E-17	6.73E-17	9.49E-17
1.47E-16	1.57E-16	1.21E-16	9.02E-17	6.67E-17	3.05E-17	1.03E-17	5.08E-18	4.46E-18	3.70E-18	2.14E-18	3.64E-17	5.67E-17	7.63E-17	9.40E-17
1.61E-16	1.71E-16	1.33E-16	1.04E-16	7.38E-17	3.30E-17	2.37E-17	8.82E-19	3.47E-20	6.36E-19	1.26E-17	5.45E-17	6.67E-17	7.39E-17	9.79E-17
1.94E-16	1.84E-16	1.59E-16	1.32E-16	8.69E-17	5.30E-17	5.21E-17	2.15E-17	1.38E-17	1.19E-17	3.01E-17	8.04E-17	1.05E-16	9.16E-17	1.25E-16
2.20E-16	2.12E-16	1.94E-16	1.48E-16	1.08E-16	8.40E-17	7.80E-17	5.37E-17	4.11E-17	5.00E-17	5.62E-17	1.06E-16	1.45E-16	1.28E-16	1.44E-16
2.30E-16	2.36E-16	2.15E-16	1.75E-16	1.26E-16	1.12E-16	9.72E-17	8.99E-17	6.67E-17	8.70E-17	9.72E-17	1.35E-16	1.66E-16	1.51E-16	1.77E-16
2.36E-16	2.56E-16	2.45E-16	2.26E-16	1.63E-16	1.29E-16	1.18E-16	1.27E-16	1.00E-16	1.46E-16	1.53E-16	1.62E-16	1.80E-16	1.67E-16	2.16E-16
2.51E-16	2.57E-16	2.75E-16	2.64E-16	2.16E-16	1.74E-16	1.68E-16	1.71E-16	1.48E-16	2.00E-16	2.10E-16	1.81E-16	1.94E-16	2.11E-16	2.53E-16
2.86E-16	2.80E-16	3.00E-16	3.02E-16	2.68E-16	2.43E-16	2.35E-16	2.26E-16	2.13E-16	2.31E-16	2.33E-16	2.10E-16	2.21E-16	2.55E-16	2.67E-16
3.49E-16	3.14E-16	3.18E-16	3.35E-16	3.23E-16	3.19E-16	3.01E-16	2.78E-16	2.60E-16	2.60E-16	2.53E-16	2.57E-16	2.49E-16	2.69E-16	2.66E-16
3.90E-16	3.48E-16	3.41E-16	3.57E-16	3.50E-16	3.47E-16	3.39E-16	3.06E-16	2.69E-16	2.86E-16	3.02E-16	2.97E-16	2.78E-16	2.88E-16	2.69E-16
4.19E-16	3.88E-16	3.76E-16	3.90E-16	3.73E-16	3.65E-16	3.69E-16	3.38E-16	2.95E-16	3.17E-16	3.42E-16	3.38E-16	3.27E-16	3.09E-16	2.89E-16
4.49E-16	4.26E-16	4.09E-16	3.98E-16	4.02E-16	4.01E-16	4.09E-16	3.74E-16	3.30E-16	3.61E-16	3.58E-16	3.71E-16	3.76E-16	3.38E-16	3.25E-16
4.71E-16	4.45E-16	4.45E-16	4.30E-16	4.33E-16	4.21E-16	4.32E-16	4.05E-16	3.60E-16	4.03E-16	3.65E-16	3.78E-16	3.97E-16	3.86E-16	3.82E-16
4.93E-16	4.53E-16	4.75E-16	4.74E-16	4.54E-16	4.24E-16	4.55E-16	4.49E-16	4.04E-16	4.29E-16	3.97E-16	3.85E-16	4.05E-16	3.98E-16	4.16E-16
4.93E-16	4.66E-16	4.88E-16	4.94E-16	4.60E-16	4.26E-16	4.84E-16	4.72E-16	4.41E-16	4.54E-16	4.44E-16	3.83E-16	4.15E-16	4.11E-16	4.28E-16
4.76E-16	4.70E-16	5.10E-16	5.09E-16	4.84E-16	4.51E-16	4.84E-16	4.86E-16	4.42E-16	4.63E-16	4.71E-16	4.12E-16	4.37E-16	4.33E-16	4.32E-16
4.62E-16	4.69E-16	4.88E-16	5.19E-16	5.07E-16	4.59E-16	4.93E-16	4.72E-16	4.21E-16	4.65E-16	4.76E-16	4.18E-16	4.63E-16	4.59E-16	4.43E-16
4.56E-16	4.71E-16	4.75E-16	5.25E-16	5.04E-16	4.67E-16	5.24E-16	4.76E-16	4.34E-16	4.51E-16	4.72E-16	4.26E-16	4.67E-16	4.71E-16	4.55E-16
4.55E-16	4.76E-16	4.52E-16	5.24E-16	5.02E-16	4.78E-16	5.43E-16	5.11E-16	4.43E-16	4.53E-16	4.79E-16	4.17E-16	4.67E-16	4.72E-16	4.49E-16
4.67E-16	4.83E-16	4.31E-16	4.93E-16	5.07E-16	5.07E-16	5.36E-16	5.26E-16	4.61E-16	4.42E-16	4.64E-16	4.20E-16	4.92E-16	4.91E-16	4.54E-16
5.00E-16	5.03E-16	4.64E-16	4.83E-16	5.04E-16	5.08E-16	5.37E-16	5.16E-16	4.93E-16	4.51E-16	4.63E-16	4.56E-16	4.96E-16	4.92E-16	4.50E-16
5.19E-16	5.43E-16	5.05E-16	5.02E-16	5.15E-16	5.14E-16	5.44E-16	5.12E-16	5.10E-16	4.70E-16	4.52E-16	4.72E-16	4.99E-16	4.87E-16	4.49E-16
5.30E-16	5.58E-16	5.13E-16	4.98E-16	5.07E-16	5.26E-16	5.51E-16	5.03E-16	5.16E-16	4.80E-16	4.75E-16	4.68E-16	4.87E-16	4.79E-16	4.64E-16

5.53E-16	5.58E-16	5.10E-16	4.89E-16	4.98E-16	5.27E-16	5.46E-16	4.90E-16	5.19E-16	4.95E-16	4.80E-16	4.74E-16	4.81E-16	4.87E-16	4.62E-16
5.45E-16	5.33E-16	4.99E-16	4.71E-16	4.88E-16	5.06E-16	5.44E-16	5.10E-16	5.26E-16	5.15E-16	4.85E-16	4.95E-16	4.79E-16	4.80E-16	4.41E-16
5.25E-16	5.20E-16	4.92E-16	4.88E-16	4.91E-16	4.65E-16	5.32E-16	5.20E-16	5.13E-16	5.13E-16	5.04E-16	5.20E-16	4.88E-16	4.66E-16	4.41E-16
4.94E-16	5.31E-16	4.81E-16	5.07E-16	4.93E-16	4.58E-16	5.00E-16	5.25E-16	5.03E-16	4.94E-16	5.00E-16	5.13E-16	4.74E-16	4.72E-16	4.41E-16
5.01E-16	5.32E-16	4.89E-16	5.24E-16	5.23E-16	4.74E-16	4.83E-16	5.20E-16	4.89E-16	4.68E-16	4.71E-16	4.81E-16	4.51E-16	4.57E-16	4.24E-16
5.32E-16	5.31E-16	5.00E-16	5.28E-16	5.28E-16	4.76E-16	4.68E-16	5.20E-16	4.75E-16	4.50E-16	4.41E-16	4.22E-16	4.10E-16	4.33E-16	4.09E-16
5.31E-16	5.38E-16	5.06E-16	5.24E-16	5.02E-16	4.73E-16	4.72E-16	4.98E-16	4.70E-16	4.25E-16	3.94E-16	3.82E-16	3.73E-16	4.15E-16	4.06E-16
5.09E-16	5.22E-16	5.02E-16	5.06E-16	4.86E-16	4.56E-16	4.72E-16	4.68E-16	4.51E-16	3.98E-16	3.57E-16	3.40E-16	3.40E-16	3.92E-16	4.09E-16
4.98E-16	4.91E-16	4.71E-16	4.75E-16	4.70E-16	4.43E-16	4.52E-16	4.56E-16	4.32E-16	3.87E-16	3.41E-16	3.24E-16	3.35E-16	3.77E-16	4.09E-16
5.02E-16	4.87E-16	4.50E-16	4.52E-16	4.49E-16	4.42E-16	4.38E-16	4.56E-16	4.21E-16	3.79E-16	3.54E-16	3.42E-16	3.35E-16	3.58E-16	3.96E-16
4.90E-16	4.75E-16	4.54E-16	4.39E-16	4.36E-16	4.26E-16	4.21E-16	4.46E-16	4.20E-16	3.87E-16	3.62E-16	3.77E-16	3.65E-16	3.65E-16	3.77E-16
4.65E-14	4.60E-14	4.39E-14	4.38E-14	4.26E-14	4.05E-14	4.02E-14	3.99E-14	3.80E-14	3.88E-14	3.82E-14	3.86E-14	3.98E-14	4.08E-14	4.17E-14

4.46E-08	4.42E-08	4.41E-08	4.51E-08	4.53E-08	4.47E-08	4.51E-08	4.70E-08	4.63E-08	4.69E-08	4.67E-08	4.63E-08	4.49E-08	4.48E-08	4.53E-08
4.43E-08	4.40E-08	4.37E-08	4.42E-08	4.41E-08	4.32E-08	4.41E-08	4.53E-08	4.46E-08	4.50E-08	4.56E-08	4.57E-08	4.42E-08	4.41E-08	4.40E-08
4.30E-08	4.32E-08	4.22E-08	4.20E-08	4.27E-08	4.21E-08	4.32E-08	4.35E-08	4.33E-08	4.30E-08	4.42E-08	4.44E-08	4.32E-08	4.32E-08	4.28E-08
4.14E-08	4.14E-08	4.00E-08	4.01E-08	4.13E-08	4.09E-08	4.22E-08	4.17E-08	4.18E-08	4.13E-08	4.24E-08	4.33E-08	4.25E-08	4.22E-08	4.18E-08
3.94E-08	3.92E-08	3.85E-08	3.86E-08	3.95E-08	3.97E-08	4.04E-08	4.04E-08	4.01E-08	3.99E-08	4.03E-08	4.16E-08	4.15E-08	4.06E-08	4.12E-08
3.76E-08	3.76E-08	3.71E-08	3.72E-08	3.79E-08	3.83E-08	3.87E-08	3.91E-08	3.86E-08	3.86E-08	3.92E-08	4.00E-08	3.99E-08	3.96E-08	4.04E-08
3.61E-08	3.62E-08	3.56E-08	3.59E-08	3.67E-08	3.69E-08	3.70E-08	3.80E-08	3.80E-08	3.77E-08	3.81E-08	3.83E-08	3.81E-08	3.82E-08	3.92E-08
3.48E-08	3.50E-08	3.46E-08	3.49E-08	3.56E-08	3.52E-08	3.55E-08	3.67E-08	3.69E-08	3.62E-08	3.71E-08	3.75E-08	3.66E-08	3.69E-08	3.75E-08
3.38E-08	3.36E-08	3.39E-08	3.41E-08	3.46E-08	3.41E-08	3.45E-08	3.50E-08	3.55E-08	3.49E-08	3.60E-08	3.60E-08	3.51E-08	3.56E-08	3.61E-08
3.26E-08	3.23E-08	3.29E-08	3.33E-08	3.34E-08	3.31E-08	3.31E-08	3.30E-08	3.38E-08	3.35E-08	3.39E-08	3.34E-08	3.38E-08	3.39E-08	3.49E-08
3.14E-08	3.12E-08	3.12E-08	3.22E-08	3.22E-08	3.19E-08	3.13E-08	3.12E-08	3.22E-08	3.25E-08	3.23E-08	3.17E-08	3.23E-08	3.22E-08	3.35E-08
3.02E-08	3.06E-08	3.01E-08	3.10E-08	3.11E-08	3.09E-08	3.03E-08	3.03E-08	3.07E-08	3.19E-08	3.16E-08	3.14E-08	3.06E-08	3.10E-08	3.16E-08
2.92E-08	2.97E-08	2.93E-08	2.99E-08	2.97E-08	2.97E-08	2.96E-08	2.92E-08	2.98E-08	3.10E-08	3.13E-08	3.08E-08	2.93E-08	2.98E-08	3.02E-08
2.78E-08	2.88E-08	2.84E-08	2.85E-08	2.87E-08	2.88E-08	2.85E-08	2.84E-08	2.96E-08	3.05E-08	3.05E-08	2.99E-08	2.86E-08	2.89E-08	2.92E-08
2.65E-08	2.73E-08	2.75E-08	2.72E-08	2.74E-08	2.75E-08	2.74E-08	2.80E-08	2.92E-08	2.89E-08	2.90E-08	2.87E-08	2.83E-08	2.81E-08	2.80E-08
2.58E-08	2.61E-08	2.64E-08	2.64E-08	2.57E-08	2.63E-08	2.72E-08	2.72E-08	2.85E-08	2.74E-08	2.73E-08	2.76E-08	2.75E-08	2.72E-08	2.67E-08
2.46E-08	2.50E-08	2.48E-08	2.59E-08	2.46E-08	2.54E-08	2.66E-08	2.60E-08	2.68E-08	2.64E-08	2.62E-08	2.65E-08	2.60E-08	2.59E-08	2.53E-08
2.32E-08	2.41E-08	2.34E-08	2.50E-08	2.37E-08	2.44E-08	2.51E-08	2.48E-08	2.48E-08	2.45E-08	2.44E-08	2.44E-08	2.45E-08	2.46E-08	2.40E-08
2.21E-08	2.28E-08	2.26E-08	2.36E-08	2.28E-08	2.36E-08	2.36E-08	2.37E-08	2.33E-08	2.31E-08	2.27E-08	2.28E-08	2.33E-08	2.35E-08	2.31E-08
2.13E-08	2.22E-08	2.20E-08	2.20E-08	2.18E-08	2.26E-08	2.25E-08	2.25E-08	2.24E-08	2.23E-08	2.16E-08	2.18E-08	2.23E-08	2.23E-08	2.22E-08
2.08E-08	2.13E-08	2.12E-08	2.11E-08	2.05E-08	2.18E-08	2.13E-08	2.12E-08	2.15E-08	2.11E-08	2.04E-08	2.07E-08	2.13E-08	2.14E-08	2.15E-08
2.02E-08	2.06E-08	2.01E-08	2.03E-08	1.95E-08	2.08E-08	2.02E-08	1.95E-08	2.02E-08	2.01E-08	1.95E-08	2.00E-08	2.04E-08	2.04E-08	2.13E-08
1.96E-08	1.99E-08	1.89E-08	1.95E-08	1.90E-08	1.96E-08	1.93E-08	1.86E-08	1.89E-08	1.92E-08	1.87E-08	1.94E-08	1.97E-08	1.97E-08	2.05E-08
1.87E-08	1.90E-08	1.84E-08	1.88E-08	1.87E-08	1.86E-08	1.89E-08	1.82E-08	1.84E-08	1.87E-08	1.80E-08	1.87E-08	1.97E-08	1.92E-08	1.96E-08
1.78E-08	1.78E-08	1.77E-08	1.80E-08	1.79E-08	1.77E-08	1.81E-08	1.74E-08	1.75E-08	1.77E-08	1.74E-08	1.79E-08	1.91E-08	1.82E-08	1.89E-08
1.69E-08	1.62E-08	1.65E-08	1.67E-08	1.69E-08	1.66E-08	1.68E-08	1.63E-08	1.61E-08	1.70E-08	1.67E-08	1.73E-08	1.66E-08	1.70E-08	1.77E-08
1.61E-08	1.53E-08	1.57E-08	1.61E-08	1.66E-08	1.57E-08	1.54E-08	1.50E-08	1.50E-08	1.59E-08	1.59E-08	1.57E-08	1.50E-08	1.58E-08	1.70E-08
1.49E-08	1.46E-08	1.53E-08	1.67E-08	1.66E-08	1.49E-08	1.43E-08	1.36E-08	1.44E-08	1.53E-08	1.53E-08	1.46E-08	1.43E-08	1.54E-08	1.57E-08
1.32E-08	1.34E-08	1.54E-08	1.70E-08	1.62E-08	1.39E-08	1.31E-08	1.28E-08	1.35E-08	1.43E-08	1.40E-08	1.43E-08	1.45E-08	1.51E-08	1.53E-08
1.15E-08	1.20E-08	1.45E-08	1.72E-08	1.60E-08	1.31E-08	1.22E-08	1.20E-08	1.26E-08	1.38E-08	1.33E-08	1.43E-08	1.44E-08	1.49E-08	1.43E-08
1.09E-08	1.13E-08	1.36E-08	1.62E-08	1.52E-08	1.21E-08	1.12E-08	1.13E-08	1.17E-08	1.32E-08	1.26E-08	1.39E-08	1.39E-08	1.42E-08	1.30E-08
1.06E-08	1.05E-08	1.17E-08	1.23E-08	1.20E-08	1.12E-08	1.08E-08	1.10E-08	1.08E-08	1.19E-08	1.16E-08	1.24E-08	1.29E-08	1.25E-08	1.18E-08
1.03E-08	9.53E-09	1.12E-08	9.84E-09	1.08E-08	1.06E-08	1.03E-08	1.08E-08	1.01E-08	1.06E-08	1.07E-08	1.06E-08	1.16E-08	1.02E-08	1.04E-08
9.63E-09	8.27E-09	1.00E-08	8.68E-09	9.48E-09	9.63E-09	9.23E-09	9.68E-09	9.09E-09	9.04E-09	9.23E-09	9.50E-09	1.03E-08	9.13E-09	9.22E-09
8.36E-09	7.55E-09	8.92E-09	7.68E-09	8.22E-09	8.38E-09	7.73E-09	8.41E-09	8.56E-09	7.68E-09	8.06E-09	8.73E-09	9.32E-09	8.28E-09	8.36E-09
7.17E-09	7.05E-09	8.01E-09	7.43E-09	7.96E-09	7.32E-09	7.02E-09	7.25E-09	7.73E-09	6.55E-09	7.74E-09	7.86E-09	8.30E-09	7.43E-09	7.64E-09
6.95E-09	6.41E-09	7.12E-09	6.76E-09	7.67E-09	6.71E-09	6.44E-09	6.39E-09	6.84E-09	5.84E-09	7.17E-09	7.09E-09	7.95E-09	6.79E-09	6.46E-09
6.47E-09	5.63E-09	6.34E-09	6.01E-09	7.07E-09	6.41E-09	5.28E-09	5.68E-09	5.79E-09	5.43E-09	6.68E-09	6.40E-09	7.48E-09	6.18E-09	4.85E-09
5.44E-09	5.17E-09	5.31E-09	5.25E-09	5.76E-09	6.09E-09	4.63E-09	5.35E-09	5.11E-09	5.00E-09	6.14E-09	6.24E-09	6.61E-09	5.46E-09	4.19E-09
4.81E-09	4.55E-09	4.49E-09	4.37E-09	4.80E-09	5.43E-09	4.57E-09	4.79E-09	4.62E-09	4.18E-09	5.18E-09	5.51E-09	4.76E-09	5.09E-09	4.22E-09
3.96E-09	3.90E-09	3.68E-09	3.99E-09	4.38E-09	4.68E-09	4.57E-09	4.33E-09	4.17E-09	3.31E-09	4.30E-09	3.87E-09	3.17E-09	4.77E-09	3.95E-09
3.04E-09	3.45E-09	2.77E-09	3.14E-09	3.47E-09	4.22E-09	4.18E-09	4.04E-09	3.58E-09	3.06E-09	3.77E-09	2.87E-09	2.25E-09	3.59E-09	3.06E-09
2.60E-09	3.22E-09	1.83E-09	2.55E-09	3.33E-09	4.12E-09	3.69E-09	3.83E-09	3.11E-09	2.78E-09	3.12E-09	1.99E-09	1.69E-09	2.12E-09	2.22E-09
2.17E-09	2.61E-09	1.09E-09	2.23E-09	2.96E-09	3.47E-09	2.61E-09	2.99E-09	2.40E-09	2.14E-09	2.51E-09	1.12E-09	1.08E-09	1.61E-09	1.29E-09
1.41E-09	1.98E-09	6.97E-10	1.50E-09	2.45E-09	2.78E-09	1.94E-09	2.53E-09	2.01E-09	1.66E-09	1.90E-09	7.55E-10	5.45E-10	1.18E-09	4.95E-10
3.10E-10	1.04E-09	-2.79E-11	6.01E-10	1.09E-09	1.31E-09	1.36E-09	1.42E-09	1.10E-09	7.98E-10	5.86E-10	-4.00E-10	-4.56E-10	-3.16E-11	-5.92E-10
-8.04E-10	2.14E-10	-2.81E-10	9.68E-11	3.61E-10	3.55E-10	8.77E-10	7.14E-10	3.84E-10	-4.09E-10	-5.34E-10	-1.17E-09	-1.32E-09	-6.92E-10	-1.12E-09
-1.71E-09	-6.80E-10	-6.73E-10	-5.30E-10	-3.81E-10	-3.18E-10	3.89E-11	-3.62E-10	-9.31E-10	-1.56E-09	-1.16E-09	-1.77E-09	-2.00E-09	-1.44E-09	-1.88E-09
-2.29E-09	-9.05E-10	-9.80E-10	-1.38E-09	-1.23E-09	-1.05E-09	-1.03E-09	-1.68E-09	-1.81E-09	-2.09E-09	-1.64E-09	-2.28E-09	-2.63E-09	-1.78E-09	-2.25E-09
-2.87E-09	-1.84E-09	-1.55E-09	-2.18E-09	-1.79E-09	-1.44E-09	-1.50E-09	-2.64E-09	-2.13E-09	-2.07E-09	-2.11E-09	-2.28E-09	-2.71E-09	-1.74E-09	-2.17E-09
-3.63E-09	-3.23E-09	-2.12E-09	-2.60E-09	-2.09E-09	-1.89E-09	-2.05E-09	-3.12E-09	-2.29E-09	-2.00E-09	-2.47E-09	-2.51E-09	-2.90E-09	-2.07E-09	-2.43E-09
-4.32E-09	-4.03E-09	-2.81E-09	-3.20E-09	-2.74E-09	-2.68E-09	-2.84E-09	-3.51E-09	-2.23E-09	-2.52E-09	-2.95E-09	-2.47E-09	-2.98E-09	-2.62E-09	-2.82E-09
-4.49E-09	-4.27E-09	-3.32E-09	-3.94E-09	-3.22E-09	-3.44E-09	-3.82E-09	-3.90E-09	-2.79E-09	-2.99E-09	-3.54E-09	-2.51E-09	-3.40E-09	-3.23E-09	-3.14E-09
-4.51E-09	-4.08E-09	-3.93E-09	-4.27E-09	-3.27E-09	-4.05E-09	-3.86E-09	-3.31E-09	-3.03E-09	-3.25E-09	-3.61E-09	-3.08E-09	-3.51E-09	-3.23E-09	-3.04E-09
-4.99E-09	-4.69E-09	-5.16E-09	-4.99E-09	-4.36E-09	-4.78E-09	-4.22E-09	-3.79E-09	-3.08E-09	-3.62E-09	-3.60E-09	-4.26E-09	-3.74E-09	-3.45E-09	-2.87E-09
-5.76E-09	-5.54E-09	-5.30E-09	-5.69E-09	-5.11E-09	-4.96E-09	-4.68E-09	-4.03E-09	-3.25E-09	-3.72E-09	-3.68E-09	-5.14E-09	-4.31E-09	-3.57E-09	-3.76E-09
-6.49E-09	-5.67E-09	-5.63E-09	-6.15E-09	-5.59E-09	-5.17E-09	-4.97E-09	-4.22E-09	-3.95E-09	-4.10E-09	-4.43E-09	-5.35E-09	-4.88E-09	-4.35E-09	-5.55E-09
-6.86E-09	-5.90E-09	-6.33E-09	-6.64E-09	-6.48E-09	-5.36E-09	-4.96E-09	-4.37E-09	-4.85E-09	-4.89E-09	-5.04E-09	-5.11E-09	-5.19E-09	-5.13E-09	-6.69E-09
-7.36E-09	-6.59E-09	-7.13E-09	-7.16E-09	-7.26E-09	-5.66E-09	-5.01E-09	-4.97E-09	-5.35E-09	-5.38E-09	-5.27E-09	-5.14E-09	-5.15E-09	-5.94E-09	-7.58E-09
-7.97E-09	-7.06E-09	-7.91E-09	-7.51E-09	-7.37E-09	-6.16E-09	-5.33E-09	-5.42E-09	-5.91E-09	-5.25E-09	-5.63E-09	-5.38E-09	-5.24E-09	-6.35E-09	-8.11E-09
-8.13E-09	-7.34E-09	-8.34E-09	-7.94E-09	-7.72E-09	-6.75E-09	-5.54E-09	-5.53E-09	-6.58E-09	-5.84E-09	-5.97E-09	-5.96E-09	-5.56E-09	-7.12E-09	-8.06E-09
-8.54E-09	-7.86E-09	-8.61E-09	-8.14E-09	-8.31E-09	-7.43E-09	-6.30E-09	-6.10E-09	-7.19E-09	-6.51E-09	-6.11E-09	-6.91E-09	-6.58E-09	-7.87E-09	-8.39E-09
-9.22E-09	-8.62E-09	-8.85E-09	-8.49E-09	-8.97E-09	-8.17E-09	-7.28E-09	-7.15E-09	-7.37E-09	-7.38E-09	-6.18E-09	-7.65E-09	-7.62E-09	-8.58E-09	-9.05E-09
-9.67E-09	-9.50E-09	-9.11E-09	-8.47E-09	-9.11E-09	-8.79E-09	-8.37E-09	-8.36E-09	-7.47E-09	-7.88E-09	-7.06E-09	-8.93E-09	-9.20E-09	-9.87E-09	-9.72E-09
-9.93E-09	-1.00E-08	-9.43E-09	-8.20E-09	-8.87E-09	-8.85E-09	-8.22E-09	-8.70E-09	-7.69E-09	-8.34E-09	-8.92E-09	-9.95E-09	-1.05E-08	-1.13E-08	-9.96E-09
-1.00E-08	-1.01E-08	-9.47E-09	-8.52E-09	-8.97E-09	-8.99E-09	-7.63E-09	-8.23E-09	-7.47E-09	-8.97E-09	-9.73E-09	-1.05E-08	-1.14E-08	-1.19E-08	-1.05E-08
-1.01E-08	-9.93E-09	-9.71E-09	-9.60E-09	-9.83E-09	-9.41E-09	-7.53E-09	-7.56E-09	-7.63E-09	-9.28E-09	-9.90E-09	-1.11E-08	-1.14E-08	-1.21E-08	-1.18E-08
-1.02E-08	-9.96E-09	-1.04E-08	-1.05E-08	-1.04E-08	-9.97E-09	-7.64E-09	-7.18E-09	-7.89E-09	-9.29E-09	-1.05E-08	-1.17E-08	-1.18E-08	-1.21E-08	-1.29E-08
-1.03E-08	-1.03E-08	-1.07E-08	-1.13E-08	-1.09E-08	-1.04E-08	-8.11E-09	-6.95E-09	-7.55E-09	-8.94E-09	-1.06E-08	-1.22E-08	-1.23E-08	-1.20E-08	-1.34E-08
-1.03E-08	-1.04E-08	-1.04E-08	-1.11E-08	-1.08E-08	-1.02E-08	-8.60E-09	-8.23E-09	-8.21E-09	-8.94E-09	-1.07E-08	-1.23E-08	-1.23E-08	-1.23E-08	-1.33E-08
-1.02E-08	-1.04E-08	-1.02E-08	-1.10E-08	-1.08E-08	-9.70E-09	-9.17E-09	-9.53E-09	-8.54E-09	-9.42E-09	-1.13E-08	-1.25E-08	-1.22E-08	-1.27E-08	-1.35E-08
-9.74E-09	-1.03E-08	-9.89E-09	-1.05E-08	-1.07E-08	-9.98E-09	-1.07E-08	-1.06E-08	-9.16E-09	-9.86E-09	-1.17E-08	-1.30E-08	-1.21E-08	-1.27E-08	-1.30E-08
-8.71E-09	-9.80E-09	-9.58E-09	-1.01E-08	-1.03E-08	-1.05E-08	-1.18E-08	-1.19E-08	-1.06E-08	-1.11E-08	-1.23E-08	-1.28E-08	-1.23E-08	-1.29E-08	-1.31E-08
-7.45E-09	-9.26E-09	-9.80E-09	-9.23E-09	-1.01E-08	-1.12E-08	-1.22E-08	-1.22E-08	-1.17E-08	-1.19E-08	-1.22E-08	-1.23E-08	-1.23E-08	-1.37E-08	-1.36E-08
-7.37E-09	-9.43E-09	-9.67E-09	-8.31E-09	-1.02E-08	-1.14E-08	-1.20E-08	-1.19E-08	-1.21E-08	-1.22E-08	-1.19E-08	-1.24E-08	-1.30E-08	-1.45E-08	-1.41E-08
-8.18E-09	-1.00E-08	-9.49E-09	-8.49E-09	-1.08E-08	-1.16E-08	-1.19E-08	-1.17E-08	-1.22E-08	-1.23E-08	-1.20E-08	-1.24E-08	-1.39E-08	-1.50E-08	-1.47E-08
-8.94E-09	-1.03E-08	-9.06E-09	-1.10E-08	-1.10E-08	-1.18E-08	-1.22E-08	-1.21E-08	-1.24E-08	-1.31E-08	-1.26E-08	-1.23E-08	-1.42E-08	-1.52E-08	-1.48E-08
-9.29E-09	-1.02E-08	-8.95E-09	-1.12E-08	-1.12E-08	-1.18E-08	-1.19E-08	-1.27E-08	-1.31E-08	-1.38E-08	-1.36E-08	-1.27E-08	-1.45E-08	-1.50E-08	-1.51E-08

-9.81E-09	-1.02E-08	-9.34E-09	-1.09E-08	-1.11E-08	-1.20E-08	-1.22E-08	-1.30E-08	-1.36E-08	-1.43E-08	-1.43E-08	-1.29E-08	-1.48E-08	-1.52E-08	-1.55E-08
-9.68E-09	-1.03E-08	-9.97E-09	-1.10E-08	-1.18E-08	-1.31E-08	-1.28E-08	-1.33E-08	-1.40E-08	-1.50E-08	-1.45E-08	-1.39E-08	-1.47E-08	-1.53E-08	-1.53E-08
-9.45E-09	-1.01E-08	-1.04E-08	-1.08E-08	-1.19E-08	-1.41E-08	-1.37E-08	-1.36E-08	-1.44E-08	-1.53E-08	-1.51E-08	-1.53E-08	-1.53E-08	-1.59E-08	-1.56E-08
-9.22E-09	-9.67E-09	-1.08E-08	-1.17E-08	-1.25E-08	-1.39E-08	-1.43E-08	-1.39E-08	-1.39E-08	-1.50E-08	-1.52E-08	-1.57E-08	-1.60E-08	-1.65E-08	-1.67E-08
-8.96E-09	-9.43E-09	-1.12E-08	-1.26E-08	-1.29E-08	-1.37E-08	-1.41E-08	-1.33E-08	-1.34E-08	-1.45E-08	-1.52E-08	-1.56E-08	-1.64E-08	-1.68E-08	-1.73E-08
-9.20E-09	-1.01E-08	-1.13E-08	-1.29E-08	-1.25E-08	-1.30E-08	-1.31E-08	-1.24E-08	-1.26E-08	-1.38E-08	-1.50E-08	-1.59E-08	-1.71E-08	-1.70E-08	-1.74E-08
-1.03E-08	-1.13E-08	-1.22E-08	-1.31E-08	-1.24E-08	-1.26E-08	-1.21E-08	-1.15E-08	-1.32E-08	-1.43E-08	-1.52E-08	-1.70E-08	-1.79E-08	-1.73E-08	-1.72E-08
-1.09E-08	-1.18E-08	-1.31E-08	-1.33E-08	-1.22E-08	-1.20E-08	-1.13E-08	-1.13E-08	-1.40E-08	-1.49E-08	-1.56E-08	-1.79E-08	-1.81E-08	-1.75E-08	-1.67E-08
-1.03E-08	-1.15E-08	-1.27E-08	-1.31E-08	-1.21E-08	-1.15E-08	-1.08E-08	-1.13E-08	-1.42E-08	-1.49E-08	-1.60E-08	-1.79E-08	-1.74E-08	-1.76E-08	-1.68E-08
-1.03E-08	-1.12E-08	-1.23E-08	-1.25E-08	-1.22E-08	-1.18E-08	-1.12E-08	-1.17E-08	-1.37E-08	-1.51E-08	-1.68E-08	-1.79E-08	-1.68E-08	-1.69E-08	-1.68E-08
-1.03E-08	-1.12E-08	-1.19E-08	-1.25E-08	-1.28E-08	-1.20E-08	-1.15E-08	-1.20E-08	-1.39E-08	-1.57E-08	-1.71E-08	-1.75E-08	-1.74E-08	-1.69E-08	-1.66E-08
-1.04E-08	-1.06E-08	-1.20E-08	-1.31E-08	-1.38E-08	-1.23E-08	-1.27E-08	-1.28E-08	-1.47E-08	-1.61E-08	-1.70E-08	-1.73E-08	-1.75E-08	-1.78E-08	-1.65E-08
-1.05E-08	-1.04E-08	-1.24E-08	-1.30E-08	-1.41E-08	-1.34E-08	-1.44E-08	-1.44E-08	-1.54E-08	-1.58E-08	-1.72E-08	-1.76E-08	-1.73E-08	-1.83E-08	-1.70E-08
-1.03E-08	-1.07E-08	-1.24E-08	-1.29E-08	-1.39E-08	-1.40E-08	-1.51E-08	-1.53E-08	-1.59E-08	-1.58E-08	-1.68E-08	-1.78E-08	-1.81E-08	-1.86E-08	-1.74E-08
-1.04E-08	-1.15E-08	-1.24E-08	-1.32E-08	-1.44E-08	-1.42E-08	-1.52E-08	-1.56E-08	-1.61E-08	-1.59E-08	-1.68E-08	-1.76E-08	-1.81E-08	-1.89E-08	-1.75E-08
-1.10E-08	-1.18E-08	-1.18E-08	-1.30E-08	-1.49E-08	-1.41E-08	-1.51E-08	-1.64E-08	-1.65E-08	-1.69E-08	-1.73E-08	-1.75E-08	-1.83E-08	-1.92E-08	-1.84E-08
-1.15E-08	-1.19E-08	-1.12E-08	-1.31E-08	-1.51E-08	-1.43E-08	-1.53E-08	-1.67E-08	-1.67E-08	-1.73E-08	-1.81E-08	-1.79E-08	-1.82E-08	-1.91E-08	-1.88E-08
-1.20E-08	-1.18E-08	-1.12E-08	-1.38E-08	-1.53E-08	-1.48E-08	-1.58E-08	-1.70E-08	-1.64E-08	-1.73E-08	-1.83E-08	-1.78E-08	-1.79E-08	-1.91E-08	-1.88E-08
-1.24E-08	-1.25E-08	-1.24E-08	-1.51E-08	-1.60E-08	-1.60E-08	-1.66E-08	-1.73E-08	-1.63E-08	-1.72E-08	-1.80E-08	-1.78E-08	-1.80E-08	-1.89E-08	-1.85E-08
-1.33E-08	-1.35E-08	-1.37E-08	-1.59E-08	-1.71E-08	-1.70E-08	-1.74E-08	-1.78E-08	-1.67E-08	-1.71E-08	-1.78E-08	-1.81E-08	-1.80E-08	-1.90E-08	-1.85E-08
-1.44E-08	-1.45E-08	-1.47E-08	-1.58E-08	-1.75E-08	-1.72E-08	-1.74E-08	-1.80E-08	-1.72E-08	-1.75E-08	-1.78E-08	-1.81E-08	-1.79E-08	-1.89E-08	-1.88E-08
-1.56E-08	-1.58E-08	-1.59E-08	-1.66E-08	-1.75E-08	-1.69E-08	-1.72E-08	-1.79E-08	-1.76E-08	-1.79E-08	-1.83E-08	-1.84E-08	-1.84E-08	-1.86E-08	-1.90E-08
-1.62E-08	-1.72E-08	-1.68E-08	-1.75E-08	-1.75E-08	-1.71E-08	-1.72E-08	-1.79E-08	-1.82E-08	-1.79E-08	-1.85E-08	-1.84E-08	-1.86E-08	-1.83E-08	-1.93E-08
-1.73E-08	-1.75E-08	-1.74E-08	-1.77E-08	-1.73E-08	-1.73E-08	-1.77E-08	-1.84E-08	-1.85E-08	-1.80E-08	-1.88E-08	-1.86E-08	-1.85E-08	-1.83E-08	-1.89E-08
-1.79E-08	-1.79E-08	-1.77E-08	-1.78E-08	-1.76E-08	-1.78E-08	-1.84E-08	-1.90E-08	-1.88E-08	-1.79E-08	-1.87E-08	-1.90E-08	-1.86E-08	-1.84E-08	-1.84E-08
-1.81E-08	-1.77E-08	-1.78E-08	-1.74E-08	-1.79E-08	-1.88E-08	-1.88E-08	-1.94E-08	-1.91E-08	-1.81E-08	-1.88E-08	-1.96E-08	-1.85E-08	-1.85E-08	-1.85E-08
-1.84E-08	-1.82E-08	-1.82E-08	-1.75E-08	-1.84E-08	-1.92E-08	-1.88E-08	-1.91E-08	-1.93E-08	-1.80E-08	-1.91E-08	-1.95E-08	-1.83E-08	-1.83E-08	-1.87E-08
-1.90E-08	-1.89E-08	-1.89E-08	-1.82E-08	-1.87E-08	-1.92E-08	-1.87E-08	-1.86E-08	-1.89E-08	-1.81E-08	-1.90E-08	-1.90E-08	-1.83E-08	-1.83E-08	-1.90E-08
-1.95E-08	-1.95E-08	-1.92E-08	-1.87E-08	-1.92E-08	-1.90E-08	-1.93E-08	-1.83E-08	-1.91E-08	-1.87E-08	-1.91E-08	-1.91E-08	-1.87E-08	-1.89E-08	-1.92E-08
-2.00E-08	-1.98E-08	-1.93E-08	-1.88E-08	-1.96E-08	-1.92E-08	-1.97E-08	-1.87E-08	-1.96E-08	-1.94E-08	-1.96E-08	-1.94E-08	-1.94E-08	-1.93E-08	-1.92E-08
-2.03E-08	-1.99E-08	-1.93E-08	-1.86E-08	-1.93E-08	-1.94E-08	-2.03E-08	-1.90E-08	-1.98E-08	-1.98E-08	-2.00E-08	-1.94E-08	-1.94E-08	-1.92E-08	-1.92E-08
-2.01E-08	-1.98E-08	-1.94E-08	-1.88E-08	-1.88E-08	-1.93E-08	-2.02E-08	-1.94E-08	-2.01E-08	-2.02E-08	-2.01E-08	-1.95E-08	-1.95E-08	-1.92E-08	-1.98E-08
-2.04E-08	-2.03E-08	-2.02E-08	-1.95E-08	-1.95E-08	-1.96E-08	-2.00E-08	-1.97E-08	-2.08E-08	-2.02E-08	-2.00E-08	-1.97E-08	-1.97E-08	-1.93E-08	-1.99E-08
-2.10E-08	-2.10E-08	-2.07E-08	-2.01E-08	-2.01E-08	-1.94E-08	-2.03E-08	-2.07E-08	-2.11E-08	-2.01E-08	-1.95E-08	-1.95E-08	-1.97E-08	-1.95E-08	-2.02E-08
-2.11E-08	-2.13E-08	-2.08E-08	-2.04E-08	-1.99E-08	-2.01E-08	-2.08E-08	-2.13E-08	-2.11E-08	-2.03E-08	-1.96E-08	-1.98E-08	-1.98E-08	-1.95E-08	-2.03E-08
-2.05E-08	-2.12E-08	-2.08E-08	-2.09E-08	-2.02E-08	-2.06E-08	-2.08E-08	-2.13E-08	-2.07E-08	-2.04E-08	-2.03E-08	-1.96E-08	-1.93E-08	-1.94E-08	-2.03E-08
-2.03E-08	-2.12E-08	-2.03E-08	-2.07E-08	-2.02E-08	-2.00E-08	-2.05E-08	-2.09E-08	-2.08E-08	-2.02E-08	-2.02E-08	-1.95E-08	-1.93E-08	-1.91E-08	-2.02E-08
-2.02E-08	-2.12E-08	-2.00E-08	-2.03E-08	-1.95E-08	-1.95E-08	-2.04E-08	-2.04E-08	-2.05E-08	-1.98E-08	-1.97E-08	-1.91E-08	-1.93E-08	-1.90E-08	-2.01E-08
-2.11E-08	-2.11E-08	-2.04E-08	-1.99E-08	-1.94E-08	-1.97E-08	-2.07E-08	-2.03E-08	-2.04E-08	-1.96E-08	-1.92E-08	-1.92E-08	-1.91E-08	-1.88E-08	-1.94E-08
-2.12E-08	-2.08E-08	-2.04E-08	-2.01E-08	-2.00E-08	-2.04E-08	-2.09E-08	-2.05E-08	-2.01E-08	-1.98E-08	-1.95E-08	-1.89E-08	-1.87E-08	-1.83E-08	-1.89E-08
-2.08E-08	-2.11E-08	-2.05E-08	-2.06E-08	-2.05E-08	-2.02E-08	-2.08E-08	-2.06E-08	-2.02E-08	-1.95E-08	-1.93E-08	-1.86E-08	-1.83E-08	-1.81E-08	-1.83E-08
-2.08E-08	-2.11E-08	-2.06E-08	-2.06E-08	-2.05E-08	-1.98E-08	-2.02E-08	-2.08E-08	-2.02E-08	-1.93E-08	-1.90E-08	-1.78E-08	-1.81E-08	-1.82E-08	-1.79E-08
-2.07E-08	-2.05E-08	-2.04E-08	-2.03E-08	-1.99E-08	-1.92E-08	-1.97E-08	-2.05E-08	-2.02E-08	-1.96E-08	-1.87E-08	-1.77E-08	-1.79E-08	-1.80E-08	-1.73E-08
-2.06E-08	-1.96E-08	-2.00E-08	-1.98E-08	-1.92E-08	-1.88E-08	-1.90E-08	-2.00E-08	-1.98E-08	-1.94E-08	-1.82E-08	-1.79E-08	-1.76E-08	-1.76E-08	-1.69E-08
-1.98E-08	-1.91E-08	-1.98E-08	-1.96E-08	-1.84E-08	-1.85E-08	-1.91E-08	-1.92E-08	-1.91E-08	-1.86E-08	-1.74E-08	-1.76E-08	-1.72E-08	-1.76E-08	-1.65E-08
-1.94E-08	-1.89E-08	-1.92E-08	-1.93E-08	-1.80E-08	-1.85E-08	-1.93E-08	-1.89E-08	-1.88E-08	-1.75E-08	-1.72E-08	-1.71E-08	-1.67E-08	-1.72E-08	-1.70E-08
-1.96E-08	-1.88E-08	-1.89E-08	-1.89E-08	-1.81E-08	-1.83E-08	-1.89E-08	-1.89E-08	-1.88E-08	-1.67E-08	-1.72E-08	-1.71E-08	-1.65E-08	-1.68E-08	-1.71E-08
-1.94E-08	-1.89E-08	-1.90E-08	-1.94E-08	-1.87E-08	-1.81E-08	-1.84E-08	-1.86E-08	-1.87E-08	-1.65E-08	-1.69E-08	-1.68E-08	-1.61E-08	-1.66E-08	-1.70E-08
-1.94E-08	-1.83E-08	-1.90E-08	-1.95E-08	-1.90E-08	-1.79E-08	-1.78E-08	-1.79E-08	-1.81E-08	-1.69E-08	-1.62E-08	-1.60E-08	-1.62E-08	-1.64E-08	-1.66E-08
-1.93E-08	-1.83E-08	-1.88E-08	-1.93E-08	-1.90E-08	-1.81E-08	-1.77E-08	-1.78E-08	-1.81E-08	-1.71E-08	-1.55E-08	-1.54E-08	-1.58E-08	-1.62E-08	-1.64E-08

1.99E-15	1.95E-15	1.95E-15	2.03E-15	2.05E-15	2.00E-15	2.04E-15	2.20E-15	2.15E-15	2.20E-15	2.18E-15	2.14E-15	2.02E-15	2.01E-15	2.05E-15
1.97E-15	1.94E-15	1.91E-15	1.95E-15	1.94E-15	1.87E-15	1.95E-15	2.06E-15	1.99E-15	2.03E-15	2.08E-15	2.09E-15	1.96E-15	1.94E-15	1.93E-15
1.85E-15	1.87E-15	1.78E-15	1.76E-15	1.82E-15	1.78E-15	1.87E-15	1.89E-15	1.88E-15	1.85E-15	1.96E-15	1.97E-15	1.87E-15	1.87E-15	1.83E-15
1.72E-15	1.71E-15	1.60E-15	1.61E-15	1.70E-15	1.67E-15	1.78E-15	1.74E-15	1.75E-15	1.71E-15	1.80E-15	1.87E-15	1.80E-15	1.78E-15	1.75E-15
1.56E-15	1.54E-15	1.49E-15	1.49E-15	1.56E-15	1.58E-15	1.63E-15	1.63E-15	1.61E-15	1.59E-15	1.63E-15	1.73E-15	1.72E-15	1.65E-15	1.70E-15
1.41E-15	1.42E-15	1.38E-15	1.38E-15	1.43E-15	1.47E-15	1.50E-15	1.53E-15	1.49E-15	1.49E-15	1.54E-15	1.60E-15	1.59E-15	1.57E-15	1.63E-15
1.30E-15	1.31E-15	1.27E-15	1.29E-15	1.35E-15	1.36E-15	1.37E-15	1.45E-15	1.44E-15	1.42E-15	1.45E-15	1.47E-15	1.45E-15	1.46E-15	1.53E-15
1.21E-15	1.22E-15	1.20E-15	1.22E-15	1.27E-15	1.24E-15	1.26E-15	1.35E-15	1.36E-15	1.31E-15	1.38E-15	1.40E-15	1.34E-15	1.36E-15	1.41E-15
1.14E-15	1.13E-15	1.15E-15	1.16E-15	1.20E-15	1.16E-15	1.19E-15	1.22E-15	1.26E-15	1.22E-15	1.30E-15	1.29E-15	1.23E-15	1.26E-15	1.30E-15
1.07E-15	1.04E-15	1.08E-15	1.11E-15	1.11E-15	1.10E-15	1.10E-15	1.09E-15	1.14E-15	1.12E-15	1.15E-15	1.11E-15	1.14E-15	1.15E-15	1.22E-15
9.85E-16	9.71E-16	9.75E-16	1.04E-15	1.04E-15	1.02E-15	9.80E-16	9.75E-16	1.03E-15	1.06E-15	1.04E-15	1.01E-15	1.04E-15	1.04E-15	1.12E-15
9.10E-16	9.34E-16	9.04E-16	9.59E-16	9.64E-16	9.54E-16	9.18E-16	9.15E-16	9.42E-16	1.02E-15	9.96E-16	9.84E-16	9.39E-16	9.59E-16	9.96E-16
8.51E-16	8.85E-16	8.59E-16	8.92E-16	8.81E-16	8.83E-16	8.78E-16	8.53E-16	8.91E-16	9.64E-16	9.82E-16	9.50E-16	8.61E-16	8.87E-16	9.13E-16
7.73E-16	8.29E-16	8.09E-16	8.12E-16	8.24E-16	8.27E-16	8.14E-16	8.09E-16	8.75E-16	9.28E-16	9.31E-16	8.92E-16	8.17E-16	8.33E-16	8.52E-16
7.02E-16	7.44E-16	7.55E-16	7.38E-16	7.50E-16	7.58E-16	7.52E-16	7.83E-16	8.52E-16	8.38E-16	8.43E-16	8.25E-16	7.99E-16	7.87E-16	7.84E-16
6.64E-16	6.81E-16	6.95E-16	6.98E-16	6.63E-16	6.94E-16	7.42E-16	7.40E-16	8.14E-16	7.49E-16	7.47E-16	7.63E-16	7.57E-16	7.41E-16	7.12E-16
6.04E-16	6.27E-16	6.15E-16	6.72E-16	6.08E-16	6.45E-16	7.05E-16	6.74E-16	7.20E-16	6.95E-16	6.87E-16	7.04E-16	6.76E-16	6.70E-16	6.38E-16
5.40E-16	5.79E-16	5.49E-16	6.23E-16	5.64E-16	5.97E-16	6.30E-16	6.15E-16	6.17E-16	6.00E-16	5.93E-16	5.94E-16	5.99E-16	6.05E-16	5.78E-16
4.89E-16	5.20E-16	5.11E-16	5.56E-16	5.22E-16	5.59E-16	5.57E-16	5.63E-16	5.42E-16	5.35E-16	5.17E-16	5.19E-16	5.42E-16	5.54E-16	5.36E-16
4.55E-16	4.93E-16	4.83E-16	4.85E-16	4.74E-16	5.10E-16	5.07E-16	5.05E-16	5.02E-16	4.99E-16	4.65E-16	4.75E-16	4.99E-16	4.96E-16	4.91E-16
4.31E-16	4.55E-16	4.51E-16	4.43E-16	4.19E-16	4.75E-16	4.53E-16	4.51E-16	4.61E-16	4.46E-16	4.17E-16	4.30E-16	4.54E-16	4.57E-16	4.64E-16
4.09E-16	4.25E-16	4.03E-16	4.11E-16	3.78E-16	4.31E-16	4.07E-16	3.80E-16	4.06E-16	4.04E-16	3.82E-16	4.00E-16	4.15E-16	4.18E-16	4.55E-16
3.86E-16	3.95E-16	3.58E-16	3.79E-16	3.62E-16	3.84E-16	3.71E-16	3.47E-16	3.59E-16	3.70E-16	3.51E-16	3.76E-16	3.87E-16	3.87E-16	4.19E-16
3.51E-16	3.62E-16	3.40E-16	3.52E-16	3.49E-16	3.46E-16	3.56E-16	3.30E-16	3.38E-16	3.51E-16	3.23E-16	3.49E-16	3.89E-16	3.70E-16	3.82E-16
3.17E-16	3.15E-16	3.13E-16	3.25E-16	3.20E-16	3.14E-16	3.29E-16	3.02E-16	3.07E-16	3.15E-16	3.04E-16	3.21E-16	3.64E-16	3.32E-16	3.57E-16
2.86E-16	2.64E-16	2.71E-16	2.78E-16	2.86E-16	2.75E-16	2.83E-16	2.67E-16	2.61E-16	2.88E-16	2.78E-16	3.00E-16	2.77E-16	2.88E-16	3.13E-16
2.60E-16	2.35E-16	2.45E-16	2.59E-16	2.74E-16	2.48E-16	2.37E-16	2.25E-16	2.25E-16	2.54E-16	2.54E-16	2.46E-16	2.24E-16	2.50E-16	2.88E-16
2.21E-16	2.14E-16	2.33E-16	2.78E-16	2.75E-16	2.23E-16	2.06E-16	1.85E-16	2.08E-16	2.34E-16	2.33E-16	2.12E-16	2.05E-16	2.38E-16	2.47E-16
1.74E-16	1.79E-16	2.36E-16	2.89E-16	2.64E-16	1.94E-16	1.72E-16	1.63E-16	1.83E-16	2.06E-16	1.95E-16	2.04E-16	2.11E-16	2.27E-16	2.34E-16
1.33E-16	1.43E-16	2.09E-16	2.96E-16	2.55E-16	1.70E-16	1.50E-16	1.45E-16	1.59E-16	1.89E-16	1.78E-16	2.05E-16	2.08E-16	2.22E-16	2.05E-16
1.19E-16	1.27E-16	1.86E-16	2.63E-16	2.32E-16	1.47E-16	1.26E-16	1.28E-16	1.37E-16	1.73E-16	1.59E-16	1.92E-16	1.92E-16	2.01E-16	1.70E-16

1.12E-16	1.11E-16	1.37E-16	1.50E-16	1.45E-16	1.25E-16	1.16E-16	1.22E-16	1.16E-16	1.42E-16	1.34E-16	1.54E-16	1.67E-16	1.55E-16	1.40E-16
1.06E-16	9.08E-17	1.24E-16	9.67E-17	1.16E-16	1.13E-16	1.06E-16	1.18E-16	1.01E-16	1.12E-16	1.14E-16	1.12E-16	1.35E-16	1.05E-16	1.08E-16
9.28E-17	6.84E-17	1.00E-16	7.53E-17	8.99E-17	9.27E-17	8.51E-17	9.36E-17	8.26E-17	8.18E-17	8.52E-17	9.03E-17	1.06E-16	8.33E-17	8.50E-17
6.99E-17	5.71E-17	7.96E-17	5.90E-17	6.76E-17	7.03E-17	5.98E-17	7.08E-17	7.32E-17	5.89E-17	6.50E-17	7.63E-17	8.69E-17	6.86E-17	6.99E-17
5.14E-17	4.97E-17	6.41E-17	5.52E-17	6.34E-17	5.36E-17	4.93E-17	5.25E-17	5.98E-17	4.28E-17	6.00E-17	6.17E-17	6.88E-17	5.52E-17	5.84E-17
4.83E-17	4.11E-17	5.06E-17	4.57E-17	5.88E-17	4.51E-17	4.15E-17	4.08E-17	4.68E-17	3.40E-17	5.15E-17	5.02E-17	6.32E-17	4.61E-17	4.17E-17
4.18E-17	3.17E-17	4.02E-17	3.62E-17	4.99E-17	4.11E-17	2.79E-17	3.23E-17	3.35E-17	2.95E-17	4.46E-17	4.10E-17	5.60E-17	3.82E-17	2.36E-17
2.96E-17	2.68E-17	2.82E-17	2.76E-17	3.32E-17	3.71E-17	2.15E-17	2.86E-17	2.61E-17	2.50E-17	3.78E-17	3.89E-17	4.36E-17	2.98E-17	1.76E-17
2.32E-17	2.07E-17	2.02E-17	1.91E-17	2.30E-17	2.95E-17	2.09E-17	2.29E-17	2.13E-17	1.75E-17	2.68E-17	3.04E-17	2.27E-17	2.59E-17	1.78E-17
1.57E-17	1.52E-17	1.35E-17	1.59E-17	1.92E-17	2.19E-17	2.09E-17	1.87E-17	1.74E-17	1.10E-17	1.84E-17	1.50E-17	1.00E-17	2.28E-17	1.56E-17
9.22E-18	1.19E-17	7.65E-18	9.83E-18	1.21E-17	1.78E-17	1.75E-17	1.63E-17	1.28E-17	9.34E-18	1.42E-17	8.23E-18	5.07E-18	1.29E-17	9.33E-18
6.79E-18	1.03E-17	3.35E-18	6.50E-18	1.11E-17	1.70E-17	1.36E-17	1.46E-17	9.64E-18	7.73E-18	9.73E-18	3.96E-18	2.85E-18	4.49E-18	4.95E-18
4.69E-18	6.83E-18	1.18E-18	4.96E-18	8.76E-18	1.20E-17	6.81E-18	8.95E-18	5.74E-18	4.60E-18	6.30E-18	1.26E-18	1.16E-18	2.60E-18	1.65E-18
1.99E-18	3.93E-18	4.86E-19	2.25E-18	6.01E-18	7.71E-18	3.75E-18	6.39E-18	4.05E-18	2.77E-18	3.59E-18	5.70E-19	2.97E-19	1.38E-18	2.45E-19
9.60E-20	1.08E-18	7.77E-22	3.62E-19	1.18E-18	1.72E-18	1.85E-18	2.02E-18	1.21E-18	6.37E-19	3.44E-19	1.60E-19	2.08E-19	9.99E-22	3.50E-19
6.46E-19	4.59E-20	7.90E-20	9.36E-21	1.30E-19	1.26E-19	7.70E-19	5.10E-19	1.48E-19	1.67E-19	2.85E-19	1.38E-18	1.75E-18	4.79E-19	1.25E-18
2.94E-18	4.62E-19	4.52E-19	2.81E-19	1.45E-19	1.01E-19	1.51E-21	1.31E-19	8.66E-19	2.42E-18	1.34E-18	3.13E-18	4.01E-18	2.08E-18	3.54E-18
5.24E-18	8.18E-19	9.61E-19	1.91E-18	1.51E-18	1.11E-18	1.07E-18	2.82E-18	3.29E-18	4.39E-18	2.69E-18	5.18E-18	6.94E-18	3.16E-18	5.07E-18
8.26E-18	3.37E-18	2.40E-18	4.75E-18	3.19E-18	2.06E-18	2.24E-18	6.96E-18	4.54E-18	4.29E-18	4.43E-18	5.22E-18	7.33E-18	3.04E-18	4.70E-18
1.32E-17	1.04E-17	4.50E-18	6.77E-18	4.36E-18	3.59E-18	4.21E-18	9.72E-18	5.23E-18	4.02E-18	6.10E-18	6.32E-18	8.39E-18	4.30E-18	5.92E-18
1.87E-17	1.62E-17	7.91E-18	1.03E-17	7.50E-18	7.21E-18	8.06E-18	1.23E-17	4.96E-18	6.35E-18	8.68E-18	6.10E-18	8.87E-18	6.87E-18	7.95E-18
2.01E-17	1.82E-17	1.10E-17	1.55E-17	1.04E-17	1.18E-17	1.46E-17	1.52E-17	7.78E-18	8.97E-18	1.25E-17	6.30E-18	1.16E-17	1.05E-17	9.88E-18
2.03E-17	1.67E-17	1.54E-17	1.82E-17	1.07E-17	1.64E-17	1.49E-17	1.10E-17	9.17E-18	1.05E-17	1.30E-17	9.49E-18	1.23E-17	1.04E-17	9.21E-18
2.49E-17	2.20E-17	2.67E-17	2.49E-17	1.90E-17	2.29E-17	1.78E-17	1.44E-17	9.47E-18	1.31E-17	1.29E-17	1.82E-17	1.40E-17	1.19E-17	8.22E-18
3.32E-17	3.07E-17	2.81E-17	3.24E-17	2.61E-17	2.46E-17	2.19E-17	1.62E-17	1.06E-17	1.38E-17	1.35E-17	2.64E-17	1.86E-17	1.28E-17	1.41E-17
4.22E-17	3.22E-17	3.16E-17	3.78E-17	3.12E-17	2.68E-17	2.47E-17	1.78E-17	1.56E-17	1.68E-17	1.96E-17	2.86E-17	2.38E-17	1.89E-17	3.08E-17
4.71E-17	3.48E-17	4.00E-17	4.40E-17	4.20E-17	2.87E-17	2.46E-17	1.91E-17	2.35E-17	2.39E-17	2.54E-17	2.61E-17	2.69E-17	2.63E-17	4.47E-17
5.42E-17	4.34E-17	5.08E-17	5.12E-17	5.27E-17	3.21E-17	2.51E-17	2.47E-17	2.86E-17	2.89E-17	2.78E-17	2.65E-17	2.65E-17	3.53E-17	5.75E-17
6.35E-17	4.99E-17	6.25E-17	5.64E-17	5.42E-17	3.80E-17	2.84E-17	2.94E-17	3.49E-17	2.76E-17	3.17E-17	2.89E-17	2.75E-17	4.04E-17	6.58E-17
6.62E-17	5.39E-17	6.95E-17	6.30E-17	5.96E-17	4.56E-17	3.06E-17	3.06E-17	4.33E-17	3.41E-17	3.57E-17	3.55E-17	3.09E-17	5.07E-17	6.49E-17
7.30E-17	6.18E-17	7.41E-17	6.62E-17	6.90E-17	5.52E-17	3.97E-17	3.72E-17	5.17E-17	4.24E-17	3.74E-17	4.78E-17	4.33E-17	6.20E-17	7.04E-17
8.50E-17	7.42E-17	7.84E-17	7.21E-17	8.04E-17	6.67E-17	5.30E-17	5.11E-17	5.43E-17	5.44E-17	3.82E-17	5.85E-17	5.80E-17	7.36E-17	8.19E-17
9.34E-17	9.02E-17	8.29E-17	7.17E-17	8.30E-17	7.72E-17	7.00E-17	6.98E-17	5.57E-17	6.20E-17	4.98E-17	7.97E-17	8.46E-17	9.75E-17	9.44E-17
9.87E-17	1.01E-16	8.90E-17	6.72E-17	7.87E-17	7.84E-17	6.75E-17	7.58E-17	5.92E-17	6.95E-17	7.95E-17	9.90E-17	1.11E-16	1.27E-16	9.92E-17
1.01E-16	1.01E-16	8.97E-17	7.27E-17	8.05E-17	8.08E-17	5.82E-17	6.78E-17	5.58E-17	8.04E-17	9.46E-17	1.10E-16	1.30E-16	1.42E-16	1.11E-16
1.02E-16	9.86E-17	9.42E-17	9.21E-17	9.65E-17	8.85E-17	5.67E-17	5.72E-17	5.82E-17	8.61E-17	9.80E-17	1.24E-16	1.30E-16	1.46E-16	1.40E-16
1.04E-16	9.92E-17	1.08E-16	1.11E-16	1.08E-16	9.94E-17	5.84E-17	5.16E-17	6.23E-17	8.64E-17	1.11E-16	1.37E-16	1.39E-16	1.47E-16	1.65E-16
1.05E-16	1.06E-16	1.15E-16	1.28E-16	1.18E-16	1.07E-16	6.58E-17	4.83E-17	5.71E-17	7.99E-17	1.13E-16	1.48E-16	1.52E-16	1.45E-16	1.78E-16
1.07E-16	1.09E-16	1.08E-16	1.24E-16	1.16E-16	1.03E-16	7.40E-17	6.77E-17	6.74E-17	8.00E-17	1.15E-16	1.51E-16	1.52E-16	1.52E-16	1.76E-16
1.03E-16	1.09E-16	1.03E-16	1.20E-16	1.17E-16	9.41E-17	8.41E-17	9.08E-17	7.30E-17	8.87E-17	1.27E-16	1.57E-16	1.48E-16	1.60E-16	1.81E-16
9.50E-17	1.07E-16	9.78E-17	1.11E-16	1.14E-16	9.96E-17	1.15E-16	1.13E-16	8.39E-17	9.73E-17	1.36E-16	1.68E-16	1.46E-16	1.61E-16	1.69E-16
7.58E-17	9.60E-17	9.18E-17	1.01E-16	1.06E-16	1.10E-16	1.40E-16	1.41E-16	1.12E-16	1.23E-16	1.52E-16	1.64E-16	1.51E-16	1.68E-16	1.73E-16
5.55E-17	8.58E-17	9.59E-17	8.51E-17	1.02E-16	1.25E-16	1.48E-16	1.48E-16	1.38E-16	1.42E-16	1.48E-16	1.52E-16	1.52E-16	1.88E-16	1.85E-16
5.43E-17	8.89E-17	9.36E-17	6.91E-17	1.05E-16	1.30E-16	1.44E-16	1.41E-16	1.46E-16	1.49E-16	1.42E-16	1.53E-16	1.70E-16	2.11E-16	2.00E-16
6.69E-17	1.01E-16	9.00E-17	7.21E-17	1.17E-16	1.35E-16	1.42E-16	1.37E-16	1.49E-16	1.52E-16	1.44E-16	1.55E-16	1.93E-16	2.26E-16	2.16E-16
7.98E-17	1.06E-16	8.20E-17	1.21E-16	1.21E-16	1.38E-16	1.49E-16	1.47E-16	1.54E-16	1.71E-16	1.58E-16	1.50E-16	2.00E-16	2.32E-16	2.20E-16
8.63E-17	1.05E-16	8.01E-17	1.26E-16	1.25E-16	1.39E-16	1.41E-16	1.61E-16	1.73E-16	1.92E-16	1.86E-16	1.61E-16	2.11E-16	2.25E-16	2.27E-16
9.63E-17	1.05E-16	8.72E-17	1.20E-16	1.23E-16	1.43E-16	1.48E-16	1.69E-16	1.84E-16	2.06E-16	2.05E-16	1.67E-16	2.19E-16	2.32E-16	2.39E-16
9.37E-17	1.07E-16	9.94E-17	1.21E-16	1.39E-16	1.71E-16	1.64E-16	1.78E-16	1.97E-16	2.26E-16	2.11E-16	1.93E-16	2.17E-16	2.36E-16	2.36E-16
8.93E-17	1.03E-16	1.08E-16	1.17E-16	1.41E-16	1.98E-16	1.87E-16	1.84E-16	2.06E-16	2.33E-16	2.27E-16	2.34E-16	2.33E-16	2.52E-16	2.45E-16
8.51E-17	9.36E-17	1.17E-16	1.37E-16	1.56E-16	1.94E-16	2.04E-16	1.94E-16	1.92E-16	2.26E-16	2.32E-16	2.45E-16	2.57E-16	2.74E-16	2.79E-16
8.03E-17	8.89E-17	1.26E-16	1.59E-16	1.66E-16	1.88E-16	1.98E-16	1.77E-16	1.80E-16	2.11E-16	2.30E-16	2.44E-16	2.68E-16	2.82E-16	2.99E-16
8.46E-17	1.03E-16	1.29E-16	1.67E-16	1.56E-16	1.69E-16	1.73E-16	1.54E-16	1.60E-16	1.92E-16	2.26E-16	2.54E-16	2.92E-16	2.90E-16	3.03E-16
1.06E-16	1.27E-16	1.49E-16	1.72E-16	1.53E-16	1.60E-16	1.48E-16	1.31E-16	1.74E-16	2.04E-16	2.31E-16	2.88E-16	3.22E-16	3.01E-16	2.96E-16
1.18E-16	1.40E-16	1.72E-16	1.76E-16	1.49E-16	1.43E-16	1.27E-16	1.28E-16	1.96E-16	2.22E-16	2.43E-16	3.22E-16	3.27E-16	3.07E-16	2.78E-16
1.06E-16	1.33E-16	1.60E-16	1.71E-16	1.47E-16	1.32E-16	1.17E-16	1.27E-16	2.01E-16	2.23E-16	2.56E-16	3.20E-16	3.04E-16	3.08E-16	2.84E-16
1.06E-16	1.26E-16	1.51E-16	1.56E-16	1.49E-16	1.39E-16	1.25E-16	1.38E-16	1.88E-16	2.29E-16	2.81E-16	3.22E-16	2.82E-16	2.87E-16	2.82E-16
1.07E-16	1.24E-16	1.42E-16	1.56E-16	1.64E-16	1.43E-16	1.32E-16	1.45E-16	1.92E-16	2.46E-16	2.92E-16	3.05E-16	3.02E-16	2.87E-16	2.77E-16
1.07E-16	1.12E-16	1.45E-16	1.71E-16	1.91E-16	1.52E-16	1.62E-16	1.63E-16	2.16E-16	2.58E-16	2.89E-16	2.99E-16	3.07E-16	3.16E-16	2.72E-16
1.10E-16	1.08E-16	1.53E-16	1.69E-16	1.99E-16	1.81E-16	2.07E-16	2.08E-16	2.38E-16	2.51E-16	2.96E-16	3.10E-16	2.99E-16	3.36E-16	2.88E-16
1.05E-16	1.14E-16	1.55E-16	1.68E-16	1.93E-16	1.95E-16	2.29E-16	2.34E-16	2.54E-16	2.50E-16	2.81E-16	3.16E-16	3.26E-16	3.46E-16	3.01E-16
1.07E-16	1.31E-16	1.53E-16	1.74E-16	2.06E-16	2.03E-16	2.31E-16	2.44E-16	2.59E-16	2.53E-16	2.82E-16	3.09E-16	3.26E-16	3.57E-16	3.05E-16
1.20E-16	1.39E-16	1.39E-16	1.69E-16	2.21E-16	2.00E-16	2.27E-16	2.70E-16	2.71E-16	2.85E-16	3.01E-16	3.07E-16	3.33E-16	3.68E-16	3.37E-16
1.32E-16	1.41E-16	1.26E-16	1.72E-16	2.28E-16	2.03E-16	2.34E-16	2.79E-16	2.78E-16	3.00E-16	3.28E-16	3.19E-16	3.32E-16	3.66E-16	3.55E-16
1.44E-16	1.38E-16	1.26E-16	1.90E-16	2.34E-16	2.19E-16	2.50E-16	2.88E-16	2.69E-16	2.98E-16	3.33E-16	3.15E-16	3.21E-16	3.65E-16	3.52E-16
1.54E-16	1.57E-16	1.55E-16	2.28E-16	2.57E-16	2.57E-16	2.77E-16	2.99E-16	2.67E-16	2.94E-16	3.23E-16	3.17E-16	3.23E-16	3.57E-16	3.42E-16
1.76E-16	1.82E-16	1.87E-16	2.52E-16	2.93E-16	2.90E-16	3.02E-16	3.16E-16	2.80E-16	2.94E-16	3.15E-16	3.29E-16	3.24E-16	3.60E-16	3.41E-16
2.08E-16	2.10E-16	2.17E-16	2.50E-16	3.06E-16	2.96E-16	3.04E-16	3.25E-16	2.95E-16	3.06E-16	3.18E-16	3.27E-16	3.20E-16	3.58E-16	3.52E-16
2.43E-16	2.51E-16	2.54E-16	2.76E-16	3.06E-16	2.84E-16	2.97E-16	3.21E-16	3.11E-16	3.21E-16	3.34E-16	3.38E-16	3.40E-16	3.45E-16	3.62E-16
2.63E-16	2.95E-16	2.83E-16	3.07E-16	3.05E-16	2.94E-16	2.96E-16	3.21E-16	3.33E-16	3.20E-16	3.43E-16	3.40E-16	3.45E-16	3.37E-16	3.71E-16
2.98E-16	3.08E-16	3.04E-16	3.15E-16	2.98E-16	3.00E-16	3.14E-16	3.38E-16	3.41E-16	3.26E-16	3.55E-16	3.46E-16	3.43E-16	3.35E-16	3.57E-16
3.19E-16	3.19E-16	3.12E-16	3.17E-16	3.08E-16	3.18E-16	3.37E-16	3.60E-16	3.52E-16	3.20E-16	3.50E-16	3.60E-16	3.48E-16	3.40E-16	3.37E-16
3.28E-16	3.15E-16	3.18E-16	3.02E-16	3.22E-16	3.52E-16	3.55E-16	3.77E-16	3.64E-16	3.27E-16	3.55E-16	3.82E-16	3.44E-16	3.42E-16	3.44E-16
3.38E-16	3.30E-16	3.31E-16	3.05E-16	3.40E-16	3.70E-16	3.53E-16	3.66E-16	3.72E-16	3.25E-16	3.65E-16	3.80E-16	3.35E-16	3.35E-16	3.51E-16
3.62E-16	3.59E-16	3.56E-16	3.30E-16	3.50E-16	3.68E-16	3.48E-16	3.45E-16	3.57E-16	3.29E-16	3.60E-16	3.62E-16	3.35E-16	3.34E-16	3.60E-16
3.81E-16	3.81E-16	3.69E-16	3.51E-16	3.69E-16	3.61E-16	3.73E-16	3.36E-16	3.66E-16	3.49E-16	3.65E-16	3.64E-16	3.51E-16	3.57E-16	3.68E-16
3.99E-16	3.93E-16	3.74E-16	3.55E-16	3.85E-16	3.67E-16	3.87E-16	3.50E-16	3.85E-16	3.77E-16	3.82E-16	3.76E-16	3.76E-16	3.72E-16	3.67E-16
4.11E-16	3.94E-16	3.74E-16	3.47E-16	3.71E-16	3.76E-16	4.11E-16	3.63E-16	3.92E-16	3.90E-16	3.99E-16	3.78E-16	3.77E-16	3.67E-16	3.70E-16
4.06E-16	3.90E-16	3.75E-16	3.52E-16	3.52E-16	3.73E-16	4.09E-16	3.77E-16	4.02E-16	4.06E-16	4.03E-16	3.82E-16	3.78E-16	3.67E-16	3.92E-16
4.18E-16	4.12E-16	4.06E-16	3.82E-16	3.81E-16	3.83E-16	3.99E-16	3.89E-16	4.32E-16	4.07E-16	3.99E-16	3.87E-16	3.86E-16	3.72E-16	3.95E-16
4.40E-16	4.43E-16	4.29E-16	4.03E-16	4.05E-16	3.76E-16	4.13E-16	4.30E-16	4.46E-16	4.04E-16	3.81E-16	3.81E-16	3.89E-16	3.78E-16	4.07E-16
4.46E-16	4.55E-16	4.34E-16	4.16E-16	3.96E-16	4.05E-16	4.32E-16	4.52E-16	4.44E-16	4.12E-16	3.86E-16	3.93E-16	3.90E-16	3.78E-16	4.12E-16
4.22E-16	4.48E-16	4.34E-16	4.35E-16	4.09E-16	4.26E-16	4.33E-16	4.53E-16	4.27E-16	4.15E-16	4.11E-16	3.86E-16	3.72E-16	3.76E-16	4.13E-16
4.13E-16	4.51E-16	4.12E-16	4.30E-16	4.08E-16	3.99E-16	4.22E-16	4.37E-16	4.34E-16	4.06E-16	4.08E-16	3.79E-16	3.74E-16	3.65E-16	4.08E-16
4.09E-16	4.50E-16	3.98E-16	4.12E-16	3.80E-16	3.82E-16	4.15E-16	4.16E-16	4.20E-16	3.91E-16	3.86E-16	3.65E-16	3.71E-16	3.62E-16	4.03E-16
4.45E-16	4.45E-16	4.14E-16	3.95E-16	3.76E-16	3.89E-16	4.28E-16	4.11E-16	4.15E-16	3.82E-16	3.69E-16	3.68E-16	3.66E-16	3.52E-16	3.78E-16

4.48E-16	4.31E-16	4.16E-16	4.05E-16	4.00E-16	4.15E-16	4.37E-16	4.19E-16	4.05E-16	3.94E-16	3.79E-16	3.58E-16	3.51E-16	3.35E-16	3.57E-16
4.32E-16	4.43E-16	4.20E-16	4.24E-16	4.21E-16	4.10E-16	4.35E-16	4.26E-16	4.10E-16	3.79E-16	3.73E-16	3.46E-16	3.36E-16	3.27E-16	3.35E-16
4.34E-16	4.44E-16	4.22E-16	4.24E-16	4.19E-16	3.93E-16	4.10E-16	4.32E-16	4.10E-16	3.73E-16	3.60E-16	3.19E-16	3.26E-16	3.31E-16	3.19E-16
4.29E-16	4.20E-16	4.15E-16	4.10E-16	3.94E-16	3.70E-16	3.88E-16	4.20E-16	4.06E-16	3.86E-16	3.48E-16	3.14E-16	3.19E-16	3.22E-16	2.98E-16
4.23E-16	3.84E-16	4.00E-16	3.92E-16	3.69E-16	3.52E-16	3.61E-16	4.00E-16	3.90E-16	3.77E-16	3.30E-16	3.21E-16	3.09E-16	3.11E-16	2.84E-16
3.94E-16	3.64E-16	3.94E-16	3.83E-16	3.40E-16	3.41E-16	3.67E-16	3.70E-16	3.66E-16	3.45E-16	3.03E-16	3.10E-16	2.97E-16	3.11E-16	2.72E-16
3.77E-16	3.56E-16	3.69E-16	3.71E-16	3.22E-16	3.41E-16	3.72E-16	3.56E-16	3.53E-16	3.06E-16	2.94E-16	2.94E-16	2.79E-16	2.97E-16	2.89E-16
3.83E-16	3.53E-16	3.56E-16	3.56E-16	3.26E-16	3.36E-16	3.57E-16	3.56E-16	3.55E-16	2.78E-16	2.94E-16	2.92E-16	2.72E-16	2.81E-16	2.92E-16
3.75E-16	3.57E-16	3.59E-16	3.75E-16	3.51E-16	3.26E-16	3.40E-16	3.47E-16	3.51E-16	2.72E-16	2.86E-16	2.81E-16	2.61E-16	2.74E-16	2.88E-16
3.77E-16	3.36E-16	3.61E-16	3.81E-16	3.59E-16	3.21E-16	3.15E-16	3.21E-16	3.29E-16	2.85E-16	2.63E-16	2.55E-16	2.62E-16	2.69E-16	2.76E-16
3.74E-16	3.35E-16	3.53E-16	3.74E-16	3.60E-16	3.28E-16	3.15E-16	3.16E-16	3.28E-16	2.94E-16	2.40E-16	2.37E-16	2.49E-16	2.64E-16	2.69E-16
4.14E-14	4.18E-14	4.16E-14	4.30E-14	4.34E-14	4.30E-14	4.37E-14	4.43E-14	4.51E-14	4.51E-14	4.61E-14	4.68E-14	4.64E-14	4.69E-14	4.75E-14

4.58E-08	4.49E-08	4.41E-08	4.49E-08	4.52E-08	4.43E-08	4.45E-08	4.43E-08	4.50E-08	4.44E-08	4.48E-08	4.49E-08	4.28E-08	4.33E-08	4.47E-08
4.46E-08	4.38E-08	4.35E-08	4.40E-08	4.44E-08	4.36E-08	4.30E-08	4.27E-08	4.39E-08	4.35E-08	4.32E-08	4.31E-08	4.14E-08	4.21E-08	4.40E-08
4.33E-08	4.29E-08	4.29E-08	4.29E-08	4.27E-08	4.25E-08	4.20E-08	4.15E-08	4.25E-08	4.23E-08	4.18E-08	4.16E-08	4.02E-08	4.06E-08	4.20E-08
4.21E-08	4.18E-08	4.14E-08	4.16E-08	4.11E-08	4.12E-08	4.09E-08	4.07E-08	4.11E-08	4.09E-08	4.08E-08	4.07E-08	3.97E-08	3.96E-08	4.05E-08
4.10E-08	4.05E-08	4.00E-08	3.98E-08	3.96E-08	3.99E-08	3.97E-08	3.95E-08	3.98E-08	3.94E-08	4.01E-08	4.02E-08	3.94E-08	3.86E-08	3.95E-08
3.98E-08	3.93E-08	3.83E-08	3.85E-08	3.83E-08	3.84E-08	3.82E-08	3.83E-08	3.87E-08	3.82E-08	3.91E-08	3.93E-08	3.88E-08	3.74E-08	3.83E-08
3.86E-08	3.81E-08	3.70E-08	3.73E-08	3.77E-08	3.72E-08	3.68E-08	3.69E-08	3.75E-08	3.78E-08	3.77E-08	3.76E-08	3.73E-08	3.61E-08	3.65E-08
3.74E-08	3.69E-08	3.60E-08	3.64E-08	3.72E-08	3.64E-08	3.60E-08	3.63E-08	3.65E-08	3.70E-08	3.63E-08	3.61E-08	3.65E-08	3.54E-08	3.58E-08
3.58E-08	3.55E-08	3.50E-08	3.55E-08	3.62E-08	3.57E-08	3.58E-08	3.56E-08	3.53E-08	3.57E-08	3.57E-08	3.50E-08	3.54E-08	3.47E-08	3.50E-08
3.43E-08	3.42E-08	3.38E-08	3.41E-08	3.46E-08	3.50E-08	3.54E-08	3.45E-08	3.38E-08	3.50E-08	3.54E-08	3.46E-08	3.43E-08	3.41E-08	3.43E-08
3.33E-08	3.31E-08	3.27E-08	3.30E-08	3.33E-08	3.37E-08	3.40E-08	3.36E-08	3.30E-08	3.42E-08	3.47E-08	3.41E-08	3.37E-08	3.40E-08	3.42E-08
3.15E-08	3.23E-08	3.18E-08	3.18E-08	3.21E-08	3.19E-08	3.29E-08	3.28E-08	3.26E-08	3.30E-08	3.35E-08	3.34E-08	3.37E-08	3.40E-08	3.45E-08
3.02E-08	3.14E-08	3.11E-08	3.08E-08	3.09E-08	3.06E-08	3.21E-08	3.24E-08	3.15E-08	3.22E-08	3.24E-08	3.31E-08	3.39E-08	3.46E-08	3.49E-08
2.95E-08	3.06E-08	3.02E-08	2.98E-08	3.00E-08	2.96E-08	3.10E-08	3.17E-08	3.10E-08	3.13E-08	3.16E-08	3.23E-08	3.35E-08	3.50E-08	3.55E-08
2.81E-08	2.90E-08	2.89E-08	2.82E-08	2.88E-08	2.87E-08	2.91E-08	3.08E-08	3.03E-08	2.99E-08	3.01E-08	3.08E-08	3.20E-08	3.44E-08	3.58E-08
2.63E-08	2.75E-08	2.79E-08	2.68E-08	2.77E-08	2.74E-08	2.77E-08	2.91E-08	2.92E-08	2.85E-08	2.82E-08	2.92E-08	3.01E-08	3.23E-08	3.46E-08
2.47E-08	2.60E-08	2.65E-08	2.59E-08	2.68E-08	2.66E-08	2.69E-08	2.75E-08	2.75E-08	2.69E-08	2.69E-08	2.74E-08	2.83E-08	2.99E-08	3.21E-08
2.32E-08	2.43E-08	2.46E-08	2.47E-08	2.56E-08	2.56E-08	2.57E-08	2.63E-08	2.58E-08	2.56E-08	2.56E-08	2.62E-08	2.67E-08	2.80E-08	2.82E-08
2.23E-08	2.30E-08	2.33E-08	2.34E-08	2.41E-08	2.46E-08	2.44E-08	2.52E-08	2.44E-08	2.46E-08	2.42E-08	2.48E-08	2.52E-08	2.64E-08	2.52E-08
2.17E-08	2.25E-08	2.23E-08	2.26E-08	2.30E-08	2.36E-08	2.29E-08	2.38E-08	2.27E-08	2.36E-08	2.27E-08	2.32E-08	2.38E-08	2.41E-08	2.32E-08
2.13E-08	2.17E-08	2.17E-08	2.17E-08	2.19E-08	2.30E-08	2.20E-08	2.25E-08	2.12E-08	2.23E-08	2.13E-08	2.15E-08	2.30E-08	2.21E-08	2.20E-08
2.10E-08	2.12E-08	2.14E-08	2.06E-08	2.08E-08	2.19E-08	2.12E-08	2.15E-08	2.00E-08	2.08E-08	2.01E-08	2.02E-08	2.16E-08	2.06E-08	2.11E-08
2.04E-08	2.10E-08	2.07E-08	1.95E-08	1.93E-08	2.01E-08	2.03E-08	2.03E-08	1.93E-08	1.94E-08	1.88E-08	1.89E-08	1.94E-08	1.97E-08	2.02E-08
1.95E-08	2.07E-08	1.97E-08	1.85E-08	1.78E-08	1.91E-08	1.97E-08	1.96E-08	1.86E-08	1.82E-08	1.78E-08	1.75E-08	1.75E-08	1.82E-08	1.82E-08
1.89E-08	1.97E-08	1.87E-08	1.76E-08	1.69E-08	1.78E-08	1.82E-08	1.86E-08	1.76E-08	1.69E-08	1.67E-08	1.65E-08	1.63E-08	1.70E-08	1.69E-08
1.77E-08	1.80E-08	1.71E-08	1.68E-08	1.58E-08	1.62E-08	1.64E-08	1.66E-08	1.66E-08	1.58E-08	1.54E-08	1.56E-08	1.56E-08	1.62E-08	1.52E-08
1.64E-08	1.62E-08	1.62E-08	1.59E-08	1.51E-08	1.52E-08	1.54E-08	1.53E-08	1.55E-08	1.52E-08	1.51E-08	1.48E-08	1.49E-08	1.51E-08	1.42E-08
1.53E-08	1.51E-08	1.53E-08	1.50E-08	1.46E-08	1.46E-08	1.45E-08	1.45E-08	1.46E-08	1.48E-08	1.46E-08	1.40E-08	1.43E-08	1.44E-08	1.35E-08
1.47E-08	1.44E-08	1.43E-08	1.40E-08	1.41E-08	1.42E-08	1.39E-08	1.35E-08	1.39E-08	1.43E-08	1.37E-08	1.33E-08	1.32E-08	1.38E-08	1.27E-08
1.36E-08	1.37E-08	1.36E-08	1.31E-08	1.32E-08	1.33E-08	1.28E-08	1.24E-08	1.29E-08	1.34E-08	1.26E-08	1.28E-08	1.20E-08	1.32E-08	1.21E-08
1.21E-08	1.24E-08	1.27E-08	1.22E-08	1.23E-08	1.23E-08	1.14E-08	1.13E-08	1.18E-08	1.19E-08	1.12E-08	1.16E-08	1.17E-08	1.30E-08	1.19E-08
1.12E-08	1.10E-08	1.11E-08	1.15E-08	1.10E-08	1.13E-08	1.03E-08	9.91E-09	9.97E-09	9.85E-09	9.55E-09	1.04E-08	1.14E-08	1.19E-08	1.15E-08
9.96E-09	1.01E-08	9.66E-09	1.11E-08	9.90E-09	1.04E-08	9.29E-09	8.44E-09	8.67E-09	8.01E-09	7.88E-09	9.47E-09	1.04E-08	1.04E-08	9.85E-09
8.70E-09	9.49E-09	8.73E-09	1.02E-08	8.78E-09	9.25E-09	8.45E-09	7.70E-09	7.35E-09	6.35E-09	6.72E-09	8.74E-09	9.07E-09	7.50E-09	8.40E-09
8.33E-09	9.15E-09	7.93E-09	8.75E-09	7.41E-09	7.44E-09	7.51E-09	7.24E-09	5.97E-09	5.25E-09	5.93E-09	8.03E-09	7.55E-09	5.78E-09	7.00E-09
8.02E-09	8.23E-09	6.90E-09	6.72E-09	5.80E-09	6.32E-09	6.38E-09	6.46E-09	5.42E-09	4.48E-09	5.49E-09	6.80E-09	6.50E-09	5.08E-09	5.42E-09
6.99E-09	7.12E-09	5.84E-09	5.44E-09	4.89E-09	5.39E-09	5.94E-09	6.16E-09	5.71E-09	4.48E-09	5.14E-09	5.49E-09	4.67E-09	4.04E-09	3.64E-09
5.44E-09	5.47E-09	4.62E-09	4.21E-09	4.38E-09	4.59E-09	5.50E-09	5.42E-09	5.43E-09	4.52E-09	4.35E-09	4.42E-09	3.45E-09	2.91E-09	2.49E-09
4.39E-09	3.82E-09	3.68E-09	3.23E-09	3.52E-09	3.44E-09	4.21E-09	4.34E-09	4.83E-09	3.67E-09	3.57E-09	3.57E-09	3.12E-09	2.01E-09	1.46E-09
4.05E-09	3.27E-09	3.31E-09	2.74E-09	2.78E-09	2.54E-09	3.01E-09	3.30E-09	3.66E-09	2.53E-09	2.53E-09	2.76E-09	3.16E-09	1.68E-09	8.25E-10
3.99E-09	2.75E-09	2.67E-09	2.22E-09	2.43E-09	1.84E-09	2.44E-09	2.55E-09	2.72E-09	1.57E-09	1.54E-09	1.81E-09	2.54E-09	1.05E-09	3.98E-10
3.09E-09	2.19E-09	1.96E-09	1.70E-09	2.28E-09	1.22E-09	2.02E-09	1.65E-09	1.86E-09	7.08E-10	7.35E-10	1.19E-09	1.42E-09	2.44E-11	-5.19E-11
2.26E-09	1.62E-09	1.75E-09	1.36E-09	1.98E-09	1.15E-09	1.92E-09	9.84E-10	1.33E-09	1.77E-10	1.27E-10	1.18E-09	8.34E-10	-5.42E-10	-8.11E-10
1.72E-09	8.76E-10	1.30E-09	1.17E-09	1.66E-09	1.41E-09	1.71E-09	5.92E-10	8.67E-10	-5.78E-10	-2.40E-10	9.39E-10	1.87E-10	-9.05E-10	-1.52E-09
1.33E-09	4.31E-10	9.28E-10	1.38E-09	1.89E-09	1.48E-09	1.35E-09	3.41E-10	8.42E-10	-5.98E-10	-7.37E-10	1.43E-10	-6.24E-10	-1.37E-09	-1.38E-09
6.29E-10	2.75E-11	4.70E-10	9.23E-10	1.21E-09	1.23E-10	5.20E-10	-8.15E-10	-3.23E-10	-1.30E-09	-1.73E-09	-1.69E-09	-1.66E-09	-2.12E-09	-1.99E-09
-2.22E-10	-2.20E-10	1.96E-10	3.55E-10	9.66E-11	-9.39E-10	-5.17E-11	-2.07E-09	-1.73E-09	-1.94E-09	-2.58E-09	-2.21E-09	-2.43E-09	-2.48E-09	-2.75E-09
-1.23E-09	-1.22E-09	-8.44E-10	-7.57E-10	-7.93E-10	-1.62E-09	-8.57E-10	-2.58E-09	-2.43E-09	-2.07E-09	-2.93E-09	-2.29E-09	-2.81E-09	-2.55E-09	-3.52E-09
-1.85E-09	-2.20E-09	-1.89E-09	-1.96E-09	-1.66E-09	-2.14E-09	-1.85E-09	-3.10E-09	-2.95E-09	-2.64E-09	-2.91E-09	-2.88E-09	-2.63E-09	-2.86E-09	-4.21E-09
-2.35E-09	-2.55E-09	-2.35E-09	-2.84E-09	-2.73E-09	-2.51E-09	-2.65E-09	-3.44E-09	-3.02E-09	-3.44E-09	-3.28E-09	-3.71E-09	-2.73E-09	-3.56E-09	-4.90E-09
-2.86E-09	-2.68E-09	-2.79E-09	-3.66E-09	-3.54E-09	-3.15E-09	-3.43E-09	-4.10E-09	-3.67E-09	-3.92E-09	-3.78E-09	-4.56E-09	-3.48E-09	-4.54E-09	-5.57E-09
-3.65E-09	-2.88E-09	-3.47E-09	-4.19E-09	-4.11E-09	-4.20E-09	-4.56E-09	-4.44E-09	-4.17E-09	-3.88E-09	-4.48E-09	-5.41E-09	-4.13E-09	-5.63E-09	-6.35E-09
-3.85E-09	-3.29E-09	-4.19E-09	-4.79E-09	-4.86E-09	-4.78E-09	-5.23E-09	-4.71E-09	-4.60E-09	-4.21E-09	-4.73E-09	-6.02E-09	-4.98E-09	-6.19E-09	-7.08E-09
-3.67E-09	-4.18E-09	-5.45E-09	-5.49E-09	-5.79E-09	-5.34E-09	-5.39E-09	-4.92E-09	-5.10E-09	-5.29E-09	-5.77E-09	-6.73E-09	-5.94E-09	-6.80E-09	-7.79E-09
-4.22E-09	-5.73E-09	-6.59E-09	-6.65E-09	-6.53E-09	-5.78E-09	-5.85E-09	-5.07E-09	-5.54E-09	-6.33E-09	-6.50E-09	-7.13E-09	-6.78E-09	-7.62E-09	-8.54E-09
-5.31E-09	-6.54E-09	-7.04E-09	-7.50E-09	-6.95E-09	-6.08E-09	-6.22E-09	-5.82E-09	-5.97E-09	-7.06E-09	-7.02E-09	-7.13E-09	-6.80E-09	-8.11E-09	-8.80E-09
-6.53E-09	-7.19E-09	-7.63E-09	-7.70E-09	-6.77E-09	-6.41E-09	-6.58E-09	-6.58E-09	-6.96E-09	-7.59E-09	-7.48E-09	-7.41E-09	-7.08E-09	-8.05E-09	-8.52E-09
-7.58E-09	-7.82E-09	-7.78E-09	-7.20E-09	-6.56E-09	-6.19E-09	-6.86E-09	-7.04E-09	-7.36E-09	-8.12E-09	-8.20E-09	-8.11E-09	-7.43E-09	-8.12E-09	-8.16E-09
-8.03E-09	-8.06E-09	-7.96E-09	-7.49E-09	-7.25E-09	-7.03E-09	-7.51E-09	-7.26E-09	-7.65E-09	-8.65E-09	-8.75E-09	-9.20E-09	-8.38E-09	-8.66E-09	-8.45E-09
-8.05E-09	-8.14E-09	-8.48E-09	-8.52E-09	-8.74E-09	-7.65E-09	-8.22E-09	-7.76E-09	-7.87E-09	-8.94E-09	-9.26E-09	-9.98E-09	-9.20E-09	-9.42E-09	-9.26E-09
-7.82E-09	-8.24E-09	-8.96E-09	-9.50E-09	-9.34E-09	-8.21E-09	-8.62E-09	-8.36E-09	-8.45E-09	-9.54E-09	-9.79E-09	-1.02E-08	-9.43E-09	-1.05E-08	-1.08E-08
-7.88E-09	-8.64E-09	-9.29E-09	-9.78E-09	-1.01E-08	-9.35E-09	-9.13E-09	-8.91E-09	-9.04E-09	-9.93E-09	-9.77E-09	-1.00E-08	-9.93E-09	-1.12E-08	-1.17E-08
-8.66E-09	-9.42E-09	-9.45E-09	-9.99E-09	-1.06E-08	-1.02E-08	-9.55E-09	-9.09E-09	-9.36E-09	-1.03E-08	-9.97E-09	-1.00E-08	-1.06E-08	-1.14E-08	-1.24E-08
-9.49E-09	-9.90E-09	-9.57E-09	-1.03E-08	-1.09E-08	-1.05E-08	-1.01E-08	-9.63E-09	-9.67E-09	-1.06E-08	-1.03E-08	-1.06E-08	-1.14E-08	-1.17E-08	-1.27E-08
-9.54E-09	-1.02E-08	-9.77E-09	-1.08E-08	-1.10E-08	-1.05E-08	-1.04E-08	-1.08E-08	-1.06E-08	-1.08E-08	-1.06E-08	-1.13E-08	-1.17E-08	-1.21E-08	-1.26E-08
-9.84E-09	-1.02E-08	-1.01E-08	-1.07E-08	-1.08E-08	-1.07E-08	-1.07E-08	-1.13E-08	-1.12E-08	-1.07E-08	-1.08E-08	-1.15E-08	-1.17E-08	-1.25E-08	-1.26E-08
-1.05E-08	-1.01E-08	-1.00E-08	-1.09E-08	-1.09E-08	-1.09E-08	-1.12E-08	-1.17E-08	-1.12E-08	-1.10E-08	-1.12E-08	-1.21E-08	-1.23E-08	-1.28E-08	-1.29E-08
-1.11E-08	-1.07E-08	-1.03E-08	-1.14E-08	-1.11E-08	-1.12E-08	-1.22E-08	-1.24E-08	-1.16E-08	-1.11E-08	-1.20E-08	-1.28E-08	-1.29E-08	-1.31E-08	-1.34E-08
-1.19E-08	-1.15E-08	-1.07E-08	-1.19E-08	-1.21E-08	-1.22E-08	-1.34E-08	-1.34E-08	-1.26E-08	-1.18E-08	-1.29E-08	-1.34E-08	-1.37E-08	-1.35E-08	-1.35E-08
-1.22E-08	-1.20E-08	-1.18E-08	-1.25E-08	-1.31E-08	-1.30E-08	-1.39E-08	-1.38E-08	-1.33E-08	-1.25E-08	-1.31E-08	-1.39E-08	-1.45E-08	-1.41E-08	-1.37E-08
-1.29E-08	-1.29E-08	-1.31E-08	-1.29E-08	-1.38E-08	-1.35E-08	-1.36E-08	-1.37E-08	-1.41E-08	-1.32E-08	-1.35E-08	-1.47E-08	-1.45E-08	-1.40E-08	-1.42E-08
-1.32E-08	-1.32E-08	-1.37E-08	-1.33E-08	-1.37E-08	-1.40E-08	-1.35E-08	-1.34E-08	-1.44E-08	-1.40E-08	-1.42E-08	-1.52E-08	-1.44E-08	-1.38E-08	-1.45E-08
-1.31E-08	-1.32E-08	-1.41E-08	-1.35E-08	-1.35E-08	-1.42E-08	-1.36E-08	-1.33E-08	-1.45E-08	-1.42E-08	-1.47E-08	-1.52E-08	-1.49E-08	-1.38E-08	-1.51E-08
-1.32E-08	-1.36E-08	-1.45E-08	-1.34E-08	-1.34E-08	-1.45E-08	-1.43E-08	-1.37E-08	-1.42E-08	-1.36E-08	-1.46E-08	-1.50E-08	-1.50E-08	-1.44E-08	-1.60E-08
-1.40E-08	-1.45E-08	-1.48E-08	-1.38E-08	-1.36E-08	-1.49E-08	-1.50E-08	-1.45E-08	-1.46E-08	-1.35E-08	-1.45E-08	-1.55E-08	-1.52E-08	-1.54E-08	-1.66E-08
-1.42E-08	-1.51E-08	-1.49E-08	-1.43E-08	-1.46E-08	-1.53E-08	-1.54E-08	-1.47E-08	-1.48E-08	-1.33E-08	-1.44E-08	-1.61E-08	-1.59E-08	-1.60E-08	-1.69E-08
-1.42E-08	-1.57E-08	-1.54E-08	-1.51E-08	-1.48E-08	-1.55E-08	-1.53E-08	-1.48E-08	-1.52E-08	-1.40E-08	-1.50E-08	-1.58E-08	-1.62E-08	-1.61E-08	-1.71E-08
-1.45E-08	-1.57E-08	-1.56E-08	-1.60E-08	-1.47E-08	-1.59E-08	-1.57E-08	-1.56E-08	-1.60E-08	-1.48E-08	-1.60E-08	-1.58E-08	-1.67E-08	-1.68E-08	-1.71E-08

-1.56E-08	-1.63E-08	-1.57E-08	-1.61E-08	-1.59E-08	-1.67E-08	-1.61E-08	-1.63E-08	-1.68E-08	-1.55E-08	-1.69E-08	-1.69E-08	-1.71E-08	-1.75E-08	-1.71E-08
-1.60E-08	-1.64E-08	-1.67E-08	-1.65E-08	-1.65E-08	-1.73E-08	-1.69E-08	-1.69E-08	-1.74E-08	-1.68E-08	-1.74E-08	-1.71E-08	-1.72E-08	-1.77E-08	-1.69E-08
-1.59E-08	-1.67E-08	-1.70E-08	-1.67E-08	-1.67E-08	-1.72E-08	-1.71E-08	-1.73E-08	-1.76E-08	-1.71E-08	-1.74E-08	-1.68E-08	-1.69E-08	-1.73E-08	-1.70E-08
-1.64E-08	-1.71E-08	-1.69E-08	-1.66E-08	-1.68E-08	-1.71E-08	-1.73E-08	-1.72E-08	-1.76E-08	-1.71E-08	-1.75E-08	-1.73E-08	-1.70E-08	-1.73E-08	-1.67E-08
-1.69E-08	-1.70E-08	-1.68E-08	-1.63E-08	-1.63E-08	-1.71E-08	-1.68E-08	-1.67E-08	-1.72E-08	-1.69E-08	-1.78E-08	-1.76E-08	-1.73E-08	-1.74E-08	-1.68E-08
-1.74E-08	-1.72E-08	-1.72E-08	-1.67E-08	-1.61E-08	-1.71E-08	-1.64E-08	-1.65E-08	-1.75E-08	-1.72E-08	-1.81E-08	-1.79E-08	-1.74E-08	-1.75E-08	-1.71E-08
-1.77E-08	-1.74E-08	-1.78E-08	-1.73E-08	-1.61E-08	-1.70E-08	-1.69E-08	-1.72E-08	-1.78E-08	-1.79E-08	-1.82E-08	-1.81E-08	-1.78E-08	-1.77E-08	-1.77E-08
-1.77E-08	-1.74E-08	-1.76E-08	-1.71E-08	-1.58E-08	-1.68E-08	-1.78E-08	-1.78E-08	-1.79E-08	-1.82E-08	-1.82E-08	-1.82E-08	-1.76E-08	-1.77E-08	-1.81E-08
-1.71E-08	-1.73E-08	-1.73E-08	-1.70E-08	-1.65E-08	-1.68E-08	-1.78E-08	-1.76E-08	-1.79E-08	-1.82E-08	-1.85E-08	-1.75E-08	-1.75E-08	-1.81E-08	-1.85E-08
-1.64E-08	-1.66E-08	-1.70E-08	-1.68E-08	-1.65E-08	-1.68E-08	-1.77E-08	-1.74E-08	-1.75E-08	-1.75E-08	-1.86E-08	-1.72E-08	-1.76E-08	-1.80E-08	-1.86E-08
-1.57E-08	-1.61E-08	-1.70E-08	-1.69E-08	-1.72E-08	-1.72E-08	-1.74E-08	-1.71E-08	-1.69E-08	-1.68E-08	-1.76E-08	-1.70E-08	-1.73E-08	-1.75E-08	-1.85E-08
-1.62E-08	-1.60E-08	-1.68E-08	-1.74E-08	-1.74E-08	-1.72E-08	-1.71E-08	-1.67E-08	-1.63E-08	-1.61E-08	-1.65E-08	-1.69E-08	-1.74E-08	-1.78E-08	-1.88E-08
-1.74E-08	-1.66E-08	-1.72E-08	-1.74E-08	-1.73E-08	-1.69E-08	-1.63E-08	-1.65E-08	-1.57E-08	-1.61E-08	-1.63E-08	-1.68E-08	-1.72E-08	-1.87E-08	-1.92E-08
-1.86E-08	-1.76E-08	-1.77E-08	-1.74E-08	-1.73E-08	-1.64E-08	-1.61E-08	-1.69E-08	-1.59E-08	-1.73E-08	-1.67E-08	-1.70E-08	-1.73E-08	-1.89E-08	-1.88E-08
-1.90E-08	-1.82E-08	-1.82E-08	-1.81E-08	-1.79E-08	-1.70E-08	-1.61E-08	-1.75E-08	-1.73E-08	-1.75E-08	-1.46E-08	-1.50E-08	-1.77E-08	-1.86E-08	-1.87E-08
-1.94E-08	-1.88E-08	-1.86E-08	-1.86E-08	-1.84E-08	-1.81E-08	-1.73E-08	-1.82E-08	-1.80E-08	-1.76E-08	-1.03E-08	-1.11E-08	-1.80E-08	-1.82E-08	-1.84E-08
-1.96E-08	-1.91E-08	-1.84E-08	-1.86E-08	-1.86E-08	-1.83E-08	-1.86E-08	-1.82E-08	-1.78E-08	-1.72E-08	-7.39E-09	-8.69E-09	-1.79E-08	-1.78E-08	-1.82E-08
-1.93E-08	-1.87E-08	-1.76E-08	-1.86E-08	-1.81E-08	-1.80E-08	-1.82E-08	-1.76E-08	-1.71E-08	-1.71E-08	-1.75E-08	-1.81E-08	-1.80E-08	-1.75E-08	-1.79E-08
-1.88E-08	-1.85E-08	-1.76E-08	-1.91E-08	-1.82E-08	-1.81E-08	-1.80E-08	-1.70E-08	-1.67E-08	-1.62E-08	-1.71E-08	-1.82E-08	-1.73E-08	-1.72E-08	-1.74E-08
-1.84E-08	-1.85E-08	-1.84E-08	-1.94E-08	-1.89E-08	-1.82E-08	-1.82E-08	-1.72E-08	-1.67E-08	-1.66E-08	-1.72E-08	-1.77E-08	-1.68E-08	-1.64E-08	-1.71E-08
-1.85E-08	-1.84E-08	-1.85E-08	-1.95E-08	-1.87E-08	-1.80E-08	-1.80E-08	-1.74E-08	-1.72E-08	-1.72E-08	-1.79E-08	-1.73E-08	-1.64E-08	-1.56E-08	-1.62E-08
-1.94E-08	-1.83E-08	-1.79E-08	-1.89E-08	-1.88E-08	-1.76E-08	-1.78E-08	-1.70E-08	-1.70E-08	-1.73E-08	-1.80E-08	-1.71E-08	-1.64E-08	-1.51E-08	-1.52E-08
-1.95E-08	-1.80E-08	-1.76E-08	-1.80E-08	-1.89E-08	-1.83E-08	-1.82E-08	-1.71E-08	-1.67E-08	-1.74E-08	-1.73E-08	-1.72E-08	-1.70E-08	-1.56E-08	-1.44E-08
-1.93E-08	-1.80E-08	-1.75E-08	-1.80E-08	-1.88E-08	-1.82E-08	-1.80E-08	-1.72E-08	-1.66E-08	-1.75E-08	-1.74E-08	-1.74E-08	-1.76E-08	-1.65E-08	-1.48E-08
-1.91E-08	-1.91E-08	-1.78E-08	-1.76E-08	-1.85E-08	-1.70E-08	-1.78E-08	-1.72E-08	-1.69E-08	-1.76E-08	-1.73E-08	-1.73E-08	-1.79E-08	-1.66E-08	-1.58E-08
-1.91E-08	-1.95E-08	-1.82E-08	-1.66E-08	-1.79E-08	-1.61E-08	-1.74E-08	-1.68E-08	-1.69E-08	-1.71E-08	-1.66E-08	-1.72E-08	-1.72E-08	-1.62E-08	-1.66E-08
-1.89E-08	-1.92E-08	-1.83E-08	-1.52E-08	-1.74E-08	-1.67E-08	-1.77E-08	-1.63E-08	-1.68E-08	-1.68E-08	-1.63E-08	-1.70E-08	-1.68E-08	-1.60E-08	-1.60E-08
-1.87E-08	-1.85E-08	-1.84E-08	-1.33E-08	-1.75E-08	-1.74E-08	-1.73E-08	-1.60E-08	-1.69E-08	-1.66E-08	-1.65E-08	-1.69E-08	-1.66E-08	-1.58E-08	-1.56E-08
-1.85E-08	-1.80E-08	-1.81E-08	-1.64E-08	-1.70E-08	-1.76E-08	-1.72E-08	-1.64E-08	-1.66E-08	-1.67E-08	-1.65E-08	-1.67E-08	-1.63E-08	-1.54E-08	-1.50E-08
-1.85E-08	-1.83E-08	-1.79E-08	-1.78E-08	-1.71E-08	-1.77E-08	-1.72E-08	-1.68E-08	-1.66E-08	-1.67E-08	-1.67E-08	-1.66E-08	-1.62E-08	-1.56E-08	-1.49E-08
-1.94E-08	-1.94E-08	-1.86E-08	-1.83E-08	-1.77E-08	-1.81E-08	-1.71E-08	-1.65E-08	-1.64E-08	-1.65E-08	-1.61E-08	-1.62E-08	-1.63E-08	-1.59E-08	-1.49E-08
-2.01E-08	-2.03E-08	-1.90E-08	-1.85E-08	-1.77E-08	-1.83E-08	-1.68E-08	-1.62E-08	-1.63E-08	-1.62E-08	-1.53E-08	-1.65E-08	-1.62E-08	-1.55E-08	-1.47E-08
-2.01E-08	-2.03E-08	-1.90E-08	-1.81E-08	-1.74E-08	-1.80E-08	-1.65E-08	-1.64E-08	-1.61E-08	-1.58E-08	-1.51E-08	-1.67E-08	-1.58E-08	-1.46E-08	-1.42E-08
-1.95E-08	-1.99E-08	-1.90E-08	-1.77E-08	-1.71E-08	-1.77E-08	-1.64E-08	-1.65E-08	-1.59E-08	-1.60E-08	-1.58E-08	-1.63E-08	-1.56E-08	-1.40E-08	-1.32E-08
-1.83E-08	-1.95E-08	-1.86E-08	-1.75E-08	-1.69E-08	-1.77E-08	-1.63E-08	-1.62E-08	-1.55E-08	-1.58E-08	-1.57E-08	-1.60E-08	-1.51E-08	-1.32E-08	-1.28E-08
-1.72E-08	-1.89E-08	-1.81E-08	-1.71E-08	-1.66E-08	-1.71E-08	-1.61E-08	-1.61E-08	-1.52E-08	-1.56E-08	-1.56E-08	-1.52E-08	-1.44E-08	-1.27E-08	-1.28E-08
-1.59E-08	-1.82E-08	-1.73E-08	-1.65E-08	-1.62E-08	-1.64E-08	-1.58E-08	-1.58E-08	-1.51E-08	-1.57E-08	-1.50E-08	-1.44E-08	-1.40E-08	-1.29E-08	-1.29E-08
-1.39E-08	-1.78E-08	-1.68E-08	-1.60E-08	-1.60E-08	-1.61E-08	-1.55E-08	-1.53E-08	-1.52E-08	-1.51E-08	-1.46E-08	-1.44E-08	-1.42E-08	-1.35E-08	-1.34E-08
-1.71E-08	-1.77E-08	-1.69E-08	-1.57E-08	-1.59E-08	-1.56E-08	-1.50E-08	-1.51E-08	-1.52E-08	-1.47E-08	-1.45E-08	-1.45E-08	-1.42E-08	-1.39E-08	-1.39E-08
-1.75E-08	-1.80E-08	-1.67E-08	-1.56E-08	-1.57E-08	-1.46E-08	-1.44E-08	-1.51E-08	-1.56E-08	-1.44E-08	-1.46E-08	-1.47E-08	-1.40E-08	-1.39E-08	-1.43E-08
-1.79E-08	-1.86E-08	-1.72E-08	-1.62E-08	-1.54E-08	-1.39E-08	-1.45E-08	-1.52E-08	-1.53E-08	-1.37E-08	-1.42E-08	-1.45E-08	-1.35E-08	-1.34E-08	-1.42E-08
-1.81E-08	-1.85E-08	-1.76E-08	-1.65E-08	-1.52E-08	-1.43E-08	-1.46E-08	-1.47E-08	-1.44E-08	-1.34E-08	-1.35E-08	-1.38E-08	-1.31E-08	-1.34E-08	-1.40E-08
-1.79E-08	-1.81E-08	-1.74E-08	-1.63E-08	-1.51E-08	-1.41E-08	-1.44E-08	-1.47E-08	-1.40E-08	-1.33E-08	-1.29E-08	-1.34E-08	-1.32E-08	-1.36E-08	-1.34E-08
-1.77E-08	-1.78E-08	-1.71E-08	-1.63E-08	-1.51E-08	-1.45E-08	-1.47E-08	-1.46E-08	-1.41E-08	-1.37E-08	-1.30E-08	-1.33E-08	-1.35E-08	-1.36E-08	-1.31E-08
-1.70E-08	-1.73E-08	-1.68E-08	-1.61E-08	-1.54E-08	-1.49E-08	-1.41E-08	-1.45E-08	-1.45E-08	-1.45E-08	-1.35E-08	-1.34E-08	-1.36E-08	-1.38E-08	-1.28E-08
-1.66E-08	-1.67E-08	-1.66E-08	-1.59E-08	-1.52E-08	-1.49E-08	-1.36E-08	-1.42E-08	-1.46E-08	-1.45E-08	-1.36E-08	-1.33E-08	-1.34E-08	-1.36E-08	-1.29E-08
-1.65E-08	-1.65E-08	-1.62E-08	-1.60E-08	-1.47E-08	-1.44E-08	-1.38E-08	-1.38E-08	-1.42E-08	-1.42E-08	-1.37E-08	-1.34E-08	-1.33E-08	-1.34E-08	-1.24E-08
-1.67E-08	-1.64E-08	-1.61E-08	-1.57E-08	-1.42E-08	-1.37E-08	-1.34E-08	-1.37E-08	-1.44E-08	-1.39E-08	-1.37E-08	-1.34E-08	-1.31E-08	-1.26E-08	-1.15E-08
-1.69E-08	-1.65E-08	-1.60E-08	-1.53E-08	-1.39E-08	-1.39E-08	-1.39E-08	-1.41E-08	-1.42E-08	-1.37E-08	-1.33E-08	-1.32E-08	-1.22E-08	-1.14E-08	-1.04E-08
-1.70E-08	-1.65E-08	-1.56E-08	-1.48E-08	-1.43E-08	-1.42E-08	-1.45E-08	-1.42E-08	-1.38E-08	-1.34E-08	-1.28E-08	-1.30E-08	-1.19E-08	-1.12E-08	-9.61E-09

2.10E-15	2.01E-15	1.95E-15	2.02E-15	2.04E-15	1.96E-15	1.98E-15	1.97E-15	2.02E-15	1.97E-15	2.01E-15	2.02E-15	1.83E-15	1.88E-15	2.00E-15
1.99E-15	1.92E-15	1.89E-15	1.94E-15	1.97E-15	1.90E-15	1.85E-15	1.82E-15	1.92E-15	1.89E-15	1.87E-15	1.86E-15	1.71E-15	1.77E-15	1.93E-15
1.87E-15	1.84E-15	1.84E-15	1.84E-15	1.83E-15	1.80E-15	1.76E-15	1.72E-15	1.81E-15	1.79E-15	1.75E-15	1.73E-15	1.61E-15	1.65E-15	1.77E-15
1.77E-15	1.75E-15	1.72E-15	1.73E-15	1.69E-15	1.70E-15	1.67E-15	1.65E-15	1.69E-15	1.67E-15	1.67E-15	1.66E-15	1.57E-15	1.57E-15	1.64E-15
1.68E-15	1.64E-15	1.60E-15	1.58E-15	1.56E-15	1.59E-15	1.57E-15	1.56E-15	1.58E-15	1.55E-15	1.61E-15	1.62E-15	1.56E-15	1.49E-15	1.56E-15
1.58E-15	1.54E-15	1.47E-15	1.48E-15	1.47E-15	1.47E-15	1.46E-15	1.47E-15	1.50E-15	1.46E-15	1.53E-15	1.54E-15	1.51E-15	1.40E-15	1.46E-15
1.49E-15	1.45E-15	1.37E-15	1.39E-15	1.42E-15	1.38E-15	1.35E-15	1.36E-15	1.41E-15	1.43E-15	1.42E-15	1.41E-15	1.39E-15	1.30E-15	1.33E-15
1.40E-15	1.36E-15	1.30E-15	1.32E-15	1.38E-15	1.33E-15	1.30E-15	1.32E-15	1.33E-15	1.37E-15	1.32E-15	1.30E-15	1.33E-15	1.25E-15	1.28E-15
1.29E-15	1.26E-15	1.23E-15	1.26E-15	1.31E-15	1.27E-15	1.28E-15	1.27E-15	1.24E-15	1.27E-15	1.27E-15	1.22E-15	1.26E-15	1.21E-15	1.23E-15
1.18E-15	1.17E-15	1.14E-15	1.16E-15	1.20E-15	1.22E-15	1.26E-15	1.19E-15	1.14E-15	1.22E-15	1.26E-15	1.19E-15	1.18E-15	1.16E-15	1.18E-15
1.11E-15	1.09E-15	1.07E-15	1.09E-15	1.11E-15	1.14E-15	1.16E-15	1.13E-15	1.09E-15	1.17E-15	1.20E-15	1.16E-15	1.13E-15	1.16E-15	1.17E-15
9.90E-16	1.04E-15	1.01E-15	1.01E-15	1.03E-15	1.02E-15	1.08E-15	1.07E-15	1.06E-15	1.09E-15	1.12E-15	1.11E-15	1.14E-15	1.15E-15	1.19E-15
9.10E-16	9.87E-16	9.68E-16	9.50E-16	9.54E-16	9.38E-16	1.03E-15	1.05E-15	9.91E-16	1.04E-15	1.05E-15	1.10E-15	1.15E-15	1.20E-15	1.22E-15
8.68E-16	9.38E-16	9.13E-16	8.90E-16	9.00E-16	8.74E-16	9.62E-16	1.00E-15	9.59E-16	9.78E-16	9.99E-16	1.04E-15	1.12E-15	1.23E-15	1.26E-15
7.92E-16	8.40E-16	8.36E-16	7.96E-16	8.31E-16	8.22E-16	8.44E-16	9.47E-16	9.20E-16	8.92E-16	9.04E-16	9.46E-16	1.03E-15	1.19E-15	1.28E-15
6.93E-16	7.58E-16	7.78E-16	7.18E-16	7.65E-16	7.53E-16	7.70E-16	8.46E-16	8.52E-16	8.15E-16	7.96E-16	8.50E-16	9.08E-16	1.05E-15	1.20E-15
6.09E-16	6.76E-16	7.03E-16	6.71E-16	7.17E-16	7.07E-16	7.24E-16	7.56E-16	7.56E-16	7.23E-16	7.22E-16	7.53E-16	7.99E-16	8.93E-16	1.03E-15
5.38E-16	5.89E-16	6.06E-16	6.11E-16	6.55E-16	6.54E-16	6.60E-16	6.92E-16	6.66E-16	6.56E-16	6.57E-16	6.88E-16	7.14E-16	7.85E-16	7.93E-16
4.97E-16	5.31E-16	5.42E-16	5.49E-16	5.82E-16	6.06E-16	5.94E-16	6.36E-16	5.97E-16	6.05E-16	5.86E-16	6.15E-16	6.37E-16	7.00E-16	6.35E-16
4.73E-16	5.04E-16	4.97E-16	5.09E-16	5.27E-16	5.58E-16	5.23E-16	5.65E-16	5.16E-16	5.59E-16	5.16E-16	5.37E-16	5.68E-16	5.81E-16	5.40E-16
4.54E-16	4.70E-16	4.72E-16	4.70E-16	4.80E-16	5.27E-16	4.83E-16	5.04E-16	4.49E-16	4.97E-16	4.52E-16	4.62E-16	5.29E-16	4.90E-16	4.82E-16
4.41E-16	4.49E-16	4.58E-16	4.23E-16	4.33E-16	4.80E-16	4.48E-16	4.62E-16	4.02E-16	4.34E-16	4.03E-16	4.06E-16	4.65E-16	4.25E-16	4.46E-16
4.17E-16	4.41E-16	4.29E-16	3.82E-16	3.72E-16	4.04E-16	4.13E-16	4.13E-16	3.72E-16	3.76E-16	3.55E-16	3.58E-16	3.78E-16	3.86E-16	4.07E-16
3.79E-16	4.27E-16	3.89E-16	3.41E-16	3.17E-16	3.65E-16	3.87E-16	3.85E-16	3.45E-16	3.30E-16	3.16E-16	3.08E-16	3.07E-16	3.33E-16	3.32E-16
3.56E-16	3.89E-16	3.49E-16	3.08E-16	2.85E-16	3.15E-16	3.30E-16	3.44E-16	3.10E-16	2.86E-16	2.78E-16	2.74E-16	2.67E-16	2.89E-16	2.84E-16
3.14E-16	3.24E-16	2.93E-16	2.81E-16	2.49E-16	2.62E-16	2.69E-16	2.75E-16	2.75E-16	2.49E-16	2.38E-16	2.43E-16	2.43E-16	2.62E-16	2.31E-16
2.68E-16	2.64E-16	2.64E-16	2.51E-16	2.27E-16	2.30E-16	2.38E-16	2.35E-16	2.41E-16	2.30E-16	2.28E-16	2.18E-16	2.23E-16	2.27E-16	2.02E-16
2.36E-16	2.28E-16	2.34E-16	2.24E-16	2.13E-16	2.12E-16	2.10E-16	2.09E-16	2.14E-16	2.19E-16	2.12E-16	1.97E-16	2.05E-16	2.07E-16	1.83E-16
2.16E-16	2.06E-16	2.05E-16	1.97E-16	2.00E-16	2.01E-16	1.94E-16	1.81E-16	1.93E-16	2.05E-16	1.88E-16	1.78E-16	1.73E-16	1.90E-16	1.62E-16
1.86E-16	1.86E-16	1.86E-16	1.73E-16	1.75E-16	1.77E-16	1.65E-16	1.54E-16	1.67E-16	1.79E-16	1.59E-16	1.63E-16	1.44E-16	1.74E-16	1.47E-16
1.46E-16	1.55E-16	1.62E-16	1.49E-16	1.50E-16	1.52E-16	1.29E-16	1.28E-16	1.38E-16	1.42E-16	1.25E-16	1.35E-16	1.36E-16	1.70E-16	1.41E-16

1.26E-16	1.22E-16	1.24E-16	1.33E-16	1.21E-16	1.29E-16	1.06E-16	9.82E-17	9.94E-17	9.71E-17	9.12E-17	1.08E-16	1.30E-16	1.42E-16	1.31E-16
9.93E-17	1.03E-16	9.33E-17	1.23E-16	9.79E-17	1.08E-16	8.63E-17	7.12E-17	7.52E-17	6.42E-17	6.22E-17	8.98E-17	1.08E-16	1.09E-16	9.71E-17
7.56E-17	9.01E-17	7.61E-17	1.04E-16	7.71E-17	8.56E-17	7.14E-17	5.92E-17	5.40E-17	4.03E-17	4.51E-17	7.64E-17	8.23E-17	5.63E-17	7.06E-17
6.93E-17	8.37E-17	6.30E-17	7.66E-17	5.50E-17	5.53E-17	5.64E-17	5.24E-17	3.57E-17	2.75E-17	3.52E-17	6.45E-17	5.70E-17	3.34E-17	4.90E-17
6.42E-17	6.77E-17	4.76E-17	4.52E-17	3.36E-17	3.99E-17	4.07E-17	4.17E-17	2.94E-17	2.01E-17	3.01E-17	4.62E-17	4.23E-17	2.58E-17	2.94E-17
4.89E-17	5.06E-17	3.42E-17	2.96E-17	2.39E-17	2.91E-17	3.53E-17	3.79E-17	3.26E-17	2.01E-17	2.65E-17	3.01E-17	2.18E-17	1.64E-17	1.32E-17
2.96E-17	3.00E-17	2.14E-17	1.78E-17	1.92E-17	2.11E-17	3.02E-17	2.94E-17	2.95E-17	2.04E-17	1.89E-17	1.95E-17	1.19E-17	8.47E-18	6.19E-18
1.93E-17	1.46E-17	1.36E-17	1.05E-17	1.24E-17	1.19E-17	1.77E-17	1.88E-17	2.33E-17	1.35E-17	1.28E-17	1.27E-17	9.73E-18	4.05E-18	2.13E-18
1.64E-17	1.07E-17	1.10E-17	7.50E-18	7.75E-18	6.46E-18	9.08E-18	1.09E-17	1.34E-17	6.38E-18	6.41E-18	7.62E-18	1.00E-17	2.81E-18	6.80E-19
1.59E-17	7.55E-18	7.15E-18	4.94E-18	5.88E-18	3.40E-18	5.94E-18	6.52E-18	7.39E-18	2.46E-18	2.38E-18	3.28E-18	6.43E-18	1.09E-18	1.58E-19
9.53E-18	4.79E-18	3.86E-18	2.88E-18	5.20E-18	1.48E-18	4.08E-18	2.72E-18	3.46E-18	5.01E-19	5.40E-19	1.42E-18	2.01E-18	5.93E-22	2.69E-21
5.13E-18	2.61E-18	3.06E-18	1.85E-18	3.93E-18	1.33E-18	3.69E-18	9.68E-19	1.78E-18	3.13E-20	1.61E-20	1.39E-18	6.96E-19	2.94E-19	6.58E-19
2.96E-18	7.68E-19	1.68E-18	1.36E-18	2.77E-18	1.98E-18	2.91E-18	3.50E-19	7.51E-19	3.35E-19	5.78E-20	8.82E-19	3.48E-20	8.19E-19	2.30E-18
1.77E-18	1.85E-19	8.62E-19	1.91E-18	3.56E-18	2.20E-18	1.82E-18	1.16E-19	7.08E-19	3.58E-19	5.43E-19	2.05E-20	3.90E-19	1.88E-18	1.89E-18
3.96E-19	7.57E-22	2.21E-19	8.52E-19	1.46E-18	1.51E-20	2.70E-19	6.64E-19	1.04E-19	1.68E-18	2.98E-18	2.86E-18	2.77E-18	4.51E-18	3.97E-18
4.94E-20	4.84E-20	3.83E-20	1.26E-19	9.33E-21	8.83E-19	2.67E-21	4.27E-18	2.98E-18	3.78E-18	6.68E-18	4.88E-18	5.90E-18	6.16E-18	7.57E-18
1.51E-18	1.48E-18	7.13E-19	5.73E-19	6.29E-19	2.63E-18	7.35E-19	6.68E-18	5.89E-18	4.29E-18	8.58E-18	5.25E-18	7.92E-18	6.52E-18	1.24E-17
3.44E-18	4.82E-18	3.56E-18	3.85E-18	2.77E-18	4.59E-18	3.42E-18	9.61E-18	8.71E-18	6.96E-18	8.49E-18	8.27E-18	6.92E-18	8.15E-18	1.77E-17
5.52E-18	6.49E-18	5.52E-18	8.09E-18	7.47E-18	6.28E-18	7.01E-18	1.18E-17	9.12E-18	1.18E-17	1.08E-17	1.38E-17	7.43E-18	1.27E-17	2.40E-17
8.20E-18	7.17E-18	7.81E-18	1.34E-17	1.25E-17	9.90E-18	1.18E-17	1.68E-17	1.35E-17	1.54E-17	1.43E-17	2.08E-17	1.21E-17	2.06E-17	3.11E-17
1.33E-17	8.30E-18	1.20E-17	1.76E-17	1.69E-17	1.76E-17	2.08E-17	1.97E-17	1.74E-17	1.51E-17	2.00E-17	2.93E-17	1.71E-17	3.17E-17	4.03E-17
1.49E-17	1.08E-17	1.76E-17	2.29E-17	2.37E-17	2.28E-17	2.73E-17	2.21E-17	2.12E-17	1.77E-17	2.24E-17	3.62E-17	2.48E-17	3.83E-17	5.02E-17
1.35E-17	1.75E-17	2.98E-17	3.02E-17	3.35E-17	2.85E-17	2.91E-17	2.43E-17	2.60E-17	2.79E-17	3.33E-17	4.53E-17	3.53E-17	4.62E-17	6.07E-17
1.78E-17	3.29E-17	4.34E-17	4.42E-17	4.27E-17	3.34E-17	3.42E-17	2.58E-17	3.06E-17	4.01E-17	4.22E-17	5.08E-17	4.59E-17	5.81E-17	7.29E-17
2.82E-17	4.27E-17	4.96E-17	5.62E-17	4.84E-17	3.70E-17	3.87E-17	3.39E-17	3.56E-17	4.99E-17	4.93E-17	5.09E-17	4.62E-17	6.58E-17	7.75E-17
4.26E-17	5.17E-17	5.82E-17	5.92E-17	4.58E-17	4.11E-17	4.33E-17	4.34E-17	4.85E-17	5.76E-17	5.59E-17	5.48E-17	5.01E-17	6.48E-17	7.25E-17
5.75E-17	6.11E-17	6.06E-17	5.18E-17	4.31E-17	3.83E-17	4.70E-17	4.96E-17	5.41E-17	6.59E-17	6.73E-17	6.58E-17	5.51E-17	6.60E-17	6.66E-17
6.45E-17	6.50E-17	6.33E-17	5.61E-17	5.26E-17	4.95E-17	5.65E-17	5.27E-17	5.85E-17	7.48E-17	7.66E-17	8.47E-17	7.02E-17	7.50E-17	7.14E-17
6.48E-17	6.63E-17	7.19E-17	7.27E-17	7.64E-17	5.86E-17	6.75E-17	6.02E-17	6.20E-17	7.99E-17	8.57E-17	9.97E-17	8.46E-17	8.88E-17	8.57E-17
6.11E-17	6.79E-17	8.03E-17	9.03E-17	8.72E-17	6.73E-17	7.42E-17	7.00E-17	7.14E-17	9.10E-17	9.59E-17	1.05E-16	8.90E-17	1.09E-16	1.17E-16
6.21E-17	7.46E-17	8.63E-17	9.57E-17	1.01E-16	8.74E-17	8.33E-17	7.94E-17	8.18E-17	9.87E-17	9.54E-17	1.00E-16	9.87E-17	1.25E-16	1.38E-16
7.50E-17	8.87E-17	8.93E-17	9.97E-17	1.11E-16	1.03E-16	9.11E-17	8.27E-17	8.76E-17	1.06E-16	9.95E-17	9.99E-17	1.13E-16	1.31E-16	1.55E-16
9.01E-17	9.81E-17	9.15E-17	1.05E-16	1.18E-16	1.10E-16	1.03E-16	9.28E-17	9.35E-17	1.12E-16	1.05E-16	1.12E-16	1.29E-16	1.36E-16	1.61E-16
9.10E-17	1.04E-16	9.55E-17	1.16E-16	1.21E-16	1.11E-16	1.09E-16	1.16E-16	1.12E-16	1.16E-16	1.13E-16	1.28E-16	1.38E-16	1.47E-16	1.60E-16
9.68E-17	1.05E-16	1.02E-16	1.14E-16	1.17E-16	1.13E-16	1.14E-16	1.28E-16	1.26E-16	1.15E-16	1.16E-16	1.33E-16	1.37E-16	1.57E-16	1.58E-16
1.11E-16	1.02E-16	1.01E-16	1.19E-16	1.19E-16	1.19E-16	1.25E-16	1.37E-16	1.26E-16	1.20E-16	1.25E-16	1.47E-16	1.50E-16	1.64E-16	1.67E-16
1.23E-16	1.14E-16	1.07E-16	1.29E-16	1.23E-16	1.25E-16	1.50E-16	1.53E-16	1.35E-16	1.24E-16	1.45E-16	1.65E-16	1.67E-16	1.72E-16	1.80E-16
1.42E-16	1.32E-16	1.14E-16	1.41E-16	1.47E-16	1.49E-16	1.80E-16	1.80E-16	1.59E-16	1.39E-16	1.68E-16	1.80E-16	1.89E-16	1.83E-16	1.81E-16
1.50E-16	1.45E-16	1.39E-16	1.56E-16	1.72E-16	1.70E-16	1.94E-16	1.91E-16	1.76E-16	1.56E-16	1.71E-16	1.92E-16	2.10E-16	1.98E-16	1.87E-16
1.67E-16	1.67E-16	1.72E-16	1.67E-16	1.90E-16	1.84E-16	1.86E-16	1.88E-16	1.99E-16	1.74E-16	1.81E-16	2.16E-16	2.11E-16	1.95E-16	2.01E-16
1.75E-16	1.73E-16	1.87E-16	1.78E-16	1.87E-16	1.96E-16	1.83E-16	1.80E-16	2.08E-16	1.96E-16	2.01E-16	2.31E-16	2.08E-16	1.90E-16	2.10E-16
1.72E-16	1.74E-16	2.00E-16	1.82E-16	1.82E-16	2.00E-16	1.86E-16	1.76E-16	2.09E-16	2.02E-16	2.15E-16	2.30E-16	2.21E-16	1.91E-16	2.27E-16
1.75E-16	1.86E-16	2.11E-16	1.81E-16	1.80E-16	2.11E-16	2.05E-16	1.89E-16	2.03E-16	1.84E-16	2.14E-16	2.24E-16	2.26E-16	2.08E-16	2.55E-16
1.95E-16	2.09E-16	2.20E-16	1.90E-16	1.85E-16	2.22E-16	2.26E-16	2.10E-16	2.14E-16	1.83E-16	2.09E-16	2.40E-16	2.30E-16	2.36E-16	2.74E-16
2.02E-16	2.28E-16	2.22E-16	2.03E-16	2.14E-16	2.33E-16	2.36E-16	2.17E-16	2.19E-16	1.76E-16	2.06E-16	2.59E-16	2.52E-16	2.57E-16	2.86E-16
2.03E-16	2.46E-16	2.36E-16	2.27E-16	2.18E-16	2.40E-16	2.35E-16	2.18E-16	2.31E-16	1.96E-16	2.26E-16	2.48E-16	2.61E-16	2.59E-16	2.93E-16
2.11E-16	2.48E-16	2.44E-16	2.55E-16	2.15E-16	2.53E-16	2.45E-16	2.42E-16	2.57E-16	2.20E-16	2.55E-16	2.51E-16	2.80E-16	2.82E-16	2.91E-16
2.44E-16	2.64E-16	2.47E-16	2.58E-16	2.52E-16	2.79E-16	2.60E-16	2.67E-16	2.82E-16	2.42E-16	2.84E-16	2.85E-16	2.92E-16	3.05E-16	2.92E-16
2.56E-16	2.69E-16	2.78E-16	2.71E-16	2.73E-16	3.00E-16	2.84E-16	2.86E-16	3.04E-16	2.82E-16	3.03E-16	2.91E-16	2.95E-16	3.13E-16	2.87E-16
2.52E-16	2.80E-16	2.89E-16	2.81E-16	2.79E-16	2.97E-16	2.91E-16	3.00E-16	3.10E-16	2.92E-16	3.04E-16	2.84E-16	2.85E-16	3.01E-16	2.87E-16
2.69E-16	2.91E-16	2.85E-16	2.76E-16	2.82E-16	2.94E-16	2.98E-16	2.95E-16	3.09E-16	2.92E-16	3.07E-16	3.00E-16	2.90E-16	3.01E-16	2.80E-16
2.86E-16	2.90E-16	2.81E-16	2.65E-16	2.66E-16	2.91E-16	2.83E-16	2.80E-16	2.96E-16	2.84E-16	3.16E-16	3.10E-16	3.00E-16	3.03E-16	2.81E-16
3.01E-16	2.96E-16	2.97E-16	2.80E-16	2.60E-16	2.92E-16	2.69E-16	2.74E-16	3.05E-16	2.94E-16	3.28E-16	3.21E-16	3.04E-16	3.07E-16	2.93E-16
3.15E-16	3.03E-16	3.17E-16	2.98E-16	2.58E-16	2.89E-16	2.84E-16	2.95E-16	3.18E-16	3.22E-16	3.31E-16	3.27E-16	3.16E-16	3.14E-16	3.14E-16
3.13E-16	3.02E-16	3.10E-16	2.92E-16	2.50E-16	2.84E-16	3.18E-16	3.16E-16	3.19E-16	3.32E-16	3.33E-16	3.30E-16	3.11E-16	3.12E-16	3.29E-16
2.91E-16	2.99E-16	2.99E-16	2.88E-16	2.72E-16	2.83E-16	3.15E-16	3.11E-16	3.20E-16	3.31E-16	3.41E-16	3.05E-16	3.08E-16	3.27E-16	3.43E-16
2.70E-16	2.76E-16	2.88E-16	2.82E-16	2.73E-16	2.82E-16	3.14E-16	3.04E-16	3.06E-16	3.06E-16	3.46E-16	2.96E-16	3.08E-16	3.24E-16	3.47E-16
2.46E-16	2.60E-16	2.88E-16	2.85E-16	2.95E-16	2.95E-16	3.04E-16	2.94E-16	2.87E-16	2.82E-16	3.08E-16	2.90E-16	3.00E-16	3.07E-16	3.42E-16
2.62E-16	2.57E-16	2.82E-16	3.02E-16	3.03E-16	2.96E-16	2.91E-16	2.79E-16	2.66E-16	2.60E-16	2.71E-16	2.84E-16	3.01E-16	3.16E-16	3.53E-16
3.04E-16	2.76E-16	2.95E-16	3.04E-16	3.00E-16	2.84E-16	2.67E-16	2.73E-16	2.47E-16	2.60E-16	2.66E-16	2.83E-16	2.96E-16	3.50E-16	3.68E-16
3.46E-16	3.11E-16	3.12E-16	3.02E-16	2.98E-16	2.70E-16	2.58E-16	2.86E-16	2.53E-16	2.98E-16	2.80E-16	2.90E-16	3.00E-16	3.58E-16	3.52E-16
3.61E-16	3.32E-16	3.33E-16	3.27E-16	3.21E-16	2.91E-16	2.60E-16	3.05E-16	2.98E-16	3.05E-16	2.13E-16	2.24E-16	3.15E-16	3.46E-16	3.50E-16
3.76E-16	3.54E-16	3.44E-16	3.47E-16	3.40E-16	3.28E-16	2.99E-16	3.31E-16	3.23E-16	3.09E-16	1.05E-16	1.23E-16	3.25E-16	3.30E-16	3.40E-16
3.83E-16	3.66E-16	3.37E-16	3.45E-16	3.46E-16	3.36E-16	3.44E-16	3.32E-16	3.15E-16	2.97E-16	5.46E-17	7.55E-17	3.22E-16	3.18E-16	3.31E-16
3.72E-16	3.48E-16	3.09E-16	3.47E-16	3.28E-16	3.25E-16	3.31E-16	3.09E-16	2.93E-16	2.91E-16	3.08E-16	3.27E-16	3.24E-16	3.06E-16	3.20E-16
3.53E-16	3.41E-16	3.10E-16	3.65E-16	3.33E-16	3.28E-16	3.23E-16	2.91E-16	2.80E-16	2.63E-16	2.92E-16	3.31E-16	2.99E-16	2.95E-16	3.04E-16
3.38E-16	3.43E-16	3.39E-16	3.78E-16	3.58E-16	3.31E-16	3.33E-16	2.96E-16	2.80E-16	2.76E-16	2.94E-16	3.15E-16	2.83E-16	2.69E-16	2.93E-16
3.43E-16	3.37E-16	3.42E-16	3.82E-16	3.51E-16	3.24E-16	3.24E-16	3.02E-16	2.97E-16	2.97E-16	3.20E-16	2.99E-16	2.67E-16	2.42E-16	2.63E-16
3.76E-16	3.36E-16	3.20E-16	3.56E-16	3.54E-16	3.11E-16	3.19E-16	2.89E-16	2.89E-16	3.00E-16	3.23E-16	2.91E-16	2.71E-16	2.27E-16	2.31E-16
3.81E-16	3.22E-16	3.10E-16	3.23E-16	3.56E-16	3.34E-16	3.32E-16	2.91E-16	2.79E-16	3.03E-16	3.01E-16	2.97E-16	2.88E-16	2.43E-16	2.07E-16
3.73E-16	3.25E-16	3.06E-16	3.24E-16	3.55E-16	3.30E-16	3.25E-16	2.97E-16	2.76E-16	3.08E-16	3.02E-16	3.03E-16	3.08E-16	2.71E-16	2.20E-16
3.64E-16	3.67E-16	3.16E-16	3.08E-16	3.42E-16	2.90E-16	3.17E-16	2.96E-16	2.85E-16	3.08E-16	2.98E-16	3.01E-16	3.19E-16	2.74E-16	2.51E-16
3.64E-16	3.81E-16	3.29E-16	2.77E-16	3.21E-16	2.60E-16	3.03E-16	2.82E-16	2.85E-16	2.92E-16	2.76E-16	2.94E-16	2.96E-16	2.63E-16	2.74E-16
3.57E-16	3.69E-16	3.37E-16	2.32E-16	3.01E-16	2.77E-16	3.12E-16	2.66E-16	2.83E-16	2.83E-16	2.67E-16	2.87E-16	2.81E-16	2.55E-16	2.57E-16
3.49E-16	3.43E-16	3.38E-16	1.77E-16	3.06E-16	3.04E-16	3.01E-16	2.55E-16	2.85E-16	2.76E-16	2.74E-16	2.85E-16	2.76E-16	2.49E-16	2.44E-16
3.43E-16	3.25E-16	3.27E-16	2.70E-16	2.89E-16	3.08E-16	2.96E-16	2.69E-16	2.77E-16	2.79E-16	2.73E-16	2.79E-16	2.66E-16	2.37E-16	2.25E-16
3.43E-16	3.36E-16	3.22E-16	3.16E-16	2.93E-16	3.12E-16	2.97E-16	2.84E-16	2.74E-16	2.78E-16	2.81E-16	2.75E-16	2.61E-16	2.42E-16	2.21E-16
3.75E-16	3.77E-16	3.45E-16	3.34E-16	3.12E-16	3.29E-16	2.94E-16	2.72E-16	2.68E-16	2.71E-16	2.59E-16	2.63E-16	2.65E-16	2.53E-16	2.23E-16
4.03E-16	4.13E-16	3.60E-16	3.42E-16	3.12E-16	3.34E-16	2.83E-16	2.64E-16	2.67E-16	2.63E-16	2.33E-16	2.72E-16	2.63E-16	2.40E-16	2.16E-16
4.05E-16	4.14E-16	3.61E-16	3.28E-16	3.04E-16	3.25E-16	2.71E-16	2.69E-16	2.59E-16	2.50E-16	2.29E-16	2.78E-16	2.50E-16	2.14E-16	2.02E-16
3.79E-16	3.95E-16	3.61E-16	3.14E-16	2.94E-16	3.13E-16	2.69E-16	2.73E-16	2.52E-16	2.55E-16	2.51E-16	2.65E-16	2.42E-16	1.97E-16	1.75E-16
3.33E-16	3.82E-16	3.46E-16	3.05E-16	2.86E-16	3.12E-16	2.66E-16	2.61E-16	2.42E-16	2.50E-16	2.47E-16	2.55E-16	2.28E-16	1.73E-16	1.65E-16
2.94E-16	3.59E-16	3.27E-16	2.92E-16	2.76E-16	2.94E-16	2.58E-16	2.60E-16	2.30E-16	2.43E-16	2.43E-16	2.31E-16	2.08E-16	1.61E-16	1.63E-16
2.53E-16	3.30E-16	2.99E-16	2.71E-16	2.61E-16	2.69E-16	2.50E-16	2.48E-16	2.29E-16	2.45E-16	2.25E-16	2.08E-16	1.96E-16	1.68E-16	1.66E-16
1.93E-16	3.15E-16	2.82E-16	2.56E-16	2.57E-16	2.60E-16	2.40E-16	2.33E-16	2.32E-16	2.29E-16	2.14E-16	2.07E-16	2.01E-16	1.82E-16	1.79E-16
2.94E-16	3.12E-16	2.86E-16	2.47E-16	2.54E-16	2.44E-16	2.24E-16	2.28E-16	2.30E-16	2.15E-16	2.11E-16	2.11E-16	2.01E-16	1.93E-16	1.93E-16

3.07E-16	3.25E-16	2.80E-16	2.45E-16	2.46E-16	2.12E-16	2.08E-16	2.27E-16	2.42E-16	2.07E-16	2.12E-16	2.15E-16	1.96E-16	1.94E-16	2.03E-16
3.19E-16	3.46E-16	2.96E-16	2.61E-16	2.37E-16	1.94E-16	2.11E-16	2.30E-16	2.33E-16	1.87E-16	2.01E-16	2.09E-16	1.83E-16	1.81E-16	2.03E-16
3.27E-16	3.42E-16	3.09E-16	2.72E-16	2.30E-16	2.05E-16	2.12E-16	2.16E-16	2.08E-16	1.81E-16	1.81E-16	1.92E-16	1.70E-16	1.80E-16	1.95E-16
3.21E-16	3.29E-16	3.02E-16	2.67E-16	2.27E-16	1.99E-16	2.09E-16	2.16E-16	1.96E-16	1.76E-16	1.67E-16	1.80E-16	1.74E-16	1.86E-16	1.80E-16
3.12E-16	3.17E-16	2.94E-16	2.66E-16	2.29E-16	2.10E-16	2.15E-16	2.14E-16	2.00E-16	1.88E-16	1.69E-16	1.76E-16	1.83E-16	1.85E-16	1.71E-16
2.88E-16	3.01E-16	2.82E-16	2.59E-16	2.39E-16	2.22E-16	1.99E-16	2.11E-16	2.11E-16	2.11E-16	1.82E-16	1.79E-16	1.84E-16	1.90E-16	1.64E-16
2.77E-16	2.79E-16	2.76E-16	2.54E-16	2.31E-16	2.21E-16	1.84E-16	2.01E-16	2.14E-16	2.09E-16	1.86E-16	1.76E-16	1.81E-16	1.85E-16	1.66E-16
2.71E-16	2.72E-16	2.61E-16	2.57E-16	2.16E-16	2.07E-16	1.90E-16	1.90E-16	2.01E-16	2.02E-16	1.88E-16	1.79E-16	1.77E-16	1.79E-16	1.53E-16
2.80E-16	2.69E-16	2.58E-16	2.47E-16	2.01E-16	1.87E-16	1.80E-16	1.87E-16	2.06E-16	1.94E-16	1.88E-16	1.79E-16	1.72E-16	1.59E-16	1.32E-16
2.85E-16	2.71E-16	2.57E-16	2.35E-16	1.93E-16	1.94E-16	1.92E-16	1.98E-16	2.02E-16	1.88E-16	1.76E-16	1.74E-16	1.50E-16	1.30E-16	1.07E-16
2.88E-16	2.71E-16	2.45E-16	2.20E-16	2.04E-16	2.02E-16	2.11E-16	2.02E-16	1.91E-16	1.80E-16	1.65E-16	1.70E-16	1.41E-16	1.25E-16	9.24E-17
4.66E-14	4.72E-14	4.58E-14	4.50E-14	4.49E-14	4.47E-14	4.45E-14	4.43E-14	4.41E-14	4.39E-14	4.36E-14	4.43E-14	4.43E-14	4.46E-14	4.57E-14

Quadrant 2 Data (16384 v

4.43E-08	4.36E-08	4.51E-08	4.44E-08	4.52E-08	4.33E-08	4.30E-08	4.14E-08	4.11E-08
4.31E-08	4.29E-08	4.38E-08	4.31E-08	4.34E-08	4.21E-08	4.10E-08	4.04E-08	4.04E-08
4.17E-08	4.20E-08	4.27E-08	4.21E-08	4.15E-08	4.07E-08	3.96E-08	3.95E-08	3.95E-08
4.11E-08	4.16E-08	4.17E-08	4.15E-08	4.04E-08	4.00E-08	3.89E-08	3.87E-08	3.88E-08
3.97E-08	4.07E-08	4.06E-08	4.03E-08	3.93E-08	3.98E-08	3.83E-08	3.79E-08	3.83E-08
3.75E-08	3.88E-08	3.92E-08	3.88E-08	3.81E-08	3.78E-08	3.77E-08	3.71E-08	3.72E-08
3.57E-08	3.64E-08	3.75E-08	3.74E-08	3.69E-08	3.70E-08	3.71E-08	3.67E-08	3.60E-08
3.51E-08	3.54E-08	3.64E-08	3.60E-08	3.56E-08	3.62E-08	3.63E-08	3.60E-08	3.51E-08
3.45E-08	3.45E-08	3.51E-08	3.46E-08	3.42E-08	3.46E-08	3.48E-08	3.43E-08	3.38E-08
3.42E-08	3.39E-08	3.38E-08	3.33E-08	3.27E-08	3.34E-08	3.35E-08	3.28E-08	3.26E-08
3.37E-08	3.35E-08	3.27E-08	3.23E-08	3.13E-08	3.26E-08	3.25E-08	3.19E-08	3.18E-08
3.41E-08	3.32E-08	3.18E-08	3.11E-08	3.01E-08	3.09E-08	3.11E-08	3.06E-08	3.07E-08
3.46E-08	3.33E-08	3.08E-08	3.01E-08	2.91E-08	2.92E-08	2.95E-08	2.95E-08	2.93E-08
3.50E-08	3.34E-08	3.04E-08	2.93E-08	2.82E-08	2.83E-08	2.86E-08	2.84E-08	2.78E-08
3.52E-08	3.32E-08	3.02E-08	2.88E-08	2.76E-08	2.74E-08	2.75E-08	2.72E-08	2.67E-08
3.34E-08	3.17E-08	3.01E-08	2.83E-08	2.71E-08	2.67E-08	2.64E-08	2.59E-08	2.55E-08
3.06E-08	2.96E-08	2.92E-08	2.80E-08	2.64E-08	2.57E-08	2.53E-08	2.41E-08	2.41E-08
2.79E-08	2.74E-08	2.79E-08	2.72E-08	2.57E-08	2.50E-08	2.39E-08	2.26E-08	2.28E-08
2.55E-08	2.53E-08	2.65E-08	2.63E-08	2.54E-08	2.43E-08	2.26E-08	2.15E-08	2.15E-08
2.32E-08	2.33E-08	2.38E-08	2.51E-08	2.47E-08	2.31E-08	2.17E-08	2.11E-08	2.08E-08
2.20E-08	2.18E-08	2.21E-08	2.36E-08	2.31E-08	2.17E-08	2.13E-08	2.08E-08	2.00E-08
2.10E-08	2.06E-08	2.08E-08	2.19E-08	2.18E-08	2.08E-08	2.06E-08	2.05E-08	1.96E-08
1.93E-08	1.91E-08	1.96E-08	2.01E-08	2.06E-08	2.00E-08	1.95E-08	1.97E-08	1.88E-08
1.82E-08	1.80E-08	1.82E-08	1.86E-08	1.88E-08	1.81E-08	1.79E-08	1.81E-08	1.79E-08
1.78E-08	1.72E-08	1.66E-08	1.63E-08	1.68E-08	1.65E-08	1.61E-08	1.58E-08	1.71E-08
1.67E-08	1.59E-08	1.51E-08	1.48E-08	1.54E-08	1.52E-08	1.50E-08	1.46E-08	1.57E-08
1.56E-08	1.42E-08	1.38E-08	1.36E-08	1.44E-08	1.40E-08	1.34E-08	1.33E-08	1.30E-08
1.46E-08	1.35E-08	1.28E-08	1.23E-08	1.38E-08	1.29E-08	1.23E-08	1.25E-08	1.18E-08
1.36E-08	1.26E-08	1.19E-08	1.15E-08	1.31E-08	1.18E-08	1.16E-08	1.15E-08	1.14E-08
1.22E-08	1.19E-08	1.10E-08	1.10E-08	1.21E-08	1.07E-08	1.10E-08	1.09E-08	1.07E-08
1.14E-08	1.06E-08	1.02E-08	1.04E-08	1.06E-08	9.62E-09	1.03E-08	1.00E-08	9.60E-09
1.05E-08	9.15E-09	9.68E-09	9.35E-09	9.13E-09	8.28E-09	8.77E-09	8.86E-09	8.46E-09
9.39E-09	8.27E-09	8.68E-09	8.37E-09	8.07E-09	6.91E-09	6.80E-09	7.63E-09	7.63E-09
7.73E-09	7.91E-09	6.86E-09	7.32E-09	6.78E-09	6.11E-09	5.64E-09	6.21E-09	6.48E-09
6.92E-09	7.43E-09	6.78E-09	6.05E-09	5.61E-09	5.55E-09	5.16E-09	5.36E-09	5.55E-09
5.82E-09	6.66E-09	6.36E-09	4.96E-09	4.73E-09	5.18E-09	4.76E-09	4.91E-09	4.56E-09
3.61E-09	4.93E-09	4.64E-09	3.83E-09	3.91E-09	4.47E-09	3.71E-09	4.00E-09	3.88E-09
1.86E-09	3.07E-09	2.91E-09	2.68E-09	3.21E-09	3.19E-09	3.10E-09	3.24E-09	2.91E-09
1.10E-09	2.28E-09	1.73E-09	2.02E-09	2.38E-09	2.42E-09	2.71E-09	1.96E-09	2.02E-09
1.07E-09	1.10E-09	9.69E-10	1.27E-09	1.36E-09	1.42E-09	1.79E-09	1.13E-09	1.05E-09
5.49E-10	3.73E-10	-9.71E-11	5.99E-10	4.63E-10	2.13E-10	9.10E-10	5.69E-10	8.50E-11
-3.59E-10	-3.02E-10	-5.36E-10	3.09E-10	-3.93E-10	-7.48E-10	-2.99E-10	-3.58E-10	-5.73E-10
-8.02E-10	-7.69E-10	-1.04E-09	-3.52E-10	-1.17E-09	-1.61E-09	-1.78E-09	-1.58E-09	-1.45E-09
-1.13E-09	-1.02E-09	-1.55E-09	-1.15E-09	-1.99E-09	-2.13E-09	-2.42E-09	-2.50E-09	-2.00E-09
-1.65E-09	-1.31E-09	-2.11E-09	-2.18E-09	-2.82E-09	-2.51E-09	-2.96E-09	-2.99E-09	-2.76E-09
-1.84E-09	-1.57E-09	-2.55E-09	-2.93E-09	-3.94E-09	-2.99E-09	-3.67E-09	-3.55E-09	-3.38E-09
-2.13E-09	-2.02E-09	-2.56E-09	-3.69E-09	-4.19E-09	-3.35E-09	-3.55E-09	-3.85E-09	-3.57E-09
-2.74E-09	-3.40E-09	-3.52E-09	-4.46E-09	-4.73E-09	-4.20E-09	-3.69E-09	-4.21E-09	-3.68E-09
-4.18E-09	-4.26E-09	-4.77E-09	-5.47E-09	-5.57E-09	-5.30E-09	-4.81E-09	-5.25E-09	-4.16E-09
-4.93E-09	-4.85E-09	-5.57E-09	-6.15E-09	-6.10E-09	-5.84E-09	-5.44E-09	-5.98E-09	-4.97E-09
-5.80E-09	-5.46E-09	-6.24E-09	-6.17E-09	-6.33E-09	-6.43E-09	-5.68E-09	-6.37E-09	-5.68E-09
-6.41E-09	-6.41E-09	-6.42E-09	-6.32E-09	-6.80E-09	-7.05E-09	-6.40E-09	-6.89E-09	-6.53E-09
-6.72E-09	-6.98E-09	-6.52E-09	-7.08E-09	-7.51E-09	-7.47E-09	-7.10E-09	-7.59E-09	-7.53E-09
-7.28E-09	-7.79E-09	-7.63E-09	-7.88E-09	-8.43E-09	-8.22E-09	-7.83E-09	-8.40E-09	-8.27E-09
-8.07E-09	-8.70E-09	-8.56E-09	-8.40E-09	-8.56E-09	-8.63E-09	-8.30E-09	-8.68E-09	-8.72E-09
-8.81E-09	-9.49E-09	-9.34E-09	-8.39E-09	-8.71E-09	-9.09E-09	-8.95E-09	-9.32E-09	-9.38E-09
-9.15E-09	-1.03E-08	-1.01E-08	-8.61E-09	-8.53E-09	-9.52E-09	-9.56E-09	-9.83E-09	-9.40E-09
-8.96E-09	-1.07E-08	-1.06E-08	-9.19E-09	-8.81E-09	-9.65E-09	-1.01E-08	-9.85E-09	-9.55E-09
-9.09E-09	-1.05E-08	-1.07E-08	-9.46E-09	-9.89E-09	-1.02E-08	-1.04E-08	-9.75E-09	-9.76E-09
-9.99E-09	-1.07E-08	-1.08E-08	-9.95E-09	-1.08E-08	-1.05E-08	-1.05E-08	-9.72E-09	-1.02E-08
-1.06E-08	-1.09E-08	-1.08E-08	-1.02E-08	-1.09E-08	-1.08E-08	-1.11E-08	-1.02E-08	-1.06E-08
-1.06E-08	-1.08E-08	-1.11E-08	-1.06E-08	-1.08E-08	-1.09E-08	-1.18E-08	-1.11E-08	-1.13E-08
-1.12E-08	-1.16E-08	-1.19E-08	-1.12E-08	-1.13E-08	-1.11E-08	-1.18E-08	-1.15E-08	-1.19E-08
-1.16E-08	-1.20E-08	-1.21E-08	-1.17E-08	-1.16E-08	-1.06E-08	-1.11E-08	-1.17E-08	-1.22E-08
-1.18E-08	-1.22E-08	-1.24E-08	-1.22E-08	-1.16E-08	-9.94E-09	-9.98E-09	-1.17E-08	-1.20E-08
-1.21E-08	-1.20E-08	-1.27E-08	-1.26E-08	-1.14E-08	-9.59E-09	-9.69E-09	-1.16E-08	-1.21E-08
-1.25E-08	-1.22E-08	-1.28E-08	-1.28E-08	-1.16E-08	-1.06E-08	-1.06E-08	-1.25E-08	-1.26E-08
-1.27E-08	-1.30E-08	-1.34E-08	-1.29E-08	-1.22E-08	-1.23E-08	-1.22E-08	-1.28E-08	-1.27E-08
-1.29E-08	-1.36E-08	-1.40E-08	-1.29E-08	-1.30E-08	-1.31E-08	-1.28E-08	-1.31E-08	-1.23E-08
-1.37E-08	-1.43E-08	-1.38E-08	-1.32E-08	-1.35E-08	-1.36E-08	-1.25E-08	-1.28E-08	-1.21E-08
-1.45E-08	-1.50E-08	-1.39E-08	-1.38E-08	-1.37E-08	-1.36E-08	-1.25E-08	-1.28E-08	-1.23E-08
-1.52E-08	-1.53E-08	-1.46E-08	-1.47E-08	-1.44E-08	-1.37E-08	-1.25E-08	-1.34E-08	-1.30E-08
-1.58E-08	-1.57E-08	-1.54E-08	-1.57E-08	-1.48E-08	-1.37E-08	-1.32E-08	-1.41E-08	-1.36E-08
-1.69E-08	-1.60E-08	-1.55E-08	-1.56E-08	-1.51E-08	-1.41E-08	-1.42E-08	-1.44E-08	-1.36E-08
-1.75E-08	-1.60E-08	-1.50E-08	-1.57E-08	-1.52E-08	-1.41E-08	-1.49E-08	-1.42E-08	-1.32E-08
-1.76E-08	-1.59E-08	-1.47E-08	-1.53E-08	-1.49E-08	-1.42E-08	-1.42E-08	-1.29E-08	-1.29E-08
-1.72E-08	-1.57E-08	-1.45E-08	-1.52E-08	-1.48E-08	-1.44E-08	-1.38E-08	-1.19E-08	-1.34E-08
-1.72E-08	-1.60E-08	-1.49E-08	-1.55E-08	-1.50E-08	-1.47E-08	-1.41E-08	-1.28E-08	-1.46E-08

-1.72E-08	-1.64E-08	-1.55E-08	-1.60E-08	-1.55E-08	-1.46E-08	-1.52E-08	-1.35E-08	-1.52E-08
-1.72E-08	-1.65E-08	-1.55E-08	-1.59E-08	-1.53E-08	-1.46E-08	-1.57E-08	-1.23E-08	-1.56E-08
-1.70E-08	-1.62E-08	-1.53E-08	-1.52E-08	-1.52E-08	-1.48E-08	-1.54E-08	-1.05E-08	-1.55E-08
-1.66E-08	-1.57E-08	-1.55E-08	-1.53E-08	-1.56E-08	-1.52E-08	-1.55E-08	-1.54E-08	-1.58E-08
-1.65E-08	-1.58E-08	-1.55E-08	-1.60E-08	-1.60E-08	-1.55E-08	-1.60E-08	-1.63E-08	-1.67E-08
-1.70E-08	-1.59E-08	-1.56E-08	-1.69E-08	-1.64E-08	-1.60E-08	-1.60E-08	-1.66E-08	-1.69E-08
-1.77E-08	-1.67E-08	-1.60E-08	-1.74E-08	-1.64E-08	-1.64E-08	-1.60E-08	-1.63E-08	-1.71E-08
-1.85E-08	-1.77E-08	-1.67E-08	-1.74E-08	-1.64E-08	-1.65E-08	-1.61E-08	-1.61E-08	-1.66E-08
-1.84E-08	-1.77E-08	-1.73E-08	-1.70E-08	-1.65E-08	-1.63E-08	-1.63E-08	-1.62E-08	-1.66E-08
-1.82E-08	-1.80E-08	-1.75E-08	-1.65E-08	-1.69E-08	-1.62E-08	-1.62E-08	-1.64E-08	-1.65E-08
-1.80E-08	-1.78E-08	-1.75E-08	-1.63E-08	-1.66E-08	-1.63E-08	-1.60E-08	-1.62E-08	-1.65E-08
-1.81E-08	-1.77E-08	-1.74E-08	-1.63E-08	-1.65E-08	-1.59E-08	-1.56E-08	-1.59E-08	-1.63E-08
-1.83E-08	-1.75E-08	-1.74E-08	-1.64E-08	-1.68E-08	-1.58E-08	-1.57E-08	-1.56E-08	-1.60E-08
-1.79E-08	-1.73E-08	-1.72E-08	-1.65E-08	-1.70E-08	-1.65E-08	-1.58E-08	-1.55E-08	-1.60E-08
-1.78E-08	-1.75E-08	-1.72E-08	-1.62E-08	-1.69E-08	-1.68E-08	-1.57E-08	-1.56E-08	-1.59E-08
-1.79E-08	-1.76E-08	-1.72E-08	-1.61E-08	-1.66E-08	-1.64E-08	-1.57E-08	-1.55E-08	-1.55E-08
-1.78E-08	-1.71E-08	-1.65E-08	-1.61E-08	-1.60E-08	-1.61E-08	-1.55E-08	-1.51E-08	-1.49E-08
-1.76E-08	-1.67E-08	-1.55E-08	-1.58E-08	-1.54E-08	-1.57E-08	-1.51E-08	-1.43E-08	-1.41E-08
-1.73E-08	-1.64E-08	-1.42E-08	-1.57E-08	-1.52E-08	-1.52E-08	-1.43E-08	-1.36E-08	-1.33E-08
-1.70E-08	-1.58E-08	-1.49E-08	-1.62E-08	-1.49E-08	-1.47E-08	-1.35E-08	-1.29E-08	-1.29E-08
-1.63E-08	-1.52E-08	-1.58E-08	-1.67E-08	-1.48E-08	-1.46E-08	-1.38E-08	-1.27E-08	-1.29E-08
-1.57E-08	-1.46E-08	-1.49E-08	-1.57E-08	-1.45E-08	-1.44E-08	-1.41E-08	-1.26E-08	-1.23E-08
-1.49E-08	-1.44E-08	-1.45E-08	-1.50E-08	-1.39E-08	-1.37E-08	-1.34E-08	-1.28E-08	-1.25E-08
-1.42E-08	-1.44E-08	-1.49E-08	-1.46E-08	-1.40E-08	-1.33E-08	-1.31E-08	-1.29E-08	-1.30E-08
-1.53E-08	-1.49E-08	-1.55E-08	-1.46E-08	-1.41E-08	-1.29E-08	-1.29E-08	-1.28E-08	-1.28E-08
-1.52E-08	-1.50E-08	-1.53E-08	-1.49E-08	-1.37E-08	-1.26E-08	-1.28E-08	-1.24E-08	-1.23E-08
-1.44E-08	-1.44E-08	-1.51E-08	-1.48E-08	-1.32E-08	-1.27E-08	-1.27E-08	-1.23E-08	-1.20E-08
-1.40E-08	-1.44E-08	-1.48E-08	-1.43E-08	-1.27E-08	-1.26E-08	-1.24E-08	-1.23E-08	-1.25E-08
-1.44E-08	-1.42E-08	-1.45E-08	-1.42E-08	-1.28E-08	-1.24E-08	-1.25E-08	-1.22E-08	-1.25E-08
-1.45E-08	-1.43E-08	-1.43E-08	-1.42E-08	-1.29E-08	-1.19E-08	-1.27E-08	-1.20E-08	-1.20E-08
-1.48E-08	-1.43E-08	-1.44E-08	-1.43E-08	-1.27E-08	-1.20E-08	-1.26E-08	-1.15E-08	-1.16E-08
-1.47E-08	-1.43E-08	-1.46E-08	-1.39E-08	-1.21E-08	-1.23E-08	-1.28E-08	-1.08E-08	-1.11E-08
-1.37E-08	-1.40E-08	-1.46E-08	-1.34E-08	-1.23E-08	-1.29E-08	-1.30E-08	-1.12E-08	-1.10E-08
-1.28E-08	-1.33E-08	-1.43E-08	-1.26E-08	-1.27E-08	-1.33E-08	-1.33E-08	-1.16E-08	-1.05E-08
-1.28E-08	-1.26E-08	-1.33E-08	-1.19E-08	-1.32E-08	-1.35E-08	-1.34E-08	-1.20E-08	-9.88E-09
-1.28E-08	-1.21E-08	-1.26E-08	-1.19E-08	-1.34E-08	-1.33E-08	-1.30E-08	-1.23E-08	-1.03E-08
-1.34E-08	-1.25E-08	-1.21E-08	-1.23E-08	-1.35E-08	-1.33E-08	-1.23E-08	-1.18E-08	-1.07E-08
-1.37E-08	-1.27E-08	-1.19E-08	-1.26E-08	-1.35E-08	-1.30E-08	-1.22E-08	-1.18E-08	-1.07E-08
-1.38E-08	-1.28E-08	-1.22E-08	-1.23E-08	-1.31E-08	-1.22E-08	-1.20E-08	-1.20E-08	-1.08E-08
-1.39E-08	-1.31E-08	-1.23E-08	-1.18E-08	-1.24E-08	-1.18E-08	-1.18E-08	-1.21E-08	-1.08E-08
-1.33E-08	-1.26E-08	-1.20E-08	-1.15E-08	-1.19E-08	-1.20E-08	-1.16E-08	-1.17E-08	-1.08E-08
-1.28E-08	-1.21E-08	-1.25E-08	-1.19E-08	-1.17E-08	-1.22E-08	-1.17E-08	-1.16E-08	-1.11E-08
-1.32E-08	-1.26E-08	-1.30E-08	-1.28E-08	-1.23E-08	-1.22E-08	-1.13E-08	-1.13E-08	-1.11E-08
-1.31E-08	-1.25E-08	-1.29E-08	-1.29E-08	-1.27E-08	-1.22E-08	-1.12E-08	-1.09E-08	-1.10E-08
-1.29E-08	-1.25E-08	-1.25E-08	-1.28E-08	-1.29E-08	-1.23E-08	-1.13E-08	-1.06E-08	-1.05E-08
-1.27E-08	-1.24E-08	-1.20E-08	-1.27E-08	-1.33E-08	-1.22E-08	-1.13E-08	-1.00E-08	-9.39E-09
-1.25E-08	-1.15E-08	-1.09E-08	-1.22E-08	-1.32E-08	-1.21E-08	-1.09E-08	-1.01E-08	-8.91E-09
-1.19E-08	-1.16E-08	-1.07E-08	-1.18E-08	-1.25E-08	-1.21E-08	-1.08E-08	-1.06E-08	-9.44E-09
-1.15E-08	-1.09E-08	-1.12E-08	-1.16E-08	-1.15E-08	-1.23E-08	-1.08E-08	-1.07E-08	-1.01E-08
-1.11E-08	-1.13E-08	-1.14E-08	-1.11E-08	-1.07E-08	-1.20E-08	-1.07E-08	-1.10E-08	-1.02E-08

Quadrant 2 Data Squared

1.97E-15	1.90E-15	2.03E-15	1.97E-15	2.04E-15	1.87E-15	1.85E-15	1.71E-15	1.69E-15
1.85E-15	1.84E-15	1.92E-15	1.85E-15	1.89E-15	1.77E-15	1.68E-15	1.63E-15	1.63E-15
1.74E-15	1.77E-15	1.82E-15	1.78E-15	1.72E-15	1.66E-15	1.57E-15	1.56E-15	1.56E-15
1.69E-15	1.73E-15	1.74E-15	1.72E-15	1.63E-15	1.60E-15	1.51E-15	1.50E-15	1.51E-15
1.57E-15	1.65E-15	1.65E-15	1.63E-15	1.54E-15	1.58E-15	1.47E-15	1.44E-15	1.46E-15
1.41E-15	1.50E-15	1.54E-15	1.50E-15	1.45E-15	1.43E-15	1.42E-15	1.38E-15	1.38E-15
1.28E-15	1.33E-15	1.41E-15	1.40E-15	1.36E-15	1.37E-15	1.38E-15	1.35E-15	1.29E-15
1.23E-15	1.25E-15	1.32E-15	1.30E-15	1.27E-15	1.31E-15	1.31E-15	1.30E-15	1.23E-15
1.19E-15	1.19E-15	1.23E-15	1.20E-15	1.17E-15	1.19E-15	1.21E-15	1.17E-15	1.14E-15
1.17E-15	1.15E-15	1.15E-15	1.11E-15	1.07E-15	1.12E-15	1.12E-15	1.08E-15	1.06E-15
1.14E-15	1.12E-15	1.07E-15	1.04E-15	9.79E-16	1.06E-15	1.06E-15	1.02E-15	1.01E-15
1.16E-15	1.10E-15	1.01E-15	9.64E-16	9.03E-16	9.56E-16	9.68E-16	9.39E-16	9.45E-16
1.20E-15	1.11E-15	9.49E-16	9.07E-16	8.49E-16	8.52E-16	8.72E-16	8.68E-16	8.58E-16
1.23E-15	1.12E-15	9.26E-16	8.58E-16	7.96E-16	7.99E-16	8.15E-16	8.05E-16	7.72E-16
1.24E-15	1.11E-15	9.14E-16	8.29E-16	7.61E-16	7.50E-16	7.54E-16	7.42E-16	7.12E-16
1.11E-15	1.01E-15	9.08E-16	7.99E-16	7.36E-16	7.12E-16	6.99E-16	6.69E-16	6.52E-16
9.37E-16	8.75E-16	8.53E-16	7.84E-16	6.95E-16	6.62E-16	6.42E-16	5.82E-16	5.79E-16
7.80E-16	7.51E-16	7.80E-16	7.42E-16	6.59E-16	6.27E-16	5.71E-16	5.12E-16	5.21E-16
6.52E-16	6.41E-16	7.01E-16	6.94E-16	6.47E-16	5.90E-16	5.10E-16	4.60E-16	4.61E-16
5.38E-16	5.41E-16	5.65E-16	6.29E-16	6.12E-16	5.32E-16	4.71E-16	4.46E-16	4.34E-16
4.86E-16	4.74E-16	4.86E-16	5.57E-16	5.35E-16	4.70E-16	4.54E-16	4.31E-16	3.98E-16
4.39E-16	4.26E-16	4.34E-16	4.81E-16	4.77E-16	4.31E-16	4.22E-16	4.19E-16	3.85E-16
3.72E-16	3.64E-16	3.84E-16	4.05E-16	4.26E-16	4.00E-16	3.81E-16	3.89E-16	3.55E-16
3.33E-16	3.25E-16	3.32E-16	3.45E-16	3.55E-16	3.29E-16	3.22E-16	3.29E-16	3.20E-16
3.16E-16	2.97E-16	2.76E-16	2.67E-16	2.82E-16	2.72E-16	2.58E-16	2.50E-16	2.94E-16
2.79E-16	2.52E-16	2.27E-16	2.19E-16	2.37E-16	2.32E-16	2.24E-16	2.13E-16	2.45E-16
2.43E-16	2.01E-16	1.89E-16	1.84E-16	2.06E-16	1.97E-16	1.79E-16	1.78E-16	1.69E-16
2.14E-16	1.82E-16	1.64E-16	1.52E-16	1.89E-16	1.66E-16	1.52E-16	1.56E-16	1.40E-16
1.84E-16	1.60E-16	1.42E-16	1.33E-16	1.72E-16	1.40E-16	1.35E-16	1.32E-16	1.30E-16
1.50E-16	1.42E-16	1.20E-16	1.21E-16	1.46E-16	1.15E-16	1.21E-16	1.18E-16	1.15E-16
1.30E-16	1.12E-16	1.04E-16	1.09E-16	1.12E-16	9.25E-17	1.06E-16	1.01E-16	9.21E-17

1.10E-16	8.37E-17	9.37E-17	8.74E-17	8.33E-17	6.86E-17	7.69E-17	7.85E-17	7.15E-17
8.81E-17	6.85E-17	7.53E-17	7.01E-17	6.52E-17	4.77E-17	4.63E-17	5.82E-17	5.81E-17
5.98E-17	6.25E-17	4.70E-17	5.36E-17	4.59E-17	3.74E-17	3.18E-17	3.85E-17	4.19E-17
4.79E-17	5.52E-17	4.59E-17	3.66E-17	3.14E-17	3.08E-17	2.66E-17	2.88E-17	3.08E-17
3.39E-17	4.43E-17	4.04E-17	2.46E-17	2.24E-17	2.68E-17	2.27E-17	2.41E-17	2.08E-17
1.31E-17	2.43E-17	2.16E-17	1.47E-17	1.53E-17	2.00E-17	1.37E-17	1.60E-17	1.51E-17
3.44E-18	9.41E-18	8.49E-18	7.19E-18	1.03E-17	1.02E-17	9.62E-18	1.05E-17	8.45E-18
1.20E-18	5.22E-18	2.98E-18	4.08E-18	5.66E-18	5.88E-18	7.32E-18	3.82E-18	4.10E-18
1.15E-18	1.21E-18	9.39E-19	1.61E-18	1.85E-18	2.03E-18	3.19E-18	1.29E-18	1.09E-18
3.01E-19	1.39E-19	9.44E-21	3.58E-19	2.14E-19	4.52E-20	8.28E-19	3.24E-19	7.23E-21
1.29E-19	9.12E-20	2.87E-19	9.53E-20	1.54E-19	5.59E-19	8.92E-20	1.28E-19	3.28E-19
6.43E-19	5.91E-19	1.09E-18	1.24E-19	1.38E-18	2.61E-18	3.18E-18	2.50E-18	2.10E-18
1.29E-18	1.04E-18	2.39E-18	1.33E-18	3.96E-18	4.52E-18	5.88E-18	6.23E-18	4.01E-18
2.73E-18	1.71E-18	4.46E-18	4.75E-18	7.97E-18	6.31E-18	8.75E-18	8.94E-18	7.60E-18
3.37E-18	2.48E-18	6.52E-18	8.61E-18	1.55E-17	8.96E-18	1.35E-17	1.26E-17	1.14E-17
4.54E-18	4.09E-18	6.58E-18	1.37E-17	1.75E-17	1.12E-17	1.26E-17	1.48E-17	1.27E-17
7.49E-18	1.16E-17	1.24E-17	1.99E-17	2.24E-17	1.77E-17	1.36E-17	1.77E-17	1.35E-17
1.75E-17	1.81E-17	2.28E-17	2.99E-17	3.10E-17	2.81E-17	2.31E-17	2.76E-17	1.73E-17
2.43E-17	2.36E-17	3.11E-17	3.78E-17	3.72E-17	3.41E-17	2.96E-17	3.58E-17	2.47E-17
3.36E-17	2.98E-17	3.89E-17	3.81E-17	4.00E-17	4.13E-17	3.22E-17	4.06E-17	3.23E-17
4.11E-17	4.11E-17	4.12E-17	3.99E-17	4.63E-17	4.97E-17	4.09E-17	4.74E-17	4.26E-17
4.52E-17	4.87E-17	4.24E-17	5.01E-17	5.64E-17	5.58E-17	5.03E-17	5.76E-17	5.67E-17
5.30E-17	6.07E-17	5.82E-17	6.20E-17	7.10E-17	6.76E-17	6.14E-17	7.06E-17	6.84E-17
6.52E-17	7.57E-17	7.33E-17	7.05E-17	7.33E-17	7.44E-17	6.89E-17	7.54E-17	7.61E-17
7.77E-17	9.01E-17	8.73E-17	7.03E-17	7.59E-17	8.27E-17	8.01E-17	8.68E-17	8.79E-17
8.37E-17	1.06E-16	1.01E-16	7.41E-17	7.28E-17	9.06E-17	9.15E-17	9.65E-17	8.84E-17
8.02E-17	1.13E-16	1.13E-16	8.44E-17	7.76E-17	9.31E-17	1.01E-16	9.71E-17	9.12E-17
8.26E-17	1.11E-16	1.15E-16	8.95E-17	9.79E-17	1.04E-16	1.08E-16	9.50E-17	9.52E-17
9.98E-17	1.14E-16	1.18E-16	9.90E-17	1.17E-16	1.10E-16	1.10E-16	9.46E-17	1.04E-16
1.13E-16	1.18E-16	1.17E-16	1.04E-16	1.19E-16	1.16E-16	1.24E-16	1.05E-16	1.12E-16
1.12E-16	1.17E-16	1.24E-16	1.12E-16	1.16E-16	1.19E-16	1.38E-16	1.22E-16	1.28E-16
1.25E-16	1.34E-16	1.42E-16	1.27E-16	1.28E-16	1.22E-16	1.40E-16	1.33E-16	1.41E-16
1.33E-16	1.44E-16	1.46E-16	1.37E-16	1.34E-16	1.12E-16	1.23E-16	1.38E-16	1.48E-16
1.39E-16	1.49E-16	1.54E-16	1.48E-16	1.34E-16	9.87E-17	9.95E-17	1.36E-16	1.45E-16
1.46E-16	1.44E-16	1.62E-16	1.59E-16	1.31E-16	9.20E-17	9.39E-17	1.35E-16	1.46E-16
1.55E-16	1.48E-16	1.65E-16	1.64E-16	1.35E-16	1.12E-16	1.12E-16	1.57E-16	1.60E-16
1.61E-16	1.68E-16	1.80E-16	1.66E-16	1.49E-16	1.50E-16	1.49E-16	1.65E-16	1.61E-16
1.66E-16	1.85E-16	1.96E-16	1.66E-16	1.68E-16	1.72E-16	1.63E-16	1.72E-16	1.52E-16
1.87E-16	2.05E-16	1.91E-16	1.74E-16	1.82E-16	1.84E-16	1.56E-16	1.63E-16	1.47E-16
2.10E-16	2.24E-16	1.94E-16	1.91E-16	1.88E-16	1.85E-16	1.56E-16	1.63E-16	1.51E-16
2.30E-16	2.35E-16	2.13E-16	2.15E-16	2.07E-16	1.88E-16	1.55E-16	1.79E-16	1.70E-16
2.50E-16	2.46E-16	2.36E-16	2.45E-16	2.18E-16	1.89E-16	1.75E-16	1.98E-16	1.84E-16
2.84E-16	2.56E-16	2.39E-16	2.42E-16	2.28E-16	1.98E-16	2.02E-16	2.06E-16	1.85E-16
3.06E-16	2.56E-16	2.25E-16	2.45E-16	2.31E-16	1.99E-16	2.21E-16	2.02E-16	1.73E-16
3.08E-16	2.52E-16	2.16E-16	2.35E-16	2.21E-16	2.02E-16	2.01E-16	1.65E-16	1.66E-16
2.94E-16	2.46E-16	2.11E-16	2.30E-16	2.18E-16	2.08E-16	1.91E-16	1.42E-16	1.80E-16
2.94E-16	2.57E-16	2.23E-16	2.41E-16	2.25E-16	2.15E-16	1.99E-16	1.63E-16	2.13E-16
2.95E-16	2.70E-16	2.41E-16	2.55E-16	2.41E-16	2.13E-16	2.30E-16	1.82E-16	2.32E-16
2.96E-16	2.72E-16	2.41E-16	2.52E-16	2.33E-16	2.14E-16	2.46E-16	1.52E-16	2.44E-16
2.89E-16	2.61E-16	2.35E-16	2.32E-16	2.31E-16	2.19E-16	2.37E-16	1.11E-16	2.39E-16
2.76E-16	2.47E-16	2.40E-16	2.34E-16	2.44E-16	2.32E-16	2.41E-16	2.37E-16	2.50E-16
2.73E-16	2.49E-16	2.42E-16	2.57E-16	2.56E-16	2.39E-16	2.55E-16	2.66E-16	2.78E-16
2.90E-16	2.51E-16	2.45E-16	2.87E-16	2.69E-16	2.57E-16	2.57E-16	2.75E-16	2.84E-16
3.15E-16	2.78E-16	2.56E-16	3.01E-16	2.70E-16	2.68E-16	2.55E-16	2.67E-16	2.91E-16
3.42E-16	3.13E-16	2.80E-16	3.02E-16	2.68E-16	2.73E-16	2.60E-16	2.58E-16	2.77E-16
3.39E-16	3.14E-16	3.00E-16	2.89E-16	2.74E-16	2.67E-16	2.65E-16	2.63E-16	2.76E-16
3.32E-16	3.23E-16	3.05E-16	2.73E-16	2.85E-16	2.63E-16	2.61E-16	2.69E-16	2.73E-16
3.25E-16	3.18E-16	3.05E-16	2.67E-16	2.75E-16	2.66E-16	2.56E-16	2.64E-16	2.72E-16
3.28E-16	3.13E-16	3.04E-16	2.67E-16	2.71E-16	2.51E-16	2.44E-16	2.53E-16	2.65E-16
3.34E-16	3.07E-16	3.01E-16	2.67E-16	2.81E-16	2.49E-16	2.46E-16	2.43E-16	2.57E-16
3.21E-16	3.00E-16	2.95E-16	2.71E-16	2.88E-16	2.72E-16	2.48E-16	2.41E-16	2.57E-16
3.18E-16	3.06E-16	2.96E-16	2.63E-16	2.84E-16	2.84E-16	2.47E-16	2.44E-16	2.53E-16
3.22E-16	3.10E-16	2.94E-16	2.58E-16	2.76E-16	2.71E-16	2.45E-16	2.41E-16	2.42E-16
3.15E-16	2.92E-16	2.72E-16	2.59E-16	2.56E-16	2.59E-16	2.40E-16	2.29E-16	2.21E-16
3.09E-16	2.78E-16	2.40E-16	2.49E-16	2.36E-16	2.46E-16	2.28E-16	2.04E-16	1.98E-16
2.99E-16	2.69E-16	2.03E-16	2.47E-16	2.31E-16	2.31E-16	2.04E-16	1.85E-16	1.76E-16
2.89E-16	2.51E-16	2.22E-16	2.63E-16	2.23E-16	2.16E-16	1.83E-16	1.66E-16	1.65E-16
2.67E-16	2.30E-16	2.49E-16	2.80E-16	2.18E-16	2.14E-16	1.91E-16	1.62E-16	1.67E-16
2.47E-16	2.13E-16	2.21E-16	2.45E-16	2.10E-16	2.08E-16	1.98E-16	1.58E-16	1.51E-16
2.21E-16	2.08E-16	2.10E-16	2.25E-16	1.92E-16	1.88E-16	1.79E-16	1.64E-16	1.56E-16
2.01E-16	2.08E-16	2.21E-16	2.13E-16	1.97E-16	1.76E-16	1.70E-16	1.67E-16	1.70E-16
2.35E-16	2.21E-16	2.40E-16	2.12E-16	1.97E-16	1.67E-16	1.66E-16	1.64E-16	1.63E-16
2.32E-16	2.24E-16	2.35E-16	2.22E-16	1.87E-16	1.58E-16	1.65E-16	1.55E-16	1.52E-16
2.08E-16	2.07E-16	2.29E-16	2.18E-16	1.74E-16	1.61E-16	1.61E-16	1.51E-16	1.45E-16
1.96E-16	2.08E-16	2.18E-16	2.05E-16	1.62E-16	1.60E-16	1.53E-16	1.50E-16	1.56E-16
2.08E-16	2.02E-16	2.11E-16	2.02E-16	1.64E-16	1.54E-16	1.57E-16	1.48E-16	1.57E-16
2.10E-16	2.05E-16	2.03E-16	2.01E-16	1.66E-16	1.41E-16	1.62E-16	1.43E-16	1.43E-16
2.19E-16	2.06E-16	2.07E-16	2.04E-16	1.61E-16	1.43E-16	1.60E-16	1.33E-16	1.35E-16
2.16E-16	2.06E-16	2.13E-16	1.93E-16	1.46E-16	1.51E-16	1.65E-16	1.16E-16	1.24E-16
1.87E-16	1.95E-16	2.15E-16	1.80E-16	1.52E-16	1.66E-16	1.69E-16	1.26E-16	1.21E-16
1.63E-16	1.77E-16	2.04E-16	1.59E-16	1.61E-16	1.77E-16	1.77E-16	1.34E-16	1.11E-16
1.64E-16	1.58E-16	1.77E-16	1.41E-16	1.73E-16	1.83E-16	1.80E-16	1.44E-16	9.75E-17
1.63E-16	1.46E-16	1.59E-16	1.41E-16	1.80E-16	1.78E-16	1.70E-16	1.51E-16	1.05E-16
1.79E-16	1.57E-16	1.46E-16	1.51E-16	1.83E-16	1.77E-16	1.50E-16	1.39E-16	1.15E-16
1.87E-16	1.61E-16	1.41E-16	1.58E-16	1.82E-16	1.70E-16	1.50E-16	1.39E-16	1.15E-16
1.91E-16	1.63E-16	1.48E-16	1.51E-16	1.73E-16	1.50E-16	1.43E-16	1.44E-16	1.16E-16

1.94E-16	1.71E-16	1.51E-16	1.39E-16	1.55E-16	1.39E-16	1.39E-16		1.46E-16	1.16E-16
1.77E-16	1.58E-16	1.45E-16	1.33E-16	1.42E-16	1.45E-16	1.34E-16		1.38E-16	1.18E-16
1.65E-16	1.46E-16	1.57E-16	1.41E-16	1.37E-16	1.48E-16	1.36E-16		1.34E-16	1.22E-16
1.75E-16	1.59E-16	1.69E-16	1.64E-16	1.51E-16	1.48E-16	1.28E-16		1.27E-16	1.23E-16
1.72E-16	1.55E-16	1.66E-16	1.66E-16	1.61E-16	1.49E-16	1.26E-16		1.20E-16	1.22E-16
1.65E-16	1.55E-16	1.56E-16	1.65E-16	1.67E-16	1.52E-16	1.28E-16		1.11E-16	1.11E-16
1.62E-16	1.54E-16	1.44E-16	1.61E-16	1.76E-16	1.49E-16	1.28E-16		1.01E-16	8.81E-17
1.55E-16	1.33E-16	1.18E-16	1.49E-16	1.73E-16	1.47E-16	1.19E-16		1.02E-16	7.94E-17
1.40E-16	1.36E-16	1.15E-16	1.39E-16	1.56E-16	1.47E-16	1.17E-16		1.13E-16	8.91E-17
1.31E-16	1.18E-16	1.25E-16	1.35E-16	1.32E-16	1.51E-16	1.16E-16		1.14E-16	1.01E-16
1.22E-16	1.27E-16	1.31E-16	1.23E-16	1.15E-16	1.45E-16	1.15E-16		1.20E-16	1.04E-16
4.48E-14	4.33E-14	4.26E-14	4.17E-14	4.04E-14	3.91E-14	3.79E-14	8.05E-12	3.65E-14	3.62E-14

SUM OF SQUARED QUADR/	8.05E-12
TOTAL NUMBER VALUES IN	16,384
AVERAGE SQUARED	4.91E-16
RMS 2.2164E-08	

alues):

4.09E-08	4.05E-08	4.07E-08	4.01E-08	4.06E-08	4.05E-08	4.04E-08	4.10E-08	3.98E-08	3.99E-08	3.96E-08	3.96E-08	3.99E-08	3.92E-08	3.91E-08
3.98E-08	3.94E-08	3.95E-08	3.97E-08	3.98E-08	3.97E-08	3.89E-08	3.97E-08	3.91E-08	3.98E-08	3.87E-08	3.85E-08	3.83E-08	3.74E-08	3.75E-08
3.89E-08	3.79E-08	3.84E-08	3.89E-08	3.89E-08	3.87E-08	3.76E-08	3.89E-08	3.84E-08	3.97E-08	3.82E-08	3.72E-08	3.71E-08	3.59E-08	3.62E-08
3.81E-08	3.69E-08	3.77E-08	3.78E-08	3.72E-08	3.72E-08	3.63E-08	3.79E-08	3.82E-08	3.94E-08	3.79E-08	3.63E-08	3.54E-08	3.39E-08	3.49E-08
3.71E-08	3.59E-08	3.66E-08	3.64E-08	3.54E-08	3.58E-08	3.50E-08	3.61E-08	3.70E-08	3.80E-08	3.69E-08	3.52E-08	3.38E-08	3.23E-08	3.38E-08
3.58E-08	3.48E-08	3.54E-08	3.52E-08	3.39E-08	3.45E-08	3.35E-08	3.39E-08	3.46E-08	3.57E-08	3.48E-08	3.38E-08	3.28E-08	3.10E-08	3.29E-08
3.45E-08	3.39E-08	3.43E-08	3.36E-08	3.25E-08	3.33E-08	3.23E-08	3.22E-08	3.17E-08	3.30E-08	3.24E-08	3.23E-08	3.16E-08	3.03E-08	3.19E-08
3.37E-08	3.34E-08	3.34E-08	3.26E-08	3.18E-08	3.20E-08	3.13E-08	3.08E-08	3.05E-08	3.13E-08	3.09E-08	3.07E-08	3.02E-08	2.94E-08	3.09E-08
3.30E-08	3.31E-08	3.23E-08	3.18E-08	3.09E-08	3.10E-08	3.04E-08	2.95E-08	2.94E-08	2.98E-08	2.93E-08	2.90E-08	2.88E-08	2.80E-08	2.94E-08
3.22E-08	3.20E-08	3.17E-08	3.12E-08	2.99E-08	3.00E-08	2.95E-08	2.85E-08	2.85E-08	2.86E-08	2.84E-08	2.73E-08	2.76E-08	2.66E-08	2.83E-08
3.11E-08	3.06E-08	3.03E-08	3.02E-08	2.92E-08	2.92E-08	2.86E-08	2.77E-08	2.79E-08	2.71E-08	2.76E-08	2.61E-08	2.64E-08	2.52E-08	2.58E-08
3.00E-08	2.93E-08	2.90E-08	2.87E-08	2.82E-08	2.82E-08	2.80E-08	2.65E-08	2.74E-08	2.61E-08	2.64E-08	2.52E-08	2.57E-08	2.44E-08	2.45E-08
2.88E-08	2.82E-08	2.74E-08	2.72E-08	2.69E-08	2.69E-08	2.71E-08	2.57E-08	2.66E-08	2.52E-08	2.53E-08	2.45E-08	2.49E-08	2.31E-08	2.35E-08
2.73E-08	2.68E-08	2.58E-08	2.56E-08	2.54E-08	2.52E-08	2.58E-08	2.48E-08	2.53E-08	2.49E-08	2.47E-08	2.39E-08	2.46E-08	2.26E-08	2.31E-08
2.62E-08	2.57E-08	2.46E-08	2.46E-08	2.39E-08	2.40E-08	2.45E-08	2.41E-08	2.39E-08	2.43E-08	2.41E-08	2.29E-08	2.33E-08	2.18E-08	2.21E-08
2.48E-08	2.47E-08	2.39E-08	2.33E-08	2.28E-08	2.30E-08	2.34E-08	2.30E-08	2.31E-08	2.32E-08	2.31E-08	2.13E-08	2.19E-08	2.06E-08	2.07E-08
2.35E-08	2.32E-08	2.23E-08	2.19E-08	2.17E-08	2.20E-08	2.24E-08	2.19E-08	2.19E-08	2.20E-08	2.12E-08	2.06E-08	2.13E-08	2.00E-08	1.91E-08
2.24E-08	2.27E-08	2.14E-08	2.13E-08	2.09E-08	2.08E-08	2.13E-08	2.10E-08	2.04E-08	2.06E-08	1.96E-08	2.00E-08	2.06E-08	1.96E-08	1.74E-08
2.15E-08	2.22E-08	2.07E-08	2.09E-08	2.00E-08	1.99E-08	2.00E-08	2.00E-08	1.93E-08	1.94E-08	1.86E-08	1.91E-08	1.97E-08	1.90E-08	1.64E-08
2.11E-08	2.13E-08	1.98E-08	1.99E-08	1.97E-08	1.94E-08	1.94E-08	1.92E-08	1.86E-08	1.85E-08	1.78E-08	1.80E-08	1.83E-08	1.77E-08	1.55E-08
2.04E-08	2.02E-08	1.87E-08	1.91E-08	1.87E-08	1.82E-08	1.86E-08	1.84E-08	1.79E-08	1.76E-08	1.70E-08	1.67E-08	1.67E-08	1.59E-08	1.44E-08
1.91E-08	1.93E-08	1.79E-08	1.80E-08	1.74E-08	1.66E-08	1.72E-08	1.77E-08	1.68E-08	1.66E-08	1.62E-08	1.58E-08	1.54E-08	1.42E-08	1.37E-08
1.80E-08	1.80E-08	1.72E-08	1.67E-08	1.62E-08	1.52E-08	1.56E-08	1.62E-08	1.57E-08	1.56E-08	1.52E-08	1.51E-08	1.40E-08	1.29E-08	1.25E-08
1.69E-08	1.69E-08	1.68E-08	1.57E-08	1.53E-08	1.44E-08	1.44E-08	1.44E-08	1.48E-08	1.49E-08	1.49E-08	1.48E-08	1.30E-08	1.20E-08	1.18E-08
1.60E-08	1.60E-08	1.62E-08	1.49E-08	1.42E-08	1.35E-08	1.32E-08	1.33E-08	1.43E-08	1.38E-08	1.39E-08	1.39E-08	1.24E-08	1.13E-08	1.12E-08
1.50E-08	1.45E-08	1.44E-08	1.36E-08	1.33E-08	1.30E-08	1.22E-08	1.28E-08	1.37E-08	1.25E-08	1.33E-08	1.35E-08	1.17E-08	1.09E-08	1.07E-08
1.42E-08	1.33E-08	1.27E-08	1.29E-08	1.23E-08	1.22E-08	1.18E-08	1.23E-08	1.27E-08	1.15E-08	1.20E-08	1.16E-08	1.05E-08	1.04E-08	9.35E-09
1.30E-08	1.25E-08	1.15E-08	1.17E-08	1.13E-08	1.16E-08	1.16E-08	1.19E-08	1.16E-08	1.05E-08	1.05E-08	1.03E-08	9.36E-09	9.95E-09	7.96E-09
1.21E-08	1.17E-08	1.06E-08	1.06E-08	1.06E-08	1.07E-08	1.13E-08	1.06E-08	9.59E-09	9.34E-09	8.93E-09	9.35E-09	8.41E-09	7.78E-09	6.84E-09
1.11E-08	1.04E-08	9.90E-09	1.02E-08	1.04E-08	1.01E-08	1.01E-08	8.86E-09	8.17E-09	8.33E-09	8.23E-09	8.44E-09	7.50E-09	7.13E-09	6.25E-09
1.01E-08	9.09E-09	9.34E-09	9.55E-09	9.29E-09	9.13E-09	8.87E-09	8.15E-09	7.06E-09	7.38E-09	7.46E-09	7.22E-09	6.77E-09	6.04E-09	6.12E-09
8.83E-09	8.14E-09	8.78E-09	8.39E-09	7.95E-09	8.43E-09	8.16E-09	7.85E-09	6.78E-09	6.58E-09	6.37E-09	6.27E-09	5.71E-09	4.72E-09	5.40E-09
7.86E-09	7.34E-09	7.76E-09	7.12E-09	6.90E-09	8.19E-09	7.91E-09	7.56E-09	6.24E-09	5.51E-09	5.22E-09	5.47E-09	4.37E-09	3.57E-09	4.83E-09
7.18E-09	6.18E-09	6.52E-09	6.13E-09	6.07E-09	7.06E-09	7.29E-09	7.14E-09	6.05E-09	4.92E-09	4.79E-09	5.06E-09	3.37E-09	2.81E-09	3.76E-09
5.89E-09	4.99E-09	5.42E-09	5.30E-09	5.78E-09	5.85E-09	5.95E-09	6.16E-09	5.44E-09	4.42E-09	4.42E-09	4.47E-09	2.55E-09	1.81E-09	3.01E-09
4.62E-09	4.03E-09	3.97E-09	4.60E-09	4.69E-09	4.90E-09	5.11E-09	5.37E-09	3.80E-09	3.69E-09	3.58E-09	3.12E-09	2.09E-09	1.35E-09	2.67E-09
3.70E-09	3.15E-09	3.14E-09	3.87E-09	3.64E-09	3.88E-09	3.89E-09	4.59E-09	2.44E-09	2.32E-09	2.22E-09	1.89E-09	1.40E-09	9.18E-10	2.12E-09
3.15E-09	2.40E-09	2.98E-09	3.35E-09	3.17E-09	3.32E-09	2.36E-09	3.11E-09	1.97E-09	1.64E-09	1.26E-09	7.68E-10	4.61E-10	5.16E-10	1.06E-09
2.33E-09	1.81E-09	2.73E-09	3.15E-09	2.96E-09	2.79E-09	1.65E-09	1.74E-09	1.70E-09	1.20E-09	9.94E-10	2.69E-10	-8.09E-11	-6.24E-10	4.42E-10
1.17E-09	1.34E-09	2.36E-09	2.96E-09	2.49E-09	2.04E-09	1.28E-09	1.00E-09	9.78E-10	6.29E-10	6.10E-10	-4.81E-10	-7.68E-10	-1.33E-09	-5.70E-10
6.95E-10	2.29E-10	1.23E-09	2.07E-09	1.66E-09	8.73E-10	4.97E-10	4.12E-10	3.65E-10	3.99E-10	1.93E-10	-1.26E-09	-1.58E-09	-1.72E-09	-1.42E-09
-1.28E-11	-9.06E-10	-1.54E-10	1.05E-10	7.76E-10	-6.78E-11	-2.54E-10	-1.17E-10	-3.85E-10	-3.79E-10	-7.14E-10	-2.25E-09	-1.94E-09	-2.41E-09	-2.43E-09
-6.81E-10	-1.59E-09	-1.18E-09	-9.93E-10	-5.99E-10	-1.17E-09	-1.34E-09	-1.29E-09	-1.56E-09	-1.57E-09	-1.95E-09	-3.51E-09	-2.97E-09	-3.27E-09	-2.80E-09
-1.21E-09	-2.00E-09	-1.67E-09	-1.69E-09	-1.80E-09	-2.11E-09	-2.00E-09	-2.59E-09	-2.72E-09	-2.63E-09	-3.09E-09	-4.23E-09	-3.42E-09	-4.00E-09	-3.14E-09
-2.28E-09	-2.41E-09	-2.49E-09	-2.40E-09	-2.67E-09	-2.82E-09	-3.01E-09	-3.78E-09	-3.52E-09	-3.57E-09	-4.15E-09	-4.73E-09	-4.45E-09	-4.52E-09	-3.54E-09
-3.60E-09	-2.58E-09	-3.11E-09	-2.92E-09	-3.09E-09	-3.01E-09	-3.65E-09	-4.62E-09	-4.30E-09	-4.45E-09	-5.21E-09	-5.21E-09	-4.81E-09	-4.82E-09	-4.20E-09
-3.92E-09	-2.82E-09	-3.41E-09	-3.48E-09	-3.53E-09	-4.06E-09	-4.44E-09	-5.09E-09	-5.06E-09	-4.85E-09	-5.86E-09	-5.66E-09	-5.37E-09	-5.36E-09	-4.83E-09
-4.13E-09	-3.34E-09	-3.87E-09	-3.77E-09	-3.47E-09	-4.02E-09	-4.66E-09	-5.41E-09	-5.49E-09	-5.30E-09	-6.02E-09	-5.86E-09	-5.80E-09	-6.06E-09	-5.29E-09
-4.52E-09	-4.21E-09	-4.18E-09	-3.95E-09	-3.54E-09	-3.98E-09	-4.64E-09	-5.85E-09	-5.65E-09	-5.44E-09	-6.21E-09	-6.41E-09	-6.53E-09	-6.43E-09	-5.72E-09
-5.45E-09	-4.62E-09	-4.54E-09	-4.24E-09	-2.96E-09	-3.61E-09	-4.44E-09	-6.10E-09	-5.61E-09	-5.89E-09	-6.25E-09	-7.08E-09	-6.66E-09	-7.08E-09	-6.09E-09
-5.85E-09	-4.34E-09	-4.44E-09	-3.90E-09	-2.42E-09	-3.35E-09	-4.13E-09	-6.03E-09	-5.79E-09	-6.63E-09	-6.36E-09	-7.12E-09	-6.54E-09	-7.73E-09	-6.89E-09
-6.03E-09	-4.32E-09	-4.48E-09	-3.62E-09	-2.36E-09	-2.94E-09	-4.24E-09	-6.12E-09	-6.02E-09	-8.08E-09	-6.55E-09	-6.86E-09	-6.75E-09	-8.23E-09	-7.27E-09
-6.37E-09	-4.74E-09	-4.85E-09	-3.96E-09	-2.87E-09	-3.15E-09	-4.31E-09	-6.36E-09	-7.30E-09	-1.01E-08	-6.95E-09	-6.63E-09	-6.79E-09	-7.89E-09	-7.20E-09
-7.27E-09	-6.10E-09	-6.31E-09	-5.75E-09	-4.93E-09	-4.97E-09	-5.68E-09	-6.56E-09	-7.90E-09	-1.05E-08	-7.55E-09	-6.90E-09	-7.13E-09	-8.31E-09	-7.78E-09
-8.10E-09	-6.70E-09	-7.54E-09	-7.53E-09	-6.55E-09	-6.70E-09	-6.88E-09	-6.78E-09	-7.24E-09	-7.82E-09	-6.74E-09	-7.43E-09	-8.50E-09	-9.04E-09	-8.59E-09
-8.45E-09	-7.14E-09	-8.27E-09	-8.52E-09	-7.70E-09	-8.20E-09	-8.02E-09	-8.14E-09	-7.31E-09	-7.03E-09	-7.50E-09	-8.49E-09	-9.34E-09	-9.81E-09	-9.29E-09
-9.09E-09	-8.01E-09	-8.89E-09	-9.01E-09	-8.82E-09	-9.13E-09	-8.82E-09	-9.27E-09	-8.34E-09	-8.35E-09	-8.90E-09	-9.31E-09	-1.00E-08	-9.38E-09	-9.96E-09
-9.69E-09	-8.91E-09	-9.54E-09	-9.84E-09	-9.52E-09	-9.92E-09	-9.36E-09	-1.01E-08	-9.57E-09	-9.19E-09	-9.60E-09	-9.92E-09	-1.06E-08	-8.69E-09	-1.06E-08
-1.01E-08	-9.65E-09	-1.04E-08	-1.06E-08	-1.03E-08	-1.04E-08	-1.04E-08	-1.06E-08	-1.01E-08	-9.90E-09	-9.95E-09	-1.02E-08	-1.09E-08	-5.12E-09	-1.04E-08
-1.03E-08	-9.81E-09	-1.11E-08	-1.08E-08	-1.09E-08	-1.06E-08	-1.05E-08	-1.07E-08	-1.05E-08	-1.04E-08	-1.03E-08	-1.08E-08	-1.13E-08	9.03E-10	-9.68E-09
-1.02E-08	-1.03E-08	-1.16E-08	-1.14E-08	-1.14E-08	-1.07E-08	-1.05E-08	-1.03E-08	-1.09E-08	-1.08E-08	-1.11E-08	-1.12E-08	-1.13E-08	3.83E-09	-9.73E-09
-1.07E-08	-1.06E-08	-1.17E-08	-1.16E-08	-1.16E-08	-1.09E-08	-1.07E-08	-1.05E-08	-1.11E-08	-1.14E-08	-1.12E-08	-1.13E-08	-1.08E-08	-9.88E-09	-1.17E-08
-1.11E-08	-1.11E-08	-1.18E-08	-1.15E-08	-1.17E-08	-1.11E-08	-1.05E-08	-1.07E-08	-1.10E-08	-1.16E-08	-1.15E-08	-1.10E-08	-1.04E-08	-1.07E-08	-1.11E-08
-1.12E-08	-1.13E-08	-1.19E-08	-1.20E-08	-1.23E-08	-1.18E-08	-1.11E-08	-1.14E-08	-1.10E-08	-1.22E-08	-1.20E-08	-1.05E-08	-1.02E-08	-1.07E-08	-1.06E-08
-1.11E-08	-1.14E-08	-1.21E-08	-1.20E-08	-1.29E-08	-1.23E-08	-1.18E-08	-1.21E-08	-1.11E-08	-1.21E-08	-1.23E-08	-1.03E-08	-1.09E-08	-1.09E-08	-1.03E-08
-1.15E-08	-1.17E-08	-1.20E-08	-1.22E-08	-1.33E-08	-1.27E-08	-1.23E-08	-1.22E-08	-1.13E-08	-1.22E-08	-1.22E-08	-1.05E-08	-1.11E-08	-1.10E-08	-1.02E-08
-1.22E-08	-1.23E-08	-1.23E-08	-1.27E-08	-1.34E-08	-1.28E-08	-1.24E-08	-1.23E-08	-1.15E-08	-1.27E-08	-1.23E-08	-1.10E-08	-1.12E-08	-1.11E-08	-1.02E-08
-1.25E-08	-1.20E-08	-1.22E-08	-1.28E-08	-1.32E-08	-1.25E-08	-1.23E-08	-1.22E-08	-1.17E-08	-1.28E-08	-1.22E-08	-1.16E-08	-1.10E-08	-1.12E-08	-1.14E-08
-1.28E-08	-1.22E-08	-1.23E-08	-1.33E-08	-1.30E-08	-1.27E-08	-1.25E-08	-1.25E-08	-1.25E-08	-1.25E-08	-1.21E-08	-1.21E-08	-1.12E-08	-1.15E-08	-1.26E-08
-1.33E-08	-1.30E-08	-1.33E-08	-1.38E-08	-1.25E-08	-1.26E-08	-1.28E-08	-1.26E-08	-1.31E-08	-1.19E-08	-1.19E-08	-1.18E-08	-1.17E-08	-1.17E-08	-1.31E-08
-1.35E-08	-1.37E-08	-1.39E-08	-1.39E-08	-1.28E-08	-1.31E-08	-1.27E-08	-1.25E-08	-1.35E-08	-1.22E-08	-1.21E-08	-1.18E-08	-1.20E-08	-1.16E-08	-1.34E-08
-1.42E-08	-1.41E-08	-1.38E-08	-1.35E-08	-1.30E-08	-1.34E-08	-1.22E-08	-1.21E-08	-1.34E-08	-1.19E-08	-1.16E-08	-1.13E-08	-1.22E-08	-1.13E-08	-1.33E-08
-1.45E-08	-1.43E-08	-1.32E-08	-1.31E-08	-1.22E-08	-1.32E-08	-1.26E-08	-1.23E-08	-1.34E-08	-1.18E-08	-1.17E-08	-1.15E-08	-1.30E-08	-1.18E-08	-1.35E-08
-1.45E-08	-1.40E-08	-1.27E-08	-1.30E-08	-1.11E-08	-1.29E-08	-1.32E-08	-1.27E-08	-1.35E-08	-1.30E-08	-1.30E-08	-1.24E-08	-1.34E-08	-1.31E-08	-1.34E-08
-1.44E-08	-1.41E-08	-1.32E-08	-1.33E-08	-1.13E-08	-1.28E-08	-1.36E-08	-1.29E-08	-1.39E-08	-1.37E-08	-1.41E-08	-1.39E-08	-1.37E-08	-1.30E-08	-1.23E-08
-1.45E-08	-1.44E-08	-1.35E-08	-1.38E-08	-1.22E-08	-1.38E-08	-1.39E-08	-1.31E-08	-1.41E-08	-1.44E-08	-1.46E-08	-1.46E-08	-1.43E-08	-1.30E-08	-1.13E-08
-1.45E-08	-1.43E-08	-1.35E-08	-1.44E-08	-1.35E-08	-1.47E-08	-1.44E-08	-1.33E-08	-1.40E-08	-1.46E-08	-1.51E-08	-1.46E-08	-1.43E-08	-1.25E-08	-1.16E-08
-1.41E-08	-1.41E-08	-1.38E-08	-1.52E-08	-1.45E-08	-1.42E-08	-1.44E-08	-1.33E-08	-1.39E-08	-1.41E-08	-1.49E-08	-1.42E-08	-1.40E-08	-1.22E-08	-1.18E-08

-1.40E-08	-1.43E-08	-1.41E-08	-1.53E-08	-1.47E-08	-1.36E-08	-1.41E-08	-1.30E-08	-1.34E-08	-1.39E-08	-1.40E-08	-1.38E-08	-1.38E-08	-1.23E-08	-1.12E-08
-1.49E-08	-1.52E-08	-1.44E-08	-1.47E-08	-1.41E-08	-1.29E-08	-1.34E-08	-1.32E-08	-1.28E-08	-1.39E-08	-1.34E-08	-1.34E-08	-1.34E-08	-1.20E-08	-1.13E-08
-1.58E-08	-1.55E-08	-1.45E-08	-1.44E-08	-1.37E-08	-1.31E-08	-1.34E-08	-1.36E-08	-1.30E-08	-1.40E-08	-1.32E-08	-1.33E-08	-1.29E-08	-1.23E-08	-1.17E-08
-1.62E-08	-1.51E-08	-1.46E-08	-1.44E-08	-1.39E-08	-1.33E-08	-1.38E-08	-1.37E-08	-1.32E-08	-1.38E-08	-1.30E-08	-1.28E-08	-1.24E-08	-1.25E-08	-1.19E-08
-1.60E-08	-1.50E-08	-1.51E-08	-1.52E-08	-1.44E-08	-1.38E-08	-1.46E-08	-1.38E-08	-1.33E-08	-1.36E-08	-1.29E-08	-1.26E-08	-1.20E-08	-1.23E-08	-1.19E-08
-1.58E-08	-1.53E-08	-1.56E-08	-1.54E-08	-1.49E-08	-1.45E-08	-1.47E-08	-1.35E-08	-1.36E-08	-1.33E-08	-1.24E-08	-1.24E-08	-1.22E-08	-1.19E-08	-1.20E-08
-1.57E-08	-1.56E-08	-1.55E-08	-1.54E-08	-1.48E-08	-1.42E-08	-1.47E-08	-1.34E-08	-1.37E-08	-1.29E-08	-1.25E-08	-1.28E-08	-1.21E-08	-1.18E-08	-1.19E-08
-1.59E-08	-1.57E-08	-1.52E-08	-1.53E-08	-1.48E-08	-1.38E-08	-1.46E-08	-1.39E-08	-1.34E-08	-1.25E-08	-1.23E-08	-1.26E-08	-1.20E-08	-1.21E-08	-1.19E-08
-1.60E-08	-1.58E-08	-1.50E-08	-1.51E-08	-1.50E-08	-1.46E-08	-1.46E-08	-1.43E-08	-1.34E-08	-1.22E-08	-1.19E-08	-1.21E-08	-1.17E-08	-1.21E-08	-1.17E-08
-1.59E-08	-1.60E-08	-1.49E-08	-1.50E-08	-1.51E-08	-1.49E-08	-1.45E-08	-1.43E-08	-1.30E-08	-1.23E-08	-1.20E-08	-1.14E-08	-1.15E-08	-1.17E-08	-1.14E-08
-1.65E-08	-1.58E-08	-1.50E-08	-1.48E-08	-1.50E-08	-1.44E-08	-1.43E-08	-1.39E-08	-1.26E-08	-1.25E-08	-1.19E-08	-1.10E-08	-1.02E-08	-1.12E-08	-1.08E-08
-1.65E-08	-1.53E-08	-1.45E-08	-1.46E-08	-1.47E-08	-1.40E-08	-1.43E-08	-1.34E-08	-1.23E-08	-1.23E-08	-1.12E-08	-1.02E-08	-9.46E-09	-1.05E-08	-9.79E-09
-1.61E-08	-1.48E-08	-1.44E-08	-1.43E-08	-1.43E-08	-1.35E-08	-1.41E-08	-1.29E-08	-1.24E-08	-1.17E-08	-1.08E-08	-9.78E-09	-9.57E-09	-1.06E-08	-9.31E-09
-1.60E-08	-1.46E-08	-1.38E-08	-1.39E-08	-1.33E-08	-1.29E-08	-1.34E-08	-1.27E-08	-1.25E-08	-1.13E-08	-1.12E-08	-1.01E-08	-9.55E-09	-1.06E-08	-8.83E-09
-1.58E-08	-1.42E-08	-1.34E-08	-1.32E-08	-1.26E-08	-1.28E-08	-1.31E-08	-1.28E-08	-1.27E-08	-1.19E-08	-1.15E-08	-1.10E-08	-9.78E-09	-9.79E-09	-8.38E-09
-1.54E-08	-1.42E-08	-1.33E-08	-1.22E-08	-1.26E-08	-1.28E-08	-1.27E-08	-1.29E-08	-1.30E-08	-1.23E-08	-1.19E-08	-1.18E-08	-1.02E-08	-9.23E-09	-7.80E-09
-1.51E-08	-1.44E-08	-1.38E-08	-1.22E-08	-1.22E-08	-1.29E-08	-1.21E-08	-1.23E-08	-1.30E-08	-1.23E-08	-1.16E-08	-1.12E-08	-1.00E-08	-9.02E-09	-7.46E-09
-1.43E-08	-1.41E-08	-1.39E-08	-1.23E-08	-1.19E-08	-1.27E-08	-1.26E-08	-1.25E-08	-1.29E-08	-1.20E-08	-1.06E-08	-1.04E-08	-9.47E-09	-8.73E-09	-7.51E-09
-1.35E-08	-1.38E-08	-1.40E-08	-1.25E-08	-1.17E-08	-1.27E-08	-1.30E-08	-1.29E-08	-1.23E-08	-1.13E-08	-9.75E-09	-9.94E-09	-9.02E-09	-8.30E-09	-7.39E-09
-1.31E-08	-1.38E-08	-1.35E-08	-1.25E-08	-1.25E-08	-1.28E-08	-1.26E-08	-1.26E-08	-1.16E-08	-1.05E-08	-9.61E-09	-9.54E-09	-8.87E-09	-8.22E-09	-7.18E-09
-1.26E-08	-1.37E-08	-1.28E-08	-1.30E-08	-1.29E-08	-1.27E-08	-1.19E-08	-1.20E-08	-1.08E-08	-9.77E-09	-9.55E-09	-8.62E-09	-8.40E-09	-7.43E-09	-6.68E-09
-1.23E-08	-1.33E-08	-1.27E-08	-1.32E-08	-1.30E-08	-1.26E-08	-1.17E-08	-1.16E-08	-1.01E-08	-9.39E-09	-9.43E-09	-7.61E-09	-7.48E-09	-6.82E-09	-6.14E-09
-1.20E-08	-1.29E-08	-1.24E-08	-1.29E-08	-1.30E-08	-1.23E-08	-1.12E-08	-1.13E-08	-9.73E-09	-9.06E-09	-8.85E-09	-7.10E-09	-7.10E-09	-6.46E-09	-6.24E-09
-1.17E-08	-1.24E-08	-1.19E-08	-1.22E-08	-1.28E-08	-1.18E-08	-1.08E-08	-1.05E-08	-9.12E-09	-8.41E-09	-8.57E-09	-7.74E-09	-7.00E-09	-6.02E-09	-6.14E-09
-1.15E-08	-1.17E-08	-1.14E-08	-1.15E-08	-1.24E-08	-1.13E-08	-1.06E-08	-1.01E-08	-9.56E-09	-8.79E-09	-8.75E-09	-7.86E-09	-7.33E-09	-6.17E-09	-5.88E-09
-1.11E-08	-1.14E-08	-1.10E-08	-1.07E-08	-1.18E-08	-1.11E-08	-1.02E-08	-9.82E-09	-1.02E-08	-9.04E-09	-8.60E-09	-7.68E-09	-7.19E-09	-5.37E-09	-5.16E-09
-1.07E-08	-1.14E-08	-1.10E-08	-1.02E-08	-1.14E-08	-1.11E-08	-9.71E-09	-9.58E-09	-1.06E-08	-9.50E-09	-8.31E-09	-8.23E-09	-7.48E-09	-4.98E-09	-4.52E-09
-1.15E-08	-1.15E-08	-1.06E-08	-1.02E-08	-1.10E-08	-1.09E-08	-1.01E-08	-9.53E-09	-1.03E-08	-9.03E-09	-8.41E-09	-8.68E-09	-7.72E-09	-4.93E-09	-4.21E-09
-1.19E-08	-1.15E-08	-1.08E-08	-1.04E-08	-1.06E-08	-1.04E-08	-1.09E-08	-9.91E-09	-1.00E-08	-8.28E-09	-7.89E-09	-8.50E-09	-7.11E-09	-5.08E-09	-5.41E-09
-1.19E-08	-1.14E-08	-1.10E-08	-1.07E-08	-1.05E-08	-9.88E-09	-1.07E-08	-1.01E-08	-9.80E-09	-7.84E-09	-7.36E-09	-7.48E-09	-5.87E-09	-4.97E-09	-6.03E-09
-1.20E-08	-1.15E-08	-1.11E-08	-1.12E-08	-1.06E-08	-9.58E-09	-1.00E-08	-1.02E-08	-9.51E-09	-7.87E-09	-7.19E-09	-7.14E-09	-6.28E-09	-5.03E-09	-6.09E-09
-1.20E-08	-1.17E-08	-1.10E-08	-1.10E-08	-1.05E-08	-9.27E-09	-9.28E-09	-9.77E-09	-9.07E-09	-8.64E-09	-7.66E-09	-6.66E-09	-6.72E-09	-4.95E-09	-6.50E-09
-1.17E-08	-1.19E-08	-1.12E-08	-1.09E-08	-1.10E-08	-9.87E-09	-9.24E-09	-9.60E-09	-8.64E-09	-9.46E-09	-8.55E-09	-5.83E-09	-6.36E-09	-5.26E-09	-6.13E-09
-1.17E-08	-1.19E-08	-1.09E-08	-1.07E-08	-1.10E-08	-1.02E-08	-9.25E-09	-9.40E-09	-8.36E-09	-8.73E-09	-8.84E-09	-5.86E-09	-6.35E-09	-5.32E-09	-6.37E-09
-1.14E-08	-1.15E-08	-1.04E-08	-1.02E-08	-1.14E-08	-1.04E-08	-9.54E-09	-9.21E-09	-9.00E-09	-8.28E-09	-8.57E-09	-7.67E-09	-7.03E-09	-6.65E-09	-6.62E-09
-1.09E-08	-1.10E-08	-9.86E-09	-1.00E-08	-1.11E-08	-1.05E-08	-9.45E-09	-8.98E-09	-8.82E-09	-8.52E-09	-8.59E-09	-7.41E-09	-7.64E-09	-7.85E-09	-6.93E-09
-1.08E-08	-1.08E-08	-9.92E-09	-9.96E-09	-1.07E-08	-1.02E-08	-8.71E-09	-8.60E-09	-8.63E-09	-8.85E-09	-8.48E-09	-7.15E-09	-7.65E-09	-8.64E-09	-7.06E-09
-1.03E-08	-1.03E-08	-1.01E-08	-1.01E-08	-1.05E-08	-9.72E-09	-8.50E-09	-8.56E-09	-8.51E-09	-9.05E-09	-8.73E-09	-8.31E-09	-8.05E-09	-8.58E-09	-7.76E-09
-1.00E-08	-1.01E-08	-1.01E-08	-9.72E-09	-1.02E-08	-9.66E-09	-9.32E-09	-9.20E-09	-8.97E-09	-9.21E-09	-8.34E-09	-8.70E-09	-8.47E-09	-8.93E-09	-8.26E-09
-9.41E-09	-1.00E-08	-9.69E-09	-9.43E-09	-1.02E-08	-9.85E-09	-9.93E-09	-9.63E-09	-9.28E-09	-9.39E-09	-8.02E-09	-9.01E-09	-8.76E-09	-8.90E-09	-8.81E-09
-9.31E-09	-9.66E-09	-9.23E-09	-9.70E-09	-1.04E-08	-1.03E-08	-1.04E-08	-9.62E-09	-9.39E-09	-9.44E-09	-7.83E-09	-9.16E-09	-8.66E-09	-8.42E-09	-8.32E-09
-1.06E-08	-9.90E-09	-9.04E-09	-9.29E-09	-1.08E-08	-9.85E-09	-1.04E-08	-1.04E-08	-9.56E-09	-9.29E-09	-8.20E-09	-9.02E-09	-8.38E-09	-8.24E-09	-7.80E-09
-1.15E-08	-9.92E-09	-9.10E-09	-9.12E-09	-1.02E-08	-9.39E-09	-1.01E-08	-1.03E-08	-9.48E-09	-9.41E-09	-8.39E-09	-8.76E-09	-8.05E-09	-8.17E-09	-7.93E-09
-1.15E-08	-1.05E-08	-9.89E-09	-9.25E-09	-9.94E-09	-9.48E-09	-1.00E-08	-1.02E-08	-9.14E-09	-9.23E-09	-8.87E-09	-8.76E-09	-8.24E-09	-8.52E-09	-7.96E-09
-1.13E-08	-1.06E-08	-1.02E-08	-9.73E-09	-9.89E-09	-9.96E-09	-9.48E-09	-9.71E-09	-8.87E-09	-8.98E-09	-9.14E-09	-8.64E-09	-8.57E-09	-8.41E-09	-7.96E-09
-1.08E-08	-1.04E-08	-1.04E-08	-1.00E-08	-9.67E-09	-1.00E-08	-9.09E-09	-8.96E-09	-8.15E-09	-8.47E-09	-8.69E-09	-8.26E-09	-8.11E-09	-8.19E-09	-7.85E-09
-9.93E-09	-1.05E-08	-1.08E-08	-1.03E-08	-9.60E-09	-9.92E-09	-8.89E-09	-8.46E-09	-7.63E-09	-8.17E-09	-8.66E-09	-7.99E-09	-7.60E-09	-8.23E-09	-8.16E-09
-9.45E-09	-1.04E-08	-1.05E-08	-1.01E-08	-9.50E-09	-9.84E-09	-8.70E-09	-8.37E-09	-8.32E-09	-8.48E-09	-9.07E-09	-7.96E-09	-7.77E-09	-8.35E-09	-8.38E-09
-9.26E-09	-9.45E-09	-1.04E-08	-9.89E-09	-9.67E-09	-1.00E-08	-8.99E-09	-8.94E-09	-8.60E-09	-8.06E-09	-9.48E-09	-7.79E-09	-7.67E-09	-8.17E-09	-8.15E-09
-9.32E-09	-9.15E-09	-1.04E-08	-9.71E-09	-9.60E-09	-9.77E-09	-8.93E-09	-8.68E-09	-8.47E-09	-8.13E-09	-8.99E-09	-8.22E-09	-7.63E-09	-7.58E-09	-7.94E-09

(16384 values):

1.67E-15	1.64E-15	1.65E-15	1.61E-15	1.65E-15	1.64E-15	1.63E-15	1.68E-15	1.58E-15	1.59E-15	1.57E-15	1.57E-15	1.59E-15	1.53E-15	1.53E-15
1.58E-15	1.55E-15	1.56E-15	1.58E-15	1.58E-15	1.58E-15	1.51E-15	1.57E-15	1.53E-15	1.58E-15	1.50E-15	1.48E-15	1.47E-15	1.40E-15	1.41E-15
1.51E-15	1.44E-15	1.47E-15	1.51E-15	1.51E-15	1.50E-15	1.42E-15	1.51E-15	1.48E-15	1.58E-15	1.46E-15	1.39E-15	1.37E-15	1.29E-15	1.31E-15
1.45E-15	1.36E-15	1.42E-15	1.43E-15	1.39E-15	1.38E-15	1.32E-15	1.44E-15	1.46E-15	1.55E-15	1.44E-15	1.32E-15	1.25E-15	1.15E-15	1.22E-15
1.38E-15	1.29E-15	1.34E-15	1.33E-15	1.26E-15	1.28E-15	1.22E-15	1.31E-15	1.37E-15	1.44E-15	1.36E-15	1.24E-15	1.14E-15	1.04E-15	1.14E-15
1.28E-15	1.21E-15	1.25E-15	1.24E-15	1.15E-15	1.19E-15	1.12E-15	1.15E-15	1.19E-15	1.27E-15	1.21E-15	1.14E-15	1.08E-15	9.61E-16	1.08E-15
1.19E-15	1.15E-15	1.18E-15	1.13E-15	1.06E-15	1.11E-15	1.04E-15	1.03E-15	1.01E-15	1.09E-15	1.05E-15	1.04E-15	9.98E-16	9.20E-16	1.01E-15
1.13E-15	1.12E-15	1.11E-15	1.07E-15	1.01E-15	1.03E-15	9.78E-16	9.51E-16	9.29E-16	9.77E-16	9.58E-16	9.43E-16	9.10E-16	8.63E-16	9.53E-16
1.09E-15	1.10E-15	1.04E-15	1.01E-15	9.56E-16	9.58E-16	9.22E-16	8.72E-16	8.63E-16	8.89E-16	8.57E-16	8.42E-16	8.28E-16	7.84E-16	8.67E-16
1.04E-15	1.03E-15	1.01E-15	9.72E-16	8.96E-16	8.98E-16	8.67E-16	8.14E-16	8.14E-16	8.19E-16	8.06E-16	7.47E-16	7.60E-16	7.09E-16	7.99E-16
9.70E-16	9.34E-16	9.17E-16	9.14E-16	8.51E-16	8.55E-16	8.16E-16	7.65E-16	7.78E-16	7.36E-16	7.61E-16	6.80E-16	6.96E-16	6.34E-16	6.65E-16
9.01E-16	8.59E-16	8.43E-16	8.26E-16	7.96E-16	7.97E-16	7.86E-16	7.03E-16	7.48E-16	6.82E-16	6.97E-16	6.33E-16	6.58E-16	5.96E-16	5.99E-16
8.27E-16	7.96E-16	7.51E-16	7.39E-16	7.23E-16	7.22E-16	7.36E-16	6.58E-16	7.08E-16	6.37E-16	6.40E-16	5.99E-16	6.22E-16	5.34E-16	5.55E-16
7.45E-16	7.19E-16	6.65E-16	6.57E-16	6.45E-16	6.37E-16	6.65E-16	6.17E-16	6.40E-16	6.18E-16	6.11E-16	5.70E-16	6.06E-16	5.09E-16	5.35E-16
6.86E-16	6.62E-16	6.05E-16	6.05E-16	5.70E-16	5.75E-16	6.02E-16	5.78E-16	5.71E-16	5.91E-16	5.80E-16	5.24E-16	5.44E-16	4.75E-16	4.90E-16
6.17E-16	6.11E-16	5.69E-16	5.45E-16	5.18E-16	5.28E-16	5.48E-16	5.31E-16	5.32E-16	5.38E-16	5.33E-16	4.54E-16	4.78E-16	4.23E-16	4.29E-16
5.52E-16	5.40E-16	4.98E-16	4.81E-16	4.71E-16	4.85E-16	5.01E-16	4.78E-16	4.81E-16	4.84E-16	4.49E-16	4.24E-16	4.52E-16	3.99E-16	3.66E-16
5.02E-16	5.16E-16	4.60E-16	4.54E-16	4.38E-16	4.31E-16	4.56E-16	4.39E-16	4.15E-16	4.25E-16	3.83E-16	4.01E-16	4.25E-16	3.85E-16	3.02E-16
4.64E-16	4.92E-16	4.28E-16	4.35E-16	4.02E-16	3.94E-16	4.00E-16	3.99E-16	3.74E-16	3.75E-16	3.46E-16	3.63E-16	3.87E-16	3.61E-16	2.70E-16
4.46E-16	4.52E-16	3.90E-16	3.95E-16	3.89E-16	3.76E-16	3.76E-16	3.67E-16	3.46E-16	3.42E-16	3.17E-16	3.22E-16	3.33E-16	3.14E-16	2.40E-16
4.15E-16	4.07E-16	3.48E-16	3.65E-16	3.50E-16	3.30E-16	3.47E-16	3.38E-16	3.19E-16	3.09E-16	2.90E-16	2.77E-16	2.79E-16	2.54E-16	2.06E-16
3.64E-16	3.71E-16	3.22E-16	3.23E-16	3.01E-16	2.76E-16	2.95E-16	3.12E-16	2.82E-16	2.74E-16	2.63E-16	2.50E-16	2.37E-16	2.02E-16	1.88E-16
3.25E-16	3.26E-16	2.97E-16	2.80E-16	2.63E-16	2.32E-16	2.43E-16	2.64E-16	2.46E-16	2.42E-16	2.32E-16	2.27E-16	1.95E-16	1.67E-16	1.55E-16
2.85E-16	2.87E-16	2.82E-16	2.46E-16	2.33E-16	2.08E-16	2.08E-16	2.07E-16	2.20E-16	2.21E-16	2.22E-16	2.19E-16	1.68E-16	1.45E-16	1.40E-16
2.57E-16	2.56E-16	2.62E-16	2.21E-16	2.03E-16	1.81E-16	1.74E-16	1.77E-16	2.04E-16	1.90E-16	1.95E-16	1.94E-16	1.53E-16	1.28E-16	1.25E-16
2.25E-16	2.12E-16	2.07E-16	1.85E-16	1.76E-16	1.68E-16	1.49E-16	1.65E-16	1.89E-16	1.57E-16	1.78E-16	1.83E-16	1.38E-16	1.18E-16	1.14E-16
2.00E-16	1.78E-16	1.60E-16	1.65E-16	1.50E-16	1.50E-16	1.40E-16	1.51E-16	1.60E-16	1.33E-16	1.44E-16	1.35E-16	1.10E-16	1.08E-16	8.74E-17
1.69E-16	1.57E-16	1.32E-16	1.36E-16	1.28E-16	1.34E-16	1.34E-16	1.42E-16	1.35E-16	1.10E-16	1.11E-16	1.05E-16	8.76E-17	9.90E-17	6.34E-17
1.45E-16	1.36E-16	1.13E-16	1.12E-16	1.13E-16	1.14E-16	1.28E-16	1.13E-16	9.20E-17	8.73E-17	7.98E-17	8.74E-17	7.08E-17	6.06E-17	4.68E-17
1.24E-16	1.08E-16	9.81E-17	1.04E-16	1.07E-16	1.02E-16	1.03E-16	7.85E-17	6.67E-17	6.94E-17	6.78E-17	7.13E-17	5.62E-17	5.08E-17	3.90E-17
1.02E-16	8.27E-17	8.73E-17	9.11E-17	8.64E-17	8.34E-17	7.86E-17	6.65E-17	4.99E-17	5.45E-17	5.57E-17	5.22E-17	4.58E-17	3.65E-17	3.74E-17

7.79E-17	6.63E-17	7.70E-17	7.04E-17	6.32E-17	7.11E-17	6.66E-17	6.17E-17	4.60E-17	4.33E-17	4.06E-17	3.93E-17	3.26E-17	2.23E-17	2.92E-17
6.18E-17	5.39E-17	6.03E-17	5.06E-17	4.77E-17	6.71E-17	6.25E-17	5.72E-17	3.90E-17	3.03E-17	2.73E-17	2.99E-17	1.91E-17	1.27E-17	2.34E-17
5.15E-17	3.82E-17	4.26E-17	3.76E-17	3.69E-17	4.98E-17	5.31E-17	5.09E-17	3.66E-17	2.42E-17	2.29E-17	2.56E-17	1.14E-17	7.87E-18	1.42E-17
3.47E-17	2.49E-17	2.94E-17	2.81E-17	3.34E-17	3.42E-17	3.53E-17	3.80E-17	2.96E-17	1.95E-17	1.95E-17	2.00E-17	6.52E-18	3.27E-18	9.08E-18
2.13E-17	1.62E-17	1.57E-17	2.12E-17	2.20E-17	2.40E-17	2.61E-17	2.88E-17	1.44E-17	1.36E-17	1.28E-17	9.73E-18	4.37E-18	1.83E-18	7.16E-18
1.37E-17	9.95E-18	9.87E-18	1.49E-17	1.32E-17	1.50E-17	1.51E-17	2.11E-17	5.95E-18	5.40E-18	4.92E-18	3.55E-18	1.95E-18	8.43E-19	4.48E-18
9.89E-18	5.76E-18	8.89E-18	1.12E-17	1.00E-17	1.10E-17	5.59E-18	9.67E-18	3.88E-18	2.69E-18	1.58E-18	5.90E-19	2.13E-19	2.66E-19	1.13E-18
5.42E-18	3.27E-18	7.47E-18	9.92E-18	8.76E-18	7.78E-18	2.73E-18	3.02E-18	2.89E-18	1.45E-18	9.88E-19	7.22E-20	6.55E-21	3.89E-19	1.96E-19
1.37E-18	1.79E-18	5.57E-18	8.75E-18	6.20E-18	4.14E-18	1.63E-18	1.00E-18	9.56E-19	3.96E-19	3.72E-19	2.31E-19	5.90E-19	1.76E-18	3.25E-19
4.83E-19	5.25E-20	1.51E-18	4.29E-18	2.75E-18	7.62E-19	2.47E-19	1.70E-19	1.33E-19	1.59E-19	3.74E-20	1.59E-18	2.49E-18	2.96E-18	2.01E-18
1.64E-22	8.21E-19	2.39E-20	1.10E-20	6.02E-19	4.59E-21	6.43E-20	1.37E-20	1.48E-19	1.44E-19	5.10E-19	5.06E-18	3.76E-18	5.79E-18	5.91E-18
4.64E-19	2.52E-18	1.38E-18	9.86E-19	3.59E-19	1.36E-18	1.81E-18	1.67E-18	2.44E-18	2.47E-18	3.81E-18	1.23E-17	8.82E-18	1.07E-17	7.83E-18
1.47E-18	4.00E-18	2.79E-18	2.86E-18	3.24E-18	4.44E-18	4.02E-18	6.72E-18	7.39E-18	6.91E-18	9.53E-18	1.79E-17	1.17E-17	1.60E-17	9.85E-18
5.21E-18	5.83E-18	6.21E-18	5.78E-18	7.12E-18	7.94E-18	9.09E-18	1.43E-17	1.24E-17	1.27E-17	1.73E-17	2.24E-17	1.98E-17	2.04E-17	1.26E-17
1.30E-17	6.64E-18	9.70E-18	8.50E-18	9.55E-18	9.07E-18	1.34E-17	2.13E-17	1.85E-17	1.98E-17	2.72E-17	2.72E-17	2.32E-17	2.32E-17	1.76E-17
1.53E-17	7.95E-18	1.16E-17	1.21E-17	1.25E-17	1.65E-17	1.97E-17	2.59E-17	2.56E-17	2.35E-17	3.44E-17	3.20E-17	2.89E-17	2.87E-17	2.33E-17
1.71E-17	1.11E-17	1.50E-17	1.42E-17	1.21E-17	1.62E-17	2.17E-17	2.93E-17	3.02E-17	2.81E-17	3.62E-17	3.43E-17	3.37E-17	3.67E-17	2.80E-17
2.04E-17	1.77E-17	1.74E-17	1.56E-17	1.25E-17	1.58E-17	2.15E-17	3.42E-17	3.19E-17	2.96E-17	3.86E-17	4.11E-17	4.27E-17	4.13E-17	3.27E-17
2.97E-17	2.13E-17	2.06E-17	1.79E-17	8.75E-18	1.30E-17	1.97E-17	3.72E-17	3.14E-17	3.47E-17	3.91E-17	5.01E-17	4.43E-17	5.01E-17	3.71E-17
3.42E-17	1.88E-17	1.97E-17	1.52E-17	5.87E-18	1.12E-17	1.71E-17	3.64E-17	3.35E-17	4.39E-17	4.05E-17	5.07E-17	4.28E-17	5.98E-17	4.75E-17
3.63E-17	1.87E-17	2.01E-17	1.31E-17	5.55E-18	8.65E-18	1.80E-17	3.75E-17	3.63E-17	6.54E-17	4.29E-17	4.71E-17	4.55E-17	6.77E-17	5.28E-17
4.06E-17	2.25E-17	2.35E-17	1.57E-17	8.22E-18	9.94E-18	1.86E-17	4.04E-17	5.33E-17	1.02E-16	4.83E-17	4.39E-17	4.61E-17	6.23E-17	5.19E-17
5.29E-17	3.72E-17	3.98E-17	3.30E-17	2.43E-17	2.47E-17	3.22E-17	4.30E-17	6.24E-17	1.10E-16	5.70E-17	4.76E-17	5.08E-17	6.90E-17	6.06E-17
6.56E-17	4.49E-17	5.68E-17	5.68E-17	4.29E-17	4.49E-17	4.73E-17	4.60E-17	5.24E-17	6.11E-17	4.54E-17	5.52E-17	7.23E-17	8.17E-17	7.38E-17
7.13E-17	5.10E-17	6.84E-17	7.26E-17	5.92E-17	6.72E-17	6.43E-17	6.62E-17	5.35E-17	4.95E-17	5.62E-17	7.21E-17	8.72E-17	9.63E-17	8.64E-17
8.26E-17	6.42E-17	7.90E-17	8.11E-17	7.77E-17	8.33E-17	7.79E-17	8.60E-17	6.96E-17	6.98E-17	7.92E-17	8.67E-17	1.01E-16	8.81E-17	9.92E-17
9.38E-17	7.94E-17	9.09E-17	9.69E-17	9.07E-17	9.84E-17	8.76E-17	1.02E-16	9.16E-17	8.44E-17	9.22E-17	9.84E-17	1.12E-16	7.54E-17	1.12E-16
1.01E-16	9.32E-17	1.07E-16	1.12E-16	1.07E-16	1.07E-16	1.09E-16	1.13E-16	1.02E-16	9.79E-17	9.91E-17	1.04E-16	1.19E-16	2.63E-17	1.08E-16
1.06E-16	9.62E-17	1.24E-16	1.16E-16	1.18E-16	1.12E-16	1.11E-16	1.14E-16	1.11E-16	1.07E-16	1.05E-16	1.17E-16	1.27E-16	8.16E-19	9.37E-17
1.05E-16	1.07E-16	1.33E-16	1.30E-16	1.31E-16	1.15E-16	1.10E-16	1.06E-16	1.20E-16	1.16E-16	1.23E-16	1.24E-16	1.28E-16	1.47E-17	9.47E-17
1.15E-16	1.13E-16	1.37E-16	1.36E-16	1.34E-16	1.19E-16	1.15E-16	1.11E-16	1.22E-16	1.30E-16	1.25E-16	1.28E-16	1.17E-16	9.76E-17	1.37E-16
1.24E-16	1.23E-16	1.40E-16	1.33E-16	1.38E-16	1.24E-16	1.10E-16	1.15E-16	1.22E-16	1.35E-16	1.31E-16	1.21E-16	1.07E-16	1.14E-16	1.24E-16
1.25E-16	1.28E-16	1.43E-16	1.43E-16	1.51E-16	1.38E-16	1.22E-16	1.30E-16	1.22E-16	1.49E-16	1.45E-16	1.10E-16	1.04E-16	1.14E-16	1.13E-16
1.23E-16	1.30E-16	1.46E-16	1.44E-16	1.67E-16	1.53E-16	1.39E-16	1.47E-16	1.22E-16	1.47E-16	1.51E-16	1.06E-16	1.20E-16	1.19E-16	1.07E-16
1.33E-16	1.37E-16	1.44E-16	1.49E-16	1.77E-16	1.62E-16	1.51E-16	1.50E-16	1.28E-16	1.50E-16	1.48E-16	1.10E-16	1.23E-16	1.22E-16	1.05E-16
1.48E-16	1.51E-16	1.51E-16	1.60E-16	1.80E-16	1.65E-16	1.54E-16	1.50E-16	1.33E-16	1.62E-16	1.51E-16	1.21E-16	1.26E-16	1.23E-16	1.03E-16
1.56E-16	1.44E-16	1.48E-16	1.64E-16	1.75E-16	1.55E-16	1.51E-16	1.50E-16	1.36E-16	1.64E-16	1.49E-16	1.34E-16	1.22E-16	1.26E-16	1.29E-16
1.65E-16	1.49E-16	1.51E-16	1.76E-16	1.68E-16	1.62E-16	1.56E-16	1.56E-16	1.55E-16	1.56E-16	1.46E-16	1.47E-16	1.26E-16	1.33E-16	1.59E-16
1.76E-16	1.68E-16	1.78E-16	1.91E-16	1.56E-16	1.59E-16	1.63E-16	1.58E-16	1.71E-16	1.43E-16	1.40E-16	1.39E-16	1.36E-16	1.38E-16	1.71E-16
1.82E-16	1.88E-16	1.94E-16	1.93E-16	1.63E-16	1.73E-16	1.60E-16	1.55E-16	1.83E-16	1.49E-16	1.46E-16	1.39E-16	1.43E-16	1.34E-16	1.79E-16
2.02E-16	1.99E-16	1.90E-16	1.83E-16	1.68E-16	1.81E-16	1.49E-16	1.46E-16	1.79E-16	1.41E-16	1.34E-16	1.27E-16	1.49E-16	1.29E-16	1.78E-16
2.10E-16	2.03E-16	1.75E-16	1.72E-16	1.49E-16	1.74E-16	1.58E-16	1.52E-16	1.79E-16	1.40E-16	1.37E-16	1.32E-16	1.68E-16	1.40E-16	1.81E-16
2.11E-16	1.95E-16	1.61E-16	1.69E-16	1.24E-16	1.67E-16	1.75E-16	1.62E-16	1.82E-16	1.68E-16	1.70E-16	1.53E-16	1.79E-16	1.71E-16	1.79E-16
2.07E-16	1.99E-16	1.73E-16	1.77E-16	1.27E-16	1.65E-16	1.84E-16	1.66E-16	1.93E-16	1.87E-16	2.00E-16	1.92E-16	1.88E-16	1.70E-16	1.51E-16
2.10E-16	2.07E-16	1.83E-16	1.90E-16	1.49E-16	1.90E-16	1.92E-16	1.73E-16	1.99E-16	2.09E-16	2.14E-16	2.14E-16	2.03E-16	1.68E-16	1.27E-16
2.10E-16	2.06E-16	1.83E-16	2.07E-16	1.83E-16	2.15E-16	2.06E-16	1.77E-16	1.96E-16	2.12E-16	2.29E-16	2.12E-16	2.05E-16	1.56E-16	1.34E-16
1.99E-16	2.00E-16	1.90E-16	2.31E-16	2.11E-16	2.02E-16	2.09E-16	1.77E-16	1.94E-16	1.99E-16	2.22E-16	2.03E-16	1.97E-16	1.49E-16	1.38E-16
1.97E-16	2.06E-16	1.99E-16	2.33E-16	2.15E-16	1.86E-16	1.98E-16	1.70E-16	1.78E-16	1.93E-16	1.96E-16	1.90E-16	1.90E-16	1.50E-16	1.26E-16
2.21E-16	2.30E-16	2.08E-16	2.17E-16	1.98E-16	1.66E-16	1.80E-16	1.75E-16	1.63E-16	1.94E-16	1.79E-16	1.79E-16	1.79E-16	1.45E-16	1.29E-16
2.50E-16	2.42E-16	2.10E-16	2.08E-16	1.87E-16	1.71E-16	1.81E-16	1.85E-16	1.70E-16	1.97E-16	1.74E-16	1.78E-16	1.66E-16	1.52E-16	1.37E-16
2.63E-16	2.29E-16	2.13E-16	2.08E-16	1.93E-16	1.78E-16	1.90E-16	1.87E-16	1.75E-16	1.90E-16	1.68E-16	1.63E-16	1.54E-16	1.56E-16	1.41E-16
2.57E-16	2.25E-16	2.27E-16	2.32E-16	2.07E-16	1.91E-16	2.12E-16	1.91E-16	1.77E-16	1.85E-16	1.66E-16	1.58E-16	1.44E-16	1.52E-16	1.43E-16
2.51E-16	2.35E-16	2.42E-16	2.38E-16	2.21E-16	2.11E-16	2.16E-16	1.83E-16	1.84E-16	1.78E-16	1.54E-16	1.53E-16	1.48E-16	1.41E-16	1.45E-16
2.47E-16	2.44E-16	2.40E-16	2.38E-16	2.18E-16	2.00E-16	2.17E-16	1.81E-16	1.87E-16	1.66E-16	1.57E-16	1.63E-16	1.46E-16	1.40E-16	1.43E-16
2.51E-16	2.46E-16	2.32E-16	2.34E-16	2.19E-16	1.91E-16	2.12E-16	1.92E-16	1.78E-16	1.57E-16	1.50E-16	1.58E-16	1.44E-16	1.45E-16	1.42E-16
2.54E-16	2.50E-16	2.26E-16	2.27E-16	2.24E-16	2.12E-16	2.13E-16	2.06E-16	1.79E-16	1.50E-16	1.41E-16	1.46E-16	1.37E-16	1.46E-16	1.37E-16
2.52E-16	2.56E-16	2.23E-16	2.25E-16	2.28E-16	2.22E-16	2.11E-16	2.04E-16	1.69E-16	1.50E-16	1.44E-16	1.31E-16	1.31E-16	1.38E-16	1.30E-16
2.74E-16	2.49E-16	2.24E-16	2.18E-16	2.24E-16	2.06E-16	2.04E-16	1.92E-16	1.58E-16	1.56E-16	1.42E-16	1.20E-16	1.04E-16	1.25E-16	1.16E-16
2.73E-16	2.35E-16	2.09E-16	2.14E-16	2.16E-16	1.96E-16	2.06E-16	1.80E-16	1.52E-16	1.50E-16	1.25E-16	1.04E-16	8.95E-17	1.10E-16	9.58E-17
2.61E-16	2.20E-16	2.08E-16	2.05E-16	2.03E-16	1.83E-16	1.98E-16	1.67E-16	1.53E-16	1.38E-16	1.16E-16	9.56E-17	9.17E-17	1.12E-16	8.67E-17
2.56E-16	2.13E-16	1.91E-16	1.92E-16	1.76E-16	1.67E-16	1.79E-16	1.61E-16	1.57E-16	1.29E-16	1.25E-16	1.01E-16	9.12E-17	1.13E-16	7.80E-17
2.49E-16	2.01E-16	1.80E-16	1.73E-16	1.58E-16	1.64E-16	1.73E-16	1.65E-16	1.60E-16	1.41E-16	1.33E-16	1.22E-16	9.57E-17	9.59E-17	7.01E-17
2.37E-16	2.00E-16	1.77E-16	1.50E-16	1.59E-16	1.65E-16	1.62E-16	1.66E-16	1.68E-16	1.51E-16	1.41E-16	1.38E-16	1.04E-16	8.52E-17	6.08E-17
2.27E-16	2.07E-16	1.92E-16	1.49E-16	1.48E-16	1.68E-16	1.47E-16	1.51E-16	1.69E-16	1.51E-16	1.35E-16	1.26E-16	1.01E-16	8.14E-17	5.57E-17
2.04E-16	1.99E-16	1.93E-16	1.52E-16	1.42E-16	1.61E-16	1.58E-16	1.55E-16	1.66E-16	1.43E-16	1.11E-16	1.09E-16	8.97E-17	7.63E-17	5.64E-17
1.83E-16	1.91E-16	1.97E-16	1.57E-16	1.36E-16	1.62E-16	1.70E-16	1.67E-16	1.52E-16	1.27E-16	9.50E-17	9.88E-17	8.14E-17	6.89E-17	5.46E-17
1.71E-16	1.91E-16	1.82E-16	1.57E-16	1.57E-16	1.64E-16	1.58E-16	1.59E-16	1.36E-16	1.10E-16	9.24E-17	9.10E-17	7.87E-17	6.75E-17	5.16E-17
1.59E-16	1.88E-16	1.64E-16	1.69E-16	1.66E-16	1.62E-16	1.41E-16	1.44E-16	1.16E-16	9.54E-17	9.13E-17	7.42E-17	7.06E-17	5.52E-17	4.47E-17
1.52E-16	1.76E-16	1.62E-16	1.74E-16	1.70E-16	1.58E-16	1.38E-16	1.34E-16	1.02E-16	8.82E-17	8.90E-17	5.80E-17	5.59E-17	4.65E-17	3.77E-17
1.43E-16	1.67E-16	1.54E-16	1.67E-16	1.69E-16	1.51E-16	1.25E-16	1.27E-16	9.47E-17	8.21E-17	7.83E-17	5.04E-17	5.04E-17	4.17E-17	3.89E-17
1.36E-16	1.55E-16	1.42E-16	1.49E-16	1.64E-16	1.40E-16	1.17E-16	1.10E-16	8.31E-17	7.08E-17	7.35E-17	5.99E-17	4.91E-17	3.63E-17	3.77E-17
1.32E-16	1.37E-16	1.30E-16	1.32E-16	1.53E-16	1.28E-16	1.11E-16	1.01E-16	9.14E-17	7.73E-17	7.65E-17	6.18E-17	5.37E-17	3.81E-17	3.46E-17
1.24E-16	1.30E-16	1.20E-16	1.14E-16	1.39E-16	1.24E-16	1.05E-16	9.63E-17	1.04E-16	8.17E-17	7.39E-17	5.90E-17	5.17E-17	2.88E-17	2.66E-17
1.15E-16	1.31E-16	1.21E-16	1.04E-16	1.30E-16	1.22E-16	9.43E-17	9.17E-17	1.13E-16	9.03E-17	6.91E-17	6.77E-17	5.60E-17	2.48E-17	2.04E-17
1.31E-16	1.32E-16	1.12E-16	1.05E-16	1.22E-16	1.19E-16	1.01E-16	9.08E-17	1.07E-16	8.15E-17	7.07E-17	7.53E-17	5.97E-17	2.43E-17	1.77E-17
1.42E-16	1.33E-16	1.16E-16	1.08E-16	1.13E-16	1.09E-16	1.20E-16	9.82E-17	1.01E-16	6.85E-17	6.22E-17	7.23E-17	5.05E-17	2.58E-17	2.92E-17
1.40E-16	1.30E-16	1.22E-16	1.14E-16	1.10E-16	9.77E-17	1.15E-16	1.02E-16	9.61E-17	6.14E-17	5.41E-17	5.59E-17	3.44E-17	2.47E-17	3.64E-17
1.45E-16	1.32E-16	1.23E-16	1.27E-16	1.13E-16	9.18E-17	1.00E-16	1.03E-16	9.05E-17	6.19E-17	5.17E-17	5.09E-17	3.94E-17	2.53E-17	3.70E-17
1.44E-16	1.36E-16	1.22E-16	1.21E-16	1.10E-16	8.60E-17	8.61E-17	9.54E-17	8.23E-17	7.46E-17	5.87E-17	4.43E-17	4.52E-17	2.45E-17	4.23E-17
1.37E-16	1.41E-16	1.26E-16	1.20E-16	1.22E-16	9.74E-17	8.54E-17	9.22E-17	7.47E-17	8.95E-17	7.30E-17	3.40E-17	4.05E-17	2.77E-17	3.75E-17
1.37E-16	1.42E-16	1.19E-16	1.14E-16	1.22E-16	1.04E-16	8.55E-17	8.84E-17	6.98E-17	7.63E-17	7.81E-17	3.43E-17	4.03E-17	2.83E-17	4.06E-17
1.29E-16	1.32E-16	1.07E-16	1.04E-16	1.30E-16	1.08E-16	9.10E-17	8.48E-17	8.09E-17	6.86E-17	7.34E-17	5.88E-17	4.94E-17	4.42E-17	4.38E-17
1.20E-16	1.20E-16	9.73E-17	1.01E-16	1.24E-16	1.11E-16	8.93E-17	8.06E-17	7.78E-17	7.27E-17	7.38E-17	5.49E-17	5.84E-17	6.17E-17	4.80E-17
1.17E-16	1.17E-16	9.84E-17	9.92E-17	1.15E-16	1.04E-16	7.59E-17	7.40E-17	7.45E-17	7.83E-17	7.19E-17	5.11E-17	5.86E-17	7.46E-17	4.98E-17
1.05E-16	1.06E-16	1.03E-16	1.03E-16	1.09E-16	9.45E-17	7.22E-17	7.33E-17	7.24E-17	8.18E-17	7.62E-17	6.91E-17	6.48E-17	7.36E-17	6.03E-17
9.99E-17	1.01E-16	1.03E-16	9.44E-17	1.04E-16	9.33E-17	8.69E-17	8.47E-17	8.05E-17	8.49E-17	6.96E-17	7.57E-17	7.17E-17	7.97E-17	6.82E-17

8.85E-17	1.01E-16	9.39E-17	8.90E-17	1.05E-16	9.70E-17	9.86E-17	9.27E-17	8.62E-17	8.82E-17	6.43E-17	8.11E-17	7.68E-17	7.92E-17	7.75E-17
8.67E-17	9.34E-17	8.52E-17	9.40E-17	1.08E-16	1.05E-16	1.08E-16	9.26E-17	8.82E-17	8.92E-17	6.13E-17	8.39E-17	7.49E-17	7.09E-17	6.92E-17
1.13E-16	9.80E-17	8.17E-17	8.63E-17	1.17E-16	9.71E-17	1.08E-16	1.08E-16	9.14E-17	8.64E-17	6.72E-17	8.14E-17	7.02E-17	6.78E-17	6.08E-17
1.31E-16	9.84E-17	8.29E-17	8.31E-17	1.03E-16	8.82E-17	1.03E-16	1.06E-16	8.98E-17	8.86E-17	7.05E-17	7.68E-17	6.49E-17	6.68E-17	6.28E-17
1.32E-16	1.09E-16	9.78E-17	8.55E-17	9.87E-17	9.00E-17	1.00E-16	1.03E-16	8.36E-17	8.52E-17	7.86E-17	7.68E-17	6.78E-17	7.26E-17	6.34E-17
1.29E-16	1.12E-16	1.03E-16	9.46E-17	9.79E-17	9.91E-17	8.99E-17	9.44E-17	7.87E-17	8.06E-17	8.36E-17	7.46E-17	7.35E-17	7.07E-17	6.33E-17
1.16E-16	1.08E-16	1.08E-16	1.00E-16	9.34E-17	1.00E-16	8.26E-17	8.02E-17	6.64E-17	7.17E-17	7.55E-17	6.83E-17	6.58E-17	6.72E-17	6.17E-17
9.87E-17	1.10E-16	1.16E-16	1.07E-16	9.22E-17	9.84E-17	7.91E-17	7.15E-17	5.82E-17	6.67E-17	7.50E-17	6.39E-17	5.78E-17	6.78E-17	6.66E-17
8.93E-17	1.08E-16	1.10E-16	1.03E-16	9.02E-17	9.69E-17	7.56E-17	7.01E-17	6.93E-17	7.20E-17	8.23E-17	6.34E-17	6.04E-17	6.98E-17	7.02E-17
8.58E-17	8.93E-17	1.08E-16	9.79E-17	9.35E-17	1.00E-16	8.09E-17	8.00E-17	7.40E-17	6.50E-17	8.99E-17	6.08E-17	5.89E-17	6.67E-17	6.65E-17
8.68E-17	8.37E-17	1.08E-16	9.44E-17	9.22E-17	9.55E-17	7.98E-17	7.54E-17	7.17E-17	6.61E-17	8.07E-17	6.75E-17	5.82E-17	5.75E-17	6.30E-17
3.52E-14	3.39E-14	3.30E-14	3.25E-14	3.15E-14	3.11E-14	3.04E-14	3.01E-14	2.95E-14	2.95E-14	2.83E-14	2.68E-14	2.61E-14	2.38E-14	2.41E-14

3.86E-08	3.82E-08	3.71E-08	3.67E-08	3.60E-08	3.56E-08	3.50E-08	3.49E-08	3.48E-08	3.41E-08	3.40E-08	3.38E-08	3.35E-08	3.30E-08	3.28E-08
3.70E-08	3.68E-08	3.60E-08	3.54E-08	3.47E-08	3.42E-08	3.39E-08	3.34E-08	3.34E-08	3.28E-08	3.28E-08	3.23E-08	3.23E-08	3.16E-08	3.15E-08
3.56E-08	3.52E-08	3.48E-08	3.41E-08	3.36E-08	3.28E-08	3.30E-08	3.26E-08	3.24E-08	3.19E-08	3.18E-08	3.11E-08	3.12E-08	3.05E-08	3.05E-08
3.42E-08	3.37E-08	3.33E-08	3.30E-08	3.26E-08	3.16E-08	3.17E-08	3.14E-08	3.12E-08	3.10E-08	3.08E-08	3.01E-08	3.00E-08	2.95E-08	2.93E-08
3.29E-08	3.23E-08	3.18E-08	3.16E-08	3.10E-08	3.02E-08	3.00E-08	3.04E-08	3.00E-08	2.96E-08	2.94E-08	2.84E-08	2.82E-08	2.85E-08	2.82E-08
3.16E-08	3.13E-08	3.08E-08	3.06E-08	2.97E-08	2.87E-08	2.90E-08	2.95E-08	2.89E-08	2.82E-08	2.79E-08	2.69E-08	2.70E-08	2.75E-08	2.70E-08
3.07E-08	3.02E-08	2.96E-08	2.92E-08	2.85E-08	2.78E-08	2.84E-08	2.87E-08	2.80E-08	2.68E-08	2.64E-08	2.60E-08	2.59E-08	2.64E-08	2.61E-08
2.96E-08	2.86E-08	2.83E-08	2.78E-08	2.71E-08	2.69E-08	2.80E-08	2.83E-08	2.68E-08	2.57E-08	2.51E-08	2.47E-08	2.48E-08	2.57E-08	2.54E-08
2.83E-08	2.72E-08	2.72E-08	2.66E-08	2.59E-08	2.58E-08	2.78E-08	2.77E-08	2.54E-08	2.45E-08	2.39E-08	2.34E-08	2.40E-08	2.47E-08	2.43E-08
2.65E-08	2.57E-08	2.59E-08	2.54E-08	2.49E-08	2.49E-08	2.72E-08	2.55E-08	2.41E-08	2.37E-08	2.29E-08	2.25E-08	2.36E-08	2.37E-08	2.32E-08
2.55E-08	2.45E-08	2.47E-08	2.45E-08	2.42E-08	2.42E-08	2.54E-08	2.32E-08	2.26E-08	2.29E-08	2.23E-08	2.21E-08	2.32E-08	2.30E-08	2.25E-08
2.49E-08	2.37E-08	2.42E-08	2.38E-08	2.37E-08	2.33E-08	2.26E-08	2.14E-08	2.09E-08	2.16E-08	2.18E-08	2.16E-08	2.19E-08	2.18E-08	2.17E-08
2.44E-08	2.35E-08	2.40E-08	2.35E-08	2.28E-08	2.26E-08	2.07E-08	1.98E-08	1.96E-08	1.98E-08	2.06E-08	2.05E-08	2.07E-08	2.06E-08	2.02E-08
2.44E-08	2.33E-08	2.36E-08	2.29E-08	2.18E-08	2.16E-08	1.94E-08	1.85E-08	1.84E-08	1.83E-08	1.91E-08	1.94E-08	1.95E-08	1.88E-08	1.87E-08
2.35E-08	2.30E-08	2.29E-08	2.11E-08	2.04E-08	2.00E-08	1.82E-08	1.75E-08	1.76E-08	1.74E-08	1.75E-08	1.81E-08	1.81E-08	1.72E-08	1.73E-08
2.17E-08	2.15E-08	2.08E-08	1.91E-08	1.89E-08	1.83E-08	1.71E-08	1.61E-08	1.66E-08	1.63E-08	1.61E-08	1.72E-08	1.65E-08	1.61E-08	1.55E-08
1.96E-08	1.94E-08	1.87E-08	1.77E-08	1.73E-08	1.67E-08	1.57E-08	1.49E-08	1.58E-08	1.56E-08	1.53E-08	1.63E-08	1.49E-08	1.46E-08	1.39E-08
1.77E-08	1.76E-08	1.67E-08	1.62E-08	1.60E-08	1.53E-08	1.50E-08	1.41E-08	1.47E-08	1.49E-08	1.47E-08	1.56E-08	1.41E-08	1.37E-08	1.29E-08
1.59E-08	1.60E-08	1.56E-08	1.55E-08	1.49E-08	1.41E-08	1.39E-08	1.36E-08	1.38E-08	1.40E-08	1.35E-08	1.46E-08	1.30E-08	1.24E-08	1.22E-08
1.45E-08	1.49E-08	1.40E-08	1.44E-08	1.36E-08	1.29E-08	1.26E-08	1.30E-08	1.28E-08	1.30E-08	1.23E-08	1.28E-08	1.20E-08	1.13E-08	1.16E-08
1.34E-08	1.43E-08	1.28E-08	1.33E-08	1.26E-08	1.18E-08	1.14E-08	1.21E-08	1.18E-08	1.19E-08	1.10E-08	1.11E-08	1.07E-08	1.07E-08	1.12E-08
1.26E-08	1.39E-08	1.20E-08	1.20E-08	1.18E-08	1.12E-08	1.06E-08	1.07E-08	1.02E-08	1.04E-08	9.98E-09	9.99E-09	9.89E-09	1.01E-08	1.09E-08
1.22E-08	1.30E-08	1.14E-08	1.11E-08	1.11E-08	1.06E-08	9.64E-09	8.50E-09	8.58E-09	8.87E-09	8.82E-09	9.20E-09	9.24E-09	9.28E-09	9.74E-09
1.14E-08	1.19E-08	1.10E-08	1.14E-08	1.03E-08	9.46E-09	8.64E-09	7.62E-09	7.24E-09	7.20E-09	7.64E-09	8.03E-09	8.23E-09	8.29E-09	8.36E-09
1.07E-08	1.07E-08	1.08E-08	1.14E-08	9.71E-09	8.42E-09	7.83E-09	7.26E-09	6.46E-09	6.32E-09	6.71E-09	7.07E-09	6.96E-09	7.22E-09	7.68E-09
1.03E-08	9.85E-09	1.05E-08	1.06E-08	8.33E-09	7.78E-09	6.96E-09	6.58E-09	5.91E-09	5.58E-09	5.73E-09	6.44E-09	5.88E-09	5.81E-09	6.43E-09
9.41E-09	9.45E-09	9.49E-09	9.20E-09	7.05E-09	7.36E-09	6.38E-09	5.28E-09	5.37E-09	4.68E-09	4.97E-09	5.13E-09	4.60E-09	4.85E-09	5.15E-09
7.68E-09	8.81E-09	8.69E-09	8.09E-09	5.83E-09	6.78E-09	5.65E-09	4.34E-09	4.61E-09	3.95E-09	4.17E-09	4.01E-09	3.83E-09	4.14E-09	4.27E-09
6.49E-09	7.70E-09	7.91E-09	7.35E-09	5.82E-09	5.93E-09	4.83E-09	4.13E-09	4.20E-09	3.62E-09	3.60E-09	3.49E-09	3.25E-09	3.11E-09	3.53E-09
5.88E-09	6.07E-09	6.49E-09	6.37E-09	5.64E-09	4.48E-09	3.62E-09	3.51E-09	3.41E-09	3.28E-09	2.70E-09	2.66E-09	2.56E-09	2.48E-09	2.68E-09
5.71E-09	5.49E-09	5.60E-09	5.41E-09	5.11E-09	3.49E-09	2.77E-09	2.76E-09	2.67E-09	2.79E-09	1.83E-09	2.11E-09	2.01E-09	2.26E-09	2.11E-09
5.42E-09	4.45E-09	4.71E-09	4.59E-09	4.53E-09	3.04E-09	1.90E-09	1.58E-09	2.13E-09	2.81E-09	1.38E-09	1.63E-09	1.59E-09	1.45E-09	1.43E-09
4.58E-09	3.47E-09	3.76E-09	4.04E-09	3.61E-09	2.01E-09	7.10E-10	1.15E-09	1.69E-09	2.23E-09	6.95E-10	8.31E-10	9.93E-10	4.88E-10	6.19E-10
3.70E-09	2.64E-09	2.52E-09	2.79E-09	2.28E-09	1.05E-09	-2.86E-10	4.40E-10	8.24E-10	1.18E-09	1.73E-10	-9.96E-11	4.61E-10	-3.33E-10	-5.17E-10
2.69E-09	2.03E-09	1.65E-09	1.47E-09	8.36E-10	-4.87E-11	-1.29E-09	-1.97E-10	-3.67E-10	2.33E-11	-1.69E-10	-7.65E-10	-2.17E-10	-1.02E-09	-1.54E-09
1.95E-09	1.22E-09	1.14E-09	7.18E-10	-5.96E-10	-1.32E-09	-2.06E-09	-1.53E-09	-2.27E-09	-1.58E-09	-1.18E-09	-1.54E-09	-1.11E-09	-2.28E-09	-2.89E-09
1.23E-09	6.91E-10	5.24E-10	3.66E-11	-1.74E-09	-2.10E-09	-2.39E-09	-2.53E-09	-3.02E-09	-2.93E-09	-2.29E-09	-2.26E-09	-2.51E-09	-3.06E-09	-3.18E-09
2.82E-10	-9.87E-11	-4.85E-11	-5.87E-10	-2.69E-09	-2.63E-09	-2.74E-09	-2.92E-09	-3.09E-09	-3.40E-09	-3.09E-09	-3.20E-09	-3.52E-09	-3.11E-09	-3.21E-09
-7.40E-10	-5.47E-10	-3.05E-10	-9.87E-10	-2.88E-09	-2.89E-09	-3.04E-09	-3.35E-09	-3.72E-09	-4.04E-09	-3.69E-09	-4.11E-09	-4.15E-09	-3.61E-09	-3.78E-09
-1.87E-09	-1.31E-09	-1.26E-09	-1.73E-09	-2.68E-09	-3.55E-09	-3.79E-09	-3.96E-09	-4.49E-09	-4.95E-09	-4.38E-09	-4.97E-09	-4.74E-09	-4.10E-09	-4.29E-09
-2.52E-09	-2.51E-09	-2.83E-09	-2.69E-09	-2.49E-09	-3.93E-09	-4.41E-09	-4.84E-09	-5.28E-09	-5.62E-09	-5.09E-09	-5.61E-09	-5.53E-09	-4.50E-09	-4.64E-09
-3.14E-09	-3.32E-09	-3.81E-09	-3.23E-09	-2.60E-09	-4.32E-09	-5.39E-09	-6.03E-09	-5.80E-09	-6.15E-09	-5.59E-09	-6.13E-09	-5.94E-09	-4.99E-09	-5.14E-09
-3.44E-09	-3.84E-09	-4.26E-09	-3.45E-09	-3.17E-09	-4.21E-09	-6.33E-09	-6.94E-09	-5.90E-09	-6.44E-09	-6.13E-09	-6.39E-09	-6.05E-09	-5.32E-09	-5.62E-09
-3.77E-09	-4.30E-09	-4.26E-09	-4.14E-09	-4.20E-09	-4.32E-09	-6.98E-09	-7.05E-09	-6.23E-09	-6.56E-09	-6.71E-09	-6.95E-09	-6.29E-09	-5.63E-09	-6.21E-09
-4.06E-09	-4.54E-09	-4.51E-09	-4.70E-09	-5.16E-09	-5.15E-09	-7.10E-09	-7.17E-09	-7.41E-09	-7.08E-09	-7.06E-09	-7.64E-09	-6.78E-09	-6.25E-09	-6.67E-09
-4.50E-09	-4.78E-09	-5.14E-09	-5.82E-09	-5.88E-09	-5.56E-09	-7.23E-09	-7.49E-09	-7.57E-09	-8.17E-09	-7.59E-09	-7.66E-09	-6.70E-09	-6.77E-09	-7.52E-09
-5.06E-09	-5.31E-09	-5.83E-09	-6.94E-09	-6.48E-09	-6.55E-09	-7.24E-09	-7.48E-09	-7.81E-09	-9.05E-09	-7.61E-09	-6.74E-09	-6.36E-09	-7.02E-09	-7.98E-09
-5.86E-09	-5.95E-09	-6.03E-09	-7.14E-09	-7.29E-09	-7.30E-09	-6.94E-09	-7.55E-09	-7.88E-09	-9.18E-09	-7.50E-09	-6.28E-09	-6.31E-09	-7.25E-09	-8.13E-09
-6.55E-09	-6.74E-09	-6.07E-09	-7.31E-09	-7.78E-09	-7.67E-09	-7.07E-09	-7.69E-09	-8.21E-09	-9.44E-09	-7.69E-09	-6.56E-09	-7.22E-09	-7.84E-09	-8.52E-09
-6.85E-09	-7.26E-09	-6.49E-09	-7.30E-09	-8.15E-09	-8.08E-09	-7.77E-09	-7.92E-09	-8.70E-09	-9.39E-09	-8.78E-09	-7.89E-09	-8.43E-09	-7.77E-09	-8.94E-09
-7.02E-09	-7.70E-09	-7.41E-09	-7.67E-09	-9.03E-09	-8.57E-09	-8.45E-09	-8.51E-09	-8.98E-09	-9.86E-09	-9.91E-09	-8.48E-09	-8.97E-09	-7.38E-09	-9.00E-09
-7.30E-09	-7.97E-09	-8.20E-09	-8.49E-09	-9.63E-09	-8.73E-09	-8.88E-09	-8.75E-09	-8.90E-09	-9.76E-09	-1.02E-08	-8.83E-09	-9.15E-09	-7.60E-09	-8.62E-09
-7.67E-09	-8.55E-09	-8.75E-09	-9.77E-09	-1.03E-08	-9.00E-09	-9.37E-09	-9.34E-09	-9.11E-09	-9.81E-09	-1.02E-08	-9.42E-09	-9.36E-09	-8.39E-09	-8.45E-09
-8.43E-09	-9.04E-09	-9.00E-09	-1.01E-08	-1.01E-08	-9.23E-09	-9.55E-09	-9.63E-09	-8.88E-09	-9.21E-09	-9.69E-09	-9.45E-09	-8.69E-09	-8.33E-09	-8.17E-09
-8.88E-09	-9.95E-09	-9.10E-09	-1.01E-08	-1.01E-08	-9.75E-09	-9.69E-09	-9.90E-09	-8.42E-09	-8.32E-09	-9.05E-09	-9.36E-09	-8.33E-09	-7.91E-09	-7.80E-09
-9.36E-09	-1.00E-08	-9.14E-09	-1.05E-08	-1.07E-08	-1.06E-08	-1.04E-08	-1.01E-08	-8.97E-09	-7.89E-09	-8.89E-09	-9.02E-09	-7.74E-09	-7.28E-09	-6.95E-09
-9.87E-09	-1.00E-08	-9.68E-09	-1.06E-08	-1.15E-08	-1.15E-08	-1.08E-08	-1.02E-08	-9.27E-09	-7.51E-09	-8.11E-09	-8.58E-09	-7.67E-09	-7.17E-09	-6.52E-09
-1.04E-08	-1.03E-08	-1.04E-08	-1.11E-08	-1.17E-08	-1.17E-08	-1.12E-08	-1.06E-08	-9.63E-09	-7.87E-09	-7.85E-09	-8.21E-09	-7.19E-09	-7.23E-09	-5.75E-09
-1.09E-08	-1.07E-08	-1.09E-08	-1.14E-08	-1.14E-08	-1.20E-08	-1.12E-08	-1.05E-08	-9.89E-09	-8.51E-09	-7.93E-09	-7.62E-09	-6.76E-09	-8.00E-09	-5.16E-09
-1.03E-08	-1.04E-08	-1.12E-08	-1.13E-08	-1.11E-08	-1.17E-08	-1.09E-08	-1.04E-08	-9.68E-09	-8.68E-09	-7.63E-09	-7.07E-09	-5.56E-09	-7.79E-09	-4.84E-09
-1.04E-08	-1.07E-08	-1.10E-08	-1.08E-08	-1.08E-08	-1.16E-08	-1.08E-08	-1.05E-08	-9.72E-09	-8.71E-09	-7.49E-09	-6.99E-09	-4.89E-09	-7.07E-09	-5.36E-09
-1.07E-08	-1.07E-08	-1.09E-08	-1.06E-08	-1.10E-08	-1.18E-08	-1.07E-08	-1.08E-08	-9.84E-09	-8.36E-09	-7.80E-09	-7.15E-09	-5.30E-09	-6.37E-09	-5.70E-09
-1.03E-08	-1.04E-08	-1.07E-08	-1.06E-08	-1.12E-08	-1.18E-08	-1.03E-08	-1.07E-08	-9.06E-09	-7.76E-09	-7.35E-09	-6.96E-09	-5.56E-09	-5.32E-09	-5.24E-09
-1.01E-08	-1.01E-08	-1.05E-08	-1.04E-08	-1.05E-08	-1.12E-08	-1.03E-08	-1.02E-08	-8.85E-09	-6.64E-09	-5.74E-09	-5.82E-09	-5.81E-09	-4.99E-09	-5.35E-09
-1.03E-08	-1.03E-08	-1.11E-08	-1.11E-08	-1.06E-08	-1.03E-08	-1.04E-08	-1.01E-08	-8.69E-09	-5.22E-09	-3.90E-09	-4.66E-09	-5.20E-09	-4.67E-09	-5.43E-09
-1.09E-08	-1.07E-08	-1.14E-08	-1.21E-08	-1.17E-08	-1.04E-08	-1.05E-08	-9.90E-09	-8.35E-09	-5.02E-09	-3.86E-09	-4.70E-09	-4.62E-09	-4.64E-09	-5.01E-09
-1.12E-08	-1.13E-08	-1.22E-08	-1.27E-08	-1.21E-08	-1.03E-08	-9.58E-09	-9.37E-09	-8.15E-09	-5.39E-09	-4.01E-09	-5.16E-09	-4.78E-09	-4.75E-09	-4.51E-09
-1.13E-08	-1.16E-08	-1.29E-08	-1.32E-08	-1.21E-08	-9.37E-09	-7.74E-09	-8.38E-09	-7.86E-09	-5.92E-09	-5.37E-09	-5.74E-09	-5.89E-09	-4.67E-09	-4.09E-09
-1.16E-08	-1.16E-08	-1.29E-08	-1.31E-08	-1.18E-08	-8.98E-09	-7.00E-09	-7.29E-09	-7.58E-09	-6.47E-09	-6.84E-09	-6.54E-09	-6.19E-09	-4.50E-09	-3.15E-09
-1.23E-08	-1.18E-08	-1.23E-08	-1.21E-08	-1.11E-08	-9.41E-09	-7.73E-09	-7.12E-09	-7.59E-09	-7.42E-09	-7.62E-09	-6.66E-09	-5.85E-09	-4.56E-09	-2.41E-09
-1.26E-08	-1.21E-08	-1.13E-08	-1.11E-08	-9.91E-09	-9.63E-09	-8.72E-09	-7.65E-09	-8.60E-09	-9.06E-09	-8.50E-09	-6.88E-09	-5.11E-09	-4.22E-09	-2.58E-09
-1.25E-08	-1.18E-08	-1.11E-08	-1.03E-08	-9.20E-09	-9.82E-09	-9.80E-09	-8.26E-09	-8.78E-09	-9.69E-09	-8.18E-09	-6.41E-09	-5.09E-09	-4.00E-09	-2.93E-09
-1.25E-08	-1.16E-08	-1.12E-08	-9.60E-09	-9.18E-09	-9.64E-09	-9.99E-09	-8.60E-09	-8.06E-09	-9.67E-09	-7.89E-09	-6.28E-09	-5.91E-09	-4.07E-09	-4.04E-09
-1.27E-08	-1.18E-08	-1.17E-08	-9.82E-09	-9.20E-09	-9.63E-09	-9.41E-09	-8.97E-09	-7.80E-09	-9.46E-09	-8.63E-09	-6.88E-09	-6.49E-09	-4.89E-09	-4.61E-09
-1.22E-08	-1.21E-08	-1.22E-08	-1.08E-08	-9.28E-09	-9.79E-09	-8.94E-09	-9.14E-09	-7.87E-09	-8.85E-09	-9.21E-09	-7.35E-09	-6.56E-09	-6.01E-09	-4.75E-09
-1.17E-08	-1.16E-08	-1.23E-08	-1.11E-08	-9.35E-09	-1.01E-08	-8.73E-09	-9.48E-09	-8.48E-09	-8.51E-09	-9.14E-09	-8.53E-09	-7.39E-09	-6.81E-09	-5.90E-09
-1.13E-08	-1.16E-08	-1.20E-08	-1.12E-08	-8.80E-09	-9.55E-09	-9.21E-09	-1.02E-08	-9.38E-09	-9.24E-09	-9.72E-09	-9.40E-09	-8.42E-09	-7.65E-09	-6.42E-09
-1.13E-08	-1.16E-08	-1.14E-08	-1.10E-08	-8.85E-09	-9.66E-09	-9.44E-09	-1.01E-08	-9.80E-09	-1.04E-08	-1.10E-08	-1.06E-08	-9.96E-09	-8.26E-09	-7.01E-09

-1.11E-08	-1.17E-08	-1.09E-08	-1.07E-08	-9.19E-09	-9.78E-09	-9.68E-09	-9.37E-09	-1.09E-08	-1.17E-08	-1.19E-08	-1.11E-08	-1.09E-08	-9.29E-09	-8.51E-09
-1.18E-08	-1.12E-08	-1.05E-08	-1.06E-08	-1.05E-08	-9.86E-09	-9.44E-09	-9.43E-09	-1.09E-08	-1.23E-08	-1.17E-08	-1.09E-08	-1.09E-08	-1.03E-08	-1.06E-08
-1.17E-08	-1.05E-08	-9.80E-09	-1.06E-08	-1.12E-08	-1.06E-08	-9.78E-09	-9.89E-09	-1.07E-08	-1.18E-08	-1.08E-08	-1.06E-08	-1.10E-08	-1.05E-08	-1.14E-08
-1.08E-08	-1.00E-08	-9.39E-09	-1.01E-08	-1.10E-08	-1.08E-08	-1.03E-08	-1.03E-08	-1.07E-08	-1.14E-08	-1.06E-08	-1.10E-08	-1.14E-08	-1.08E-08	-1.15E-08
-1.12E-08	-1.06E-08	-1.03E-08	-9.99E-09	-1.07E-08	-1.02E-08	-1.01E-08	-1.13E-08	-1.13E-08	-1.13E-08	-1.10E-08	-1.13E-08	-1.18E-08	-1.10E-08	-1.15E-08
-1.18E-08	-1.16E-08	-1.12E-08	-1.02E-08	-1.03E-08	-1.01E-08	-1.08E-08	-1.22E-08	-1.17E-08	-1.11E-08	-1.11E-08	-1.16E-08	-1.20E-08	-1.10E-08	-1.13E-08
-1.17E-08	-1.20E-08	-1.09E-08	-1.10E-08	-1.08E-08	-1.07E-08	-1.14E-08	-1.22E-08	-1.15E-08	-1.08E-08	-1.07E-08	-1.15E-08	-1.17E-08	-1.06E-08	-1.05E-08
-1.15E-08	-1.16E-08	-1.05E-08	-1.13E-08	-1.12E-08	-1.07E-08	-1.11E-08	-1.11E-08	-1.12E-08	-1.06E-08	-1.01E-08	-1.13E-08	-1.09E-08	-9.69E-09	-9.67E-09
-1.11E-08	-1.15E-08	-1.05E-08	-1.15E-08	-1.11E-08	-1.00E-08	-1.05E-08	-1.05E-08	-1.07E-08	-1.03E-08	-9.59E-09	-1.04E-08	-1.03E-08	-9.60E-09	-9.48E-09
-1.12E-08	-1.08E-08	-1.00E-08	-1.06E-08	-1.05E-08	-1.02E-08	-1.03E-08	-9.80E-09	-9.48E-09	-9.07E-09	-8.20E-09	-8.96E-09	-9.69E-09	-9.84E-09	-9.50E-09
-1.03E-08	-1.00E-08	-9.33E-09	-9.43E-09	-1.01E-08	-1.04E-08	-9.88E-09	-9.51E-09	-8.46E-09	-6.87E-09	-6.12E-09	-7.17E-09	-9.08E-09	-9.60E-09	-9.19E-09
-9.65E-09	-9.23E-09	-8.82E-09	-8.40E-09	-9.61E-09	-1.01E-08	-9.13E-09	-9.01E-09	-7.73E-09	-5.16E-09	-4.08E-09	-5.74E-09	-7.79E-09	-8.95E-09	-8.66E-09
-9.19E-09	-9.28E-09	-9.13E-09	-8.68E-09	-8.97E-09	-9.58E-09	-8.13E-09	-7.81E-09	-6.52E-09	-3.63E-09	-2.40E-09	-3.92E-09	-6.41E-09	-7.84E-09	-8.00E-09
-8.24E-09	-9.40E-09	-9.10E-09	-8.74E-09	-8.43E-09	-8.41E-09	-7.15E-09	-6.29E-09	-5.02E-09	-3.36E-09	-1.68E-09	-2.56E-09	-5.35E-09	-6.91E-09	-7.28E-09
-7.45E-09	-8.92E-09	-8.75E-09	-8.65E-09	-7.86E-09	-7.72E-09	-6.45E-09	-4.74E-09	-4.39E-09	-3.11E-09	-2.05E-09	-2.32E-09	-5.01E-09	-6.66E-09	-6.87E-09
-6.90E-09	-7.67E-09	-7.98E-09	-8.38E-09	-7.38E-09	-7.39E-09	-5.56E-09	-3.99E-09	-3.61E-09	-3.55E-09	-2.76E-09	-3.43E-09	-5.29E-09	-6.66E-09	-6.37E-09
-5.79E-09	-6.20E-09	-7.15E-09	-7.70E-09	-6.40E-09	-6.05E-09	-4.48E-09	-3.30E-09	-3.72E-09	-3.90E-09	-3.27E-09	-3.32E-09	-5.12E-09	-6.67E-09	-6.61E-09
-4.99E-09	-5.21E-09	-5.94E-09	-6.08E-09	-4.61E-09	-4.53E-09	-3.21E-09	-2.40E-09	-2.97E-09	-2.87E-09	-2.65E-09	-2.15E-09	-4.52E-09	-6.18E-09	-6.66E-09
-5.59E-09	-5.48E-09	-4.80E-09	-4.01E-09	-2.54E-09	-2.40E-09	-1.38E-09	-2.33E-10	-9.50E-10	-9.47E-10	-1.30E-09	-6.35E-10	-3.21E-09	-4.97E-09	-6.05E-09
-5.97E-09	-5.77E-09	-2.97E-09	-1.11E-09	4.48E-11	1.14E-09	1.53E-09	2.38E-09	2.04E-09	1.80E-09	7.87E-10	1.41E-09	-1.61E-09	-3.55E-09	-5.04E-09
-6.36E-09	-5.03E-09	-1.71E-09	8.95E-10	3.09E-09	6.08E-09	5.40E-09	5.74E-09	5.12E-09	4.67E-09	3.29E-09	3.68E-09	-1.64E-10	-2.94E-09	-4.11E-09
-6.32E-09	-4.27E-09	-3.44E-10	2.55E-09	6.31E-09	1.11E-08	9.32E-09	7.92E-09	7.29E-09	6.67E-09	4.75E-09	4.32E-09	2.38E-10	-2.83E-09	-3.62E-09
-6.49E-09	-4.01E-09	-8.47E-11	3.51E-09	7.94E-09	1.31E-08	1.19E-08	9.44E-09	8.11E-09	7.61E-09	4.83E-09	3.70E-09	1.36E-10	-2.88E-09	-3.53E-09
-6.29E-09	-3.88E-09	-1.05E-09	3.58E-09	7.84E-09	1.09E-08	1.14E-08	9.65E-09	6.68E-09	6.39E-09	3.21E-09	2.28E-09	-7.74E-10	-2.94E-09	-3.16E-09
-5.62E-09	-3.40E-09	-1.49E-09	2.50E-09	6.30E-09	8.39E-09	9.65E-09	7.82E-09	4.53E-09	4.24E-09	1.09E-09	1.54E-10	-9.76E-10	-2.65E-09	-2.70E-09
-4.45E-09	-2.45E-09	-1.44E-09	1.50E-09	3.90E-09	6.02E-09	6.32E-09	5.94E-09	3.32E-09	2.90E-09	-4.20E-10	-1.36E-09	-1.01E-09	-2.04E-09	-2.48E-09
-3.65E-09	-1.99E-09	-1.64E-09	1.16E-10	1.95E-09	2.84E-09	2.98E-09	4.43E-09	3.03E-09	2.28E-09	-8.75E-10	-1.82E-09	-1.14E-09	-1.55E-09	-2.62E-09
-3.69E-09	-2.58E-09	-2.72E-09	-1.14E-09	6.93E-10	1.12E-09	9.93E-10	2.87E-09	2.85E-09	1.98E-09	-1.02E-09	-2.12E-09	-1.97E-09	-1.38E-09	-2.27E-09
-4.23E-09	-3.45E-09	-3.19E-09	-1.86E-09	1.03E-09	7.13E-10	8.20E-10	2.37E-09	3.01E-09	2.05E-09	-5.84E-10	-2.34E-09	-2.39E-09	-1.05E-09	-2.09E-09
-4.77E-09	-3.44E-09	-3.86E-09	-2.03E-09	1.35E-09	1.49E-09	1.78E-09	3.93E-09	3.83E-09	2.95E-09	8.52E-11	-2.21E-09	-2.14E-09	-1.55E-09	-2.53E-09
-5.18E-09	-3.60E-09	-3.59E-09	-1.30E-09	2.68E-09	2.86E-09	3.51E-09	6.25E-09	6.09E-09	4.63E-09	3.93E-10	-1.88E-09	-2.09E-09	-2.51E-09	-2.75E-09
-5.18E-09	-3.74E-09	-3.19E-09	-7.30E-10	3.53E-09	3.93E-09	4.99E-09	9.16E-09	8.91E-09	5.57E-09	9.37E-10	-1.19E-09	-2.40E-09	-1.99E-09	-2.35E-09
-4.70E-09	-3.65E-09	-2.63E-09	-6.59E-10	2.89E-09	3.68E-09	5.41E-09	1.04E-08	1.05E-08	5.22E-09	1.32E-09	-7.58E-10	-1.63E-09	-1.43E-09	-2.37E-09
-4.26E-09	-3.59E-09	-2.57E-09	-1.21E-09	8.41E-10	1.31E-09	3.72E-09	7.84E-09	9.19E-09	4.21E-09	1.14E-09	-2.13E-10	-1.20E-09	-1.45E-09	-3.00E-09
-4.59E-09	-3.85E-09	-2.92E-09	-2.22E-09	-1.35E-09	-1.46E-09	1.18E-09	3.97E-09	4.20E-09	2.06E-09	1.06E-09	6.10E-10	-8.89E-10	-1.10E-09	-2.50E-09
-4.84E-09	-3.78E-09	-4.42E-09	-3.71E-09	-3.88E-09	-3.04E-09	-1.35E-09	-6.65E-10	-3.62E-10	-8.87E-11	6.79E-10	9.44E-10	-8.73E-10	-5.99E-11	-1.72E-09
-5.20E-09	-4.78E-09	-4.95E-09	-5.25E-09	-5.36E-09	-4.00E-09	-3.04E-09	-3.21E-09	-2.90E-09	-2.04E-09	-6.20E-10	-1.26E-09	-1.48E-09	1.14E-10	-1.19E-09
-6.23E-09	-5.87E-09	-5.54E-09	-6.45E-09	-6.36E-09	-4.75E-09	-4.45E-09	-5.36E-09	-4.55E-09	-3.81E-09	-2.54E-09	-2.70E-09	-2.14E-09	-5.74E-10	-1.67E-09
-6.92E-09	-6.61E-09	-5.75E-09	-6.78E-09	-6.93E-09	-5.96E-09	-6.02E-09	-6.78E-09	-5.53E-09	-5.29E-09	-4.15E-09	-3.50E-09	-3.21E-09	-1.95E-09	-2.80E-09
-7.70E-09	-7.33E-09	-6.69E-09	-7.25E-09	-7.60E-09	-6.87E-09	-7.28E-09	-7.20E-09	-6.14E-09	-5.74E-09	-5.22E-09	-4.30E-09	-3.80E-09	-3.67E-09	-4.60E-09
-8.18E-09	-7.63E-09	-7.41E-09	-7.79E-09	-7.84E-09	-7.26E-09	-7.98E-09	-7.86E-09	-6.65E-09	-5.92E-09	-5.61E-09	-4.89E-09	-3.85E-09	-4.55E-09	-4.74E-09
-8.19E-09	-7.88E-09	-8.14E-09	-8.55E-09	-8.15E-09	-7.62E-09	-7.76E-09	-7.95E-09	-7.26E-09	-6.24E-09	-4.84E-09	-4.82E-09	-3.70E-09	-4.20E-09	-4.58E-09
-8.07E-09	-7.60E-09	-8.44E-09	-8.92E-09	-8.09E-09	-7.49E-09	-7.46E-09	-7.79E-09	-7.92E-09	-6.76E-09	-4.79E-09	-5.51E-09	-4.68E-09	-4.02E-09	-4.18E-09
-8.06E-09	-7.90E-09	-8.39E-09	-8.80E-09	-8.47E-09	-7.30E-09	-6.87E-09	-7.31E-09	-7.85E-09	-6.77E-09	-5.41E-09	-6.11E-09	-5.68E-09	-4.65E-09	-3.93E-09
-7.71E-09	-8.37E-09	-8.49E-09	-8.63E-09	-8.55E-09	-7.68E-09	-7.11E-09	-7.14E-09	-7.21E-09	-6.50E-09	-5.72E-09	-6.13E-09	-5.99E-09	-4.88E-09	-4.21E-09
-7.28E-09	-8.55E-09	-8.24E-09	-8.29E-09	-8.50E-09	-7.73E-09	-7.34E-09	-7.19E-09	-6.98E-09	-6.33E-09	-5.84E-09	-6.02E-09	-5.84E-09	-4.88E-09	-5.08E-09
-7.30E-09	-8.43E-09	-8.11E-09	-8.21E-09	-8.45E-09	-7.98E-09	-7.44E-09	-7.42E-09	-6.94E-09	-6.31E-09	-6.19E-09	-5.98E-09	-5.62E-09	-4.92E-09	-5.26E-09
-7.47E-09	-8.27E-09	-7.82E-09	-8.01E-09	-8.32E-09	-8.09E-09	-7.82E-09	-7.69E-09	-7.12E-09	-6.86E-09	-6.32E-09	-5.99E-09	-5.95E-09	-4.98E-09	-4.72E-09
-7.75E-09	-7.54E-09	-7.04E-09	-7.92E-09	-8.42E-09	-7.96E-09	-7.83E-09	-7.76E-09	-7.11E-09	-6.82E-09	-6.11E-09	-5.99E-09	-5.97E-09	-5.46E-09	-4.47E-09
-7.74E-09	-6.90E-09	-6.36E-09	-7.83E-09	-8.44E-09	-7.77E-09	-7.69E-09	-7.85E-09	-6.98E-09	-6.76E-09	-5.93E-09	-5.75E-09	-5.76E-09	-5.52E-09	-4.81E-09

1.49E-15	1.46E-15	1.38E-15	1.34E-15	1.30E-15	1.27E-15	1.23E-15	1.22E-15	1.21E-15	1.16E-15	1.16E-15	1.14E-15	1.12E-15	1.09E-15	1.08E-15
1.37E-15	1.36E-15	1.30E-15	1.26E-15	1.20E-15	1.17E-15	1.15E-15	1.12E-15	1.12E-15	1.07E-15	1.08E-15	1.04E-15	1.04E-15	9.99E-16	9.90E-16
1.27E-15	1.24E-15	1.21E-15	1.16E-15	1.13E-15	1.07E-15	1.09E-15	1.06E-15	1.05E-15	1.02E-15	1.01E-15	9.67E-16	9.72E-16	9.30E-16	9.30E-16
1.17E-15	1.14E-15	1.11E-15	1.09E-15	1.06E-15	1.00E-15	1.00E-15	9.87E-16	9.75E-16	9.62E-16	9.46E-16	9.04E-16	8.99E-16	8.71E-16	8.60E-16
1.08E-15	1.04E-15	1.01E-15	1.00E-15	9.62E-16	9.10E-16	8.98E-16	9.22E-16	9.02E-16	8.75E-16	8.63E-16	8.04E-16	7.95E-16	8.10E-16	7.97E-16
9.96E-16	9.77E-16	9.46E-16	9.35E-16	8.81E-16	8.26E-16	8.44E-16	8.71E-16	8.37E-16	7.93E-16	7.79E-16	7.24E-16	7.27E-16	7.56E-16	7.31E-16
9.45E-16	9.10E-16	8.74E-16	8.54E-16	8.14E-16	7.75E-16	8.04E-16	8.25E-16	7.83E-16	7.17E-16	6.98E-16	6.78E-16	6.71E-16	6.99E-16	6.81E-16
8.78E-16	8.18E-16	8.04E-16	7.72E-16	7.33E-16	7.24E-16	7.83E-16	8.01E-16	7.18E-16	6.61E-16	6.31E-16	6.10E-16	6.13E-16	6.61E-16	6.46E-16
7.98E-16	7.40E-16	7.41E-16	7.06E-16	6.68E-16	6.66E-16	7.74E-16	7.69E-16	6.48E-16	6.02E-16	5.70E-16	5.49E-16	5.74E-16	6.12E-16	5.89E-16
7.04E-16	6.61E-16	6.69E-16	6.47E-16	6.21E-16	6.20E-16	7.39E-16	6.51E-16	5.82E-16	5.63E-16	5.22E-16	5.07E-16	5.57E-16	5.61E-16	5.41E-16
6.49E-16	6.02E-16	6.12E-16	5.99E-16	5.87E-16	5.86E-16	6.44E-16	5.39E-16	5.09E-16	5.26E-16	4.98E-16	4.89E-16	5.37E-16	5.30E-16	5.08E-16
6.18E-16	5.63E-16	5.84E-16	5.65E-16	5.62E-16	5.41E-16	5.11E-16	4.57E-16	4.38E-16	4.65E-16	4.75E-16	4.67E-16	4.81E-16	4.76E-16	4.70E-16
5.96E-16	5.50E-16	5.74E-16	5.51E-16	5.21E-16	5.10E-16	4.30E-16	3.91E-16	3.83E-16	3.91E-16	4.23E-16	4.20E-16	4.30E-16	4.23E-16	4.10E-16
5.96E-16	5.43E-16	5.56E-16	5.25E-16	4.75E-16	4.65E-16	3.76E-16	3.43E-16	3.37E-16	3.37E-16	3.64E-16	3.75E-16	3.80E-16	3.55E-16	3.50E-16
5.51E-16	5.31E-16	5.26E-16	4.47E-16	4.15E-16	4.00E-16	3.32E-16	3.05E-16	3.08E-16	3.02E-16	3.05E-16	3.27E-16	3.29E-16	2.96E-16	2.99E-16
4.70E-16	4.62E-16	4.34E-16	3.66E-16	3.58E-16	3.33E-16	2.91E-16	2.58E-16	2.77E-16	2.66E-16	2.58E-16	2.95E-16	2.73E-16	2.59E-16	2.40E-16
3.84E-16	3.76E-16	3.50E-16	3.13E-16	2.99E-16	2.79E-16	2.46E-16	2.21E-16	2.48E-16	2.44E-16	2.35E-16	2.67E-16	2.21E-16	2.13E-16	1.95E-16
3.12E-16	3.09E-16	2.80E-16	2.64E-16	2.55E-16	2.34E-16	2.24E-16	1.99E-16	2.17E-16	2.23E-16	2.16E-16	2.43E-16	1.98E-16	1.87E-16	1.66E-16
2.52E-16	2.55E-16	2.42E-16	2.41E-16	2.21E-16	1.99E-16	1.93E-16	1.85E-16	1.91E-16	1.96E-16	1.83E-16	2.12E-16	1.70E-16	1.54E-16	1.49E-16
2.09E-16	2.21E-16	1.96E-16	2.08E-16	1.84E-16	1.67E-16	1.59E-16	1.70E-16	1.63E-16	1.70E-16	1.50E-16	1.64E-16	1.44E-16	1.28E-16	1.35E-16
1.79E-16	2.05E-16	1.63E-16	1.76E-16	1.59E-16	1.38E-16	1.31E-16	1.47E-16	1.39E-16	1.41E-16	1.21E-16	1.24E-16	1.14E-16	1.14E-16	1.26E-16
1.59E-16	1.94E-16	1.44E-16	1.44E-16	1.38E-16	1.26E-16	1.12E-16	1.14E-16	1.05E-16	1.08E-16	9.95E-17	9.97E-17	9.79E-17	1.02E-16	1.18E-16
1.49E-16	1.69E-16	1.29E-16	1.24E-16	1.22E-16	1.13E-16	9.30E-17	7.23E-17	7.37E-17	7.87E-17	7.77E-17	8.46E-17	8.54E-17	8.62E-17	9.49E-17
1.29E-16	1.42E-16	1.20E-16	1.29E-16	1.07E-16	8.95E-17	7.46E-17	5.81E-17	5.24E-17	5.19E-17	5.83E-17	6.45E-17	6.77E-17	6.87E-17	6.99E-17
1.14E-16	1.16E-16	1.17E-16	1.29E-16	9.43E-17	7.09E-17	6.12E-17	5.27E-17	4.17E-17	4.00E-17	4.50E-17	5.00E-17	4.85E-17	5.22E-17	5.91E-17
1.05E-16	9.70E-17	1.10E-16	1.12E-16	6.94E-17	6.05E-17	4.84E-17	4.33E-17	3.49E-17	3.12E-17	3.28E-17	4.15E-17	3.46E-17	3.38E-17	4.14E-17
8.86E-17	8.92E-17	9.01E-17	8.46E-17	4.97E-17	5.42E-17	4.07E-17	2.79E-17	2.88E-17	2.19E-17	2.47E-17	2.63E-17	2.12E-17	2.36E-17	2.66E-17
5.91E-17	7.76E-17	7.55E-17	6.55E-17	3.40E-17	4.59E-17	3.19E-17	1.89E-17	2.12E-17	1.56E-17	1.74E-17	1.61E-17	1.46E-17	1.71E-17	1.82E-17
4.21E-17	5.92E-17	6.25E-17	5.40E-17	3.39E-17	3.52E-17	2.34E-17	1.70E-17	1.77E-17	1.31E-17	1.30E-17	1.22E-17	1.05E-17	9.67E-18	1.25E-17
3.45E-17	3.69E-17	4.21E-17	4.05E-17	3.18E-17	2.01E-17	1.31E-17	1.23E-17	1.16E-17	1.07E-17	7.31E-18	7.05E-18	6.58E-18	6.15E-18	7.18E-18
3.26E-17	3.01E-17	3.13E-17	2.92E-17	2.61E-17	1.22E-17	7.65E-18	7.60E-18	7.15E-18	7.77E-18	3.34E-18	4.43E-18	4.04E-18	5.10E-18	4.47E-18

2.94E-17	1.98E-17	2.22E-17	2.10E-17	2.05E-17	9.24E-18	3.60E-18	2.48E-18	4.54E-18	7.89E-18	1.92E-18	2.65E-18	2.53E-18	2.09E-18	2.04E-18
2.10E-17	1.21E-17	1.42E-17	1.63E-17	1.31E-17	4.03E-18	5.04E-19	1.33E-18	2.85E-18	4.97E-18	4.83E-19	6.90E-19	9.86E-19	2.38E-19	3.84E-19
1.37E-17	6.98E-18	6.36E-18	7.79E-18	5.18E-18	1.11E-18	8.21E-20	1.94E-19	6.80E-19	1.38E-18	3.00E-20	9.92E-21	2.13E-19	1.11E-19	2.67E-19
7.23E-18	4.10E-18	2.74E-18	2.16E-18	6.99E-19	2.37E-21	1.66E-18	3.90E-20	1.35E-19	5.41E-22	2.87E-20	5.86E-19	4.71E-20	1.04E-18	2.38E-18
3.80E-18	1.49E-18	1.30E-18	5.15E-19	3.55E-19	1.73E-18	4.25E-18	2.34E-18	5.17E-18	2.49E-18	1.38E-18	2.38E-18	1.24E-18	5.21E-18	8.36E-18
1.51E-18	4.78E-19	2.75E-19	1.34E-21	3.04E-18	4.40E-18	5.73E-18	6.38E-18	9.10E-18	8.58E-18	5.25E-18	5.12E-18	6.28E-18	9.36E-18	1.01E-17
7.97E-20	9.73E-21	2.35E-21	3.45E-19	7.21E-18	6.92E-18	7.50E-18	8.53E-18	9.58E-18	1.15E-17	9.56E-18	1.02E-17	1.24E-17	9.69E-18	1.03E-17
5.47E-19	2.99E-19	9.30E-20	9.73E-19	8.30E-18	8.37E-18	9.26E-18	1.12E-17	1.38E-17	1.63E-17	1.36E-17	1.69E-17	1.72E-17	1.30E-17	1.43E-17
3.50E-18	1.72E-18	1.59E-18	2.99E-18	7.17E-18	1.26E-17	1.44E-17	1.57E-17	2.02E-17	2.45E-17	1.92E-17	2.47E-17	2.25E-17	1.68E-17	1.84E-17
6.36E-18	6.29E-18	7.99E-18	7.25E-18	6.19E-18	1.54E-17	1.95E-17	2.35E-17	2.78E-17	3.16E-17	2.60E-17	3.15E-17	3.06E-17	2.03E-17	2.16E-17
9.86E-18	1.10E-17	1.45E-17	1.05E-17	6.78E-18	1.87E-17	2.91E-17	3.64E-17	3.36E-17	3.78E-17	3.12E-17	3.76E-17	3.52E-17	2.49E-17	2.64E-17
1.18E-17	1.48E-17	1.82E-17	1.19E-17	1.00E-17	1.77E-17	4.00E-17	4.82E-17	3.48E-17	4.15E-17	3.76E-17	4.08E-17	3.66E-17	2.84E-17	3.16E-17
1.42E-17	1.85E-17	1.81E-17	1.72E-17	1.76E-17	1.86E-17	4.87E-17	4.97E-17	3.88E-17	4.30E-17	4.50E-17	4.83E-17	3.95E-17	3.17E-17	3.85E-17
1.65E-17	2.06E-17	2.04E-17	2.21E-17	2.66E-17	2.65E-17	5.04E-17	5.14E-17	5.48E-17	5.01E-17	4.98E-17	5.84E-17	4.59E-17	3.91E-17	4.45E-17
2.03E-17	2.29E-17	2.64E-17	3.39E-17	3.46E-17	3.09E-17	5.23E-17	5.61E-17	5.73E-17	6.68E-17	5.76E-17	5.86E-17	4.49E-17	4.58E-17	5.66E-17
2.56E-17	2.82E-17	3.40E-17	4.81E-17	4.20E-17	4.30E-17	5.25E-17	5.59E-17	6.09E-17	8.19E-17	5.79E-17	4.54E-17	4.04E-17	4.92E-17	6.37E-17
3.43E-17	3.54E-17	3.64E-17	5.09E-17	5.32E-17	5.34E-17	4.82E-17	5.70E-17	6.20E-17	8.42E-17	5.62E-17	3.94E-17	3.99E-17	5.26E-17	6.61E-17
4.29E-17	4.55E-17	3.69E-17	5.35E-17	6.05E-17	5.88E-17	5.01E-17	5.91E-17	6.74E-17	8.90E-17	5.91E-17	4.31E-17	5.22E-17	6.14E-17	7.26E-17
4.70E-17	5.27E-17	4.21E-17	5.33E-17	6.64E-17	6.52E-17	6.04E-17	6.28E-17	7.57E-17	8.81E-17	7.71E-17	6.22E-17	7.11E-17	6.04E-17	8.00E-17
4.92E-17	5.93E-17	5.48E-17	5.89E-17	8.16E-17	7.34E-17	7.14E-17	7.25E-17	8.07E-17	9.72E-17	9.81E-17	7.19E-17	8.04E-17	5.44E-17	8.10E-17
5.32E-17	6.36E-17	6.73E-17	7.20E-17	9.27E-17	7.63E-17	7.88E-17	7.65E-17	7.92E-17	9.53E-17	1.04E-16	7.80E-17	8.37E-17	5.78E-17	7.43E-17
5.88E-17	7.31E-17	7.66E-17	9.55E-17	1.06E-16	8.11E-17	8.79E-17	8.73E-17	8.30E-17	9.61E-17	1.05E-16	8.87E-17	8.77E-17	7.03E-17	7.13E-17
7.10E-17	8.17E-17	8.09E-17	1.03E-16	1.02E-16	8.52E-17	9.12E-17	9.28E-17	7.89E-17	8.48E-17	9.38E-17	8.92E-17	7.55E-17	6.95E-17	6.67E-17
7.89E-17	9.90E-17	8.29E-17	1.02E-16	1.02E-16	9.51E-17	9.39E-17	9.79E-17	7.09E-17	6.92E-17	8.19E-17	8.75E-17	6.94E-17	6.26E-17	6.09E-17
8.77E-17	1.00E-16	8.36E-17	1.09E-16	1.15E-16	1.13E-16	1.08E-16	1.03E-16	8.05E-17	6.23E-17	7.91E-17	8.14E-17	5.99E-17	5.30E-17	4.83E-17
9.74E-17	1.01E-16	9.37E-17	1.12E-16	1.32E-16	1.31E-16	1.17E-16	1.05E-16	8.60E-17	5.64E-17	6.58E-17	7.36E-17	5.89E-17	5.14E-17	4.25E-17
1.09E-16	1.05E-16	1.08E-16	1.23E-16	1.36E-16	1.37E-16	1.25E-16	1.13E-16	9.28E-17	6.19E-17	6.16E-17	6.74E-17	5.17E-17	5.23E-17	3.31E-17
1.18E-16	1.14E-16	1.18E-16	1.30E-16	1.31E-16	1.44E-16	1.25E-16	1.10E-16	9.79E-17	7.23E-17	6.28E-17	5.80E-17	4.56E-17	6.40E-17	2.67E-17
1.07E-16	1.08E-16	1.24E-16	1.28E-16	1.23E-16	1.38E-16	1.18E-16	1.08E-16	9.37E-17	7.53E-17	5.82E-17	5.00E-17	3.10E-17	6.07E-17	2.34E-17
1.09E-16	1.14E-16	1.21E-16	1.17E-16	1.16E-16	1.34E-16	1.16E-16	1.11E-16	9.45E-17	7.59E-17	5.62E-17	4.89E-17	2.39E-17	5.00E-17	2.88E-17
1.14E-16	1.14E-16	1.19E-16	1.13E-16	1.21E-16	1.38E-16	1.14E-16	1.17E-16	9.68E-17	6.99E-17	6.08E-17	5.11E-17	2.81E-17	4.05E-17	3.25E-17
1.05E-16	1.09E-16	1.14E-16	1.12E-16	1.25E-16	1.38E-16	1.07E-16	1.15E-16	8.21E-17	6.03E-17	5.41E-17	4.85E-17	3.09E-17	2.83E-17	2.75E-17
1.01E-16	1.02E-16	1.11E-16	1.08E-16	1.10E-16	1.26E-16	1.06E-16	1.05E-16	7.83E-17	4.41E-17	3.29E-17	3.39E-17	3.37E-17	2.49E-17	2.87E-17
1.07E-16	1.06E-16	1.23E-16	1.23E-16	1.12E-16	1.06E-16	1.08E-16	1.02E-16	7.55E-17	2.73E-17	1.52E-17	2.17E-17	2.70E-17	2.18E-17	2.95E-17
1.18E-16	1.14E-16	1.30E-16	1.46E-16	1.37E-16	1.08E-16	1.11E-16	9.81E-17	6.97E-17	2.52E-17	1.49E-17	2.21E-17	2.13E-17	2.15E-17	2.51E-17
1.25E-16	1.28E-16	1.48E-16	1.61E-16	1.46E-16	1.06E-16	9.18E-17	8.78E-17	6.65E-17	2.90E-17	1.61E-17	2.66E-17	2.28E-17	2.25E-17	2.04E-17
1.28E-16	1.34E-16	1.65E-16	1.74E-16	1.46E-16	8.77E-17	5.99E-17	7.02E-17	6.18E-17	3.50E-17	2.89E-17	3.30E-17	3.47E-17	2.18E-17	1.67E-17
1.34E-16	1.34E-16	1.66E-16	1.71E-16	1.39E-16	8.07E-17	4.90E-17	5.31E-17	5.74E-17	4.19E-17	4.68E-17	4.27E-17	3.83E-17	2.03E-17	9.93E-18
1.51E-16	1.40E-16	1.50E-16	1.47E-16	1.22E-16	8.86E-17	5.97E-17	5.08E-17	5.77E-17	5.50E-17	5.80E-17	4.43E-17	3.42E-17	2.08E-17	5.80E-18
1.59E-16	1.46E-16	1.27E-16	1.23E-16	9.82E-17	9.28E-17	7.60E-17	5.85E-17	7.40E-17	8.20E-17	7.22E-17	4.73E-17	2.61E-17	1.78E-17	6.67E-18
1.55E-16	1.39E-16	1.22E-16	1.05E-16	8.46E-17	9.65E-17	9.59E-17	6.81E-17	7.70E-17	9.40E-17	6.68E-17	4.11E-17	2.59E-17	1.60E-17	8.57E-18
1.55E-16	1.35E-16	1.25E-16	9.22E-17	8.42E-17	9.29E-17	9.99E-17	7.39E-17	6.49E-17	9.35E-17	6.23E-17	3.95E-17	3.50E-17	1.66E-17	1.63E-17
1.62E-16	1.40E-16	1.36E-16	9.65E-17	8.47E-17	9.27E-17	8.86E-17	8.04E-17	6.09E-17	8.96E-17	7.45E-17	4.73E-17	4.21E-17	2.40E-17	2.12E-17
1.50E-16	1.46E-16	1.48E-16	1.16E-16	8.61E-17	9.58E-17	8.00E-17	8.36E-17	6.19E-17	7.83E-17	8.48E-17	5.40E-17	4.31E-17	3.61E-17	2.26E-17
1.36E-16	1.35E-16	1.51E-16	1.23E-16	8.74E-17	1.01E-16	7.61E-17	8.99E-17	7.18E-17	7.24E-17	8.35E-17	7.28E-17	5.46E-17	4.64E-17	3.48E-17
1.28E-16	1.35E-16	1.45E-16	1.26E-16	7.75E-17	9.13E-17	8.48E-17	1.04E-16	8.80E-17	8.55E-17	9.45E-17	8.83E-17	7.09E-17	5.85E-17	4.13E-17
1.27E-16	1.36E-16	1.31E-16	1.22E-16	7.83E-17	9.33E-17	8.91E-17	1.03E-16	9.60E-17	1.09E-16	1.22E-16	1.13E-16	9.92E-17	6.82E-17	4.91E-17
1.23E-16	1.38E-16	1.20E-16	1.15E-16	8.45E-17	9.56E-17	9.36E-17	8.78E-17	1.19E-16	1.37E-16	1.42E-16	1.24E-16	1.18E-16	8.62E-17	7.24E-17
1.39E-16	1.25E-16	1.09E-16	1.12E-16	1.09E-16	9.73E-17	8.90E-17	8.89E-17	1.18E-16	1.51E-16	1.38E-16	1.19E-16	1.18E-16	1.06E-16	1.13E-16
1.36E-16	1.10E-16	9.60E-17	1.12E-16	1.25E-16	1.13E-16	9.57E-17	9.78E-17	1.14E-16	1.40E-16	1.16E-16	1.13E-16	1.20E-16	1.11E-16	1.29E-16
1.17E-16	9.99E-17	8.82E-17	1.03E-16	1.20E-16	1.17E-16	1.06E-16	1.05E-16	1.14E-16	1.30E-16	1.11E-16	1.21E-16	1.30E-16	1.16E-16	1.33E-16
1.25E-16	1.13E-16	1.07E-16	9.99E-17	1.14E-16	1.04E-16	1.02E-16	1.27E-16	1.28E-16	1.27E-16	1.21E-16	1.28E-16	1.38E-16	1.21E-16	1.32E-16
1.40E-16	1.35E-16	1.25E-16	1.05E-16	1.07E-16	1.02E-16	1.16E-16	1.49E-16	1.38E-16	1.24E-16	1.22E-16	1.35E-16	1.44E-16	1.21E-16	1.28E-16
1.36E-16	1.43E-16	1.20E-16	1.20E-16	1.17E-16	1.14E-16	1.31E-16	1.48E-16	1.32E-16	1.16E-16	1.14E-16	1.33E-16	1.36E-16	1.13E-16	1.11E-16
1.31E-16	1.34E-16	1.10E-16	1.27E-16	1.26E-16	1.14E-16	1.23E-16	1.22E-16	1.25E-16	1.12E-16	1.02E-16	1.27E-16	1.19E-16	9.38E-17	9.35E-17
1.24E-16	1.32E-16	1.10E-16	1.33E-16	1.23E-16	1.01E-16	1.10E-16	1.09E-16	1.14E-16	1.05E-16	9.19E-17	1.08E-16	1.07E-16	9.21E-17	8.98E-17
1.25E-16	1.17E-16	1.00E-16	1.12E-16	1.11E-16	1.04E-16	1.06E-16	9.61E-17	8.98E-17	8.22E-17	6.72E-17	8.03E-17	9.39E-17	9.68E-17	9.02E-17
1.06E-16	1.00E-16	8.70E-17	8.90E-17	1.02E-16	1.09E-16	9.76E-17	9.05E-17	7.16E-17	4.72E-17	3.74E-17	5.14E-17	8.24E-17	9.22E-17	8.45E-17
9.32E-17	8.52E-17	7.78E-17	7.06E-17	9.23E-17	1.01E-16	8.33E-17	8.11E-17	5.98E-17	2.66E-17	1.66E-17	3.29E-17	6.07E-17	8.02E-17	7.50E-17
8.44E-17	8.61E-17	8.33E-17	7.54E-17	8.05E-17	9.17E-17	6.62E-17	6.10E-17	4.26E-17	1.32E-17	5.77E-18	1.54E-17	4.11E-17	6.15E-17	6.39E-17
6.78E-17	8.84E-17	8.29E-17	7.64E-17	7.10E-17	7.08E-17	5.11E-17	3.96E-17	2.52E-17	1.13E-17	2.82E-18	6.54E-18	2.87E-17	4.77E-17	5.29E-17
5.54E-17	7.95E-17	7.66E-17	7.48E-17	6.17E-17	5.96E-17	4.16E-17	2.24E-17	1.93E-17	9.66E-18	4.21E-18	5.37E-18	2.51E-17	4.44E-17	4.72E-17
4.77E-17	5.88E-17	6.37E-17	7.03E-17	5.45E-17	5.46E-17	3.09E-17	1.59E-17	1.30E-17	1.26E-17	7.62E-18	1.17E-17	2.80E-17	4.44E-17	4.06E-17
3.36E-17	3.85E-17	5.12E-17	5.92E-17	4.10E-17	3.66E-17	2.00E-17	1.09E-17	1.39E-17	1.52E-17	1.07E-17	1.10E-17	2.62E-17	4.45E-17	4.37E-17
2.49E-17	2.71E-17	3.53E-17	3.70E-17	2.12E-17	2.06E-17	1.03E-17	5.75E-18	8.82E-18	8.23E-18	7.03E-18	4.63E-18	2.04E-17	3.81E-17	4.43E-17
3.13E-17	3.01E-17	2.31E-17	1.60E-17	6.45E-18	5.78E-18	1.89E-18	5.43E-20	9.02E-19	8.97E-19	1.68E-18	4.03E-19	1.03E-17	2.47E-17	3.66E-17
3.57E-17	3.33E-17	8.80E-18	1.23E-18	2.01E-21	1.31E-18	2.33E-18	5.69E-18	4.18E-18	3.24E-18	6.19E-19	1.99E-18	2.60E-18	1.26E-17	2.54E-17
4.05E-17	2.53E-17	2.92E-18	8.02E-19	9.55E-18	3.70E-17	2.92E-17	3.30E-17	2.62E-17	2.18E-17	1.08E-17	1.35E-17	2.68E-20	8.63E-18	1.69E-17
3.99E-17	1.82E-17	1.18E-19	6.52E-18	3.98E-17	1.23E-16	8.68E-17	6.27E-17	5.31E-17	4.44E-17	2.25E-17	1.86E-17	5.67E-20	8.02E-18	1.31E-17
4.21E-17	1.61E-17	7.17E-21	1.23E-17	6.31E-17	1.71E-16	1.42E-16	8.91E-17	6.58E-17	5.80E-17	2.33E-17	1.37E-17	1.84E-20	8.29E-18	1.25E-17
3.96E-17	1.50E-17	1.11E-18	1.28E-17	6.15E-17	1.18E-16	1.31E-16	9.31E-17	4.46E-17	4.09E-17	1.03E-17	5.18E-18	5.99E-19	8.64E-18	1.00E-17
3.16E-17	1.16E-17	2.22E-18	6.26E-18	3.97E-17	7.04E-17	9.32E-17	6.11E-17	2.05E-17	1.80E-17	1.19E-18	2.37E-20	9.52E-19	7.02E-18	7.28E-18
1.98E-17	6.02E-18	2.08E-18	2.25E-18	1.52E-17	3.63E-17	3.99E-17	3.53E-17	1.10E-17	8.40E-18	1.76E-19	1.85E-18	1.01E-18	4.16E-18	6.15E-18
1.34E-17	3.97E-18	2.68E-18	1.34E-20	3.81E-18	8.07E-18	8.88E-18	1.96E-17	9.19E-18	5.18E-18	7.66E-19	3.30E-18	1.29E-18	2.39E-18	6.86E-18
1.36E-17	6.66E-18	7.37E-18	1.31E-18	4.81E-19	1.25E-18	9.86E-19	8.25E-18	8.15E-18	3.90E-18	1.03E-18	4.49E-18	3.86E-18	1.90E-18	5.14E-18
1.79E-17	1.19E-17	1.02E-17	3.45E-18	1.05E-18	5.09E-19	6.72E-19	5.63E-18	9.03E-18	4.22E-18	3.41E-19	5.49E-18	5.72E-18	1.09E-18	4.38E-18
2.27E-17	1.18E-17	1.49E-17	4.11E-18	1.82E-18	2.23E-18	3.18E-18	1.55E-17	1.46E-17	8.70E-18	7.25E-21	4.88E-18	4.60E-18	2.40E-18	6.42E-18
2.68E-17	1.30E-17	1.29E-17	1.69E-18	7.17E-18	8.21E-18	1.23E-17	3.90E-17	3.71E-17	2.14E-17	1.54E-19	3.53E-18	4.38E-18	6.32E-18	7.56E-18
2.68E-17	1.40E-17	1.02E-17	5.33E-19	1.24E-17	1.54E-17	2.49E-17	8.38E-17	7.94E-17	3.10E-17	8.79E-19	1.41E-18	5.77E-18	3.98E-18	5.52E-18
2.21E-17	1.33E-17	6.94E-18	4.34E-19	8.36E-18	1.35E-17	2.92E-17	1.08E-16	1.10E-16	2.73E-17	1.73E-18	5.75E-19	2.67E-18	2.06E-18	5.62E-18
1.81E-17	1.29E-17	6.61E-18	1.47E-18	7.08E-19	1.72E-18	1.39E-17	6.15E-17	8.44E-17	1.77E-17	1.30E-18	4.52E-20	1.43E-18	2.11E-18	8.97E-18
2.10E-17	1.48E-17	8.54E-18	4.95E-18	1.81E-18	2.12E-18	1.40E-18	1.58E-17	1.76E-17	4.26E-18	1.13E-18	3.72E-19	7.90E-19	1.20E-18	6.23E-18
2.34E-17	1.43E-17	1.96E-17	1.38E-17	1.51E-17	9.25E-18	1.82E-18	4.42E-19	1.31E-19	7.87E-21	4.61E-19	8.91E-19	7.61E-19	3.58E-21	2.97E-18
2.70E-17	2.28E-17	2.45E-17	2.75E-17	2.87E-17	1.60E-17	9.23E-18	1.03E-17	8.43E-18	4.18E-18	3.84E-19	1.59E-18	2.18E-18	1.30E-20	1.42E-18
3.88E-17	3.45E-17	3.07E-17	4.16E-17	4.05E-17	2.25E-17	1.98E-17	2.87E-17	2.07E-17	1.45E-17	6.43E-18	7.31E-18	4.60E-18	3.29E-19	2.80E-18
4.79E-17	4.37E-17	3.30E-17	4.60E-17	4.81E-17	3.55E-17	3.63E-17	4.59E-17	3.06E-17	2.80E-17	1.73E-17	1.23E-17	1.03E-17	3.78E-18	7.84E-18

5.93E-17	5.37E-17	4.47E-17	5.26E-17	5.78E-17	4.72E-17	5.30E-17	5.18E-17	3.77E-17	3.29E-17	2.73E-17	1.85E-17	1.45E-17	1.35E-17	2.12E-17
6.69E-17	5.83E-17	5.49E-17	6.06E-17	6.14E-17	5.27E-17	6.37E-17	6.18E-17	4.42E-17	3.50E-17	3.15E-17	2.39E-17	1.48E-17	2.07E-17	2.25E-17
6.72E-17	6.21E-17	6.62E-17	7.31E-17	6.63E-17	5.81E-17	6.03E-17	6.32E-17	5.28E-17	3.89E-17	2.34E-17	2.32E-17	1.37E-17	1.76E-17	2.10E-17
6.52E-17	5.78E-17	7.13E-17	7.96E-17	6.54E-17	5.61E-17	5.57E-17	6.07E-17	6.28E-17	4.57E-17	2.30E-17	3.03E-17	2.19E-17	1.62E-17	1.75E-17
6.49E-17	6.24E-17	7.04E-17	7.75E-17	7.17E-17	5.33E-17	4.72E-17	5.35E-17	6.17E-17	4.58E-17	2.93E-17	3.73E-17	3.23E-17	2.16E-17	1.54E-17
5.94E-17	7.01E-17	7.20E-17	7.44E-17	7.30E-17	5.90E-17	5.05E-17	5.10E-17	5.20E-17	4.23E-17	3.28E-17	3.75E-17	3.59E-17	2.38E-17	1.77E-17
5.30E-17	7.31E-17	6.79E-17	6.87E-17	7.22E-17	5.97E-17	5.38E-17	5.16E-17	4.87E-17	4.01E-17	3.41E-17	3.63E-17	3.41E-17	2.38E-17	2.58E-17
5.32E-17	7.10E-17	6.57E-17	6.74E-17	7.14E-17	6.37E-17	5.54E-17	5.51E-17	4.82E-17	3.98E-17	3.83E-17	3.58E-17	3.16E-17	2.42E-17	2.77E-17
5.59E-17	6.84E-17	6.11E-17	6.42E-17	6.93E-17	6.55E-17	6.11E-17	5.92E-17	5.07E-17	4.70E-17	3.99E-17	3.59E-17	3.54E-17	2.48E-17	2.23E-17
6.00E-17	5.69E-17	4.95E-17	6.27E-17	7.08E-17	6.34E-17	6.14E-17	6.02E-17	5.06E-17	4.65E-17	3.73E-17	3.59E-17	3.56E-17	2.98E-17	1.99E-17
5.99E-17	4.76E-17	4.04E-17	6.12E-17	7.12E-17	6.04E-17	5.91E-17	6.16E-17	4.87E-17	4.57E-17	3.52E-17	3.30E-17	3.32E-17	3.04E-17	2.32E-17
2.30E-14	2.24E-14	2.16E-14	2.12E-14	2.04E-14	1.98E-14	1.93E-14	1.89E-14	1.78E-14	1.69E-14	1.59E-14	1.56E-14	1.54E-14	1.50E-14	1.48E-14

3.39E-08	3.50E-08	3.39E-08	3.33E-08	3.39E-08	3.34E-08	3.34E-08	3.35E-08	3.23E-08	3.32E-08	3.37E-08	3.33E-08	3.22E-08	3.06E-08	2.96E-08
3.25E-08	3.30E-08	3.23E-08	3.20E-08	3.25E-08	3.20E-08	3.18E-08	3.18E-08	3.14E-08	3.22E-08	3.22E-08	3.21E-08	3.07E-08	2.90E-08	2.80E-08
3.11E-08	3.14E-08	3.05E-08	3.10E-08	3.13E-08	3.08E-08	3.08E-08	3.07E-08	3.08E-08	3.09E-08	3.03E-08	3.03E-08	2.91E-08	2.75E-08	2.64E-08
2.97E-08	2.99E-08	2.93E-08	2.97E-08	3.00E-08	2.95E-08	2.96E-08	2.96E-08	3.00E-08	2.93E-08	2.90E-08	2.88E-08	2.77E-08	2.65E-08	2.48E-08
2.84E-08	2.88E-08	2.81E-08	2.87E-08	2.83E-08	2.79E-08	2.80E-08	2.81E-08	2.86E-08	2.79E-08	2.74E-08	2.73E-08	2.65E-08	2.57E-08	2.34E-08
2.73E-08	2.79E-08	2.71E-08	2.79E-08	2.70E-08	2.67E-08	2.65E-08	2.65E-08	2.70E-08	2.67E-08	2.61E-08	2.57E-08	2.55E-08	2.50E-08	2.29E-08
2.61E-08	2.68E-08	2.62E-08	2.61E-08	2.59E-08	2.59E-08	2.57E-08	2.54E-08	2.59E-08	2.58E-08	2.46E-08	2.42E-08	2.39E-08	2.40E-08	2.22E-08
2.49E-08	2.52E-08	2.51E-08	2.49E-08	2.50E-08	2.53E-08	2.49E-08	2.41E-08	2.50E-08	2.50E-08	2.32E-08	2.26E-08	2.21E-08	2.26E-08	2.11E-08
2.38E-08	2.37E-08	2.36E-08	2.36E-08	2.40E-08	2.48E-08	2.38E-08	2.30E-08	2.34E-08	2.37E-08	2.21E-08	2.17E-08	2.04E-08	2.10E-08	1.99E-08
2.27E-08	2.27E-08	2.25E-08	2.26E-08	2.28E-08	2.36E-08	2.20E-08	2.19E-08	2.13E-08	2.19E-08	2.11E-08	2.07E-08	1.88E-08	1.92E-08	1.80E-08
2.12E-08	2.16E-08	2.12E-08	2.12E-08	2.14E-08	2.19E-08	2.06E-08	2.08E-08	1.97E-08	2.02E-08	1.96E-08	1.94E-08	1.75E-08	1.79E-08	1.63E-08
2.00E-08	2.02E-08	1.98E-08	1.97E-08	1.98E-08	2.05E-08	1.91E-08	1.97E-08	1.85E-08	1.87E-08	1.82E-08	1.82E-08	1.66E-08	1.71E-08	1.52E-08
1.89E-08	1.89E-08	1.85E-08	1.89E-08	1.86E-08	1.94E-08	1.81E-08	1.84E-08	1.81E-08	1.76E-08	1.72E-08	1.72E-08	1.58E-08	1.53E-08	1.41E-08
1.73E-08	1.72E-08	1.75E-08	1.80E-08	1.80E-08	1.83E-08	1.75E-08	1.68E-08	1.68E-08	1.68E-08	1.62E-08	1.58E-08	1.53E-08	1.43E-08	1.37E-08
1.58E-08	1.60E-08	1.66E-08	1.72E-08	1.68E-08	1.71E-08	1.69E-08	1.61E-08	1.62E-08	1.60E-08	1.54E-08	1.48E-08	1.43E-08	1.34E-08	1.30E-08
1.46E-08	1.48E-08	1.56E-08	1.60E-08	1.57E-08	1.61E-08	1.61E-08	1.54E-08	1.48E-08	1.47E-08	1.41E-08	1.40E-08	1.34E-08	1.27E-08	1.27E-08
1.35E-08	1.41E-08	1.45E-08	1.47E-08	1.45E-08	1.47E-08	1.48E-08	1.42E-08	1.32E-08	1.31E-08	1.32E-08	1.34E-08	1.22E-08	1.18E-08	1.23E-08
1.27E-08	1.32E-08	1.35E-08	1.32E-08	1.30E-08	1.33E-08	1.27E-08	1.31E-08	1.21E-08	1.22E-08	1.26E-08	1.23E-08	1.12E-08	1.12E-08	1.30E-08
1.19E-08	1.22E-08	1.27E-08	1.22E-08	1.19E-08	1.17E-08	1.18E-08	1.21E-08	1.17E-08	1.14E-08	1.21E-08	1.15E-08	1.07E-08	1.15E-08	1.59E-08
1.14E-08	1.10E-08	1.20E-08	1.13E-08	1.11E-08	1.11E-08	1.11E-08	1.14E-08	1.16E-08	1.07E-08	1.13E-08	1.09E-08	1.00E-08	1.31E-08	2.30E-08
1.09E-08	9.98E-09	1.12E-08	1.07E-08	1.04E-08	1.06E-08	1.04E-08	1.07E-08	1.08E-08	1.00E-08	9.87E-09	9.50E-09	8.95E-09	1.58E-08	3.30E-08
1.02E-08	9.34E-09	1.06E-08	1.06E-08	1.02E-08	1.04E-08	1.01E-08	1.00E-08	9.61E-09	8.67E-09	8.57E-09	7.95E-09	8.14E-09	1.60E-08	2.87E-08
9.20E-09	8.59E-09	9.62E-09	9.65E-09	9.95E-09	9.70E-09	9.10E-09	8.85E-09	8.82E-09	7.74E-09	7.81E-09	6.66E-09	7.55E-09	7.59E-09	9.82E-09
8.06E-09	8.21E-09	8.91E-09	8.36E-09	9.10E-09	8.74E-09	8.22E-09	7.67E-09	8.31E-09	7.15E-09	7.26E-09	5.89E-09	6.97E-09	6.97E-09	4.74E-09
7.37E-09	7.91E-09	7.80E-09	7.59E-09	8.05E-09	7.64E-09	7.66E-09	6.90E-09	7.81E-09	6.17E-09	6.35E-09	5.06E-09	5.48E-09	5.75E-09	4.21E-09
6.65E-09	7.19E-09	6.78E-09	6.83E-09	6.86E-09	6.52E-09	6.77E-09	6.01E-09	6.66E-09	5.44E-09	5.17E-09	4.37E-09	4.48E-09	4.23E-09	2.69E-09
5.77E-09	5.75E-09	5.56E-09	6.00E-09	5.76E-09	5.45E-09	5.82E-09	5.47E-09	5.92E-09	4.68E-09	4.25E-09	3.39E-09	3.28E-09	3.51E-09	1.79E-09
4.88E-09	4.55E-09	4.29E-09	5.01E-09	4.84E-09	4.59E-09	5.01E-09	5.13E-09	5.46E-09	3.97E-09	3.24E-09	2.79E-09	2.71E-09	2.56E-09	1.30E-09
3.92E-09	3.50E-09	2.94E-09	3.68E-09	4.13E-09	3.88E-09	4.25E-09	4.62E-09	4.49E-09	3.45E-09	2.45E-09	2.04E-09	1.88E-09	1.66E-09	8.53E-10
3.04E-09	2.54E-09	2.20E-09	3.05E-09	2.56E-09	2.59E-09	3.08E-09	3.22E-09	3.21E-09	2.35E-09	1.43E-09	1.49E-09	1.26E-09	1.74E-09	8.80E-10
2.17E-09	1.92E-09	2.08E-09	2.31E-09	1.67E-09	1.46E-09	1.98E-09	1.80E-09	2.06E-09	1.35E-09	7.86E-10	1.10E-09	8.86E-10	1.71E-09	8.90E-10
1.79E-09	1.27E-09	2.00E-09	1.45E-09	1.55E-09	1.27E-09	1.05E-09	1.11E-09	1.24E-09	6.25E-10	4.08E-10	3.93E-10	4.27E-10	1.70E-09	9.91E-10
9.39E-10	5.29E-10	2.12E-09	9.31E-10	1.66E-09	1.13E-09	3.88E-10	3.80E-10	7.98E-10	-3.51E-10	-3.64E-10	-1.73E-10	-1.77E-10	1.22E-09	7.50E-10
-6.17E-11	2.77E-10	1.77E-09	6.71E-10	1.58E-09	7.88E-10	-2.14E-10	-9.81E-13	1.18E-10	-5.18E-10	-1.06E-09	-8.37E-10	-1.00E-09	2.25E-10	-3.52E-10
-1.19E-09	-1.11E-11	1.73E-09	4.77E-10	1.05E-09	4.08E-10	-9.98E-10	-7.77E-10	-6.54E-10	-7.65E-10	-1.57E-09	-1.64E-09	-1.83E-09	-1.37E-09	-1.48E-09
-2.08E-09	-4.21E-10	1.16E-09	-5.43E-11	-6.28E-10	-1.06E-09	-1.86E-09	-1.74E-09	-1.38E-09	-1.42E-09	-2.15E-09	-2.10E-09	-1.93E-09	-1.93E-09	-2.61E-09
-2.83E-09	-1.16E-09	-4.14E-10	-1.31E-09	-1.68E-09	-2.25E-09	-2.06E-09	-2.00E-09	-2.42E-09	-2.11E-09	-2.29E-09	-2.44E-09	-2.03E-09	-2.54E-09	-3.38E-09
-3.59E-09	-2.20E-09	-2.14E-09	-2.66E-09	-2.81E-09	-3.05E-09	-2.27E-09	-2.53E-09	-3.29E-09	-2.60E-09	-2.79E-09	-2.52E-09	-2.31E-09	-3.71E-09	-3.92E-09
-4.00E-09	-3.76E-09	-3.19E-09	-3.06E-09	-3.70E-09	-3.82E-09	-2.26E-09	-3.06E-09	-3.95E-09	-3.81E-09	-3.44E-09	-3.24E-09	-3.06E-09	-4.48E-09	-4.43E-09
-4.39E-09	-4.52E-09	-3.60E-09	-2.96E-09	-4.03E-09	-3.99E-09	-2.37E-09	-3.60E-09	-4.71E-09	-4.23E-09	-4.21E-09	-4.31E-09	-3.74E-09	-5.31E-09	-5.21E-09
-4.71E-09	-5.05E-09	-3.81E-09	-2.85E-09	-3.99E-09	-4.25E-09	-3.56E-09	-4.21E-09	-5.03E-09	-4.42E-09	-4.88E-09	-5.14E-09	-4.94E-09	-5.98E-09	-5.88E-09
-5.07E-09	-5.41E-09	-4.28E-09	-3.11E-09	-4.38E-09	-4.56E-09	-4.58E-09	-4.58E-09	-5.04E-09	-4.67E-09	-5.42E-09	-5.64E-09	-6.00E-09	-6.84E-09	-6.28E-09
-5.34E-09	-5.39E-09	-5.07E-09	-4.21E-09	-5.02E-09	-5.32E-09	-5.02E-09	-5.05E-09	-5.33E-09	-4.98E-09	-5.40E-09	-5.69E-09	-6.20E-09	-7.43E-09	-6.72E-09
-5.89E-09	-5.71E-09	-5.83E-09	-5.10E-09	-5.52E-09	-5.80E-09	-5.36E-09	-5.31E-09	-5.45E-09	-5.23E-09	-5.55E-09	-5.44E-09	-6.29E-09	-7.49E-09	-6.84E-09
-6.79E-09	-6.52E-09	-6.79E-09	-5.79E-09	-6.17E-09	-6.03E-09	-5.66E-09	-5.02E-09	-5.37E-09	-5.61E-09	-5.86E-09	-5.41E-09	-5.91E-09	-7.17E-09	-6.92E-09
-7.33E-09	-6.58E-09	-7.02E-09	-6.12E-09	-6.54E-09	-6.04E-09	-5.88E-09	-5.34E-09	-5.64E-09	-6.05E-09	-6.25E-09	-5.66E-09	-5.58E-09	-6.82E-09	-6.95E-09
-7.71E-09	-6.84E-09	-6.70E-09	-6.16E-09	-6.37E-09	-6.24E-09	-6.39E-09	-5.78E-09	-6.10E-09	-6.49E-09	-6.39E-09	-6.05E-09	-5.71E-09	-6.55E-09	-6.87E-09
-8.07E-09	-7.17E-09	-6.61E-09	-6.33E-09	-6.27E-09	-6.35E-09	-6.43E-09	-6.74E-09	-6.66E-09	-7.21E-09	-7.00E-09	-6.29E-09	-6.50E-09	-6.85E-09	-6.90E-09
-8.32E-09	-7.56E-09	-6.56E-09	-6.14E-09	-5.75E-09	-6.08E-09	-6.64E-09	-6.46E-09	-6.70E-09	-7.40E-09	-7.65E-09	-6.52E-09	-7.10E-09	-6.99E-09	-6.76E-09
-8.86E-09	-7.52E-09	-6.98E-09	-6.12E-09	-5.55E-09	-6.21E-09	-7.23E-09	-6.35E-09	-7.07E-09	-7.23E-09	-7.70E-09	-6.42E-09	-6.87E-09	-7.49E-09	-6.90E-09
-9.28E-09	-7.44E-09	-7.00E-09	-5.80E-09	-5.47E-09	-6.70E-09	-7.60E-09	-6.87E-09	-7.42E-09	-7.25E-09	-7.34E-09	-6.89E-09	-6.93E-09	-8.01E-09	-7.63E-09
-9.15E-09	-7.63E-09	-6.81E-09	-6.54E-09	-6.35E-09	-7.32E-09	-7.89E-09	-7.85E-09	-8.13E-09	-7.16E-09	-7.34E-09	-6.99E-09	-7.19E-09	-8.26E-09	-7.84E-09
-8.96E-09	-8.50E-09	-8.02E-09	-7.44E-09	-7.97E-09	-8.21E-09	-8.83E-09	-8.65E-09	-8.50E-09	-7.22E-09	-7.46E-09	-7.41E-09	-7.70E-09	-8.60E-09	-8.15E-09
-8.25E-09	-8.18E-09	-8.89E-09	-8.14E-09	-8.69E-09	-8.64E-09	-9.25E-09	-9.33E-09	-8.61E-09	-7.32E-09	-7.21E-09	-7.83E-09	-8.25E-09	-8.32E-09	-7.98E-09
-7.90E-09	-7.52E-09	-9.52E-09	-9.06E-09	-9.06E-09	-9.23E-09	-9.60E-09	-9.08E-09	-8.70E-09	-7.62E-09	-6.98E-09	-8.51E-09	-8.45E-09	-7.92E-09	-7.64E-09
-8.09E-09	-7.33E-09	-9.75E-09	-9.49E-09	-9.08E-09	-9.44E-09	-9.66E-09	-8.95E-09	-8.78E-09	-7.70E-09	-7.13E-09	-8.49E-09	-8.28E-09	-7.12E-09	-6.71E-09
-7.31E-09	-7.64E-09	-9.52E-09	-9.31E-09	-8.44E-09	-9.70E-09	-9.32E-09	-8.92E-09	-8.81E-09	-7.96E-09	-7.93E-09	-8.43E-09	-7.55E-09	-6.49E-09	-6.75E-09
-6.38E-09	-7.33E-09	-8.95E-09	-8.83E-09	-8.08E-09	-9.75E-09	-9.08E-09	-8.82E-09	-9.18E-09	-8.97E-09	-8.59E-09	-8.13E-09	-7.46E-09	-6.58E-09	-6.98E-09
-5.70E-09	-7.65E-09	-8.76E-09	-8.58E-09	-8.71E-09	-9.66E-09	-8.60E-09	-8.37E-09	-9.35E-09	-9.23E-09	-9.29E-09	-8.36E-09	-7.67E-09	-7.18E-09	-7.58E-09
-4.96E-09	-8.06E-09	-8.97E-09	-8.84E-09	-9.10E-09	-9.32E-09	-8.18E-09	-7.78E-09	-8.86E-09	-8.89E-09	-9.16E-09	-8.66E-09	-8.34E-09	-7.34E-09	-7.58E-09
-4.78E-09	-7.68E-09	-8.46E-09	-8.70E-09	-8.92E-09	-8.96E-09	-8.20E-09	-7.31E-09	-8.20E-09	-8.83E-09	-8.53E-09	-8.34E-09	-7.88E-09	-6.45E-09	-6.74E-09
-4.87E-09	-7.13E-09	-7.59E-09	-7.56E-09	-7.80E-09	-8.17E-09	-7.47E-09	-6.63E-09	-7.47E-09	-8.62E-09	-8.03E-09	-7.93E-09	-7.00E-09	-6.03E-09	-5.64E-09
-5.36E-09	-7.24E-09	-7.13E-09	-6.90E-09	-7.09E-09	-7.38E-09	-7.54E-09	-6.57E-09	-6.42E-09	-8.19E-09	-7.72E-09	-6.96E-09	-6.32E-09	-5.20E-09	-4.58E-09
-5.47E-09	-7.24E-09	-7.27E-09	-7.12E-09	-6.98E-09	-6.70E-09	-7.15E-09	-6.32E-09	-5.85E-09	-7.56E-09	-7.09E-09	-6.14E-09	-5.49E-09	-4.42E-09	-3.66E-09
-4.87E-09	-5.96E-09	-6.85E-09	-6.95E-09	-6.74E-09	-5.95E-09	-6.50E-09	-6.44E-09	-5.29E-09	-6.55E-09	-6.71E-09	-5.89E-09	-4.99E-09	-4.13E-09	-4.41E-09
-3.22E-09	-4.24E-09	-5.55E-09	-6.45E-09	-6.10E-09	-5.67E-09	-5.94E-09	-5.96E-09	-4.53E-09	-5.37E-09	-6.72E-09	-5.45E-09	-3.61E-09	-4.03E-09	-5.29E-09
-2.08E-09	-3.25E-09	-3.92E-09	-5.45E-09	-5.90E-09	-5.14E-09	-5.58E-09	-5.25E-09	-4.12E-09	-4.49E-09	-6.47E-09	-5.63E-09	-2.99E-09	-3.79E-09	-5.77E-09
-8.57E-10	-1.62E-09	-2.58E-09	-5.08E-09	-6.20E-09	-4.75E-09	-4.60E-09	-4.47E-09	-4.16E-09	-3.83E-09	-6.29E-09	-5.92E-09	-3.99E-09	-3.32E-09	-5.34E-09
-9.31E-10	2.95E-10	-1.43E-09	-4.20E-09	-6.11E-09	-4.56E-09	-4.73E-09	-4.81E-09	-4.46E-09	-3.55E-09	-5.67E-09	-6.57E-09	-5.26E-09	-4.06E-09	-5.25E-09
-7.00E-10	1.06E-09	-1.13E-09	-3.57E-09	-6.03E-09	-4.63E-09	-5.34E-09	-6.36E-09	-5.05E-09	-3.56E-09	-4.77E-09	-6.76E-09	-5.62E-09	-5.08E-09	-6.07E-09
-4.27E-10	5.76E-10	-1.95E-09	-4.04E-09	-5.81E-09	-5.13E-09	-6.20E-09	-8.04E-09	-6.95E-09	-5.04E-09	-4.76E-09	-6.48E-09	-5.59E-09	-5.80E-09	-6.71E-09
-5.26E-10	-5.60E-11	-2.52E-09	-4.75E-09	-5.83E-09	-5.26E-09	-6.73E-09	-9.12E-09	-8.11E-09	-6.65E-09	-5.69E-09	-6.18E-09	-6.27E-09	-6.45E-09	-6.69E-09
-2.70E-09	-1.61E-09	-3.19E-09	-5.30E-09	-6.03E-09	-5.36E-09	-7.93E-09	-9.50E-09	-8.79E-09	-7.70E-09	-6.97E-09	-6.83E-09	-7.64E-09	-7.04E-09	-6.92E-09
-4.53E-09	-4.16E-09	-3.81E-09	-6.07E-09	-6.68E-09	-6.42E-09	-9.38E-09	-1.02E-08	-1.00E-08	-8.36E-09	-7.83E-09	-8.24E-09	-8.59E-09	-7.41E-09	-7.75E-09
-5.03E-09	-5.69E-09	-4.68E-09	-6.79E-09	-7.53E-09	-7.67E-09	-1.02E-08	-1.05E-08	-1.05E-08	-8.86E-09	-8.92E-09	-8.96E-09	-8.91E-09	-7.93E-09	-8.01E-09
-5.69E-09	-6.59E-09	-6.13E-09	-7.01E-09	-8.31E-09	-8.78E-09	-1.03E-08	-1.02E-08	-1.01E-08	-8.94E-09	-9.03E-09	-8.84E-09	-8.63E-09	-8.12E-09	-7.92E-09
-6.35E-09	-7.68E-09	-7.45E-09	-7.83E-09	-8.90E-09	-9.17E-09	-1.03E-08	-9.89E-09	-9.64E-09	-8.87E-09	-8.45E-09	-8.19E-09	-7.98E-09	-7.74E-09	-7.32E-09
-6.97E-09	-8.23E-09	-8.38E-09	-8.75E-09	-9.26E-09	-9.23E-09	-9.83E-09	-9.73E-09	-9.23E-09	-9.19E-09	-7.66E-09	-7.58E-09	-7.39E-09	-7.15E-09	-6.90E-09

-8.38E-09	-8.78E-09	-9.20E-09	-8.99E-09	-8.85E-09	-8.40E-09	-9.04E-09	-9.40E-09	-8.88E-09	-8.40E-09	-6.67E-09	-6.88E-09	-7.17E-09	-6.47E-09	-7.05E-09
-9.82E-09	-9.68E-09	-9.61E-09	-8.99E-09	-7.88E-09	-7.29E-09	-7.97E-09	-8.54E-09	-8.80E-09	-7.83E-09	-6.40E-09	-7.20E-09	-6.82E-09	-6.75E-09	-7.44E-09
-1.09E-08	-1.02E-08	-9.75E-09	-8.99E-09	-7.65E-09	-6.86E-09	-6.81E-09	-7.66E-09	-8.43E-09	-7.35E-09	-6.06E-09	-7.38E-09	-7.12E-09	-7.26E-09	-7.90E-09
-1.10E-08	-1.06E-08	-1.01E-08	-9.27E-09	-8.50E-09	-7.57E-09	-6.47E-09	-7.62E-09	-8.70E-09	-7.43E-09	-6.59E-09	-8.06E-09	-8.39E-09	-7.90E-09	-8.50E-09
-1.07E-08	-1.08E-08	-1.03E-08	-9.66E-09	-9.05E-09	-8.70E-09	-7.95E-09	-8.51E-09	-8.99E-09	-8.29E-09	-7.76E-09	-8.95E-09	-9.36E-09	-8.89E-09	-9.03E-09
-1.07E-08	-1.04E-08	-1.01E-08	-9.79E-09	-9.57E-09	-9.04E-09	-8.82E-09	-8.39E-09	-8.48E-09	-8.61E-09	-8.71E-09	-9.55E-09	-9.86E-09	-9.34E-09	-9.59E-09
-1.05E-08	-1.03E-08	-1.02E-08	-9.34E-09	-9.30E-09	-8.90E-09	-8.49E-09	-7.63E-09	-7.75E-09	-8.45E-09	-8.50E-09	-9.39E-09	-9.42E-09	-8.34E-09	-8.95E-09
-1.01E-08	-1.01E-08	-1.01E-08	-8.99E-09	-8.64E-09	-8.05E-09	-7.92E-09	-7.92E-09	-7.70E-09	-8.33E-09	-9.11E-09	-9.12E-09	-8.64E-09	-7.09E-09	-8.57E-09
-9.82E-09	-9.90E-09	-9.95E-09	-8.13E-09	-7.62E-09	-7.44E-09	-7.35E-09	-7.99E-09	-7.89E-09	-7.95E-09	-9.24E-09	-8.71E-09	-7.38E-09	-5.86E-09	-7.68E-09
-9.77E-09	-9.26E-09	-9.52E-09	-7.98E-09	-7.37E-09	-7.10E-09	-6.97E-09	-7.13E-09	-7.63E-09	-7.89E-09	-8.64E-09	-8.19E-09	-6.17E-09	-4.44E-09	-6.94E-09
-9.03E-09	-8.92E-09	-9.66E-09	-8.76E-09	-7.91E-09	-6.96E-09	-6.20E-09	-6.00E-09	-7.21E-09	-7.95E-09	-8.55E-09	-7.51E-09	-5.82E-09	-5.20E-09	-6.98E-09
-8.87E-09	-8.83E-09	-9.18E-09	-8.54E-09	-8.36E-09	-7.39E-09	-6.54E-09	-5.75E-09	-6.84E-09	-7.88E-09	-8.65E-09	-7.91E-09	-6.90E-09	-6.93E-09	-7.74E-09
-8.32E-09	-8.51E-09	-8.18E-09	-8.11E-09	-8.07E-09	-7.85E-09	-6.99E-09	-5.52E-09	-6.37E-09	-7.48E-09	-8.53E-09	-8.38E-09	-7.80E-09	-7.97E-09	-8.37E-09
-7.74E-09	-8.09E-09	-8.02E-09	-7.54E-09	-7.41E-09	-7.91E-09	-7.06E-09	-5.54E-09	-6.43E-09	-7.11E-09	-7.76E-09	-7.87E-09	-8.47E-09	-8.56E-09	-8.52E-09
-7.29E-09	-7.58E-09	-8.02E-09	-7.56E-09	-6.48E-09	-7.91E-09	-7.32E-09	-5.97E-09	-5.80E-09	-6.58E-09	-6.84E-09	-7.33E-09	-8.53E-09	-8.52E-09	-8.33E-09
-6.45E-09	-7.47E-09	-7.97E-09	-7.67E-09	-5.93E-09	-7.94E-09	-7.48E-09	-6.63E-09	-5.50E-09	-6.37E-09	-5.98E-09	-6.80E-09	-7.87E-09	-8.27E-09	-8.17E-09
-5.61E-09	-7.31E-09	-7.54E-09	-7.45E-09	-5.46E-09	-7.57E-09	-6.90E-09	-6.53E-09	-6.50E-09	-6.48E-09	-5.39E-09	-6.38E-09	-7.42E-09	-8.03E-09	-7.68E-09
-6.10E-09	-7.33E-09	-6.94E-09	-6.91E-09	-5.47E-09	-6.74E-09	-6.03E-09	-6.23E-09	-6.59E-09	-6.65E-09	-5.74E-09	-7.34E-09	-7.54E-09	-7.19E-09	-7.48E-09
-5.70E-09	-6.60E-09	-6.08E-09	-6.00E-09	-5.04E-09	-5.92E-09	-5.16E-09	-5.57E-09	-5.94E-09	-6.14E-09	-5.32E-09	-7.31E-09	-7.31E-09	-6.50E-09	-7.19E-09
-5.14E-09	-5.50E-09	-5.08E-09	-5.09E-09	-5.02E-09	-5.26E-09	-4.65E-09	-5.11E-09	-5.70E-09	-5.86E-09	-4.48E-09	-6.29E-09	-6.23E-09	-6.68E-09	-6.85E-09
-4.39E-09	-4.60E-09	-4.77E-09	-5.03E-09	-5.01E-09	-4.88E-09	-4.74E-09	-4.71E-09	-5.51E-09	-5.71E-09	-4.07E-09	-5.49E-09	-5.23E-09	-6.29E-09	-6.76E-09
-4.82E-09	-5.07E-09	-4.82E-09	-4.91E-09	-4.34E-09	-4.99E-09	-4.94E-09	-4.30E-09	-4.91E-09	-5.44E-09	-4.75E-09	-5.29E-09	-4.48E-09	-6.22E-09	-7.15E-09
-4.94E-09	-5.50E-09	-4.33E-09	-4.99E-09	-4.14E-09	-4.67E-09	-4.69E-09	-4.30E-09	-3.78E-09	-4.91E-09	-5.38E-09	-5.46E-09	-4.34E-09	-5.80E-09	-7.34E-09
-4.76E-09	-5.39E-09	-4.46E-09	-5.40E-09	-4.53E-09	-4.45E-09	-3.74E-09	-4.91E-09	-3.60E-09	-4.99E-09	-5.73E-09	-5.81E-09	-4.12E-09	-5.04E-09	-6.27E-09
-4.53E-09	-5.03E-09	-4.71E-09	-5.58E-09	-4.69E-09	-4.75E-09	-3.98E-09	-5.31E-09	-3.11E-09	-5.80E-09	-5.84E-09	-5.37E-09	-3.02E-09	-3.92E-09	-5.40E-09
-4.16E-09	-4.54E-09	-4.85E-09	-5.53E-09	-5.09E-09	-5.03E-09	-4.36E-09	-5.17E-09	-5.88E-09	-6.13E-09	-5.58E-09	-4.75E-09	-2.38E-09	-3.13E-09	-4.02E-09
-3.08E-09	-4.03E-09	-4.82E-09	-5.23E-09	-4.95E-09	-4.74E-09	-4.52E-09	-4.76E-09	-5.46E-09	-5.51E-09	-5.63E-09	-4.74E-09	-2.62E-09	-3.45E-09	-3.48E-09
-2.22E-09	-3.44E-09	-4.44E-09	-4.48E-09	-4.25E-09	-4.04E-09	-4.23E-09	-4.63E-09	-5.41E-09	-5.39E-09	-5.67E-09	-4.95E-09	-4.12E-09	-4.41E-09	-3.53E-09
-1.82E-09	-3.40E-09	-4.36E-09	-4.16E-09	-3.35E-09	-3.44E-09	-3.36E-09	-5.14E-09	-5.60E-09	-5.81E-09	-5.58E-09	-5.36E-09	-5.24E-09	-5.00E-09	-4.17E-09
-2.54E-09	-3.58E-09	-4.42E-09	-4.24E-09	-2.92E-09	-3.14E-09	-3.26E-09	-5.99E-09	-6.06E-09	-6.15E-09	-5.50E-09	-5.66E-09	-5.18E-09	-5.02E-09	-4.76E-09
-3.06E-09	-3.92E-09	-4.57E-09	-4.90E-09	-3.94E-09	-3.41E-09	-3.37E-09	-5.99E-09	-5.84E-09	-5.96E-09	-5.35E-09	-5.52E-09	-4.42E-09	-4.48E-09	-4.65E-09
-3.22E-09	-4.48E-09	-4.59E-09	-5.31E-09	-4.62E-09	-4.25E-09	-3.46E-09	-4.77E-09	-5.08E-09	-5.56E-09	-5.23E-09	-4.90E-09	-3.11E-09	-3.66E-09	-4.52E-09
-3.57E-09	-4.81E-09	-4.32E-09	-5.00E-09	-4.68E-09	-4.95E-09	-3.15E-09	-3.41E-09	-4.71E-09	-5.03E-09	-4.78E-09	-4.58E-09	-2.93E-09	-3.99E-09	-4.91E-09
-3.50E-09	-4.64E-09	-3.72E-09	-4.47E-09	-4.20E-09	-4.81E-09	-3.79E-09	-3.49E-09	-4.50E-09	-4.77E-09	-4.48E-09	-3.70E-09	-3.82E-09	-5.23E-09	-5.40E-09
-3.22E-09	-4.30E-09	-3.52E-09	-4.19E-09	-3.79E-09	-4.41E-09	-4.34E-09	-4.34E-09	-4.44E-09	-4.40E-09	-4.64E-09	-3.02E-09	-3.46E-09	-5.30E-09	-5.78E-09
-3.08E-09	-4.45E-09	-4.44E-09	-4.43E-09	-3.76E-09	-4.19E-09	-4.56E-09	-4.87E-09	-4.28E-09	-3.92E-09	-4.37E-09	-2.29E-09	-3.11E-09	-4.89E-09	-5.48E-09
-3.65E-09	-4.66E-09	-4.38E-09	-4.47E-09	-4.55E-09	-4.10E-09	-4.67E-09	-4.78E-09	-3.93E-09	-3.53E-09	-4.14E-09	-2.28E-09	-2.48E-09	-4.48E-09	-4.73E-09
-3.55E-09	-4.34E-09	-3.58E-09	-3.85E-09	-4.36E-09	-4.35E-09	-4.78E-09	-4.68E-09	-3.81E-09	-3.73E-09	-4.20E-09	-2.31E-09	-2.39E-09	-3.71E-09	-3.46E-09
-3.45E-09	-3.45E-09	-3.27E-09	-3.48E-09	-4.03E-09	-4.71E-09	-4.96E-09	-4.37E-09	-3.66E-09	-3.63E-09	-4.01E-09	-2.57E-09	-2.64E-09	-2.48E-09	-1.52E-09
-3.81E-09	-2.93E-09	-3.03E-09	-3.64E-09	-3.84E-09	-4.58E-09	-4.51E-09	-3.80E-09	-3.63E-09	-3.55E-09	-3.66E-09	-3.12E-09	-3.14E-09	-1.83E-09	-2.26E-10
-4.74E-09	-3.59E-09	-3.19E-09	-2.97E-09	-3.48E-09	-4.37E-09	-3.79E-09	-3.34E-09	-3.79E-09	-3.69E-09	-3.43E-09	-3.76E-09	-2.93E-09	-2.34E-09	-2.27E-10
-5.53E-09	-4.39E-09	-3.50E-09	-2.71E-09	-3.44E-09	-4.06E-09	-3.66E-09	-3.10E-09	-3.98E-09	-3.49E-09	-3.03E-09	-3.86E-09	-3.20E-09	-2.57E-09	-6.54E-10
-5.34E-09	-4.19E-09	-3.78E-09	-3.18E-09	-3.70E-09	-3.92E-09	-3.27E-09	-3.10E-09	-3.98E-09	-3.29E-09	-2.65E-09	-3.23E-09	-3.00E-09	-2.70E-09	-8.23E-10
-4.70E-09	-4.07E-09	-4.13E-09	-4.07E-09	-4.25E-09	-3.51E-09	-2.91E-09	-3.06E-09	-3.49E-09	-2.90E-09	-2.09E-09	-2.64E-09	-2.39E-09	-2.44E-09	-1.52E-09
-4.71E-09	-4.58E-09	-4.25E-09	-4.32E-09	-4.19E-09	-2.78E-09	-2.63E-09	-3.31E-09	-3.09E-09	-2.62E-09	-2.21E-09	-1.90E-09	-1.51E-09	-1.43E-09	-1.88E-09
-4.58E-09	-4.67E-09	-4.36E-09	-3.91E-09	-3.90E-09	-2.96E-09	-2.54E-09	-2.98E-09	-2.73E-09	-2.03E-09	-1.42E-09	-1.02E-09	-8.01E-10	-5.42E-10	-1.09E-09
-4.42E-09	-4.43E-09	-4.43E-09	-3.95E-09	-3.50E-09	-2.82E-09	-2.41E-09	-2.62E-09	-2.27E-09	-1.22E-09	-4.44E-10	4.85E-10	2.78E-11	6.32E-10	-3.34E-10
-4.13E-09	-4.12E-09	-4.15E-09	-3.64E-09	-3.04E-09	-2.60E-09	-1.80E-09	-1.86E-09	-1.33E-09	1.41E-10	1.25E-09	2.10E-09	1.41E-09	9.42E-10	9.97E-10
-3.90E-09	-4.04E-09	-3.31E-09	-2.84E-09	-2.36E-09	-2.01E-09	-1.24E-09	-6.65E-10	1.98E-10	2.53E-09	3.97E-09	5.03E-09	3.84E-09	2.92E-09	2.69E-09
-4.08E-09	-3.52E-09	-2.66E-09	-2.20E-09	-2.17E-09	-2.28E-09	-8.48E-10	6.90E-10	2.06E-09	5.04E-09	7.86E-09	9.57E-09	7.43E-09	5.72E-09	5.36E-09

1.15E-15	1.23E-15	1.15E-15	1.11E-15	1.15E-15	1.12E-15	1.11E-15	1.12E-15	1.05E-15	1.10E-15	1.14E-15	1.11E-15	1.04E-15	9.37E-16	8.77E-16
1.05E-15	1.09E-15	1.05E-15	1.03E-15	1.06E-15	1.02E-15	1.01E-15	1.01E-15	9.86E-16	1.04E-15	1.03E-15	1.03E-15	9.43E-16	8.43E-16	7.85E-16
9.65E-16	9.83E-16	9.31E-16	9.64E-16	9.77E-16	9.46E-16	9.47E-16	9.43E-16	9.46E-16	9.54E-16	9.20E-16	9.16E-16	8.46E-16	7.57E-16	6.96E-16
8.82E-16	8.96E-16	8.56E-16	8.84E-16	8.97E-16	8.69E-16	8.79E-16	8.77E-16	9.02E-16	8.58E-16	8.41E-16	8.31E-16	7.66E-16	7.01E-16	6.15E-16
8.05E-16	8.31E-16	7.90E-16	8.23E-16	8.03E-16	7.78E-16	7.82E-16	7.87E-16	8.16E-16	7.77E-16	7.52E-16	7.46E-16	7.03E-16	6.62E-16	5.49E-16
7.44E-16	7.78E-16	7.33E-16	7.81E-16	7.28E-16	7.12E-16	7.04E-16	7.03E-16	7.28E-16	7.15E-16	6.83E-16	6.62E-16	6.50E-16	6.27E-16	5.26E-16
6.80E-16	7.19E-16	6.87E-16	6.83E-16	6.72E-16	6.73E-16	6.58E-16	6.45E-16	6.71E-16	6.65E-16	6.05E-16	5.84E-16	5.70E-16	5.75E-16	4.95E-16
6.18E-16	6.33E-16	6.28E-16	6.19E-16	6.26E-16	6.42E-16	6.21E-16	5.82E-16	6.25E-16	6.25E-16	5.37E-16	5.12E-16	4.89E-16	5.09E-16	4.45E-16
5.69E-16	5.63E-16	5.57E-16	5.56E-16	5.77E-16	6.15E-16	5.68E-16	5.29E-16	5.49E-16	5.61E-16	4.87E-16	4.69E-16	4.17E-16	4.39E-16	3.95E-16
5.15E-16	5.13E-16	5.04E-16	5.09E-16	5.19E-16	5.57E-16	4.84E-16	4.79E-16	4.52E-16	4.79E-16	4.44E-16	4.27E-16	3.54E-16	3.69E-16	3.24E-16
4.51E-16	4.66E-16	4.49E-16	4.48E-16	4.59E-16	4.78E-16	4.23E-16	4.31E-16	3.88E-16	4.06E-16	3.82E-16	3.74E-16	3.08E-16	3.20E-16	2.67E-16
3.99E-16	4.09E-16	3.91E-16	3.89E-16	3.93E-16	4.20E-16	3.65E-16	3.90E-16	3.43E-16	3.49E-16	3.31E-16	3.33E-16	2.76E-16	2.92E-16	2.32E-16
3.55E-16	3.57E-16	3.43E-16	3.56E-16	3.47E-16	3.78E-16	3.28E-16	3.38E-16	3.26E-16	3.08E-16	2.96E-16	2.95E-16	2.51E-16	2.34E-16	1.99E-16
2.98E-16	2.97E-16	3.06E-16	3.24E-16	3.23E-16	3.35E-16	3.08E-16	2.83E-16	2.84E-16	2.81E-16	2.62E-16	2.49E-16	2.33E-16	2.05E-16	1.88E-16
2.51E-16	2.57E-16	2.75E-16	2.94E-16	2.82E-16	2.93E-16	2.86E-16	2.59E-16	2.61E-16	2.58E-16	2.36E-16	2.20E-16	2.05E-16	1.81E-16	1.69E-16
2.15E-16	2.19E-16	2.44E-16	2.55E-16	2.45E-16	2.58E-16	2.59E-16	2.36E-16	2.18E-16	2.16E-16	1.97E-16	1.95E-16	1.80E-16	1.62E-16	1.60E-16
1.83E-16	1.98E-16	2.09E-16	2.17E-16	2.10E-16	2.16E-16	2.19E-16	2.02E-16	1.74E-16	1.73E-16	1.74E-16	1.80E-16	1.49E-16	1.40E-16	1.52E-16
1.61E-16	1.76E-16	1.81E-16	1.74E-16	1.70E-16	1.77E-16	1.62E-16	1.72E-16	1.47E-16	1.49E-16	1.59E-16	1.52E-16	1.25E-16	1.25E-16	1.69E-16
1.42E-16	1.49E-16	1.62E-16	1.48E-16	1.40E-16	1.37E-16	1.39E-16	1.47E-16	1.36E-16	1.29E-16	1.47E-16	1.33E-16	1.14E-16	1.31E-16	2.53E-16
1.31E-16	1.22E-16	1.44E-16	1.29E-16	1.23E-16	1.24E-16	1.23E-16	1.29E-16	1.35E-16	1.14E-16	1.28E-16	1.18E-16	1.00E-16	1.72E-16	5.27E-16
1.19E-16	9.95E-17	1.26E-16	1.14E-16	1.08E-16	1.12E-16	1.08E-16	1.15E-16	1.16E-16	1.01E-16	9.74E-17	9.02E-17	8.01E-17	2.51E-16	1.09E-15
1.04E-16	8.72E-17	1.13E-16	1.12E-16	1.04E-16	1.07E-16	1.02E-16	9.99E-17	9.23E-17	7.51E-17	7.34E-17	6.32E-17	6.63E-17	2.56E-16	8.24E-16
8.46E-17	7.38E-17	9.26E-17	9.31E-17	9.91E-17	9.42E-17	8.29E-17	7.84E-17	7.78E-17	5.99E-17	6.09E-17	4.44E-17	5.70E-17	5.76E-17	9.65E-17
6.50E-17	6.73E-17	7.94E-17	6.99E-17	8.27E-17	7.64E-17	6.76E-17	5.88E-17	6.90E-17	5.11E-17	5.27E-17	3.47E-17	4.86E-17	4.85E-17	2.25E-17
5.43E-17	6.26E-17	6.08E-17	5.77E-17	6.48E-17	5.84E-17	5.87E-17	4.77E-17	6.10E-17	3.80E-17	4.03E-17	2.56E-17	3.01E-17	3.31E-17	1.77E-17
4.42E-17	5.17E-17	4.60E-17	4.67E-17	4.70E-17	4.25E-17	4.58E-17	3.61E-17	4.43E-17	2.96E-17	2.67E-17	1.91E-17	2.00E-17	1.79E-17	7.23E-18
3.32E-17	3.31E-17	3.09E-17	3.60E-17	3.31E-17	2.97E-17	3.38E-17	3.00E-17	3.50E-17	2.19E-17	1.80E-17	1.15E-17	1.08E-17	1.23E-17	3.19E-18
2.38E-17	2.07E-17	1.84E-17	2.51E-17	2.34E-17	2.11E-17	2.51E-17	2.64E-17	2.98E-17	1.58E-17	1.05E-17	7.79E-18	7.36E-18	6.56E-18	1.69E-18
1.54E-17	1.23E-17	8.62E-18	1.35E-17	1.71E-17	1.51E-17	1.81E-17	2.14E-17	2.01E-17	1.19E-17	6.00E-18	4.15E-18	3.55E-18	2.77E-18	7.27E-19
9.27E-18	6.46E-18	4.84E-18	9.33E-18	6.56E-18	6.71E-18	9.51E-18	1.04E-17	1.03E-17	5.53E-18	2.04E-18	2.21E-18	1.58E-18	3.02E-18	7.74E-19
4.70E-18	3.68E-18	4.34E-18	5.33E-18	2.80E-18	2.12E-18	3.92E-18	3.25E-18	4.26E-18	1.82E-18	6.18E-19	1.21E-18	7.84E-19	2.93E-18	7.91E-19

3.21E-18	1.61E-18	3.98E-18	2.11E-18	2.39E-18	1.62E-18	1.11E-18	1.24E-18	1.53E-18	3.91E-19	1.67E-19	1.54E-19	1.82E-19	2.90E-18	9.82E-19
8.82E-19	2.80E-19	4.51E-18	8.67E-19	2.75E-18	1.27E-18	1.50E-19	1.45E-19	6.36E-19	1.23E-19	1.32E-19	2.99E-20	3.14E-20	1.50E-18	5.62E-19
3.81E-21	7.67E-20	3.12E-18	4.50E-19	2.49E-18	6.20E-19	4.60E-20	9.61E-25	1.38E-20	2.68E-19	1.12E-18	7.00E-19	1.01E-18	5.08E-20	1.24E-19
1.41E-18	1.23E-22	2.98E-18	2.28E-19	1.10E-18	1.66E-19	9.95E-19	6.04E-19	4.28E-19	5.86E-19	2.46E-18	2.68E-18	3.36E-18	1.87E-18	2.18E-18
4.33E-18	1.78E-19	1.35E-18	2.95E-21	3.94E-19	1.13E-18	3.46E-18	3.02E-18	1.89E-18	2.02E-18	4.64E-18	4.39E-18	3.74E-18	3.71E-18	6.82E-18
8.02E-18	1.35E-18	1.71E-19	1.72E-18	2.83E-18	5.05E-18	4.25E-18	3.99E-18	5.85E-18	4.44E-18	5.25E-18	5.96E-18	4.13E-18	6.47E-18	1.14E-17
1.29E-17	4.83E-18	4.57E-18	7.05E-18	7.90E-18	9.31E-18	5.13E-18	6.40E-18	1.08E-17	6.77E-18	7.76E-18	6.37E-18	5.34E-18	1.37E-17	1.54E-17
1.60E-17	1.41E-17	1.02E-17	9.34E-18	1.37E-17	1.46E-17	5.10E-18	9.39E-18	1.56E-17	1.45E-17	1.18E-17	1.05E-17	9.33E-18	2.01E-17	1.96E-17
1.93E-17	2.04E-17	1.30E-17	8.75E-18	1.62E-17	1.59E-17	5.63E-18	1.30E-17	2.21E-17	1.79E-17	1.77E-17	1.86E-17	1.40E-17	2.82E-17	2.72E-17
2.22E-17	2.55E-17	1.45E-17	8.14E-18	1.59E-17	1.81E-17	1.27E-17	1.77E-17	2.53E-17	1.95E-17	2.38E-17	2.64E-17	2.44E-17	3.58E-17	3.45E-17
2.57E-17	2.92E-17	1.83E-17	9.70E-18	1.92E-17	2.08E-17	2.10E-17	2.10E-17	2.54E-17	2.18E-17	2.94E-17	3.18E-17	3.60E-17	4.68E-17	3.94E-17
2.86E-17	2.90E-17	2.57E-17	1.77E-17	2.52E-17	2.83E-17	2.52E-17	2.55E-17	2.84E-17	2.48E-17	2.91E-17	3.24E-17	3.85E-17	5.51E-17	4.51E-17
3.47E-17	3.26E-17	3.40E-17	2.60E-17	3.05E-17	3.37E-17	2.88E-17	2.82E-17	2.97E-17	2.74E-17	3.08E-17	2.96E-17	3.95E-17	5.61E-17	4.68E-17
4.61E-17	4.25E-17	4.61E-17	3.35E-17	3.81E-17	3.64E-17	3.21E-17	2.52E-17	2.88E-17	3.15E-17	3.43E-17	2.93E-17	3.50E-17	5.14E-17	4.79E-17
5.38E-17	4.33E-17	4.93E-17	3.75E-17	4.27E-17	3.65E-17	3.46E-17	2.86E-17	3.18E-17	3.66E-17	3.91E-17	3.21E-17	3.11E-17	4.65E-17	4.84E-17
5.94E-17	4.68E-17	4.48E-17	3.79E-17	4.06E-17	3.89E-17	4.08E-17	3.34E-17	3.72E-17	4.21E-17	4.08E-17	3.66E-17	3.26E-17	4.28E-17	4.72E-17
6.51E-17	5.15E-17	4.37E-17	4.01E-17	3.93E-17	4.03E-17	4.13E-17	4.54E-17	4.44E-17	5.20E-17	4.90E-17	3.96E-17	4.22E-17	4.69E-17	4.76E-17
6.92E-17	5.71E-17	4.30E-17	3.78E-17	3.30E-17	3.69E-17	4.41E-17	4.17E-17	4.48E-17	5.48E-17	5.86E-17	4.25E-17	5.04E-17	4.89E-17	4.57E-17
7.86E-17	5.65E-17	4.87E-17	3.75E-17	3.08E-17	3.86E-17	5.23E-17	4.03E-17	5.00E-17	5.23E-17	5.93E-17	4.12E-17	4.72E-17	5.60E-17	4.76E-17
8.62E-17	5.54E-17	4.90E-17	3.37E-17	3.00E-17	4.49E-17	5.77E-17	4.72E-17	5.51E-17	5.26E-17	5.39E-17	4.74E-17	4.81E-17	6.41E-17	5.82E-17
8.37E-17	5.81E-17	4.64E-17	4.27E-17	4.03E-17	5.36E-17	6.22E-17	6.16E-17	6.60E-17	5.13E-17	5.39E-17	4.88E-17	5.17E-17	6.83E-17	6.14E-17
8.04E-17	7.22E-17	6.44E-17	5.53E-17	6.35E-17	6.74E-17	7.80E-17	7.49E-17	7.22E-17	5.21E-17	5.56E-17	5.50E-17	5.93E-17	7.39E-17	6.64E-17
6.80E-17	6.70E-17	7.90E-17	6.63E-17	7.55E-17	7.47E-17	8.55E-17	8.70E-17	7.42E-17	5.35E-17	5.21E-17	6.13E-17	6.81E-17	6.93E-17	6.37E-17
6.24E-17	5.65E-17	9.07E-17	8.20E-17	8.21E-17	8.51E-17	9.21E-17	8.25E-17	7.56E-17	5.81E-17	4.87E-17	7.24E-17	7.14E-17	6.27E-17	5.84E-17
6.55E-17	5.38E-17	9.51E-17	9.01E-17	8.25E-17	8.91E-17	9.33E-17	8.01E-17	7.71E-17	5.93E-17	5.09E-17	7.20E-17	6.85E-17	5.07E-17	4.50E-17
5.34E-17	5.84E-17	9.07E-17	8.67E-17	7.12E-17	9.42E-17	8.69E-17	7.96E-17	7.75E-17	6.33E-17	6.29E-17	7.11E-17	5.70E-17	4.21E-17	4.56E-17
4.07E-17	5.37E-17	8.00E-17	7.79E-17	6.52E-17	9.51E-17	8.24E-17	7.78E-17	8.42E-17	8.04E-17	7.38E-17	6.61E-17	5.57E-17	4.33E-17	4.87E-17
3.25E-17	5.84E-17	7.68E-17	7.36E-17	7.58E-17	9.34E-17	7.40E-17	7.01E-17	8.74E-17	8.52E-17	8.64E-17	6.99E-17	5.88E-17	5.15E-17	5.75E-17
2.46E-17	6.50E-17	8.05E-17	7.82E-17	8.28E-17	8.69E-17	6.69E-17	6.05E-17	7.85E-17	7.90E-17	8.38E-17	7.50E-17	6.95E-17	5.39E-17	5.74E-17
2.28E-17	5.90E-17	7.15E-17	7.58E-17	7.96E-17	8.04E-17	6.72E-17	5.34E-17	6.73E-17	7.79E-17	7.28E-17	6.96E-17	6.21E-17	4.16E-17	4.54E-17
2.37E-17	5.09E-17	5.76E-17	5.71E-17	6.09E-17	6.68E-17	5.58E-17	4.40E-17	5.59E-17	7.43E-17	6.46E-17	6.29E-17	4.90E-17	3.63E-17	3.18E-17
2.87E-17	5.24E-17	5.09E-17	4.76E-17	5.02E-17	5.45E-17	5.68E-17	4.31E-17	4.12E-17	6.71E-17	5.96E-17	4.84E-17	3.99E-17	2.70E-17	2.10E-17
2.99E-17	5.24E-17	5.28E-17	5.06E-17	4.87E-17	4.49E-17	5.11E-17	4.00E-17	3.42E-17	5.71E-17	5.02E-17	3.76E-17	3.01E-17	1.95E-17	1.34E-17
2.37E-17	3.55E-17	4.70E-17	4.83E-17	4.55E-17	3.54E-17	4.23E-17	4.15E-17	2.80E-17	4.30E-17	4.50E-17	3.47E-17	2.49E-17	1.71E-17	1.95E-17
1.04E-17	1.79E-17	3.08E-17	4.17E-17	3.72E-17	3.21E-17	3.53E-17	3.55E-17	2.05E-17	2.88E-17	4.52E-17	2.97E-17	1.30E-17	1.63E-17	2.80E-17
4.31E-18	1.06E-17	1.54E-17	2.97E-17	3.48E-17	2.64E-17	3.11E-17	2.76E-17	1.70E-17	2.01E-17	4.18E-17	3.17E-17	8.91E-18	1.44E-17	3.33E-17
7.35E-19	2.64E-18	6.64E-18	2.58E-17	3.85E-17	2.25E-17	2.12E-17	2.00E-17	1.73E-17	1.47E-17	3.95E-17	3.50E-17	1.59E-17	1.10E-17	2.85E-17
8.68E-19	8.68E-20	2.06E-18	1.76E-17	3.73E-17	2.08E-17	2.24E-17	2.31E-17	1.98E-17	1.26E-17	3.21E-17	4.31E-17	2.76E-17	1.65E-17	2.76E-17
4.90E-19	1.13E-18	1.28E-18	1.28E-17	3.63E-17	2.14E-17	2.85E-17	4.05E-17	2.55E-17	1.27E-17	2.28E-17	4.56E-17	3.16E-17	2.59E-17	3.68E-17
1.82E-19	3.32E-19	3.79E-18	1.63E-17	3.38E-17	2.63E-17	3.84E-17	6.47E-17	4.83E-17	2.54E-17	2.27E-17	4.21E-17	3.13E-17	3.36E-17	4.51E-17
2.77E-19	3.14E-21	6.33E-18	2.25E-17	3.40E-17	2.76E-17	4.53E-17	8.32E-17	6.58E-17	4.42E-17	3.23E-17	3.82E-17	3.94E-17	4.16E-17	4.48E-17
7.31E-18	2.58E-18	1.02E-17	2.81E-17	3.64E-17	2.88E-17	6.29E-17	9.02E-17	7.73E-17	5.93E-17	4.86E-17	4.67E-17	5.84E-17	4.95E-17	4.79E-17
2.05E-17	1.73E-17	1.45E-17	3.69E-17	4.46E-17	4.12E-17	8.80E-17	1.03E-16	1.00E-16	7.00E-17	6.13E-17	6.79E-17	7.37E-17	5.49E-17	6.01E-17
2.53E-17	3.24E-17	2.19E-17	4.60E-17	5.68E-17	5.88E-17	1.03E-16	1.10E-16	1.10E-16	7.86E-17	7.96E-17	8.04E-17	7.93E-17	6.29E-17	6.41E-17
3.24E-17	4.34E-17	3.75E-17	4.92E-17	6.90E-17	7.71E-17	1.05E-16	1.05E-16	1.03E-16	8.00E-17	8.16E-17	7.81E-17	7.45E-17	6.59E-17	6.28E-17
4.03E-17	5.89E-17	5.56E-17	6.13E-17	7.91E-17	8.41E-17	1.07E-16	9.79E-17	9.29E-17	7.86E-17	7.14E-17	6.72E-17	6.36E-17	5.99E-17	5.36E-17
4.85E-17	6.77E-17	7.02E-17	7.66E-17	8.57E-17	8.53E-17	9.66E-17	9.47E-17	8.52E-17	8.45E-17	5.87E-17	5.74E-17	5.46E-17	5.11E-17	4.76E-17
7.03E-17	7.71E-17	8.47E-17	8.09E-17	7.83E-17	7.05E-17	8.17E-17	8.83E-17	7.88E-17	7.06E-17	4.45E-17	4.74E-17	5.15E-17	4.18E-17	4.96E-17
9.65E-17	9.37E-17	9.24E-17	8.08E-17	6.20E-17	5.31E-17	6.35E-17	7.30E-17	7.74E-17	6.13E-17	4.09E-17	5.19E-17	4.66E-17	4.56E-17	5.54E-17
1.19E-16	1.04E-16	9.50E-17	8.09E-17	5.86E-17	4.71E-17	4.64E-17	5.87E-17	7.11E-17	5.40E-17	3.67E-17	5.45E-17	5.07E-17	5.27E-17	6.24E-17
1.22E-16	1.12E-16	1.01E-16	8.59E-17	7.22E-17	5.73E-17	4.18E-17	5.81E-17	7.57E-17	5.52E-17	4.34E-17	6.50E-17	7.04E-17	6.24E-17	7.23E-17
1.14E-16	1.16E-16	1.05E-16	9.33E-17	8.20E-17	7.56E-17	6.32E-17	7.24E-17	8.08E-17	6.87E-17	6.01E-17	8.00E-17	8.77E-17	7.91E-17	8.16E-17
1.16E-16	1.09E-16	1.01E-16	9.58E-17	9.15E-17	8.17E-17	7.78E-17	7.05E-17	7.19E-17	7.42E-17	7.58E-17	9.13E-17	9.71E-17	8.73E-17	9.21E-17
1.09E-16	1.06E-16	1.04E-16	8.73E-17	8.65E-17	7.92E-17	7.22E-17	5.82E-17	6.01E-17	7.13E-17	7.23E-17	8.82E-17	8.87E-17	6.96E-17	8.01E-17
1.02E-16	1.01E-16	1.02E-16	8.08E-17	7.47E-17	6.49E-17	6.27E-17	6.27E-17	5.93E-17	6.93E-17	8.30E-17	8.32E-17	7.46E-17	5.03E-17	7.35E-17
9.65E-17	9.79E-17	9.91E-17	6.60E-17	5.81E-17	5.54E-17	5.40E-17	6.39E-17	6.23E-17	6.31E-17	8.53E-17	7.58E-17	5.44E-17	3.44E-17	5.90E-17
9.55E-17	8.57E-17	9.07E-17	6.38E-17	5.43E-17	5.04E-17	4.86E-17	5.09E-17	5.82E-17	6.23E-17	7.47E-17	6.71E-17	3.81E-17	1.97E-17	4.81E-17
8.16E-17	7.95E-17	9.33E-17	7.68E-17	6.26E-17	4.85E-17	3.85E-17	3.60E-17	5.20E-17	6.33E-17	7.30E-17	5.65E-17	3.38E-17	2.71E-17	4.87E-17
7.86E-17	7.80E-17	8.43E-17	7.30E-17	6.98E-17	5.46E-17	4.28E-17	3.31E-17	4.68E-17	6.22E-17	7.49E-17	6.25E-17	4.76E-17	4.80E-17	5.99E-17
6.92E-17	7.24E-17	6.70E-17	6.58E-17	6.51E-17	6.17E-17	4.88E-17	3.04E-17	4.06E-17	5.60E-17	7.28E-17	7.02E-17	6.09E-17	6.36E-17	7.01E-17
5.99E-17	6.55E-17	6.43E-17	5.69E-17	5.49E-17	6.25E-17	4.98E-17	3.07E-17	4.14E-17	5.05E-17	6.01E-17	6.20E-17	7.18E-17	7.32E-17	7.25E-17
5.31E-17	5.74E-17	6.43E-17	5.71E-17	4.20E-17	6.25E-17	5.36E-17	3.57E-17	3.37E-17	4.32E-17	4.68E-17	5.37E-17	7.28E-17	7.27E-17	6.94E-17
4.16E-17	5.58E-17	6.35E-17	5.89E-17	3.52E-17	6.30E-17	5.60E-17	4.40E-17	3.02E-17	4.06E-17	3.57E-17	4.63E-17	6.20E-17	6.84E-17	6.67E-17
3.14E-17	5.35E-17	5.68E-17	5.55E-17	2.98E-17	5.73E-17	4.77E-17	4.27E-17	4.22E-17	4.20E-17	2.90E-17	4.08E-17	5.51E-17	6.45E-17	5.90E-17
3.72E-17	5.38E-17	4.81E-17	4.77E-17	2.99E-17	4.55E-17	3.63E-17	3.88E-17	4.34E-17	4.42E-17	3.29E-17	5.38E-17	5.68E-17	5.17E-17	5.59E-17
3.25E-17	4.35E-17	3.70E-17	3.61E-17	2.54E-17	3.50E-17	2.66E-17	3.10E-17	3.53E-17	3.76E-17	2.83E-17	5.34E-17	5.35E-17	4.22E-17	5.16E-17
2.64E-17	3.03E-17	2.58E-17	2.59E-17	2.52E-17	2.77E-17	2.17E-17	2.61E-17	3.25E-17	3.43E-17	2.00E-17	3.96E-17	3.88E-17	4.46E-17	4.70E-17
1.92E-17	2.12E-17	2.28E-17	2.53E-17	2.51E-17	2.38E-17	2.25E-17	2.22E-17	3.04E-17	3.26E-17	1.65E-17	3.02E-17	2.74E-17	3.95E-17	4.57E-17
2.32E-17	2.57E-17	2.32E-17	2.41E-17	1.88E-17	2.49E-17	2.44E-17	1.85E-17	2.41E-17	2.96E-17	2.25E-17	2.80E-17	2.01E-17	3.87E-17	5.11E-17
2.44E-17	3.03E-17	1.88E-17	2.49E-17	1.71E-17	2.18E-17	2.20E-17	1.85E-17	1.43E-17	2.41E-17	2.90E-17	2.98E-17	1.88E-17	3.36E-17	5.39E-17
2.27E-17	2.91E-17	1.99E-17	2.92E-17	2.05E-17	1.98E-17	1.40E-17	2.41E-17	1.30E-17	2.49E-17	3.28E-17	3.38E-17	1.70E-17	2.54E-17	3.93E-17
2.05E-17	2.53E-17	2.22E-17	3.11E-17	2.20E-17	2.26E-17	1.58E-17	2.82E-17	9.67E-18	3.36E-17	3.41E-17	2.89E-17	9.12E-18	1.53E-17	2.92E-17
1.73E-17	2.06E-17	2.35E-17	3.06E-17	2.59E-17	2.53E-17	1.90E-17	2.68E-17	3.46E-17	3.76E-17	3.12E-17	2.25E-17	5.68E-18	9.81E-18	1.62E-17
9.49E-18	1.63E-17	2.32E-17	2.74E-17	2.45E-17	2.25E-17	2.05E-17	2.27E-17	2.98E-17	3.03E-17	3.17E-17	2.25E-17	6.86E-18	1.19E-17	1.21E-17
4.94E-18	1.18E-17	1.97E-17	2.00E-17	1.81E-17	1.64E-17	1.79E-17	2.14E-17	2.92E-17	2.90E-17	3.21E-17	2.45E-17	1.69E-17	1.94E-17	1.24E-17
3.32E-18	1.16E-17	1.90E-17	1.73E-17	1.12E-17	1.18E-17	1.13E-17	2.65E-17	3.13E-17	3.37E-17	3.11E-17	2.88E-17	2.74E-17	2.50E-17	1.74E-17
6.45E-18	1.28E-17	1.95E-17	1.80E-17	8.53E-18	9.83E-18	1.06E-17	3.58E-17	3.68E-17	3.79E-17	3.03E-17	3.21E-17	2.69E-17	2.52E-17	2.27E-17
9.35E-18	1.54E-17	2.09E-17	2.40E-17	1.55E-17	1.16E-17	1.14E-17	3.58E-17	3.41E-17	3.55E-17	2.86E-17	3.05E-17	1.95E-17	2.00E-17	2.16E-17
1.04E-17	2.00E-17	2.11E-17	2.82E-17	2.14E-17	1.81E-17	1.20E-17	2.27E-17	2.58E-17	3.10E-17	2.73E-17	2.41E-17	9.66E-18	1.34E-17	2.04E-17
1.27E-17	2.31E-17	1.86E-17	2.50E-17	2.19E-17	2.45E-17	9.95E-18	1.17E-17	2.22E-17	2.53E-17	2.28E-17	2.10E-17	8.56E-18	1.59E-17	2.41E-17
1.23E-17	2.15E-17	1.38E-17	2.00E-17	1.77E-17	2.31E-17	1.43E-17	1.22E-17	2.02E-17	2.28E-17	2.01E-17	1.37E-17	1.46E-17	2.73E-17	2.92E-17
1.03E-17	1.85E-17	1.24E-17	1.76E-17	1.43E-17	1.95E-17	1.88E-17	1.88E-17	1.97E-17	1.93E-17	2.15E-17	9.10E-18	1.19E-17	2.81E-17	3.34E-17
9.49E-18	1.98E-17	1.98E-17	1.97E-17	1.41E-17	1.76E-17	2.08E-17	2.37E-17	1.83E-17	1.54E-17	1.91E-17	5.25E-18	9.64E-18	2.39E-17	3.01E-17
1.33E-17	2.17E-17	1.92E-17	2.00E-17	2.07E-17	1.68E-17	2.18E-17	2.29E-17	1.55E-17	1.25E-17	1.71E-17	5.19E-18	6.15E-18	2.01E-17	2.24E-17
1.26E-17	1.88E-17	1.28E-17	1.48E-17	1.91E-17	1.89E-17	2.29E-17	2.19E-17	1.45E-17	1.39E-17	1.77E-17	5.33E-18	5.71E-18	1.38E-17	1.20E-17
1.19E-17	1.19E-17	1.07E-17	1.21E-17	1.62E-17	2.22E-17	2.46E-17	1.91E-17	1.34E-17	1.32E-17	1.61E-17	6.62E-18	6.95E-18	6.14E-18	2.32E-18

1.45E-17	8.61E-18	9.18E-18	1.32E-17	1.47E-17	2.10E-17	2.04E-17	1.44E-17	1.32E-17	1.26E-17	1.34E-17	9.74E-18	9.84E-18	3.34E-18	5.10E-20
2.24E-17	1.29E-17	1.02E-17	8.84E-18	1.21E-17	1.91E-17	1.44E-17	1.12E-17	1.43E-17	1.36E-17	1.18E-17	1.41E-17	8.59E-18	5.49E-18	5.13E-20
3.06E-17	1.93E-17	1.22E-17	7.37E-18	1.18E-17	1.65E-17	1.34E-17	9.58E-18	1.59E-17	1.22E-17	9.20E-18	1.49E-17	1.02E-17	6.60E-18	4.28E-19
2.85E-17	1.75E-17	1.43E-17	1.01E-17	1.37E-17	1.54E-17	1.07E-17	9.60E-18	1.59E-17	1.08E-17	7.04E-18	1.05E-17	9.00E-18	7.28E-18	6.77E-19
2.21E-17	1.66E-17	1.70E-17	1.65E-17	1.81E-17	1.23E-17	8.46E-18	9.36E-18	1.22E-17	8.43E-18	4.36E-18	6.95E-18	5.73E-18	5.95E-18	2.32E-18
2.22E-17	2.10E-17	1.81E-17	1.87E-17	1.75E-17	7.71E-18	6.90E-18	1.10E-17	9.55E-18	6.87E-18	4.89E-18	3.60E-18	2.29E-18	2.03E-18	3.54E-18
2.09E-17	2.18E-17	1.90E-17	1.53E-17	1.52E-17	8.73E-18	6.44E-18	8.86E-18	7.48E-18	4.14E-18	2.02E-18	1.04E-18	6.41E-19	2.94E-19	1.19E-18
1.96E-17	1.96E-17	1.96E-17	1.56E-17	1.23E-17	7.97E-18	5.82E-18	6.89E-18	5.17E-18	1.48E-18	1.97E-19	2.35E-19	7.72E-22	4.00E-19	1.12E-19
1.71E-17	1.70E-17	1.72E-17	1.33E-17	9.26E-18	6.75E-18	3.24E-18	3.46E-18	1.78E-18	2.00E-20	1.56E-18	4.43E-18	1.99E-18	8.88E-19	9.95E-19
1.52E-17	1.63E-17	1.09E-17	8.05E-18	5.59E-18	4.03E-18	1.55E-18	4.43E-19	3.92E-20	6.41E-18	1.57E-17	2.53E-17	1.48E-17	8.55E-18	7.25E-18
1.67E-17	1.24E-17	7.06E-18	4.84E-18	4.71E-18	5.18E-18	7.18E-19	4.76E-19	4.25E-18	2.54E-17	6.19E-17	9.15E-17	5.52E-17	3.28E-17	2.87E-17
1.46E-14	1.51E-14	1.50E-14	1.49E-14	1.49E-14	1.50E-14	1.46E-14	1.45E-14	1.45E-14	1.43E-14	1.38E-14	1.36E-14	1.24E-14	1.24E-14	1.37E-14

3.03E-08	2.96E-08	2.96E-08	2.99E-08	2.97E-08	2.92E-08	2.87E-08	2.86E-08	2.82E-08	2.83E-08	2.80E-08	2.74E-08	2.75E-08	2.73E-08	2.69E-08
2.90E-08	2.83E-08	2.84E-08	2.84E-08	2.79E-08	2.81E-08	2.71E-08	2.74E-08	2.71E-08	2.74E-08	2.68E-08	2.61E-08	2.60E-08	2.64E-08	2.58E-08
2.73E-08	2.69E-08	2.68E-08	2.65E-08	2.64E-08	2.65E-08	2.58E-08	2.59E-08	2.64E-08	2.69E-08	2.59E-08	2.49E-08	2.51E-08	2.54E-08	2.45E-08
2.61E-08	2.57E-08	2.57E-08	2.50E-08	2.47E-08	2.50E-08	2.46E-08	2.47E-08	2.55E-08	2.59E-08	2.50E-08	2.40E-08	2.45E-08	2.47E-08	2.37E-08
2.46E-08	2.49E-08	2.55E-08	2.44E-08	2.38E-08	2.37E-08	2.35E-08	2.35E-08	2.42E-08	2.43E-08	2.35E-08	2.30E-08	2.36E-08	2.39E-08	2.31E-08
2.39E-08	2.48E-08	2.52E-08	2.36E-08	2.25E-08	2.24E-08	2.23E-08	2.20E-08	2.22E-08	2.21E-08	2.17E-08	2.19E-08	2.25E-08	2.28E-08	2.18E-08
2.31E-08	2.42E-08	2.50E-08	2.22E-08	2.14E-08	2.14E-08	2.12E-08	2.11E-08	2.06E-08	2.04E-08	2.03E-08	2.12E-08	2.14E-08	2.13E-08	2.03E-08
2.16E-08	2.35E-08	2.53E-08	2.07E-08	1.98E-08	2.03E-08	2.02E-08	2.01E-08	1.96E-08	1.94E-08	1.93E-08	2.02E-08	2.03E-08	1.99E-08	1.90E-08
2.00E-08	2.07E-08	2.17E-08	1.93E-08	1.88E-08	1.89E-08	1.92E-08	1.89E-08	1.87E-08	1.92E-08	1.88E-08	1.94E-08	1.94E-08	1.88E-08	1.80E-08
1.83E-08	1.84E-08	1.88E-08	1.82E-08	1.79E-08	1.77E-08	1.81E-08	1.79E-08	1.82E-08	1.89E-08	1.80E-08	1.86E-08	1.81E-08	1.78E-08	1.72E-08
1.70E-08	1.72E-08	1.79E-08	1.77E-08	1.68E-08	1.67E-08	1.72E-08	1.70E-08	1.75E-08	1.84E-08	1.74E-08	1.73E-08	1.69E-08	1.71E-08	1.67E-08
1.61E-08	1.64E-08	1.74E-08	1.69E-08	1.58E-08	1.55E-08	1.61E-08	1.58E-08	1.62E-08	1.71E-08	1.66E-08	1.59E-08	1.59E-08	1.65E-08	1.60E-08
1.54E-08	1.55E-08	1.60E-08	1.55E-08	1.48E-08	1.46E-08	1.47E-08	1.46E-08	1.49E-08	1.59E-08	1.49E-08	1.50E-08	1.45E-08	1.50E-08	1.50E-08
1.48E-08	1.42E-08	1.41E-08	1.44E-08	1.40E-08	1.43E-08	1.35E-08	1.43E-08	1.41E-08	1.46E-08	1.41E-08	1.40E-08	1.37E-08	1.37E-08	1.41E-08
1.40E-08	1.30E-08	1.26E-08	1.37E-08	1.32E-08	1.38E-08	1.33E-08	1.41E-08	1.35E-08	1.34E-08	1.32E-08	1.29E-08	1.27E-08	1.27E-08	1.26E-08
1.32E-08	1.21E-08	1.18E-08	1.31E-08	1.21E-08	1.31E-08	1.34E-08	1.41E-08	1.34E-08	1.31E-08	1.24E-08	1.22E-08	1.19E-08	1.19E-08	1.16E-08
1.25E-08	1.12E-08	1.12E-08	1.17E-08	1.09E-08	1.26E-08	1.32E-08	1.37E-08	1.33E-08	1.27E-08	1.18E-08	1.16E-08	1.12E-08	1.07E-08	1.08E-08
1.15E-08	1.04E-08	1.04E-08	1.03E-08	9.90E-09	1.19E-08	1.27E-08	1.25E-08	1.26E-08	1.23E-08	1.14E-08	1.15E-08	1.10E-08	1.02E-08	1.03E-08
1.08E-08	9.51E-09	9.13E-09	9.20E-09	9.15E-09	1.05E-08	1.11E-08	1.17E-08	1.12E-08	1.10E-08	1.05E-08	1.12E-08	1.06E-08	9.81E-09	9.38E-09
9.99E-09	8.99E-09	8.51E-09	8.41E-09	8.78E-09	9.35E-09	9.84E-09	1.03E-08	1.01E-08	9.94E-09	9.66E-09	1.07E-08	9.76E-09	9.29E-09	8.95E-09
9.49E-09	8.80E-09	8.52E-09	8.56E-09	8.89E-09	8.96E-09	9.28E-09	9.75E-09	9.60E-09	9.09E-09	9.13E-09	9.36E-09	8.93E-09	9.00E-09	9.10E-09
8.81E-09	8.10E-09	8.33E-09	8.34E-09	8.79E-09	8.92E-09	9.25E-09	8.95E-09	8.82E-09	8.13E-09	8.33E-09	7.89E-09	8.58E-09	8.55E-09	8.18E-09
7.67E-09	7.22E-09	7.16E-09	7.28E-09	7.30E-09	8.47E-09	8.18E-09	8.31E-09	7.51E-09	7.13E-09	7.69E-09	7.44E-09	8.14E-09	7.45E-09	6.85E-09
6.58E-09	6.37E-09	5.98E-09	6.48E-09	7.01E-09	8.00E-09	7.50E-09	7.84E-09	6.71E-09	7.00E-09	7.17E-09	7.07E-09	6.87E-09	6.10E-09	5.86E-09
4.89E-09	5.82E-09	4.90E-09	5.96E-09	6.05E-09	7.23E-09	7.00E-09	7.35E-09	6.53E-09	6.24E-09	6.29E-09	6.09E-09	5.55E-09	4.88E-09	4.45E-09
3.49E-09	4.67E-09	3.56E-09	4.81E-09	5.78E-09	6.94E-09	7.03E-09	7.15E-09	6.42E-09	4.99E-09	4.98E-09	4.77E-09	4.68E-09	3.74E-09	3.38E-09
2.29E-09	3.52E-09	2.93E-09	4.05E-09	5.23E-09	6.62E-09	6.62E-09	6.10E-09	5.32E-09	3.76E-09	3.87E-09	3.49E-09	3.64E-09	2.81E-09	2.33E-09
1.71E-09	2.50E-09	2.37E-09	3.40E-09	4.53E-09	5.61E-09	4.58E-09	4.10E-09	4.07E-09	2.96E-09	3.39E-09	2.79E-09	2.93E-09	2.47E-09	1.41E-09
1.35E-09	2.10E-09	1.86E-09	2.39E-09	3.48E-09	3.77E-09	2.82E-09	3.25E-09	2.98E-09	2.02E-09	2.66E-09	1.75E-09	1.81E-09	2.19E-09	8.83E-10
1.23E-09	1.85E-09	1.06E-09	2.02E-09	2.45E-09	2.90E-09	2.39E-09	2.57E-09	2.22E-09	1.63E-09	1.81E-09	1.17E-09	1.43E-09	1.81E-09	6.24E-10
1.12E-09	1.70E-09	4.29E-10	8.32E-10	1.65E-09	2.10E-09	1.73E-09	1.14E-09	1.12E-09	1.06E-09	8.98E-10	3.50E-10	1.23E-09	1.82E-09	3.16E-10
1.50E-09	1.50E-09	1.58E-11	-1.84E-10	9.85E-10	7.19E-10	7.17E-10	-6.63E-11	4.54E-11	-6.41E-11	1.31E-10	-1.91E-10	8.94E-10	1.51E-09	1.97E-10
2.00E-09	1.27E-09	-3.48E-10	-7.45E-10	2.65E-10	-2.60E-10	-5.72E-10	-9.07E-10	-6.07E-10	-1.18E-09	-1.15E-10	-6.60E-10	3.98E-10	9.90E-10	2.07E-10
1.11E-09	7.62E-10	-1.09E-09	-1.35E-09	-5.84E-10	-1.09E-09	-1.17E-09	-1.16E-09	-1.04E-09	-1.38E-09	-3.01E-10	-1.08E-09	4.60E-10	2.91E-10	-3.70E-10
-3.35E-10	-4.66E-11	-1.89E-09	-1.99E-09	-1.25E-09	-1.81E-09	-1.82E-09	-1.19E-09	-1.31E-09	-1.59E-09	-1.06E-09	-1.73E-09	-1.32E-10	-6.53E-10	-6.04E-10
-1.47E-09	-1.34E-09	-2.05E-09	-2.10E-09	-1.51E-09	-2.38E-09	-2.12E-09	-1.88E-09	-1.87E-09	-2.01E-09	-2.03E-09	-1.76E-09	-8.38E-10	-1.34E-09	-1.52E-09
-1.94E-09	-1.63E-09	-2.09E-09	-2.19E-09	-2.01E-09	-3.02E-09	-2.45E-09	-2.57E-09	-2.36E-09	-2.38E-09	-2.32E-09	-2.07E-09	-1.36E-09	-1.61E-09	-2.27E-09
-2.72E-09	-2.88E-09	-2.45E-09	-3.46E-09	-3.47E-09	-3.80E-09	-2.98E-09	-2.89E-09	-3.12E-09	-2.64E-09	-2.56E-09	-2.39E-09	-1.52E-09	-2.02E-09	-2.68E-09
-3.94E-09	-4.19E-09	-3.93E-09	-4.55E-09	-4.44E-09	-4.58E-09	-3.58E-09	-3.26E-09	-3.74E-09	-2.75E-09	-2.42E-09	-2.11E-09	-1.89E-09	-2.44E-09	-3.18E-09
-4.83E-09	-5.09E-09	-5.32E-09	-5.04E-09	-5.09E-09	-5.33E-09	-3.94E-09	-3.59E-09	-4.29E-09	-3.24E-09	-2.13E-09	-1.75E-09	-2.22E-09	-2.52E-09	-3.53E-09
-5.28E-09	-5.30E-09	-5.99E-09	-5.44E-09	-5.47E-09	-5.64E-09	-4.26E-09	-4.46E-09	-5.07E-09	-4.02E-09	-2.27E-09	-1.66E-09	-1.96E-09	-2.81E-09	-3.67E-09
-5.96E-09	-5.70E-09	-6.27E-09	-5.64E-09	-5.65E-09	-5.41E-09	-5.00E-09	-4.93E-09	-5.23E-09	-4.42E-09	-3.07E-09	-1.93E-09	-2.40E-09	-3.81E-09	-4.38E-09
-6.43E-09	-6.08E-09	-6.33E-09	-6.19E-09	-5.68E-09	-5.72E-09	-5.82E-09	-5.60E-09	-5.09E-09	-4.82E-09	-3.87E-09	-2.45E-09	-3.47E-09	-3.99E-09	-4.99E-09
-6.57E-09	-6.33E-09	-6.69E-09	-6.90E-09	-5.59E-09	-5.54E-09	-5.95E-09	-6.09E-09	-4.68E-09	-4.84E-09	-3.77E-09	-2.92E-09	-4.16E-09	-4.21E-09	-4.67E-09
-6.23E-09	-6.72E-09	-6.95E-09	-6.81E-09	-5.62E-09	-6.13E-09	-5.63E-09	-6.01E-09	-4.63E-09	-4.91E-09	-4.04E-09	-3.70E-09	-4.86E-09	-4.56E-09	-4.06E-09
-6.12E-09	-6.92E-09	-6.87E-09	-6.87E-09	-5.94E-09	-6.48E-09	-5.63E-09	-5.44E-09	-5.60E-09	-5.62E-09	-4.94E-09	-4.88E-09	-5.55E-09	-4.63E-09	-4.08E-09
-6.43E-09	-7.02E-09	-6.95E-09	-7.05E-09	-6.45E-09	-6.59E-09	-6.59E-09	-5.64E-09	-5.84E-09	-6.03E-09	-5.75E-09	-5.89E-09	-5.58E-09	-4.14E-09	-3.35E-09
-6.83E-09	-6.91E-09	-7.03E-09	-7.14E-09	-6.67E-09	-6.90E-09	-7.07E-09	-5.97E-09	-5.99E-09	-5.86E-09	-6.35E-09	-6.29E-09	-5.43E-09	-3.32E-09	-2.03E-09
-6.88E-09	-7.23E-09	-7.07E-09	-7.18E-09	-7.09E-09	-7.26E-09	-7.15E-09	-6.04E-09	-6.36E-09	-5.70E-09	-6.25E-09	-6.32E-09	-5.23E-09	-2.87E-09	-1.64E-09
-6.90E-09	-6.90E-09	-7.05E-09	-7.25E-09	-7.92E-09	-7.66E-09	-7.13E-09	-5.73E-09	-6.61E-09	-6.21E-09	-6.25E-09	-6.21E-09	-5.58E-09	-2.85E-09	-1.39E-09
-6.72E-09	-7.09E-09	-7.29E-09	-7.10E-09	-8.23E-09	-7.67E-09	-6.85E-09	-5.64E-09	-6.48E-09	-6.31E-09	-6.10E-09	-6.02E-09	-6.05E-09	-4.77E-09	-1.47E-09
-6.73E-09	-7.28E-09	-7.66E-09	-7.16E-09	-7.67E-09	-6.97E-09	-6.30E-09	-5.75E-09	-6.51E-09	-6.20E-09	-5.95E-09	-6.19E-09	-6.41E-09	-5.52E-09	-2.40E-09
-7.26E-09	-7.32E-09	-7.65E-09	-6.77E-09	-6.95E-09	-6.84E-09	-6.15E-09	-5.57E-09	-6.17E-09	-5.63E-09	-5.90E-09	-6.05E-09	-6.88E-09	-6.06E-09	-3.45E-09
-7.13E-09	-7.06E-09	-7.45E-09	-6.25E-09	-6.63E-09	-6.76E-09	-6.31E-09	-5.27E-09	-5.70E-09	-4.92E-09	-5.53E-09	-5.80E-09	-6.93E-09	-6.52E-09	-4.95E-09
-6.08E-09	-6.12E-09	-6.68E-09	-5.83E-09	-6.37E-09	-5.96E-09	-6.00E-09	-5.34E-09	-4.90E-09	-4.09E-09	-4.83E-09	-5.43E-09	-6.56E-09	-6.52E-09	-5.23E-09
-4.72E-09	-4.52E-09	-5.38E-09	-4.89E-09	-5.58E-09	-4.48E-09	-4.82E-09	-5.50E-09	-5.22E-09	-4.76E-09	-4.93E-09	-5.03E-09	-5.81E-09	-6.22E-09	-5.16E-09
-3.89E-09	-3.93E-09	-4.52E-09	-4.70E-09	-4.49E-09	-3.22E-09	-3.66E-09	-5.66E-09	-5.58E-09	-5.51E-09	-4.99E-09	-4.55E-09	-4.73E-09	-5.33E-09	-5.03E-09
-5.37E-09	-4.41E-09	-4.29E-09	-4.92E-09	-4.06E-09	-2.86E-09	-2.71E-09	-5.08E-09	-5.69E-09	-5.67E-09	-4.77E-09	-3.53E-09	-3.80E-09	-4.98E-09	-4.98E-09
-6.01E-09	-5.35E-09	-4.58E-09	-5.12E-09	-4.40E-09	-2.68E-09	-2.70E-09	-5.30E-09	-5.99E-09	-5.62E-09	-4.33E-09	-2.94E-09	-3.80E-09	-5.04E-09	-5.25E-09
-5.55E-09	-5.31E-09	-5.39E-09	-5.18E-09	-4.71E-09	-3.30E-09	-4.03E-09	-5.48E-09	-5.60E-09	-5.63E-09	-4.38E-09	-3.74E-09	-4.73E-09	-4.90E-09	-5.16E-09
-4.72E-09	-4.10E-09	-5.41E-09	-5.82E-09	-5.79E-09	-4.93E-09	-4.94E-09	-5.68E-09	-5.29E-09	-5.69E-09	-5.22E-09	-4.73E-09	-5.02E-09	-4.16E-09	-4.00E-09
-3.77E-09	-3.30E-09	-4.74E-09	-6.13E-09	-6.49E-09	-6.46E-09	-6.14E-09	-6.14E-09	-5.21E-09	-5.68E-09	-6.00E-09	-4.98E-09	-4.64E-09	-3.44E-09	-2.70E-09
-2.86E-09	-3.79E-09	-5.00E-09	-6.72E-09	-7.07E-09	-7.36E-09	-7.18E-09	-7.03E-09	-5.97E-09	-5.98E-09	-5.77E-09	-5.19E-09	-4.06E-09	-3.56E-09	-2.74E-09
-3.07E-09	-4.93E-09	-6.14E-09	-7.18E-09	-7.52E-09	-7.70E-09	-7.64E-09	-7.53E-09	-6.49E-09	-6.10E-09	-5.77E-09	-5.10E-09	-3.64E-09	-4.29E-09	-3.24E-09
-3.84E-09	-5.91E-09	-6.84E-09	-7.61E-09	-7.95E-09	-7.83E-09	-7.65E-09	-7.54E-09	-6.46E-09	-6.20E-09	-5.98E-09	-5.00E-09	-3.22E-09	-4.36E-09	-2.52E-09
-5.44E-09	-6.89E-09	-7.11E-09	-7.82E-09	-8.21E-09	-7.84E-09	-7.56E-09	-7.52E-09	-6.24E-09	-6.46E-09	-5.89E-09	-5.45E-09	-4.37E-09	-4.12E-09	-1.41E-09
-6.61E-09	-7.43E-09	-6.84E-09	-7.84E-09	-8.07E-09	-7.20E-09	-7.64E-09	-7.82E-09	-6.98E-09	-6.87E-09	-6.33E-09	-5.61E-09	-4.92E-09	-4.38E-09	-1.49E-09
-6.78E-09	-7.29E-09	-7.06E-09	-7.69E-09	-7.82E-09	-6.98E-09	-7.66E-09	-7.92E-09	-7.41E-09	-7.02E-09	-6.38E-09	-4.90E-09	-5.04E-09	-4.48E-09	-3.98E-09
-6.79E-09	-7.41E-09	-7.54E-09	-7.45E-09	-7.55E-09	-7.46E-09	-7.57E-09	-7.07E-09	-6.96E-09	-6.66E-09	-6.02E-09	-4.88E-09	-5.16E-09	-4.34E-09	-5.29E-09
-6.57E-09	-7.46E-09	-7.72E-09	-7.61E-09	-7.63E-09	-7.71E-09	-7.41E-09	-6.22E-09	-6.12E-09	-5.63E-09	-5.83E-09	-5.75E-09	-5.39E-09	-4.72E-09	-5.30E-09
-6.01E-09	-7.14E-09	-7.46E-09	-8.15E-09	-8.16E-09	-8.26E-09	-7.74E-09	-5.58E-09	-5.08E-09	-5.07E-09	-6.07E-09	-6.46E-09	-6.35E-09	-5.72E-09	-6.07E-09
-4.63E-09	-6.59E-09	-7.68E-09	-8.53E-09	-7.92E-09	-8.52E-09	-7.60E-09	-5.59E-09	-4.50E-09	-5.27E-09	-5.77E-09	-6.52E-09	-6.70E-09	-6.56E-09	-6.49E-09
-3.72E-09	-6.84E-09	-8.07E-09	-8.12E-09	-7.08E-09	-8.06E-09	-7.29E-09	-6.13E-09	-5.21E-09	-5.72E-09	-5.18E-09	-6.41E-09	-6.57E-09	-6.53E-09	-6.31E-09
-5.32E-09	-7.18E-09	-8.13E-09	-7.46E-09	-6.02E-09	-7.52E-09	-6.83E-09	-7.04E-09	-6.70E-09	-6.39E-09	-5.49E-09	-6.81E-09	-6.74E-09	-6.58E-09	-6.39E-09
-6.27E-09	-7.33E-09	-7.39E-09	-7.00E-09	-6.33E-09	-7.37E-09	-7.04E-09	-7.47E-09	-7.32E-09	-7.53E-09	-6.87E-09	-8.00E-09	-6.82E-09	-6.42E-09	-5.75E-09
-5.96E-09	-6.99E-09	-6.67E-09	-6.11E-09	-6.70E-09	-7.17E-09	-7.31E-09	-7.25E-09	-7.24E-09	-7.68E-09	-7.33E-09	-8.07E-09	-6.15E-09	-5.32E-09	-5.00E-09
-5.91E-09	-6.37E-09	-5.99E-09	-5.48E-09	-5.92E-09	-6.85E-09	-8.15E-09	-7.29E-09	-6.82E-09	-7.34E-09	-6.49E-09	-7.46E-09	-5.10E-09	-4.08E-09	-4.50E-09
-6.55E-09	-5.70E-09	-5.12E-09	-6.02E-09	-6.16E-09	-7.13E-09	-8.69E-09	-7.83E-09	-7.78E-09	-6.94E-09	-5.44E-09	-6.44E-09	-4.74E-09	-3.52E-09	-4.21E-09

-6.20E-09	-5.39E-09	-5.02E-09	-6.12E-09	-6.88E-09	-8.20E-09	-8.61E-09	-7.80E-09	-7.97E-09	-7.05E-09	-5.69E-09	-5.84E-09	-5.34E-09	-4.48E-09	-4.82E-09
-6.07E-09	-5.57E-09	-5.22E-09	-6.09E-09	-6.90E-09	-8.34E-09	-8.21E-09	-7.89E-09	-7.51E-09	-6.90E-09	-5.77E-09	-5.99E-09	-5.97E-09	-5.62E-09	-5.30E-09
-6.35E-09	-5.92E-09	-4.81E-09	-6.13E-09	-6.74E-09	-8.34E-09	-8.05E-09	-7.75E-09	-6.90E-09	-7.05E-09	-6.34E-09	-6.47E-09	-6.21E-09	-6.02E-09	-5.28E-09
-7.42E-09	-6.70E-09	-4.36E-09	-6.06E-09	-5.74E-09	-7.43E-09	-7.40E-09	-6.73E-09	-6.17E-09	-6.84E-09	-6.49E-09	-6.17E-09	-6.40E-09	-6.02E-09	-5.55E-09
-8.04E-09	-6.46E-09	-4.98E-09	-6.31E-09	-6.12E-09	-6.82E-09	-6.73E-09	-5.53E-09	-5.89E-09	-6.18E-09	-5.99E-09	-5.42E-09	-6.12E-09	-5.45E-09	-5.42E-09
-8.21E-09	-6.33E-09	-5.70E-09	-6.18E-09	-6.95E-09	-6.66E-09	-6.18E-09	-5.07E-09	-6.02E-09	-5.17E-09	-5.07E-09	-4.96E-09	-5.45E-09	-4.81E-09	-4.91E-09
-7.91E-09	-6.52E-09	-7.59E-09	-7.07E-09	-7.92E-09	-6.88E-09	-5.72E-09	-4.94E-09	-5.41E-09	-4.40E-09	-4.67E-09	-4.86E-09	-4.79E-09	-4.39E-09	-3.86E-09
-8.33E-09	-7.47E-09	-8.66E-09	-7.73E-09	-8.24E-09	-7.20E-09	-5.75E-09	-4.32E-09	-4.69E-09	-5.15E-09	-4.88E-09	-4.75E-09	-4.47E-09	-3.87E-09	-2.87E-09
-8.51E-09	-8.01E-09	-8.99E-09	-7.65E-09	-8.26E-09	-7.95E-09	-6.81E-09	-4.82E-09	-4.90E-09	-5.89E-09	-5.55E-09	-4.52E-09	-4.77E-09	-3.71E-09	-1.73E-09
-8.71E-09	-7.92E-09	-8.23E-09	-7.08E-09	-7.85E-09	-8.27E-09	-6.80E-09	-5.53E-09	-5.64E-09	-6.28E-09	-5.43E-09	-4.56E-09	-4.59E-09	-3.43E-09	-1.07E-09
-8.07E-09	-7.31E-09	-7.37E-09	-6.56E-09	-7.06E-09	-7.92E-09	-6.45E-09	-6.46E-09	-5.98E-09	-6.53E-09	-5.52E-09	-4.59E-09	-4.31E-09	-3.30E-09	-1.11E-09
-7.51E-09	-6.75E-09	-6.59E-09	-5.44E-09	-6.23E-09	-7.49E-09	-6.99E-09	-7.02E-09	-5.62E-09	-6.29E-09	-6.23E-09	-5.68E-09	-4.23E-09	-2.99E-09	-7.26E-10
-6.87E-09	-5.88E-09	-5.31E-09	-5.00E-09	-6.15E-09	-6.82E-09	-6.72E-09	-7.16E-09	-5.17E-09	-5.77E-09	-6.11E-09	-5.60E-09	-3.16E-09	-3.16E-09	-8.89E-10
-6.10E-09	-4.83E-09	-4.39E-09	-5.25E-09	-6.60E-09	-6.18E-09	-6.12E-09	-6.36E-09	-4.97E-09	-5.28E-09	-5.70E-09	-5.15E-09	-3.00E-09	-3.38E-09	-2.28E-09
-6.28E-09	-5.16E-09	-4.08E-09	-5.44E-09	-6.39E-09	-5.10E-09	-5.55E-09	-5.73E-09	-4.56E-09	-4.39E-09	-4.95E-09	-5.17E-09	-4.27E-09	-4.38E-09	-3.62E-09
-7.19E-09	-6.01E-09	-4.62E-09	-5.47E-09	-6.41E-09	-4.79E-09	-5.38E-09	-5.86E-09	-4.85E-09	-3.59E-09	-4.18E-09	-4.93E-09	-4.76E-09	-5.20E-09	-4.77E-09
-7.28E-09	-5.88E-09	-5.01E-09	-4.53E-09	-5.56E-09	-4.90E-09	-5.79E-09	-6.29E-09	-5.71E-09	-4.05E-09	-3.87E-09	-4.43E-09	-4.52E-09	-5.10E-09	-5.27E-09
-7.11E-09	-4.87E-09	-4.86E-09	-4.45E-09	-4.87E-09	-5.03E-09	-5.95E-09	-5.61E-09	-5.22E-09	-4.57E-09	-3.58E-09	-3.95E-09	-3.72E-09	-5.25E-09	-5.29E-09
-6.74E-09	-5.28E-09	-5.13E-09	-4.74E-09	-5.22E-09	-4.97E-09	-5.18E-09	-4.43E-09	-3.95E-09	-3.79E-09	-4.03E-09	-4.07E-09	-4.21E-09	-5.60E-09	-4.83E-09
-6.43E-09	-6.29E-09	-5.07E-09	-4.39E-09	-4.86E-09	-4.57E-09	-4.10E-09	-3.69E-09	-2.94E-09	-3.75E-09	-4.34E-09	-3.77E-09	-4.63E-09	-5.54E-09	-5.09E-09
-6.30E-09	-6.77E-09	-5.42E-09	-4.28E-09	-3.64E-09	-3.37E-09	-2.42E-09	-2.43E-09	-2.37E-09	-3.78E-09	-3.71E-09	-2.87E-09	-4.21E-09	-5.37E-09	-4.88E-09
-6.52E-09	-6.89E-09	-6.37E-09	-4.91E-09	-3.02E-09	-2.10E-09	-2.29E-09	-2.46E-09	-1.85E-09	-3.75E-09	-4.11E-09	-2.71E-09	-3.66E-09	-5.14E-09	-4.57E-09
-6.74E-09	-6.63E-09	-7.05E-09	-5.75E-09	-3.34E-09	-2.16E-09	-2.79E-09	-2.97E-09	-2.43E-09	-3.48E-09	-4.71E-09	-3.24E-09	-3.75E-09	-5.07E-09	-4.33E-09
-6.55E-09	-6.31E-09	-6.77E-09	-5.96E-09	-2.91E-09	-2.21E-09	-3.21E-09	-3.81E-09	-2.48E-09	-3.00E-09	-4.58E-09	-3.16E-09	-4.25E-09	-5.05E-09	-4.61E-09
-5.59E-09	-5.91E-09	-5.72E-09	-5.27E-09	-3.05E-09	-2.96E-09	-4.36E-09	-3.89E-09	-3.11E-09	-3.39E-09	-3.88E-09	-3.88E-09	-4.85E-09	-4.04E-09	-4.38E-09
-4.67E-09	-5.11E-09	-4.54E-09	-4.89E-09	-4.90E-09	-4.60E-09	-5.73E-09	-4.05E-09	-3.27E-09	-3.54E-09	-3.20E-09	-4.81E-09	-4.45E-09	-3.37E-09	-3.89E-09
-4.63E-09	-5.00E-09	-4.60E-09	-5.03E-09	-5.36E-09	-6.15E-09	-6.11E-09	-3.99E-09	-3.93E-09	-2.79E-09	-2.80E-09	-4.71E-09	-4.68E-09	-4.23E-09	-4.48E-09
-4.82E-09	-5.41E-09	-4.71E-09	-4.44E-09	-4.79E-09	-6.14E-09	-5.57E-09	-3.53E-09	-2.86E-09	-2.96E-09	-2.97E-09	-4.92E-09	-4.95E-09	-5.10E-09	-4.84E-09
-4.52E-09	-4.41E-09	-3.68E-09	-4.15E-09	-4.22E-09	-5.59E-09	-4.47E-09	-3.72E-09	-2.46E-09	-3.35E-09	-3.99E-09	-5.39E-09	-5.21E-09	-4.84E-09	-4.91E-09
-4.46E-09	-3.65E-09	-3.98E-09	-5.21E-09	-4.80E-09	-4.59E-09	-2.86E-09	-2.93E-09	-2.21E-09	-4.31E-09	-4.92E-09	-5.16E-09	-4.56E-09	-3.83E-09	-4.61E-09
-4.89E-09	-3.65E-09	-5.31E-09	-5.96E-09	-4.60E-09	-3.82E-09	-1.27E-09	-1.51E-09	-2.12E-09	-4.21E-09	-5.05E-09	-5.18E-09	-4.10E-09	-3.23E-09	-4.33E-09
-4.95E-09	-4.89E-09	-5.56E-09	-5.85E-09	-3.99E-09	-3.69E-09	-1.64E-09	-1.64E-09	-1.71E-09	-4.41E-09	-5.01E-09	-5.02E-09	-4.39E-09	-3.72E-09	-4.54E-09
-5.26E-09	-5.64E-09	-5.67E-09	-5.47E-09	-3.26E-09	-4.07E-09	-3.39E-09	-3.57E-09	-3.18E-09	-4.95E-09	-4.85E-09	-4.65E-09	-4.83E-09	-4.68E-09	-4.41E-09
-5.56E-09	-5.84E-09	-5.57E-09	-5.25E-09	-2.88E-09	-4.16E-09	-4.24E-09	-4.51E-09	-4.50E-09	-5.02E-09	-4.21E-09	-4.98E-09	-4.62E-09	-4.74E-09	-4.61E-09
-5.71E-09	-5.29E-09	-5.94E-09	-5.79E-09	-3.73E-09	-4.92E-09	-4.90E-09	-4.89E-09	-4.62E-09	-4.97E-09	-3.90E-09	-5.26E-09	-4.67E-09	-4.93E-09	-4.65E-09
-4.96E-09	-4.89E-09	-5.94E-09	-5.82E-09	-4.24E-09	-4.97E-09	-4.62E-09	-4.80E-09	-4.56E-09	-4.96E-09	-4.06E-09	-5.29E-09	-4.46E-09	-4.98E-09	-4.90E-09
-3.82E-09	-4.24E-09	-4.83E-09	-5.35E-09	-4.25E-09	-4.44E-09	-4.22E-09	-4.37E-09	-4.67E-09	-4.89E-09	-4.52E-09	-5.39E-09	-4.72E-09	-4.58E-09	-3.77E-09
-2.45E-09	-2.89E-09	-3.47E-09	-4.60E-09	-4.05E-09	-3.51E-09	-3.82E-09	-4.18E-09	-4.34E-09	-4.86E-09	-5.25E-09	-4.92E-09	-4.20E-09	-3.51E-09	-2.89E-09
-5.95E-10	-1.53E-09	-2.39E-09	-3.92E-09	-3.35E-09	-1.95E-09	-3.03E-09	-4.11E-09	-4.08E-09	-4.90E-09	-5.35E-09	-4.34E-09	-3.44E-09	-2.62E-09	-1.94E-09
2.53E-10	-8.99E-10	-2.13E-09	-3.44E-09	-3.00E-09	-1.67E-09	-2.74E-09	-4.57E-09	-3.52E-09	-4.61E-09	-5.54E-09	-4.17E-09	-2.88E-09	-2.80E-09	-2.36E-09
-1.12E-10	-1.28E-09	-2.11E-09	-2.80E-09	-3.13E-09	-2.67E-09	-2.85E-09	-4.16E-09	-2.84E-09	-3.71E-09	-4.61E-09	-3.57E-09	-2.64E-09	-3.49E-09	-3.24E-09
-4.82E-10	-1.20E-09	-1.95E-09	-2.31E-09	-3.42E-09	-3.19E-09	-3.29E-09	-3.50E-09	-2.28E-09	-2.54E-09	-3.23E-09	-1.86E-09	-2.02E-09	-3.05E-09	-3.23E-09
-6.71E-10	-8.98E-10	-1.77E-09	-2.05E-09	-3.12E-09	-3.11E-09	-3.26E-09	-3.14E-09	-2.08E-09	-1.31E-09	-1.43E-09	3.37E-10	-1.06E-09	-2.03E-09	-2.38E-09
-2.64E-10	-6.64E-10	-1.81E-09	-1.63E-09	-2.33E-09	-3.02E-09	-3.14E-09	-2.80E-09	-1.95E-09	-2.91E-10	-3.93E-10	1.70E-09	-6.69E-10	-1.82E-09	-2.30E-09
-7.91E-10	-1.42E-09	-1.43E-09	-1.05E-09	-1.30E-09	-2.18E-09	-3.02E-09	-2.89E-09	-2.66E-09	-8.16E-10	-3.94E-10	2.05E-09	6.02E-10	-1.51E-09	-2.27E-09
-5.75E-10	-1.03E-09	-8.15E-10	-5.61E-11	-1.27E-10	-1.63E-09	-2.85E-09	-3.32E-09	-3.15E-09	-1.96E-09	-1.79E-10	2.52E-09	1.48E-09	-8.29E-10	-1.78E-09
-3.67E-10	3.68E-10	5.33E-10	1.48E-09	6.66E-10	-2.17E-09	-2.65E-09	-3.37E-09	-3.24E-09	-2.14E-09	-1.79E-10	1.78E-09	6.70E-10	-3.94E-10	-1.58E-09
1.04E-09	1.64E-09	3.00E-09	2.74E-09	1.37E-09	-1.69E-09	-2.08E-09	-2.77E-09	-2.83E-09	-2.13E-09	-6.07E-10	3.73E-10	-8.26E-10	-6.52E-11	-1.45E-09
3.07E-09	3.55E-09	4.72E-09	3.00E-09	1.68E-09	-3.68E-10	-9.90E-10	-1.43E-09	-2.06E-09	-1.79E-09	-3.79E-10	1.97E-10	-1.42E-09	-1.35E-09	-1.32E-09
4.13E-09	5.08E-09	5.44E-09	4.82E-09	3.05E-09	1.54E-09	4.66E-10	4.11E-10	-9.53E-10	-7.67E-10	2.97E-10	1.14E-09	-1.38E-09	-2.59E-09	-9.56E-10

9.17E-16	8.75E-16	8.78E-16	8.96E-16	8.83E-16	8.54E-16	8.26E-16	8.19E-16	7.98E-16	8.01E-16	7.84E-16	7.51E-16	7.59E-16	7.43E-16	7.21E-16
8.40E-16	8.03E-16	8.08E-16	8.04E-16	7.80E-16	7.87E-16	7.37E-16	7.51E-16	7.36E-16	7.49E-16	7.18E-16	6.81E-16	6.74E-16	6.97E-16	6.64E-16
7.44E-16	7.26E-16	7.18E-16	7.00E-16	6.95E-16	7.01E-16	6.65E-16	6.73E-16	6.96E-16	7.23E-16	6.72E-16	6.21E-16	6.29E-16	6.46E-16	6.02E-16
6.82E-16	6.60E-16	6.60E-16	6.26E-16	6.11E-16	6.23E-16	6.06E-16	6.09E-16	6.48E-16	6.73E-16	6.27E-16	5.76E-16	5.99E-16	6.09E-16	5.61E-16
6.03E-16	6.20E-16	6.48E-16	5.94E-16	5.65E-16	5.60E-16	5.52E-16	5.52E-16	5.84E-16	5.92E-16	5.51E-16	5.31E-16	5.55E-16	5.71E-16	5.31E-16
5.70E-16	6.16E-16	6.37E-16	5.57E-16	5.06E-16	5.04E-16	4.98E-16	4.85E-16	4.94E-16	4.88E-16	4.71E-16	4.82E-16	5.04E-16	5.21E-16	4.74E-16
5.33E-16	5.85E-16	6.25E-16	4.92E-16	4.56E-16	4.59E-16	4.49E-16	4.45E-16	4.26E-16	4.18E-16	4.11E-16	4.49E-16	4.57E-16	4.55E-16	4.12E-16
4.68E-16	5.54E-16	6.40E-16	4.28E-16	3.93E-16	4.10E-16	4.07E-16	4.06E-16	3.83E-16	3.78E-16	3.72E-16	4.09E-16	4.11E-16	3.97E-16	3.63E-16
3.99E-16	4.27E-16	4.72E-16	3.74E-16	3.55E-16	3.58E-16	3.69E-16	3.56E-16	3.51E-16	3.69E-16	3.53E-16	3.77E-16	3.75E-16	3.55E-16	3.25E-16
3.37E-16	3.39E-16	3.52E-16	3.31E-16	3.20E-16	3.15E-16	3.28E-16	3.21E-16	3.31E-16	3.59E-16	3.25E-16	3.46E-16	3.28E-16	3.15E-16	2.96E-16
2.90E-16	2.97E-16	3.22E-16	3.15E-16	2.83E-16	2.79E-16	2.94E-16	2.90E-16	3.06E-16	3.37E-16	3.04E-16	2.99E-16	2.87E-16	2.92E-16	2.80E-16
2.59E-16	2.68E-16	3.02E-16	2.87E-16	2.50E-16	2.40E-16	2.58E-16	2.48E-16	2.64E-16	2.94E-16	2.75E-16	2.53E-16	2.53E-16	2.71E-16	2.57E-16
2.37E-16	2.40E-16	2.57E-16	2.41E-16	2.19E-16	2.13E-16	2.15E-16	2.12E-16	2.22E-16	2.53E-16	2.22E-16	2.24E-16	2.11E-16	2.25E-16	2.26E-16
2.20E-16	2.02E-16	1.97E-16	2.08E-16	1.97E-16	2.04E-16	1.82E-16	2.04E-16	1.99E-16	2.15E-16	1.98E-16	1.96E-16	1.88E-16	1.87E-16	1.99E-16
1.96E-16	1.69E-16	1.59E-16	1.89E-16	1.73E-16	1.91E-16	1.78E-16	1.99E-16	1.81E-16	1.80E-16	1.75E-16	1.67E-16	1.61E-16	1.62E-16	1.59E-16
1.73E-16	1.46E-16	1.40E-16	1.71E-16	1.47E-16	1.73E-16	1.80E-16	1.99E-16	1.79E-16	1.71E-16	1.55E-16	1.49E-16	1.41E-16	1.42E-16	1.35E-16
1.56E-16	1.26E-16	1.26E-16	1.38E-16	1.20E-16	1.58E-16	1.75E-16	1.87E-16	1.77E-16	1.62E-16	1.40E-16	1.35E-16	1.25E-16	1.14E-16	1.17E-16
1.32E-16	1.08E-16	1.08E-16	1.07E-16	9.81E-17	1.42E-16	1.61E-16	1.55E-16	1.59E-16	1.51E-16	1.30E-16	1.33E-16	1.20E-16	1.04E-16	1.06E-16
1.16E-16	9.04E-17	8.34E-17	8.47E-17	8.37E-17	1.10E-16	1.24E-16	1.37E-16	1.25E-16	1.21E-16	1.10E-16	1.25E-16	1.13E-16	9.63E-17	8.80E-17
9.97E-17	8.08E-17	7.24E-17	7.07E-17	7.71E-17	8.74E-17	9.68E-17	1.05E-16	1.03E-16	9.88E-17	9.33E-17	1.14E-16	9.52E-17	8.64E-17	8.01E-17
9.00E-17	7.74E-17	7.26E-17	7.32E-17	7.91E-17	8.03E-17	8.61E-17	9.50E-17	9.21E-17	8.26E-17	8.34E-17	8.75E-17	7.97E-17	8.10E-17	8.27E-17
7.77E-17	6.56E-17	6.95E-17	6.96E-17	7.73E-17	7.95E-17	8.55E-17	8.01E-17	7.78E-17	6.61E-17	6.93E-17	6.23E-17	7.37E-17	7.30E-17	6.69E-17
5.88E-17	5.21E-17	5.13E-17	5.31E-17	5.33E-17	7.17E-17	6.70E-17	6.90E-17	5.65E-17	5.09E-17	5.91E-17	5.53E-17	6.62E-17	5.55E-17	4.69E-17
4.33E-17	4.05E-17	3.57E-17	4.20E-17	4.91E-17	6.40E-17	5.63E-17	6.15E-17	4.51E-17	4.90E-17	5.15E-17	5.00E-17	4.72E-17	3.72E-17	3.43E-17
2.39E-17	3.38E-17	2.41E-17	3.56E-17	3.66E-17	5.22E-17	4.89E-17	5.40E-17	4.26E-17	3.89E-17	3.96E-17	3.70E-17	3.08E-17	2.38E-17	1.98E-17
1.22E-17	2.18E-17	1.26E-17	2.32E-17	3.34E-17	4.81E-17	4.95E-17	5.11E-17	4.12E-17	2.49E-17	2.48E-17	2.27E-17	2.19E-17	1.40E-17	1.14E-17
5.26E-18	1.24E-17	8.56E-18	1.64E-17	2.73E-17	4.38E-17	4.38E-17	3.72E-17	2.83E-17	1.42E-17	1.50E-17	1.22E-17	1.32E-17	7.91E-18	5.45E-18
2.94E-18	6.23E-18	5.63E-18	1.16E-17	2.05E-17	3.15E-17	2.09E-17	1.68E-17	1.66E-17	8.77E-18	1.15E-17	7.79E-18	8.57E-18	6.09E-18	1.99E-18
1.83E-18	4.43E-18	3.47E-18	5.73E-18	1.21E-17	1.42E-17	7.98E-18	1.05E-17	8.85E-18	4.07E-18	7.06E-18	3.06E-18	3.26E-18	4.80E-18	7.80E-19
1.50E-18	3.44E-18	1.12E-18	4.10E-18	5.98E-18	8.43E-18	5.72E-18	6.62E-18	4.94E-18	2.66E-18	3.28E-18	1.36E-18	2.03E-18	3.26E-18	3.89E-19
1.25E-18	2.90E-18	1.84E-19	6.92E-19	2.73E-18	4.41E-18	3.00E-18	1.31E-18	1.26E-18	1.13E-18	8.06E-19	1.23E-19	1.50E-18	3.33E-18	1.00E-19

2.25E-18	2.25E-18	2.51E-22	3.39E-20	9.70E-19	5.17E-19	5.14E-19	4.39E-21	2.06E-21	4.11E-21	1.73E-20	3.64E-20	8.00E-19	2.29E-18	3.86E-20
4.00E-18	1.60E-18	1.21E-19	5.55E-19	7.03E-20	6.75E-20	3.27E-19	8.22E-19	3.68E-19	1.38E-18	1.32E-20	4.36E-19	1.58E-19	9.80E-19	4.29E-20
1.24E-18	5.80E-19	1.20E-18	1.83E-18	3.41E-19	1.19E-18	1.37E-18	1.34E-18	1.07E-18	1.91E-18	9.06E-20	1.16E-18	2.11E-19	8.44E-20	1.37E-19
1.12E-19	2.17E-21	3.58E-18	3.95E-18	1.57E-18	3.26E-18	3.31E-18	1.42E-18	1.73E-18	2.54E-18	1.13E-18	2.98E-18	1.73E-20	4.27E-19	3.65E-19
2.16E-18	1.81E-18	4.20E-18	4.41E-18	2.28E-18	5.64E-18	4.48E-18	3.55E-18	3.49E-18	4.05E-18	4.12E-18	3.11E-18	7.03E-19	1.78E-18	2.31E-18
3.78E-18	2.64E-18	4.35E-18	4.79E-18	4.04E-18	9.13E-18	6.00E-18	6.59E-18	5.55E-18	5.67E-18	5.40E-18	4.29E-18	1.85E-18	2.60E-18	5.17E-18
7.39E-18	8.28E-18	6.00E-18	1.20E-17	1.20E-17	1.44E-17	8.90E-18	8.34E-18	9.72E-18	6.98E-18	6.54E-18	5.73E-18	2.32E-18	4.06E-18	7.21E-18
1.55E-17	1.76E-17	1.54E-17	2.07E-17	1.97E-17	2.10E-17	1.28E-17	1.06E-17	1.40E-17	7.54E-18	5.85E-18	4.46E-18	3.57E-18	5.96E-18	1.01E-17
2.33E-17	2.59E-17	2.83E-17	2.54E-17	2.59E-17	2.84E-17	1.55E-17	1.29E-17	1.84E-17	1.05E-17	4.52E-18	3.06E-18	4.94E-18	6.35E-18	1.25E-17
2.78E-17	2.81E-17	3.59E-17	2.96E-17	2.99E-17	3.18E-17	1.82E-17	1.99E-17	2.57E-17	1.62E-17	5.14E-18	2.74E-18	3.84E-18	7.87E-18	1.35E-17
3.55E-17	3.25E-17	3.93E-17	3.18E-17	3.19E-17	2.92E-17	2.50E-17	2.43E-17	2.73E-17	1.95E-17	9.41E-18	3.73E-18	5.78E-18	1.45E-17	1.92E-17
4.14E-17	3.70E-17	4.01E-17	3.84E-17	3.22E-17	3.27E-17	3.39E-17	3.14E-17	2.59E-17	2.33E-17	1.50E-17	6.02E-18	1.20E-17	1.59E-17	2.49E-17
4.32E-17	4.00E-17	4.47E-17	4.76E-17	3.12E-17	3.07E-17	3.54E-17	3.71E-17	2.19E-17	2.34E-17	1.42E-17	8.50E-18	1.73E-17	1.77E-17	2.18E-17
3.89E-17	4.52E-17	4.83E-17	4.64E-17	3.16E-17	3.76E-17	3.17E-17	3.62E-17	2.15E-17	2.41E-17	1.63E-17	1.37E-17	2.36E-17	2.08E-17	1.65E-17
3.75E-17	4.78E-17	4.71E-17	4.72E-17	3.53E-17	4.20E-17	3.17E-17	2.95E-17	3.14E-17	3.15E-17	2.44E-17	2.38E-17	3.08E-17	2.14E-17	1.67E-17
4.13E-17	4.93E-17	4.84E-17	4.97E-17	4.15E-17	4.35E-17	4.34E-17	3.18E-17	3.41E-17	3.64E-17	3.31E-17	3.47E-17	3.12E-17	1.71E-17	1.12E-17
4.67E-17	4.77E-17	4.94E-17	5.09E-17	4.45E-17	4.76E-17	4.99E-17	3.57E-17	3.58E-17	3.44E-17	4.04E-17	3.96E-17	2.94E-17	1.10E-17	4.10E-18
4.73E-17	5.23E-17	5.01E-17	5.15E-17	5.03E-17	5.27E-17	5.12E-17	3.64E-17	4.05E-17	3.25E-17	3.91E-17	3.99E-17	2.73E-17	8.26E-18	2.69E-18
4.77E-17	4.76E-17	4.97E-17	5.25E-17	6.28E-17	5.87E-17	5.09E-17	3.29E-17	4.37E-17	3.86E-17	3.91E-17	3.86E-17	3.11E-17	8.14E-18	1.93E-18
4.52E-17	5.03E-17	5.32E-17	5.04E-17	6.78E-17	5.88E-17	4.70E-17	3.18E-17	4.19E-17	3.99E-17	3.72E-17	3.62E-17	3.66E-17	2.28E-17	2.16E-18
4.53E-17	5.30E-17	5.87E-17	5.13E-17	5.89E-17	4.86E-17	3.97E-17	3.30E-17	4.23E-17	3.84E-17	3.54E-17	3.83E-17	4.11E-17	3.05E-17	5.75E-18
5.27E-17	5.35E-17	5.85E-17	4.59E-17	4.83E-17	4.68E-17	3.79E-17	3.11E-17	3.81E-17	3.17E-17	3.48E-17	3.66E-17	4.73E-17	3.67E-17	1.19E-17
5.08E-17	4.99E-17	5.56E-17	3.90E-17	4.40E-17	4.57E-17	3.98E-17	2.77E-17	3.25E-17	2.42E-17	3.05E-17	3.36E-17	4.80E-17	4.26E-17	2.45E-17
3.70E-17	3.74E-17	4.47E-17	3.40E-17	4.06E-17	3.56E-17	3.59E-17	2.85E-17	2.40E-17	1.67E-17	2.33E-17	2.95E-17	4.30E-17	4.25E-17	2.74E-17
2.23E-17	2.05E-17	2.90E-17	2.39E-17	3.12E-17	2.01E-17	2.32E-17	3.03E-17	2.72E-17	2.26E-17	2.44E-17	2.53E-17	3.38E-17	3.87E-17	2.66E-17
1.52E-17	1.54E-17	2.04E-17	2.21E-17	2.02E-17	1.04E-17	1.34E-17	3.20E-17	3.11E-17	3.03E-17	2.49E-17	2.07E-17	2.24E-17	2.84E-17	2.53E-17
2.88E-17	1.94E-17	1.84E-17	2.42E-17	1.65E-17	8.16E-18	7.32E-18	2.58E-17	3.23E-17	3.21E-17	2.28E-17	1.25E-17	1.44E-17	2.48E-17	2.48E-17
3.61E-17	2.87E-17	2.10E-17	2.62E-17	1.93E-17	7.17E-18	7.28E-18	2.80E-17	3.58E-17	3.16E-17	1.87E-17	8.67E-18	1.45E-17	2.54E-17	2.75E-17
3.08E-17	2.82E-17	2.90E-17	2.68E-17	2.22E-17	1.09E-17	1.63E-17	3.00E-17	3.14E-17	3.17E-17	1.92E-17	1.40E-17	2.24E-17	2.40E-17	2.66E-17
2.22E-17	1.68E-17	2.93E-17	3.39E-17	3.36E-17	2.43E-17	2.44E-17	3.23E-17	2.80E-17	3.24E-17	2.73E-17	2.23E-17	2.52E-17	1.73E-17	1.60E-17
1.42E-17	1.09E-17	2.25E-17	3.75E-17	4.21E-17	4.17E-17	3.77E-17	3.77E-17	2.71E-17	3.23E-17	3.60E-17	2.48E-17	2.16E-17	1.18E-17	7.27E-18
8.21E-18	1.44E-17	2.50E-17	4.51E-17	5.00E-17	5.42E-17	5.16E-17	4.94E-17	3.57E-17	3.57E-17	3.33E-17	2.70E-17	1.65E-17	1.26E-17	7.51E-18
9.41E-18	2.43E-17	3.77E-17	5.15E-17	5.65E-17	5.93E-17	5.84E-17	5.67E-17	4.21E-17	3.72E-17	3.33E-17	2.61E-17	1.32E-17	1.84E-17	1.05E-17
1.48E-17	3.49E-17	4.69E-17	5.79E-17	6.32E-17	6.14E-17	5.85E-17	5.68E-17	4.17E-17	3.84E-17	3.58E-17	2.50E-17	1.04E-17	1.90E-17	6.35E-18
2.95E-17	4.75E-17	5.06E-17	6.11E-17	6.73E-17	6.14E-17	5.72E-17	5.65E-17	3.89E-17	4.18E-17	3.47E-17	2.97E-17	1.91E-17	1.69E-17	1.98E-18
4.37E-17	5.52E-17	4.68E-17	6.15E-17	6.51E-17	5.18E-17	5.84E-17	6.12E-17	4.88E-17	4.73E-17	4.01E-17	3.15E-17	2.42E-17	1.92E-17	2.22E-18
4.60E-17	5.32E-17	4.98E-17	5.92E-17	6.12E-17	4.87E-17	5.87E-17	6.28E-17	5.49E-17	4.93E-17	4.07E-17	2.40E-17	2.54E-17	2.01E-17	1.59E-17
4.60E-17	5.48E-17	5.68E-17	5.55E-17	5.70E-17	5.57E-17	5.73E-17	4.99E-17	4.84E-17	4.44E-17	3.62E-17	2.38E-17	2.67E-17	1.88E-17	2.80E-17
4.32E-17	5.56E-17	5.96E-17	5.79E-17	5.82E-17	5.95E-17	5.49E-17	3.86E-17	3.75E-17	3.18E-17	3.39E-17	3.31E-17	2.91E-17	2.23E-17	2.81E-17
3.61E-17	5.10E-17	5.56E-17	6.64E-17	6.67E-17	6.83E-17	5.99E-17	3.12E-17	2.58E-17	2.57E-17	3.69E-17	4.17E-17	4.04E-17	3.27E-17	3.68E-17
2.15E-17	4.34E-17	5.89E-17	7.28E-17	6.26E-17	7.26E-17	5.78E-17	3.12E-17	2.03E-17	2.78E-17	3.33E-17	4.25E-17	4.48E-17	4.30E-17	4.21E-17
1.38E-17	4.68E-17	6.51E-17	6.59E-17	5.01E-17	6.49E-17	5.31E-17	3.76E-17	2.71E-17	3.27E-17	2.68E-17	4.11E-17	4.31E-17	4.27E-17	3.99E-17
2.84E-17	5.15E-17	6.61E-17	5.56E-17	3.63E-17	5.65E-17	4.66E-17	4.96E-17	4.49E-17	4.08E-17	3.01E-17	4.64E-17	4.54E-17	4.34E-17	4.08E-17
3.93E-17	5.38E-17	5.46E-17	4.90E-17	4.01E-17	5.44E-17	4.96E-17	5.58E-17	5.35E-17	5.66E-17	4.72E-17	6.40E-17	4.65E-17	4.13E-17	3.31E-17
3.56E-17	4.88E-17	4.45E-17	3.74E-17	4.49E-17	5.14E-17	5.35E-17	5.25E-17	5.23E-17	5.90E-17	5.37E-17	6.52E-17	3.78E-17	2.83E-17	2.50E-17
3.50E-17	4.06E-17	3.59E-17	3.00E-17	3.51E-17	4.69E-17	6.64E-17	5.31E-17	4.66E-17	5.38E-17	4.21E-17	5.56E-17	2.60E-17	1.66E-17	2.03E-17
4.29E-17	3.25E-17	2.62E-17	3.62E-17	3.80E-17	5.08E-17	7.56E-17	6.13E-17	6.06E-17	4.82E-17	2.96E-17	4.15E-17	2.24E-17	1.24E-17	1.77E-17
3.84E-17	2.90E-17	2.52E-17	3.75E-17	4.73E-17	6.72E-17	7.41E-17	6.08E-17	6.34E-17	4.97E-17	3.24E-17	3.41E-17	2.85E-17	2.00E-17	2.32E-17
3.69E-17	3.10E-17	2.73E-17	3.71E-17	4.77E-17	6.95E-17	6.74E-17	6.23E-17	5.64E-17	4.76E-17	3.33E-17	3.58E-17	3.57E-17	3.16E-17	2.81E-17
4.04E-17	3.50E-17	2.31E-17	3.76E-17	4.54E-17	6.95E-17	6.48E-17	6.00E-17	4.76E-17	4.96E-17	4.02E-17	4.18E-17	3.86E-17	3.63E-17	2.79E-17
5.51E-17	4.49E-17	1.90E-17	3.67E-17	3.30E-17	5.52E-17	5.48E-17	4.52E-17	3.81E-17	4.68E-17	4.21E-17	3.80E-17	4.10E-17	3.63E-17	3.09E-17
6.47E-17	4.17E-17	2.48E-17	3.98E-17	3.75E-17	4.64E-17	4.53E-17	3.06E-17	3.47E-17	3.82E-17	3.59E-17	2.93E-17	3.75E-17	2.97E-17	2.94E-17
6.75E-17	4.00E-17	3.25E-17	3.82E-17	4.84E-17	4.44E-17	3.81E-17	2.57E-17	3.62E-17	2.68E-17	2.57E-17	2.46E-17	2.97E-17	2.31E-17	2.41E-17
6.26E-17	4.26E-17	5.77E-17	4.99E-17	6.27E-17	4.73E-17	3.27E-17	2.44E-17	2.92E-17	1.94E-17	2.18E-17	2.36E-17	2.29E-17	1.93E-17	1.49E-17
6.94E-17	5.58E-17	7.50E-17	5.97E-17	6.79E-17	5.18E-17	3.31E-17	1.87E-17	2.20E-17	2.65E-17	2.38E-17	2.26E-17	2.00E-17	1.50E-17	8.25E-18
7.25E-17	6.42E-17	8.08E-17	5.85E-17	6.82E-17	6.32E-17	4.64E-17	2.32E-17	2.40E-17	3.47E-17	3.08E-17	2.04E-17	2.28E-17	1.37E-17	2.98E-18
7.59E-17	6.27E-17	6.77E-17	5.02E-17	6.16E-17	6.83E-17	4.63E-17	3.06E-17	3.19E-17	3.94E-17	2.95E-17	2.08E-17	2.11E-17	1.18E-17	1.15E-18
6.51E-17	5.35E-17	5.42E-17	4.30E-17	4.98E-17	6.27E-17	4.16E-17	4.17E-17	3.57E-17	4.27E-17	3.05E-17	2.10E-17	1.86E-17	1.09E-17	1.22E-18
5.64E-17	4.55E-17	4.34E-17	2.96E-17	3.88E-17	5.61E-17	4.88E-17	4.93E-17	3.16E-17	3.96E-17	3.89E-17	3.22E-17	1.79E-17	8.92E-18	5.28E-19
4.72E-17	3.46E-17	2.82E-17	2.50E-17	3.78E-17	4.66E-17	4.52E-17	5.13E-17	2.68E-17	3.33E-17	3.73E-17	3.13E-17	9.97E-18	1.00E-17	7.90E-19
3.72E-17	2.33E-17	1.92E-17	2.75E-17	4.35E-17	3.82E-17	3.75E-17	4.04E-17	2.47E-17	2.78E-17	3.24E-17	2.65E-17	8.98E-18	1.14E-17	5.20E-18
3.94E-17	2.67E-17	1.66E-17	2.96E-17	4.08E-17	2.60E-17	3.08E-17	3.28E-17	2.08E-17	1.93E-17	2.45E-17	2.67E-17	1.82E-17	1.92E-17	1.31E-17
5.17E-17	3.61E-17	2.14E-17	2.99E-17	4.11E-17	2.30E-17	2.89E-17	3.44E-17	2.35E-17	1.29E-17	1.75E-17	2.43E-17	2.26E-17	2.71E-17	2.28E-17
5.30E-17	3.46E-17	2.51E-17	2.05E-17	3.10E-17	2.40E-17	3.35E-17	3.96E-17	3.26E-17	1.64E-17	1.50E-17	1.96E-17	2.04E-17	2.60E-17	2.78E-17
5.05E-17	2.37E-17	2.37E-17	1.98E-17	2.38E-17	2.53E-17	3.54E-17	3.15E-17	2.72E-17	2.09E-17	1.28E-17	1.56E-17	1.38E-17	2.76E-17	2.80E-17
4.54E-17	2.79E-17	2.63E-17	2.25E-17	2.73E-17	2.47E-17	2.68E-17	1.96E-17	1.56E-17	1.44E-17	1.62E-17	1.66E-17	1.77E-17	3.13E-17	2.34E-17
4.13E-17	3.96E-17	2.57E-17	1.93E-17	2.37E-17	2.08E-17	1.68E-17	1.36E-17	8.67E-18	1.40E-17	1.88E-17	1.42E-17	2.15E-17	3.07E-17	2.59E-17
3.97E-17	4.58E-17	2.93E-17	1.83E-17	1.33E-17	1.14E-17	5.88E-18	5.90E-18	5.62E-18	1.43E-17	1.38E-17	8.21E-18	1.77E-17	2.88E-17	2.39E-17
4.25E-17	4.75E-17	4.05E-17	2.41E-17	9.14E-18	4.40E-18	5.22E-18	6.06E-18	3.41E-18	1.40E-17	1.69E-17	7.37E-18	1.34E-17	2.64E-17	2.09E-17
4.54E-17	4.40E-17	4.97E-17	3.30E-17	1.12E-17	4.65E-18	7.77E-18	8.80E-18	5.92E-18	1.21E-17	2.22E-17	1.05E-17	1.41E-17	2.57E-17	1.87E-17
4.29E-17	3.98E-17	4.58E-17	3.56E-17	8.49E-18	4.89E-18	1.03E-17	1.45E-17	6.15E-18	8.98E-18	2.09E-17	1.00E-17	1.81E-17	2.55E-17	2.12E-17
3.13E-17	3.50E-17	3.27E-17	2.77E-17	9.32E-18	8.76E-18	1.90E-17	1.51E-17	9.69E-18	1.15E-17	1.50E-17	1.51E-17	2.35E-17	1.63E-17	1.92E-17
2.18E-17	2.62E-17	2.06E-17	2.39E-17	2.40E-17	2.12E-17	3.29E-17	1.64E-17	1.07E-17	1.25E-17	1.02E-17	2.32E-17	1.98E-17	1.13E-17	1.52E-17
2.15E-17	2.50E-17	2.12E-17	2.53E-17	2.87E-17	3.78E-17	3.74E-17	1.59E-17	1.54E-17	7.76E-18	7.83E-18	2.21E-17	2.19E-17	1.79E-17	2.00E-17
2.32E-17	2.93E-17	2.22E-17	1.97E-17	2.30E-17	3.77E-17	3.10E-17	1.25E-17	8.19E-18	8.79E-18	8.79E-18	2.42E-17	2.45E-17	2.60E-17	2.34E-17
2.04E-17	1.94E-17	1.36E-17	1.72E-17	1.78E-17	3.12E-17	2.00E-17	1.38E-17	6.07E-18	1.12E-17	1.59E-17	2.91E-17	2.71E-17	2.34E-17	2.41E-17
1.99E-17	1.33E-17	1.58E-17	2.72E-17	2.30E-17	2.11E-17	8.16E-18	8.58E-18	4.89E-18	1.86E-17	2.43E-17	2.66E-17	2.08E-17	1.47E-17	2.13E-17
2.39E-17	1.33E-17	2.82E-17	3.56E-17	2.11E-17	1.46E-17	1.62E-18	2.27E-18	4.47E-18	1.77E-17	2.55E-17	2.69E-17	1.68E-17	1.04E-17	1.88E-17
2.45E-17	2.40E-17	3.09E-17	3.42E-17	1.59E-17	1.36E-17	2.69E-18	2.70E-18	2.94E-18	1.95E-17	2.51E-17	2.52E-17	1.93E-17	1.39E-17	2.06E-17
2.76E-17	3.18E-17	3.22E-17	2.99E-17	1.06E-17	1.65E-17	1.15E-17	1.27E-17	1.01E-17	2.45E-17	2.35E-17	2.16E-17	2.33E-17	2.19E-17	1.95E-17
3.10E-17	3.41E-17	3.10E-17	2.75E-17	8.28E-18	1.73E-17	1.80E-17	2.03E-17	2.03E-17	2.52E-17	1.77E-17	2.48E-17	2.14E-17	2.24E-17	2.12E-17
3.26E-17	2.80E-17	3.53E-17	3.35E-17	1.39E-17	2.42E-17	2.40E-17	2.39E-17	2.14E-17	2.47E-17	1.52E-17	2.77E-17	2.18E-17	2.43E-17	2.16E-17
2.46E-17	2.39E-17	3.53E-17	3.39E-17	1.80E-17	2.47E-17	2.14E-17	2.30E-17	2.08E-17	2.46E-17	1.65E-17	2.80E-17	1.99E-17	2.48E-17	2.40E-17
1.46E-17	1.80E-17	2.33E-17	2.86E-17	1.81E-17	1.97E-17	1.78E-17	1.91E-17	2.18E-17	2.39E-17	2.05E-17	2.90E-17	2.22E-17	2.10E-17	1.42E-17
6.00E-18	8.37E-18	1.21E-17	2.12E-17	1.64E-17	1.23E-17	1.46E-17	1.75E-17	1.89E-17	2.37E-17	2.75E-17	2.42E-17	1.76E-17	1.23E-17	8.35E-18
3.54E-19	2.34E-18	5.73E-18	1.53E-17	1.12E-17	3.79E-18	9.16E-18	1.69E-17	1.66E-17	2.41E-17	2.86E-17	1.89E-17	1.18E-17	6.85E-18	3.75E-18

6.42E-20	8.08E-19	4.55E-18	1.18E-17	8.98E-18	2.80E-18	7.52E-18	2.09E-17	1.24E-17	2.13E-17	3.07E-17	1.74E-17	8.32E-18	7.82E-18	5.57E-18
1.27E-20	1.64E-18	4.46E-18	7.82E-18	9.81E-18	7.16E-18	8.13E-18	1.73E-17	8.07E-18	1.38E-17	2.12E-17	1.27E-17	6.94E-18	1.22E-17	1.05E-17
2.33E-19	1.44E-18	3.79E-18	5.33E-18	1.17E-17	1.01E-17	1.08E-17	1.22E-17	5.18E-18	6.47E-18	1.04E-17	3.44E-18	4.08E-18	9.29E-18	1.04E-17
4.51E-19	8.06E-19	3.14E-18	4.22E-18	9.74E-18	9.67E-18	1.06E-17	9.87E-18	4.31E-18	1.71E-18	2.04E-18	1.13E-19	1.13E-18	4.14E-18	5.68E-18
7.00E-20	4.40E-19	3.29E-18	2.65E-18	5.44E-18	9.12E-18	9.85E-18	7.85E-18	3.82E-18	8.47E-20	1.54E-19	2.89E-18	4.47E-19	3.33E-18	5.27E-18
6.26E-19	2.01E-18	2.04E-18	1.11E-18	1.69E-18	4.74E-18	9.13E-18	8.36E-18	7.08E-18	6.65E-19	1.55E-19	4.19E-18	3.62E-19	2.29E-18	5.16E-18
3.30E-19	1.07E-18	6.65E-19	3.15E-21	1.62E-20	2.67E-18	8.10E-18	1.10E-17	9.90E-18	3.86E-18	3.20E-20	6.36E-18	2.20E-18	6.88E-19	3.15E-18
1.35E-19	1.35E-19	2.84E-19	2.19E-18	4.44E-19	4.70E-18	7.02E-18	1.13E-17	1.05E-17	4.60E-18	3.21E-20	3.16E-18	4.48E-19	1.55E-19	2.51E-18
1.08E-18	2.68E-18	9.01E-18	7.50E-18	1.89E-18	2.85E-18	4.31E-18	7.66E-18	8.03E-18	4.52E-18	3.69E-19	1.39E-19	6.82E-19	4.25E-21	2.11E-18
9.43E-18	1.26E-17	2.23E-17	8.99E-18	2.82E-18	1.35E-19	9.81E-19	2.05E-18	4.23E-18	3.20E-18	1.44E-19	3.90E-20	2.00E-18	1.82E-18	1.73E-18
1.70E-17	2.58E-17	2.96E-17	2.33E-17	9.32E-18	2.37E-18	2.17E-19	1.69E-19	9.09E-19	5.89E-19	8.80E-20	1.31E-18	1.90E-18	6.70E-18	9.15E-19
1.12E-14	1.12E-14	1.16E-14	1.10E-14	1.06E-14	1.10E-14	1.07E-14	1.05E-14	1.01E-14	1.02E-14	9.62E-15	9.49E-15	9.27E-15	9.08E-15	8.37E-15

2.68E-08	2.69E-08	2.57E-08	2.48E-08	2.53E-08	2.53E-08	2.42E-08	2.48E-08	2.43E-08	2.39E-08	2.44E-08	2.36E-08	2.25E-08	2.19E-08	2.22E-08
2.57E-08	2.62E-08	2.47E-08	2.38E-08	2.42E-08	2.40E-08	2.36E-08	2.37E-08	2.35E-08	2.27E-08	2.29E-08	2.22E-08	2.11E-08	2.03E-08	2.08E-08
2.46E-08	2.49E-08	2.37E-08	2.27E-08	2.27E-08	2.24E-08	2.30E-08	2.29E-08	2.25E-08	2.15E-08	2.15E-08	2.08E-08	2.01E-08	1.95E-08	1.98E-08
2.34E-08	2.28E-08	2.29E-08	2.17E-08	2.16E-08	2.15E-08	2.21E-08	2.17E-08	2.13E-08	2.05E-08	2.03E-08	1.97E-08	1.92E-08	1.92E-08	1.94E-08
2.19E-08	2.13E-08	2.13E-08	2.03E-08	2.04E-08	2.07E-08	2.06E-08	2.00E-08	1.96E-08	1.94E-08	1.91E-08	1.85E-08	1.83E-08	1.86E-08	1.87E-08
2.07E-08	2.00E-08	1.99E-08	1.95E-08	1.96E-08	1.98E-08	1.93E-08	1.83E-08	1.79E-08	1.81E-08	1.76E-08	1.69E-08	1.74E-08	1.74E-08	1.67E-08
1.94E-08	1.88E-08	1.87E-08	1.85E-08	1.86E-08	1.88E-08	1.81E-08	1.66E-08	1.62E-08	1.69E-08	1.60E-08	1.55E-08	1.58E-08	1.49E-08	1.47E-08
1.82E-08	1.80E-08	1.75E-08	1.77E-08	1.75E-08	1.77E-08	1.67E-08	1.52E-08	1.49E-08	1.53E-08	1.45E-08	1.46E-08	1.42E-08	1.32E-08	1.32E-08
1.72E-08	1.74E-08	1.64E-08	1.64E-08	1.66E-08	1.63E-08	1.53E-08	1.41E-08	1.43E-08	1.44E-08	1.35E-08	1.35E-08	1.27E-08	1.21E-08	1.21E-08
1.65E-08	1.65E-08	1.57E-08	1.56E-08	1.59E-08	1.52E-08	1.45E-08	1.37E-08	1.37E-08	1.41E-08	1.27E-08	1.25E-08	1.18E-08	1.11E-08	1.14E-08
1.58E-08	1.55E-08	1.52E-08	1.48E-08	1.46E-08	1.42E-08	1.33E-08	1.30E-08	1.35E-08	1.34E-08	1.15E-08	1.14E-08	1.10E-08	1.03E-08	1.06E-08
1.50E-08	1.45E-08	1.51E-08	1.41E-08	1.36E-08	1.31E-08	1.26E-08	1.22E-08	1.34E-08	1.17E-08	1.06E-08	1.06E-08	9.70E-09	9.17E-09	8.94E-09
1.38E-08	1.39E-08	1.47E-08	1.35E-08	1.30E-08	1.24E-08	1.20E-08	1.13E-08	1.17E-08	1.00E-08	9.49E-09	9.72E-09	8.81E-09	8.27E-09	7.64E-09
1.32E-08	1.29E-08	1.38E-08	1.29E-08	1.24E-08	1.22E-08	1.16E-08	1.08E-08	1.01E-08	8.84E-09	8.11E-09	8.58E-09	7.98E-09	7.08E-09	6.98E-09
1.22E-08	1.21E-08	1.28E-08	1.23E-08	1.19E-08	1.21E-08	1.09E-08	9.77E-09	9.14E-09	7.87E-09	7.51E-09	7.32E-09	6.45E-09	6.06E-09	6.57E-09
1.09E-08	1.10E-08	1.19E-08	1.17E-08	1.14E-08	1.16E-08	9.92E-09	8.89E-09	8.31E-09	7.28E-09	7.25E-09	7.02E-09	5.45E-09	4.95E-09	6.53E-09
1.04E-08	1.00E-08	1.09E-08	1.06E-08	1.12E-08	1.10E-08	9.16E-09	8.35E-09	8.11E-09	7.16E-09	7.02E-09	6.82E-09	4.65E-09	4.38E-09	6.29E-09
1.04E-08	9.32E-09	1.03E-08	1.01E-08	1.07E-08	1.00E-08	8.39E-09	7.77E-09	7.52E-09	7.21E-09	6.45E-09	5.57E-09	4.07E-09	4.09E-09	3.85E-09
9.73E-09	8.99E-09	9.58E-09	1.01E-08	9.66E-09	8.75E-09	7.98E-09	7.42E-09	7.39E-09	7.12E-09	6.37E-09	4.71E-09	3.80E-09	4.32E-09	3.19E-09
8.95E-09	8.65E-09	8.61E-09	9.36E-09	8.51E-09	7.87E-09	7.55E-09	6.95E-09	7.19E-09	6.57E-09	6.09E-09	4.45E-09	3.79E-09	4.96E-09	3.06E-09
8.13E-09	8.47E-09	7.75E-09	8.57E-09	7.69E-09	7.14E-09	6.67E-09	6.14E-09	5.94E-09	5.12E-09	5.36E-09	4.28E-09	3.78E-09	5.02E-09	2.87E-09
6.94E-09	7.35E-09	6.69E-09	7.62E-09	6.85E-09	6.32E-09	5.49E-09	5.34E-09	4.71E-09	4.13E-09	4.14E-09	3.81E-09	3.81E-09	4.47E-09	2.77E-09
6.11E-09	6.45E-09	5.91E-09	6.59E-09	6.37E-09	5.89E-09	4.84E-09	4.92E-09	3.96E-09	3.29E-09	3.57E-09	3.20E-09	3.60E-09	4.24E-09	2.74E-09
5.63E-09	5.41E-09	5.34E-09	5.86E-09	5.72E-09	5.27E-09	4.56E-09	4.71E-09	3.34E-09	2.88E-09	2.91E-09	2.67E-09	3.17E-09	3.47E-09	2.64E-09
4.73E-09	4.18E-09	4.90E-09	5.11E-09	5.00E-09	4.53E-09	3.85E-09	4.19E-09	2.80E-09	2.21E-09	2.31E-09	2.39E-09	3.12E-09	2.72E-09	2.76E-09
3.48E-09	3.58E-09	4.40E-09	4.47E-09	4.77E-09	3.99E-09	3.19E-09	3.35E-09	2.27E-09	2.22E-09	1.77E-09	2.18E-09	2.86E-09	2.30E-09	2.73E-09
2.65E-09	2.88E-09	3.61E-09	3.75E-09	4.09E-09	3.40E-09	2.71E-09	2.77E-09	1.51E-09	1.90E-09	1.59E-09	1.83E-09	2.69E-09	2.53E-09	2.68E-09
2.05E-09	1.89E-09	2.56E-09	2.99E-09	3.19E-09	2.94E-09	2.38E-09	2.30E-09	1.01E-09	1.41E-09	1.41E-09	1.48E-09	2.72E-09	2.30E-09	2.10E-09
1.22E-09	1.20E-09	1.83E-09	1.98E-09	2.22E-09	2.56E-09	1.90E-09	1.59E-09	5.93E-10	7.00E-10	1.40E-09	1.27E-09	2.34E-09	1.98E-09	1.69E-09
5.46E-10	6.89E-10	9.67E-10	1.05E-09	1.44E-09	2.23E-09	1.75E-09	1.05E-09	4.98E-10	5.08E-10	1.57E-09	9.95E-10	1.72E-09	1.76E-09	1.55E-09
4.40E-10	5.35E-10	7.85E-10	9.01E-10	8.93E-10	1.57E-09	1.45E-09	5.51E-10	3.12E-10	2.31E-10	1.59E-09	8.04E-10	1.45E-09	1.72E-09	1.90E-09
5.34E-10	3.68E-10	9.53E-10	8.38E-10	8.31E-10	9.07E-10	5.88E-10	-6.57E-11	4.63E-10	2.69E-11	1.11E-09	8.05E-10	1.31E-09	1.47E-09	2.06E-09
4.42E-10	3.28E-10	9.92E-10	3.12E-10	8.02E-10	9.12E-10	1.41E-10	-2.04E-10	3.30E-10	2.52E-11	1.34E-09	9.87E-10	1.56E-09	1.55E-09	2.02E-09
8.12E-11	4.77E-11	3.99E-10	3.44E-10	8.03E-10	9.60E-10	-4.75E-12	-1.25E-10	6.18E-10	6.09E-10	1.61E-09	1.83E-09	1.71E-09	1.78E-09	1.57E-09
-3.54E-10	-6.02E-10	5.50E-10	4.34E-10	6.46E-10	4.63E-10	-1.08E-10	-6.00E-11	3.26E-10	4.63E-10	1.74E-09	2.13E-09	1.80E-09	1.66E-09	1.17E-09
-7.53E-10	-9.93E-10	6.85E-10	4.69E-10	4.88E-10	-3.13E-11	-3.00E-10	-5.25E-10	-3.34E-10	1.62E-10	1.28E-09	2.11E-09	1.75E-09	1.16E-09	1.14E-09
-1.10E-09	-8.11E-10	3.97E-10	2.45E-10	-3.78E-10	-8.61E-10	-8.83E-10	-8.46E-10	-7.37E-10	3.44E-10	9.94E-10	1.72E-09	1.27E-09	6.39E-10	9.46E-10
-1.87E-09	-1.13E-09	9.41E-11	-8.65E-10	-1.69E-09	-1.61E-09	-1.04E-09	-8.55E-10	-1.43E-09	4.33E-10	9.68E-10	1.70E-09	1.47E-09	5.68E-10	1.32E-09
-2.33E-09	-1.73E-09	-9.92E-10	-1.82E-09	-1.99E-09	-1.75E-09	-9.26E-10	-7.70E-10	-1.50E-09	2.13E-10	8.31E-10	1.64E-09	1.64E-09	1.08E-09	1.33E-09
-2.58E-09	-2.28E-09	-2.27E-09	-2.33E-09	-1.79E-09	-1.87E-09	-8.39E-10	-9.96E-10	-1.14E-09	2.56E-10	4.61E-10	1.16E-09	1.63E-09	1.22E-09	1.12E-09
-2.83E-09	-2.67E-09	-3.00E-09	-2.83E-09	-2.00E-09	-2.39E-09	-8.92E-10	-1.17E-09	-1.18E-09	4.98E-11	3.88E-10	8.18E-10	1.69E-09	1.93E-09	1.19E-09
-3.92E-09	-3.81E-09	-4.02E-09	-3.35E-09	-2.76E-09	-2.55E-09	-1.12E-09	-1.54E-09	-1.77E-09	-8.15E-10	4.66E-10	7.04E-10	1.12E-09	1.42E-09	1.23E-09
-4.41E-09	-3.47E-09	-3.53E-09	-3.06E-09	-2.84E-09	-2.56E-09	-1.42E-09	-1.33E-09	-1.90E-09	-1.16E-09	-3.46E-10	7.66E-10	1.10E-09	1.31E-09	9.69E-10
-4.13E-09	-2.43E-09	-2.80E-09	-3.35E-09	-3.01E-09	-2.48E-09	-1.35E-09	-9.71E-10	-1.14E-09	-1.10E-09	-8.90E-10	8.79E-10	1.33E-09	1.52E-09	7.72E-10
-3.25E-09	-1.83E-09	-2.35E-09	-2.99E-09	-2.78E-09	-2.26E-09	-1.65E-09	-1.07E-09	-1.09E-09	-1.04E-09	-7.59E-10	8.39E-10	1.49E-09	2.27E-09	1.23E-09
-2.69E-09	-1.15E-09	-1.85E-09	-2.08E-09	-2.02E-09	-2.01E-09	-2.16E-09	-1.13E-09	-1.46E-09	-5.03E-10	-4.46E-10	1.12E-09	1.37E-09	2.14E-09	1.19E-09
-2.02E-09	-6.90E-10	-1.70E-09	-1.34E-09	-1.57E-09	-1.77E-09	-2.57E-09	-1.14E-09	-1.38E-09	6.43E-10	3.79E-10	1.36E-09	7.06E-10	1.80E-09	1.32E-09
-1.73E-09	-9.18E-10	-1.87E-09	-1.47E-09	-1.25E-09	-1.73E-09	-2.55E-09	-1.21E-09	-1.06E-09	8.57E-10	1.26E-09	1.76E-09	5.66E-10	1.21E-09	3.74E-10
-1.82E-09	-1.78E-09	-2.37E-09	-2.24E-09	-1.41E-09	-2.57E-09	-2.40E-09	-1.41E-09	-5.90E-10	8.15E-10	1.30E-09	1.72E-09	8.05E-10	9.27E-10	1.39E-10
-2.44E-09	-2.09E-09	-2.12E-09	-2.73E-09	-2.18E-09	-3.17E-09	-2.83E-09	-1.92E-09	-1.65E-10	6.42E-10	4.85E-10	4.93E-10	7.98E-10	1.34E-09	1.82E-09
-2.57E-09	-2.37E-09	-2.20E-09	-3.50E-09	-2.70E-09	-3.49E-09	-3.10E-09	-2.31E-09	-2.03E-10	-3.27E-10	-1.31E-09	-1.88E-10	6.26E-10	9.91E-10	1.72E-09
-3.68E-09	-3.35E-09	-2.80E-09	-3.46E-09	-3.47E-09	-3.58E-09	-3.71E-09	-3.17E-09	-1.35E-09	-1.74E-09	-1.74E-09	-7.82E-10	5.89E-11	4.31E-10	1.05E-09
-4.41E-09	-3.90E-09	-3.47E-09	-3.17E-09	-3.65E-09	-3.46E-09	-4.38E-09	-3.66E-09	-1.55E-09	-1.63E-09	-1.21E-09	-1.10E-09	-5.19E-10	-4.05E-10	3.19E-10
-4.82E-09	-4.22E-09	-3.83E-09	-3.22E-09	-3.76E-09	-3.68E-09	-4.12E-09	-3.45E-09	-1.08E-09	-8.19E-10	-6.42E-10	-6.67E-10	-5.75E-10	-9.45E-10	-4.42E-10
-4.56E-09	-3.85E-09	-3.73E-09	-2.72E-09	-3.44E-09	-2.98E-09	-2.71E-09	-2.75E-09	-8.53E-10	-2.62E-10	-3.35E-10	-3.77E-10	-9.10E-10	-8.69E-10	-8.47E-10
-4.47E-09	-3.00E-09	-2.45E-09	-1.89E-09	-2.43E-09	-2.17E-09	-1.55E-09	-1.75E-09	-1.24E-09	-1.04E-09	-4.61E-10	-3.09E-10	-1.03E-09	-4.20E-10	-5.80E-10
-4.81E-09	-2.28E-09	-1.76E-09	-1.27E-09	-1.75E-09	-1.51E-09	-1.36E-09	-1.15E-09	-1.88E-09	-2.06E-09	-1.48E-09	-8.83E-10	-1.49E-09	-4.47E-10	-4.14E-10
-4.76E-09	-2.01E-09	-2.20E-09	-1.38E-09	-1.15E-09	-1.89E-09	-1.67E-09	-1.41E-09	-1.84E-09	-2.60E-09	-2.06E-09	-1.52E-09	-2.36E-09	-1.10E-09	-9.55E-10
-4.54E-09	-3.07E-09	-3.28E-09	-1.86E-09	-1.59E-09	-2.63E-09	-2.49E-09	-1.72E-09	-1.93E-09	-3.10E-09	-2.38E-09	-1.78E-09	-2.62E-09	-1.88E-09	-1.15E-09
-3.89E-09	-3.61E-09	-3.82E-09	-1.76E-09	-1.67E-09	-2.89E-09	-1.84E-09	-1.72E-09	-2.58E-09	-3.44E-09	-2.65E-09	-1.51E-09	-2.53E-09	-1.88E-09	-1.10E-09
-2.74E-09	-3.35E-09	-3.87E-09	-2.25E-09	-2.19E-09	-2.68E-09	-1.42E-09	-1.66E-09	-2.98E-09	-3.14E-09	-2.36E-09	-1.48E-09	-2.56E-09	-1.85E-09	-6.92E-10
-2.23E-09	-3.02E-09	-4.20E-09	-3.88E-09	-2.67E-09	-3.17E-09	-1.80E-09	-1.58E-09	-2.17E-09	-2.30E-09	-1.79E-09	-1.32E-09	-2.08E-09	-1.35E-09	-7.71E-11
-2.41E-09	-3.58E-09	-4.72E-09	-4.50E-09	-3.05E-09	-3.37E-09	-1.09E-09	-5.86E-10	-1.17E-09	-1.28E-09	-1.12E-09	-1.28E-09	-1.26E-09	-7.98E-10	3.69E-10
-1.99E-09	-3.56E-09	-5.06E-09	-4.76E-09	-3.66E-09	-3.11E-09	-1.48E-09	-3.76E-10	-2.06E-12	-4.81E-10	-6.22E-10	-8.20E-10	-1.96E-10	-1.92E-10	9.20E-11
-1.91E-09	-3.54E-09	-5.40E-09	-4.71E-09	-3.57E-09	-2.32E-09	-1.50E-09	-1.34E-10	2.84E-10	-5.45E-10	-4.08E-10	-8.18E-10	5.55E-10	-1.48E-10	1.68E-10
-2.38E-09	-3.33E-09	-4.24E-09	-4.55E-09	-3.32E-09	-1.95E-09	-4.86E-10	-1.38E-10	1.90E-10	-1.22E-09	-1.03E-09	-1.49E-09	2.93E-10	-2.67E-11	-7.44E-10
-2.63E-09	-3.47E-09	-4.31E-09	-4.59E-09	-3.67E-09	-1.34E-09	-1.21E-10	-3.12E-10	-1.57E-10	-1.83E-09	-1.79E-09	-2.09E-09	-4.50E-10	-6.22E-10	-9.51E-10
-4.29E-09	-4.65E-09	-5.10E-09	-4.87E-09	-3.38E-09	-9.69E-10	-1.47E-10	-7.49E-11	-8.97E-10	-2.09E-09	-2.20E-09	-2.16E-09	-1.38E-09	-6.77E-10	-1.84E-10
-5.69E-09	-5.90E-09	-5.85E-09	-4.96E-09	-3.46E-09	-8.66E-10	-2.21E-10	-1.71E-10	-2.10E-09	-2.34E-09	-2.33E-09	-1.73E-09	-1.64E-09	-6.03E-11	3.21E-10
-5.89E-09	-6.38E-09	-6.02E-09	-5.16E-09	-3.81E-09	-1.80E-09	-8.88E-10	-1.20E-09	-2.56E-09	-1.70E-09	-2.21E-09	-1.83E-09	-1.91E-09	-6.50E-10	3.28E-10
-6.17E-09	-6.38E-09	-5.70E-09	-5.30E-09	-4.20E-09	-3.71E-09	-1.96E-09	-1.63E-09	-1.59E-09	-1.05E-09	-2.15E-09	-2.31E-09	-1.88E-09	-1.69E-09	-9.93E-10
-6.28E-09	-5.94E-09	-5.05E-09	-4.92E-09	-4.07E-09	-4.17E-09	-2.96E-09	-1.35E-09	-1.56E-10	-1.07E-09	-1.53E-09	-2.14E-09	-2.04E-09	-2.31E-09	-1.88E-09
-6.24E-09	-6.12E-09	-4.35E-09	-4.03E-09	-3.44E-09	-4.03E-09	-3.47E-09	-1.87E-09	-1.20E-09	-1.85E-09	-1.64E-09	-2.26E-09	-2.49E-09	-2.28E-09	-1.76E-09
-5.63E-09	-6.04E-09	-4.20E-09	-3.36E-09	-3.17E-09	-4.19E-09	-2.89E-09	-2.16E-09	-2.86E-09	-3.52E-09	-2.64E-09	-2.02E-09	-2.72E-09	-2.26E-09	-1.63E-09
-4.71E-09	-5.58E-09	-4.24E-09	-3.81E-09	-3.53E-09	-4.37E-09	-2.81E-09	-2.25E-09	-3.66E-09	-4.23E-09	-3.13E-09	-2.09E-09	-2.74E-09	-2.18E-09	-1.59E-09
-4.66E-09	-4.97E-09	-4.05E-09	-3.08E-09	-3.47E-09	-4.50E-09	-3.12E-09	-2.09E-09	-3.43E-09	-4.14E-09	-3.14E-09	-2.01E-09	-2.56E-09	-1.99E-09	-1.31E-09
-4.98E-09	-4.67E-09	-3.32E-09	-2.06E-09	-3.39E-09	-4.47E-09	-3.22E-09	-2.53E-09	-3.51E-09	-3.78E-09	-2.95E-09	-1.62E-09	-1.75E-09	-1.56E-09	-1.27E-09
-4.64E-09	-4.69E-09	-3.26E-09	-2.86E-09	-3.75E-09	-4.33E-09	-2.73E-09	-2.68E-09	-3.37E-09	-3.25E-09	-2.69E-09	-1.49E-09	-1.21E-09	-1.51E-09	-1.15E-09

-4.79E-09	-4.79E-09	-3.72E-09	-4.56E-09	-4.21E-09	-3.57E-09	-2.61E-09	-3.24E-09	-2.92E-09	-2.83E-09	-1.97E-09	-1.38E-09	-4.71E-10	-7.43E-10	-1.11E-09
-5.11E-09	-4.71E-09	-4.05E-09	-4.71E-09	-4.14E-09	-3.20E-09	-3.35E-09	-3.75E-09	-2.33E-09	-1.92E-09	-1.24E-09	-8.98E-10	4.50E-10	1.31E-11	-6.23E-10
-4.93E-09	-4.46E-09	-4.05E-09	-4.74E-09	-4.55E-09	-3.88E-09	-4.35E-09	-3.81E-09	-1.53E-09	-9.96E-10	-4.35E-10	-4.41E-10	1.29E-09	4.87E-10	-6.19E-10
-4.29E-09	-4.67E-09	-4.27E-09	-4.70E-09	-4.52E-09	-3.92E-09	-4.53E-09	-3.16E-09	-4.43E-10	1.04E-09	8.53E-10	-1.87E-10	1.41E-09	5.43E-10	6.63E-11
-3.77E-09	-4.30E-09	-3.99E-09	-4.56E-09	-4.20E-09	-3.09E-09	-4.40E-09	-2.18E-09	1.26E-09	3.92E-09	2.65E-09	4.51E-10	9.15E-10	-1.93E-10	4.51E-11
-3.42E-09	-4.13E-09	-4.49E-09	-4.50E-09	-4.27E-09	-3.28E-09	-3.92E-09	-1.18E-09	2.21E-09	5.57E-09	3.88E-09	8.02E-10	-3.40E-10	-7.05E-10	1.17E-10
-3.31E-09	-4.30E-09	-4.89E-09	-4.69E-09	-3.87E-09	-2.77E-09	-2.96E-09	-4.21E-10	1.43E-09	4.15E-09	2.85E-09	1.90E-10	-1.25E-09	-1.41E-09	-1.25E-10
-3.48E-09	-3.70E-09	-4.23E-09	-3.91E-09	-3.98E-09	-2.78E-09	-2.02E-09	-1.48E-09	2.60E-10	1.52E-09	9.66E-10	-6.43E-10	-1.85E-09	-1.84E-09	-7.13E-10
-2.98E-09	-2.89E-09	-3.17E-09	-2.61E-09	-3.47E-09	-3.24E-09	-2.28E-09	-2.40E-09	-1.28E-09	-9.27E-10	-1.23E-09	-2.11E-09	-1.94E-09	-1.27E-09	-1.43E-09
-1.97E-09	-2.56E-09	-2.99E-09	-1.61E-09	-2.59E-09	-2.84E-09	-2.84E-09	-3.10E-09	-2.51E-09	-2.12E-09	-2.18E-09	-2.54E-09	-1.53E-09	-5.99E-10	-1.53E-09
-1.69E-09	-3.31E-09	-3.57E-09	-1.43E-09	-2.07E-09	-2.46E-09	-2.73E-09	-3.28E-09	-2.99E-09	-2.33E-09	-2.11E-09	-2.02E-09	-1.71E-09	-2.17E-10	-1.59E-09
-1.94E-09	-3.27E-09	-4.02E-09	-2.56E-09	-2.33E-09	-2.23E-09	-2.64E-09	-3.54E-09	-3.12E-09	-2.03E-09	-1.79E-09	-9.50E-10	-1.16E-09	-1.14E-09	-1.81E-09
-2.15E-09	-2.91E-09	-3.56E-09	-3.22E-09	-3.16E-09	-2.20E-09	-2.24E-09	-3.22E-09	-2.85E-09	-1.83E-09	-1.52E-09	-8.95E-10	-1.14E-09	-2.24E-09	-1.92E-09
-2.52E-09	-3.01E-09	-2.59E-09	-2.88E-09	-2.95E-09	-1.58E-09	-1.31E-09	-2.76E-09	-2.67E-09	-1.27E-09	-1.92E-09	-1.64E-09	-1.71E-09	-2.46E-09	-1.82E-09
-3.63E-09	-3.55E-09	-2.64E-09	-2.21E-09	-2.55E-09	-1.77E-09	-4.11E-10	-1.87E-09	-2.63E-09	-2.23E-09	-2.26E-09	-2.78E-09	-2.35E-09	-2.21E-09	-1.58E-09
-4.02E-09	-4.00E-09	-3.15E-09	-1.78E-09	-2.03E-09	-1.79E-09	-5.12E-11	-1.70E-09	-2.91E-09	-3.42E-09	-2.58E-09	-2.60E-09	-2.60E-09	-1.71E-09	-1.29E-09
-4.67E-09	-4.67E-09	-3.75E-09	-1.82E-09	-2.14E-09	-2.15E-09	-1.15E-09	-1.98E-09	-3.42E-09	-3.34E-09	-3.13E-09	-2.17E-09	-2.26E-09	-1.40E-09	-1.05E-09
-4.87E-09	-5.02E-09	-4.41E-09	-3.30E-09	-3.68E-09	-3.30E-09	-3.00E-09	-2.75E-09	-3.27E-09	-3.47E-09	-3.25E-09	-1.93E-09	-1.45E-09	-1.25E-09	-1.29E-09
-4.63E-09	-5.26E-09	-4.51E-09	-4.53E-09	-5.01E-09	-3.89E-09	-3.62E-09	-2.72E-09	-2.66E-09	-3.09E-09	-2.62E-09	-1.56E-09	-1.69E-09	-1.26E-09	-1.16E-09
-4.54E-09	-5.01E-09	-4.82E-09	-5.10E-09	-5.24E-09	-4.01E-09	-3.72E-09	-2.46E-09	-1.64E-09	-2.76E-09	-2.04E-09	-1.64E-09	-2.39E-09	-2.19E-09	-1.88E-09
-4.54E-09	-4.54E-09	-4.65E-09	-5.00E-09	-5.13E-09	-4.60E-09	-3.85E-09	-2.57E-09	-1.50E-09	-2.45E-09	-1.57E-09	-1.71E-09	-2.48E-09	-2.78E-09	-2.33E-09
-3.88E-09	-3.07E-09	-4.28E-09	-4.80E-09	-4.75E-09	-5.03E-09	-4.33E-09	-3.19E-09	-2.27E-09	-2.15E-09	-1.69E-09	-1.64E-09	-1.74E-09	-2.00E-09	-1.65E-09
-3.37E-09	-2.02E-09	-3.52E-09	-4.63E-09	-4.53E-09	-4.73E-09	-4.48E-09	-3.76E-09	-3.17E-09	-2.00E-09	-2.01E-09	-1.39E-09	-4.49E-10	-5.52E-10	-1.11E-09
-2.91E-09	-2.03E-09	-2.98E-09	-4.02E-09	-4.78E-09	-4.55E-09	-4.43E-09	-4.00E-09	-3.76E-09	-2.49E-09	-2.22E-09	-1.54E-09	-2.28E-10	5.14E-10	-6.19E-10
-2.76E-09	-2.47E-09	-3.19E-09	-3.56E-09	-4.86E-09	-4.37E-09	-4.47E-09	-4.05E-09	-3.84E-09	-2.73E-09	-2.95E-09	-2.74E-09	-1.33E-09	2.51E-11	-8.67E-10
-3.03E-09	-2.80E-09	-4.35E-09	-3.89E-09	-4.71E-09	-3.64E-09	-4.23E-09	-3.83E-09	-3.37E-09	-2.11E-09	-2.91E-09	-3.18E-09	-2.47E-09	-1.77E-09	-2.09E-09
-3.84E-09	-3.55E-09	-5.11E-09	-4.28E-09	-4.38E-09	-3.35E-09	-4.21E-09	-3.51E-09	-2.61E-09	-1.21E-09	-2.11E-09	-3.44E-09	-3.37E-09	-3.61E-09	-2.49E-09
-4.50E-09	-4.31E-09	-5.49E-09	-4.51E-09	-4.21E-09	-3.62E-09	-4.00E-09	-3.07E-09	-1.81E-09	-1.49E-09	-2.68E-09	-3.64E-09	-3.67E-09	-3.84E-09	-2.49E-09
-4.11E-09	-4.54E-09	-5.09E-09	-4.27E-09	-4.31E-09	-3.88E-09	-4.01E-09	-2.92E-09	-1.87E-09	-2.96E-09	-3.50E-09	-4.08E-09	-3.85E-09	-3.67E-09	-2.57E-09
-3.19E-09	-3.94E-09	-3.62E-09	-3.94E-09	-4.20E-09	-4.14E-09	-3.93E-09	-2.85E-09	-2.51E-09	-3.74E-09	-4.09E-09	-3.86E-09	-3.35E-09	-3.39E-09	-2.46E-09
-2.46E-09	-2.94E-09	-2.55E-09	-3.79E-09	-4.34E-09	-4.45E-09	-3.91E-09	-3.04E-09	-3.50E-09	-3.65E-09	-3.45E-09	-3.46E-09	-2.92E-09	-3.43E-09	-2.00E-09
-2.39E-09	-2.31E-09	-2.95E-09	-3.67E-09	-4.30E-09	-4.96E-09	-4.37E-09	-3.20E-09	-3.48E-09	-3.58E-09	-3.08E-09	-3.44E-09	-3.18E-09	-3.55E-09	-1.62E-09
-2.91E-09	-2.71E-09	-3.11E-09	-3.74E-09	-4.28E-09	-5.17E-09	-4.72E-09	-3.20E-09	-2.74E-09	-4.04E-09	-3.41E-09	-3.72E-09	-3.56E-09	-3.84E-09	-1.61E-09
-3.71E-09	-3.58E-09	-4.15E-09	-4.17E-09	-3.75E-09	-4.81E-09	-4.52E-09	-2.81E-09	-2.65E-09	-3.76E-09	-3.66E-09	-4.29E-09	-3.73E-09	-3.94E-09	-1.37E-09
-3.31E-09	-4.21E-09	-4.23E-09	-4.43E-09	-3.99E-09	-4.45E-09	-4.12E-09	-2.82E-09	-2.86E-09	-3.28E-09	-3.93E-09	-4.27E-09	-4.30E-09	-4.33E-09	-2.05E-09
-3.31E-09	-4.67E-09	-3.99E-09	-4.79E-09	-4.63E-09	-3.97E-09	-3.25E-09	-2.94E-09	-2.09E-09	-2.71E-09	-3.74E-09	-3.93E-09	-3.79E-09	-4.34E-09	-3.47E-09
-2.96E-09	-4.61E-09	-3.54E-09	-4.76E-09	-4.74E-09	-3.85E-09	-2.73E-09	-2.71E-09	-1.71E-09	-2.58E-09	-3.02E-09	-3.04E-09	-3.05E-09	-3.85E-09	-3.61E-09
-3.00E-09	-4.20E-09	-3.36E-09	-4.61E-09	-4.84E-09	-3.99E-09	-3.10E-09	-3.29E-09	-2.22E-09	-3.04E-09	-1.95E-09	-2.36E-09	-2.50E-09	-2.67E-09	-2.32E-09
-3.54E-09	-3.93E-09	-3.34E-09	-4.02E-09	-4.57E-09	-4.45E-09	-3.11E-09	-3.66E-09	-3.63E-09	-3.22E-09	-1.79E-09	-2.07E-09	-1.42E-09	-1.31E-09	-4.40E-10
-4.30E-09	-4.15E-09	-3.76E-09	-3.49E-09	-4.06E-09	-4.32E-09	-3.10E-09	-3.78E-09	-3.84E-09	-2.72E-09	-2.28E-09	-2.13E-09	-8.29E-10	1.56E-10	4.37E-10
-4.61E-09	-4.21E-09	-3.77E-09	-3.15E-09	-3.46E-09	-3.72E-09	-2.20E-09	-3.79E-09	-3.14E-09	-2.10E-09	-1.98E-09	-1.99E-09	-1.02E-09	-2.49E-10	-8.73E-10
-4.55E-09	-4.03E-09	-3.50E-09	-3.37E-09	-2.91E-09	-2.97E-09	-2.05E-09	-3.80E-09	-3.00E-09	-1.53E-09	-1.42E-09	-2.29E-09	-1.15E-09	-1.87E-09	-1.90E-09
-4.33E-09	-3.93E-09	-3.37E-09	-3.56E-09	-2.78E-09	-2.88E-09	-2.02E-09	-3.29E-09	-2.87E-09	-1.47E-09	-1.73E-09	-2.74E-09	-1.56E-09	-2.72E-09	-2.81E-09
-4.32E-09	-3.75E-09	-3.36E-09	-3.30E-09	-2.66E-09	-2.93E-09	-1.87E-09	-2.48E-09	-2.35E-09	-7.66E-10	-2.06E-09	-2.54E-09	-2.07E-09	-3.07E-09	-3.25E-09
-3.82E-09	-3.39E-09	-3.03E-09	-2.43E-09	-1.71E-09	-2.40E-09	-1.47E-09	-1.53E-09	-1.32E-09	-1.23E-09	-2.29E-09	-2.76E-09	-2.67E-09	-3.12E-09	-4.08E-09
-2.91E-09	-3.08E-09	-2.26E-09	-1.32E-09	-7.53E-10	-1.04E-09	-1.08E-09	-1.22E-09	-6.38E-10	-1.65E-09	-2.73E-09	-2.75E-09	-3.41E-09	-3.23E-09	-4.45E-09
-2.27E-09	-2.33E-09	-9.62E-10	-5.18E-11	-2.44E-11	-4.10E-11	-1.34E-09	-2.28E-09	-1.68E-09	-2.14E-09	-3.67E-09	-3.62E-09	-3.91E-09	-3.45E-09	-4.39E-09
-1.20E-09	-1.33E-09	1.30E-10	-1.24E-10	-2.28E-10	-5.12E-10	-1.79E-09	-3.15E-09	-2.65E-09	-4.02E-09	-4.12E-09	-4.31E-09	-4.14E-09	-3.52E-09	-4.38E-09
-2.50E-10	1.59E-10	4.21E-10	1.54E-10	-4.14E-10	-6.32E-10	-1.66E-09	-3.24E-09	-2.81E-09	-4.43E-09	-4.43E-09	-4.68E-09	-4.18E-09	-3.24E-09	-4.32E-09
1.18E-09	1.99E-09	2.01E-09	1.44E-09	2.28E-11	-8.57E-11	-1.76E-09	-2.43E-09	-3.14E-09	-4.47E-09	-4.78E-09	-5.03E-09	-4.49E-09	-3.16E-09	-4.15E-09

7.16E-16	7.25E-16	6.59E-16	6.16E-16	6.42E-16	6.41E-16	5.86E-16	6.17E-16	5.89E-16	5.71E-16	5.96E-16	5.55E-16	5.07E-16	4.79E-16	4.92E-16
6.62E-16	6.88E-16	6.10E-16	5.67E-16	5.84E-16	5.74E-16	5.57E-16	5.61E-16	5.50E-16	5.16E-16	5.23E-16	4.94E-16	4.47E-16	4.11E-16	4.34E-16
6.07E-16	6.18E-16	5.61E-16	5.15E-16	5.15E-16	5.00E-16	5.28E-16	5.23E-16	5.06E-16	4.60E-16	4.64E-16	4.31E-16	4.04E-16	3.82E-16	3.92E-16
5.45E-16	5.22E-16	5.22E-16	4.71E-16	4.67E-16	4.61E-16	4.87E-16	4.72E-16	4.52E-16	4.19E-16	4.12E-16	3.87E-16	3.68E-16	3.67E-16	3.77E-16
4.81E-16	4.52E-16	4.54E-16	4.14E-16	4.16E-16	4.30E-16	4.24E-16	3.99E-16	3.85E-16	3.76E-16	3.65E-16	3.41E-16	3.34E-16	3.46E-16	3.48E-16
4.27E-16	3.99E-16	3.95E-16	3.82E-16	3.82E-16	3.90E-16	3.74E-16	3.34E-16	3.21E-16	3.28E-16	3.11E-16	2.86E-16	3.03E-16	3.03E-16	2.78E-16
3.75E-16	3.52E-16	3.49E-16	3.43E-16	3.45E-16	3.54E-16	3.26E-16	2.75E-16	2.63E-16	2.87E-16	2.57E-16	2.41E-16	2.51E-16	2.23E-16	2.15E-16
3.33E-16	3.24E-16	3.08E-16	3.13E-16	3.08E-16	3.13E-16	2.81E-16	2.31E-16	2.23E-16	2.34E-16	2.09E-16	2.14E-16	2.02E-16	1.74E-16	1.75E-16
2.95E-16	3.03E-16	2.70E-16	2.69E-16	2.76E-16	2.64E-16	2.36E-16	1.99E-16	2.03E-16	2.07E-16	1.82E-16	1.83E-16	1.60E-16	1.47E-16	1.46E-16
2.71E-16	2.71E-16	2.48E-16	2.43E-16	2.53E-16	2.32E-16	2.11E-16	1.87E-16	1.89E-16	1.99E-16	1.60E-16	1.56E-16	1.40E-16	1.24E-16	1.30E-16
2.49E-16	2.41E-16	2.31E-16	2.19E-16	2.13E-16	2.00E-16	1.76E-16	1.69E-16	1.81E-16	1.80E-16	1.32E-16	1.31E-16	1.20E-16	1.06E-16	1.12E-16
2.25E-16	2.11E-16	2.28E-16	1.98E-16	1.84E-16	1.72E-16	1.58E-16	1.48E-16	1.79E-16	1.37E-16	1.12E-16	1.12E-16	9.41E-17	8.41E-17	8.00E-17
1.90E-16	1.93E-16	2.17E-16	1.81E-16	1.70E-16	1.53E-16	1.44E-16	1.28E-16	1.38E-16	1.01E-16	9.01E-17	9.44E-17	7.77E-17	6.84E-17	5.83E-17
1.75E-16	1.66E-16	1.90E-16	1.66E-16	1.54E-16	1.50E-16	1.35E-16	1.16E-16	1.03E-16	7.82E-17	6.58E-17	7.35E-17	6.37E-17	5.01E-17	4.88E-17
1.48E-16	1.45E-16	1.64E-16	1.50E-16	1.41E-16	1.46E-16	1.18E-16	9.55E-17	8.36E-17	6.19E-17	5.64E-17	5.36E-17	4.16E-17	3.67E-17	4.32E-17
1.20E-16	1.22E-16	1.42E-16	1.36E-16	1.31E-16	1.34E-16	9.84E-17	7.91E-17	6.91E-17	5.31E-17	5.26E-17	4.92E-17	2.97E-17	2.45E-17	4.27E-17
1.09E-16	1.00E-16	1.20E-16	1.13E-16	1.26E-16	1.21E-16	8.39E-17	6.97E-17	6.58E-17	5.12E-17	4.93E-17	4.65E-17	2.17E-17	1.92E-17	3.95E-17
1.09E-16	8.68E-17	1.06E-16	1.03E-16	1.15E-16	1.01E-16	7.03E-17	6.03E-17	5.66E-17	5.20E-17	4.16E-17	3.10E-17	1.66E-17	1.68E-17	1.48E-17
9.48E-17	8.08E-17	9.17E-17	1.01E-16	9.34E-17	7.66E-17	6.37E-17	5.51E-17	5.46E-17	5.07E-17	4.06E-17	2.22E-17	1.44E-17	1.87E-17	1.01E-17
8.00E-17	7.48E-17	7.41E-17	8.75E-17	7.24E-17	6.20E-17	5.70E-17	4.83E-17	5.17E-17	4.32E-17	3.71E-17	1.98E-17	1.44E-17	2.46E-17	9.36E-18
6.60E-17	7.18E-17	6.01E-17	7.35E-17	5.92E-17	5.09E-17	4.45E-17	3.77E-17	3.52E-17	2.62E-17	2.87E-17	1.83E-17	1.43E-17	2.52E-17	8.24E-18
4.82E-17	5.40E-17	4.47E-17	5.81E-17	4.70E-17	3.99E-17	3.01E-17	2.85E-17	2.22E-17	1.71E-17	1.71E-17	1.45E-17	1.45E-17	1.99E-17	7.65E-18
3.73E-17	4.16E-17	3.49E-17	4.35E-17	4.06E-17	3.46E-17	2.34E-17	2.42E-17	1.56E-17	1.08E-17	1.27E-17	1.02E-17	1.30E-17	1.80E-17	7.50E-18
3.17E-17	2.93E-17	2.85E-17	3.43E-17	3.28E-17	2.78E-17	2.08E-17	2.22E-17	1.12E-17	8.31E-18	8.44E-18	7.11E-18	1.00E-17	1.20E-17	6.95E-18
2.24E-17	1.74E-17	2.40E-17	2.61E-17	2.50E-17	2.06E-17	1.48E-17	1.75E-17	7.83E-18	4.91E-18	5.32E-18	5.73E-18	9.71E-18	7.42E-18	7.62E-18
1.21E-17	1.28E-17	1.93E-17	2.00E-17	2.27E-17	1.59E-17	1.02E-17	1.13E-17	5.17E-18	4.92E-18	3.15E-18	4.75E-18	8.19E-18	5.29E-18	7.47E-18
7.05E-18	8.28E-18	1.31E-17	1.41E-17	1.67E-17	1.15E-17	7.37E-18	7.65E-18	2.28E-18	3.61E-18	2.52E-18	3.36E-18	7.23E-18	6.38E-18	7.20E-18
4.19E-18	3.57E-18	6.55E-18	8.95E-18	1.02E-17	8.62E-18	5.66E-18	5.28E-18	1.03E-18	1.99E-18	1.98E-18	2.19E-18	7.41E-18	5.30E-18	4.40E-18
1.48E-18	1.44E-18	3.36E-18	3.92E-18	4.92E-18	6.58E-18	3.63E-18	2.51E-18	3.51E-19	4.90E-19	1.95E-18	1.61E-18	5.47E-18	3.91E-18	2.86E-18
2.98E-19	4.75E-19	9.36E-19	1.11E-18	2.07E-18	4.97E-18	3.07E-18	1.10E-18	2.48E-19	2.58E-19	2.45E-18	9.90E-19	2.94E-18	3.11E-18	2.39E-18
1.93E-19	2.86E-19	6.17E-19	8.11E-19	7.97E-19	2.46E-18	2.10E-18	3.04E-19	9.71E-20	5.33E-20	2.53E-18	6.47E-19	2.11E-18	2.95E-18	3.60E-18

2.85E-19	1.35E-19	9.09E-19	7.02E-19	6.90E-19	8.22E-19	3.46E-19	4.31E-21	2.14E-19	7.23E-22	1.22E-18	6.48E-19	1.73E-18	2.15E-18	4.26E-18
1.95E-19	1.08E-19	9.84E-19	9.71E-20	6.42E-19	8.33E-19	1.98E-20	4.16E-20	1.09E-19	6.33E-22	1.80E-18	9.74E-19	2.42E-18	2.39E-18	4.08E-18
6.60E-21	2.27E-21	1.59E-19	1.18E-19	6.45E-19	9.21E-19	2.25E-23	1.57E-20	3.82E-19	3.70E-19	2.59E-18	3.33E-18	2.94E-18	3.18E-18	2.45E-18
1.25E-19	3.63E-19	3.02E-19	1.88E-19	4.18E-19	2.14E-19	1.16E-20	3.60E-21	1.06E-19	2.15E-19	3.03E-18	4.53E-18	3.23E-18	2.74E-18	1.38E-18
5.67E-19	9.86E-19	4.70E-19	2.20E-19	2.39E-19	9.77E-22	9.00E-20	2.76E-19	1.11E-19	2.63E-20	1.65E-18	4.46E-18	3.08E-18	1.36E-18	1.31E-18
1.21E-18	6.57E-19	1.58E-19	5.99E-20	1.43E-19	7.41E-19	7.80E-19	7.15E-19	5.43E-19	1.18E-19	9.88E-19	2.95E-18	1.61E-18	4.08E-19	8.94E-19
3.50E-18	1.28E-18	8.85E-21	7.48E-19	2.87E-18	2.60E-18	1.07E-18	7.31E-19	2.05E-18	1.87E-19	9.38E-19	2.88E-18	2.16E-18	3.23E-19	1.73E-18
5.45E-18	2.99E-18	9.85E-19	3.32E-18	3.96E-18	3.05E-18	8.57E-19	5.92E-19	2.26E-18	4.53E-20	6.91E-19	2.70E-18	2.68E-18	1.16E-18	1.76E-18
6.64E-18	5.21E-18	5.16E-18	5.43E-18	3.21E-18	3.48E-18	7.03E-19	9.91E-19	1.31E-18	6.57E-20	2.13E-19	1.35E-18	2.66E-18	1.49E-18	1.25E-18
8.00E-18	7.12E-18	8.98E-18	7.99E-18	3.99E-18	5.73E-18	7.96E-19	1.36E-18	1.38E-18	2.48E-21	1.51E-19	6.69E-19	2.86E-18	3.73E-18	1.43E-18
1.53E-17	1.45E-17	1.62E-17	1.12E-17	7.63E-18	6.51E-18	1.26E-18	2.36E-18	3.14E-18	6.64E-19	2.18E-19	4.96E-19	1.26E-18	2.01E-18	1.50E-18
1.94E-17	1.20E-17	1.25E-17	9.37E-18	8.04E-18	6.53E-18	2.01E-18	1.77E-18	3.61E-18	1.34E-18	1.20E-19	5.87E-19	1.22E-18	1.71E-18	9.38E-19
1.71E-17	5.89E-18	7.82E-18	1.12E-17	9.05E-18	6.14E-18	1.83E-18	9.43E-19	1.30E-18	1.22E-18	7.93E-19	7.72E-19	1.77E-18	2.32E-18	5.96E-19
1.06E-17	3.33E-18	5.51E-18	8.96E-18	7.73E-18	5.13E-18	2.72E-18	1.14E-18	1.18E-18	1.08E-18	5.76E-19	7.03E-19	2.21E-18	5.15E-18	1.50E-18
7.22E-18	1.31E-18	3.43E-18	4.34E-18	4.10E-18	4.05E-18	4.67E-18	1.28E-18	2.13E-18	2.53E-19	1.99E-19	1.26E-18	1.87E-18	4.57E-18	1.41E-18
4.07E-18	4.76E-19	2.88E-18	1.80E-18	2.47E-18	3.13E-18	6.60E-18	1.29E-18	1.91E-18	4.14E-19	1.44E-19	1.85E-18	4.99E-19	3.23E-18	1.75E-18
2.99E-18	8.44E-19	3.48E-18	2.16E-18	1.56E-18	2.98E-18	6.52E-18	1.46E-18	1.12E-18	7.34E-19	1.58E-18	3.09E-18	3.20E-19	1.46E-18	1.40E-19
3.33E-18	3.18E-18	5.61E-18	5.00E-18	1.98E-18	6.60E-18	5.78E-18	2.00E-18	3.49E-19	6.64E-19	1.68E-18	2.95E-18	6.49E-19	8.60E-19	1.93E-20
5.96E-18	4.36E-18	4.47E-18	7.47E-18	4.76E-18	1.00E-17	8.01E-18	3.70E-18	2.73E-20	4.12E-19	2.35E-19	2.43E-19	6.36E-19	1.81E-18	3.30E-18
6.61E-18	5.63E-18	4.84E-18	1.22E-17	7.29E-18	1.22E-17	9.63E-18	5.35E-18	4.11E-20	1.07E-19	1.72E-18	3.55E-20	3.92E-19	9.83E-19	2.97E-18
1.36E-17	1.12E-17	7.84E-18	1.20E-17	1.21E-17	1.28E-17	1.38E-17	1.01E-17	1.83E-18	3.02E-18	3.03E-18	6.12E-19	3.47E-21	1.86E-19	1.09E-18
1.94E-17	1.52E-17	1.21E-17	1.01E-17	1.33E-17	1.20E-17	1.92E-17	1.34E-17	2.40E-18	2.64E-18	1.46E-18	1.22E-18	2.70E-19	1.64E-19	1.02E-19
2.32E-17	1.78E-17	1.47E-17	1.04E-17	1.41E-17	1.36E-17	1.70E-17	1.19E-17	1.16E-18	6.70E-19	4.12E-19	4.45E-19	3.31E-19	8.94E-19	1.95E-19
2.08E-17	1.48E-17	1.39E-17	7.40E-18	1.19E-17	8.88E-18	7.34E-18	7.54E-18	7.28E-19	6.86E-20	1.12E-19	1.42E-19	8.28E-19	7.55E-19	7.18E-19
2.00E-17	9.00E-18	6.00E-18	3.55E-18	5.92E-18	4.72E-18	2.42E-18	3.06E-18	1.53E-18	1.07E-18	2.13E-19	9.57E-20	1.06E-18	1.76E-19	3.36E-19
2.31E-17	5.19E-18	3.08E-18	1.62E-18	3.08E-18	2.28E-18	1.85E-18	1.32E-18	3.55E-18	4.22E-18	2.20E-18	7.80E-19	2.22E-18	2.00E-19	1.71E-19
2.26E-17	4.05E-18	4.84E-18	1.91E-18	1.33E-18	3.59E-18	2.79E-18	2.00E-18	3.39E-18	6.77E-18	4.24E-18	2.32E-18	5.56E-18	1.22E-18	9.12E-19
2.06E-17	9.41E-18	1.08E-17	3.45E-18	2.54E-18	6.91E-18	6.18E-18	2.95E-18	3.74E-18	9.60E-18	5.64E-18	3.17E-18	6.88E-18	3.54E-18	1.31E-18
1.52E-17	1.31E-17	1.46E-17	3.08E-18	2.77E-18	8.36E-18	3.37E-18	2.95E-18	6.67E-18	1.18E-17	7.03E-18	2.27E-18	6.38E-18	3.52E-18	1.21E-18
7.49E-18	1.13E-17	1.50E-17	5.08E-18	4.80E-18	7.19E-18	2.02E-18	2.76E-18	8.88E-18	9.87E-18	5.57E-18	2.18E-18	6.55E-18	3.42E-18	4.78E-19
4.99E-18	9.15E-18	1.77E-17	1.51E-17	7.13E-18	1.00E-17	3.25E-18	2.50E-18	4.70E-18	5.31E-18	3.19E-18	1.74E-18	4.32E-18	1.83E-18	5.94E-21
5.80E-18	1.28E-17	2.23E-17	2.02E-17	9.29E-18	1.13E-17	1.19E-18	3.44E-19	1.36E-18	1.63E-18	1.26E-18	1.63E-18	1.59E-18	6.36E-19	1.36E-19
3.95E-18	1.27E-17	2.56E-17	2.27E-17	1.34E-17	9.69E-18	2.18E-18	1.41E-19	4.25E-24	2.31E-19	3.87E-19	6.72E-19	3.83E-20	3.68E-20	8.46E-21
3.65E-18	1.25E-17	2.92E-17	2.21E-17	1.28E-17	5.37E-18	2.25E-18	1.79E-20	8.04E-20	2.97E-19	1.66E-19	6.69E-19	3.08E-19	2.20E-20	2.82E-20
5.68E-18	1.11E-17	1.80E-17	2.07E-17	1.10E-17	3.81E-18	2.36E-19	1.90E-20	3.62E-20	1.50E-18	1.05E-18	2.22E-18	8.58E-20	7.13E-22	5.54E-19
6.91E-18	1.21E-17	1.86E-17	2.10E-17	1.34E-17	1.80E-18	1.47E-20	9.72E-20	2.47E-20	3.35E-18	3.20E-18	4.37E-18	2.03E-19	3.87E-19	9.03E-19
1.84E-17	2.16E-17	2.60E-17	2.37E-17	1.14E-17	9.40E-19	2.16E-20	5.62E-21	8.05E-19	4.37E-18	4.83E-18	4.65E-18	1.90E-18	4.58E-19	3.40E-20
3.24E-17	3.48E-17	3.43E-17	2.46E-17	1.20E-17	7.50E-19	4.88E-20	2.91E-20	4.41E-18	5.49E-18	5.42E-18	2.98E-18	2.70E-18	3.64E-21	1.03E-19
3.47E-17	4.07E-17	3.62E-17	2.66E-17	1.45E-17	3.24E-18	7.88E-19	1.45E-18	6.56E-18	2.87E-18	4.87E-18	3.36E-18	3.64E-18	4.23E-19	1.07E-19
3.80E-17	4.07E-17	3.25E-17	2.81E-17	1.77E-17	1.38E-17	3.85E-18	2.67E-18	2.54E-18	1.09E-18	4.64E-18	5.32E-18	3.54E-18	2.85E-18	9.86E-19
3.95E-17	3.52E-17	2.55E-17	2.42E-17	1.65E-17	1.74E-17	8.78E-18	1.81E-18	2.44E-20	1.14E-18	2.35E-18	4.58E-18	4.15E-18	5.33E-18	3.53E-18
3.90E-17	3.74E-17	1.90E-17	1.62E-17	1.18E-17	1.63E-17	1.20E-17	3.49E-18	1.44E-18	3.42E-18	2.68E-18	5.13E-18	6.18E-18	5.18E-18	3.09E-18
3.17E-17	3.65E-17	1.76E-17	1.13E-17	1.01E-17	1.76E-17	8.36E-18	4.69E-18	8.17E-18	1.24E-17	6.95E-18	4.09E-18	7.43E-18	5.13E-18	2.66E-18
2.22E-17	3.12E-17	1.79E-17	1.45E-17	1.25E-17	1.91E-17	7.91E-18	5.07E-18	1.34E-17	1.79E-17	9.80E-18	4.37E-18	7.48E-18	4.77E-18	2.52E-18
2.17E-17	2.47E-17	1.64E-17	9.49E-18	1.20E-17	2.03E-17	9.76E-18	4.38E-18	1.17E-17	1.71E-17	9.84E-18	4.04E-18	6.58E-18	3.96E-18	1.72E-18
2.48E-17	2.18E-17	1.10E-17	4.26E-18	1.15E-17	2.00E-17	1.04E-17	6.39E-18	1.23E-17	1.43E-17	8.73E-18	2.63E-18	3.06E-18	2.42E-18	1.60E-18
2.15E-17	2.20E-17	1.06E-17	8.16E-18	1.40E-17	1.88E-17	7.43E-18	7.17E-18	1.13E-17	1.05E-17	7.22E-18	2.21E-18	1.46E-18	2.28E-18	1.32E-18
2.29E-17	2.29E-17	1.38E-17	2.08E-17	1.77E-17	1.27E-17	6.81E-18	1.05E-17	8.53E-18	7.98E-18	3.87E-18	1.92E-18	2.22E-19	5.52E-19	1.23E-18
2.61E-17	2.21E-17	1.64E-17	2.22E-17	1.72E-17	1.03E-17	1.12E-17	1.41E-17	5.44E-18	3.67E-18	1.53E-18	8.07E-19	2.02E-19	1.72E-22	3.88E-19
2.44E-17	1.99E-17	1.64E-17	2.25E-17	2.07E-17	1.50E-17	1.90E-17	1.46E-17	2.33E-18	9.93E-19	1.90E-19	1.95E-19	1.66E-18	2.37E-19	3.83E-19
1.84E-17	2.18E-17	1.82E-17	2.21E-17	2.04E-17	1.54E-17	2.05E-17	9.96E-18	1.96E-19	1.09E-18	7.28E-19	3.48E-20	2.00E-18	2.94E-19	4.40E-21
1.42E-17	1.85E-17	1.59E-17	2.08E-17	1.76E-17	9.54E-18	1.94E-17	4.76E-18	1.60E-18	1.53E-17	7.05E-18	2.04E-19	8.37E-19	3.74E-20	2.03E-21
1.17E-17	1.70E-17	2.01E-17	2.03E-17	1.82E-17	1.07E-17	1.54E-17	1.39E-18	4.88E-18	3.10E-17	1.50E-17	6.43E-19	1.16E-19	4.97E-19	1.37E-20
1.10E-17	1.85E-17	2.39E-17	2.20E-17	1.50E-17	7.65E-18	8.76E-18	1.78E-19	2.04E-18	1.72E-17	8.15E-18	3.59E-20	1.56E-18	2.00E-18	1.56E-20
1.21E-17	1.37E-17	1.79E-17	1.53E-17	1.59E-17	7.73E-18	4.08E-18	2.20E-18	6.74E-20	2.32E-18	9.32E-19	4.14E-19	3.44E-18	3.40E-18	5.08E-19
8.90E-18	8.38E-18	1.00E-17	6.82E-18	1.20E-17	1.05E-17	5.21E-18	5.74E-18	1.63E-18	8.59E-19	1.52E-18	4.44E-18	3.77E-18	1.61E-18	2.03E-18
3.87E-18	6.56E-18	8.92E-18	2.60E-18	6.69E-18	8.07E-18	8.09E-18	9.64E-18	6.29E-18	4.48E-18	4.76E-18	6.45E-18	2.36E-18	3.59E-19	2.35E-18
2.84E-18	1.10E-17	1.28E-17	2.04E-18	4.27E-18	6.07E-18	7.45E-18	1.07E-17	8.97E-18	5.43E-18	4.46E-18	4.09E-18	2.92E-18	4.70E-20	2.53E-18
3.77E-18	1.07E-17	1.62E-17	6.58E-18	5.43E-18	4.98E-18	6.98E-18	1.25E-17	9.74E-18	4.10E-18	3.21E-18	9.02E-19	1.36E-18	1.30E-18	3.26E-18
4.61E-18	8.48E-18	1.27E-17	1.04E-17	1.00E-17	4.83E-18	5.02E-18	1.04E-17	8.11E-18	3.36E-18	2.31E-18	8.00E-19	1.30E-18	5.00E-18	3.69E-18
6.35E-18	9.09E-18	6.70E-18	8.30E-18	8.68E-18	2.49E-18	1.71E-18	7.62E-18	7.15E-18	1.60E-18	3.67E-18	2.68E-18	2.94E-18	6.06E-18	3.30E-18
1.31E-17	1.26E-17	6.95E-18	4.87E-18	6.49E-18	3.13E-18	1.69E-19	3.51E-18	6.92E-18	4.97E-18	5.12E-18	7.74E-18	5.52E-18	4.90E-18	2.50E-18
1.61E-17	1.60E-17	9.95E-18	3.18E-18	4.10E-18	3.21E-18	2.62E-21	2.89E-18	8.50E-18	1.17E-17	6.63E-18	6.78E-18	6.78E-18	2.93E-18	1.66E-18
2.18E-17	2.18E-17	1.41E-17	3.31E-18	4.59E-18	4.64E-18	1.32E-18	3.92E-18	1.17E-17	1.12E-17	9.78E-18	4.71E-18	5.09E-18	1.95E-18	1.11E-18
2.37E-17	2.52E-17	1.94E-17	1.09E-17	1.35E-17	1.09E-17	9.02E-18	7.56E-18	1.07E-17	1.20E-17	1.05E-17	3.74E-18	2.10E-18	1.55E-18	1.66E-18
2.15E-17	2.77E-17	2.04E-17	2.06E-17	2.51E-17	1.51E-17	1.31E-17	7.40E-18	7.08E-18	9.57E-18	6.86E-18	2.42E-18	2.85E-18	1.60E-18	1.34E-18
2.06E-17	2.51E-17	2.32E-17	2.60E-17	2.74E-17	1.61E-17	1.38E-17	6.07E-18	2.68E-18	7.62E-18	4.15E-18	2.68E-18	5.73E-18	4.81E-18	3.52E-18
2.06E-17	2.06E-17	2.17E-17	2.50E-17	2.63E-17	2.12E-17	1.48E-17	6.59E-18	2.25E-18	5.99E-18	2.46E-18	2.92E-18	6.16E-18	7.71E-18	5.43E-18
1.51E-17	9.43E-18	1.83E-17	2.31E-17	2.26E-17	2.53E-17	1.87E-17	1.02E-17	5.14E-18	4.62E-18	2.87E-18	2.68E-18	3.02E-18	4.00E-18	2.73E-18
1.13E-17	4.08E-18	1.24E-17	2.14E-17	2.05E-17	2.23E-17	2.00E-17	1.42E-17	1.01E-17	4.01E-18	4.05E-18	1.93E-18	2.01E-19	3.05E-19	1.23E-18
8.47E-18	4.11E-18	8.85E-18	1.61E-17	2.29E-17	2.07E-17	1.96E-17	1.60E-17	1.41E-17	6.20E-18	4.94E-18	2.37E-18	5.18E-20	2.64E-19	3.83E-19
7.63E-18	6.08E-18	1.02E-17	1.27E-17	2.36E-17	1.91E-17	2.00E-17	1.64E-17	1.48E-17	7.43E-18	8.73E-18	7.50E-18	1.77E-18	6.30E-22	7.52E-19
9.16E-18	7.83E-18	1.89E-17	1.51E-17	2.21E-17	1.32E-17	1.79E-17	1.46E-17	1.14E-17	4.44E-18	8.47E-18	1.01E-17	6.09E-18	3.14E-18	4.39E-18
1.47E-17	1.26E-17	2.61E-17	1.83E-17	1.92E-17	1.12E-17	1.77E-17	1.23E-17	6.84E-18	1.47E-18	4.45E-18	1.18E-17	1.13E-17	1.30E-17	6.18E-18
2.02E-17	1.86E-17	3.02E-17	2.03E-17	1.77E-17	1.31E-17	1.60E-17	9.44E-18	3.26E-18	2.23E-18	7.17E-18	1.33E-17	1.35E-17	1.48E-17	6.20E-18
1.69E-17	2.06E-17	2.59E-17	1.82E-17	1.85E-17	1.51E-17	1.61E-17	8.55E-18	3.51E-18	8.79E-18	1.22E-17	1.67E-17	1.49E-17	1.35E-17	6.61E-18
1.02E-17	1.55E-17	1.31E-17	1.55E-17	1.76E-17	1.72E-17	1.54E-17	8.12E-18	6.30E-18	1.40E-17	1.67E-17	1.49E-17	1.12E-17	1.15E-17	6.07E-18
6.03E-18	8.64E-18	6.48E-18	1.44E-17	1.88E-17	1.98E-17	1.53E-17	9.26E-18	1.23E-17	1.34E-17	1.19E-17	1.20E-17	8.54E-18	1.18E-17	4.00E-18
5.73E-18	5.32E-18	8.72E-18	1.35E-17	1.85E-17	2.46E-17	1.91E-17	1.03E-17	1.21E-17	1.28E-17	9.51E-18	1.18E-17	1.01E-17	1.26E-17	2.64E-18
8.46E-18	7.34E-18	9.66E-18	1.40E-17	1.83E-17	2.67E-17	2.23E-17	1.02E-17	7.49E-18	1.64E-17	1.16E-17	1.39E-17	1.26E-17	1.47E-17	2.58E-18
1.38E-17	1.28E-17	1.72E-17	1.74E-17	1.40E-17	2.31E-17	2.04E-17	7.92E-18	7.02E-18	1.42E-17	1.34E-17	1.84E-17	1.39E-17	1.55E-17	1.87E-18
1.10E-17	1.78E-17	1.79E-17	1.96E-17	1.59E-17	1.98E-17	1.70E-17	7.98E-18	8.19E-18	1.08E-17	1.55E-17	1.82E-17	1.85E-17	1.88E-17	4.18E-18
1.10E-17	2.18E-17	1.59E-17	2.29E-17	2.14E-17	1.58E-17	1.06E-17	8.62E-18	4.37E-18	7.33E-18	1.40E-17	1.55E-17	1.44E-17	1.89E-17	1.20E-17
8.76E-18	2.13E-17	1.25E-17	2.27E-17	2.25E-17	1.48E-17	7.45E-18	7.35E-18	2.93E-18	6.64E-18	9.11E-18	9.24E-18	9.30E-18	1.48E-17	1.30E-17
9.01E-18	1.76E-17	1.13E-17	2.13E-17	2.34E-17	1.59E-17	9.59E-18	1.08E-17	4.91E-18	9.26E-18	3.82E-18	5.57E-18	6.26E-18	7.11E-18	5.38E-18
1.25E-17	1.54E-17	1.11E-17	1.62E-17	2.09E-17	1.98E-17	9.68E-18	1.34E-17	1.32E-17	1.04E-17	3.19E-18	4.30E-18	2.02E-18	1.71E-18	1.93E-19

1.85E-17	1.72E-17	1.41E-17	1.22E-17	1.65E-17	1.86E-17	9.63E-18	1.43E-17	1.47E-17	7.42E-18	5.22E-18	4.52E-18	6.88E-19	2.44E-20	1.91E-19
2.12E-17	1.77E-17	1.42E-17	9.95E-18	1.20E-17	1.38E-17	4.85E-18	1.44E-17	9.84E-18	4.40E-18	3.93E-18	3.96E-18	1.04E-18	6.19E-20	7.63E-19
2.07E-17	1.62E-17	1.23E-17	1.14E-17	8.46E-18	8.81E-18	4.22E-18	1.44E-17	9.01E-18	2.33E-18	2.02E-18	5.25E-18	1.32E-18	3.48E-18	3.60E-18
1.87E-17	1.54E-17	1.13E-17	1.27E-17	7.76E-18	8.28E-18	4.08E-18	1.08E-17	8.23E-18	2.15E-18	2.99E-18	7.48E-18	2.42E-18	7.37E-18	7.89E-18
1.86E-17	1.41E-17	1.13E-17	1.09E-17	7.07E-18	8.61E-18	3.50E-18	6.17E-18	5.54E-18	5.87E-19	4.23E-18	6.45E-18	4.29E-18	9.40E-18	1.05E-17
1.46E-17	1.15E-17	9.15E-18	5.92E-18	2.91E-18	5.76E-18	2.16E-18	2.33E-18	1.75E-18	1.51E-18	5.24E-18	7.62E-18	7.12E-18	9.71E-18	1.67E-17
8.45E-18	9.46E-18	5.10E-18	1.74E-18	5.68E-19	1.09E-18	1.16E-18	1.48E-18	4.07E-19	2.71E-18	7.48E-18	7.55E-18	1.16E-17	1.04E-17	1.98E-17
5.15E-18	5.43E-18	9.26E-19	2.68E-21	5.96E-22	1.68E-21	1.79E-18	5.18E-18	2.83E-18	4.57E-18	1.35E-17	1.31E-17	1.53E-17	1.19E-17	1.93E-17
1.43E-18	1.78E-18	1.68E-20	1.55E-20	5.18E-20	2.63E-19	3.20E-18	9.90E-18	7.02E-18	1.62E-17	1.70E-17	1.86E-17	1.72E-17	1.24E-17	1.92E-17
6.26E-20	2.52E-20	1.77E-19	2.38E-20	1.71E-19	3.99E-19	2.76E-18	1.05E-17	7.90E-18	1.96E-17	1.96E-17	2.19E-17	1.75E-17	1.05E-17	1.87E-17
1.38E-18	3.96E-18	4.03E-18	2.06E-18	5.21E-22	7.34E-21	3.10E-18	5.89E-18	9.87E-18	2.00E-17	2.29E-17	2.53E-17	2.02E-17	9.98E-18	1.72E-17
7.74E-15	7.61E-15	7.43E-15	7.02E-15	6.91E-15	6.66E-15	6.01E-15	5.49E-15	5.24E-15	5.04E-15	4.74E-15	4.46E-15	4.14E-15	3.92E-15	3.82E-15

2.28E-08	2.26E-08	2.10E-08	2.05E-08	1.90E-08	1.89E-08	1.84E-08	1.87E-08	1.87E-08	1.72E-08	1.58E-08	1.56E-08	1.43E-08	1.38E-08	1.28E-08	1.25E-08
2.14E-08	2.10E-08	1.97E-08	1.92E-08	1.79E-08	1.74E-08	1.71E-08	1.79E-08	1.73E-08	1.57E-08	1.48E-08	1.43E-08	1.37E-08	1.29E-08	1.16E-08	1.19E-08
1.99E-08	1.92E-08	1.81E-08	1.77E-08	1.62E-08	1.63E-08	1.61E-08	1.65E-08	1.53E-08	1.46E-08	1.40E-08	1.34E-08	1.28E-08	1.21E-08	1.09E-08	1.16E-08
1.83E-08	1.72E-08	1.65E-08	1.63E-08	1.52E-08	1.54E-08	1.54E-08	1.53E-08	1.41E-08	1.39E-08	1.30E-08	1.25E-08	1.20E-08	1.11E-08	1.03E-08	1.13E-08
1.72E-08	1.61E-08	1.53E-08	1.55E-08	1.48E-08	1.46E-08	1.49E-08	1.44E-08	1.32E-08	1.29E-08	1.22E-08	1.19E-08	1.13E-08	9.70E-09	9.15E-09	1.03E-08
1.54E-08	1.50E-08	1.42E-08	1.46E-08	1.41E-08	1.39E-08	1.39E-08	1.35E-08	1.26E-08	1.17E-08	1.15E-08	1.06E-08	9.78E-09	8.67E-09	8.00E-09	8.84E-09
1.40E-08	1.34E-08	1.33E-08	1.36E-08	1.35E-08	1.31E-08	1.31E-08	1.24E-08	1.18E-08	1.07E-08	1.06E-08	9.67E-09	8.63E-09	7.99E-09	7.29E-09	7.89E-09
1.30E-08	1.20E-08	1.21E-08	1.29E-08	1.27E-08	1.23E-08	1.23E-08	1.14E-08	1.10E-08	1.01E-08	9.27E-09	9.32E-09	8.23E-09	7.52E-09	6.72E-09	7.04E-09
1.18E-08	1.06E-08	1.11E-08	1.17E-08	1.17E-08	1.07E-08	1.12E-08	1.04E-08	9.80E-09	9.48E-09	8.32E-09	9.01E-09	8.28E-09	7.74E-09	6.22E-09	6.37E-09
1.06E-08	9.78E-09	1.04E-08	1.06E-08	1.14E-08	9.85E-09	1.01E-08	9.53E-09	9.05E-09	9.07E-09	7.72E-09	8.86E-09	8.49E-09	7.81E-09	6.03E-09	5.77E-09
9.53E-09	9.21E-09	1.00E-08	9.84E-09	9.62E-09	9.17E-09	9.09E-09	8.19E-09	7.73E-09	8.35E-09	7.58E-09	9.12E-09	8.87E-09	7.68E-09	5.48E-09	5.28E-09
8.26E-09	8.90E-09	9.83E-09	8.95E-09	8.61E-09	8.22E-09	8.07E-09	6.83E-09	6.47E-09	7.27E-09	6.81E-09	8.25E-09	8.45E-09	7.15E-09	5.09E-09	5.11E-09
7.28E-09	8.51E-09	9.40E-09	8.26E-09	7.87E-09	7.28E-09	6.88E-09	5.99E-09	5.52E-09	6.04E-09	5.45E-09	6.65E-09	7.24E-09	6.39E-09	4.85E-09	5.00E-09
6.46E-09	8.04E-09	7.94E-09	7.40E-09	7.20E-09	6.62E-09	6.45E-09	5.55E-09	5.17E-09	5.33E-09	4.41E-09	5.04E-09	5.26E-09	5.84E-09	4.80E-09	4.79E-09
5.69E-09	6.63E-09	6.42E-09	6.57E-09	6.33E-09	6.36E-09	6.03E-09	5.15E-09	4.63E-09	4.37E-09	4.05E-09	4.25E-09	4.17E-09	5.16E-09	4.31E-09	4.24E-09
4.84E-09	5.38E-09	5.70E-09	6.31E-09	5.92E-09	6.12E-09	5.75E-09	4.45E-09	3.58E-09	3.57E-09	4.12E-09	3.97E-09	3.91E-09	4.26E-09	3.74E-09	3.96E-09
4.41E-09	4.86E-09	5.87E-09	6.13E-09	6.15E-09	6.33E-09	5.53E-09	3.98E-09	3.18E-09	2.96E-09	5.57E-09	4.08E-09	3.68E-09	3.61E-09	3.43E-09	3.62E-09
4.46E-09	4.32E-09	5.18E-09	5.06E-09	5.63E-09	6.02E-09	4.97E-09	4.01E-09	3.08E-09	2.33E-09	8.13E-09	4.67E-09	4.05E-09	3.66E-09	3.29E-09	3.07E-09
4.30E-09	3.73E-09	4.07E-09	3.55E-09	4.48E-09	4.59E-09	4.08E-09	3.85E-09	3.22E-09	3.42E-09	1.10E-08	5.31E-09	4.43E-09	3.72E-09	3.21E-09	3.25E-09
3.82E-09	3.03E-09	3.29E-09	2.82E-09	3.21E-09	3.54E-09	3.34E-09	2.99E-09	2.70E-09	3.54E-09	5.65E-09	5.01E-09	4.15E-09	3.31E-09	2.90E-09	3.36E-09
3.56E-09	2.87E-09	2.88E-09	2.17E-09	2.63E-09	2.78E-09	2.53E-09	2.41E-09	2.35E-09	2.91E-09	3.57E-09	4.34E-09	3.14E-09	2.75E-09	2.60E-09	3.60E-09
2.87E-09	2.84E-09	2.93E-09	2.10E-09	2.46E-09	2.41E-09	1.83E-09	1.98E-09	1.83E-09	2.40E-09	2.75E-09	4.04E-09	2.12E-09	2.52E-09	2.59E-09	3.98E-09
2.66E-09	2.57E-09	3.69E-09	3.46E-09	3.45E-09	1.97E-09	1.90E-09	2.12E-09	1.68E-09	1.96E-09	2.46E-09	3.49E-09	2.33E-09	2.88E-09	2.55E-09	3.38E-09
2.56E-09	2.15E-09	4.75E-09	5.23E-09	4.54E-09	1.50E-09	1.62E-09	1.70E-09	1.79E-09	2.35E-09	2.09E-09	2.93E-09	2.77E-09	2.40E-09	2.72E-09	2.88E-09
2.56E-09	1.98E-09	3.62E-09	4.16E-09	4.32E-09	1.38E-09	1.64E-09	1.80E-09	1.73E-09	2.78E-09	1.88E-09	2.46E-09	2.67E-09	2.60E-09	3.00E-09	3.08E-09
2.39E-09	2.10E-09	2.42E-09	1.90E-09	2.09E-09	1.41E-09	1.72E-09	1.61E-09	1.70E-09	3.15E-09	1.93E-09	2.38E-09	2.37E-09	3.02E-09	3.67E-09	3.33E-09
2.35E-09	1.92E-09	2.38E-09	1.66E-09	1.61E-09	1.67E-09	2.17E-09	1.86E-09	2.24E-09	3.13E-09	1.99E-09	2.43E-09	2.30E-09	3.33E-09	3.64E-09	3.55E-09
2.50E-09	2.10E-09	2.39E-09	1.42E-09	1.34E-09	1.76E-09	2.72E-09	1.99E-09	2.17E-09	3.25E-09	2.37E-09	2.61E-09	2.83E-09	3.78E-09	3.52E-09	3.57E-09
3.11E-09	2.38E-09	2.35E-09	1.06E-09	1.13E-09	1.78E-09	2.72E-09	2.09E-09	2.05E-09	3.30E-09	2.48E-09	2.75E-09	3.30E-09	3.98E-09	3.30E-09	3.37E-09
3.81E-09	3.08E-09	2.72E-09	1.02E-09	1.15E-09	1.75E-09	2.07E-09	1.89E-09	1.63E-09	2.82E-09	2.34E-09	2.57E-09	2.93E-09	3.42E-09	3.29E-09	3.40E-09
3.46E-09	3.20E-09	2.71E-09	1.08E-09	1.72E-09	1.48E-09	1.47E-09	2.12E-09	1.73E-09	2.38E-09	2.24E-09	2.12E-09	2.40E-09	2.82E-09	3.52E-09	3.66E-09
2.52E-09	2.85E-09	2.46E-09	1.68E-09	2.41E-09	1.68E-09	1.52E-09	2.65E-09	2.22E-09	2.25E-09	2.08E-09	2.29E-09	2.33E-09	2.54E-09	3.72E-09	3.66E-09
2.49E-09	3.00E-09	2.69E-09	2.37E-09	2.81E-09	1.99E-09	1.80E-09	3.30E-09	2.88E-09	2.36E-09	2.27E-09	2.62E-09	2.41E-09	2.88E-09	3.78E-09	3.67E-09
1.81E-09	2.60E-09	2.60E-09	2.60E-09	2.43E-09	2.21E-09	2.33E-09	3.97E-09	3.54E-09	2.65E-09	2.64E-09	3.03E-09	2.85E-09	3.19E-09	3.80E-09	3.89E-09
1.69E-09	2.05E-09	1.87E-09	2.11E-09	2.09E-09	2.50E-09	2.64E-09	3.98E-09	4.06E-09	3.31E-09	3.08E-09	2.95E-09	2.67E-09	3.04E-09	4.76E-09	4.08E-09
1.69E-09	1.85E-09	1.40E-09	1.85E-09	1.86E-09	2.39E-09	2.55E-09	3.30E-09	4.01E-09	3.65E-09	3.36E-09	3.17E-09	2.88E-09	3.22E-09	5.12E-09	4.24E-09
1.77E-09	1.57E-09	1.27E-09	2.00E-09	1.65E-09	2.25E-09	2.58E-09	2.52E-09	3.78E-09	4.00E-09	3.52E-09	3.49E-09	3.17E-09	3.30E-09	4.72E-09	4.16E-09
1.86E-09	1.42E-09	1.13E-09	1.75E-09	1.45E-09	2.19E-09	2.18E-09	2.27E-09	2.97E-09	4.31E-09	3.34E-09	3.90E-09	3.43E-09	3.08E-09	4.40E-09	4.12E-09
1.81E-09	1.19E-09	9.77E-10	1.38E-09	1.19E-09	2.16E-09	2.07E-09	2.25E-09	2.64E-09	4.20E-09	3.47E-09	3.54E-09	3.42E-09	3.24E-09	3.92E-09	4.08E-09
1.25E-09	6.74E-10	8.11E-10	1.31E-09	1.19E-09	2.11E-09	1.35E-09	2.05E-09	2.43E-09	3.57E-09	3.74E-09	3.40E-09	3.18E-09	3.57E-09	3.20E-09	4.05E-09
8.48E-10	3.99E-10	7.51E-10	1.09E-09	9.02E-10	1.97E-09	8.95E-10	1.36E-09	1.75E-09	3.37E-09	3.25E-09	3.26E-09	2.97E-09	3.41E-09	3.07E-09	3.56E-09
6.92E-10	5.21E-10	8.23E-10	6.81E-10	1.91E-10	1.41E-09	1.02E-09	1.17E-09	1.67E-09	2.82E-09	2.77E-09	2.98E-09	2.90E-09	3.44E-09	3.40E-09	3.41E-09
1.21E-09	8.10E-10	1.01E-09	5.23E-10	-8.47E-11	5.87E-10	1.02E-09	1.23E-09	2.22E-09	2.44E-09	2.76E-09	2.69E-09	2.98E-09	3.73E-09	3.86E-09	3.75E-09
1.19E-09	1.02E-09	9.67E-10	6.72E-10	9.38E-13	3.46E-10	9.97E-10	1.45E-09	2.51E-09	2.30E-09	2.90E-09	2.34E-09	3.27E-09	3.68E-09	4.06E-09	4.09E-09
9.14E-10	1.17E-09	1.28E-09	8.61E-10	1.15E-10	8.78E-10	1.18E-09	1.15E-09	2.08E-09	2.28E-09	2.92E-09	2.14E-09	3.30E-09	3.63E-09	4.26E-09	3.77E-09
8.42E-10	1.49E-09	2.10E-09	9.27E-10	1.02E-10	1.25E-09	1.63E-09	1.71E-09	2.19E-09	2.40E-09	2.70E-09	2.05E-09	2.78E-09	3.41E-09	4.16E-09	3.31E-09
1.25E-09	2.13E-09	2.43E-09	8.08E-10	3.12E-10	1.19E-09	2.09E-09	2.34E-09	2.51E-09	2.72E-09	2.61E-09	1.87E-09	2.09E-09	2.80E-09	4.13E-09	3.05E-09
1.06E-09	2.69E-09	2.25E-09	7.85E-10	4.47E-10	9.51E-10	1.84E-09	2.30E-09	3.03E-09	3.34E-09	3.06E-09	2.16E-09	2.34E-09	2.62E-09	4.39E-09	3.32E-09
1.44E-09	3.40E-09	1.99E-09	9.67E-10	8.45E-10	8.25E-10	1.88E-09	2.33E-09	3.52E-09	3.12E-09	3.05E-09	2.67E-09	2.70E-09	2.72E-09	4.45E-09	4.08E-09
2.62E-09	3.94E-09	1.91E-09	1.36E-09	1.27E-09	1.44E-09	1.97E-09	2.53E-09	4.43E-09	3.16E-09	3.07E-09	3.13E-09	3.08E-09	3.05E-09	4.28E-09	4.17E-09
1.64E-09	3.08E-09	1.51E-09	1.84E-09	1.24E-09	1.57E-09	1.65E-09	2.15E-09	3.56E-09	2.66E-09	3.24E-09	3.28E-09	3.45E-09	3.56E-09	4.05E-09	3.40E-09
7.07E-10	1.93E-09	8.26E-10	1.49E-09	8.74E-10	1.24E-09	1.10E-09	1.58E-09	2.85E-09	2.60E-09	3.51E-09	3.31E-09	4.06E-09	4.08E-09	4.11E-09	2.99E-09
2.95E-10	1.01E-09	2.91E-10	1.08E-09	3.97E-10	8.36E-10	1.39E-09	7.21E-10	2.23E-09	3.02E-09	4.06E-09	3.46E-09	4.05E-09	4.03E-09	4.00E-09	2.92E-09
-2.27E-10	5.91E-10	6.68E-11	6.39E-11	1.86E-10	1.08E-10	1.48E-09	2.03E-10	1.85E-09	2.84E-09	4.52E-09	3.58E-09	3.61E-09	4.21E-09	3.54E-09	2.90E-09
-4.16E-10	-2.26E-10	8.83E-11	-6.23E-10	-2.88E-10	-2.58E-10	1.07E-09	-1.09E-10	6.89E-10	2.21E-09	3.34E-09	3.31E-09	3.08E-09	3.82E-09	3.18E-09	3.15E-09
-3.74E-10	-3.55E-10	2.61E-10	-9.39E-10	-1.88E-10	-2.21E-10	9.06E-10	-3.52E-10	-1.55E-10	1.74E-09	1.44E-09	2.54E-09	2.61E-09	3.56E-09	3.16E-09	3.53E-09
5.47E-11	-3.45E-10	4.98E-10	-5.23E-10	4.52E-10	-1.86E-10	5.14E-10	-3.55E-10	-6.49E-11	1.55E-09	7.65E-10	1.90E-09	2.15E-09	3.09E-09	3.33E-09	3.33E-09
-4.11E-10	-1.37E-10	7.70E-10	1.14E-10	1.04E-09	1.33E-10	6.83E-10	2.19E-10	2.89E-10	1.28E-09	6.58E-10	1.41E-09	1.99E-09	3.43E-09	3.15E-09	2.77E-09
-1.49E-09	-4.56E-10	1.13E-10	9.18E-11	1.00E-09	8.49E-10	1.28E-09	3.49E-10	4.07E-10	1.67E-09	8.87E-10	1.01E-09	2.26E-09	4.13E-09	3.88E-09	3.02E-09
-1.28E-09	-2.59E-10	6.78E-11	2.32E-10	9.95E-10	1.35E-09	1.87E-09	6.34E-10	9.38E-10	2.33E-09	1.22E-09	1.18E-09	3.02E-09	5.35E-09	5.28E-09	4.02E-09
-1.09E-09	2.49E-12	-2.95E-10	5.61E-11	9.02E-10	8.01E-10	1.10E-09	1.09E-09	1.62E-09	2.79E-09	1.91E-09	2.02E-09	3.56E-09	6.09E-09	5.71E-09	4.24E-09
-8.96E-10	-6.98E-10	-9.32E-10	-3.74E-10	7.13E-10	4.56E-10	3.60E-10	1.03E-09	2.29E-09	3.39E-09	2.54E-09	2.59E-09	3.63E-09	5.36E-09	5.17E-09	4.07E-09
-4.02E-10	-9.54E-10	-7.88E-10	-3.85E-10	4.05E-10	5.59E-10	6.29E-10	1.02E-09	3.14E-09	4.37E-09	3.31E-09	3.15E-09	3.94E-09	4.08E-09	3.54E-09	3.44E-09
-6.35E-10	-7.30E-10	-4.54E-10	5.96E-10	8.77E-10	1.18E-09	1.53E-09	2.40E-09	3.82E-09	4.61E-09	4.40E-09	3.87E-09	4.70E-09	4.39E-09	3.34E-09	3.87E-09
-5.86E-10	-2.20E-10	-2.66E-10	1.42E-09	2.05E-09	2.23E-09	2.83E-09	3.89E-09	4.83E-09	4.36E-09	5.41E-09	5.51E-09	5.90E-09	5.54E-09	4.48E-09	5.16E-09
-4.62E-10	8.85E-11	8.48E-11	2.06E-09	3.32E-09	3.63E-09	4.03E-09	4.81E-09	5.58E-09	3.70E-09	5.46E-09	6.64E-09	7.36E-09	7.31E-09	5.52E-09	6.55E-09
-6.79E-10	3.89E-12	-2.14E-11	1.66E-09	3.34E-09	3.66E-09	3.45E-09	6.03E-09	6.21E-09	4.40E-09	5.68E-09	6.81E-09	8.85E-09	8.36E-09	6.17E-09	6.46E-09
-7.61E-10	-2.79E-10	-3.62E-10	7.43E-10	1.90E-09	2.60E-09	3.23E-09	6.46E-09	7.90E-09	5.31E-09	6.25E-09	7.68E-09	9.58E-09	8.37E-09	6.10E-09	5.28E-09
-4.17E-10	-5.19E-10	-3.15E-10	7.38E-10	1.80E-09	2.55E-09	3.33E-09	5.65E-09	8.41E-09	4.77E-09	6.44E-09	8.33E-09	8.94E-09	7.23E-09	5.23E-09	4.74E-09
-6.55E-10	-6.08E-10	-3.06E-10	1.45E-09	2.90E-09	4.07E-09	3.94E-09	4.39E-09	7.16E-09	3.57E-09	5.20E-09	6.97E-09	5.67E-09	5.06E-09	3.69E-09	4.37E-09
-8.96E-10	-5.94E-10	5.91E-11	2.43E-09	4.54E-09	5.98E-09	4.30E-09	4.24E-09	4.27E-09	2.40E-09	3.26E-09	4.59E-09	3.96E-09	3.48E-09	2.95E-09	3.28E-09
-1.08E-09	-2.64E-10	3.58E-10	3.08E-09	5.97E-09	6.41E-09	4.21E-09	4.01E-09	3.44E-09	2.12E-09	2.30E-09	2.39E-09	2.82E-09	2.99E-09	2.89E-09	2.87E-09
-1.27E-09	2.77E-10	8.60E-11	2.60E-09	5.68E-09	4.60E-09	3.00E-09	3.87E-09	4.10E-09	2.49E-09	2.52E-09	1.76E-09	2.61E-09	3.14E-09	3.38E-09	3.08E-09
-9.95E-10	6.80E-10	-1.07E-10	1.16E-09	2.72E-09	2.48E-09	2.08E-09	3.51E-09	4.02E-09	3.23E-09	3.03E-09	1.97E-09	3.50E-09	3.72E-09	4.43E-09	3.72E-09
-9.77E-10	-1.16E-10	1.58E-11	7.08E-10	9.11E-10	1.78E-09	2.56E-09	4.02E-09	4.65E-09	4.09E-09	3.50E-09	2.61E-09	4.78E-09	4.45E-09	4.63E-09	4.14E-09
-1.10E-09	-9.30E-10	2.66E-11	5.32E-10	5.35E-10	2.37E-09	4.18E-09	5.14E-09	5.47E-09	4.01E-09	3.50E-09	3.17E-09	5.10E-09	4.75E-09	4.00E-09	4.28E-09
-1.47E-09	-9.04E-10	-5.89E-10	9.67E-11	4.19E-10	3.02E-09	4.68E-09	5.00E-09	4.89E-09	3.24E-09	2.98E-09	3.07E-09	4.28E-09	3.60E-09	3.16E-09	4.89E-09
-1.44E-09	-7.92E-10	-1.11E-09	-3.48E-10	3.06E-10	2.14E-09	2.90E-09	2.93E-09	2.75E-09	2.62E-09	2.86E-09	2.98E-09	3.16E-09	2.44E-09	2.45E-09	3.87E-09

-1.03E-09	-4.63E-10	-1.02E-09	-2.53E-10	-4.61E-10	7.33E-10	1.37E-09	1.93E-09	1.95E-09	1.92E-09	2.27E-09	1.99E-09	2.28E-09	1.28E-09	1.48E-09	2.13E-09
-7.45E-10	-6.54E-10	-1.19E-09	-3.60E-10	-1.09E-09	-2.36E-10	1.10E-09	1.39E-09	8.50E-10	9.70E-10	1.31E-09	3.32E-10	1.78E-09	5.77E-10	1.05E-09	1.95E-09
-1.07E-09	-9.86E-10	-1.41E-09	-6.67E-10	-1.36E-09	-3.99E-10	8.88E-10	2.39E-10	-1.17E-10	2.86E-10	1.80E-10	1.07E-11	1.86E-09	7.36E-10	1.45E-09	2.80E-09
-7.43E-10	-6.14E-10	-1.25E-09	-5.89E-11	-1.23E-09	-4.01E-10	8.15E-10	-4.54E-11	-3.75E-10	4.82E-10	-3.20E-10	-2.61E-10	7.69E-10	2.80E-10	1.58E-09	3.97E-09
-9.62E-11	-2.14E-10	-6.61E-10	8.78E-10	-2.46E-10	-1.63E-10	-3.17E-10	-4.84E-10	-6.45E-10	8.42E-10	-6.35E-10	-5.03E-10	-4.31E-10	-9.64E-10	1.05E-09	3.56E-09
4.30E-11	-1.15E-10	-4.32E-10	1.35E-09	8.88E-10	1.41E-10	-6.80E-10	-6.53E-10	-9.88E-10	3.34E-10	-7.98E-10	-6.28E-10	-4.96E-10	-1.13E-09	5.20E-10	2.78E-09
4.25E-11	-1.14E-10	-4.84E-10	1.42E-09	9.34E-10	2.76E-10	-6.74E-10	-2.97E-10	-8.67E-10	-2.47E-10	-1.18E-09	-8.44E-10	-2.24E-10	-7.60E-10	4.00E-10	1.38E-09
-3.70E-10	-2.18E-10	-6.83E-10	4.16E-10	-1.99E-10	2.68E-10	-3.47E-10	5.02E-11	-6.87E-10	-5.83E-10	-1.33E-09	-8.44E-10	8.04E-11	-2.00E-10	5.83E-10	7.63E-10
-2.75E-10	-5.60E-10	-1.21E-09	-1.34E-09	-1.40E-09	6.71E-12	-2.98E-10	9.54E-10	1.11E-10	-1.34E-09	-1.84E-09	-1.65E-09	4.10E-10	4.58E-10	7.12E-10	6.82E-10
-1.93E-10	-1.13E-09	-1.10E-09	-1.24E-09	-1.37E-09	-3.10E-10	-1.05E-10	1.18E-09	3.72E-10	-1.52E-09	-2.25E-09	-2.04E-09	4.57E-10	5.68E-10	4.36E-10	5.40E-10
-1.15E-09	-1.77E-09	-6.63E-10	-1.10E-09	-1.10E-09	-4.40E-10	-1.23E-10	1.03E-09	1.72E-10	-1.33E-09	-2.15E-09	-1.95E-09	4.59E-10	-5.64E-11	1.17E-10	5.36E-10
-1.13E-09	-1.47E-09	-1.13E-10	-5.74E-10	-8.38E-10	-2.90E-10	5.00E-11	-3.28E-10	-8.28E-10	-1.32E-09	-1.93E-09	-1.56E-09	6.20E-10	1.52E-10	7.80E-10	1.36E-09
-9.95E-10	-1.06E-09	-2.83E-10	-5.12E-10	-7.09E-10	-6.71E-10	-4.27E-10	-1.53E-09	-1.45E-09	-1.31E-09	-1.87E-09	-7.90E-10	3.60E-10	3.44E-10	1.50E-09	1.58E-09
-9.57E-10	-9.01E-10	1.89E-10	-4.33E-10	-6.53E-10	-1.09E-09	-9.91E-10	-1.76E-09	-1.56E-09	-1.26E-09	-1.61E-09	-3.17E-12	-6.47E-10	1.24E-10	8.15E-10	5.68E-10
-6.49E-10	-6.81E-11	5.12E-10	-2.96E-10	5.76E-11	-6.65E-10	-1.05E-09	-1.29E-09	-1.75E-09	-1.24E-09	-1.40E-09	4.27E-11	-8.92E-10	4.76E-11	4.51E-10	4.67E-10
-2.17E-11	9.11E-10	8.95E-10	-1.62E-11	4.40E-10	-3.37E-10	-1.34E-09	-1.04E-09	-1.70E-09	-5.04E-10	-8.26E-10	1.76E-10	-1.20E-09	-5.65E-10	1.20E-09	7.76E-10
-1.68E-10	1.25E-09	2.92E-10	-8.39E-10	8.15E-12	-6.72E-10	-1.52E-09	-1.21E-09	-1.28E-09	2.09E-10	2.43E-11	7.48E-10	-1.37E-09	-1.40E-09	2.40E-09	1.17E-09
-1.41E-09	-7.09E-10	-6.36E-10	-1.76E-09	-1.07E-09	-1.43E-09	-1.60E-09	-1.70E-09	-1.05E-09	-5.78E-11	8.28E-10	1.46E-09	-1.21E-09	-1.73E-09	1.55E-09	6.15E-10
-2.10E-09	-1.66E-09	-6.43E-10	-1.79E-09	-1.63E-09	-1.61E-09	-1.64E-09	-2.02E-09	-9.03E-10	2.80E-10	2.31E-10	5.07E-10	-1.67E-09	-1.75E-09	-1.59E-10	-2.63E-10
-2.21E-09	-1.81E-09	-5.61E-10	-1.30E-09	-2.00E-09	-1.41E-09	-1.99E-09	-2.42E-09	-1.66E-09	1.39E-10	-5.29E-11	-1.48E-10	-2.28E-09	-1.43E-09	-7.83E-10	-3.99E-10
-1.99E-09	-1.67E-09	-1.23E-09	-1.53E-09	-1.96E-09	-1.84E-09	-1.89E-09	-2.40E-09	-1.75E-09	4.98E-10	-3.15E-10	-1.29E-09	-2.77E-09	-1.41E-09	-1.22E-09	-7.44E-10
-1.59E-09	-1.75E-09	-2.40E-09	-2.16E-09	-2.15E-09	-1.89E-09	-1.42E-09	-1.77E-09	-7.55E-10	8.75E-11	-4.59E-10	-1.50E-09	-2.89E-09	-1.85E-09	-1.30E-09	-8.77E-10
-1.54E-09	-2.28E-09	-2.59E-09	-2.23E-09	-1.87E-09	-1.04E-09	-6.73E-10	-1.29E-09	-2.40E-10	-5.84E-10	-6.45E-10	-1.43E-09	-2.04E-09	-1.60E-09	-8.89E-10	-5.91E-10
-1.79E-09	-2.48E-09	-2.48E-09	-1.96E-09	-1.84E-09	-8.23E-10	-5.17E-10	-1.45E-09	-6.26E-10	-7.01E-10	-6.23E-10	-1.33E-09	-1.21E-09	-9.86E-10	-1.06E-09	-2.51E-10
-1.77E-09	-1.76E-09	-1.63E-09	-1.47E-09	-1.76E-09	-1.31E-09	-1.36E-09	-2.15E-09	-1.22E-09	-1.60E-09	-1.61E-09	-1.13E-09	-5.51E-10	-8.87E-11	-5.35E-10	2.97E-10
-2.31E-09	-9.61E-10	-5.34E-10	-5.58E-10	-1.41E-09	-1.32E-09	-1.73E-09	-2.21E-09	-2.14E-09	-1.70E-09	-1.68E-09	-4.43E-10	-1.25E-10	3.46E-10	7.18E-10	8.52E-10
-2.38E-09	-2.63E-10	-2.56E-10	-4.47E-11	-1.23E-09	-9.52E-10	-1.34E-09	-2.00E-09	-2.12E-09	-1.21E-09	-8.31E-10	5.27E-10	-3.59E-10	3.96E-10	1.69E-09	1.27E-09
-1.98E-09	-5.76E-10	-8.57E-10	-4.10E-10	-1.40E-09	-1.49E-09	-1.15E-09	-1.96E-09	-6.96E-10	-7.14E-10	-1.16E-10	5.91E-10	-5.69E-10	3.48E-10	2.09E-09	2.43E-09
-1.88E-09	-1.09E-09	-1.80E-09	-2.11E-09	-1.84E-09	-1.55E-09	-1.25E-09	-1.24E-09	2.45E-12	9.36E-11	-7.84E-12	1.06E-09	-6.49E-10	2.53E-10	2.62E-09	3.82E-09
-2.25E-09	-2.24E-09	-2.33E-09	-2.45E-09	-2.02E-09	-1.78E-09	-1.00E-09	-4.92E-10	3.95E-10	5.25E-10	4.04E-10	1.70E-09	-2.04E-10	6.80E-10	3.51E-09	5.51E-09
-3.19E-09	-2.86E-09	-2.58E-09	-2.19E-09	-2.09E-09	-2.37E-09	-5.32E-10	3.46E-10	6.33E-10	3.15E-10	9.97E-10	1.57E-09	-1.13E-10	1.27E-09	4.70E-09	6.49E-09
-2.99E-09	-2.23E-09	-1.97E-09	-2.03E-09	-2.18E-09	-2.48E-09	-1.09E-09	-7.30E-11	1.13E-09	-4.14E-10	9.51E-10	1.01E-09	-3.68E-10	1.31E-09	4.92E-09	5.15E-09
-2.07E-09	-1.45E-09	-1.06E-09	-1.77E-09	-1.83E-09	-2.07E-09	-4.22E-10	-3.81E-11	1.34E-09	-7.68E-10	3.92E-10	7.48E-11	-2.72E-10	8.72E-10	4.52E-09	3.12E-09
-1.23E-09	-7.08E-10	-8.85E-10	-2.10E-09	-1.84E-09	-9.60E-10	5.12E-11	-1.83E-10	4.58E-10	-1.15E-09	1.78E-11	-6.48E-10	-4.82E-10	7.54E-10	3.46E-09	1.77E-09
-1.26E-09	-7.68E-10	-9.96E-10	-2.06E-09	-1.57E-09	1.60E-10	-5.91E-10	-6.13E-10	-1.31E-09	-1.51E-09	-3.32E-11	-4.53E-10	-2.66E-10	3.40E-10	5.42E-10	-2.23E-10
-2.41E-09	-1.40E-09	-7.45E-10	-1.48E-09	-8.58E-10	-3.70E-10	-1.51E-09	-7.23E-10	-1.66E-09	-1.54E-09	-4.79E-10	-7.37E-10	-3.03E-10	1.04E-10	-1.62E-09	-2.33E-09
-3.08E-09	-2.58E-09	-1.73E-09	-8.04E-10	-1.07E-09	-1.70E-09	-1.95E-09	-6.14E-10	-1.95E-09	-2.18E-09	-1.47E-09	-1.67E-09	-2.84E-10	-6.89E-10	-2.48E-09	-4.01E-09
-2.58E-09	-3.25E-09	-3.16E-09	-1.14E-09	-1.66E-09	-2.36E-09	-1.90E-09	-8.11E-10	-2.20E-09	-2.41E-09	-2.36E-09	-2.62E-09	-4.80E-10	-1.71E-09	-3.63E-09	-4.56E-09
-7.30E-10	-2.25E-09	-3.28E-09	-1.48E-09	-2.07E-09	-2.94E-09	-1.72E-09	-1.41E-09	-2.35E-09	-2.66E-09	-2.95E-09	-3.46E-09	-1.79E-09	-3.53E-09	-4.39E-09	-5.04E-09
1.45E-09	-9.05E-10	-2.17E-09	-8.48E-10	-2.06E-09	-3.00E-09	-1.80E-09	-1.84E-09	-2.22E-09	-3.19E-09	-3.49E-09	-3.96E-09	-3.19E-09	-4.31E-09	-4.59E-09	-5.41E-09
1.72E-09	-3.25E-10	-4.63E-10	-8.67E-10	-1.17E-09	-2.65E-09	-2.33E-09	-1.97E-09	-1.89E-09	-3.44E-09	-4.14E-09	-4.17E-09	-3.33E-09	-4.38E-09	-4.66E-09	-5.46E-09
6.02E-11	-3.98E-10	-2.58E-11	-1.50E-09	-1.15E-09	-2.73E-09	-3.33E-09	-2.14E-09	-2.10E-09	-3.98E-09	-4.60E-09	-4.18E-09	-3.14E-09	-4.32E-09	-4.45E-09	-5.36E-09
-2.30E-09	-1.68E-09	-1.08E-09	-2.94E-09	-2.41E-09	-3.51E-09	-3.82E-09	-3.08E-09	-3.52E-09	-4.68E-09	-4.76E-09	-4.25E-09	-2.96E-09	-4.45E-09	-4.89E-09	-5.40E-09
-3.57E-09	-3.29E-09	-3.21E-09	-3.97E-09	-4.20E-09	-4.74E-09	-4.58E-09	-4.11E-09	-4.83E-09	-4.66E-09	-4.56E-09	-4.04E-09	-3.43E-09	-4.69E-09	-5.51E-09	-5.46E-09
-4.00E-09	-4.22E-09	-4.07E-09	-4.36E-09	-4.64E-09	-5.18E-09	-5.02E-09	-4.82E-09	-5.40E-09	-4.94E-09	-5.03E-09	-4.46E-09	-4.34E-09	-4.98E-09	-6.01E-09	-6.29E-09
-4.23E-09	-4.47E-09	-4.27E-09	-4.67E-09	-4.88E-09	-5.35E-09	-5.37E-09	-5.30E-09	-5.63E-09	-5.58E-09	-5.62E-09	-5.76E-09	-5.84E-09	-6.29E-09	-6.37E-09	-7.22E-09
-4.27E-09	-4.54E-09	-4.28E-09	-5.02E-09	-5.17E-09	-5.77E-09	-5.51E-09	-5.60E-09	-5.96E-09	-6.18E-09	-6.22E-09	-6.74E-09	-6.58E-09	-6.47E-09	-6.61E-09	-7.48E-09
-4.70E-09	-5.10E-09	-4.60E-09	-5.02E-09	-5.16E-09	-5.80E-09	-5.70E-09	-5.80E-09	-6.29E-09	-6.10E-09	-6.40E-09	-6.93E-09	-6.96E-09	-6.38E-09	-6.84E-09	-7.71E-09
-5.07E-09	-5.65E-09	-4.78E-09	-4.85E-09	-5.30E-09	-5.71E-09	-5.49E-09	-5.69E-09	-6.40E-09	-6.14E-09	-6.46E-09	-6.79E-09	-7.29E-09	-6.76E-09	-7.05E-09	-7.43E-09
-4.92E-09	-5.44E-09	-4.66E-09	-4.84E-09	-5.62E-09	-5.84E-09	-5.34E-09	-6.22E-09	-6.68E-09	-6.18E-09	-6.59E-09	-6.52E-09	-6.86E-09	-7.02E-09	-6.94E-09	-7.19E-09

5.21E-16	5.11E-16	4.40E-16	4.21E-16	3.63E-16	3.56E-16	3.38E-16	3.49E-16	3.50E-16	2.94E-16	2.50E-16	2.42E-16	2.05E-16	1.90E-16	1.63E-16	1.56E-16
4.56E-16	4.41E-16	3.87E-16	3.70E-16	3.20E-16	3.04E-16	2.93E-16	3.22E-16	2.99E-16	2.47E-16	2.20E-16	2.04E-16	1.89E-16	1.66E-16	1.35E-16	1.42E-16
3.97E-16	3.68E-16	3.27E-16	3.13E-16	2.62E-16	2.65E-16	2.60E-16	2.72E-16	2.34E-16	2.12E-16	1.97E-16	1.80E-16	1.65E-16	1.45E-16	1.20E-16	1.35E-16
3.35E-16	2.94E-16	2.72E-16	2.66E-16	2.31E-16	2.36E-16	2.36E-16	2.35E-16	1.99E-16	1.93E-16	1.70E-16	1.55E-16	1.45E-16	1.23E-16	1.07E-16	1.27E-16
2.97E-16	2.60E-16	2.35E-16	2.40E-16	2.19E-16	2.14E-16	2.22E-16	2.08E-16	1.74E-16	1.67E-16	1.49E-16	1.41E-16	1.28E-16	9.42E-17	8.37E-17	1.07E-16
2.37E-16	2.25E-16	2.02E-16	2.12E-16	1.98E-16	1.92E-16	1.94E-16	1.81E-16	1.60E-16	1.38E-16	1.32E-16	1.12E-16	9.56E-17	7.51E-17	6.40E-17	7.82E-17
1.97E-16	1.79E-16	1.76E-16	1.84E-16	1.83E-16	1.73E-16	1.72E-16	1.55E-16	1.39E-16	1.15E-16	1.11E-16	9.35E-17	7.45E-17	6.38E-17	5.31E-17	6.22E-17
1.70E-16	1.43E-16	1.47E-16	1.66E-16	1.61E-16	1.51E-16	1.51E-16	1.30E-16	1.21E-16	1.01E-16	8.60E-17	8.69E-17	6.77E-17	5.66E-17	4.52E-17	4.95E-17
1.40E-16	1.13E-16	1.23E-16	1.37E-16	1.38E-16	1.14E-16	1.25E-16	1.08E-16	9.60E-17	8.98E-17	6.93E-17	8.13E-17	6.86E-17	6.00E-17	3.86E-17	4.05E-17
1.13E-16	9.56E-17	1.08E-16	1.12E-16	1.30E-16	9.70E-17	1.03E-16	9.08E-17	8.19E-17	8.22E-17	5.96E-17	7.85E-17	7.21E-17	6.10E-17	3.64E-17	3.32E-17
9.08E-17	8.49E-17	1.01E-16	9.68E-17	9.26E-17	8.40E-17	8.27E-17	6.70E-17	5.98E-17	6.97E-17	5.74E-17	8.32E-17	7.87E-17	5.90E-17	3.00E-17	2.79E-17
6.83E-17	7.93E-17	9.66E-17	8.02E-17	7.41E-17	6.75E-17	6.51E-17	4.67E-17	4.19E-17	5.29E-17	4.64E-17	6.80E-17	7.14E-17	5.11E-17	2.59E-17	2.61E-17
5.29E-17	7.24E-17	8.84E-17	6.82E-17	6.20E-17	5.31E-17	4.73E-17	3.58E-17	3.05E-17	3.65E-17	2.97E-17	4.42E-17	5.24E-17	4.08E-17	2.35E-17	2.50E-17
4.17E-17	6.46E-17	6.31E-17	5.47E-17	5.19E-17	4.38E-17	4.16E-17	3.07E-17	2.67E-17	2.84E-17	1.94E-17	2.54E-17	2.77E-17	3.41E-17	2.31E-17	2.30E-17
3.24E-17	4.39E-17	4.12E-17	4.32E-17	4.00E-17	4.04E-17	3.64E-17	2.65E-17	2.14E-17	1.91E-17	1.64E-17	1.81E-17	1.74E-17	2.66E-17	1.86E-17	1.80E-17
2.34E-17	2.89E-17	3.25E-17	3.98E-17	3.51E-17	3.75E-17	3.30E-17	1.98E-17	1.28E-17	1.27E-17	1.70E-17	1.57E-17	1.53E-17	1.81E-17	1.40E-17	1.57E-17
1.94E-17	2.36E-17	3.45E-17	3.75E-17	3.79E-17	4.01E-17	3.06E-17	1.59E-17	1.01E-17	8.78E-18	3.10E-17	1.67E-17	1.36E-17	1.30E-17	1.17E-17	1.31E-17
1.99E-17	1.86E-17	2.69E-17	2.56E-17	3.17E-17	3.62E-17	2.47E-17	1.61E-17	9.51E-18	5.44E-18	6.61E-17	2.18E-17	1.64E-17	1.34E-17	1.08E-17	9.44E-18
1.85E-17	1.39E-17	1.66E-17	1.26E-17	2.01E-17	2.10E-17	1.66E-17	1.48E-17	1.04E-17	1.17E-17	1.21E-16	2.82E-17	1.96E-17	1.38E-17	1.03E-17	1.06E-17
1.46E-17	9.17E-18	1.08E-17	7.96E-18	1.03E-17	1.26E-17	1.11E-17	8.96E-18	7.28E-18	1.26E-17	3.19E-17	2.51E-17	1.72E-17	1.09E-17	8.39E-18	1.13E-17
1.27E-17	8.26E-18	8.30E-18	4.69E-18	6.94E-18	7.74E-18	6.38E-18	5.82E-18	5.54E-18	8.45E-18	1.27E-17	1.88E-17	9.87E-18	7.54E-18	6.74E-18	1.30E-17
8.24E-18	8.04E-18	8.61E-18	4.40E-18	6.06E-18	5.79E-18	3.34E-18	3.93E-18	3.34E-18	5.78E-18	7.59E-18	1.63E-17	4.51E-18	6.37E-18	6.72E-18	1.58E-17
7.06E-18	6.63E-18	1.36E-17	1.19E-17	1.19E-17	3.88E-18	3.62E-18	4.48E-18	2.82E-18	3.82E-18	6.07E-18	1.21E-17	5.43E-18	8.27E-18	6.48E-18	1.14E-17
6.53E-18	4.64E-18	2.25E-17	2.74E-17	2.06E-17	2.25E-18	2.61E-18	2.89E-18	3.21E-18	5.50E-18	4.38E-18	8.60E-18	7.66E-18	5.76E-18	7.39E-18	8.30E-18
6.58E-18	3.90E-18	1.31E-17	1.73E-17	1.87E-17	1.91E-18	2.70E-18	3.22E-18	2.98E-18	7.72E-18	3.52E-18	6.06E-18	7.11E-18	6.77E-18	9.02E-18	9.50E-18
5.71E-18	4.41E-18	5.86E-18	3.62E-18	4.38E-18	2.00E-18	2.96E-18	2.60E-18	2.91E-18	9.92E-18	3.74E-18	5.67E-18	5.63E-18	9.13E-18	1.35E-17	1.11E-17
5.52E-18	3.68E-18	5.66E-18	2.75E-18	2.61E-18	2.81E-18	4.70E-18	3.46E-18	5.02E-18	9.77E-18	3.94E-18	5.92E-18	5.29E-18	1.11E-17	1.32E-17	1.26E-17
6.26E-18	4.43E-18	5.70E-18	2.03E-18	1.78E-18	3.12E-18	7.41E-18	3.97E-18	4.71E-18	1.05E-17	5.63E-18	6.82E-18	8.03E-18	1.43E-17	1.24E-17	1.28E-17
9.66E-18	5.65E-18	5.53E-18	1.12E-18	1.27E-18	3.15E-18	7.38E-18	4.35E-18	4.19E-18	1.09E-17	6.13E-18	7.55E-18	1.09E-17	1.59E-17	1.09E-17	1.13E-17
1.45E-17	9.46E-18	7.38E-18	1.04E-18	1.33E-18	3.07E-18	4.27E-18	3.58E-18	2.65E-18	7.96E-18	5.46E-18	6.62E-18	8.61E-18	1.17E-17	1.08E-17	1.16E-17
1.20E-17	1.02E-17	7.36E-18	1.16E-18	2.95E-18	2.19E-18	2.15E-18	4.49E-18	2.98E-18	5.66E-18	5.02E-18	4.48E-18	5.78E-18	7.93E-18	1.24E-17	1.34E-17

6.33E-18	8.11E-18	6.04E-18	2.81E-18	5.79E-18	2.83E-18	2.31E-18	7.04E-18	4.92E-18	5.08E-18	4.31E-18	5.25E-18	5.45E-18	6.45E-18	1.38E-17	1.34E-17
6.20E-18	9.03E-18	7.23E-18	5.63E-18	7.91E-18	3.97E-18	3.25E-18	1.09E-17	8.29E-18	5.59E-18	5.13E-18	6.85E-18	5.80E-18	8.32E-18	1.43E-17	1.35E-17
3.26E-18	6.78E-18	6.79E-18	6.74E-18	5.89E-18	4.88E-18	5.45E-18	1.58E-17	1.25E-17	7.04E-18	6.96E-18	9.17E-18	8.15E-18	1.02E-17	1.44E-17	1.52E-17
2.85E-18	4.20E-18	3.48E-18	4.46E-18	4.39E-18	6.26E-18	6.98E-18	1.59E-17	1.65E-17	1.09E-17	9.50E-18	8.72E-18	7.15E-18	9.23E-18	2.27E-17	1.66E-17
2.85E-18	3.41E-18	1.96E-18	3.41E-18	3.47E-18	5.71E-18	6.48E-18	1.09E-17	1.61E-17	1.33E-17	1.13E-17	1.00E-17	8.30E-18	1.04E-17	2.62E-17	1.80E-17
3.13E-18	2.46E-18	1.60E-18	4.01E-18	2.73E-18	5.04E-18	6.66E-18	6.34E-18	1.43E-17	1.60E-17	1.24E-17	1.21E-17	1.01E-17	1.09E-17	2.22E-17	1.73E-17
3.45E-18	2.02E-18	1.27E-18	3.07E-18	2.10E-18	4.77E-18	4.77E-18	5.16E-18	8.79E-18	1.86E-17	1.11E-17	1.52E-17	1.17E-17	9.47E-18	1.94E-17	1.70E-17
3.28E-18	1.41E-18	9.54E-19	1.91E-18	1.43E-18	4.66E-18	4.29E-18	5.06E-18	7.00E-18	1.76E-17	1.21E-17	1.25E-17	1.17E-17	1.05E-17	1.53E-17	1.67E-17
1.57E-18	4.54E-19	6.57E-19	1.72E-18	1.41E-18	4.47E-18	1.83E-18	4.19E-18	5.92E-18	1.27E-17	1.40E-17	1.16E-17	1.01E-17	1.28E-17	1.03E-17	1.64E-17
7.20E-19	1.59E-19	5.64E-19	1.20E-18	8.14E-19	3.89E-18	8.01E-19	1.85E-18	3.05E-18	1.14E-17	1.06E-17	1.06E-17	8.79E-18	1.16E-17	9.44E-18	1.27E-17
4.79E-19	2.71E-19	6.77E-19	4.63E-19	3.66E-20	1.98E-18	1.05E-18	1.38E-18	2.80E-18	7.96E-18	7.67E-18	8.85E-18	8.44E-18	1.18E-17	1.16E-17	1.16E-17
1.47E-18	6.55E-19	1.02E-18	2.74E-19	7.17E-21	3.45E-19	1.03E-18	1.51E-18	4.94E-18	5.95E-18	7.59E-18	7.26E-18	8.88E-18	1.39E-17	1.49E-17	1.41E-17
1.41E-18	1.03E-18	9.35E-19	4.51E-19	8.80E-25	1.20E-19	9.93E-19	2.10E-18	6.31E-18	5.27E-18	8.41E-18	5.46E-18	1.07E-17	1.35E-17	1.65E-17	1.68E-17
8.36E-19	1.37E-18	1.65E-18	7.42E-19	1.31E-20	7.70E-19	1.38E-18	1.32E-18	4.31E-18	5.18E-18	8.56E-18	4.59E-18	1.09E-17	1.32E-17	1.82E-17	1.42E-17
7.09E-19	2.21E-18	4.41E-18	8.60E-19	1.04E-20	1.57E-18	2.67E-18	2.93E-18	4.79E-18	5.77E-18	7.27E-18	4.20E-18	7.70E-18	1.16E-17	1.73E-17	1.09E-17
1.56E-18	4.56E-18	5.89E-18	6.54E-19	9.76E-20	1.41E-18	4.38E-18	5.48E-18	6.28E-18	7.41E-18	6.80E-18	3.48E-18	4.37E-18	7.85E-18	1.71E-17	9.30E-18
1.12E-18	7.24E-18	5.05E-18	6.17E-19	2.00E-19	9.04E-19	3.40E-18	5.29E-18	9.19E-18	1.12E-17	9.33E-18	4.65E-18	5.50E-18	6.85E-18	1.93E-17	1.10E-17
2.08E-18	1.16E-17	3.95E-18	9.35E-19	7.15E-19	6.81E-19	3.54E-18	5.43E-18	1.24E-17	9.74E-18	9.27E-18	7.14E-18	7.28E-18	7.39E-18	1.98E-17	1.66E-17
6.87E-18	1.56E-17	3.65E-18	1.86E-18	1.61E-18	2.06E-18	3.86E-18	6.42E-18	1.96E-17	1.00E-17	9.45E-18	9.81E-18	9.51E-18	9.32E-18	1.83E-17	1.74E-17
2.68E-18	9.47E-18	2.29E-18	3.40E-18	1.53E-18	2.48E-18	2.74E-18	4.64E-18	1.27E-17	7.08E-18	1.05E-17	1.08E-17	1.19E-17	1.27E-17	1.64E-17	1.16E-17
5.00E-19	3.73E-18	6.82E-19	2.21E-18	7.63E-19	1.53E-18	1.22E-18	2.49E-18	8.10E-18	6.76E-18	1.23E-17	1.10E-17	1.65E-17	1.66E-17	1.69E-17	8.92E-18
8.68E-20	1.01E-18	8.49E-20	1.16E-18	1.57E-19	6.99E-19	1.92E-18	5.20E-19	4.96E-18	9.14E-18	1.65E-17	1.19E-17	1.64E-17	1.63E-17	1.60E-17	8.54E-18
5.16E-20	3.49E-19	4.46E-21	4.08E-21	3.45E-20	1.17E-20	2.18E-18	4.12E-20	3.41E-18	8.08E-18	2.04E-17	1.28E-17	1.31E-17	1.78E-17	1.25E-17	8.41E-18
1.73E-19	5.12E-20	7.79E-21	3.88E-19	8.32E-20	6.67E-20	1.15E-18	1.19E-20	4.75E-19	4.89E-18	1.11E-17	1.10E-17	9.47E-18	1.46E-17	1.01E-17	9.91E-18
1.40E-19	1.26E-19	6.83E-20	8.81E-19	3.54E-20	4.90E-20	8.20E-19	1.24E-19	2.42E-20	3.04E-18	2.08E-18	6.46E-18	6.80E-18	1.26E-17	1.00E-17	1.25E-17
2.99E-21	1.19E-19	2.48E-19	2.73E-19	2.04E-19	3.45E-20	2.64E-19	1.26E-19	4.21E-21	2.40E-18	5.85E-19	3.62E-18	4.63E-18	9.57E-18	1.11E-17	1.11E-17
1.69E-19	1.88E-20	5.92E-19	1.29E-20	1.08E-18	1.77E-20	4.66E-19	4.79E-20	8.35E-20	1.63E-18	4.33E-19	1.98E-18	3.97E-18	1.18E-17	9.95E-18	7.69E-18
2.21E-18	2.08E-19	1.27E-20	8.42E-21	9.99E-19	7.21E-19	1.65E-18	1.22E-19	1.66E-19	2.79E-18	7.86E-19	1.01E-18	5.11E-18	1.71E-17	1.50E-17	9.10E-18
1.63E-18	6.69E-20	4.60E-21	5.39E-20	9.90E-19	1.82E-18	3.50E-18	4.02E-19	8.80E-19	5.43E-18	1.50E-18	1.40E-18	9.14E-18	2.86E-17	2.78E-17	1.62E-17
1.20E-18	6.18E-24	8.71E-20	3.15E-21	8.14E-19	6.41E-19	1.20E-18	1.18E-18	2.61E-18	7.76E-18	3.65E-18	4.07E-18	1.27E-17	3.71E-17	3.27E-17	1.80E-17
8.03E-19	4.87E-19	8.68E-19	1.40E-19	5.08E-19	2.08E-19	1.30E-19	1.05E-18	5.23E-18	1.15E-17	6.48E-18	6.69E-18	1.32E-17	2.87E-17	2.68E-17	1.66E-17
1.61E-19	9.10E-19	6.21E-19	1.48E-19	1.64E-19	3.13E-19	3.96E-19	1.05E-18	9.87E-18	1.91E-17	1.10E-17	9.90E-18	1.55E-17	1.67E-17	1.25E-17	1.18E-17
4.03E-19	5.33E-19	2.06E-19	3.55E-19	7.70E-19	1.38E-18	2.34E-18	5.77E-18	1.46E-17	2.12E-17	1.93E-17	1.49E-17	2.21E-17	1.93E-17	1.12E-17	1.50E-17
3.44E-19	4.86E-20	7.10E-20	2.01E-18	4.18E-18	4.97E-18	8.00E-18	1.51E-17	2.33E-17	1.90E-17	2.93E-17	3.03E-17	3.48E-17	3.07E-17	2.01E-17	2.66E-17
2.13E-19	7.84E-21	7.19E-21	4.24E-18	1.10E-17	1.32E-17	1.62E-17	2.31E-17	3.12E-17	1.37E-17	2.99E-17	4.41E-17	5.41E-17	5.35E-17	3.04E-17	4.29E-17
4.61E-19	1.52E-23	4.59E-22	2.74E-18	1.12E-17	1.34E-17	1.19E-17	3.63E-17	3.85E-17	1.94E-17	3.23E-17	4.63E-17	7.83E-17	7.00E-17	3.80E-17	4.18E-17
5.78E-19	7.78E-20	1.31E-19	5.51E-19	3.60E-18	6.74E-18	1.04E-17	4.17E-17	6.24E-17	2.82E-17	3.91E-17	5.90E-17	9.17E-17	7.00E-17	3.72E-17	2.78E-17
1.74E-19	2.70E-19	9.92E-20	5.45E-19	3.24E-18	6.52E-18	1.11E-17	3.19E-17	7.07E-17	2.28E-17	4.15E-17	6.94E-17	8.00E-17	5.23E-17	2.74E-17	2.25E-17
4.29E-19	3.69E-19	9.33E-20	2.10E-18	8.38E-18	1.66E-17	1.56E-17	1.93E-17	5.13E-17	1.28E-17	2.70E-17	4.86E-17	3.21E-17	2.56E-17	1.36E-17	1.91E-17
8.03E-19	3.53E-19	3.49E-21	5.92E-18	2.06E-17	3.58E-17	1.85E-17	1.80E-17	1.82E-17	5.76E-18	1.06E-17	2.10E-17	1.57E-17	1.21E-17	8.70E-18	1.07E-17
1.16E-18	6.96E-20	1.28E-19	9.51E-18	3.56E-17	4.10E-17	1.77E-17	1.61E-17	1.18E-17	4.51E-18	5.28E-18	5.71E-18	7.93E-18	8.96E-18	8.38E-18	8.26E-18
1.62E-18	7.65E-20	7.39E-21	6.77E-18	3.23E-17	2.11E-17	9.00E-18	1.50E-17	1.68E-17	6.21E-18	6.33E-18	3.11E-18	6.83E-18	9.89E-18	1.14E-17	9.48E-18
9.90E-19	4.62E-19	1.14E-20	1.35E-18	7.37E-18	6.15E-18	4.34E-18	1.23E-17	1.62E-17	1.04E-17	9.19E-18	3.87E-18	1.23E-17	1.38E-17	1.96E-17	1.38E-17
9.54E-19	1.35E-20	2.51E-22	5.02E-19	8.30E-19	3.15E-18	6.56E-18	1.62E-17	2.16E-17	1.67E-17	1.23E-17	6.80E-18	2.29E-17	1.98E-17	2.14E-17	1.72E-17
1.21E-18	8.64E-19	7.10E-22	2.83E-19	2.87E-19	5.62E-18	1.75E-17	2.65E-17	2.99E-17	1.61E-17	1.22E-17	1.00E-17	2.60E-17	2.25E-17	1.60E-17	1.83E-17
2.17E-18	8.17E-19	3.47E-19	9.35E-21	1.75E-19	9.11E-18	2.19E-17	2.50E-17	2.39E-17	1.05E-17	8.86E-18	9.43E-18	1.83E-17	1.30E-17	9.99E-18	2.39E-17
2.07E-18	6.27E-19	1.23E-18	1.21E-19	9.38E-20	4.57E-18	8.39E-18	8.59E-18	7.57E-18	6.88E-18	8.17E-18	8.86E-18	1.00E-17	5.95E-18	6.01E-18	1.50E-17
1.07E-18	2.14E-19	1.04E-18	6.39E-20	2.12E-19	5.38E-19	1.87E-18	3.73E-18	3.81E-18	3.69E-18	5.14E-18	3.98E-18	5.21E-18	1.63E-18	2.20E-18	4.55E-18
5.55E-19	4.27E-19	1.43E-18	1.29E-19	1.18E-18	5.59E-20	1.21E-18	1.94E-18	7.22E-19	9.40E-19	1.72E-18	1.10E-19	3.17E-18	3.33E-19	1.11E-18	3.80E-18
1.14E-18	9.72E-19	2.00E-18	4.45E-19	1.84E-18	1.59E-19	7.88E-19	5.71E-20	1.37E-20	8.20E-20	3.23E-20	1.14E-22	3.45E-18	5.41E-19	2.11E-18	7.84E-18
5.53E-19	3.77E-19	1.55E-18	3.47E-21	1.51E-18	1.61E-19	6.64E-19	2.06E-21	1.41E-19	2.32E-19	1.02E-19	6.83E-20	5.91E-19	7.85E-20	2.49E-18	1.58E-17
9.25E-21	4.56E-20	4.37E-19	7.71E-19	6.03E-20	2.66E-20	1.00E-19	2.34E-19	4.16E-19	7.10E-19	4.03E-19	2.53E-19	1.86E-19	9.29E-19	1.11E-18	1.27E-17
1.85E-21	1.32E-20	1.87E-19	1.81E-18	7.89E-19	1.99E-20	4.62E-19	4.26E-19	9.76E-19	1.12E-19	6.37E-19	3.95E-19	2.46E-19	1.28E-18	2.70E-19	7.71E-18
1.81E-21	1.31E-20	2.34E-19	2.01E-18	8.72E-19	7.63E-20	4.55E-19	8.83E-20	7.51E-19	6.08E-20	1.40E-18	7.13E-19	5.00E-20	5.78E-19	1.60E-19	1.91E-18
1.37E-19	4.73E-20	4.67E-19	1.73E-19	3.95E-20	7.17E-20	1.20E-19	2.52E-21	4.72E-19	3.40E-19	1.76E-18	7.12E-19	6.47E-21	3.98E-20	3.40E-19	5.82E-19
7.56E-20	3.14E-19	1.46E-18	1.79E-18	1.97E-18	4.50E-23	8.86E-20	9.09E-19	1.23E-20	1.79E-18	3.37E-18	2.71E-18	1.68E-19	2.10E-19	5.07E-19	4.65E-19
3.72E-20	1.29E-18	1.21E-18	1.55E-18	1.86E-18	9.62E-20	1.10E-20	1.38E-18	1.38E-19	2.30E-18	5.07E-18	4.15E-18	2.09E-19	3.22E-19	1.90E-19	2.92E-19
1.33E-18	3.14E-18	4.39E-19	1.20E-18	1.20E-18	1.93E-19	1.52E-20	1.06E-18	2.97E-20	1.77E-18	4.63E-18	3.81E-18	2.11E-19	3.18E-21	1.37E-20	2.88E-19
1.27E-18	2.15E-18	1.28E-20	3.29E-19	7.02E-19	8.40E-20	2.50E-21	1.07E-19	6.85E-19	1.75E-18	3.73E-18	2.42E-18	3.84E-19	2.30E-20	6.08E-19	1.86E-18
9.90E-19	1.13E-18	8.03E-20	2.62E-19	5.03E-19	4.50E-19	1.83E-19	2.33E-18	2.09E-18	1.71E-18	3.50E-18	6.24E-19	1.30E-19	1.18E-19	2.24E-18	2.51E-18
9.16E-19	8.12E-19	3.57E-20	1.87E-19	4.26E-19	1.18E-18	9.82E-19	3.09E-18	2.44E-18	1.60E-18	2.61E-18	1.00E-23	4.19E-19	1.54E-20	6.64E-19	3.22E-19
4.21E-19	4.64E-21	2.62E-19	8.78E-20	3.32E-21	4.42E-19	1.11E-18	1.67E-18	3.05E-18	1.53E-18	1.96E-18	1.83E-21	7.96E-19	2.27E-21	2.03E-19	2.18E-19
4.70E-22	8.30E-19	8.02E-19	2.62E-22	1.93E-19	1.13E-19	1.80E-18	1.07E-18	2.88E-18	2.54E-19	6.83E-19	3.11E-20	1.44E-18	3.19E-19	1.44E-18	6.02E-19
2.84E-20	1.57E-18	8.52E-20	7.03E-19	6.64E-23	4.52E-19	2.31E-18	1.45E-18	1.65E-18	4.38E-20	5.88E-22	5.59E-19	1.88E-18	1.95E-18	5.77E-18	1.37E-18
1.98E-18	5.03E-19	4.05E-19	3.09E-18	1.15E-18	2.05E-18	2.55E-18	2.88E-18	1.11E-18	3.35E-21	6.86E-19	2.14E-18	1.47E-18	3.00E-18	2.40E-18	3.79E-19
4.41E-18	2.75E-18	4.13E-19	3.21E-18	2.66E-18	2.59E-18	2.70E-18	4.06E-18	8.15E-19	7.84E-20	5.33E-20	2.57E-19	2.78E-18	3.06E-18	2.54E-20	6.93E-20
4.87E-18	3.29E-18	3.15E-19	1.70E-18	4.00E-18	1.98E-18	3.95E-18	5.87E-18	2.75E-18	1.94E-20	2.80E-21	2.19E-20	5.18E-18	2.05E-18	6.13E-19	1.59E-19
3.98E-18	2.80E-18	1.52E-18	2.34E-18	3.85E-18	3.39E-18	3.58E-18	5.77E-18	3.05E-18	2.48E-19	9.95E-20	1.65E-18	7.66E-18	2.00E-18	1.48E-18	5.54E-19
2.51E-18	3.05E-18	5.76E-18	4.64E-18	4.61E-18	3.56E-18	2.01E-18	3.12E-18	5.70E-19	7.66E-21	2.11E-19	2.26E-18	8.38E-18	3.41E-18	1.68E-18	7.69E-19
2.37E-18	5.18E-18	6.70E-18	4.99E-18	3.51E-18	1.07E-18	4.53E-19	1.66E-18	5.77E-20	3.41E-19	4.16E-19	2.06E-18	4.14E-18	2.57E-18	7.91E-19	3.49E-19
3.21E-18	6.17E-18	6.14E-18	3.83E-18	3.38E-18	6.77E-19	2.67E-19	2.09E-18	3.92E-19	4.91E-19	3.88E-19	1.78E-18	1.47E-18	9.72E-19	1.13E-18	6.31E-20
3.15E-18	3.09E-18	2.65E-18	2.16E-18	3.10E-18	1.71E-18	1.86E-18	4.62E-18	1.50E-18	2.56E-18	2.58E-18	1.28E-18	3.04E-19	7.86E-21	2.86E-19	8.82E-20
5.32E-18	9.23E-19	2.85E-19	3.11E-19	1.99E-18	1.74E-18	2.98E-18	4.86E-18	4.58E-18	2.89E-18	2.81E-18	1.96E-19	1.56E-20	1.19E-19	5.16E-19	7.25E-19
5.64E-18	6.94E-20	6.54E-20	2.00E-21	1.52E-18	9.05E-19	1.80E-18	4.02E-18	4.50E-18	1.45E-18	6.91E-19	2.78E-19	1.29E-19	1.56E-19	2.86E-18	1.63E-18
3.92E-18	3.31E-19	7.34E-19	1.68E-19	1.96E-18	2.21E-18	1.33E-18	3.85E-18	4.84E-19	5.09E-19	1.36E-20	3.50E-19	3.23E-19	1.21E-19	4.35E-18	5.88E-18
3.53E-18	1.19E-18	3.25E-18	4.43E-18	3.37E-18	2.41E-18	1.57E-18	1.53E-18	6.01E-24	8.76E-21	6.14E-23	1.13E-18	4.21E-19	6.42E-20	6.84E-18	1.46E-17
5.05E-18	5.02E-18	5.42E-18	5.99E-18	4.07E-18	3.15E-18	1.00E-18	2.43E-19	1.56E-19	2.75E-19	1.63E-19	2.89E-18	4.16E-20	4.62E-19	1.23E-17	3.03E-17
1.02E-17	8.20E-18	6.65E-18	4.82E-18	4.38E-18	5.62E-18	2.83E-19	1.19E-19	4.01E-19	9.90E-20	9.93E-19	2.48E-18	1.27E-20	1.63E-18	2.21E-17	4.21E-17
8.94E-18	4.99E-18	3.90E-18	4.11E-18	4.74E-18	6.16E-18	1.19E-18	5.33E-21	1.27E-18	1.71E-19	9.04E-19	1.03E-18	1.36E-19	1.72E-18	2.42E-17	2.66E-17
4.28E-18	2.11E-18	1.12E-18	3.13E-18	3.34E-18	4.30E-18	1.78E-19	1.45E-21	1.79E-18	5.89E-19	1.54E-19	5.60E-21	7.39E-20	7.60E-19	2.04E-17	9.73E-18
1.51E-18	5.02E-19	7.83E-19	4.39E-18	3.40E-18	9.21E-19	2.62E-21	3.34E-20	2.10E-19	1.33E-18	3.17E-22	4.20E-19	2.33E-19	5.69E-19	1.19E-17	3.12E-18
1.59E-18	5.90E-19	9.93E-19	4.23E-18	2.47E-18	2.55E-20	3.49E-19	3.76E-19	1.72E-18	2.29E-18	1.10E-21	2.05E-19	7.08E-20	1.16E-19	2.93E-19	4.97E-20
5.79E-18	1.96E-18	5.55E-19	2.20E-18	7.36E-19	1.37E-19	2.28E-18	5.23E-19	2.76E-18	2.36E-18	2.30E-19	5.43E-19	9.15E-20	1.09E-20	2.64E-18	5.42E-18
9.48E-18	6.65E-18	3.00E-18	6.47E-19	1.15E-18	2.90E-18	3.81E-18	3.77E-19	3.80E-18	4.74E-18	2.15E-18	2.78E-18	8.09E-20	4.75E-19	6.17E-18	1.61E-17
6.64E-18	1.06E-17	9.96E-18	1.31E-18	2.76E-18	5.57E-18	3.59E-18	6.57E-19	4.82E-18	5.83E-18	5.56E-18	6.84E-18	2.31E-19	2.93E-18	1.32E-17	2.08E-17
5.32E-19	5.07E-18	1.08E-17	2.18E-18	4.29E-18	8.67E-18	2.95E-18	2.00E-18	5.51E-18	7.07E-18	8.69E-18	1.20E-17	3.20E-18	1.25E-17	1.92E-17	2.54E-17

2.10E-18	8.19E-19	4.70E-18	7.20E-19	4.23E-18	9.01E-18	3.25E-18	3.37E-18	4.92E-18	1.02E-17	1.21E-17	1.57E-17	1.02E-17	1.86E-17	2.11E-17	2.93E-17
2.95E-18	1.06E-19	2.15E-19	7.52E-19	1.36E-18	7.02E-18	5.41E-18	3.89E-18	3.58E-18	1.18E-17	1.71E-17	1.74E-17	1.11E-17	1.92E-17	2.17E-17	2.98E-17
3.62E-21	1.58E-19	6.63E-22	2.25E-18	1.32E-18	7.43E-18	1.11E-17	4.58E-18	4.42E-18	1.58E-17	2.12E-17	1.74E-17	9.83E-18	1.86E-17	1.98E-17	2.88E-17
5.30E-18	2.82E-18	1.16E-18	8.62E-18	5.82E-18	1.23E-17	1.46E-17	9.47E-18	1.24E-17	2.19E-17	2.27E-17	1.80E-17	8.74E-18	1.98E-17	2.39E-17	2.92E-17
1.27E-17	1.08E-17	1.03E-17	1.58E-17	1.77E-17	2.25E-17	2.10E-17	1.69E-17	2.33E-17	2.17E-17	2.08E-17	1.63E-17	1.18E-17	2.20E-17	3.04E-17	2.98E-17
1.60E-17	1.78E-17	1.65E-17	1.90E-17	2.16E-17	2.69E-17	2.52E-17	2.32E-17	2.92E-17	2.44E-17	2.53E-17	1.99E-17	1.88E-17	2.48E-17	3.61E-17	3.95E-17
1.79E-17	2.00E-17	1.83E-17	2.18E-17	2.38E-17	2.86E-17	2.88E-17	2.81E-17	3.17E-17	3.11E-17	3.16E-17	3.32E-17	3.41E-17	3.96E-17	4.06E-17	5.22E-17
1.83E-17	2.07E-17	1.83E-17	2.52E-17	2.67E-17	3.33E-17	3.04E-17	3.13E-17	3.55E-17	3.82E-17	3.87E-17	4.54E-17	4.33E-17	4.19E-17	4.38E-17	5.60E-17
2.21E-17	2.60E-17	2.11E-17	2.52E-17	2.66E-17	3.36E-17	3.24E-17	3.37E-17	3.95E-17	3.72E-17	4.09E-17	4.80E-17	4.84E-17	4.07E-17	4.68E-17	5.95E-17
2.57E-17	3.19E-17	2.29E-17	2.35E-17	2.81E-17	3.26E-17	3.01E-17	3.24E-17	4.10E-17	3.77E-17	4.17E-17	4.61E-17	5.32E-17	4.57E-17	4.97E-17	5.52E-17
2.42E-17	2.96E-17	2.17E-17	2.35E-17	3.16E-17	3.41E-17	2.85E-17	3.87E-17	4.46E-17	3.82E-17	4.35E-17	4.26E-17	4.70E-17	4.93E-17	4.81E-17	5.17E-17
3.67E-15	3.49E-15	3.31E-15	3.30E-15	3.19E-15	3.14E-15	3.08E-15	3.11E-15	3.11E-15	2.81E-15	2.89E-15	2.84E-15	2.78E-15	2.67E-15	2.51E-15	2.71E-15

1.18E-08	1.11E-08	1.13E-08	1.06E-08	1.01E-08	9.97E-09	9.49E-09	9.28E-09	8.02E-09	7.87E-09	7.66E-09	7.60E-09	6.90E-09	6.46E-09	6.39E-09
1.15E-08	1.09E-08	1.07E-08	1.02E-08	9.97E-09	9.17E-09	8.59E-09	8.09E-09	7.44E-09	7.31E-09	6.88E-09	6.82E-09	6.79E-09	6.36E-09	6.00E-09
1.14E-08	1.10E-08	1.05E-08	9.99E-09	9.23E-09	8.58E-09	7.96E-09	7.23E-09	6.83E-09	6.73E-09	6.48E-09	6.42E-09	6.77E-09	5.72E-09	5.45E-09
1.11E-08	1.01E-08	9.76E-09	9.17E-09	8.21E-09	8.39E-09	7.48E-09	7.05E-09	6.61E-09	6.75E-09	6.60E-09	6.34E-09	6.18E-09	5.11E-09	4.85E-09
1.05E-08	8.97E-09	8.03E-09	8.10E-09	7.82E-09	8.22E-09	6.92E-09	6.49E-09	6.14E-09	6.53E-09	6.51E-09	6.06E-09	5.77E-09	4.80E-09	4.83E-09
8.99E-09	8.39E-09	7.41E-09	7.23E-09	7.10E-09	7.03E-09	6.49E-09	5.71E-09	5.82E-09	6.25E-09	6.18E-09	5.47E-09	5.35E-09	4.78E-09	5.07E-09
7.56E-09	7.79E-09	6.67E-09	6.10E-09	6.23E-09	5.98E-09	6.23E-09	5.18E-09	5.39E-09	5.57E-09	6.02E-09	5.09E-09	4.92E-09	5.05E-09	4.99E-09
6.72E-09	6.86E-09	6.27E-09	5.54E-09	5.31E-09	5.23E-09	5.72E-09	4.77E-09	5.08E-09	4.64E-09	6.00E-09	4.84E-09	4.77E-09	4.95E-09	4.51E-09
6.22E-09	6.00E-09	5.92E-09	5.49E-09	4.82E-09	4.71E-09	4.94E-09	4.22E-09	5.29E-09	4.26E-09	5.71E-09	4.69E-09	4.76E-09	4.16E-09	3.80E-09
6.10E-09	5.40E-09	5.35E-09	5.15E-09	4.75E-09	4.89E-09	5.03E-09	4.05E-09	4.71E-09	4.10E-09	5.24E-09	4.38E-09	4.36E-09	3.73E-09	3.45E-09
6.32E-09	5.12E-09	4.66E-09	4.84E-09	5.09E-09	5.32E-09	5.15E-09	4.49E-09	4.03E-09	3.98E-09	4.78E-09	4.22E-09	4.20E-09	3.54E-09	3.33E-09
6.00E-09	5.17E-09	4.54E-09	4.76E-09	5.51E-09	6.44E-09	5.59E-09	4.69E-09	4.00E-09	3.39E-09	3.52E-09	3.79E-09	3.74E-09	3.99E-09	3.31E-09
5.25E-09	5.46E-09	5.23E-09	5.22E-09	5.62E-09	6.43E-09	5.86E-09	5.07E-09	4.35E-09	2.90E-09	2.68E-09	3.66E-09	3.61E-09	4.13E-09	3.53E-09
5.15E-09	5.63E-09	5.11E-09	5.34E-09	5.47E-09	5.23E-09	5.40E-09	5.36E-09	4.56E-09	2.79E-09	2.44E-09	3.40E-09	3.49E-09	3.72E-09	3.30E-09
4.85E-09	4.76E-09	4.46E-09	4.76E-09	4.93E-09	4.08E-09	4.57E-09	4.83E-09	4.05E-09	2.93E-09	2.49E-09	3.31E-09	3.33E-09	3.49E-09	3.22E-09
4.70E-09	4.09E-09	4.23E-09	4.49E-09	4.67E-09	3.47E-09	3.92E-09	3.84E-09	3.40E-09	3.09E-09	2.75E-09	3.17E-09	3.28E-09	3.44E-09	3.05E-09
4.20E-09	3.60E-09	3.80E-09	4.22E-09	4.09E-09	3.11E-09	3.39E-09	3.24E-09	3.08E-09	2.96E-09	2.71E-09	3.48E-09	3.51E-09	3.30E-09	3.07E-09
3.19E-09	3.71E-09	3.70E-09	3.79E-09	3.83E-09	2.54E-09	3.00E-09	3.36E-09	3.19E-09	2.98E-09	3.10E-09	3.85E-09	4.00E-09	3.75E-09	3.20E-09
2.95E-09	3.54E-09	3.67E-09	3.65E-09	4.28E-09	2.84E-09	3.07E-09	3.69E-09	3.51E-09	3.49E-09	3.73E-09	4.87E-09	4.47E-09	4.53E-09	3.96E-09
3.34E-09	3.29E-09	4.05E-09	3.72E-09	4.27E-09	3.68E-09	3.29E-09	4.20E-09	3.96E-09	3.89E-09	4.25E-09	5.91E-09	5.06E-09	4.95E-09	4.25E-09
3.66E-09	3.33E-09	4.55E-09	3.57E-09	3.96E-09	4.17E-09	3.05E-09	4.44E-09	4.70E-09	4.64E-09	4.62E-09	6.21E-09	5.75E-09	5.64E-09	4.48E-09
3.51E-09	3.60E-09	4.14E-09	3.40E-09	3.72E-09	3.99E-09	3.08E-09	4.70E-09	5.55E-09	5.19E-09	4.77E-09	5.47E-09	6.11E-09	5.91E-09	4.45E-09
3.57E-09	3.62E-09	4.16E-09	3.43E-09	3.49E-09	3.34E-09	3.44E-09	5.41E-09	5.50E-09	5.03E-09	5.00E-09	4.48E-09	5.57E-09	5.08E-09	4.43E-09
3.39E-09	3.81E-09	4.36E-09	3.66E-09	3.23E-09	3.28E-09	3.67E-09	5.09E-09	4.66E-09	4.40E-09	4.23E-09	3.89E-09	4.45E-09	3.88E-09	4.22E-09
3.39E-09	3.26E-09	3.88E-09	4.12E-09	3.37E-09	3.42E-09	3.82E-09	4.83E-09	4.45E-09	4.02E-09	3.57E-09	3.50E-09	4.13E-09	3.72E-09	4.10E-09
3.23E-09	2.70E-09	3.13E-09	3.85E-09	3.45E-09	3.78E-09	4.00E-09	4.66E-09	4.65E-09	3.60E-09	3.82E-09	3.86E-09	4.25E-09	3.87E-09	4.08E-09
3.45E-09	2.69E-09	3.07E-09	3.48E-09	3.44E-09	4.13E-09	4.06E-09	4.11E-09	4.19E-09	3.80E-09	4.16E-09	4.69E-09	4.50E-09	3.95E-09	4.70E-09
4.11E-09	3.23E-09	3.27E-09	3.71E-09	3.83E-09	4.34E-09	4.11E-09	3.54E-09	3.91E-09	4.33E-09	4.70E-09	4.99E-09	4.89E-09	4.16E-09	5.44E-09
4.08E-09	3.71E-09	3.73E-09	3.84E-09	3.96E-09	4.37E-09	4.13E-09	3.78E-09	3.98E-09	4.72E-09	5.16E-09	4.90E-09	5.09E-09	4.69E-09	5.57E-09
3.87E-09	4.07E-09	4.28E-09	4.19E-09	3.88E-09	4.14E-09	4.30E-09	3.99E-09	4.16E-09	4.52E-09	5.37E-09	5.01E-09	5.38E-09	5.28E-09	4.87E-09
4.01E-09	4.46E-09	4.54E-09	4.26E-09	3.73E-09	4.26E-09	4.20E-09	4.17E-09	4.08E-09	4.24E-09	5.55E-09	5.71E-09	5.98E-09	5.72E-09	4.95E-09
4.24E-09	4.85E-09	4.51E-09	4.69E-09	4.36E-09	4.79E-09	4.84E-09	4.22E-09	3.92E-09	4.50E-09	5.74E-09	6.17E-09	6.59E-09	5.91E-09	5.32E-09
3.87E-09	4.46E-09	4.52E-09	5.63E-09	4.91E-09	5.68E-09	5.50E-09	4.72E-09	4.13E-09	4.69E-09	5.76E-09	6.26E-09	6.54E-09	5.51E-09	5.63E-09
4.37E-09	4.26E-09	4.74E-09	6.05E-09	5.19E-09	6.32E-09	6.26E-09	5.55E-09	4.88E-09	5.02E-09	5.44E-09	6.09E-09	5.88E-09	5.59E-09	5.81E-09
5.01E-09	4.64E-09	5.06E-09	6.18E-09	5.67E-09	6.81E-09	6.64E-09	5.97E-09	5.24E-09	5.12E-09	5.38E-09	6.34E-09	5.48E-09	5.79E-09	6.28E-09
5.68E-09	4.84E-09	5.36E-09	6.59E-09	6.41E-09	7.25E-09	6.67E-09	6.23E-09	5.29E-09	5.38E-09	5.70E-09	6.02E-09	6.00E-09	6.16E-09	6.38E-09
4.25E-09	4.84E-09	5.55E-09	6.39E-09	7.00E-09	7.07E-09	6.51E-09	6.47E-09	5.91E-09	5.64E-09	6.04E-09	5.85E-09	5.63E-09	5.81E-09	6.52E-09
4.14E-09	4.60E-09	5.62E-09	5.41E-09	5.79E-09	6.36E-09	6.30E-09	6.67E-09	6.61E-09	6.37E-09	6.61E-09	6.09E-09	5.35E-09	5.76E-09	6.74E-09
4.30E-09	4.74E-09	4.95E-09	5.08E-09	5.02E-09	5.94E-09	6.11E-09	6.17E-09	6.84E-09	7.10E-09	7.14E-09	6.07E-09	5.54E-09	5.96E-09	6.73E-09
4.92E-09	5.15E-09	4.25E-09	5.09E-09	4.83E-09	5.88E-09	5.59E-09	5.57E-09	6.49E-09	7.42E-09	7.44E-09	6.55E-09	5.95E-09	6.44E-09	6.67E-09
4.83E-09	5.15E-09	4.45E-09	4.87E-09	4.53E-09	5.52E-09	5.59E-09	5.39E-09	6.06E-09	6.75E-09	6.97E-09	6.51E-09	6.43E-09	6.46E-09	6.92E-09
3.97E-09	4.84E-09	4.42E-09	4.54E-09	4.23E-09	5.58E-09	5.41E-09	5.66E-09	6.21E-09	6.78E-09	6.94E-09	6.85E-09	7.05E-09	6.76E-09	7.26E-09
3.80E-09	4.65E-09	4.36E-09	4.49E-09	4.34E-09	5.51E-09	5.60E-09	6.01E-09	6.71E-09	6.65E-09	6.93E-09	6.80E-09	7.08E-09	7.21E-09	7.78E-09
3.93E-09	4.41E-09	4.27E-09	4.69E-09	4.41E-09	5.36E-09	5.52E-09	6.15E-09	7.07E-09	6.74E-09	6.65E-09	6.65E-09	7.02E-09	7.19E-09	8.08E-09
3.81E-09	4.45E-09	4.41E-09	4.89E-09	4.66E-09	5.46E-09	5.27E-09	6.34E-09	7.49E-09	6.96E-09	6.26E-09	6.46E-09	7.33E-09	7.55E-09	8.39E-09
3.68E-09	4.41E-09	4.44E-09	5.51E-09	4.88E-09	5.68E-09	4.96E-09	6.33E-09	7.16E-09	7.27E-09	6.57E-09	6.62E-09	6.87E-09	7.42E-09	8.17E-09
3.42E-09	4.32E-09	4.63E-09	6.08E-09	5.70E-09	5.83E-09	4.74E-09	5.92E-09	6.27E-09	7.59E-09	6.77E-09	6.18E-09	6.55E-09	7.15E-09	8.26E-09
3.67E-09	4.64E-09	4.66E-09	6.21E-09	6.83E-09	5.97E-09	5.29E-09	5.53E-09	5.75E-09	6.56E-09	6.57E-09	5.76E-09	6.52E-09	7.64E-09	8.56E-09
4.16E-09	4.78E-09	4.42E-09	5.84E-09	7.45E-09	6.32E-09	5.39E-09	5.36E-09	5.47E-09	6.33E-09	6.11E-09	5.80E-09	6.75E-09	8.11E-09	8.86E-09
4.51E-09	4.94E-09	4.27E-09	5.38E-09	6.82E-09	5.76E-09	5.21E-09	5.16E-09	5.22E-09	6.28E-09	6.31E-09	6.05E-09	7.18E-09	8.15E-09	8.36E-09
3.95E-09	4.40E-09	3.95E-09	4.47E-09	6.19E-09	4.90E-09	5.01E-09	5.36E-09	5.53E-09	6.64E-09	6.72E-09	6.47E-09	7.32E-09	7.35E-09	7.47E-09
3.13E-09	3.72E-09	4.15E-09	4.04E-09	5.95E-09	4.36E-09	4.83E-09	5.19E-09	5.90E-09	7.25E-09	7.12E-09	7.11E-09	7.09E-09	6.97E-09	7.22E-09
2.79E-09	3.35E-09	4.06E-09	4.01E-09	4.96E-09	4.34E-09	4.43E-09	5.09E-09	6.33E-09	7.84E-09	7.18E-09	7.55E-09	6.92E-09	6.80E-09	7.29E-09
2.90E-09	3.45E-09	4.26E-09	3.90E-09	4.10E-09	4.51E-09	4.61E-09	4.83E-09	6.88E-09	7.74E-09	6.36E-09	7.15E-09	7.08E-09	6.92E-09	7.16E-09
3.31E-09	3.61E-09	3.68E-09	3.44E-09	3.84E-09	4.83E-09	5.05E-09	4.42E-09	6.43E-09	6.68E-09	5.52E-09	6.40E-09	7.27E-09	7.32E-09	6.72E-09
3.42E-09	3.82E-09	3.43E-09	3.08E-09	4.20E-09	4.93E-09	5.05E-09	4.15E-09	5.41E-09	5.63E-09	5.01E-09	5.68E-09	6.88E-09	7.41E-09	6.62E-09
2.98E-09	3.72E-09	3.73E-09	3.65E-09	4.33E-09	4.87E-09	5.01E-09	4.43E-09	5.03E-09	5.23E-09	5.17E-09	5.62E-09	6.37E-09	7.31E-09	6.62E-09
2.79E-09	3.34E-09	3.61E-09	4.33E-09	4.51E-09	4.33E-09	5.24E-09	4.58E-09	4.60E-09	5.27E-09	5.68E-09	5.94E-09	6.08E-09	6.83E-09	6.75E-09
3.02E-09	3.98E-09	4.74E-09	4.99E-09	5.50E-09	4.85E-09	4.90E-09	4.39E-09	4.59E-09	5.36E-09	5.88E-09	6.12E-09	5.67E-09	6.34E-09	6.85E-09
4.00E-09	5.37E-09	6.05E-09	5.63E-09	6.20E-09	5.29E-09	4.99E-09	4.91E-09	4.74E-09	5.36E-09	5.74E-09	5.95E-09	5.78E-09	6.52E-09	6.89E-09
4.56E-09	5.85E-09	7.23E-09	7.11E-09	7.02E-09	6.04E-09	6.21E-09	5.65E-09	5.13E-09	5.45E-09	5.73E-09	5.88E-09	6.03E-09	6.79E-09	6.81E-09
4.07E-09	5.85E-09	6.84E-09	7.51E-09	7.21E-09	6.82E-09	7.94E-09	6.78E-09	5.15E-09	5.35E-09	5.50E-09	5.73E-09	6.37E-09	6.28E-09	7.04E-09
3.79E-09	5.02E-09	6.11E-09	6.75E-09	6.66E-09	7.73E-09	8.95E-09	7.32E-09	5.26E-09	5.27E-09	5.48E-09	5.60E-09	6.23E-09	6.08E-09	7.37E-09
4.20E-09	4.65E-09	5.78E-09	6.73E-09	6.90E-09	8.31E-09	8.59E-09	7.17E-09	5.02E-09	4.87E-09	5.54E-09	5.62E-09	5.51E-09	6.06E-09	6.89E-09
5.10E-09	5.40E-09	6.31E-09	7.16E-09	6.67E-09	7.84E-09	7.19E-09	6.34E-09	4.84E-09	4.77E-09	5.50E-09	5.64E-09	5.00E-09	5.80E-09	6.66E-09
5.63E-09	6.45E-09	7.34E-09	6.59E-09	5.47E-09	6.89E-09	6.27E-09	5.86E-09	4.79E-09	4.58E-09	5.02E-09	5.58E-09	4.88E-09	6.03E-09	6.36E-09
5.94E-09	6.64E-09	6.79E-09	5.18E-09	3.71E-09	5.35E-09	6.35E-09	5.50E-09	4.72E-09	4.22E-09	4.93E-09	5.68E-09	5.17E-09	6.21E-09	6.21E-09
5.46E-09	5.52E-09	5.50E-09	4.21E-09	2.61E-09	3.82E-09	5.16E-09	4.61E-09	4.38E-09	4.41E-09	5.19E-09	5.48E-09	5.52E-09	6.06E-09	5.82E-09
5.49E-09	4.67E-09	5.56E-09	4.46E-09	2.65E-09	3.23E-09	4.16E-09	4.07E-09	4.45E-09	4.98E-09	5.67E-09	5.56E-09	5.84E-09	5.51E-09	5.47E-09
5.33E-09	4.59E-09	5.62E-09	4.62E-09	3.08E-09	3.32E-09	4.89E-09	4.60E-09	5.23E-09	5.40E-09	5.66E-09	6.07E-09	5.83E-09	5.48E-09	5.41E-09
3.42E-09	4.21E-09	5.38E-09	4.55E-09	3.96E-09	3.84E-09	5.66E-09	5.20E-09	6.54E-09	5.76E-09	5.70E-09	6.48E-09	5.60E-09	5.81E-09	5.87E-09
2.75E-09	3.90E-09	6.02E-09	4.59E-09	4.11E-09	3.86E-09	5.76E-09	5.38E-09	7.46E-09	6.53E-09	5.29E-09	6.54E-09	5.63E-09	6.13E-09	6.01E-09
2.70E-09	3.94E-09	5.89E-09	4.52E-09	4.19E-09	3.84E-09	4.33E-09	5.07E-09	6.18E-09	6.20E-09	4.53E-09	5.33E-09	5.24E-09	6.39E-09	5.65E-09
3.54E-09	4.26E-09	5.06E-09	4.18E-09	3.84E-09	3.01E-09	3.44E-09	5.02E-09	4.59E-09	5.34E-09	4.13E-09	4.43E-09	4.77E-09	5.93E-09	4.81E-09
4.62E-09	6.11E-09	4.80E-09	3.58E-09	2.80E-09	2.42E-09	2.99E-09	4.13E-09	2.83E-09	3.93E-09	3.85E-09	4.19E-09	5.07E-09	5.54E-09	4.93E-09
5.89E-09	7.57E-09	4.69E-09	2.71E-09	2.43E-09	2.28E-09	2.82E-09	3.04E-09	2.15E-09	3.55E-09	4.21E-09	4.65E-09	5.70E-09	5.71E-09	5.20E-09
5.93E-09	6.47E-09	4.01E-09	2.84E-09	2.35E-09	1.79E-09	2.76E-09	3.20E-09	2.65E-09	4.46E-09	5.24E-09	5.04E-09	5.08E-09	5.38E-09	4.76E-09
4.91E-09	3.98E-09	3.26E-09	2.60E-09	2.47E-09	2.13E-09	2.61E-09	3.52E-09	3.06E-09	4.90E-09	6.22E-09	5.06E-09	4.54E-09	4.80E-09	4.20E-09

3.50E-09	2.55E-09	2.86E-09	2.52E-09	2.54E-09	2.61E-09	2.80E-09	3.70E-09	3.40E-09	4.37E-09	5.32E-09	4.67E-09	3.69E-09	4.14E-09	3.90E-09
3.05E-09	2.26E-09	3.07E-09	3.61E-09	3.52E-09	2.85E-09	3.67E-09	4.27E-09	4.06E-09	4.27E-09	4.10E-09	4.59E-09	3.70E-09	3.55E-09	3.53E-09
2.56E-09	1.68E-09	3.15E-09	4.50E-09	4.33E-09	3.55E-09	4.78E-09	4.87E-09	5.21E-09	4.67E-09	4.27E-09	4.70E-09	4.35E-09	3.50E-09	3.77E-09
1.91E-09	1.45E-09	3.27E-09	5.02E-09	4.32E-09	3.94E-09	5.76E-09	4.95E-09	5.71E-09	4.70E-09	4.63E-09	5.00E-09	5.04E-09	3.76E-09	4.05E-09
1.03E-09	1.34E-09	3.66E-09	4.74E-09	4.21E-09	4.28E-09	4.95E-09	3.96E-09	4.75E-09	4.18E-09	4.51E-09	5.22E-09	5.05E-09	3.83E-09	3.97E-09
6.31E-10	1.29E-09	2.79E-09	3.84E-09	4.55E-09	4.40E-09	3.10E-09	3.01E-09	3.51E-09	3.49E-09	3.76E-09	4.44E-09	4.58E-09	3.93E-09	3.81E-09
5.05E-10	1.38E-09	1.96E-09	3.36E-09	3.92E-09	3.60E-09	1.75E-09	1.87E-09	2.39E-09	2.18E-09	3.17E-09	3.57E-09	3.87E-09	3.51E-09	3.34E-09
9.40E-10	7.98E-10	7.36E-10	2.63E-09	3.45E-09	3.00E-09	1.18E-09	1.28E-09	1.69E-09	1.94E-09	2.92E-09	3.28E-09	3.32E-09	3.28E-09	3.48E-09
1.38E-09	4.65E-10	-2.45E-10	1.49E-09	2.87E-09	3.00E-09	1.10E-09	1.26E-09	1.65E-09	2.03E-09	2.75E-09	2.88E-09	2.56E-09	3.07E-09	3.30E-09
1.81E-09	4.49E-10	-5.73E-10	8.24E-10	2.53E-09	3.05E-09	1.53E-09	9.31E-10	1.40E-09	2.26E-09	2.76E-09	2.38E-09	2.04E-09	3.19E-09	2.88E-09
2.30E-09	1.09E-09	-4.47E-10	1.16E-09	2.79E-09	3.74E-09	2.11E-09	7.80E-10	1.22E-09	2.22E-09	2.56E-09	1.99E-09	2.32E-09	3.50E-09	2.72E-09
2.43E-09	1.77E-09	-2.78E-11	1.43E-09	2.43E-09	3.42E-09	2.32E-09	8.35E-10	1.17E-09	1.56E-09	1.99E-09	1.54E-09	2.61E-09	3.53E-09	2.86E-09
2.34E-09	1.68E-09	3.18E-10	1.22E-09	2.19E-09	2.47E-09	1.72E-09	4.06E-10	1.05E-09	1.43E-09	2.18E-09	1.79E-09	2.91E-09	2.82E-09	2.71E-09
9.74E-10	1.02E-09	8.43E-10	1.80E-09	2.45E-09	1.29E-09	-2.28E-10	-1.18E-10	8.39E-10	1.59E-09	2.53E-09	2.18E-09	2.77E-09	2.36E-09	2.51E-09
3.33E-10	1.32E-09	1.43E-09	2.20E-09	2.28E-09	3.08E-10	-4.18E-10	-2.18E-10	1.28E-09	1.79E-09	2.36E-09	2.11E-09	2.55E-09	2.08E-09	2.22E-09
3.40E-10	2.15E-09	1.39E-09	1.63E-09	1.22E-09	-2.00E-10	-1.31E-10	4.42E-10	2.12E-09	1.96E-09	2.58E-09	1.83E-09	2.31E-09	1.73E-09	1.75E-09
-5.42E-11	1.33E-09	1.07E-09	4.85E-10	2.08E-10	2.18E-11	4.35E-10	1.33E-09	2.76E-09	2.32E-09	2.71E-09	1.56E-09	2.21E-09	1.84E-09	1.45E-09
-5.52E-10	1.90E-10	6.20E-10	-4.65E-10	-2.45E-10	4.54E-10	9.70E-10	2.15E-09	2.77E-09	2.45E-09	2.57E-09	1.78E-09	2.76E-09	2.75E-09	2.09E-09
-3.91E-10	1.07E-10	3.10E-10	-6.93E-10	-3.51E-10	7.24E-10	1.54E-09	2.78E-09	2.85E-09	1.74E-09	2.32E-09	2.04E-09	3.25E-09	3.73E-09	3.22E-09
4.08E-10	3.80E-10	3.06E-10	-3.54E-10	3.80E-11	1.29E-09	2.56E-09	3.56E-09	3.64E-09	2.18E-09	2.59E-09	2.20E-09	3.44E-09	3.92E-09	3.41E-09
7.46E-10	3.88E-10	3.67E-11	-8.01E-10	3.30E-10	1.95E-09	3.86E-09	4.93E-09	5.15E-09	3.54E-09	3.47E-09	2.80E-09	3.04E-09	3.19E-09	2.57E-09
4.59E-10	2.78E-11	-4.85E-10	-4.71E-10	7.43E-10	2.30E-09	5.37E-09	7.33E-09	7.76E-09	5.14E-09	3.91E-09	3.15E-09	2.62E-09	2.84E-09	2.22E-09
1.03E-09	-5.23E-10	-4.05E-10	4.71E-10	1.36E-09	2.64E-09	5.94E-09	9.94E-09	1.05E-08	6.68E-09	3.85E-09	2.92E-09	2.40E-09	2.06E-09	1.59E-09
8.92E-10	-3.38E-10	-4.14E-10	8.74E-10	1.66E-09	3.02E-09	5.74E-09	9.92E-09	1.03E-08	6.70E-09	3.41E-09	2.40E-09	2.00E-09	1.23E-09	1.41E-09
7.15E-10	-3.33E-10	-1.00E-09	3.50E-11	1.15E-09	2.91E-09	3.75E-09	5.82E-09	6.86E-09	4.37E-09	2.33E-09	1.54E-09	1.24E-09	5.50E-10	9.27E-10
9.30E-11	-7.55E-10	-1.21E-09	-4.05E-10	2.99E-10	1.82E-09	2.11E-09	3.19E-09	3.40E-09	1.93E-09	9.65E-10	5.88E-10	4.16E-10	1.02E-10	4.16E-10
5.42E-10	-7.65E-11	-1.23E-09	-8.89E-10	-6.44E-10	7.58E-10	1.28E-09	2.11E-09	1.15E-09	3.70E-10	-6.71E-10	-7.24E-10	-4.96E-10	-2.03E-10	7.63E-12
1.45E-09	8.27E-10	-9.65E-10	-9.89E-10	-1.05E-09	1.29E-10	3.93E-10	5.21E-10	-6.77E-10	-6.22E-10	-1.55E-09	-1.01E-09	-8.79E-10	-2.41E-10	-6.15E-11
2.37E-09	1.67E-09	-3.04E-10	-1.12E-09	-9.67E-10	-7.70E-11	2.59E-12	2.03E-11	-1.57E-09	-9.21E-10	-1.38E-09	-1.16E-09	-4.07E-10	-1.96E-10	-4.79E-10
2.48E-09	2.66E-09	6.44E-10	-8.05E-10	-1.43E-09	-1.39E-09	-4.15E-10	-2.04E-10	-1.87E-09	-1.28E-09	-1.38E-09	-1.39E-09	-3.12E-10	-9.98E-10	-6.31E-10
3.15E-09	2.33E-09	1.52E-09	-4.17E-10	-1.39E-09	-1.94E-09	-1.24E-09	-8.48E-10	-2.06E-09	-1.31E-09	-1.15E-09	-2.04E-09	-8.40E-10	-1.91E-09	-1.13E-09
2.81E-09	1.25E-09	1.57E-09	-5.86E-10	-7.55E-10	-2.00E-09	-1.93E-09	-1.11E-09	-2.22E-09	-1.82E-09	-1.51E-09	-2.18E-09	-1.86E-09	-2.51E-09	-2.11E-09
1.53E-09	1.54E-11	1.84E-10	-1.56E-09	-1.07E-09	-1.80E-09	-2.38E-09	-1.01E-09	-1.77E-09	-2.46E-09	-2.17E-09	-2.18E-09	-2.66E-09	-2.89E-09	-2.61E-09
-3.02E-10	-1.55E-09	-7.62E-10	-2.14E-09	-1.54E-09	-1.27E-09	-2.41E-09	-1.47E-09	-1.76E-09	-2.73E-09	-2.66E-09	-2.63E-09	-2.90E-09	-3.28E-09	-2.57E-09
-1.42E-09	-2.37E-09	-1.41E-09	-1.85E-09	-1.51E-09	-1.32E-09	-2.49E-09	-1.93E-09	-3.00E-09	-2.90E-09	-2.87E-09	-3.20E-09	-3.41E-09	-3.84E-09	-2.93E-09
-2.51E-09	-3.14E-09	-2.30E-09	-1.93E-09	-2.40E-09	-2.52E-09	-3.45E-09	-2.73E-09	-3.61E-09	-3.12E-09	-3.16E-09	-3.70E-09	-4.00E-09	-4.35E-09	-3.62E-09
-3.81E-09	-3.62E-09	-2.30E-09	-1.69E-09	-3.04E-09	-4.43E-09	-4.24E-09	-4.21E-09	-3.86E-09	-3.53E-09	-3.34E-09	-4.43E-09	-4.78E-09	-4.59E-09	-4.28E-09
-4.44E-09	-3.99E-09	-2.78E-09	-2.41E-09	-3.68E-09	-5.38E-09	-5.02E-09	-5.23E-09	-4.26E-09	-3.90E-09	-4.51E-09	-5.17E-09	-5.48E-09	-4.76E-09	-5.31E-09
-4.97E-09	-4.20E-09	-4.06E-09	-3.69E-09	-4.58E-09	-5.77E-09	-5.49E-09	-5.74E-09	-5.15E-09	-4.64E-09	-5.25E-09	-5.17E-09	-5.89E-09	-4.94E-09	-5.56E-09
-5.61E-09	-4.96E-09	-4.70E-09	-4.75E-09	-5.47E-09	-6.06E-09	-6.32E-09	-6.44E-09	-5.76E-09	-5.31E-09	-5.36E-09	-4.83E-09	-5.86E-09	-5.26E-09	-5.32E-09
-5.63E-09	-5.46E-09	-5.34E-09	-5.69E-09	-5.82E-09	-6.04E-09	-6.59E-09	-6.69E-09	-6.39E-09	-5.94E-09	-5.65E-09	-5.10E-09	-6.01E-09	-5.70E-09	-5.52E-09
-5.32E-09	-5.84E-09	-6.05E-09	-6.39E-09	-6.11E-09	-6.10E-09	-6.63E-09	-7.06E-09	-6.75E-09	-6.76E-09	-6.21E-09	-5.69E-09	-6.46E-09	-5.94E-09	-6.29E-09
-4.86E-09	-6.35E-09	-6.27E-09	-6.99E-09	-6.39E-09	-6.60E-09	-6.91E-09	-7.51E-09	-7.29E-09	-7.08E-09	-6.39E-09	-5.93E-09	-6.63E-09	-6.49E-09	-6.67E-09
-5.53E-09	-6.53E-09	-6.41E-09	-7.37E-09	-6.99E-09	-7.28E-09	-6.99E-09	-8.03E-09	-7.53E-09	-6.65E-09	-6.78E-09	-6.50E-09	-6.57E-09	-6.72E-09	-7.12E-09
-6.54E-09	-6.74E-09	-6.67E-09	-7.21E-09	-6.87E-09	-7.39E-09	-7.34E-09	-8.49E-09	-7.67E-09	-6.34E-09	-7.06E-09	-6.77E-09	-7.07E-09	-6.75E-09	-7.53E-09
-6.92E-09	-7.10E-09	-6.77E-09	-7.47E-09	-7.18E-09	-7.30E-09	-7.67E-09	-8.60E-09	-7.50E-09	-6.56E-09	-7.05E-09	-7.18E-09	-7.52E-09	-7.11E-09	-7.56E-09
-7.40E-09	-7.43E-09	-7.05E-09	-7.61E-09	-7.34E-09	-7.75E-09	-7.84E-09	-8.69E-09	-7.41E-09	-6.68E-09	-7.26E-09	-7.16E-09	-7.50E-09	-7.39E-09	-7.05E-09
-7.99E-09	-8.16E-09	-7.68E-09	-7.85E-09	-7.16E-09	-7.79E-09	-7.86E-09	-8.44E-09	-8.03E-09	-7.38E-09	-8.03E-09	-7.63E-09	-7.82E-09	-7.83E-09	-6.83E-09
-7.57E-09	-8.10E-09	-7.93E-09	-7.66E-09	-7.00E-09	-7.88E-09	-7.97E-09	-8.65E-09	-8.14E-09	-7.75E-09	-8.80E-09	-8.18E-09	-8.16E-09	-7.91E-09	-6.85E-09
-6.92E-09	-7.79E-09	-8.10E-09	-7.89E-09	-7.33E-09	-8.52E-09	-8.55E-09	-8.81E-09	-8.05E-09	-7.99E-09	-9.00E-09	-8.38E-09	-8.17E-09	-7.83E-09	-7.09E-09

1.38E-16	1.23E-16	1.28E-16	1.11E-16	1.02E-16	9.93E-17	9.01E-17	8.62E-17	6.43E-17	6.19E-17	5.86E-17	5.78E-17	4.77E-17	4.17E-17	4.09E-17
1.32E-16	1.19E-16	1.14E-16	1.05E-16	9.95E-17	8.41E-17	7.38E-17	6.54E-17	5.53E-17	5.34E-17	4.74E-17	4.65E-17	4.61E-17	4.05E-17	3.60E-17
1.29E-16	1.20E-16	1.11E-16	9.99E-17	8.52E-17	7.36E-17	6.34E-17	5.22E-17	4.67E-17	4.53E-17	4.20E-17	4.12E-17	4.59E-17	3.27E-17	2.97E-17
1.24E-16	1.03E-16	9.52E-17	8.40E-17	6.74E-17	7.03E-17	5.59E-17	4.97E-17	4.36E-17	4.55E-17	4.35E-17	4.02E-17	3.81E-17	2.61E-17	2.35E-17
1.10E-16	8.04E-17	6.44E-17	6.57E-17	6.12E-17	6.76E-17	4.78E-17	4.21E-17	3.77E-17	4.27E-17	4.24E-17	3.67E-17	3.33E-17	2.30E-17	2.33E-17
8.08E-17	7.03E-17	5.49E-17	5.23E-17	5.04E-17	4.95E-17	4.22E-17	3.26E-17	3.39E-17	3.91E-17	3.82E-17	2.99E-17	2.86E-17	2.29E-17	2.57E-17
5.72E-17	6.07E-17	4.45E-17	3.72E-17	3.89E-17	3.58E-17	3.88E-17	2.68E-17	2.91E-17	3.10E-17	3.62E-17	2.59E-17	2.42E-17	2.55E-17	2.49E-17
4.52E-17	4.71E-17	3.93E-17	3.07E-17	2.82E-17	2.73E-17	3.28E-17	2.27E-17	2.58E-17	2.15E-17	3.60E-17	2.34E-17	2.27E-17	2.45E-17	2.04E-17
3.87E-17	3.60E-17	3.50E-17	3.02E-17	2.32E-17	2.22E-17	2.44E-17	1.78E-17	2.79E-17	1.82E-17	3.26E-17	2.20E-17	2.27E-17	1.73E-17	1.45E-17
3.72E-17	2.92E-17	2.87E-17	2.65E-17	2.25E-17	2.39E-17	2.53E-17	1.64E-17	2.22E-17	1.68E-17	2.75E-17	1.92E-17	1.90E-17	1.39E-17	1.19E-17
4.00E-17	2.62E-17	2.17E-17	2.34E-17	2.60E-17	2.83E-17	2.65E-17	2.01E-17	1.63E-17	1.58E-17	2.28E-17	1.78E-17	1.76E-17	1.25E-17	1.11E-17
3.60E-17	2.68E-17	2.06E-17	2.27E-17	3.03E-17	4.15E-17	3.13E-17	2.20E-17	1.60E-17	1.15E-17	1.24E-17	1.44E-17	1.40E-17	1.59E-17	1.09E-17
2.76E-17	2.98E-17	2.73E-17	2.73E-17	3.16E-17	4.14E-17	3.43E-17	2.57E-17	1.90E-17	8.43E-18	7.17E-18	1.34E-17	1.30E-17	1.70E-17	1.24E-17
2.65E-17	3.17E-17	2.61E-17	2.85E-17	3.00E-17	2.73E-17	2.91E-17	2.88E-17	2.08E-17	7.80E-18	5.97E-18	1.15E-17	1.22E-17	1.39E-17	1.09E-17
2.36E-17	2.26E-17	1.99E-17	2.27E-17	2.44E-17	1.67E-17	2.09E-17	2.33E-17	1.64E-17	8.59E-18	6.21E-18	1.10E-17	1.11E-17	1.22E-17	1.03E-17
2.21E-17	1.68E-17	1.79E-17	2.02E-17	2.18E-17	1.21E-17	1.54E-17	1.48E-17	1.16E-17	9.53E-18	7.58E-18	1.00E-17	1.08E-17	1.19E-17	9.31E-18
1.76E-17	1.29E-17	1.44E-17	1.78E-17	1.67E-17	9.65E-18	1.15E-17	1.05E-17	9.46E-18	8.75E-18	7.32E-18	1.21E-17	1.23E-17	1.09E-17	9.44E-18
1.02E-17	1.37E-17	1.37E-17	1.44E-17	1.47E-17	6.46E-18	8.98E-18	1.13E-17	1.02E-17	8.91E-18	9.63E-18	1.48E-17	1.60E-17	1.41E-17	1.02E-17
8.72E-18	1.25E-17	1.35E-17	1.33E-17	1.84E-17	8.06E-18	9.42E-18	1.36E-17	1.23E-17	1.22E-17	1.39E-17	2.37E-17	2.00E-17	2.05E-17	1.57E-17
1.11E-17	1.09E-17	1.64E-17	1.38E-17	1.82E-17	1.36E-17	1.08E-17	1.76E-17	1.57E-17	1.51E-17	1.80E-17	3.50E-17	2.56E-17	2.45E-17	1.81E-17
1.34E-17	1.11E-17	2.07E-17	1.27E-17	1.57E-17	1.74E-17	9.29E-18	1.98E-17	2.21E-17	2.15E-17	2.14E-17	3.86E-17	3.31E-17	3.18E-17	2.01E-17
1.23E-17	1.29E-17	1.72E-17	1.15E-17	1.39E-17	1.59E-17	9.49E-18	2.21E-17	3.08E-17	2.69E-17	2.28E-17	2.99E-17	3.73E-17	3.49E-17	1.98E-17
1.27E-17	1.31E-17	1.73E-17	1.18E-17	1.22E-17	1.12E-17	1.18E-17	2.92E-17	3.03E-17	2.53E-17	2.50E-17	2.00E-17	3.10E-17	2.58E-17	1.96E-17
1.15E-17	1.45E-17	1.90E-17	1.34E-17	1.04E-17	1.08E-17	1.35E-17	2.59E-17	2.17E-17	1.93E-17	1.79E-17	1.51E-17	1.98E-17	1.51E-17	1.78E-17
1.15E-17	1.07E-17	1.51E-17	1.70E-17	1.13E-17	1.17E-17	1.46E-17	2.33E-17	1.98E-17	1.61E-17	1.28E-17	1.22E-17	1.71E-17	1.38E-17	1.68E-17
1.04E-17	7.29E-18	9.82E-18	1.48E-17	1.19E-17	1.43E-17	1.60E-17	2.17E-17	2.16E-17	1.30E-17	1.46E-17	1.49E-17	1.81E-17	1.50E-17	1.67E-17
1.19E-17	7.22E-18	9.40E-18	1.21E-17	1.18E-17	1.71E-17	1.65E-17	1.69E-17	1.75E-17	1.44E-17	1.73E-17	2.20E-17	2.03E-17	1.56E-17	2.21E-17
1.69E-17	1.05E-17	1.07E-17	1.37E-17	1.47E-17	1.89E-17	1.69E-17	1.25E-17	1.53E-17	1.87E-17	2.21E-17	2.49E-17	2.39E-17	1.73E-17	2.96E-17
1.67E-17	1.38E-17	1.39E-17	1.47E-17	1.57E-17	1.91E-17	1.70E-17	1.43E-17	1.59E-17	2.23E-17	2.66E-17	2.40E-17	2.59E-17	2.20E-17	3.10E-17
1.50E-17	1.66E-17	1.84E-17	1.76E-17	1.50E-17	1.72E-17	1.85E-17	1.60E-17	1.73E-17	2.04E-17	2.89E-17	2.51E-17	2.90E-17	2.78E-17	2.37E-17
1.60E-17	1.99E-17	2.07E-17	1.82E-17	1.39E-17	1.81E-17	1.77E-17	1.74E-17	1.67E-17	1.79E-17	3.08E-17	3.26E-17	3.58E-17	3.27E-17	2.45E-17

1.80E-17	2.35E-17	2.03E-17	2.20E-17	1.90E-17	2.30E-17	2.34E-17	1.78E-17	1.53E-17	2.02E-17	3.29E-17	3.81E-17	4.34E-17	3.50E-17	2.83E-17
1.50E-17	1.99E-17	2.04E-17	3.17E-17	2.41E-17	3.22E-17	3.02E-17	2.23E-17	1.71E-17	2.20E-17	3.32E-17	3.92E-17	4.27E-17	3.04E-17	3.17E-17
1.91E-17	1.81E-17	2.25E-17	3.66E-17	2.70E-17	3.99E-17	3.92E-17	3.08E-17	2.38E-17	2.52E-17	2.96E-17	3.71E-17	3.46E-17	3.12E-17	3.38E-17
2.51E-17	2.16E-17	2.56E-17	3.82E-17	3.21E-17	4.63E-17	4.40E-17	3.56E-17	2.75E-17	2.62E-17	2.89E-17	4.02E-17	3.01E-17	3.35E-17	3.94E-17
3.22E-17	2.34E-17	2.87E-17	4.34E-17	4.11E-17	5.25E-17	4.45E-17	3.88E-17	2.80E-17	2.90E-17	3.25E-17	3.62E-17	3.60E-17	3.80E-17	4.08E-17
1.81E-17	2.34E-17	3.08E-17	4.08E-17	4.90E-17	5.00E-17	4.24E-17	4.19E-17	3.49E-17	3.18E-17	3.65E-17	3.43E-17	3.17E-17	3.38E-17	4.26E-17
1.71E-17	2.11E-17	3.16E-17	2.93E-17	3.36E-17	4.04E-17	3.97E-17	4.45E-17	4.37E-17	4.05E-17	4.37E-17	3.71E-17	2.86E-17	3.32E-17	4.54E-17
1.85E-17	2.25E-17	2.45E-17	2.58E-17	2.52E-17	3.52E-17	3.74E-17	3.81E-17	4.68E-17	5.03E-17	5.09E-17	3.68E-17	3.07E-17	3.55E-17	4.52E-17
2.42E-17	2.65E-17	1.81E-17	2.59E-17	2.33E-17	3.46E-17	3.12E-17	3.11E-17	4.21E-17	5.51E-17	5.53E-17	4.29E-17	3.54E-17	4.15E-17	4.45E-17
2.33E-17	2.66E-17	1.98E-17	2.37E-17	2.05E-17	3.05E-17	3.13E-17	2.90E-17	3.67E-17	4.55E-17	4.86E-17	4.23E-17	4.14E-17	4.18E-17	4.79E-17
1.57E-17	2.34E-17	1.95E-17	2.06E-17	1.79E-17	3.11E-17	2.93E-17	3.20E-17	3.86E-17	4.60E-17	4.81E-17	4.69E-17	4.98E-17	4.56E-17	5.28E-17
1.45E-17	2.16E-17	1.90E-17	2.02E-17	1.89E-17	3.03E-17	3.14E-17	3.61E-17	4.50E-17	4.42E-17	4.80E-17	4.63E-17	5.01E-17	5.20E-17	6.05E-17
1.55E-17	1.94E-17	1.82E-17	2.20E-17	1.94E-17	2.88E-17	3.04E-17	3.78E-17	4.99E-17	4.54E-17	4.42E-17	4.42E-17	4.92E-17	5.17E-17	6.53E-17
1.45E-17	1.98E-17	1.94E-17	2.40E-17	2.17E-17	2.98E-17	2.78E-17	4.03E-17	5.60E-17	4.84E-17	3.92E-17	4.17E-17	5.37E-17	5.71E-17	7.03E-17
1.35E-17	1.95E-17	1.97E-17	3.03E-17	2.39E-17	3.23E-17	2.46E-17	4.01E-17	5.12E-17	5.29E-17	4.31E-17	4.38E-17	4.72E-17	5.50E-17	6.68E-17
1.17E-17	1.87E-17	2.14E-17	3.69E-17	3.25E-17	3.40E-17	2.25E-17	3.51E-17	3.93E-17	5.77E-17	4.59E-17	3.82E-17	4.29E-17	5.11E-17	6.82E-17
1.35E-17	2.16E-17	2.17E-17	3.86E-17	4.67E-17	3.57E-17	2.80E-17	3.06E-17	3.30E-17	4.31E-17	4.31E-17	3.32E-17	4.25E-17	5.84E-17	7.33E-17
1.73E-17	2.29E-17	1.96E-17	3.41E-17	5.56E-17	3.99E-17	2.91E-17	2.87E-17	2.99E-17	4.00E-17	3.74E-17	3.37E-17	4.55E-17	6.58E-17	7.84E-17
2.03E-17	2.44E-17	1.82E-17	2.90E-17	4.65E-17	3.32E-17	2.71E-17	2.67E-17	2.73E-17	3.94E-17	3.98E-17	3.66E-17	5.16E-17	6.64E-17	6.99E-17
1.56E-17	1.94E-17	1.56E-17	2.00E-17	3.83E-17	2.40E-17	2.51E-17	2.87E-17	3.06E-17	4.42E-17	4.52E-17	4.18E-17	5.36E-17	5.41E-17	5.57E-17
9.78E-18	1.38E-17	1.73E-17	1.63E-17	3.54E-17	1.90E-17	2.34E-17	2.69E-17	3.48E-17	5.25E-17	5.07E-17	5.05E-17	5.02E-17	4.86E-17	5.22E-17
7.81E-18	1.12E-17	1.65E-17	1.61E-17	2.46E-17	1.88E-17	1.97E-17	2.59E-17	4.01E-17	6.14E-17	5.16E-17	5.70E-17	4.79E-17	4.62E-17	5.32E-17
8.44E-18	1.19E-17	1.82E-17	1.52E-17	1.68E-17	2.04E-17	2.12E-17	2.33E-17	4.73E-17	5.99E-17	4.05E-17	5.11E-17	5.01E-17	4.78E-17	5.13E-17
1.10E-17	1.30E-17	1.36E-17	1.19E-17	1.47E-17	2.33E-17	2.55E-17	1.95E-17	4.13E-17	4.46E-17	3.05E-17	4.09E-17	5.29E-17	5.35E-17	4.51E-17
1.17E-17	1.46E-17	1.18E-17	9.49E-18	1.76E-17	2.43E-17	2.55E-17	1.72E-17	2.93E-17	3.17E-17	2.51E-17	3.22E-17	4.73E-17	5.50E-17	4.38E-17
8.86E-18	1.39E-17	1.39E-17	1.33E-17	1.87E-17	2.37E-17	2.51E-17	1.96E-17	2.53E-17	2.73E-17	2.67E-17	3.16E-17	4.06E-17	5.34E-17	4.38E-17
7.81E-18	1.12E-17	1.30E-17	1.88E-17	2.03E-17	1.88E-17	2.75E-17	2.10E-17	2.11E-17	2.78E-17	3.23E-17	3.53E-17	3.70E-17	4.66E-17	4.55E-17
9.13E-18	1.59E-17	2.25E-17	2.49E-17	3.02E-17	2.35E-17	2.40E-17	1.92E-17	2.11E-17	2.88E-17	3.46E-17	3.74E-17	3.22E-17	4.02E-17	4.70E-17
1.60E-17	2.88E-17	3.66E-17	3.17E-17	3.85E-17	2.80E-17	2.49E-17	2.42E-17	2.24E-17	2.87E-17	3.29E-17	3.54E-17	3.34E-17	4.25E-17	4.75E-17
2.08E-17	3.43E-17	5.23E-17	5.06E-17	4.93E-17	3.65E-17	3.86E-17	3.19E-17	2.63E-17	2.97E-17	3.28E-17	3.46E-17	3.64E-17	4.61E-17	4.63E-17
1.66E-17	3.42E-17	4.68E-17	5.64E-17	5.19E-17	4.65E-17	6.31E-17	4.60E-17	2.65E-17	2.86E-17	3.03E-17	3.28E-17	4.05E-17	3.95E-17	4.95E-17
1.44E-17	2.52E-17	3.74E-17	4.55E-17	4.44E-17	5.98E-17	8.00E-17	5.36E-17	2.77E-17	2.78E-17	3.00E-17	3.14E-17	3.88E-17	3.70E-17	5.43E-17
1.77E-17	2.16E-17	3.35E-17	4.53E-17	4.76E-17	6.90E-17	7.38E-17	5.13E-17	2.52E-17	2.37E-17	3.07E-17	3.15E-17	3.03E-17	3.67E-17	4.74E-17
2.60E-17	2.92E-17	3.99E-17	5.13E-17	4.44E-17	6.14E-17	5.16E-17	4.02E-17	2.34E-17	2.28E-17	3.02E-17	3.19E-17	2.50E-17	3.37E-17	4.43E-17
3.17E-17	4.17E-17	5.39E-17	4.35E-17	2.99E-17	4.75E-17	3.93E-17	3.43E-17	2.30E-17	2.09E-17	2.52E-17	3.11E-17	2.38E-17	3.63E-17	4.05E-17
3.53E-17	4.41E-17	4.61E-17	2.68E-17	1.38E-17	2.86E-17	4.03E-17	3.03E-17	2.23E-17	1.78E-17	2.43E-17	3.23E-17	2.67E-17	3.86E-17	3.86E-17
2.98E-17	3.05E-17	3.03E-17	1.78E-17	6.83E-18	1.46E-17	2.66E-17	2.13E-17	1.92E-17	1.95E-17	2.69E-17	3.01E-17	3.05E-17	3.67E-17	3.39E-17
3.01E-17	2.18E-17	3.09E-17	1.99E-17	7.00E-18	1.05E-17	1.73E-17	1.66E-17	1.98E-17	2.48E-17	3.22E-17	3.09E-17	3.42E-17	3.04E-17	2.99E-17
2.84E-17	2.11E-17	3.15E-17	2.14E-17	9.48E-18	1.10E-17	2.39E-17	2.12E-17	2.74E-17	2.91E-17	3.21E-17	3.68E-17	3.39E-17	3.01E-17	2.93E-17
1.17E-17	1.78E-17	2.89E-17	2.07E-17	1.57E-17	1.48E-17	3.20E-17	2.71E-17	4.28E-17	3.32E-17	3.25E-17	4.19E-17	3.14E-17	3.37E-17	3.45E-17
7.54E-18	1.52E-17	3.62E-17	2.11E-17	1.69E-17	1.49E-17	3.32E-17	2.89E-17	5.57E-17	4.26E-17	2.80E-17	4.28E-17	3.17E-17	3.76E-17	3.61E-17
7.29E-18	1.56E-17	3.47E-17	2.05E-17	1.76E-17	1.47E-17	1.87E-17	2.57E-17	3.82E-17	3.84E-17	2.05E-17	2.85E-17	2.75E-17	4.08E-17	3.19E-17
1.25E-17	1.82E-17	2.56E-17	1.74E-17	1.48E-17	9.04E-18	1.18E-17	2.52E-17	2.11E-17	2.85E-17	1.71E-17	1.96E-17	2.27E-17	3.52E-17	2.32E-17
2.14E-17	3.74E-17	2.30E-17	1.28E-17	7.84E-18	5.84E-18	8.95E-18	1.70E-17	7.99E-18	1.54E-17	1.48E-17	1.75E-17	2.57E-17	3.07E-17	2.43E-17
3.47E-17	5.73E-17	2.20E-17	7.36E-18	5.89E-18	5.19E-18	7.94E-18	9.22E-18	4.62E-18	1.26E-17	1.78E-17	2.16E-17	3.25E-17	3.26E-17	2.71E-17
3.51E-17	4.18E-17	1.61E-17	8.06E-18	5.50E-18	3.22E-18	7.63E-18	1.03E-17	7.05E-18	1.99E-17	2.75E-17	2.54E-17	2.58E-17	2.90E-17	2.26E-17
2.41E-17	1.59E-17	1.06E-17	6.75E-18	6.08E-18	4.53E-18	6.83E-18	1.24E-17	9.35E-18	2.40E-17	3.87E-17	2.57E-17	2.07E-17	2.30E-17	1.76E-17
1.23E-17	6.49E-18	8.20E-18	6.34E-18	6.47E-18	6.79E-18	7.85E-18	1.37E-17	1.16E-17	1.91E-17	2.84E-17	2.18E-17	1.36E-17	1.71E-17	1.52E-17
9.28E-18	5.13E-18	9.39E-18	1.30E-17	1.24E-17	8.14E-18	1.34E-17	1.82E-17	1.65E-17	1.82E-17	1.68E-17	2.11E-17	1.37E-17	1.26E-17	1.25E-17
6.53E-18	2.83E-18	9.94E-18	2.02E-17	1.88E-17	1.26E-17	2.29E-17	2.37E-17	2.71E-17	2.18E-17	1.82E-17	2.21E-17	1.89E-17	1.23E-17	1.42E-17
3.63E-18	2.11E-18	1.07E-17	2.52E-17	1.87E-17	1.55E-17	3.32E-17	2.45E-17	3.26E-17	2.21E-17	2.14E-17	2.50E-17	2.54E-17	1.42E-17	1.64E-17
1.07E-18	1.79E-18	1.34E-17	2.25E-17	1.77E-17	1.83E-17	2.45E-17	1.57E-17	2.25E-17	1.74E-17	2.03E-17	2.72E-17	2.55E-17	1.46E-17	1.58E-17
3.98E-19	1.68E-18	7.77E-18	1.48E-17	2.07E-17	1.93E-17	9.62E-18	9.06E-18	1.23E-17	1.22E-17	1.42E-17	1.97E-17	2.10E-17	1.55E-17	1.45E-17
2.55E-19	1.91E-18	3.84E-18	1.13E-17	1.53E-17	1.30E-17	3.07E-18	3.49E-18	5.72E-18	4.77E-18	1.01E-17	1.28E-17	1.50E-17	1.23E-17	1.12E-17
8.83E-19	6.36E-19	5.42E-19	6.92E-18	1.19E-17	9.00E-18	1.39E-18	1.64E-18	2.87E-18	3.78E-18	8.55E-18	1.07E-17	1.10E-17	1.08E-17	1.21E-17
1.90E-18	2.16E-19	6.01E-20	2.21E-18	8.21E-18	9.02E-18	1.21E-18	1.60E-18	2.74E-18	4.10E-18	7.57E-18	8.28E-18	6.54E-18	9.42E-18	1.09E-17
3.28E-18	2.02E-19	3.28E-19	6.79E-19	6.40E-18	9.31E-18	2.33E-18	8.66E-19	1.95E-18	5.13E-18	7.59E-18	5.65E-18	4.16E-18	1.02E-17	8.29E-18
5.31E-18	1.20E-18	1.99E-19	1.34E-18	7.77E-18	1.40E-17	4.44E-18	6.08E-19	1.49E-18	4.93E-18	6.56E-18	3.95E-18	5.39E-18	1.22E-17	7.38E-18
5.93E-18	3.14E-18	7.73E-22	2.03E-18	5.92E-18	1.17E-17	5.37E-18	6.97E-19	1.36E-18	2.42E-18	3.95E-18	2.37E-18	6.83E-18	1.25E-17	8.17E-18
5.47E-18	2.83E-18	1.01E-19	1.48E-18	4.82E-18	6.10E-18	2.95E-18	1.65E-19	1.10E-18	2.04E-18	4.76E-18	3.19E-18	8.47E-18	7.93E-18	7.35E-18
9.49E-19	1.05E-18	7.10E-19	3.25E-18	6.01E-18	1.67E-18	5.19E-20	1.39E-20	7.04E-19	2.52E-18	6.39E-18	4.76E-18	7.68E-18	5.56E-18	6.32E-18
1.11E-19	1.74E-18	2.04E-18	4.84E-18	5.20E-18	9.47E-20	1.74E-19	4.77E-20	1.63E-18	3.20E-18	5.58E-18	4.44E-18	6.50E-18	4.32E-18	4.91E-18
1.16E-19	4.63E-18	1.93E-18	2.66E-18	1.49E-18	4.01E-20	1.73E-20	1.95E-19	4.48E-18	3.84E-18	6.65E-18	3.35E-18	5.32E-18	2.99E-18	3.07E-18
2.94E-21	1.76E-18	1.14E-18	2.35E-19	4.31E-20	4.76E-22	1.90E-19	1.76E-18	7.61E-18	5.39E-18	7.34E-18	2.42E-18	4.88E-18	3.40E-18	2.10E-18
3.05E-19	3.61E-20	3.84E-19	2.16E-19	6.02E-20	2.06E-19	9.41E-19	4.63E-18	7.68E-18	6.00E-18	6.59E-18	3.17E-18	7.62E-18	7.58E-18	4.35E-18
1.53E-19	1.14E-20	9.59E-20	4.80E-19	1.23E-19	5.24E-19	2.38E-18	7.72E-18	8.15E-18	3.02E-18	5.38E-18	4.17E-18	1.06E-17	1.39E-17	1.04E-17
1.66E-19	1.45E-19	9.33E-20	1.25E-19	1.44E-21	1.66E-18	6.57E-18	1.27E-17	1.32E-17	4.76E-18	6.72E-18	4.85E-18	1.19E-17	1.54E-17	1.16E-17
5.57E-19	1.50E-19	1.34E-21	6.42E-19	1.09E-19	3.81E-18	1.49E-17	2.43E-17	2.65E-17	1.26E-17	1.20E-17	7.82E-18	9.22E-18	1.01E-17	6.61E-18
2.10E-19	7.75E-22	2.35E-19	2.22E-19	5.52E-19	5.28E-18	2.88E-17	5.37E-17	6.03E-17	2.64E-17	1.53E-17	9.89E-18	6.85E-18	8.07E-18	4.93E-18
1.06E-18	2.74E-19	1.64E-19	2.21E-19	1.84E-18	6.98E-18	3.53E-17	9.89E-17	1.10E-16	4.46E-17	1.48E-17	8.51E-18	5.78E-18	4.23E-18	2.52E-18
7.96E-19	1.14E-19	1.71E-19	7.64E-19	2.77E-18	9.11E-18	3.29E-17	9.85E-17	1.05E-16	4.49E-17	1.16E-17	5.77E-18	4.00E-18	1.52E-18	2.00E-18
5.11E-19	1.11E-19	1.01E-18	1.23E-21	1.31E-18	8.48E-18	1.41E-17	3.38E-17	4.71E-17	1.91E-17	5.43E-18	2.37E-18	1.54E-18	3.02E-19	8.59E-19
8.64E-21	5.71E-19	1.46E-18	1.64E-19	8.91E-20	3.32E-18	4.45E-18	1.01E-17	1.16E-17	3.73E-18	9.32E-19	3.46E-19	1.73E-19	1.04E-20	1.73E-19
2.94E-19	5.85E-21	1.50E-18	7.90E-19	4.14E-19	5.75E-19	1.64E-18	4.44E-18	1.32E-18	1.37E-19	4.51E-19	5.25E-19	2.46E-19	4.13E-20	5.82E-23
2.10E-18	6.84E-19	9.31E-19	9.79E-19	1.10E-18	1.67E-20	1.55E-19	2.71E-19	4.59E-19	3.86E-19	2.41E-18	1.01E-18	7.73E-19	5.81E-20	3.79E-21
5.63E-18	2.78E-18	9.26E-20	1.26E-18	9.35E-19	5.93E-21	6.73E-24	4.13E-22	2.47E-18	8.48E-19	1.91E-18	1.34E-18	1.65E-19	3.86E-20	2.30E-19
6.14E-18	7.06E-18	4.14E-19	6.48E-19	2.06E-18	1.93E-18	1.72E-19	4.15E-20	3.51E-18	1.64E-18	1.90E-18	1.94E-18	9.71E-20	9.95E-19	3.98E-19
9.91E-18	5.43E-18	2.32E-18	1.73E-19	1.92E-18	3.78E-18	1.54E-18	7.19E-19	4.25E-18	1.71E-18	1.33E-18	4.14E-18	7.06E-19	3.66E-18	1.29E-18
7.90E-18	1.55E-18	2.48E-18	3.44E-19	5.70E-19	3.99E-18	3.71E-18	1.24E-18	4.91E-18	3.30E-18	2.27E-18	4.77E-18	3.46E-18	6.28E-18	4.47E-18
2.35E-18	2.36E-22	3.39E-20	2.43E-18	1.13E-18	3.24E-18	5.64E-18	1.02E-18	3.12E-18	6.07E-18	4.70E-18	4.74E-18	7.09E-18	8.36E-18	6.80E-18
9.11E-20	2.40E-18	5.80E-19	4.56E-18	2.38E-18	1.62E-18	5.81E-18	2.15E-18	3.10E-18	7.43E-18	7.09E-18	6.94E-18	8.41E-18	1.08E-17	6.59E-18
2.03E-18	5.61E-18	2.00E-18	3.42E-18	2.28E-18	1.75E-18	6.18E-18	3.71E-18	9.02E-18	8.38E-18	8.25E-18	1.02E-17	1.17E-17	1.48E-17	8.59E-18
6.32E-18	9.86E-18	5.27E-18	3.71E-18	5.75E-18	6.35E-18	1.19E-17	7.46E-18	1.30E-17	9.71E-18	9.99E-18	1.37E-17	1.60E-17	1.89E-17	1.31E-17
1.46E-17	1.31E-17	5.29E-18	2.84E-18	9.23E-18	1.96E-17	1.80E-17	1.77E-17	1.49E-17	1.24E-17	1.12E-17	1.96E-17	2.28E-17	2.10E-17	1.83E-17
1.97E-17	1.59E-17	7.75E-18	5.79E-18	1.36E-17	2.90E-17	2.52E-17	2.73E-17	1.81E-17	1.52E-17	2.04E-17	2.68E-17	3.00E-17	2.26E-17	2.82E-17
2.47E-17	1.77E-17	1.65E-17	1.37E-17	2.10E-17	3.33E-17	3.02E-17	3.30E-17	2.65E-17	2.16E-17	2.76E-17	2.68E-17	3.47E-17	2.44E-17	3.09E-17

3.14E-17	2.46E-17	2.21E-17	2.25E-17	2.99E-17	3.67E-17	3.99E-17	4.14E-17	3.32E-17	2.82E-17	2.87E-17	2.33E-17	3.43E-17	2.77E-17	2.83E-17
3.17E-17	2.98E-17	2.85E-17	3.24E-17	3.38E-17	3.64E-17	4.35E-17	4.48E-17	4.09E-17	3.53E-17	3.19E-17	2.61E-17	3.61E-17	3.25E-17	3.05E-17
2.84E-17	3.41E-17	3.67E-17	4.08E-17	3.74E-17	3.72E-17	4.39E-17	4.98E-17	4.56E-17	4.57E-17	3.86E-17	3.24E-17	4.18E-17	3.53E-17	3.96E-17
2.36E-17	4.04E-17	3.94E-17	4.88E-17	4.08E-17	4.35E-17	4.77E-17	5.64E-17	5.31E-17	5.01E-17	4.08E-17	3.51E-17	4.39E-17	4.21E-17	4.44E-17
3.06E-17	4.26E-17	4.11E-17	5.43E-17	4.89E-17	5.30E-17	4.89E-17	6.45E-17	5.67E-17	4.43E-17	4.59E-17	4.23E-17	4.31E-17	4.51E-17	5.06E-17
4.28E-17	4.54E-17	4.45E-17	5.20E-17	4.72E-17	5.46E-17	5.38E-17	7.21E-17	5.88E-17	4.01E-17	4.99E-17	4.58E-17	4.99E-17	4.56E-17	5.67E-17
4.79E-17	5.04E-17	4.59E-17	5.58E-17	5.15E-17	5.33E-17	5.89E-17	7.39E-17	5.63E-17	4.31E-17	4.96E-17	5.15E-17	5.66E-17	5.05E-17	5.72E-17
5.48E-17	5.53E-17	4.98E-17	5.79E-17	5.38E-17	6.00E-17	6.15E-17	7.55E-17	5.49E-17	4.47E-17	5.27E-17	5.13E-17	5.62E-17	5.45E-17	4.97E-17
6.38E-17	6.66E-17	5.90E-17	6.16E-17	5.13E-17	6.06E-17	6.19E-17	7.12E-17	6.46E-17	5.45E-17	6.45E-17	5.83E-17	6.11E-17	6.14E-17	4.67E-17
5.73E-17	6.57E-17	6.29E-17	5.86E-17	4.90E-17	6.20E-17	6.35E-17	7.48E-17	6.63E-17	6.01E-17	7.74E-17	6.70E-17	6.65E-17	6.25E-17	4.69E-17
4.79E-17	6.07E-17	6.56E-17	6.23E-17	5.37E-17	7.25E-17	7.31E-17	7.76E-17	6.48E-17	6.39E-17	8.09E-17	7.02E-17	6.68E-17	6.12E-17	5.02E-17
2.75E-15	2.86E-15	2.89E-15	2.98E-15	2.92E-15	3.16E-15	3.27E-15	3.45E-15	3.43E-15	3.24E-15	3.33E-15	3.35E-15	3.49E-15	3.54E-15	3.57E-15

6.31E-09	6.17E-09	6.59E-09	4.60E-09	3.46E-09	3.71E-09	3.63E-09	2.96E-09	2.66E-09	2.00E-10	-4.42E-10	-9.57E-10	-2.51E-09	-2.40E-09	-3.15E-09
5.99E-09	5.13E-09	5.35E-09	4.04E-09	2.96E-09	2.95E-09	2.57E-09	2.25E-09	2.04E-09	-2.48E-10	-6.88E-10	-1.02E-09	-2.64E-09	-2.61E-09	-2.95E-09
5.17E-09	4.36E-09	4.07E-09	4.03E-09	2.71E-09	2.37E-09	2.09E-09	2.10E-09	2.32E-09	3.85E-10	-2.17E-10	-7.84E-10	-2.36E-09	-2.32E-09	-3.13E-09
4.34E-09	3.86E-09	3.95E-09	3.43E-09	2.40E-09	2.16E-09	2.28E-09	1.93E-09	1.99E-09	9.38E-10	2.73E-10	-9.13E-10	-2.51E-09	-1.94E-09	-3.21E-09
4.16E-09	3.56E-09	3.65E-09	2.80E-09	1.77E-09	2.06E-09	3.02E-09	1.47E-09	1.08E-09	1.08E-09	5.29E-10	-1.10E-09	-2.19E-09	-1.85E-09	-2.85E-09
4.23E-09	3.23E-09	3.44E-09	2.14E-09	1.63E-09	2.08E-09	3.25E-09	9.51E-10	1.81E-10	9.67E-10	4.51E-10	-1.13E-09	-1.22E-09	-1.81E-09	-2.31E-09
4.12E-09	2.78E-09	2.83E-09	1.79E-09	1.32E-09	1.82E-09	5.89E-10	1.04E-09	1.54E-10	8.26E-10	7.05E-11	-1.20E-09	2.64E-11	-1.56E-09	-2.11E-09
3.77E-09	2.71E-09	2.35E-09	1.56E-09	1.28E-09	1.58E-09	4.56E-10	1.30E-09	4.33E-10	2.49E-10	-4.86E-10	-1.44E-09	2.99E-09	-1.59E-09	-1.55E-09
3.68E-09	2.74E-09	1.94E-09	1.71E-09	1.75E-09	1.41E-09	9.14E-10	1.67E-09	6.18E-10	1.65E-10	-9.04E-10	-1.80E-09	8.49E-09	-1.33E-09	-8.58E-10
3.34E-09	2.48E-09	1.95E-09	1.95E-09	1.88E-09	1.29E-09	1.40E-09	1.84E-09	2.80E-10	2.05E-10	-1.03E-09	-1.55E-09	1.36E-08	-9.29E-10	-6.81E-10
2.82E-09	1.97E-09	2.15E-09	2.45E-09	2.01E-09	1.39E-09	1.84E-09	2.05E-09	8.72E-10	5.48E-10	-7.12E-10	-7.66E-10	5.06E-09	-1.15E-10	-3.65E-11
2.69E-09	2.00E-09	2.30E-09	2.74E-09	2.06E-09	1.43E-09	2.01E-09	2.19E-09	1.73E-09	1.18E-09	-1.57E-10	3.73E-10	-2.34E-10	4.77E-10	5.32E-10
2.72E-09	2.11E-09	2.63E-09	3.02E-09	1.98E-09	1.48E-09	1.88E-09	2.13E-09	1.89E-09	1.56E-09	4.66E-10	4.30E-10	-2.48E-10	2.30E-11	4.22E-10
2.60E-09	2.36E-09	2.64E-09	2.39E-09	1.75E-09	1.72E-09	2.26E-09	2.62E-09	1.92E-09	2.03E-09	1.22E-09	1.12E-09	-8.09E-11	-2.98E-10	6.25E-10
2.72E-09	2.46E-09	2.49E-09	2.30E-09	2.27E-09	2.57E-09	2.91E-09	2.75E-09	1.85E-09	2.53E-09	2.01E-09	1.72E-09	8.73E-11	5.19E-10	4.44E-10
3.06E-09	2.31E-09	2.09E-09	2.33E-09	2.77E-09	3.58E-09	3.60E-09	2.72E-09	1.94E-09	2.77E-09	2.01E-09	8.52E-10	-7.43E-11	9.90E-10	1.46E-09
3.55E-09	2.37E-09	2.28E-09	2.41E-09	3.36E-09	4.01E-09	3.25E-09	2.53E-09	2.24E-09	2.53E-09	2.22E-09	9.46E-10	1.13E-10	1.04E-09	2.34E-09
3.90E-09	2.76E-09	2.50E-09	2.77E-09	3.43E-09	3.72E-09	3.16E-09	2.82E-09	2.38E-09	2.62E-09	2.62E-09	1.47E-09	9.88E-10	1.38E-09	1.72E-09
3.89E-09	3.43E-09	3.13E-09	3.15E-09	4.04E-09	4.18E-09	3.84E-09	3.33E-09	2.73E-09	3.02E-09	2.92E-09	2.34E-09	1.71E-09	1.56E-09	1.47E-09
3.85E-09	3.66E-09	3.79E-09	3.41E-09	4.22E-09	4.19E-09	4.37E-09	3.63E-09	3.54E-09	3.17E-09	3.18E-09	2.82E-09	1.90E-09	1.43E-09	1.78E-09
3.73E-09	3.50E-09	3.40E-09	3.37E-09	4.06E-09	3.39E-09	4.49E-09	4.06E-09	4.25E-09	3.34E-09	3.47E-09	3.22E-09	1.95E-09	1.73E-09	1.78E-09
4.07E-09	4.04E-09	3.41E-09	3.15E-09	4.15E-09	3.60E-09	4.86E-09	4.20E-09	4.48E-09	4.22E-09	4.18E-09	3.67E-09	2.40E-09	2.07E-09	2.01E-09
4.63E-09	4.72E-09	3.78E-09	3.09E-09	3.99E-09	3.81E-09	4.91E-09	4.39E-09	4.93E-09	5.17E-09	4.84E-09	3.94E-09	2.90E-09	2.47E-09	2.57E-09
4.73E-09	5.09E-09	3.94E-09	3.31E-09	4.00E-09	4.30E-09	4.79E-09	4.41E-09	5.06E-09	5.43E-09	4.89E-09	4.06E-09	2.79E-09	2.58E-09	2.61E-09
4.17E-09	4.35E-09	3.76E-09	3.57E-09	3.95E-09	4.19E-09	5.04E-09	4.04E-09	4.91E-09	5.08E-09	4.60E-09	4.03E-09	3.04E-09	3.02E-09	2.87E-09
4.39E-09	4.08E-09	3.82E-09	3.80E-09	3.67E-09	4.03E-09	4.47E-09	4.11E-09	4.63E-09	4.35E-09	4.43E-09	4.16E-09	3.72E-09	3.96E-09	3.66E-09
5.01E-09	4.11E-09	3.98E-09	4.05E-09	3.54E-09	4.36E-09	4.64E-09	4.30E-09	4.98E-09	4.70E-09	4.75E-09	4.28E-09	4.13E-09	4.80E-09	4.46E-09
5.35E-09	4.37E-09	4.31E-09	4.23E-09	3.75E-09	4.52E-09	4.75E-09	4.56E-09	5.34E-09	5.55E-09	5.54E-09	4.54E-09	4.07E-09	5.02E-09	4.65E-09
5.45E-09	4.83E-09	5.06E-09	4.44E-09	4.36E-09	4.77E-09	4.73E-09	4.59E-09	5.48E-09	6.18E-09	5.77E-09	4.75E-09	4.04E-09	4.95E-09	4.82E-09
4.73E-09	4.98E-09	5.88E-09	5.48E-09	5.06E-09	5.02E-09	4.57E-09	4.72E-09	5.11E-09	6.15E-09	5.62E-09	4.66E-09	4.35E-09	5.41E-09	5.73E-09
5.05E-09	5.44E-09	6.12E-09	6.64E-09	5.70E-09	5.30E-09	5.03E-09	5.05E-09	5.54E-09	6.32E-09	5.51E-09	4.79E-09	4.87E-09	6.14E-09	6.81E-09
5.31E-09	5.30E-09	5.89E-09	7.26E-09	5.96E-09	5.39E-09	5.05E-09	5.25E-09	5.65E-09	6.60E-09	5.73E-09	5.24E-09	5.30E-09	6.34E-09	6.72E-09
5.37E-09	5.16E-09	5.76E-09	6.41E-09	5.81E-09	5.92E-09	5.39E-09	5.35E-09	5.96E-09	6.74E-09	6.17E-09	5.49E-09	5.66E-09	6.37E-09	6.17E-09
5.56E-09	5.26E-09	5.37E-09	6.14E-09	6.08E-09	6.66E-09	6.14E-09	5.84E-09	6.30E-09	6.96E-09	6.21E-09	5.98E-09	5.91E-09	6.87E-09	6.22E-09
5.92E-09	5.76E-09	5.95E-09	7.03E-09	6.89E-09	7.10E-09	7.07E-09	6.36E-09	6.63E-09	6.77E-09	6.20E-09	6.70E-09	6.50E-09	7.50E-09	6.73E-09
6.13E-09	6.92E-09	6.61E-09	8.13E-09	8.26E-09	8.02E-09	8.05E-09	6.67E-09	6.79E-09	6.70E-09	6.50E-09	7.34E-09	7.32E-09	8.42E-09	7.78E-09
6.77E-09	7.84E-09	7.80E-09	8.87E-09	9.45E-09	9.07E-09	8.58E-09	7.27E-09	6.91E-09	6.43E-09	6.80E-09	7.91E-09	7.99E-09	9.50E-09	8.69E-09
7.91E-09	8.66E-09	9.26E-09	9.86E-09	1.01E-08	9.14E-09	8.10E-09	7.51E-09	6.94E-09	6.18E-09	6.98E-09	8.37E-09	8.58E-09	1.02E-08	9.53E-09
8.07E-09	9.70E-09	1.07E-08	1.11E-08	1.11E-08	8.96E-09	7.46E-09	7.65E-09	7.16E-09	6.45E-09	7.29E-09	8.98E-09	8.74E-09	1.01E-08	9.86E-09
7.99E-09	1.07E-08	1.22E-08	1.32E-08	1.25E-08	9.49E-09	7.34E-09	7.73E-09	7.66E-09	6.80E-09	7.63E-09	8.87E-09	9.10E-09	9.90E-09	9.62E-09
8.10E-09	1.17E-08	1.31E-08	1.47E-08	1.31E-08	9.64E-09	7.38E-09	7.85E-09	8.08E-09	7.40E-09	8.11E-09	8.80E-09	8.91E-09	9.80E-09	8.90E-09
8.01E-09	1.18E-08	1.24E-08	1.36E-08	1.24E-08	9.26E-09	7.38E-09	7.83E-09	7.68E-09	7.40E-09	8.77E-09	8.92E-09	8.25E-09	9.61E-09	8.37E-09
8.22E-09	1.19E-08	1.14E-08	1.06E-08	1.01E-08	8.41E-09	7.69E-09	7.49E-09	7.25E-09	7.36E-09	8.74E-09	9.74E-09	8.43E-09	8.82E-09	8.13E-09
7.91E-09	1.06E-08	1.02E-08	8.65E-09	7.91E-09	8.08E-09	8.23E-09	7.14E-09	7.24E-09	7.51E-09	9.06E-09	1.09E-08	8.70E-09	8.57E-09	8.43E-09
7.95E-09	9.85E-09	1.07E-08	8.95E-09	7.66E-09	7.67E-09	8.38E-09	7.10E-09	7.33E-09	8.12E-09	9.55E-09	1.06E-08	8.31E-09	8.66E-09	8.42E-09
8.57E-09	9.69E-09	1.11E-08	9.10E-09	8.05E-09	7.63E-09	8.21E-09	7.20E-09	7.53E-09	8.93E-09	1.03E-08	9.49E-09	8.22E-09	9.04E-09	8.57E-09
9.45E-09	1.07E-08	1.07E-08	8.53E-09	8.48E-09	7.49E-09	8.07E-09	7.56E-09	7.58E-09	9.22E-09	1.08E-08	9.17E-09	8.60E-09	8.87E-09	8.33E-09
1.03E-08	1.18E-08	1.07E-08	8.39E-09	8.36E-09	7.35E-09	8.04E-09	7.69E-09	7.81E-09	9.18E-09	1.05E-08	8.89E-09	8.48E-09	8.17E-09	7.87E-09
1.08E-08	1.24E-08	1.12E-08	8.38E-09	8.00E-09	7.31E-09	8.28E-09	7.71E-09	8.23E-09	9.60E-09	9.64E-09	8.82E-09	8.20E-09	7.91E-09	7.97E-09
1.00E-08	1.08E-08	9.77E-09	8.21E-09	7.97E-09	7.50E-09	7.90E-09	7.84E-09	8.26E-09	9.54E-09	8.90E-09	8.96E-09	8.14E-09	8.04E-09	8.64E-09
8.92E-09	9.78E-09	8.88E-09	8.33E-09	7.97E-09	7.63E-09	7.92E-09	8.17E-09	8.41E-09	9.57E-09	8.97E-09	9.27E-09	8.44E-09	8.41E-09	9.21E-09
8.17E-09	8.12E-09	8.06E-09	8.55E-09	8.13E-09	7.82E-09	7.98E-09	8.45E-09	8.10E-09	9.13E-09	9.07E-09	9.32E-09	8.31E-09	8.78E-09	1.05E-08
7.59E-09	6.98E-09	7.56E-09	8.73E-09	8.28E-09	7.60E-09	8.40E-09	8.46E-09	7.80E-09	8.46E-09	8.98E-09	9.14E-09	8.43E-09	9.11E-09	1.17E-08
7.17E-09	6.78E-09	7.06E-09	8.93E-09	8.48E-09	7.70E-09	8.67E-09	8.72E-09	8.05E-09	8.53E-09	9.32E-09	9.45E-09	9.27E-09	9.65E-09	1.09E-08
7.25E-09	6.92E-09	7.25E-09	9.43E-09	9.27E-09	8.43E-09	8.81E-09	9.20E-09	8.57E-09	9.32E-09	9.68E-09	9.45E-09	1.03E-08	9.99E-09	1.13E-08
7.53E-09	7.15E-09	7.64E-09	9.77E-09	9.89E-09	9.06E-09	8.66E-09	9.48E-09	8.48E-09	9.77E-09	9.88E-09	1.02E-08	1.11E-08	1.06E-08	1.20E-08
7.53E-09	7.40E-09	7.76E-09	8.91E-09	9.79E-09	9.25E-09	8.64E-09	9.60E-09	8.51E-09	9.22E-09	9.88E-09	1.04E-08	1.07E-08	1.10E-08	1.11E-08
7.25E-09	7.13E-09	7.49E-09	8.36E-09	8.82E-09	8.66E-09	8.74E-09	1.02E-08	9.59E-09	9.53E-09	9.70E-09	9.64E-09	1.02E-08	1.03E-08	1.02E-08
7.22E-09	6.64E-09	7.80E-09	7.83E-09	8.02E-09	7.91E-09	8.71E-09	1.07E-08	1.02E-08	1.03E-08	9.93E-09	9.37E-09	9.64E-09	9.82E-09	9.59E-09
7.42E-09	6.51E-09	7.58E-09	7.76E-09	7.59E-09	7.45E-09	8.53E-09	9.78E-09	9.86E-09	9.92E-09	1.02E-08	9.08E-09	9.35E-09	9.86E-09	9.24E-09
7.51E-09	6.84E-09	7.74E-09	7.63E-09	7.24E-09	7.39E-09	8.45E-09	8.93E-09	8.79E-09	8.53E-09	9.75E-09	9.12E-09	9.46E-09	9.70E-09	9.19E-09
7.56E-09	6.83E-09	7.93E-09	7.27E-09	7.27E-09	7.79E-09	8.73E-09	8.59E-09	8.43E-09	7.75E-09	9.16E-09	9.11E-09	9.23E-09	9.41E-09	9.17E-09
7.53E-09	7.39E-09	8.06E-09	7.37E-09	7.54E-09	7.88E-09	8.32E-09	7.95E-09	8.10E-09	7.80E-09	8.91E-09	8.67E-09	9.32E-09	9.39E-09	9.56E-09
7.79E-09	7.65E-09	7.63E-09	7.31E-09	7.40E-09	7.43E-09	7.56E-09	7.80E-09	8.15E-09	8.35E-09	8.71E-09	8.91E-09	9.46E-09	9.49E-09	9.79E-09
7.67E-09	7.06E-09	6.76E-09	6.94E-09	7.07E-09	7.44E-09	7.32E-09	8.06E-09	8.26E-09	8.72E-09	8.91E-09	9.02E-09	9.89E-09	9.62E-09	1.00E-08
7.49E-09	6.74E-09	6.38E-09	6.85E-09	6.84E-09	7.43E-09	7.75E-09	8.64E-09	8.66E-09	8.31E-09	9.00E-09	9.10E-09	1.01E-08	9.60E-09	1.00E-08
7.43E-09	6.76E-09	6.27E-09	6.33E-09	6.58E-09	7.26E-09	8.25E-09	8.77E-09	8.80E-09	8.27E-09	9.37E-09	9.26E-09	9.96E-09	9.79E-09	9.58E-09
7.08E-09	6.89E-09	6.12E-09	6.03E-09	6.41E-09	7.56E-09	8.34E-09	8.26E-09	8.70E-09	8.40E-09	8.86E-09	9.10E-09	9.92E-09	9.92E-09	9.56E-09
6.60E-09	7.20E-09	6.55E-09	6.14E-09	6.26E-09	8.22E-09	8.71E-09	8.22E-09	8.54E-09	8.33E-09	8.23E-09	9.05E-09	9.88E-09	9.77E-09	9.77E-09
5.85E-09	6.41E-09	7.26E-09	6.09E-09	6.11E-09	8.08E-09	8.46E-09	8.24E-09	8.04E-09	8.16E-09	8.08E-09	9.00E-09	9.38E-09	9.46E-09	1.01E-08
5.17E-09	5.69E-09	6.84E-09	6.05E-09	5.96E-09	7.50E-09	7.57E-09	7.70E-09	7.54E-09	7.97E-09	8.00E-09	8.59E-09	9.33E-09	9.04E-09	1.00E-08
4.87E-09	5.04E-09	5.40E-09	5.57E-09	6.19E-09	7.09E-09	6.88E-09	7.11E-09	7.28E-09	7.73E-09	7.81E-09	8.08E-09	9.21E-09	9.06E-09	1.03E-08
4.59E-09	4.69E-09	5.18E-09	5.14E-09	6.62E-09	6.67E-09	6.51E-09	6.31E-09	7.42E-09	7.43E-09	7.89E-09	7.84E-09	8.70E-09	9.16E-09	1.03E-08
4.75E-09	4.94E-09	5.22E-09	4.96E-09	6.71E-09	6.10E-09	6.01E-09	6.44E-09	7.20E-09	7.36E-09	7.82E-09	8.12E-09	8.41E-09	8.96E-09	1.00E-08
5.32E-09	4.74E-09	5.14E-09	4.69E-09	6.24E-09	5.87E-09	5.86E-09	6.97E-09	7.56E-09	7.83E-09	7.90E-09	8.34E-09	8.23E-09	8.80E-09	9.63E-09
5.37E-09	4.32E-09	4.66E-09	4.72E-09	5.97E-09	6.27E-09	5.95E-09	7.25E-09	7.88E-09	7.86E-09	8.16E-09	8.65E-09	8.09E-09	8.58E-09	9.84E-09
4.34E-09	4.29E-09	4.39E-09	4.75E-09	5.85E-09	6.84E-09	6.46E-09	7.62E-09	7.14E-09	7.84E-09	8.18E-09	8.88E-09	7.07E-09	8.45E-09	9.90E-09
3.98E-09	4.50E-09	4.40E-09	5.06E-09	6.30E-09	6.88E-09	6.82E-09	8.22E-09	7.14E-09	7.90E-09	7.98E-09	8.68E-09	5.52E-09	8.31E-09	9.75E-09

3.80E-09	4.49E-09	4.38E-09	5.47E-09	6.23E-09	7.16E-09	7.00E-09	8.08E-09	7.23E-09	8.21E-09	7.81E-09	8.60E-09	4.26E-09	8.52E-09	9.69E-09
3.91E-09	4.33E-09	4.70E-09	6.01E-09	6.47E-09	8.00E-09	7.00E-09	7.12E-09	6.83E-09	7.53E-09	7.37E-09	8.22E-09	7.92E-09	9.09E-09	9.40E-09
4.02E-09	4.61E-09	4.54E-09	6.60E-09	7.17E-09	7.97E-09	6.67E-09	6.47E-09	6.07E-09	6.80E-09	6.72E-09	7.74E-09	8.47E-09	8.88E-09	9.36E-09
3.73E-09	4.62E-09	4.07E-09	5.61E-09	6.86E-09	6.85E-09	5.70E-09	6.04E-09	5.65E-09	6.60E-09	5.92E-09	7.53E-09	8.14E-09	8.65E-09	9.30E-09
3.51E-09	4.65E-09	3.87E-09	4.78E-09	5.50E-09	5.51E-09	4.99E-09	5.53E-09	5.46E-09	6.05E-09	5.03E-09	7.38E-09	6.79E-09	8.18E-09	8.80E-09
3.86E-09	4.97E-09	3.87E-09	4.31E-09	5.03E-09	5.19E-09	5.02E-09	4.99E-09	5.60E-09	5.87E-09	3.60E-09	6.87E-09	5.97E-09	7.37E-09	7.90E-09
3.64E-09	4.04E-09	3.42E-09	4.15E-09	4.44E-09	4.92E-09	5.11E-09	4.90E-09	6.01E-09	6.18E-09	5.16E-09	6.36E-09	5.86E-09	6.39E-09	6.98E-09
3.54E-09	3.39E-09	2.71E-09	3.63E-09	3.88E-09	4.74E-09	5.40E-09	5.22E-09	6.45E-09	6.81E-09	6.26E-09	6.13E-09	5.83E-09	6.09E-09	6.52E-09
3.48E-09	3.32E-09	2.24E-09	3.89E-09	3.93E-09	5.01E-09	5.86E-09	5.94E-09	6.61E-09	6.42E-09	6.44E-09	6.08E-09	5.79E-09	5.69E-09	6.73E-09
3.31E-09	3.14E-09	2.37E-09	4.09E-09	4.37E-09	5.33E-09	6.05E-09	6.52E-09	6.01E-09	5.42E-09	5.53E-09	5.59E-09	5.47E-09	5.79E-09	6.77E-09
2.50E-09	3.13E-09	2.28E-09	3.35E-09	4.27E-09	5.60E-09	6.69E-09	7.19E-09	6.23E-09	5.20E-09	5.45E-09	5.53E-09	5.28E-09	6.11E-09	6.38E-09
1.90E-09	2.79E-09	1.98E-09	2.58E-09	3.96E-09	5.15E-09	6.53E-09	6.92E-09	6.10E-09	5.01E-09	5.09E-09	5.44E-09	5.19E-09	6.52E-09	6.15E-09
1.65E-09	2.51E-09	2.04E-09	2.42E-09	3.39E-09	4.91E-09	5.07E-09	6.04E-09	5.35E-09	4.09E-09	4.78E-09	5.39E-09	5.35E-09	6.06E-09	6.40E-09
1.36E-09	2.28E-09	2.42E-09	2.87E-09	3.07E-09	4.15E-09	4.51E-09	5.34E-09	4.65E-09	3.78E-09	4.87E-09	5.42E-09	5.00E-09	5.31E-09	6.40E-09
1.26E-09	1.85E-09	2.58E-09	3.32E-09	2.79E-09	3.56E-09	4.06E-09	4.57E-09	4.27E-09	3.50E-09	4.61E-09	5.16E-09	4.41E-09	4.79E-09	6.13E-09
1.32E-09	1.59E-09	2.10E-09	3.28E-09	3.03E-09	3.52E-09	3.75E-09	4.10E-09	4.17E-09	3.37E-09	4.48E-09	5.29E-09	4.35E-09	4.44E-09	5.02E-09
1.58E-09	1.58E-09	2.18E-09	3.14E-09	3.47E-09	3.32E-09	3.57E-09	3.89E-09	4.41E-09	3.50E-09	4.54E-09	5.45E-09	5.21E-09	4.66E-09	4.40E-09
2.05E-09	2.02E-09	2.52E-09	2.64E-09	3.41E-09	3.08E-09	3.44E-09	3.18E-09	3.98E-09	3.73E-09	4.51E-09	5.02E-09	4.81E-09	4.63E-09	4.11E-09
2.45E-09	2.13E-09	2.26E-09	2.08E-09	2.98E-09	2.94E-09	2.89E-09	2.82E-09	3.86E-09	3.44E-09	3.68E-09	4.39E-09	4.74E-09	4.88E-09	4.14E-09
2.00E-09	1.86E-09	1.67E-09	1.92E-09	2.45E-09	2.26E-09	2.54E-09	2.99E-09	3.63E-09	2.91E-09	3.34E-09	4.29E-09	4.93E-09	5.05E-09	4.22E-09
1.58E-09	1.70E-09	1.21E-09	1.61E-09	1.65E-09	1.72E-09	2.47E-09	2.92E-09	3.11E-09	3.24E-09	3.40E-09	4.07E-09	4.70E-09	4.55E-09	4.18E-09
1.38E-09	1.26E-09	7.17E-10	1.31E-09	1.25E-09	1.37E-09	1.98E-09	3.13E-09	3.38E-09	3.90E-09	3.43E-09	4.04E-09	4.58E-09	3.73E-09	4.58E-09
1.26E-09	9.22E-10	1.40E-10	5.16E-10	9.54E-10	1.10E-09	1.37E-09	2.84E-09	3.57E-09	3.92E-09	3.46E-09	3.66E-09	3.61E-09	3.40E-09	4.38E-09
1.54E-09	3.61E-10	-2.22E-10	4.93E-10	7.42E-10	8.68E-10	8.71E-10	1.61E-09	2.71E-09	2.77E-09	3.14E-09	3.47E-09	2.58E-09	2.97E-09	3.26E-09
8.25E-10	-1.78E-10	-3.53E-10	3.99E-10	8.17E-10	6.56E-10	5.10E-10	1.11E-09	1.95E-09	1.68E-09	2.59E-09	3.18E-09	2.22E-09	2.65E-09	2.15E-09
9.34E-11	-4.54E-10	-2.20E-10	-1.31E-10	4.75E-10	4.13E-10	1.41E-10	4.16E-10	1.47E-09	9.51E-10	1.77E-09	2.67E-09	1.78E-09	2.13E-09	1.56E-09
-3.84E-10	-6.27E-10	3.14E-10	-2.06E-10	-3.24E-10	3.29E-10	-7.82E-11	1.74E-10	1.15E-09	6.58E-10	1.05E-09	2.23E-09	1.33E-09	1.62E-09	1.29E-09
-7.81E-10	-2.80E-10	5.23E-10	-2.44E-10	-5.29E-10	3.32E-10	-2.04E-10	-2.12E-10	6.76E-10	2.92E-10	5.25E-10	1.53E-09	8.34E-10	1.32E-09	8.47E-10
-1.16E-09	-8.04E-10	6.93E-11	-1.15E-10	-3.67E-10	3.96E-10	-2.98E-10	-8.16E-10	5.43E-10	1.12E-10	2.99E-10	1.09E-09	7.07E-10	9.33E-10	5.33E-10
-1.21E-09	-1.45E-09	-1.05E-09	-2.30E-10	-4.28E-10	-2.47E-11	-8.07E-10	-1.36E-09	2.47E-10	2.22E-10	1.50E-10	7.13E-10	5.65E-10	7.18E-10	4.03E-10
-1.52E-09	-1.85E-09	-1.72E-09	-9.15E-10	-1.21E-09	-7.17E-10	-1.09E-09	-1.70E-09	-1.37E-10	-1.61E-10	1.51E-10	4.49E-12	5.18E-10	6.51E-10	2.48E-11
-2.02E-09	-2.55E-09	-2.48E-09	-1.96E-09	-2.29E-09	-1.24E-09	-1.62E-09	-2.16E-09	-9.46E-10	-5.96E-10	8.15E-11	-3.94E-10	3.62E-10	2.23E-10	-7.19E-10
-2.76E-09	-3.24E-09	-3.04E-09	-2.51E-09	-2.68E-09	-1.68E-09	-2.15E-09	-2.36E-09	-1.61E-09	-7.42E-10	-4.52E-10	-1.02E-09	3.50E-10	-4.42E-10	-1.21E-09
-3.19E-09	-3.56E-09	-3.43E-09	-3.25E-09	-2.83E-09	-2.20E-09	-2.51E-09	-2.56E-09	-2.13E-09	-1.67E-09	-1.55E-09	-1.46E-09	-7.12E-10	-1.07E-09	-1.43E-09
-3.64E-09	-4.02E-09	-3.96E-09	-4.04E-09	-3.67E-09	-3.10E-09	-3.33E-09	-2.87E-09	-2.88E-09	-2.49E-09	-2.51E-09	-1.77E-09	-1.87E-09	-1.41E-09	-1.76E-09
-4.36E-09	-4.72E-09	-4.34E-09	-4.76E-09	-4.38E-09	-3.92E-09	-3.82E-09	-3.12E-09	-3.57E-09	-3.36E-09	-3.18E-09	-2.14E-09	-3.06E-09	-1.94E-09	-1.98E-09
-5.18E-09	-5.35E-09	-4.49E-09	-5.12E-09	-4.80E-09	-4.23E-09	-4.17E-09	-3.73E-09	-4.29E-09	-4.14E-09	-3.64E-09	-3.50E-09	-3.92E-09	-2.74E-09	-2.27E-09
-5.30E-09	-5.26E-09	-5.10E-09	-5.21E-09	-5.21E-09	-4.45E-09	-4.67E-09	-4.69E-09	-4.76E-09	-4.40E-09	-4.34E-09	-4.30E-09	-4.37E-09	-2.88E-09	-2.42E-09
-5.66E-09	-4.81E-09	-5.30E-09	-5.63E-09	-5.46E-09	-4.93E-09	-5.04E-09	-5.47E-09	-4.87E-09	-4.83E-09	-4.75E-09	-4.50E-09	-4.83E-09	-3.40E-09	-2.40E-09
-5.41E-09	-4.12E-09	-5.15E-09	-5.94E-09	-5.78E-09	-5.36E-09	-5.36E-09	-5.35E-09	-4.81E-09	-5.03E-09	-5.23E-09	-5.07E-09	-5.33E-09	-4.04E-09	-2.67E-09
-5.39E-09	-4.13E-09	-5.50E-09	-6.34E-09	-6.19E-09	-5.53E-09	-5.35E-09	-4.96E-09	-4.88E-09	-5.34E-09	-5.67E-09	-5.19E-09	-5.55E-09	-4.63E-09	-3.89E-09
-5.91E-09	-5.39E-09	-6.41E-09	-6.77E-09	-6.58E-09	-5.81E-09	-5.36E-09	-5.04E-09	-4.89E-09	-5.64E-09	-5.75E-09	-5.62E-09	-5.81E-09	-4.75E-09	-4.51E-09
-6.69E-09	-6.92E-09	-7.09E-09	-6.93E-09	-6.68E-09	-6.16E-09	-5.35E-09	-5.52E-09	-5.50E-09	-6.04E-09	-5.77E-09	-5.99E-09	-5.99E-09	-4.84E-09	-4.27E-09
-7.55E-09	-7.24E-09	-7.11E-09	-6.92E-09	-6.81E-09	-6.31E-09	-5.96E-09	-6.10E-09	-6.30E-09	-6.38E-09	-6.41E-09	-6.19E-09	-6.26E-09	-5.25E-09	-4.28E-09
-7.91E-09	-7.45E-09	-7.36E-09	-7.45E-09	-7.19E-09	-6.70E-09	-6.93E-09	-6.59E-09	-6.71E-09	-6.47E-09	-7.03E-09	-6.39E-09	-6.24E-09	-6.35E-09	-5.03E-09
-8.05E-09	-7.92E-09	-7.98E-09	-8.05E-09	-7.32E-09	-7.30E-09	-7.95E-09	-7.28E-09	-6.99E-09	-6.72E-09	-7.14E-09	-6.66E-09	-6.50E-09	-6.90E-09	-5.71E-09
-7.93E-09	-7.95E-09	-8.39E-09	-8.22E-09	-7.42E-09	-8.28E-09	-8.44E-09	-7.85E-09	-7.69E-09	-7.43E-09	-7.49E-09	-6.52E-09	-7.10E-09	-7.29E-09	-6.17E-09
-7.52E-09	-7.60E-09	-8.48E-09	-7.99E-09	-7.66E-09	-9.00E-09	-8.30E-09	-8.38E-09	-8.33E-09	-8.20E-09	-7.90E-09	-7.25E-09	-7.62E-09	-7.63E-09	-6.99E-09
-7.31E-09	-7.67E-09	-8.64E-09	-7.89E-09	-8.00E-09	-9.37E-09	-8.56E-09	-8.63E-09	-8.90E-09	-8.62E-09	-8.45E-09	-8.36E-09	-8.08E-09	-8.04E-09	-7.45E-09
-7.69E-09	-8.00E-09	-8.71E-09	-8.11E-09	-8.17E-09	-9.67E-09	-8.92E-09	-8.91E-09	-9.34E-09	-9.38E-09	-9.16E-09	-9.08E-09	-8.62E-09	-8.03E-09	-7.97E-09

3.98E-17	3.81E-17	4.34E-17	2.12E-17	1.20E-17	1.37E-17	1.32E-17	8.74E-18	7.10E-18	3.99E-20	1.96E-19	9.15E-19	6.28E-18	5.76E-18	9.95E-18
3.58E-17	2.63E-17	2.86E-17	1.63E-17	8.79E-18	8.71E-18	6.61E-18	5.04E-18	4.17E-18	6.13E-20	4.73E-19	1.05E-18	6.96E-18	6.84E-18	8.72E-18
2.67E-17	1.90E-17	1.66E-17	1.62E-17	7.34E-18	5.62E-18	4.38E-18	4.42E-18	5.37E-18	1.48E-19	4.72E-20	6.14E-19	5.55E-18	5.38E-18	9.78E-18
1.88E-17	1.49E-17	1.56E-17	1.18E-17	5.77E-18	4.68E-18	5.19E-18	3.72E-18	3.95E-18	8.79E-19	7.45E-20	8.33E-19	6.30E-18	3.76E-18	1.03E-17
1.73E-17	1.27E-17	1.33E-17	7.84E-18	3.15E-18	4.22E-18	9.10E-18	2.16E-18	1.17E-18	1.16E-18	2.80E-19	1.22E-18	4.80E-18	3.44E-18	8.11E-18
1.79E-17	1.04E-17	1.19E-17	4.58E-18	2.67E-18	4.31E-18	1.05E-17	9.04E-19	3.29E-20	9.35E-19	2.04E-19	1.28E-18	1.48E-18	3.27E-18	5.32E-18
1.69E-17	7.74E-18	8.01E-18	3.21E-18	1.75E-18	3.31E-18	3.47E-19	1.09E-18	2.36E-20	6.82E-19	4.97E-21	1.44E-18	6.96E-22	2.44E-18	4.47E-18
1.42E-17	7.35E-18	5.54E-18	2.45E-18	1.64E-18	2.48E-18	2.08E-19	1.69E-18	1.88E-19	6.21E-20	2.36E-19	2.07E-18	8.93E-18	2.53E-18	2.39E-18
1.36E-17	7.51E-18	3.76E-18	2.91E-18	3.06E-18	2.00E-18	8.36E-19	2.81E-18	3.82E-19	2.72E-20	8.17E-19	3.23E-18	7.22E-17	1.78E-18	7.37E-19
1.12E-17	6.15E-18	3.79E-18	3.82E-18	3.53E-18	1.66E-18	1.96E-18	3.38E-18	7.81E-20	4.22E-20	1.05E-18	2.41E-18	1.84E-16	8.62E-19	4.64E-19
7.95E-18	3.90E-18	4.62E-18	6.01E-18	4.04E-18	1.93E-18	3.37E-18	4.20E-18	7.60E-19	3.00E-19	5.07E-19	5.86E-19	2.56E-17	1.33E-20	1.33E-21
7.26E-18	3.98E-18	5.30E-18	7.51E-18	4.24E-18	2.05E-18	4.04E-18	4.79E-18	2.99E-18	1.38E-18	2.46E-20	1.39E-19	5.46E-20	2.28E-19	2.83E-19
7.41E-18	4.47E-18	6.89E-18	9.12E-18	3.93E-18	2.19E-18	3.53E-18	4.55E-18	3.59E-18	2.44E-18	2.17E-19	1.85E-19	6.14E-20	5.27E-22	1.78E-19
6.78E-18	5.56E-18	6.98E-18	5.71E-18	3.08E-18	2.98E-18	5.10E-18	6.86E-18	3.70E-18	4.13E-18	1.48E-18	1.25E-18	6.55E-21	8.89E-20	3.91E-19
7.38E-18	6.03E-18	6.22E-18	5.30E-18	5.15E-18	6.62E-18	8.48E-18	7.57E-18	3.42E-18	6.40E-18	4.04E-18	2.96E-18	7.62E-21	2.69E-19	1.97E-19
9.35E-18	5.31E-18	4.38E-18	5.41E-18	7.70E-18	1.28E-17	1.30E-17	7.39E-18	3.76E-18	7.65E-18	4.05E-18	7.26E-19	5.52E-21	9.81E-19	2.13E-18
1.26E-17	5.60E-18	5.18E-18	5.80E-18	1.13E-17	1.61E-17	1.06E-17	6.39E-18	5.03E-18	6.40E-18	4.93E-18	8.95E-19	1.27E-20	1.09E-18	5.47E-18
1.52E-17	7.59E-18	6.27E-18	7.65E-18	1.18E-17	1.38E-17	9.97E-18	7.94E-18	5.66E-18	6.87E-18	6.88E-18	2.17E-18	9.77E-19	1.90E-18	2.97E-18
1.51E-17	1.17E-17	9.80E-18	9.93E-18	1.64E-17	1.75E-17	1.47E-17	1.11E-17	7.43E-18	9.10E-18	8.53E-18	5.46E-18	2.94E-18	2.44E-18	2.17E-18
1.48E-17	1.34E-17	1.44E-17	1.16E-17	1.78E-17	1.75E-17	1.91E-17	1.32E-17	1.26E-17	1.01E-17	1.01E-17	7.95E-18	3.61E-18	2.04E-18	3.15E-18
1.39E-17	1.22E-17	1.15E-17	1.14E-17	1.65E-17	1.15E-17	2.01E-17	1.65E-17	1.80E-17	1.12E-17	1.21E-17	1.04E-17	3.79E-18	2.99E-18	3.16E-18
1.66E-17	1.63E-17	1.16E-17	9.93E-18	1.72E-17	1.30E-17	2.36E-17	1.77E-17	2.01E-17	1.78E-17	1.75E-17	1.35E-17	5.76E-18	4.28E-18	4.05E-18
2.14E-17	2.23E-17	1.43E-17	9.54E-18	1.59E-17	1.46E-17	2.41E-17	1.92E-17	2.43E-17	2.67E-17	2.35E-17	1.55E-17	8.41E-18	6.12E-18	6.59E-18
2.23E-17	2.59E-17	1.55E-17	1.10E-17	1.60E-17	1.85E-17	2.29E-17	1.94E-17	2.56E-17	2.95E-17	2.39E-17	1.65E-17	7.80E-18	6.67E-18	6.81E-18
1.74E-17	1.90E-17	1.41E-17	1.28E-17	1.56E-17	1.75E-17	2.54E-17	1.63E-17	2.41E-17	2.58E-17	2.12E-17	1.63E-17	9.22E-18	9.09E-18	8.25E-18
1.93E-17	1.66E-17	1.46E-17	1.45E-17	1.35E-17	1.62E-17	2.00E-17	1.69E-17	2.14E-17	1.89E-17	1.96E-17	1.73E-17	1.38E-17	1.56E-17	1.34E-17
2.51E-17	1.69E-17	1.58E-17	1.64E-17	1.26E-17	1.90E-17	2.16E-17	1.85E-17	2.48E-17	2.21E-17	2.26E-17	1.83E-17	1.71E-17	2.30E-17	1.99E-17
2.86E-17	1.91E-17	1.85E-17	1.79E-17	1.40E-17	2.04E-17	2.26E-17	2.08E-17	2.85E-17	3.08E-17	3.07E-17	2.06E-17	1.66E-17	2.52E-17	2.17E-17
2.97E-17	2.33E-17	2.56E-17	1.97E-17	1.90E-17	2.27E-17	2.24E-17	2.11E-17	3.00E-17	3.82E-17	3.33E-17	2.26E-17	1.63E-17	2.45E-17	2.33E-17
2.24E-17	2.48E-17	3.46E-17	3.00E-17	2.56E-17	2.53E-17	2.09E-17	2.23E-17	2.62E-17	3.78E-17	3.16E-17	2.17E-17	1.90E-17	2.93E-17	3.29E-17
2.55E-17	2.96E-17	3.74E-17	4.41E-17	3.25E-17	2.81E-17	2.53E-17	2.55E-17	3.07E-17	3.99E-17	3.03E-17	2.29E-17	2.37E-17	3.77E-17	4.64E-17

2.82E-17	2.81E-17	3.47E-17	5.27E-17	3.55E-17	2.91E-17	2.55E-17	2.76E-17	3.19E-17	4.35E-17	3.29E-17	2.74E-17	2.81E-17	4.02E-17	4.52E-17
2.88E-17	2.66E-17	3.32E-17	4.10E-17	3.38E-17	3.50E-17	2.90E-17	2.86E-17	3.55E-17	4.54E-17	3.81E-17	3.02E-17	3.21E-17	4.06E-17	3.81E-17
3.10E-17	2.77E-17	2.89E-17	3.77E-17	3.70E-17	4.44E-17	3.77E-17	3.41E-17	3.97E-17	4.84E-17	3.85E-17	3.58E-17	3.49E-17	4.72E-17	3.87E-17
3.50E-17	3.31E-17	3.53E-17	4.94E-17	4.74E-17	5.04E-17	5.00E-17	4.04E-17	4.40E-17	4.59E-17	3.85E-17	4.49E-17	4.23E-17	5.62E-17	4.53E-17
3.76E-17	4.79E-17	4.37E-17	6.62E-17	6.82E-17	6.44E-17	6.48E-17	4.44E-17	4.61E-17	4.50E-17	4.23E-17	5.38E-17	5.36E-17	7.09E-17	6.05E-17
4.58E-17	6.15E-17	6.08E-17	7.87E-17	8.94E-17	8.22E-17	7.36E-17	5.29E-17	4.77E-17	4.13E-17	4.62E-17	6.25E-17	6.39E-17	9.03E-17	7.55E-17
6.26E-17	7.50E-17	8.57E-17	9.73E-17	1.02E-16	8.36E-17	6.55E-17	5.65E-17	4.82E-17	3.81E-17	4.88E-17	7.01E-17	7.36E-17	1.03E-16	9.08E-17
6.51E-17	9.41E-17	1.15E-16	1.24E-16	1.23E-16	8.03E-17	5.57E-17	5.85E-17	5.13E-17	4.16E-17	5.32E-17	8.07E-17	7.64E-17	1.01E-16	9.73E-17
6.39E-17	1.15E-16	1.48E-16	1.74E-16	1.56E-16	9.00E-17	5.38E-17	5.98E-17	5.87E-17	4.63E-17	5.83E-17	7.87E-17	8.29E-17	9.81E-17	9.25E-17
6.56E-17	1.36E-16	1.71E-16	2.17E-16	1.71E-16	9.28E-17	5.45E-17	6.17E-17	6.53E-17	5.47E-17	6.58E-17	7.75E-17	7.94E-17	9.61E-17	7.91E-17
6.41E-17	1.40E-16	1.55E-16	1.85E-16	1.53E-16	8.58E-17	5.45E-17	6.12E-17	5.90E-17	5.48E-17	7.70E-17	7.97E-17	6.80E-17	9.23E-17	7.01E-17
6.76E-17	1.43E-16	1.30E-16	1.13E-16	1.01E-16	7.07E-17	5.92E-17	5.61E-17	5.26E-17	5.41E-17	7.64E-17	9.49E-17	7.11E-17	7.78E-17	6.61E-17
6.25E-17	1.12E-16	1.04E-16	7.48E-17	6.25E-17	6.52E-17	6.77E-17	5.10E-17	5.24E-17	5.64E-17	8.21E-17	1.19E-16	7.57E-17	7.34E-17	7.11E-17
6.32E-17	9.70E-17	1.14E-16	8.02E-17	5.86E-17	5.89E-17	7.03E-17	5.04E-17	5.37E-17	6.59E-17	9.11E-17	1.13E-16	6.91E-17	7.50E-17	7.08E-17
7.34E-17	9.39E-17	1.23E-16	8.28E-17	6.48E-17	5.82E-17	6.74E-17	5.19E-17	5.68E-17	7.98E-17	1.06E-16	9.01E-17	6.76E-17	8.16E-17	7.34E-17
8.94E-17	1.14E-16	1.15E-16	7.28E-17	7.20E-17	5.61E-17	6.51E-17	5.71E-17	5.75E-17	8.51E-17	1.16E-16	8.41E-17	7.39E-17	7.87E-17	6.93E-17
1.07E-16	1.39E-16	1.15E-16	7.04E-17	6.98E-17	5.40E-17	6.46E-17	5.91E-17	6.10E-17	8.42E-17	1.10E-16	7.90E-17	7.19E-17	6.67E-17	6.19E-17
1.16E-16	1.53E-16	1.26E-16	7.03E-17	6.41E-17	5.34E-17	6.85E-17	5.94E-17	6.78E-17	9.21E-17	9.28E-17	7.78E-17	6.73E-17	6.26E-17	6.35E-17
1.00E-16	1.16E-16	9.55E-17	6.74E-17	6.35E-17	5.62E-17	6.25E-17	6.15E-17	6.82E-17	9.10E-17	7.93E-17	8.03E-17	6.63E-17	6.46E-17	7.46E-17
7.96E-17	9.57E-17	7.88E-17	6.94E-17	6.35E-17	5.82E-17	6.27E-17	6.68E-17	7.07E-17	9.17E-17	8.05E-17	8.60E-17	7.12E-17	7.08E-17	8.49E-17
6.68E-17	6.59E-17	6.50E-17	7.31E-17	6.61E-17	6.11E-17	6.37E-17	7.14E-17	6.55E-17	8.33E-17	8.23E-17	8.68E-17	6.91E-17	7.71E-17	1.10E-16
5.76E-17	4.87E-17	5.72E-17	7.62E-17	6.86E-17	5.78E-17	7.05E-17	7.15E-17	6.09E-17	7.16E-17	8.07E-17	8.35E-17	7.10E-17	8.31E-17	1.36E-16
5.14E-17	4.60E-17	4.98E-17	7.98E-17	7.18E-17	5.93E-17	7.51E-17	7.61E-17	6.47E-17	7.28E-17	8.68E-17	8.92E-17	8.60E-17	9.31E-17	1.19E-16
5.25E-17	4.79E-17	5.26E-17	8.90E-17	8.59E-17	7.11E-17	7.76E-17	8.47E-17	7.34E-17	8.70E-17	9.37E-17	8.93E-17	1.05E-16	9.98E-17	1.28E-16
5.66E-17	5.11E-17	5.83E-17	9.55E-17	9.78E-17	8.20E-17	7.50E-17	8.98E-17	7.18E-17	9.55E-17	9.77E-17	1.05E-16	1.23E-16	1.13E-16	1.45E-16
5.67E-17	5.48E-17	6.03E-17	7.94E-17	9.59E-17	8.55E-17	7.46E-17	9.21E-17	7.25E-17	8.50E-17	9.75E-17	1.09E-16	1.15E-16	1.22E-16	1.23E-16
5.26E-17	5.08E-17	5.61E-17	6.98E-17	7.77E-17	7.51E-17	7.63E-17	1.04E-16	9.20E-17	9.07E-17	9.41E-17	9.30E-17	1.04E-16	1.06E-16	1.05E-16
5.22E-17	4.42E-17	6.09E-17	6.14E-17	6.44E-17	6.26E-17	7.58E-17	1.14E-16	1.04E-16	1.06E-16	9.86E-17	8.79E-17	9.28E-17	9.65E-17	9.20E-17
5.50E-17	4.24E-17	5.75E-17	6.03E-17	5.75E-17	5.55E-17	7.27E-17	9.56E-17	9.73E-17	9.85E-17	1.03E-16	8.25E-17	8.75E-17	9.72E-17	8.53E-17
5.63E-17	4.68E-17	6.00E-17	5.83E-17	5.25E-17	5.47E-17	7.14E-17	7.97E-17	7.73E-17	7.27E-17	9.50E-17	8.32E-17	8.95E-17	9.41E-17	8.44E-17
5.71E-17	4.66E-17	6.29E-17	5.28E-17	5.29E-17	6.06E-17	7.62E-17	7.38E-17	7.10E-17	6.01E-17	8.40E-17	8.31E-17	8.51E-17	8.85E-17	8.41E-17
5.67E-17	5.46E-17	6.50E-17	5.44E-17	5.69E-17	6.20E-17	6.93E-17	6.32E-17	6.56E-17	6.08E-17	7.94E-17	7.51E-17	8.68E-17	8.81E-17	9.14E-17
6.06E-17	5.86E-17	5.82E-17	5.35E-17	5.47E-17	5.51E-17	5.72E-17	6.08E-17	6.64E-17	6.97E-17	7.59E-17	7.95E-17	8.94E-17	9.01E-17	9.58E-17
5.88E-17	4.99E-17	4.58E-17	4.81E-17	5.00E-17	5.53E-17	5.35E-17	6.49E-17	6.82E-17	7.61E-17	7.93E-17	8.13E-17	9.79E-17	9.26E-17	1.00E-16
5.62E-17	4.54E-17	4.07E-17	4.69E-17	4.68E-17	5.52E-17	6.00E-17	7.46E-17	7.50E-17	6.91E-17	8.09E-17	8.28E-17	1.01E-16	9.22E-17	1.01E-16
5.53E-17	4.57E-17	3.93E-17	4.01E-17	4.32E-17	5.28E-17	6.81E-17	7.69E-17	7.75E-17	6.84E-17	8.77E-17	8.57E-17	9.92E-17	9.58E-17	9.17E-17
5.01E-17	4.75E-17	3.74E-17	3.64E-17	4.11E-17	5.71E-17	6.95E-17	6.83E-17	7.57E-17	7.06E-17	7.84E-17	8.29E-17	9.84E-17	9.85E-17	9.14E-17
4.36E-17	5.19E-17	4.28E-17	3.77E-17	3.92E-17	6.76E-17	7.59E-17	6.76E-17	7.29E-17	6.94E-17	6.78E-17	8.19E-17	9.76E-17	9.54E-17	9.54E-17
3.42E-17	4.11E-17	5.27E-17	3.71E-17	3.74E-17	6.52E-17	7.15E-17	6.78E-17	6.47E-17	6.67E-17	6.52E-17	8.10E-17	8.79E-17	8.94E-17	1.02E-16
2.68E-17	3.24E-17	4.68E-17	3.66E-17	3.55E-17	5.62E-17	5.73E-17	5.92E-17	5.69E-17	6.35E-17	6.40E-17	7.37E-17	8.71E-17	8.17E-17	1.01E-16
2.37E-17	2.54E-17	2.92E-17	3.10E-17	3.83E-17	5.03E-17	4.73E-17	5.05E-17	5.29E-17	5.97E-17	6.11E-17	6.53E-17	8.47E-17	8.21E-17	1.06E-16
2.11E-17	2.20E-17	2.69E-17	2.64E-17	4.39E-17	4.45E-17	4.23E-17	3.98E-17	5.51E-17	5.52E-17	6.22E-17	6.15E-17	7.57E-17	8.39E-17	1.07E-16
2.26E-17	2.44E-17	2.73E-17	2.46E-17	4.50E-17	3.72E-17	3.61E-17	4.14E-17	5.18E-17	5.42E-17	6.11E-17	6.59E-17	7.07E-17	8.03E-17	1.01E-16
2.83E-17	2.25E-17	2.65E-17	2.20E-17	3.89E-17	3.44E-17	3.43E-17	4.86E-17	5.72E-17	6.13E-17	6.24E-17	6.96E-17	6.77E-17	7.75E-17	9.28E-17
2.88E-17	1.86E-17	2.17E-17	2.23E-17	3.56E-17	3.93E-17	3.55E-17	5.25E-17	6.22E-17	6.18E-17	6.66E-17	7.48E-17	6.55E-17	7.37E-17	9.69E-17
1.89E-17	1.84E-17	1.92E-17	2.26E-17	3.42E-17	4.68E-17	4.17E-17	5.80E-17	5.10E-17	6.15E-17	6.69E-17	7.88E-17	5.00E-17	7.14E-17	9.79E-17
1.59E-17	2.02E-17	1.94E-17	2.56E-17	3.97E-17	4.73E-17	4.65E-17	6.75E-17	5.09E-17	6.25E-17	6.37E-17	7.53E-17	3.04E-17	6.90E-17	9.51E-17
1.45E-17	2.01E-17	1.92E-17	3.00E-17	3.88E-17	5.13E-17	4.89E-17	6.53E-17	5.22E-17	6.74E-17	6.10E-17	7.40E-17	1.82E-17	7.27E-17	9.39E-17
1.53E-17	1.88E-17	2.20E-17	3.61E-17	4.19E-17	6.40E-17	4.90E-17	5.07E-17	4.66E-17	5.67E-17	5.44E-17	6.76E-17	6.28E-17	8.26E-17	8.83E-17
1.61E-17	2.12E-17	2.06E-17	4.35E-17	5.15E-17	6.36E-17	4.44E-17	4.19E-17	3.68E-17	4.62E-17	4.51E-17	6.00E-17	7.17E-17	7.89E-17	8.76E-17
1.39E-17	2.13E-17	1.66E-17	3.15E-17	4.71E-17	4.69E-17	3.25E-17	3.65E-17	3.19E-17	4.36E-17	3.51E-17	5.68E-17	6.63E-17	7.48E-17	8.64E-17
1.23E-17	2.16E-17	1.49E-17	2.29E-17	3.02E-17	3.04E-17	2.49E-17	3.06E-17	2.98E-17	3.66E-17	2.53E-17	5.45E-17	4.61E-17	6.70E-17	7.74E-17
1.49E-17	2.47E-17	1.49E-17	1.86E-17	2.53E-17	2.70E-17	2.52E-17	2.49E-17	3.14E-17	3.45E-17	1.30E-17	4.71E-17	3.57E-17	5.44E-17	6.24E-17
1.32E-17	1.64E-17	1.17E-17	1.72E-17	1.97E-17	2.42E-17	2.61E-17	2.40E-17	3.61E-17	3.82E-17	2.66E-17	4.05E-17	3.44E-17	4.08E-17	4.87E-17
1.25E-17	1.15E-17	7.33E-18	1.32E-17	1.50E-17	2.24E-17	2.91E-17	2.73E-17	4.16E-17	4.64E-17	3.91E-17	3.76E-17	3.40E-17	3.71E-17	4.26E-17
1.21E-17	1.11E-17	5.02E-18	1.51E-17	1.54E-17	2.51E-17	3.43E-17	3.53E-17	4.38E-17	4.12E-17	4.15E-17	3.70E-17	3.35E-17	3.24E-17	4.54E-17
1.10E-17	9.86E-18	5.60E-18	1.67E-17	1.91E-17	2.84E-17	3.66E-17	4.24E-17	3.61E-17	2.94E-17	3.05E-17	3.13E-17	3.00E-17	3.35E-17	4.58E-17
6.25E-18	9.81E-18	5.20E-18	1.12E-17	1.82E-17	3.14E-17	4.48E-17	5.17E-17	3.88E-17	2.70E-17	2.97E-17	3.05E-17	2.78E-17	3.74E-17	4.06E-17
3.60E-18	7.76E-18	3.92E-18	6.65E-18	1.57E-17	2.65E-17	4.26E-17	4.79E-17	3.72E-17	2.51E-17	2.59E-17	2.96E-17	2.69E-17	4.25E-17	3.78E-17
2.73E-18	6.29E-18	4.18E-18	5.87E-18	1.15E-17	2.41E-17	2.57E-17	3.65E-17	2.86E-17	1.67E-17	2.29E-17	2.91E-17	2.86E-17	3.67E-17	4.09E-17
1.85E-18	5.19E-18	5.85E-18	8.24E-18	9.45E-18	1.72E-17	2.04E-17	2.85E-17	2.16E-17	1.43E-17	2.37E-17	2.93E-17	2.50E-17	2.81E-17	4.10E-17
1.58E-18	3.42E-18	6.65E-18	1.11E-17	7.76E-18	1.27E-17	1.65E-17	2.09E-17	1.82E-17	1.22E-17	2.12E-17	2.67E-17	1.94E-17	2.29E-17	3.75E-17
1.75E-18	2.54E-18	4.41E-18	1.08E-17	9.20E-18	1.24E-17	1.41E-17	1.68E-17	1.74E-17	1.13E-17	2.01E-17	2.80E-17	1.89E-17	1.97E-17	2.52E-17
2.51E-18	2.50E-18	4.73E-18	9.83E-18	1.21E-17	1.10E-17	1.27E-17	1.52E-17	1.95E-17	1.22E-17	2.06E-17	2.97E-17	2.72E-17	2.18E-17	1.93E-17
4.21E-18	4.10E-18	6.35E-18	6.97E-18	1.16E-17	9.50E-18	1.18E-17	1.01E-17	1.58E-17	1.39E-17	2.04E-17	2.52E-17	2.32E-17	2.14E-17	1.69E-17
6.00E-18	4.54E-18	5.11E-18	4.32E-18	8.88E-18	8.64E-18	8.37E-18	7.93E-18	1.49E-17	1.18E-17	1.35E-17	1.93E-17	2.25E-17	2.38E-17	1.72E-17
3.99E-18	3.48E-18	2.78E-18	3.68E-18	6.00E-18	5.11E-18	6.44E-18	8.94E-18	1.32E-17	8.45E-18	1.11E-17	1.84E-17	2.43E-17	2.55E-17	1.78E-17
2.48E-18	2.88E-18	1.47E-18	2.60E-18	2.72E-18	2.95E-18	6.10E-18	8.53E-18	9.64E-18	1.05E-17	1.16E-17	1.66E-17	2.21E-17	2.07E-17	1.75E-17
1.91E-18	1.59E-18	5.14E-19	1.71E-18	1.57E-18	1.87E-18	3.90E-18	9.82E-18	1.14E-17	1.52E-17	1.18E-17	1.63E-17	2.10E-17	1.39E-17	2.10E-17
1.58E-18	8.50E-19	1.97E-20	2.67E-19	9.10E-19	1.21E-18	1.86E-18	8.05E-18	1.28E-17	1.54E-17	1.19E-17	1.34E-17	1.30E-17	1.16E-17	1.92E-17
2.37E-18	1.31E-19	4.93E-20	2.43E-19	5.51E-19	7.54E-19	7.59E-19	2.61E-18	7.33E-18	7.66E-18	9.85E-18	1.20E-17	6.66E-18	8.82E-18	1.06E-17
6.81E-19	3.17E-20	1.24E-19	1.59E-19	6.67E-19	4.30E-19	2.60E-19	1.24E-18	3.81E-18	2.83E-18	6.69E-18	1.01E-17	4.94E-18	7.02E-18	4.61E-18
8.73E-21	2.06E-19	4.85E-20	1.72E-20	2.26E-19	1.70E-19	1.98E-20	1.73E-19	2.15E-18	9.05E-19	3.15E-18	7.15E-18	3.17E-18	4.52E-18	2.43E-18
1.48E-19	3.93E-19	9.84E-20	4.24E-20	1.05E-19	1.08E-19	6.12E-21	3.03E-20	1.31E-18	4.33E-19	1.10E-18	4.97E-18	1.77E-18	2.63E-18	1.68E-18
6.10E-19	7.86E-20	2.74E-19	5.93E-20	2.80E-19	1.10E-19	4.16E-20	4.48E-20	4.57E-19	8.51E-20	2.76E-19	2.34E-18	6.95E-19	1.73E-18	7.18E-19
1.34E-18	6.46E-19	4.81E-21	1.32E-20	1.35E-19	1.57E-19	8.87E-20	6.65E-19	2.95E-19	1.25E-20	8.91E-20	1.19E-18	5.00E-19	8.70E-19	2.84E-19
1.46E-18	2.11E-18	1.10E-18	5.28E-20	1.83E-19	6.11E-22	6.51E-19	1.86E-18	6.09E-20	4.91E-20	2.26E-20	5.09E-19	3.20E-19	5.16E-19	1.63E-19
2.31E-18	3.42E-18	2.97E-18	8.37E-19	1.46E-18	5.14E-19	1.20E-18	2.89E-18	1.88E-20	2.58E-20	2.29E-20	2.02E-23	2.68E-19	4.24E-19	6.15E-22
4.10E-18	6.48E-18	6.15E-18	3.84E-18	5.26E-18	1.53E-18	2.64E-18	4.66E-18	8.94E-19	3.55E-19	6.64E-21	1.56E-19	1.31E-19	4.99E-20	5.17E-19
7.65E-18	1.05E-17	9.25E-18	6.31E-18	7.17E-18	2.82E-18	4.63E-18	5.59E-18	2.60E-18	5.50E-19	2.05E-19	1.03E-18	1.23E-19	1.96E-19	1.45E-18
1.02E-17	1.27E-17	1.18E-17	1.06E-17	8.00E-18	4.83E-18	6.30E-18	6.57E-18	4.53E-18	2.78E-18	2.39E-18	2.12E-18	5.06E-19	1.15E-18	2.05E-18
1.33E-17	1.62E-17	1.57E-17	1.64E-17	1.35E-17	9.62E-18	1.11E-17	8.24E-18	8.29E-18	6.22E-18	6.30E-18	3.14E-18	3.48E-18	1.98E-18	3.11E-18
1.90E-17	2.23E-17	1.89E-17	2.27E-17	1.92E-17	1.53E-17	1.46E-17	9.72E-18	1.27E-17	1.13E-17	1.01E-17	4.60E-18	9.38E-18	3.75E-18	3.92E-18
2.68E-17	2.86E-17	2.02E-17	2.62E-17	2.31E-17	1.79E-17	1.74E-17	1.39E-17	1.84E-17	1.71E-17	1.33E-17	1.22E-17	1.54E-17	7.52E-18	5.16E-18
2.81E-17	2.76E-17	2.60E-17	2.72E-17	2.71E-17	1.98E-17	2.18E-17	2.20E-17	2.27E-17	1.94E-17	1.89E-17	1.85E-17	1.91E-17	8.31E-18	5.86E-18
3.20E-17	2.31E-17	2.81E-17	3.17E-17	2.98E-17	2.43E-17	2.54E-17	2.99E-17	2.38E-17	2.34E-17	2.26E-17	2.02E-17	2.33E-17	1.16E-17	5.76E-18

2.93E-17	1.70E-17	2.65E-17	3.53E-17	3.34E-17	2.87E-17	2.87E-17	2.86E-17	2.32E-17	2.53E-17	2.73E-17	2.57E-17	2.84E-17	1.63E-17	7.14E-18
2.91E-17	1.70E-17	3.02E-17	4.03E-17	3.84E-17	3.05E-17	2.86E-17	2.46E-17	2.38E-17	2.85E-17	3.22E-17	2.69E-17	3.08E-17	2.15E-17	1.51E-17
3.50E-17	2.90E-17	4.10E-17	4.59E-17	4.33E-17	3.37E-17	2.87E-17	2.54E-17	2.40E-17	3.18E-17	3.30E-17	3.16E-17	3.37E-17	2.26E-17	2.03E-17
4.47E-17	4.79E-17	5.03E-17	4.81E-17	4.46E-17	3.80E-17	2.86E-17	3.04E-17	3.02E-17	3.65E-17	3.33E-17	3.59E-17	3.59E-17	2.34E-17	1.83E-17
5.70E-17	5.25E-17	5.06E-17	4.79E-17	4.64E-17	3.98E-17	3.56E-17	3.72E-17	3.97E-17	4.07E-17	4.11E-17	3.84E-17	3.92E-17	2.76E-17	1.83E-17
6.26E-17	5.56E-17	5.41E-17	5.55E-17	5.17E-17	4.48E-17	4.81E-17	4.34E-17	4.50E-17	4.19E-17	4.94E-17	4.08E-17	3.89E-17	4.04E-17	2.53E-17
6.48E-17	6.27E-17	6.37E-17	6.47E-17	5.36E-17	5.33E-17	6.32E-17	5.31E-17	4.89E-17	4.52E-17	5.10E-17	4.44E-17	4.23E-17	4.76E-17	3.26E-17
6.29E-17	6.33E-17	7.04E-17	6.75E-17	5.51E-17	6.85E-17	7.12E-17	6.16E-17	5.91E-17	5.52E-17	5.61E-17	4.25E-17	5.04E-17	5.31E-17	3.81E-17
5.66E-17	5.78E-17	7.18E-17	6.39E-17	5.87E-17	8.11E-17	6.89E-17	7.03E-17	6.94E-17	6.72E-17	6.25E-17	5.25E-17	5.81E-17	5.83E-17	4.89E-17
5.35E-17	5.88E-17	7.46E-17	6.22E-17	6.40E-17	8.78E-17	7.33E-17	7.45E-17	7.92E-17	7.44E-17	7.14E-17	6.99E-17	6.53E-17	6.47E-17	5.55E-17
5.91E-17	6.40E-17	7.59E-17	6.58E-17	6.67E-17	9.36E-17	7.95E-17	7.94E-17	8.73E-17	8.79E-17	8.39E-17	8.25E-17	7.43E-17	6.44E-17	6.35E-17
3.94E-15	4.33E-15	4.52E-15	4.62E-15	4.55E-15	4.42E-15	4.45E-15	4.56E-15	4.55E-15	4.78E-15	5.07E-15	5.32E-15	5.38E-15	5.55E-15	5.83E-15

Quadrant 3 Data (16384 value

-4.34E-09	-4.36E-09	-6.57E-09	-8.89E-09	-9.86E-09	-3.62E-08	-3.64E-08
-3.77E-09	-3.61E-09	-5.04E-09	-7.76E-09	-8.28E-09	-3.57E-08	-3.58E-08
-3.86E-09	-3.47E-09	-4.36E-09	-6.50E-09	-7.44E-09	-3.54E-08	-3.46E-08
-4.40E-09	-3.83E-09	-4.93E-09	-6.88E-09	-8.11E-09	-3.49E-08	-3.43E-08
-4.44E-09	-4.50E-09	-5.36E-09	-7.30E-09	-7.87E-09	-3.44E-08	-3.36E-08
-4.10E-09	-4.71E-09	-5.78E-09	-6.96E-09	-7.09E-09	-3.38E-08	-3.36E-08
-3.69E-09	-3.99E-09	-5.47E-09	-6.27E-09	-6.05E-09	-3.30E-08	-3.35E-08
-3.30E-09	-3.63E-09	-5.03E-09	-5.76E-09	-5.83E-09	-3.28E-08	-3.32E-08
-2.70E-09	-3.62E-09	-4.72E-09	-5.63E-09	-5.58E-09	-3.33E-08	-3.31E-08
-2.18E-09	-3.32E-09	-3.87E-09	-5.08E-09	-4.93E-09	-3.28E-08	-3.29E-08
-1.49E-09	-2.99E-09	-3.43E-09	-3.98E-09	-3.71E-09	-3.26E-08	-3.26E-08
-8.79E-10	-2.38E-09	-2.51E-09	-2.78E-09	-2.89E-09	-3.23E-08	-3.25E-08
-6.18E-10	-1.42E-09	-1.41E-09	-1.77E-09	-1.99E-09	-3.23E-08	-3.25E-08
2.50E-11	-5.02E-10	-4.89E-10	-1.04E-09	-1.22E-09	-3.18E-08	-3.18E-08
1.00E-09	-3.02E-10	-2.91E-10	-1.00E-09	-1.11E-09	-3.16E-08	-3.11E-08
2.10E-09	-1.91E-10	-6.21E-10	-1.12E-09	-6.03E-10	-3.11E-08	-3.11E-08
2.62E-09	2.89E-10	-3.32E-10	-6.13E-10	7.19E-12	-3.06E-08	-3.09E-08
2.69E-09	1.15E-09	4.96E-10	2.17E-10	6.11E-11	-3.00E-08	-3.05E-08
2.55E-09	2.01E-09	1.35E-09	7.50E-10	2.29E-10	-2.99E-08	-2.99E-08
1.86E-09	2.31E-09	1.25E-09	1.08E-09	4.15E-10	-2.93E-08	-2.92E-08
1.74E-09	1.84E-09	1.17E-09	1.48E-09	1.03E-09	-2.86E-08	-2.81E-08
1.71E-09	1.94E-09	1.86E-09	2.15E-09	2.12E-09	-2.80E-08	-2.77E-08
1.92E-09	2.57E-09	2.29E-09	2.98E-09	3.11E-09	-2.77E-08	-2.76E-08
2.16E-09	2.86E-09	2.78E-09	3.53E-09	3.95E-09	-2.74E-08	-2.69E-08
2.63E-09	3.25E-09	3.18E-09	4.12E-09	4.80E-09	-2.68E-08	-2.63E-08
3.37E-09	3.85E-09	3.88E-09	5.04E-09	5.54E-09	-2.64E-08	-2.63E-08
3.89E-09	4.64E-09	4.50E-09	5.04E-09	5.34E-09	-2.59E-08	-2.58E-08
4.23E-09	5.03E-09	4.46E-09	4.79E-09	5.20E-09	-2.54E-08	-2.58E-08
4.77E-09	5.28E-09	4.97E-09	5.33E-09	5.64E-09	-2.48E-08	-2.51E-08
5.72E-09	5.46E-09	5.89E-09	6.09E-09	6.34E-09	-2.45E-08	-2.43E-08
5.93E-09	5.76E-09	6.68E-09	7.04E-09	7.19E-09	-2.40E-08	-2.35E-08
5.87E-09	6.25E-09	7.10E-09	7.49E-09	7.81E-09	-2.37E-08	-2.33E-08
6.22E-09	6.88E-09	7.56E-09	7.89E-09	8.51E-09	-2.30E-08	-2.26E-08
6.97E-09	7.92E-09	8.14E-09	8.72E-09	9.77E-09	-2.21E-08	-2.23E-08
7.82E-09	8.90E-09	9.04E-09	9.90E-09	1.18E-08	-2.13E-08	-2.13E-08
9.11E-09	1.11E-08	1.09E-08	1.17E-08	1.39E-08	-2.06E-08	-1.99E-08
1.09E-08	1.36E-08	1.31E-08	1.36E-08	1.53E-08	-1.97E-08	-1.93E-08
1.22E-08	1.47E-08	1.45E-08	1.50E-08	1.58E-08	-1.86E-08	-1.88E-08
1.27E-08	1.39E-08	1.46E-08	1.52E-08	1.53E-08	-1.83E-08	-1.82E-08
1.20E-08	1.27E-08	1.43E-08	1.57E-08	1.46E-08	-1.78E-08	-1.74E-08
1.05E-08	1.14E-08	1.38E-08	1.50E-08	1.34E-08	-1.70E-08	-1.65E-08
9.52E-09	1.02E-08	1.25E-08	1.34E-08	1.22E-08	-1.63E-08	-1.63E-08
8.82E-09	9.24E-09	1.11E-08	1.17E-08	1.14E-08	-1.58E-08	-1.56E-08
8.11E-09	8.59E-09	9.97E-09	1.03E-08	1.11E-08	-1.52E-08	-1.51E-08
8.01E-09	8.49E-09	9.61E-09	9.82E-09	1.08E-08	-1.47E-08	-1.49E-08
8.15E-09	9.07E-09	9.97E-09	1.03E-08	1.08E-08	-1.44E-08	-1.46E-08
8.50E-09	9.43E-09	1.03E-08	1.07E-08	1.11E-08	-1.43E-08	-1.41E-08
8.99E-09	9.93E-09	1.08E-08	1.10E-08	1.15E-08	-1.38E-08	-1.36E-08
9.77E-09	1.04E-08	1.09E-08	1.09E-08	1.20E-08	-1.31E-08	-1.33E-08
1.00E-08	1.00E-08	1.06E-08	1.05E-08	1.17E-08	-1.28E-08	-1.30E-08
1.04E-08	9.79E-09	1.01E-08	9.80E-09	1.16E-08	-1.26E-08	-1.26E-08
1.12E-08	1.03E-08	1.02E-08	1.03E-08	1.18E-08	-1.22E-08	-1.18E-08
1.16E-08	1.04E-08	1.05E-08	1.07E-08	1.20E-08	-1.15E-08	-1.07E-08
1.06E-08	1.03E-08	1.07E-08	1.10E-08	1.19E-08	-1.04E-08	-9.82E-09
1.11E-08	1.03E-08	1.09E-08	1.16E-08	1.23E-08	-9.60E-09	-8.72E-09
1.15E-08	1.02E-08	1.08E-08	1.17E-08	1.27E-08	-8.41E-09	-7.52E-09
1.11E-08	9.99E-09	1.06E-08	1.19E-08	1.31E-08	-6.76E-09	-5.93E-09
1.04E-08	9.90E-09	1.03E-08	1.19E-08	1.36E-08	-6.34E-09	-4.86E-09
1.00E-08	1.00E-08	1.04E-08	1.23E-08	1.36E-08	-7.01E-09	-6.24E-09
1.01E-08	1.05E-08	1.07E-08	1.28E-08	1.35E-08	-7.08E-09	-6.56E-09
1.01E-08	1.10E-08	1.10E-08	1.30E-08	1.36E-08	-6.81E-09	-5.83E-09
1.04E-08	1.14E-08	1.11E-08	1.28E-08	1.41E-08	-6.18E-09	-4.96E-09
1.10E-08	1.15E-08	1.11E-08	1.28E-08	1.42E-08	-4.87E-09	-4.11E-09
1.12E-08	1.12E-08	1.11E-08	1.26E-08	1.42E-08	-3.86E-09	-3.84E-09
1.12E-08	1.09E-08	1.12E-08	1.24E-08	1.37E-08	-3.22E-09	-3.50E-09
1.02E-08	1.12E-08	1.10E-08	1.23E-08	1.34E-08	-2.78E-09	-2.94E-09
9.80E-09	1.10E-08	1.11E-08	1.25E-08	1.35E-08	-2.42E-09	-2.55E-09
9.74E-09	1.08E-08	1.12E-08	1.23E-08	1.36E-08	-1.53E-09	-2.02E-09
1.00E-08	1.06E-08	1.09E-08	1.19E-08	1.34E-08	-8.02E-10	-1.24E-09
1.05E-08	1.06E-08	1.10E-08	1.21E-08	1.34E-08	-1.99E-10	-4.19E-10
1.12E-08	1.10E-08	1.13E-08	1.16E-08	1.33E-08	5.21E-10	6.52E-10
1.18E-08	1.14E-08	1.14E-08	1.16E-08	1.32E-08	1.53E-09	1.67E-09
1.20E-08	1.19E-08	1.15E-08	1.16E-08	1.31E-08	2.40E-09	2.02E-09
1.14E-08	1.15E-08	1.11E-08	1.15E-08	1.31E-08	3.05E-09	2.50E-09
1.02E-08	1.06E-08	1.09E-08	1.15E-08	1.30E-08	3.78E-09	2.85E-09
9.17E-09	1.00E-08	1.11E-08	1.18E-08	1.35E-08	4.38E-09	3.41E-09
8.78E-09	9.68E-09	1.11E-08	1.20E-08	1.36E-08	5.02E-09	4.19E-09
8.60E-09	9.57E-09	1.08E-08	1.17E-08	1.28E-08	5.75E-09	4.64E-09

8.57E-09	9.66E-09	1.02E-08	1.08E-08	1.17E-08	6.46E-09	5.33E-09
8.58E-09	9.78E-09	9.81E-09	1.02E-08	1.13E-08	6.88E-09	5.75E-09
8.91E-09	1.01E-08	1.01E-08	1.05E-08	1.15E-08	7.62E-09	6.49E-09
9.02E-09	9.43E-09	9.95E-09	1.04E-08	1.16E-08	8.21E-09	7.59E-09
8.76E-09	8.54E-09	9.60E-09	1.04E-08	1.14E-08	9.05E-09	8.90E-09
8.37E-09	8.59E-09	9.43E-09	9.88E-09	1.17E-08	1.01E-08	1.01E-08
7.74E-09	8.81E-09	9.13E-09	9.66E-09	1.14E-08	1.09E-08	1.08E-08
7.50E-09	8.50E-09	8.52E-09	9.37E-09	1.08E-08	1.19E-08	1.15E-08
7.87E-09	8.13E-09	8.17E-09	8.96E-09	1.05E-08	1.25E-08	1.25E-08
7.38E-09	7.46E-09	7.83E-09	8.92E-09	9.98E-09	1.29E-08	1.36E-08
6.69E-09	7.27E-09	7.69E-09	8.41E-09	9.47E-09	1.37E-08	1.44E-08
6.09E-09	6.99E-09	7.55E-09	7.99E-09	9.21E-09	1.51E-08	1.53E-08
6.16E-09	6.84E-09	7.30E-09	7.90E-09	9.39E-09	1.54E-08	1.60E-08
6.90E-09	6.89E-09	7.25E-09	7.90E-09	8.93E-09	1.57E-08	1.65E-08
7.01E-09	6.73E-09	6.94E-09	7.86E-09	8.51E-09	1.64E-08	1.71E-08
6.48E-09	6.40E-09	6.58E-09	7.38E-09	7.84E-09	1.74E-08	1.79E-08
5.64E-09	6.37E-09	6.31E-09	6.49E-09	7.06E-09	1.87E-08	1.90E-08
5.13E-09	6.26E-09	5.62E-09	6.00E-09	6.85E-09	1.98E-08	1.98E-08
4.50E-09	5.63E-09	5.17E-09	6.36E-09	7.11E-09	2.08E-08	2.07E-08
3.90E-09	4.55E-09	4.63E-09	6.81E-09	7.15E-09	2.22E-08	2.16E-08
3.43E-09	4.20E-09	4.41E-09	6.90E-09	7.43E-09	2.33E-08	2.24E-08
3.53E-09	3.70E-09	4.06E-09	6.93E-09	7.54E-09	2.42E-08	2.37E-08
3.81E-09	3.72E-09	3.92E-09	6.14E-09	7.08E-09	2.52E-08	2.44E-08
3.54E-09	3.71E-09	3.44E-09	4.84E-09	5.51E-09	2.58E-08	2.52E-08
2.97E-09	3.30E-09	3.00E-09	4.37E-09	4.67E-09	2.63E-08	2.63E-08
2.29E-09	2.89E-09	3.02E-09	4.03E-09	4.00E-09	2.74E-08	2.76E-08
1.63E-09	2.42E-09	3.29E-09	3.60E-09	3.76E-09	2.89E-08	2.88E-08
1.49E-09	2.17E-09	3.46E-09	3.36E-09	3.79E-09	3.03E-08	3.00E-08
9.64E-10	1.25E-09	3.34E-09	3.23E-09	3.74E-09	3.17E-08	3.18E-08
3.01E-10	6.45E-10	2.81E-09	2.96E-09	3.64E-09	3.37E-08	3.35E-08
1.84E-11	3.65E-10	1.96E-09	2.70E-09	3.10E-09	3.47E-08	3.53E-08
-5.17E-10	-9.82E-11	1.67E-09	2.13E-09	2.64E-09	3.54E-08	3.69E-08
-9.10E-10	-5.21E-10	8.44E-10	9.86E-10	1.97E-09	3.70E-08	3.77E-08
-9.98E-10	-1.05E-09	2.95E-10	2.74E-10	6.45E-10	3.89E-08	3.77E-08
-1.56E-09	-1.15E-09	1.06E-10	-1.83E-10	7.25E-11	4.06E-08	3.91E-08
-1.58E-09	-1.20E-09	-4.36E-10	-5.05E-10	-8.77E-11	4.15E-08	4.09E-08
-1.44E-09	-1.60E-09	-1.22E-09	-9.12E-10	-1.99E-10	4.20E-08	4.23E-08
-1.97E-09	-2.28E-09	-1.84E-09	-1.60E-09	-9.97E-10	4.25E-08	4.26E-08
-2.20E-09	-2.60E-09	-2.73E-09	-2.26E-09	-1.85E-09	4.33E-08	4.34E-08
-2.31E-09	-2.89E-09	-3.11E-09	-3.09E-09	-2.92E-09	4.43E-08	4.44E-08
-3.14E-09	-3.46E-09	-3.68E-09	-3.55E-09	-4.06E-09	4.55E-08	4.55E-08
-3.75E-09	-4.33E-09	-4.60E-09	-3.85E-09	-4.31E-09	4.75E-08	4.63E-08
-4.07E-09	-5.08E-09	-5.20E-09	-4.11E-09	-5.17E-09	4.89E-08	4.77E-08
-4.33E-09	-5.87E-09	-5.12E-09	-4.95E-09	-5.99E-09	5.02E-08	4.91E-08
-4.83E-09	-6.02E-09	-5.44E-09	-5.91E-09	-6.70E-09	5.14E-08	5.08E-08
-5.92E-09	-6.14E-09	-5.84E-09	-6.92E-09	-7.83E-09	5.24E-08	5.23E-08
-6.67E-09	-6.64E-09	-6.98E-09	-7.55E-09	-8.68E-09	5.32E-08	5.31E-08
-6.89E-09	-7.16E-09	-7.76E-09	-7.84E-09	-9.54E-09	5.42E-08	5.39E-08
-7.15E-09	-7.68E-09	-8.03E-09	-8.41E-09	-9.99E-09	5.50E-08	5.49E-08
-7.21E-09	-8.15E-09	-7.94E-09	-8.88E-09	-9.91E-09	5.63E-08	5.63E-08

Quadrant 3 Data Squared (16

1.88E-17	1.90E-17	4.32E-17	7.91E-17	9.71E-17	1.31E-15	1.33E-15
1.42E-17	1.31E-17	2.54E-17	6.02E-17	6.86E-17	1.27E-15	1.28E-15
1.49E-17	1.20E-17	1.90E-17	4.22E-17	5.54E-17	1.26E-15	1.20E-15
1.93E-17	1.47E-17	2.43E-17	4.74E-17	6.57E-17	1.22E-15	1.17E-15
1.97E-17	2.03E-17	2.88E-17	5.32E-17	6.20E-17	1.18E-15	1.13E-15
1.68E-17	2.21E-17	3.35E-17	4.85E-17	5.02E-17	1.15E-15	1.13E-15
1.36E-17	1.59E-17	2.99E-17	3.94E-17	3.66E-17	1.09E-15	1.12E-15
1.09E-17	1.32E-17	2.53E-17	3.32E-17	3.40E-17	1.08E-15	1.10E-15
7.29E-18	1.31E-17	2.23E-17	3.17E-17	3.12E-17	1.11E-15	1.09E-15
4.73E-18	1.10E-17	1.50E-17	2.58E-17	2.43E-17	1.07E-15	1.09E-15
2.22E-18	8.92E-18	1.18E-17	1.59E-17	1.37E-17	1.07E-15	1.06E-15
7.72E-19	5.68E-18	6.31E-18	7.73E-18	8.32E-18	1.04E-15	1.06E-15
3.82E-19	2.02E-18	1.99E-18	3.12E-18	3.96E-18	1.04E-15	1.06E-15
6.25E-22	2.52E-19	2.39E-19	1.08E-18	1.48E-18	1.01E-15	1.01E-15
1.00E-18	9.14E-20	8.46E-20	1.00E-18	1.23E-18	1.00E-15	9.69E-16
4.39E-18	3.64E-20	3.85E-19	1.27E-18	3.63E-19	9.69E-16	9.67E-16
6.84E-18	8.37E-20	1.10E-19	3.76E-19	5.17E-23	9.38E-16	9.52E-16
7.22E-18	1.32E-18	2.46E-19	4.71E-20	3.74E-21	9.01E-16	9.31E-16
6.49E-18	4.03E-18	1.83E-18	5.63E-19	5.26E-20	8.96E-16	8.94E-16
3.47E-18	5.31E-18	1.57E-18	1.16E-18	1.72E-19	8.58E-16	8.50E-16
3.02E-18	3.37E-18	1.37E-18	2.20E-18	1.05E-18	8.19E-16	7.92E-16
2.93E-18	3.77E-18	3.47E-18	4.61E-18	4.49E-18	7.86E-16	7.67E-16
3.68E-18	6.62E-18	5.23E-18	8.88E-18	9.69E-18	7.69E-16	7.59E-16
4.68E-18	8.20E-18	7.75E-18	1.25E-17	1.56E-17	7.48E-16	7.23E-16
6.93E-18	1.06E-17	1.01E-17	1.70E-17	2.30E-17	7.20E-16	6.90E-16
1.13E-17	1.48E-17	1.50E-17	2.54E-17	3.07E-17	6.98E-16	6.91E-16
1.51E-17	2.15E-17	2.02E-17	2.54E-17	2.85E-17	6.73E-16	6.67E-16
1.79E-17	2.53E-17	1.99E-17	2.30E-17	2.70E-17	6.46E-16	6.63E-16
2.27E-17	2.79E-17	2.47E-17	2.84E-17	3.18E-17	6.14E-16	6.32E-16
3.27E-17	2.98E-17	3.47E-17	3.71E-17	4.02E-17	5.99E-16	5.89E-16
3.51E-17	3.32E-17	4.46E-17	4.95E-17	5.17E-17	5.77E-16	5.54E-16

3.45E-17	3.91E-17	5.04E-17	5.62E-17	6.10E-17	5.60E-16	5.42E-16
3.87E-17	4.73E-17	5.72E-17	6.23E-17	7.23E-17	5.27E-16	5.12E-16
4.86E-17	6.28E-17	6.63E-17	7.61E-17	9.54E-17	4.86E-16	4.97E-16
6.12E-17	7.93E-17	8.17E-17	9.79E-17	1.39E-16	4.52E-16	4.55E-16
8.29E-17	1.22E-16	1.19E-16	1.36E-16	1.93E-16	4.24E-16	3.96E-16
1.19E-16	1.86E-16	1.72E-16	1.85E-16	2.33E-16	3.87E-16	3.74E-16
1.48E-16	2.15E-16	2.10E-16	2.24E-16	2.48E-16	3.46E-16	3.55E-16
1.62E-16	1.93E-16	2.13E-16	2.31E-16	2.34E-16	3.35E-16	3.30E-16
1.45E-16	1.61E-16	2.05E-16	2.45E-16	2.14E-16	3.15E-16	3.01E-16
1.11E-16	1.30E-16	1.91E-16	2.25E-16	1.80E-16	2.89E-16	2.73E-16
9.06E-17	1.03E-16	1.57E-16	1.79E-16	1.49E-16	2.66E-16	2.64E-16
7.78E-17	8.53E-17	1.24E-16	1.36E-16	1.29E-16	2.49E-16	2.44E-16
6.58E-17	7.39E-17	9.94E-17	1.07E-16	1.24E-16	2.32E-16	2.28E-16
6.42E-17	7.21E-17	9.23E-17	9.64E-17	1.16E-16	2.15E-16	2.22E-16
6.64E-17	8.23E-17	9.94E-17	1.06E-16	1.17E-16	2.07E-16	2.14E-16
7.23E-17	8.89E-17	1.06E-16	1.15E-16	1.23E-16	2.04E-16	2.00E-16
8.07E-17	9.87E-17	1.17E-16	1.20E-16	1.33E-16	1.91E-16	1.84E-16
9.55E-17	1.07E-16	1.19E-16	1.20E-16	1.43E-16	1.72E-16	1.77E-16
1.00E-16	1.01E-16	1.12E-16	1.09E-16	1.36E-16	1.63E-16	1.68E-16
1.09E-16	9.59E-17	1.02E-16	9.60E-17	1.35E-16	1.60E-16	1.58E-16
1.25E-16	1.06E-16	1.04E-16	1.06E-16	1.39E-16	1.49E-16	1.40E-16
1.34E-16	1.09E-16	1.09E-16	1.14E-16	1.44E-16	1.31E-16	1.15E-16
1.12E-16	1.06E-16	1.15E-16	1.21E-16	1.43E-16	1.09E-16	9.65E-17
1.23E-16	1.06E-16	1.19E-16	1.36E-16	1.52E-16	9.21E-17	7.61E-17
1.32E-16	1.03E-16	1.16E-16	1.37E-16	1.62E-16	7.07E-17	5.65E-17
1.24E-16	9.98E-17	1.13E-16	1.42E-16	1.72E-16	4.57E-17	3.52E-17
1.09E-16	9.80E-17	1.07E-16	1.42E-16	1.85E-16	4.02E-17	2.37E-17
1.00E-16	1.01E-16	1.08E-16	1.50E-16	1.86E-16	4.91E-17	3.89E-17
1.03E-16	1.10E-16	1.15E-16	1.64E-16	1.84E-16	5.02E-17	4.30E-17
1.01E-16	1.22E-16	1.22E-16	1.68E-16	1.86E-16	4.64E-17	3.40E-17
1.07E-16	1.31E-16	1.24E-16	1.64E-16	1.99E-16	3.82E-17	2.46E-17
1.22E-16	1.33E-16	1.23E-16	1.63E-16	2.02E-16	2.37E-17	1.69E-17
1.26E-16	1.25E-16	1.23E-16	1.59E-16	2.01E-16	1.49E-17	1.47E-17
1.25E-16	1.19E-16	1.25E-16	1.54E-16	1.87E-16	1.04E-17	1.23E-17
1.04E-16	1.24E-16	1.20E-16	1.51E-16	1.79E-16	7.71E-18	8.62E-18
9.61E-17	1.22E-16	1.23E-16	1.57E-16	1.82E-16	5.85E-18	6.49E-18
9.49E-17	1.16E-16	1.26E-16	1.52E-16	1.84E-16	2.34E-18	4.07E-18
1.00E-16	1.12E-16	1.19E-16	1.42E-16	1.81E-16	6.44E-19	1.53E-18
1.10E-16	1.12E-16	1.21E-16	1.45E-16	1.80E-16	3.96E-20	1.76E-19
1.26E-16	1.22E-16	1.28E-16	1.35E-16	1.77E-16	2.72E-19	4.25E-19
1.39E-16	1.30E-16	1.31E-16	1.35E-16	1.75E-16	2.34E-18	2.80E-18
1.43E-16	1.42E-16	1.32E-16	1.34E-16	1.72E-16	5.77E-18	4.08E-18
1.30E-16	1.33E-16	1.24E-16	1.31E-16	1.70E-16	9.32E-18	6.26E-18
1.05E-16	1.13E-16	1.20E-16	1.31E-16	1.69E-16	1.43E-17	8.14E-18
8.40E-17	1.01E-16	1.24E-16	1.38E-16	1.82E-16	1.92E-17	1.16E-17
7.72E-17	9.37E-17	1.23E-16	1.45E-16	1.85E-16	2.52E-17	1.76E-17
7.40E-17	9.16E-17	1.17E-16	1.36E-16	1.63E-16	3.30E-17	2.15E-17
7.35E-17	9.33E-17	1.04E-16	1.17E-16	1.36E-16	4.17E-17	2.84E-17
7.36E-17	9.57E-17	9.62E-17	1.04E-16	1.28E-16	4.73E-17	3.31E-17
7.93E-17	1.02E-16	1.01E-16	1.11E-16	1.32E-16	5.80E-17	4.21E-17
8.13E-17	8.90E-17	9.90E-17	1.07E-16	1.35E-16	6.75E-17	5.77E-17
7.67E-17	7.30E-17	9.21E-17	1.07E-16	1.31E-16	8.19E-17	7.92E-17
7.01E-17	7.37E-17	8.90E-17	9.76E-17	1.36E-16	1.02E-16	1.02E-16
5.99E-17	7.76E-17	8.33E-17	9.33E-17	1.31E-16	1.19E-16	1.18E-16
5.62E-17	7.23E-17	7.26E-17	8.79E-17	1.16E-16	1.42E-16	1.31E-16
6.20E-17	6.60E-17	6.68E-17	8.03E-17	1.10E-16	1.57E-16	1.57E-16
5.44E-17	5.56E-17	6.14E-17	7.95E-17	9.96E-17	1.67E-16	1.84E-16
4.47E-17	5.28E-17	5.92E-17	7.07E-17	8.97E-17	1.89E-16	2.08E-16
3.71E-17	4.89E-17	5.70E-17	6.38E-17	8.49E-17	2.27E-16	2.33E-16
3.79E-17	4.68E-17	5.33E-17	6.24E-17	8.83E-17	2.36E-16	2.55E-16
4.76E-17	4.75E-17	5.26E-17	6.24E-17	7.98E-17	2.45E-16	2.73E-16
4.91E-17	4.53E-17	4.82E-17	6.18E-17	7.24E-17	2.70E-16	2.91E-16
4.19E-17	4.09E-17	4.34E-17	5.45E-17	6.14E-17	3.04E-16	3.19E-16
3.18E-17	4.05E-17	3.98E-17	4.21E-17	4.98E-17	3.50E-16	3.61E-16
2.64E-17	3.91E-17	3.16E-17	3.59E-17	4.69E-17	3.93E-16	3.94E-16
2.03E-17	3.17E-17	2.67E-17	4.04E-17	5.05E-17	4.31E-16	4.30E-16
1.52E-17	2.07E-17	2.14E-17	4.64E-17	5.12E-17	4.93E-16	4.67E-16
1.18E-17	1.76E-17	1.94E-17	4.76E-17	5.53E-17	5.43E-16	5.04E-16
1.25E-17	1.37E-17	1.65E-17	4.80E-17	5.69E-17	5.84E-16	5.61E-16
1.45E-17	1.39E-17	1.54E-17	3.78E-17	5.01E-17	6.33E-16	5.97E-16
1.25E-17	1.37E-17	1.18E-17	2.34E-17	3.04E-17	6.68E-16	6.36E-16
8.85E-18	1.09E-17	8.99E-18	1.91E-17	2.18E-17	6.92E-16	6.90E-16
5.22E-18	8.32E-18	9.11E-18	1.63E-17	1.60E-17	7.48E-16	7.63E-16
2.64E-18	5.86E-18	1.08E-17	1.30E-17	1.42E-17	8.34E-16	8.27E-16
2.22E-18	4.70E-18	1.19E-17	1.13E-17	1.44E-17	9.17E-16	9.01E-16
9.30E-19	1.57E-18	1.11E-17	1.04E-17	1.40E-17	1.01E-15	1.01E-15
9.07E-20	4.16E-19	7.89E-18	8.77E-18	1.33E-17	1.14E-15	1.12E-15
3.39E-22	1.33E-19	3.85E-18	7.28E-18	9.63E-18	1.20E-15	1.25E-15
2.67E-19	9.65E-21	2.78E-18	4.55E-18	6.97E-18	1.25E-15	1.36E-15
8.29E-19	2.72E-19	7.12E-19	9.72E-19	3.90E-18	1.37E-15	1.42E-15
9.95E-19	1.10E-18	8.68E-20	7.53E-20	4.16E-19	1.51E-15	1.42E-15
2.45E-18	1.33E-18	1.12E-20	3.35E-20	5.26E-21	1.65E-15	1.53E-15
2.49E-18	1.43E-18	1.90E-19	2.55E-19	7.68E-21	1.72E-15	1.67E-15
2.08E-18	2.55E-18	1.49E-18	8.32E-19	3.94E-20	1.77E-15	1.79E-15
3.88E-18	5.19E-18	3.39E-18	2.55E-18	9.94E-19	1.81E-15	1.81E-15
4.84E-18	6.77E-18	7.44E-18	5.12E-18	3.41E-18	1.87E-15	1.89E-15

5.34E-18	8.38E-18	9.67E-18	9.55E-18	8.51E-18		1.96E-15	1.97E-15
9.83E-18	1.20E-17	1.36E-17	1.26E-17	1.65E-17		2.07E-15	2.07E-15
1.41E-17	1.88E-17	2.12E-17	1.48E-17	1.86E-17		2.25E-15	2.15E-15
1.66E-17	2.59E-17	2.70E-17	1.69E-17	2.68E-17		2.39E-15	2.27E-15
1.88E-17	3.45E-17	2.62E-17	2.45E-17	3.59E-17		2.52E-15	2.41E-15
2.33E-17	3.62E-17	2.96E-17	3.50E-17	4.49E-17		2.64E-15	2.58E-15
3.50E-17	3.77E-17	3.41E-17	4.79E-17	6.13E-17		2.74E-15	2.73E-15
4.45E-17	4.41E-17	4.87E-17	5.69E-17	7.53E-17		2.83E-15	2.82E-15
4.75E-17	5.12E-17	6.03E-17	6.14E-17	9.10E-17		2.94E-15	2.91E-15
5.12E-17	5.90E-17	6.45E-17	7.07E-17	9.99E-17		3.03E-15	3.01E-15
5.20E-17	6.65E-17	6.30E-17	7.89E-17	9.83E-17		3.17E-15	3.17E-15
6.58E-15	7.31E-15	8.04E-15	9.51E-15	1.14E-14	1.46E-12	9.09E-14	8.98E-14

SUM OF SQUARED QUADRAI	1.46E-12
TOTAL NUMBER VALUES IN (	16,384
AVERAGE SQUARED	8.92E-17
RMS	9.4455E-09

es):

-3.60E-08	-3.59E-08	-3.54E-08	-3.50E-08	-3.58E-08	-3.57E-08	-3.52E-08	-3.52E-08	-3.52E-08	-3.48E-08	-3.47E-08	-3.36E-08	-3.33E-08	-3.37E-08
-3.58E-08	-3.62E-08	-3.58E-08	-3.48E-08	-3.58E-08	-3.57E-08	-3.52E-08	-3.50E-08	-3.53E-08	-3.46E-08	-3.46E-08	-3.41E-08	-3.31E-08	-3.39E-08
-3.47E-08	-3.54E-08	-3.56E-08	-3.48E-08	-3.55E-08	-3.54E-08	-3.46E-08	-3.45E-08	-3.52E-08	-3.46E-08	-3.47E-08	-3.40E-08	-3.27E-08	-3.35E-08
-3.39E-08	-3.45E-08	-3.50E-08	-3.52E-08	-3.51E-08	-3.48E-08	-3.36E-08	-3.36E-08	-3.47E-08	-3.43E-08	-3.45E-08	-3.41E-08	-3.24E-08	-3.28E-08
-3.33E-08	-3.37E-08	-3.47E-08	-3.50E-08	-3.44E-08	-3.38E-08	-3.30E-08	-3.31E-08	-3.41E-08	-3.39E-08	-3.42E-08	-3.37E-08	-3.18E-08	-3.17E-08
-3.33E-08	-3.33E-08	-3.43E-08	-3.41E-08	-3.33E-08	-3.32E-08	-3.35E-08	-3.30E-08	-3.39E-08	-3.39E-08	-3.37E-08	-3.31E-08	-3.14E-08	-3.12E-08
-3.37E-08	-3.31E-08	-3.36E-08	-3.36E-08	-3.29E-08	-3.29E-08	-3.40E-08	-3.33E-08	-3.40E-08	-3.37E-08	-3.37E-08	-3.30E-08	-3.18E-08	-3.12E-08
-3.42E-08	-3.28E-08	-3.31E-08	-3.30E-08	-3.26E-08	-3.20E-08	-3.36E-08	-3.38E-08	-3.39E-08	-3.27E-08	-3.32E-08	-3.27E-08	-3.21E-08	-3.15E-08
-3.40E-08	-3.25E-08	-3.27E-08	-3.28E-08	-3.29E-08	-3.15E-08	-3.33E-08	-3.33E-08	-3.33E-08	-3.16E-08	-3.23E-08	-3.22E-08	-3.18E-08	-3.10E-08
-3.37E-08	-3.26E-08	-3.24E-08	-3.28E-08	-3.28E-08	-3.21E-08	-3.29E-08	-3.27E-08	-3.31E-08	-3.17E-08	-3.19E-08	-3.19E-08	-3.16E-08	-3.03E-08
-3.36E-08	-3.23E-08	-3.25E-08	-3.28E-08	-3.29E-08	-3.23E-08	-3.28E-08	-3.24E-08	-3.29E-08	-3.15E-08	-3.17E-08	-3.18E-08	-3.11E-08	-3.01E-08
-3.34E-08	-3.21E-08	-3.27E-08	-3.25E-08	-3.27E-08	-3.21E-08	-3.23E-08	-3.21E-08	-3.23E-08	-3.12E-08	-3.14E-08	-3.13E-08	-3.08E-08	-3.02E-08
-3.29E-08	-3.15E-08	-3.19E-08	-3.20E-08	-3.25E-08	-3.10E-08	-3.16E-08	-3.13E-08	-3.17E-08	-3.07E-08	-3.11E-08	-3.07E-08	-3.06E-08	-3.02E-08
-3.22E-08	-3.10E-08	-3.11E-08	-3.09E-08	-3.15E-08	-2.99E-08	-3.08E-08	-3.08E-08	-3.11E-08	-3.04E-08	-3.10E-08	-3.02E-08	-3.03E-08	-2.99E-08
-3.20E-08	-3.07E-08	-3.05E-08	-3.03E-08	-3.07E-08	-3.02E-08	-3.04E-08	-3.04E-08	-3.04E-08	-2.98E-08	-3.07E-08	-2.95E-08	-3.00E-08	-2.97E-08
-3.19E-08	-3.07E-08	-3.07E-08	-3.07E-08	-3.03E-08	-3.02E-08	-3.01E-08	-2.96E-08	-2.99E-08	-2.91E-08	-3.04E-08	-2.87E-08	-3.00E-08	-2.96E-08
-3.15E-08	-3.07E-08	-3.07E-08	-3.05E-08	-3.00E-08	-2.97E-08	-2.94E-08	-2.92E-08	-2.92E-08	-2.89E-08	-2.97E-08	-2.80E-08	-2.94E-08	-2.96E-08
-3.04E-08	-3.02E-08	-3.03E-08	-3.02E-08	-2.93E-08	-2.93E-08	-2.90E-08	-2.94E-08	-2.90E-08	-2.89E-08	-2.89E-08	-2.76E-08	-2.91E-08	-2.96E-08
-2.96E-08	-2.94E-08	-2.97E-08	-2.99E-08	-2.90E-08	-2.91E-08	-2.87E-08	-2.89E-08	-2.88E-08	-2.89E-08	-2.87E-08	-2.75E-08	-2.85E-08	-2.92E-08
-2.88E-08	-2.90E-08	-2.94E-08	-2.95E-08	-2.88E-08	-2.86E-08	-2.79E-08	-2.80E-08	-2.85E-08	-2.85E-08	-2.84E-08	-2.75E-08	-2.77E-08	-2.85E-08
-2.80E-08	-2.85E-08	-2.91E-08	-2.90E-08	-2.84E-08	-2.82E-08	-2.73E-08	-2.74E-08	-2.79E-08	-2.77E-08	-2.80E-08	-2.77E-08	-2.77E-08	-2.76E-08
-2.71E-08	-2.76E-08	-2.84E-08	-2.83E-08	-2.79E-08	-2.77E-08	-2.71E-08	-2.71E-08	-2.75E-08	-2.73E-08	-2.74E-08	-2.76E-08	-2.76E-08	-2.65E-08
-2.66E-08	-2.71E-08	-2.79E-08	-2.76E-08	-2.75E-08	-2.69E-08	-2.63E-08	-2.65E-08	-2.71E-08	-2.65E-08	-2.67E-08	-2.72E-08	-2.67E-08	-2.52E-08
-2.65E-08	-2.69E-08	-2.76E-08	-2.75E-08	-2.72E-08	-2.63E-08	-2.60E-08	-2.62E-08	-2.66E-08	-2.58E-08	-2.60E-08	-2.62E-08	-2.53E-08	-2.43E-08
-2.62E-08	-2.65E-08	-2.68E-08	-2.70E-08	-2.63E-08	-2.55E-08	-2.58E-08	-2.59E-08	-2.56E-08	-2.56E-08	-2.60E-08	-2.57E-08	-2.39E-08	-2.39E-08
-2.56E-08	-2.59E-08	-2.64E-08	-2.59E-08	-2.55E-08	-2.49E-08	-2.56E-08	-2.55E-08	-2.49E-08	-2.56E-08	-2.59E-08	-2.60E-08	-2.36E-08	-2.40E-08
-2.52E-08	-2.53E-08	-2.57E-08	-2.48E-08	-2.48E-08	-2.50E-08	-2.54E-08	-2.50E-08	-2.44E-08	-2.52E-08	-2.57E-08	-2.60E-08	-2.43E-08	-2.37E-08
-2.55E-08	-2.46E-08	-2.50E-08	-2.42E-08	-2.40E-08	-2.46E-08	-2.47E-08	-2.45E-08	-2.39E-08	-2.46E-08	-2.51E-08	-2.55E-08	-2.46E-08	-2.40E-08
-2.47E-08	-2.41E-08	-2.39E-08	-2.34E-08	-2.31E-08	-2.37E-08	-2.37E-08	-2.42E-08	-2.39E-08	-2.41E-08	-2.46E-08	-2.47E-08	-2.42E-08	-2.39E-08
-2.39E-08	-2.34E-08	-2.28E-08	-2.18E-08	-2.16E-08	-2.26E-08	-2.31E-08	-2.37E-08	-2.33E-08	-2.35E-08	-2.41E-08	-2.40E-08	-2.36E-08	-2.34E-08
-2.37E-08	-2.27E-08	-2.17E-08	-2.00E-08	-2.01E-08	-2.13E-08	-2.27E-08	-2.30E-08	-2.28E-08	-2.35E-08	-2.37E-08	-2.39E-08	-2.29E-08	-2.27E-08
-2.31E-08	-2.20E-08	-2.06E-08	-1.86E-08	-1.87E-08	-2.02E-08	-2.18E-08	-2.23E-08	-2.23E-08	-2.30E-08	-2.29E-08	-2.37E-08	-2.24E-08	-2.22E-08
-2.26E-08	-2.13E-08	-1.98E-08	-1.79E-08	-1.83E-08	-1.97E-08	-2.13E-08	-2.16E-08	-2.18E-08	-2.21E-08	-2.20E-08	-2.29E-08	-2.18E-08	-2.16E-08
-2.22E-08	-2.06E-08	-1.96E-08	-1.83E-08	-1.90E-08	-1.95E-08	-2.11E-08	-2.10E-08	-2.14E-08	-2.19E-08	-2.15E-08	-2.22E-08	-2.17E-08	-2.08E-08
-2.13E-08	-2.05E-08	-1.97E-08	-1.90E-08	-1.93E-08	-1.97E-08	-2.07E-08	-2.04E-08	-2.06E-08	-2.09E-08	-2.09E-08	-2.14E-08	-2.12E-08	-2.06E-08
-1.99E-08	-2.01E-08	-1.96E-08	-1.88E-08	-1.94E-08	-1.96E-08	-2.00E-08	-1.99E-08	-1.93E-08	-1.96E-08	-2.00E-08	-2.05E-08	-2.07E-08	-2.07E-08
-1.84E-08	-1.90E-08	-1.91E-08	-1.80E-08	-1.90E-08	-1.98E-08	-1.94E-08	-1.91E-08	-1.82E-08	-1.80E-08	-1.88E-08	-1.97E-08	-2.00E-08	-2.00E-08
-1.78E-08	-1.85E-08	-1.79E-08	-1.73E-08	-1.84E-08	-1.89E-08	-1.86E-08	-1.82E-08	-1.77E-08	-1.75E-08	-1.84E-08	-1.94E-08	-1.92E-08	-1.92E-08
-1.80E-08	-1.80E-08	-1.72E-08	-1.64E-08	-1.75E-08	-1.79E-08	-1.78E-08	-1.73E-08	-1.74E-08	-1.72E-08	-1.81E-08	-1.89E-08	-1.84E-08	-1.84E-08
-1.81E-08	-1.77E-08	-1.70E-08	-1.58E-08	-1.68E-08	-1.73E-08	-1.67E-08	-1.66E-08	-1.69E-08	-1.66E-08	-1.71E-08	-1.76E-08	-1.80E-08	-1.77E-08
-1.76E-08	-1.69E-08	-1.66E-08	-1.53E-08	-1.58E-08	-1.62E-08	-1.56E-08	-1.56E-08	-1.63E-08	-1.58E-08	-1.59E-08	-1.63E-08	-1.73E-08	-1.67E-08
-1.70E-08	-1.60E-08	-1.62E-08	-1.57E-08	-1.50E-08	-1.53E-08	-1.49E-08	-1.47E-08	-1.53E-08	-1.51E-08	-1.50E-08	-1.53E-08	-1.63E-08	-1.58E-08
-1.61E-08	-1.53E-08	-1.57E-08	-1.58E-08	-1.44E-08	-1.50E-08	-1.44E-08	-1.42E-08	-1.47E-08	-1.41E-08	-1.42E-08	-1.45E-08	-1.56E-08	-1.49E-08
-1.55E-08	-1.48E-08	-1.49E-08	-1.52E-08	-1.40E-08	-1.47E-08	-1.39E-08	-1.36E-08	-1.37E-08	-1.29E-08	-1.39E-08	-1.42E-08	-1.51E-08	-1.39E-08
-1.49E-08	-1.44E-08	-1.46E-08	-1.42E-08	-1.35E-08	-1.41E-08	-1.31E-08	-1.29E-08	-1.27E-08	-1.17E-08	-1.34E-08	-1.40E-08	-1.43E-08	-1.31E-08
-1.46E-08	-1.44E-08	-1.41E-08	-1.35E-08	-1.30E-08	-1.31E-08	-1.25E-08	-1.19E-08	-1.23E-08	-1.17E-08	-1.29E-08	-1.37E-08	-1.36E-08	-1.21E-08
-1.44E-08	-1.39E-08	-1.33E-08	-1.28E-08	-1.24E-08	-1.28E-08	-1.23E-08	-1.16E-08	-1.17E-08	-1.17E-08	-1.23E-08	-1.31E-08	-1.26E-08	-1.14E-08
-1.39E-08	-1.36E-08	-1.27E-08	-1.24E-08	-1.22E-08	-1.28E-08	-1.16E-08	-1.11E-08	-1.13E-08	-1.13E-08	-1.19E-08	-1.25E-08	-1.20E-08	-1.04E-08
-1.32E-08	-1.28E-08	-1.22E-08	-1.22E-08	-1.25E-08	-1.23E-08	-1.11E-08	-1.07E-08	-1.08E-08	-1.11E-08	-1.15E-08	-1.19E-08	-1.17E-08	-1.01E-08
-1.24E-08	-1.24E-08	-1.15E-08	-1.24E-08	-1.23E-08	-1.20E-08	-1.06E-08	-1.10E-08	-1.09E-08	-1.09E-08	-1.12E-08	-1.12E-08	-1.16E-08	-9.82E-09
-1.16E-08	-1.21E-08	-1.08E-08	-1.19E-08	-1.16E-08	-1.16E-08	-1.05E-08	-1.14E-08	-1.12E-08	-1.03E-08	-1.07E-08	-1.04E-08	-1.13E-08	-9.45E-09
-1.13E-08	-1.12E-08	-1.03E-08	-1.14E-08	-1.10E-08	-1.14E-08	-1.12E-08	-1.15E-08	-1.14E-08	-1.06E-08	-1.08E-08	-1.02E-08	-1.06E-08	-9.82E-09
-1.07E-08	-1.07E-08	-1.07E-08	-1.11E-08	-1.08E-08	-1.19E-08	-1.17E-08	-1.18E-08	-1.10E-08	-1.04E-08	-1.09E-08	-9.68E-09	-9.79E-09	-8.83E-09
-1.02E-08	-1.06E-08	-1.07E-08	-1.09E-08	-1.04E-08	-1.15E-08	-1.13E-08	-1.11E-08	-1.04E-08	-1.01E-08	-1.05E-08	-9.10E-09	-9.00E-09	-8.35E-09
-9.85E-09	-1.05E-08	-1.08E-08	-1.06E-08	-1.03E-08	-1.07E-08	-1.07E-08	-1.05E-08	-9.97E-09	-9.55E-09	-9.78E-09	-8.60E-09	-8.61E-09	-8.51E-09
-8.74E-09	-1.01E-08	-1.03E-08	-1.01E-08	-1.05E-08	-1.02E-08	-1.03E-08	-1.03E-08	-9.31E-09	-9.37E-09	-8.61E-09	-8.72E-09	-8.25E-09	-8.62E-09
-7.42E-09	-9.13E-09	-9.29E-09	-9.78E-09	-1.04E-08	-9.26E-09	-9.23E-09	-9.71E-09	-8.64E-09	-9.04E-09	-7.93E-09	-8.04E-09	-7.47E-09	-8.17E-09
-6.54E-09	-8.23E-09	-8.05E-09	-8.97E-09	-9.89E-09	-8.31E-09	-8.31E-09	-8.82E-09	-8.39E-09	-8.92E-09	-7.83E-09	-7.25E-09	-7.23E-09	-7.66E-09
-6.31E-09	-6.83E-09	-7.05E-09	-7.89E-09	-9.11E-09	-8.06E-09	-8.33E-09	-8.67E-09	-8.15E-09	-8.21E-09	-7.14E-09	-6.96E-09	-7.39E-09	-7.17E-09
-5.79E-09	-6.24E-09	-6.02E-09	-7.35E-09	-7.92E-09	-7.69E-09	-7.75E-09	-8.19E-09	-7.67E-09	-7.22E-09	-6.38E-09	-7.14E-09	-6.80E-09	-6.28E-09
-5.00E-09	-5.59E-09	-5.50E-09	-7.06E-09	-6.87E-09	-6.87E-09	-6.92E-09	-7.58E-09	-6.73E-09	-5.83E-09	-5.74E-09	-6.41E-09	-5.90E-09	-5.52E-09
-4.59E-09	-4.79E-09	-4.62E-09	-6.12E-09	-5.64E-09	-5.88E-09	-5.98E-09	-6.75E-09	-5.90E-09	-5.12E-09	-5.07E-09	-5.83E-09	-5.10E-09	-4.45E-09
-3.90E-09	-4.31E-09	-4.38E-09	-5.26E-09	-4.93E-09	-5.25E-09	-5.12E-09	-5.56E-09	-4.86E-09	-4.81E-09	-3.71E-09	-4.69E-09	-4.56E-09	-3.42E-09
-3.09E-09	-3.82E-09	-3.80E-09	-4.65E-09	-4.73E-09	-4.57E-09	-4.46E-09	-4.40E-09	-4.12E-09	-3.72E-09	-2.57E-09	-3.74E-09	-4.03E-09	-3.23E-09
-2.69E-09	-2.93E-09	-3.17E-09	-3.63E-09	-3.82E-09	-3.56E-09	-3.90E-09	-3.33E-09	-3.03E-09	-2.45E-09	-1.97E-09	-2.92E-09	-3.29E-09	-2.73E-09
-1.97E-09	-2.55E-09	-2.66E-09	-2.77E-09	-2.47E-09	-2.58E-09	-2.87E-09	-2.57E-09	-1.98E-09	-1.90E-09	-2.19E-09	-2.14E-09	-2.18E-09	-1.76E-09
-1.22E-09	-1.65E-09	-1.89E-09	-1.61E-09	-1.11E-09	-2.05E-09	-1.73E-09	-1.91E-09	-9.98E-10	-1.71E-09	-2.17E-09	-1.59E-09	-1.26E-09	-1.57E-09
-7.72E-10	-7.46E-10	-9.88E-10	-5.98E-10	9.69E-11	-1.24E-09	-9.14E-10	-1.38E-09	-6.29E-10	-1.02E-09	-1.07E-09	-1.26E-09	-3.79E-10	-1.19E-09
-2.44E-10	-1.94E-10	-3.72E-10	2.64E-10	9.01E-10	-4.75E-11	-5.29E-10	-8.33E-10	-2.17E-10	2.36E-11	8.30E-11	-6.55E-10	9.16E-11	-3.88E-10
4.83E-10	2.57E-10	-2.07E-10	5.01E-10	1.68E-09	9.88E-10	3.87E-11	1.57E-10	4.14E-10	8.59E-10	3.81E-10	4.41E-10	7.31E-10	5.17E-10
8.71E-10	5.58E-10	3.67E-10	1.39E-09	2.34E-09	1.63E-09	9.31E-10	8.67E-10	6.21E-10	1.07E-09	7.59E-10	1.48E-09	1.23E-09	1.00E-09
1.53E-09	1.07E-09	1.50E-09	2.21E-09	2.41E-09	1.98E-09	1.71E-09	1.69E-09	1.29E-09	1.98E-09	1.18E-09	2.29E-09	1.75E-09	1.71E-09
2.77E-09	2.26E-09	1.91E-09	2.46E-09	2.71E-09	2.68E-09	2.03E-09	2.20E-09	1.75E-09	3.42E-09	1.83E-09	3.06E-09	2.36E-09	2.28E-09
4.09E-09	3.37E-09	2.52E-09	2.62E-09	3.04E-09	3.09E-09	2.32E-09	2.61E-09	2.57E-09	3.41E-09	2.75E-09	3.79E-09	2.90E-09	2.77E-09
4.84E-09	4.23E-09	3.55E-09	3.52E-09	3.45E-09	3.99E-09	3.30E-09	3.62E-09	3.03E-09	3.73E-09	3.64E-09	4.37E-09	3.96E-09	3.42E-09
4.77E-09	4.71E-09	4.72E-09	4.97E-09	4.30E-09	4.76E-09	4.28E-09	4.52E-09	3.84E-09	4.48E-09	4.40E-09	4.79E-09	4.55E-09	4.41E-09
4.81E-09	5.16E-09	5.57E-09	5.68E-09	4.85E-09	5.61E-09	4.95E-09	5.37E-09	4.73E-09	5.52E-09	5.14E-09	4.75E-09	4.80E-09	5.57E-09
5.38E-09	5.92E-09	5.87E-09	5.91E-09	5.28E-09	6.48E-09	5.14E-09	6.45E-09	5.67E-09	6.10E-09	5.87E-09	5.22E-09	5.36E-09	5.57E-09

5.62E-09	6.35E-09	6.38E-09	6.55E-09	5.87E-09	6.78E-09	5.80E-09	7.39E-09	6.19E-09	6.27E-09	6.64E-09	5.84E-09	5.19E-09	5.35E-09
6.10E-09	6.87E-09	6.89E-09	7.59E-09	6.62E-09	6.62E-09	6.70E-09	6.91E-09	6.83E-09	6.28E-09	7.35E-09	6.60E-09	5.44E-09	5.34E-09
6.69E-09	7.76E-09	7.35E-09	7.97E-09	7.46E-09	7.17E-09	7.31E-09	7.76E-09	7.63E-09	6.80E-09	8.23E-09	7.37E-09	6.27E-09	6.07E-09
7.79E-09	8.26E-09	7.85E-09	8.56E-09	8.61E-09	7.81E-09	8.04E-09	8.47E-09	7.97E-09	7.20E-09	8.78E-09	7.92E-09	7.47E-09	7.55E-09
9.02E-09	9.16E-09	8.62E-09	9.41E-09	9.86E-09	8.62E-09	8.98E-09	8.91E-09	8.48E-09	7.85E-09	8.71E-09	8.28E-09	8.64E-09	8.70E-09
9.74E-09	9.96E-09	9.44E-09	1.01E-08	1.02E-08	9.18E-09	9.42E-09	9.30E-09	9.16E-09	8.53E-09	9.40E-09	8.60E-09	8.91E-09	9.01E-09
9.84E-09	1.06E-08	1.01E-08	1.05E-08	9.92E-09	9.52E-09	9.89E-09	9.78E-09	9.58E-09	9.48E-09	9.77E-09	8.71E-09	8.73E-09	9.08E-09
1.07E-08	1.12E-08	1.10E-08	1.12E-08	1.07E-08	1.04E-08	1.08E-08	1.05E-08	1.07E-08	1.05E-08	1.03E-08	9.55E-09	9.66E-09	9.67E-09
1.19E-08	1.22E-08	1.18E-08	1.21E-08	1.16E-08	1.16E-08	1.21E-08	1.14E-08	1.19E-08	1.11E-08	1.11E-08	1.08E-08	1.07E-08	1.07E-08
1.34E-08	1.35E-08	1.28E-08	1.32E-08	1.27E-08	1.24E-08	1.34E-08	1.23E-08	1.29E-08	1.22E-08	1.21E-08	1.26E-08	1.20E-08	1.12E-08
1.45E-08	1.46E-08	1.37E-08	1.37E-08	1.36E-08	1.29E-08	1.39E-08	1.29E-08	1.34E-08	1.32E-08	1.31E-08	1.40E-08	1.32E-08	1.16E-08
1.53E-08	1.52E-08	1.41E-08	1.45E-08	1.45E-08	1.38E-08	1.42E-08	1.39E-08	1.40E-08	1.38E-08	1.37E-08	1.46E-08	1.38E-08	1.24E-08
1.57E-08	1.61E-08	1.47E-08	1.53E-08	1.54E-08	1.47E-08	1.52E-08	1.48E-08	1.47E-08	1.46E-08	1.47E-08	1.48E-08	1.41E-08	1.33E-08
1.62E-08	1.65E-08	1.58E-08	1.62E-08	1.70E-08	1.60E-08	1.65E-08	1.58E-08	1.55E-08	1.54E-08	1.60E-08	1.57E-08	1.49E-08	1.43E-08
1.69E-08	1.73E-08	1.73E-08	1.71E-08	1.81E-08	1.69E-08	1.76E-08	1.66E-08	1.64E-08	1.62E-08	1.67E-08	1.65E-08	1.57E-08	1.54E-08
1.76E-08	1.84E-08	1.83E-08	1.77E-08	1.87E-08	1.78E-08	1.80E-08	1.76E-08	1.71E-08	1.72E-08	1.79E-08	1.78E-08	1.66E-08	1.63E-08
1.87E-08	1.95E-08	1.97E-08	1.86E-08	1.92E-08	1.88E-08	1.88E-08	1.84E-08	1.81E-08	1.79E-08	1.92E-08	1.91E-08	1.77E-08	1.70E-08
1.99E-08	2.02E-08	2.09E-08	2.04E-08	2.04E-08	1.97E-08	1.96E-08	1.92E-08	1.89E-08	1.87E-08	2.00E-08	2.02E-08	1.86E-08	1.75E-08
2.12E-08	2.11E-08	2.23E-08	2.19E-08	2.13E-08	2.03E-08	2.01E-08	1.99E-08	2.00E-08	1.93E-08	2.02E-08	2.10E-08	1.97E-08	1.84E-08
2.20E-08	2.18E-08	2.25E-08	2.19E-08	2.13E-08	2.10E-08	2.14E-08	2.10E-08	2.10E-08	2.02E-08	2.04E-08	2.13E-08	2.09E-08	1.91E-08
2.26E-08	2.24E-08	2.23E-08	2.16E-08	2.19E-08	2.22E-08	2.25E-08	2.23E-08	2.22E-08	2.15E-08	2.11E-08	2.15E-08	2.19E-08	1.99E-08
2.36E-08	2.30E-08	2.31E-08	2.23E-08	2.26E-08	2.32E-08	2.31E-08	2.34E-08	2.29E-08	2.31E-08	2.22E-08	2.22E-08	2.25E-08	2.10E-08
2.44E-08	2.38E-08	2.40E-08	2.30E-08	2.37E-08	2.40E-08	2.35E-08	2.40E-08	2.34E-08	2.46E-08	2.35E-08	2.26E-08	2.26E-08	2.22E-08
2.57E-08	2.53E-08	2.53E-08	2.40E-08	2.46E-08	2.50E-08	2.46E-08	2.51E-08	2.43E-08	2.58E-08	2.51E-08	2.36E-08	2.37E-08	2.32E-08
2.71E-08	2.68E-08	2.66E-08	2.56E-08	2.60E-08	2.62E-08	2.61E-08	2.63E-08	2.54E-08	2.64E-08	2.60E-08	2.48E-08	2.48E-08	2.43E-08
2.85E-08	2.71E-08	2.72E-08	2.74E-08	2.73E-08	2.74E-08	2.73E-08	2.71E-08	2.63E-08	2.69E-08	2.65E-08	2.56E-08	2.62E-08	2.57E-08
2.92E-08	2.80E-08	2.85E-08	2.84E-08	2.84E-08	2.85E-08	2.74E-08	2.79E-08	2.71E-08	2.75E-08	2.72E-08	2.72E-08	2.70E-08	2.74E-08
3.02E-08	2.95E-08	2.97E-08	2.94E-08	2.95E-08	2.90E-08	2.84E-08	2.91E-08	2.81E-08	2.84E-08	2.80E-08	2.88E-08	2.85E-08	2.87E-08
3.17E-08	3.15E-08	3.12E-08	3.04E-08	3.07E-08	3.03E-08	2.96E-08	3.07E-08	2.96E-08	2.94E-08	2.94E-08	3.02E-08	2.97E-08	2.92E-08
3.31E-08	3.36E-08	3.30E-08	3.14E-08	3.16E-08	3.16E-08	3.11E-08	3.24E-08	3.13E-08	3.07E-08	3.11E-08	3.11E-08	3.06E-08	3.05E-08
3.44E-08	3.50E-08	3.43E-08	3.26E-08	3.22E-08	3.27E-08	3.26E-08	3.37E-08	3.27E-08	3.19E-08	3.26E-08	3.17E-08	3.13E-08	3.16E-08
3.57E-08	3.62E-08	3.51E-08	3.41E-08	3.30E-08	3.38E-08	3.41E-08	3.44E-08	3.39E-08	3.30E-08	3.35E-08	3.25E-08	3.25E-08	3.24E-08
3.69E-08	3.68E-08	3.59E-08	3.56E-08	3.46E-08	3.52E-08	3.57E-08	3.55E-08	3.45E-08	3.43E-08	3.46E-08	3.37E-08	3.38E-08	3.32E-08
3.76E-08	3.70E-08	3.68E-08	3.66E-08	3.63E-08	3.64E-08	3.66E-08	3.64E-08	3.58E-08	3.56E-08	3.56E-08	3.52E-08	3.49E-08	3.45E-08
3.87E-08	3.78E-08	3.82E-08	3.77E-08	3.73E-08	3.79E-08	3.75E-08	3.71E-08	3.74E-08	3.68E-08	3.64E-08	3.66E-08	3.67E-08	3.62E-08
4.06E-08	3.91E-08	3.95E-08	3.84E-08	3.82E-08	3.93E-08	3.85E-08	3.78E-08	3.89E-08	3.84E-08	3.76E-08	3.81E-08	3.84E-08	3.79E-08
4.21E-08	4.07E-08	4.03E-08	3.99E-08	3.92E-08	4.00E-08	3.96E-08	3.90E-08	4.03E-08	4.01E-08	3.91E-08	3.97E-08	3.95E-08	3.92E-08
4.22E-08	4.14E-08	4.13E-08	4.11E-08	4.02E-08	4.11E-08	4.06E-08	4.02E-08	4.16E-08	4.10E-08	4.03E-08	4.10E-08	4.03E-08	4.01E-08
4.23E-08	4.22E-08	4.24E-08	4.20E-08	4.16E-08	4.25E-08	4.19E-08	4.17E-08	4.23E-08	4.15E-08	4.17E-08	4.22E-08	4.15E-08	4.12E-08
4.33E-08	4.36E-08	4.35E-08	4.32E-08	4.27E-08	4.36E-08	4.30E-08	4.32E-08	4.33E-08	4.20E-08	4.27E-08	4.34E-08	4.28E-08	4.22E-08
4.45E-08	4.46E-08	4.47E-08	4.42E-08	4.38E-08	4.43E-08	4.45E-08	4.49E-08	4.43E-08	4.32E-08	4.40E-08	4.42E-08	4.36E-08	4.28E-08
4.52E-08	4.52E-08	4.55E-08	4.55E-08	4.51E-08	4.56E-08	4.61E-08	4.61E-08	4.54E-08	4.45E-08	4.49E-08	4.53E-08	4.43E-08	4.39E-08
4.63E-08	4.63E-08	4.67E-08	4.69E-08	4.67E-08	4.68E-08	4.75E-08	4.73E-08	4.62E-08	4.55E-08	4.57E-08	4.63E-08	4.55E-08	4.55E-08
4.80E-08	4.78E-08	4.81E-08	4.83E-08	4.77E-08	4.79E-08	4.83E-08	4.79E-08	4.74E-08	4.69E-08	4.66E-08	4.70E-08	4.75E-08	4.74E-08
4.94E-08	4.92E-08	4.91E-08	4.89E-08	4.92E-08	4.92E-08	4.87E-08	4.88E-08	4.85E-08	4.83E-08	4.79E-08	4.82E-08	4.95E-08	4.90E-08
5.08E-08	5.05E-08	5.03E-08	5.02E-08	5.08E-08	5.06E-08	4.99E-08	5.01E-08	4.97E-08	4.95E-08	4.89E-08	4.95E-08	5.09E-08	5.04E-08
5.19E-08	5.14E-08	5.15E-08	5.18E-08	5.23E-08	5.18E-08	5.14E-08	5.13E-08	5.12E-08	5.11E-08	5.05E-08	5.03E-08	5.10E-08	5.12E-08
5.31E-08	5.29E-08	5.27E-08	5.35E-08	5.37E-08	5.32E-08	5.27E-08	5.30E-08	5.29E-08	5.26E-08	5.24E-08	5.18E-08	5.18E-08	5.23E-08
5.40E-08	5.45E-08	5.41E-08	5.53E-08	5.52E-08	5.46E-08	5.43E-08	5.44E-08	5.49E-08	5.42E-08	5.44E-08	5.32E-08	5.28E-08	5.36E-08
5.55E-08	5.60E-08	5.60E-08	5.70E-08	5.69E-08	5.61E-08	5.61E-08	5.54E-08	5.58E-08	5.61E-08	5.57E-08	5.47E-08	5.42E-08	5.49E-08

384 values):

1.30E-15	1.29E-15	1.26E-15	1.23E-15	1.28E-15	1.27E-15	1.24E-15	1.24E-15	1.24E-15	1.21E-15	1.21E-15	1.13E-15	1.11E-15	1.13E-15
1.28E-15	1.31E-15	1.28E-15	1.21E-15	1.28E-15	1.28E-15	1.24E-15	1.23E-15	1.25E-15	1.20E-15	1.20E-15	1.16E-15	1.10E-15	1.15E-15
1.20E-15	1.26E-15	1.27E-15	1.21E-15	1.26E-15	1.25E-15	1.20E-15	1.19E-15	1.24E-15	1.20E-15	1.20E-15	1.16E-15	1.07E-15	1.12E-15
1.15E-15	1.19E-15	1.22E-15	1.24E-15	1.23E-15	1.21E-15	1.13E-15	1.13E-15	1.20E-15	1.17E-15	1.19E-15	1.16E-15	1.05E-15	1.07E-15
1.11E-15	1.14E-15	1.20E-15	1.23E-15	1.18E-15	1.14E-15	1.09E-15	1.10E-15	1.16E-15	1.15E-15	1.17E-15	1.13E-15	1.01E-15	1.01E-15
1.11E-15	1.11E-15	1.18E-15	1.16E-15	1.11E-15	1.11E-15	1.12E-15	1.09E-15	1.15E-15	1.15E-15	1.13E-15	1.10E-15	9.86E-16	9.76E-16
1.13E-15	1.09E-15	1.13E-15	1.13E-15	1.08E-15	1.08E-15	1.16E-15	1.11E-15	1.16E-15	1.14E-15	1.13E-15	1.09E-15	1.01E-15	9.72E-16
1.17E-15	1.08E-15	1.09E-15	1.09E-15	1.06E-15	1.02E-15	1.13E-15	1.14E-15	1.15E-15	1.07E-15	1.10E-15	1.07E-15	1.03E-15	9.93E-16
1.16E-15	1.06E-15	1.07E-15	1.07E-15	1.08E-15	9.91E-16	1.11E-15	1.11E-15	1.11E-15	9.99E-16	1.04E-15	1.04E-15	1.01E-15	9.62E-16
1.14E-15	1.06E-15	1.05E-15	1.08E-15	1.08E-15	1.03E-15	1.08E-15	1.07E-15	1.10E-15	1.01E-15	1.02E-15	1.02E-15	9.97E-16	9.20E-16
1.13E-15	1.05E-15	1.06E-15	1.07E-15	1.08E-15	1.04E-15	1.07E-15	1.05E-15	1.08E-15	9.91E-16	1.01E-15	1.01E-15	9.65E-16	9.09E-16
1.11E-15	1.03E-15	1.07E-15	1.06E-15	1.07E-15	1.03E-15	1.04E-15	1.03E-15	1.05E-15	9.71E-16	9.86E-16	9.79E-16	9.47E-16	9.15E-16
1.08E-15	9.93E-16	1.02E-15	1.02E-15	1.05E-15	9.60E-16	9.98E-16	9.79E-16	1.01E-15	9.43E-16	9.64E-16	9.40E-16	9.34E-16	9.14E-16
1.04E-15	9.63E-16	9.65E-16	9.56E-16	9.94E-16	8.97E-16	9.49E-16	9.46E-16	9.70E-16	9.26E-16	9.61E-16	9.10E-16	9.20E-16	8.91E-16
1.02E-15	9.41E-16	9.33E-16	9.18E-16	9.44E-16	9.14E-16	9.25E-16	9.23E-16	9.27E-16	8.91E-16	9.42E-16	8.73E-16	9.02E-16	8.83E-16
1.02E-15	9.44E-16	9.40E-16	9.42E-16	9.20E-16	9.10E-16	9.03E-16	8.77E-16	8.92E-16	8.49E-16	9.26E-16	8.26E-16	8.98E-16	8.75E-16
9.91E-16	9.42E-16	9.44E-16	9.29E-16	8.99E-16	8.83E-16	8.62E-16	8.55E-16	8.55E-16	8.34E-16	8.84E-16	7.86E-16	8.67E-16	8.79E-16
9.25E-16	9.10E-16	9.19E-16	9.15E-16	8.60E-16	8.57E-16	8.43E-16	8.63E-16	8.42E-16	8.36E-16	8.37E-16	7.62E-16	8.47E-16	8.73E-16
8.77E-16	8.64E-16	8.85E-16	8.93E-16	8.39E-16	8.45E-16	8.26E-16	8.34E-16	8.29E-16	8.35E-16	8.26E-16	7.56E-16	8.10E-16	8.50E-16
8.30E-16	8.38E-16	8.62E-16	8.69E-16	8.27E-16	8.19E-16	7.76E-16	7.82E-16	8.14E-16	8.14E-16	8.06E-16	7.55E-16	7.68E-16	8.12E-16
7.81E-16	8.10E-16	8.46E-16	8.39E-16	8.09E-16	7.92E-16	7.46E-16	7.49E-16	7.78E-16	7.69E-16	7.83E-16	7.66E-16	7.66E-16	7.60E-16
7.34E-16	7.62E-16	8.09E-16	7.98E-16	7.77E-16	7.67E-16	7.33E-16	7.36E-16	7.55E-16	7.43E-16	7.52E-16	7.61E-16	7.60E-16	7.04E-16
7.10E-16	7.34E-16	7.80E-16	7.62E-16	7.59E-16	7.22E-16	6.91E-16	7.04E-16	7.37E-16	7.04E-16	7.12E-16	7.40E-16	7.12E-16	6.34E-16
7.02E-16	7.22E-16	7.61E-16	7.57E-16	7.40E-16	6.90E-16	6.74E-16	6.85E-16	7.07E-16	6.67E-16	6.74E-16	6.88E-16	6.40E-16	5.91E-16
6.87E-16	7.02E-16	7.19E-16	7.27E-16	6.90E-16	6.49E-16	6.66E-16	6.72E-16	6.57E-16	6.55E-16	6.73E-16	6.62E-16	5.71E-16	5.73E-16
6.54E-16	6.68E-16	6.96E-16	6.69E-16	6.50E-16	6.20E-16	6.55E-16	6.49E-16	6.22E-16	6.58E-16	6.69E-16	6.73E-16	5.59E-16	5.75E-16
6.36E-16	6.42E-16	6.61E-16	6.15E-16	6.17E-16	6.23E-16	6.46E-16	6.27E-16	5.93E-16	6.37E-16	6.62E-16	6.75E-16	5.92E-16	5.62E-16
6.49E-16	6.05E-16	6.23E-16	5.88E-16	5.76E-16	6.05E-16	6.09E-16	5.99E-16	5.71E-16	6.05E-16	6.32E-16	6.52E-16	6.04E-16	5.77E-16
6.12E-16	5.81E-16	5.73E-16	5.46E-16	5.32E-16	5.63E-16	5.60E-16	5.83E-16	5.72E-16	5.81E-16	6.06E-16	6.12E-16	5.85E-16	5.71E-16
5.73E-16	5.49E-16	5.20E-16	4.76E-16	4.65E-16	5.10E-16	5.36E-16	5.63E-16	5.44E-16	5.53E-16	5.83E-16	5.76E-16	5.58E-16	5.48E-16
5.60E-16	5.15E-16	4.72E-16	4.01E-16	4.04E-16	4.52E-16	5.14E-16	5.29E-16	5.20E-16	5.53E-16	5.62E-16	5.69E-16	5.24E-16	5.15E-16

5.32E-16	4.83E-16	4.26E-16	3.47E-16	3.51E-16	4.07E-16	4.74E-16	4.99E-16	4.95E-16	5.29E-16	5.23E-16	5.63E-16	5.00E-16	4.91E-16
5.09E-16	4.54E-16	3.94E-16	3.20E-16	3.34E-16	3.89E-16	4.53E-16	4.66E-16	4.74E-16	4.89E-16	4.84E-16	5.24E-16	4.77E-16	4.67E-16
4.92E-16	4.26E-16	3.86E-16	3.36E-16	3.60E-16	3.82E-16	4.46E-16	4.39E-16	4.59E-16	4.79E-16	4.61E-16	4.92E-16	4.73E-16	4.34E-16
4.52E-16	4.21E-16	3.89E-16	3.59E-16	3.74E-16	3.87E-16	4.30E-16	4.15E-16	4.24E-16	4.37E-16	4.38E-16	4.56E-16	4.49E-16	4.26E-16
3.98E-16	4.03E-16	3.83E-16	3.53E-16	3.76E-16	3.85E-16	4.01E-16	3.98E-16	3.71E-16	3.84E-16	4.01E-16	4.21E-16	4.30E-16	4.27E-16
3.38E-16	3.62E-16	3.63E-16	3.25E-16	3.60E-16	3.93E-16	3.77E-16	3.66E-16	3.32E-16	3.25E-16	3.55E-16	3.89E-16	3.99E-16	3.98E-16
3.17E-16	3.42E-16	3.21E-16	2.98E-16	3.39E-16	3.56E-16	3.46E-16	3.30E-16	3.15E-16	3.05E-16	3.39E-16	3.76E-16	3.70E-16	3.70E-16
3.24E-16	3.23E-16	2.95E-16	2.68E-16	3.05E-16	3.19E-16	3.17E-16	2.99E-16	3.04E-16	2.96E-16	3.26E-16	3.56E-16	3.39E-16	3.37E-16
3.29E-16	3.13E-16	2.89E-16	2.48E-16	2.83E-16	2.98E-16	2.78E-16	2.75E-16	2.86E-16	2.75E-16	2.93E-16	3.10E-16	3.23E-16	3.12E-16
3.10E-16	2.85E-16	2.75E-16	2.35E-16	2.50E-16	2.62E-16	2.43E-16	2.44E-16	2.67E-16	2.50E-16	2.53E-16	2.67E-16	2.99E-16	2.81E-16
2.90E-16	2.57E-16	2.63E-16	2.48E-16	2.25E-16	2.36E-16	2.22E-16	2.17E-16	2.34E-16	2.28E-16	2.25E-16	2.33E-16	2.65E-16	2.51E-16
2.60E-16	2.34E-16	2.47E-16	2.48E-16	2.08E-16	2.24E-16	2.06E-16	2.01E-16	2.17E-16	2.00E-16	2.02E-16	2.10E-16	2.44E-16	2.21E-16
2.40E-16	2.19E-16	2.23E-16	2.31E-16	1.95E-16	2.15E-16	1.93E-16	1.85E-16	1.88E-16	1.67E-16	1.92E-16	2.00E-16	2.27E-16	1.92E-16
2.23E-16	2.09E-16	2.14E-16	2.03E-16	1.82E-16	2.00E-16	1.71E-16	1.65E-16	1.61E-16	1.37E-16	1.80E-16	1.97E-16	2.05E-16	1.71E-16
2.14E-16	2.07E-16	1.98E-16	1.83E-16	1.70E-16	1.72E-16	1.57E-16	1.41E-16	1.51E-16	1.37E-16	1.67E-16	1.88E-16	1.84E-16	1.47E-16
2.07E-16	1.94E-16	1.76E-16	1.65E-16	1.54E-16	1.65E-16	1.50E-16	1.35E-16	1.38E-16	1.36E-16	1.52E-16	1.70E-16	1.58E-16	1.30E-16
1.94E-16	1.84E-16	1.62E-16	1.55E-16	1.48E-16	1.64E-16	1.35E-16	1.22E-16	1.27E-16	1.27E-16	1.42E-16	1.57E-16	1.44E-16	1.08E-16
1.75E-16	1.64E-16	1.50E-16	1.49E-16	1.56E-16	1.52E-16	1.23E-16	1.15E-16	1.16E-16	1.24E-16	1.32E-16	1.41E-16	1.38E-16	1.01E-16
1.53E-16	1.53E-16	1.33E-16	1.53E-16	1.51E-16	1.45E-16	1.13E-16	1.22E-16	1.20E-16	1.18E-16	1.24E-16	1.25E-16	1.34E-16	9.65E-17
1.36E-16	1.47E-16	1.16E-16	1.41E-16	1.34E-16	1.35E-16	1.11E-16	1.29E-16	1.26E-16	1.07E-16	1.15E-16	1.09E-16	1.27E-16	8.93E-17
1.28E-16	1.26E-16	1.07E-16	1.31E-16	1.22E-16	1.30E-16	1.25E-16	1.33E-16	1.30E-16	1.12E-16	1.16E-16	1.04E-16	1.12E-16	9.64E-17
1.14E-16	1.14E-16	1.14E-16	1.24E-16	1.16E-16	1.41E-16	1.36E-16	1.39E-16	1.22E-16	1.08E-16	1.19E-16	9.37E-17	9.58E-17	7.79E-17
1.03E-16	1.11E-16	1.15E-16	1.20E-16	1.08E-16	1.33E-16	1.27E-16	1.24E-16	1.09E-16	1.02E-16	1.10E-16	8.28E-17	8.10E-17	6.96E-17
9.69E-17	1.11E-16	1.16E-16	1.11E-16	1.06E-16	1.14E-16	1.15E-16	1.11E-16	9.94E-17	9.13E-17	9.56E-17	7.39E-17	7.41E-17	7.24E-17
7.64E-17	1.03E-16	1.06E-16	1.03E-16	1.11E-16	1.04E-16	1.05E-16	1.06E-16	8.68E-17	8.78E-17	7.42E-17	7.60E-17	6.81E-17	7.42E-17
5.50E-17	8.33E-17	8.64E-17	9.57E-17	1.09E-16	8.57E-17	8.51E-17	9.42E-17	7.47E-17	8.16E-17	6.28E-17	6.47E-17	5.58E-17	6.67E-17
4.27E-17	6.77E-17	6.48E-17	8.05E-17	9.77E-17	6.90E-17	6.90E-17	7.78E-17	7.05E-17	7.96E-17	6.12E-17	5.25E-17	5.22E-17	5.87E-17
3.98E-17	4.67E-17	4.97E-17	6.23E-17	8.30E-17	6.50E-17	6.93E-17	7.51E-17	6.64E-17	6.73E-17	5.10E-17	4.85E-17	5.46E-17	5.14E-17
3.35E-17	3.90E-17	3.62E-17	5.40E-17	6.27E-17	5.92E-17	6.01E-17	6.70E-17	5.88E-17	5.22E-17	4.08E-17	5.10E-17	4.62E-17	3.94E-17
2.50E-17	3.13E-17	3.02E-17	4.98E-17	4.72E-17	4.72E-17	4.79E-17	5.75E-17	4.53E-17	3.40E-17	3.30E-17	4.11E-17	3.48E-17	3.05E-17
2.10E-17	2.30E-17	2.13E-17	3.75E-17	3.18E-17	3.45E-17	3.58E-17	4.56E-17	3.48E-17	2.62E-17	2.57E-17	3.40E-17	2.60E-17	1.98E-17
1.52E-17	1.86E-17	1.92E-17	2.77E-17	2.43E-17	2.75E-17	2.62E-17	3.09E-17	2.36E-17	2.31E-17	1.38E-17	2.20E-17	2.08E-17	1.17E-17
9.58E-18	1.46E-17	1.44E-17	2.16E-17	2.24E-17	2.08E-17	1.99E-17	1.94E-17	1.70E-17	1.38E-17	6.63E-18	1.40E-17	1.63E-17	1.04E-17
7.25E-18	8.58E-18	1.01E-17	1.32E-17	1.46E-17	1.27E-17	1.52E-17	1.11E-17	9.20E-18	5.98E-18	3.87E-18	8.54E-18	1.08E-17	7.44E-18
3.89E-18	6.51E-18	7.08E-18	7.70E-18	6.11E-18	6.67E-18	8.22E-18	6.60E-18	3.92E-18	3.63E-18	4.79E-18	4.58E-18	4.77E-18	3.11E-18
1.48E-18	2.73E-18	3.56E-18	2.60E-18	1.23E-18	4.18E-18	2.98E-18	3.65E-18	9.96E-19	2.93E-18	4.71E-18	2.52E-18	1.59E-18	2.45E-18
5.96E-19	5.57E-19	9.77E-19	3.58E-19	9.38E-21	1.53E-18	8.36E-19	1.92E-18	3.95E-19	1.05E-18	1.14E-18	1.58E-18	1.44E-19	1.41E-18
5.96E-20	3.76E-20	1.38E-19	6.96E-20	8.11E-19	2.25E-21	2.80E-19	6.94E-19	4.71E-20	5.59E-22	6.89E-21	4.29E-19	8.40E-21	1.51E-19
2.33E-19	6.60E-20	4.30E-20	2.51E-19	2.82E-18	9.75E-19	1.49E-21	2.46E-20	1.72E-19	7.38E-19	1.45E-19	1.94E-19	5.35E-19	2.67E-19
7.59E-19	3.11E-19	1.35E-19	1.93E-18	5.48E-18	2.65E-18	8.66E-19	7.52E-19	3.86E-19	1.15E-18	5.75E-19	2.19E-18	1.52E-18	1.01E-18
2.33E-18	1.15E-18	2.25E-18	4.86E-18	5.79E-18	3.94E-18	2.93E-18	2.87E-18	1.67E-18	3.91E-18	1.39E-18	5.27E-18	3.05E-18	2.91E-18
7.69E-18	5.10E-18	3.63E-18	6.04E-18	7.35E-18	7.18E-18	4.12E-18	4.85E-18	3.07E-18	1.17E-17	3.34E-18	9.37E-18	5.57E-18	5.19E-18
1.67E-17	1.13E-17	6.36E-18	6.85E-18	9.23E-18	9.57E-18	5.39E-18	6.81E-18	6.59E-18	1.17E-17	7.55E-18	1.44E-17	8.42E-18	7.69E-18
2.35E-17	1.79E-17	1.26E-17	1.24E-17	1.19E-17	1.60E-17	1.09E-17	1.31E-17	9.17E-18	1.39E-17	1.33E-17	1.91E-17	1.57E-17	1.17E-17
2.27E-17	2.22E-17	2.23E-17	2.47E-17	1.85E-17	2.27E-17	1.83E-17	2.05E-17	1.48E-17	2.01E-17	1.94E-17	2.29E-17	2.07E-17	1.95E-17
2.31E-17	2.66E-17	3.10E-17	3.22E-17	2.35E-17	3.15E-17	2.45E-17	2.88E-17	2.23E-17	3.04E-17	2.65E-17	2.25E-17	2.31E-17	3.10E-17
2.89E-17	3.50E-17	3.45E-17	3.50E-17	2.79E-17	4.20E-17	2.64E-17	4.16E-17	3.21E-17	3.72E-17	3.45E-17	2.73E-17	2.87E-17	3.10E-17
3.16E-17	4.03E-17	4.07E-17	4.30E-17	3.45E-17	4.60E-17	3.36E-17	5.47E-17	3.84E-17	3.93E-17	4.40E-17	3.41E-17	2.69E-17	2.86E-17
3.72E-17	4.72E-17	4.74E-17	5.77E-17	4.38E-17	4.38E-17	4.48E-17	4.78E-17	4.67E-17	3.94E-17	5.41E-17	4.36E-17	2.96E-17	2.85E-17
4.48E-17	6.02E-17	5.40E-17	6.36E-17	5.56E-17	5.14E-17	5.34E-17	6.03E-17	5.83E-17	4.63E-17	6.77E-17	5.43E-17	3.93E-17	3.68E-17
6.07E-17	6.82E-17	6.17E-17	7.32E-17	7.41E-17	6.09E-17	6.47E-17	7.17E-17	6.34E-17	5.19E-17	7.71E-17	6.27E-17	5.58E-17	5.70E-17
8.13E-17	8.39E-17	7.43E-17	8.86E-17	9.72E-17	7.44E-17	8.06E-17	7.95E-17	7.19E-17	6.16E-17	7.59E-17	6.86E-17	7.47E-17	7.58E-17
9.49E-17	9.92E-17	8.90E-17	1.02E-16	1.04E-16	8.44E-17	8.88E-17	8.65E-17	8.39E-17	7.28E-17	8.84E-17	7.40E-17	7.93E-17	8.12E-17
9.68E-17	1.12E-16	1.03E-16	1.11E-16	9.84E-17	9.06E-17	9.79E-17	9.56E-17	9.18E-17	8.98E-17	9.55E-17	7.59E-17	7.62E-17	8.25E-17
1.14E-16	1.26E-16	1.20E-16	1.26E-16	1.14E-16	1.09E-16	1.16E-16	1.10E-16	1.14E-16	1.10E-16	1.06E-16	9.13E-17	9.33E-17	9.35E-17
1.42E-16	1.50E-16	1.40E-16	1.46E-16	1.34E-16	1.35E-16	1.47E-16	1.31E-16	1.42E-16	1.24E-16	1.24E-16	1.17E-16	1.15E-16	1.15E-16
1.80E-16	1.84E-16	1.63E-16	1.74E-16	1.61E-16	1.54E-16	1.79E-16	1.52E-16	1.65E-16	1.49E-16	1.46E-16	1.59E-16	1.44E-16	1.26E-16
2.10E-16	2.12E-16	1.88E-16	1.88E-16	1.84E-16	1.66E-16	1.93E-16	1.66E-16	1.78E-16	1.74E-16	1.71E-16	1.97E-16	1.74E-16	1.35E-16
2.34E-16	2.32E-16	1.98E-16	2.10E-16	2.11E-16	1.90E-16	2.02E-16	1.92E-16	1.97E-16	1.92E-16	1.87E-16	2.14E-16	1.91E-16	1.53E-16
2.46E-16	2.59E-16	2.15E-16	2.33E-16	2.38E-16	2.17E-16	2.32E-16	2.19E-16	2.16E-16	2.12E-16	2.16E-16	2.20E-16	1.99E-16	1.76E-16
2.62E-16	2.71E-16	2.51E-16	2.63E-16	2.89E-16	2.56E-16	2.73E-16	2.49E-16	2.41E-16	2.36E-16	2.55E-16	2.47E-16	2.23E-16	2.03E-16
2.86E-16	2.99E-16	3.00E-16	2.94E-16	3.28E-16	2.84E-16	3.10E-16	2.77E-16	2.67E-16	2.64E-16	2.78E-16	2.71E-16	2.45E-16	2.37E-16
3.11E-16	3.37E-16	3.36E-16	3.12E-16	3.49E-16	3.17E-16	3.26E-16	3.11E-16	2.91E-16	2.96E-16	3.19E-16	3.17E-16	2.76E-16	2.67E-16
3.50E-16	3.79E-16	3.88E-16	3.45E-16	3.69E-16	3.54E-16	3.52E-16	3.39E-16	3.27E-16	3.22E-16	3.69E-16	3.65E-16	3.14E-16	2.89E-16
3.98E-16	4.09E-16	4.37E-16	4.15E-16	4.15E-16	3.88E-16	3.83E-16	3.67E-16	3.58E-16	3.51E-16	4.00E-16	4.08E-16	3.45E-16	3.07E-16
4.49E-16	4.44E-16	4.96E-16	4.79E-16	4.52E-16	4.13E-16	4.04E-16	3.95E-16	3.98E-16	3.73E-16	4.06E-16	4.42E-16	3.86E-16	3.39E-16
4.83E-16	4.74E-16	5.04E-16	4.81E-16	4.55E-16	4.43E-16	4.56E-16	4.42E-16	4.40E-16	4.08E-16	4.16E-16	4.53E-16	4.35E-16	3.63E-16
5.10E-16	5.03E-16	4.96E-16	4.68E-16	4.80E-16	4.93E-16	5.07E-16	4.96E-16	4.93E-16	4.61E-16	4.45E-16	4.62E-16	4.80E-16	3.97E-16
5.59E-16	5.30E-16	5.36E-16	4.99E-16	5.12E-16	5.38E-16	5.32E-16	5.48E-16	5.23E-16	5.33E-16	4.93E-16	4.92E-16	5.07E-16	4.39E-16
5.97E-16	5.65E-16	5.78E-16	5.30E-16	5.60E-16	5.77E-16	5.53E-16	5.77E-16	5.46E-16	6.05E-16	5.51E-16	5.10E-16	5.13E-16	4.93E-16
6.60E-16	6.39E-16	6.42E-16	5.77E-16	6.06E-16	6.27E-16	6.05E-16	6.29E-16	5.89E-16	6.65E-16	6.31E-16	5.59E-16	5.60E-16	5.37E-16
7.36E-16	7.18E-16	7.05E-16	6.57E-16	6.73E-16	6.84E-16	6.82E-16	6.92E-16	6.43E-16	6.96E-16	6.77E-16	6.14E-16	6.17E-16	5.90E-16
8.12E-16	7.33E-16	7.42E-16	7.49E-16	7.47E-16	7.52E-16	7.44E-16	7.36E-16	6.90E-16	7.22E-16	7.02E-16	6.55E-16	6.87E-16	6.60E-16
8.51E-16	7.82E-16	8.14E-16	8.08E-16	8.04E-16	8.13E-16	7.52E-16	7.76E-16	7.32E-16	7.57E-16	7.40E-16	7.38E-16	7.31E-16	7.48E-16
9.15E-16	8.72E-16	8.84E-16	8.66E-16	8.68E-16	8.44E-16	8.06E-16	8.45E-16	7.91E-16	8.06E-16	7.86E-16	8.27E-16	8.11E-16	8.22E-16
1.01E-15	9.91E-16	9.75E-16	9.25E-16	9.42E-16	9.20E-16	8.75E-16	9.44E-16	8.79E-16	8.65E-16	8.67E-16	9.14E-16	8.84E-16	8.55E-16
1.10E-15	1.13E-15	1.09E-15	9.86E-16	9.97E-16	1.00E-15	9.70E-16	1.05E-15	9.78E-16	9.45E-16	9.65E-16	9.67E-16	9.34E-16	9.29E-16
1.18E-15	1.23E-15	1.17E-15	1.06E-15	1.04E-15	1.07E-15	1.06E-15	1.13E-15	1.07E-15	1.02E-15	1.06E-15	1.01E-15	9.82E-16	1.00E-15
1.27E-15	1.31E-15	1.23E-15	1.16E-15	1.09E-15	1.14E-15	1.17E-15	1.19E-15	1.15E-15	1.09E-15	1.12E-15	1.05E-15	1.06E-15	1.05E-15
1.36E-15	1.35E-15	1.29E-15	1.26E-15	1.20E-15	1.24E-15	1.28E-15	1.26E-15	1.19E-15	1.18E-15	1.20E-15	1.14E-15	1.14E-15	1.10E-15
1.42E-15	1.37E-15	1.36E-15	1.34E-15	1.32E-15	1.32E-15	1.34E-15	1.32E-15	1.28E-15	1.26E-15	1.27E-15	1.24E-15	1.22E-15	1.19E-15
1.50E-15	1.43E-15	1.46E-15	1.42E-15	1.39E-15	1.43E-15	1.41E-15	1.38E-15	1.40E-15	1.35E-15	1.33E-15	1.34E-15	1.35E-15	1.31E-15
1.65E-15	1.53E-15	1.56E-15	1.48E-15	1.46E-15	1.54E-15	1.48E-15	1.43E-15	1.51E-15	1.47E-15	1.41E-15	1.45E-15	1.47E-15	1.44E-15
1.77E-15	1.65E-15	1.63E-15	1.59E-15	1.54E-15	1.60E-15	1.57E-15	1.52E-15	1.63E-15	1.60E-15	1.53E-15	1.58E-15	1.56E-15	1.54E-15
1.78E-15	1.71E-15	1.70E-15	1.69E-15	1.62E-15	1.69E-15	1.65E-15	1.61E-15	1.73E-15	1.68E-15	1.63E-15	1.68E-15	1.62E-15	1.61E-15
1.79E-15	1.78E-15	1.80E-15	1.77E-15	1.73E-15	1.80E-15	1.75E-15	1.74E-15	1.79E-15	1.72E-15	1.74E-15	1.78E-15	1.72E-15	1.70E-15

1.88E-15	1.90E-15	1.89E-15	1.87E-15	1.82E-15	1.90E-15	1.85E-15	1.87E-15	1.87E-15	1.77E-15	1.82E-15	1.88E-15	1.83E-15	1.78E-15
1.98E-15	1.99E-15	2.00E-15	1.96E-15	1.92E-15	1.96E-15	1.98E-15	2.01E-15	1.96E-15	1.87E-15	1.93E-15	1.96E-15	1.90E-15	1.83E-15
2.04E-15	2.04E-15	2.07E-15	2.07E-15	2.04E-15	2.08E-15	2.13E-15	2.12E-15	2.06E-15	1.98E-15	2.01E-15	2.05E-15	1.96E-15	1.93E-15
2.14E-15	2.14E-15	2.18E-15	2.20E-15	2.18E-15	2.19E-15	2.25E-15	2.23E-15	2.14E-15	2.07E-15	2.08E-15	2.14E-15	2.07E-15	2.07E-15
2.30E-15	2.29E-15	2.31E-15	2.33E-15	2.28E-15	2.30E-15	2.34E-15	2.30E-15	2.25E-15	2.20E-15	2.17E-15	2.21E-15	2.26E-15	2.24E-15
2.44E-15	2.42E-15	2.41E-15	2.39E-15	2.42E-15	2.43E-15	2.38E-15	2.38E-15	2.36E-15	2.33E-15	2.29E-15	2.33E-15	2.45E-15	2.40E-15
2.58E-15	2.55E-15	2.53E-15	2.52E-15	2.58E-15	2.56E-15	2.49E-15	2.51E-15	2.47E-15	2.45E-15	2.39E-15	2.45E-15	2.59E-15	2.54E-15
2.69E-15	2.64E-15	2.65E-15	2.69E-15	2.73E-15	2.69E-15	2.64E-15	2.63E-15	2.62E-15	2.61E-15	2.55E-15	2.53E-15	2.60E-15	2.62E-15
2.82E-15	2.80E-15	2.78E-15	2.87E-15	2.88E-15	2.83E-15	2.78E-15	2.81E-15	2.80E-15	2.77E-15	2.74E-15	2.68E-15	2.69E-15	2.74E-15
2.92E-15	2.97E-15	2.93E-15	3.06E-15	3.05E-15	2.98E-15	2.95E-15	2.95E-15	3.01E-15	2.94E-15	2.96E-15	2.83E-15	2.79E-15	2.88E-15
3.08E-15	3.13E-15	3.13E-15	3.25E-15	3.24E-15	3.15E-15	3.15E-15	3.06E-15	3.11E-15	3.15E-15	3.10E-15	2.99E-15	2.94E-15	3.02E-15
8.85E-14	8.71E-14	8.70E-14	8.58E-14	8.53E-14	8.51E-14	8.49E-14	8.47E-14	8.45E-14	8.28E-14	8.34E-14	8.28E-14	8.13E-14	7.96E-14

-3.36E-08	-3.29E-08	-3.37E-08	-3.48E-08	-3.38E-08	-3.36E-08	-3.28E-08	-3.23E-08	-3.18E-08	-3.12E-08	-3.25E-08	-3.12E-08	-3.15E-08	-3.06E-08
-3.34E-08	-3.30E-08	-3.38E-08	-3.45E-08	-3.41E-08	-3.33E-08	-3.23E-08	-3.18E-08	-3.14E-08	-3.09E-08	-3.26E-08	-3.16E-08	-3.17E-08	-3.11E-08
-3.30E-08	-3.28E-08	-3.36E-08	-3.41E-08	-3.38E-08	-3.28E-08	-3.20E-08	-3.12E-08	-3.13E-08	-3.09E-08	-3.22E-08	-3.22E-08	-3.20E-08	-3.17E-08
-3.23E-08	-3.28E-08	-3.29E-08	-3.34E-08	-3.33E-08	-3.25E-08	-3.18E-08	-3.10E-08	-3.10E-08	-3.09E-08	-3.17E-08	-3.19E-08	-3.22E-08	-3.20E-08
-3.19E-08	-3.23E-08	-3.21E-08	-3.26E-08	-3.25E-08	-3.24E-08	-3.17E-08	-3.10E-08	-3.15E-08	-3.07E-08	-3.11E-08	-3.13E-08	-3.20E-08	-3.21E-08
-3.15E-08	-3.19E-08	-3.13E-08	-3.16E-08	-3.19E-08	-3.20E-08	-3.21E-08	-3.14E-08	-3.14E-08	-3.07E-08	-3.08E-08	-3.01E-08	-3.15E-08	-3.16E-08
-3.13E-08	-3.16E-08	-3.08E-08	-3.13E-08	-3.20E-08	-3.17E-08	-3.19E-08	-3.13E-08	-3.10E-08	-3.07E-08	-3.05E-08	-2.97E-08	-3.09E-08	-3.12E-08
-3.15E-08	-3.16E-08	-3.07E-08	-3.13E-08	-3.23E-08	-3.17E-08	-3.11E-08	-3.04E-08	-3.03E-08	-3.06E-08	-3.06E-08	-3.01E-08	-3.06E-08	-3.09E-08
-3.15E-08	-3.17E-08	-3.09E-08	-3.14E-08	-3.17E-08	-3.12E-08	-3.07E-08	-3.06E-08	-3.02E-08	-3.04E-08	-3.07E-08	-3.02E-08	-3.04E-08	-3.04E-08
-3.10E-08	-3.12E-08	-3.13E-08	-3.10E-08	-3.10E-08	-3.05E-08	-3.02E-08	-3.07E-08	-3.05E-08	-3.00E-08	-3.04E-08	-3.02E-08	-2.99E-08	-3.00E-08
-3.03E-08	-3.06E-08	-3.13E-08	-3.06E-08	-3.04E-08	-2.95E-08	-3.02E-08	-3.06E-08	-3.06E-08	-2.95E-08	-3.00E-08	-3.00E-08	-2.89E-08	-2.95E-08
-2.97E-08	-3.01E-08	-3.07E-08	-3.03E-08	-3.00E-08	-2.91E-08	-3.01E-08	-2.99E-08	-2.98E-08	-2.89E-08	-2.91E-08	-2.95E-08	-2.83E-08	-2.89E-08
-2.88E-08	-2.97E-08	-3.03E-08	-2.97E-08	-2.95E-08	-2.88E-08	-2.92E-08	-2.90E-08	-2.91E-08	-2.84E-08	-2.89E-08	-2.91E-08	-2.82E-08	-2.84E-08
-2.79E-08	-2.94E-08	-3.00E-08	-2.95E-08	-2.94E-08	-2.90E-08	-2.85E-08	-2.83E-08	-2.81E-08	-2.80E-08	-2.86E-08	-2.88E-08	-2.80E-08	-2.81E-08
-2.83E-08	-2.90E-08	-2.94E-08	-2.94E-08	-2.94E-08	-2.89E-08	-2.84E-08	-2.82E-08	-2.84E-08	-2.79E-08	-2.81E-08	-2.83E-08	-2.80E-08	-2.77E-08
-2.91E-08	-2.90E-08	-2.89E-08	-2.91E-08	-2.94E-08	-2.88E-08	-2.88E-08	-2.84E-08	-2.85E-08	-2.78E-08	-2.77E-08	-2.82E-08	-2.74E-08	-2.72E-08
-2.93E-08	-2.90E-08	-2.86E-08	-2.87E-08	-2.87E-08	-2.82E-08	-2.83E-08	-2.77E-08	-2.79E-08	-2.73E-08	-2.75E-08	-2.77E-08	-2.65E-08	-2.68E-08
-2.90E-08	-2.92E-08	-2.86E-08	-2.81E-08	-2.78E-08	-2.79E-08	-2.78E-08	-2.71E-08	-2.77E-08	-2.72E-08	-2.74E-08	-2.73E-08	-2.61E-08	-2.60E-08
-2.86E-08	-2.90E-08	-2.84E-08	-2.80E-08	-2.75E-08	-2.74E-08	-2.76E-08	-2.71E-08	-2.74E-08	-2.72E-08	-2.67E-08	-2.67E-08	-2.56E-08	-2.53E-08
-2.78E-08	-2.81E-08	-2.79E-08	-2.76E-08	-2.71E-08	-2.69E-08	-2.72E-08	-2.78E-08	-2.78E-08	-2.73E-08	-2.61E-08	-2.58E-08	-2.52E-08	-2.48E-08
-2.69E-08	-2.73E-08	-2.74E-08	-2.66E-08	-2.63E-08	-2.61E-08	-2.63E-08	-2.84E-08	-2.81E-08	-2.70E-08	-2.54E-08	-2.50E-08	-2.50E-08	-2.50E-08
-2.65E-08	-2.65E-08	-2.65E-08	-2.56E-08	-2.53E-08	-2.55E-08	-2.58E-08	-2.82E-08	-2.80E-08	-2.70E-08	-2.54E-08	-2.48E-08	-2.49E-08	-2.50E-08
-2.56E-08	-2.61E-08	-2.58E-08	-2.49E-08	-2.46E-08	-2.49E-08	-2.59E-08	-2.72E-08	-2.75E-08	-2.65E-08	-2.50E-08	-2.53E-08	-2.44E-08	-2.49E-08
-2.46E-08	-2.56E-08	-2.54E-08	-2.47E-08	-2.47E-08	-2.47E-08	-2.60E-08	-2.66E-08	-2.67E-08	-2.58E-08	-2.46E-08	-2.52E-08	-2.41E-08	-2.48E-08
-2.40E-08	-2.48E-08	-2.48E-08	-2.54E-08	-2.48E-08	-2.47E-08	-2.59E-08	-2.58E-08	-2.59E-08	-2.49E-08	-2.44E-08	-2.52E-08	-2.45E-08	-2.46E-08
-2.38E-08	-2.41E-08	-2.44E-08	-2.52E-08	-2.42E-08	-2.45E-08	-2.55E-08	-2.51E-08	-2.51E-08	-2.49E-08	-2.41E-08	-2.51E-08	-2.49E-08	-2.43E-08
-2.37E-08	-2.32E-08	-2.38E-08	-2.44E-08	-2.36E-08	-2.37E-08	-2.45E-08	-2.46E-08	-2.44E-08	-2.47E-08	-2.37E-08	-2.45E-08	-2.44E-08	-2.41E-08
-2.37E-08	-2.27E-08	-2.30E-08	-2.34E-08	-2.32E-08	-2.31E-08	-2.36E-08	-2.39E-08	-2.38E-08	-2.45E-08	-2.33E-08	-2.33E-08	-2.36E-08	-2.38E-08
-2.35E-08	-2.30E-08	-2.29E-08	-2.29E-08	-2.26E-08	-2.26E-08	-2.30E-08	-2.33E-08	-2.34E-08	-2.42E-08	-2.26E-08	-2.19E-08	-2.29E-08	-2.34E-08
-2.33E-08	-2.32E-08	-2.31E-08	-2.26E-08	-2.22E-08	-2.21E-08	-2.27E-08	-2.31E-08	-2.33E-08	-2.39E-08	-2.26E-08	-2.17E-08	-2.27E-08	-2.32E-08
-2.28E-08	-2.29E-08	-2.26E-08	-2.24E-08	-2.18E-08	-2.20E-08	-2.21E-08	-2.23E-08	-2.30E-08	-2.35E-08	-2.30E-08	-2.28E-08	-2.27E-08	-2.29E-08
-2.21E-08	-2.28E-08	-2.23E-08	-2.21E-08	-2.14E-08	-2.17E-08	-2.13E-08	-2.17E-08	-2.23E-08	-2.26E-08	-2.27E-08	-2.25E-08	-2.20E-08	-2.20E-08
-2.16E-08	-2.23E-08	-2.18E-08	-2.13E-08	-2.13E-08	-2.12E-08	-2.06E-08	-2.10E-08	-2.16E-08	-2.18E-08	-2.17E-08	-2.14E-08	-2.09E-08	-2.09E-08
-2.12E-08	-2.16E-08	-2.15E-08	-2.08E-08	-2.12E-08	-2.07E-08	-2.00E-08	-2.07E-08	-2.07E-08	-2.08E-08	-2.07E-08	-2.04E-08	-2.00E-08	-2.05E-08
-2.04E-08	-2.10E-08	-2.08E-08	-2.00E-08	-2.07E-08	-2.06E-08	-1.92E-08	-2.04E-08	-1.96E-08	-2.02E-08	-1.99E-08	-1.97E-08	-1.93E-08	-2.01E-08
-2.01E-08	-2.02E-08	-1.98E-08	-1.90E-08	-1.97E-08	-1.99E-08	-1.87E-08	-2.00E-08	-1.88E-08	-1.92E-08	-1.95E-08	-1.94E-08	-1.90E-08	-1.96E-08
-1.98E-08	-1.97E-08	-1.89E-08	-1.86E-08	-1.92E-08	-1.92E-08	-1.87E-08	-1.91E-08	-1.85E-08	-1.81E-08	-1.85E-08	-1.89E-08	-1.83E-08	-1.91E-08
-1.90E-08	-1.90E-08	-1.81E-08	-1.81E-08	-1.88E-08	-1.86E-08	-1.87E-08	-1.82E-08	-1.79E-08	-1.74E-08	-1.77E-08	-1.80E-08	-1.75E-08	-1.82E-08
-1.81E-08	-1.85E-08	-1.73E-08	-1.78E-08	-1.84E-08	-1.83E-08	-1.80E-08	-1.77E-08	-1.78E-08	-1.62E-08	-1.73E-08	-1.73E-08	-1.73E-08	-1.73E-08
-1.74E-08	-1.76E-08	-1.65E-08	-1.72E-08	-1.80E-08	-1.77E-08	-1.72E-08	-1.67E-08	-1.71E-08	-1.59E-08	-1.70E-08	-1.67E-08	-1.67E-08	-1.67E-08
-1.66E-08	-1.68E-08	-1.59E-08	-1.62E-08	-1.69E-08	-1.70E-08	-1.63E-08	-1.50E-08	-1.60E-08	-1.63E-08	-1.62E-08	-1.63E-08	-1.63E-08	-1.60E-08
-1.56E-08	-1.60E-08	-1.56E-08	-1.58E-08	-1.57E-08	-1.63E-08	-1.52E-08	-1.42E-08	-1.47E-08	-1.58E-08	-1.56E-08	-1.51E-08	-1.56E-08	-1.53E-08
-1.44E-08	-1.48E-08	-1.49E-08	-1.52E-08	-1.44E-08	-1.51E-08	-1.44E-08	-1.39E-08	-1.44E-08	-1.49E-08	-1.51E-08	-1.41E-08	-1.47E-08	-1.47E-08
-1.31E-08	-1.38E-08	-1.38E-08	-1.39E-08	-1.38E-08	-1.44E-08	-1.39E-08	-1.46E-08	-1.42E-08	-1.43E-08	-1.45E-08	-1.37E-08	-1.43E-08	-1.42E-08
-1.23E-08	-1.28E-08	-1.34E-08	-1.31E-08	-1.31E-08	-1.36E-08	-1.38E-08	-1.37E-08	-1.36E-08	-1.36E-08	-1.39E-08	-1.40E-08	-1.39E-08	-1.38E-08
-1.14E-08	-1.19E-08	-1.28E-08	-1.28E-08	-1.27E-08	-1.27E-08	-1.32E-08	-1.28E-08	-1.27E-08	-1.28E-08	-1.34E-08	-1.36E-08	-1.32E-08	-1.30E-08
-1.09E-08	-1.16E-08	-1.27E-08	-1.21E-08	-1.18E-08	-1.16E-08	-1.22E-08	-1.21E-08	-1.20E-08	-1.24E-08	-1.30E-08	-1.29E-08	-1.23E-08	-1.20E-08
-1.07E-08	-1.16E-08	-1.22E-08	-1.13E-08	-1.08E-08	-1.10E-08	-1.14E-08	-1.21E-08	-1.20E-08	-1.21E-08	-1.20E-08	-1.17E-08	-1.10E-08	-1.11E-08
-1.04E-08	-1.10E-08	-1.14E-08	-1.01E-08	-9.96E-09	-1.09E-08	-1.11E-08	-1.17E-08	-1.18E-08	-1.17E-08	-1.03E-08	-1.06E-08	-1.05E-08	-1.04E-08
-9.94E-09	-1.02E-08	-1.05E-08	-8.93E-09	-9.51E-09	-1.04E-08	-1.06E-08	-1.08E-08	-1.11E-08	-1.09E-08	-7.51E-09	-9.36E-09	-1.05E-08	-1.05E-08
-9.69E-09	-9.47E-09	-9.64E-09	-8.55E-09	-9.38E-09	-9.97E-09	-1.09E-08	-1.05E-08	-1.05E-08	-9.94E-09	-2.91E-09	-7.13E-09	-1.03E-08	-9.98E-09
-9.54E-09	-9.03E-09	-9.14E-09	-8.17E-09	-9.72E-09	-1.00E-08	-1.05E-08	-9.69E-09	-9.81E-09	-9.32E-09	3.34E-09	-2.56E-09	-9.78E-09	-9.08E-09
-9.02E-09	-8.72E-09	-8.64E-09	-7.80E-09	-9.07E-09	-9.12E-09	-9.64E-09	-8.98E-09	-8.87E-09	-8.82E-09	3.29E-09	3.60E-09	-9.10E-09	-9.20E-09
-8.32E-09	-7.95E-09	-8.24E-09	-8.26E-09	-8.35E-09	-8.12E-09	-9.25E-09	-8.14E-09	-8.42E-09	-7.76E-09	-2.78E-09	2.79E-09	-8.63E-09	-9.09E-09
-8.15E-09	-7.28E-09	-7.63E-09	-8.44E-09	-7.71E-09	-7.82E-09	-8.88E-09	-7.94E-09	-7.94E-09	-6.96E-09	-7.86E-09	-4.26E-09	-7.68E-09	-8.30E-09
-7.94E-09	-7.07E-09	-7.40E-09	-8.22E-09	-7.14E-09	-7.23E-09	-8.05E-09	-7.64E-09	-6.93E-09	-6.81E-09	-6.99E-09	-7.55E-09	-6.79E-09	-7.60E-09
-8.05E-09	-7.10E-09	-7.60E-09	-7.33E-09	-7.04E-09	-6.86E-09	-7.17E-09	-7.02E-09	-5.65E-09	-6.66E-09	-6.42E-09	-7.06E-09	-6.05E-09	-6.98E-09
-7.59E-09	-6.99E-09	-7.51E-09	-6.50E-09	-6.81E-09	-7.03E-09	-6.79E-09	-6.29E-09	-5.01E-09	-6.51E-09	-6.39E-09	-6.64E-09	-5.68E-09	-6.17E-09
-6.73E-09	-6.26E-09	-6.82E-09	-6.34E-09	-6.22E-09	-6.20E-09	-6.28E-09	-5.96E-09	-5.29E-09	-5.96E-09	-5.53E-09	-5.81E-09	-4.97E-09	-5.41E-09
-5.67E-09	-5.51E-09	-6.58E-09	-5.72E-09	-5.56E-09	-5.29E-09	-5.78E-09	-5.74E-09	-5.08E-09	-5.22E-09	-4.63E-09	-5.16E-09	-4.51E-09	-4.85E-09
-4.81E-09	-5.15E-09	-5.81E-09	-5.11E-09	-4.78E-09	-4.37E-09	-4.68E-09	-5.27E-09	-4.45E-09	-4.31E-09	-3.97E-09	-4.75E-09	-4.53E-09	-4.26E-09
-4.55E-09	-4.43E-09	-4.94E-09	-4.56E-09	-4.59E-09	-3.45E-09	-3.59E-09	-4.50E-09	-3.72E-09	-2.99E-09	-3.61E-09	-4.49E-09	-3.95E-09	-4.04E-09
-4.06E-09	-3.50E-09	-4.29E-09	-4.15E-09	-4.35E-09	-3.24E-09	-3.21E-09	-3.72E-09	-3.10E-09	-2.79E-09	-3.57E-09	-3.62E-09	-2.95E-09	-3.61E-09
-3.52E-09	-2.81E-09	-3.49E-09	-3.63E-09	-3.09E-09	-2.75E-09	-2.82E-09	-2.99E-09	-2.49E-09	-2.80E-09	-3.19E-09	-2.42E-09	-2.49E-09	-3.00E-09
-3.02E-09	-2.46E-09	-2.66E-09	-2.80E-09	-1.91E-09	-2.29E-09	-2.24E-09	-2.85E-09	-2.33E-09	-2.39E-09	-2.52E-09	-1.58E-09	-1.86E-09	-2.05E-09
-2.16E-09	-2.50E-09	-1.75E-09	-1.84E-09	-1.14E-09	-1.99E-09	-1.94E-09	-2.88E-09	-2.21E-09	-1.09E-09	-1.95E-09	-9.65E-10	-1.08E-09	-1.31E-09
-1.51E-09	-2.40E-09	-9.23E-10	-6.61E-10	-5.93E-10	-1.53E-09	-1.49E-09	-2.47E-09	-1.89E-09	-6.01E-11	-1.26E-09	-1.92E-10	-3.47E-12	-8.11E-10
-6.22E-10	-1.81E-09	-5.19E-10	4.50E-10	-1.75E-10	-1.11E-09	-1.08E-09	-1.74E-09	-1.21E-09	-3.21E-10	-6.04E-10	-4.72E-10	3.24E-10	-2.09E-10
1.26E-10	-9.54E-10	7.69E-11	5.69E-10	2.43E-10	-2.47E-10	-4.31E-10	-7.50E-10	-4.09E-10	-2.20E-10	-5.01E-10	-2.88E-10	6.01E-10	4.24E-10
3.49E-10	3.13E-11	6.06E-10	3.90E-10	9.25E-10	6.92E-10	2.56E-10	-2.09E-10	3.12E-10	4.86E-10	3.46E-11	2.07E-10	1.09E-09	1.03E-09
8.58E-10	7.73E-10	1.18E-09	8.16E-10	1.09E-09	1.37E-09	3.24E-10	2.32E-10	1.02E-09	1.08E-09	9.90E-10	7.88E-10	1.94E-09	1.47E-09
1.18E-09	1.73E-09	2.20E-09	1.85E-09	1.65E-09	1.51E-09	9.36E-10	1.25E-09	1.45E-09	1.65E-09	1.77E-09	1.53E-09	2.86E-09	2.34E-09
1.44E-09	2.34E-09	2.72E-09	2.60E-09	1.56E-09	1.78E-09	1.57E-09	2.18E-09	1.66E-09	2.24E-09	1.67E-09	2.19E-09	2.80E-09	2.72E-09
2.33E-09	2.64E-09	3.31E-09	2.47E-09	1.47E-09	2.32E-09	2.15E-09	2.92E-09	2.40E-09	2.79E-09	1.90E-09	2.03E-09	2.29E-09	2.94E-09
3.24E-09	2.97E-09	3.78E-09	2.52E-09	2.01E-09	3.21E-09	3.10E-09	3.55E-09	3.13E-09	3.84E-09	3.09E-09	2.59E-09	2.99E-09	3.62E-09
4.19E-09	3.58E-09	3.98E-09	2.98E-09	2.57E-09	3.67E-09	3.80E-09	4.02E-09	3.84E-09	4.31E-09	3.90E-09	3.29E-09	4.07E-09	4.42E-09
4.77E-09	4.45E-09	4.67E-09	3.51E-09	3.19E-09	3.94E-09	4.34E-09	4.35E-09	4.30E-09	4.73E-09	4.49E-09	4.15E-09	5.08E-09	4.81E-09
5.41E-09	4.95E-09	5.41E-09	4.39E-09	4.28E-09	4.64E-09	4.93E-09	4.63E-09	4.89E-09	5.32E-09	5.19E-09	5.31E-09	5.99E-09	5.36E-09

5.56E-09	5.33E-09	5.90E-09	5.31E-09	5.60E-09	5.96E-09	5.33E-09	5.10E-09	5.78E-09	6.30E-09	5.80E-09	5.90E-09	5.90E-09	6.03E-09
5.65E-09	6.14E-09	6.25E-09	6.32E-09	7.11E-09	6.97E-09	6.35E-09	6.13E-09	6.74E-09	6.67E-09	6.25E-09	6.22E-09	5.91E-09	6.68E-09
6.30E-09	6.86E-09	7.07E-09	7.58E-09	8.00E-09	7.04E-09	6.90E-09	7.30E-09	7.27E-09	6.75E-09	6.76E-09	6.53E-09	6.24E-09	7.19E-09
7.44E-09	7.44E-09	7.84E-09	8.46E-09	8.36E-09	7.30E-09	7.69E-09	8.34E-09	8.11E-09	7.34E-09	7.19E-09	7.05E-09	6.70E-09	7.83E-09
8.69E-09	8.24E-09	8.58E-09	8.33E-09	9.00E-09	8.15E-09	8.55E-09	8.92E-09	8.44E-09	8.32E-09	8.05E-09	7.59E-09	7.61E-09	8.84E-09
9.16E-09	9.10E-09	9.20E-09	8.85E-09	9.41E-09	8.76E-09	8.74E-09	9.33E-09	8.74E-09	9.18E-09	8.89E-09	8.14E-09	8.57E-09	9.18E-09
9.65E-09	1.00E-08	9.67E-09	9.32E-09	9.81E-09	8.88E-09	8.94E-09	9.41E-09	9.15E-09	9.76E-09	9.34E-09	9.15E-09	9.49E-09	9.85E-09
1.05E-08	1.04E-08	1.02E-08	9.96E-09	9.97E-09	9.37E-09	9.36E-09	9.80E-09	9.61E-09	1.04E-08	1.01E-08	1.01E-08	1.02E-08	1.07E-08
1.11E-08	1.07E-08	1.06E-08	1.06E-08	1.03E-08	1.02E-08	9.92E-09	1.05E-08	1.06E-08	1.08E-08	1.08E-08	1.06E-08	1.11E-08	1.14E-08
1.12E-08	1.09E-08	1.11E-08	1.20E-08	1.08E-08	1.09E-08	1.05E-08	1.09E-08	1.15E-08	1.11E-08	1.20E-08	1.09E-08	1.16E-08	1.19E-08
1.15E-08	1.14E-08	1.20E-08	1.31E-08	1.16E-08	1.19E-08	1.17E-08	1.15E-08	1.22E-08	1.22E-08	1.29E-08	1.22E-08	1.20E-08	1.24E-08
1.21E-08	1.23E-08	1.24E-08	1.37E-08	1.22E-08	1.22E-08	1.21E-08	1.28E-08	1.33E-08	1.33E-08	1.35E-08	1.33E-08	1.28E-08	1.26E-08
1.28E-08	1.36E-08	1.33E-08	1.35E-08	1.29E-08	1.29E-08	1.29E-08	1.43E-08	1.47E-08	1.41E-08	1.44E-08	1.44E-08	1.39E-08	1.28E-08
1.40E-08	1.47E-08	1.42E-08	1.37E-08	1.36E-08	1.35E-08	1.39E-08	1.55E-08	1.58E-08	1.46E-08	1.50E-08	1.51E-08	1.49E-08	1.40E-08
1.54E-08	1.55E-08	1.51E-08	1.48E-08	1.48E-08	1.46E-08	1.51E-08	1.65E-08	1.64E-08	1.52E-08	1.53E-08	1.56E-08	1.59E-08	1.53E-08
1.67E-08	1.64E-08	1.60E-08	1.58E-08	1.59E-08	1.57E-08	1.62E-08	1.71E-08	1.69E-08	1.59E-08	1.60E-08	1.61E-08	1.62E-08	1.58E-08
1.76E-08	1.74E-08	1.66E-08	1.65E-08	1.67E-08	1.66E-08	1.73E-08	1.69E-08	1.71E-08	1.67E-08	1.65E-08	1.72E-08	1.66E-08	1.67E-08
1.84E-08	1.78E-08	1.73E-08	1.75E-08	1.78E-08	1.74E-08	1.81E-08	1.76E-08	1.75E-08	1.71E-08	1.69E-08	1.79E-08	1.70E-08	1.79E-08
1.90E-08	1.87E-08	1.84E-08	1.86E-08	1.88E-08	1.84E-08	1.89E-08	1.83E-08	1.79E-08	1.79E-08	1.71E-08	1.88E-08	1.83E-08	1.84E-08
1.99E-08	1.99E-08	1.98E-08	1.96E-08	1.95E-08	1.95E-08	1.96E-08	1.87E-08	1.86E-08	1.87E-08	1.72E-08	1.95E-08	1.92E-08	1.88E-08
2.10E-08	2.14E-08	2.11E-08	2.07E-08	2.05E-08	2.03E-08	2.01E-08	1.94E-08	1.94E-08	1.98E-08	1.84E-08	2.02E-08	1.96E-08	1.91E-08
2.21E-08	2.25E-08	2.16E-08	2.17E-08	2.11E-08	2.08E-08	2.06E-08	2.04E-08	2.02E-08	2.06E-08	1.96E-08	2.05E-08	2.02E-08	1.98E-08
2.26E-08	2.30E-08	2.25E-08	2.27E-08	2.21E-08	2.18E-08	2.16E-08	2.18E-08	2.18E-08	2.15E-08	2.06E-08	2.09E-08	2.09E-08	2.07E-08
2.30E-08	2.36E-08	2.35E-08	2.37E-08	2.37E-08	2.31E-08	2.29E-08	2.33E-08	2.33E-08	2.28E-08	2.17E-08	2.17E-08	2.18E-08	2.20E-08
2.38E-08	2.46E-08	2.49E-08	2.47E-08	2.50E-08	2.42E-08	2.42E-08	2.38E-08	2.38E-08	2.40E-08	2.32E-08	2.29E-08	2.29E-08	2.29E-08
2.50E-08	2.55E-08	2.63E-08	2.59E-08	2.60E-08	2.54E-08	2.53E-08	2.49E-08	2.41E-08	2.50E-08	2.45E-08	2.43E-08	2.47E-08	2.41E-08
2.62E-08	2.66E-08	2.75E-08	2.70E-08	2.69E-08	2.63E-08	2.67E-08	2.59E-08	2.50E-08	2.58E-08	2.59E-08	2.57E-08	2.64E-08	2.52E-08
2.76E-08	2.77E-08	2.80E-08	2.76E-08	2.77E-08	2.70E-08	2.80E-08	2.70E-08	2.59E-08	2.68E-08	2.70E-08	2.67E-08	2.71E-08	2.62E-08
2.91E-08	2.88E-08	2.86E-08	2.86E-08	2.86E-08	2.80E-08	2.85E-08	2.80E-08	2.66E-08	2.75E-08	2.75E-08	2.73E-08	2.77E-08	2.70E-08
2.97E-08	3.02E-08	2.98E-08	3.00E-08	2.98E-08	2.92E-08	2.88E-08	2.89E-08	2.77E-08	2.84E-08	2.81E-08	2.84E-08	2.81E-08	2.85E-08
3.07E-08	3.18E-08	3.11E-08	3.09E-08	3.07E-08	3.04E-08	2.96E-08	3.00E-08	2.93E-08	2.94E-08	2.89E-08	2.91E-08	2.87E-08	2.93E-08
3.17E-08	3.35E-08	3.27E-08	3.21E-08	3.20E-08	3.15E-08	3.10E-08	3.11E-08	3.05E-08	3.02E-08	2.99E-08	2.99E-08	2.98E-08	3.03E-08
3.28E-08	3.35E-08	3.41E-08	3.36E-08	3.33E-08	3.30E-08	3.19E-08	3.23E-08	3.14E-08	3.12E-08	3.08E-08	3.11E-08	3.09E-08	3.12E-08
3.43E-08	3.44E-08	3.52E-08	3.50E-08	3.44E-08	3.44E-08	3.31E-08	3.31E-08	3.26E-08	3.24E-08	3.19E-08	3.21E-08	3.20E-08	3.23E-08
3.60E-08	3.54E-08	3.62E-08	3.64E-08	3.56E-08	3.54E-08	3.42E-08	3.42E-08	3.38E-08	3.40E-08	3.30E-08	3.31E-08	3.28E-08	3.31E-08
3.75E-08	3.61E-08	3.71E-08	3.75E-08	3.64E-08	3.62E-08	3.55E-08	3.50E-08	3.51E-08	3.53E-08	3.43E-08	3.40E-08	3.38E-08	3.41E-08
3.87E-08	3.72E-08	3.78E-08	3.79E-08	3.70E-08	3.67E-08	3.64E-08	3.58E-08	3.67E-08	3.66E-08	3.53E-08	3.52E-08	3.47E-08	3.50E-08
3.94E-08	3.89E-08	3.91E-08	3.83E-08	3.78E-08	3.77E-08	3.74E-08	3.66E-08	3.81E-08	3.83E-08	3.67E-08	3.64E-08	3.61E-08	3.63E-08
4.06E-08	4.05E-08	4.07E-08	3.95E-08	3.91E-08	3.90E-08	3.86E-08	3.81E-08	3.97E-08	3.92E-08	3.81E-08	3.76E-08	3.80E-08	3.79E-08
4.17E-08	4.18E-08	4.18E-08	4.12E-08	4.04E-08	4.06E-08	4.02E-08	3.98E-08	4.12E-08	4.02E-08	3.95E-08	3.90E-08	3.96E-08	3.91E-08
4.27E-08	4.31E-08	4.23E-08	4.28E-08	4.17E-08	4.22E-08	4.16E-08	4.14E-08	4.24E-08	4.14E-08	4.05E-08	4.03E-08	4.06E-08	4.04E-08
4.39E-08	4.41E-08	4.31E-08	4.33E-08	4.31E-08	4.37E-08	4.29E-08	4.28E-08	4.34E-08	4.25E-08	4.16E-08	4.14E-08	4.16E-08	4.10E-08
4.56E-08	4.55E-08	4.43E-08	4.39E-08	4.44E-08	4.50E-08	4.37E-08	4.41E-08	4.43E-08	4.35E-08	4.28E-08	4.25E-08	4.29E-08	4.24E-08
4.74E-08	4.70E-08	4.57E-08	4.50E-08	4.59E-08	4.60E-08	4.50E-08	4.55E-08	4.52E-08	4.48E-08	4.41E-08	4.33E-08	4.36E-08	4.38E-08
4.93E-08	4.91E-08	4.72E-08	4.66E-08	4.73E-08	4.69E-08	4.69E-08	4.66E-08	4.60E-08	4.59E-08	4.60E-08	4.47E-08	4.50E-08	4.48E-08
5.05E-08	5.10E-08	4.85E-08	4.82E-08	4.87E-08	4.81E-08	4.85E-08	4.74E-08	4.70E-08	4.68E-08	4.75E-08	4.60E-08	4.64E-08	4.58E-08
5.16E-08	5.15E-08	4.99E-08	4.95E-08	4.98E-08	4.93E-08	4.99E-08	4.85E-08	4.84E-08	4.82E-08	4.77E-08	4.72E-08	4.78E-08	4.75E-08
5.18E-08	5.13E-08	5.12E-08	5.09E-08	5.11E-08	5.04E-08	5.07E-08	4.98E-08	5.01E-08	4.93E-08	4.92E-08	4.88E-08	4.91E-08	4.88E-08
5.25E-08	5.20E-08	5.22E-08	5.25E-08	5.24E-08	5.18E-08	5.18E-08	5.11E-08	5.14E-08	5.08E-08	5.04E-08	5.05E-08	5.07E-08	5.04E-08
5.41E-08	5.37E-08	5.35E-08	5.38E-08	5.37E-08	5.37E-08	5.35E-08	5.29E-08	5.29E-08	5.27E-08	5.18E-08	5.21E-08	5.19E-08	5.21E-08

1.13E-15	1.08E-15	1.13E-15	1.21E-15	1.14E-15	1.13E-15	1.07E-15	1.04E-15	1.01E-15	9.76E-16	1.06E-15	9.75E-16	9.90E-16	9.35E-16
1.11E-15	1.09E-15	1.14E-15	1.19E-15	1.16E-15	1.11E-15	1.04E-15	1.01E-15	9.84E-16	9.57E-16	1.06E-15	1.00E-15	1.00E-15	9.69E-16
1.09E-15	1.08E-15	1.13E-15	1.16E-15	1.15E-15	1.08E-15	1.02E-15	9.73E-16	9.79E-16	9.56E-16	1.04E-15	1.04E-15	1.02E-15	1.00E-15
1.04E-15	1.07E-15	1.08E-15	1.11E-15	1.11E-15	1.06E-15	1.01E-15	9.61E-16	9.59E-16	9.54E-16	1.00E-15	1.02E-15	1.04E-15	1.02E-15
1.02E-15	1.04E-15	1.03E-15	1.06E-15	1.06E-15	1.05E-15	1.00E-15	9.60E-16	9.92E-16	9.41E-16	9.70E-16	9.77E-16	1.02E-15	1.03E-15
9.94E-16	1.02E-15	9.82E-16	1.00E-15	1.02E-15	1.03E-15	1.03E-15	9.87E-16	9.84E-16	9.44E-16	9.51E-16	9.05E-16	9.92E-16	1.00E-15
9.83E-16	1.00E-15	9.46E-16	9.82E-16	1.02E-15	1.01E-15	1.02E-15	9.78E-16	9.63E-16	9.40E-16	9.29E-16	8.85E-16	9.55E-16	9.71E-16
9.90E-16	1.00E-15	9.40E-16	9.80E-16	1.04E-15	1.01E-15	9.67E-16	9.25E-16	9.19E-16	9.36E-16	9.37E-16	9.06E-16	9.38E-16	9.57E-16
9.95E-16	1.01E-15	9.56E-16	9.86E-16	1.00E-15	9.76E-16	9.39E-16	9.35E-16	9.14E-16	9.27E-16	9.45E-16	9.09E-16	9.21E-16	9.24E-16
9.60E-16	9.74E-16	9.80E-16	9.64E-16	9.63E-16	9.32E-16	9.14E-16	9.44E-16	9.30E-16	9.00E-16	9.23E-16	9.13E-16	8.94E-16	9.00E-16
9.20E-16	9.38E-16	9.79E-16	9.39E-16	9.25E-16	8.68E-16	9.12E-16	9.35E-16	9.34E-16	8.69E-16	9.00E-16	8.98E-16	8.35E-16	8.72E-16
8.83E-16	9.08E-16	9.44E-16	9.18E-16	8.99E-16	8.48E-16	9.09E-16	8.96E-16	8.90E-16	8.33E-16	8.48E-16	8.68E-16	7.99E-16	8.37E-16
8.29E-16	8.83E-16	9.18E-16	8.82E-16	8.70E-16	8.32E-16	8.55E-16	8.42E-16	8.44E-16	8.04E-16	8.34E-16	8.49E-16	7.93E-16	8.09E-16
7.81E-16	8.64E-16	8.97E-16	8.71E-16	8.65E-16	8.40E-16	8.10E-16	8.01E-16	7.92E-16	7.81E-16	8.17E-16	8.27E-16	7.86E-16	7.87E-16
8.03E-16	8.41E-16	8.67E-16	8.65E-16	8.66E-16	8.38E-16	8.06E-16	7.96E-16	8.06E-16	7.81E-16	7.87E-16	8.03E-16	7.81E-16	7.67E-16
8.48E-16	8.42E-16	8.34E-16	8.48E-16	8.62E-16	8.30E-16	8.29E-16	8.06E-16	8.11E-16	7.71E-16	7.69E-16	7.96E-16	7.50E-16	7.41E-16
8.56E-16	8.41E-16	8.17E-16	8.22E-16	8.22E-16	7.95E-16	8.00E-16	7.66E-16	7.80E-16	7.43E-16	7.58E-16	7.67E-16	7.05E-16	7.19E-16
8.39E-16	8.51E-16	8.16E-16	7.90E-16	7.76E-16	7.77E-16	7.71E-16	7.36E-16	7.65E-16	7.37E-16	7.49E-16	7.43E-16	6.82E-16	6.78E-16
8.19E-16	8.39E-16	8.06E-16	7.83E-16	7.55E-16	7.49E-16	7.60E-16	7.36E-16	7.52E-16	7.42E-16	7.11E-16	7.12E-16	6.55E-16	6.39E-16
7.70E-16	7.90E-16	7.76E-16	7.64E-16	7.32E-16	7.23E-16	7.39E-16	7.72E-16	7.75E-16	7.44E-16	6.84E-16	6.64E-16	6.37E-16	6.16E-16
7.22E-16	7.47E-16	7.50E-16	7.09E-16	6.94E-16	6.81E-16	6.93E-16	8.09E-16	7.89E-16	7.28E-16	6.46E-16	6.27E-16	6.27E-16	6.25E-16
7.02E-16	7.05E-16	7.02E-16	6.56E-16	6.39E-16	6.53E-16	6.64E-16	7.95E-16	7.83E-16	7.28E-16	6.44E-16	6.13E-16	6.19E-16	6.24E-16
6.55E-16	6.80E-16	6.64E-16	6.19E-16	6.06E-16	6.22E-16	6.70E-16	7.42E-16	7.55E-16	7.01E-16	6.25E-16	6.42E-16	5.97E-16	6.18E-16
6.03E-16	6.56E-16	6.46E-16	6.11E-16	6.11E-16	6.08E-16	6.73E-16	7.06E-16	7.13E-16	6.64E-16	6.04E-16	6.37E-16	5.82E-16	6.13E-16
5.77E-16	6.13E-16	6.15E-16	6.43E-16	6.17E-16	6.10E-16	6.70E-16	6.66E-16	6.71E-16	6.21E-16	5.97E-16	6.36E-16	5.98E-16	6.05E-16
5.68E-16	5.81E-16	5.95E-16	6.33E-16	5.87E-16	6.01E-16	6.49E-16	6.30E-16	6.29E-16	6.21E-16	5.82E-16	6.30E-16	6.20E-16	5.91E-16
5.63E-16	5.40E-16	5.65E-16	5.94E-16	5.57E-16	5.62E-16	6.01E-16	6.06E-16	5.97E-16	6.10E-16	5.64E-16	6.02E-16	5.95E-16	5.83E-16
5.61E-16	5.15E-16	5.30E-16	5.49E-16	5.37E-16	5.34E-16	5.59E-16	5.71E-16	5.67E-16	6.01E-16	5.44E-16	5.41E-16	5.59E-16	5.66E-16
5.54E-16	5.30E-16	5.27E-16	5.24E-16	5.12E-16	5.11E-16	5.28E-16	5.42E-16	5.49E-16	5.84E-16	5.09E-16	4.81E-16	5.23E-16	5.50E-16
5.43E-16	5.37E-16	5.32E-16	5.12E-16	4.95E-16	4.88E-16	5.15E-16	5.32E-16	5.43E-16	5.71E-16	5.12E-16	4.71E-16	5.17E-16	5.39E-16
5.20E-16	5.26E-16	5.11E-16	5.02E-16	4.74E-16	4.83E-16	4.89E-16	4.99E-16	5.28E-16	5.52E-16	5.28E-16	5.19E-16	5.13E-16	5.25E-16

4.91E-16	5.21E-16	4.98E-16	4.89E-16	4.58E-16	4.69E-16	4.52E-16	4.69E-16	4.97E-16	5.10E-16	5.16E-16	5.05E-16	4.83E-16	4.82E-16
4.67E-16	4.99E-16	4.74E-16	4.56E-16	4.52E-16	4.48E-16	4.24E-16	4.42E-16	4.66E-16	4.73E-16	4.71E-16	4.58E-16	4.38E-16	4.35E-16
4.51E-16	4.66E-16	4.60E-16	4.34E-16	4.48E-16	4.28E-16	4.02E-16	4.26E-16	4.30E-16	4.33E-16	4.27E-16	4.15E-16	3.99E-16	4.20E-16
4.18E-16	4.40E-16	4.31E-16	4.02E-16	4.27E-16	4.25E-16	3.69E-16	4.18E-16	3.86E-16	4.07E-16	3.95E-16	3.90E-16	3.72E-16	4.05E-16
4.04E-16	4.07E-16	3.91E-16	3.61E-16	3.89E-16	3.97E-16	3.49E-16	4.01E-16	3.55E-16	3.67E-16	3.80E-16	3.77E-16	3.62E-16	3.86E-16
3.93E-16	3.89E-16	3.57E-16	3.44E-16	3.68E-16	3.70E-16	3.50E-16	3.66E-16	3.43E-16	3.27E-16	3.43E-16	3.57E-16	3.36E-16	3.66E-16
3.59E-16	3.61E-16	3.28E-16	3.26E-16	3.55E-16	3.47E-16	3.48E-16	3.33E-16	3.19E-16	3.02E-16	3.14E-16	3.24E-16	3.07E-16	3.33E-16
3.28E-16	3.42E-16	3.00E-16	3.16E-16	3.39E-16	3.34E-16	3.25E-16	3.15E-16	3.17E-16	2.62E-16	2.98E-16	2.98E-16	3.00E-16	3.00E-16
3.02E-16	3.09E-16	2.73E-16	2.95E-16	3.23E-16	3.14E-16	2.96E-16	2.78E-16	2.92E-16	2.52E-16	2.91E-16	2.78E-16	2.81E-16	2.77E-16
2.76E-16	2.84E-16	2.53E-16	2.63E-16	2.85E-16	2.90E-16	2.66E-16	2.25E-16	2.57E-16	2.66E-16	2.63E-16	2.67E-16	2.67E-16	2.56E-16
2.44E-16	2.56E-16	2.43E-16	2.48E-16	2.48E-16	2.65E-16	2.31E-16	2.01E-16	2.15E-16	2.49E-16	2.42E-16	2.29E-16	2.45E-16	2.35E-16
2.08E-16	2.19E-16	2.23E-16	2.32E-16	2.08E-16	2.28E-16	2.07E-16	1.94E-16	2.07E-16	2.23E-16	2.27E-16	1.99E-16	2.17E-16	2.16E-16
1.73E-16	1.91E-16	1.92E-16	1.94E-16	1.89E-16	2.08E-16	1.93E-16	2.14E-16	2.01E-16	2.05E-16	2.09E-16	1.88E-16	2.05E-16	2.03E-16
1.51E-16	1.64E-16	1.79E-16	1.72E-16	1.72E-16	1.84E-16	1.90E-16	1.88E-16	1.84E-16	1.86E-16	1.94E-16	1.97E-16	1.94E-16	1.91E-16
1.29E-16	1.40E-16	1.65E-16	1.63E-16	1.60E-16	1.61E-16	1.75E-16	1.64E-16	1.60E-16	1.63E-16	1.81E-16	1.85E-16	1.74E-16	1.69E-16
1.19E-16	1.36E-16	1.62E-16	1.47E-16	1.39E-16	1.35E-16	1.48E-16	1.47E-16	1.43E-16	1.53E-16	1.68E-16	1.66E-16	1.51E-16	1.44E-16
1.15E-16	1.35E-16	1.49E-16	1.27E-16	1.16E-16	1.20E-16	1.30E-16	1.47E-16	1.43E-16	1.47E-16	1.44E-16	1.36E-16	1.22E-16	1.23E-16
1.07E-16	1.22E-16	1.31E-16	1.02E-16	9.92E-17	1.18E-16	1.22E-16	1.36E-16	1.40E-16	1.37E-16	1.06E-16	1.12E-16	1.11E-16	1.08E-16
9.89E-17	1.03E-16	1.09E-16	7.97E-17	9.04E-17	1.07E-16	1.13E-16	1.17E-16	1.23E-16	1.19E-16	5.64E-17	8.76E-17	1.11E-16	1.10E-16
9.38E-17	8.97E-17	9.30E-17	7.32E-17	8.80E-17	9.93E-17	1.18E-16	1.10E-16	1.10E-16	9.88E-17	8.49E-18	5.09E-17	1.05E-16	9.97E-17
9.09E-17	8.15E-17	8.36E-17	6.67E-17	9.45E-17	1.01E-16	1.10E-16	9.40E-17	9.63E-17	8.69E-17	1.12E-17	6.53E-18	9.57E-17	8.25E-17
8.14E-17	7.61E-17	7.46E-17	6.09E-17	8.23E-17	8.32E-17	9.30E-17	8.07E-17	7.87E-17	7.77E-17	1.08E-17	1.30E-17	8.29E-17	8.46E-17
6.93E-17	6.32E-17	6.79E-17	6.83E-17	6.97E-17	6.59E-17	8.56E-17	6.62E-17	7.09E-17	6.02E-17	7.75E-18	7.77E-18	7.45E-17	8.26E-17
6.64E-17	5.30E-17	5.83E-17	7.12E-17	5.95E-17	6.12E-17	7.88E-17	6.31E-17	6.30E-17	4.84E-17	6.18E-17	1.82E-17	5.90E-17	6.89E-17
6.30E-17	5.00E-17	5.47E-17	6.76E-17	5.10E-17	5.23E-17	6.48E-17	5.83E-17	4.80E-17	4.64E-17	4.89E-17	5.70E-17	4.60E-17	5.78E-17
6.48E-17	5.04E-17	5.77E-17	5.37E-17	4.96E-17	4.71E-17	5.14E-17	4.93E-17	3.19E-17	4.43E-17	4.12E-17	4.98E-17	3.67E-17	4.87E-17
5.76E-17	4.88E-17	5.65E-17	4.22E-17	4.63E-17	4.95E-17	4.62E-17	3.95E-17	2.51E-17	4.24E-17	4.09E-17	4.41E-17	3.22E-17	3.81E-17
4.53E-17	3.92E-17	4.65E-17	4.02E-17	3.87E-17	3.84E-17	3.94E-17	3.55E-17	2.80E-17	3.55E-17	3.06E-17	3.38E-17	2.47E-17	2.93E-17
3.21E-17	3.03E-17	4.33E-17	3.27E-17	3.10E-17	2.80E-17	3.34E-17	3.29E-17	2.58E-17	2.72E-17	2.15E-17	2.66E-17	2.04E-17	2.36E-17
2.31E-17	2.66E-17	3.37E-17	2.61E-17	2.28E-17	1.91E-17	2.19E-17	2.78E-17	1.98E-17	1.86E-17	1.58E-17	2.26E-17	2.05E-17	1.81E-17
2.07E-17	1.96E-17	2.44E-17	2.08E-17	2.11E-17	1.19E-17	1.29E-17	2.02E-17	1.39E-17	8.97E-18	1.31E-17	2.01E-17	1.56E-17	1.63E-17
1.65E-17	1.23E-17	1.84E-17	1.72E-17	1.89E-17	1.05E-17	1.03E-17	1.39E-17	9.59E-18	7.80E-18	1.27E-17	1.31E-17	8.68E-18	1.31E-17
1.24E-17	7.89E-18	1.22E-17	1.31E-17	9.55E-18	7.57E-18	7.98E-18	8.93E-18	6.20E-18	7.83E-18	1.02E-17	5.84E-18	6.19E-18	9.03E-18
9.12E-18	6.07E-18	7.10E-18	7.86E-18	3.63E-18	5.25E-18	5.02E-18	8.11E-18	5.41E-18	5.71E-18	6.36E-18	2.50E-18	3.46E-18	4.20E-18
4.65E-18	6.23E-18	3.06E-18	3.40E-18	1.30E-18	3.97E-18	3.78E-18	8.29E-18	4.88E-18	1.20E-18	3.80E-18	9.32E-19	1.16E-18	1.73E-18
2.27E-18	5.75E-18	8.51E-19	4.37E-19	3.52E-19	2.34E-18	2.23E-18	6.12E-18	3.56E-18	3.61E-21	1.58E-18	3.68E-20	1.20E-23	6.57E-19
3.87E-19	3.29E-18	2.69E-19	2.03E-19	3.06E-20	1.24E-18	1.17E-18	3.02E-18	1.47E-18	1.03E-19	3.65E-19	2.22E-19	1.05E-19	4.35E-20
1.60E-20	9.10E-19	5.92E-21	3.23E-19	5.91E-20	6.10E-20	1.85E-19	5.62E-19	1.68E-19	4.83E-20	2.51E-19	8.32E-20	3.61E-19	1.80E-19
1.22E-19	9.81E-22	3.67E-19	1.52E-19	8.55E-19	4.78E-19	6.55E-20	4.37E-20	9.73E-20	2.36E-19	1.19E-21	4.29E-20	1.20E-18	1.06E-18
7.36E-19	5.98E-19	1.40E-18	6.66E-19	1.19E-18	1.86E-18	1.05E-19	5.38E-20	1.04E-18	1.17E-18	9.81E-19	6.21E-19	3.78E-18	2.17E-18
1.39E-18	3.01E-18	4.83E-18	3.40E-18	2.71E-18	2.29E-18	8.76E-19	1.57E-18	2.09E-18	2.71E-18	3.12E-18	2.33E-18	8.17E-18	5.47E-18
2.08E-18	5.49E-18	7.42E-18	6.74E-18	2.44E-18	3.19E-18	2.48E-18	4.77E-18	2.74E-18	5.01E-18	2.79E-18	4.79E-18	7.85E-18	7.40E-18
5.44E-18	6.97E-18	1.09E-17	6.08E-18	2.16E-18	5.40E-18	4.64E-18	8.51E-18	5.74E-18	7.76E-18	3.61E-18	4.10E-18	5.26E-18	8.66E-18
1.05E-17	8.79E-18	1.43E-17	6.37E-18	4.06E-18	1.03E-17	9.61E-18	1.26E-17	9.78E-18	1.48E-17	9.53E-18	6.71E-18	8.93E-18	1.31E-17
1.75E-17	1.28E-17	1.58E-17	8.88E-18	6.58E-18	1.34E-17	1.44E-17	1.62E-17	1.48E-17	1.86E-17	1.52E-17	1.08E-17	1.66E-17	1.95E-17
2.28E-17	1.98E-17	2.18E-17	1.23E-17	1.02E-17	1.55E-17	1.88E-17	1.89E-17	1.85E-17	2.23E-17	2.01E-17	1.72E-17	2.58E-17	2.31E-17
2.93E-17	2.45E-17	2.93E-17	1.93E-17	1.84E-17	2.16E-17	2.43E-17	2.14E-17	2.40E-17	2.83E-17	2.69E-17	2.82E-17	3.59E-17	2.87E-17
3.09E-17	2.84E-17	3.49E-17	2.82E-17	3.14E-17	3.56E-17	2.84E-17	2.60E-17	3.34E-17	3.97E-17	3.36E-17	3.49E-17	3.48E-17	3.63E-17
3.19E-17	3.77E-17	3.90E-17	4.00E-17	5.06E-17	4.86E-17	4.03E-17	3.76E-17	4.54E-17	4.45E-17	3.91E-17	3.87E-17	3.50E-17	4.46E-17
3.97E-17	4.70E-17	4.99E-17	5.75E-17	6.39E-17	4.95E-17	4.76E-17	5.32E-17	5.29E-17	4.55E-17	4.57E-17	4.27E-17	3.89E-17	5.17E-17
5.54E-17	5.54E-17	6.14E-17	7.15E-17	6.99E-17	5.33E-17	5.91E-17	6.96E-17	6.58E-17	5.39E-17	5.17E-17	4.97E-17	4.49E-17	6.13E-17
7.55E-17	6.79E-17	7.37E-17	6.94E-17	8.10E-17	6.64E-17	7.31E-17	7.96E-17	7.12E-17	6.93E-17	6.48E-17	5.76E-17	5.79E-17	7.81E-17
8.40E-17	8.28E-17	8.47E-17	7.84E-17	8.86E-17	7.68E-17	7.64E-17	8.70E-17	7.64E-17	8.42E-17	7.90E-17	6.62E-17	7.34E-17	8.43E-17
9.30E-17	1.01E-16	9.35E-17	8.69E-17	9.62E-17	7.89E-17	8.00E-17	8.85E-17	8.37E-17	9.52E-17	8.73E-17	8.37E-17	9.00E-17	9.71E-17
1.09E-16	1.09E-16	1.04E-16	9.91E-17	9.94E-17	8.78E-17	8.76E-17	9.60E-17	9.23E-17	1.08E-16	1.01E-16	1.02E-16	1.05E-16	1.15E-16
1.22E-16	1.15E-16	1.12E-16	1.12E-16	1.06E-16	1.05E-16	9.85E-17	1.10E-16	1.13E-16	1.17E-16	1.18E-16	1.13E-16	1.24E-16	1.29E-16
1.25E-16	1.19E-16	1.24E-16	1.45E-16	1.17E-16	1.20E-16	1.11E-16	1.19E-16	1.33E-16	1.24E-16	1.43E-16	1.18E-16	1.35E-16	1.41E-16
1.33E-16	1.30E-16	1.45E-16	1.72E-16	1.36E-16	1.41E-16	1.37E-16	1.33E-16	1.48E-16	1.49E-16	1.67E-16	1.48E-16	1.44E-16	1.55E-16
1.46E-16	1.51E-16	1.54E-16	1.88E-16	1.49E-16	1.49E-16	1.46E-16	1.63E-16	1.77E-16	1.77E-16	1.81E-16	1.77E-16	1.63E-16	1.59E-16
1.64E-16	1.85E-16	1.78E-16	1.82E-16	1.66E-16	1.66E-16	1.66E-16	2.04E-16	2.17E-16	1.99E-16	2.06E-16	2.07E-16	1.92E-16	1.65E-16
1.95E-16	2.16E-16	2.01E-16	1.88E-16	1.86E-16	1.83E-16	1.92E-16	2.40E-16	2.48E-16	2.12E-16	2.24E-16	2.27E-16	2.22E-16	1.95E-16
2.38E-16	2.41E-16	2.29E-16	2.18E-16	2.19E-16	2.14E-16	2.28E-16	2.74E-16	2.70E-16	2.30E-16	2.35E-16	2.45E-16	2.53E-16	2.35E-16
2.79E-16	2.70E-16	2.55E-16	2.49E-16	2.53E-16	2.46E-16	2.62E-16	2.94E-16	2.85E-16	2.54E-16	2.57E-16	2.60E-16	2.62E-16	2.48E-16
3.09E-16	3.03E-16	2.75E-16	2.72E-16	2.79E-16	2.75E-16	2.98E-16	2.87E-16	2.94E-16	2.79E-16	2.71E-16	2.94E-16	2.74E-16	2.78E-16
3.38E-16	3.19E-16	3.01E-16	3.05E-16	3.17E-16	3.01E-16	3.28E-16	3.09E-16	3.05E-16	2.91E-16	2.86E-16	3.19E-16	2.91E-16	3.20E-16
3.62E-16	3.49E-16	3.40E-16	3.44E-16	3.55E-16	3.37E-16	3.58E-16	3.33E-16	3.21E-16	3.20E-16	2.92E-16	3.54E-16	3.34E-16	3.37E-16
3.96E-16	3.96E-16	3.92E-16	3.82E-16	3.81E-16	3.81E-16	3.83E-16	3.51E-16	3.46E-16	3.51E-16	2.97E-16	3.80E-16	3.67E-16	3.55E-16
4.41E-16	4.56E-16	4.44E-16	4.29E-16	4.19E-16	4.11E-16	4.03E-16	3.75E-16	3.76E-16	3.91E-16	3.37E-16	4.10E-16	3.83E-16	3.67E-16
4.88E-16	5.08E-16	4.69E-16	4.73E-16	4.46E-16	4.35E-16	4.24E-16	4.16E-16	4.09E-16	4.26E-16	3.85E-16	4.21E-16	4.09E-16	3.91E-16
5.11E-16	5.28E-16	5.07E-16	5.16E-16	4.91E-16	4.74E-16	4.65E-16	4.75E-16	4.74E-16	4.62E-16	4.24E-16	4.38E-16	4.37E-16	4.30E-16
5.29E-16	5.59E-16	5.53E-16	5.62E-16	5.63E-16	5.35E-16	5.27E-16	5.42E-16	5.41E-16	5.19E-16	4.70E-16	4.70E-16	4.74E-16	4.85E-16
5.67E-16	6.07E-16	6.22E-16	6.11E-16	6.26E-16	5.85E-16	5.86E-16	5.68E-16	5.68E-16	5.78E-16	5.37E-16	5.23E-16	5.24E-16	5.23E-16
6.23E-16	6.52E-16	6.90E-16	6.70E-16	6.74E-16	6.44E-16	6.39E-16	6.20E-16	5.82E-16	6.24E-16	5.98E-16	5.90E-16	6.08E-16	5.83E-16
6.86E-16	7.08E-16	7.54E-16	7.32E-16	7.25E-16	6.91E-16	7.14E-16	6.72E-16	6.23E-16	6.68E-16	6.71E-16	6.63E-16	6.98E-16	6.37E-16
7.59E-16	7.65E-16	7.82E-16	7.61E-16	7.67E-16	7.30E-16	7.82E-16	7.27E-16	6.71E-16	7.16E-16	7.29E-16	7.13E-16	7.36E-16	6.86E-16
8.47E-16	8.30E-16	8.18E-16	8.19E-16	8.21E-16	7.85E-16	8.14E-16	7.82E-16	7.09E-16	7.59E-16	7.59E-16	7.46E-16	7.68E-16	7.31E-16
8.81E-16	9.11E-16	8.86E-16	9.01E-16	8.90E-16	8.54E-16	8.29E-16	8.33E-16	7.67E-16	8.09E-16	7.91E-16	8.06E-16	7.88E-16	8.10E-16
9.39E-16	1.01E-15	9.68E-16	9.57E-16	9.44E-16	9.26E-16	8.78E-16	8.98E-16	8.56E-16	8.62E-16	8.34E-16	8.49E-16	8.26E-16	8.60E-16
1.01E-15	1.12E-15	1.07E-15	1.03E-15	1.02E-15	9.93E-16	9.58E-16	9.68E-16	9.28E-16	9.13E-16	8.92E-16	8.92E-16	8.85E-16	9.18E-16
1.08E-15	1.12E-15	1.16E-15	1.13E-15	1.11E-15	1.09E-15	1.02E-15	1.04E-15	9.88E-16	9.70E-16	9.51E-16	9.65E-16	9.57E-16	9.73E-16
1.18E-15	1.19E-15	1.24E-15	1.23E-15	1.18E-15	1.18E-15	1.10E-15	1.09E-15	1.06E-15	1.05E-15	1.02E-15	1.03E-15	1.02E-15	1.04E-15
1.29E-15	1.25E-15	1.31E-15	1.33E-15	1.27E-15	1.26E-15	1.17E-15	1.17E-15	1.15E-15	1.16E-15	1.09E-15	1.09E-15	1.08E-15	1.10E-15
1.40E-15	1.31E-15	1.38E-15	1.41E-15	1.32E-15	1.31E-15	1.26E-15	1.23E-15	1.23E-15	1.25E-15	1.17E-15	1.16E-15	1.15E-15	1.16E-15
1.50E-15	1.38E-15	1.43E-15	1.44E-15	1.37E-15	1.35E-15	1.32E-15	1.28E-15	1.35E-15	1.34E-15	1.25E-15	1.24E-15	1.20E-15	1.22E-15
1.55E-15	1.51E-15	1.53E-15	1.47E-15	1.43E-15	1.42E-15	1.40E-15	1.34E-15	1.46E-15	1.46E-15	1.35E-15	1.32E-15	1.30E-15	1.32E-15
1.65E-15	1.64E-15	1.65E-15	1.56E-15	1.53E-15	1.52E-15	1.49E-15	1.45E-15	1.57E-15	1.54E-15	1.45E-15	1.41E-15	1.44E-15	1.44E-15

1.74E-15	1.75E-15	1.75E-15	1.70E-15	1.63E-15	1.65E-15	1.62E-15	1.58E-15	1.70E-15	1.61E-15	1.56E-15	1.52E-15	1.57E-15	1.53E-15
1.82E-15	1.86E-15	1.79E-15	1.83E-15	1.74E-15	1.78E-15	1.73E-15	1.71E-15	1.80E-15	1.71E-15	1.64E-15	1.62E-15	1.65E-15	1.63E-15
1.93E-15	1.94E-15	1.86E-15	1.87E-15	1.86E-15	1.91E-15	1.84E-15	1.83E-15	1.88E-15	1.81E-15	1.73E-15	1.72E-15	1.73E-15	1.68E-15
2.08E-15	2.07E-15	1.96E-15	1.92E-15	1.97E-15	2.03E-15	1.91E-15	1.95E-15	1.97E-15	1.89E-15	1.83E-15	1.81E-15	1.84E-15	1.80E-15
2.25E-15	2.21E-15	2.09E-15	2.02E-15	2.11E-15	2.11E-15	2.03E-15	2.07E-15	2.04E-15	2.00E-15	1.95E-15	1.88E-15	1.90E-15	1.91E-15
2.43E-15	2.41E-15	2.23E-15	2.17E-15	2.24E-15	2.20E-15	2.19E-15	2.18E-15	2.11E-15	2.10E-15	2.11E-15	2.00E-15	2.02E-15	2.01E-15
2.55E-15	2.60E-15	2.35E-15	2.32E-15	2.37E-15	2.32E-15	2.35E-15	2.25E-15	2.21E-15	2.19E-15	2.26E-15	2.11E-15	2.15E-15	2.10E-15
2.66E-15	2.65E-15	2.49E-15	2.45E-15	2.48E-15	2.43E-15	2.49E-15	2.35E-15	2.34E-15	2.32E-15	2.28E-15	2.23E-15	2.28E-15	2.25E-15
2.68E-15	2.63E-15	2.62E-15	2.60E-15	2.61E-15	2.54E-15	2.57E-15	2.48E-15	2.51E-15	2.43E-15	2.42E-15	2.38E-15	2.41E-15	2.38E-15
2.76E-15	2.71E-15	2.72E-15	2.76E-15	2.75E-15	2.69E-15	2.69E-15	2.62E-15	2.64E-15	2.58E-15	2.54E-15	2.55E-15	2.57E-15	2.55E-15
2.93E-15	2.88E-15	2.86E-15	2.89E-15	2.88E-15	2.88E-15	2.86E-15	2.80E-15	2.80E-15	2.78E-15	2.69E-15	2.72E-15	2.70E-15	2.72E-15
7.84E-14	7.89E-14	7.80E-14	7.74E-14	7.68E-14	7.58E-14	7.51E-14	7.45E-14	7.45E-14	7.33E-14	7.16E-14	7.11E-14	7.11E-14	7.11E-14

-3.10E-08	-3.15E-08	-3.16E-08	-3.11E-08	-3.11E-08	-3.15E-08	-3.14E-08	-3.10E-08	-3.06E-08	-2.92E-08	-2.96E-08	-3.03E-08	-3.01E-08	-2.98E-08
-3.07E-08	-3.09E-08	-3.16E-08	-3.15E-08	-3.17E-08	-3.20E-08	-3.09E-08	-3.11E-08	-3.07E-08	-2.94E-08	-3.00E-08	-3.03E-08	-3.02E-08	-3.00E-08
-3.12E-08	-3.06E-08	-3.12E-08	-3.15E-08	-3.21E-08	-3.17E-08	-3.04E-08	-3.12E-08	-3.10E-08	-2.91E-08	-2.96E-08	-3.00E-08	-2.99E-08	-2.98E-08
-3.15E-08	-3.08E-08	-3.09E-08	-3.14E-08	-3.19E-08	-3.16E-08	-3.08E-08	-3.16E-08	-3.09E-08	-2.90E-08	-2.93E-08	-2.92E-08	-2.94E-08	-2.93E-08
-3.13E-08	-3.11E-08	-2.99E-08	-3.09E-08	-3.16E-08	-3.11E-08	-3.14E-08	-3.15E-08	-3.10E-08	-2.87E-08	-2.89E-08	-2.87E-08	-2.90E-08	-2.89E-08
-3.10E-08	-3.08E-08	-2.94E-08	-3.05E-08	-3.13E-08	-3.07E-08	-3.10E-08	-3.14E-08	-3.08E-08	-2.84E-08	-2.88E-08	-2.87E-08	-2.91E-08	-2.88E-08
-3.05E-08	-3.02E-08	-2.93E-08	-3.02E-08	-3.08E-08	-3.04E-08	-3.07E-08	-3.11E-08	-3.09E-08	-2.87E-08	-2.88E-08	-2.88E-08	-2.91E-08	-2.87E-08
-3.05E-08	-2.98E-08	-2.99E-08	-3.01E-08	-3.04E-08	-3.01E-08	-3.05E-08	-3.04E-08	-3.04E-08	-2.91E-08	-2.91E-08	-2.86E-08	-2.89E-08	-2.81E-08
-3.02E-08	-2.93E-08	-3.04E-08	-3.08E-08	-3.00E-08	-3.01E-08	-3.04E-08	-2.96E-08	-3.03E-08	-2.89E-08	-2.88E-08	-2.81E-08	-2.80E-08	-2.79E-08
-3.00E-08	-2.91E-08	-3.04E-08	-3.07E-08	-2.97E-08	-2.98E-08	-3.00E-08	-2.92E-08	-3.00E-08	-2.84E-08	-2.82E-08	-2.74E-08	-2.77E-08	-2.75E-08
-2.95E-08	-2.90E-08	-2.97E-08	-3.01E-08	-2.93E-08	-2.91E-08	-2.94E-08	-2.90E-08	-2.94E-08	-2.80E-08	-2.79E-08	-2.71E-08	-2.77E-08	-2.70E-08
-2.88E-08	-2.88E-08	-2.92E-08	-2.94E-08	-2.86E-08	-2.86E-08	-2.84E-08	-2.86E-08	-2.84E-08	-2.76E-08	-2.76E-08	-2.71E-08	-2.74E-08	-2.67E-08
-2.81E-08	-2.83E-08	-2.87E-08	-2.90E-08	-2.84E-08	-2.83E-08	-2.76E-08	-2.77E-08	-2.75E-08	-2.70E-08	-2.75E-08	-2.67E-08	-2.71E-08	-2.65E-08
-2.74E-08	-2.76E-08	-2.81E-08	-2.86E-08	-2.82E-08	-2.83E-08	-2.67E-08	-2.70E-08	-2.65E-08	-2.64E-08	-2.74E-08	-2.62E-08	-2.68E-08	-2.59E-08
-2.75E-08	-2.77E-08	-2.78E-08	-2.81E-08	-2.76E-08	-2.76E-08	-2.66E-08	-2.67E-08	-2.59E-08	-2.53E-08	-2.69E-08	-2.61E-08	-2.64E-08	-2.55E-08
-2.74E-08	-2.79E-08	-2.75E-08	-2.75E-08	-2.68E-08	-2.68E-08	-2.59E-08	-2.63E-08	-2.63E-08	-2.53E-08	-2.65E-08	-2.57E-08	-2.61E-08	-2.55E-08
-2.72E-08	-2.75E-08	-2.69E-08	-2.71E-08	-2.68E-08	-2.65E-08	-2.53E-08	-2.62E-08	-2.65E-08	-2.55E-08	-2.59E-08	-2.56E-08	-2.54E-08	-2.52E-08
-2.68E-08	-2.71E-08	-2.66E-08	-2.69E-08	-2.66E-08	-2.66E-08	-2.52E-08	-2.60E-08	-2.61E-08	-2.54E-08	-2.55E-08	-2.46E-08	-2.44E-08	-2.47E-08
-2.61E-08	-2.69E-08	-2.60E-08	-2.65E-08	-2.60E-08	-2.60E-08	-2.53E-08	-2.58E-08	-2.58E-08	-2.51E-08	-2.56E-08	-2.39E-08	-2.41E-08	-2.43E-08
-2.58E-08	-2.66E-08	-2.60E-08	-2.58E-08	-2.52E-08	-2.52E-08	-2.51E-08	-2.55E-08	-2.56E-08	-2.45E-08	-2.52E-08	-2.41E-08	-2.40E-08	-2.41E-08
-2.58E-08	-2.67E-08	-2.59E-08	-2.51E-08	-2.45E-08	-2.45E-08	-2.46E-08	-2.52E-08	-2.52E-08	-2.49E-08	-2.47E-08	-2.44E-08	-2.38E-08	-2.37E-08
-2.58E-08	-2.65E-08	-2.56E-08	-2.48E-08	-2.41E-08	-2.36E-08	-2.40E-08	-2.47E-08	-2.40E-08	-2.48E-08	-2.44E-08	-2.43E-08	-2.34E-08	-2.33E-08
-2.56E-08	-2.65E-08	-2.48E-08	-2.43E-08	-2.35E-08	-2.28E-08	-2.32E-08	-2.39E-08	-2.30E-08	-2.40E-08	-2.38E-08	-2.35E-08	-2.26E-08	-2.29E-08
-2.53E-08	-2.59E-08	-2.45E-08	-2.40E-08	-2.32E-08	-2.22E-08	-2.24E-08	-2.32E-08	-2.21E-08	-2.33E-08	-2.34E-08	-2.25E-08	-2.19E-08	-2.22E-08
-2.47E-08	-2.48E-08	-2.43E-08	-2.39E-08	-2.30E-08	-2.23E-08	-2.19E-08	-2.29E-08	-2.17E-08	-2.33E-08	-2.26E-08	-2.14E-08	-2.13E-08	-2.14E-08
-2.38E-08	-2.41E-08	-2.36E-08	-2.34E-08	-2.28E-08	-2.23E-08	-2.23E-08	-2.27E-08	-2.21E-08	-2.27E-08	-2.20E-08	-2.11E-08	-2.15E-08	-2.12E-08
-2.36E-08	-2.36E-08	-2.27E-08	-2.27E-08	-2.22E-08	-2.19E-08	-2.23E-08	-2.22E-08	-2.18E-08	-2.21E-08	-2.13E-08	-2.09E-08	-2.14E-08	-2.08E-08
-2.37E-08	-2.31E-08	-2.19E-08	-2.19E-08	-2.14E-08	-2.18E-08	-2.21E-08	-2.20E-08	-2.14E-08	-2.13E-08	-2.06E-08	-2.06E-08	-2.09E-08	-2.01E-08
-2.32E-08	-2.26E-08	-2.14E-08	-2.17E-08	-2.08E-08	-2.17E-08	-2.14E-08	-2.16E-08	-2.11E-08	-2.04E-08	-2.00E-08	-2.02E-08	-1.97E-08	-1.89E-08
-2.26E-08	-2.19E-08	-2.06E-08	-2.16E-08	-2.06E-08	-2.11E-08	-2.07E-08	-2.13E-08	-2.06E-08	-1.97E-08	-1.89E-08	-2.02E-08	-1.85E-08	-1.81E-08
-2.20E-08	-2.12E-08	-2.09E-08	-2.10E-08	-2.00E-08	-2.01E-08	-2.07E-08	-2.04E-08	-2.01E-08	-1.93E-08	-1.81E-08	-1.96E-08	-1.83E-08	-1.76E-08
-2.14E-08	-2.02E-08	-2.07E-08	-2.03E-08	-1.94E-08	-1.92E-08	-2.02E-08	-1.95E-08	-1.96E-08	-1.91E-08	-1.80E-08	-1.92E-08	-1.77E-08	-1.81E-08
-2.09E-08	-1.99E-08	-2.02E-08	-1.98E-08	-1.87E-08	-1.84E-08	-1.95E-08	-1.84E-08	-1.89E-08	-1.86E-08	-1.84E-08	-1.87E-08	-1.66E-08	-1.75E-08
-2.04E-08	-1.95E-08	-1.95E-08	-1.92E-08	-1.84E-08	-1.85E-08	-1.87E-08	-1.78E-08	-1.84E-08	-1.80E-08	-1.80E-08	-1.82E-08	-1.66E-08	-1.67E-08
-2.03E-08	-1.91E-08	-1.90E-08	-1.85E-08	-1.85E-08	-1.81E-08	-1.83E-08	-1.72E-08	-1.80E-08	-1.75E-08	-1.75E-08	-1.77E-08	-1.66E-08	-1.66E-08
-1.95E-08	-1.86E-08	-1.84E-08	-1.81E-08	-1.84E-08	-1.78E-08	-1.75E-08	-1.67E-08	-1.74E-08	-1.72E-08	-1.68E-08	-1.71E-08	-1.65E-08	-1.68E-08
-1.90E-08	-1.80E-08	-1.79E-08	-1.77E-08	-1.78E-08	-1.72E-08	-1.64E-08	-1.60E-08	-1.66E-08	-1.64E-08	-1.58E-08	-1.62E-08	-1.63E-08	-1.63E-08
-1.80E-08	-1.74E-08	-1.77E-08	-1.73E-08	-1.71E-08	-1.66E-08	-1.58E-08	-1.54E-08	-1.57E-08	-1.57E-08	-1.50E-08	-1.56E-08	-1.56E-08	-1.54E-08
-1.71E-08	-1.70E-08	-1.71E-08	-1.69E-08	-1.63E-08	-1.60E-08	-1.54E-08	-1.53E-08	-1.51E-08	-1.50E-08	-1.47E-08	-1.53E-08	-1.50E-08	-1.50E-08
-1.63E-08	-1.64E-08	-1.63E-08	-1.63E-08	-1.58E-08	-1.56E-08	-1.48E-08	-1.51E-08	-1.51E-08	-1.48E-08	-1.48E-08	-1.48E-08	-1.46E-08	-1.44E-08
-1.56E-08	-1.58E-08	-1.55E-08	-1.57E-08	-1.52E-08	-1.52E-08	-1.48E-08	-1.47E-08	-1.50E-08	-1.46E-08	-1.41E-08	-1.40E-08	-1.38E-08	-1.36E-08
-1.51E-08	-1.49E-08	-1.44E-08	-1.47E-08	-1.45E-08	-1.46E-08	-1.49E-08	-1.42E-08	-1.48E-08	-1.41E-08	-1.33E-08	-1.27E-08	-1.29E-08	-1.26E-08
-1.42E-08	-1.41E-08	-1.39E-08	-1.39E-08	-1.39E-08	-1.42E-08	-1.46E-08	-1.44E-08	-1.44E-08	-1.32E-08	-1.26E-08	-1.20E-08	-1.20E-08	-1.22E-08
-1.36E-08	-1.39E-08	-1.40E-08	-1.37E-08	-1.37E-08	-1.39E-08	-1.39E-08	-1.38E-08	-1.42E-08	-1.30E-08	-1.29E-08	-1.26E-08	-1.17E-08	-1.24E-08
-1.34E-08	-1.36E-08	-1.35E-08	-1.36E-08	-1.33E-08	-1.34E-08	-1.32E-08	-1.32E-08	-1.36E-08	-1.30E-08	-1.32E-08	-1.31E-08	-1.25E-08	-1.30E-08
-1.27E-08	-1.29E-08	-1.29E-08	-1.29E-08	-1.27E-08	-1.26E-08	-1.25E-08	-1.27E-08	-1.27E-08	-1.28E-08	-1.31E-08	-1.32E-08	-1.31E-08	-1.28E-08
-1.22E-08	-1.22E-08	-1.21E-08	-1.20E-08	-1.19E-08	-1.16E-08	-1.22E-08	-1.23E-08	-1.23E-08	-1.24E-08	-1.28E-08	-1.23E-08	-1.28E-08	-1.24E-08
-1.17E-08	-1.13E-08	-1.07E-08	-1.14E-08	-1.15E-08	-1.12E-08	-1.14E-08	-1.15E-08	-1.19E-08	-1.22E-08	-1.25E-08	-1.14E-08	-1.20E-08	-1.19E-08
-1.12E-08	-1.07E-08	-9.88E-09	-1.06E-08	-1.07E-08	-1.12E-08	-1.05E-08	-1.06E-08	-1.13E-08	-1.17E-08	-1.22E-08	-1.07E-08	-1.12E-08	-1.11E-08
-1.09E-08	-1.03E-08	-9.73E-09	-9.31E-09	-9.96E-09	-1.07E-08	-1.02E-08	-1.05E-08	-1.10E-08	-1.15E-08	-1.18E-08	-1.04E-08	-1.06E-08	-1.05E-08
-1.08E-08	-1.02E-08	-9.49E-09	-8.64E-09	-9.30E-09	-1.07E-08	-1.00E-08	-1.06E-08	-1.06E-08	-1.08E-08	-1.09E-08	-9.95E-09	-1.02E-08	-1.02E-08
-1.02E-08	-9.92E-09	-8.40E-09	-7.95E-09	-8.99E-09	-1.02E-08	-9.27E-09	-1.00E-08	-9.65E-09	-1.04E-08	-1.02E-08	-9.90E-09	-9.70E-09	-9.52E-09
-9.52E-09	-8.84E-09	-7.13E-09	-7.68E-09	-9.14E-09	-9.18E-09	-8.49E-09	-9.28E-09	-9.16E-09	-1.00E-08	-9.23E-09	-9.32E-09	-9.18E-09	-8.43E-09
-8.95E-09	-7.95E-09	-6.75E-09	-7.95E-09	-8.85E-09	-8.56E-09	-8.10E-09	-8.78E-09	-9.05E-09	-9.20E-09	-8.57E-09	-8.62E-09	-8.49E-09	-8.50E-09
-8.37E-09	-8.06E-09	-7.20E-09	-7.27E-09	-8.24E-09	-8.29E-09	-8.49E-09	-8.17E-09	-8.13E-09	-8.60E-09	-7.90E-09	-7.78E-09	-8.93E-09	-8.22E-09
-7.98E-09	-7.99E-09	-7.48E-09	-6.38E-09	-7.48E-09	-7.71E-09	-8.03E-09	-7.03E-09	-7.23E-09	-7.81E-09	-6.79E-09	-6.79E-09	-8.24E-09	-7.84E-09
-7.41E-09	-7.58E-09	-7.45E-09	-6.54E-09	-6.76E-09	-6.99E-09	-7.21E-09	-6.29E-09	-6.56E-09	-7.28E-09	-5.52E-09	-5.76E-09	-7.26E-09	-7.47E-09
-6.70E-09	-6.60E-09	-6.54E-09	-5.79E-09	-5.83E-09	-6.85E-09	-6.85E-09	-5.70E-09	-5.70E-09	-6.48E-09	-5.80E-09	-5.23E-09	-6.18E-09	-6.80E-09
-5.75E-09	-5.58E-09	-5.23E-09	-5.20E-09	-5.48E-09	-6.10E-09	-6.51E-09	-5.49E-09	-5.40E-09	-5.80E-09	-5.54E-09	-4.48E-09	-5.07E-09	-6.06E-09
-5.01E-09	-5.12E-09	-4.33E-09	-4.63E-09	-5.10E-09	-5.47E-09	-5.52E-09	-5.24E-09	-4.53E-09	-4.80E-09	-4.63E-09	-2.94E-09	-3.77E-09	-5.23E-09
-4.33E-09	-4.97E-09	-4.51E-09	-3.72E-09	-4.64E-09	-4.81E-09	-4.73E-09	-5.45E-09	-3.62E-09	-3.84E-09	-3.62E-09	-2.20E-09	-3.10E-09	-4.11E-09
-3.70E-09	-4.51E-09	-4.99E-09	-3.40E-09	-4.22E-09	-4.36E-09	-4.34E-09	-4.77E-09	-3.71E-09	-3.85E-09	-3.47E-09	-2.31E-09	-2.96E-09	-3.38E-09
-3.12E-09	-3.86E-09	-4.18E-09	-3.29E-09	-3.80E-09	-3.67E-09	-4.03E-09	-4.03E-09	-3.64E-09	-3.81E-09	-3.61E-09	-3.29E-09	-3.48E-09	-3.79E-09
-2.10E-09	-2.94E-09	-3.25E-09	-3.61E-09	-3.33E-09	-2.97E-09	-3.64E-09	-3.08E-09	-2.71E-09	-3.08E-09	-2.94E-09	-2.93E-09	-2.96E-09	-3.90E-09
-1.15E-09	-2.25E-09	-2.46E-09	-3.57E-09	-3.06E-09	-2.13E-09	-3.02E-09	-2.34E-09	-1.57E-09	-2.40E-09	-2.15E-09	-2.52E-09	-2.19E-09	-3.27E-09
-9.14E-10	-1.43E-09	-1.77E-09	-2.60E-09	-2.35E-09	-1.43E-09	-2.38E-09	-1.64E-09	-6.11E-10	-1.41E-09	-1.57E-09	-1.92E-09	-1.35E-09	-2.53E-09
-8.03E-10	-7.40E-10	-1.20E-09	-1.45E-09	-1.32E-09	-8.95E-10	-1.57E-09	-1.28E-09	-6.95E-10	-1.03E-09	-1.40E-09	-1.43E-09	-1.23E-09	-1.21E-09
-1.90E-10	-2.52E-11	-5.79E-10	-4.97E-10	-4.85E-10	-2.47E-10	-7.11E-10	-6.08E-10	-8.19E-11	-4.17E-10	-6.62E-10	-8.66E-10	-6.91E-10	-1.36E-10
7.72E-10	8.92E-10	3.14E-10	3.41E-10	2.64E-10	6.29E-10	1.55E-11	3.95E-10	7.24E-10	1.09E-10	1.26E-10	-1.90E-11	4.84E-10	5.08E-10
2.14E-09	1.72E-09	1.31E-09	1.61E-09	1.24E-09	2.04E-09	5.09E-10	1.54E-09	1.49E-09	9.33E-10	8.14E-10	1.26E-09	1.49E-09	9.37E-10
2.47E-09	2.47E-09	2.18E-09	3.26E-09	2.63E-09	3.18E-09	1.15E-09	1.93E-09	2.28E-09	1.82E-09	2.06E-09	2.12E-09	2.06E-09	1.34E-09
2.73E-09	2.90E-09	3.35E-09	4.52E-09	3.37E-09	2.81E-09	1.88E-09	2.54E-09	2.59E-09	2.47E-09	2.99E-09	2.36E-09	1.90E-09	1.45E-09
2.78E-09	3.59E-09	4.99E-09	5.39E-09	4.00E-09	2.73E-09	3.00E-09	3.04E-09	2.53E-09	2.87E-09	3.30E-09	2.39E-09	2.18E-09	1.55E-09
2.89E-09	4.16E-09	5.30E-09	5.43E-09	4.74E-09	3.64E-09	3.98E-09	3.46E-09	2.43E-09	3.20E-09	3.44E-09	2.61E-09	2.52E-09	2.20E-09
3.50E-09	4.61E-09	4.60E-09	4.42E-09	5.16E-09	4.32E-09	4.28E-09	3.77E-09	2.80E-09	3.46E-09	3.33E-09	3.02E-09	3.49E-09	3.22E-09
3.65E-09	4.40E-09	4.57E-09	4.38E-09	5.53E-09	4.84E-09	4.40E-09	4.31E-09	3.27E-09	4.16E-09	3.95E-09	3.74E-09	3.88E-09	3.56E-09
4.11E-09	4.79E-09	4.76E-09	4.58E-09	5.91E-09	5.40E-09	4.94E-09	5.11E-09	4.23E-09	4.67E-09	4.43E-09	4.35E-09	4.26E-09	3.92E-09
4.60E-09	5.83E-09	5.13E-09	5.30E-09	6.28E-09	6.02E-09	5.83E-09	5.71E-09	5.43E-09	5.34E-09	4.72E-09	4.61E-09	4.55E-09	4.31E-09

5.54E-09	6.44E-09	5.69E-09	5.71E-09	6.62E-09	6.65E-09	6.60E-09	6.23E-09	6.37E-09	5.58E-09	5.18E-09	4.91E-09	5.28E-09	5.17E-09
6.88E-09	6.92E-09	6.12E-09	6.07E-09	7.09E-09	6.89E-09	7.04E-09	6.14E-09	6.36E-09	5.93E-09	5.61E-09	5.33E-09	5.93E-09	6.08E-09
8.05E-09	7.66E-09	6.79E-09	6.33E-09	7.13E-09	7.35E-09	7.52E-09	6.58E-09	6.51E-09	6.64E-09	6.14E-09	6.21E-09	6.96E-09	7.14E-09
8.40E-09	8.35E-09	7.65E-09	7.25E-09	7.73E-09	7.60E-09	7.72E-09	7.22E-09	6.88E-09	7.46E-09	6.92E-09	6.95E-09	7.90E-09	7.80E-09
8.97E-09	9.29E-09	8.82E-09	8.14E-09	8.09E-09	8.49E-09	8.39E-09	7.61E-09	7.68E-09	8.57E-09	7.70E-09	7.58E-09	8.30E-09	8.67E-09
9.60E-09	9.81E-09	9.24E-09	8.79E-09	8.54E-09	8.68E-09	9.11E-09	8.38E-09	8.38E-09	9.33E-09	8.62E-09	8.39E-09	9.12E-09	8.98E-09
9.78E-09	1.03E-08	9.91E-09	9.27E-09	9.03E-09	9.22E-09	9.76E-09	9.54E-09	9.11E-09	9.59E-09	8.96E-09	9.18E-09	9.61E-09	8.88E-09
1.04E-08	1.08E-08	1.06E-08	1.00E-08	9.86E-09	9.95E-09	1.03E-08	1.01E-08	9.96E-09	1.04E-08	9.27E-09	9.94E-09	9.44E-09	9.31E-09
1.15E-08	1.08E-08	1.11E-08	1.05E-08	1.07E-08	1.09E-08	1.09E-08	1.03E-08	1.07E-08	1.13E-08	1.02E-08	1.09E-08	1.01E-08	1.02E-08
1.21E-08	1.13E-08	1.15E-08	1.14E-08	1.17E-08	1.17E-08	1.13E-08	1.07E-08	1.13E-08	1.20E-08	1.07E-08	1.13E-08	1.09E-08	1.12E-08
1.21E-08	1.17E-08	1.20E-08	1.18E-08	1.19E-08	1.19E-08	1.21E-08	1.17E-08	1.20E-08	1.20E-08	1.13E-08	1.12E-08	1.17E-08	1.19E-08
1.24E-08	1.25E-08	1.28E-08	1.23E-08	1.24E-08	1.26E-08	1.29E-08	1.28E-08	1.23E-08	1.18E-08	1.18E-08	1.16E-08	1.23E-08	1.26E-08
1.28E-08	1.33E-08	1.40E-08	1.33E-08	1.32E-08	1.34E-08	1.33E-08	1.31E-08	1.27E-08	1.21E-08	1.28E-08	1.25E-08	1.28E-08	1.33E-08
1.38E-08	1.38E-08	1.45E-08	1.41E-08	1.39E-08	1.40E-08	1.34E-08	1.35E-08	1.30E-08	1.28E-08	1.35E-08	1.36E-08	1.37E-08	1.44E-08
1.51E-08	1.46E-08	1.47E-08	1.45E-08	1.43E-08	1.46E-08	1.36E-08	1.41E-08	1.38E-08	1.39E-08	1.41E-08	1.44E-08	1.43E-08	1.55E-08
1.54E-08	1.52E-08	1.51E-08	1.51E-08	1.48E-08	1.54E-08	1.44E-08	1.52E-08	1.49E-08	1.54E-08	1.45E-08	1.51E-08	1.49E-08	1.61E-08
1.57E-08	1.62E-08	1.57E-08	1.52E-08	1.54E-08	1.60E-08	1.53E-08	1.60E-08	1.60E-08	1.62E-08	1.56E-08	1.61E-08	1.57E-08	1.67E-08
1.67E-08	1.76E-08	1.66E-08	1.59E-08	1.59E-08	1.67E-08	1.65E-08	1.66E-08	1.73E-08	1.65E-08	1.66E-08	1.66E-08	1.66E-08	1.70E-08
1.76E-08	1.84E-08	1.73E-08	1.70E-08	1.70E-08	1.73E-08	1.75E-08	1.75E-08	1.78E-08	1.74E-08	1.70E-08	1.73E-08	1.76E-08	1.72E-08
1.83E-08	1.86E-08	1.77E-08	1.80E-08	1.75E-08	1.81E-08	1.80E-08	1.78E-08	1.81E-08	1.82E-08	1.77E-08	1.83E-08	1.85E-08	1.84E-08
1.90E-08	1.91E-08	1.84E-08	1.91E-08	1.83E-08	1.87E-08	1.84E-08	1.85E-08	1.90E-08	1.89E-08	1.87E-08	1.88E-08	1.90E-08	1.98E-08
2.01E-08	1.99E-08	1.93E-08	2.02E-08	1.94E-08	1.99E-08	1.93E-08	1.95E-08	2.01E-08	1.92E-08	1.95E-08	1.92E-08	1.92E-08	2.04E-08
2.16E-08	2.06E-08	2.05E-08	2.12E-08	2.08E-08	2.09E-08	2.05E-08	2.04E-08	2.14E-08	1.96E-08	2.04E-08	2.01E-08	2.02E-08	2.02E-08
2.26E-08	2.17E-08	2.16E-08	2.21E-08	2.18E-08	2.18E-08	2.19E-08	2.12E-08	2.24E-08	2.09E-08	2.16E-08	2.10E-08	2.11E-08	1.99E-08
2.32E-08	2.30E-08	2.30E-08	2.28E-08	2.28E-08	2.27E-08	2.33E-08	2.23E-08	2.32E-08	2.23E-08	2.27E-08	2.24E-08	2.17E-08	2.05E-08
2.39E-08	2.41E-08	2.40E-08	2.35E-08	2.36E-08	2.36E-08	2.41E-08	2.33E-08	2.44E-08	2.31E-08	2.35E-08	2.30E-08	2.26E-08	2.16E-08
2.45E-08	2.50E-08	2.50E-08	2.42E-08	2.44E-08	2.43E-08	2.42E-08	2.48E-08	2.46E-08	2.38E-08	2.37E-08	2.30E-08	2.33E-08	2.27E-08
2.56E-08	2.61E-08	2.57E-08	2.53E-08	2.53E-08	2.53E-08	2.50E-08	2.63E-08	2.52E-08	2.40E-08	2.43E-08	2.40E-08	2.40E-08	2.42E-08
2.71E-08	2.69E-08	2.62E-08	2.65E-08	2.62E-08	2.60E-08	2.52E-08	2.68E-08	2.60E-08	2.44E-08	2.51E-08	2.51E-08	2.49E-08	2.53E-08
2.86E-08	2.76E-08	2.71E-08	2.76E-08	2.73E-08	2.69E-08	2.62E-08	2.70E-08	2.69E-08	2.54E-08	2.58E-08	2.57E-08	2.53E-08	2.60E-08
2.99E-08	2.88E-08	2.81E-08	2.81E-08	2.81E-08	2.80E-08	2.74E-08	2.78E-08	2.78E-08	2.66E-08	2.71E-08	2.69E-08	2.65E-08	2.63E-08
3.07E-08	3.02E-08	2.96E-08	2.92E-08	2.92E-08	2.91E-08	2.84E-08	2.90E-08	2.86E-08	2.77E-08	2.83E-08	2.79E-08	2.80E-08	2.70E-08
3.14E-08	3.14E-08	3.10E-08	3.02E-08	2.99E-08	3.01E-08	2.99E-08	2.98E-08	2.96E-08	2.90E-08	2.96E-08	2.88E-08	2.92E-08	2.80E-08
3.25E-08	3.18E-08	3.19E-08	3.12E-08	3.09E-08	3.10E-08	3.11E-08	3.06E-08	3.07E-08	3.05E-08	3.04E-08	2.95E-08	3.07E-08	2.96E-08
3.33E-08	3.26E-08	3.28E-08	3.26E-08	3.22E-08	3.20E-08	3.21E-08	3.20E-08	3.17E-08	3.10E-08	3.09E-08	3.02E-08	3.16E-08	3.06E-08
3.44E-08	3.37E-08	3.38E-08	3.38E-08	3.35E-08	3.32E-08	3.34E-08	3.31E-08	3.26E-08	3.24E-08	3.19E-08	3.15E-08	3.21E-08	3.14E-08
3.54E-08	3.49E-08	3.47E-08	3.50E-08	3.48E-08	3.42E-08	3.42E-08	3.39E-08	3.37E-08	3.39E-08	3.30E-08	3.24E-08	3.30E-08	3.25E-08
3.64E-08	3.58E-08	3.54E-08	3.58E-08	3.56E-08	3.53E-08	3.50E-08	3.50E-08	3.44E-08	3.50E-08	3.41E-08	3.32E-08	3.39E-08	3.37E-08
3.76E-08	3.68E-08	3.64E-08	3.66E-08	3.67E-08	3.61E-08	3.62E-08	3.64E-08	3.53E-08	3.54E-08	3.50E-08	3.44E-08	3.46E-08	3.45E-08
3.89E-08	3.82E-08	3.77E-08	3.76E-08	3.80E-08	3.71E-08	3.74E-08	3.75E-08	3.63E-08	3.63E-08	3.58E-08	3.55E-08	3.54E-08	3.53E-08
4.02E-08	3.95E-08	3.93E-08	3.92E-08	3.95E-08	3.84E-08	3.87E-08	3.86E-08	3.76E-08	3.77E-08	3.70E-08	3.67E-08	3.63E-08	3.65E-08
4.10E-08	4.07E-08	4.06E-08	4.06E-08	4.10E-08	3.96E-08	3.99E-08	3.99E-08	3.93E-08	3.91E-08	3.83E-08	3.78E-08	3.76E-08	3.79E-08
4.24E-08	4.22E-08	4.13E-08	4.19E-08	4.15E-08	4.07E-08	4.09E-08	4.11E-08	4.08E-08	4.05E-08	3.96E-08	3.89E-08	3.90E-08	3.92E-08
4.35E-08	4.33E-08	4.26E-08	4.29E-08	4.19E-08	4.18E-08	4.19E-08	4.20E-08	4.19E-08	4.15E-08	4.10E-08	4.02E-08	4.04E-08	4.03E-08
4.44E-08	4.49E-08	4.38E-08	4.43E-08	4.32E-08	4.28E-08	4.34E-08	4.29E-08	4.31E-08	4.27E-08	4.24E-08	4.14E-08	4.19E-08	4.17E-08
4.58E-08	4.63E-08	4.52E-08	4.53E-08	4.46E-08	4.40E-08	4.44E-08	4.38E-08	4.38E-08	4.35E-08	4.34E-08	4.30E-08	4.32E-08	4.28E-08
4.73E-08	4.77E-08	4.64E-08	4.61E-08	4.60E-08	4.53E-08	4.60E-08	4.53E-08	4.44E-08	4.49E-08	4.45E-08	4.43E-08	4.39E-08	4.34E-08
4.87E-08	4.87E-08	4.81E-08	4.73E-08	4.72E-08	4.68E-08	4.75E-08	4.66E-08	4.57E-08	4.65E-08	4.55E-08	4.54E-08	4.49E-08	4.45E-08
4.98E-08	4.95E-08	4.97E-08	4.90E-08	4.85E-08	4.84E-08	4.89E-08	4.79E-08	4.71E-08	4.75E-08	4.68E-08	4.66E-08	4.61E-08	4.58E-08
5.12E-08	5.09E-08	5.16E-08	5.08E-08	5.03E-08	5.01E-08	5.01E-08	4.91E-08	4.82E-08	4.84E-08	4.79E-08	4.78E-08	4.74E-08	4.72E-08

9.62E-16	9.90E-16	9.95E-16	9.70E-16	9.68E-16	9.92E-16	9.86E-16	9.61E-16	9.34E-16	8.52E-16	8.77E-16	9.21E-16	9.07E-16	8.86E-16
9.44E-16	9.58E-16	9.96E-16	9.95E-16	1.01E-15	1.02E-15	9.58E-16	9.64E-16	9.45E-16	8.66E-16	8.97E-16	9.18E-16	9.14E-16	9.00E-16
9.74E-16	9.35E-16	9.74E-16	9.93E-16	1.03E-15	1.01E-15	9.23E-16	9.76E-16	9.63E-16	8.47E-16	8.76E-16	8.99E-16	8.92E-16	8.89E-16
9.91E-16	9.50E-16	9.53E-16	9.85E-16	1.02E-15	1.00E-15	9.51E-16	9.97E-16	9.53E-16	8.43E-16	8.59E-16	8.51E-16	8.66E-16	8.60E-16
9.81E-16	9.65E-16	8.96E-16	9.52E-16	9.98E-16	9.68E-16	9.84E-16	9.94E-16	9.59E-16	8.23E-16	8.34E-16	8.22E-16	8.40E-16	8.37E-16
9.64E-16	9.46E-16	8.64E-16	9.29E-16	9.78E-16	9.44E-16	9.62E-16	9.85E-16	9.47E-16	8.05E-16	8.27E-16	8.22E-16	8.47E-16	8.32E-16
9.30E-16	9.12E-16	8.61E-16	9.15E-16	9.50E-16	9.22E-16	9.40E-16	9.66E-16	9.53E-16	8.22E-16	8.29E-16	8.29E-16	8.48E-16	8.22E-16
9.29E-16	8.90E-16	8.94E-16	9.07E-16	9.26E-16	9.08E-16	9.33E-16	9.27E-16	9.27E-16	8.49E-16	8.44E-16	8.18E-16	8.33E-16	7.90E-16
9.11E-16	8.60E-16	9.22E-16	9.47E-16	9.01E-16	9.08E-16	9.22E-16	8.78E-16	9.17E-16	8.36E-16	8.30E-16	7.90E-16	7.85E-16	7.79E-16
8.97E-16	8.47E-16	9.22E-16	9.43E-16	8.80E-16	8.87E-16	8.99E-16	8.50E-16	9.00E-16	8.08E-16	7.96E-16	7.51E-16	7.65E-16	7.54E-16
8.70E-16	8.39E-16	8.84E-16	9.06E-16	8.56E-16	8.48E-16	8.64E-16	8.43E-16	8.63E-16	7.84E-16	7.81E-16	7.34E-16	7.67E-16	7.28E-16
8.31E-16	8.29E-16	8.50E-16	8.64E-16	8.20E-16	8.16E-16	8.07E-16	8.16E-16	8.04E-16	7.62E-16	7.62E-16	7.34E-16	7.50E-16	7.13E-16
7.92E-16	8.00E-16	8.22E-16	8.43E-16	8.08E-16	8.03E-16	7.60E-16	7.67E-16	7.54E-16	7.30E-16	7.58E-16	7.12E-16	7.36E-16	7.00E-16
7.52E-16	7.63E-16	7.90E-16	8.19E-16	7.96E-16	8.00E-16	7.15E-16	7.29E-16	7.01E-16	6.97E-16	7.52E-16	6.85E-16	7.16E-16	6.70E-16
7.56E-16	7.67E-16	7.75E-16	7.91E-16	7.60E-16	7.64E-16	7.06E-16	7.15E-16	6.72E-16	6.42E-16	7.22E-16	6.80E-16	6.97E-16	6.52E-16
7.49E-16	7.77E-16	7.57E-16	7.56E-16	7.19E-16	7.20E-16	6.71E-16	6.93E-16	6.92E-16	6.41E-16	7.01E-16	6.63E-16	6.80E-16	6.51E-16
7.38E-16	7.55E-16	7.22E-16	7.33E-16	7.17E-16	7.04E-16	6.42E-16	6.88E-16	7.02E-16	6.49E-16	6.71E-16	6.53E-16	6.46E-16	6.36E-16
7.18E-16	7.37E-16	7.08E-16	7.25E-16	7.05E-16	7.06E-16	6.36E-16	6.78E-16	6.83E-16	6.47E-16	6.48E-16	6.07E-16	5.95E-16	6.13E-16
6.80E-16	7.21E-16	6.77E-16	7.04E-16	6.76E-16	6.78E-16	6.40E-16	6.65E-16	6.67E-16	6.31E-16	6.55E-16	5.69E-16	5.79E-16	5.89E-16
6.67E-16	7.09E-16	6.74E-16	6.64E-16	6.35E-16	6.35E-16	6.31E-16	6.48E-16	6.56E-16	6.02E-16	6.35E-16	5.80E-16	5.75E-16	5.79E-16
6.65E-16	7.12E-16	6.73E-16	6.30E-16	5.98E-16	6.01E-16	6.08E-16	6.33E-16	6.34E-16	6.22E-16	6.10E-16	5.93E-16	5.65E-16	5.62E-16
6.66E-16	7.04E-16	6.55E-16	6.17E-16	5.79E-16	5.58E-16	5.77E-16	6.11E-16	5.77E-16	6.15E-16	5.97E-16	5.90E-16	5.48E-16	5.43E-16
6.54E-16	7.02E-16	6.16E-16	5.91E-16	5.52E-16	5.21E-16	5.36E-16	5.70E-16	5.30E-16	5.74E-16	5.65E-16	5.53E-16	5.09E-16	5.23E-16
6.38E-16	6.69E-16	6.02E-16	5.76E-16	5.37E-16	4.91E-16	5.00E-16	5.40E-16	4.87E-16	5.43E-16	5.46E-16	5.04E-16	4.80E-16	4.93E-16
6.10E-16	6.16E-16	5.89E-16	5.69E-16	5.29E-16	4.97E-16	4.79E-16	5.23E-16	4.72E-16	5.41E-16	5.13E-16	4.57E-16	4.54E-16	4.58E-16
5.65E-16	5.80E-16	5.58E-16	5.50E-16	5.18E-16	4.98E-16	4.99E-16	5.13E-16	4.87E-16	5.17E-16	4.85E-16	4.46E-16	4.64E-16	4.50E-16
5.59E-16	5.59E-16	5.15E-16	5.17E-16	4.95E-16	4.79E-16	4.99E-16	4.95E-16	4.75E-16	4.88E-16	4.54E-16	4.37E-16	4.57E-16	4.31E-16
5.61E-16	5.34E-16	4.81E-16	4.79E-16	4.56E-16	4.73E-16	4.88E-16	4.84E-16	4.58E-16	4.54E-16	4.25E-16	4.26E-16	4.37E-16	4.03E-16
5.38E-16	5.13E-16	4.59E-16	4.69E-16	4.34E-16	4.71E-16	4.57E-16	4.68E-16	4.44E-16	4.16E-16	4.01E-16	4.09E-16	3.87E-16	3.58E-16
5.11E-16	4.81E-16	4.25E-16	4.68E-16	4.23E-16	4.44E-16	4.28E-16	4.52E-16	4.24E-16	3.87E-16	3.56E-16	4.06E-16	3.44E-16	3.27E-16
4.83E-16	4.51E-16	4.39E-16	4.43E-16	3.99E-16	4.03E-16	4.28E-16	4.18E-16	4.03E-16	3.72E-16	3.27E-16	3.85E-16	3.35E-16	3.09E-16

4.58E-16	4.06E-16	4.28E-16	4.11E-16	3.77E-16	3.70E-16	4.09E-16	3.80E-16	3.83E-16	3.64E-16	3.24E-16	3.68E-16	3.12E-16	3.28E-16
4.37E-16	3.95E-16	4.07E-16	3.90E-16	3.51E-16	3.37E-16	3.81E-16	3.38E-16	3.58E-16	3.45E-16	3.38E-16	3.50E-16	2.76E-16	3.06E-16
4.18E-16	3.80E-16	3.82E-16	3.69E-16	3.38E-16	3.42E-16	3.51E-16	3.18E-16	3.37E-16	3.22E-16	3.23E-16	3.29E-16	2.77E-16	2.80E-16
4.10E-16	3.65E-16	3.62E-16	3.44E-16	3.41E-16	3.27E-16	3.33E-16	2.96E-16	3.23E-16	3.06E-16	3.08E-16	3.13E-16	2.74E-16	2.76E-16
3.81E-16	3.46E-16	3.37E-16	3.27E-16	3.40E-16	3.16E-16	3.08E-16	2.79E-16	3.04E-16	2.96E-16	2.83E-16	2.93E-16	2.72E-16	2.82E-16
3.60E-16	3.22E-16	3.20E-16	3.13E-16	3.17E-16	2.96E-16	2.70E-16	2.55E-16	2.75E-16	2.68E-16	2.50E-16	2.62E-16	2.64E-16	2.64E-16
3.25E-16	3.04E-16	3.12E-16	2.99E-16	2.91E-16	2.75E-16	2.48E-16	2.37E-16	2.47E-16	2.46E-16	2.26E-16	2.42E-16	2.43E-16	2.37E-16
2.92E-16	2.90E-16	2.93E-16	2.85E-16	2.65E-16	2.55E-16	2.36E-16	2.35E-16	2.27E-16	2.25E-16	2.15E-16	2.35E-16	2.26E-16	2.25E-16
2.66E-16	2.69E-16	2.65E-16	2.65E-16	2.51E-16	2.44E-16	2.18E-16	2.27E-16	2.29E-16	2.20E-16	2.18E-16	2.18E-16	2.12E-16	2.08E-16
2.44E-16	2.49E-16	2.40E-16	2.47E-16	2.31E-16	2.31E-16	2.20E-16	2.17E-16	2.25E-16	2.13E-16	2.00E-16	1.96E-16	1.91E-16	1.85E-16
2.27E-16	2.23E-16	2.08E-16	2.16E-16	2.11E-16	2.13E-16	2.21E-16	2.03E-16	2.20E-16	1.98E-16	1.76E-16	1.60E-16	1.66E-16	1.58E-16
2.02E-16	2.00E-16	1.94E-16	1.93E-16	1.93E-16	2.02E-16	2.13E-16	2.08E-16	2.08E-16	1.74E-16	1.59E-16	1.45E-16	1.43E-16	1.50E-16
1.86E-16	1.94E-16	1.97E-16	1.87E-16	1.88E-16	1.92E-16	1.94E-16	1.91E-16	2.02E-16	1.69E-16	1.67E-16	1.60E-16	1.37E-16	1.54E-16
1.81E-16	1.86E-16	1.84E-16	1.86E-16	1.78E-16	1.80E-16	1.74E-16	1.75E-16	1.86E-16	1.69E-16	1.74E-16	1.71E-16	1.55E-16	1.69E-16
1.62E-16	1.65E-16	1.65E-16	1.68E-16	1.62E-16	1.59E-16	1.57E-16	1.61E-16	1.62E-16	1.64E-16	1.72E-16	1.73E-16	1.71E-16	1.64E-16
1.50E-16	1.48E-16	1.45E-16	1.43E-16	1.42E-16	1.33E-16	1.48E-16	1.52E-16	1.51E-16	1.54E-16	1.65E-16	1.53E-16	1.64E-16	1.53E-16
1.38E-16	1.28E-16	1.15E-16	1.29E-16	1.32E-16	1.26E-16	1.31E-16	1.33E-16	1.42E-16	1.49E-16	1.56E-16	1.30E-16	1.45E-16	1.42E-16
1.25E-16	1.14E-16	9.75E-17	1.12E-16	1.15E-16	1.24E-16	1.11E-16	1.11E-16	1.27E-16	1.37E-16	1.48E-16	1.14E-16	1.26E-16	1.22E-16
1.19E-16	1.07E-16	9.47E-17	8.66E-17	9.92E-17	1.15E-16	1.04E-16	1.11E-16	1.21E-16	1.31E-16	1.40E-16	1.09E-16	1.13E-16	1.10E-16
1.16E-16	1.04E-16	9.00E-17	7.47E-17	8.65E-17	1.15E-16	1.01E-16	1.13E-16	1.12E-16	1.17E-16	1.20E-16	9.90E-17	1.03E-16	1.04E-16
1.05E-16	9.83E-17	7.06E-17	6.31E-17	8.08E-17	1.04E-16	8.59E-17	9.99E-17	9.31E-17	1.09E-16	1.03E-16	9.80E-17	9.41E-17	9.06E-17
9.06E-17	7.81E-17	5.08E-17	5.89E-17	8.35E-17	8.42E-17	7.21E-17	8.61E-17	8.38E-17	1.00E-16	8.53E-17	8.70E-17	8.42E-17	7.11E-17
8.01E-17	6.32E-17	4.55E-17	6.32E-17	7.83E-17	7.33E-17	6.56E-17	7.71E-17	8.19E-17	8.47E-17	7.35E-17	7.43E-17	7.20E-17	7.23E-17
7.00E-17	6.49E-17	5.19E-17	5.29E-17	6.79E-17	6.86E-17	7.21E-17	6.67E-17	6.60E-17	7.40E-17	6.23E-17	6.05E-17	7.98E-17	6.75E-17
6.37E-17	6.39E-17	5.60E-17	4.07E-17	5.59E-17	5.95E-17	6.45E-17	4.95E-17	5.22E-17	6.11E-17	4.61E-17	4.61E-17	6.78E-17	6.14E-17
5.49E-17	5.75E-17	5.54E-17	4.28E-17	4.57E-17	4.89E-17	5.20E-17	3.96E-17	4.31E-17	5.30E-17	3.05E-17	3.32E-17	5.27E-17	5.57E-17
4.48E-17	4.36E-17	4.27E-17	3.35E-17	3.39E-17	4.69E-17	4.69E-17	3.25E-17	3.24E-17	4.20E-17	3.36E-17	2.73E-17	3.82E-17	4.63E-17
3.30E-17	3.11E-17	2.74E-17	2.70E-17	3.01E-17	3.72E-17	4.24E-17	3.01E-17	2.91E-17	3.37E-17	3.07E-17	2.01E-17	2.57E-17	3.67E-17
2.51E-17	2.62E-17	1.87E-17	2.15E-17	2.60E-17	2.99E-17	3.05E-17	2.75E-17	2.05E-17	2.31E-17	2.14E-17	8.65E-18	1.42E-17	2.73E-17
1.87E-17	2.47E-17	2.04E-17	1.39E-17	2.16E-17	2.32E-17	2.24E-17	2.97E-17	1.31E-17	1.48E-17	1.31E-17	4.86E-18	9.60E-18	1.69E-17
1.37E-17	2.04E-17	2.49E-17	1.16E-17	1.78E-17	1.90E-17	1.88E-17	2.27E-17	1.37E-17	1.48E-17	1.20E-17	5.35E-18	8.77E-18	1.14E-17
9.76E-18	1.49E-17	1.74E-17	1.08E-17	1.44E-17	1.35E-17	1.63E-17	1.62E-17	1.33E-17	1.45E-17	1.31E-17	1.08E-17	1.21E-17	1.44E-17
4.42E-18	8.62E-18	1.06E-17	1.30E-17	1.11E-17	8.82E-18	1.33E-17	9.46E-18	7.32E-18	9.49E-18	8.67E-18	8.56E-18	8.75E-18	1.52E-17
1.32E-18	5.06E-18	6.06E-18	1.27E-17	9.37E-18	4.52E-18	9.15E-18	5.47E-18	2.47E-18	5.76E-18	4.62E-18	6.37E-18	4.81E-18	1.07E-17
8.35E-19	2.05E-18	3.14E-18	6.77E-18	5.55E-18	2.03E-18	5.67E-18	2.68E-18	3.74E-19	1.99E-18	2.47E-18	3.67E-18	1.83E-18	6.38E-18
6.45E-19	5.47E-19	1.45E-18	2.10E-18	1.74E-18	8.02E-19	2.47E-18	1.63E-18	4.83E-19	1.06E-18	1.95E-18	2.05E-18	1.52E-18	1.47E-18
3.63E-20	6.37E-22	3.36E-19	2.47E-19	2.36E-19	6.12E-20	5.05E-19	3.70E-19	6.70E-21	1.74E-19	4.38E-19	7.50E-19	4.78E-19	1.85E-20
5.96E-19	7.95E-19	9.85E-20	1.17E-19	6.96E-20	3.96E-19	2.40E-22	1.56E-19	5.24E-19	1.19E-20	1.58E-20	3.60E-22	2.34E-19	2.58E-19
4.60E-18	2.97E-18	1.71E-18	2.58E-18	1.53E-18	4.18E-18	2.59E-19	2.39E-18	2.22E-18	8.70E-19	6.63E-19	1.60E-18	2.23E-18	8.77E-19
6.09E-18	6.08E-18	4.77E-18	1.06E-17	6.93E-18	1.01E-17	1.32E-18	3.74E-18	5.20E-18	3.31E-18	4.24E-18	4.49E-18	4.24E-18	1.81E-18
7.43E-18	8.40E-18	1.12E-17	2.05E-17	1.13E-17	7.89E-18	3.55E-18	6.43E-18	6.70E-18	6.09E-18	8.94E-18	5.59E-18	3.60E-18	2.09E-18
7.72E-18	1.29E-17	2.49E-17	2.91E-17	1.60E-17	7.44E-18	9.00E-18	9.27E-18	6.40E-18	8.23E-18	1.09E-17	5.73E-18	4.76E-18	2.41E-18
8.37E-18	1.73E-17	2.81E-17	2.95E-17	2.25E-17	1.33E-17	1.59E-17	1.20E-17	5.89E-18	1.03E-17	1.18E-17	6.82E-18	6.36E-18	4.86E-18
1.23E-17	2.12E-17	2.12E-17	1.95E-17	2.66E-17	1.86E-17	1.84E-17	1.42E-17	7.84E-18	1.19E-17	1.11E-17	9.12E-18	1.21E-17	1.04E-17
1.33E-17	1.93E-17	2.09E-17	1.92E-17	3.06E-17	2.34E-17	1.94E-17	1.85E-17	1.07E-17	1.73E-17	1.56E-17	1.40E-17	1.50E-17	1.27E-17
1.69E-17	2.29E-17	2.27E-17	2.10E-17	3.49E-17	2.91E-17	2.44E-17	2.61E-17	1.79E-17	2.18E-17	1.96E-17	1.90E-17	1.82E-17	1.54E-17
2.12E-17	3.40E-17	2.63E-17	2.81E-17	3.95E-17	3.62E-17	3.40E-17	3.26E-17	2.95E-17	2.85E-17	2.23E-17	2.12E-17	2.07E-17	1.86E-17
3.07E-17	4.15E-17	3.23E-17	3.26E-17	4.38E-17	4.43E-17	4.35E-17	3.88E-17	4.06E-17	3.12E-17	2.68E-17	2.41E-17	2.79E-17	2.67E-17
4.74E-17	4.78E-17	3.74E-17	3.68E-17	5.03E-17	4.74E-17	4.96E-17	3.76E-17	4.04E-17	3.51E-17	3.15E-17	2.84E-17	3.52E-17	3.69E-17
6.48E-17	5.87E-17	4.61E-17	4.01E-17	5.08E-17	5.40E-17	5.66E-17	4.33E-17	4.24E-17	4.41E-17	3.78E-17	3.85E-17	4.85E-17	5.09E-17
7.06E-17	6.97E-17	5.86E-17	5.25E-17	5.97E-17	5.77E-17	5.96E-17	5.21E-17	4.73E-17	5.56E-17	4.79E-17	4.83E-17	6.24E-17	6.08E-17
8.05E-17	8.64E-17	7.78E-17	6.63E-17	6.55E-17	7.22E-17	7.04E-17	5.79E-17	5.89E-17	7.34E-17	5.93E-17	5.75E-17	6.89E-17	7.53E-17
9.22E-17	9.63E-17	8.55E-17	7.73E-17	7.29E-17	7.53E-17	8.30E-17	7.03E-17	7.03E-17	8.70E-17	7.43E-17	7.03E-17	8.32E-17	8.06E-17
9.56E-17	1.06E-16	9.82E-17	8.60E-17	8.16E-17	8.50E-17	9.52E-17	9.11E-17	8.29E-17	9.19E-17	8.03E-17	8.43E-17	9.24E-17	7.88E-17
1.07E-16	1.17E-16	1.13E-16	1.01E-16	9.73E-17	9.90E-17	1.06E-16	1.02E-16	9.93E-17	1.08E-16	8.58E-17	9.88E-17	8.92E-17	8.66E-17
1.33E-16	1.17E-16	1.23E-16	1.11E-16	1.15E-16	1.18E-16	1.19E-16	1.07E-16	1.14E-16	1.29E-16	1.04E-16	1.19E-16	1.02E-16	1.04E-16
1.47E-16	1.27E-16	1.32E-16	1.30E-16	1.37E-16	1.36E-16	1.28E-16	1.14E-16	1.29E-16	1.43E-16	1.15E-16	1.28E-16	1.19E-16	1.25E-16
1.48E-16	1.37E-16	1.45E-16	1.39E-16	1.42E-16	1.42E-16	1.47E-16	1.36E-16	1.45E-16	1.44E-16	1.27E-16	1.25E-16	1.36E-16	1.42E-16
1.55E-16	1.56E-16	1.64E-16	1.52E-16	1.53E-16	1.58E-16	1.68E-16	1.63E-16	1.53E-16	1.39E-16	1.40E-16	1.34E-16	1.50E-16	1.58E-16
1.64E-16	1.76E-16	1.96E-16	1.77E-16	1.75E-16	1.80E-16	1.77E-16	1.71E-16	1.61E-16	1.46E-16	1.64E-16	1.57E-16	1.65E-16	1.78E-16
1.91E-16	1.91E-16	2.11E-16	1.99E-16	1.94E-16	1.95E-16	1.79E-16	1.82E-16	1.69E-16	1.65E-16	1.82E-16	1.86E-16	1.88E-16	2.08E-16
2.27E-16	2.14E-16	2.16E-16	2.12E-16	2.05E-16	2.15E-16	1.85E-16	2.00E-16	1.89E-16	1.93E-16	1.97E-16	2.07E-16	2.06E-16	2.41E-16
2.38E-16	2.32E-16	2.29E-16	2.28E-16	2.20E-16	2.36E-16	2.07E-16	2.30E-16	2.21E-16	2.38E-16	2.11E-16	2.28E-16	2.22E-16	2.59E-16
2.47E-16	2.64E-16	2.45E-16	2.32E-16	2.37E-16	2.56E-16	2.36E-16	2.55E-16	2.55E-16	2.61E-16	2.44E-16	2.60E-16	2.46E-16	2.78E-16
2.79E-16	3.09E-16	2.75E-16	2.52E-16	2.54E-16	2.80E-16	2.72E-16	2.76E-16	2.99E-16	2.73E-16	2.76E-16	2.76E-16	2.77E-16	2.89E-16
3.08E-16	3.38E-16	3.00E-16	2.89E-16	2.88E-16	2.99E-16	3.08E-16	3.08E-16	3.15E-16	3.04E-16	2.88E-16	2.98E-16	3.08E-16	2.97E-16
3.35E-16	3.45E-16	3.12E-16	3.25E-16	3.08E-16	3.27E-16	3.25E-16	3.16E-16	3.28E-16	3.32E-16	3.15E-16	3.35E-16	3.42E-16	3.40E-16
3.62E-16	3.64E-16	3.37E-16	3.66E-16	3.35E-16	3.50E-16	3.38E-16	3.42E-16	3.62E-16	3.59E-16	3.50E-16	3.54E-16	3.61E-16	3.91E-16
4.06E-16	3.95E-16	3.74E-16	4.09E-16	3.77E-16	3.97E-16	3.72E-16	3.79E-16	4.05E-16	3.68E-16	3.80E-16	3.70E-16	3.69E-16	4.16E-16
4.65E-16	4.25E-16	4.20E-16	4.50E-16	4.31E-16	4.35E-16	4.18E-16	4.16E-16	4.56E-16	3.86E-16	4.15E-16	4.03E-16	4.08E-16	4.07E-16
5.10E-16	4.70E-16	4.68E-16	4.86E-16	4.73E-16	4.74E-16	4.79E-16	4.48E-16	5.04E-16	4.38E-16	4.67E-16	4.41E-16	4.44E-16	3.97E-16
5.40E-16	5.30E-16	5.30E-16	5.19E-16	5.19E-16	5.16E-16	5.41E-16	4.97E-16	5.40E-16	4.97E-16	5.14E-16	5.02E-16	4.71E-16	4.19E-16
5.72E-16	5.80E-16	5.74E-16	5.53E-16	5.57E-16	5.58E-16	5.82E-16	5.45E-16	5.96E-16	5.33E-16	5.51E-16	5.28E-16	5.10E-16	4.66E-16
6.01E-16	6.27E-16	6.25E-16	5.85E-16	5.95E-16	5.92E-16	5.87E-16	6.16E-16	6.03E-16	5.65E-16	5.60E-16	5.31E-16	5.41E-16	5.17E-16
6.56E-16	6.80E-16	6.62E-16	6.41E-16	6.38E-16	6.43E-16	6.26E-16	6.94E-16	6.35E-16	5.78E-16	5.92E-16	5.78E-16	5.77E-16	5.86E-16
7.33E-16	7.24E-16	6.87E-16	7.02E-16	6.88E-16	6.78E-16	6.37E-16	7.16E-16	6.78E-16	5.94E-16	6.30E-16	6.31E-16	6.19E-16	6.41E-16
8.19E-16	7.59E-16	7.32E-16	7.64E-16	7.45E-16	7.25E-16	6.87E-16	7.29E-16	7.26E-16	6.45E-16	6.67E-16	6.63E-16	6.42E-16	6.76E-16
8.95E-16	8.28E-16	7.88E-16	7.90E-16	7.88E-16	7.84E-16	7.49E-16	7.73E-16	7.72E-16	7.08E-16	7.32E-16	7.21E-16	7.00E-16	6.94E-16
9.44E-16	9.13E-16	8.74E-16	8.51E-16	8.50E-16	8.45E-16	8.09E-16	8.41E-16	8.19E-16	7.67E-16	8.03E-16	7.81E-16	7.82E-16	7.31E-16
9.89E-16	9.88E-16	9.60E-16	9.12E-16	8.94E-16	9.06E-16	8.94E-16	8.86E-16	8.74E-16	8.39E-16	8.73E-16	8.30E-16	8.55E-16	7.84E-16
1.06E-15	1.01E-15	1.02E-15	9.75E-16	9.53E-16	9.61E-16	9.66E-16	9.36E-16	9.41E-16	9.29E-16	9.23E-16	8.72E-16	9.43E-16	8.75E-16
1.11E-15	1.07E-15	1.07E-15	1.06E-15	1.03E-15	1.02E-15	1.03E-15	1.02E-15	1.01E-15	9.63E-16	9.54E-16	9.11E-16	1.00E-15	9.34E-16
1.18E-15	1.14E-15	1.14E-15	1.14E-15	1.12E-15	1.10E-15	1.12E-15	1.10E-15	1.06E-15	1.05E-15	1.02E-15	9.93E-16	1.03E-15	9.83E-16
1.26E-15	1.21E-15	1.21E-15	1.22E-15	1.21E-15	1.17E-15	1.17E-15	1.15E-15	1.13E-15	1.15E-15	1.09E-15	1.05E-15	1.09E-15	1.06E-15
1.33E-15	1.28E-15	1.25E-15	1.28E-15	1.27E-15	1.25E-15	1.23E-15	1.23E-15	1.19E-15	1.22E-15	1.16E-15	1.10E-15	1.15E-15	1.14E-15
1.42E-15	1.36E-15	1.33E-15	1.34E-15	1.35E-15	1.31E-15	1.31E-15	1.33E-15	1.25E-15	1.25E-15	1.22E-15	1.18E-15	1.20E-15	1.19E-15

1.52E-15	1.46E-15	1.42E-15	1.41E-15	1.44E-15	1.38E-15	1.40E-15	1.41E-15	1.32E-15	1.32E-15	1.28E-15	1.26E-15	1.25E-15	1.25E-15
1.61E-15	1.56E-15	1.54E-15	1.54E-15	1.56E-15	1.47E-15	1.50E-15	1.49E-15	1.42E-15	1.42E-15	1.37E-15	1.35E-15	1.32E-15	1.33E-15
1.68E-15	1.66E-15	1.65E-15	1.65E-15	1.68E-15	1.57E-15	1.60E-15	1.59E-15	1.55E-15	1.53E-15	1.47E-15	1.43E-15	1.41E-15	1.44E-15
1.79E-15	1.78E-15	1.71E-15	1.76E-15	1.72E-15	1.65E-15	1.67E-15	1.69E-15	1.66E-15	1.64E-15	1.57E-15	1.52E-15	1.52E-15	1.53E-15
1.89E-15	1.88E-15	1.81E-15	1.84E-15	1.76E-15	1.74E-15	1.76E-15	1.76E-15	1.76E-15	1.72E-15	1.68E-15	1.61E-15	1.64E-15	1.62E-15
1.97E-15	2.02E-15	1.92E-15	1.96E-15	1.87E-15	1.83E-15	1.88E-15	1.84E-15	1.86E-15	1.83E-15	1.80E-15	1.71E-15	1.76E-15	1.74E-15
2.10E-15	2.14E-15	2.04E-15	2.05E-15	1.99E-15	1.93E-15	1.97E-15	1.92E-15	1.92E-15	1.89E-15	1.88E-15	1.85E-15	1.87E-15	1.84E-15
2.24E-15	2.28E-15	2.16E-15	2.13E-15	2.11E-15	2.05E-15	2.12E-15	2.05E-15	1.97E-15	2.01E-15	1.98E-15	1.96E-15	1.93E-15	1.88E-15
2.37E-15	2.37E-15	2.32E-15	2.24E-15	2.23E-15	2.19E-15	2.26E-15	2.18E-15	2.09E-15	2.16E-15	2.07E-15	2.06E-15	2.01E-15	1.98E-15
2.48E-15	2.45E-15	2.47E-15	2.40E-15	2.36E-15	2.34E-15	2.39E-15	2.30E-15	2.22E-15	2.26E-15	2.19E-15	2.18E-15	2.13E-15	2.10E-15
2.63E-15	2.59E-15	2.66E-15	2.58E-15	2.53E-15	2.51E-15	2.51E-15	2.41E-15	2.32E-15	2.34E-15	2.30E-15	2.29E-15	2.25E-15	2.23E-15
7.07E-14	6.99E-14	6.83E-14	6.82E-14	6.71E-14	6.63E-14	6.59E-14	6.57E-14	6.47E-14	6.27E-14	6.20E-14	6.06E-14	6.06E-14	5.98E-14

-2.83E-08	-2.80E-08	-2.82E-08	-2.78E-08	-2.85E-08	-2.76E-08	-2.73E-08	-2.70E-08	-2.57E-08	-2.57E-08	-2.52E-08	-2.45E-08	-2.46E-08	-2.50E-08
-2.89E-08	-2.83E-08	-2.86E-08	-2.76E-08	-2.80E-08	-2.73E-08	-2.68E-08	-2.69E-08	-2.55E-08	-2.57E-08	-2.51E-08	-2.48E-08	-2.50E-08	-2.53E-08
-2.93E-08	-2.86E-08	-2.86E-08	-2.75E-08	-2.76E-08	-2.73E-08	-2.66E-08	-2.65E-08	-2.51E-08	-2.54E-08	-2.53E-08	-2.44E-08	-2.55E-08	-2.54E-08
-2.87E-08	-2.83E-08	-2.85E-08	-2.76E-08	-2.75E-08	-2.70E-08	-2.70E-08	-2.64E-08	-2.54E-08	-2.53E-08	-2.54E-08	-2.41E-08	-2.53E-08	-2.49E-08
-2.82E-08	-2.77E-08	-2.82E-08	-2.75E-08	-2.69E-08	-2.61E-08	-2.64E-08	-2.63E-08	-2.58E-08	-2.52E-08	-2.51E-08	-2.38E-08	-2.45E-08	-2.43E-08
-2.76E-08	-2.75E-08	-2.72E-08	-2.71E-08	-2.63E-08	-2.59E-08	-2.56E-08	-2.67E-08	-2.58E-08	-2.55E-08	-2.43E-08	-2.31E-08	-2.35E-08	-2.35E-08
-2.74E-08	-2.72E-08	-2.68E-08	-2.64E-08	-2.54E-08	-2.52E-08	-2.53E-08	-2.64E-08	-2.58E-08	-2.55E-08	-2.36E-08	-2.25E-08	-2.27E-08	-2.32E-08
-2.71E-08	-2.66E-08	-2.65E-08	-2.58E-08	-2.48E-08	-2.49E-08	-2.53E-08	-2.64E-08	-2.54E-08	-2.54E-08	-2.31E-08	-2.22E-08	-2.28E-08	-2.30E-08
-2.71E-08	-2.61E-08	-2.63E-08	-2.57E-08	-2.54E-08	-2.54E-08	-2.52E-08	-2.64E-08	-2.48E-08	-2.47E-08	-2.22E-08	-2.18E-08	-2.27E-08	-2.26E-08
-2.67E-08	-2.60E-08	-2.58E-08	-2.58E-08	-2.55E-08	-2.56E-08	-2.49E-08	-2.59E-08	-2.44E-08	-2.35E-08	-2.12E-08	-2.09E-08	-2.24E-08	-2.28E-08
-2.66E-08	-2.59E-08	-2.55E-08	-2.58E-08	-2.50E-08	-2.52E-08	-2.46E-08	-2.49E-08	-2.37E-08	-2.27E-08	-2.09E-08	-2.09E-08	-2.18E-08	-2.26E-08
-2.64E-08	-2.58E-08	-2.56E-08	-2.52E-08	-2.51E-08	-2.51E-08	-2.44E-08	-2.39E-08	-2.31E-08	-2.20E-08	-2.13E-08	-2.14E-08	-2.18E-08	-2.26E-08
-2.60E-08	-2.55E-08	-2.54E-08	-2.48E-08	-2.49E-08	-2.49E-08	-2.42E-08	-2.32E-08	-2.25E-08	-2.24E-08	-2.19E-08	-2.12E-08	-2.17E-08	-2.23E-08
-2.59E-08	-2.51E-08	-2.51E-08	-2.51E-08	-2.45E-08	-2.46E-08	-2.36E-08	-2.23E-08	-2.20E-08	-2.23E-08	-2.14E-08	-2.16E-08	-2.18E-08	-2.22E-08
-2.57E-08	-2.51E-08	-2.47E-08	-2.49E-08	-2.41E-08	-2.37E-08	-2.26E-08	-2.17E-08	-2.16E-08	-2.18E-08	-2.11E-08	-2.22E-08	-2.16E-08	-2.21E-08
-2.54E-08	-2.49E-08	-2.43E-08	-2.44E-08	-2.34E-08	-2.20E-08	-2.18E-08	-2.12E-08	-2.14E-08	-2.16E-08	-2.16E-08	-2.21E-08	-2.16E-08	-2.22E-08
-2.49E-08	-2.45E-08	-2.38E-08	-2.35E-08	-2.25E-08	-2.10E-08	-2.16E-08	-2.14E-08	-2.17E-08	-2.16E-08	-2.18E-08	-2.20E-08	-2.16E-08	-2.19E-08
-2.43E-08	-2.43E-08	-2.35E-08	-2.27E-08	-2.20E-08	-2.10E-08	-2.18E-08	-2.17E-08	-2.17E-08	-2.14E-08	-2.18E-08	-2.16E-08	-2.13E-08	-2.13E-08
-2.38E-08	-2.40E-08	-2.32E-08	-2.22E-08	-2.17E-08	-2.15E-08	-2.17E-08	-2.14E-08	-2.11E-08	-2.08E-08	-2.16E-08	-2.11E-08	-2.08E-08	-2.06E-08
-2.33E-08	-2.36E-08	-2.27E-08	-2.23E-08	-2.16E-08	-2.11E-08	-2.13E-08	-2.10E-08	-2.05E-08	-2.03E-08	-2.14E-08	-2.08E-08	-1.99E-08	-2.01E-08
-2.29E-08	-2.33E-08	-2.24E-08	-2.19E-08	-2.14E-08	-2.08E-08	-2.08E-08	-2.03E-08	-2.01E-08	-2.06E-08	-2.09E-08	-2.08E-08	-1.92E-08	-1.96E-08
-2.26E-08	-2.32E-08	-2.19E-08	-2.12E-08	-2.08E-08	-2.04E-08	-2.05E-08	-1.97E-08	-2.01E-08	-2.04E-08	-2.04E-08	-2.05E-08	-1.91E-08	-1.97E-08
-2.26E-08	-2.25E-08	-2.14E-08	-2.06E-08	-2.02E-08	-1.99E-08	-2.00E-08	-1.92E-08	-1.98E-08	-1.98E-08	-2.01E-08	-1.99E-08	-1.95E-08	-1.93E-08
-2.16E-08	-2.18E-08	-2.09E-08	-2.06E-08	-2.02E-08	-2.01E-08	-1.94E-08	-1.90E-08	-1.96E-08	-1.89E-08	-1.94E-08	-1.92E-08	-1.90E-08	-1.86E-08
-2.10E-08	-2.13E-08	-2.09E-08	-2.05E-08	-1.96E-08	-1.97E-08	-1.91E-08	-1.88E-08	-1.96E-08	-1.80E-08	-1.87E-08	-1.86E-08	-1.83E-08	-1.85E-08
-2.11E-08	-2.11E-08	-2.04E-08	-2.02E-08	-1.90E-08	-1.91E-08	-1.84E-08	-1.84E-08	-1.91E-08	-1.78E-08	-1.86E-08	-1.80E-08	-1.79E-08	-1.80E-08
-2.08E-08	-2.08E-08	-2.01E-08	-1.97E-08	-1.91E-08	-1.87E-08	-1.76E-08	-1.78E-08	-1.86E-08	-1.74E-08	-1.81E-08	-1.79E-08	-1.77E-08	-1.77E-08
-1.99E-08	-2.02E-08	-1.93E-08	-1.89E-08	-1.89E-08	-1.87E-08	-1.78E-08	-1.72E-08	-1.82E-08	-1.75E-08	-1.77E-08	-1.78E-08	-1.75E-08	-1.70E-08
-1.91E-08	-1.95E-08	-1.83E-08	-1.75E-08	-1.82E-08	-1.84E-08	-1.84E-08	-1.72E-08	-1.77E-08	-1.74E-08	-1.74E-08	-1.77E-08	-1.77E-08	-1.63E-08
-1.86E-08	-1.90E-08	-1.75E-08	-1.70E-08	-1.77E-08	-1.80E-08	-1.83E-08	-1.71E-08	-1.74E-08	-1.70E-08	-1.69E-08	-1.70E-08	-1.76E-08	-1.60E-08
-1.83E-08	-1.83E-08	-1.74E-08	-1.72E-08	-1.74E-08	-1.74E-08	-1.75E-08	-1.72E-08	-1.73E-08	-1.68E-08	-1.65E-08	-1.68E-08	-1.68E-08	-1.58E-08
-1.77E-08	-1.76E-08	-1.71E-08	-1.72E-08	-1.74E-08	-1.72E-08	-1.72E-08	-1.66E-08	-1.70E-08	-1.64E-08	-1.62E-08	-1.64E-08	-1.62E-08	-1.52E-08
-1.72E-08	-1.73E-08	-1.69E-08	-1.68E-08	-1.70E-08	-1.69E-08	-1.70E-08	-1.64E-08	-1.67E-08	-1.55E-08	-1.59E-08	-1.58E-08	-1.56E-08	-1.43E-08
-1.70E-08	-1.69E-08	-1.66E-08	-1.66E-08	-1.67E-08	-1.63E-08	-1.66E-08	-1.63E-08	-1.63E-08	-1.49E-08	-1.54E-08	-1.53E-08	-1.54E-08	-1.38E-08
-1.68E-08	-1.64E-08	-1.65E-08	-1.64E-08	-1.59E-08	-1.59E-08	-1.61E-08	-1.60E-08	-1.59E-08	-1.47E-08	-1.48E-08	-1.46E-08	-1.49E-08	-1.35E-08
-1.63E-08	-1.64E-08	-1.65E-08	-1.61E-08	-1.52E-08	-1.55E-08	-1.58E-08	-1.54E-08	-1.57E-08	-1.47E-08	-1.45E-08	-1.44E-08	-1.46E-08	-1.34E-08
-1.55E-08	-1.61E-08	-1.61E-08	-1.56E-08	-1.49E-08	-1.49E-08	-1.57E-08	-1.50E-08	-1.54E-08	-1.44E-08	-1.45E-08	-1.43E-08	-1.45E-08	-1.34E-08
-1.48E-08	-1.52E-08	-1.55E-08	-1.50E-08	-1.45E-08	-1.46E-08	-1.54E-08	-1.46E-08	-1.52E-08	-1.43E-08	-1.44E-08	-1.40E-08	-1.37E-08	-1.35E-08
-1.46E-08	-1.45E-08	-1.50E-08	-1.45E-08	-1.48E-08	-1.43E-08	-1.50E-08	-1.42E-08	-1.50E-08	-1.41E-08	-1.38E-08	-1.36E-08	-1.35E-08	-1.34E-08
-1.41E-08	-1.41E-08	-1.49E-08	-1.41E-08	-1.49E-08	-1.42E-08	-1.51E-08	-1.45E-08	-1.48E-08	-1.37E-08	-1.33E-08	-1.32E-08	-1.39E-08	-1.33E-08
-1.33E-08	-1.38E-08	-1.46E-08	-1.44E-08	-1.49E-08	-1.43E-08	-1.49E-08	-1.47E-08	-1.44E-08	-1.32E-08	-1.30E-08	-1.30E-08	-1.38E-08	-1.32E-08
-1.24E-08	-1.37E-08	-1.46E-08	-1.41E-08	-1.50E-08	-1.43E-08	-1.43E-08	-1.43E-08	-1.36E-08	-1.26E-08	-1.27E-08	-1.32E-08	-1.33E-08	-1.31E-08
-1.23E-08	-1.33E-08	-1.42E-08	-1.36E-08	-1.46E-08	-1.40E-08	-1.39E-08	-1.36E-08	-1.29E-08	-1.28E-08	-1.30E-08	-1.33E-08	-1.31E-08	-1.30E-08
-1.27E-08	-1.33E-08	-1.37E-08	-1.30E-08	-1.41E-08	-1.31E-08	-1.36E-08	-1.32E-08	-1.24E-08	-1.30E-08	-1.27E-08	-1.31E-08	-1.29E-08	-1.28E-08
-1.29E-08	-1.33E-08	-1.31E-08	-1.27E-08	-1.32E-08	-1.23E-08	-1.29E-08	-1.25E-08	-1.24E-08	-1.28E-08	-1.20E-08	-1.25E-08	-1.19E-08	-1.22E-08
-1.27E-08	-1.29E-08	-1.24E-08	-1.23E-08	-1.20E-08	-1.16E-08	-1.16E-08	-1.19E-08	-1.27E-08	-1.26E-08	-1.11E-08	-1.20E-08	-1.08E-08	-1.18E-08
-1.25E-08	-1.21E-08	-1.13E-08	-1.18E-08	-1.12E-08	-1.12E-08	-9.97E-09	-1.10E-08	-1.25E-08	-1.17E-08	-1.02E-08	-1.14E-08	-9.54E-09	-1.11E-08
-1.20E-08	-1.13E-08	-1.02E-08	-1.11E-08	-1.04E-08	-1.07E-08	-8.14E-09	-9.45E-09	-1.17E-08	-1.07E-08	-9.51E-09	-1.11E-08	-9.11E-09	-1.10E-08
-1.16E-08	-1.06E-08	-9.57E-09	-1.08E-08	-9.77E-09	-1.03E-08	-7.37E-09	-9.27E-09	-1.11E-08	-1.05E-08	-9.72E-09	-1.14E-08	-1.03E-08	-1.09E-08
-1.08E-08	-9.94E-09	-9.17E-09	-1.06E-08	-9.01E-09	-1.01E-08	-9.76E-09	-9.68E-09	-1.06E-08	-1.10E-08	-1.05E-08	-1.12E-08	-1.11E-08	-1.05E-08
-9.66E-09	-9.04E-09	-8.48E-09	-9.34E-09	-9.19E-09	-9.61E-09	-9.61E-09	-9.42E-09	-1.02E-08	-1.10E-08	-1.01E-08	-1.08E-08	-1.10E-08	-1.04E-08
-8.53E-09	-8.13E-09	-8.22E-09	-7.85E-09	-8.44E-09	-8.53E-09	-8.58E-09	-9.18E-09	-9.30E-09	-1.02E-08	-9.40E-09	-1.03E-08	-1.07E-08	-1.02E-08
-8.04E-09	-7.97E-09	-8.13E-09	-7.03E-09	-8.14E-09	-8.21E-09	-7.93E-09	-8.64E-09	-8.16E-09	-9.38E-09	-8.55E-09	-9.84E-09	-9.74E-09	-9.54E-09
-7.81E-09	-7.95E-09	-7.11E-09	-6.47E-09	-7.84E-09	-7.96E-09	-7.52E-09	-7.72E-09	-7.36E-09	-8.78E-09	-7.43E-09	-8.70E-09	-8.53E-09	-8.78E-09
-8.43E-09	-8.20E-09	-7.77E-09	-6.83E-09	-7.21E-09	-7.46E-09	-6.79E-09	-7.09E-09	-6.39E-09	-7.99E-09	-6.56E-09	-7.81E-09	-7.32E-09	-7.94E-09
-8.26E-09	-7.66E-09	-7.32E-09	-8.01E-09	-6.60E-09	-6.82E-09	-6.26E-09	-6.79E-09	-6.23E-09	-7.19E-09	-6.49E-09	-6.88E-09	-6.55E-09	-7.00E-09
-7.80E-09	-7.52E-09	-7.19E-09	-7.59E-09	-5.89E-09	-6.15E-09	-6.26E-09	-6.86E-09	-6.14E-09	-6.66E-09	-6.45E-09	-6.51E-09	-6.45E-09	-6.34E-09
-7.06E-09	-7.16E-09	-6.85E-09	-6.91E-09	-5.49E-09	-5.91E-09	-5.91E-09	-6.06E-09	-5.95E-09	-6.24E-09	-5.82E-09	-6.01E-09	-5.83E-09	-6.04E-09
-6.23E-09	-6.64E-09	-6.09E-09	-5.68E-09	-5.11E-09	-5.56E-09	-5.46E-09	-5.78E-09	-5.37E-09	-5.42E-09	-4.90E-09	-5.43E-09	-5.86E-09	-5.29E-09
-5.57E-09	-6.05E-09	-5.26E-09	-4.79E-09	-4.88E-09	-5.68E-09	-5.27E-09	-5.48E-09	-4.89E-09	-5.00E-09	-4.40E-09	-4.98E-09	-5.49E-09	-4.54E-09
-4.54E-09	-5.49E-09	-4.28E-09	-4.66E-09	-4.41E-09	-5.30E-09	-5.11E-09	-4.99E-09	-4.37E-09	-4.84E-09	-3.95E-09	-4.65E-09	-4.50E-09	-3.91E-09
-3.78E-09	-4.86E-09	-3.76E-09	-4.37E-09	-3.67E-09	-4.96E-09	-5.14E-09	-4.82E-09	-4.66E-09	-4.78E-09	-3.55E-09	-3.89E-09	-3.47E-09	-3.34E-09
-3.45E-09	-4.24E-09	-3.97E-09	-4.04E-09	-3.22E-09	-4.54E-09	-4.51E-09	-4.49E-09	-4.31E-09	-4.52E-09	-3.89E-09	-3.10E-09	-2.09E-09	-2.20E-09
-3.58E-09	-3.91E-09	-3.76E-09	-3.78E-09	-3.03E-09	-3.63E-09	-3.62E-09	-3.64E-09	-3.75E-09	-3.81E-09	-3.63E-09	-2.21E-09	-6.22E-10	-5.47E-10
-3.60E-09	-3.17E-09	-3.15E-09	-3.65E-09	-2.93E-09	-2.83E-09	-3.10E-09	-3.22E-09	-2.87E-09	-2.56E-09	-2.71E-09	-1.17E-09	8.79E-10	1.05E-09
-3.07E-09	-2.84E-09	-2.80E-09	-3.01E-09	-2.61E-09	-2.37E-09	-2.75E-09	-2.68E-09	-2.47E-09	-1.56E-09	-1.75E-09	-2.42E-10	1.42E-09	1.99E-09
-2.41E-09	-2.50E-09	-2.11E-09	-1.91E-09	-2.12E-09	-1.67E-09	-1.85E-09	-2.31E-09	-1.79E-09	-1.29E-09	-1.11E-09	7.79E-10	1.18E-09	2.48E-09
-1.54E-09	-1.71E-09	-1.25E-09	-1.16E-09	-1.46E-09	-7.20E-10	-1.12E-09	-1.93E-09	-8.47E-10	-7.48E-10	-1.66E-10	2.07E-09	1.45E-09	2.52E-09
-3.05E-10	-9.01E-10	-2.67E-10	-2.73E-10	-2.51E-10	2.10E-10	-8.47E-10	-1.19E-09	-2.46E-10	-1.81E-10	3.92E-10	1.90E-09	1.42E-09	1.88E-09
1.08E-09	-2.45E-10	7.88E-10	3.17E-10	6.49E-10	5.07E-10	-6.07E-10	-2.44E-10	6.08E-10	1.07E-09	1.41E-09	1.68E-09	1.11E-09	1.74E-09
1.73E-09	4.21E-10	1.44E-09	1.50E-09	1.10E-09	6.53E-10	-2.06E-10	6.35E-10	1.11E-09	1.93E-09	2.49E-09	1.83E-09	1.41E-09	1.90E-09
2.01E-09	1.34E-09	2.11E-09	2.02E-09	1.32E-09	9.33E-10	3.01E-10	1.28E-09	1.48E-09	1.95E-09	1.82E-09	1.48E-09	1.55E-09	1.94E-09
1.79E-09	1.94E-09	2.81E-09	2.19E-09	1.85E-09	1.69E-09	1.02E-09	1.61E-09	1.94E-09	2.38E-09	1.70E-09	1.64E-09	1.43E-09	1.32E-09
1.95E-09	2.29E-09	3.00E-09	2.66E-09	2.38E-09	2.45E-09	1.92E-09	2.04E-09	2.15E-09	2.77E-09	2.12E-09	1.74E-09	1.55E-09	1.45E-09
2.64E-09	2.73E-09	2.98E-09	3.04E-09	2.71E-09	2.81E-09	2.56E-09	2.69E-09	2.77E-09	2.94E-09	2.50E-09	1.53E-09	1.91E-09	1.60E-09
3.45E-09	3.32E-09	2.83E-09	3.06E-09	2.57E-09	3.27E-09	3.47E-09	3.24E-09	3.54E-09	3.58E-09	2.99E-09	1.95E-09	2.42E-09	2.14E-09
3.63E-09	3.76E-09	3.29E-09	3.40E-09	2.93E-09	4.06E-09	3.64E-09	4.01E-09	4.23E-09	3.99E-09	3.69E-09	2.78E-09	3.10E-09	2.51E-09
4.04E-09	4.05E-09	4.00E-09	3.94E-09	3.33E-09	4.49E-09	4.04E-09	4.81E-09	4.47E-09	4.63E-09	4.18E-09	3.66E-09	3.87E-09	2.75E-09

4.80E-09	4.92E-09	4.20E-09	4.33E-09	3.96E-09	5.36E-09	4.35E-09	4.79E-09	4.78E-09	5.31E-09	4.69E-09	4.33E-09	4.10E-09	3.28E-09
5.61E-09	5.81E-09	4.54E-09	4.79E-09	4.48E-09	5.78E-09	4.67E-09	4.94E-09	5.04E-09	5.31E-09	5.14E-09	4.63E-09	4.57E-09	3.93E-09
6.23E-09	6.43E-09	5.48E-09	5.31E-09	4.87E-09	5.97E-09	5.45E-09	5.44E-09	5.76E-09	5.48E-09	5.17E-09	4.85E-09	5.06E-09	4.59E-09
6.48E-09	6.79E-09	6.26E-09	5.71E-09	5.18E-09	6.26E-09	6.02E-09	6.88E-09	6.82E-09	5.90E-09	5.50E-09	5.39E-09	5.78E-09	5.55E-09
7.14E-09	7.13E-09	6.98E-09	6.71E-09	5.89E-09	6.98E-09	6.92E-09	8.17E-09	7.79E-09	6.43E-09	6.31E-09	5.65E-09	6.11E-09	5.86E-09
7.91E-09	7.92E-09	7.67E-09	7.57E-09	7.08E-09	8.10E-09	8.30E-09	8.61E-09	8.12E-09	6.67E-09	7.12E-09	6.00E-09	6.40E-09	6.18E-09
8.44E-09	8.97E-09	8.82E-09	8.31E-09	7.93E-09	8.89E-09	8.42E-09	8.17E-09	8.07E-09	7.09E-09	7.56E-09	7.10E-09	6.88E-09	6.83E-09
8.91E-09	1.01E-08	9.52E-09	9.00E-09	8.12E-09	9.10E-09	7.94E-09	7.71E-09	7.98E-09	7.08E-09	7.88E-09	8.17E-09	7.12E-09	7.59E-09
9.71E-09	1.11E-08	9.81E-09	9.83E-09	8.78E-09	9.26E-09	8.61E-09	7.93E-09	8.61E-09	7.46E-09	7.76E-09	8.14E-09	7.40E-09	8.01E-09
1.08E-08	1.15E-08	9.99E-09	1.08E-08	9.34E-09	9.71E-09	9.68E-09	9.32E-09	9.52E-09	8.48E-09	8.22E-09	8.63E-09	7.96E-09	8.13E-09
1.18E-08	1.25E-08	1.07E-08	1.12E-08	1.05E-08	1.08E-08	1.10E-08	1.06E-08	1.02E-08	9.63E-09	9.32E-09	9.72E-09	8.48E-09	8.69E-09
1.21E-08	1.28E-08	1.17E-08	1.15E-08	1.17E-08	1.18E-08	1.14E-08	1.12E-08	1.09E-08	1.03E-08	1.00E-08	1.03E-08	8.97E-09	9.27E-09
1.31E-08	1.30E-08	1.28E-08	1.24E-08	1.26E-08	1.23E-08	1.18E-08	1.20E-08	1.17E-08	1.10E-08	1.05E-08	1.10E-08	9.64E-09	9.51E-09
1.41E-08	1.39E-08	1.32E-08	1.31E-08	1.28E-08	1.27E-08	1.24E-08	1.24E-08	1.24E-08	1.15E-08	1.12E-08	1.18E-08	1.05E-08	1.02E-08
1.52E-08	1.46E-08	1.35E-08	1.37E-08	1.33E-08	1.31E-08	1.26E-08	1.29E-08	1.31E-08	1.22E-08	1.21E-08	1.27E-08	1.09E-08	1.11E-08
1.58E-08	1.53E-08	1.41E-08	1.41E-08	1.40E-08	1.38E-08	1.34E-08	1.37E-08	1.39E-08	1.29E-08	1.27E-08	1.34E-08	1.17E-08	1.19E-08
1.60E-08	1.64E-08	1.47E-08	1.46E-08	1.44E-08	1.47E-08	1.45E-08	1.48E-08	1.52E-08	1.41E-08	1.32E-08	1.38E-08	1.27E-08	1.28E-08
1.66E-08	1.73E-08	1.57E-08	1.56E-08	1.51E-08	1.57E-08	1.53E-08	1.56E-08	1.62E-08	1.55E-08	1.41E-08	1.44E-08	1.38E-08	1.35E-08
1.75E-08	1.75E-08	1.64E-08	1.65E-08	1.60E-08	1.68E-08	1.58E-08	1.62E-08	1.66E-08	1.63E-08	1.48E-08	1.50E-08	1.53E-08	1.39E-08
1.82E-08	1.79E-08	1.70E-08	1.73E-08	1.69E-08	1.77E-08	1.67E-08	1.66E-08	1.68E-08	1.62E-08	1.58E-08	1.58E-08	1.63E-08	1.42E-08
1.83E-08	1.83E-08	1.77E-08	1.82E-08	1.80E-08	1.88E-08	1.76E-08	1.76E-08	1.76E-08	1.68E-08	1.68E-08	1.64E-08	1.66E-08	1.54E-08
1.88E-08	1.90E-08	1.82E-08	1.93E-08	1.95E-08	1.95E-08	1.85E-08	1.83E-08	1.82E-08	1.77E-08	1.75E-08	1.72E-08	1.76E-08	1.66E-08
1.92E-08	1.95E-08	1.88E-08	2.01E-08	2.05E-08	1.96E-08	1.92E-08	1.90E-08	1.87E-08	1.87E-08	1.78E-08	1.75E-08	1.87E-08	1.77E-08
1.97E-08	2.04E-08	1.97E-08	2.06E-08	2.14E-08	2.01E-08	1.97E-08	1.97E-08	1.95E-08	2.00E-08	1.85E-08	1.86E-08	1.97E-08	1.83E-08
2.06E-08	2.05E-08	2.06E-08	2.10E-08	2.20E-08	2.06E-08	2.03E-08	2.03E-08	2.01E-08	2.08E-08	1.97E-08	1.96E-08	2.01E-08	1.91E-08
2.16E-08	2.12E-08	2.18E-08	2.16E-08	2.20E-08	2.14E-08	2.09E-08	2.10E-08	2.10E-08	2.11E-08	2.11E-08	2.06E-08	2.00E-08	2.01E-08
2.25E-08	2.20E-08	2.23E-08	2.24E-08	2.25E-08	2.26E-08	2.22E-08	2.20E-08	2.19E-08	2.17E-08	2.18E-08	2.13E-08	2.05E-08	2.11E-08
2.36E-08	2.30E-08	2.33E-08	2.37E-08	2.36E-08	2.35E-08	2.36E-08	2.30E-08	2.26E-08	2.27E-08	2.27E-08	2.23E-08	2.19E-08	2.21E-08
2.48E-08	2.42E-08	2.42E-08	2.45E-08	2.48E-08	2.43E-08	2.50E-08	2.45E-08	2.31E-08	2.37E-08	2.36E-08	2.33E-08	2.33E-08	2.32E-08
2.57E-08	2.51E-08	2.51E-08	2.56E-08	2.58E-08	2.49E-08	2.64E-08	2.53E-08	2.37E-08	2.45E-08	2.39E-08	2.42E-08	2.43E-08	2.43E-08
2.62E-08	2.59E-08	2.61E-08	2.64E-08	2.69E-08	2.57E-08	2.68E-08	2.53E-08	2.48E-08	2.56E-08	2.47E-08	2.54E-08	2.51E-08	2.52E-08
2.68E-08	2.70E-08	2.73E-08	2.70E-08	2.76E-08	2.65E-08	2.76E-08	2.58E-08	2.59E-08	2.66E-08	2.57E-08	2.61E-08	2.56E-08	2.61E-08
2.79E-08	2.81E-08	2.83E-08	2.79E-08	2.82E-08	2.76E-08	2.86E-08	2.66E-08	2.66E-08	2.73E-08	2.65E-08	2.64E-08	2.59E-08	2.65E-08
2.94E-08	2.93E-08	2.92E-08	2.85E-08	2.88E-08	2.85E-08	2.88E-08	2.76E-08	2.78E-08	2.85E-08	2.75E-08	2.69E-08	2.63E-08	2.71E-08
3.05E-08	3.02E-08	3.01E-08	2.94E-08	2.98E-08	2.95E-08	2.94E-08	2.89E-08	2.90E-08	2.94E-08	2.87E-08	2.77E-08	2.71E-08	2.78E-08
3.17E-08	3.11E-08	3.15E-08	3.04E-08	3.06E-08	3.08E-08	3.07E-08	3.01E-08	2.99E-08	3.02E-08	2.96E-08	2.89E-08	2.83E-08	2.88E-08
3.27E-08	3.20E-08	3.27E-08	3.11E-08	3.18E-08	3.22E-08	3.20E-08	3.10E-08	3.09E-08	3.07E-08	2.99E-08	2.96E-08	3.00E-08	2.97E-08
3.38E-08	3.30E-08	3.33E-08	3.20E-08	3.27E-08	3.29E-08	3.31E-08	3.18E-08	3.20E-08	3.16E-08	3.08E-08	3.07E-08	3.13E-08	3.05E-08
3.43E-08	3.39E-08	3.40E-08	3.31E-08	3.33E-08	3.32E-08	3.34E-08	3.28E-08	3.28E-08	3.18E-08	3.17E-08	3.17E-08	3.15E-08	3.17E-08
3.52E-08	3.49E-08	3.46E-08	3.45E-08	3.41E-08	3.42E-08	3.40E-08	3.39E-08	3.36E-08	3.29E-08	3.28E-08	3.25E-08	3.19E-08	3.23E-08
3.65E-08	3.60E-08	3.55E-08	3.54E-08	3.55E-08	3.50E-08	3.48E-08	3.50E-08	3.44E-08	3.43E-08	3.42E-08	3.34E-08	3.26E-08	3.31E-08
3.78E-08	3.72E-08	3.69E-08	3.68E-08	3.71E-08	3.59E-08	3.62E-08	3.63E-08	3.51E-08	3.58E-08	3.64E-08	3.45E-08	3.38E-08	3.43E-08
3.92E-08	3.89E-08	3.81E-08	3.81E-08	3.81E-08	3.71E-08	3.73E-08	3.71E-08	3.61E-08	3.67E-08	3.87E-08	3.55E-08	3.50E-08	3.55E-08
4.03E-08	4.01E-08	3.95E-08	3.97E-08	3.92E-08	3.80E-08	3.84E-08	3.81E-08	3.79E-08	3.77E-08	3.80E-08	3.69E-08	3.61E-08	3.63E-08
4.14E-08	4.12E-08	4.05E-08	4.12E-08	4.03E-08	3.91E-08	3.91E-08	3.95E-08	3.96E-08	3.91E-08	3.80E-08	3.86E-08	3.75E-08	3.74E-08
4.23E-08	4.24E-08	4.18E-08	4.23E-08	4.13E-08	4.01E-08	4.00E-08	4.04E-08	4.09E-08	4.00E-08	3.93E-08	4.06E-08	3.95E-08	3.92E-08
4.36E-08	4.36E-08	4.35E-08	4.31E-08	4.22E-08	4.15E-08	4.14E-08	4.14E-08	4.16E-08	4.10E-08	4.06E-08	4.21E-08	4.16E-08	4.11E-08
4.48E-08	4.51E-08	4.53E-08	4.45E-08	4.36E-08	4.28E-08	4.28E-08	4.28E-08	4.21E-08	4.24E-08	4.16E-08	4.24E-08	4.24E-08	4.24E-08
4.64E-08	4.65E-08	4.66E-08	4.57E-08	4.51E-08	4.42E-08	4.41E-08	4.43E-08	4.33E-08	4.40E-08	4.31E-08	4.36E-08	4.30E-08	4.31E-08
4.78E-08	4.75E-08	4.74E-08	4.72E-08	4.61E-08	4.57E-08	4.53E-08	4.61E-08	4.53E-08	4.50E-08	4.44E-08	4.49E-08	4.44E-08	4.41E-08

8.00E-16	7.85E-16	7.96E-16	7.70E-16	8.10E-16	7.61E-16	7.45E-16	7.30E-16	6.60E-16	6.61E-16	6.34E-16	6.03E-16	6.03E-16	6.26E-16
8.38E-16	8.03E-16	8.18E-16	7.63E-16	7.85E-16	7.44E-16	7.20E-16	7.22E-16	6.53E-16	6.62E-16	6.29E-16	6.14E-16	6.26E-16	6.40E-16
8.56E-16	8.20E-16	8.15E-16	7.58E-16	7.61E-16	7.47E-16	7.10E-16	7.00E-16	6.31E-16	6.46E-16	6.38E-16	5.96E-16	6.52E-16	6.43E-16
8.25E-16	8.03E-16	8.12E-16	7.64E-16	7.55E-16	7.27E-16	7.29E-16	6.96E-16	6.48E-16	6.39E-16	6.47E-16	5.81E-16	6.41E-16	6.19E-16
7.93E-16	7.66E-16	7.96E-16	7.59E-16	7.25E-16	6.83E-16	6.99E-16	6.93E-16	6.63E-16	6.35E-16	6.28E-16	5.65E-16	6.01E-16	5.90E-16
7.64E-16	7.54E-16	7.40E-16	7.33E-16	6.90E-16	6.69E-16	6.55E-16	7.16E-16	6.66E-16	6.51E-16	5.91E-16	5.36E-16	5.53E-16	5.54E-16
7.52E-16	7.41E-16	7.21E-16	6.97E-16	6.47E-16	6.34E-16	6.40E-16	6.97E-16	6.67E-16	6.52E-16	5.59E-16	5.08E-16	5.14E-16	5.39E-16
7.32E-16	7.10E-16	7.01E-16	6.67E-16	6.16E-16	6.20E-16	6.38E-16	6.99E-16	6.47E-16	6.47E-16	5.35E-16	4.92E-16	5.19E-16	5.27E-16
7.33E-16	6.83E-16	6.90E-16	6.60E-16	6.44E-16	6.46E-16	6.35E-16	6.98E-16	6.15E-16	6.11E-16	4.93E-16	4.77E-16	5.13E-16	5.10E-16
7.15E-16	6.76E-16	6.66E-16	6.68E-16	6.52E-16	6.54E-16	6.18E-16	6.68E-16	5.95E-16	5.52E-16	4.50E-16	4.38E-16	5.01E-16	5.18E-16
7.05E-16	6.70E-16	6.49E-16	6.64E-16	6.24E-16	6.34E-16	6.06E-16	6.22E-16	5.61E-16	5.15E-16	4.37E-16	4.36E-16	4.73E-16	5.09E-16
6.97E-16	6.64E-16	6.55E-16	6.37E-16	6.29E-16	6.32E-16	5.94E-16	5.70E-16	5.34E-16	4.85E-16	4.56E-16	4.57E-16	4.74E-16	5.13E-16
6.76E-16	6.51E-16	6.47E-16	6.15E-16	6.19E-16	6.21E-16	5.86E-16	5.37E-16	5.05E-16	5.01E-16	4.82E-16	4.48E-16	4.69E-16	4.96E-16
6.68E-16	6.32E-16	6.28E-16	6.28E-16	5.99E-16	6.03E-16	5.56E-16	4.98E-16	4.83E-16	4.96E-16	4.56E-16	4.67E-16	4.77E-16	4.95E-16
6.61E-16	6.28E-16	6.12E-16	6.20E-16	5.81E-16	5.62E-16	5.12E-16	4.71E-16	4.66E-16	4.75E-16	4.44E-16	4.93E-16	4.66E-16	4.90E-16
6.44E-16	6.22E-16	5.92E-16	5.97E-16	5.48E-16	4.85E-16	4.77E-16	4.48E-16	4.56E-16	4.65E-16	4.65E-16	4.89E-16	4.66E-16	4.91E-16
6.20E-16	6.01E-16	5.66E-16	5.54E-16	5.08E-16	4.42E-16	4.67E-16	4.59E-16	4.69E-16	4.66E-16	4.73E-16	4.83E-16	4.67E-16	4.78E-16
5.91E-16	5.92E-16	5.52E-16	5.15E-16	4.84E-16	4.40E-16	4.74E-16	4.69E-16	4.69E-16	4.58E-16	4.76E-16	4.67E-16	4.53E-16	4.54E-16
5.67E-16	5.76E-16	5.37E-16	4.92E-16	4.70E-16	4.61E-16	4.69E-16	4.57E-16	4.44E-16	4.33E-16	4.68E-16	4.43E-16	4.33E-16	4.26E-16
5.45E-16	5.59E-16	5.16E-16	4.96E-16	4.66E-16	4.44E-16	4.55E-16	4.41E-16	4.19E-16	4.13E-16	4.59E-16	4.32E-16	3.96E-16	4.05E-16
5.24E-16	5.43E-16	5.00E-16	4.81E-16	4.57E-16	4.34E-16	4.34E-16	4.13E-16	4.04E-16	4.22E-16	4.35E-16	4.32E-16	3.70E-16	3.82E-16
5.10E-16	5.39E-16	4.79E-16	4.51E-16	4.33E-16	4.18E-16	4.21E-16	3.87E-16	4.04E-16	4.16E-16	4.17E-16	4.21E-16	3.65E-16	3.87E-16
5.10E-16	5.06E-16	4.57E-16	4.26E-16	4.10E-16	3.97E-16	4.00E-16	3.69E-16	3.90E-16	3.91E-16	4.04E-16	3.95E-16	3.79E-16	3.72E-16
4.69E-16	4.74E-16	4.38E-16	4.26E-16	4.07E-16	4.05E-16	3.78E-16	3.60E-16	3.86E-16	3.56E-16	3.76E-16	3.67E-16	3.62E-16	3.46E-16
4.43E-16	4.56E-16	4.35E-16	4.20E-16	3.85E-16	3.87E-16	3.67E-16	3.52E-16	3.84E-16	3.25E-16	3.51E-16	3.46E-16	3.37E-16	3.43E-16
4.46E-16	4.45E-16	4.18E-16	4.07E-16	3.61E-16	3.65E-16	3.38E-16	3.39E-16	3.66E-16	3.16E-16	3.47E-16	3.24E-16	3.22E-16	3.25E-16
4.34E-16	4.34E-16	4.06E-16	3.90E-16	3.66E-16	3.50E-16	3.11E-16	3.18E-16	3.46E-16	3.02E-16	3.27E-16	3.22E-16	3.15E-16	3.12E-16
3.97E-16	4.09E-16	3.73E-16	3.57E-16	3.56E-16	3.50E-16	3.17E-16	2.95E-16	3.31E-16	3.07E-16	3.14E-16	3.16E-16	3.08E-16	2.89E-16
3.65E-16	3.80E-16	3.36E-16	3.05E-16	3.33E-16	3.40E-16	3.38E-16	2.94E-16	3.14E-16	3.02E-16	3.01E-16	3.13E-16	3.15E-16	2.67E-16
3.46E-16	3.61E-16	3.08E-16	2.88E-16	3.12E-16	3.24E-16	3.33E-16	2.93E-16	3.03E-16	2.90E-16	2.86E-16	2.90E-16	3.09E-16	2.56E-16
3.35E-16	3.33E-16	3.02E-16	2.97E-16	3.04E-16	3.04E-16	3.06E-16	2.97E-16	2.98E-16	2.83E-16	2.72E-16	2.81E-16	2.83E-16	2.48E-16

3.14E-16	3.09E-16	2.91E-16	2.95E-16	3.02E-16	2.95E-16	2.96E-16	2.76E-16	2.88E-16	2.68E-16	2.64E-16	2.67E-16	2.62E-16	2.32E-16
2.96E-16	2.98E-16	2.85E-16	2.82E-16	2.91E-16	2.84E-16	2.88E-16	2.68E-16	2.78E-16	2.40E-16	2.53E-16	2.49E-16	2.45E-16	2.06E-16
2.88E-16	2.84E-16	2.77E-16	2.76E-16	2.79E-16	2.64E-16	2.77E-16	2.66E-16	2.67E-16	2.21E-16	2.37E-16	2.35E-16	2.36E-16	1.90E-16
2.83E-16	2.69E-16	2.72E-16	2.70E-16	2.53E-16	2.52E-16	2.58E-16	2.57E-16	2.54E-16	2.16E-16	2.18E-16	2.12E-16	2.21E-16	1.83E-16
2.67E-16	2.69E-16	2.73E-16	2.59E-16	2.31E-16	2.41E-16	2.50E-16	2.37E-16	2.47E-16	2.16E-16	2.09E-16	2.07E-16	2.14E-16	1.79E-16
2.40E-16	2.59E-16	2.58E-16	2.44E-16	2.22E-16	2.22E-16	2.46E-16	2.25E-16	2.38E-16	2.08E-16	2.09E-16	2.05E-16	2.10E-16	1.81E-16
2.19E-16	2.32E-16	2.40E-16	2.26E-16	2.12E-16	2.13E-16	2.37E-16	2.13E-16	2.31E-16	2.04E-16	2.08E-16	1.95E-16	1.87E-16	1.83E-16
2.14E-16	2.09E-16	2.24E-16	2.09E-16	2.19E-16	2.05E-16	2.24E-16	2.01E-16	2.24E-16	1.99E-16	1.91E-16	1.84E-16	1.81E-16	1.80E-16
1.99E-16	1.98E-16	2.22E-16	1.99E-16	2.22E-16	2.03E-16	2.27E-16	2.09E-16	2.19E-16	1.89E-16	1.77E-16	1.75E-16	1.92E-16	1.77E-16
1.77E-16	1.90E-16	2.14E-16	2.09E-16	2.21E-16	2.05E-16	2.21E-16	2.15E-16	2.07E-16	1.75E-16	1.70E-16	1.69E-16	1.90E-16	1.74E-16
1.54E-16	1.88E-16	2.12E-16	2.00E-16	2.25E-16	2.04E-16	2.06E-16	2.04E-16	1.86E-16	1.59E-16	1.61E-16	1.75E-16	1.78E-16	1.71E-16
1.52E-16	1.77E-16	2.02E-16	1.86E-16	2.12E-16	1.95E-16	1.92E-16	1.86E-16	1.66E-16	1.65E-16	1.70E-16	1.76E-16	1.73E-16	1.70E-16
1.62E-16	1.76E-16	1.87E-16	1.69E-16	1.99E-16	1.72E-16	1.84E-16	1.75E-16	1.53E-16	1.70E-16	1.63E-16	1.72E-16	1.66E-16	1.64E-16
1.66E-16	1.78E-16	1.72E-16	1.63E-16	1.75E-16	1.51E-16	1.67E-16	1.57E-16	1.55E-16	1.64E-16	1.45E-16	1.57E-16	1.42E-16	1.48E-16
1.62E-16	1.66E-16	1.55E-16	1.52E-16	1.45E-16	1.36E-16	1.34E-16	1.41E-16	1.62E-16	1.59E-16	1.24E-16	1.45E-16	1.17E-16	1.40E-16
1.57E-16	1.46E-16	1.27E-16	1.40E-16	1.25E-16	1.26E-16	9.95E-17	1.20E-16	1.56E-16	1.36E-16	1.04E-16	1.30E-16	9.10E-17	1.23E-16
1.44E-16	1.27E-16	1.04E-16	1.23E-16	1.08E-16	1.15E-16	6.63E-17	8.93E-17	1.38E-16	1.14E-16	9.04E-17	1.23E-16	8.29E-17	1.21E-16
1.35E-16	1.13E-16	9.15E-17	1.17E-16	9.54E-17	1.05E-16	5.43E-17	8.60E-17	1.23E-16	1.10E-16	9.45E-17	1.30E-16	1.05E-16	1.20E-16
1.17E-16	9.87E-17	8.40E-17	1.13E-16	8.12E-17	1.02E-16	9.52E-17	9.37E-17	1.12E-16	1.22E-16	1.11E-16	1.26E-16	1.24E-16	1.09E-16
9.33E-17	8.16E-17	7.19E-17	8.72E-17	8.45E-17	9.24E-17	9.24E-17	8.86E-17	1.05E-16	1.20E-16	1.03E-16	1.16E-16	1.21E-16	1.07E-16
7.28E-17	6.61E-17	6.76E-17	6.17E-17	7.13E-17	7.28E-17	7.37E-17	8.43E-17	8.65E-17	1.04E-16	8.83E-17	1.06E-16	1.14E-16	1.05E-16
6.46E-17	6.35E-17	6.60E-17	4.95E-17	6.63E-17	6.74E-17	6.29E-17	7.46E-17	6.67E-17	8.79E-17	7.31E-17	9.69E-17	9.48E-17	9.10E-17
6.10E-17	6.32E-17	5.05E-17	4.19E-17	6.15E-17	6.33E-17	5.65E-17	5.97E-17	5.42E-17	7.72E-17	5.52E-17	7.56E-17	7.27E-17	7.71E-17
7.11E-17	6.73E-17	6.03E-17	4.67E-17	5.20E-17	5.56E-17	4.61E-17	5.03E-17	4.08E-17	6.38E-17	4.30E-17	6.09E-17	5.36E-17	6.31E-17
6.81E-17	5.87E-17	5.35E-17	6.41E-17	4.36E-17	4.65E-17	3.92E-17	4.62E-17	3.88E-17	5.17E-17	4.21E-17	4.73E-17	4.29E-17	4.90E-17
6.08E-17	5.66E-17	5.18E-17	5.75E-17	3.47E-17	3.79E-17	3.92E-17	4.70E-17	3.78E-17	4.43E-17	4.16E-17	4.23E-17	4.16E-17	4.01E-17
4.98E-17	5.13E-17	4.69E-17	4.77E-17	3.01E-17	3.49E-17	3.50E-17	3.67E-17	3.54E-17	3.89E-17	3.39E-17	3.61E-17	3.40E-17	3.64E-17
3.88E-17	4.40E-17	3.71E-17	3.23E-17	2.61E-17	3.09E-17	2.98E-17	3.34E-17	2.88E-17	2.94E-17	2.40E-17	2.94E-17	3.43E-17	2.80E-17
3.10E-17	3.66E-17	2.77E-17	2.29E-17	2.38E-17	3.22E-17	2.78E-17	3.01E-17	2.39E-17	2.50E-17	1.94E-17	2.48E-17	3.01E-17	2.06E-17
2.06E-17	3.01E-17	1.83E-17	2.17E-17	1.95E-17	2.81E-17	2.61E-17	2.49E-17	1.91E-17	2.34E-17	1.56E-17	2.17E-17	2.02E-17	1.53E-17
1.43E-17	2.36E-17	1.41E-17	1.91E-17	1.35E-17	2.46E-17	2.64E-17	2.33E-17	2.17E-17	2.29E-17	1.26E-17	1.51E-17	1.20E-17	1.12E-17
1.19E-17	1.80E-17	1.57E-17	1.63E-17	1.04E-17	2.06E-17	2.03E-17	2.02E-17	1.86E-17	2.05E-17	1.51E-17	9.63E-18	4.36E-18	4.84E-18
1.28E-17	1.53E-17	1.42E-17	1.43E-17	9.17E-18	1.31E-17	1.31E-17	1.32E-17	1.40E-17	1.45E-17	1.32E-17	4.90E-18	3.86E-19	2.99E-19
1.30E-17	1.01E-17	9.89E-18	1.33E-17	8.61E-18	8.02E-18	9.58E-18	1.03E-17	8.25E-18	6.57E-18	7.33E-18	1.36E-18	7.73E-19	1.10E-18
9.45E-18	8.05E-18	7.82E-18	9.05E-18	6.79E-18	5.63E-18	7.56E-18	7.18E-18	6.09E-18	2.42E-18	3.07E-18	5.88E-20	2.01E-18	3.98E-18
5.82E-18	6.27E-18	4.47E-18	3.66E-18	4.50E-18	2.80E-18	3.42E-18	5.33E-18	3.19E-18	1.66E-18	1.23E-18	6.07E-19	1.40E-18	6.14E-18
2.38E-18	2.93E-18	1.56E-18	1.35E-18	2.13E-18	5.18E-19	1.25E-18	3.74E-18	7.17E-19	5.59E-19	2.74E-20	4.29E-18	2.11E-18	6.37E-18
9.32E-20	8.12E-19	7.12E-20	7.46E-20	6.31E-20	4.41E-20	7.17E-19	1.41E-18	6.05E-20	3.27E-20	1.54E-19	3.60E-18	2.02E-18	3.55E-18
1.17E-18	5.98E-20	6.21E-19	1.00E-19	4.21E-19	2.57E-19	3.68E-19	5.95E-20	3.69E-19	1.15E-18	1.99E-18	2.81E-18	1.22E-18	3.04E-18
2.99E-18	1.77E-19	2.07E-18	2.24E-18	1.22E-18	4.27E-19	4.26E-20	4.03E-19	1.24E-18	3.72E-18	6.20E-18	3.35E-18	2.00E-18	3.59E-18
4.05E-18	1.79E-18	4.45E-18	4.06E-18	1.74E-18	8.70E-19	9.03E-20	1.63E-18	2.20E-18	3.81E-18	3.33E-18	2.20E-18	2.39E-18	3.78E-18
3.19E-18	3.75E-18	7.89E-18	4.80E-18	3.42E-18	2.87E-18	1.04E-18	2.58E-18	3.77E-18	5.64E-18	2.90E-18	2.70E-18	2.06E-18	1.74E-18
3.82E-18	5.24E-18	8.99E-18	7.09E-18	5.67E-18	5.99E-18	3.69E-18	4.18E-18	4.63E-18	7.65E-18	4.51E-18	3.02E-18	2.40E-18	2.12E-18
6.99E-18	7.44E-18	8.90E-18	9.22E-18	7.37E-18	7.90E-18	6.54E-18	7.25E-18	7.69E-18	8.62E-18	6.25E-18	2.35E-18	3.63E-18	2.56E-18
1.19E-17	1.10E-17	7.98E-18	9.39E-18	6.58E-18	1.07E-17	1.20E-17	1.05E-17	1.26E-17	1.28E-17	8.93E-18	3.80E-18	5.88E-18	4.57E-18
1.32E-17	1.41E-17	1.08E-17	1.16E-17	8.56E-18	1.65E-17	1.33E-17	1.61E-17	1.79E-17	1.59E-17	1.36E-17	7.72E-18	9.59E-18	6.29E-18
1.63E-17	1.64E-17	1.60E-17	1.55E-17	1.11E-17	2.01E-17	1.64E-17	2.31E-17	1.99E-17	2.15E-17	1.75E-17	1.34E-17	1.49E-17	7.57E-18
2.30E-17	2.42E-17	1.76E-17	1.87E-17	1.57E-17	2.87E-17	1.89E-17	2.29E-17	2.29E-17	2.81E-17	2.20E-17	1.87E-17	1.68E-17	1.07E-17
3.15E-17	3.37E-17	2.06E-17	2.29E-17	2.01E-17	3.34E-17	2.18E-17	2.44E-17	2.54E-17	2.82E-17	2.64E-17	2.14E-17	2.09E-17	1.55E-17
3.89E-17	4.13E-17	3.00E-17	2.81E-17	2.37E-17	3.56E-17	2.97E-17	2.96E-17	3.32E-17	3.00E-17	2.68E-17	2.35E-17	2.56E-17	2.11E-17
4.20E-17	4.61E-17	3.91E-17	3.26E-17	2.68E-17	3.91E-17	3.62E-17	4.74E-17	4.65E-17	3.48E-17	3.02E-17	2.90E-17	3.34E-17	3.08E-17
5.10E-17	5.09E-17	4.87E-17	4.50E-17	3.47E-17	4.87E-17	4.78E-17	6.68E-17	6.06E-17	4.14E-17	3.98E-17	3.19E-17	3.73E-17	3.43E-17
6.26E-17	6.28E-17	5.89E-17	5.73E-17	5.01E-17	6.57E-17	6.89E-17	7.42E-17	6.60E-17	4.45E-17	5.07E-17	3.61E-17	4.09E-17	3.81E-17
7.12E-17	8.05E-17	7.78E-17	6.91E-17	6.29E-17	7.91E-17	7.09E-17	6.67E-17	6.52E-17	5.03E-17	5.71E-17	5.04E-17	4.74E-17	4.66E-17
7.93E-17	1.02E-16	9.06E-17	8.10E-17	6.60E-17	8.28E-17	6.31E-17	5.94E-17	6.36E-17	5.01E-17	6.21E-17	6.67E-17	5.07E-17	5.77E-17
9.42E-17	1.24E-16	9.62E-17	9.65E-17	7.71E-17	8.58E-17	7.41E-17	6.28E-17	7.41E-17	5.56E-17	6.03E-17	6.63E-17	5.48E-17	6.41E-17
1.17E-16	1.32E-16	9.97E-17	1.18E-16	8.73E-17	9.42E-17	9.38E-17	8.68E-17	9.06E-17	7.19E-17	6.75E-17	7.45E-17	6.34E-17	6.60E-17
1.40E-16	1.56E-16	1.15E-16	1.26E-16	1.10E-16	1.17E-16	1.21E-16	1.12E-16	1.04E-16	9.27E-17	8.68E-17	9.46E-17	7.19E-17	7.54E-17
1.47E-16	1.64E-16	1.36E-16	1.31E-16	1.36E-16	1.39E-16	1.29E-16	1.25E-16	1.19E-16	1.07E-16	1.00E-16	1.06E-16	8.04E-17	8.60E-17
1.72E-16	1.68E-16	1.63E-16	1.53E-16	1.59E-16	1.52E-16	1.39E-16	1.44E-16	1.37E-16	1.21E-16	1.11E-16	1.20E-16	9.30E-17	9.05E-17
1.99E-16	1.93E-16	1.75E-16	1.71E-16	1.64E-16	1.61E-16	1.54E-16	1.54E-16	1.54E-16	1.32E-16	1.25E-16	1.40E-16	1.10E-16	1.04E-16
2.32E-16	2.13E-16	1.82E-16	1.87E-16	1.76E-16	1.71E-16	1.58E-16	1.67E-16	1.73E-16	1.49E-16	1.45E-16	1.60E-16	1.19E-16	1.23E-16
2.49E-16	2.33E-16	1.99E-16	1.99E-16	1.95E-16	1.90E-16	1.79E-16	1.88E-16	1.94E-16	1.66E-16	1.61E-16	1.80E-16	1.38E-16	1.42E-16
2.57E-16	2.69E-16	2.17E-16	2.14E-16	2.06E-16	2.15E-16	2.09E-16	2.18E-16	2.31E-16	2.00E-16	1.75E-16	1.90E-16	1.61E-16	1.64E-16
2.76E-16	3.00E-16	2.46E-16	2.43E-16	2.27E-16	2.47E-16	2.34E-16	2.43E-16	2.64E-16	2.41E-16	1.99E-16	2.07E-16	1.91E-16	1.82E-16
3.06E-16	3.08E-16	2.68E-16	2.73E-16	2.55E-16	2.82E-16	2.51E-16	2.62E-16	2.74E-16	2.64E-16	2.20E-16	2.27E-16	2.34E-16	1.92E-16
3.32E-16	3.21E-16	2.90E-16	2.98E-16	2.84E-16	3.12E-16	2.78E-16	2.74E-16	2.82E-16	2.61E-16	2.49E-16	2.50E-16	2.65E-16	2.03E-16
3.36E-16	3.36E-16	3.15E-16	3.33E-16	3.24E-16	3.52E-16	3.10E-16	3.10E-16	3.10E-16	2.84E-16	2.81E-16	2.70E-16	2.74E-16	2.38E-16
3.53E-16	3.59E-16	3.33E-16	3.73E-16	3.79E-16	3.81E-16	3.43E-16	3.33E-16	3.31E-16	3.14E-16	3.08E-16	2.96E-16	3.11E-16	2.76E-16
3.70E-16	3.81E-16	3.52E-16	4.05E-16	4.21E-16	3.84E-16	3.68E-16	3.61E-16	3.50E-16	3.49E-16	3.16E-16	3.07E-16	3.50E-16	3.13E-16
3.89E-16	4.15E-16	3.87E-16	4.26E-16	4.58E-16	4.03E-16	3.88E-16	3.88E-16	3.79E-16	3.98E-16	3.43E-16	3.45E-16	3.89E-16	3.36E-16
4.24E-16	4.22E-16	4.25E-16	4.41E-16	4.83E-16	4.23E-16	4.12E-16	4.14E-16	4.05E-16	4.34E-16	3.88E-16	3.83E-16	4.03E-16	3.64E-16
4.68E-16	4.51E-16	4.74E-16	4.67E-16	4.85E-16	4.59E-16	4.37E-16	4.43E-16	4.42E-16	4.47E-16	4.43E-16	4.25E-16	4.00E-16	4.03E-16
5.06E-16	4.85E-16	4.99E-16	5.02E-16	5.07E-16	5.11E-16	4.93E-16	4.85E-16	4.78E-16	4.69E-16	4.76E-16	4.54E-16	4.22E-16	4.44E-16
5.57E-16	5.30E-16	5.45E-16	5.61E-16	5.55E-16	5.51E-16	5.59E-16	5.28E-16	5.09E-16	5.16E-16	5.16E-16	4.97E-16	4.81E-16	4.90E-16
6.17E-16	5.86E-16	5.87E-16	6.00E-16	6.14E-16	5.91E-16	6.23E-16	6.01E-16	5.32E-16	5.61E-16	5.55E-16	5.44E-16	5.41E-16	5.37E-16
6.58E-16	6.29E-16	6.30E-16	6.56E-16	6.67E-16	6.18E-16	6.97E-16	6.42E-16	5.62E-16	5.98E-16	5.71E-16	5.84E-16	5.91E-16	5.90E-16
6.88E-16	6.69E-16	6.84E-16	6.99E-16	7.26E-16	6.60E-16	7.20E-16	6.42E-16	6.14E-16	6.55E-16	6.08E-16	6.43E-16	6.32E-16	6.36E-16
7.18E-16	7.29E-16	7.45E-16	7.30E-16	7.62E-16	7.05E-16	7.62E-16	6.68E-16	6.71E-16	7.08E-16	6.58E-16	6.84E-16	6.57E-16	6.82E-16
7.77E-16	7.88E-16	8.00E-16	7.79E-16	7.94E-16	7.63E-16	8.17E-16	7.09E-16	7.06E-16	7.46E-16	7.00E-16	6.95E-16	6.71E-16	7.03E-16
8.65E-16	8.56E-16	8.50E-16	8.15E-16	8.31E-16	8.14E-16	8.29E-16	7.61E-16	7.72E-16	8.10E-16	7.58E-16	7.25E-16	6.93E-16	7.34E-16
9.30E-16	9.11E-16	9.09E-16	8.62E-16	8.87E-16	8.73E-16	8.62E-16	8.34E-16	8.43E-16	8.64E-16	8.24E-16	7.68E-16	7.32E-16	7.72E-16
1.01E-15	9.66E-16	9.92E-16	9.22E-16	9.39E-16	9.51E-16	9.43E-16	9.05E-16	8.92E-16	9.14E-16	8.75E-16	8.34E-16	8.03E-16	8.31E-16
1.07E-15	1.03E-15	1.07E-15	9.65E-16	1.01E-15	1.04E-15	1.02E-15	9.59E-16	9.58E-16	9.42E-16	8.96E-16	8.77E-16	9.00E-16	8.82E-16
1.14E-15	1.09E-15	1.11E-15	1.03E-15	1.07E-15	1.08E-15	1.10E-15	1.01E-15	1.02E-15	9.96E-16	9.47E-16	9.43E-16	9.77E-16	9.33E-16
1.18E-15	1.15E-15	1.15E-15	1.10E-15	1.11E-15	1.10E-15	1.12E-15	1.07E-15	1.07E-15	1.01E-15	1.01E-15	1.01E-15	9.93E-16	1.01E-15

1.24E-15	1.22E-15	1.20E-15	1.19E-15	1.17E-15	1.17E-15	1.15E-15	1.15E-15	1.13E-15	1.08E-15	1.08E-15	1.06E-15	1.02E-15	1.05E-15
1.33E-15	1.30E-15	1.26E-15	1.26E-15	1.26E-15	1.22E-15	1.21E-15	1.23E-15	1.18E-15	1.18E-15	1.17E-15	1.12E-15	1.06E-15	1.10E-15
1.43E-15	1.38E-15	1.36E-15	1.35E-15	1.38E-15	1.29E-15	1.31E-15	1.32E-15	1.23E-15	1.28E-15	1.32E-15	1.19E-15	1.14E-15	1.18E-15
1.54E-15	1.51E-15	1.45E-15	1.45E-15	1.45E-15	1.38E-15	1.39E-15	1.38E-15	1.31E-15	1.35E-15	1.50E-15	1.26E-15	1.22E-15	1.26E-15
1.62E-15	1.61E-15	1.56E-15	1.58E-15	1.53E-15	1.45E-15	1.47E-15	1.45E-15	1.44E-15	1.42E-15	1.45E-15	1.36E-15	1.30E-15	1.32E-15
1.71E-15	1.70E-15	1.64E-15	1.70E-15	1.63E-15	1.53E-15	1.53E-15	1.56E-15	1.57E-15	1.53E-15	1.44E-15	1.49E-15	1.41E-15	1.40E-15
1.79E-15	1.80E-15	1.75E-15	1.79E-15	1.70E-15	1.61E-15	1.60E-15	1.63E-15	1.67E-15	1.60E-15	1.54E-15	1.65E-15	1.56E-15	1.53E-15
1.90E-15	1.90E-15	1.89E-15	1.86E-15	1.78E-15	1.72E-15	1.71E-15	1.72E-15	1.73E-15	1.68E-15	1.65E-15	1.77E-15	1.73E-15	1.69E-15
2.01E-15	2.03E-15	2.06E-15	1.98E-15	1.90E-15	1.83E-15	1.83E-15	1.83E-15	1.77E-15	1.80E-15	1.73E-15	1.80E-15	1.80E-15	1.80E-15
2.15E-15	2.16E-15	2.17E-15	2.09E-15	2.03E-15	1.95E-15	1.95E-15	1.97E-15	1.88E-15	1.94E-15	1.86E-15	1.90E-15	1.85E-15	1.86E-15
2.29E-15	2.25E-15	2.25E-15	2.22E-15	2.13E-15	2.09E-15	2.05E-15	2.12E-15	2.05E-15	2.03E-15	1.97E-15	2.01E-15	1.97E-15	1.94E-15
5.85E-14	5.79E-14	5.66E-14	5.55E-14	5.45E-14	5.32E-14	5.27E-14	5.19E-14	5.09E-14	5.01E-14	4.84E-14	4.80E-14	4.71E-14	4.69E-14

-2.47E-08	-2.44E-08	-2.46E-08	-2.40E-08	-2.34E-08	-2.36E-08	-2.26E-08	-2.22E-08	-2.29E-08	-2.30E-08	-2.33E-08	-2.33E-08	-2.26E-08	-2.18E-08
-2.52E-08	-2.49E-08	-2.47E-08	-2.38E-08	-2.37E-08	-2.38E-08	-2.27E-08	-2.19E-08	-2.24E-08	-2.26E-08	-2.26E-08	-2.29E-08	-2.26E-08	-2.22E-08
-2.50E-08	-2.48E-08	-2.49E-08	-2.32E-08	-2.31E-08	-2.33E-08	-2.26E-08	-2.24E-08	-2.25E-08	-2.18E-08	-2.16E-08	-2.21E-08	-2.17E-08	-2.21E-08
-2.46E-08	-2.47E-08	-2.47E-08	-2.27E-08	-2.28E-08	-2.23E-08	-2.20E-08	-2.26E-08	-2.27E-08	-2.15E-08	-2.10E-08	-2.12E-08	-2.07E-08	-2.18E-08
-2.39E-08	-2.43E-08	-2.40E-08	-2.24E-08	-2.25E-08	-2.11E-08	-2.17E-08	-2.25E-08	-2.24E-08	-2.15E-08	-2.09E-08	-2.09E-08	-2.06E-08	-2.16E-08
-2.33E-08	-2.34E-08	-2.31E-08	-2.22E-08	-2.18E-08	-2.10E-08	-2.14E-08	-2.32E-08	-2.26E-08	-2.16E-08	-2.14E-08	-2.10E-08	-2.08E-08	-2.13E-08
-2.27E-08	-2.29E-08	-2.23E-08	-2.21E-08	-2.18E-08	-2.15E-08	-2.15E-08	-2.37E-08	-2.30E-08	-2.17E-08	-2.17E-08	-2.08E-08	-2.04E-08	-2.03E-08
-2.25E-08	-2.26E-08	-2.16E-08	-2.22E-08	-2.15E-08	-2.15E-08	-2.19E-08	-2.38E-08	-2.28E-08	-2.17E-08	-2.15E-08	-2.03E-08	-1.94E-08	-1.96E-08
-2.22E-08	-2.20E-08	-2.11E-08	-2.19E-08	-2.16E-08	-2.15E-08	-2.23E-08	-2.33E-08	-2.20E-08	-2.12E-08	-2.14E-08	-1.99E-08	-1.88E-08	-1.90E-08
-2.18E-08	-2.17E-08	-2.14E-08	-2.20E-08	-2.13E-08	-2.15E-08	-2.17E-08	-2.24E-08	-2.17E-08	-2.10E-08	-2.16E-08	-2.01E-08	-1.89E-08	-1.86E-08
-2.18E-08	-2.15E-08	-2.17E-08	-2.23E-08	-2.09E-08	-2.10E-08	-2.12E-08	-2.21E-08	-2.14E-08	-2.09E-08	-2.10E-08	-2.03E-08	-1.89E-08	-1.86E-08
-2.19E-08	-2.11E-08	-2.14E-08	-2.19E-08	-2.09E-08	-2.04E-08	-2.02E-08	-2.15E-08	-2.06E-08	-2.06E-08	-2.03E-08	-2.00E-08	-1.85E-08	-1.90E-08
-2.19E-08	-2.09E-08	-2.12E-08	-2.12E-08	-2.09E-08	-2.02E-08	-1.95E-08	-2.08E-08	-2.03E-08	-2.00E-08	-1.99E-08	-1.98E-08	-1.83E-08	-1.88E-08
-2.19E-08	-2.12E-08	-2.13E-08	-2.08E-08	-2.09E-08	-2.04E-08	-1.92E-08	-1.99E-08	-1.94E-08	-1.93E-08	-1.94E-08	-1.92E-08	-1.82E-08	-1.79E-08
-2.19E-08	-2.16E-08	-2.14E-08	-2.07E-08	-2.07E-08	-2.01E-08	-1.89E-08	-1.82E-08	-1.80E-08	-1.88E-08	-1.90E-08	-1.87E-08	-1.84E-08	-1.74E-08
-2.14E-08	-2.14E-08	-2.12E-08	-2.06E-08	-2.04E-08	-1.99E-08	-1.88E-08	-1.71E-08	-1.70E-08	-1.85E-08	-1.91E-08	-1.86E-08	-1.89E-08	-1.76E-08
-2.08E-08	-2.11E-08	-2.09E-08	-2.06E-08	-1.98E-08	-1.93E-08	-1.85E-08	-1.71E-08	-1.74E-08	-1.84E-08	-1.89E-08	-1.85E-08	-1.90E-08	-1.84E-08
-2.05E-08	-2.04E-08	-2.05E-08	-2.02E-08	-1.91E-08	-1.86E-08	-1.83E-08	-1.78E-08	-1.85E-08	-1.85E-08	-1.86E-08	-1.80E-08	-1.84E-08	-1.84E-08
-1.99E-08	-1.95E-08	-1.95E-08	-1.92E-08	-1.84E-08	-1.84E-08	-1.78E-08	-1.80E-08	-1.90E-08	-1.83E-08	-1.84E-08	-1.81E-08	-1.81E-08	-1.79E-08
-1.93E-08	-1.87E-08	-1.88E-08	-1.81E-08	-1.78E-08	-1.81E-08	-1.77E-08	-1.77E-08	-1.90E-08	-1.86E-08	-1.83E-08	-1.84E-08	-1.80E-08	-1.77E-08
-1.91E-08	-1.82E-08	-1.87E-08	-1.75E-08	-1.76E-08	-1.82E-08	-1.75E-08	-1.76E-08	-1.90E-08	-1.86E-08	-1.81E-08	-1.78E-08	-1.76E-08	-1.74E-08
-1.92E-08	-1.82E-08	-1.85E-08	-1.80E-08	-1.77E-08	-1.79E-08	-1.74E-08	-1.74E-08	-1.88E-08	-1.87E-08	-1.80E-08	-1.74E-08	-1.65E-08	-1.69E-08
-1.92E-08	-1.82E-08	-1.82E-08	-1.87E-08	-1.79E-08	-1.73E-08	-1.78E-08	-1.72E-08	-1.83E-08	-1.76E-08	-1.74E-08	-1.66E-08	-1.61E-08	-1.63E-08
-1.89E-08	-1.78E-08	-1.76E-08	-1.84E-08	-1.78E-08	-1.72E-08	-1.80E-08	-1.75E-08	-1.78E-08	-1.69E-08	-1.69E-08	-1.62E-08	-1.67E-08	-1.59E-08
-1.84E-08	-1.70E-08	-1.73E-08	-1.79E-08	-1.78E-08	-1.71E-08	-1.81E-08	-1.73E-08	-1.75E-08	-1.65E-08	-1.69E-08	-1.58E-08	-1.65E-08	-1.51E-08
-1.77E-08	-1.64E-08	-1.72E-08	-1.77E-08	-1.70E-08	-1.65E-08	-1.76E-08	-1.71E-08	-1.73E-08	-1.62E-08	-1.64E-08	-1.59E-08	-1.56E-08	-1.43E-08
-1.70E-08	-1.70E-08	-1.74E-08	-1.76E-08	-1.65E-08	-1.62E-08	-1.72E-08	-1.67E-08	-1.71E-08	-1.60E-08	-1.57E-08	-1.52E-08	-1.40E-08	-1.45E-08
-1.65E-08	-1.71E-08	-1.75E-08	-1.70E-08	-1.67E-08	-1.60E-08	-1.66E-08	-1.63E-08	-1.69E-08	-1.55E-08	-1.48E-08	-1.42E-08	-1.20E-08	-1.45E-08
-1.62E-08	-1.67E-08	-1.71E-08	-1.65E-08	-1.67E-08	-1.64E-08	-1.63E-08	-1.56E-08	-1.61E-08	-1.49E-08	-1.39E-08	-1.36E-08	-1.06E-08	-1.39E-08
-1.61E-08	-1.63E-08	-1.62E-08	-1.60E-08	-1.63E-08	-1.63E-08	-1.57E-08	-1.50E-08	-1.50E-08	-1.42E-08	-1.32E-08	-1.35E-08	-1.37E-08	-1.41E-08
-1.57E-08	-1.62E-08	-1.57E-08	-1.56E-08	-1.57E-08	-1.54E-08	-1.52E-08	-1.43E-08	-1.43E-08	-1.42E-08	-1.41E-08	-1.31E-08	-1.37E-08	-1.39E-08
-1.53E-08	-1.59E-08	-1.57E-08	-1.52E-08	-1.52E-08	-1.46E-08	-1.47E-08	-1.40E-08	-1.39E-08	-1.43E-08	-1.38E-08	-1.29E-08	-1.37E-08	-1.35E-08
-1.51E-08	-1.54E-08	-1.49E-08	-1.45E-08	-1.48E-08	-1.40E-08	-1.42E-08	-1.41E-08	-1.34E-08	-1.37E-08	-1.36E-08	-1.28E-08	-1.35E-08	-1.32E-08
-1.47E-08	-1.45E-08	-1.43E-08	-1.42E-08	-1.44E-08	-1.34E-08	-1.33E-08	-1.36E-08	-1.33E-08	-1.29E-08	-1.26E-08	-1.27E-08	-1.34E-08	-1.32E-08
-1.42E-08	-1.42E-08	-1.38E-08	-1.41E-08	-1.41E-08	-1.34E-08	-1.26E-08	-1.27E-08	-1.28E-08	-1.27E-08	-1.19E-08	-1.23E-08	-1.30E-08	-1.29E-08
-1.38E-08	-1.38E-08	-1.39E-08	-1.39E-08	-1.38E-08	-1.35E-08	-1.19E-08	-1.21E-08	-1.26E-08	-1.25E-08	-1.21E-08	-1.21E-08	-1.24E-08	-1.23E-08
-1.35E-08	-1.38E-08	-1.39E-08	-1.36E-08	-1.34E-08	-1.32E-08	-1.16E-08	-1.20E-08	-1.28E-08	-1.29E-08	-1.26E-08	-1.24E-08	-1.18E-08	-1.19E-08
-1.34E-08	-1.37E-08	-1.35E-08	-1.29E-08	-1.26E-08	-1.28E-08	-1.15E-08	-1.22E-08	-1.28E-08	-1.28E-08	-1.25E-08	-1.26E-08	-1.18E-08	-1.15E-08
-1.34E-08	-1.35E-08	-1.31E-08	-1.28E-08	-1.21E-08	-1.25E-08	-1.17E-08	-1.22E-08	-1.22E-08	-1.24E-08	-1.22E-08	-1.21E-08	-1.16E-08	-1.10E-08
-1.29E-08	-1.34E-08	-1.30E-08	-1.25E-08	-1.20E-08	-1.20E-08	-1.15E-08	-1.24E-08	-1.14E-08	-1.18E-08	-1.20E-08	-1.13E-08	-1.03E-08	-1.03E-08
-1.27E-08	-1.31E-08	-1.31E-08	-1.23E-08	-1.23E-08	-1.23E-08	-1.17E-08	-1.23E-08	-1.14E-08	-1.15E-08	-1.18E-08	-1.01E-08	-9.53E-09	-1.01E-08
-1.26E-08	-1.27E-08	-1.30E-08	-1.25E-08	-1.27E-08	-1.26E-08	-1.16E-08	-1.17E-08	-1.09E-08	-1.07E-08	-1.09E-08	-9.23E-09	-8.47E-09	-9.83E-09
-1.28E-08	-1.30E-08	-1.33E-08	-1.26E-08	-1.29E-08	-1.29E-08	-1.13E-08	-1.10E-08	-1.03E-08	-9.95E-09	-1.04E-08	-9.23E-09	-8.39E-09	-9.36E-09
-1.27E-08	-1.33E-08	-1.33E-08	-1.23E-08	-1.27E-08	-1.25E-08	-1.03E-08	-1.01E-08	-9.89E-09	-1.03E-08	-1.06E-08	-9.62E-09	-8.73E-09	-9.02E-09
-1.20E-08	-1.27E-08	-1.26E-08	-1.19E-08	-1.19E-08	-1.19E-08	-9.09E-09	-8.79E-09	-9.29E-09	-9.79E-09	-9.92E-09	-9.62E-09	-9.12E-09	-8.71E-09
-1.12E-08	-1.17E-08	-1.17E-08	-1.16E-08	-1.14E-08	-1.09E-08	-8.41E-09	-6.93E-09	-8.79E-09	-8.85E-09	-8.54E-09	-9.77E-09	-9.42E-09	-8.58E-09
-1.05E-08	-1.12E-08	-1.08E-08	-1.08E-08	-1.08E-08	-1.08E-08	-8.71E-09	-7.20E-09	-8.79E-09	-8.84E-09	-7.80E-09	-9.51E-09	-9.27E-09	-9.25E-09
-1.07E-08	-1.13E-08	-1.15E-08	-1.14E-08	-1.15E-08	-1.05E-08	-9.10E-09	-9.49E-09	-8.77E-09	-8.72E-09	-7.78E-09	-9.25E-09	-8.62E-09	-8.64E-09
-1.12E-08	-1.10E-08	-1.15E-08	-1.07E-08	-1.07E-08	-9.82E-09	-9.13E-09	-9.09E-09	-8.16E-09	-8.77E-09	-8.05E-09	-9.05E-09	-8.23E-09	-8.21E-09
-1.07E-08	-1.07E-08	-1.06E-08	-1.01E-08	-1.02E-08	-9.50E-09	-9.23E-09	-8.85E-09	-8.24E-09	-8.77E-09	-8.43E-09	-8.35E-09	-7.56E-09	-7.96E-09
-1.02E-08	-1.03E-08	-9.91E-09	-9.58E-09	-1.00E-08	-9.24E-09	-8.98E-09	-8.55E-09	-8.80E-09	-8.85E-09	-8.41E-09	-7.71E-09	-7.17E-09	-7.90E-09
-9.87E-09	-9.75E-09	-9.64E-09	-9.19E-09	-9.49E-09	-8.71E-09	-8.29E-09	-8.56E-09	-9.01E-09	-8.48E-09	-8.08E-09	-7.55E-09	-7.11E-09	-7.69E-09
-9.07E-09	-8.63E-09	-9.13E-09	-8.61E-09	-8.61E-09	-7.65E-09	-7.65E-09	-8.16E-09	-8.29E-09	-7.97E-09	-7.62E-09	-7.10E-09	-6.88E-09	-7.18E-09
-8.40E-09	-7.54E-09	-8.00E-09	-7.45E-09	-7.54E-09	-7.13E-09	-6.98E-09	-7.24E-09	-7.46E-09	-7.30E-09	-6.60E-09	-6.21E-09	-6.14E-09	-6.12E-09
-7.54E-09	-6.16E-09	-6.53E-09	-6.26E-09	-6.10E-09	-6.25E-09	-5.95E-09	-6.12E-09	-6.12E-09	-6.41E-09	-5.53E-09	-5.18E-09	-5.14E-09	-4.98E-09
-6.55E-09	-5.83E-09	-5.55E-09	-5.14E-09	-4.92E-09	-4.87E-09	-4.94E-09	-4.77E-09	-4.87E-09	-5.19E-09	-4.43E-09	-4.43E-09	-4.25E-09	-3.98E-09
-6.11E-09	-5.56E-09	-5.84E-09	-4.53E-09	-4.04E-09	-3.88E-09	-4.47E-09	-3.70E-09	-3.82E-09	-3.80E-09	-3.53E-09	-3.60E-09	-3.54E-09	-2.94E-09
-5.65E-09	-4.86E-09	-5.46E-09	-4.01E-09	-3.77E-09	-3.26E-09	-3.58E-09	-3.16E-09	-3.22E-09	-2.30E-09	-2.44E-09	-2.43E-09	-2.67E-09	-1.60E-09
-5.00E-09	-4.36E-09	-4.73E-09	-3.47E-09	-3.50E-09	-2.75E-09	-2.51E-09	-2.22E-09	-2.07E-09	-1.28E-09	-1.15E-09	-1.16E-09	-1.75E-09	-4.51E-10
-4.41E-09	-3.68E-09	-4.11E-09	-3.33E-09	-3.21E-09	-2.41E-09	-1.48E-09	-6.61E-10	-9.24E-10	-3.08E-10	1.00E-10	-2.07E-10	-8.79E-10	3.15E-10
-3.93E-09	-2.80E-09	-3.34E-09	-2.96E-09	-2.48E-09	-1.89E-09	-4.63E-10	9.50E-10	5.07E-10	7.84E-10	1.08E-09	4.92E-10	-1.46E-10	7.91E-10
-3.17E-09	-2.29E-09	-2.59E-09	-1.85E-09	-1.18E-09	-7.40E-10	8.04E-10	2.32E-09	2.27E-09	2.03E-09	1.75E-09	9.24E-10	6.75E-10	7.35E-10
-2.22E-09	-1.31E-09	-1.49E-09	-7.45E-10	1.88E-10	5.59E-10	1.59E-09	2.94E-09	3.32E-09	2.68E-09	2.52E-09	1.03E-09	1.01E-09	2.83E-10
-6.57E-10	-2.37E-10	6.66E-11	4.02E-10	1.37E-09	1.42E-09	2.28E-09	3.09E-09	3.11E-09	2.49E-09	2.38E-09	1.59E-09	1.11E-09	2.38E-10
1.57E-09	1.51E-09	1.66E-09	1.81E-09	2.10E-09	1.82E-09	2.89E-09	2.96E-09	2.16E-09	1.48E-09	1.70E-09	2.06E-09	8.83E-10	1.13E-10
3.07E-09	2.45E-09	2.62E-09	2.19E-09	2.31E-09	2.15E-09	2.05E-09	1.89E-09	1.34E-09	1.76E-10	6.34E-10	2.29E-09	4.41E-11	-1.43E-10
3.44E-09	2.65E-09	3.08E-09	1.92E-09	1.76E-09	1.96E-09	7.75E-10	7.70E-10	1.19E-09	-3.50E-10	5.08E-10	7.05E-10	-9.13E-10	-6.07E-10
3.25E-09	3.06E-09	2.80E-09	1.46E-09	1.34E-09	1.04E-09	-4.31E-10	3.63E-10	3.88E-10	-1.15E-09	-8.91E-10	-6.19E-10	-1.50E-09	-1.02E-09
2.53E-09	2.91E-09	2.34E-09	1.09E-09	7.54E-10	1.81E-10	-1.16E-09	-2.81E-11	-4.47E-10	-1.93E-09	-1.91E-09	-1.44E-09	-1.85E-09	-1.62E-09
1.51E-09	2.05E-09	1.98E-09	7.24E-10	2.97E-10	1.57E-10	-1.09E-09	-6.96E-10	-1.02E-09	-1.97E-09	-2.06E-09	-1.59E-09	-1.87E-09	-1.91E-09
1.30E-09	1.73E-09	1.94E-09	8.20E-10	-2.87E-10	4.98E-10	-7.38E-10	-7.42E-10	-9.30E-10	-1.42E-09	-1.42E-09	-1.36E-09	-1.69E-09	-1.58E-09
9.49E-10	1.24E-09	1.50E-09	9.07E-10	-4.44E-10	-1.51E-10	-5.86E-10	-5.51E-10	-7.57E-10	-9.40E-10	-1.19E-09	-8.89E-10	-1.32E-09	-1.21E-09
4.87E-10	2.94E-10	7.67E-10	5.13E-10	-3.16E-10	-5.29E-12	-4.19E-10	-5.41E-10	-5.23E-10	-6.01E-10	-8.79E-10	-5.58E-10	-8.51E-10	-8.83E-10
7.17E-10	3.49E-10	7.14E-10	4.13E-10	2.04E-10	2.72E-10	1.80E-11	-1.97E-10	-1.40E-10	-3.84E-10	-3.27E-10	-4.53E-10	-3.19E-10	-3.01E-10
8.39E-10	7.80E-10	1.15E-09	1.01E-09	6.37E-10	6.38E-10	5.08E-10	2.94E-11	1.38E-10	4.60E-11	7.57E-11	-1.94E-10	-3.69E-11	1.82E-10
1.02E-09	1.17E-09	1.51E-09	1.13E-09	9.45E-10	1.10E-09	1.04E-09	3.63E-10	2.26E-10	6.54E-10	-8.33E-12	-1.71E-11	6.98E-11	3.91E-10
1.44E-09	1.33E-09	1.61E-09	1.04E-09	1.27E-09	1.34E-09	1.49E-09	6.98E-10	5.71E-10	8.21E-10	2.57E-10	-1.28E-10	7.78E-10	6.84E-10
1.90E-09	1.59E-09	1.78E-09	1.30E-09	2.22E-09	1.47E-09	1.98E-09	1.16E-09	1.10E-09	1.10E-09	5.56E-10	4.65E-10	1.15E-09	8.98E-10

2.60E-09	2.13E-09	2.33E-09	2.12E-09	3.24E-09	2.12E-09	2.36E-09	1.94E-09	1.70E-09	1.39E-09	1.00E-09	1.31E-09	2.00E-09	1.10E-09
3.25E-09	3.06E-09	3.31E-09	2.70E-09	4.02E-09	2.55E-09	2.62E-09	2.56E-09	2.38E-09	1.68E-09	1.22E-09	2.06E-09	3.02E-09	1.91E-09
4.19E-09	3.90E-09	4.31E-09	3.53E-09	4.26E-09	2.92E-09	3.10E-09	3.01E-09	2.83E-09	2.02E-09	2.19E-09	2.79E-09	3.07E-09	2.11E-09
4.81E-09	4.55E-09	4.22E-09	3.87E-09	4.48E-09	3.71E-09	3.64E-09	3.47E-09	3.14E-09	2.14E-09	2.74E-09	3.31E-09	3.12E-09	2.41E-09
4.94E-09	5.08E-09	4.60E-09	4.35E-09	4.58E-09	4.39E-09	4.06E-09	3.59E-09	3.64E-09	2.94E-09	3.26E-09	3.49E-09	3.27E-09	2.99E-09
5.38E-09	5.62E-09	4.98E-09	4.99E-09	4.54E-09	4.69E-09	4.24E-09	3.96E-09	4.23E-09	3.92E-09	3.47E-09	3.81E-09	3.70E-09	3.57E-09
6.03E-09	6.12E-09	5.52E-09	5.47E-09	4.59E-09	5.01E-09	4.57E-09	4.14E-09	4.77E-09	4.78E-09	3.26E-09	3.97E-09	4.30E-09	4.34E-09
7.08E-09	6.54E-09	6.48E-09	6.22E-09	4.85E-09	5.00E-09	5.09E-09	4.91E-09	5.35E-09	4.78E-09	4.21E-09	4.10E-09	4.73E-09	5.19E-09
7.70E-09	6.88E-09	6.85E-09	6.75E-09	3.58E-09	4.87E-09	5.81E-09	5.81E-09	5.63E-09	5.25E-09	5.36E-09	4.68E-09	4.73E-09	5.63E-09
8.06E-09	7.04E-09	7.31E-09	6.73E-09	2.82E-09	4.98E-09	6.69E-09	7.09E-09	6.48E-09	6.09E-09	6.32E-09	5.51E-09	5.63E-09	6.55E-09
8.56E-09	7.59E-09	7.82E-09	7.67E-09	4.69E-09	6.50E-09	7.44E-09	7.71E-09	7.72E-09	6.69E-09	6.99E-09	7.04E-09	6.74E-09	7.04E-09
9.64E-09	8.33E-09	7.95E-09	8.80E-09	7.61E-09	7.98E-09	7.97E-09	7.87E-09	8.27E-09	7.55E-09	8.10E-09	8.13E-09	7.56E-09	7.73E-09
1.02E-08	9.22E-09	8.59E-09	9.52E-09	9.07E-09	8.50E-09	8.53E-09	8.40E-09	8.90E-09	8.75E-09	9.06E-09	8.67E-09	8.33E-09	8.39E-09
1.08E-08	1.04E-08	9.47E-09	9.70E-09	1.01E-08	9.11E-09	9.18E-09	9.52E-09	1.03E-08	9.95E-09	9.81E-09	9.33E-09	8.75E-09	9.09E-09
1.19E-08	1.19E-08	1.00E-08	1.06E-08	1.09E-08	9.89E-09	1.02E-08	1.08E-08	1.10E-08	1.01E-08	1.08E-08	9.92E-09	9.19E-09	9.57E-09
1.28E-08	1.28E-08	1.08E-08	1.15E-08	1.18E-08	1.10E-08	1.13E-08	1.19E-08	1.19E-08	1.14E-08	1.21E-08	1.09E-08	9.52E-09	9.83E-09
1.30E-08	1.29E-08	1.15E-08	1.21E-08	1.23E-08	1.23E-08	1.22E-08	1.27E-08	1.37E-08	1.38E-08	1.35E-08	1.25E-08	1.05E-08	1.06E-08
1.36E-08	1.29E-08	1.24E-08	1.29E-08	1.27E-08	1.28E-08	1.27E-08	1.39E-08	1.55E-08	1.62E-08	1.59E-08	1.42E-08	1.16E-08	1.09E-08
1.43E-08	1.39E-08	1.31E-08	1.32E-08	1.34E-08	1.35E-08	1.36E-08	1.51E-08	1.65E-08	1.79E-08	1.76E-08	1.53E-08	1.27E-08	1.18E-08
1.45E-08	1.50E-08	1.42E-08	1.43E-08	1.40E-08	1.44E-08	1.46E-08	1.60E-08	1.69E-08	1.87E-08	1.83E-08	1.62E-08	1.38E-08	1.28E-08
1.52E-08	1.66E-08	1.58E-08	1.55E-08	1.53E-08	1.56E-08	1.51E-08	1.67E-08	1.70E-08	1.81E-08	1.79E-08	1.68E-08	1.49E-08	1.40E-08
1.65E-08	1.82E-08	1.71E-08	1.66E-08	1.66E-08	1.65E-08	1.57E-08	1.68E-08	1.70E-08	1.80E-08	1.73E-08	1.69E-08	1.57E-08	1.49E-08
1.75E-08	1.88E-08	1.84E-08	1.74E-08	1.79E-08	1.73E-08	1.62E-08	1.71E-08	1.69E-08	1.82E-08	1.70E-08	1.66E-08	1.64E-08	1.54E-08
1.81E-08	1.81E-08	1.92E-08	1.81E-08	1.89E-08	1.80E-08	1.71E-08	1.77E-08	1.73E-08	1.75E-08	1.68E-08	1.68E-08	1.68E-08	1.58E-08
1.90E-08	1.84E-08	1.99E-08	1.88E-08	1.97E-08	1.91E-08	1.79E-08	1.84E-08	1.80E-08	1.85E-08	1.76E-08	1.77E-08	1.78E-08	1.66E-08
2.03E-08	1.94E-08	2.06E-08	1.96E-08	1.98E-08	1.99E-08	1.88E-08	1.92E-08	1.88E-08	1.99E-08	1.86E-08	1.82E-08	1.84E-08	1.75E-08
2.15E-08	2.05E-08	2.12E-08	1.99E-08	2.07E-08	2.03E-08	2.01E-08	2.02E-08	1.94E-08	2.04E-08	1.94E-08	1.87E-08	1.90E-08	1.82E-08
2.20E-08	2.13E-08	2.19E-08	2.09E-08	2.14E-08	2.11E-08	2.07E-08	2.10E-08	1.99E-08	2.05E-08	1.99E-08	1.91E-08	1.94E-08	1.91E-08
2.23E-08	2.22E-08	2.28E-08	2.19E-08	2.24E-08	2.21E-08	2.14E-08	2.18E-08	2.11E-08	2.11E-08	2.08E-08	1.97E-08	1.98E-08	2.00E-08
2.33E-08	2.32E-08	2.38E-08	2.26E-08	2.30E-08	2.31E-08	2.19E-08	2.22E-08	2.28E-08	2.20E-08	2.21E-08	2.03E-08	2.07E-08	2.06E-08
2.42E-08	2.39E-08	2.46E-08	2.37E-08	2.40E-08	2.41E-08	2.26E-08	2.28E-08	2.40E-08	2.29E-08	2.28E-08	2.10E-08	2.17E-08	2.14E-08
2.46E-08	2.51E-08	2.56E-08	2.51E-08	2.54E-08	2.53E-08	2.36E-08	2.39E-08	2.43E-08	2.34E-08	2.31E-08	2.23E-08	2.22E-08	2.21E-08
2.57E-08	2.60E-08	2.61E-08	2.58E-08	2.59E-08	2.57E-08	2.45E-08	2.48E-08	2.47E-08	2.41E-08	2.37E-08	2.32E-08	2.32E-08	2.28E-08
2.65E-08	2.65E-08	2.70E-08	2.64E-08	2.66E-08	2.61E-08	2.55E-08	2.57E-08	2.54E-08	2.51E-08	2.47E-08	2.42E-08	2.44E-08	2.39E-08
2.74E-08	2.71E-08	2.78E-08	2.72E-08	2.74E-08	2.72E-08	2.67E-08	2.68E-08	2.59E-08	2.56E-08	2.51E-08	2.53E-08	2.54E-08	2.48E-08
2.85E-08	2.77E-08	2.86E-08	2.78E-08	2.80E-08	2.87E-08	2.82E-08	2.79E-08	2.68E-08	2.65E-08	2.57E-08	2.63E-08	2.63E-08	2.61E-08
2.94E-08	2.89E-08	2.96E-08	2.89E-08	2.88E-08	2.93E-08	2.87E-08	2.86E-08	2.79E-08	2.77E-08	2.67E-08	2.75E-08	2.74E-08	2.70E-08
3.04E-08	3.02E-08	3.04E-08	2.99E-08	2.98E-08	2.96E-08	2.97E-08	2.93E-08	2.88E-08	2.91E-08	2.81E-08	2.85E-08	2.81E-08	2.80E-08
3.16E-08	3.15E-08	3.15E-08	3.12E-08	3.05E-08	3.05E-08	3.09E-08	2.99E-08	2.99E-08	3.01E-08	2.92E-08	2.97E-08	2.87E-08	2.88E-08
3.27E-08	3.28E-08	3.25E-08	3.26E-08	3.15E-08	3.16E-08	3.19E-08	3.09E-08	3.05E-08	3.09E-08	3.03E-08	3.10E-08	2.96E-08	2.96E-08
3.34E-08	3.36E-08	3.34E-08	3.37E-08	3.28E-08	3.24E-08	3.26E-08	3.20E-08	3.16E-08	3.15E-08	3.12E-08	3.17E-08	3.08E-08	3.08E-08
3.39E-08	3.44E-08	3.45E-08	3.53E-08	3.39E-08	3.33E-08	3.36E-08	3.32E-08	3.28E-08	3.22E-08	3.22E-08	3.24E-08	3.18E-08	3.20E-08
3.49E-08	3.50E-08	3.60E-08	3.67E-08	3.53E-08	3.49E-08	3.46E-08	3.37E-08	3.41E-08	3.33E-08	3.34E-08	3.36E-08	3.25E-08	3.28E-08
3.63E-08	3.59E-08	3.66E-08	3.71E-08	3.62E-08	3.62E-08	3.53E-08	3.44E-08	3.55E-08	3.41E-08	3.48E-08	3.45E-08	3.34E-08	3.37E-08
3.70E-08	3.73E-08	3.73E-08	3.74E-08	3.69E-08	3.70E-08	3.61E-08	3.54E-08	3.68E-08	3.53E-08	3.56E-08	3.55E-08	3.47E-08	3.48E-08
3.89E-08	3.87E-08	3.81E-08	3.83E-08	3.79E-08	3.73E-08	3.72E-08	3.69E-08	3.77E-08	3.65E-08	3.66E-08	3.67E-08	3.58E-08	3.63E-08
4.11E-08	4.00E-08	3.91E-08	3.92E-08	3.92E-08	3.81E-08	3.86E-08	3.80E-08	3.84E-08	3.78E-08	3.78E-08	3.80E-08	3.75E-08	3.79E-08
4.23E-08	4.11E-08	4.04E-08	4.05E-08	4.03E-08	3.95E-08	4.01E-08	3.92E-08	3.95E-08	3.89E-08	3.92E-08	3.94E-08	3.90E-08	3.94E-08
4.31E-08	4.19E-08	4.17E-08	4.18E-08	4.17E-08	4.10E-08	4.11E-08	4.02E-08	4.06E-08	4.03E-08	4.03E-08	4.02E-08	3.99E-08	4.02E-08
4.43E-08	4.30E-08	4.29E-08	4.33E-08	4.26E-08	4.21E-08	4.16E-08	4.12E-08	4.20E-08	4.14E-08	4.12E-08	4.08E-08	4.12E-08	4.10E-08

6.09E-16	5.96E-16	6.08E-16	5.77E-16	5.50E-16	5.56E-16	5.10E-16	4.94E-16	5.26E-16	5.28E-16	5.43E-16	5.43E-16	5.09E-16	4.75E-16
6.33E-16	6.19E-16	6.12E-16	5.65E-16	5.59E-16	5.64E-16	5.14E-16	4.80E-16	5.03E-16	5.09E-16	5.10E-16	5.25E-16	5.09E-16	4.95E-16
6.25E-16	6.16E-16	6.19E-16	5.38E-16	5.36E-16	5.41E-16	5.10E-16	5.01E-16	5.07E-16	4.76E-16	4.68E-16	4.87E-16	4.71E-16	4.88E-16
6.06E-16	6.10E-16	6.11E-16	5.17E-16	5.20E-16	4.96E-16	4.83E-16	5.09E-16	5.17E-16	4.62E-16	4.41E-16	4.48E-16	4.30E-16	4.74E-16
5.69E-16	5.89E-16	5.74E-16	5.01E-16	5.04E-16	4.47E-16	4.69E-16	5.07E-16	5.04E-16	4.61E-16	4.38E-16	4.37E-16	4.25E-16	4.67E-16
5.41E-16	5.49E-16	5.33E-16	4.94E-16	4.77E-16	4.41E-16	4.58E-16	5.37E-16	5.11E-16	4.66E-16	4.58E-16	4.42E-16	4.31E-16	4.52E-16
5.14E-16	5.23E-16	4.97E-16	4.90E-16	4.73E-16	4.63E-16	4.64E-16	5.64E-16	5.28E-16	4.72E-16	4.73E-16	4.31E-16	4.16E-16	4.12E-16
5.06E-16	5.09E-16	4.69E-16	4.93E-16	4.64E-16	4.63E-16	4.81E-16	5.65E-16	5.18E-16	4.70E-16	4.64E-16	4.12E-16	3.78E-16	3.85E-16
4.91E-16	4.85E-16	4.45E-16	4.81E-16	4.65E-16	4.64E-16	4.98E-16	5.42E-16	4.83E-16	4.51E-16	4.56E-16	3.97E-16	3.52E-16	3.60E-16
4.75E-16	4.70E-16	4.56E-16	4.84E-16	4.54E-16	4.62E-16	4.72E-16	5.02E-16	4.72E-16	4.40E-16	4.67E-16	4.06E-16	3.56E-16	3.45E-16
4.76E-16	4.64E-16	4.71E-16	4.96E-16	4.37E-16	4.43E-16	4.49E-16	4.89E-16	4.57E-16	4.38E-16	4.41E-16	4.12E-16	3.57E-16	3.44E-16
4.80E-16	4.43E-16	4.60E-16	4.79E-16	4.38E-16	4.15E-16	4.09E-16	4.63E-16	4.25E-16	4.26E-16	4.14E-16	3.98E-16	3.42E-16	3.60E-16
4.78E-16	4.38E-16	4.50E-16	4.49E-16	4.37E-16	4.10E-16	3.79E-16	4.34E-16	4.12E-16	4.01E-16	3.94E-16	3.92E-16	3.34E-16	3.54E-16
4.81E-16	4.51E-16	4.54E-16	4.32E-16	4.39E-16	4.17E-16	3.69E-16	3.95E-16	3.76E-16	3.71E-16	3.78E-16	3.68E-16	3.30E-16	3.21E-16
4.79E-16	4.67E-16	4.58E-16	4.27E-16	4.29E-16	4.06E-16	3.55E-16	3.31E-16	3.23E-16	3.52E-16	3.62E-16	3.51E-16	3.39E-16	3.01E-16
4.58E-16	4.60E-16	4.47E-16	4.26E-16	4.16E-16	3.97E-16	3.53E-16	2.92E-16	2.91E-16	3.42E-16	3.64E-16	3.45E-16	3.56E-16	3.09E-16
4.33E-16	4.43E-16	4.37E-16	4.25E-16	3.93E-16	3.74E-16	3.42E-16	2.93E-16	3.04E-16	3.38E-16	3.57E-16	3.44E-16	3.60E-16	3.39E-16
4.20E-16	4.16E-16	4.21E-16	4.08E-16	3.66E-16	3.45E-16	3.35E-16	3.16E-16	3.43E-16	3.43E-16	3.47E-16	3.23E-16	3.40E-16	3.38E-16
3.95E-16	3.81E-16	3.82E-16	3.69E-16	3.37E-16	3.37E-16	3.19E-16	3.23E-16	3.61E-16	3.36E-16	3.39E-16	3.27E-16	3.26E-16	3.19E-16
3.71E-16	3.51E-16	3.52E-16	3.27E-16	3.18E-16	3.28E-16	3.15E-16	3.14E-16	3.62E-16	3.45E-16	3.36E-16	3.39E-16	3.24E-16	3.13E-16
3.63E-16	3.29E-16	3.49E-16	3.07E-16	3.09E-16	3.31E-16	3.06E-16	3.10E-16	3.59E-16	3.48E-16	3.28E-16	3.18E-16	3.10E-16	3.04E-16
3.70E-16	3.31E-16	3.43E-16	3.23E-16	3.15E-16	3.19E-16	3.03E-16	3.04E-16	3.54E-16	3.50E-16	3.23E-16	3.04E-16	2.71E-16	2.84E-16
3.70E-16	3.30E-16	3.30E-16	3.50E-16	3.21E-16	3.00E-16	3.18E-16	2.98E-16	3.36E-16	3.10E-16	3.03E-16	2.74E-16	2.58E-16	2.64E-16
3.58E-16	3.16E-16	3.08E-16	3.39E-16	3.18E-16	2.97E-16	3.24E-16	3.07E-16	3.18E-16	2.86E-16	2.84E-16	2.62E-16	2.77E-16	2.53E-16
3.39E-16	2.90E-16	2.98E-16	3.19E-16	3.17E-16	2.92E-16	3.27E-16	2.99E-16	3.08E-16	2.73E-16	2.84E-16	2.49E-16	2.73E-16	2.27E-16
3.14E-16	2.71E-16	2.95E-16	3.14E-16	2.88E-16	2.71E-16	3.09E-16	2.94E-16	2.99E-16	2.63E-16	2.68E-16	2.54E-16	2.44E-16	2.04E-16
2.88E-16	2.88E-16	3.04E-16	3.09E-16	2.73E-16	2.64E-16	2.97E-16	2.79E-16	2.92E-16	2.55E-16	2.47E-16	2.31E-16	1.96E-16	2.10E-16
2.72E-16	2.91E-16	3.06E-16	2.89E-16	2.78E-16	2.56E-16	2.77E-16	2.64E-16	2.84E-16	2.39E-16	2.19E-16	2.00E-16	1.43E-16	2.09E-16
2.63E-16	2.77E-16	2.93E-16	2.74E-16	2.78E-16	2.69E-16	2.65E-16	2.45E-16	2.58E-16	2.21E-16	1.94E-16	1.85E-16	1.12E-16	1.92E-16
2.59E-16	2.65E-16	2.61E-16	2.58E-16	2.65E-16	2.65E-16	2.48E-16	2.25E-16	2.26E-16	2.01E-16	1.74E-16	1.81E-16	1.88E-16	1.99E-16
2.48E-16	2.63E-16	2.47E-16	2.43E-16	2.46E-16	2.39E-16	2.30E-16	2.04E-16	2.06E-16	2.03E-16	2.00E-16	1.72E-16	1.88E-16	1.94E-16

2.34E-16	2.53E-16	2.48E-16	2.30E-16	2.31E-16	2.12E-16	2.16E-16	1.97E-16	1.93E-16	2.04E-16	1.90E-16	1.66E-16	1.87E-16	1.83E-16
2.29E-16	2.38E-16	2.22E-16	2.12E-16	2.18E-16	1.96E-16	2.02E-16	1.98E-16	1.79E-16	1.88E-16	1.84E-16	1.64E-16	1.82E-16	1.75E-16
2.16E-16	2.09E-16	2.05E-16	2.01E-16	2.07E-16	1.80E-16	1.77E-16	1.86E-16	1.78E-16	1.67E-16	1.58E-16	1.62E-16	1.78E-16	1.74E-16
2.02E-16	2.01E-16	1.89E-16	1.99E-16	1.98E-16	1.80E-16	1.59E-16	1.62E-16	1.64E-16	1.61E-16	1.41E-16	1.51E-16	1.69E-16	1.66E-16
1.90E-16	1.89E-16	1.94E-16	1.93E-16	1.91E-16	1.81E-16	1.42E-16	1.46E-16	1.59E-16	1.57E-16	1.46E-16	1.47E-16	1.54E-16	1.51E-16
1.83E-16	1.91E-16	1.92E-16	1.86E-16	1.80E-16	1.75E-16	1.33E-16	1.43E-16	1.64E-16	1.66E-16	1.58E-16	1.54E-16	1.40E-16	1.42E-16
1.81E-16	1.89E-16	1.81E-16	1.67E-16	1.59E-16	1.63E-16	1.32E-16	1.49E-16	1.63E-16	1.65E-16	1.57E-16	1.59E-16	1.40E-16	1.33E-16
1.78E-16	1.81E-16	1.72E-16	1.64E-16	1.46E-16	1.55E-16	1.36E-16	1.50E-16	1.49E-16	1.55E-16	1.50E-16	1.46E-16	1.35E-16	1.21E-16
1.66E-16	1.79E-16	1.69E-16	1.57E-16	1.43E-16	1.45E-16	1.32E-16	1.54E-16	1.31E-16	1.40E-16	1.44E-16	1.27E-16	1.07E-16	1.06E-16
1.60E-16	1.72E-16	1.73E-16	1.52E-16	1.51E-16	1.52E-16	1.36E-16	1.51E-16	1.29E-16	1.32E-16	1.39E-16	1.01E-16	9.07E-17	1.02E-16
1.59E-16	1.62E-16	1.70E-16	1.56E-16	1.61E-16	1.60E-16	1.35E-16	1.37E-16	1.20E-16	1.14E-16	1.19E-16	8.51E-17	7.17E-17	9.66E-17
1.64E-16	1.70E-16	1.77E-16	1.59E-16	1.67E-16	1.66E-16	1.28E-16	1.22E-16	1.06E-16	9.90E-17	1.09E-16	8.52E-17	7.04E-17	8.76E-17
1.61E-16	1.77E-16	1.76E-16	1.51E-16	1.60E-16	1.57E-16	1.07E-16	1.02E-16	9.78E-17	1.07E-16	1.13E-16	9.25E-17	7.61E-17	8.13E-17
1.45E-16	1.60E-16	1.58E-16	1.42E-16	1.42E-16	1.42E-16	8.26E-17	7.73E-17	8.63E-17	9.58E-17	9.85E-17	9.25E-17	8.31E-17	7.59E-17
1.26E-16	1.38E-16	1.38E-16	1.34E-16	1.29E-16	1.19E-16	7.07E-17	4.80E-17	7.72E-17	7.84E-17	7.30E-17	9.54E-17	8.87E-17	7.37E-17
1.10E-16	1.26E-16	1.16E-16	1.16E-16	1.17E-16	1.16E-16	7.58E-17	5.18E-17	7.73E-17	7.81E-17	6.08E-17	9.04E-17	8.60E-17	8.56E-17
1.14E-16	1.27E-16	1.31E-16	1.30E-16	1.32E-16	1.10E-16	8.28E-17	9.00E-17	7.69E-17	7.60E-17	6.05E-17	8.57E-17	7.43E-17	7.46E-17
1.24E-16	1.20E-16	1.33E-16	1.15E-16	1.16E-16	9.65E-17	8.33E-17	8.26E-17	6.66E-17	7.69E-17	6.49E-17	8.19E-17	6.77E-17	6.73E-17
1.15E-16	1.14E-16	1.12E-16	1.01E-16	1.05E-16	9.03E-17	8.52E-17	7.82E-17	6.79E-17	7.70E-17	7.10E-17	6.97E-17	5.71E-17	6.34E-17
1.05E-16	1.05E-16	9.83E-17	9.19E-17	1.00E-16	8.54E-17	8.06E-17	7.30E-17	7.74E-17	7.83E-17	7.07E-17	5.94E-17	5.14E-17	6.24E-17
9.74E-17	9.51E-17	9.30E-17	8.44E-17	9.02E-17	7.59E-17	6.86E-17	7.32E-17	8.11E-17	7.19E-17	6.52E-17	5.70E-17	5.06E-17	5.91E-17
8.23E-17	7.46E-17	8.34E-17	7.41E-17	7.42E-17	5.86E-17	5.85E-17	6.66E-17	6.87E-17	6.36E-17	5.80E-17	5.04E-17	4.73E-17	5.16E-17
7.05E-17	5.68E-17	6.40E-17	5.54E-17	5.68E-17	5.09E-17	4.88E-17	5.25E-17	5.56E-17	5.33E-17	4.35E-17	3.86E-17	3.77E-17	3.75E-17
5.68E-17	3.79E-17	4.26E-17	3.92E-17	3.73E-17	3.90E-17	3.54E-17	3.75E-17	3.75E-17	4.11E-17	3.06E-17	2.69E-17	2.64E-17	2.48E-17
4.29E-17	3.40E-17	3.08E-17	2.65E-17	2.42E-17	2.38E-17	2.44E-17	2.27E-17	2.37E-17	2.69E-17	1.96E-17	1.96E-17	1.81E-17	1.58E-17
3.73E-17	3.09E-17	3.41E-17	2.05E-17	1.63E-17	1.51E-17	2.00E-17	1.37E-17	1.46E-17	1.44E-17	1.25E-17	1.30E-17	1.25E-17	8.66E-18
3.20E-17	2.36E-17	2.98E-17	1.61E-17	1.42E-17	1.07E-17	1.28E-17	9.99E-18	1.04E-17	5.29E-18	5.96E-18	5.89E-18	7.15E-18	2.56E-18
2.50E-17	1.90E-17	2.23E-17	1.21E-17	1.22E-17	7.56E-18	6.30E-18	4.94E-18	4.27E-18	1.63E-18	1.32E-18	1.34E-18	3.05E-18	2.03E-19
1.94E-17	1.35E-17	1.69E-17	1.11E-17	1.03E-17	5.81E-18	2.20E-18	4.37E-19	8.54E-19	9.50E-20	1.01E-20	4.30E-20	7.73E-19	9.93E-20
1.54E-17	7.85E-18	1.12E-17	8.78E-18	6.16E-18	3.57E-18	2.14E-19	9.03E-19	2.57E-19	6.15E-19	1.18E-18	2.42E-19	2.14E-20	6.26E-19
1.01E-17	5.26E-18	6.70E-18	3.41E-18	1.40E-18	5.47E-19	6.47E-19	5.38E-18	5.14E-18	4.13E-18	3.06E-18	8.53E-19	4.55E-19	5.41E-19
4.94E-18	1.71E-18	2.22E-18	5.56E-19	3.54E-20	3.12E-19	2.52E-18	8.64E-18	1.10E-17	7.20E-18	6.35E-18	1.07E-18	1.01E-18	8.03E-20
4.32E-19	5.63E-20	4.43E-21	1.62E-19	1.87E-18	2.03E-18	5.20E-18	9.56E-18	9.66E-18	6.22E-18	5.65E-18	2.53E-18	1.23E-18	5.68E-20
2.47E-18	2.28E-18	2.75E-18	3.29E-18	4.39E-18	3.32E-18	8.34E-18	8.77E-18	4.68E-18	2.19E-18	2.90E-18	4.24E-18	7.80E-19	1.29E-20
9.40E-18	5.99E-18	6.87E-18	4.78E-18	5.36E-18	4.63E-18	4.22E-18	3.57E-18	1.81E-18	3.10E-20	4.01E-19	5.23E-18	1.94E-21	2.05E-20
1.18E-17	7.00E-18	9.48E-18	3.69E-18	3.10E-18	3.83E-18	6.01E-19	5.92E-19	1.43E-18	1.23E-19	2.58E-19	4.97E-19	8.34E-19	3.69E-19
1.05E-17	9.34E-18	7.82E-18	2.12E-18	1.79E-18	1.08E-18	1.86E-19	1.32E-19	1.50E-19	1.31E-18	7.94E-19	3.83E-19	2.24E-18	1.04E-18
6.40E-18	8.44E-18	5.48E-18	1.20E-18	5.69E-19	3.29E-20	1.34E-18	7.92E-22	2.00E-19	3.71E-18	3.64E-18	2.08E-18	3.42E-18	2.63E-18
2.28E-18	4.21E-18	3.90E-18	5.25E-19	8.84E-20	2.46E-20	1.18E-18	4.85E-19	1.04E-18	3.86E-18	4.23E-18	2.52E-18	3.49E-18	3.64E-18
1.70E-18	2.99E-18	3.75E-18	6.73E-19	8.26E-20	2.48E-19	5.45E-19	5.51E-19	8.65E-19	2.02E-18	2.02E-18	1.84E-18	2.87E-18	2.49E-18
9.01E-19	1.53E-18	2.26E-18	8.22E-19	1.97E-19	2.27E-20	3.43E-19	3.04E-19	5.73E-19	8.83E-19	1.41E-18	7.91E-19	1.75E-18	1.46E-18
2.37E-19	8.64E-20	5.89E-19	2.63E-19	9.98E-20	2.79E-23	1.75E-19	2.93E-19	2.73E-19	3.62E-19	7.72E-19	3.12E-19	7.25E-19	7.80E-19
5.15E-19	1.22E-19	5.09E-19	1.71E-19	4.18E-20	7.39E-20	3.24E-22	3.90E-20	1.95E-20	1.48E-19	1.07E-19	2.05E-19	1.02E-19	9.04E-20
7.04E-19	6.09E-19	1.32E-18	1.02E-18	4.06E-19	4.07E-19	2.58E-19	8.64E-22	1.92E-20	2.11E-21	5.72E-21	3.75E-20	1.36E-21	3.32E-20
1.04E-18	1.38E-18	2.29E-18	1.27E-18	8.92E-19	1.22E-18	1.08E-18	1.31E-19	5.12E-20	4.28E-19	6.93E-23	2.93E-22	4.87E-21	1.53E-19
2.08E-18	1.76E-18	2.61E-18	1.09E-18	1.62E-18	1.78E-18	2.22E-18	4.88E-19	3.26E-19	6.74E-19	6.63E-20	1.64E-20	6.05E-19	4.67E-19
3.60E-18	2.54E-18	3.17E-18	1.68E-18	4.94E-18	2.18E-18	3.94E-18	1.33E-18	1.21E-18	1.21E-18	3.10E-19	2.16E-19	1.32E-18	8.06E-19
6.78E-18	4.55E-18	5.45E-18	4.51E-18	1.05E-17	4.51E-18	5.58E-18	3.78E-18	2.88E-18	1.94E-18	1.01E-18	1.72E-18	3.99E-18	1.22E-18
1.06E-17	9.38E-18	1.09E-17	7.27E-18	1.61E-17	6.48E-18	6.87E-18	6.55E-18	5.67E-18	2.83E-18	1.49E-18	4.23E-18	9.09E-18	3.65E-18
1.76E-17	1.52E-17	1.86E-17	1.25E-17	1.81E-17	8.55E-18	9.62E-18	9.05E-18	8.04E-18	4.07E-18	4.81E-18	7.77E-18	9.41E-18	4.46E-18
2.31E-17	2.07E-17	1.78E-17	1.49E-17	2.01E-17	1.38E-17	1.33E-17	1.21E-17	9.86E-18	4.57E-18	7.51E-18	1.10E-17	9.71E-18	5.79E-18
2.44E-17	2.58E-17	2.12E-17	1.89E-17	2.10E-17	1.93E-17	1.65E-17	1.29E-17	1.33E-17	8.67E-18	1.06E-17	1.22E-17	1.07E-17	8.95E-18
2.90E-17	3.15E-17	2.48E-17	2.49E-17	2.06E-17	2.20E-17	1.79E-17	1.57E-17	1.79E-17	1.53E-17	1.20E-17	1.45E-17	1.37E-17	1.27E-17
3.64E-17	3.75E-17	3.05E-17	2.99E-17	2.11E-17	2.51E-17	2.09E-17	1.71E-17	2.28E-17	2.28E-17	1.06E-17	1.58E-17	1.85E-17	1.88E-17
5.02E-17	4.28E-17	4.20E-17	3.86E-17	2.35E-17	2.50E-17	2.59E-17	2.41E-17	2.86E-17	2.29E-17	1.78E-17	1.68E-17	2.24E-17	2.69E-17
5.92E-17	4.73E-17	4.69E-17	4.55E-17	1.28E-17	2.38E-17	3.38E-17	3.37E-17	3.17E-17	2.76E-17	2.87E-17	2.19E-17	2.24E-17	3.17E-17
6.49E-17	4.95E-17	5.35E-17	4.52E-17	7.92E-18	2.48E-17	4.48E-17	5.03E-17	4.20E-17	3.71E-17	3.99E-17	3.03E-17	3.17E-17	4.29E-17
7.32E-17	5.76E-17	6.12E-17	5.88E-17	2.20E-17	4.22E-17	5.54E-17	5.94E-17	5.96E-17	4.47E-17	4.88E-17	4.96E-17	4.54E-17	4.96E-17
9.30E-17	6.95E-17	6.33E-17	7.74E-17	5.80E-17	6.37E-17	6.35E-17	6.20E-17	6.84E-17	5.70E-17	6.56E-17	6.62E-17	5.71E-17	5.98E-17
1.04E-16	8.50E-17	7.39E-17	9.07E-17	8.23E-17	7.22E-17	7.28E-17	7.06E-17	7.92E-17	7.66E-17	8.20E-17	7.52E-17	6.94E-17	7.04E-17
1.17E-16	1.09E-16	8.97E-17	9.41E-17	1.01E-16	8.30E-17	8.42E-17	9.06E-17	1.06E-16	9.90E-17	9.62E-17	8.70E-17	7.66E-17	8.26E-17
1.42E-16	1.43E-16	1.00E-16	1.13E-16	1.18E-16	9.77E-17	1.04E-16	1.17E-16	1.21E-16	1.03E-16	1.16E-16	9.84E-17	8.45E-17	9.15E-17
1.65E-16	1.63E-16	1.16E-16	1.32E-16	1.39E-16	1.22E-16	1.28E-16	1.43E-16	1.43E-16	1.30E-16	1.45E-16	1.19E-16	9.06E-17	9.66E-17
1.69E-16	1.66E-16	1.31E-16	1.47E-16	1.51E-16	1.50E-16	1.48E-16	1.60E-16	1.89E-16	1.89E-16	1.82E-16	1.55E-16	1.11E-16	1.13E-16
1.86E-16	1.67E-16	1.53E-16	1.67E-16	1.60E-16	1.64E-16	1.62E-16	1.93E-16	2.41E-16	2.63E-16	2.54E-16	2.01E-16	1.35E-16	1.19E-16
2.06E-16	1.93E-16	1.70E-16	1.74E-16	1.79E-16	1.81E-16	1.84E-16	2.28E-16	2.71E-16	3.22E-16	3.11E-16	2.35E-16	1.61E-16	1.40E-16
2.10E-16	2.26E-16	2.02E-16	2.05E-16	1.96E-16	2.07E-16	2.13E-16	2.56E-16	2.84E-16	3.48E-16	3.35E-16	2.61E-16	1.89E-16	1.63E-16
2.31E-16	2.76E-16	2.49E-16	2.40E-16	2.35E-16	2.45E-16	2.29E-16	2.78E-16	2.89E-16	3.29E-16	3.22E-16	2.82E-16	2.21E-16	1.95E-16
2.71E-16	3.33E-16	2.92E-16	2.77E-16	2.76E-16	2.72E-16	2.48E-16	2.83E-16	2.89E-16	3.23E-16	3.01E-16	2.87E-16	2.48E-16	2.21E-16
3.06E-16	3.52E-16	3.37E-16	3.03E-16	3.22E-16	2.98E-16	2.64E-16	2.93E-16	2.85E-16	3.33E-16	2.89E-16	2.74E-16	2.69E-16	2.38E-16
3.27E-16	3.29E-16	3.70E-16	3.28E-16	3.57E-16	3.25E-16	2.91E-16	3.13E-16	2.99E-16	3.05E-16	2.82E-16	2.82E-16	2.84E-16	2.51E-16
3.61E-16	3.40E-16	3.97E-16	3.52E-16	3.88E-16	3.66E-16	3.21E-16	3.37E-16	3.24E-16	3.42E-16	3.11E-16	3.14E-16	3.18E-16	2.76E-16
4.12E-16	3.78E-16	4.24E-16	3.84E-16	3.93E-16	3.94E-16	3.54E-16	3.68E-16	3.54E-16	3.96E-16	3.45E-16	3.32E-16	3.38E-16	3.05E-16
4.62E-16	4.22E-16	4.50E-16	3.98E-16	4.30E-16	4.12E-16	4.02E-16	4.08E-16	3.75E-16	4.15E-16	3.78E-16	3.50E-16	3.63E-16	3.33E-16
4.84E-16	4.56E-16	4.79E-16	4.38E-16	4.58E-16	4.43E-16	4.30E-16	4.43E-16	3.97E-16	4.22E-16	3.95E-16	3.64E-16	3.76E-16	3.66E-16
4.99E-16	4.91E-16	5.22E-16	4.78E-16	5.01E-16	4.89E-16	4.58E-16	4.74E-16	4.44E-16	4.45E-16	4.34E-16	3.90E-16	3.94E-16	3.99E-16
5.41E-16	5.37E-16	5.67E-16	5.12E-16	5.27E-16	5.31E-16	4.80E-16	4.93E-16	5.18E-16	4.85E-16	4.87E-16	4.13E-16	4.29E-16	4.26E-16
5.86E-16	5.71E-16	6.04E-16	5.64E-16	5.78E-16	5.80E-16	5.13E-16	5.19E-16	5.76E-16	5.24E-16	5.22E-16	4.41E-16	4.72E-16	4.59E-16
6.07E-16	6.28E-16	6.56E-16	6.28E-16	6.44E-16	6.40E-16	5.57E-16	5.70E-16	5.92E-16	5.47E-16	5.34E-16	4.95E-16	4.92E-16	4.87E-16
6.63E-16	6.76E-16	6.82E-16	6.68E-16	6.72E-16	6.61E-16	5.98E-16	6.16E-16	6.09E-16	5.82E-16	5.63E-16	5.37E-16	5.36E-16	5.20E-16
7.03E-16	7.04E-16	7.27E-16	6.98E-16	7.07E-16	6.82E-16	6.50E-16	6.62E-16	6.43E-16	6.30E-16	6.08E-16	5.84E-16	5.95E-16	5.72E-16
7.49E-16	7.34E-16	7.72E-16	7.39E-16	7.51E-16	7.40E-16	7.16E-16	7.16E-16	6.71E-16	6.58E-16	6.28E-16	6.39E-16	6.44E-16	6.17E-16
8.15E-16	7.65E-16	8.21E-16	7.73E-16	7.83E-16	8.22E-16	7.93E-16	7.76E-16	7.18E-16	7.03E-16	6.61E-16	6.89E-16	6.90E-16	6.81E-16
8.62E-16	8.37E-16	8.77E-16	8.37E-16	8.30E-16	8.56E-16	8.24E-16	8.17E-16	7.78E-16	7.65E-16	7.14E-16	7.58E-16	7.50E-16	7.31E-16
9.26E-16	9.10E-16	9.23E-16	8.96E-16	8.87E-16	8.78E-16	8.84E-16	8.59E-16	8.31E-16	8.44E-16	7.89E-16	8.15E-16	7.90E-16	7.82E-16
9.97E-16	9.95E-16	9.94E-16	9.72E-16	9.32E-16	9.28E-16	9.52E-16	8.93E-16	8.93E-16	9.05E-16	8.53E-16	8.82E-16	8.23E-16	8.29E-16

1.07E-15	1.07E-15	1.06E-15	1.06E-15	9.91E-16	9.98E-16	1.02E-15	9.53E-16	9.33E-16	9.53E-16	9.16E-16	9.59E-16	8.78E-16	8.77E-16
1.12E-15	1.13E-15	1.12E-15	1.14E-15	1.07E-15	1.05E-15	1.06E-15	1.02E-15	9.97E-16	9.92E-16	9.74E-16	1.01E-15	9.50E-16	9.47E-16
1.15E-15	1.18E-15	1.19E-15	1.24E-15	1.15E-15	1.11E-15	1.13E-15	1.10E-15	1.08E-15	1.04E-15	1.04E-15	1.05E-15	1.01E-15	1.02E-15
1.22E-15	1.22E-15	1.30E-15	1.35E-15	1.25E-15	1.22E-15	1.19E-15	1.14E-15	1.16E-15	1.11E-15	1.12E-15	1.13E-15	1.05E-15	1.08E-15
1.32E-15	1.29E-15	1.34E-15	1.37E-15	1.31E-15	1.31E-15	1.24E-15	1.19E-15	1.26E-15	1.16E-15	1.21E-15	1.19E-15	1.11E-15	1.13E-15
1.37E-15	1.39E-15	1.39E-15	1.40E-15	1.36E-15	1.37E-15	1.31E-15	1.25E-15	1.36E-15	1.24E-15	1.27E-15	1.26E-15	1.20E-15	1.21E-15
1.51E-15	1.50E-15	1.45E-15	1.46E-15	1.44E-15	1.39E-15	1.38E-15	1.36E-15	1.42E-15	1.33E-15	1.34E-15	1.34E-15	1.28E-15	1.32E-15
1.69E-15	1.60E-15	1.53E-15	1.54E-15	1.53E-15	1.45E-15	1.49E-15	1.44E-15	1.47E-15	1.43E-15	1.43E-15	1.45E-15	1.41E-15	1.44E-15
1.79E-15	1.69E-15	1.64E-15	1.64E-15	1.62E-15	1.56E-15	1.61E-15	1.53E-15	1.56E-15	1.52E-15	1.54E-15	1.55E-15	1.52E-15	1.55E-15
1.86E-15	1.76E-15	1.74E-15	1.75E-15	1.73E-15	1.68E-15	1.69E-15	1.61E-15	1.65E-15	1.63E-15	1.63E-15	1.61E-15	1.59E-15	1.62E-15
1.96E-15	1.84E-15	1.84E-15	1.87E-15	1.81E-15	1.78E-15	1.73E-15	1.70E-15	1.76E-15	1.72E-15	1.69E-15	1.66E-15	1.70E-15	1.68E-15
4.59E-14	4.51E-14	4.51E-14	4.40E-14	4.31E-14	4.20E-14	4.07E-14	4.07E-14	4.10E-14	4.00E-14	3.93E-14	3.81E-14	3.65E-14	3.63E-14

-2.17E-08	-2.25E-08	-2.26E-08	-2.26E-08	-2.17E-08	-2.15E-08	-2.12E-08	-2.06E-08	-2.06E-08	-2.05E-08	-2.01E-08	-2.09E-08	-2.09E-08	-2.04E-08
-2.21E-08	-2.22E-08	-2.19E-08	-2.16E-08	-2.11E-08	-2.14E-08	-2.10E-08	-2.07E-08	-2.09E-08	-2.06E-08	-2.00E-08	-2.07E-08	-2.12E-08	-2.07E-08
-2.24E-08	-2.21E-08	-2.16E-08	-2.11E-08	-2.03E-08	-2.06E-08	-2.09E-08	-2.09E-08	-2.09E-08	-2.08E-08	-2.00E-08	-2.09E-08	-2.10E-08	-2.03E-08
-2.21E-08	-2.18E-08	-2.16E-08	-2.08E-08	-1.99E-08	-2.04E-08	-2.04E-08	-2.05E-08	-2.08E-08	-2.05E-08	-2.05E-08	-2.11E-08	-2.10E-08	-2.01E-08
-2.13E-08	-2.11E-08	-2.09E-08	-2.03E-08	-1.96E-08	-2.06E-08	-2.03E-08	-2.02E-08	-2.03E-08	-1.99E-08	-2.02E-08	-2.08E-08	-2.06E-08	-2.01E-08
-2.07E-08	-2.06E-08	-2.01E-08	-1.91E-08	-1.89E-08	-2.02E-08	-2.02E-08	-2.03E-08	-2.04E-08	-1.96E-08	-1.98E-08	-2.02E-08	-2.04E-08	-2.02E-08
-2.04E-08	-2.03E-08	-1.96E-08	-1.85E-08	-1.87E-08	-2.00E-08	-1.98E-08	-2.01E-08	-2.04E-08	-2.01E-08	-2.02E-08	-2.02E-08	-2.01E-08	-1.98E-08
-2.04E-08	-2.02E-08	-1.92E-08	-1.80E-08	-1.85E-08	-1.97E-08	-1.98E-08	-2.01E-08	-2.02E-08	-2.00E-08	-2.03E-08	-2.03E-08	-2.02E-08	-1.96E-08
-1.98E-08	-2.02E-08	-1.91E-08	-1.80E-08	-1.86E-08	-1.98E-08	-2.03E-08	-2.05E-08	-2.01E-08	-2.00E-08	-1.99E-08	-2.00E-08	-1.99E-08	-1.93E-08
-1.93E-08	-1.98E-08	-1.91E-08	-1.87E-08	-1.90E-08	-2.00E-08	-2.05E-08	-2.07E-08	-2.02E-08	-1.97E-08	-1.93E-08	-1.94E-08	-1.92E-08	-1.87E-08
-1.86E-08	-1.97E-08	-1.90E-08	-1.94E-08	-1.93E-08	-1.99E-08	-2.04E-08	-2.02E-08	-1.97E-08	-1.91E-08	-1.86E-08	-1.86E-08	-1.84E-08	-1.83E-08
-1.89E-08	-1.99E-08	-1.91E-08	-1.94E-08	-1.92E-08	-1.95E-08	-2.00E-08	-1.96E-08	-1.92E-08	-1.89E-08	-1.82E-08	-1.82E-08	-1.77E-08	-1.78E-08
-1.91E-08	-1.99E-08	-1.95E-08	-1.95E-08	-1.89E-08	-1.91E-08	-1.96E-08	-1.94E-08	-1.92E-08	-1.91E-08	-1.81E-08	-1.77E-08	-1.70E-08	-1.73E-08
-1.90E-08	-1.95E-08	-1.96E-08	-1.94E-08	-1.88E-08	-1.87E-08	-1.91E-08	-1.94E-08	-1.97E-08	-1.89E-08	-1.79E-08	-1.69E-08	-1.62E-08	-1.72E-08
-1.83E-08	-1.94E-08	-1.94E-08	-1.95E-08	-1.91E-08	-1.88E-08	-1.86E-08	-1.87E-08	-1.96E-08	-1.87E-08	-1.79E-08	-1.58E-08	-1.63E-08	-1.73E-08
-1.76E-08	-1.88E-08	-1.91E-08	-1.90E-08	-1.88E-08	-1.89E-08	-1.77E-08	-1.84E-08	-1.88E-08	-1.83E-08	-1.77E-08	-1.58E-08	-1.61E-08	-1.73E-08
-1.72E-08	-1.83E-08	-1.93E-08	-1.85E-08	-1.81E-08	-1.93E-08	-1.85E-08	-1.81E-08	-1.78E-08	-1.76E-08	-1.78E-08	-1.66E-08	-1.58E-08	-1.67E-08
-1.75E-08	-1.81E-08	-1.89E-08	-1.86E-08	-1.84E-08	-1.90E-08	-1.80E-08	-1.76E-08	-1.73E-08	-1.72E-08	-1.78E-08	-1.65E-08	-1.60E-08	-1.61E-08
-1.71E-08	-1.77E-08	-1.85E-08	-1.80E-08	-1.84E-08	-1.82E-08	-1.76E-08	-1.72E-08	-1.70E-08	-1.68E-08	-1.75E-08	-1.62E-08	-1.58E-08	-1.56E-08
-1.68E-08	-1.75E-08	-1.82E-08	-1.79E-08	-1.80E-08	-1.72E-08	-1.67E-08	-1.67E-08	-1.65E-08	-1.63E-08	-1.62E-08	-1.52E-08	-1.55E-08	-1.57E-08
-1.72E-08	-1.75E-08	-1.75E-08	-1.74E-08	-1.72E-08	-1.62E-08	-1.61E-08	-1.63E-08	-1.63E-08	-1.58E-08	-1.54E-08	-1.44E-08	-1.50E-08	-1.56E-08
-1.72E-08	-1.72E-08	-1.70E-08	-1.67E-08	-1.66E-08	-1.56E-08	-1.57E-08	-1.58E-08	-1.54E-08	-1.50E-08	-1.50E-08	-1.44E-08	-1.52E-08	-1.57E-08
-1.67E-08	-1.64E-08	-1.67E-08	-1.62E-08	-1.60E-08	-1.51E-08	-1.51E-08	-1.49E-08	-1.47E-08	-1.47E-08	-1.49E-08	-1.45E-08	-1.53E-08	-1.52E-08
-1.59E-08	-1.58E-08	-1.66E-08	-1.58E-08	-1.57E-08	-1.43E-08	-1.46E-08	-1.43E-08	-1.37E-08	-1.43E-08	-1.44E-08	-1.45E-08	-1.48E-08	-1.44E-08
-1.48E-08	-1.55E-08	-1.57E-08	-1.50E-08	-1.54E-08	-1.38E-08	-1.39E-08	-1.40E-08	-1.28E-08	-1.34E-08	-1.39E-08	-1.47E-08	-1.44E-08	-1.38E-08
-1.41E-08	-1.52E-08	-1.50E-08	-1.46E-08	-1.54E-08	-1.38E-08	-1.30E-08	-1.35E-08	-1.25E-08	-1.25E-08	-1.32E-08	-1.43E-08	-1.38E-08	-1.35E-08
-1.44E-08	-1.54E-08	-1.47E-08	-1.45E-08	-1.52E-08	-1.38E-08	-1.25E-08	-1.33E-08	-1.32E-08	-1.22E-08	-1.27E-08	-1.32E-08	-1.31E-08	-1.33E-08
-1.46E-08	-1.50E-08	-1.49E-08	-1.45E-08	-1.48E-08	-1.36E-08	-1.25E-08	-1.33E-08	-1.35E-08	-1.25E-08	-1.24E-08	-1.28E-08	-1.26E-08	-1.32E-08
-1.41E-08	-1.44E-08	-1.50E-08	-1.44E-08	-1.43E-08	-1.34E-08	-1.28E-08	-1.34E-08	-1.35E-08	-1.29E-08	-1.24E-08	-1.26E-08	-1.26E-08	-1.30E-08
-1.38E-08	-1.36E-08	-1.43E-08	-1.38E-08	-1.42E-08	-1.29E-08	-1.26E-08	-1.31E-08	-1.29E-08	-1.25E-08	-1.21E-08	-1.22E-08	-1.22E-08	-1.28E-08
-1.34E-08	-1.29E-08	-1.37E-08	-1.32E-08	-1.35E-08	-1.26E-08	-1.22E-08	-1.26E-08	-1.24E-08	-1.22E-08	-1.22E-08	-1.16E-08	-1.19E-08	-1.24E-08
-1.33E-08	-1.25E-08	-1.28E-08	-1.29E-08	-1.25E-08	-1.21E-08	-1.14E-08	-1.25E-08	-1.21E-08	-1.16E-08	-1.21E-08	-1.09E-08	-1.18E-08	-1.14E-08
-1.32E-08	-1.23E-08	-1.19E-08	-1.21E-08	-1.21E-08	-1.19E-08	-1.01E-08	-1.15E-08	-1.19E-08	-1.12E-08	-1.16E-08	-1.01E-08	-1.15E-08	-1.04E-08
-1.27E-08	-1.19E-08	-1.12E-08	-1.10E-08	-1.13E-08	-1.11E-08	-8.96E-09	-1.08E-08	-1.17E-08	-1.11E-08	-1.10E-08	-1.02E-08	-1.09E-08	-9.88E-09
-1.23E-08	-1.13E-08	-1.08E-08	-1.03E-08	-1.03E-08	-9.92E-09	-8.08E-09	-1.05E-08	-1.16E-08	-1.08E-08	-1.08E-08	-1.06E-08	-1.06E-08	-9.53E-09
-1.23E-08	-1.07E-08	-1.01E-08	-9.60E-09	-9.42E-09	-9.03E-09	-7.92E-09	-1.02E-08	-1.15E-08	-1.09E-08	-1.10E-08	-1.05E-08	-1.01E-08	-9.13E-09
-1.19E-08	-1.04E-08	-9.53E-09	-9.16E-09	-8.78E-09	-8.77E-09	-8.34E-09	-9.86E-09	-1.13E-08	-1.06E-08	-1.05E-08	-1.04E-08	-1.03E-08	-9.77E-09
-1.12E-08	-9.94E-09	-8.88E-09	-8.78E-09	-7.98E-09	-7.83E-09	-7.25E-09	-9.09E-09	-1.06E-08	-1.03E-08	-1.05E-08	-1.08E-08	-1.15E-08	-1.09E-08
-1.08E-08	-9.50E-09	-8.25E-09	-7.73E-09	-6.50E-09	-5.49E-09	-5.79E-09	-7.97E-09	-1.08E-08	-1.07E-08	-1.11E-08	-1.13E-08	-1.27E-08	-1.13E-08
-1.03E-08	-9.37E-09	-7.89E-09	-6.78E-09	-4.16E-09	-3.44E-09	-3.86E-09	-6.53E-09	-1.08E-08	-1.12E-08	-1.22E-08	-1.17E-08	-1.39E-08	-1.17E-08
-1.02E-08	-9.07E-09	-8.04E-09	-7.39E-09	-4.72E-09	-4.36E-09	-2.02E-09	-4.36E-09	-8.02E-09	-9.30E-09	-1.30E-08	-1.28E-08	-1.54E-08	-1.20E-08
-9.98E-09	-8.64E-09	-7.61E-09	-8.17E-09	-6.33E-09	-6.15E-09	-2.15E-09	-3.25E-09	-4.89E-09	-7.50E-09	-1.14E-08	-1.31E-08	-1.49E-08	-1.30E-08
-9.42E-09	-8.19E-09	-7.54E-09	-7.95E-09	-7.89E-09	-7.70E-09	-3.63E-09	-3.60E-09	-3.85E-09	-6.26E-09	-8.54E-09	-1.15E-08	-1.36E-08	-1.38E-08
-9.41E-09	-8.49E-09	-7.93E-09	-7.47E-09	-7.62E-09	-7.62E-09	-5.70E-09	-5.03E-09	-4.12E-09	-4.76E-09	-7.28E-09	-9.57E-09	-1.20E-08	-1.29E-08
-9.23E-09	-9.24E-09	-8.49E-09	-7.19E-09	-7.36E-09	-7.32E-09	-7.40E-09	-6.96E-09	-5.82E-09	-4.94E-09	-6.99E-09	-8.73E-09	-1.03E-08	-1.15E-08
-9.08E-09	-9.36E-09	-8.85E-09	-7.65E-09	-7.46E-09	-7.25E-09	-8.53E-09	-7.79E-09	-6.66E-09	-6.29E-09	-6.90E-09	-8.09E-09	-9.55E-09	-1.01E-08
-8.88E-09	-9.06E-09	-8.73E-09	-7.83E-09	-7.69E-09	-7.37E-09	-9.19E-09	-7.85E-09	-7.08E-09	-7.72E-09	-7.14E-09	-7.72E-09	-8.35E-09	-8.16E-09
-8.56E-09	-8.91E-09	-8.36E-09	-8.40E-09	-7.80E-09	-7.46E-09	-9.72E-09	-7.86E-09	-6.87E-09	-8.11E-09	-7.42E-09	-7.45E-09	-7.45E-09	-8.05E-09
-8.03E-09	-8.48E-09	-8.04E-09	-8.04E-09	-7.31E-09	-7.07E-09	-1.03E-08	-7.91E-09	-6.44E-09	-7.60E-09	-6.67E-09	-6.21E-09	-6.82E-09	-7.41E-09
-7.48E-09	-8.51E-09	-7.85E-09	-7.16E-09	-6.41E-09	-6.71E-09	-9.53E-09	-7.65E-09	-5.56E-09	-6.29E-09	-5.23E-09	-4.86E-09	-5.53E-09	-6.29E-09
-7.13E-09	-7.80E-09	-7.19E-09	-5.91E-09	-5.72E-09	-6.26E-09	-8.51E-09	-5.39E-09	-4.26E-09	-4.73E-09	-3.66E-09	-2.33E-09	-3.98E-09	-5.12E-09
-6.90E-09	-7.03E-09	-6.89E-09	-5.31E-09	-5.21E-09	-5.43E-09	-6.93E-09	-3.52E-09	-2.41E-09	-2.73E-09	-2.14E-09	1.24E-11	-2.25E-09	-2.58E-09
-6.72E-09	-6.75E-09	-6.68E-09	-5.27E-09	-4.75E-09	-4.47E-09	-4.49E-09	-2.95E-09	-4.36E-10	5.07E-10	3.42E-10	2.11E-09	1.03E-09	3.19E-09
-6.07E-09	-6.53E-09	-5.95E-09	-5.28E-09	-5.18E-09	-4.25E-09	-3.96E-09	-2.70E-09	1.81E-10	2.88E-09	3.19E-09	5.05E-09	5.61E-09	7.27E-09
-5.26E-09	-5.30E-09	-4.60E-09	-4.60E-09	-5.18E-09	-4.63E-09	-3.75E-09	-2.53E-09	-7.42E-10	1.39E-09	3.39E-09	5.68E-09	7.65E-09	9.47E-09
-3.98E-09	-3.81E-09	-3.49E-09	-3.67E-09	-4.44E-09	-4.14E-09	-3.73E-09	-2.96E-09	-1.61E-09	-8.41E-10	5.42E-10	2.54E-09	5.37E-09	6.59E-09
-2.67E-09	-2.71E-09	-2.56E-09	-2.74E-09	-3.21E-09	-3.05E-09	-3.85E-09	-4.13E-09	-2.45E-09	-2.66E-09	-1.44E-09	2.61E-10	3.04E-09	4.38E-09
-1.67E-09	-1.61E-09	-1.61E-09	-1.45E-09	-1.82E-09	-2.14E-09	-3.65E-09	-4.39E-09	-3.48E-09	-3.60E-09	-2.14E-09	-3.33E-10	2.57E-09	4.00E-09
-2.31E-10	-7.06E-10	-8.00E-10	-2.45E-10	-3.12E-10	-1.01E-09	-2.94E-09	-4.15E-09	-3.97E-09	-4.06E-09	-2.57E-09	-6.94E-10	1.86E-09	2.65E-09
9.14E-10	3.59E-10	6.24E-11	-1.37E-11	3.41E-10	-8.47E-10	-3.07E-09	-3.98E-09	-4.05E-09	-4.25E-09	-2.73E-09	-1.49E-09	-9.28E-11	-4.96E-10
1.18E-09	7.38E-10	2.84E-10	-8.28E-10	-4.11E-10	-1.11E-09	-3.44E-09	-4.00E-09	-3.91E-09	-3.81E-09	-3.00E-09	-2.18E-09	-1.36E-09	-2.21E-09
6.39E-10	4.55E-10	-3.76E-10	-1.68E-09	-2.29E-09	-1.67E-09	-2.83E-09	-3.56E-09	-3.38E-09	-3.41E-09	-2.95E-09	-2.51E-09	-2.48E-09	-2.90E-09
-5.18E-11	-1.62E-10	-1.10E-09	-2.18E-09	-3.23E-09	-2.49E-09	-2.31E-09	-2.88E-09	-3.01E-09	-3.43E-09	-3.03E-09	-2.82E-09	-2.99E-09	-3.12E-09
1.23E-11	-3.47E-10	-1.70E-09	-2.50E-09	-3.43E-09	-3.32E-09	-2.02E-09	-2.12E-09	-2.67E-09	-3.83E-09	-3.20E-09	-2.80E-09	-3.32E-09	-3.19E-09
2.25E-10	-7.28E-10	-2.31E-09	-2.58E-09	-3.52E-09	-3.33E-09	-1.85E-09	-1.46E-09	-2.35E-09	-3.71E-09	-3.27E-09	-2.90E-09	-3.46E-09	-3.37E-09
7.53E-11	-1.38E-09	-2.20E-09	-2.51E-09	-3.65E-09	-2.98E-09	-2.09E-09	-9.23E-10	-2.04E-09	-3.50E-09	-3.41E-09	-2.91E-09	-3.69E-09	-3.55E-09
5.03E-11	-1.71E-09	-1.94E-09	-2.36E-09	-3.34E-09	-2.13E-09	-2.30E-09	-1.38E-09	-2.12E-09	-3.12E-09	-3.43E-09	-3.23E-09	-3.44E-09	-3.20E-09
-1.15E-09	-1.91E-09	-2.03E-09	-1.99E-09	-3.28E-09	-2.34E-09	-2.49E-09	-2.07E-09	-2.32E-09	-2.35E-09	-3.33E-09	-3.50E-09	-3.11E-09	-3.16E-09
-1.77E-09	-1.77E-09	-2.10E-09	-1.46E-09	-3.14E-09	-2.70E-09	-2.92E-09	-2.34E-09	-2.27E-09	-2.29E-09	-2.80E-09	-3.74E-09	-3.56E-09	-3.44E-09
-1.77E-09	-1.63E-09	-1.71E-09	-1.25E-09	-2.74E-09	-2.54E-09	-2.34E-09	-1.99E-09	-2.09E-09	-2.53E-09	-2.51E-09	-3.45E-09	-3.66E-09	-3.39E-09
-1.23E-09	-1.31E-09	-1.16E-09	-1.09E-09	-1.87E-09	-2.12E-09	-2.09E-09	-1.68E-09	-1.93E-09	-2.30E-09	-2.07E-09	-2.83E-09	-3.59E-09	-2.99E-09
-1.03E-09	-7.05E-10	-6.72E-10	-7.16E-10	-1.35E-09	-1.51E-09	-1.91E-09	-1.36E-09	-1.75E-09	-2.31E-09	-2.27E-09	-2.23E-09	-3.24E-09	-2.59E-09
-1.01E-09	8.37E-11	1.09E-10	-4.67E-10	-1.05E-09	-1.15E-09	-1.65E-09	-1.20E-09	-1.61E-09	-2.02E-09	-2.36E-09	-1.76E-09	-2.75E-09	-2.15E-09
-5.14E-10	9.72E-10	1.07E-09	2.40E-10	-3.83E-10	-1.13E-09	-1.66E-09	-1.21E-09	-1.54E-09	-1.66E-09	-1.81E-09	-1.52E-09	-1.90E-09	-1.37E-09
-1.11E-10	1.83E-09	1.66E-09	5.78E-10	2.07E-10	-9.57E-10	-1.51E-09	-9.13E-10	-9.72E-10	-1.17E-09	-1.27E-09	-1.29E-09	-1.66E-09	-6.73E-10
-6.70E-11	2.14E-09	1.46E-09	7.39E-10	8.64E-10	-3.03E-10	-6.33E-10	2.58E-10	-3.73E-10	-7.07E-10	-7.65E-10	-1.23E-09	-1.45E-09	-7.38E-11
3.48E-10	1.54E-09	8.83E-10	7.16E-10	1.52E-09	6.84E-10	3.52E-10	9.41E-10	3.08E-10	-2.71E-10	4.39E-10	-9.41E-10	-1.24E-09	-2.14E-10
8.01E-10	1.45E-09	7.03E-10	6.36E-10	2.05E-09	1.03E-09	7.99E-10	9.98E-10	4.60E-10	2.10E-10	1.26E-09	-3.55E-10	-8.58E-10	-1.72E-11

1.16E-09	1.39E-09	9.31E-10	7.77E-10	1.49E-09	8.17E-10	1.16E-09	1.37E-09	3.76E-10	4.53E-10	1.58E-09	3.84E-10	-4.06E-10	3.08E-10
1.66E-09	1.56E-09	1.98E-09	1.14E-09	1.03E-09	8.03E-10	1.09E-09	1.38E-09	6.40E-10	7.13E-10	2.12E-09	4.90E-10	-7.72E-11	5.98E-10
2.05E-09	2.23E-09	2.85E-09	1.70E-09	1.57E-09	1.33E-09	1.16E-09	1.54E-09	1.46E-09	1.20E-09	1.34E-09	7.29E-10	1.47E-10	1.11E-09
2.64E-09	2.91E-09	3.48E-09	1.95E-09	2.28E-09	1.82E-09	1.31E-09	1.69E-09	2.03E-09	1.63E-09	1.69E-09	7.59E-10	4.96E-10	1.58E-09
3.19E-09	3.53E-09	3.54E-09	2.65E-09	3.33E-09	2.42E-09	1.68E-09	1.87E-09	2.20E-09	1.61E-09	2.30E-09	1.22E-09	9.93E-10	2.14E-09
3.61E-09	4.00E-09	3.81E-09	2.96E-09	4.00E-09	3.11E-09	2.31E-09	2.54E-09	2.35E-09	2.11E-09	2.47E-09	1.76E-09	1.77E-09	2.56E-09
4.21E-09	4.70E-09	4.25E-09	3.77E-09	4.56E-09	3.77E-09	3.14E-09	3.38E-09	2.55E-09	2.75E-09	2.74E-09	2.37E-09	2.54E-09	2.91E-09
4.90E-09	4.82E-09	4.49E-09	4.44E-09	4.69E-09	4.45E-09	3.74E-09	3.84E-09	3.31E-09	3.53E-09	3.41E-09	2.81E-09	3.22E-09	3.52E-09
5.00E-09	5.18E-09	4.62E-09	4.98E-09	4.87E-09	4.94E-09	4.46E-09	3.92E-09	3.87E-09	4.19E-09	4.01E-09	3.33E-09	3.94E-09	4.02E-09
5.82E-09	5.89E-09	5.38E-09	5.61E-09	5.24E-09	5.62E-09	4.93E-09	4.55E-09	4.53E-09	4.82E-09	4.35E-09	3.51E-09	4.65E-09	4.26E-09
7.15E-09	6.72E-09	6.13E-09	5.90E-09	5.65E-09	6.08E-09	5.26E-09	5.57E-09	5.41E-09	5.38E-09	4.84E-09	4.00E-09	4.94E-09	4.88E-09
8.21E-09	7.38E-09	6.90E-09	6.32E-09	6.38E-09	6.63E-09	5.66E-09	6.56E-09	5.78E-09	5.45E-09	5.20E-09	4.81E-09	5.27E-09	5.55E-09
8.47E-09	7.73E-09	8.12E-09	7.28E-09	7.09E-09	6.87E-09	6.32E-09	6.90E-09	6.25E-09	5.60E-09	6.12E-09	5.56E-09	5.80E-09	6.49E-09
8.77E-09	8.42E-09	9.19E-09	8.40E-09	7.91E-09	7.38E-09	6.96E-09	7.53E-09	7.26E-09	6.61E-09	6.99E-09	6.27E-09	6.55E-09	6.78E-09
9.39E-09	8.86E-09	9.44E-09	9.44E-09	8.45E-09	8.45E-09	8.11E-09	8.51E-09	8.51E-09	7.73E-09	7.87E-09	7.27E-09	7.47E-09	6.94E-09
9.81E-09	9.27E-09	9.37E-09	9.75E-09	9.28E-09	9.55E-09	9.34E-09	9.43E-09	9.45E-09	8.38E-09	8.76E-09	8.15E-09	8.18E-09	7.69E-09
1.05E-08	1.02E-08	9.98E-09	1.01E-08	1.02E-08	1.07E-08	1.08E-08	1.02E-08	1.02E-08	9.29E-09	9.77E-09	8.81E-09	8.55E-09	8.29E-09
1.09E-08	1.10E-08	1.06E-08	1.07E-08	1.08E-08	1.15E-08	1.23E-08	1.13E-08	1.12E-08	1.06E-08	1.08E-08	9.63E-09	9.50E-09	8.80E-09
1.20E-08	1.17E-08	1.14E-08	1.15E-08	1.13E-08	1.18E-08	1.27E-08	1.19E-08	1.19E-08	1.12E-08	1.15E-08	1.07E-08	1.02E-08	9.28E-09
1.30E-08	1.26E-08	1.20E-08	1.22E-08	1.22E-08	1.23E-08	1.29E-08	1.24E-08	1.26E-08	1.19E-08	1.19E-08	1.17E-08	1.09E-08	9.93E-09
1.42E-08	1.37E-08	1.27E-08	1.32E-08	1.33E-08	1.30E-08	1.33E-08	1.31E-08	1.35E-08	1.30E-08	1.20E-08	1.22E-08	1.13E-08	1.05E-08
1.52E-08	1.46E-08	1.34E-08	1.36E-08	1.41E-08	1.35E-08	1.38E-08	1.40E-08	1.44E-08	1.39E-08	1.24E-08	1.27E-08	1.21E-08	1.16E-08
1.60E-08	1.53E-08	1.46E-08	1.42E-08	1.45E-08	1.44E-08	1.47E-08	1.47E-08	1.53E-08	1.43E-08	1.33E-08	1.33E-08	1.32E-08	1.25E-08
1.67E-08	1.64E-08	1.61E-08	1.53E-08	1.47E-08	1.49E-08	1.55E-08	1.50E-08	1.51E-08	1.46E-08	1.41E-08	1.40E-08	1.41E-08	1.38E-08
1.73E-08	1.72E-08	1.73E-08	1.60E-08	1.51E-08	1.57E-08	1.61E-08	1.56E-08	1.53E-08	1.50E-08	1.51E-08	1.46E-08	1.53E-08	1.48E-08
1.81E-08	1.74E-08	1.77E-08	1.66E-08	1.59E-08	1.67E-08	1.68E-08	1.60E-08	1.63E-08	1.58E-08	1.58E-08	1.53E-08	1.59E-08	1.49E-08
1.86E-08	1.82E-08	1.86E-08	1.75E-08	1.72E-08	1.78E-08	1.75E-08	1.67E-08	1.71E-08	1.67E-08	1.65E-08	1.59E-08	1.59E-08	1.56E-08
1.96E-08	1.91E-08	1.93E-08	1.84E-08	1.82E-08	1.80E-08	1.83E-08	1.74E-08	1.78E-08	1.72E-08	1.72E-08	1.68E-08	1.67E-08	1.63E-08
2.06E-08	2.00E-08	1.96E-08	1.94E-08	1.86E-08	1.84E-08	1.91E-08	1.85E-08	1.84E-08	1.79E-08	1.80E-08	1.80E-08	1.78E-08	1.68E-08
2.13E-08	2.08E-08	2.01E-08	2.02E-08	1.94E-08	1.92E-08	1.98E-08	1.91E-08	1.88E-08	1.84E-08	1.87E-08	1.91E-08	1.87E-08	1.77E-08
2.19E-08	2.15E-08	2.12E-08	2.08E-08	2.00E-08	2.00E-08	2.01E-08	1.97E-08	1.94E-08	1.92E-08	1.95E-08	1.95E-08	1.92E-08	1.87E-08
2.26E-08	2.21E-08	2.23E-08	2.19E-08	2.07E-08	2.08E-08	2.05E-08	2.06E-08	2.04E-08	2.02E-08	2.04E-08	2.02E-08	2.00E-08	1.97E-08
2.32E-08	2.30E-08	2.35E-08	2.32E-08	2.16E-08	2.16E-08	2.12E-08	2.15E-08	2.15E-08	2.12E-08	2.14E-08	2.14E-08	2.10E-08	2.07E-08
2.42E-08	2.41E-08	2.44E-08	2.40E-08	2.29E-08	2.25E-08	2.20E-08	2.25E-08	2.22E-08	2.18E-08	2.23E-08	2.32E-08	2.18E-08	2.17E-08
2.52E-08	2.55E-08	2.59E-08	2.48E-08	2.40E-08	2.38E-08	2.28E-08	2.32E-08	2.33E-08	2.31E-08	2.31E-08	2.46E-08	2.25E-08	2.27E-08
2.59E-08	2.66E-08	2.63E-08	2.55E-08	2.53E-08	2.46E-08	2.41E-08	2.45E-08	2.44E-08	2.46E-08	2.36E-08	2.38E-08	2.39E-08	2.28E-08
2.66E-08	2.72E-08	2.65E-08	2.65E-08	2.65E-08	2.56E-08	2.52E-08	2.54E-08	2.51E-08	2.56E-08	2.45E-08	2.38E-08	2.44E-08	2.31E-08
2.72E-08	2.72E-08	2.69E-08	2.72E-08	2.73E-08	2.64E-08	2.61E-08	2.62E-08	2.57E-08	2.59E-08	2.51E-08	2.44E-08	2.46E-08	2.40E-08
2.82E-08	2.82E-08	2.81E-08	2.80E-08	2.82E-08	2.74E-08	2.66E-08	2.69E-08	2.67E-08	2.64E-08	2.60E-08	2.56E-08	2.58E-08	2.53E-08
2.93E-08	2.93E-08	2.94E-08	2.91E-08	2.94E-08	2.83E-08	2.77E-08	2.77E-08	2.75E-08	2.72E-08	2.69E-08	2.66E-08	2.67E-08	2.67E-08
3.02E-08	3.02E-08	3.05E-08	3.01E-08	2.99E-08	2.91E-08	2.87E-08	2.88E-08	2.86E-08	2.87E-08	2.80E-08	2.77E-08	2.78E-08	2.75E-08
3.11E-08	3.11E-08	3.11E-08	3.09E-08	3.06E-08	3.00E-08	2.98E-08	2.99E-08	2.93E-08	2.99E-08	2.90E-08	2.90E-08	2.88E-08	2.85E-08
3.24E-08	3.23E-08	3.21E-08	3.15E-08	3.13E-08	3.13E-08	3.07E-08	3.10E-08	2.99E-08	3.08E-08	3.00E-08	2.98E-08	2.98E-08	2.94E-08
3.36E-08	3.31E-08	3.31E-08	3.27E-08	3.25E-08	3.26E-08	3.14E-08	3.13E-08	3.05E-08	3.14E-08	3.10E-08	3.09E-08	3.03E-08	3.02E-08
3.45E-08	3.41E-08	3.41E-08	3.37E-08	3.38E-08	3.34E-08	3.21E-08	3.21E-08	3.14E-08	3.18E-08	3.19E-08	3.21E-08	3.10E-08	3.08E-08
3.58E-08	3.52E-08	3.48E-08	3.43E-08	3.47E-08	3.46E-08	3.33E-08	3.33E-08	3.22E-08	3.22E-08	3.28E-08	3.31E-08	3.20E-08	3.19E-08
3.75E-08	3.68E-08	3.58E-08	3.55E-08	3.54E-08	3.57E-08	3.50E-08	3.44E-08	3.35E-08	3.28E-08	3.36E-08	3.36E-08	3.34E-08	3.30E-08
3.90E-08	3.83E-08	3.67E-08	3.62E-08	3.61E-08	3.66E-08	3.62E-08	3.53E-08	3.51E-08	3.42E-08	3.47E-08	3.41E-08	3.47E-08	3.40E-08
3.94E-08	3.94E-08	3.76E-08	3.71E-08	3.70E-08	3.72E-08	3.72E-08	3.65E-08	3.67E-08	3.61E-08	3.59E-08	3.50E-08	3.55E-08	3.51E-08
4.05E-08	4.08E-08	3.85E-08	3.82E-08	3.80E-08	3.79E-08	3.83E-08	3.78E-08	3.82E-08	3.79E-08	3.69E-08	3.64E-08	3.65E-08	3.60E-08

4.72E-16	5.06E-16	5.13E-16	5.09E-16	4.71E-16	4.64E-16	4.47E-16	4.24E-16	4.26E-16	4.21E-16	4.03E-16	4.38E-16	4.35E-16	4.17E-16
4.87E-16	4.93E-16	4.78E-16	4.68E-16	4.45E-16	4.56E-16	4.41E-16	4.30E-16	4.36E-16	4.24E-16	3.99E-16	4.28E-16	4.47E-16	4.29E-16
5.03E-16	4.89E-16	4.67E-16	4.45E-16	4.12E-16	4.23E-16	4.36E-16	4.35E-16	4.37E-16	4.33E-16	3.99E-16	4.35E-16	4.43E-16	4.12E-16
4.88E-16	4.76E-16	4.65E-16	4.34E-16	3.98E-16	4.16E-16	4.16E-16	4.19E-16	4.31E-16	4.22E-16	4.18E-16	4.45E-16	4.40E-16	4.05E-16
4.53E-16	4.44E-16	4.36E-16	4.10E-16	3.84E-16	4.23E-16	4.13E-16	4.09E-16	4.12E-16	3.95E-16	4.08E-16	4.32E-16	4.23E-16	4.04E-16
4.30E-16	4.23E-16	4.03E-16	3.65E-16	3.57E-16	4.06E-16	4.08E-16	4.14E-16	4.17E-16	3.82E-16	3.91E-16	4.09E-16	4.14E-16	4.09E-16
4.17E-16	4.13E-16	3.86E-16	3.41E-16	3.48E-16	3.99E-16	3.93E-16	4.05E-16	4.17E-16	4.03E-16	4.09E-16	4.08E-16	4.03E-16	3.91E-16
4.16E-16	4.07E-16	3.70E-16	3.25E-16	3.42E-16	3.88E-16	3.91E-16	4.02E-16	4.07E-16	4.01E-16	4.14E-16	4.12E-16	4.08E-16	3.84E-16
3.92E-16	4.09E-16	3.63E-16	3.23E-16	3.45E-16	3.93E-16	4.11E-16	4.21E-16	4.04E-16	3.98E-16	3.97E-16	4.00E-16	3.97E-16	3.74E-16
3.71E-16	3.93E-16	3.66E-16	3.50E-16	3.61E-16	3.98E-16	4.21E-16	4.28E-16	4.10E-16	3.90E-16	3.72E-16	3.75E-16	3.68E-16	3.50E-16
3.47E-16	3.87E-16	3.60E-16	3.78E-16	3.72E-16	3.96E-16	4.16E-16	4.08E-16	3.87E-16	3.66E-16	3.46E-16	3.47E-16	3.37E-16	3.35E-16
3.56E-16	3.96E-16	3.65E-16	3.78E-16	3.69E-16	3.82E-16	4.01E-16	3.86E-16	3.68E-16	3.58E-16	3.31E-16	3.32E-16	3.13E-16	3.16E-16
3.64E-16	3.94E-16	3.80E-16	3.82E-16	3.58E-16	3.63E-16	3.84E-16	3.78E-16	3.68E-16	3.64E-16	3.28E-16	3.13E-16	2.88E-16	3.00E-16
3.63E-16	3.82E-16	3.84E-16	3.78E-16	3.52E-16	3.50E-16	3.66E-16	3.78E-16	3.88E-16	3.57E-16	3.21E-16	2.87E-16	2.64E-16	2.97E-16
3.36E-16	3.75E-16	3.75E-16	3.82E-16	3.67E-16	3.55E-16	3.46E-16	3.51E-16	3.83E-16	3.51E-16	3.21E-16	2.50E-16	2.65E-16	3.00E-16
3.09E-16	3.55E-16	3.64E-16	3.60E-16	3.53E-16	3.57E-16	3.14E-16	3.37E-16	3.53E-16	3.35E-16	3.14E-16	2.50E-16	2.60E-16	3.00E-16
2.96E-16	3.36E-16	3.71E-16	3.41E-16	3.28E-16	3.71E-16	3.40E-16	3.28E-16	3.18E-16	3.10E-16	3.19E-16	2.76E-16	2.49E-16	2.78E-16
3.06E-16	3.29E-16	3.58E-16	3.47E-16	3.37E-16	3.62E-16	3.24E-16	3.10E-16	3.00E-16	2.96E-16	3.15E-16	2.74E-16	2.56E-16	2.60E-16
2.93E-16	3.14E-16	3.43E-16	3.25E-16	3.40E-16	3.31E-16	3.10E-16	2.96E-16	2.87E-16	2.81E-16	3.07E-16	2.61E-16	2.51E-16	2.45E-16
2.84E-16	3.06E-16	3.31E-16	3.20E-16	3.24E-16	2.96E-16	2.80E-16	2.78E-16	2.73E-16	2.66E-16	2.62E-16	2.30E-16	2.39E-16	2.45E-16
2.94E-16	3.07E-16	3.05E-16	3.02E-16	2.96E-16	2.63E-16	2.59E-16	2.66E-16	2.65E-16	2.50E-16	2.38E-16	2.09E-16	2.24E-16	2.44E-16
2.97E-16	2.95E-16	2.90E-16	2.80E-16	2.75E-16	2.44E-16	2.45E-16	2.50E-16	2.38E-16	2.25E-16	2.24E-16	2.06E-16	2.31E-16	2.45E-16
2.80E-16	2.68E-16	2.80E-16	2.61E-16	2.57E-16	2.29E-16	2.28E-16	2.22E-16	2.16E-16	2.15E-16	2.22E-16	2.09E-16	2.34E-16	2.31E-16
2.52E-16	2.51E-16	2.74E-16	2.51E-16	2.47E-16	2.05E-16	2.12E-16	2.04E-16	1.87E-16	2.04E-16	2.07E-16	2.09E-16	2.20E-16	2.07E-16
2.19E-16	2.41E-16	2.47E-16	2.26E-16	2.38E-16	1.90E-16	1.94E-16	1.95E-16	1.65E-16	1.78E-16	1.92E-16	2.16E-16	2.07E-16	1.90E-16
2.00E-16	2.31E-16	2.26E-16	2.14E-16	2.36E-16	1.90E-16	1.70E-16	1.83E-16	1.57E-16	1.56E-16	1.73E-16	2.05E-16	1.91E-16	1.82E-16
2.06E-16	2.37E-16	2.17E-16	2.12E-16	2.31E-16	1.89E-16	1.57E-16	1.78E-16	1.73E-16	1.49E-16	1.62E-16	1.75E-16	1.70E-16	1.77E-16
2.15E-16	2.25E-16	2.23E-16	2.10E-16	2.18E-16	1.85E-16	1.55E-16	1.76E-16	1.82E-16	1.57E-16	1.55E-16	1.63E-16	1.58E-16	1.75E-16
2.00E-16	2.08E-16	2.24E-16	2.06E-16	2.03E-16	1.78E-16	1.64E-16	1.79E-16	1.82E-16	1.66E-16	1.54E-16	1.59E-16	1.59E-16	1.70E-16
1.92E-16	1.86E-16	2.06E-16	1.89E-16	2.00E-16	1.67E-16	1.59E-16	1.71E-16	1.67E-16	1.55E-16	1.46E-16	1.49E-16	1.50E-16	1.65E-16
1.78E-16	1.67E-16	1.88E-16	1.75E-16	1.83E-16	1.58E-16	1.49E-16	1.59E-16	1.55E-16	1.49E-16	1.49E-16	1.35E-16	1.41E-16	1.55E-16

1.77E-16	1.57E-16	1.64E-16	1.68E-16	1.57E-16	1.47E-16	1.29E-16	1.56E-16	1.45E-16	1.35E-16	1.46E-16	1.18E-16	1.40E-16	1.30E-16
1.73E-16	1.50E-16	1.42E-16	1.46E-16	1.46E-16	1.42E-16	1.02E-16	1.32E-16	1.41E-16	1.26E-16	1.35E-16	1.02E-16	1.33E-16	1.09E-16
1.62E-16	1.42E-16	1.26E-16	1.21E-16	1.27E-16	1.24E-16	8.03E-17	1.17E-16	1.36E-16	1.22E-16	1.21E-16	1.04E-16	1.20E-16	9.77E-17
1.52E-16	1.28E-16	1.16E-16	1.06E-16	1.05E-16	9.84E-17	6.53E-17	1.10E-16	1.34E-16	1.17E-16	1.18E-16	1.12E-16	1.13E-16	9.09E-17
1.52E-16	1.15E-16	1.02E-16	9.22E-17	8.87E-17	8.15E-17	6.27E-17	1.05E-16	1.31E-16	1.20E-16	1.21E-16	1.10E-16	1.02E-16	8.34E-17
1.41E-16	1.08E-16	9.09E-17	8.39E-17	7.70E-17	7.68E-17	6.95E-17	9.72E-17	1.27E-16	1.13E-16	1.09E-16	1.08E-16	1.05E-16	9.54E-17
1.26E-16	9.87E-17	7.88E-17	7.72E-17	6.36E-17	6.13E-17	5.25E-17	8.26E-17	1.12E-16	1.06E-16	1.11E-16	1.18E-16	1.33E-16	1.19E-16
1.17E-16	9.02E-17	6.81E-17	5.97E-17	4.23E-17	3.02E-17	3.35E-17	6.35E-17	1.17E-16	1.15E-16	1.24E-16	1.29E-16	1.62E-16	1.27E-16
1.06E-16	8.78E-17	6.23E-17	4.59E-17	1.73E-17	1.18E-17	1.49E-17	4.26E-17	1.18E-16	1.26E-16	1.49E-16	1.37E-16	1.94E-16	1.36E-16
1.04E-16	8.22E-17	6.47E-17	5.46E-17	2.22E-17	1.90E-17	4.08E-18	1.90E-17	6.43E-17	8.65E-17	1.69E-16	1.63E-16	2.36E-16	1.44E-16
9.96E-17	7.46E-17	5.79E-17	6.67E-17	4.00E-17	3.78E-17	4.64E-18	1.06E-17	2.39E-17	5.63E-17	1.29E-16	1.73E-16	2.22E-16	1.70E-16
8.86E-17	6.71E-17	5.68E-17	6.32E-17	6.22E-17	5.92E-17	1.32E-17	1.30E-17	1.49E-17	3.92E-17	7.29E-17	1.33E-16	1.86E-16	1.91E-16
8.85E-17	7.22E-17	6.29E-17	5.58E-17	5.81E-17	5.81E-17	3.25E-17	2.53E-17	1.70E-17	2.27E-17	5.31E-17	9.15E-17	1.45E-16	1.67E-16
8.51E-17	8.54E-17	7.21E-17	5.17E-17	5.42E-17	5.36E-17	5.48E-17	4.85E-17	3.39E-17	2.44E-17	4.88E-17	7.62E-17	1.07E-16	1.31E-16
8.24E-17	8.76E-17	7.83E-17	5.85E-17	5.57E-17	5.26E-17	7.27E-17	6.07E-17	4.44E-17	3.96E-17	4.76E-17	6.54E-17	9.12E-17	1.01E-16
7.88E-17	8.22E-17	7.62E-17	6.13E-17	5.91E-17	5.43E-17	8.45E-17	6.17E-17	5.01E-17	5.95E-17	5.10E-17	5.96E-17	6.98E-17	6.66E-17
7.33E-17	7.93E-17	6.99E-17	7.05E-17	6.08E-17	5.57E-17	9.44E-17	6.18E-17	4.73E-17	6.57E-17	5.50E-17	5.55E-17	5.55E-17	6.48E-17
6.45E-17	7.20E-17	6.47E-17	6.47E-17	5.35E-17	5.00E-17	1.05E-16	6.25E-17	4.15E-17	5.78E-17	4.45E-17	3.86E-17	4.65E-17	5.49E-17
5.60E-17	7.24E-17	6.17E-17	5.13E-17	4.11E-17	4.51E-17	9.09E-17	5.85E-17	3.09E-17	3.96E-17	2.74E-17	2.36E-17	3.06E-17	3.96E-17
5.08E-17	6.09E-17	5.16E-17	3.50E-17	3.28E-17	3.92E-17	7.25E-17	2.91E-17	1.81E-17	2.24E-17	1.34E-17	5.41E-18	1.58E-17	2.62E-17
4.76E-17	4.95E-17	4.75E-17	2.82E-17	2.72E-17	2.94E-17	4.80E-17	1.24E-17	5.83E-18	7.44E-18	4.58E-18	1.55E-22	5.06E-18	6.66E-18
4.51E-17	4.55E-17	4.46E-17	2.78E-17	2.26E-17	1.99E-17	2.02E-17	8.71E-18	1.91E-19	2.57E-19	1.17E-19	4.44E-18	1.06E-18	1.02E-17
3.68E-17	4.26E-17	3.54E-17	2.79E-17	2.69E-17	1.80E-17	1.57E-17	7.27E-18	3.27E-20	8.31E-18	1.02E-17	2.55E-17	3.14E-17	5.29E-17
2.77E-17	2.81E-17	2.12E-17	2.12E-17	2.68E-17	2.15E-17	1.40E-17	6.42E-18	5.50E-19	1.94E-18	1.15E-17	3.23E-17	5.86E-17	8.96E-17
1.58E-17	1.45E-17	1.22E-17	1.35E-17	1.97E-17	1.71E-17	1.39E-17	8.77E-18	2.60E-18	7.08E-19	2.94E-19	6.45E-18	2.88E-17	4.35E-17
7.14E-18	7.35E-18	6.55E-18	7.52E-18	1.03E-17	9.33E-18	1.48E-17	1.70E-17	6.02E-18	7.09E-18	2.07E-18	6.84E-20	9.26E-18	1.92E-17
2.78E-18	2.58E-18	2.60E-18	2.10E-18	3.32E-18	4.56E-18	1.33E-17	1.93E-17	1.21E-17	1.30E-17	4.59E-18	1.11E-19	6.61E-18	1.60E-17
5.34E-20	4.98E-19	6.41E-19	5.98E-20	9.73E-20	1.03E-18	8.67E-18	1.73E-17	1.58E-17	1.64E-17	6.61E-18	4.82E-19	3.47E-18	7.00E-18
8.36E-19	1.29E-19	3.89E-21	1.88E-22	1.16E-19	7.17E-19	9.42E-18	1.59E-17	1.64E-17	1.81E-17	7.45E-18	2.22E-18	8.62E-21	2.46E-19
1.38E-18	5.45E-19	8.07E-20	6.86E-19	1.69E-19	1.22E-18	1.18E-17	1.60E-17	1.53E-17	1.45E-17	9.00E-18	4.74E-18	1.85E-18	4.87E-18
4.09E-19	2.07E-19	1.41E-19	2.82E-18	5.24E-18	2.79E-18	8.03E-18	1.27E-17	1.14E-17	1.16E-17	8.69E-18	6.30E-18	6.17E-18	8.43E-18
2.68E-21	2.61E-20	1.22E-18	4.74E-18	1.05E-17	6.20E-18	5.35E-18	8.27E-18	9.04E-18	1.17E-17	9.16E-18	7.94E-18	8.94E-18	9.75E-18
1.51E-22	1.21E-19	2.89E-18	6.25E-18	1.18E-17	1.10E-17	4.06E-18	4.47E-18	7.11E-18	1.47E-17	1.02E-17	7.87E-18	1.10E-17	1.02E-17
5.08E-20	5.29E-19	5.31E-18	6.63E-18	1.24E-17	1.11E-17	3.42E-18	2.14E-18	5.53E-18	1.38E-17	1.07E-17	8.44E-18	1.20E-17	1.13E-17
5.67E-21	1.90E-18	4.83E-18	6.29E-18	1.33E-17	8.85E-18	4.37E-18	8.52E-19	4.18E-18	1.23E-17	1.16E-17	8.48E-18	1.36E-17	1.26E-17
2.53E-21	2.94E-18	3.78E-18	5.57E-18	1.11E-17	4.55E-18	5.27E-18	1.89E-18	4.47E-18	9.72E-18	1.17E-17	1.04E-17	1.19E-17	1.02E-17
1.32E-18	3.64E-18	4.12E-18	3.96E-18	1.08E-17	5.48E-18	6.19E-18	4.30E-18	5.39E-18	5.51E-18	1.11E-17	1.22E-17	9.69E-18	9.98E-18
3.12E-18	3.15E-18	4.39E-18	2.14E-18	9.86E-18	7.31E-18	8.54E-18	5.46E-18	5.17E-18	5.27E-18	7.83E-18	1.40E-17	1.27E-17	1.18E-17
3.13E-18	2.65E-18	2.92E-18	1.55E-18	7.51E-18	6.47E-18	5.50E-18	3.98E-18	4.36E-18	6.38E-18	6.32E-18	1.19E-17	1.34E-17	1.15E-17
1.52E-18	1.72E-18	1.35E-18	1.18E-18	3.49E-18	4.50E-18	4.38E-18	2.82E-18	3.71E-18	5.30E-18	4.27E-18	8.03E-18	1.29E-17	8.95E-18
1.07E-18	4.97E-19	4.52E-19	5.13E-19	1.83E-18	2.29E-18	3.66E-18	1.84E-18	3.05E-18	5.36E-18	5.14E-18	4.96E-18	1.05E-17	6.72E-18
1.01E-18	7.00E-21	1.19E-20	2.18E-19	1.10E-18	1.33E-18	2.74E-18	1.43E-18	2.58E-18	4.08E-18	5.59E-18	3.08E-18	7.57E-18	4.61E-18
2.65E-19	9.45E-19	1.15E-18	5.78E-20	1.47E-19	1.27E-18	2.76E-18	1.46E-18	2.37E-18	2.77E-18	3.29E-18	2.32E-18	3.60E-18	1.87E-18
1.24E-20	3.35E-18	2.76E-18	3.34E-19	4.29E-20	9.17E-19	2.27E-18	8.34E-19	9.45E-19	1.36E-18	1.60E-18	1.67E-18	2.76E-18	4.53E-19
4.50E-21	4.58E-18	2.13E-18	5.47E-19	7.46E-19	9.21E-20	4.00E-19	6.66E-20	1.39E-19	5.00E-19	5.86E-19	1.51E-18	2.09E-18	5.45E-21
1.21E-19	2.38E-18	7.80E-19	5.13E-19	2.32E-18	4.68E-19	1.24E-19	8.86E-19	9.47E-20	7.35E-20	1.93E-19	8.85E-19	1.54E-18	4.57E-20
6.41E-19	2.09E-18	4.94E-19	4.04E-19	4.20E-18	1.05E-18	6.38E-19	9.96E-19	2.11E-19	4.41E-20	1.59E-18	1.26E-19	7.37E-19	2.96E-22
1.34E-18	1.94E-18	8.68E-19	6.04E-19	2.23E-18	6.67E-19	1.36E-18	1.87E-18	1.41E-19	2.05E-19	2.49E-18	1.48E-19	1.65E-19	9.51E-20
2.75E-18	2.44E-18	3.92E-18	1.31E-18	1.07E-18	6.45E-19	1.19E-18	1.89E-18	4.09E-19	5.09E-19	4.49E-18	2.40E-19	5.95E-21	3.58E-19
4.22E-18	4.96E-18	8.11E-18	2.88E-18	2.46E-18	1.78E-18	1.35E-18	2.38E-18	2.12E-18	1.44E-18	1.81E-18	5.32E-19	2.15E-20	1.22E-18
6.95E-18	8.46E-18	1.21E-17	3.79E-18	5.19E-18	3.30E-18	1.72E-18	2.86E-18	4.12E-18	2.67E-18	2.87E-18	5.76E-19	2.47E-19	2.49E-18
1.02E-17	1.25E-17	1.25E-17	7.01E-18	1.11E-17	5.88E-18	2.81E-18	3.51E-18	4.85E-18	2.59E-18	5.30E-18	1.48E-18	9.86E-19	4.56E-18
1.30E-17	1.60E-17	1.45E-17	8.76E-18	1.60E-17	9.69E-18	5.36E-18	6.44E-18	5.50E-18	4.44E-18	6.10E-18	3.08E-18	3.12E-18	6.57E-18
1.77E-17	2.21E-17	1.80E-17	1.42E-17	2.08E-17	1.42E-17	9.89E-18	1.14E-17	6.51E-18	7.55E-18	7.52E-18	5.62E-18	6.44E-18	8.45E-18
2.40E-17	2.33E-17	2.01E-17	1.97E-17	2.20E-17	1.98E-17	1.40E-17	1.48E-17	1.09E-17	1.25E-17	1.16E-17	7.89E-18	1.04E-17	1.24E-17
2.50E-17	2.68E-17	2.14E-17	2.48E-17	2.37E-17	2.44E-17	1.99E-17	1.53E-17	1.49E-17	1.76E-17	1.61E-17	1.11E-17	1.55E-17	1.61E-17
3.39E-17	3.47E-17	2.89E-17	3.15E-17	2.74E-17	3.15E-17	2.43E-17	2.07E-17	2.06E-17	2.32E-17	1.89E-17	1.23E-17	2.17E-17	1.82E-17
5.11E-17	4.52E-17	3.76E-17	3.48E-17	3.19E-17	3.70E-17	2.76E-17	3.10E-17	2.92E-17	2.90E-17	2.34E-17	1.60E-17	2.44E-17	2.39E-17
6.73E-17	5.45E-17	4.76E-17	4.00E-17	4.07E-17	4.40E-17	3.20E-17	4.31E-17	3.34E-17	2.97E-17	2.71E-17	2.31E-17	2.78E-17	3.08E-17
7.18E-17	5.97E-17	6.60E-17	5.30E-17	5.02E-17	4.72E-17	4.00E-17	4.77E-17	3.90E-17	3.14E-17	3.74E-17	3.09E-17	3.37E-17	4.21E-17
7.69E-17	7.08E-17	8.45E-17	7.05E-17	6.25E-17	5.44E-17	4.85E-17	5.67E-17	5.28E-17	4.36E-17	4.89E-17	3.93E-17	4.30E-17	4.59E-17
8.81E-17	7.84E-17	8.91E-17	8.91E-17	7.14E-17	7.15E-17	6.58E-17	7.24E-17	7.24E-17	5.97E-17	6.19E-17	5.28E-17	5.58E-17	4.81E-17
9.62E-17	8.59E-17	8.77E-17	9.51E-17	8.61E-17	9.12E-17	8.72E-17	8.90E-17	8.93E-17	7.02E-17	7.67E-17	6.64E-17	6.69E-17	5.92E-17
1.10E-16	1.04E-16	9.96E-17	1.03E-16	1.04E-16	1.14E-16	1.16E-16	1.04E-16	1.04E-16	8.63E-17	9.55E-17	7.77E-17	7.31E-17	6.88E-17
1.19E-16	1.20E-16	1.12E-16	1.15E-16	1.17E-16	1.33E-16	1.52E-16	1.27E-16	1.26E-16	1.12E-16	1.17E-16	9.28E-17	9.03E-17	7.74E-17
1.43E-16	1.36E-16	1.30E-16	1.32E-16	1.27E-16	1.40E-16	1.61E-16	1.43E-16	1.41E-16	1.25E-16	1.33E-16	1.14E-16	1.04E-16	8.62E-17
1.70E-16	1.58E-16	1.45E-16	1.48E-16	1.49E-16	1.51E-16	1.66E-16	1.53E-16	1.58E-16	1.42E-16	1.41E-16	1.38E-16	1.19E-16	9.85E-17
2.02E-16	1.88E-16	1.62E-16	1.75E-16	1.78E-16	1.68E-16	1.78E-16	1.73E-16	1.82E-16	1.68E-16	1.44E-16	1.48E-16	1.27E-16	1.10E-16
2.32E-16	2.12E-16	1.79E-16	1.86E-16	2.00E-16	1.82E-16	1.92E-16	1.96E-16	2.07E-16	1.92E-16	1.54E-16	1.61E-16	1.48E-16	1.35E-16
2.57E-16	2.36E-16	2.15E-16	2.01E-16	2.12E-16	2.06E-16	2.16E-16	2.16E-16	2.33E-16	2.04E-16	1.78E-16	1.76E-16	1.73E-16	1.57E-16
2.77E-16	2.68E-16	2.60E-16	2.35E-16	2.15E-16	2.21E-16	2.40E-16	2.24E-16	2.27E-16	2.13E-16	2.00E-16	1.95E-16	1.99E-16	1.90E-16
3.00E-16	2.95E-16	2.99E-16	2.57E-16	2.29E-16	2.47E-16	2.58E-16	2.42E-16	2.33E-16	2.26E-16	2.27E-16	2.14E-16	2.34E-16	2.18E-16
3.26E-16	3.03E-16	3.12E-16	2.75E-16	2.52E-16	2.78E-16	2.83E-16	2.56E-16	2.65E-16	2.50E-16	2.51E-16	2.35E-16	2.52E-16	2.21E-16
3.45E-16	3.31E-16	3.45E-16	3.06E-16	2.96E-16	3.16E-16	3.05E-16	2.80E-16	2.92E-16	2.77E-16	2.71E-16	2.54E-16	2.54E-16	2.42E-16
3.82E-16	3.64E-16	3.71E-16	3.40E-16	3.30E-16	3.24E-16	3.35E-16	3.03E-16	3.16E-16	2.97E-16	2.95E-16	2.82E-16	2.80E-16	2.67E-16
4.23E-16	3.98E-16	3.83E-16	3.77E-16	3.44E-16	3.39E-16	3.66E-16	3.43E-16	3.37E-16	3.19E-16	3.24E-16	3.24E-16	3.18E-16	2.83E-16
4.53E-16	4.35E-16	4.03E-16	4.09E-16	3.75E-16	3.70E-16	3.90E-16	3.66E-16	3.55E-16	3.38E-16	3.51E-16	3.66E-16	3.49E-16	3.14E-16
4.81E-16	4.60E-16	4.49E-16	4.32E-16	4.01E-16	3.99E-16	4.04E-16	3.88E-16	3.78E-16	3.67E-16	3.82E-16	3.81E-16	3.70E-16	3.50E-16
5.10E-16	4.88E-16	4.99E-16	4.79E-16	4.26E-16	4.31E-16	4.20E-16	4.23E-16	4.15E-16	4.08E-16	4.15E-16	4.09E-16	4.00E-16	3.88E-16
5.37E-16	5.30E-16	5.50E-16	5.40E-16	4.65E-16	4.65E-16	4.50E-16	4.63E-16	4.64E-16	4.48E-16	4.58E-16	4.58E-16	4.43E-16	4.28E-16
5.86E-16	5.79E-16	5.97E-16	5.76E-16	5.24E-16	5.05E-16	4.86E-16	5.05E-16	4.92E-16	4.76E-16	4.98E-16	5.38E-16	4.76E-16	4.73E-16
6.34E-16	6.48E-16	6.72E-16	6.15E-16	5.76E-16	5.69E-16	5.19E-16	5.38E-16	5.42E-16	5.35E-16	5.35E-16	6.07E-16	5.07E-16	5.14E-16
6.71E-16	7.10E-16	6.94E-16	6.50E-16	6.40E-16	6.06E-16	5.81E-16	5.98E-16	5.96E-16	6.03E-16	5.59E-16	5.66E-16	5.73E-16	5.21E-16
7.06E-16	7.39E-16	7.05E-16	7.04E-16	7.02E-16	6.58E-16	6.33E-16	6.44E-16	6.31E-16	6.57E-16	5.98E-16	5.65E-16	5.94E-16	5.32E-16
7.37E-16	7.40E-16	7.26E-16	7.40E-16	7.47E-16	6.95E-16	6.81E-16	6.84E-16	6.59E-16	6.72E-16	6.31E-16	5.98E-16	6.06E-16	5.77E-16
7.96E-16	7.97E-16	7.90E-16	7.87E-16	7.96E-16	7.52E-16	7.10E-16	7.23E-16	7.11E-16	6.97E-16	6.73E-16	6.55E-16	6.63E-16	6.41E-16

8.59E-16	8.59E-16	8.64E-16	8.44E-16	8.62E-16	8.00E-16	7.66E-16	7.68E-16	7.59E-16	7.42E-16	7.25E-16	7.09E-16	7.15E-16	7.12E-16
9.13E-16	9.09E-16	9.29E-16	9.03E-16	8.96E-16	8.48E-16	8.25E-16	8.29E-16	8.19E-16	8.22E-16	7.86E-16	7.66E-16	7.74E-16	7.54E-16
9.66E-16	9.70E-16	9.68E-16	9.52E-16	9.37E-16	9.00E-16	8.87E-16	8.95E-16	8.57E-16	8.94E-16	8.43E-16	8.40E-16	8.29E-16	8.11E-16
1.05E-15	1.04E-15	1.03E-15	9.95E-16	9.81E-16	9.83E-16	9.45E-16	9.60E-16	8.95E-16	9.48E-16	8.97E-16	8.90E-16	8.88E-16	8.66E-16
1.13E-15	1.10E-15	1.09E-15	1.07E-15	1.05E-15	1.06E-15	9.84E-16	9.78E-16	9.30E-16	9.86E-16	9.60E-16	9.56E-16	9.20E-16	9.10E-16
1.19E-15	1.17E-15	1.16E-15	1.14E-15	1.14E-15	1.11E-15	1.03E-15	1.03E-15	9.87E-16	1.01E-15	1.02E-15	1.03E-15	9.64E-16	9.49E-16
1.28E-15	1.24E-15	1.21E-15	1.18E-15	1.20E-15	1.20E-15	1.11E-15	1.11E-15	1.03E-15	1.03E-15	1.07E-15	1.09E-15	1.02E-15	1.02E-15
1.40E-15	1.36E-15	1.28E-15	1.26E-15	1.25E-15	1.27E-15	1.22E-15	1.18E-15	1.12E-15	1.07E-15	1.13E-15	1.13E-15	1.11E-15	1.09E-15
1.52E-15	1.46E-15	1.34E-15	1.31E-15	1.30E-15	1.34E-15	1.31E-15	1.24E-15	1.23E-15	1.17E-15	1.21E-15	1.16E-15	1.20E-15	1.15E-15
1.55E-15	1.55E-15	1.42E-15	1.38E-15	1.37E-15	1.38E-15	1.38E-15	1.33E-15	1.35E-15	1.30E-15	1.29E-15	1.22E-15	1.26E-15	1.23E-15
1.64E-15	1.67E-15	1.48E-15	1.46E-15	1.44E-15	1.44E-15	1.47E-15	1.43E-15	1.46E-15	1.43E-15	1.36E-15	1.32E-15	1.34E-15	1.30E-15
3.61E-14	3.59E-14	3.49E-14	3.36E-14	3.29E-14	3.25E-14	3.18E-14	3.16E-14	3.13E-14	3.07E-14	3.05E-14	3.01E-14	3.04E-14	2.95E-14

-2.00E-08	-2.02E-08	-1.95E-08	-1.95E-08	-1.92E-08	-1.89E-08	-1.87E-08	-1.87E-08	-1.88E-08	-1.88E-08	-1.85E-08	-1.78E-08	-1.81E-08	-1.79E-08
-2.03E-08	-1.99E-08	-1.98E-08	-1.98E-08	-1.91E-08	-1.81E-08	-1.89E-08	-1.85E-08	-1.82E-08	-1.85E-08	-1.84E-08	-1.78E-08	-1.78E-08	-1.76E-08
-2.03E-08	-1.96E-08	-1.99E-08	-1.97E-08	-1.89E-08	-1.83E-08	-1.90E-08	-1.79E-08	-1.73E-08	-1.79E-08	-1.80E-08	-1.72E-08	-1.71E-08	-1.70E-08
-2.00E-08	-1.95E-08	-1.96E-08	-1.96E-08	-1.90E-08	-1.87E-08	-1.88E-08	-1.71E-08	-1.69E-08	-1.73E-08	-1.74E-08	-1.73E-08	-1.67E-08	-1.66E-08
-2.00E-08	-2.00E-08	-1.95E-08	-1.94E-08	-1.93E-08	-1.91E-08	-1.82E-08	-1.69E-08	-1.70E-08	-1.72E-08	-1.69E-08	-1.69E-08	-1.62E-08	-1.59E-08
-1.98E-08	-2.02E-08	-1.98E-08	-1.92E-08	-1.91E-08	-1.90E-08	-1.79E-08	-1.68E-08	-1.71E-08	-1.74E-08	-1.67E-08	-1.64E-08	-1.57E-08	-1.58E-08
-1.95E-08	-1.98E-08	-1.95E-08	-1.90E-08	-1.86E-08	-1.86E-08	-1.78E-08	-1.71E-08	-1.73E-08	-1.75E-08	-1.68E-08	-1.65E-08	-1.57E-08	-1.56E-08
-1.90E-08	-1.90E-08	-1.89E-08	-1.89E-08	-1.85E-08	-1.83E-08	-1.76E-08	-1.73E-08	-1.75E-08	-1.75E-08	-1.68E-08	-1.64E-08	-1.57E-08	-1.55E-08
-1.88E-08	-1.86E-08	-1.83E-08	-1.84E-08	-1.82E-08	-1.78E-08	-1.72E-08	-1.74E-08	-1.74E-08	-1.73E-08	-1.66E-08	-1.60E-08	-1.52E-08	-1.52E-08
-1.84E-08	-1.86E-08	-1.83E-08	-1.83E-08	-1.81E-08	-1.75E-08	-1.68E-08	-1.69E-08	-1.72E-08	-1.69E-08	-1.59E-08	-1.57E-08	-1.52E-08	-1.53E-08
-1.77E-08	-1.81E-08	-1.82E-08	-1.85E-08	-1.84E-08	-1.76E-08	-1.65E-08	-1.64E-08	-1.62E-08	-1.62E-08	-1.53E-08	-1.52E-08	-1.53E-08	-1.52E-08
-1.73E-08	-1.79E-08	-1.80E-08	-1.83E-08	-1.80E-08	-1.77E-08	-1.68E-08	-1.63E-08	-1.55E-08	-1.55E-08	-1.45E-08	-1.44E-08	-1.49E-08	-1.52E-08
-1.69E-08	-1.79E-08	-1.81E-08	-1.79E-08	-1.72E-08	-1.71E-08	-1.67E-08	-1.65E-08	-1.57E-08	-1.53E-08	-1.41E-08	-1.38E-08	-1.40E-08	-1.46E-08
-1.63E-08	-1.74E-08	-1.79E-08	-1.76E-08	-1.64E-08	-1.63E-08	-1.63E-08	-1.61E-08	-1.53E-08	-1.51E-08	-1.43E-08	-1.40E-08	-1.36E-08	-1.36E-08
-1.61E-08	-1.65E-08	-1.74E-08	-1.71E-08	-1.57E-08	-1.56E-08	-1.58E-08	-1.55E-08	-1.49E-08	-1.47E-08	-1.41E-08	-1.44E-08	-1.39E-08	-1.32E-08
-1.59E-08	-1.53E-08	-1.64E-08	-1.62E-08	-1.45E-08	-1.52E-08	-1.53E-08	-1.53E-08	-1.48E-08	-1.44E-08	-1.39E-08	-1.40E-08	-1.38E-08	-1.32E-08
-1.56E-08	-1.43E-08	-1.51E-08	-1.53E-08	-1.35E-08	-1.45E-08	-1.48E-08	-1.47E-08	-1.49E-08	-1.46E-08	-1.38E-08	-1.36E-08	-1.37E-08	-1.38E-08
-1.57E-08	-1.46E-08	-1.44E-08	-1.49E-08	-1.26E-08	-1.44E-08	-1.46E-08	-1.42E-08	-1.41E-08	-1.43E-08	-1.35E-08	-1.34E-08	-1.35E-08	-1.33E-08
-1.57E-08	-1.55E-08	-1.53E-08	-1.52E-08	-1.37E-08	-1.46E-08	-1.46E-08	-1.41E-08	-1.34E-08	-1.38E-08	-1.31E-08	-1.30E-08	-1.29E-08	-1.28E-08
-1.55E-08	-1.57E-08	-1.58E-08	-1.59E-08	-1.48E-08	-1.49E-08	-1.45E-08	-1.35E-08	-1.25E-08	-1.34E-08	-1.29E-08	-1.27E-08	-1.25E-08	-1.25E-08
-1.51E-08	-1.55E-08	-1.51E-08	-1.57E-08	-1.49E-08	-1.45E-08	-1.41E-08	-1.32E-08	-1.21E-08	-1.27E-08	-1.29E-08	-1.25E-08	-1.23E-08	-1.24E-08
-1.53E-08	-1.52E-08	-1.48E-08	-1.53E-08	-1.43E-08	-1.37E-08	-1.37E-08	-1.33E-08	-1.23E-08	-1.20E-08	-1.28E-08	-1.21E-08	-1.22E-08	-1.22E-08
-1.50E-08	-1.48E-08	-1.46E-08	-1.47E-08	-1.36E-08	-1.32E-08	-1.33E-08	-1.33E-08	-1.24E-08	-1.21E-08	-1.22E-08	-1.17E-08	-1.20E-08	-1.19E-08
-1.42E-08	-1.42E-08	-1.41E-08	-1.39E-08	-1.32E-08	-1.32E-08	-1.33E-08	-1.29E-08	-1.22E-08	-1.19E-08	-1.15E-08	-1.13E-08	-1.12E-08	-1.14E-08
-1.37E-08	-1.36E-08	-1.32E-08	-1.30E-08	-1.29E-08	-1.31E-08	-1.29E-08	-1.25E-08	-1.20E-08	-1.15E-08	-1.11E-08	-1.09E-08	-1.07E-08	-1.07E-08
-1.36E-08	-1.29E-08	-1.26E-08	-1.24E-08	-1.25E-08	-1.24E-08	-1.23E-08	-1.21E-08	-1.17E-08	-1.09E-08	-1.10E-08	-1.09E-08	-1.02E-08	-1.02E-08
-1.35E-08	-1.27E-08	-1.24E-08	-1.22E-08	-1.16E-08	-1.13E-08	-1.15E-08	-1.17E-08	-1.14E-08	-1.08E-08	-1.09E-08	-1.05E-08	-9.93E-09	-9.77E-09
-1.33E-08	-1.24E-08	-1.22E-08	-1.21E-08	-1.08E-08	-1.03E-08	-1.09E-08	-1.12E-08	-1.12E-08	-1.08E-08	-1.02E-08	-9.83E-09	-9.13E-09	-9.11E-09
-1.28E-08	-1.22E-08	-1.17E-08	-1.19E-08	-1.04E-08	-9.31E-09	-1.06E-08	-1.08E-08	-1.07E-08	-1.02E-08	-9.27E-09	-8.90E-09	-8.01E-09	-8.79E-09
-1.24E-08	-1.22E-08	-1.15E-08	-1.16E-08	-1.08E-08	-9.83E-09	-1.03E-08	-1.04E-08	-1.01E-08	-9.02E-09	-8.52E-09	-8.42E-09	-7.41E-09	-8.64E-09
-1.15E-08	-1.14E-08	-1.13E-08	-1.12E-08	-1.14E-08	-1.08E-08	-1.02E-08	-9.96E-09	-9.88E-09	-8.32E-09	-8.39E-09	-8.46E-09	-8.34E-09	-8.12E-09
-1.04E-08	-1.08E-08	-1.08E-08	-1.07E-08	-1.10E-08	-1.09E-08	-1.01E-08	-9.55E-09	-9.98E-09	-8.78E-09	-8.47E-09	-8.53E-09	-8.37E-09	-7.39E-09
-9.71E-09	-1.03E-08	-1.01E-08	-1.03E-08	-1.07E-08	-1.06E-08	-1.01E-08	-9.14E-09	-9.86E-09	-9.08E-09	-8.05E-09	-8.39E-09	-8.52E-09	-6.75E-09
-9.19E-09	-9.86E-09	-9.64E-09	-9.97E-09	-1.02E-08	-1.02E-08	-9.46E-09	-8.96E-09	-9.55E-09	-8.87E-09	-8.29E-09	-8.54E-09	-8.07E-09	-6.12E-09
-8.75E-09	-1.02E-08	-9.29E-09	-9.62E-09	-9.78E-09	-9.74E-09	-8.40E-09	-8.27E-09	-8.92E-09	-8.77E-09	-8.42E-09	-8.37E-09	-7.38E-09	-5.70E-09
-9.34E-09	-9.86E-09	-9.13E-09	-9.43E-09	-9.73E-09	-9.34E-09	-7.78E-09	-7.71E-09	-8.78E-09	-8.48E-09	-8.46E-09	-8.26E-09	-7.26E-09	-5.69E-09
-1.00E-08	-9.81E-09	-9.16E-09	-9.21E-09	-8.96E-09	-8.59E-09	-7.24E-09	-7.35E-09	-8.58E-09	-8.37E-09	-8.44E-09	-8.27E-09	-6.99E-09	-5.81E-09
-1.04E-08	-9.67E-09	-9.00E-09	-8.83E-09	-8.05E-09	-7.71E-09	-6.77E-09	-6.85E-09	-8.00E-09	-8.08E-09	-8.34E-09	-8.66E-09	-6.84E-09	-6.01E-09
-1.06E-08	-9.51E-09	-8.82E-09	-8.05E-09	-6.95E-09	-6.61E-09	-6.14E-09	-6.85E-09	-7.18E-09	-7.42E-09	-8.22E-09	-8.41E-09	-7.36E-09	-6.05E-09
-1.05E-08	-9.43E-09	-8.89E-09	-7.56E-09	-6.01E-09	-5.90E-09	-5.63E-09	-6.80E-09	-6.64E-09	-6.44E-09	-7.89E-09	-8.05E-09	-7.33E-09	-6.44E-09
-1.07E-08	-9.61E-09	-9.80E-09	-8.08E-09	-5.78E-09	-5.41E-09	-5.91E-09	-6.73E-09	-6.20E-09	-5.54E-09	-7.42E-09	-7.83E-09	-7.42E-09	-6.64E-09
-1.15E-08	-1.00E-08	-1.03E-08	-8.83E-09	-6.86E-09	-5.93E-09	-6.50E-09	-6.77E-09	-6.02E-09	-4.69E-09	-7.33E-09	-7.76E-09	-7.17E-09	-6.75E-09
-1.28E-08	-1.07E-08	-1.06E-08	-8.97E-09	-8.35E-09	-7.50E-09	-6.90E-09	-6.52E-09	-6.15E-09	-4.14E-09	-6.99E-09	-8.03E-09	-7.03E-09	-7.17E-09
-1.34E-08	-1.08E-08	-1.07E-08	-9.14E-09	-9.01E-09	-8.29E-09	-7.11E-09	-6.19E-09	-5.97E-09	-5.01E-09	-6.81E-09	-8.37E-09	-7.09E-09	-7.52E-09
-1.27E-08	-1.09E-08	-1.04E-08	-9.52E-09	-9.01E-09	-8.38E-09	-7.29E-09	-5.69E-09	-5.74E-09	-6.17E-09	-7.13E-09	-8.16E-09	-7.19E-09	-7.80E-09
-1.21E-08	-1.16E-08	-1.03E-08	-9.83E-09	-9.27E-09	-8.63E-09	-7.90E-09	-5.33E-09	-6.36E-09	-7.60E-09	-8.01E-09	-8.30E-09	-7.81E-09	-7.71E-09
-1.05E-08	-1.13E-08	-1.08E-08	-1.00E-08	-9.78E-09	-8.65E-09	-8.08E-09	-5.94E-09	-7.75E-09	-8.70E-09	-9.16E-09	-8.67E-09	-8.90E-09	-8.39E-09
-9.87E-09	-9.82E-09	-9.94E-09	-9.97E-09	-9.01E-09	-7.78E-09	-6.84E-09	-6.47E-09	-8.21E-09	-9.56E-09	-9.91E-09	-8.68E-09	-9.81E-09	-9.21E-09
-9.08E-09	-7.42E-09	-7.37E-09	-8.07E-09	-4.92E-09	-5.11E-09	-6.26E-09	-7.11E-09	-8.51E-09	-9.46E-09	-9.92E-09	-8.59E-09	-9.99E-09	-9.62E-09
-6.38E-09	-5.11E-09	-4.36E-09	-3.99E-09	7.89E-11	-3.06E-09	-5.80E-09	-7.51E-09	-8.63E-09	-9.23E-09	-9.39E-09	-8.36E-09	-1.02E-08	-9.82E-09
-5.05E-09	-3.73E-09	-1.72E-09	1.22E-09	4.64E-09	-2.19E-09	-5.63E-09	-7.66E-09	-8.34E-09	-8.97E-09	-8.44E-09	-8.72E-09	-1.02E-08	-9.58E-09
-3.10E-09	-1.66E-09	2.93E-09	6.02E-09	5.19E-09	-1.48E-09	-5.58E-09	-7.22E-09	-7.76E-09	-8.65E-09	-8.16E-09	-8.48E-09	-9.45E-09	-9.02E-09
1.22E-09	2.26E-09	8.87E-09	1.04E-08	4.78E-09	-1.75E-09	-4.85E-09	-6.34E-09	-7.22E-09	-7.99E-09	-7.69E-09	-8.04E-09	-8.56E-09	-8.44E-09
6.72E-09	7.58E-09	1.29E-08	1.04E-08	4.41E-09	-2.21E-09	-4.24E-09	-5.44E-09	-6.77E-09	-7.51E-09	-7.33E-09	-8.32E-09	-8.32E-09	-8.43E-09
8.87E-09	9.67E-09	1.06E-08	7.80E-09	3.79E-09	-2.29E-09	-4.40E-09	-4.98E-09	-6.08E-09	-7.36E-09	-7.24E-09	-7.97E-09	-8.18E-09	-8.67E-09
7.06E-09	6.64E-09	5.87E-09	4.20E-09	1.80E-09	-1.83E-09	-3.39E-09	-4.75E-09	-5.55E-09	-7.42E-09	-7.18E-09	-7.26E-09	-7.57E-09	-8.80E-09
5.54E-09	4.64E-09	3.22E-09	1.52E-09	1.03E-10	-1.87E-09	-3.36E-09	-4.98E-09	-5.56E-09	-6.73E-09	-6.63E-09	-6.41E-09	-6.98E-09	-8.35E-09
4.78E-09	3.34E-09	1.82E-09	2.76E-10	-1.17E-09	-1.98E-09	-3.52E-09	-4.87E-09	-5.02E-09	-5.62E-09	-5.66E-09	-5.24E-09	-6.63E-09	-7.66E-09
3.01E-09	1.55E-09	3.01E-10	-4.14E-10	-1.59E-09	-2.57E-09	-3.71E-09	-4.30E-09	-4.40E-09	-4.68E-09	-5.06E-09	-4.81E-09	-5.98E-09	-6.92E-09
4.28E-10	3.90E-10	-2.25E-10	-9.41E-10	-1.66E-09	-2.58E-09	-3.11E-09	-3.66E-09	-3.95E-09	-4.11E-09	-4.62E-09	-4.62E-09	-5.77E-09	-6.19E-09
-9.96E-10	-5.50E-10	-5.46E-10	-1.43E-09	-1.71E-09	-1.87E-09	-2.84E-09	-3.57E-09	-3.32E-09	-3.82E-09	-4.56E-09	-4.31E-09	-5.40E-09	-5.70E-09
-1.59E-09	-1.04E-09	-8.26E-10	-1.67E-09	-1.72E-09	-1.27E-09	-2.89E-09	-3.30E-09	-3.17E-09	-3.85E-09	-4.54E-09	-3.66E-09	-4.63E-09	-5.02E-09
-1.83E-09	-1.61E-09	-1.16E-09	-1.76E-09	-1.84E-09	-1.44E-09	-2.89E-09	-3.16E-09	-3.11E-09	-3.77E-09	-4.20E-09	-3.49E-09	-4.38E-09	-4.64E-09
-2.30E-09	-2.01E-09	-1.49E-09	-1.90E-09	-2.24E-09	-1.92E-09	-2.51E-09	-2.85E-09	-3.24E-09	-3.65E-09	-3.96E-09	-3.67E-09	-4.78E-09	-4.71E-09
-2.75E-09	-2.55E-09	-2.36E-09	-2.28E-09	-2.41E-09	-1.91E-09	-2.59E-09	-3.26E-09	-3.24E-09	-4.03E-09	-3.94E-09	-3.32E-09	-4.59E-09	-4.81E-09
-3.44E-09	-2.72E-09	-2.43E-09	-2.73E-09	-2.49E-09	-1.94E-09	-2.87E-09	-3.25E-09	-3.36E-09	-3.95E-09	-3.71E-09	-3.64E-09	-4.06E-09	-4.42E-09
-3.66E-09	-2.89E-09	-2.75E-09	-2.78E-09	-2.70E-09	-2.24E-09	-2.70E-09	-2.81E-09	-2.65E-09	-3.22E-09	-3.32E-09	-3.39E-09	-3.74E-09	-4.05E-09
-3.77E-09	-3.12E-09	-3.01E-09	-2.76E-09	-2.77E-09	-2.54E-09	-1.99E-09	-2.56E-09	-1.90E-09	-2.52E-09	-3.11E-09	-2.95E-09	-3.37E-09	-3.47E-09
-3.66E-09	-2.96E-09	-3.16E-09	-2.86E-09	-2.58E-09	-2.54E-09	-1.68E-09	-2.35E-09	-1.66E-09	-1.97E-09	-2.82E-09	-2.46E-09	-2.69E-09	-2.97E-09
-3.21E-09	-2.57E-09	-3.01E-09	-2.56E-09	-2.48E-09	-2.70E-09	-1.78E-09	-2.30E-09	-1.83E-09	-1.84E-09	-2.13E-09	-1.51E-09	-2.09E-09	-2.61E-09
-2.61E-09	-2.44E-09	-2.75E-09	-2.01E-09	-2.37E-09	-2.34E-09	-1.81E-09	-2.20E-09	-1.80E-09	-1.70E-09	-1.73E-09	-6.78E-10	-1.56E-09	-2.44E-09
-2.76E-09	-2.43E-09	-2.31E-09	-2.12E-09	-2.24E-09	-1.81E-09	-1.69E-09	-2.04E-09	-1.69E-09	-1.29E-09	-1.36E-09	-9.48E-10	-1.21E-09	-2.15E-09
-2.34E-09	-2.14E-09	-1.90E-09	-1.69E-09	-1.91E-09	-1.39E-09	-1.00E-09	-1.79E-09	-1.37E-09	-5.39E-10	-1.40E-09	-1.05E-09	-1.07E-09	-1.80E-09
-2.10E-09	-1.82E-09	-1.29E-09	-1.13E-09	-1.84E-09	-1.15E-09	-2.32E-10	-1.30E-09	-1.11E-09	-1.58E-10	-1.39E-09	-9.46E-10	-8.96E-10	-1.18E-09
-1.86E-09	-1.58E-09	-1.07E-09	-1.10E-09	-1.30E-09	-8.07E-10	4.87E-10	-7.81E-10	-7.22E-10	-1.90E-12	-7.72E-10	-4.74E-10	-9.73E-10	-8.66E-10
-1.03E-09	-1.40E-09	-1.05E-09	-1.21E-09	-1.02E-09	-7.07E-10	4.80E-10	-4.26E-10	-4.02E-10	1.64E-10	-1.79E-10	-3.47E-10	-4.25E-10	-6.68E-10
-1.78E-10	-7.80E-10	-6.64E-10	-1.16E-09	-7.89E-10	-7.87E-10	7.42E-10	2.77E-10	-4.50E-11	-5.64E-11	4.36E-10	7.00E-11	-1.98E-11	-4.31E-10
-1.03E-11	-1.72E-10	-2.75E-10	-1.03E-09	-6.33E-10	-7.05E-10	8.42E-10	5.69E-10	6.89E-10	1.75E-10	1.05E-09	4.50E-10	4.03E-10	3.60E-10

1.48E-10	1.59E-10	-3.56E-10	-5.93E-10	-3.56E-10	-2.21E-10	9.87E-10	6.11E-10	8.16E-10	9.02E-10	1.36E-09	6.91E-10	1.08E-09	7.95E-10
2.24E-10	3.50E-10	-2.43E-10	-1.42E-10	-3.52E-10	-6.97E-11	1.09E-09	1.39E-09	1.31E-09	1.53E-09	1.27E-09	9.01E-10	1.14E-09	1.10E-09
5.73E-10	8.77E-10	1.31E-10	7.57E-11	-1.11E-10	1.27E-10	1.50E-09	1.89E-09	1.77E-09	2.14E-09	1.41E-09	1.19E-09	1.20E-09	1.50E-09
1.02E-09	1.15E-09	5.93E-10	4.48E-10	3.82E-10	4.43E-10	2.47E-09	2.24E-09	1.92E-09	2.74E-09	1.93E-09	1.94E-09	1.74E-09	1.77E-09
1.73E-09	1.82E-09	1.10E-09	1.38E-09	1.30E-09	9.01E-10	3.12E-09	2.69E-09	2.44E-09	2.94E-09	2.40E-09	2.69E-09	2.35E-09	2.21E-09
2.28E-09	2.21E-09	1.96E-09	2.13E-09	2.00E-09	1.78E-09	3.09E-09	3.09E-09	2.95E-09	3.45E-09	3.39E-09	3.42E-09	3.10E-09	2.93E-09
2.55E-09	2.80E-09	2.80E-09	2.44E-09	2.52E-09	2.63E-09	3.15E-09	3.37E-09	3.32E-09	3.61E-09	4.24E-09	3.93E-09	3.74E-09	3.73E-09
3.26E-09	3.17E-09	3.35E-09	3.30E-09	3.00E-09	3.49E-09	3.66E-09	3.80E-09	4.00E-09	3.98E-09	4.85E-09	4.59E-09	4.44E-09	3.91E-09
4.03E-09	3.51E-09	4.04E-09	4.37E-09	3.47E-09	4.10E-09	4.33E-09	4.39E-09	4.54E-09	4.73E-09	5.00E-09	5.43E-09	4.86E-09	4.20E-09
4.83E-09	4.41E-09	4.95E-09	5.26E-09	4.54E-09	4.70E-09	4.95E-09	5.27E-09	5.07E-09	5.76E-09	5.63E-09	6.29E-09	5.16E-09	4.55E-09
5.24E-09	5.51E-09	6.41E-09	5.69E-09	5.47E-09	5.35E-09	5.62E-09	6.10E-09	5.66E-09	6.51E-09	6.28E-09	7.04E-09	5.86E-09	5.17E-09
5.56E-09	6.06E-09	6.95E-09	6.08E-09	5.95E-09	6.26E-09	6.31E-09	6.64E-09	6.38E-09	7.12E-09	7.05E-09	7.11E-09	6.61E-09	5.79E-09
5.96E-09	6.38E-09	5.85E-09	6.15E-09	6.69E-09	6.90E-09	6.88E-09	7.01E-09	7.03E-09	7.52E-09	7.62E-09	7.47E-09	7.42E-09	6.38E-09
6.41E-09	6.98E-09	6.62E-09	6.73E-09	7.28E-09	7.44E-09	7.49E-09	7.59E-09	7.68E-09	8.02E-09	8.06E-09	8.15E-09	8.34E-09	6.97E-09
7.21E-09	7.94E-09	7.55E-09	7.43E-09	7.52E-09	7.91E-09	8.07E-09	8.21E-09	8.34E-09	8.11E-09	8.24E-09	8.60E-09	8.81E-09	7.74E-09
7.94E-09	8.92E-09	8.35E-09	7.84E-09	7.85E-09	8.54E-09	7.90E-09	8.77E-09	8.82E-09	9.06E-09	8.70E-09	9.53E-09	9.35E-09	8.57E-09
8.47E-09	9.22E-09	8.60E-09	8.40E-09	8.46E-09	9.15E-09	8.23E-09	9.38E-09	9.55E-09	9.84E-09	9.31E-09	1.03E-08	9.44E-09	8.95E-09
8.64E-09	9.40E-09	8.89E-09	8.96E-09	9.12E-09	9.50E-09	8.73E-09	1.00E-08	1.02E-08	1.05E-08	9.73E-09	1.07E-08	9.80E-09	9.41E-09
9.07E-09	9.77E-09	9.27E-09	9.51E-09	9.81E-09	9.87E-09	9.55E-09	1.08E-08	1.08E-08	1.06E-08	1.01E-08	1.11E-08	1.06E-08	1.02E-08
9.61E-09	1.04E-08	9.68E-09	1.01E-08	1.10E-08	1.06E-08	1.05E-08	1.09E-08	1.10E-08	1.07E-08	1.06E-08	1.13E-08	1.14E-08	1.10E-08
1.04E-08	1.06E-08	1.02E-08	1.06E-08	1.20E-08	1.12E-08	1.12E-08	1.16E-08	1.17E-08	1.10E-08	1.12E-08	1.16E-08	1.19E-08	1.20E-08
1.18E-08	1.13E-08	1.09E-08	1.16E-08	1.29E-08	1.19E-08	1.20E-08	1.23E-08	1.25E-08	1.21E-08	1.22E-08	1.23E-08	1.22E-08	1.29E-08
1.31E-08	1.25E-08	1.21E-08	1.23E-08	1.34E-08	1.29E-08	1.29E-08	1.31E-08	1.32E-08	1.31E-08	1.33E-08	1.28E-08	1.31E-08	1.34E-08
1.45E-08	1.33E-08	1.26E-08	1.30E-08	1.38E-08	1.39E-08	1.35E-08	1.37E-08	1.38E-08	1.38E-08	1.36E-08	1.33E-08	1.41E-08	1.46E-08
1.49E-08	1.37E-08	1.31E-08	1.35E-08	1.41E-08	1.44E-08	1.41E-08	1.42E-08	1.44E-08	1.41E-08	1.43E-08	1.38E-08	1.47E-08	1.51E-08
1.51E-08	1.45E-08	1.42E-08	1.43E-08	1.44E-08	1.51E-08	1.45E-08	1.48E-08	1.51E-08	1.50E-08	1.53E-08	1.46E-08	1.53E-08	1.54E-08
1.54E-08	1.52E-08	1.51E-08	1.53E-08	1.52E-08	1.55E-08	1.50E-08	1.53E-08	1.59E-08	1.59E-08	1.62E-08	1.57E-08	1.60E-08	1.58E-08
1.60E-08	1.58E-08	1.62E-08	1.62E-08	1.58E-08	1.62E-08	1.60E-08	1.60E-08	1.67E-08	1.67E-08	1.70E-08	1.66E-08	1.65E-08	1.67E-08
1.67E-08	1.62E-08	1.69E-08	1.69E-08	1.61E-08	1.71E-08	1.71E-08	1.69E-08	1.76E-08	1.78E-08	1.79E-08	1.74E-08	1.74E-08	1.79E-08
1.81E-08	1.70E-08	1.76E-08	1.78E-08	1.70E-08	1.76E-08	1.81E-08	1.79E-08	1.84E-08	1.89E-08	1.88E-08	1.82E-08	1.85E-08	1.87E-08
1.92E-08	1.77E-08	1.78E-08	1.79E-08	1.76E-08	1.88E-08	1.90E-08	1.87E-08	1.93E-08	2.01E-08	1.97E-08	1.89E-08	1.92E-08	1.93E-08
1.97E-08	1.85E-08	1.89E-08	1.83E-08	1.83E-08	1.97E-08	1.99E-08	1.94E-08	2.00E-08	2.11E-08	2.08E-08	1.98E-08	1.99E-08	2.02E-08
2.00E-08	1.94E-08	2.00E-08	1.90E-08	1.93E-08	2.00E-08	2.07E-08	2.05E-08	2.11E-08	2.19E-08	2.18E-08	2.10E-08	2.13E-08	2.13E-08
2.11E-08	2.03E-08	2.09E-08	2.03E-08	2.04E-08	2.11E-08	2.15E-08	2.16E-08	2.19E-08	2.26E-08	2.25E-08	2.19E-08	2.24E-08	2.24E-08
2.17E-08	2.14E-08	2.18E-08	2.18E-08	2.18E-08	2.25E-08	2.27E-08	2.25E-08	2.25E-08	2.30E-08	2.34E-08	2.29E-08	2.32E-08	2.28E-08
2.27E-08	2.24E-08	2.22E-08	2.28E-08	2.28E-08	2.40E-08	2.40E-08	2.35E-08	2.34E-08	2.39E-08	2.41E-08	2.40E-08	2.43E-08	2.36E-08
2.37E-08	2.35E-08	2.28E-08	2.35E-08	2.34E-08	2.44E-08	2.46E-08	2.44E-08	2.42E-08	2.45E-08	2.49E-08	2.51E-08	2.56E-08	2.48E-08
2.47E-08	2.45E-08	2.39E-08	2.41E-08	2.44E-08	2.48E-08	2.51E-08	2.49E-08	2.54E-08	2.53E-08	2.57E-08	2.57E-08	2.60E-08	2.58E-08
2.51E-08	2.55E-08	2.49E-08	2.49E-08	2.52E-08	2.55E-08	2.60E-08	2.60E-08	2.66E-08	2.60E-08	2.68E-08	2.67E-08	2.68E-08	2.70E-08
2.59E-08	2.62E-08	2.61E-08	2.57E-08	2.56E-08	2.57E-08	2.66E-08	2.67E-08	2.72E-08	2.66E-08	2.77E-08	2.74E-08	2.82E-08	2.88E-08
2.67E-08	2.71E-08	2.72E-08	2.66E-08	2.63E-08	2.65E-08	2.74E-08	2.76E-08	2.82E-08	2.76E-08	2.81E-08	2.79E-08	2.93E-08	3.07E-08
2.77E-08	2.77E-08	2.82E-08	2.74E-08	2.71E-08	2.76E-08	2.81E-08	2.87E-08	2.92E-08	2.85E-08	2.90E-08	2.88E-08	3.04E-08	3.27E-08
2.88E-08	2.85E-08	2.89E-08	2.84E-08	2.81E-08	2.84E-08	2.87E-08	2.99E-08	3.02E-08	2.94E-08	2.98E-08	2.98E-08	3.17E-08	3.37E-08
3.01E-08	2.93E-08	2.92E-08	2.97E-08	2.93E-08	2.96E-08	2.93E-08	2.99E-08	3.06E-08	3.01E-08	3.04E-08	3.06E-08	3.22E-08	3.32E-08
3.11E-08	3.02E-08	3.01E-08	3.11E-08	3.03E-08	3.10E-08	3.03E-08	3.03E-08	3.10E-08	3.09E-08	3.12E-08	3.16E-08	3.21E-08	3.30E-08
3.20E-08	3.13E-08	3.11E-08	3.28E-08	3.13E-08	3.21E-08	3.10E-08	3.09E-08	3.16E-08	3.17E-08	3.19E-08	3.20E-08	3.20E-08	3.31E-08
3.28E-08	3.24E-08	3.18E-08	3.28E-08	3.17E-08	3.26E-08	3.22E-08	3.19E-08	3.24E-08	3.27E-08	3.25E-08	3.26E-08	3.25E-08	3.32E-08
3.38E-08	3.36E-08	3.26E-08	3.30E-08	3.28E-08	3.33E-08	3.33E-08	3.32E-08	3.38E-08	3.36E-08	3.33E-08	3.36E-08	3.32E-08	3.37E-08
3.48E-08	3.48E-08	3.35E-08	3.39E-08	3.39E-08	3.41E-08	3.40E-08	3.45E-08	3.46E-08	3.47E-08	3.44E-08	3.50E-08	3.39E-08	3.42E-08
3.59E-08	3.55E-08	3.47E-08	3.49E-08	3.49E-08	3.51E-08	3.47E-08	3.55E-08	3.55E-08	3.55E-08	3.55E-08	3.59E-08	3.49E-08	3.51E-08

3.98E-16	4.08E-16	3.81E-16	3.79E-16	3.70E-16	3.57E-16	3.48E-16	3.48E-16	3.54E-16	3.54E-16	3.43E-16	3.17E-16	3.27E-16	3.22E-16
4.14E-16	3.98E-16	3.92E-16	3.94E-16	3.63E-16	3.29E-16	3.57E-16	3.42E-16	3.33E-16	3.43E-16	3.40E-16	3.18E-16	3.17E-16	3.09E-16
4.12E-16	3.82E-16	3.97E-16	3.86E-16	3.58E-16	3.36E-16	3.60E-16	3.19E-16	3.00E-16	3.22E-16	3.23E-16	2.97E-16	2.93E-16	2.89E-16
4.00E-16	3.81E-16	3.84E-16	3.83E-16	3.59E-16	3.49E-16	3.54E-16	2.92E-16	2.86E-16	2.99E-16	3.03E-16	2.98E-16	2.79E-16	2.77E-16
4.00E-16	3.99E-16	3.81E-16	3.75E-16	3.71E-16	3.63E-16	3.30E-16	2.87E-16	2.91E-16	2.96E-16	2.86E-16	2.87E-16	2.63E-16	2.54E-16
3.91E-16	4.09E-16	3.90E-16	3.70E-16	3.66E-16	3.61E-16	3.22E-16	2.81E-16	2.92E-16	3.04E-16	2.80E-16	2.68E-16	2.47E-16	2.50E-16
3.79E-16	3.90E-16	3.82E-16	3.62E-16	3.46E-16	3.45E-16	3.17E-16	2.92E-16	3.00E-16	3.07E-16	2.82E-16	2.72E-16	2.46E-16	2.44E-16
3.63E-16	3.61E-16	3.56E-16	3.58E-16	3.44E-16	3.33E-16	3.10E-16	3.01E-16	3.06E-16	3.06E-16	2.84E-16	2.68E-16	2.45E-16	2.40E-16
3.54E-16	3.45E-16	3.34E-16	3.40E-16	3.33E-16	3.15E-16	2.96E-16	3.02E-16	3.03E-16	2.98E-16	2.77E-16	2.57E-16	2.32E-16	2.30E-16
3.38E-16	3.46E-16	3.35E-16	3.35E-16	3.29E-16	3.05E-16	2.81E-16	2.86E-16	2.97E-16	2.85E-16	2.54E-16	2.46E-16	2.32E-16	2.35E-16
3.12E-16	3.29E-16	3.33E-16	3.43E-16	3.37E-16	3.10E-16	2.71E-16	2.68E-16	2.64E-16	2.62E-16	2.34E-16	2.30E-16	2.35E-16	2.31E-16
2.99E-16	3.22E-16	3.25E-16	3.36E-16	3.22E-16	3.12E-16	2.82E-16	2.67E-16	2.41E-16	2.39E-16	2.11E-16	2.08E-16	2.22E-16	2.31E-16
2.86E-16	3.22E-16	3.27E-16	3.21E-16	2.96E-16	2.92E-16	2.81E-16	2.72E-16	2.46E-16	2.33E-16	1.98E-16	1.91E-16	1.95E-16	2.12E-16
2.67E-16	3.04E-16	3.19E-16	3.09E-16	2.70E-16	2.66E-16	2.66E-16	2.58E-16	2.33E-16	2.29E-16	2.05E-16	1.96E-16	1.84E-16	1.84E-16
2.59E-16	2.73E-16	3.01E-16	2.93E-16	2.45E-16	2.44E-16	2.49E-16	2.41E-16	2.21E-16	2.17E-16	2.00E-16	2.06E-16	1.94E-16	1.75E-16
2.53E-16	2.34E-16	2.70E-16	2.61E-16	2.11E-16	2.30E-16	2.33E-16	2.33E-16	2.20E-16	2.08E-16	1.94E-16	1.96E-16	1.91E-16	1.74E-16
2.42E-16	2.05E-16	2.29E-16	2.34E-16	1.83E-16	2.11E-16	2.18E-16	2.16E-16	2.21E-16	2.14E-16	1.90E-16	1.85E-16	1.87E-16	1.90E-16
2.47E-16	2.12E-16	2.08E-16	2.22E-16	1.59E-16	2.06E-16	2.14E-16	2.02E-16	1.99E-16	2.05E-16	1.81E-16	1.80E-16	1.82E-16	1.76E-16
2.47E-16	2.39E-16	2.33E-16	2.32E-16	1.87E-16	2.12E-16	2.13E-16	1.98E-16	1.80E-16	1.91E-16	1.71E-16	1.69E-16	1.66E-16	1.64E-16
2.40E-16	2.47E-16	2.49E-16	2.52E-16	2.20E-16	2.23E-16	2.12E-16	1.82E-16	1.57E-16	1.80E-16	1.66E-16	1.62E-16	1.56E-16	1.57E-16
2.29E-16	2.39E-16	2.29E-16	2.47E-16	2.21E-16	2.11E-16	1.98E-16	1.74E-16	1.45E-16	1.60E-16	1.67E-16	1.57E-16	1.52E-16	1.53E-16
2.34E-16	2.30E-16	2.20E-16	2.33E-16	2.05E-16	1.88E-16	1.87E-16	1.78E-16	1.51E-16	1.45E-16	1.64E-16	1.47E-16	1.48E-16	1.50E-16
2.25E-16	2.20E-16	2.12E-16	2.17E-16	1.86E-16	1.74E-16	1.77E-16	1.76E-16	1.54E-16	1.47E-16	1.48E-16	1.38E-16	1.45E-16	1.42E-16
2.02E-16	2.03E-16	1.98E-16	1.92E-16	1.74E-16	1.73E-16	1.77E-16	1.65E-16	1.50E-16	1.42E-16	1.33E-16	1.28E-16	1.26E-16	1.31E-16
1.89E-16	1.85E-16	1.75E-16	1.70E-16	1.67E-16	1.72E-16	1.68E-16	1.56E-16	1.43E-16	1.33E-16	1.24E-16	1.19E-16	1.14E-16	1.15E-16
1.84E-16	1.68E-16	1.59E-16	1.54E-16	1.56E-16	1.55E-16	1.51E-16	1.47E-16	1.37E-16	1.19E-16	1.21E-16	1.19E-16	1.04E-16	1.03E-16
1.81E-16	1.62E-16	1.54E-16	1.49E-16	1.34E-16	1.28E-16	1.32E-16	1.37E-16	1.31E-16	1.17E-16	1.20E-16	1.09E-16	9.86E-17	9.54E-17
1.76E-16	1.55E-16	1.48E-16	1.46E-16	1.17E-16	1.05E-16	1.20E-16	1.26E-16	1.25E-16	1.17E-16	1.03E-16	9.66E-17	8.33E-17	8.30E-17
1.64E-16	1.48E-16	1.36E-16	1.41E-16	1.09E-16	8.67E-17	1.13E-16	1.16E-16	1.15E-16	1.04E-16	8.60E-17	7.92E-17	6.41E-17	7.73E-17
1.54E-16	1.48E-16	1.32E-16	1.34E-16	1.17E-16	9.66E-17	1.06E-16	1.09E-16	1.02E-16	8.13E-17	7.26E-17	7.09E-17	5.49E-17	7.46E-17
1.32E-16	1.31E-16	1.28E-16	1.26E-16	1.29E-16	1.17E-16	1.03E-16	9.93E-17	9.75E-17	6.92E-17	7.05E-17	7.16E-17	6.96E-17	6.59E-17

1.08E-16	1.16E-16	1.17E-16	1.16E-16	1.20E-16	1.20E-16	1.02E-16	9.13E-17	9.97E-17	7.71E-17	7.17E-17	7.28E-17	7.00E-17	5.46E-17
9.44E-17	1.07E-16	1.03E-16	1.06E-16	1.14E-16	1.13E-16	1.03E-16	8.35E-17	9.73E-17	8.24E-17	6.48E-17	7.03E-17	7.25E-17	4.55E-17
8.45E-17	9.72E-17	9.29E-17	9.95E-17	1.04E-16	1.04E-16	8.96E-17	8.03E-17	9.11E-17	7.87E-17	6.87E-17	7.30E-17	6.52E-17	3.75E-17
7.65E-17	1.04E-16	8.64E-17	9.26E-17	9.56E-17	9.49E-17	7.06E-17	6.84E-17	7.96E-17	7.70E-17	7.08E-17	7.01E-17	5.45E-17	3.25E-17
8.71E-17	9.72E-17	8.33E-17	8.90E-17	9.47E-17	8.71E-17	6.06E-17	5.95E-17	7.72E-17	7.19E-17	7.15E-17	6.82E-17	5.27E-17	3.24E-17
1.01E-16	9.63E-17	8.40E-17	8.49E-17	8.03E-17	7.39E-17	5.25E-17	5.41E-17	7.37E-17	7.01E-17	7.12E-17	6.84E-17	4.88E-17	3.37E-17
1.08E-16	9.35E-17	8.10E-17	7.81E-17	6.48E-17	5.94E-17	4.58E-17	4.69E-17	6.40E-17	6.53E-17	6.96E-17	7.49E-17	4.68E-17	3.61E-17
1.12E-16	9.03E-17	7.78E-17	6.48E-17	4.83E-17	4.37E-17	3.77E-17	4.69E-17	5.15E-17	5.51E-17	6.76E-17	7.07E-17	5.41E-17	3.67E-17
1.10E-16	8.90E-17	7.90E-17	5.71E-17	3.61E-17	3.48E-17	3.17E-17	4.63E-17	4.41E-17	4.15E-17	6.22E-17	6.48E-17	5.38E-17	4.15E-17
1.14E-16	9.24E-17	9.60E-17	6.52E-17	3.34E-17	2.92E-17	3.49E-17	4.53E-17	3.85E-17	3.07E-17	5.51E-17	6.13E-17	5.51E-17	4.41E-17
1.32E-16	1.00E-16	1.05E-16	7.79E-17	4.71E-17	3.52E-17	4.22E-17	4.59E-17	3.62E-17	2.20E-17	5.37E-17	6.03E-17	5.13E-17	4.55E-17
1.64E-16	1.15E-16	1.12E-16	8.04E-17	6.98E-17	5.63E-17	4.76E-17	4.25E-17	3.78E-17	1.72E-17	4.89E-17	6.44E-17	4.94E-17	5.14E-17
1.81E-16	1.17E-16	1.13E-16	8.35E-17	8.11E-17	6.87E-17	5.06E-17	3.83E-17	3.57E-17	2.51E-17	4.64E-17	7.00E-17	5.03E-17	5.65E-17
1.60E-16	1.19E-16	1.09E-16	9.06E-17	8.11E-17	7.01E-17	5.31E-17	3.24E-17	3.29E-17	3.81E-17	5.08E-17	6.65E-17	5.17E-17	6.08E-17
1.47E-16	1.35E-16	1.07E-16	9.66E-17	8.58E-17	7.45E-17	6.24E-17	2.84E-17	4.04E-17	5.77E-17	6.42E-17	6.89E-17	6.10E-17	5.94E-17
1.11E-16	1.29E-16	1.16E-16	1.01E-16	9.56E-17	7.48E-17	6.53E-17	3.52E-17	6.00E-17	7.57E-17	8.38E-17	7.52E-17	7.91E-17	7.04E-17
9.74E-17	9.64E-17	9.89E-17	9.94E-17	8.12E-17	6.06E-17	4.68E-17	4.19E-17	6.75E-17	9.15E-17	9.82E-17	7.54E-17	9.63E-17	8.49E-17
8.24E-17	5.51E-17	5.42E-17	6.51E-17	2.42E-17	2.61E-17	3.91E-17	5.06E-17	7.25E-17	8.95E-17	9.85E-17	7.38E-17	9.97E-17	9.26E-17
4.08E-17	2.61E-17	1.90E-17	1.59E-17	6.23E-21	9.36E-18	3.36E-17	5.64E-17	7.45E-17	8.52E-17	8.82E-17	6.99E-17	1.04E-16	9.65E-17
2.55E-17	1.39E-17	2.96E-18	1.48E-18	2.16E-17	4.80E-18	3.17E-17	5.86E-17	6.96E-17	8.04E-17	7.12E-17	7.60E-17	1.03E-16	9.18E-17
9.60E-18	2.76E-18	8.57E-18	3.63E-17	2.69E-17	2.18E-18	3.12E-17	5.21E-17	6.03E-17	7.49E-17	6.65E-17	7.19E-17	8.93E-17	8.14E-17
1.48E-18	5.11E-18	7.87E-17	1.09E-16	2.29E-17	3.07E-18	2.35E-17	4.02E-17	5.21E-17	6.38E-17	5.92E-17	6.47E-17	7.32E-17	7.13E-17
4.52E-17	5.75E-17	1.65E-16	1.08E-16	1.95E-17	4.89E-18	1.80E-17	2.96E-17	4.58E-17	5.64E-17	5.37E-17	6.92E-17	6.93E-17	7.10E-17
7.86E-17	9.36E-17	1.12E-16	6.08E-17	1.44E-17	5.23E-18	1.93E-17	2.48E-17	3.69E-17	5.42E-17	5.25E-17	6.35E-17	6.69E-17	7.52E-17
4.99E-17	4.41E-17	3.45E-17	1.76E-17	3.25E-18	3.34E-18	1.15E-17	2.25E-17	3.08E-17	5.51E-17	5.16E-17	5.27E-17	5.73E-17	7.75E-17
3.07E-17	2.15E-17	1.04E-17	2.32E-18	1.06E-20	3.51E-18	1.13E-17	2.48E-17	3.09E-17	4.53E-17	4.39E-17	4.11E-17	4.88E-17	6.97E-17
2.29E-17	1.12E-17	3.30E-18	7.62E-20	1.38E-18	3.93E-18	1.24E-17	2.37E-17	2.52E-17	3.16E-17	3.20E-17	2.74E-17	4.39E-17	5.86E-17
9.05E-18	2.41E-18	9.09E-20	1.72E-19	2.52E-18	6.58E-18	1.38E-17	1.85E-17	1.93E-17	2.19E-17	2.56E-17	2.31E-17	3.57E-17	4.79E-17
1.84E-19	1.52E-19	5.08E-20	8.86E-19	2.75E-18	6.67E-18	9.66E-18	1.34E-17	1.56E-17	1.69E-17	2.14E-17	2.13E-17	3.33E-17	3.83E-17
9.92E-19	3.03E-19	2.98E-19	2.05E-18	2.93E-18	3.50E-18	8.05E-18	1.27E-17	1.10E-17	1.46E-17	2.08E-17	1.86E-17	2.91E-17	3.25E-17
2.53E-18	1.09E-18	6.82E-19	2.77E-18	2.95E-18	1.61E-18	8.33E-18	1.09E-17	1.00E-17	1.49E-17	2.06E-17	1.34E-17	2.15E-17	2.52E-17
3.34E-18	2.60E-18	1.34E-18	3.11E-18	3.37E-18	2.08E-18	8.37E-18	1.00E-17	9.67E-18	1.42E-17	1.76E-17	1.22E-17	1.92E-17	2.15E-17
5.27E-18	4.04E-18	2.23E-18	3.61E-18	5.04E-18	3.67E-18	6.28E-18	8.11E-18	1.05E-17	1.33E-17	1.57E-17	1.34E-17	2.29E-17	2.22E-17
7.57E-18	6.51E-18	5.59E-18	5.19E-18	5.80E-18	3.65E-18	6.69E-18	1.06E-17	1.05E-17	1.62E-17	1.55E-17	1.10E-17	2.10E-17	2.31E-17
1.18E-17	7.41E-18	5.91E-18	7.45E-18	6.21E-18	3.76E-18	8.25E-18	1.05E-17	1.13E-17	1.56E-17	1.37E-17	1.33E-17	1.65E-17	1.96E-17
1.34E-17	8.34E-18	7.55E-18	7.70E-18	7.28E-18	5.01E-18	7.27E-18	7.91E-18	7.02E-18	1.04E-17	1.10E-17	1.15E-17	1.40E-17	1.64E-17
1.42E-17	9.74E-18	9.06E-18	7.60E-18	7.69E-18	6.46E-18	3.95E-18	6.58E-18	3.63E-18	6.36E-18	9.67E-18	8.73E-18	1.14E-17	1.21E-17
1.34E-17	8.79E-18	1.00E-17	8.17E-18	6.67E-18	6.44E-18	2.84E-18	5.51E-18	2.76E-18	3.90E-18	7.97E-18	6.04E-18	7.24E-18	8.83E-18
1.03E-17	6.62E-18	9.04E-18	6.55E-18	6.16E-18	7.30E-18	3.16E-18	5.28E-18	3.34E-18	3.40E-18	4.53E-18	2.29E-18	4.35E-18	6.84E-18
6.82E-18	5.97E-18	7.56E-18	4.03E-18	5.63E-18	5.49E-18	3.29E-18	4.85E-18	3.24E-18	2.89E-18	2.98E-18	4.59E-19	2.42E-18	5.97E-18
7.63E-18	5.89E-18	5.33E-18	4.48E-18	5.02E-18	3.29E-18	2.86E-18	4.18E-18	2.86E-18	1.67E-18	1.84E-18	8.99E-19	1.47E-18	4.64E-18
5.48E-18	4.59E-18	3.60E-18	2.87E-18	3.64E-18	1.93E-18	1.01E-18	3.22E-18	1.87E-18	2.91E-19	1.96E-18	1.11E-18	1.14E-18	3.22E-18
4.42E-18	3.31E-18	1.65E-18	1.28E-18	3.38E-18	1.32E-18	5.39E-20	1.70E-18	1.24E-18	2.48E-20	1.92E-18	8.94E-19	8.02E-19	1.39E-18
3.46E-18	2.51E-18	1.15E-18	1.21E-18	1.70E-18	6.52E-19	2.37E-19	6.10E-19	5.21E-19	3.63E-24	5.96E-19	2.25E-19	9.48E-19	7.49E-19
1.06E-18	1.95E-18	1.11E-18	1.46E-18	1.04E-18	5.00E-19	2.31E-19	1.82E-19	1.62E-19	2.70E-20	3.20E-20	1.20E-19	1.80E-19	4.47E-19
3.16E-20	6.08E-19	4.40E-19	1.34E-18	6.23E-19	6.19E-19	5.51E-19	7.65E-20	2.03E-21	3.18E-21	1.90E-19	4.90E-21	3.91E-22	1.86E-19
1.05E-22	2.97E-20	7.59E-20	1.07E-18	4.01E-19	4.97E-19	7.09E-19	3.23E-19	4.74E-19	3.06E-20	1.10E-18	2.02E-19	1.62E-19	1.30E-19
2.19E-20	2.52E-20	1.26E-19	3.51E-19	1.26E-19	4.88E-20	9.73E-19	3.73E-19	6.65E-19	8.14E-19	1.86E-18	4.78E-19	1.17E-18	6.32E-19
5.00E-20	1.23E-19	5.89E-20	2.02E-20	1.24E-19	4.86E-21	1.19E-18	1.92E-18	1.71E-18	2.33E-18	1.62E-18	8.11E-19	1.30E-18	1.21E-18
3.29E-19	7.69E-19	1.71E-20	5.74E-21	1.22E-20	1.61E-20	2.26E-18	3.56E-18	3.14E-18	4.58E-18	1.98E-18	1.42E-18	1.45E-18	2.24E-18
1.05E-18	1.32E-18	3.52E-19	2.01E-19	1.46E-19	1.96E-19	6.11E-18	5.02E-18	3.69E-18	7.50E-18	3.74E-18	3.76E-18	3.01E-18	3.14E-18
2.98E-18	3.30E-18	1.21E-18	1.91E-18	1.68E-18	8.13E-19	9.76E-18	7.24E-18	5.97E-18	8.62E-18	5.75E-18	7.22E-18	5.50E-18	4.89E-18
5.21E-18	4.87E-18	3.85E-18	4.52E-18	3.98E-18	3.17E-18	9.55E-18	9.53E-18	8.70E-18	1.19E-17	1.15E-17	1.17E-17	9.62E-18	8.58E-18
6.49E-18	7.83E-18	7.85E-18	5.98E-18	6.33E-18	6.92E-18	9.93E-18	1.13E-17	1.10E-17	1.30E-17	1.80E-17	1.55E-17	1.40E-17	1.39E-17
1.06E-17	1.00E-17	1.12E-17	1.09E-17	8.98E-18	1.22E-17	1.34E-17	1.44E-17	1.60E-17	1.58E-17	2.35E-17	2.11E-17	1.98E-17	1.53E-17
1.62E-17	1.24E-17	1.63E-17	1.91E-17	1.20E-17	1.68E-17	1.87E-17	1.93E-17	2.06E-17	2.24E-17	2.50E-17	2.95E-17	2.36E-17	1.76E-17
2.33E-17	1.95E-17	2.46E-17	2.77E-17	2.06E-17	2.21E-17	2.45E-17	2.78E-17	2.57E-17	3.32E-17	3.17E-17	3.96E-17	2.66E-17	2.07E-17
2.75E-17	3.03E-17	4.11E-17	3.24E-17	2.99E-17	2.86E-17	3.16E-17	3.73E-17	3.21E-17	4.24E-17	3.95E-17	4.96E-17	3.43E-17	2.67E-17
3.09E-17	3.67E-17	4.83E-17	3.70E-17	3.54E-17	3.92E-17	3.98E-17	4.40E-17	4.07E-17	5.07E-17	4.97E-17	5.05E-17	4.37E-17	3.35E-17
3.55E-17	4.07E-17	3.42E-17	3.79E-17	4.48E-17	4.77E-17	4.74E-17	4.91E-17	4.95E-17	5.66E-17	5.81E-17	5.58E-17	5.51E-17	4.07E-17
4.11E-17	4.87E-17	4.38E-17	4.53E-17	5.30E-17	5.54E-17	5.61E-17	5.76E-17	5.90E-17	6.44E-17	6.50E-17	6.65E-17	6.95E-17	4.85E-17
5.20E-17	6.31E-17	5.70E-17	5.52E-17	5.66E-17	6.26E-17	6.52E-17	6.75E-17	6.96E-17	6.58E-17	6.79E-17	7.39E-17	7.76E-17	5.99E-17
6.30E-17	7.96E-17	6.97E-17	6.15E-17	6.16E-17	7.29E-17	6.24E-17	7.68E-17	7.79E-17	8.22E-17	7.57E-17	9.08E-17	8.75E-17	7.34E-17
7.18E-17	8.50E-17	7.40E-17	7.06E-17	7.16E-17	8.38E-17	6.78E-17	8.80E-17	9.12E-17	9.68E-17	8.67E-17	1.06E-16	8.91E-17	8.00E-17
7.47E-17	8.83E-17	7.91E-17	8.03E-17	8.31E-17	9.02E-17	7.63E-17	1.00E-16	1.04E-16	1.10E-16	9.47E-17	1.15E-16	9.60E-17	8.86E-17
8.22E-17	9.55E-17	8.58E-17	9.05E-17	9.63E-17	9.74E-17	9.12E-17	1.17E-16	1.16E-16	1.12E-16	1.01E-16	1.23E-16	1.12E-16	1.05E-16
9.24E-17	1.08E-16	9.38E-17	1.03E-16	1.21E-16	1.13E-16	1.10E-16	1.19E-16	1.22E-16	1.15E-16	1.12E-16	1.28E-16	1.30E-16	1.20E-16
1.08E-16	1.12E-16	1.03E-16	1.13E-16	1.43E-16	1.25E-16	1.25E-16	1.34E-16	1.38E-16	1.21E-16	1.26E-16	1.34E-16	1.40E-16	1.45E-16
1.40E-16	1.29E-16	1.19E-16	1.34E-16	1.66E-16	1.43E-16	1.45E-16	1.50E-16	1.57E-16	1.47E-16	1.49E-16	1.51E-16	1.48E-16	1.66E-16
1.72E-16	1.57E-16	1.46E-16	1.50E-16	1.79E-16	1.68E-16	1.66E-16	1.72E-16	1.74E-16	1.72E-16	1.78E-16	1.64E-16	1.71E-16	1.80E-16
2.09E-16	1.77E-16	1.59E-16	1.69E-16	1.90E-16	1.92E-16	1.81E-16	1.88E-16	1.90E-16	1.92E-16	1.86E-16	1.76E-16	1.99E-16	2.13E-16
2.22E-16	1.88E-16	1.72E-16	1.81E-16	1.98E-16	2.08E-16	1.99E-16	2.02E-16	2.08E-16	1.98E-16	2.05E-16	1.91E-16	2.16E-16	2.28E-16
2.29E-16	2.11E-16	2.01E-16	2.03E-16	2.06E-16	2.29E-16	2.10E-16	2.18E-16	2.27E-16	2.26E-16	2.34E-16	2.14E-16	2.34E-16	2.38E-16
2.37E-16	2.30E-16	2.29E-16	2.35E-16	2.32E-16	2.40E-16	2.24E-16	2.35E-16	2.52E-16	2.53E-16	2.62E-16	2.46E-16	2.58E-16	2.50E-16
2.57E-16	2.50E-16	2.61E-16	2.64E-16	2.48E-16	2.63E-16	2.56E-16	2.57E-16	2.78E-16	2.80E-16	2.89E-16	2.75E-16	2.73E-16	2.79E-16
2.79E-16	2.63E-16	2.84E-16	2.87E-16	2.60E-16	2.92E-16	2.93E-16	2.85E-16	3.11E-16	3.16E-16	3.20E-16	3.01E-16	3.02E-16	3.19E-16
3.27E-16	2.89E-16	3.08E-16	3.16E-16	2.88E-16	3.10E-16	3.28E-16	3.19E-16	3.37E-16	3.59E-16	3.54E-16	3.32E-16	3.44E-16	3.50E-16
3.69E-16	3.14E-16	3.18E-16	3.20E-16	3.08E-16	3.53E-16	3.59E-16	3.49E-16	3.72E-16	4.03E-16	3.88E-16	3.56E-16	3.70E-16	3.74E-16
3.89E-16	3.43E-16	3.57E-16	3.36E-16	3.35E-16	3.89E-16	3.96E-16	3.77E-16	3.98E-16	4.45E-16	4.33E-16	3.92E-16	3.97E-16	4.10E-16
4.01E-16	3.76E-16	3.99E-16	3.63E-16	3.73E-16	4.02E-16	4.30E-16	4.19E-16	4.46E-16	4.79E-16	4.73E-16	4.40E-16	4.54E-16	4.54E-16
4.44E-16	4.12E-16	4.37E-16	4.14E-16	4.16E-16	4.43E-16	4.60E-16	4.66E-16	4.79E-16	5.10E-16	5.07E-16	4.78E-16	5.00E-16	5.01E-16
4.73E-16	4.58E-16	4.74E-16	4.74E-16	4.73E-16	5.05E-16	5.14E-16	5.07E-16	5.07E-16	5.30E-16	5.47E-16	5.23E-16	5.38E-16	5.18E-16
5.18E-16	5.04E-16	4.95E-16	5.19E-16	5.20E-16	5.76E-16	5.78E-16	5.53E-16	5.46E-16	5.69E-16	5.79E-16	5.74E-16	5.93E-16	5.57E-16
5.61E-16	5.51E-16	5.21E-16	5.54E-16	5.49E-16	5.94E-16	6.08E-16	5.95E-16	5.84E-16	6.02E-16	6.18E-16	6.28E-16	6.53E-16	6.13E-16
6.09E-16	5.99E-16	5.71E-16	5.83E-16	5.95E-16	6.17E-16	6.30E-16	6.21E-16	6.43E-16	6.39E-16	6.62E-16	6.62E-16	6.77E-16	6.66E-16
6.32E-16	6.49E-16	6.19E-16	6.20E-16	6.35E-16	6.49E-16	6.76E-16	6.74E-16	7.07E-16	6.74E-16	7.18E-16	7.15E-16	7.20E-16	7.31E-16

6.71E-16	6.86E-16	6.80E-16	6.63E-16	6.54E-16	6.60E-16	7.09E-16	7.12E-16	7.38E-16	7.07E-16	7.65E-16	7.53E-16	7.94E-16	8.28E-16
7.14E-16	7.33E-16	7.39E-16	7.10E-16	6.90E-16	7.01E-16	7.50E-16	7.61E-16	7.94E-16	7.64E-16	7.91E-16	7.76E-16	8.60E-16	9.40E-16
7.65E-16	7.68E-16	7.98E-16	7.49E-16	7.36E-16	7.60E-16	7.88E-16	8.21E-16	8.54E-16	8.12E-16	8.43E-16	8.27E-16	9.27E-16	1.07E-15
8.32E-16	8.14E-16	8.33E-16	8.07E-16	7.89E-16	8.09E-16	8.25E-16	8.96E-16	9.14E-16	8.62E-16	8.87E-16	8.88E-16	1.00E-15	1.13E-15
9.04E-16	8.60E-16	8.53E-16	8.81E-16	8.57E-16	8.74E-16	8.59E-16	8.95E-16	9.38E-16	9.09E-16	9.24E-16	9.34E-16	1.04E-15	1.11E-15
9.70E-16	9.10E-16	9.03E-16	9.65E-16	9.16E-16	9.61E-16	9.15E-16	9.16E-16	9.59E-16	9.54E-16	9.73E-16	9.98E-16	1.03E-15	1.09E-15
1.03E-15	9.79E-16	9.67E-16	1.07E-15	9.78E-16	1.03E-15	9.64E-16	9.57E-16	9.96E-16	1.01E-15	1.02E-15	1.03E-15	1.02E-15	1.09E-15
1.07E-15	1.05E-15	1.01E-15	1.07E-15	1.00E-15	1.06E-15	1.04E-15	1.02E-15	1.05E-15	1.07E-15	1.06E-15	1.06E-15	1.05E-15	1.10E-15
1.14E-15	1.13E-15	1.07E-15	1.09E-15	1.07E-15	1.11E-15	1.11E-15	1.10E-15	1.14E-15	1.13E-15	1.11E-15	1.13E-15	1.11E-15	1.14E-15
1.21E-15	1.21E-15	1.12E-15	1.15E-15	1.15E-15	1.16E-15	1.16E-15	1.19E-15	1.20E-15	1.20E-15	1.18E-15	1.22E-15	1.15E-15	1.17E-15
1.29E-15	1.26E-15	1.20E-15	1.22E-15	1.22E-15	1.23E-15	1.20E-15	1.26E-15	1.26E-15	1.26E-15	1.26E-15	1.29E-15	1.22E-15	1.23E-15
2.89E-14	2.80E-14	2.77E-14	2.77E-14	2.63E-14	2.67E-14	2.65E-14	2.65E-14	2.70E-14	2.71E-14	2.71E-14	2.68E-14	2.70E-14	2.75E-14

-1.67E-08	-1.55E-08	-1.55E-08	-1.56E-08	-1.60E-08	-1.63E-08	-1.65E-08	-1.59E-08	-1.51E-08	-1.44E-08	-1.45E-08	-1.40E-08	-1.43E-08	-1.36E-08	-1.39E-08
-1.69E-08	-1.63E-08	-1.58E-08	-1.56E-08	-1.57E-08	-1.66E-08	-1.63E-08	-1.57E-08	-1.54E-08	-1.46E-08	-1.38E-08	-1.40E-08	-1.40E-08	-1.30E-08	-1.38E-08
-1.71E-08	-1.68E-08	-1.61E-08	-1.55E-08	-1.54E-08	-1.64E-08	-1.63E-08	-1.55E-08	-1.57E-08	-1.52E-08	-1.42E-08	-1.43E-08	-1.37E-08	-1.33E-08	-1.35E-08
-1.71E-08	-1.67E-08	-1.56E-08	-1.58E-08	-1.56E-08	-1.58E-08	-1.59E-08	-1.51E-08	-1.55E-08	-1.53E-08	-1.46E-08	-1.45E-08	-1.38E-08	-1.34E-08	-1.31E-08
-1.66E-08	-1.63E-08	-1.55E-08	-1.60E-08	-1.57E-08	-1.52E-08	-1.56E-08	-1.46E-08	-1.49E-08	-1.49E-08	-1.44E-08	-1.41E-08	-1.41E-08	-1.32E-08	-1.28E-08
-1.64E-08	-1.63E-08	-1.54E-08	-1.60E-08	-1.54E-08	-1.51E-08	-1.52E-08	-1.43E-08	-1.42E-08	-1.45E-08	-1.41E-08	-1.34E-08	-1.41E-08	-1.35E-08	-1.29E-08
-1.62E-08	-1.60E-08	-1.54E-08	-1.56E-08	-1.54E-08	-1.50E-08	-1.51E-08	-1.39E-08	-1.37E-08	-1.42E-08	-1.40E-08	-1.32E-08	-1.41E-08	-1.38E-08	-1.28E-08
-1.56E-08	-1.56E-08	-1.52E-08	-1.51E-08	-1.53E-08	-1.49E-08	-1.53E-08	-1.37E-08	-1.36E-08	-1.37E-08	-1.37E-08	-1.31E-08	-1.39E-08	-1.37E-08	-1.24E-08
-1.51E-08	-1.54E-08	-1.53E-08	-1.47E-08	-1.50E-08	-1.44E-08	-1.52E-08	-1.37E-08	-1.35E-08	-1.30E-08	-1.31E-08	-1.29E-08	-1.32E-08	-1.27E-08	-1.18E-08
-1.46E-08	-1.51E-08	-1.53E-08	-1.45E-08	-1.50E-08	-1.40E-08	-1.51E-08	-1.36E-08	-1.30E-08	-1.24E-08	-1.24E-08	-1.25E-08	-1.28E-08	-1.18E-08	-1.11E-08
-1.44E-08	-1.45E-08	-1.50E-08	-1.42E-08	-1.48E-08	-1.41E-08	-1.49E-08	-1.35E-08	-1.30E-08	-1.24E-08	-1.24E-08	-1.24E-08	-1.25E-08	-1.12E-08	-1.08E-08
-1.43E-08	-1.42E-08	-1.42E-08	-1.39E-08	-1.42E-08	-1.39E-08	-1.45E-08	-1.33E-08	-1.30E-08	-1.26E-08	-1.23E-08	-1.21E-08	-1.24E-08	-1.14E-08	-1.09E-08
-1.41E-08	-1.41E-08	-1.36E-08	-1.33E-08	-1.38E-08	-1.36E-08	-1.42E-08	-1.29E-08	-1.27E-08	-1.23E-08	-1.20E-08	-1.17E-08	-1.18E-08	-1.18E-08	-1.09E-08
-1.36E-08	-1.39E-08	-1.38E-08	-1.33E-08	-1.38E-08	-1.33E-08	-1.37E-08	-1.26E-08	-1.22E-08	-1.18E-08	-1.17E-08	-1.14E-08	-1.13E-08	-1.16E-08	-1.07E-08
-1.34E-08	-1.36E-08	-1.34E-08	-1.32E-08	-1.40E-08	-1.35E-08	-1.34E-08	-1.25E-08	-1.20E-08	-1.16E-08	-1.15E-08	-1.11E-08	-1.11E-08	-1.11E-08	-1.05E-08
-1.37E-08	-1.37E-08	-1.31E-08	-1.26E-08	-1.34E-08	-1.32E-08	-1.32E-08	-1.24E-08	-1.20E-08	-1.17E-08	-1.13E-08	-1.09E-08	-1.08E-08	-1.06E-08	-1.03E-08
-1.36E-08	-1.37E-08	-1.31E-08	-1.24E-08	-1.30E-08	-1.21E-08	-1.25E-08	-1.19E-08	-1.17E-08	-1.17E-08	-1.13E-08	-1.09E-08	-1.03E-08	-9.93E-09	-9.82E-09
-1.34E-08	-1.31E-08	-1.28E-08	-1.22E-08	-1.26E-08	-1.14E-08	-1.15E-08	-1.10E-08	-1.12E-08	-1.11E-08	-1.10E-08	-1.09E-08	-1.04E-08	-9.49E-09	-9.38E-09
-1.28E-08	-1.24E-08	-1.22E-08	-1.12E-08	-1.14E-08	-1.01E-08	-1.06E-08	-1.09E-08	-1.09E-08	-1.04E-08	-1.03E-08	-1.07E-08	-1.02E-08	-9.30E-09	-9.34E-09
-1.25E-08	-1.19E-08	-1.17E-08	-1.06E-08	-1.04E-08	-9.27E-09	-1.02E-08	-1.08E-08	-1.07E-08	-1.06E-08	-1.01E-08	-1.01E-08	-9.90E-09	-9.68E-09	-9.44E-09
-1.23E-08	-1.14E-08	-1.14E-08	-1.06E-08	-1.01E-08	-9.14E-09	-9.93E-09	-1.08E-08	-1.05E-08	-1.04E-08	-1.01E-08	-1.00E-08	-9.86E-09	-9.46E-09	-9.15E-09
-1.17E-08	-1.13E-08	-1.12E-08	-1.11E-08	-1.03E-08	-9.56E-09	-9.99E-09	-1.10E-08	-1.05E-08	-9.83E-09	-9.61E-09	-9.50E-09	-9.46E-09	-9.28E-09	-9.08E-09
-1.13E-08	-1.13E-08	-1.08E-08	-1.10E-08	-1.10E-08	-1.06E-08	-1.05E-08	-1.11E-08	-9.93E-09	-9.46E-09	-9.10E-09	-8.87E-09	-8.82E-09	-8.81E-09	-8.88E-09
-1.06E-08	-1.13E-08	-1.05E-08	-1.08E-08	-1.06E-08	-1.12E-08	-1.06E-08	-1.06E-08	-9.29E-09	-9.03E-09	-8.69E-09	-8.04E-09	-8.13E-09	-8.37E-09	-8.48E-09
-1.00E-08	-1.07E-08	-9.79E-09	-9.60E-09	-9.37E-09	-1.08E-08	-1.02E-08	-1.02E-08	-8.49E-09	-8.52E-09	-8.70E-09	-7.82E-09	-7.49E-09	-8.19E-09	-8.30E-09
-9.79E-09	-1.02E-08	-9.01E-09	-8.74E-09	-8.85E-09	-1.01E-08	-9.38E-09	-9.38E-09	-8.45E-09	-8.32E-09	-8.40E-09	-8.00E-09	-7.06E-09	-7.87E-09	-8.20E-09
-9.49E-09	-9.53E-09	-7.69E-09	-8.01E-09	-8.31E-09	-8.85E-09	-8.41E-09	-8.31E-09	-8.29E-09	-7.83E-09	-7.99E-09	-7.12E-09	-5.96E-09	-7.38E-09	-7.81E-09
-8.96E-09	-9.35E-09	-5.84E-09	-7.49E-09	-7.33E-09	-7.52E-09	-7.41E-09	-7.64E-09	-7.30E-09	-6.82E-09	-7.60E-09	-6.06E-09	-5.01E-09	-7.18E-09	-7.87E-09
-8.48E-09	-8.51E-09	-6.19E-09	-7.08E-09	-6.12E-09	-6.02E-09	-5.98E-09	-6.68E-09	-6.09E-09	-5.99E-09	-6.77E-09	-5.90E-09	-4.79E-09	-7.62E-09	-8.43E-09
-7.68E-09	-7.15E-09	-6.64E-09	-6.22E-09	-4.42E-09	-4.24E-09	-4.21E-09	-5.24E-09	-5.07E-09	-4.88E-09	-5.26E-09	-5.98E-09	-5.72E-09	-7.58E-09	-7.99E-09
-6.66E-09	-6.08E-09	-5.37E-09	-3.99E-09	-1.93E-09	-1.68E-09	-2.06E-09	-3.06E-09	-3.61E-09	-3.63E-09	-3.60E-09	-6.21E-09	-6.39E-09	-7.33E-09	-7.13E-09
-5.81E-09	-5.35E-09	-3.76E-09	-1.48E-09	1.26E-09	1.57E-09	1.06E-09	3.47E-10	-7.97E-10	-1.69E-09	-2.38E-09	-5.60E-09	-6.20E-09	-6.98E-09	-6.84E-09
-4.88E-09	-4.41E-09	-2.22E-09	5.96E-10	5.03E-09	5.33E-09	5.15E-09	4.37E-09	2.71E-09	1.34E-09	-9.77E-10	-4.11E-09	-5.52E-09	-5.96E-09	-6.09E-09
-4.51E-09	-3.82E-09	-1.62E-09	1.86E-09	7.37E-09	9.33E-09	9.02E-09	8.81E-09	6.70E-09	5.05E-09	9.18E-10	-2.53E-09	-4.23E-09	-5.73E-09	-5.75E-09
-4.31E-09	-2.79E-09	-8.75E-10	2.37E-09	7.80E-09	1.12E-08	1.12E-08	1.19E-08	1.07E-08	9.25E-09	3.09E-09	-1.21E-09	-3.48E-09	-5.19E-09	-5.56E-09
-4.35E-09	-2.29E-09	-2.59E-10	2.97E-09	8.18E-09	1.11E-08	1.12E-08	1.25E-08	1.36E-08	1.21E-08	4.95E-09	-5.05E-10	-2.95E-09	-4.68E-09	-5.64E-09
-4.66E-09	-2.24E-09	2.90E-10	3.43E-09	8.73E-09	1.03E-08	1.09E-08	1.17E-08	1.36E-08	1.14E-08	4.55E-09	-1.62E-10	-3.13E-09	-4.81E-09	-5.20E-09
-5.39E-09	-2.38E-09	4.87E-10	3.74E-09	7.57E-09	8.51E-09	8.96E-09	9.41E-09	1.10E-08	7.38E-09	2.65E-09	-1.05E-09	-3.70E-09	-4.99E-09	-5.33E-09
-5.83E-09	-2.76E-09	-1.11E-10	3.05E-09	5.58E-09	5.27E-09	5.06E-09	5.53E-09	6.82E-09	3.40E-09	3.67E-10	-2.47E-09	-4.39E-09	-4.84E-09	-5.56E-09
-5.84E-09	-3.72E-09	-2.41E-09	1.15E-10	1.56E-09	1.45E-09	1.48E-09	1.54E-09	2.97E-09	6.50E-11	-1.81E-09	-3.53E-09	-4.84E-09	-5.48E-09	-5.73E-09
-5.90E-09	-5.04E-09	-4.31E-09	-2.74E-09	-2.27E-09	-1.92E-09	-1.36E-09	-1.02E-09	-1.46E-09	-2.78E-09	-3.99E-09	-4.29E-09	-5.34E-09	-6.03E-09	-5.92E-09
-6.33E-09	-6.13E-09	-5.21E-09	-4.23E-09	-4.78E-09	-3.74E-09	-3.63E-09	-2.23E-09	-3.27E-09	-4.57E-09	-5.47E-09	-5.22E-09	-5.64E-09	-6.17E-09	-5.42E-09
-6.55E-09	-7.36E-09	-6.00E-09	-5.72E-09	-6.05E-09	-4.70E-09	-4.38E-09	-2.83E-09	-3.51E-09	-5.17E-09	-5.67E-09	-5.74E-09	-6.02E-09	-6.26E-09	-4.95E-09
-6.78E-09	-7.87E-09	-6.82E-09	-6.39E-09	-6.52E-09	-5.71E-09	-5.37E-09	-4.13E-09	-3.92E-09	-5.65E-09	-6.41E-09	-6.04E-09	-6.38E-09	-6.58E-09	-5.00E-09
-7.14E-09	-8.05E-09	-6.99E-09	-6.15E-09	-7.10E-09	-6.95E-09	-6.95E-09	-5.13E-09	-5.03E-09	-6.65E-09	-6.52E-09	-6.19E-09	-7.23E-09	-7.34E-09	-5.43E-09
-7.21E-09	-8.24E-09	-7.58E-09	-6.89E-09	-7.75E-09	-7.71E-09	-8.20E-09	-6.46E-09	-5.97E-09	-7.76E-09	-6.49E-09	-6.73E-09	-7.87E-09	-7.97E-09	-7.05E-09
-7.44E-09	-8.18E-09	-8.58E-09	-7.85E-09	-8.23E-09	-8.31E-09	-9.16E-09	-7.95E-09	-6.74E-09	-8.51E-09	-6.64E-09	-7.27E-09	-8.33E-09	-8.57E-09	-7.28E-09
-8.75E-09	-8.56E-09	-9.08E-09	-8.45E-09	-8.39E-09	-8.27E-09	-9.90E-09	-8.82E-09	-8.03E-09	-8.93E-09	-8.17E-09	-7.32E-09	-8.28E-09	-8.95E-09	-7.48E-09
-9.74E-09	-9.01E-09	-9.28E-09	-8.74E-09	-8.53E-09	-8.61E-09	-1.02E-08	-9.57E-09	-9.20E-09	-9.32E-09	-9.22E-09	-7.50E-09	-8.26E-09	-9.09E-09	-7.74E-09
-9.87E-09	-9.54E-09	-9.31E-09	-8.45E-09	-8.76E-09	-8.89E-09	-1.02E-08	-1.00E-08	-9.37E-09	-9.72E-09	-9.91E-09	-8.08E-09	-8.86E-09	-9.19E-09	-8.24E-09
-9.63E-09	-8.99E-09	-8.69E-09	-8.15E-09	-8.54E-09	-9.13E-09	-9.88E-09	-1.02E-08	-9.85E-09	-1.01E-08	-9.78E-09	-8.63E-09	-9.33E-09	-9.48E-09	-8.57E-09
-9.41E-09	-8.30E-09	-8.83E-09	-8.29E-09	-8.62E-09	-9.30E-09	-9.55E-09	-1.04E-08	-1.07E-08	-1.07E-08	-9.86E-09	-9.18E-09	-9.72E-09	-9.26E-09	-9.16E-09
-8.85E-09	-8.30E-09	-9.15E-09	-8.53E-09	-8.57E-09	-9.55E-09	-9.51E-09	-1.06E-08	-1.17E-08	-1.10E-08	-1.05E-08	-9.57E-09	-9.81E-09	-9.38E-09	-9.78E-09
-8.71E-09	-8.08E-09	-9.31E-09	-8.79E-09	-8.57E-09	-1.01E-08	-9.76E-09	-1.03E-08	-1.14E-08	-1.14E-08	-1.09E-08	-9.80E-09	-9.55E-09	-9.33E-09	-9.72E-09
-8.54E-09	-8.11E-09	-9.11E-09	-9.11E-09	-8.68E-09	-1.02E-08	-9.88E-09	-1.03E-08	-1.09E-08	-1.15E-08	-1.07E-08	-9.57E-09	-9.27E-09	-8.68E-09	-8.71E-09
-8.06E-09	-7.95E-09	-8.64E-09	-8.82E-09	-8.80E-09	-9.64E-09	-9.65E-09	-1.03E-08	-1.07E-08	-1.09E-08	-1.00E-08	-9.08E-09	-8.60E-09	-7.59E-09	-7.67E-09
-7.14E-09	-7.02E-09	-8.02E-09	-8.67E-09	-8.73E-09	-9.18E-09	-9.18E-09	-9.45E-09	-1.01E-08	-1.06E-08	-9.11E-09	-8.22E-09	-7.94E-09	-7.34E-09	-7.67E-09
-7.13E-09	-6.84E-09	-7.64E-09	-8.14E-09	-8.00E-09	-8.61E-09	-8.44E-09	-8.76E-09	-8.88E-09	-9.73E-09	-8.35E-09	-7.16E-09	-7.80E-09	-7.48E-09	-7.86E-09
-7.08E-09	-6.41E-09	-6.93E-09	-7.46E-09	-6.88E-09	-7.66E-09	-7.38E-09	-8.06E-09	-8.06E-09	-8.68E-09	-7.90E-09	-6.89E-09	-7.76E-09	-7.94E-09	-7.60E-09
-6.65E-09	-5.45E-09	-5.74E-09	-6.74E-09	-5.09E-09	-6.90E-09	-6.79E-09	-7.61E-09	-7.42E-09	-7.41E-09	-6.94E-09	-6.34E-09	-6.87E-09	-7.66E-09	-6.79E-09
-5.83E-09	-4.83E-09	-4.96E-09	-5.85E-09	-5.82E-09	-6.27E-09	-6.14E-09	-7.03E-09	-6.79E-09	-6.18E-09	-5.89E-09	-5.59E-09	-6.80E-09	-6.64E-09	-5.97E-09
-5.42E-09	-4.72E-09	-4.76E-09	-5.25E-09	-5.48E-09	-5.94E-09	-5.75E-09	-6.25E-09	-6.20E-09	-5.00E-09	-4.27E-09	-4.83E-09	-6.15E-09	-6.01E-09	-5.85E-09
-5.31E-09	-4.56E-09	-4.97E-09	-4.98E-09	-4.86E-09	-5.26E-09	-5.34E-09	-5.92E-09	-5.65E-09	-3.69E-09	-3.48E-09	-4.36E-09	-5.54E-09	-5.29E-09	-5.63E-09
-4.97E-09	-4.30E-09	-4.39E-09	-5.19E-09	-4.65E-09	-4.59E-09	-4.81E-09	-5.30E-09	-5.27E-09	-3.46E-09	-3.60E-09	-4.30E-09	-5.09E-09	-4.95E-09	-5.22E-09
-4.57E-09	-3.93E-09	-4.02E-09	-5.09E-09	-4.12E-09	-3.99E-09	-4.48E-09	-4.25E-09	-4.89E-09	-4.12E-09	-4.41E-09	-4.49E-09	-4.44E-09	-4.92E-09	-4.39E-09
-4.07E-09	-3.81E-09	-3.40E-09	-4.54E-09	-4.34E-09	-3.71E-09	-3.87E-09	-3.36E-09	-4.41E-09	-4.45E-09	-4.55E-09	-4.23E-09	-3.60E-09	-4.03E-09	-3.83E-09
-3.86E-09	-3.80E-09	-3.49E-09	-4.25E-09	-4.71E-09	-3.71E-09	-3.00E-09	-3.23E-09	-4.16E-09	-3.63E-09	-4.11E-09	-4.16E-09	-3.22E-09	-3.55E-09	-3.51E-09
-3.97E-09	-3.63E-09	-3.37E-09	-4.14E-09	-4.23E-09	-3.45E-09	-2.37E-09	-3.40E-09	-3.94E-09	-2.88E-09	-3.83E-09	-3.73E-09	-3.18E-09	-3.38E-09	-3.25E-09
-3.99E-09	-3.42E-09	-3.22E-09	-3.94E-09	-3.60E-09	-2.79E-09	-2.84E-09	-3.42E-09	-3.52E-09	-2.10E-09	-3.36E-09	-3.31E-09	-2.75E-09	-3.17E-09	-2.90E-09
-3.43E-09	-3.09E-09	-3.10E-09	-3.49E-09	-3.64E-09	-2.97E-09	-3.40E-09	-3.38E-09	-3.15E-09	-1.61E-09	-2.92E-09	-2.42E-09	-2.49E-09	-2.64E-09	-2.12E-09
-2.93E-09	-2.70E-09	-2.67E-09	-2.83E-09	-3.34E-09	-3.15E-09	-3.26E-09	-3.26E-09	-2.82E-09	-1.68E-09	-2.09E-09	-1.60E-09	-2.09E-09	-1.92E-09	-2.27E-09
-2.32E-09	-2.00E-09	-1.81E-09	-2.28E-09	-3.18E-09	-3.27E-09	-2.29E-09	-2.67E-09	-2.29E-09	-1.82E-09	-1.57E-09	-1.13E-09	-1.57E-09	-1.50E-09	-2.45E-09
-1.55E-09	-1.06E-09	-1.20E-09	-1.92E-09	-2.24E-09	-2.49E-09	-1.49E-09	-1.65E-09	-1.35E-09	-1.43E-09	-1.17E-09	-8.75E-10	-9.75E-10	-1.19E-09	-2.02E-09
-9.23E-10	-8.43E-10	-1.10E-09	-1.56E-09	-1.16E-09	-1.37E-09	-1.16E-09	-1.15E-09	-8.53E-10	-1.25E-09	-1.20E-09	-7.52E-10	-1.07E-09	-9.88E-10	-1.25E-09
-1.33E-09	-5.11E-10	-9.48E-10	-1.16E-09	-6.87E-10	-7.35E-10	-1.02E-09	-1.05E-09	-7.49E-10	-1.16E-09	-8.67E-10	-1.01E-09	-9.29E-10	-6.21E-10	-8.05E-10
4.62E-10	-4.67E-10	-7.35E-10	-7.45E-10	-2.13E-10	-4.73E-10	-6.18E-10	-6.17E-10	-5.32E-11	-8.97E-10	-3.89E-10	-5.26E-10	-5.66E-10	1.11E-11	-6.07E-10
3.63E-10	-6.14E-10	-5.75E-10	-4.63E-10	-1.52E-10	4.83E-13	2.42E-11	-4.15E-10	6.41E-10	-7.24E-10	-4.18E-11	7.90E-11	5.58E-11	5.51E-10	-3.97E-10
8.32E-11	-2.95E-10	-3.05E-10	1.26E-10	-4.68E-10	5.46E-10	3.11E-10	-1.46E-10	2.53E-10	-4.37E-10	4.69E-12	6.50E-10	9.15E-10	9.37E-10	3.50E-10

4.82E-10	2.98E-10	3.44E-10	3.76E-10	-2.74E-10	8.69E-10	6.73E-10	4.58E-10	3.67E-10	3.53E-10	3.58E-10	8.20E-10	1.86E-09	1.73E-09	1.18E-09
9.69E-10	6.95E-10	1.04E-09	5.28E-10	2.83E-10	9.14E-10	9.76E-10	1.04E-09	6.83E-10	1.31E-09	8.34E-10	1.03E-09	2.51E-09	2.61E-09	1.69E-09
1.02E-09	1.50E-09	1.45E-09	1.06E-09	1.12E-09	9.96E-10	1.01E-09	1.41E-09	8.90E-10	1.21E-09	1.23E-09	1.12E-09	2.22E-09	2.70E-09	2.54E-09
1.54E-09	1.91E-09	1.71E-09	1.78E-09	1.82E-09	1.09E-09	1.23E-09	1.03E-09	1.33E-09	9.43E-10	1.79E-09	1.58E-09	1.91E-09	2.82E-09	3.10E-09
2.11E-09	2.61E-09	2.59E-09	2.24E-09	2.28E-09	1.43E-09	1.48E-09	1.14E-09	1.98E-09	1.20E-09	2.42E-09	2.47E-09	2.16E-09	3.41E-09	3.47E-09
2.72E-09	3.16E-09	2.99E-09	2.49E-09	2.62E-09	1.97E-09	1.93E-09	1.43E-09	2.69E-09	2.18E-09	2.95E-09	3.08E-09	3.00E-09	4.02E-09	3.97E-09
3.42E-09	3.55E-09	3.40E-09	2.61E-09	2.74E-09	2.51E-09	2.42E-09	1.83E-09	2.41E-09	3.23E-09	3.52E-09	3.64E-09	3.58E-09	4.73E-09	4.60E-09
4.08E-09	3.97E-09	3.67E-09	2.90E-09	3.02E-09	2.41E-09	2.75E-09	2.61E-09	3.00E-09	3.94E-09	3.85E-09	3.85E-09	3.38E-09	4.67E-09	4.42E-09
4.66E-09	4.38E-09	4.39E-09	3.73E-09	3.41E-09	3.08E-09	2.98E-09	3.48E-09	3.60E-09	3.91E-09	4.49E-09	3.84E-09	3.90E-09	4.41E-09	4.57E-09
5.53E-09	4.81E-09	5.08E-09	4.09E-09	4.04E-09	3.37E-09	3.70E-09	4.27E-09	4.08E-09	4.50E-09	5.53E-09	4.30E-09	4.76E-09	4.78E-09	5.13E-09
6.34E-09	5.20E-09	5.20E-09	4.59E-09	4.75E-09	4.15E-09	4.25E-09	4.73E-09	4.32E-09	5.16E-09	4.63E-09	4.75E-09	5.51E-09	5.17E-09	5.38E-09
7.07E-09	5.71E-09	5.52E-09	5.16E-09	5.91E-09	5.05E-09	4.65E-09	5.19E-09	4.73E-09	5.20E-09	4.62E-09	5.21E-09	6.03E-09	5.19E-09	5.69E-09
7.07E-09	6.30E-09	6.17E-09	5.70E-09	6.69E-09	5.95E-09	5.76E-09	5.77E-09	5.26E-09	5.30E-09	5.12E-09	5.52E-09	6.46E-09	5.53E-09	6.36E-09
7.91E-09	6.95E-09	6.53E-09	6.14E-09	6.57E-09	6.89E-09	6.18E-09	6.09E-09	5.64E-09	5.78E-09	5.73E-09	5.89E-09	6.17E-09	5.81E-09	6.91E-09
8.80E-09	7.99E-09	7.07E-09	6.59E-09	7.29E-09	7.06E-09	6.29E-09	6.55E-09	6.37E-09	6.46E-09	6.41E-09	6.71E-09	6.22E-09	6.27E-09	7.81E-09
9.51E-09	8.64E-09	7.90E-09	7.55E-09	7.95E-09	7.83E-09	6.33E-09	7.05E-09	6.69E-09	6.87E-09	6.86E-09	7.52E-09	7.07E-09	7.12E-09	8.27E-09
9.25E-09	9.05E-09	8.56E-09	8.71E-09	8.24E-09	8.85E-09	6.66E-09	7.90E-09	7.61E-09	7.70E-09	7.64E-09	7.63E-09	8.40E-09	8.45E-09	8.78E-09
9.55E-09	9.49E-09	9.00E-09	9.29E-09	8.52E-09	8.77E-09	7.70E-09	8.91E-09	8.64E-09	8.60E-09	8.70E-09	8.79E-09	9.05E-09	9.52E-09	9.34E-09
1.02E-08	1.03E-08	9.91E-09	9.30E-09	8.68E-09	9.10E-09	8.92E-09	9.48E-09	9.30E-09	9.20E-09	9.58E-09	9.94E-09	9.50E-09	9.88E-09	9.78E-09
1.09E-08	1.11E-08	1.03E-08	9.64E-09	9.57E-09	9.91E-09	9.54E-09	9.80E-09	1.06E-08	9.85E-09	1.03E-08	1.09E-08	1.03E-08	1.03E-08	1.05E-08
1.16E-08	1.16E-08	1.09E-08	1.03E-08	1.05E-08	1.05E-08	1.05E-08	1.04E-08	1.11E-08	1.08E-08	1.11E-08	1.16E-08	1.14E-08	1.16E-08	1.12E-08
1.23E-08	1.23E-08	1.19E-08	1.11E-08	1.15E-08	1.07E-08	1.14E-08	1.11E-08	1.17E-08	1.17E-08	1.22E-08	1.20E-08	1.27E-08	1.31E-08	1.20E-08
1.31E-08	1.31E-08	1.27E-08	1.19E-08	1.25E-08	1.14E-08	1.24E-08	1.23E-08	1.20E-08	1.25E-08	1.28E-08	1.28E-08	1.42E-08	1.45E-08	1.30E-08
1.33E-08	1.35E-08	1.36E-08	1.26E-08	1.29E-08	1.29E-08	1.34E-08	1.30E-08	1.24E-08	1.30E-08	1.35E-08	1.36E-08	1.58E-08	1.57E-08	1.38E-08
1.37E-08	1.45E-08	1.43E-08	1.38E-08	1.40E-08	1.41E-08	1.42E-08	1.34E-08	1.31E-08	1.37E-08	1.43E-08	1.43E-08	1.63E-08	1.64E-08	1.40E-08
1.46E-08	1.54E-08	1.53E-08	1.47E-08	1.45E-08	1.48E-08	1.48E-08	1.42E-08	1.38E-08	1.46E-08	1.50E-08	1.47E-08	1.51E-08	1.64E-08	1.43E-08
1.55E-08	1.59E-08	1.58E-08	1.55E-08	1.55E-08	1.51E-08	1.57E-08	1.51E-08	1.45E-08	1.58E-08	1.55E-08	1.53E-08	1.55E-08	1.52E-08	1.45E-08
1.65E-08	1.65E-08	1.65E-08	1.66E-08	1.60E-08	1.56E-08	1.61E-08	1.61E-08	1.55E-08	1.66E-08	1.61E-08	1.59E-08	1.61E-08	1.56E-08	1.53E-08
1.75E-08	1.72E-08	1.71E-08	1.70E-08	1.64E-08	1.62E-08	1.67E-08	1.66E-08	1.62E-08	1.70E-08	1.68E-08	1.63E-08	1.66E-08	1.64E-08	1.62E-08
1.80E-08	1.81E-08	1.79E-08	1.76E-08	1.73E-08	1.71E-08	1.73E-08	1.66E-08	1.69E-08	1.69E-08	1.75E-08	1.71E-08	1.74E-08	1.75E-08	1.70E-08
1.86E-08	1.90E-08	1.87E-08	1.83E-08	1.81E-08	1.84E-08	1.79E-08	1.72E-08	1.77E-08	1.74E-08	1.80E-08	1.76E-08	1.82E-08	1.83E-08	1.76E-08
1.93E-08	1.95E-08	1.95E-08	1.90E-08	1.88E-08	1.95E-08	1.90E-08	1.84E-08	1.85E-08	1.79E-08	1.89E-08	1.84E-08	1.88E-08	1.86E-08	1.81E-08
2.06E-08	2.04E-08	2.02E-08	1.93E-08	1.92E-08	1.95E-08	1.97E-08	1.97E-08	1.92E-08	1.88E-08	1.97E-08	1.94E-08	1.94E-08	1.95E-08	1.92E-08
2.14E-08	2.08E-08	2.08E-08	1.99E-08	1.97E-08	1.98E-08	2.01E-08	2.05E-08	1.94E-08	1.95E-08	2.04E-08	1.99E-08	2.01E-08	2.03E-08	2.04E-08
2.22E-08	2.16E-08	2.16E-08	2.07E-08	2.03E-08	2.03E-08	2.08E-08	2.10E-08	2.00E-08	1.97E-08	2.06E-08	2.05E-08	2.07E-08	2.10E-08	2.12E-08
2.33E-08	2.28E-08	2.25E-08	2.16E-08	2.15E-08	2.10E-08	2.15E-08	2.12E-08	2.05E-08	2.03E-08	2.09E-08	2.12E-08	2.11E-08	2.18E-08	2.19E-08
2.43E-08	2.38E-08	2.36E-08	2.29E-08	2.23E-08	2.13E-08	2.17E-08	2.16E-08	2.08E-08	2.09E-08	2.12E-08	2.19E-08	2.23E-08	2.28E-08	2.25E-08
2.54E-08	2.52E-08	2.47E-08	2.42E-08	2.28E-08	2.15E-08	2.19E-08	2.25E-08	2.16E-08	2.15E-08	2.18E-08	2.28E-08	2.31E-08	2.37E-08	2.31E-08
2.66E-08	2.68E-08	2.61E-08	2.52E-08	2.35E-08	2.26E-08	2.28E-08	2.33E-08	2.23E-08	2.21E-08	2.22E-08	2.35E-08	2.37E-08	2.38E-08	2.39E-08
2.79E-08	2.83E-08	2.73E-08	2.66E-08	2.46E-08	2.41E-08	2.38E-08	2.38E-08	2.31E-08	2.26E-08	2.28E-08	2.37E-08	2.41E-08	2.41E-08	2.44E-08
2.94E-08	2.94E-08	2.81E-08	2.74E-08	2.57E-08	2.53E-08	2.45E-08	2.45E-08	2.40E-08	2.32E-08	2.34E-08	2.41E-08	2.43E-08	2.44E-08	2.47E-08
3.11E-08	3.00E-08	2.88E-08	2.82E-08	2.68E-08	2.64E-08	2.58E-08	2.53E-08	2.48E-08	2.41E-08	2.45E-08	2.49E-08	2.47E-08	2.50E-08	2.47E-08
3.17E-08	3.07E-08	2.91E-08	2.90E-08	2.78E-08	2.78E-08	2.68E-08	2.65E-08	2.62E-08	2.53E-08	2.57E-08	2.55E-08	2.55E-08	2.55E-08	2.53E-08
3.15E-08	3.08E-08	2.94E-08	3.03E-08	2.88E-08	2.88E-08	2.80E-08	2.78E-08	2.75E-08	2.68E-08	2.68E-08	2.65E-08	2.62E-08	2.62E-08	2.64E-08
3.14E-08	3.13E-08	3.02E-08	3.12E-08	2.93E-08	3.01E-08	2.93E-08	2.94E-08	2.85E-08	2.81E-08	2.77E-08	2.77E-08	2.72E-08	2.73E-08	2.75E-08
3.21E-08	3.15E-08	3.07E-08	3.13E-08	3.01E-08	3.09E-08	3.04E-08	3.08E-08	2.96E-08	2.95E-08	2.91E-08	2.90E-08	2.84E-08	2.87E-08	2.86E-08
3.30E-08	3.21E-08	3.16E-08	3.19E-08	3.07E-08	3.11E-08	3.16E-08	3.19E-08	3.05E-08	3.07E-08	3.04E-08	3.00E-08	3.00E-08	2.98E-08	3.00E-08
3.36E-08	3.27E-08	3.26E-08	3.26E-08	3.13E-08	3.18E-08	3.27E-08	3.24E-08	3.12E-08	3.15E-08	3.16E-08	3.15E-08	3.17E-08	3.10E-08	3.11E-08
3.42E-08	3.34E-08	3.34E-08	3.32E-08	3.21E-08	3.26E-08	3.38E-08	3.28E-08	3.21E-08	3.24E-08	3.30E-08	3.28E-08	3.30E-08	3.22E-08	3.21E-08
3.52E-08	3.42E-08	3.43E-08	3.39E-08	3.31E-08	3.33E-08	3.41E-08	3.33E-08	3.32E-08	3.35E-08	3.38E-08	3.40E-08	3.43E-08	3.32E-08	3.33E-08

2.77E-16	2.39E-16	2.39E-16	2.43E-16	2.56E-16	2.66E-16	2.73E-16	2.53E-16	2.28E-16	2.08E-16	2.09E-16	1.95E-16	2.06E-16	1.86E-16	1.93E-16
2.85E-16	2.66E-16	2.51E-16	2.43E-16	2.46E-16	2.76E-16	2.67E-16	2.46E-16	2.37E-16	2.12E-16	1.91E-16	1.95E-16	1.95E-16	1.70E-16	1.91E-16
2.94E-16	2.83E-16	2.60E-16	2.41E-16	2.38E-16	2.70E-16	2.67E-16	2.42E-16	2.46E-16	2.31E-16	2.00E-16	2.04E-16	1.87E-16	1.77E-16	1.82E-16
2.94E-16	2.79E-16	2.42E-16	2.51E-16	2.43E-16	2.48E-16	2.52E-16	2.27E-16	2.41E-16	2.33E-16	2.12E-16	2.10E-16	1.91E-16	1.78E-16	1.71E-16
2.75E-16	2.64E-16	2.41E-16	2.57E-16	2.46E-16	2.31E-16	2.44E-16	2.13E-16	2.21E-16	2.22E-16	2.09E-16	1.99E-16	1.99E-16	1.75E-16	1.65E-16
2.68E-16	2.65E-16	2.37E-16	2.56E-16	2.39E-16	2.28E-16	2.32E-16	2.05E-16	2.02E-16	2.11E-16	2.00E-16	1.81E-16	1.99E-16	1.82E-16	1.67E-16
2.62E-16	2.57E-16	2.38E-16	2.44E-16	2.36E-16	2.24E-16	2.29E-16	1.92E-16	1.87E-16	2.01E-16	1.96E-16	1.74E-16	1.98E-16	1.91E-16	1.65E-16
2.44E-16	2.44E-16	2.31E-16	2.30E-16	2.34E-16	2.22E-16	2.33E-16	1.87E-16	1.86E-16	1.87E-16	1.88E-16	1.70E-16	1.93E-16	1.87E-16	1.53E-16
2.29E-16	2.37E-16	2.35E-16	2.16E-16	2.25E-16	2.06E-16	2.31E-16	1.87E-16	1.83E-16	1.69E-16	1.71E-16	1.65E-16	1.74E-16	1.61E-16	1.39E-16
2.13E-16	2.28E-16	2.35E-16	2.10E-16	2.26E-16	1.97E-16	2.28E-16	1.86E-16	1.68E-16	1.53E-16	1.54E-16	1.57E-16	1.63E-16	1.39E-16	1.24E-16
2.06E-16	2.11E-16	2.24E-16	2.01E-16	2.19E-16	2.00E-16	2.22E-16	1.81E-16	1.68E-16	1.53E-16	1.54E-16	1.54E-16	1.57E-16	1.25E-16	1.17E-16
2.03E-16	2.01E-16	2.01E-16	1.92E-16	2.02E-16	1.94E-16	2.11E-16	1.76E-16	1.70E-16	1.59E-16	1.52E-16	1.47E-16	1.54E-16	1.31E-16	1.18E-16
1.99E-16	1.98E-16	1.85E-16	1.77E-16	1.91E-16	1.86E-16	2.01E-16	1.68E-16	1.61E-16	1.51E-16	1.44E-16	1.36E-16	1.40E-16	1.40E-16	1.19E-16
1.86E-16	1.94E-16	1.90E-16	1.77E-16	1.90E-16	1.78E-16	1.88E-16	1.58E-16	1.48E-16	1.39E-16	1.37E-16	1.31E-16	1.28E-16	1.35E-16	1.14E-16
1.79E-16	1.84E-16	1.79E-16	1.73E-16	1.95E-16	1.82E-16	1.81E-16	1.57E-16	1.45E-16	1.34E-16	1.33E-16	1.22E-16	1.23E-16	1.24E-16	1.09E-16
1.88E-16	1.87E-16	1.72E-16	1.58E-16	1.80E-16	1.73E-16	1.75E-16	1.54E-16	1.43E-16	1.38E-16	1.27E-16	1.19E-16	1.17E-16	1.11E-16	1.05E-16
1.84E-16	1.88E-16	1.71E-16	1.55E-16	1.69E-16	1.46E-16	1.57E-16	1.42E-16	1.36E-16	1.37E-16	1.28E-16	1.19E-16	1.07E-16	9.86E-17	9.65E-17
1.79E-16	1.72E-16	1.63E-16	1.48E-16	1.59E-16	1.29E-16	1.31E-16	1.22E-16	1.25E-16	1.23E-16	1.20E-16	1.18E-16	1.08E-16	9.00E-17	8.80E-17
1.65E-16	1.54E-16	1.49E-16	1.26E-16	1.29E-16	1.03E-16	1.11E-16	1.18E-16	1.18E-16	1.09E-16	1.06E-16	1.15E-16	1.05E-16	8.65E-17	8.73E-17
1.57E-16	1.42E-16	1.38E-16	1.13E-16	1.09E-16	8.59E-17	1.03E-16	1.16E-16	1.15E-16	1.11E-16	1.01E-16	1.03E-16	9.81E-17	9.37E-17	8.92E-17
1.50E-16	1.30E-16	1.29E-16	1.12E-16	1.01E-16	8.35E-17	9.87E-17	1.18E-16	1.10E-16	1.07E-16	1.02E-16	1.00E-16	9.72E-17	8.95E-17	8.38E-17
1.38E-16	1.27E-16	1.26E-16	1.23E-16	1.06E-16	9.14E-17	9.98E-17	1.20E-16	1.10E-16	9.66E-17	9.23E-17	9.02E-17	8.94E-17	8.60E-17	8.24E-17
1.27E-16	1.27E-16	1.17E-16	1.21E-16	1.21E-16	1.12E-16	1.10E-16	1.24E-16	9.86E-17	8.96E-17	8.28E-17	7.86E-17	7.78E-17	7.76E-17	7.89E-17
1.12E-16	1.27E-16	1.10E-16	1.16E-16	1.13E-16	1.25E-16	1.13E-16	1.13E-16	8.63E-17	8.16E-17	7.55E-17	6.46E-17	6.61E-17	7.00E-17	7.19E-17
1.01E-16	1.15E-16	9.59E-17	9.23E-17	8.78E-17	1.16E-16	1.03E-16	1.04E-16	7.21E-17	7.25E-17	7.57E-17	6.12E-17	5.62E-17	6.71E-17	6.90E-17
9.58E-17	1.04E-16	8.12E-17	7.64E-17	7.84E-17	1.01E-16	8.79E-17	8.79E-17	7.14E-17	6.92E-17	7.05E-17	6.39E-17	4.99E-17	6.19E-17	6.72E-17
9.00E-17	9.08E-17	5.92E-17	6.42E-17	6.90E-17	7.83E-17	7.07E-17	6.91E-17	6.87E-17	6.13E-17	6.39E-17	5.08E-17	3.55E-17	5.45E-17	6.10E-17
8.03E-17	8.74E-17	3.41E-17	5.60E-17	5.37E-17	5.66E-17	5.49E-17	5.84E-17	5.33E-17	4.65E-17	5.78E-17	3.68E-17	2.51E-17	5.15E-17	6.20E-17
7.20E-17	7.24E-17	3.83E-17	5.01E-17	3.74E-17	3.62E-17	3.58E-17	4.46E-17	3.70E-17	3.59E-17	4.58E-17	3.48E-17	2.29E-17	5.81E-17	7.11E-17
5.90E-17	5.11E-17	4.41E-17	3.87E-17	1.95E-17	1.79E-17	1.78E-17	2.75E-17	2.57E-17	2.38E-17	2.77E-17	3.58E-17	3.27E-17	5.74E-17	6.39E-17
4.43E-17	3.69E-17	2.88E-17	1.59E-17	3.73E-18	2.82E-18	4.24E-18	9.37E-18	1.30E-17	1.32E-17	1.30E-17	3.86E-17	4.08E-17	5.38E-17	5.08E-17

3.37E-17	2.86E-17	1.41E-17	2.18E-18	1.58E-18	2.47E-18	1.13E-18	1.21E-19	6.35E-19	2.84E-18	5.65E-18	3.14E-17	3.85E-17	4.87E-17	4.67E-17
2.38E-17	1.94E-17	4.92E-18	3.55E-19	2.53E-17	2.85E-17	2.65E-17	1.91E-17	7.36E-18	1.79E-18	9.55E-19	1.69E-17	3.05E-17	3.55E-17	3.70E-17
2.04E-17	1.46E-17	2.62E-18	3.44E-18	5.44E-17	8.71E-17	8.14E-17	7.76E-17	4.49E-17	2.55E-17	8.42E-19	6.43E-18	1.79E-17	3.28E-17	3.31E-17
1.86E-17	7.79E-18	7.66E-19	5.62E-18	6.09E-17	1.26E-16	1.27E-16	1.42E-16	1.14E-16	8.56E-17	9.57E-18	1.46E-18	1.21E-17	2.69E-17	3.09E-17
1.89E-17	5.24E-18	6.72E-20	8.83E-18	6.70E-17	1.24E-16	1.27E-16	1.56E-16	1.86E-16	1.46E-16	2.45E-17	2.55E-19	8.70E-18	2.19E-17	3.18E-17
2.17E-17	5.01E-18	8.41E-20	1.18E-17	7.62E-17	1.07E-16	1.18E-16	1.38E-16	1.84E-16	1.30E-16	2.07E-17	2.61E-20	9.78E-18	2.31E-17	2.71E-17
2.90E-17	5.65E-18	2.37E-19	1.40E-17	5.73E-17	7.24E-17	8.02E-17	8.86E-17	1.20E-16	5.45E-17	7.02E-18	1.10E-18	1.37E-17	2.49E-17	2.84E-17
3.40E-17	7.64E-18	1.24E-20	9.28E-18	3.11E-17	2.78E-17	2.56E-17	3.06E-17	4.65E-17	1.16E-17	1.34E-19	6.11E-18	1.93E-17	2.34E-17	3.09E-17
3.41E-17	1.39E-17	5.81E-18	1.33E-20	2.44E-18	2.10E-18	2.20E-18	2.38E-18	8.82E-18	4.23E-21	3.27E-18	1.24E-17	2.34E-17	3.00E-17	3.29E-17
3.48E-17	2.54E-17	1.86E-17	7.50E-18	5.14E-18	3.69E-18	1.84E-18	1.04E-18	2.14E-18	7.73E-18	1.59E-17	1.84E-17	2.85E-17	3.63E-17	3.51E-17
4.01E-17	3.76E-17	2.71E-17	1.79E-17	2.29E-17	1.40E-17	1.32E-17	4.99E-18	1.07E-17	2.09E-17	2.99E-17	2.72E-17	3.18E-17	3.81E-17	2.94E-17
4.29E-17	5.41E-17	3.60E-17	3.27E-17	3.66E-17	2.21E-17	1.91E-17	8.02E-18	1.23E-17	2.67E-17	3.21E-17	3.29E-17	3.63E-17	3.92E-17	2.45E-17
4.59E-17	6.19E-17	4.64E-17	4.08E-17	4.25E-17	3.26E-17	2.88E-17	1.70E-17	1.54E-17	3.19E-17	4.11E-17	3.64E-17	4.08E-17	4.33E-17	2.50E-17
5.10E-17	6.47E-17	4.88E-17	3.79E-17	5.05E-17	4.83E-17	4.83E-17	2.63E-17	2.53E-17	4.42E-17	4.25E-17	3.83E-17	5.23E-17	5.38E-17	2.95E-17
5.20E-17	6.78E-17	5.75E-17	4.74E-17	6.01E-17	5.94E-17	6.72E-17	4.17E-17	3.56E-17	6.02E-17	4.21E-17	4.52E-17	6.19E-17	6.36E-17	4.97E-17
5.54E-17	6.69E-17	7.36E-17	6.16E-17	6.77E-17	6.90E-17	8.40E-17	6.32E-17	4.55E-17	7.24E-17	4.41E-17	5.29E-17	6.94E-17	7.35E-17	5.30E-17
7.66E-17	7.32E-17	8.25E-17	7.14E-17	7.03E-17	6.83E-17	9.79E-17	7.77E-17	6.45E-17	7.97E-17	6.67E-17	5.36E-17	6.85E-17	8.02E-17	5.60E-17
9.49E-17	8.13E-17	8.61E-17	7.65E-17	7.28E-17	7.41E-17	1.05E-16	9.16E-17	8.46E-17	8.68E-17	8.51E-17	5.62E-17	6.82E-17	8.27E-17	6.00E-17
9.75E-17	9.10E-17	8.67E-17	7.13E-17	7.68E-17	7.90E-17	1.04E-16	1.00E-16	8.77E-17	9.45E-17	9.82E-17	6.53E-17	7.85E-17	8.44E-17	6.79E-17
9.28E-17	8.08E-17	7.55E-17	6.64E-17	7.29E-17	8.33E-17	9.76E-17	1.05E-16	9.70E-17	1.02E-16	9.57E-17	7.44E-17	8.71E-17	8.99E-17	7.35E-17
8.85E-17	6.89E-17	7.80E-17	6.87E-17	7.44E-17	8.66E-17	9.12E-17	1.08E-16	1.14E-16	1.15E-16	9.71E-17	8.42E-17	9.45E-17	8.57E-17	8.40E-17
7.83E-17	6.89E-17	8.37E-17	7.27E-17	7.35E-17	9.12E-17	9.05E-17	1.13E-16	1.37E-16	1.21E-16	1.11E-16	9.16E-17	9.62E-17	8.81E-17	9.57E-17
7.59E-17	6.53E-17	8.67E-17	7.73E-17	7.34E-17	1.01E-16	9.53E-17	1.06E-16	1.31E-16	1.31E-16	1.19E-16	9.60E-17	9.13E-17	8.71E-17	9.46E-17
7.29E-17	6.58E-17	8.30E-17	8.30E-17	7.53E-17	1.03E-16	9.75E-17	1.06E-16	1.19E-16	1.32E-16	1.14E-16	9.17E-17	8.60E-17	7.53E-17	7.58E-17
6.49E-17	6.32E-17	7.46E-17	7.78E-17	7.74E-17	9.30E-17	9.32E-17	1.07E-16	1.14E-16	1.19E-16	1.00E-16	8.25E-17	7.39E-17	5.76E-17	5.88E-17
5.10E-17	4.93E-17	6.43E-17	7.52E-17	7.61E-17	8.43E-17	8.42E-17	8.94E-17	1.01E-16	1.12E-16	8.29E-17	6.76E-17	6.31E-17	5.39E-17	5.88E-17
5.08E-17	4.67E-17	5.84E-17	6.62E-17	6.40E-17	7.42E-17	7.12E-17	7.68E-17	7.88E-17	9.47E-17	6.97E-17	5.13E-17	6.08E-17	5.60E-17	6.18E-17
5.01E-17	4.11E-17	4.80E-17	5.56E-17	4.74E-17	5.86E-17	5.44E-17	6.50E-17	6.50E-17	7.53E-17	6.24E-17	4.74E-17	6.02E-17	6.31E-17	5.77E-17
4.42E-17	2.97E-17	3.29E-17	4.55E-17	2.59E-17	4.76E-17	4.61E-17	5.79E-17	5.50E-17	5.49E-17	4.82E-17	4.02E-17	4.72E-17	5.87E-17	4.62E-17
3.40E-17	2.33E-17	2.46E-17	3.43E-17	3.39E-17	3.93E-17	3.78E-17	4.95E-17	4.61E-17	3.82E-17	3.46E-17	3.13E-17	4.62E-17	4.42E-17	3.56E-17
2.93E-17	2.23E-17	2.27E-17	2.76E-17	3.00E-17	3.53E-17	3.31E-17	3.90E-17	3.84E-17	2.50E-17	1.82E-17	2.33E-17	3.78E-17	3.61E-17	3.43E-17
2.82E-17	2.08E-17	2.47E-17	2.48E-17	2.36E-17	2.77E-17	2.85E-17	3.50E-17	3.19E-17	1.36E-17	1.21E-17	1.90E-17	3.07E-17	2.80E-17	3.17E-17
2.47E-17	1.85E-17	1.93E-17	2.69E-17	2.16E-17	2.11E-17	2.31E-17	2.81E-17	2.78E-17	1.19E-17	1.30E-17	1.85E-17	2.59E-17	2.45E-17	2.72E-17
2.08E-17	1.54E-17	1.62E-17	2.59E-17	1.70E-17	1.59E-17	2.01E-17	1.81E-17	2.39E-17	1.70E-17	1.95E-17	2.02E-17	1.97E-17	2.42E-17	1.93E-17
1.66E-17	1.45E-17	1.16E-17	2.06E-17	1.88E-17	1.38E-17	1.50E-17	1.13E-17	1.95E-17	1.98E-17	2.07E-17	1.79E-17	1.30E-17	1.63E-17	1.46E-17
1.49E-17	1.44E-17	1.22E-17	1.81E-17	2.22E-17	1.38E-17	9.01E-18	1.04E-17	1.73E-17	1.32E-17	1.69E-17	1.73E-17	1.04E-17	1.26E-17	1.24E-17
1.58E-17	1.32E-17	1.13E-17	1.72E-17	1.79E-17	1.19E-17	5.63E-18	1.15E-17	1.55E-17	8.32E-18	1.47E-17	1.39E-17	1.01E-17	1.14E-17	1.05E-17
1.59E-17	1.17E-17	1.04E-17	1.56E-17	1.30E-17	7.80E-18	8.05E-18	1.17E-17	1.24E-17	4.39E-18	1.13E-17	1.10E-17	7.55E-18	1.00E-17	8.44E-18
1.18E-17	9.56E-18	9.62E-18	1.22E-17	1.32E-17	8.80E-18	1.16E-17	1.14E-17	9.94E-18	2.59E-18	8.50E-18	5.84E-18	6.20E-18	6.95E-18	4.49E-18
8.61E-18	7.27E-18	7.15E-18	8.00E-18	1.12E-17	9.90E-18	1.06E-17	1.06E-17	7.97E-18	2.84E-18	4.35E-18	2.57E-18	4.37E-18	3.68E-18	5.17E-18
5.40E-18	4.00E-18	3.28E-18	5.19E-18	1.01E-17	1.07E-17	5.26E-18	7.11E-18	5.26E-18	3.33E-18	2.48E-18	1.27E-18	2.45E-18	2.25E-18	6.01E-18
2.40E-18	1.13E-18	1.43E-18	3.69E-18	5.01E-18	6.18E-18	2.23E-18	2.72E-18	1.82E-18	2.04E-18	1.37E-18	7.65E-19	9.50E-19	1.41E-18	4.10E-18
8.51E-19	7.11E-19	1.21E-18	2.44E-18	1.34E-18	1.87E-18	1.36E-18	1.32E-18	7.27E-19	1.56E-18	1.45E-18	5.65E-19	1.15E-18	9.76E-19	1.57E-18
1.78E-20	2.61E-19	8.98E-19	1.36E-18	4.72E-19	5.40E-19	1.04E-18	1.11E-18	5.61E-19	1.35E-18	7.52E-19	1.03E-18	8.63E-19	3.85E-19	6.47E-19
2.13E-19	2.19E-19	5.40E-19	5.55E-19	4.55E-20	2.23E-19	3.82E-19	3.80E-19	2.83E-21	8.04E-19	1.51E-19	2.77E-19	3.20E-19	1.24E-22	3.69E-19
1.32E-19	3.77E-19	3.31E-19	2.14E-19	2.30E-20	2.33E-25	5.88E-22	1.72E-19	4.11E-19	5.23E-19	1.74E-21	6.24E-21	3.12E-21	3.04E-19	1.57E-19
6.93E-21	8.71E-20	9.31E-20	1.59E-20	2.19E-19	2.98E-19	9.70E-20	2.13E-20	6.39E-20	1.91E-19	2.20E-23	4.22E-19	8.38E-19	8.78E-19	1.22E-19
2.32E-19	8.86E-20	1.18E-19	1.42E-19	7.53E-20	7.54E-19	4.53E-19	2.09E-19	1.35E-19	1.25E-19	1.28E-19	6.73E-19	3.47E-18	2.99E-18	1.40E-18
9.39E-19	4.83E-19	1.08E-18	2.79E-19	8.02E-20	8.35E-19	9.53E-19	1.08E-18	4.66E-19	1.71E-18	6.96E-19	1.06E-18	6.32E-18	6.83E-18	2.85E-18
1.04E-18	2.25E-18	2.11E-18	1.12E-18	1.25E-18	9.92E-19	1.02E-18	2.00E-18	7.92E-19	1.45E-18	1.52E-18	1.25E-18	4.94E-18	7.29E-18	6.44E-18
2.37E-18	3.63E-18	2.93E-18	3.18E-18	3.33E-18	1.19E-18	1.51E-18	1.05E-18	1.76E-18	8.89E-19	3.21E-18	2.51E-18	3.65E-18	7.95E-18	9.61E-18
4.46E-18	6.81E-18	6.71E-18	5.03E-18	5.20E-18	2.05E-18	2.18E-18	1.30E-18	3.91E-18	1.44E-18	5.83E-18	6.10E-18	4.65E-18	1.16E-17	1.20E-17
7.38E-18	1.00E-17	8.94E-18	6.19E-18	6.87E-18	3.89E-18	3.74E-18	2.04E-18	7.21E-18	4.77E-18	8.70E-18	9.50E-18	8.98E-18	1.62E-17	1.57E-17
1.17E-17	1.26E-17	1.16E-17	6.84E-18	7.49E-18	6.31E-18	5.88E-18	3.37E-18	5.78E-18	1.04E-17	1.24E-17	1.33E-17	1.28E-17	2.24E-17	2.11E-17
1.66E-17	1.58E-17	1.35E-17	8.41E-18	9.14E-18	5.79E-18	7.56E-18	6.79E-18	9.01E-18	1.55E-17	1.48E-17	1.49E-17	1.14E-17	2.18E-17	1.96E-17
2.17E-17	1.92E-17	1.93E-17	1.39E-17	1.16E-17	9.49E-18	8.87E-18	1.21E-17	1.29E-17	1.53E-17	2.02E-17	1.47E-17	1.52E-17	1.95E-17	2.09E-17
3.06E-17	2.31E-17	2.58E-17	1.67E-17	1.63E-17	1.14E-17	1.37E-17	1.82E-17	1.67E-17	2.03E-17	3.05E-17	1.85E-17	2.26E-17	2.29E-17	2.63E-17
4.02E-17	2.71E-17	2.70E-17	2.10E-17	2.25E-17	1.72E-17	1.81E-17	2.24E-17	1.87E-17	2.66E-17	2.14E-17	2.25E-17	3.03E-17	2.67E-17	2.89E-17
5.00E-17	3.26E-17	3.05E-17	2.67E-17	3.49E-17	2.55E-17	2.17E-17	2.70E-17	2.24E-17	2.70E-17	2.13E-17	2.72E-17	3.63E-17	2.69E-17	3.24E-17
5.00E-17	3.96E-17	3.81E-17	3.25E-17	4.48E-17	3.54E-17	3.32E-17	3.33E-17	2.76E-17	2.81E-17	2.62E-17	3.05E-17	4.18E-17	3.06E-17	4.04E-17
6.26E-17	4.83E-17	4.27E-17	3.77E-17	4.32E-17	4.74E-17	3.83E-17	3.71E-17	3.18E-17	3.34E-17	3.28E-17	3.46E-17	3.81E-17	3.38E-17	4.77E-17
7.75E-17	6.39E-17	4.99E-17	4.34E-17	5.31E-17	4.98E-17	3.96E-17	4.29E-17	4.06E-17	4.17E-17	4.10E-17	4.50E-17	3.87E-17	3.93E-17	6.09E-17
9.04E-17	7.46E-17	6.24E-17	5.70E-17	6.31E-17	6.13E-17	4.01E-17	4.97E-17	4.47E-17	4.73E-17	4.71E-17	5.66E-17	4.99E-17	5.08E-17	6.84E-17
8.55E-17	8.19E-17	7.32E-17	7.59E-17	6.80E-17	7.83E-17	4.44E-17	6.24E-17	5.79E-17	5.93E-17	5.84E-17	5.83E-17	7.05E-17	7.13E-17	7.71E-17
9.13E-17	9.00E-17	8.10E-17	8.63E-17	7.26E-17	7.69E-17	5.93E-17	7.93E-17	7.47E-17	7.40E-17	7.57E-17	7.73E-17	8.20E-17	9.07E-17	8.72E-17
1.05E-16	1.05E-16	9.81E-17	8.65E-17	7.53E-17	8.29E-17	7.95E-17	8.99E-17	8.65E-17	8.47E-17	9.18E-17	9.87E-17	9.02E-17	9.77E-17	9.57E-17
1.20E-16	1.23E-16	1.06E-16	9.30E-17	9.15E-17	9.82E-17	9.10E-17	9.60E-17	1.12E-16	9.70E-17	1.05E-16	1.19E-16	1.05E-16	1.06E-16	1.10E-16
1.34E-16	1.35E-16	1.18E-16	1.06E-16	1.11E-16	1.11E-16	1.10E-16	1.09E-16	1.24E-16	1.17E-16	1.23E-16	1.35E-16	1.30E-16	1.34E-16	1.26E-16
1.50E-16	1.50E-16	1.41E-16	1.22E-16	1.32E-16	1.15E-16	1.30E-16	1.24E-16	1.36E-16	1.36E-16	1.48E-16	1.45E-16	1.61E-16	1.73E-16	1.45E-16
1.71E-16	1.72E-16	1.62E-16	1.41E-16	1.55E-16	1.30E-16	1.54E-16	1.52E-16	1.45E-16	1.56E-16	1.65E-16	1.64E-16	2.00E-16	2.10E-16	1.68E-16
1.76E-16	1.83E-16	1.85E-16	1.58E-16	1.67E-16	1.66E-16	1.78E-16	1.69E-16	1.55E-16	1.70E-16	1.81E-16	1.85E-16	2.50E-16	2.46E-16	1.91E-16
1.89E-16	2.11E-16	2.06E-16	1.90E-16	1.97E-16	2.00E-16	2.00E-16	1.79E-16	1.72E-16	1.88E-16	2.05E-16	2.04E-16	2.66E-16	2.70E-16	1.97E-16
2.13E-16	2.37E-16	2.33E-16	2.15E-16	2.09E-16	2.18E-16	2.20E-16	2.01E-16	1.90E-16	2.14E-16	2.25E-16	2.15E-16	2.29E-16	2.70E-16	2.06E-16
2.41E-16	2.52E-16	2.51E-16	2.40E-16	2.39E-16	2.29E-16	2.45E-16	2.27E-16	2.09E-16	2.50E-16	2.39E-16	2.36E-16	2.41E-16	2.32E-16	2.09E-16
2.73E-16	2.73E-16	2.74E-16	2.75E-16	2.57E-16	2.42E-16	2.59E-16	2.60E-16	2.40E-16	2.75E-16	2.59E-16	2.54E-16	2.60E-16	2.42E-16	2.33E-16
3.05E-16	2.97E-16	2.93E-16	2.89E-16	2.68E-16	2.62E-16	2.79E-16	2.74E-16	2.61E-16	2.90E-16	2.82E-16	2.66E-16	2.75E-16	2.69E-16	2.62E-16
3.24E-16	3.28E-16	3.20E-16	3.08E-16	3.00E-16	2.94E-16	2.98E-16	2.76E-16	2.84E-16	2.84E-16	3.05E-16	2.91E-16	3.04E-16	3.05E-16	2.88E-16
3.44E-16	3.59E-16	3.51E-16	3.34E-16	3.29E-16	3.39E-16	3.22E-16	2.97E-16	3.14E-16	3.02E-16	3.23E-16	3.08E-16	3.33E-16	3.35E-16	3.11E-16
3.73E-16	3.82E-16	3.80E-16	3.61E-16	3.53E-16	3.79E-16	3.60E-16	3.39E-16	3.41E-16	3.21E-16	3.56E-16	3.39E-16	3.54E-16	3.45E-16	3.28E-16
4.23E-16	4.15E-16	4.09E-16	3.73E-16	3.70E-16	3.82E-16	3.88E-16	3.88E-16	3.68E-16	3.54E-16	3.89E-16	3.75E-16	3.74E-16	3.82E-16	3.69E-16
4.59E-16	4.31E-16	4.32E-16	3.95E-16	3.88E-16	3.94E-16	4.03E-16	4.19E-16	3.77E-16	3.78E-16	4.14E-16	3.95E-16	4.05E-16	4.12E-16	4.14E-16
4.92E-16	4.67E-16	4.69E-16	4.27E-16	4.12E-16	4.13E-16	4.35E-16	4.41E-16	4.01E-16	3.87E-16	4.23E-16	4.22E-16	4.27E-16	4.41E-16	4.48E-16
5.44E-16	5.19E-16	5.08E-16	4.68E-16	4.61E-16	4.41E-16	4.60E-16	4.50E-16	4.22E-16	4.10E-16	4.36E-16	4.48E-16	4.45E-16	4.75E-16	4.81E-16
5.93E-16	5.66E-16	5.58E-16	5.24E-16	4.99E-16	4.52E-16	4.70E-16	4.66E-16	4.34E-16	4.35E-16	4.51E-16	4.78E-16	4.98E-16	5.21E-16	5.05E-16
6.47E-16	6.36E-16	6.09E-16	5.84E-16	5.21E-16	4.61E-16	4.81E-16	5.07E-16	4.65E-16	4.63E-16	4.73E-16	5.19E-16	5.35E-16	5.60E-16	5.35E-16
7.06E-16	7.19E-16	6.79E-16	6.33E-16	5.54E-16	5.10E-16	5.19E-16	5.43E-16	4.99E-16	4.88E-16	4.91E-16	5.51E-16	5.63E-16	5.69E-16	5.71E-16

7.80E-16	7.99E-16	7.43E-16	7.09E-16	6.07E-16	5.83E-16	5.68E-16	5.66E-16	5.35E-16	5.09E-16	5.19E-16	5.60E-16	5.81E-16	5.79E-16	5.95E-16
8.65E-16	8.63E-16	7.89E-16	7.50E-16	6.60E-16	6.38E-16	6.00E-16	6.01E-16	5.75E-16	5.40E-16	5.48E-16	5.82E-16	5.92E-16	5.94E-16	6.11E-16
9.64E-16	9.01E-16	8.27E-16	7.94E-16	7.19E-16	6.99E-16	6.63E-16	6.41E-16	6.17E-16	5.83E-16	6.00E-16	6.20E-16	6.13E-16	6.24E-16	6.09E-16
1.01E-15	9.40E-16	8.46E-16	8.40E-16	7.71E-16	7.70E-16	7.19E-16	7.02E-16	6.85E-16	6.41E-16	6.61E-16	6.53E-16	6.51E-16	6.51E-16	6.39E-16
9.90E-16	9.48E-16	8.67E-16	9.19E-16	8.28E-16	8.32E-16	7.83E-16	7.71E-16	7.57E-16	7.17E-16	7.16E-16	7.04E-16	6.85E-16	6.88E-16	6.99E-16
9.85E-16	9.80E-16	9.14E-16	9.76E-16	8.60E-16	9.07E-16	8.58E-16	8.63E-16	8.13E-16	7.87E-16	7.70E-16	7.70E-16	7.40E-16	7.46E-16	7.57E-16
1.03E-15	9.90E-16	9.45E-16	9.81E-16	9.06E-16	9.52E-16	9.23E-16	9.48E-16	8.77E-16	8.68E-16	8.48E-16	8.39E-16	8.07E-16	8.25E-16	8.17E-16
1.09E-15	1.03E-15	9.97E-16	1.02E-15	9.46E-16	9.67E-16	1.00E-15	1.01E-15	9.30E-16	9.40E-16	9.23E-16	9.01E-16	8.98E-16	8.89E-16	8.99E-16
1.13E-15	1.07E-15	1.06E-15	1.06E-15	9.78E-16	1.01E-15	1.07E-15	1.05E-15	9.71E-16	9.90E-16	9.95E-16	9.94E-16	1.00E-15	9.64E-16	9.68E-16
1.17E-15	1.12E-15	1.12E-15	1.10E-15	1.03E-15	1.06E-15	1.15E-15	1.07E-15	1.03E-15	1.05E-15	1.09E-15	1.08E-15	1.09E-15	1.04E-15	1.03E-15
1.24E-15	1.17E-15	1.17E-15	1.15E-15	1.10E-15	1.11E-15	1.16E-15	1.11E-15	1.10E-15	1.13E-15	1.14E-15	1.15E-15	1.18E-15	1.10E-15	1.11E-15
2.62E-14	2.53E-14	2.41E-14	2.34E-14	2.27E-14	2.27E-14	2.30E-14	2.25E-14	2.16E-14	2.12E-14	2.09E-14	2.06E-14	2.12E-14	2.12E-14	2.06E-14

-1.31E-08	-1.24E-08	-1.25E-08	-1.16E-08	-1.09E-08	-9.18E-09	-1.09E-08	-1.14E-08	-1.15E-08	-1.03E-08	-1.04E-08	-1.16E-08	-1.10E-08
-1.27E-08	-1.17E-08	-1.20E-08	-1.12E-08	-1.08E-08	-9.19E-09	-1.06E-08	-1.08E-08	-1.12E-08	-1.03E-08	-9.88E-09	-1.09E-08	-1.00E-08
-1.27E-08	-1.19E-08	-1.22E-08	-1.11E-08	-1.08E-08	-9.81E-09	-1.06E-08	-1.05E-08	-1.06E-08	-1.05E-08	-9.07E-09	-9.99E-09	-8.81E-09
-1.30E-08	-1.23E-08	-1.22E-08	-1.13E-08	-1.11E-08	-9.82E-09	-1.08E-08	-1.04E-08	-1.07E-08	-1.08E-08	-9.00E-09	-9.34E-09	-8.34E-09
-1.31E-08	-1.22E-08	-1.19E-08	-1.13E-08	-1.13E-08	-1.04E-08	-1.07E-08	-1.02E-08	-1.09E-08	-1.02E-08	-9.83E-09	-9.46E-09	-8.87E-09
-1.28E-08	-1.19E-08	-1.18E-08	-1.16E-08	-1.16E-08	-1.13E-08	-1.07E-08	-1.04E-08	-1.10E-08	-1.01E-08	-1.02E-08	-1.00E-08	-9.36E-09
-1.24E-08	-1.16E-08	-1.16E-08	-1.20E-08	-1.19E-08	-1.13E-08	-1.07E-08	-1.01E-08	-1.07E-08	-1.02E-08	-9.70E-09	-1.04E-08	-9.80E-09
-1.18E-08	-1.11E-08	-1.12E-08	-1.17E-08	-1.18E-08	-1.10E-08	-1.03E-08	-1.07E-08	-1.06E-08	-1.00E-08	-9.67E-09	-1.05E-08	-9.91E-09
-1.14E-08	-1.08E-08	-1.07E-08	-1.12E-08	-1.12E-08	-1.06E-08	-1.03E-08	-1.05E-08	-1.02E-08	-9.98E-09	-9.99E-09	-1.04E-08	-9.89E-09
-1.12E-08	-1.09E-08	-1.09E-08	-1.09E-08	-1.08E-08	-9.87E-09	-9.33E-09	-1.00E-08	-1.01E-08	-1.02E-08	-1.02E-08	-1.00E-08	-9.51E-09
-1.09E-08	-1.07E-08	-1.09E-08	-1.07E-08	-1.04E-08	-9.54E-09	-8.78E-09	-9.93E-09	-1.00E-08	-1.04E-08	-1.00E-08	-9.38E-09	-8.73E-09
-1.05E-08	-1.00E-08	-1.04E-08	-1.01E-08	-9.72E-09	-9.29E-09	-9.07E-09	-1.00E-08	-1.00E-08	-1.02E-08	-9.29E-09	-8.62E-09	-8.25E-09
-1.03E-08	-1.00E-08	-9.43E-09	-9.33E-09	-9.36E-09	-9.18E-09	-8.94E-09	-1.00E-08	-9.90E-09	-9.73E-09	-8.54E-09	-8.11E-09	-8.23E-09
-1.01E-08	-9.49E-09	-8.84E-09	-9.19E-09	-8.99E-09	-9.24E-09	-8.79E-09	-1.00E-08	-9.85E-09	-9.33E-09	-8.10E-09	-7.88E-09	-8.58E-09
-1.00E-08	-9.22E-09	-8.49E-09	-9.49E-09	-8.74E-09	-9.19E-09	-8.77E-09	-1.00E-08	-9.85E-09	-9.68E-09	-8.13E-09	-7.66E-09	-8.42E-09
-1.01E-08	-9.25E-09	-8.44E-09	-9.07E-09	-8.52E-09	-8.71E-09	-9.08E-09	-9.48E-09	-9.25E-09	-9.65E-09	-8.10E-09	-7.47E-09	-8.34E-09
-1.01E-08	-9.39E-09	-8.41E-09	-9.20E-09	-8.93E-09	-8.37E-09	-8.93E-09	-8.99E-09	-8.78E-09	-9.49E-09	-8.08E-09	-7.59E-09	-8.43E-09
-9.84E-09	-9.40E-09	-8.87E-09	-9.67E-09	-9.17E-09	-8.55E-09	-8.97E-09	-8.87E-09	-8.72E-09	-9.25E-09	-8.03E-09	-7.81E-09	-7.86E-09
-9.45E-09	-9.31E-09	-9.44E-09	-9.87E-09	-9.15E-09	-8.81E-09	-9.12E-09	-8.78E-09	-8.83E-09	-9.12E-09	-8.18E-09	-7.69E-09	-7.49E-09
-9.07E-09	-9.37E-09	-9.49E-09	-1.01E-08	-9.22E-09	-9.29E-09	-9.09E-09	-8.90E-09	-8.68E-09	-9.00E-09	-8.20E-09	-7.37E-09	-7.31E-09
-8.43E-09	-8.87E-09	-9.05E-09	-9.93E-09	-9.28E-09	-9.45E-09	-8.96E-09	-8.79E-09	-8.65E-09	-9.09E-09	-8.07E-09	-7.31E-09	-7.21E-09
-7.71E-09	-8.34E-09	-8.16E-09	-9.05E-09	-8.97E-09	-9.20E-09	-8.59E-09	-8.44E-09	-8.56E-09	-8.78E-09	-8.21E-09	-7.75E-09	-7.50E-09
-7.70E-09	-8.20E-09	-7.51E-09	-8.20E-09	-8.19E-09	-8.65E-09	-8.31E-09	-8.12E-09	-7.88E-09	-8.35E-09	-7.71E-09	-7.90E-09	-7.89E-09
-7.47E-09	-8.19E-09	-7.45E-09	-8.01E-09	-8.03E-09	-8.28E-09	-8.23E-09	-7.84E-09	-7.36E-09	-8.00E-09	-7.29E-09	-7.81E-09	-8.08E-09
-7.30E-09	-7.92E-09	-7.49E-09	-7.99E-09	-8.46E-09	-8.40E-09	-8.30E-09	-7.69E-09	-7.12E-09	-7.65E-09	-7.36E-09	-7.74E-09	-7.85E-09
-7.03E-09	-7.73E-09	-7.79E-09	-7.94E-09	-8.29E-09	-8.36E-09	-8.30E-09	-7.82E-09	-7.21E-09	-7.40E-09	-7.23E-09	-7.58E-09	-7.54E-09
-6.41E-09	-7.42E-09	-7.70E-09	-7.77E-09	-8.27E-09	-7.98E-09	-7.81E-09	-7.49E-09	-7.49E-09	-7.41E-09	-7.44E-09	-7.45E-09	-7.16E-09
-6.08E-09	-7.45E-09	-7.53E-09	-7.37E-09	-8.05E-09	-7.83E-09	-7.56E-09	-7.19E-09	-7.25E-09	-7.12E-09	-7.69E-09	-7.68E-09	-7.30E-09
-6.32E-09	-6.94E-09	-6.97E-09	-6.89E-09	-7.55E-09	-7.44E-09	-7.57E-09	-7.37E-09	-7.29E-09	-7.28E-09	-7.68E-09	-7.83E-09	-7.68E-09
-6.21E-09	-6.19E-09	-6.57E-09	-6.52E-09	-6.41E-09	-6.66E-09	-7.65E-09	-7.32E-09	-7.18E-09	-7.56E-09	-8.01E-09	-7.93E-09	-7.53E-09
-5.71E-09	-6.33E-09	-6.75E-09	-6.54E-09	-5.73E-09	-6.38E-09	-7.69E-09	-7.11E-09	-7.03E-09	-7.72E-09	-7.91E-09	-7.58E-09	-7.45E-09
-6.42E-09	-6.89E-09	-7.23E-09	-6.65E-09	-6.36E-09	-6.53E-09	-7.49E-09	-6.78E-09	-6.71E-09	-7.59E-09	-7.31E-09	-7.23E-09	-6.91E-09
-6.36E-09	-6.84E-09	-7.13E-09	-6.58E-09	-6.31E-09	-6.40E-09	-7.14E-09	-7.01E-09	-6.21E-09	-7.11E-09	-6.96E-09	-6.87E-09	-6.51E-09
-5.76E-09	-6.63E-09	-6.75E-09	-6.57E-09	-6.25E-09	-6.69E-09	-7.14E-09	-7.02E-09	-6.07E-09	-6.85E-09	-7.01E-09	-6.84E-09	-6.56E-09
-5.43E-09	-6.33E-09	-6.45E-09	-6.53E-09	-6.40E-09	-7.13E-09	-7.36E-09	-7.12E-09	-6.67E-09	-6.89E-09	-6.90E-09	-6.74E-09	-7.03E-09
-5.69E-09	-6.63E-09	-6.53E-09	-6.50E-09	-6.55E-09	-7.61E-09	-7.72E-09	-7.39E-09	-7.29E-09	-7.08E-09	-6.66E-09	-6.57E-09	-7.16E-09
-6.07E-09	-6.59E-09	-6.64E-09	-6.08E-09	-6.63E-09	-7.64E-09	-7.35E-09	-7.64E-09	-7.20E-09	-7.45E-09	-6.24E-09	-6.39E-09	-7.20E-09
-6.11E-09	-6.32E-09	-6.54E-09	-6.04E-09	-6.35E-09	-7.34E-09	-7.12E-09	-7.71E-09	-7.23E-09	-7.59E-09	-6.66E-09	-6.58E-09	-7.13E-09
-6.21E-09	-6.28E-09	-6.37E-09	-6.27E-09	-6.07E-09	-6.94E-09	-6.67E-09	-7.35E-09	-6.95E-09	-7.43E-09	-7.10E-09	-6.61E-09	-6.97E-09
-6.24E-09	-5.73E-09	-6.05E-09	-6.78E-09	-6.53E-09	-6.83E-09	-6.57E-09	-7.09E-09	-6.65E-09	-7.09E-09	-6.93E-09	-6.08E-09	-6.86E-09
-6.05E-09	-5.64E-09	-6.12E-09	-6.93E-09	-6.94E-09	-6.59E-09	-6.54E-09	-6.86E-09	-6.10E-09	-6.63E-09	-7.09E-09	-6.34E-09	-6.91E-09
-5.66E-09	-5.62E-09	-6.68E-09	-6.74E-09	-6.69E-09	-6.42E-09	-6.22E-09	-6.63E-09	-5.99E-09	-6.08E-09	-7.25E-09	-6.60E-09	-6.79E-09
-5.05E-09	-6.04E-09	-7.23E-09	-6.53E-09	-6.34E-09	-6.20E-09	-5.88E-09	-6.49E-09	-6.09E-09	-4.56E-09	-6.92E-09	-7.25E-09	-6.76E-09
-5.28E-09	-6.51E-09	-7.27E-09	-6.08E-09	-5.71E-09	-6.08E-09	-5.45E-09	-7.02E-09	-5.72E-09	-1.93E-09	-6.73E-09	-7.55E-09	-6.57E-09
-5.37E-09	-6.95E-09	-7.38E-09	-6.33E-09	-5.84E-09	-5.82E-09	-5.47E-09	-6.79E-09	-4.79E-09	1.02E-09	-6.86E-09	-7.77E-09	-7.07E-09
-6.18E-09	-7.19E-09	-7.29E-09	-6.87E-09	-6.71E-09	-6.06E-09	-5.37E-09	-6.22E-09	-6.81E-09	-4.80E-09	-6.39E-09	-7.48E-09	-7.37E-09
-6.61E-09	-7.42E-09	-7.23E-09	-7.66E-09	-7.61E-09	-6.53E-09	-5.98E-09	-5.79E-09	-7.17E-09	-7.44E-09	-6.20E-09	-7.36E-09	-7.86E-09
-7.20E-09	-7.61E-09	-7.48E-09	-8.05E-09	-7.80E-09	-6.76E-09	-6.42E-09	-6.50E-09	-7.55E-09	-7.02E-09	-6.53E-09	-7.03E-09	-7.95E-09
-7.91E-09	-7.68E-09	-7.92E-09	-8.06E-09	-7.56E-09	-6.71E-09	-7.38E-09	-7.10E-09	-7.82E-09	-6.95E-09	-6.81E-09	-7.12E-09	-7.95E-09
-8.59E-09	-8.24E-09	-8.12E-09	-8.00E-09	-7.42E-09	-7.22E-09	-7.63E-09	-7.94E-09	-8.38E-09	-7.41E-09	-6.78E-09	-7.35E-09	-7.81E-09
-8.83E-09	-8.67E-09	-8.46E-09	-8.19E-09	-7.87E-09	-7.74E-09	-7.77E-09	-8.55E-09	-8.93E-09	-7.71E-09	-7.33E-09	-7.53E-09	-7.63E-09
-8.79E-09	-9.08E-09	-8.87E-09	-8.51E-09	-8.31E-09	-8.32E-09	-7.88E-09	-8.50E-09	-8.55E-09	-7.51E-09	-7.58E-09	-7.66E-09	-7.64E-09
-8.90E-09	-9.34E-09	-9.02E-09	-9.04E-09	-7.97E-09	-8.53E-09	-7.93E-09	-8.45E-09	-8.46E-09	-7.47E-09	-7.21E-09	-7.51E-09	-7.64E-09
-8.69E-09	-9.00E-09	-9.10E-09	-8.95E-09	-7.44E-09	-8.49E-09	-7.92E-09	-8.38E-09	-8.10E-09	-7.69E-09	-6.94E-09	-7.71E-09	-7.69E-09
-7.86E-09	-8.26E-09	-8.86E-09	-8.42E-09	-7.23E-09	-8.23E-09	-7.85E-09	-7.81E-09	-7.45E-09	-7.54E-09	-7.06E-09	-7.84E-09	-7.65E-09
-7.59E-09	-8.01E-09	-8.17E-09	-7.85E-09	-7.52E-09	-7.93E-09	-7.89E-09	-7.42E-09	-7.15E-09	-6.97E-09	-7.54E-09	-7.73E-09	-7.70E-09
-8.10E-09	-8.14E-09	-7.57E-09	-7.27E-09	-7.22E-09	-7.93E-09	-7.29E-09	-7.40E-09	-7.14E-09	-6.60E-09	-6.98E-09	-7.50E-09	-8.11E-09
-8.50E-09	-8.06E-09	-7.13E-09	-6.99E-09	-7.23E-09	-7.61E-09	-6.80E-09	-7.36E-09	-6.85E-09	-6.08E-09	-6.24E-09	-6.52E-09	-7.81E-09
-8.08E-09	-7.79E-09	-6.66E-09	-7.03E-09	-6.64E-09	-6.49E-09	-6.29E-09	-6.84E-09	-6.02E-09	-5.49E-09	-5.97E-09	-5.28E-09	-6.89E-09
-7.32E-09	-7.42E-09	-6.24E-09	-6.57E-09	-6.07E-09	-5.49E-09	-5.76E-09	-6.43E-09	-4.86E-09	-4.99E-09	-5.53E-09	-4.47E-09	-6.23E-09
-6.74E-09	-6.70E-09	-5.30E-09	-5.65E-09	-5.60E-09	-4.86E-09	-5.53E-09	-5.99E-09	-4.56E-09	-4.18E-09	-4.61E-09	-4.19E-09	-5.91E-09
-6.06E-09	-6.14E-09	-4.54E-09	-5.32E-09	-4.97E-09	-5.07E-09	-5.17E-09	-5.31E-09	-4.28E-09	-4.22E-09	-4.23E-09	-4.62E-09	-5.67E-09
-5.42E-09	-5.58E-09	-4.46E-09	-5.39E-09	-4.87E-09	-4.66E-09	-4.45E-09	-4.94E-09	-3.56E-09	-4.31E-09	-4.45E-09	-4.43E-09	-5.30E-09
-4.84E-09	-4.75E-09	-4.02E-09	-5.14E-09	-4.37E-09	-4.14E-09	-3.90E-09	-4.71E-09	-3.15E-09	-3.69E-09	-4.02E-09	-3.81E-09	-4.76E-09
-4.20E-09	-3.99E-09	-3.47E-09	-4.48E-09	-4.00E-09	-3.73E-09	-4.13E-09	-4.46E-09	-3.27E-09	-3.45E-09	-3.15E-09	-3.35E-09	-3.79E-09
-3.64E-09	-3.49E-09	-3.26E-09	-3.52E-09	-3.11E-09	-3.37E-09	-4.33E-09	-4.08E-09	-2.96E-09	-3.17E-09	-2.98E-09	-3.02E-09	-3.57E-09
-3.11E-09	-2.95E-09	-2.97E-09	-3.04E-09	-2.51E-09	-3.07E-09	-4.22E-09	-3.73E-09	-2.47E-09	-2.76E-09	-2.82E-09	-3.01E-09	-3.82E-09
-2.49E-09	-2.20E-09	-2.49E-09	-2.54E-09	-2.25E-09	-2.81E-09	-3.67E-09	-3.66E-09	-2.24E-09	-2.23E-09	-2.72E-09	-2.71E-09	-3.55E-09
-1.86E-09	-1.92E-09	-2.36E-09	-2.00E-09	-1.65E-09	-2.63E-09	-3.12E-09	-3.37E-09	-2.22E-09	-1.90E-09	-2.64E-09	-2.23E-09	-3.12E-09
-1.72E-09	-1.81E-09	-1.96E-09	-1.71E-09	-1.22E-09	-2.18E-09	-2.48E-09	-2.89E-09	-2.01E-09	-1.94E-09	-2.76E-09	-2.34E-09	-2.91E-09
-2.04E-09	-1.78E-09	-1.61E-09	-1.39E-09	-1.10E-09	-1.92E-09	-2.03E-09	-2.10E-09	-1.33E-09	-1.13E-09	-2.68E-09	-2.54E-09	-2.71E-09
-1.89E-09	-1.78E-09	-1.26E-09	-6.93E-10	-1.10E-09	-1.45E-09	-1.68E-09	-1.28E-09	-9.37E-10	-8.81E-10	-1.98E-09	-2.35E-09	-2.26E-09
-1.48E-09	-1.58E-09	-8.43E-10	-7.96E-12	-8.69E-10	-1.27E-09	-1.80E-09	-8.85E-10	-1.44E-09	-1.49E-09	-1.77E-09	-1.78E-09	-1.52E-09
-1.43E-09	-9.50E-10	-2.99E-10	5.97E-10	-5.66E-10	-7.31E-10	-1.23E-09	-5.75E-10	-1.30E-09	-1.47E-09	-1.56E-09	-1.34E-09	-1.04E-09
-8.50E-10	-5.64E-10	1.79E-10	8.22E-10	4.55E-12	-1.88E-10	-7.84E-10	-8.71E-10	-9.61E-10	-7.55E-10	-7.72E-10	-6.42E-10	-1.52E-10
-5.93E-10	-1.59E-10	2.51E-10	9.24E-10	2.16E-10	6.69E-11	-2.12E-10	-6.34E-10	-6.03E-10	-1.16E-10	6.55E-11	1.38E-11	7.53E-10
-1.79E-10	3.42E-10	1.23E-10	7.59E-10	3.41E-11	3.43E-10	5.82E-11	4.71E-11	-1.74E-10	5.21E-10	3.19E-10	8.22E-10	9.53E-10
2.88E-10	8.24E-10	6.99E-10	6.85E-10	2.75E-10	9.58E-10	3.11E-10	7.22E-10	6.94E-10	7.03E-10	6.18E-10	8.86E-10	8.16E-10

8.11E-10	1.53E-09	1.23E-09	1.17E-09	8.72E-10	1.52E-09	7.53E-10	1.64E-09	9.59E-10	1.03E-09	8.79E-10	4.40E-10	7.97E-10
1.00E-09	2.27E-09	1.60E-09	1.35E-09	1.04E-09	1.42E-09	1.21E-09	2.33E-09	7.97E-10	9.09E-10	8.04E-10	4.16E-10	1.14E-09
1.38E-09	2.56E-09	1.94E-09	1.90E-09	1.01E-09	1.23E-09	1.23E-09	2.35E-09	8.36E-10	9.36E-10	1.10E-09	6.02E-10	1.53E-09
1.86E-09	2.65E-09	1.84E-09	1.79E-09	1.43E-09	1.41E-09	1.49E-09	2.18E-09	1.13E-09	1.46E-09	1.42E-09	1.34E-09	2.29E-09
2.69E-09	2.83E-09	2.19E-09	1.87E-09	1.72E-09	1.70E-09	1.97E-09	2.37E-09	1.57E-09	1.65E-09	1.80E-09	1.40E-09	2.67E-09
3.70E-09	3.18E-09	2.80E-09	2.79E-09	2.23E-09	2.09E-09	2.52E-09	2.75E-09	2.22E-09	2.09E-09	2.41E-09	1.51E-09	2.09E-09
3.79E-09	3.43E-09	3.45E-09	3.47E-09	2.78E-09	2.37E-09	3.26E-09	2.96E-09	2.47E-09	2.44E-09	3.05E-09	2.00E-09	2.34E-09
3.70E-09	3.65E-09	4.13E-09	3.89E-09	2.98E-09	2.73E-09	3.54E-09	3.38E-09	3.00E-09	2.97E-09	3.90E-09	2.96E-09	2.82E-09
4.06E-09	3.90E-09	4.50E-09	4.82E-09	3.72E-09	3.78E-09	4.55E-09	4.21E-09	3.61E-09	3.47E-09	4.99E-09	4.03E-09	3.40E-09
4.69E-09	4.50E-09	4.56E-09	5.20E-09	4.75E-09	4.65E-09	4.82E-09	5.03E-09	4.02E-09	3.87E-09	5.30E-09	5.03E-09	3.87E-09
5.06E-09	5.41E-09	4.87E-09	4.95E-09	5.78E-09	4.80E-09	4.98E-09	5.20E-09	4.16E-09	4.23E-09	5.32E-09	5.31E-09	4.66E-09
5.12E-09	5.94E-09	5.58E-09	4.83E-09	5.55E-09	5.29E-09	5.33E-09	5.22E-09	4.76E-09	5.02E-09	5.38E-09	5.20E-09	5.12E-09
5.88E-09	6.55E-09	6.44E-09	5.54E-09	5.69E-09	6.16E-09	5.55E-09	5.33E-09	5.54E-09	5.43E-09	5.52E-09	5.11E-09	5.36E-09
6.61E-09	6.63E-09	6.80E-09	6.10E-09	6.10E-09	6.50E-09	5.85E-09	5.75E-09	6.14E-09	5.68E-09	5.84E-09	5.26E-09	5.35E-09
7.86E-09	7.01E-09	7.06E-09	6.76E-09	6.67E-09	6.72E-09	6.31E-09	6.21E-09	6.48E-09	5.74E-09	6.10E-09	5.85E-09	6.46E-09
8.18E-09	7.61E-09	7.59E-09	7.47E-09	7.22E-09	6.86E-09	6.82E-09	6.78E-09	7.00E-09	6.58E-09	6.73E-09	6.96E-09	8.14E-09
8.12E-09	8.24E-09	8.04E-09	8.37E-09	7.60E-09	7.26E-09	7.64E-09	7.63E-09	7.46E-09	7.32E-09	7.46E-09	7.98E-09	9.06E-09
8.41E-09	9.05E-09	8.37E-09	8.50E-09	8.08E-09	7.66E-09	8.23E-09	7.92E-09	7.85E-09	7.86E-09	8.28E-09	8.43E-09	7.75E-09
8.85E-09	9.34E-09	8.80E-09	8.67E-09	8.84E-09	8.19E-09	8.66E-09	8.01E-09	8.29E-09	8.34E-09	9.09E-09	8.96E-09	8.06E-09
9.80E-09	9.57E-09	9.57E-09	9.64E-09	9.62E-09	9.06E-09	9.14E-09	8.53E-09	9.11E-09	9.19E-09	9.52E-09	9.23E-09	8.68E-09
1.08E-08	1.03E-08	1.06E-08	1.07E-08	1.04E-08	9.82E-09	9.98E-09	9.37E-09	1.02E-08	9.77E-09	9.98E-09	9.59E-09	8.98E-09
1.18E-08	1.12E-08	1.14E-08	1.14E-08	1.12E-08	1.10E-08	1.07E-08	1.03E-08	1.06E-08	1.03E-08	1.02E-08	9.80E-09	9.08E-09
1.27E-08	1.17E-08	1.19E-08	1.16E-08	1.17E-08	1.17E-08	1.14E-08	1.12E-08	1.10E-08	1.10E-08	1.05E-08	1.04E-08	1.02E-08
1.34E-08	1.23E-08	1.27E-08	1.21E-08	1.22E-08	1.25E-08	1.18E-08	1.17E-08	1.20E-08	1.15E-08	1.14E-08	1.13E-08	1.13E-08
1.40E-08	1.31E-08	1.34E-08	1.31E-08	1.25E-08	1.30E-08	1.21E-08	1.21E-08	1.28E-08	1.22E-08	1.20E-08	1.24E-08	1.20E-08
1.44E-08	1.37E-08	1.43E-08	1.42E-08	1.35E-08	1.39E-08	1.26E-08	1.32E-08	1.33E-08	1.29E-08	1.28E-08	1.31E-08	1.27E-08
1.48E-08	1.41E-08	1.52E-08	1.54E-08	1.48E-08	1.49E-08	1.36E-08	1.43E-08	1.40E-08	1.32E-08	1.37E-08	1.42E-08	1.37E-08
1.54E-08	1.53E-08	1.61E-08	1.62E-08	1.58E-08	1.58E-08	1.47E-08	1.52E-08	1.49E-08	1.40E-08	1.45E-08	1.52E-08	1.44E-08
1.63E-08	1.64E-08	1.66E-08	1.68E-08	1.64E-08	1.70E-08	1.57E-08	1.62E-08	1.58E-08	1.47E-08	1.44E-08	1.53E-08	1.49E-08
1.68E-08	1.72E-08	1.73E-08	1.78E-08	1.75E-08	1.78E-08	1.66E-08	1.70E-08	1.65E-08	1.56E-08	1.55E-08	1.58E-08	1.55E-08
1.72E-08	1.77E-08	1.83E-08	1.88E-08	1.82E-08	1.78E-08	1.72E-08	1.70E-08	1.69E-08	1.64E-08	1.65E-08	1.69E-08	1.65E-08
1.75E-08	1.80E-08	1.89E-08	1.93E-08	1.90E-08	1.88E-08	1.77E-08	1.76E-08	1.74E-08	1.69E-08	1.71E-08	1.76E-08	1.72E-08
1.83E-08	1.91E-08	1.99E-08	2.00E-08	1.96E-08	1.97E-08	1.85E-08	1.82E-08	1.81E-08	1.76E-08	1.76E-08	1.79E-08	1.74E-08
1.96E-08	2.04E-08	2.10E-08	2.02E-08	2.00E-08	2.01E-08	1.94E-08	1.94E-08	1.87E-08	1.84E-08	1.79E-08	1.78E-08	1.77E-08
2.05E-08	2.08E-08	2.10E-08	2.07E-08	2.06E-08	2.05E-08	2.05E-08	2.05E-08	1.95E-08	1.92E-08	1.86E-08	1.81E-08	1.86E-08
2.11E-08	2.18E-08	2.15E-08	2.14E-08	2.12E-08	2.13E-08	2.15E-08	2.15E-08	2.01E-08	1.98E-08	1.90E-08	1.84E-08	1.94E-08
2.20E-08	2.25E-08	2.22E-08	2.21E-08	2.21E-08	2.24E-08	2.24E-08	2.21E-08	2.10E-08	2.03E-08	1.96E-08	1.91E-08	2.06E-08
2.30E-08	2.31E-08	2.27E-08	2.32E-08	2.33E-08	2.32E-08	2.31E-08	2.24E-08	2.18E-08	2.13E-08	2.04E-08	2.02E-08	2.11E-08
2.36E-08	2.34E-08	2.33E-08	2.40E-08	2.35E-08	2.36E-08	2.34E-08	2.28E-08	2.26E-08	2.23E-08	2.12E-08	2.14E-08	2.17E-08
2.40E-08	2.39E-08	2.34E-08	2.42E-08	2.38E-08	2.38E-08	2.36E-08	2.35E-08	2.34E-08	2.30E-08	2.21E-08	2.22E-08	2.19E-08
2.44E-08	2.41E-08	2.40E-08	2.44E-08	2.43E-08	2.42E-08	2.43E-08	2.40E-08	2.38E-08	2.38E-08	2.32E-08	2.32E-08	2.31E-08
2.46E-08	2.47E-08	2.45E-08	2.52E-08	2.49E-08	2.48E-08	2.51E-08	2.45E-08	2.45E-08	2.45E-08	2.39E-08	2.42E-08	2.43E-08
2.56E-08	2.51E-08	2.52E-08	2.53E-08	2.57E-08	2.55E-08	2.58E-08	2.55E-08	2.55E-08	2.54E-08	2.46E-08	2.46E-08	2.50E-08
2.65E-08	2.62E-08	2.62E-08	2.60E-08	2.63E-08	2.61E-08	2.67E-08	2.65E-08	2.64E-08	2.63E-08	2.55E-08	2.52E-08	2.54E-08
2.72E-08	2.77E-08	2.73E-08	2.68E-08	2.65E-08	2.66E-08	2.76E-08	2.76E-08	2.72E-08	2.70E-08	2.65E-08	2.62E-08	2.60E-08
2.87E-08	2.86E-08	2.80E-08	2.75E-08	2.70E-08	2.75E-08	2.85E-08	2.87E-08	2.82E-08	2.77E-08	2.73E-08	2.72E-08	2.69E-08
3.04E-08	2.92E-08	2.83E-08	2.82E-08	2.79E-08	2.86E-08	2.99E-08	3.03E-08	2.89E-08	2.80E-08	2.82E-08	2.82E-08	2.78E-08
3.16E-08	3.01E-08	2.93E-08	2.93E-08	2.91E-08	2.96E-08	3.13E-08	3.11E-08	2.97E-08	2.88E-08	2.90E-08	2.88E-08	2.84E-08
3.22E-08	3.11E-08	3.05E-08	3.04E-08	3.02E-08	3.06E-08	3.21E-08	3.14E-08	3.04E-08	3.01E-08	2.99E-08	2.94E-08	2.92E-08
3.34E-08	3.24E-08	3.18E-08	3.14E-08	3.15E-08	3.17E-08	3.30E-08	3.19E-08	3.07E-08	3.11E-08	3.09E-08	3.05E-08	3.04E-08

1.73E-16	1.54E-16	1.55E-16	1.35E-16	1.18E-16	8.43E-17	1.20E-16	1.30E-16	1.33E-16	1.07E-16	1.09E-16	1.35E-16	1.20E-16
1.62E-16	1.38E-16	1.44E-16	1.26E-16	1.16E-16	8.44E-17	1.12E-16	1.18E-16	1.25E-16	1.06E-16	9.75E-17	1.18E-16	1.01E-16
1.61E-16	1.42E-16	1.49E-16	1.24E-16	1.17E-16	9.63E-17	1.13E-16	1.11E-16	1.12E-16	1.11E-16	8.22E-17	9.99E-17	7.76E-17
1.69E-16	1.50E-16	1.49E-16	1.28E-16	1.23E-16	9.65E-17	1.17E-16	1.08E-16	1.15E-16	1.16E-16	8.10E-17	8.72E-17	6.96E-17
1.72E-16	1.50E-16	1.41E-16	1.27E-16	1.27E-16	1.09E-16	1.15E-16	1.05E-16	1.18E-16	1.05E-16	9.66E-17	8.95E-17	7.87E-17
1.64E-16	1.42E-16	1.40E-16	1.35E-16	1.35E-16	1.28E-16	1.15E-16	1.09E-16	1.20E-16	1.03E-16	1.03E-16	1.00E-16	8.76E-17
1.54E-16	1.35E-16	1.35E-16	1.43E-16	1.43E-16	1.27E-16	1.14E-16	1.03E-16	1.15E-16	1.04E-16	9.41E-17	1.08E-16	9.60E-17
1.40E-16	1.23E-16	1.26E-16	1.36E-16	1.39E-16	1.21E-16	1.07E-16	1.13E-16	1.12E-16	1.00E-16	9.34E-17	1.09E-16	9.83E-17
1.31E-16	1.16E-16	1.14E-16	1.26E-16	1.26E-16	1.12E-16	1.07E-16	1.10E-16	1.05E-16	9.96E-17	9.98E-17	1.08E-16	9.79E-17
1.25E-16	1.18E-16	1.19E-16	1.19E-16	1.17E-16	9.75E-17	8.71E-17	1.01E-16	1.02E-16	1.04E-16	1.04E-16	1.00E-16	9.03E-17
1.18E-16	1.13E-16	1.18E-16	1.15E-16	1.09E-16	9.09E-17	7.72E-17	9.86E-17	1.00E-16	1.08E-16	1.00E-16	8.80E-17	7.62E-17
1.09E-16	1.00E-16	1.08E-16	1.02E-16	9.45E-17	8.62E-17	8.22E-17	1.00E-16	1.01E-16	1.04E-16	8.63E-17	7.43E-17	6.80E-17
1.07E-16	1.00E-16	8.89E-17	8.70E-17	8.76E-17	8.42E-17	7.99E-17	9.99E-17	9.81E-17	9.46E-17	7.29E-17	6.57E-17	6.77E-17
1.03E-16	9.01E-17	7.81E-17	8.45E-17	8.08E-17	8.55E-17	7.72E-17	1.00E-16	9.71E-17	8.71E-17	6.56E-17	6.21E-17	7.37E-17
1.00E-16	8.50E-17	7.20E-17	9.01E-17	7.65E-17	8.45E-17	7.69E-17	1.00E-16	9.71E-17	9.37E-17	6.60E-17	5.87E-17	7.09E-17
1.03E-16	8.55E-17	7.12E-17	8.23E-17	7.25E-17	7.59E-17	8.25E-17	8.99E-17	8.56E-17	9.31E-17	6.56E-17	5.58E-17	6.96E-17
1.02E-16	8.83E-17	7.07E-17	8.46E-17	7.97E-17	7.01E-17	7.98E-17	8.09E-17	7.72E-17	9.01E-17	6.53E-17	5.76E-17	7.11E-17
9.69E-17	8.83E-17	7.87E-17	9.34E-17	8.40E-17	7.30E-17	8.04E-17	7.86E-17	7.60E-17	8.55E-17	6.45E-17	6.10E-17	6.17E-17
8.93E-17	8.67E-17	8.91E-17	9.75E-17	8.37E-17	7.76E-17	8.31E-17	7.70E-17	7.79E-17	8.32E-17	6.70E-17	5.92E-17	5.62E-17
8.22E-17	8.78E-17	9.00E-17	1.01E-16	8.50E-17	8.62E-17	8.26E-17	7.92E-17	7.53E-17	8.10E-17	6.72E-17	5.43E-17	5.34E-17
7.11E-17	7.86E-17	8.20E-17	9.85E-17	8.61E-17	8.93E-17	8.02E-17	7.73E-17	7.49E-17	8.26E-17	6.51E-17	5.34E-17	5.20E-17
5.95E-17	6.96E-17	6.65E-17	8.19E-17	8.05E-17	8.46E-17	7.39E-17	7.12E-17	7.33E-17	7.72E-17	6.75E-17	6.01E-17	5.63E-17
5.92E-17	6.72E-17	5.65E-17	6.72E-17	6.71E-17	7.49E-17	6.91E-17	6.60E-17	6.20E-17	6.98E-17	5.94E-17	6.24E-17	6.22E-17
5.59E-17	6.71E-17	5.55E-17	6.41E-17	6.45E-17	6.86E-17	6.77E-17	6.15E-17	5.42E-17	6.40E-17	5.31E-17	6.10E-17	6.53E-17
5.34E-17	6.27E-17	5.61E-17	6.39E-17	7.15E-17	7.06E-17	6.89E-17	5.91E-17	5.08E-17	5.86E-17	5.41E-17	5.99E-17	6.16E-17
4.95E-17	5.97E-17	6.06E-17	6.30E-17	6.87E-17	6.99E-17	6.89E-17	6.11E-17	5.21E-17	5.47E-17	5.23E-17	5.75E-17	5.68E-17
4.10E-17	5.51E-17	5.94E-17	6.04E-17	6.83E-17	6.37E-17	6.10E-17	5.61E-17	5.61E-17	5.49E-17	5.53E-17	5.55E-17	5.13E-17
3.69E-17	5.55E-17	5.67E-17	5.44E-17	6.49E-17	6.13E-17	5.72E-17	5.17E-17	5.26E-17	5.07E-17	5.92E-17	5.90E-17	5.33E-17
3.99E-17	4.81E-17	4.86E-17	4.75E-17	5.70E-17	5.53E-17	5.74E-17	5.43E-17	5.31E-17	5.30E-17	5.90E-17	6.14E-17	5.90E-17
3.85E-17	3.83E-17	4.32E-17	4.26E-17	4.11E-17	4.44E-17	5.85E-17	5.36E-17	5.16E-17	5.72E-17	6.41E-17	6.29E-17	5.67E-17
3.25E-17	4.01E-17	4.55E-17	4.28E-17	3.28E-17	4.07E-17	5.91E-17	5.05E-17	4.94E-17	5.97E-17	6.26E-17	5.75E-17	5.55E-17

4.12E-17	4.74E-17	5.23E-17	4.42E-17	4.05E-17	4.26E-17	5.61E-17	4.60E-17	4.51E-17	5.76E-17	5.34E-17	5.22E-17	4.78E-17
4.04E-17	4.68E-17	5.08E-17	4.34E-17	3.98E-17	4.10E-17	5.10E-17	4.92E-17	3.85E-17	5.06E-17	4.85E-17	4.72E-17	4.24E-17
3.32E-17	4.40E-17	4.55E-17	4.32E-17	3.91E-17	4.47E-17	5.10E-17	4.93E-17	3.69E-17	4.70E-17	4.91E-17	4.67E-17	4.30E-17
2.95E-17	4.00E-17	4.16E-17	4.26E-17	4.09E-17	5.08E-17	5.42E-17	5.07E-17	4.45E-17	4.75E-17	4.76E-17	4.54E-17	4.95E-17
3.23E-17	4.40E-17	4.26E-17	4.23E-17	4.29E-17	5.79E-17	5.96E-17	5.46E-17	5.31E-17	5.01E-17	4.43E-17	4.32E-17	5.12E-17
3.69E-17	4.34E-17	4.41E-17	3.69E-17	4.40E-17	5.84E-17	5.40E-17	5.83E-17	5.18E-17	5.56E-17	3.90E-17	4.08E-17	5.18E-17
3.73E-17	3.99E-17	4.27E-17	3.64E-17	4.03E-17	5.39E-17	5.06E-17	5.94E-17	5.22E-17	5.76E-17	4.43E-17	4.33E-17	5.09E-17
3.86E-17	3.95E-17	4.05E-17	3.93E-17	3.68E-17	4.82E-17	4.45E-17	5.41E-17	4.82E-17	5.53E-17	5.04E-17	4.37E-17	4.86E-17
3.89E-17	3.29E-17	3.66E-17	4.59E-17	4.27E-17	4.66E-17	4.32E-17	5.02E-17	4.42E-17	5.03E-17	4.80E-17	3.69E-17	4.71E-17
3.66E-17	3.18E-17	3.75E-17	4.80E-17	4.82E-17	4.34E-17	4.28E-17	4.71E-17	3.72E-17	4.40E-17	5.02E-17	4.02E-17	4.78E-17
3.20E-17	3.15E-17	4.46E-17	4.54E-17	4.47E-17	4.12E-17	3.87E-17	4.39E-17	3.59E-17	3.69E-17	5.26E-17	4.35E-17	4.60E-17
2.56E-17	3.65E-17	5.23E-17	4.27E-17	4.01E-17	3.85E-17	3.45E-17	4.21E-17	3.71E-17	2.08E-17	4.78E-17	5.25E-17	4.57E-17
2.78E-17	4.24E-17	5.28E-17	3.69E-17	3.26E-17	3.69E-17	2.97E-17	4.93E-17	3.27E-17	3.71E-18	4.53E-17	5.70E-17	4.32E-17
2.88E-17	4.83E-17	5.45E-17	4.01E-17	3.41E-17	3.38E-17	2.99E-17	4.61E-17	2.29E-17	1.05E-18	4.71E-17	6.03E-17	5.00E-17
3.81E-17	5.17E-17	5.32E-17	4.71E-17	4.51E-17	3.67E-17	2.88E-17	3.86E-17	4.64E-17	2.30E-17	4.08E-17	5.60E-17	5.43E-17
4.37E-17	5.50E-17	5.22E-17	5.87E-17	5.80E-17	4.26E-17	3.58E-17	3.36E-17	5.15E-17	5.54E-17	3.84E-17	5.42E-17	6.18E-17
5.18E-17	5.79E-17	5.60E-17	6.47E-17	6.08E-17	4.58E-17	4.12E-17	4.22E-17	5.70E-17	4.93E-17	4.26E-17	4.94E-17	6.33E-17
6.26E-17	5.90E-17	6.27E-17	6.49E-17	5.71E-17	4.50E-17	5.45E-17	5.04E-17	6.11E-17	4.83E-17	4.63E-17	5.07E-17	6.31E-17
7.38E-17	6.79E-17	6.60E-17	6.40E-17	5.50E-17	5.22E-17	5.83E-17	6.30E-17	7.02E-17	5.49E-17	4.60E-17	5.40E-17	6.11E-17
7.79E-17	7.51E-17	7.16E-17	6.71E-17	6.19E-17	5.98E-17	6.03E-17	7.31E-17	7.97E-17	5.94E-17	5.38E-17	5.67E-17	5.82E-17
7.72E-17	8.25E-17	7.86E-17	7.24E-17	6.91E-17	6.93E-17	6.21E-17	7.23E-17	7.32E-17	5.64E-17	5.74E-17	5.87E-17	5.84E-17
7.93E-17	8.72E-17	8.13E-17	8.17E-17	6.35E-17	7.28E-17	6.30E-17	7.15E-17	7.16E-17	5.58E-17	5.19E-17	5.64E-17	5.84E-17
7.55E-17	8.09E-17	8.29E-17	8.02E-17	5.53E-17	7.21E-17	6.28E-17	7.03E-17	6.57E-17	5.91E-17	4.82E-17	5.94E-17	5.91E-17
6.17E-17	6.82E-17	7.85E-17	7.08E-17	5.23E-17	6.77E-17	6.17E-17	6.10E-17	5.54E-17	5.68E-17	4.98E-17	6.14E-17	5.84E-17
5.76E-17	6.42E-17	6.67E-17	6.17E-17	5.65E-17	6.29E-17	6.22E-17	5.51E-17	5.11E-17	4.86E-17	5.68E-17	5.98E-17	5.93E-17
6.56E-17	6.62E-17	5.73E-17	5.29E-17	5.21E-17	6.29E-17	5.31E-17	5.47E-17	5.09E-17	4.35E-17	4.87E-17	5.63E-17	6.58E-17
7.23E-17	6.49E-17	5.08E-17	4.88E-17	5.22E-17	5.80E-17	4.63E-17	5.42E-17	4.70E-17	3.70E-17	3.90E-17	4.25E-17	6.10E-17
6.54E-17	6.07E-17	4.43E-17	4.94E-17	4.41E-17	4.21E-17	3.96E-17	4.68E-17	3.63E-17	3.02E-17	3.56E-17	2.79E-17	4.74E-17
5.36E-17	5.50E-17	3.90E-17	4.31E-17	3.68E-17	3.01E-17	3.32E-17	4.13E-17	2.36E-17	2.49E-17	3.06E-17	2.00E-17	3.88E-17
4.54E-17	4.49E-17	2.81E-17	3.19E-17	3.14E-17	2.36E-17	3.06E-17	3.59E-17	2.08E-17	1.75E-17	2.12E-17	1.75E-17	3.49E-17
3.67E-17	3.77E-17	2.06E-17	2.83E-17	2.47E-17	2.57E-17	2.68E-17	2.82E-17	1.83E-17	1.78E-17	1.79E-17	2.13E-17	3.21E-17
2.94E-17	3.11E-17	1.99E-17	2.90E-17	2.37E-17	2.17E-17	1.98E-17	2.44E-17	1.27E-17	1.86E-17	1.98E-17	1.96E-17	2.81E-17
2.34E-17	2.26E-17	1.62E-17	2.64E-17	1.91E-17	1.71E-17	1.52E-17	2.22E-17	9.92E-18	1.37E-17	1.62E-17	1.46E-17	2.26E-17
1.76E-17	1.59E-17	1.20E-17	2.01E-17	1.60E-17	1.39E-17	1.70E-17	1.99E-17	1.07E-17	1.19E-17	9.95E-18	1.12E-17	1.44E-17
1.32E-17	1.22E-17	1.07E-17	1.24E-17	9.69E-18	1.14E-17	1.87E-17	1.67E-17	8.77E-18	1.01E-17	8.88E-18	9.11E-18	1.28E-17
9.69E-18	8.72E-18	8.84E-18	9.23E-18	6.29E-18	9.43E-18	1.78E-17	1.39E-17	6.11E-18	7.64E-18	7.93E-18	9.04E-18	1.46E-17
6.20E-18	4.85E-18	6.22E-18	6.45E-18	5.04E-18	7.91E-18	1.35E-17	1.34E-17	5.03E-18	4.98E-18	7.37E-18	7.34E-18	1.26E-17
3.46E-18	3.69E-18	5.58E-18	4.02E-18	2.72E-18	6.92E-18	9.72E-18	1.14E-17	4.91E-18	3.62E-18	6.96E-18	4.96E-18	9.71E-18
2.97E-18	3.28E-18	3.86E-18	2.94E-18	1.49E-18	4.74E-18	6.13E-18	8.33E-18	4.03E-18	3.75E-18	7.61E-18	5.48E-18	8.48E-18
4.18E-18	3.18E-18	2.58E-18	1.94E-18	1.20E-18	3.69E-18	4.11E-18	4.41E-18	1.76E-18	1.27E-18	7.16E-18	6.43E-18	7.32E-18
3.58E-18	3.16E-18	1.59E-18	4.80E-19	1.22E-18	2.11E-18	2.82E-18	1.64E-18	8.78E-19	7.77E-19	3.90E-18	5.54E-18	5.12E-18
2.18E-18	2.49E-18	7.11E-19	6.33E-23	7.56E-19	1.62E-18	3.24E-18	7.83E-19	2.08E-18	2.22E-18	3.12E-18	3.17E-18	2.30E-18
2.04E-18	9.02E-19	8.93E-20	3.57E-19	3.20E-19	5.34E-19	1.51E-18	3.30E-19	1.69E-18	2.15E-18	2.43E-18	1.78E-18	1.08E-18
7.23E-19	3.19E-19	3.21E-20	6.76E-19	2.07E-23	3.55E-20	6.15E-19	7.59E-19	9.24E-19	5.71E-19	5.95E-19	4.12E-19	2.30E-20
3.52E-19	2.54E-20	6.32E-20	8.54E-19	4.67E-20	4.47E-21	4.50E-20	4.02E-19	3.64E-19	1.34E-20	4.28E-21	1.89E-22	5.67E-19
3.19E-20	1.17E-19	1.51E-20	5.77E-19	1.16E-21	1.18E-19	3.38E-21	2.22E-21	3.01E-20	2.72E-19	1.02E-19	6.76E-19	9.08E-19
8.31E-20	6.79E-19	4.89E-19	4.69E-19	7.56E-20	9.17E-19	9.68E-20	5.21E-19	4.82E-19	4.95E-19	3.82E-19	7.85E-19	6.66E-19
6.57E-19	2.33E-18	1.52E-18	1.37E-18	7.61E-19	2.30E-18	5.67E-19	2.70E-18	9.19E-19	1.06E-18	7.73E-19	1.94E-19	6.35E-19
1.01E-18	5.14E-18	2.57E-18	1.82E-18	1.08E-18	2.02E-18	1.45E-18	5.45E-18	6.35E-19	8.27E-19	6.46E-19	1.73E-19	1.30E-18
1.91E-18	6.55E-18	3.76E-18	3.60E-18	1.02E-18	1.52E-18	1.52E-18	5.53E-18	6.99E-19	8.76E-19	1.21E-18	3.63E-19	2.34E-18
3.47E-18	7.01E-18	3.39E-18	3.22E-18	2.06E-18	1.99E-18	2.21E-18	4.76E-18	1.28E-18	2.13E-18	2.01E-18	1.81E-18	5.23E-18
7.24E-18	8.03E-18	4.81E-18	3.50E-18	2.97E-18	2.87E-18	3.90E-18	5.61E-18	2.47E-18	2.73E-18	3.24E-18	1.95E-18	7.15E-18
1.37E-17	1.01E-17	7.86E-18	7.76E-18	4.96E-18	4.38E-18	6.37E-18	7.56E-18	4.92E-18	4.36E-18	5.81E-18	2.29E-18	4.37E-18
1.43E-17	1.18E-17	1.19E-17	1.20E-17	7.73E-18	5.60E-18	1.06E-17	8.78E-18	6.09E-18	5.98E-18	9.30E-18	4.00E-18	5.47E-18
1.37E-17	1.33E-17	1.71E-17	1.52E-17	8.89E-18	7.43E-18	1.25E-17	1.14E-17	8.99E-18	8.81E-18	1.52E-17	8.73E-18	7.93E-18
1.65E-17	1.52E-17	2.02E-17	2.32E-17	1.39E-17	1.43E-17	2.07E-17	1.78E-17	1.31E-17	1.20E-17	2.49E-17	1.62E-17	1.16E-17
2.20E-17	2.03E-17	2.08E-17	2.71E-17	2.26E-17	2.17E-17	2.32E-17	2.53E-17	1.61E-17	1.50E-17	2.81E-17	2.53E-17	1.50E-17
2.56E-17	2.93E-17	2.37E-17	2.45E-17	3.35E-17	2.30E-17	2.48E-17	2.71E-17	1.73E-17	1.79E-17	2.84E-17	2.82E-17	2.17E-17
2.62E-17	3.52E-17	3.11E-17	2.33E-17	3.08E-17	2.80E-17	2.84E-17	2.72E-17	2.27E-17	2.52E-17	2.89E-17	2.70E-17	2.63E-17
3.46E-17	4.29E-17	4.15E-17	3.07E-17	3.24E-17	3.79E-17	3.08E-17	2.84E-17	3.07E-17	2.95E-17	3.05E-17	2.61E-17	2.87E-17
4.37E-17	4.40E-17	4.62E-17	3.72E-17	3.72E-17	4.22E-17	3.42E-17	3.30E-17	3.77E-17	3.23E-17	3.42E-17	2.77E-17	2.86E-17
6.18E-17	4.92E-17	4.98E-17	4.57E-17	4.45E-17	4.52E-17	3.99E-17	3.85E-17	4.20E-17	3.30E-17	3.72E-17	3.42E-17	4.18E-17
6.69E-17	5.78E-17	5.76E-17	5.58E-17	5.21E-17	4.71E-17	4.66E-17	4.60E-17	4.90E-17	4.33E-17	4.52E-17	4.85E-17	6.63E-17
6.59E-17	6.78E-17	6.46E-17	7.01E-17	5.77E-17	5.27E-17	5.84E-17	5.83E-17	5.56E-17	5.35E-17	5.56E-17	6.37E-17	8.20E-17
7.07E-17	8.20E-17	7.01E-17	7.22E-17	6.53E-17	5.87E-17	6.78E-17	6.27E-17	6.17E-17	6.18E-17	6.86E-17	7.10E-17	6.00E-17
7.83E-17	8.73E-17	7.75E-17	7.52E-17	7.82E-17	6.71E-17	7.50E-17	6.41E-17	6.88E-17	6.96E-17	8.27E-17	8.02E-17	6.49E-17
9.60E-17	9.16E-17	9.15E-17	9.29E-17	9.25E-17	8.21E-17	8.35E-17	7.27E-17	8.30E-17	8.45E-17	9.06E-17	8.52E-17	7.54E-17
1.16E-16	1.07E-16	1.12E-16	1.15E-16	1.08E-16	9.64E-17	9.96E-17	8.79E-17	1.04E-16	9.54E-17	9.95E-17	9.20E-17	8.06E-17
1.40E-16	1.26E-16	1.30E-16	1.30E-16	1.25E-16	1.20E-16	1.15E-16	1.06E-16	1.11E-16	1.06E-16	1.03E-16	9.61E-17	8.24E-17
1.61E-16	1.38E-16	1.42E-16	1.35E-16	1.36E-16	1.38E-16	1.29E-16	1.26E-16	1.21E-16	1.20E-16	1.10E-16	1.09E-16	1.03E-16
1.80E-16	1.52E-16	1.60E-16	1.46E-16	1.48E-16	1.57E-16	1.39E-16	1.36E-16	1.45E-16	1.33E-16	1.29E-16	1.29E-16	1.28E-16
1.95E-16	1.71E-16	1.80E-16	1.72E-16	1.56E-16	1.70E-16	1.46E-16	1.47E-16	1.63E-16	1.50E-16	1.45E-16	1.53E-16	1.45E-16
2.07E-16	1.88E-16	2.03E-16	2.02E-16	1.81E-16	1.94E-16	1.59E-16	1.74E-16	1.78E-16	1.66E-16	1.65E-16	1.70E-16	1.60E-16
2.18E-16	1.99E-16	2.32E-16	2.38E-16	2.20E-16	2.21E-16	1.86E-16	2.04E-16	1.96E-16	1.75E-16	1.89E-16	2.00E-16	1.87E-16
2.36E-16	2.33E-16	2.58E-16	2.63E-16	2.51E-16	2.49E-16	2.15E-16	2.30E-16	2.22E-16	1.96E-16	2.09E-16	2.30E-16	2.07E-16
2.66E-16	2.70E-16	2.74E-16	2.83E-16	2.70E-16	2.88E-16	2.45E-16	2.61E-16	2.51E-16	2.16E-16	2.08E-16	2.35E-16	2.21E-16
2.83E-16	2.97E-16	3.01E-16	3.17E-16	3.07E-16	3.18E-16	2.75E-16	2.90E-16	2.72E-16	2.44E-16	2.41E-16	2.49E-16	2.40E-16
2.96E-16	3.14E-16	3.35E-16	3.52E-16	3.33E-16	3.16E-16	2.97E-16	2.90E-16	2.84E-16	2.67E-16	2.74E-16	2.85E-16	2.72E-16
3.06E-16	3.24E-16	3.57E-16	3.73E-16	3.63E-16	3.52E-16	3.13E-16	3.11E-16	3.01E-16	2.86E-16	2.91E-16	3.10E-16	2.96E-16
3.37E-16	3.66E-16	3.95E-16	4.02E-16	3.85E-16	3.90E-16	3.42E-16	3.32E-16	3.28E-16	3.09E-16	3.09E-16	3.21E-16	3.04E-16
3.84E-16	4.16E-16	4.40E-16	4.08E-16	4.00E-16	4.03E-16	3.75E-16	3.76E-16	3.51E-16	3.38E-16	3.21E-16	3.17E-16	3.14E-16
4.20E-16	4.32E-16	4.39E-16	4.27E-16	4.24E-16	4.19E-16	4.22E-16	4.22E-16	3.78E-16	3.69E-16	3.44E-16	3.27E-16	3.46E-16
4.45E-16	4.75E-16	4.60E-16	4.59E-16	4.51E-16	4.56E-16	4.63E-16	4.63E-16	4.05E-16	3.93E-16	3.61E-16	3.38E-16	3.76E-16
4.83E-16	5.08E-16	4.92E-16	4.90E-16	4.90E-16	5.03E-16	5.04E-16	4.90E-16	4.43E-16	4.14E-16	3.85E-16	3.65E-16	4.25E-16
5.28E-16	5.31E-16	5.16E-16	5.36E-16	5.42E-16	5.38E-16	5.33E-16	5.02E-16	4.76E-16	4.54E-16	4.18E-16	4.08E-16	4.47E-16
5.57E-16	5.46E-16	5.42E-16	5.77E-16	5.51E-16	5.56E-16	5.49E-16	5.19E-16	5.13E-16	4.99E-16	4.47E-16	4.56E-16	4.70E-16

5.76E-16	5.71E-16	5.50E-16	5.87E-16	5.69E-16	5.67E-16	5.56E-16	5.52E-16	5.47E-16	5.29E-16	4.89E-16	4.95E-16	4.78E-16	
5.93E-16	5.80E-16	5.78E-16	5.98E-16	5.89E-16	5.85E-16	5.92E-16	5.76E-16	5.64E-16	5.64E-16	5.38E-16	5.39E-16	5.32E-16	
6.06E-16	6.08E-16	6.02E-16	6.33E-16	6.21E-16	6.14E-16	6.31E-16	6.00E-16	6.00E-16	5.98E-16	5.73E-16	5.87E-16	5.89E-16	
6.53E-16	6.32E-16	6.34E-16	6.42E-16	6.60E-16	6.51E-16	6.64E-16	6.49E-16	6.49E-16	6.43E-16	6.06E-16	6.07E-16	6.23E-16	
7.01E-16	6.87E-16	6.86E-16	6.74E-16	6.89E-16	6.82E-16	7.15E-16	7.01E-16	6.97E-16	6.93E-16	6.51E-16	6.37E-16	6.44E-16	
7.40E-16	7.65E-16	7.45E-16	7.18E-16	7.04E-16	7.07E-16	7.60E-16	7.62E-16	7.42E-16	7.31E-16	7.00E-16	6.85E-16	6.76E-16	
8.24E-16	8.21E-16	7.85E-16	7.55E-16	7.30E-16	7.58E-16	8.12E-16	8.24E-16	7.95E-16	7.69E-16	7.48E-16	7.38E-16	7.25E-16	
9.22E-16	8.53E-16	8.02E-16	7.97E-16	7.81E-16	8.21E-16	8.93E-16	9.20E-16	8.36E-16	7.82E-16	7.95E-16	7.94E-16	7.72E-16	
9.96E-16	9.07E-16	8.60E-16	8.60E-16	8.48E-16	8.74E-16	9.78E-16	9.70E-16	8.84E-16	8.27E-16	8.43E-16	8.29E-16	8.04E-16	
1.04E-15	9.66E-16	9.29E-16	9.23E-16	9.12E-16	9.39E-16	1.03E-15	9.86E-16	9.23E-16	9.03E-16	8.94E-16	8.65E-16	8.54E-16	
1.12E-15	1.05E-15	1.01E-15	9.85E-16	9.91E-16	1.01E-15	1.09E-15	1.02E-15	9.42E-16	9.66E-16	9.53E-16	9.31E-16	9.21E-16	
1.99E-14	1.96E-14	1.94E-14	1.94E-14	1.89E-14	1.88E-14	1.90E-14	1.90E-14	1.81E-14	1.75E-14	1.70E-14	1.69E-14	1.69E-14	6.04E-12

SUM OF SQUARED QUADRA	6.04E-12
TOTAL NUMBER VALUES IN (	16,384
AVERAGE SQUARED	3.69E-16
RMS	1.9207E-08

Quadrant 4 Data (16384 values):

-1.05E-08	-9.97E-09	-9.16E-09	-9.72E-09	-1.04E-08	-9.35E-09	-9.59E-09	-9.73E-09
-1.03E-08	-9.50E-09	-9.36E-09	-9.23E-09	-9.80E-09	-9.17E-09	-9.27E-09	-9.53E-09
-9.41E-09	-9.29E-09	-9.63E-09	-9.13E-09	-9.15E-09	-8.97E-09	-9.01E-09	-9.25E-09
-8.92E-09	-9.52E-09	-9.91E-09	-9.18E-09	-9.16E-09	-9.10E-09	-9.22E-09	-9.32E-09
-8.91E-09	-1.01E-08	-1.03E-08	-9.32E-09	-9.26E-09	-8.92E-09	-9.17E-09	-9.23E-09
-9.11E-09	-1.01E-08	-1.04E-08	-9.10E-09	-9.45E-09	-9.09E-09	-8.96E-09	-9.30E-09
-9.56E-09	-1.03E-08	-9.84E-09	-8.38E-09	-9.37E-09	-9.12E-09	-8.74E-09	-9.33E-09
-9.77E-09	-1.00E-08	-8.94E-09	-7.85E-09	-8.56E-09	-8.98E-09	-8.24E-09	-9.62E-09
-9.48E-09	-9.48E-09	-7.77E-09	-7.55E-09	-8.17E-09	-9.02E-09	-8.08E-09	-9.56E-09
-9.16E-09	-8.94E-09	-7.52E-09	-7.70E-09	-8.48E-09	-9.32E-09	-8.26E-09	-9.24E-09
-8.79E-09	-8.88E-09	-7.62E-09	-8.20E-09	-8.93E-09	-9.26E-09	-7.78E-09	-8.58E-09
-8.49E-09	-8.48E-09	-7.48E-09	-8.43E-09	-9.20E-09	-8.83E-09	-7.59E-09	-7.83E-09
-8.48E-09	-8.64E-09	-7.66E-09	-8.15E-09	-8.75E-09	-8.56E-09	-7.80E-09	-7.49E-09
-8.73E-09	-8.60E-09	-7.71E-09	-8.08E-09	-8.22E-09	-7.93E-09	-7.78E-09	-7.65E-09
-8.47E-09	-7.90E-09	-7.30E-09	-7.67E-09	-7.76E-09	-7.68E-09	-7.94E-09	-7.82E-09
-8.19E-09	-7.57E-09	-7.22E-09	-7.25E-09	-7.28E-09	-7.32E-09	-7.50E-09	-7.55E-09
-7.78E-09	-7.37E-09	-6.90E-09	-6.92E-09	-6.81E-09	-7.01E-09	-6.88E-09	-7.01E-09
-7.31E-09	-6.87E-09	-6.86E-09	-7.02E-09	-6.55E-09	-6.92E-09	-6.79E-09	-6.72E-09
-6.92E-09	-6.14E-09	-6.82E-09	-7.23E-09	-7.04E-09	-6.66E-09	-6.71E-09	-6.68E-09
-6.85E-09	-5.70E-09	-6.68E-09	-7.61E-09	-7.63E-09	-6.71E-09	-6.67E-09	-6.12E-09
-7.00E-09	-6.26E-09	-7.00E-09	-7.77E-09	-7.36E-09	-6.41E-09	-6.52E-09	-5.80E-09
-7.35E-09	-6.93E-09	-6.94E-09	-7.58E-09	-6.76E-09	-6.05E-09	-6.76E-09	-6.22E-09
-7.72E-09	-7.07E-09	-6.70E-09	-7.46E-09	-7.04E-09	-6.54E-09	-7.31E-09	-7.11E-09
-8.11E-09	-6.85E-09	-6.68E-09	-7.66E-09	-7.63E-09	-7.28E-09	-7.45E-09	-7.57E-09
-8.11E-09	-6.80E-09	-6.71E-09	-7.77E-09	-7.50E-09	-7.31E-09	-7.28E-09	-7.61E-09
-7.81E-09	-6.71E-09	-7.00E-09	-7.52E-09	-6.93E-09	-7.26E-09	-6.94E-09	-7.29E-09
-7.73E-09	-6.93E-09	-7.22E-09	-7.28E-09	-6.56E-09	-6.95E-09	-6.52E-09	-7.06E-09
-7.63E-09	-7.29E-09	-7.20E-09	-6.98E-09	-6.60E-09	-6.87E-09	-6.35E-09	-7.35E-09
-7.93E-09	-7.66E-09	-7.25E-09	-6.68E-09	-6.68E-09	-7.04E-09	-6.59E-09	-7.65E-09
-7.87E-09	-7.79E-09	-7.79E-09	-6.73E-09	-6.36E-09	-6.94E-09	-6.20E-09	-7.43E-09
-7.51E-09	-7.57E-09	-7.89E-09	-7.04E-09	-6.72E-09	-6.87E-09	-6.45E-09	-7.05E-09
-6.91E-09	-7.56E-09	-7.75E-09	-6.93E-09	-6.55E-09	-6.62E-09	-6.10E-09	-6.99E-09
-6.46E-09	-7.50E-09	-7.43E-09	-6.81E-09	-6.31E-09	-6.44E-09	-5.81E-09	-6.91E-09
-6.68E-09	-7.55E-09	-7.40E-09	-7.13E-09	-6.62E-09	-6.71E-09	-5.85E-09	-6.84E-09
-6.95E-09	-7.52E-09	-7.60E-09	-7.24E-09	-6.82E-09	-6.76E-09	-6.37E-09	-7.06E-09
-7.03E-09	-7.69E-09	-7.75E-09	-7.21E-09	-6.65E-09	-6.35E-09	-6.46E-09	-7.42E-09
-7.29E-09	-7.81E-09	-7.68E-09	-7.26E-09	-6.27E-09	-5.98E-09	-6.39E-09	-7.46E-09
-7.34E-09	-7.54E-09	-7.24E-09	-6.92E-09	-6.13E-09	-6.36E-09	-6.53E-09	-7.27E-09
-6.83E-09	-6.92E-09	-6.49E-09	-6.84E-09	-6.47E-09	-6.91E-09	-6.60E-09	-7.08E-09
-6.73E-09	-6.61E-09	-6.39E-09	-6.77E-09	-6.34E-09	-7.01E-09	-6.52E-09	-6.92E-09
-6.98E-09	-7.01E-09	-6.73E-09	-6.47E-09	-6.59E-09	-6.96E-09	-6.44E-09	-6.46E-09
-6.72E-09	-7.34E-09	-6.96E-09	-6.12E-09	-6.94E-09	-6.94E-09	-6.39E-09	-6.29E-09
-6.73E-09	-7.61E-09	-7.22E-09	-6.43E-09	-7.25E-09	-7.24E-09	-6.40E-09	-6.09E-09
-6.66E-09	-7.13E-09	-7.12E-09	-6.21E-09	-6.77E-09	-7.32E-09	-6.31E-09	-5.87E-09
-6.75E-09	-6.93E-09	-6.87E-09	-5.57E-09	-6.33E-09	-7.58E-09	-6.90E-09	-6.00E-09
-7.09E-09	-6.60E-09	-6.49E-09	-6.12E-09	-5.68E-09	-7.69E-09	-6.98E-09	-6.38E-09
-7.22E-09	-6.44E-09	-6.62E-09	-6.91E-09	-7.16E-09	-7.74E-09	-6.73E-09	-7.04E-09
-7.26E-09	-6.18E-09	-6.26E-09	-6.83E-09	-7.53E-09	-7.64E-09	-6.93E-09	-7.06E-09
-7.89E-09	-6.64E-09	-6.39E-09	-6.98E-09	-7.69E-09	-7.80E-09	-6.87E-09	-7.39E-09
-8.12E-09	-7.52E-09	-6.97E-09	-7.01E-09	-7.42E-09	-7.48E-09	-6.79E-09	-7.28E-09
-8.10E-09	-7.72E-09	-7.45E-09	-6.88E-09	-7.51E-09	-7.08E-09	-6.65E-09	-6.83E-09
-8.17E-09	-8.17E-09	-7.74E-09	-7.02E-09	-7.74E-09	-7.15E-09	-6.69E-09	-6.73E-09
-8.02E-09	-8.05E-09	-7.56E-09	-7.16E-09	-7.90E-09	-7.33E-09	-7.02E-09	-7.00E-09
-7.87E-09	-7.50E-09	-7.28E-09	-7.16E-09	-7.63E-09	-7.08E-09	-7.21E-09	-7.37E-09
-7.37E-09	-6.71E-09	-7.08E-09	-7.43E-09	-7.47E-09	-6.89E-09	-7.11E-09	-7.28E-09
-7.35E-09	-6.30E-09	-7.13E-09	-7.41E-09	-7.04E-09	-6.66E-09	-6.87E-09	-6.78E-09
-7.64E-09	-6.24E-09	-6.55E-09	-7.14E-09	-7.01E-09	-6.52E-09	-6.21E-09	-5.88E-09
-7.42E-09	-6.22E-09	-6.42E-09	-7.11E-09	-6.73E-09	-6.29E-09	-5.65E-09	-5.35E-09
-7.31E-09	-6.09E-09	-6.10E-09	-6.68E-09	-6.21E-09	-5.98E-09	-5.13E-09	-4.74E-09
-6.78E-09	-5.63E-09	-5.30E-09	-6.37E-09	-5.64E-09	-5.72E-09	-4.83E-09	-4.54E-09
-6.37E-09	-5.56E-09	-4.99E-09	-6.29E-09	-5.46E-09	-5.20E-09	-4.86E-09	-4.78E-09
-6.09E-09	-5.35E-09	-4.78E-09	-5.69E-09	-4.70E-09	-4.59E-09	-5.54E-09	-4.80E-09
-5.56E-09	-4.57E-09	-4.38E-09	-5.03E-09	-4.17E-09	-4.44E-09	-5.38E-09	-5.01E-09
-4.77E-09	-3.88E-09	-4.20E-09	-4.98E-09	-4.59E-09	-4.71E-09	-5.54E-09	-4.55E-09
-3.93E-09	-3.15E-09	-4.00E-09	-4.30E-09	-4.41E-09	-4.55E-09	-5.52E-09	-3.76E-09
-3.56E-09	-3.04E-09	-3.55E-09	-3.64E-09	-3.95E-09	-4.24E-09	-5.14E-09	-3.25E-09
-3.43E-09	-2.87E-09	-3.58E-09	-3.75E-09	-3.69E-09	-3.82E-09	-4.49E-09	-3.34E-09
-3.33E-09	-3.04E-09	-3.76E-09	-3.74E-09	-3.29E-09	-3.24E-09	-3.90E-09	-2.99E-09
-3.34E-09	-2.93E-09	-3.56E-09	-3.41E-09	-2.83E-09	-3.35E-09	-3.46E-09	-2.91E-09
-2.83E-09	-2.52E-09	-2.90E-09	-2.70E-09	-2.06E-09	-3.24E-09	-2.87E-09	-2.67E-09
-2.51E-09	-2.39E-09	-2.18E-09	-1.90E-09	-1.26E-09	-2.66E-09	-2.68E-09	-2.59E-09
-1.79E-09	-2.07E-09	-1.63E-09	-1.57E-09	-1.18E-09	-2.26E-09	-2.09E-09	-2.35E-09
-1.62E-09	-1.95E-09	-1.67E-09	-1.35E-09	-9.93E-10	-1.65E-09	-1.71E-09	-1.99E-09
-1.42E-09	-1.64E-09	-1.58E-09	-1.12E-09	-2.83E-10	-1.16E-09	-1.62E-09	-1.58E-09
-9.10E-10	-1.26E-09	-1.40E-09	-1.33E-09	3.95E-12	-1.04E-09	-1.30E-09	-1.19E-09
-5.50E-10	-1.05E-09	-1.43E-09	-1.31E-09	-1.59E-10	-1.04E-09	-1.27E-09	-1.09E-09
-2.70E-10	-6.76E-10	-1.01E-09	-1.23E-09	-7.82E-10	-8.50E-10	-1.21E-09	-1.09E-09
2.47E-10	-4.44E-10	-1.42E-10	-7.34E-10	-3.16E-10	-4.49E-10	-9.16E-10	-8.64E-10

6.87E-10	2.03E-10	3.22E-10	9.58E-11	3.59E-10	-3.29E-10	-2.12E-10	-4.19E-10
1.29E-09	5.40E-10	5.30E-10	6.14E-10	9.10E-10	5.07E-11	3.87E-10	7.44E-11
1.33E-09	5.70E-10	9.86E-10	7.52E-10	9.83E-10	2.16E-10	7.61E-10	7.86E-10
1.54E-09	1.03E-09	1.15E-09	1.06E-09	6.68E-10	6.49E-10	9.57E-10	1.49E-09
2.08E-09	1.93E-09	1.68E-09	1.82E-09	1.20E-09	8.62E-10	1.13E-09	1.85E-09
2.46E-09	2.56E-09	2.41E-09	2.58E-09	1.84E-09	1.62E-09	1.34E-09	1.70E-09
2.89E-09	3.14E-09	2.92E-09	3.23E-09	2.40E-09	2.28E-09	2.02E-09	1.67E-09
3.18E-09	3.29E-09	3.39E-09	3.48E-09	2.85E-09	3.02E-09	2.26E-09	2.15E-09
3.30E-09	3.35E-09	3.13E-09	3.34E-09	3.38E-09	3.61E-09	2.22E-09	2.48E-09
3.80E-09	3.92E-09	3.60E-09	3.01E-09	3.56E-09	4.36E-09	2.61E-09	2.81E-09
4.61E-09	4.46E-09	3.83E-09	3.26E-09	3.72E-09	4.38E-09	2.84E-09	3.16E-09
5.56E-09	4.66E-09	4.43E-09	3.79E-09	3.82E-09	4.25E-09	3.28E-09	3.34E-09
5.92E-09	4.97E-09	4.79E-09	4.29E-09	3.66E-09	4.70E-09	3.92E-09	3.63E-09
6.06E-09	5.33E-09	5.44E-09	4.86E-09	4.52E-09	5.20E-09	4.17E-09	4.08E-09
6.49E-09	5.69E-09	6.09E-09	5.75E-09	5.12E-09	5.17E-09	4.54E-09	4.73E-09
7.12E-09	6.04E-09	6.79E-09	6.50E-09	5.25E-09	5.21E-09	5.52E-09	5.14E-09
7.85E-09	6.77E-09	7.22E-09	6.47E-09	5.62E-09	5.95E-09	6.53E-09	5.97E-09
8.15E-09	7.54E-09	7.39E-09	6.79E-09	5.84E-09	6.75E-09	7.14E-09	6.49E-09
8.49E-09	8.40E-09	7.56E-09	7.04E-09	6.43E-09	7.58E-09	7.44E-09	6.99E-09
8.73E-09	8.87E-09	7.83E-09	7.49E-09	7.33E-09	7.84E-09	7.91E-09	7.65E-09
8.97E-09	9.45E-09	7.98E-09	8.00E-09	7.93E-09	8.21E-09	8.64E-09	8.24E-09
9.06E-09	9.91E-09	8.66E-09	8.67E-09	8.92E-09	9.12E-09	8.46E-09	8.81E-09
9.50E-09	1.06E-08	9.56E-09	9.36E-09	9.80E-09	9.75E-09	8.38E-09	9.57E-09
1.04E-08	1.10E-08	1.04E-08	1.03E-08	1.01E-08	1.05E-08	9.22E-09	9.88E-09
1.13E-08	1.14E-08	1.12E-08	1.13E-08	1.07E-08	1.10E-08	9.75E-09	9.94E-09
1.19E-08	1.23E-08	1.16E-08	1.20E-08	1.13E-08	1.15E-08	1.02E-08	1.05E-08
1.29E-08	1.30E-08	1.20E-08	1.22E-08	1.19E-08	1.21E-08	1.14E-08	1.11E-08
1.40E-08	1.36E-08	1.25E-08	1.26E-08	1.26E-08	1.28E-08	1.24E-08	1.20E-08
1.48E-08	1.42E-08	1.35E-08	1.33E-08	1.30E-08	1.33E-08	1.30E-08	1.33E-08
1.56E-08	1.47E-08	1.42E-08	1.41E-08	1.36E-08	1.36E-08	1.35E-08	1.37E-08
1.61E-08	1.52E-08	1.51E-08	1.50E-08	1.46E-08	1.42E-08	1.36E-08	1.38E-08
1.64E-08	1.60E-08	1.58E-08	1.56E-08	1.54E-08	1.49E-08	1.43E-08	1.43E-08
1.69E-08	1.68E-08	1.65E-08	1.65E-08	1.61E-08	1.57E-08	1.53E-08	1.50E-08
1.77E-08	1.75E-08	1.73E-08	1.73E-08	1.71E-08	1.65E-08	1.64E-08	1.61E-08
1.85E-08	1.79E-08	1.82E-08	1.78E-08	1.79E-08	1.71E-08	1.73E-08	1.69E-08
1.91E-08	1.85E-08	1.88E-08	1.84E-08	1.87E-08	1.82E-08	1.85E-08	1.74E-08
1.94E-08	1.91E-08	1.94E-08	1.93E-08	1.94E-08	1.91E-08	1.96E-08	1.85E-08
2.05E-08	1.99E-08	2.00E-08	1.97E-08	2.01E-08	1.97E-08	2.05E-08	1.95E-08
2.13E-08	2.07E-08	2.06E-08	2.07E-08	2.05E-08	2.05E-08	2.12E-08	2.07E-08
2.19E-08	2.13E-08	2.15E-08	2.19E-08	2.10E-08	2.11E-08	2.22E-08	2.19E-08
2.24E-08	2.18E-08	2.20E-08	2.26E-08	2.18E-08	2.21E-08	2.30E-08	2.32E-08
2.34E-08	2.25E-08	2.25E-08	2.30E-08	2.29E-08	2.30E-08	2.38E-08	2.37E-08
2.44E-08	2.35E-08	2.35E-08	2.42E-08	2.41E-08	2.37E-08	2.45E-08	2.42E-08
2.54E-08	2.50E-08	2.46E-08	2.50E-08	2.47E-08	2.45E-08	2.50E-08	2.48E-08
2.65E-08	2.58E-08	2.60E-08	2.61E-08	2.55E-08	2.54E-08	2.55E-08	2.57E-08
2.72E-08	2.69E-08	2.71E-08	2.70E-08	2.65E-08	2.67E-08	2.61E-08	2.68E-08
2.83E-08	2.77E-08	2.81E-08	2.80E-08	2.73E-08	2.77E-08	2.72E-08	2.77E-08
2.92E-08	2.87E-08	2.94E-08	2.91E-08	2.84E-08	2.88E-08	2.85E-08	2.86E-08
2.99E-08	2.96E-08	3.06E-08	3.00E-08	2.92E-08	2.99E-08	2.94E-08	2.93E-08
3.07E-08	3.12E-08	3.08E-08	3.10E-08	3.05E-08	3.01E-08	3.03E-08	2.99E-08

Quadrant 4 Data Squared (16384 values):

1.11E-16	9.94E-17	8.40E-17	9.45E-17	1.09E-16	8.75E-17	9.20E-17	9.46E-17
1.05E-16	9.03E-17	8.76E-17	8.51E-17	9.61E-17	8.41E-17	8.59E-17	9.07E-17
8.86E-17	8.62E-17	9.27E-17	8.33E-17	8.38E-17	8.04E-17	8.13E-17	8.56E-17
7.96E-17	9.06E-17	9.81E-17	8.43E-17	8.39E-17	8.29E-17	8.50E-17	8.69E-17
7.94E-17	1.02E-16	1.07E-16	8.69E-17	8.57E-17	7.95E-17	8.40E-17	8.52E-17
8.29E-17	1.03E-16	1.08E-16	8.28E-17	8.93E-17	8.27E-17	8.03E-17	8.65E-17
9.15E-17	1.06E-16	9.69E-17	7.02E-17	8.79E-17	8.31E-17	7.64E-17	8.71E-17
9.55E-17	1.01E-16	8.00E-17	6.16E-17	7.33E-17	8.06E-17	6.79E-17	9.25E-17
8.98E-17	9.00E-17	6.04E-17	5.70E-17	6.67E-17	8.14E-17	6.52E-17	9.13E-17
8.39E-17	7.99E-17	5.66E-17	5.92E-17	7.20E-17	8.68E-17	6.82E-17	8.53E-17
7.73E-17	7.88E-17	5.80E-17	6.72E-17	7.97E-17	8.57E-17	6.05E-17	7.36E-17
7.21E-17	7.18E-17	5.59E-17	7.11E-17	8.46E-17	7.80E-17	5.76E-17	6.14E-17
7.20E-17	7.46E-17	5.86E-17	6.64E-17	7.66E-17	7.33E-17	6.09E-17	5.61E-17
7.62E-17	7.40E-17	5.94E-17	6.52E-17	6.75E-17	6.28E-17	6.05E-17	5.85E-17
7.17E-17	6.24E-17	5.32E-17	5.89E-17	6.03E-17	5.90E-17	6.30E-17	6.11E-17
6.71E-17	5.73E-17	5.21E-17	5.25E-17	5.30E-17	5.36E-17	5.63E-17	5.70E-17
6.05E-17	5.44E-17	4.76E-17	4.78E-17	4.63E-17	4.91E-17	4.73E-17	4.92E-17
5.34E-17	4.73E-17	4.71E-17	4.92E-17	4.29E-17	4.78E-17	4.60E-17	4.51E-17
4.79E-17	3.77E-17	4.65E-17	5.22E-17	4.95E-17	4.43E-17	4.50E-17	4.46E-17
4.70E-17	3.25E-17	4.47E-17	5.79E-17	5.82E-17	4.51E-17	4.45E-17	3.74E-17
4.90E-17	3.92E-17	4.90E-17	6.04E-17	5.41E-17	4.11E-17	4.25E-17	3.36E-17
5.40E-17	4.80E-17	4.82E-17	5.74E-17	4.58E-17	3.66E-17	4.57E-17	3.87E-17
5.96E-17	4.99E-17	4.49E-17	5.56E-17	4.96E-17	4.27E-17	5.34E-17	5.05E-17
6.58E-17	4.69E-17	4.47E-17	5.87E-17	5.81E-17	5.30E-17	5.55E-17	5.74E-17
6.57E-17	4.62E-17	4.51E-17	6.04E-17	5.62E-17	5.34E-17	5.30E-17	5.79E-17
6.11E-17	4.50E-17	4.90E-17	5.65E-17	4.80E-17	5.27E-17	4.81E-17	5.31E-17
5.97E-17	4.81E-17	5.21E-17	5.30E-17	4.31E-17	4.83E-17	4.25E-17	4.98E-17
5.82E-17	5.31E-17	5.19E-17	4.87E-17	4.36E-17	4.73E-17	4.03E-17	5.41E-17
6.28E-17	5.86E-17	5.25E-17	4.46E-17	4.46E-17	4.95E-17	4.34E-17	5.85E-17
6.19E-17	6.06E-17	6.06E-17	4.52E-17	4.05E-17	4.82E-17	3.85E-17	5.52E-17
5.64E-17	5.73E-17	6.23E-17	4.96E-17	4.52E-17	4.71E-17	4.16E-17	4.97E-17

4.77E-17	5.72E-17	6.01E-17	4.80E-17	4.29E-17	4.38E-17	3.72E-17	4.89E-17
4.17E-17	5.63E-17	5.52E-17	4.64E-17	3.98E-17	4.14E-17	3.37E-17	4.78E-17
4.46E-17	5.70E-17	5.48E-17	5.09E-17	4.38E-17	4.50E-17	3.42E-17	4.68E-17
4.83E-17	5.66E-17	5.78E-17	5.25E-17	4.65E-17	4.56E-17	4.05E-17	4.99E-17
4.95E-17	5.91E-17	6.01E-17	5.19E-17	4.43E-17	4.04E-17	4.17E-17	5.51E-17
5.32E-17	6.09E-17	5.90E-17	5.26E-17	3.93E-17	3.57E-17	4.08E-17	5.57E-17
5.39E-17	5.69E-17	5.24E-17	4.79E-17	3.76E-17	4.05E-17	4.27E-17	5.28E-17
4.67E-17	4.79E-17	4.21E-17	4.68E-17	4.19E-17	4.78E-17	4.36E-17	5.02E-17
4.53E-17	4.38E-17	4.09E-17	4.58E-17	4.03E-17	4.92E-17	4.25E-17	4.79E-17
4.87E-17	4.91E-17	4.52E-17	4.19E-17	4.34E-17	4.85E-17	4.15E-17	4.17E-17
4.52E-17	5.38E-17	4.84E-17	3.74E-17	4.81E-17	4.82E-17	4.09E-17	3.96E-17
4.52E-17	5.79E-17	5.21E-17	4.14E-17	5.26E-17	5.24E-17	4.10E-17	3.71E-17
4.43E-17	5.09E-17	5.07E-17	3.86E-17	4.59E-17	5.36E-17	3.99E-17	3.44E-17
4.56E-17	4.80E-17	4.72E-17	3.10E-17	4.01E-17	5.75E-17	4.76E-17	3.59E-17
5.03E-17	4.35E-17	4.21E-17	3.75E-17	3.23E-17	5.91E-17	4.87E-17	4.07E-17
5.21E-17	4.14E-17	4.38E-17	4.78E-17	5.13E-17	5.98E-17	4.52E-17	4.96E-17
5.27E-17	3.82E-17	3.92E-17	4.66E-17	5.67E-17	5.83E-17	4.80E-17	4.98E-17
6.22E-17	4.41E-17	4.09E-17	4.87E-17	5.92E-17	6.08E-17	4.73E-17	5.45E-17
6.60E-17	5.65E-17	4.86E-17	4.92E-17	5.51E-17	5.60E-17	4.61E-17	5.30E-17
6.57E-17	5.96E-17	5.55E-17	4.74E-17	5.64E-17	5.02E-17	4.43E-17	4.67E-17
6.68E-17	6.68E-17	5.99E-17	4.93E-17	5.99E-17	5.11E-17	4.48E-17	4.52E-17
6.43E-17	6.49E-17	5.72E-17	5.13E-17	6.24E-17	5.37E-17	4.93E-17	4.90E-17
6.19E-17	5.63E-17	5.30E-17	5.13E-17	5.83E-17	5.01E-17	5.21E-17	5.43E-17
5.43E-17	4.50E-17	5.01E-17	5.52E-17	5.58E-17	4.75E-17	5.05E-17	5.30E-17
5.41E-17	3.97E-17	5.09E-17	5.49E-17	4.95E-17	4.44E-17	4.71E-17	4.60E-17
5.83E-17	3.90E-17	4.29E-17	5.10E-17	4.91E-17	4.25E-17	3.86E-17	3.46E-17
5.50E-17	3.87E-17	4.12E-17	5.05E-17	4.53E-17	3.95E-17	3.19E-17	2.86E-17
5.34E-17	3.71E-17	3.72E-17	4.46E-17	3.86E-17	3.58E-17	2.63E-17	2.25E-17
4.59E-17	3.17E-17	2.81E-17	4.06E-17	3.18E-17	3.27E-17	2.33E-17	2.06E-17
4.05E-17	3.09E-17	2.49E-17	3.95E-17	2.98E-17	2.71E-17	2.36E-17	2.29E-17
3.71E-17	2.87E-17	2.29E-17	3.24E-17	2.21E-17	2.10E-17	3.07E-17	2.30E-17
3.09E-17	2.09E-17	1.92E-17	2.54E-17	1.73E-17	1.97E-17	2.89E-17	2.51E-17
2.28E-17	1.51E-17	1.76E-17	2.48E-17	2.10E-17	2.22E-17	3.07E-17	2.07E-17
1.55E-17	9.92E-18	1.60E-17	1.85E-17	1.95E-17	2.07E-17	3.05E-17	1.42E-17
1.27E-17	9.24E-18	1.26E-17	1.33E-17	1.56E-17	1.80E-17	2.64E-17	1.05E-17
1.18E-17	8.22E-18	1.28E-17	1.41E-17	1.36E-17	1.46E-17	2.02E-17	1.12E-17
1.11E-17	9.26E-18	1.42E-17	1.40E-17	1.08E-17	1.05E-17	1.52E-17	8.97E-18
1.12E-17	8.60E-18	1.27E-17	1.16E-17	8.02E-18	1.12E-17	1.20E-17	8.44E-18
8.02E-18	6.37E-18	8.39E-18	7.30E-18	4.24E-18	1.05E-17	8.26E-18	7.12E-18
6.30E-18	5.73E-18	4.74E-18	3.61E-18	1.60E-18	7.09E-18	7.20E-18	6.70E-18
3.22E-18	4.28E-18	2.64E-18	2.45E-18	1.39E-18	5.11E-18	4.37E-18	5.52E-18
2.62E-18	3.80E-18	2.78E-18	1.83E-18	9.87E-19	2.71E-18	2.94E-18	3.97E-18
2.01E-18	2.71E-18	2.50E-18	1.26E-18	7.99E-20	1.33E-18	2.61E-18	2.51E-18
8.28E-19	1.60E-18	1.95E-18	1.77E-18	1.56E-23	1.08E-18	1.69E-18	1.43E-18
3.02E-19	1.10E-18	2.04E-18	1.70E-18	2.53E-20	1.09E-18	1.61E-18	1.18E-18
7.32E-20	4.57E-19	1.02E-18	1.51E-18	6.12E-19	7.22E-19	1.46E-18	1.18E-18
6.09E-20	1.98E-19	2.01E-20	5.38E-19	9.98E-20	2.01E-19	8.38E-19	7.47E-19
4.72E-19	4.14E-20	1.04E-19	9.17E-21	1.29E-19	1.08E-19	4.48E-20	1.75E-19
1.66E-18	2.92E-19	2.81E-19	3.77E-19	8.29E-19	2.57E-21	1.50E-19	5.54E-21
1.77E-18	3.24E-19	9.72E-19	5.65E-19	9.66E-19	4.67E-20	5.79E-19	6.17E-19
2.38E-18	1.07E-18	1.33E-18	1.12E-18	4.47E-19	4.21E-19	9.16E-19	2.21E-18
4.32E-18	3.73E-18	2.82E-18	3.32E-18	1.44E-18	7.43E-19	1.29E-18	3.43E-18
6.07E-18	6.53E-18	5.81E-18	6.65E-18	3.40E-18	2.62E-18	1.80E-18	2.90E-18
8.36E-18	9.87E-18	8.54E-18	1.05E-17	5.77E-18	5.18E-18	4.08E-18	2.80E-18
1.01E-17	1.09E-17	1.15E-17	1.21E-17	8.14E-18	9.13E-18	5.09E-18	4.61E-18
1.09E-17	1.12E-17	9.79E-18	1.12E-17	1.14E-17	1.31E-17	4.92E-18	6.17E-18
1.44E-17	1.54E-17	1.29E-17	9.07E-18	1.27E-17	1.90E-17	6.79E-18	7.92E-18
2.12E-17	1.99E-17	1.47E-17	1.06E-17	1.38E-17	1.92E-17	8.07E-18	9.99E-18
3.09E-17	2.17E-17	1.96E-17	1.43E-17	1.46E-17	1.81E-17	1.08E-17	1.11E-17
3.51E-17	2.47E-17	2.29E-17	1.84E-17	1.34E-17	2.21E-17	1.54E-17	1.32E-17
3.67E-17	2.84E-17	2.95E-17	2.36E-17	2.04E-17	2.70E-17	1.74E-17	1.66E-17
4.21E-17	3.24E-17	3.71E-17	3.31E-17	2.62E-17	2.67E-17	2.06E-17	2.24E-17
5.07E-17	3.65E-17	4.62E-17	4.22E-17	2.76E-17	2.71E-17	3.05E-17	2.64E-17
6.16E-17	4.59E-17	5.21E-17	4.18E-17	3.16E-17	3.53E-17	4.27E-17	3.57E-17
6.64E-17	5.69E-17	5.47E-17	4.61E-17	3.41E-17	4.55E-17	5.10E-17	4.21E-17
7.21E-17	7.06E-17	5.72E-17	4.95E-17	4.13E-17	5.74E-17	5.53E-17	4.89E-17
7.62E-17	7.86E-17	6.12E-17	5.61E-17	5.38E-17	6.15E-17	6.26E-17	5.85E-17
8.05E-17	8.93E-17	6.37E-17	6.41E-17	6.29E-17	6.75E-17	7.47E-17	6.80E-17
8.22E-17	9.82E-17	7.50E-17	7.52E-17	7.95E-17	8.31E-17	7.16E-17	7.77E-17
9.02E-17	1.12E-16	9.14E-17	8.75E-17	9.60E-17	9.50E-17	7.03E-17	9.16E-17
1.09E-16	1.21E-16	1.08E-16	1.06E-16	1.02E-16	1.11E-16	8.50E-17	9.77E-17
1.28E-16	1.31E-16	1.25E-16	1.27E-16	1.15E-16	1.22E-16	9.50E-17	9.88E-17
1.42E-16	1.51E-16	1.36E-16	1.44E-16	1.27E-16	1.33E-16	1.04E-16	1.11E-16
1.67E-16	1.68E-16	1.44E-16	1.49E-16	1.42E-16	1.48E-16	1.30E-16	1.24E-16
1.95E-16	1.86E-16	1.57E-16	1.58E-16	1.58E-16	1.65E-16	1.54E-16	1.44E-16
2.18E-16	2.02E-16	1.81E-16	1.78E-16	1.68E-16	1.76E-16	1.68E-16	1.77E-16
2.42E-16	2.16E-16	2.03E-16	1.99E-16	1.86E-16	1.85E-16	1.83E-16	1.87E-16
2.58E-16	2.31E-16	2.28E-16	2.24E-16	2.14E-16	2.01E-16	1.86E-16	1.89E-16
2.69E-16	2.55E-16	2.50E-16	2.45E-16	2.37E-16	2.22E-16	2.03E-16	2.06E-16
2.87E-16	2.82E-16	2.71E-16	2.72E-16	2.60E-16	2.48E-16	2.35E-16	2.25E-16
3.15E-16	3.05E-16	3.00E-16	3.00E-16	2.91E-16	2.73E-16	2.68E-16	2.58E-16
3.42E-16	3.22E-16	3.31E-16	3.17E-16	3.22E-16	2.93E-16	3.00E-16	2.84E-16
3.63E-16	3.43E-16	3.52E-16	3.38E-16	3.51E-16	3.30E-16	3.42E-16	3.03E-16
3.76E-16	3.65E-16	3.75E-16	3.74E-16	3.77E-16	3.63E-16	3.83E-16	3.41E-16
4.20E-16	3.95E-16	4.02E-16	3.87E-16	4.04E-16	3.89E-16	4.20E-16	3.79E-16
4.52E-16	4.30E-16	4.25E-16	4.28E-16	4.19E-16	4.19E-16	4.51E-16	4.30E-16

4.78E-16	4.52E-16	4.60E-16	4.78E-16	4.41E-16	4.44E-16	4.93E-16	4.78E-16
5.03E-16	4.76E-16	4.82E-16	5.12E-16	4.76E-16	4.90E-16	5.30E-16	5.37E-16
5.48E-16	5.05E-16	5.07E-16	5.28E-16	5.24E-16	5.30E-16	5.68E-16	5.61E-16
5.96E-16	5.54E-16	5.50E-16	5.84E-16	5.79E-16	5.60E-16	6.01E-16	5.86E-16
6.44E-16	6.23E-16	6.05E-16	6.24E-16	6.12E-16	5.99E-16	6.23E-16	6.14E-16
7.00E-16	6.68E-16	6.78E-16	6.79E-16	6.50E-16	6.43E-16	6.50E-16	6.59E-16
7.41E-16	7.22E-16	7.33E-16	7.31E-16	7.03E-16	7.12E-16	6.83E-16	7.17E-16
8.02E-16	7.69E-16	7.90E-16	7.86E-16	7.47E-16	7.69E-16	7.37E-16	7.68E-16
8.51E-16	8.23E-16	8.66E-16	8.45E-16	8.04E-16	8.31E-16	8.10E-16	8.18E-16
8.97E-16	8.77E-16	9.33E-16	8.99E-16	8.55E-16	8.94E-16	8.67E-16	8.60E-16
9.44E-16	9.74E-16	9.47E-16	9.60E-16	9.32E-16	9.07E-16	9.15E-16	8.94E-16
1.67E-14	1.60E-14	1.58E-14	1.57E-14	1.53E-14	1.53E-14	1.50E-14	1.51E-14

-9.03E-09	-8.54E-09	-8.69E-09	-8.72E-09	-8.69E-09	-8.28E-09	-7.63E-09	-6.88E-09	-7.23E-09	-7.45E-09	-6.92E-09	-6.84E-09	-7.44E-09	-8.12E-09
-9.12E-09	-8.85E-09	-8.66E-09	-8.77E-09	-7.92E-09	-8.20E-09	-7.77E-09	-7.49E-09	-7.06E-09	-7.40E-09	-7.58E-09	-7.22E-09	-7.52E-09	-8.04E-09
-8.86E-09	-9.32E-09	-8.37E-09	-8.58E-09	-7.61E-09	-7.90E-09	-7.43E-09	-8.28E-09	-7.36E-09	-7.37E-09	-7.59E-09	-7.28E-09	-7.34E-09	-7.77E-09
-8.60E-09	-9.37E-09	-8.39E-09	-8.58E-09	-7.69E-09	-7.39E-09	-7.28E-09	-8.39E-09	-7.81E-09	-7.42E-09	-6.94E-09	-7.27E-09	-6.65E-09	-7.28E-09
-8.66E-09	-9.29E-09	-8.51E-09	-8.96E-09	-8.70E-09	-7.39E-09	-7.29E-09	-7.55E-09	-7.65E-09	-7.13E-09	-6.09E-09	-6.79E-09	-6.21E-09	-6.58E-09
-8.87E-09	-8.98E-09	-8.57E-09	-9.29E-09	-9.04E-09	-7.84E-09	-7.44E-09	-7.06E-09	-7.23E-09	-6.74E-09	-6.19E-09	-6.22E-09	-5.56E-09	-5.96E-09
-9.11E-09	-8.26E-09	-8.00E-09	-9.15E-09	-9.19E-09	-8.08E-09	-7.38E-09	-6.96E-09	-6.74E-09	-6.66E-09	-6.44E-09	-6.42E-09	-5.66E-09	-6.14E-09
-9.21E-09	-7.67E-09	-7.85E-09	-8.87E-09	-8.85E-09	-7.94E-09	-7.37E-09	-7.12E-09	-6.78E-09	-7.03E-09	-6.36E-09	-7.43E-09	-5.94E-09	-6.53E-09
-9.30E-09	-7.76E-09	-8.48E-09	-8.55E-09	-8.36E-09	-7.64E-09	-7.45E-09	-7.15E-09	-7.10E-09	-7.07E-09	-6.18E-09	-7.48E-09	-6.75E-09	-7.08E-09
-9.00E-09	-7.64E-09	-8.42E-09	-8.34E-09	-7.73E-09	-7.45E-09	-7.62E-09	-7.33E-09	-7.14E-09	-7.35E-09	-6.51E-09	-7.39E-09	-7.20E-09	-7.38E-09
-8.43E-09	-8.15E-09	-8.15E-09	-7.79E-09	-7.81E-09	-7.02E-09	-7.34E-09	-6.97E-09	-6.93E-09	-7.31E-09	-6.63E-09	-7.38E-09	-7.08E-09	-7.35E-09
-7.72E-09	-7.91E-09	-7.57E-09	-6.97E-09	-7.62E-09	-6.82E-09	-6.88E-09	-7.01E-09	-6.75E-09	-7.45E-09	-6.95E-09	-7.11E-09	-7.04E-09	-7.47E-09
-7.40E-09	-7.80E-09	-6.96E-09	-6.61E-09	-7.72E-09	-7.24E-09	-6.85E-09	-6.96E-09	-6.69E-09	-7.50E-09	-7.02E-09	-6.59E-09	-7.27E-09	-8.12E-09
-7.29E-09	-7.63E-09	-6.92E-09	-7.28E-09	-8.10E-09	-7.41E-09	-6.77E-09	-6.91E-09	-6.95E-09	-7.62E-09	-7.11E-09	-6.09E-09	-6.95E-09	-8.08E-09
-7.05E-09	-7.55E-09	-7.10E-09	-7.78E-09	-8.07E-09	-7.25E-09	-6.69E-09	-6.64E-09	-6.81E-09	-7.64E-09	-6.45E-09	-5.76E-09	-6.64E-09	-7.65E-09
-7.15E-09	-7.76E-09	-7.52E-09	-7.67E-09	-7.69E-09	-6.75E-09	-6.64E-09	-6.60E-09	-6.87E-09	-6.93E-09	-6.13E-09	-5.81E-09	-6.83E-09	-7.39E-09
-7.26E-09	-7.70E-09	-7.74E-09	-7.35E-09	-6.96E-09	-6.76E-09	-6.83E-09	-6.77E-09	-7.12E-09	-6.84E-09	-6.39E-09	-5.81E-09	-6.90E-09	-6.85E-09
-7.09E-09	-7.24E-09	-7.34E-09	-6.94E-09	-6.57E-09	-6.94E-09	-6.91E-09	-6.93E-09	-6.99E-09	-6.84E-09	-6.92E-09	-6.26E-09	-6.31E-09	-6.34E-09
-6.65E-09	-6.54E-09	-6.86E-09	-6.66E-09	-6.77E-09	-6.98E-09	-7.18E-09	-7.19E-09	-7.00E-09	-6.76E-09	-7.06E-09	-6.27E-09	-6.11E-09	-6.18E-09
-6.10E-09	-6.16E-09	-6.45E-09	-6.66E-09	-7.06E-09	-7.01E-09	-7.19E-09	-7.19E-09	-7.20E-09	-6.80E-09	-6.96E-09	-6.40E-09	-6.38E-09	-6.08E-09
-5.65E-09	-5.87E-09	-6.45E-09	-7.09E-09	-7.02E-09	-7.08E-09	-6.93E-09	-6.60E-09	-7.31E-09	-7.03E-09	-6.99E-09	-6.83E-09	-6.69E-09	-6.17E-09
-5.60E-09	-6.09E-09	-6.88E-09	-7.42E-09	-7.67E-09	-7.53E-09	-6.89E-09	-6.21E-09	-6.95E-09	-6.69E-09	-6.82E-09	-7.15E-09	-7.00E-09	-6.28E-09
-6.54E-09	-6.72E-09	-7.43E-09	-7.59E-09	-7.84E-09	-7.74E-09	-6.76E-09	-6.31E-09	-7.08E-09	-6.31E-09	-6.73E-09	-7.25E-09	-6.88E-09	-6.36E-09
-7.18E-09	-6.93E-09	-7.53E-09	-7.46E-09	-7.33E-09	-7.78E-09	-6.85E-09	-6.80E-09	-6.71E-09	-6.02E-09	-6.28E-09	-7.23E-09	-6.81E-09	-6.52E-09
-7.40E-09	-6.92E-09	-7.15E-09	-7.04E-09	-7.16E-09	-7.94E-09	-7.14E-09	-6.73E-09	-6.21E-09	-5.97E-09	-5.77E-09	-7.12E-09	-7.19E-09	-6.97E-09
-7.41E-09	-6.87E-09	-6.83E-09	-6.76E-09	-7.17E-09	-7.87E-09	-7.21E-09	-6.64E-09	-5.48E-09	-5.72E-09	-5.64E-09	-7.01E-09	-7.56E-09	-7.61E-09
-7.56E-09	-6.96E-09	-6.53E-09	-6.54E-09	-7.07E-09	-7.57E-09	-6.99E-09	-6.63E-09	-5.09E-09	-5.73E-09	-5.77E-09	-6.72E-09	-7.95E-09	-7.82E-09
-7.48E-09	-6.82E-09	-6.10E-09	-6.17E-09	-7.11E-09	-7.20E-09	-6.86E-09	-7.05E-09	-5.52E-09	-6.02E-09	-6.41E-09	-6.99E-09	-7.80E-09	-7.65E-09
-7.46E-09	-6.49E-09	-5.88E-09	-6.25E-09	-7.29E-09	-6.98E-09	-6.88E-09	-6.96E-09	-5.97E-09	-6.53E-09	-6.86E-09	-7.49E-09	-7.58E-09	-7.91E-09
-7.24E-09	-6.32E-09	-6.26E-09	-6.69E-09	-7.75E-09	-7.34E-09	-6.76E-09	-6.97E-09	-6.07E-09	-6.86E-09	-6.72E-09	-7.59E-09	-7.35E-09	-8.16E-09
-6.93E-09	-6.29E-09	-6.41E-09	-6.77E-09	-7.87E-09	-7.26E-09	-6.57E-09	-7.05E-09	-6.74E-09	-7.24E-09	-6.60E-09	-7.63E-09	-7.39E-09	-8.39E-09
-6.78E-09	-6.19E-09	-6.64E-09	-6.86E-09	-7.81E-09	-7.21E-09	-6.70E-09	-6.91E-09	-7.65E-09	-7.83E-09	-7.19E-09	-7.95E-09	-7.48E-09	-8.25E-09
-6.95E-09	-6.11E-09	-6.75E-09	-6.76E-09	-7.69E-09	-6.99E-09	-7.00E-09	-6.97E-09	-7.75E-09	-8.05E-09	-7.89E-09	-8.18E-09	-7.79E-09	-8.08E-09
-6.88E-09	-6.38E-09	-6.76E-09	-6.58E-09	-7.34E-09	-6.96E-09	-7.22E-09	-6.94E-09	-7.46E-09	-7.91E-09	-8.19E-09	-7.86E-09	-7.84E-09	-7.56E-09
-6.91E-09	-6.46E-09	-6.58E-09	-6.65E-09	-7.19E-09	-7.14E-09	-7.07E-09	-6.84E-09	-6.93E-09	-7.73E-09	-8.26E-09	-7.60E-09	-7.60E-09	-7.06E-09
-7.05E-09	-6.71E-09	-6.44E-09	-6.62E-09	-6.82E-09	-7.13E-09	-7.09E-09	-6.88E-09	-6.44E-09	-7.37E-09	-7.90E-09	-7.58E-09	-7.79E-09	-6.70E-09
-6.88E-09	-6.58E-09	-6.52E-09	-6.62E-09	-6.71E-09	-6.87E-09	-6.75E-09	-6.91E-09	-6.50E-09	-6.84E-09	-7.39E-09	-7.52E-09	-7.92E-09	-7.25E-09
-6.80E-09	-6.51E-09	-6.73E-09	-6.95E-09	-6.80E-09	-6.84E-09	-6.26E-09	-6.69E-09	-6.56E-09	-6.58E-09	-7.11E-09	-7.51E-09	-7.89E-09	-7.93E-09
-6.30E-09	-6.36E-09	-6.95E-09	-7.14E-09	-6.71E-09	-6.38E-09	-6.48E-09	-7.07E-09	-6.86E-09	-6.24E-09	-7.02E-09	-7.65E-09	-7.67E-09	-7.77E-09
-5.92E-09	-6.51E-09	-6.86E-09	-7.01E-09	-6.75E-09	-6.27E-09	-6.64E-09	-7.13E-09	-7.03E-09	-6.42E-09	-7.13E-09	-7.55E-09	-7.60E-09	-7.58E-09
-6.31E-09	-6.82E-09	-6.80E-09	-6.76E-09	-7.04E-09	-6.46E-09	-6.72E-09	-6.58E-09	-7.24E-09	-6.37E-09	-7.30E-09	-7.53E-09	-7.67E-09	-7.78E-09
-6.44E-09	-7.10E-09	-6.48E-09	-6.30E-09	-6.84E-09	-6.52E-09	-6.55E-09	-6.20E-09	-7.09E-09	-6.78E-09	-6.88E-09	-6.84E-09	-7.57E-09	-7.84E-09
-6.89E-09	-7.10E-09	-6.29E-09	-6.04E-09	-6.66E-09	-6.48E-09	-6.99E-09	-6.55E-09	-6.85E-09	-6.75E-09	-6.59E-09	-6.60E-09	-7.03E-09	-7.75E-09
-7.03E-09	-6.90E-09	-6.08E-09	-6.10E-09	-6.80E-09	-6.51E-09	-7.78E-09	-6.55E-09	-6.54E-09	-6.55E-09	-6.62E-09	-6.73E-09	-6.44E-09	-7.17E-09
-6.85E-09	-6.68E-09	-6.00E-09	-6.39E-09	-6.51E-09	-6.52E-09	-7.47E-09	-6.05E-09	-6.22E-09	-6.16E-09	-6.56E-09	-6.79E-09	-6.03E-09	-6.27E-09
-6.44E-09	-6.46E-09	-5.94E-09	-6.53E-09	-6.14E-09	-6.49E-09	-6.76E-09	-5.68E-09	-5.69E-09	-5.63E-09	-6.11E-09	-6.59E-09	-6.05E-09	-5.48E-09
-6.28E-09	-6.32E-09	-6.37E-09	-6.18E-09	-6.16E-09	-6.28E-09	-6.21E-09	-5.78E-09	-5.43E-09	-5.41E-09	-5.62E-09	-6.52E-09	-6.23E-09	-5.21E-09
-6.25E-09	-6.57E-09	-6.51E-09	-6.04E-09	-6.29E-09	-6.35E-09	-6.47E-09	-5.90E-09	-5.32E-09	-5.99E-09	-5.81E-09	-6.69E-09	-6.34E-09	-5.75E-09
-6.83E-09	-6.85E-09	-6.64E-09	-6.26E-09	-6.48E-09	-6.16E-09	-6.53E-09	-5.99E-09	-5.39E-09	-6.27E-09	-5.91E-09	-6.63E-09	-6.60E-09	-5.84E-09
-6.84E-09	-6.97E-09	-6.95E-09	-6.56E-09	-6.12E-09	-5.98E-09	-6.21E-09	-5.74E-09	-5.60E-09	-6.53E-09	-6.35E-09	-6.65E-09	-6.63E-09	-5.98E-09
-6.69E-09	-7.14E-09	-7.14E-09	-6.55E-09	-5.87E-09	-5.63E-09	-5.88E-09	-5.82E-09	-6.06E-09	-6.27E-09	-6.42E-09	-6.71E-09	-6.80E-09	-6.00E-09
-6.80E-09	-6.91E-09	-6.98E-09	-6.18E-09	-5.34E-09	-5.34E-09	-5.07E-09	-5.81E-09	-6.09E-09	-6.22E-09	-6.06E-09	-6.41E-09	-6.67E-09	-6.12E-09
-6.86E-09	-6.54E-09	-6.31E-09	-5.82E-09	-4.75E-09	-5.11E-09	-4.46E-09	-5.84E-09	-5.83E-09	-5.75E-09	-5.84E-09	-6.08E-09	-6.50E-09	-6.28E-09
-6.73E-09	-6.20E-09	-6.52E-09	-6.39E-09	-5.03E-09	-4.79E-09	-3.54E-09	-5.61E-09	-6.04E-09	-5.61E-09	-5.85E-09	-5.94E-09	-6.37E-09	-5.99E-09
-6.45E-09	-6.21E-09	-6.72E-09	-6.36E-09	-5.33E-09	-4.66E-09	-2.96E-09	-5.02E-09	-5.69E-09	-5.15E-09	-5.77E-09	-5.76E-09	-5.89E-09	-6.05E-09
-6.33E-09	-6.09E-09	-6.73E-09	-5.71E-09	-5.42E-09	-4.92E-09	-2.48E-09	-5.03E-09	-5.31E-09	-4.61E-09	-5.30E-09	-5.69E-09	-5.35E-09	-5.95E-09
-6.02E-09	-6.03E-09	-6.27E-09	-5.22E-09	-5.66E-09	-5.19E-09	-2.99E-09	-5.08E-09	-5.13E-09	-4.44E-09	-5.08E-09	-5.41E-09	-5.84E-09	-5.68E-09
-5.75E-09	-5.61E-09	-5.47E-09	-5.20E-09	-5.57E-09	-5.43E-09	-4.26E-09	-5.21E-09	-4.85E-09	-5.03E-09	-5.56E-09	-5.15E-09	-5.66E-09	-5.56E-09
-5.57E-09	-5.10E-09	-5.13E-09	-5.23E-09	-5.58E-09	-5.35E-09	-4.81E-09	-5.21E-09	-4.80E-09	-5.41E-09	-5.73E-09	-5.01E-09	-5.48E-09	-5.85E-09
-5.41E-09	-4.66E-09	-4.92E-09	-5.15E-09	-5.69E-09	-5.13E-09	-4.98E-09	-5.39E-09	-5.11E-09	-5.83E-09	-5.60E-09	-5.61E-09	-5.32E-09	-5.48E-09
-5.26E-09	-4.79E-09	-4.68E-09	-4.90E-09	-5.38E-09	-4.83E-09	-5.03E-09	-5.13E-09	-4.93E-09	-5.69E-09	-5.64E-09	-6.05E-09	-5.14E-09	-5.28E-09
-4.96E-09	-4.93E-09	-4.42E-09	-4.60E-09	-4.92E-09	-4.60E-09	-4.83E-09	-5.43E-09	-4.64E-09	-5.42E-09	-5.39E-09	-5.89E-09	-5.28E-09	-5.57E-09
-5.01E-09	-4.82E-09	-4.84E-09	-3.99E-09	-4.92E-09	-5.02E-09	-4.57E-09	-5.67E-09	-4.73E-09	-5.27E-09	-5.22E-09	-5.63E-09	-5.23E-09	-5.73E-09
-4.28E-09	-4.32E-09	-4.50E-09	-3.58E-09	-4.68E-09	-4.70E-09	-4.39E-09	-5.67E-09	-5.26E-09	-5.15E-09	-5.37E-09	-4.82E-09	-5.23E-09	-5.84E-09
-3.56E-09	-3.63E-09	-4.08E-09	-3.43E-09	-4.51E-09	-4.18E-09	-4.06E-09	-5.25E-09	-5.28E-09	-4.84E-09	-5.19E-09	-4.20E-09	-5.25E-09	-5.51E-09
-2.96E-09	-3.23E-09	-3.43E-09	-3.92E-09	-4.39E-09	-3.91E-09	-3.84E-09	-4.90E-09	-4.78E-09	-4.45E-09	-4.59E-09	-4.75E-09	-5.23E-09	-5.41E-09
-3.00E-09	-3.35E-09	-2.88E-09	-3.77E-09	-4.06E-09	-4.19E-09	-3.82E-09	-4.73E-09	-4.49E-09	-3.99E-09	-4.24E-09	-5.08E-09	-5.24E-09	-4.85E-09
-2.97E-09	-3.41E-09	-2.95E-09	-3.67E-09	-3.60E-09	-4.06E-09	-4.23E-09	-4.86E-09	-4.65E-09	-3.67E-09	-3.87E-09	-5.22E-09	-5.13E-09	-5.20E-09
-2.50E-09	-3.14E-09	-2.95E-09	-3.70E-09	-3.29E-09	-3.77E-09	-3.84E-09	-4.71E-09	-4.41E-09	-3.62E-09	-3.94E-09	-5.12E-09	-4.85E-09	-5.41E-09
-2.23E-09	-3.03E-09	-2.48E-09	-3.00E-09	-3.08E-09	-3.52E-09	-3.55E-09	-4.13E-09	-4.29E-09	-3.65E-09	-4.13E-09	-5.11E-09	-4.76E-09	-5.08E-09
-2.46E-09	-2.94E-09	-1.94E-09	-2.49E-09	-2.65E-09	-3.30E-09	-3.35E-09	-3.83E-09	-4.09E-09	-3.87E-09	-4.45E-09	-4.46E-09	-4.57E-09	-4.98E-09
-2.40E-09	-2.66E-09	-1.96E-09	-1.87E-09	-2.40E-09	-3.08E-09	-2.88E-09	-3.24E-09	-3.71E-09	-4.23E-09	-4.39E-09	-3.70E-09	-3.93E-09	-4.82E-09
-2.54E-09	-2.64E-09	-2.15E-09	-1.42E-09	-2.24E-09	-2.81E-09	-2.92E-09	-2.86E-09	-3.51E-09	-3.97E-09	-4.19E-09	-3.31E-09	-3.52E-09	-4.32E-09
-2.66E-09	-2.59E-09	-2.08E-09	-9.68E-10	-2.18E-09	-2.34E-09	-2.46E-09	-2.44E-09	-2.94E-09	-3.50E-09	-3.63E-09	-3.02E-09	-3.58E-09	-4.11E-09
-2.54E-09	-2.39E-09	-2.24E-09	-1.20E-09	-2.13E-09	-1.78E-09	-1.90E-09	-2.07E-09	-2.68E-09	-3.15E-09	-3.81E-09	-3.23E-09	-3.57E-09	-3.79E-09
-2.06E-09	-1.75E-09	-1.76E-09	-1.34E-09	-2.30E-09	-1.49E-09	-1.77E-09	-1.94E-09	-2.92E-09	-2.98E-09	-3.59E-09	-3.20E-09	-3.33E-09	-3.75E-09
-1.58E-09	-1.43E-09	-1.32E-09	-1.42E-09	-2.07E-09	-1.32E-09	-1.78E-09	-2.29E-09	-3.29E-09	-2.72E-09	-3.15E-09	-2.72E-09	-2.80E-09	-3.81E-09
-7.90E-10	-1.26E-09	-1.26E-09	-1.16E-09	-1.55E-09	-5.25E-10	-1.35E-09	-2.13E-09	-2.83E-09	-1.91E-09	-2.70E-09	-1.89E-09	-2.12E-09	-3.25E-09

2.17E-11	-1.03E-09	-7.82E-10	-1.08E-09	-1.14E-09	-8.74E-10	-8.17E-10	-1.75E-09	-1.61E-09	-1.24E-09	-2.76E-09	-2.05E-09	-1.78E-09	-2.90E-09
5.25E-10	-4.53E-10	-2.51E-10	-6.09E-10	-7.08E-10	-2.81E-11	-2.14E-10	-1.31E-09	-7.03E-10	-1.43E-09	-2.59E-09	-2.23E-09	-1.55E-09	-2.53E-09
4.93E-10	1.40E-10	2.03E-10	-4.99E-10	-2.11E-10	1.02E-09	2.24E-10	-9.22E-10	-4.47E-10	-1.53E-09	-1.97E-09	-1.34E-09	-9.88E-10	-2.19E-09
1.08E-09	7.76E-10	9.31E-10	5.52E-11	3.50E-10	1.26E-09	3.02E-10	-3.45E-10	-6.80E-11	-1.19E-09	-1.57E-09	-7.46E-10	-7.87E-10	-1.67E-09
1.33E-09	1.07E-09	1.28E-09	4.71E-10	6.74E-10	7.43E-10	4.20E-10	9.79E-11	3.51E-10	-6.89E-10	-1.31E-09	-4.40E-10	-6.90E-10	-1.03E-09
1.59E-09	1.66E-09	1.00E-09	5.34E-10	5.85E-10	-1.53E-10	3.01E-10	4.44E-10	5.60E-10	-9.00E-11	-6.94E-10	-6.00E-10	-7.95E-10	-7.76E-10
1.87E-09	1.81E-09	7.39E-10	8.67E-10	5.90E-10	3.90E-11	4.57E-10	5.99E-10	3.78E-10	5.85E-10	9.90E-11	-7.74E-10	-8.87E-10	-7.39E-10
2.21E-09	2.06E-09	1.07E-09	1.72E-09	1.19E-09	8.48E-10	6.40E-10	4.70E-10	9.53E-10	1.49E-09	5.51E-10	-1.61E-11	-4.59E-10	-4.57E-10
2.69E-09	2.59E-09	1.97E-09	2.48E-09	2.22E-09	1.84E-09	1.06E-09	9.44E-10	9.46E-10	1.53E-09	9.58E-10	8.84E-10	-4.85E-11	-6.81E-11
2.89E-09	2.92E-09	2.84E-09	2.78E-09	2.77E-09	2.41E-09	1.71E-09	1.75E-09	9.14E-10	8.50E-10	1.50E-09	1.38E-09	4.21E-10	5.30E-10
3.14E-09	3.06E-09	2.80E-09	3.07E-09	2.50E-09	2.56E-09	1.30E-09	2.19E-09	1.31E-09	8.25E-10	1.45E-09	1.43E-09	9.47E-10	7.08E-10
3.45E-09	3.44E-09	2.70E-09	3.10E-09	2.14E-09	2.69E-09	1.34E-09	2.43E-09	1.58E-09	1.27E-09	1.48E-09	1.48E-09	1.20E-09	1.01E-09
3.77E-09	4.21E-09	2.93E-09	3.14E-09	2.16E-09	2.55E-09	1.86E-09	2.71E-09	2.49E-09	1.98E-09	1.63E-09	1.46E-09	1.21E-09	1.10E-09
4.53E-09	4.75E-09	3.80E-09	3.54E-09	2.69E-09	2.83E-09	2.33E-09	2.80E-09	2.88E-09	2.56E-09	2.12E-09	1.47E-09	1.37E-09	1.35E-09
4.90E-09	4.78E-09	4.67E-09	4.57E-09	3.25E-09	3.52E-09	2.96E-09	3.60E-09	3.37E-09	3.22E-09	2.63E-09	2.23E-09	1.33E-09	1.81E-09
5.07E-09	5.13E-09	5.53E-09	5.05E-09	4.37E-09	4.36E-09	3.54E-09	4.42E-09	3.65E-09	3.28E-09	3.10E-09	2.99E-09	2.20E-09	2.47E-09
5.79E-09	6.06E-09	6.11E-09	6.23E-09	5.54E-09	4.96E-09	4.22E-09	4.67E-09	3.99E-09	3.17E-09	3.48E-09	3.54E-09	3.15E-09	3.03E-09
6.50E-09	6.52E-09	6.75E-09	6.55E-09	6.16E-09	5.38E-09	4.50E-09	4.89E-09	4.35E-09	3.50E-09	3.65E-09	3.85E-09	3.06E-09	3.33E-09
6.83E-09	7.05E-09	7.12E-09	6.09E-09	7.11E-09	5.83E-09	5.26E-09	5.91E-09	5.43E-09	4.12E-09	4.38E-09	4.07E-09	3.07E-09	3.86E-09
7.25E-09	7.36E-09	7.33E-09	6.20E-09	9.54E-09	6.36E-09	6.34E-09	6.15E-09	6.19E-09	4.96E-09	5.00E-09	4.27E-09	3.58E-09	4.55E-09
8.09E-09	7.77E-09	8.18E-09	6.59E-09	1.36E-08	7.22E-09	6.92E-09	5.94E-09	6.71E-09	5.79E-09	5.70E-09	4.79E-09	4.64E-09	5.08E-09
9.16E-09	8.53E-09	8.52E-09	7.19E-09	1.70E-08	8.05E-09	7.31E-09	6.20E-09	7.42E-09	6.49E-09	6.24E-09	5.38E-09	5.75E-09	5.67E-09
9.49E-09	9.22E-09	8.65E-09	7.76E-09	9.71E-09	9.02E-09	7.98E-09	6.70E-09	6.27E-09	7.11E-09	6.87E-09	6.02E-09	6.75E-09	6.39E-09
9.70E-09	1.01E-08	9.26E-09	8.48E-09	8.58E-09	9.16E-09	8.74E-09	7.52E-09	7.07E-09	7.20E-09	7.44E-09	6.80E-09	7.34E-09	6.92E-09
9.94E-09	1.09E-08	9.45E-09	9.31E-09	9.16E-09	9.08E-09	9.01E-09	8.21E-09	7.90E-09	7.81E-09	8.19E-09	8.04E-09	7.90E-09	7.69E-09
1.07E-08	1.13E-08	1.01E-08	1.01E-08	9.62E-09	9.22E-09	9.23E-09	8.75E-09	8.46E-09	8.47E-09	9.01E-09	9.02E-09	8.52E-09	8.41E-09
1.14E-08	1.14E-08	1.14E-08	1.12E-08	1.05E-08	1.00E-08	9.80E-09	9.88E-09	8.96E-09	9.45E-09	9.73E-09	9.83E-09	9.35E-09	9.46E-09
1.24E-08	1.21E-08	1.20E-08	1.22E-08	1.13E-08	1.02E-08	1.08E-08	1.06E-08	9.81E-09	1.06E-08	1.02E-08	1.03E-08	1.03E-08	1.04E-08
1.32E-08	1.25E-08	1.24E-08	1.33E-08	1.21E-08	1.08E-08	1.15E-08	1.13E-08	1.09E-08	1.12E-08	1.07E-08	1.11E-08	1.13E-08	1.09E-08
1.36E-08	1.30E-08	1.30E-08	1.42E-08	1.28E-08	1.18E-08	1.22E-08	1.20E-08	1.18E-08	1.16E-08	1.17E-08	1.23E-08	1.22E-08	1.17E-08
1.38E-08	1.40E-08	1.38E-08	1.43E-08	1.32E-08	1.28E-08	1.25E-08	1.20E-08	1.25E-08	1.27E-08	1.31E-08	1.35E-08	1.34E-08	1.30E-08
1.44E-08	1.48E-08	1.43E-08	1.45E-08	1.42E-08	1.36E-08	1.32E-08	1.27E-08	1.36E-08	1.43E-08	1.44E-08	1.35E-08	1.36E-08	1.42E-08
1.53E-08	1.56E-08	1.50E-08	1.53E-08	1.54E-08	1.47E-08	1.37E-08	1.37E-08	1.48E-08	1.53E-08	1.50E-08	1.42E-08	1.43E-08	1.40E-08
1.60E-08	1.68E-08	1.58E-08	1.62E-08	1.63E-08	1.59E-08	1.46E-08	1.46E-08	1.58E-08	1.53E-08	1.55E-08	1.54E-08	1.49E-08	1.34E-08
1.64E-08	1.77E-08	1.66E-08	1.66E-08	1.71E-08	1.78E-08	1.55E-08	1.55E-08	1.64E-08	1.57E-08	1.66E-08	1.59E-08	1.50E-08	1.38E-08
1.72E-08	1.79E-08	1.73E-08	1.72E-08	1.79E-08	1.87E-08	1.62E-08	1.59E-08	1.66E-08	1.66E-08	1.71E-08	1.71E-08	1.59E-08	1.49E-08
1.82E-08	1.82E-08	1.78E-08	1.80E-08	1.83E-08	1.81E-08	1.70E-08	1.70E-08	1.74E-08	1.74E-08	1.75E-08	1.71E-08	1.72E-08	1.62E-08
1.90E-08	1.84E-08	1.85E-08	1.90E-08	1.87E-08	1.87E-08	1.78E-08	1.79E-08	1.82E-08	1.81E-08	1.78E-08	1.76E-08	1.85E-08	1.76E-08
1.96E-08	1.91E-08	1.96E-08	1.99E-08	1.93E-08	1.93E-08	1.85E-08	1.88E-08	1.88E-08	1.83E-08	1.84E-08	1.85E-08	1.96E-08	1.90E-08
2.06E-08	2.01E-08	2.05E-08	2.02E-08	1.97E-08	2.01E-08	1.93E-08	1.98E-08	1.95E-08	1.87E-08	1.89E-08	1.94E-08	1.99E-08	1.95E-08
2.16E-08	2.09E-08	2.15E-08	2.07E-08	2.04E-08	2.07E-08	2.02E-08	2.07E-08	2.01E-08	1.93E-08	1.96E-08	1.98E-08	2.01E-08	1.98E-08
2.24E-08	2.17E-08	2.21E-08	2.16E-08	2.12E-08	2.13E-08	2.13E-08	2.17E-08	2.12E-08	2.07E-08	2.10E-08	2.07E-08	2.05E-08	2.08E-08
2.31E-08	2.26E-08	2.26E-08	2.24E-08	2.23E-08	2.21E-08	2.22E-08	2.29E-08	2.26E-08	2.20E-08	2.27E-08	2.19E-08	2.14E-08	2.16E-08
2.41E-08	2.34E-08	2.32E-08	2.33E-08	2.31E-08	2.30E-08	2.37E-08	2.41E-08	2.39E-08	2.35E-08	2.38E-08	2.31E-08	2.25E-08	2.24E-08
2.54E-08	2.45E-08	2.44E-08	2.42E-08	2.36E-08	2.39E-08	2.51E-08	2.54E-08	2.51E-08	2.46E-08	2.41E-08	2.42E-08	2.39E-08	2.31E-08
2.65E-08	2.57E-08	2.58E-08	2.56E-08	2.43E-08	2.48E-08	2.56E-08	2.58E-08	2.56E-08	2.58E-08	2.46E-08	2.56E-08	2.52E-08	2.39E-08
2.75E-08	2.66E-08	2.66E-08	2.66E-08	2.53E-08	2.57E-08	2.65E-08	2.61E-08	2.62E-08	2.63E-08	2.57E-08	2.74E-08	2.63E-08	2.55E-08
2.82E-08	2.75E-08	2.73E-08	2.75E-08	2.63E-08	2.65E-08	2.75E-08	2.70E-08	2.71E-08	2.69E-08	2.70E-08	2.73E-08	2.70E-08	2.69E-08
2.90E-08	2.85E-08	2.84E-08	2.85E-08	2.75E-08	2.78E-08	2.79E-08	2.81E-08	2.80E-08	2.79E-08	2.81E-08	2.79E-08	2.84E-08	2.84E-08
2.97E-08	2.97E-08	2.96E-08	2.94E-08	2.88E-08	2.92E-08	2.93E-08	2.93E-08	2.91E-08	2.88E-08	2.92E-08	2.93E-08	2.98E-08	2.98E-08

8.16E-17	7.29E-17	7.55E-17	7.60E-17	7.55E-17	6.86E-17	5.82E-17	4.74E-17	5.22E-17	5.54E-17	4.79E-17	4.68E-17	5.54E-17	6.59E-17
8.33E-17	7.84E-17	7.50E-17	7.70E-17	6.27E-17	6.72E-17	6.03E-17	5.61E-17	4.98E-17	5.48E-17	5.74E-17	5.21E-17	5.66E-17	6.46E-17
7.85E-17	8.69E-17	7.00E-17	7.36E-17	5.79E-17	6.24E-17	5.52E-17	6.86E-17	5.42E-17	5.43E-17	5.76E-17	5.31E-17	5.39E-17	6.03E-17
7.39E-17	8.78E-17	7.04E-17	7.37E-17	5.92E-17	5.46E-17	5.30E-17	7.04E-17	6.10E-17	5.50E-17	4.81E-17	5.29E-17	4.42E-17	5.30E-17
7.50E-17	8.63E-17	7.24E-17	8.03E-17	7.57E-17	5.46E-17	5.31E-17	5.70E-17	5.85E-17	5.09E-17	3.71E-17	4.61E-17	3.86E-17	4.33E-17
7.88E-17	8.07E-17	7.34E-17	8.63E-17	8.17E-17	6.14E-17	5.54E-17	4.98E-17	5.23E-17	4.54E-17	3.83E-17	3.86E-17	3.09E-17	3.55E-17
8.30E-17	6.82E-17	6.40E-17	8.37E-17	8.44E-17	6.52E-17	5.44E-17	4.84E-17	4.55E-17	4.44E-17	4.15E-17	4.13E-17	3.20E-17	3.76E-17
8.48E-17	5.88E-17	6.16E-17	7.86E-17	7.83E-17	6.31E-17	5.43E-17	5.07E-17	4.60E-17	4.94E-17	4.05E-17	5.51E-17	3.53E-17	4.26E-17
8.65E-17	6.02E-17	7.19E-17	7.32E-17	6.99E-17	5.84E-17	5.55E-17	5.11E-17	5.04E-17	5.00E-17	3.81E-17	5.60E-17	4.55E-17	5.01E-17
8.10E-17	5.84E-17	7.09E-17	6.95E-17	5.98E-17	5.56E-17	5.81E-17	5.37E-17	5.10E-17	5.40E-17	4.24E-17	5.46E-17	5.19E-17	5.44E-17
7.10E-17	6.65E-17	6.65E-17	6.07E-17	6.10E-17	4.93E-17	5.38E-17	4.86E-17	4.81E-17	5.35E-17	4.39E-17	5.44E-17	5.01E-17	5.41E-17
5.96E-17	6.26E-17	5.73E-17	4.86E-17	5.81E-17	4.65E-17	4.73E-17	4.91E-17	4.55E-17	5.56E-17	4.82E-17	5.05E-17	4.95E-17	5.59E-17
5.47E-17	6.08E-17	4.84E-17	4.37E-17	5.96E-17	5.24E-17	4.70E-17	4.84E-17	4.48E-17	5.62E-17	4.92E-17	4.34E-17	5.28E-17	6.60E-17
5.31E-17	5.83E-17	4.79E-17	5.29E-17	6.56E-17	5.49E-17	4.58E-17	4.77E-17	4.83E-17	5.80E-17	5.05E-17	3.71E-17	4.83E-17	6.52E-17
4.97E-17	5.70E-17	5.05E-17	6.05E-17	6.52E-17	5.26E-17	4.47E-17	4.41E-17	4.64E-17	5.83E-17	4.16E-17	3.31E-17	4.41E-17	5.86E-17
5.11E-17	6.03E-17	5.66E-17	5.89E-17	5.91E-17	4.56E-17	4.41E-17	4.35E-17	4.72E-17	4.80E-17	3.76E-17	3.38E-17	4.67E-17	5.47E-17
5.27E-17	5.93E-17	5.99E-17	5.40E-17	4.84E-17	4.57E-17	4.67E-17	4.59E-17	5.07E-17	4.68E-17	4.08E-17	3.38E-17	4.76E-17	4.69E-17
5.02E-17	5.24E-17	5.39E-17	4.81E-17	4.31E-17	4.81E-17	4.77E-17	4.80E-17	4.89E-17	4.68E-17	4.79E-17	3.91E-17	3.98E-17	4.01E-17
4.42E-17	4.28E-17	4.70E-17	4.44E-17	4.58E-17	4.87E-17	5.15E-17	5.18E-17	4.90E-17	4.57E-17	4.99E-17	3.93E-17	3.73E-17	3.82E-17
3.72E-17	3.79E-17	4.16E-17	4.43E-17	4.98E-17	4.92E-17	5.16E-17	5.17E-17	5.18E-17	4.62E-17	4.85E-17	4.09E-17	4.06E-17	3.70E-17
3.19E-17	3.45E-17	4.17E-17	5.03E-17	4.93E-17	5.01E-17	4.80E-17	4.35E-17	5.34E-17	4.95E-17	4.88E-17	4.66E-17	4.48E-17	3.81E-17
3.14E-17	3.71E-17	4.73E-17	5.50E-17	5.89E-17	5.67E-17	4.75E-17	3.86E-17	4.83E-17	4.48E-17	4.65E-17	5.11E-17	4.90E-17	3.94E-17
4.28E-17	4.52E-17	5.52E-17	5.75E-17	6.15E-17	5.99E-17	4.57E-17	3.98E-17	5.02E-17	3.98E-17	4.53E-17	5.25E-17	4.73E-17	4.05E-17
5.15E-17	4.80E-17	5.66E-17	5.57E-17	5.37E-17	6.06E-17	4.69E-17	4.62E-17	4.50E-17	3.62E-17	3.94E-17	5.22E-17	4.63E-17	4.25E-17
5.48E-17	4.79E-17	5.11E-17	4.96E-17	5.13E-17	6.31E-17	5.10E-17	4.54E-17	3.85E-17	3.56E-17	3.32E-17	5.07E-17	5.17E-17	4.86E-17
5.49E-17	4.72E-17	4.67E-17	4.57E-17	5.14E-17	6.20E-17	5.20E-17	4.41E-17	3.00E-17	3.28E-17	3.18E-17	4.91E-17	5.71E-17	5.79E-17
5.72E-17	4.85E-17	4.27E-17	4.28E-17	5.00E-17	5.73E-17	4.89E-17	4.40E-17	2.59E-17	3.28E-17	3.33E-17	4.51E-17	6.32E-17	6.12E-17
5.59E-17	4.65E-17	3.72E-17	3.80E-17	5.05E-17	5.18E-17	4.70E-17	4.96E-17	3.04E-17	3.63E-17	4.11E-17	4.88E-17	6.09E-17	5.85E-17
5.56E-17	4.21E-17	3.46E-17	3.90E-17	5.32E-17	4.87E-17	4.73E-17	4.85E-17	3.57E-17	4.27E-17	4.71E-17	5.60E-17	5.75E-17	6.25E-17
5.24E-17	3.99E-17	3.92E-17	4.47E-17	6.01E-17	5.39E-17	4.57E-17	4.86E-17	3.68E-17	4.71E-17	4.52E-17	5.76E-17	5.40E-17	6.65E-17
4.80E-17	3.96E-17	4.10E-17	4.58E-17	6.19E-17	5.28E-17	4.32E-17	4.97E-17	4.55E-17	5.24E-17	4.36E-17	5.82E-17	5.46E-17	7.04E-17

4.60E-17	3.83E-17	4.41E-17	4.71E-17	6.10E-17	5.21E-17	4.49E-17	4.78E-17	5.85E-17	6.14E-17	5.17E-17	6.32E-17	5.60E-17	6.80E-17
4.83E-17	3.73E-17	4.55E-17	4.56E-17	5.92E-17	4.89E-17	4.90E-17	4.86E-17	6.01E-17	6.48E-17	6.22E-17	6.70E-17	6.07E-17	6.53E-17
4.73E-17	4.08E-17	4.57E-17	4.34E-17	5.38E-17	4.85E-17	5.21E-17	4.81E-17	5.57E-17	6.25E-17	6.71E-17	6.18E-17	6.15E-17	5.71E-17
4.78E-17	4.17E-17	4.33E-17	4.42E-17	5.17E-17	5.10E-17	5.00E-17	4.67E-17	4.80E-17	5.98E-17	6.82E-17	5.78E-17	5.78E-17	4.98E-17
4.97E-17	4.51E-17	4.15E-17	4.39E-17	4.66E-17	5.09E-17	5.03E-17	4.73E-17	4.14E-17	5.43E-17	6.25E-17	5.75E-17	6.07E-17	4.49E-17
4.73E-17	4.33E-17	4.25E-17	4.38E-17	4.50E-17	4.72E-17	4.56E-17	4.78E-17	4.22E-17	4.67E-17	5.46E-17	5.66E-17	6.28E-17	5.26E-17
4.63E-17	4.23E-17	4.53E-17	4.83E-17	4.63E-17	4.68E-17	3.92E-17	4.47E-17	4.30E-17	4.33E-17	5.06E-17	5.64E-17	6.22E-17	6.28E-17
3.97E-17	4.05E-17	4.84E-17	5.10E-17	4.50E-17	4.07E-17	4.19E-17	4.99E-17	4.70E-17	3.89E-17	4.93E-17	5.85E-17	5.89E-17	6.04E-17
3.51E-17	4.24E-17	4.70E-17	4.91E-17	4.56E-17	3.93E-17	4.40E-17	5.08E-17	4.94E-17	4.12E-17	5.08E-17	5.71E-17	5.78E-17	5.75E-17
3.98E-17	4.65E-17	4.62E-17	4.56E-17	4.95E-17	4.18E-17	4.51E-17	4.33E-17	5.24E-17	4.06E-17	5.33E-17	5.67E-17	5.89E-17	6.06E-17
4.15E-17	5.04E-17	4.20E-17	3.97E-17	4.68E-17	4.25E-17	4.29E-17	3.84E-17	5.03E-17	4.60E-17	4.73E-17	4.67E-17	5.73E-17	6.15E-17
4.74E-17	5.04E-17	3.96E-17	3.65E-17	4.44E-17	4.20E-17	4.89E-17	4.29E-17	4.69E-17	4.55E-17	4.35E-17	4.35E-17	4.94E-17	6.01E-17
4.95E-17	4.77E-17	3.70E-17	3.72E-17	4.62E-17	4.24E-17	6.05E-17	4.30E-17	4.28E-17	4.30E-17	4.39E-17	4.53E-17	4.15E-17	5.14E-17
4.69E-17	4.46E-17	3.60E-17	4.08E-17	4.24E-17	4.25E-17	5.58E-17	3.66E-17	3.87E-17	3.79E-17	4.31E-17	4.61E-17	3.63E-17	3.93E-17
4.14E-17	4.17E-17	3.53E-17	4.27E-17	3.78E-17	4.21E-17	4.57E-17	3.23E-17	3.24E-17	3.17E-17	3.73E-17	4.35E-17	3.66E-17	3.00E-17
3.94E-17	3.99E-17	4.06E-17	3.82E-17	3.79E-17	3.94E-17	3.86E-17	3.34E-17	2.95E-17	2.93E-17	3.16E-17	4.26E-17	3.89E-17	2.72E-17
3.91E-17	4.32E-17	4.24E-17	3.65E-17	3.96E-17	4.04E-17	4.19E-17	3.49E-17	2.83E-17	3.59E-17	3.37E-17	4.47E-17	4.02E-17	3.31E-17
4.66E-17	4.69E-17	4.41E-17	3.91E-17	4.20E-17	3.80E-17	4.26E-17	3.59E-17	2.90E-17	3.93E-17	3.50E-17	4.40E-17	4.36E-17	3.41E-17
4.68E-17	4.86E-17	4.84E-17	4.30E-17	3.74E-17	3.58E-17	3.86E-17	3.30E-17	3.13E-17	4.26E-17	4.03E-17	4.42E-17	4.40E-17	3.57E-17
4.48E-17	5.10E-17	5.10E-17	4.29E-17	3.45E-17	3.17E-17	3.45E-17	3.39E-17	3.67E-17	3.93E-17	4.12E-17	4.50E-17	4.62E-17	3.59E-17
4.62E-17	4.78E-17	4.87E-17	3.82E-17	2.85E-17	2.85E-17	2.57E-17	3.38E-17	3.71E-17	3.87E-17	3.67E-17	4.11E-17	4.45E-17	3.74E-17
4.70E-17	4.28E-17	3.99E-17	3.38E-17	2.25E-17	2.61E-17	1.98E-17	3.41E-17	3.40E-17	3.31E-17	3.41E-17	3.70E-17	4.22E-17	3.95E-17
4.53E-17	3.84E-17	4.25E-17	4.08E-17	2.53E-17	2.29E-17	1.25E-17	3.15E-17	3.65E-17	3.15E-17	3.42E-17	3.52E-17	4.06E-17	3.59E-17
4.16E-17	3.86E-17	4.52E-17	4.05E-17	2.84E-17	2.17E-17	8.77E-18	2.52E-17	3.24E-17	2.65E-17	3.33E-17	3.32E-17	3.47E-17	3.66E-17
4.00E-17	3.71E-17	4.52E-17	3.26E-17	2.94E-17	2.42E-17	6.16E-18	2.53E-17	2.82E-17	2.12E-17	2.81E-17	3.23E-17	2.86E-17	3.54E-17
3.63E-17	3.64E-17	3.93E-17	2.72E-17	3.21E-17	2.70E-17	8.96E-18	2.58E-17	2.63E-17	1.97E-17	2.59E-17	2.93E-17	3.41E-17	3.22E-17
3.31E-17	3.15E-17	2.99E-17	2.71E-17	3.10E-17	2.94E-17	1.82E-17	2.71E-17	2.35E-17	2.53E-17	3.10E-17	2.65E-17	3.20E-17	3.10E-17
3.10E-17	2.60E-17	2.63E-17	2.74E-17	3.11E-17	2.86E-17	2.31E-17	2.71E-17	2.30E-17	2.93E-17	3.28E-17	2.51E-17	3.00E-17	3.42E-17
2.93E-17	2.17E-17	2.42E-17	2.65E-17	3.24E-17	2.63E-17	2.48E-17	2.91E-17	2.61E-17	3.40E-17	3.14E-17	3.14E-17	2.83E-17	3.00E-17
2.77E-17	2.29E-17	2.19E-17	2.40E-17	2.90E-17	2.34E-17	2.53E-17	2.64E-17	2.43E-17	3.23E-17	3.18E-17	3.66E-17	2.64E-17	2.78E-17
2.46E-17	2.43E-17	1.95E-17	2.12E-17	2.42E-17	2.12E-17	2.33E-17	2.95E-17	2.15E-17	2.94E-17	2.90E-17	3.47E-17	2.79E-17	3.10E-17
2.51E-17	2.33E-17	2.35E-17	1.60E-17	2.42E-17	2.52E-17	2.09E-17	3.22E-17	2.23E-17	2.78E-17	2.73E-17	3.17E-17	2.73E-17	3.28E-17
1.83E-17	1.86E-17	2.03E-17	1.28E-17	2.19E-17	2.21E-17	1.93E-17	3.21E-17	2.77E-17	2.65E-17	2.89E-17	2.32E-17	2.73E-17	3.41E-17
1.27E-17	1.31E-17	1.66E-17	1.18E-17	2.03E-17	1.75E-17	1.65E-17	2.76E-17	2.78E-17	2.34E-17	2.69E-17	1.77E-17	2.76E-17	3.04E-17
8.77E-18	1.04E-17	1.18E-17	1.54E-17	1.93E-17	1.53E-17	1.47E-17	2.40E-17	2.28E-17	1.98E-17	2.11E-17	2.26E-17	2.74E-17	2.93E-17
8.99E-18	1.12E-17	8.32E-18	1.42E-17	1.65E-17	1.76E-17	1.46E-17	2.24E-17	2.02E-17	1.60E-17	1.80E-17	2.58E-17	2.74E-17	2.35E-17
8.84E-18	1.16E-17	8.73E-18	1.35E-17	1.30E-17	1.64E-17	1.79E-17	2.36E-17	2.16E-17	1.35E-17	1.49E-17	2.73E-17	2.64E-17	2.71E-17
6.23E-18	9.88E-18	8.70E-18	1.37E-17	1.08E-17	1.42E-17	1.47E-17	2.22E-17	1.95E-17	1.31E-17	1.55E-17	2.62E-17	2.35E-17	2.93E-17
4.96E-18	9.20E-18	6.15E-18	8.98E-18	9.48E-18	1.24E-17	1.26E-17	1.71E-17	1.84E-17	1.34E-17	1.71E-17	2.61E-17	2.27E-17	2.58E-17
6.07E-18	8.63E-18	3.76E-18	6.18E-18	7.04E-18	1.09E-17	1.13E-17	1.46E-17	1.67E-17	1.50E-17	1.98E-17	1.99E-17	2.09E-17	2.48E-17
5.76E-18	7.08E-18	3.84E-18	3.51E-18	5.75E-18	9.47E-18	8.32E-18	1.05E-17	1.38E-17	1.79E-17	1.93E-17	1.37E-17	1.54E-17	2.33E-17
6.45E-18	6.98E-18	4.60E-18	2.02E-18	5.01E-18	7.89E-18	8.51E-18	8.20E-18	1.23E-17	1.58E-17	1.76E-17	1.10E-17	1.24E-17	1.87E-17
7.09E-18	6.71E-18	4.34E-18	9.36E-19	4.76E-18	5.48E-18	6.06E-18	5.95E-18	8.62E-18	1.22E-17	1.32E-17	9.14E-18	1.28E-17	1.69E-17
6.43E-18	5.73E-18	5.01E-18	1.43E-18	4.54E-18	3.17E-18	3.61E-18	4.28E-18	7.18E-18	9.94E-18	1.45E-17	1.04E-17	1.28E-17	1.43E-17
4.26E-18	3.07E-18	3.10E-18	1.79E-18	5.29E-18	2.22E-18	3.13E-18	3.75E-18	8.52E-18	8.86E-18	1.29E-17	1.03E-17	1.11E-17	1.40E-17
2.51E-18	2.05E-18	1.74E-18	2.01E-18	4.30E-18	1.75E-18	3.17E-18	5.26E-18	1.08E-17	7.40E-18	9.91E-18	7.41E-18	7.85E-18	1.45E-17
6.24E-19	1.60E-18	1.58E-18	1.35E-18	2.40E-18	2.76E-19	1.83E-18	4.56E-18	8.01E-18	3.66E-18	7.28E-18	3.56E-18	4.48E-18	1.06E-17
4.73E-22	1.07E-18	6.11E-19	1.17E-18	1.30E-18	7.64E-19	6.67E-19	3.06E-18	2.58E-18	1.54E-18	7.63E-18	4.21E-18	3.15E-18	8.40E-18
2.75E-19	2.05E-19	6.28E-20	3.71E-19	5.01E-19	7.90E-22	4.57E-20	1.70E-18	4.94E-19	2.04E-18	6.73E-18	4.97E-18	2.41E-18	6.43E-18
2.43E-19	1.97E-20	4.13E-20	2.49E-19	4.44E-20	1.03E-18	5.02E-20	8.50E-19	1.99E-19	2.35E-18	3.90E-18	1.79E-18	9.77E-19	4.79E-18
1.16E-18	6.02E-19	8.66E-19	3.05E-21	1.22E-19	1.59E-18	9.10E-20	1.19E-19	4.62E-21	1.42E-18	2.45E-18	5.56E-19	6.19E-19	2.80E-18
1.76E-18	1.14E-18	1.63E-18	2.22E-19	4.54E-19	5.52E-19	1.77E-19	9.58E-21	1.23E-19	4.75E-19	1.71E-18	1.93E-19	4.76E-19	1.06E-18
2.54E-18	2.76E-18	1.00E-18	2.85E-19	3.43E-19	2.34E-20	9.07E-20	1.97E-19	3.14E-19	8.10E-21	4.81E-19	3.60E-19	6.32E-19	6.02E-19
3.49E-18	3.28E-18	5.46E-19	7.52E-19	3.48E-19	1.52E-21	2.09E-19	3.59E-19	1.43E-19	3.42E-19	9.81E-21	6.00E-19	7.87E-19	5.46E-19
4.89E-18	4.23E-18	1.14E-18	2.98E-18	1.42E-18	7.19E-19	4.09E-19	2.21E-19	9.08E-19	2.21E-18	3.04E-19	2.59E-22	2.11E-19	2.09E-19
7.26E-18	6.71E-18	3.88E-18	6.14E-18	4.93E-18	3.37E-18	1.12E-18	8.91E-19	8.95E-19	2.33E-18	9.18E-19	7.82E-19	2.35E-21	4.64E-21
8.38E-18	8.54E-18	8.08E-18	7.72E-18	7.67E-18	5.80E-18	2.92E-18	3.08E-18	8.35E-19	7.22E-19	2.24E-18	1.91E-18	1.77E-19	2.81E-19
9.88E-18	9.39E-18	7.85E-18	9.40E-18	6.23E-18	6.56E-18	1.69E-18	4.80E-18	1.71E-18	6.80E-19	2.11E-18	2.06E-18	8.97E-19	5.01E-19
1.19E-17	1.19E-17	7.27E-18	9.58E-18	4.58E-18	7.22E-18	1.80E-18	5.92E-18	2.51E-18	1.60E-18	2.18E-18	2.20E-18	1.45E-18	1.02E-18
1.42E-17	1.77E-17	8.61E-18	9.85E-18	4.66E-18	6.48E-18	3.45E-18	7.35E-18	6.22E-18	3.91E-18	2.67E-18	2.13E-18	1.46E-18	1.21E-18
2.05E-17	2.26E-17	1.45E-17	1.25E-17	7.23E-18	8.01E-18	5.42E-18	7.83E-18	8.30E-18	6.53E-18	4.51E-18	2.15E-18	1.88E-18	1.81E-18
2.41E-17	2.29E-17	2.18E-17	2.09E-17	1.05E-17	1.24E-17	8.75E-18	1.30E-17	1.14E-17	1.04E-17	6.89E-18	4.96E-18	1.76E-18	3.26E-18
2.57E-17	2.63E-17	3.06E-17	2.55E-17	1.91E-17	1.90E-17	1.25E-17	1.96E-17	1.33E-17	1.08E-17	9.59E-18	8.92E-18	4.85E-18	6.08E-18
3.35E-17	3.67E-17	3.73E-17	3.88E-17	3.06E-17	2.46E-17	1.78E-17	2.18E-17	1.59E-17	1.00E-17	1.21E-17	1.25E-17	9.93E-18	9.21E-18
4.23E-17	4.25E-17	4.55E-17	4.29E-17	3.80E-17	2.90E-17	2.03E-17	2.39E-17	1.90E-17	1.22E-17	1.33E-17	1.48E-17	9.38E-18	1.11E-17
4.66E-17	4.97E-17	5.07E-17	3.71E-17	5.05E-17	3.40E-17	2.76E-17	3.49E-17	2.95E-17	1.70E-17	1.92E-17	1.66E-17	9.41E-18	1.49E-17
5.26E-17	5.41E-17	5.37E-17	3.84E-17	9.10E-17	4.04E-17	4.02E-17	3.79E-17	3.83E-17	2.46E-17	2.50E-17	1.82E-17	1.28E-17	2.07E-17
6.54E-17	6.04E-17	6.69E-17	4.34E-17	1.86E-16	5.21E-17	4.80E-17	3.53E-17	4.50E-17	3.35E-17	3.25E-17	2.29E-17	2.15E-17	2.58E-17
8.39E-17	7.28E-17	7.25E-17	5.17E-17	2.90E-16	6.48E-17	5.35E-17	3.85E-17	5.51E-17	4.22E-17	3.90E-17	2.89E-17	3.31E-17	3.21E-17
9.00E-17	8.49E-17	7.49E-17	6.03E-17	9.43E-17	8.14E-17	6.37E-17	4.48E-17	3.93E-17	5.06E-17	4.71E-17	3.62E-17	4.56E-17	4.08E-17
9.41E-17	1.03E-16	8.58E-17	7.19E-17	7.36E-17	8.39E-17	7.64E-17	5.66E-17	4.99E-17	5.19E-17	5.53E-17	4.63E-17	5.39E-17	4.78E-17
9.88E-17	1.20E-16	8.92E-17	8.66E-17	8.39E-17	8.24E-17	8.11E-17	6.75E-17	6.25E-17	6.11E-17	6.71E-17	6.47E-17	6.23E-17	5.92E-17
1.14E-16	1.27E-16	1.02E-16	1.01E-16	9.25E-17	8.50E-17	8.53E-17	7.66E-17	7.15E-17	7.17E-17	8.12E-17	8.14E-17	7.27E-17	7.07E-17
1.30E-16	1.30E-16	1.30E-16	1.25E-16	1.09E-16	1.00E-16	9.61E-17	9.76E-17	8.04E-17	8.93E-17	9.47E-17	9.67E-17	8.74E-17	8.95E-17
1.55E-16	1.45E-16	1.45E-16	1.49E-16	1.27E-16	1.03E-16	1.16E-16	1.12E-16	9.63E-17	1.11E-16	1.05E-16	1.06E-16	1.06E-16	1.09E-16
1.73E-16	1.56E-16	1.53E-16	1.78E-16	1.47E-16	1.17E-16	1.32E-16	1.29E-16	1.18E-16	1.24E-16	1.14E-16	1.24E-16	1.28E-16	1.18E-16
1.86E-16	1.69E-16	1.69E-16	2.00E-16	1.64E-16	1.40E-16	1.48E-16	1.45E-16	1.39E-16	1.35E-16	1.36E-16	1.51E-16	1.49E-16	1.38E-16
1.92E-16	1.96E-16	1.92E-16	2.05E-16	1.74E-16	1.64E-16	1.57E-16	1.45E-16	1.56E-16	1.62E-16	1.71E-16	1.82E-16	1.80E-16	1.70E-16
2.08E-16	2.20E-16	2.05E-16	2.11E-16	2.00E-16	1.86E-16	1.74E-16	1.62E-16	1.84E-16	2.04E-16	2.07E-16	1.83E-16	1.85E-16	2.02E-16
2.35E-16	2.43E-16	2.25E-16	2.33E-16	2.37E-16	2.15E-16	1.88E-16	1.88E-16	2.18E-16	2.35E-16	2.25E-16	2.02E-16	2.04E-16	1.95E-16
2.57E-16	2.84E-16	2.49E-16	2.61E-16	2.66E-16	2.54E-16	2.13E-16	2.13E-16	2.49E-16	2.33E-16	2.39E-16	2.37E-16	2.22E-16	1.80E-16
2.70E-16	3.13E-16	2.76E-16	2.74E-16	2.92E-16	3.15E-16	2.40E-16	2.41E-16	2.67E-16	2.48E-16	2.74E-16	2.51E-16	2.26E-16	1.90E-16
2.97E-16	3.22E-16	3.01E-16	2.94E-16	3.19E-16	3.49E-16	2.63E-16	2.54E-16	2.76E-16	2.76E-16	2.92E-16	2.92E-16	2.53E-16	2.22E-16
3.30E-16	3.30E-16	3.18E-16	3.24E-16	3.35E-16	3.27E-16	2.91E-16	2.90E-16	3.02E-16	3.04E-16	3.06E-16	2.93E-16	2.96E-16	2.64E-16
3.61E-16	3.38E-16	3.44E-16	3.60E-16	3.50E-16	3.49E-16	3.16E-16	3.20E-16	3.31E-16	3.28E-16	3.16E-16	3.11E-16	3.42E-16	3.10E-16
3.86E-16	3.64E-16	3.83E-16	3.95E-16	3.71E-16	3.72E-16	3.40E-16	3.55E-16	3.53E-16	3.33E-16	3.38E-16	3.41E-16	3.83E-16	3.59E-16

4.23E-16	4.03E-16	4.22E-16	4.09E-16	3.89E-16	4.05E-16	3.71E-16	3.91E-16	3.80E-16	3.49E-16	3.57E-16	3.77E-16	3.94E-16	3.78E-16
4.67E-16	4.36E-16	4.63E-16	4.29E-16	4.16E-16	4.28E-16	4.09E-16	4.29E-16	4.03E-16	3.74E-16	3.85E-16	3.94E-16	4.06E-16	3.92E-16
5.03E-16	4.71E-16	4.90E-16	4.68E-16	4.48E-16	4.52E-16	4.53E-16	4.71E-16	4.47E-16	4.27E-16	4.43E-16	4.28E-16	4.22E-16	4.32E-16
5.35E-16	5.11E-16	5.13E-16	5.03E-16	4.98E-16	4.91E-16	4.91E-16	5.24E-16	5.13E-16	4.84E-16	5.14E-16	4.81E-16	4.56E-16	4.66E-16
5.83E-16	5.46E-16	5.36E-16	5.41E-16	5.34E-16	5.29E-16	5.59E-16	5.82E-16	5.73E-16	5.51E-16	5.66E-16	5.32E-16	5.07E-16	5.01E-16
6.44E-16	5.98E-16	5.95E-16	5.85E-16	5.57E-16	5.72E-16	6.31E-16	6.44E-16	6.32E-16	6.03E-16	5.79E-16	5.84E-16	5.72E-16	5.33E-16
7.02E-16	6.60E-16	6.65E-16	6.56E-16	5.93E-16	6.17E-16	6.53E-16	6.65E-16	6.58E-16	6.67E-16	6.05E-16	6.57E-16	6.37E-16	5.71E-16
7.57E-16	7.09E-16	7.09E-16	7.06E-16	6.39E-16	6.62E-16	7.01E-16	6.82E-16	6.87E-16	6.94E-16	6.60E-16	7.50E-16	6.90E-16	6.51E-16
7.95E-16	7.57E-16	7.48E-16	7.54E-16	6.93E-16	7.03E-16	7.56E-16	7.30E-16	7.36E-16	7.26E-16	7.28E-16	7.44E-16	7.30E-16	7.23E-16
8.40E-16	8.15E-16	8.07E-16	8.10E-16	7.59E-16	7.74E-16	7.79E-16	7.87E-16	7.84E-16	7.79E-16	7.88E-16	7.80E-16	8.08E-16	8.04E-16
8.80E-16	8.82E-16	8.76E-16	8.67E-16	8.28E-16	8.52E-16	8.60E-16	8.56E-16	8.47E-16	8.32E-16	8.52E-16	8.60E-16	8.90E-16	8.87E-16
1.45E-14	1.41E-14	1.38E-14	1.38E-14	1.38E-14	1.32E-14	1.27E-14	1.29E-14	1.28E-14	1.26E-14	1.27E-14	1.29E-14	1.28E-14	1.26E-14

-7.60E-09	-7.27E-09	-7.56E-09	-6.94E-09	-6.76E-09	-5.41E-09	-5.75E-09	-5.50E-09	-5.44E-09	-4.76E-09	-3.92E-09	-3.31E-09	-2.43E-09	-2.14E-09
-7.43E-09	-7.08E-09	-6.94E-09	-6.79E-09	-6.83E-09	-5.38E-09	-5.29E-09	-4.73E-09	-5.17E-09	-4.41E-09	-4.05E-09	-3.34E-09	-2.46E-09	-1.35E-09
-7.03E-09	-7.06E-09	-6.41E-09	-6.27E-09	-6.59E-09	-5.26E-09	-5.43E-09	-4.30E-09	-4.49E-09	-4.41E-09	-4.33E-09	-2.95E-09	-2.53E-09	-1.15E-09
-6.37E-09	-6.61E-09	-6.29E-09	-6.13E-09	-6.19E-09	-5.45E-09	-5.57E-09	-4.82E-09	-4.91E-09	-4.21E-09	-4.07E-09	-3.04E-09	-2.85E-09	-1.97E-09
-5.81E-09	-5.77E-09	-6.48E-09	-6.22E-09	-6.06E-09	-5.75E-09	-5.72E-09	-5.37E-09	-5.25E-09	-4.27E-09	-3.72E-09	-3.27E-09	-3.05E-09	-2.46E-09
-5.64E-09	-5.28E-09	-6.91E-09	-6.95E-09	-6.13E-09	-6.10E-09	-5.83E-09	-5.16E-09	-5.40E-09	-4.82E-09	-4.24E-09	-3.26E-09	-3.14E-09	-2.55E-09
-6.45E-09	-6.01E-09	-7.38E-09	-7.22E-09	-6.31E-09	-6.52E-09	-6.24E-09	-5.19E-09	-5.46E-09	-5.10E-09	-4.75E-09	-3.17E-09	-3.08E-09	-2.68E-09
-6.83E-09	-7.07E-09	-7.64E-09	-7.72E-09	-6.60E-09	-7.00E-09	-6.28E-09	-5.62E-09	-5.55E-09	-5.55E-09	-4.90E-09	-3.25E-09	-3.16E-09	-2.80E-09
-7.21E-09	-7.42E-09	-7.60E-09	-7.70E-09	-6.72E-09	-7.16E-09	-6.52E-09	-6.22E-09	-5.98E-09	-5.57E-09	-4.84E-09	-3.68E-09	-3.36E-09	-2.86E-09
-7.66E-09	-7.04E-09	-7.82E-09	-7.88E-09	-7.37E-09	-6.95E-09	-6.58E-09	-6.10E-09	-6.29E-09	-5.18E-09	-4.63E-09	-4.05E-09	-3.11E-09	-3.00E-09
-7.66E-09	-6.93E-09	-7.63E-09	-7.05E-09	-7.29E-09	-6.88E-09	-6.40E-09	-5.74E-09	-5.84E-09	-4.96E-09	-4.98E-09	-4.51E-09	-3.51E-09	-3.32E-09
-7.63E-09	-6.54E-09	-7.03E-09	-6.52E-09	-6.97E-09	-6.50E-09	-6.04E-09	-5.26E-09	-5.66E-09	-4.90E-09	-5.10E-09	-5.19E-09	-4.16E-09	-3.85E-09
-8.23E-09	-6.41E-09	-6.75E-09	-6.20E-09	-6.52E-09	-6.53E-09	-6.05E-09	-5.04E-09	-5.27E-09	-4.74E-09	-5.16E-09	-5.48E-09	-4.58E-09	-4.25E-09
-7.96E-09	-6.91E-09	-6.99E-09	-6.77E-09	-6.34E-09	-7.01E-09	-6.43E-09	-5.27E-09	-4.87E-09	-4.49E-09	-5.18E-09	-5.66E-09	-4.36E-09	-4.49E-09
-7.64E-09	-7.23E-09	-6.24E-09	-6.82E-09	-6.23E-09	-6.98E-09	-6.79E-09	-5.27E-09	-4.87E-09	-4.13E-09	-4.86E-09	-5.56E-09	-4.11E-09	-4.34E-09
-7.34E-09	-7.06E-09	-5.25E-09	-6.52E-09	-6.65E-09	-6.99E-09	-6.98E-09	-5.38E-09	-4.42E-09	-3.92E-09	-4.80E-09	-4.75E-09	-3.79E-09	-4.56E-09
-7.14E-09	-6.75E-09	-4.96E-09	-6.18E-09	-6.70E-09	-6.88E-09	-6.70E-09	-5.36E-09	-4.95E-09	-4.60E-09	-5.22E-09	-4.16E-09	-3.38E-09	-4.36E-09
-6.70E-09	-6.56E-09	-5.87E-09	-6.23E-09	-6.70E-09	-7.10E-09	-6.19E-09	-5.38E-09	-5.55E-09	-5.53E-09	-5.35E-09	-4.41E-09	-3.36E-09	-3.87E-09
-6.52E-09	-7.02E-09	-6.52E-09	-6.76E-09	-6.84E-09	-6.70E-09	-5.90E-09	-5.46E-09	-5.65E-09	-5.58E-09	-5.31E-09	-4.57E-09	-4.43E-09	-3.82E-09
-6.16E-09	-7.15E-09	-6.76E-09	-6.72E-09	-6.44E-09	-5.70E-09	-5.83E-09	-5.73E-09	-5.29E-09	-5.35E-09	-5.14E-09	-4.73E-09	-5.67E-09	-4.31E-09
-6.05E-09	-6.96E-09	-6.83E-09	-6.66E-09	-6.24E-09	-5.69E-09	-5.89E-09	-5.72E-09	-5.08E-09	-5.23E-09	-5.21E-09	-5.31E-09	-5.86E-09	-4.84E-09
-5.96E-09	-6.70E-09	-6.19E-09	-6.91E-09	-6.74E-09	-6.16E-09	-6.22E-09	-6.04E-09	-5.59E-09	-5.45E-09	-5.55E-09	-5.94E-09	-5.76E-09	-5.21E-09
-6.67E-09	-6.80E-09	-5.38E-09	-6.70E-09	-7.05E-09	-6.29E-09	-6.54E-09	-6.44E-09	-5.99E-09	-5.82E-09	-5.72E-09	-5.74E-09	-5.62E-09	-5.13E-09
-7.05E-09	-7.10E-09	-4.60E-09	-6.87E-09	-6.81E-09	-6.37E-09	-6.79E-09	-6.60E-09	-6.20E-09	-5.90E-09	-5.74E-09	-5.75E-09	-5.20E-09	-5.46E-09
-7.36E-09	-7.25E-09	-5.27E-09	-6.60E-09	-6.95E-09	-6.65E-09	-6.67E-09	-6.60E-09	-6.44E-09	-6.41E-09	-5.94E-09	-6.07E-09	-5.48E-09	-6.88E-09
-7.65E-09	-7.08E-09	-6.84E-09	-6.67E-09	-7.20E-09	-7.03E-09	-6.53E-09	-6.39E-09	-6.75E-09	-6.65E-09	-6.11E-09	-6.28E-09	-5.44E-09	-8.21E-09
-7.63E-09	-7.40E-09	-6.97E-09	-6.72E-09	-7.41E-09	-7.40E-09	-6.34E-09	-6.20E-09	-6.56E-09	-6.41E-09	-6.18E-09	-6.41E-09	-5.82E-09	-7.92E-09
-7.60E-09	-7.65E-09	-7.29E-09	-6.94E-09	-7.76E-09	-7.33E-09	-6.46E-09	-6.38E-09	-6.23E-09	-6.37E-09	-6.30E-09	-5.97E-09	-5.91E-09	-6.63E-09
-7.72E-09	-7.70E-09	-7.53E-09	-7.24E-09	-7.86E-09	-6.88E-09	-6.71E-09	-6.38E-09	-6.10E-09	-6.79E-09	-6.17E-09	-5.61E-09	-5.85E-09	-6.54E-09
-7.97E-09	-7.84E-09	-7.68E-09	-7.31E-09	-7.91E-09	-7.13E-09	-7.09E-09	-6.77E-09	-6.54E-09	-7.14E-09	-6.52E-09	-5.82E-09	-5.97E-09	-6.35E-09
-7.84E-09	-7.67E-09	-7.55E-09	-7.15E-09	-7.96E-09	-7.48E-09	-7.48E-09	-6.95E-09	-6.40E-09	-7.02E-09	-6.68E-09	-6.55E-09	-6.19E-09	-6.25E-09
-7.75E-09	-7.34E-09	-7.21E-09	-7.09E-09	-8.21E-09	-7.75E-09	-7.42E-09	-7.17E-09	-6.47E-09	-6.98E-09	-6.69E-09	-6.68E-09	-6.41E-09	-5.97E-09
-7.48E-09	-7.76E-09	-7.27E-09	-7.71E-09	-8.51E-09	-7.86E-09	-7.24E-09	-7.26E-09	-6.52E-09	-6.42E-09	-6.24E-09	-6.40E-09	-6.72E-09	-5.88E-09
-7.35E-09	-8.07E-09	-7.78E-09	-8.11E-09	-8.33E-09	-7.62E-09	-7.00E-09	-7.53E-09	-6.83E-09	-6.57E-09	-5.64E-09	-6.27E-09	-6.66E-09	-6.22E-09
-7.55E-09	-8.12E-09	-7.76E-09	-7.91E-09	-7.96E-09	-7.14E-09	-7.02E-09	-7.88E-09	-7.28E-09	-7.14E-09	-6.00E-09	-6.13E-09	-6.55E-09	-6.55E-09
-7.49E-09	-8.24E-09	-7.86E-09	-7.74E-09	-7.69E-09	-6.96E-09	-7.41E-09	-7.89E-09	-7.80E-09	-7.01E-09	-6.30E-09	-6.14E-09	-6.46E-09	-6.47E-09
-8.04E-09	-8.31E-09	-8.40E-09	-7.55E-09	-7.56E-09	-7.26E-09	-7.56E-09	-8.06E-09	-7.64E-09	-6.55E-09	-6.48E-09	-6.64E-09	-6.67E-09	-6.48E-09
-8.60E-09	-8.28E-09	-8.31E-09	-7.33E-09	-7.59E-09	-7.52E-09	-7.59E-09	-8.20E-09	-7.59E-09	-6.12E-09	-6.55E-09	-7.32E-09	-7.07E-09	-6.50E-09
-8.37E-09	-8.09E-09	-8.00E-09	-6.77E-09	-7.44E-09	-7.71E-09	-7.64E-09	-7.87E-09	-7.58E-09	-5.75E-09	-6.96E-09	-7.64E-09	-7.59E-09	-6.72E-09
-8.14E-09	-7.73E-09	-7.52E-09	-7.77E-09	-7.36E-09	-7.51E-09	-7.64E-09	-7.48E-09	-6.99E-09	-5.76E-09	-7.02E-09	-7.36E-09	-7.66E-09	-6.57E-09
-7.99E-09	-7.81E-09	-7.48E-09	-7.73E-09	-7.28E-09	-7.67E-09	-7.95E-09	-7.55E-09	-6.93E-09	-5.90E-09	-6.93E-09	-7.22E-09	-7.49E-09	-6.38E-09
-8.10E-09	-7.67E-09	-7.67E-09	-8.00E-09	-7.31E-09	-7.81E-09	-7.81E-09	-7.50E-09	-7.13E-09	-6.05E-09	-6.66E-09	-7.20E-09	-7.51E-09	-6.77E-09
-8.12E-09	-7.82E-09	-7.79E-09	-7.92E-09	-7.42E-09	-8.17E-09	-7.75E-09	-6.95E-09	-6.91E-09	-6.38E-09	-6.96E-09	-7.05E-09	-7.33E-09	-7.14E-09
-7.33E-09	-7.75E-09	-7.90E-09	-7.78E-09	-7.54E-09	-8.01E-09	-7.81E-09	-6.73E-09	-6.72E-09	-6.28E-09	-6.88E-09	-6.81E-09	-7.03E-09	-6.98E-09
-6.85E-09	-7.43E-09	-7.63E-09	-8.10E-09	-7.89E-09	-7.50E-09	-7.44E-09	-6.84E-09	-7.08E-09	-6.61E-09	-6.78E-09	-6.40E-09	-6.52E-09	-6.80E-09
-6.27E-09	-7.32E-09	-7.16E-09	-7.85E-09	-7.80E-09	-7.40E-09	-7.24E-09	-7.10E-09	-7.64E-09	-7.39E-09	-7.18E-09	-6.69E-09	-6.26E-09	-6.54E-09
-5.97E-09	-7.55E-09	-7.01E-09	-7.54E-09	-7.56E-09	-7.49E-09	-7.31E-09	-7.46E-09	-7.97E-09	-7.49E-09	-7.45E-09	-6.85E-09	-6.46E-09	-6.63E-09
-6.12E-09	-7.35E-09	-7.16E-09	-7.38E-09	-7.23E-09	-7.45E-09	-7.37E-09	-6.91E-09	-7.85E-09	-7.19E-09	-7.21E-09	-7.03E-09	-6.72E-09	-6.89E-09
-5.61E-09	-6.90E-09	-7.47E-09	-7.59E-09	-6.86E-09	-6.93E-09	-7.30E-09	-6.02E-09	-7.70E-09	-6.93E-09	-7.26E-09	-7.31E-09	-6.93E-09	-6.81E-09
-5.98E-09	-7.01E-09	-7.54E-09	-7.52E-09	-6.69E-09	-6.44E-09	-6.80E-09	-3.12E-09	-7.14E-09	-6.55E-09	-7.15E-09	-7.22E-09	-6.81E-09	-6.79E-09
-6.45E-09	-7.06E-09	-7.66E-09	-7.43E-09	-6.75E-09	-5.75E-09	-6.03E-09	8.70E-10	-6.92E-09	-6.21E-09	-6.97E-09	-7.21E-09	-6.67E-09	-6.71E-09
-6.94E-09	-6.88E-09	-7.17E-09	-7.13E-09	-6.31E-09	-6.45E-09	-5.34E-09	1.68E-09	-6.68E-09	-6.16E-09	-6.99E-09	-7.17E-09	-6.34E-09	-6.44E-09
-6.76E-09	-6.50E-09	-6.48E-09	-6.54E-09	-6.22E-09	-6.87E-09	-6.25E-09	-6.41E-09	-6.76E-09	-6.14E-09	-7.15E-09	-6.92E-09	-6.34E-09	-6.22E-09
-6.50E-09	-6.33E-09	-6.49E-09	-6.55E-09	-6.31E-09	-6.79E-09	-6.53E-09	-6.65E-09	-6.77E-09	-5.90E-09	-6.97E-09	-6.69E-09	-6.23E-09	-6.01E-09
-6.40E-09	-6.50E-09	-6.67E-09	-6.72E-09	-6.60E-09	-6.58E-09	-6.57E-09	-6.65E-09	-6.83E-09	-6.10E-09	-7.09E-09	-6.10E-09	-5.99E-09	-5.99E-09
-6.39E-09	-6.49E-09	-7.22E-09	-7.30E-09	-7.28E-09	-6.76E-09	-7.04E-09	-6.64E-09	-6.71E-09	-6.63E-09	-6.80E-09	-5.22E-09	-5.65E-09	-5.97E-09
-6.06E-09	-6.33E-09	-7.13E-09	-7.35E-09	-7.19E-09	-6.96E-09	-6.91E-09	-6.35E-09	-6.57E-09	-6.27E-09	-6.18E-09	-5.36E-09	-5.90E-09	-6.12E-09
-5.76E-09	-6.46E-09	-6.92E-09	-7.14E-09	-6.91E-09	-6.65E-09	-6.41E-09	-6.14E-09	-6.14E-09	-5.53E-09	-5.47E-09	-5.48E-09	-6.39E-09	-6.35E-09
-5.72E-09	-6.76E-09	-6.74E-09	-7.23E-09	-6.69E-09	-6.20E-09	-6.03E-09	-6.15E-09	-5.51E-09	-5.39E-09	-5.36E-09	-5.77E-09	-6.43E-09	-6.74E-09
-5.87E-09	-6.48E-09	-6.83E-09	-6.77E-09	-6.56E-09	-5.99E-09	-6.10E-09	-6.12E-09	-5.15E-09	-5.83E-09	-5.26E-09	-5.43E-09	-6.11E-09	-6.77E-09
-6.57E-09	-5.94E-09	-6.65E-09	-6.04E-09	-6.13E-09	-5.40E-09	-5.54E-09	-6.06E-09	-5.05E-09	-6.16E-09	-5.02E-09	-5.87E-09	-5.95E-09	-6.19E-09
-6.62E-09	-5.35E-09	-6.23E-09	-5.43E-09	-5.84E-09	-5.07E-09	-5.23E-09	-6.09E-09	-5.61E-09	-6.35E-09	-4.95E-09	-5.70E-09	-5.45E-09	-5.72E-09
-6.07E-09	-4.98E-09	-6.20E-09	-5.54E-09	-6.26E-09	-5.76E-09	-5.23E-09	-6.05E-09	-5.32E-09	-6.28E-09	-5.30E-09	-5.65E-09	-5.49E-09	-5.48E-09
-5.99E-09	-5.39E-09	-6.32E-09	-6.00E-09	-6.64E-09	-5.92E-09	-5.57E-09	-5.99E-09	-4.85E-09	-6.09E-09	-5.22E-09	-5.59E-09	-6.07E-09	-5.63E-09
-5.97E-09	-5.75E-09	-6.58E-09	-6.41E-09	-6.29E-09	-5.94E-09	-5.47E-09	-5.64E-09	-4.90E-09	-5.85E-09	-4.89E-09	-5.44E-09	-6.13E-09	-5.43E-09
-5.99E-09	-6.25E-09	-6.76E-09	-6.62E-09	-5.96E-09	-5.74E-09	-5.10E-09	-5.56E-09	-5.44E-09	-5.52E-09	-5.02E-09	-5.16E-09	-5.86E-09	-5.02E-09
-5.93E-09	-5.93E-09	-6.48E-09	-6.34E-09	-6.00E-09	-5.65E-09	-4.42E-09	-5.49E-09	-5.12E-09	-4.99E-09	-5.21E-09	-4.81E-09	-5.56E-09	-4.61E-09
-5.96E-09	-5.76E-09	-6.02E-09	-6.11E-09	-6.22E-09	-5.22E-09	-3.88E-09	-4.76E-09	-4.42E-09	-4.65E-09	-4.75E-09	-4.09E-09	-5.42E-09	-4.41E-09
-5.42E-09	-5.88E-09	-5.97E-09	-5.92E-09	-6.04E-09	-4.78E-09	-3.41E-09	-4.20E-09	-3.71E-09	-4.40E-09	-4.25E-09	-3.80E-09	-5.20E-09	-4.93E-09
-4.94E-09	-4.98E-09	-5.61E-09	-5.56E-09	-5.42E-09	-3.90E-09	-3.21E-09	-4.25E-09	-3.25E-09	-4.22E-09	-3.99E-09	-3.89E-09	-4.88E-09	-4.55E-09
-4.81E-09	-4.05E-09	-5.12E-09	-4.68E-09	-4.84E-09	-3.77E-09	-3.18E-09	-4.54E-09	-3.92E-09	-4.36E-09	-4.08E-09	-3.94E-09	-4.22E-09	-4.06E-09
-4.69E-09	-4.15E-09	-4.67E-09	-3.98E-09	-4.48E-09	-3.74E-09	-3.81E-09	-4.49E-09	-4.21E-09	-4.50E-09	-4.19E-09	-4.16E-09	-4.04E-09	-4.06E-09
-4.14E-09	-4.01E-09	-4.48E-09	-3.98E-09	-3.86E-09	-3.31E-09	-3.82E-09	-4.42E-09	-3.95E-09	-4.38E-09	-4.39E-09	-3.93E-09	-3.87E-09	-4.17E-09
-3.62E-09	-3.68E-09	-4.58E-09	-3.71E-09	-3.60E-09	-3.42E-09	-3.76E-09	-4.03E-09	-3.81E-09	-4.03E-09	-4.16E-09	-3.81E-09	-3.87E-09	-4.03E-09
-3.34E-09	-4.17E-09	-4.66E-09	-3.59E-09	-3.51E-09	-3.63E-09	-3.76E-09	-3.63E-09	-3.42E-09	-4.14E-09	-4.07E-09	-3.80E-09	-4.09E-09	-4.01E-09
-3.55E-09	-4.26E-09	-4.55E-09	-4.01E-09	-3.83E-09	-4.01E-09	-4.25E-09	-3.29E-09	-3.31E-09	-4.07E-09	-4.02E-09	-3.64E-09	-4.40E-09	-3.91E-09
-3.57E-09	-4.00E-09	-4.07E-09	-3.89E-09	-3.67E-09	-4.00E-09	-4.15E-09	-3.10E-09	-3.55E-09	-3.68E-09	-3.48E-09	-3.11E-09	-4.31E-09	-3.67E-09
-3.42E-09	-3.20E-09	-3.23E-09	-3.29E-09	-3.54E-09	-3.89E-09	-3.74E-09	-3.17E-09	-3.54E-09	-3.42E-09	-3.06E-09	-2.60E-09	-3.72E-09	-3.18E-09

-2.86E-09	-2.69E-09	-2.76E-09	-3.02E-09	-3.64E-09	-3.51E-09	-3.38E-09	-3.23E-09	-3.42E-09	-3.36E-09	-2.66E-09	-2.01E-09	-3.26E-09	-2.97E-09
-1.84E-09	-2.09E-09	-2.87E-09	-2.86E-09	-3.32E-09	-3.03E-09	-2.94E-09	-3.39E-09	-3.30E-09	-3.23E-09	-2.30E-09	-1.93E-09	-2.81E-09	-2.94E-09
-1.18E-09	-2.00E-09	-2.80E-09	-2.88E-09	-2.89E-09	-2.47E-09	-2.82E-09	-2.96E-09	-3.11E-09	-2.96E-09	-1.75E-09	-2.13E-09	-2.67E-09	-2.82E-09
-6.59E-10	-1.77E-09	-2.72E-09	-2.62E-09	-2.38E-09	-1.97E-09	-2.37E-09	-2.51E-09	-3.07E-09	-2.54E-09	-1.54E-09	-2.53E-09	-2.41E-09	-2.67E-09
-6.39E-11	-1.59E-09	-2.58E-09	-2.38E-09	-2.05E-09	-1.67E-09	-2.36E-09	-2.30E-09	-2.67E-09	-2.14E-09	-2.02E-09	-2.57E-09	-1.90E-09	-2.41E-09
3.09E-10	-1.26E-09	-2.26E-09	-2.06E-09	-1.87E-09	-1.45E-09	-2.70E-09	-2.20E-09	-2.15E-09	-1.85E-09	-2.09E-09	-1.98E-09	-1.24E-09	-2.08E-09
-1.27E-10	-9.23E-10	-1.80E-09	-1.36E-09	-1.79E-09	-1.32E-09	-2.52E-09	-1.93E-09	-1.47E-09	-1.43E-09	-1.73E-09	-1.52E-09	-7.61E-10	-1.46E-09
-4.75E-10	-5.46E-10	-1.08E-09	-9.35E-10	-1.08E-09	-1.32E-09	-1.79E-09	-1.48E-09	-1.16E-09	-9.96E-10	-1.47E-09	-1.28E-09	-4.57E-10	-5.83E-10
-2.30E-10	-1.08E-10	-8.06E-10	-8.40E-10	-4.50E-10	-7.62E-10	-7.36E-10	-7.15E-10	-8.11E-10	-7.43E-10	-1.35E-09	-1.17E-09	-6.35E-10	-5.76E-10
5.82E-10	3.60E-10	-4.79E-10	-3.37E-10	5.04E-10	-1.42E-10	3.18E-11	1.44E-10	2.49E-11	-3.17E-10	-1.11E-09	-7.74E-10	-7.00E-10	-6.91E-10
8.16E-10	7.10E-10	-2.81E-11	1.68E-10	1.05E-09	6.38E-10	7.26E-10	8.32E-10	8.78E-10	4.38E-10	-5.53E-10	-5.77E-10	-4.14E-10	-2.04E-10
1.06E-09	1.18E-09	6.73E-10	5.52E-10	1.20E-09	5.04E-10	1.41E-09	1.37E-09	1.30E-09	8.33E-10	1.13E-10	-4.87E-10	-1.03E-10	2.93E-11
1.19E-09	1.80E-09	1.35E-09	1.05E-09	9.42E-10	6.63E-10	6.81E-10	1.28E-09	1.37E-09	7.22E-10	6.06E-10	1.15E-10	3.23E-12	5.34E-10
1.18E-09	1.68E-09	2.00E-09	1.48E-09	1.08E-09	6.15E-10	5.56E-10	1.43E-09	1.12E-09	4.77E-11	8.24E-10	6.22E-10	6.14E-10	9.29E-10
1.27E-09	1.93E-09	2.84E-09	1.72E-09	1.54E-09	1.06E-09	7.90E-10	1.95E-09	1.22E-09	2.17E-12	8.43E-10	1.45E-09	1.12E-09	1.44E-09
1.65E-09	2.48E-09	3.47E-09	1.68E-09	1.71E-09	1.45E-09	1.13E-09	2.42E-09	1.21E-09	9.66E-10	1.44E-09	2.21E-09	1.91E-09	2.14E-09
2.30E-09	2.83E-09	3.32E-09	2.08E-09	2.20E-09	1.99E-09	1.48E-09	2.42E-09	2.00E-09	1.86E-09	1.56E-09	2.33E-09	2.50E-09	2.38E-09
2.68E-09	3.84E-09	3.72E-09	2.60E-09	3.61E-09	2.80E-09	2.54E-09	2.81E-09	3.24E-09	2.57E-09	1.80E-09	2.01E-09	2.62E-09	2.07E-09
3.15E-09	3.83E-09	3.59E-09	3.12E-09	4.23E-09	3.00E-09	3.42E-09	3.10E-09	3.76E-09	3.15E-09	2.12E-09	2.66E-09	2.64E-09	2.28E-09
3.82E-09	3.75E-09	4.08E-09	3.59E-09	4.08E-09	3.75E-09	4.07E-09	3.27E-09	3.77E-09	3.41E-09	2.87E-09	3.63E-09	2.91E-09	2.86E-09
4.50E-09	4.27E-09	4.61E-09	4.22E-09	4.53E-09	4.48E-09	4.71E-09	3.84E-09	3.88E-09	3.83E-09	3.36E-09	4.87E-09	3.53E-09	3.47E-09
5.61E-09	4.98E-09	5.31E-09	5.09E-09	5.34E-09	4.82E-09	5.17E-09	4.32E-09	4.39E-09	4.22E-09	4.09E-09	5.18E-09	3.94E-09	3.57E-09
6.51E-09	6.11E-09	6.17E-09	5.50E-09	5.94E-09	5.29E-09	5.36E-09	4.78E-09	5.12E-09	5.08E-09	5.09E-09	5.47E-09	4.95E-09	4.29E-09
7.27E-09	6.87E-09	6.97E-09	6.27E-09	6.28E-09	6.00E-09	6.04E-09	4.90E-09	5.86E-09	5.73E-09	5.97E-09	6.68E-09	5.71E-09	5.14E-09
8.00E-09	7.45E-09	7.75E-09	7.01E-09	6.87E-09	6.65E-09	6.41E-09	5.76E-09	6.24E-09	6.37E-09	6.90E-09	7.32E-09	6.12E-09	5.87E-09
8.43E-09	7.94E-09	8.30E-09	7.28E-09	7.49E-09	7.37E-09	7.08E-09	6.67E-09	7.52E-09	7.07E-09	7.07E-09	6.73E-09	6.42E-09	7.10E-09
9.21E-09	8.55E-09	9.08E-09	7.52E-09	7.63E-09	7.87E-09	7.83E-09	7.34E-09	8.29E-09	7.60E-09	7.49E-09	7.05E-09	6.90E-09	7.82E-09
1.04E-08	9.22E-09	9.04E-09	8.44E-09	8.50E-09	8.40E-09	8.43E-09	8.09E-09	8.73E-09	7.81E-09	8.29E-09	7.59E-09	7.47E-09	8.01E-09
1.08E-08	9.79E-09	9.39E-09	9.39E-09	9.12E-09	9.13E-09	8.76E-09	8.94E-09	9.12E-09	8.64E-09	9.01E-09	8.30E-09	8.04E-09	8.53E-09
1.13E-08	1.04E-08	1.00E-08	1.02E-08	9.79E-09	9.77E-09	9.65E-09	9.84E-09	9.76E-09	9.17E-09	1.00E-08	9.17E-09	8.89E-09	9.25E-09
1.18E-08	1.13E-08	1.07E-08	1.11E-08	1.06E-08	1.05E-08	1.06E-08	1.12E-08	1.06E-08	1.00E-08	1.09E-08	1.00E-08	1.00E-08	1.04E-08
1.28E-08	1.27E-08	1.18E-08	1.24E-08	1.13E-08	1.12E-08	1.14E-08	1.22E-08	1.15E-08	1.09E-08	1.15E-08	1.09E-08	1.10E-08	1.15E-08
1.32E-08	1.36E-08	1.30E-08	1.32E-08	1.21E-08	1.24E-08	1.23E-08	1.24E-08	1.21E-08	1.20E-08	1.20E-08	1.18E-08	1.22E-08	1.21E-08
1.32E-08	1.40E-08	1.36E-08	1.40E-08	1.32E-08	1.36E-08	1.32E-08	1.30E-08	1.34E-08	1.33E-08	1.28E-08	1.30E-08	1.32E-08	1.29E-08
1.40E-08	1.47E-08	1.43E-08	1.47E-08	1.42E-08	1.47E-08	1.40E-08	1.34E-08	1.43E-08	1.42E-08	1.35E-08	1.41E-08	1.40E-08	1.36E-08
1.49E-08	1.54E-08	1.52E-08	1.55E-08	1.52E-08	1.52E-08	1.50E-08	1.42E-08	1.50E-08	1.45E-08	1.45E-08	1.48E-08	1.48E-08	1.44E-08
1.62E-08	1.60E-08	1.64E-08	1.65E-08	1.57E-08	1.57E-08	1.60E-08	1.54E-08	1.55E-08	1.52E-08	1.58E-08	1.52E-08	1.52E-08	1.54E-08
1.72E-08	1.67E-08	1.73E-08	1.74E-08	1.64E-08	1.62E-08	1.71E-08	1.61E-08	1.61E-08	1.64E-08	1.68E-08	1.59E-08	1.60E-08	1.63E-08
1.83E-08	1.77E-08	1.77E-08	1.76E-08	1.74E-08	1.70E-08	1.79E-08	1.71E-08	1.70E-08	1.70E-08	1.73E-08	1.70E-08	1.70E-08	1.73E-08
1.90E-08	1.84E-08	1.84E-08	1.81E-08	1.83E-08	1.81E-08	1.89E-08	1.79E-08	1.80E-08	1.79E-08	1.81E-08	1.81E-08	1.82E-08	1.84E-08
1.95E-08	1.92E-08	1.92E-08	1.94E-08	1.94E-08	1.93E-08	1.95E-08	1.89E-08	1.89E-08	1.89E-08	1.91E-08	1.92E-08	1.94E-08	1.89E-08
2.01E-08	2.00E-08	2.03E-08	2.06E-08	2.04E-08	2.02E-08	2.03E-08	2.00E-08	1.98E-08	1.97E-08	2.00E-08	2.01E-08	2.00E-08	1.95E-08
2.11E-08	2.10E-08	2.15E-08	2.21E-08	2.13E-08	2.12E-08	2.11E-08	2.05E-08	2.06E-08	2.07E-08	2.09E-08	2.07E-08	2.06E-08	2.04E-08
2.23E-08	2.22E-08	2.25E-08	2.32E-08	2.26E-08	2.24E-08	2.22E-08	2.13E-08	2.17E-08	2.19E-08	2.19E-08	2.14E-08	2.16E-08	2.16E-08
2.32E-08	2.35E-08	2.39E-08	2.37E-08	2.37E-08	2.34E-08	2.28E-08	2.23E-08	2.26E-08	2.32E-08	2.28E-08	2.21E-08	2.27E-08	2.29E-08
2.41E-08	2.46E-08	2.47E-08	2.49E-08	2.47E-08	2.44E-08	2.36E-08	2.31E-08	2.34E-08	2.42E-08	2.37E-08	2.30E-08	2.40E-08	2.41E-08
2.56E-08	2.58E-08	2.57E-08	2.60E-08	2.61E-08	2.54E-08	2.45E-08	2.42E-08	2.44E-08	2.52E-08	2.46E-08	2.43E-08	2.53E-08	2.51E-08
2.71E-08	2.67E-08	2.69E-08	2.73E-08	2.77E-08	2.66E-08	2.58E-08	2.55E-08	2.55E-08	2.65E-08	2.57E-08	2.56E-08	2.68E-08	2.57E-08
2.82E-08	2.78E-08	2.77E-08	2.79E-08	2.84E-08	2.77E-08	2.75E-08	2.70E-08	2.68E-08	2.77E-08	2.69E-08	2.71E-08	2.80E-08	2.66E-08
2.92E-08	2.88E-08	2.84E-08	2.91E-08	2.95E-08	2.87E-08	2.93E-08	2.84E-08	2.82E-08	2.87E-08	2.80E-08	2.82E-08	2.87E-08	2.78E-08

5.77E-17	5.29E-17	5.71E-17	4.81E-17	4.57E-17	2.93E-17	3.30E-17	3.02E-17	2.96E-17	2.27E-17	1.54E-17	1.09E-17	5.93E-18	4.60E-18
5.52E-17	5.01E-17	4.81E-17	4.61E-17	4.67E-17	2.89E-17	2.79E-17	2.24E-17	2.67E-17	1.95E-17	1.64E-17	1.11E-17	6.06E-18	1.83E-18
4.95E-17	4.98E-17	4.11E-17	3.94E-17	4.34E-17	2.77E-17	2.94E-17	1.85E-17	2.02E-17	1.95E-17	1.88E-17	8.73E-18	6.42E-18	1.33E-18
4.06E-17	4.37E-17	3.96E-17	3.75E-17	3.83E-17	2.97E-17	3.10E-17	2.33E-17	2.41E-17	1.78E-17	1.65E-17	9.23E-18	8.10E-18	3.89E-18
3.37E-17	3.33E-17	4.20E-17	3.86E-17	3.68E-17	3.31E-17	3.27E-17	2.88E-17	2.76E-17	1.82E-17	1.39E-17	1.07E-17	9.33E-18	6.07E-18
3.18E-17	2.79E-17	4.78E-17	4.83E-17	3.75E-17	3.72E-17	3.40E-17	2.66E-17	2.92E-17	2.32E-17	1.80E-17	1.06E-17	9.86E-18	6.50E-18
4.16E-17	3.61E-17	5.44E-17	5.22E-17	3.98E-17	4.25E-17	3.90E-17	2.69E-17	2.98E-17	2.61E-17	2.25E-17	1.01E-17	9.49E-18	7.18E-18
4.67E-17	5.00E-17	5.83E-17	5.96E-17	4.36E-17	4.90E-17	3.95E-17	3.16E-17	3.08E-17	3.08E-17	2.40E-17	1.06E-17	1.00E-17	7.84E-18
5.19E-17	5.50E-17	5.78E-17	5.93E-17	4.51E-17	5.13E-17	4.25E-17	3.86E-17	3.58E-17	3.10E-17	2.34E-17	1.35E-17	1.13E-17	8.18E-18
5.87E-17	4.96E-17	6.11E-17	6.20E-17	5.43E-17	4.84E-17	4.33E-17	3.73E-17	3.95E-17	2.68E-17	2.15E-17	1.64E-17	9.66E-18	8.99E-18
5.86E-17	4.80E-17	5.82E-17	4.97E-17	5.31E-17	4.73E-17	4.10E-17	3.29E-17	3.41E-17	2.46E-17	2.48E-17	2.04E-17	1.23E-17	1.10E-17
5.82E-17	4.27E-17	4.94E-17	4.25E-17	4.86E-17	4.23E-17	3.65E-17	2.77E-17	3.21E-17	2.40E-17	2.60E-17	2.69E-17	1.73E-17	1.48E-17
6.77E-17	4.10E-17	4.56E-17	3.84E-17	4.25E-17	4.26E-17	3.66E-17	2.54E-17	2.78E-17	2.24E-17	2.66E-17	3.01E-17	2.10E-17	1.80E-17
6.33E-17	4.77E-17	4.89E-17	4.58E-17	4.02E-17	4.92E-17	4.13E-17	2.78E-17	2.37E-17	2.02E-17	2.69E-17	3.20E-17	1.90E-17	2.01E-17
5.84E-17	5.23E-17	3.90E-17	4.66E-17	3.89E-17	4.87E-17	4.61E-17	2.78E-17	2.37E-17	1.71E-17	2.36E-17	3.09E-17	1.69E-17	1.88E-17
5.38E-17	4.99E-17	2.76E-17	4.25E-17	4.42E-17	4.88E-17	4.88E-17	2.90E-17	1.95E-17	1.53E-17	2.30E-17	2.26E-17	1.43E-17	2.08E-17
5.10E-17	4.55E-17	2.46E-17	3.81E-17	4.49E-17	4.73E-17	4.49E-17	2.88E-17	2.45E-17	2.12E-17	2.73E-17	1.73E-17	1.14E-17	1.90E-17
4.49E-17	4.30E-17	3.44E-17	3.88E-17	4.49E-17	5.04E-17	3.84E-17	2.90E-17	3.08E-17	3.05E-17	2.86E-17	1.95E-17	1.13E-17	1.50E-17
4.25E-17	4.93E-17	4.26E-17	4.57E-17	4.69E-17	4.49E-17	3.48E-17	2.99E-17	3.20E-17	3.11E-17	2.82E-17	2.09E-17	1.96E-17	1.46E-17
3.80E-17	5.11E-17	4.57E-17	4.51E-17	4.15E-17	3.25E-17	3.40E-17	3.28E-17	2.79E-17	2.86E-17	2.64E-17	2.24E-17	3.21E-17	1.86E-17
3.66E-17	4.85E-17	4.66E-17	4.44E-17	3.89E-17	3.24E-17	3.47E-17	3.28E-17	2.58E-17	2.74E-17	2.72E-17	2.82E-17	3.44E-17	2.34E-17
3.55E-17	4.50E-17	3.83E-17	4.77E-17	4.54E-17	3.79E-17	3.86E-17	3.65E-17	3.12E-17	2.97E-17	3.08E-17	3.53E-17	3.32E-17	2.72E-17
4.45E-17	4.62E-17	2.89E-17	4.49E-17	4.97E-17	3.96E-17	4.28E-17	4.14E-17	3.59E-17	3.38E-17	3.27E-17	3.29E-17	3.16E-17	2.63E-17
4.98E-17	5.04E-17	2.12E-17	4.72E-17	4.63E-17	4.06E-17	4.61E-17	4.36E-17	3.84E-17	3.48E-17	3.30E-17	3.30E-17	2.71E-17	2.99E-17
5.42E-17	5.25E-17	2.77E-17	4.36E-17	4.84E-17	4.42E-17	4.45E-17	4.35E-17	4.14E-17	4.11E-17	3.53E-17	3.69E-17	3.00E-17	4.74E-17
5.85E-17	5.01E-17	4.67E-17	4.45E-17	5.19E-17	4.94E-17	4.26E-17	4.08E-17	4.56E-17	4.43E-17	3.73E-17	3.94E-17	2.95E-17	6.74E-17
5.82E-17	5.48E-17	4.86E-17	4.52E-17	5.49E-17	5.48E-17	4.02E-17	3.84E-17	4.31E-17	4.11E-17	3.81E-17	4.11E-17	3.39E-17	6.27E-17
5.77E-17	5.85E-17	5.31E-17	4.82E-17	6.02E-17	5.37E-17	4.17E-17	4.08E-17	3.88E-17	4.06E-17	3.97E-17	3.57E-17	3.49E-17	4.39E-17
5.96E-17	5.93E-17	5.67E-17	5.24E-17	6.18E-17	4.73E-17	4.50E-17	4.07E-17	3.72E-17	4.61E-17	3.81E-17	3.15E-17	3.42E-17	4.27E-17
6.36E-17	6.14E-17	5.90E-17	5.35E-17	6.26E-17	5.09E-17	5.03E-17	4.58E-17	4.28E-17	5.10E-17	4.24E-17	3.39E-17	3.56E-17	4.03E-17
6.14E-17	5.89E-17	5.70E-17	5.11E-17	6.34E-17	5.60E-17	5.60E-17	4.83E-17	4.10E-17	4.92E-17	4.47E-17	4.28E-17	3.83E-17	3.91E-17

6.00E-17	5.39E-17	5.20E-17	5.03E-17	6.75E-17	6.01E-17	5.51E-17	5.15E-17	4.18E-17	4.87E-17	4.47E-17	4.46E-17	4.11E-17	3.56E-17
5.60E-17	6.02E-17	5.29E-17	5.95E-17	7.24E-17	6.17E-17	5.24E-17	5.27E-17	4.25E-17	4.12E-17	3.89E-17	4.10E-17	4.51E-17	3.46E-17
5.40E-17	6.51E-17	6.05E-17	6.58E-17	6.94E-17	5.80E-17	4.90E-17	5.66E-17	4.66E-17	4.32E-17	3.18E-17	3.93E-17	4.44E-17	3.87E-17
5.69E-17	6.59E-17	6.02E-17	6.26E-17	6.33E-17	5.09E-17	4.93E-17	6.21E-17	5.30E-17	5.10E-17	3.60E-17	3.76E-17	4.29E-17	4.29E-17
5.61E-17	6.79E-17	6.18E-17	5.99E-17	5.92E-17	4.84E-17	5.50E-17	6.23E-17	6.09E-17	4.92E-17	3.97E-17	3.77E-17	4.17E-17	4.18E-17
6.46E-17	6.91E-17	7.05E-17	5.70E-17	5.72E-17	5.27E-17	5.72E-17	6.50E-17	5.84E-17	4.29E-17	4.20E-17	4.40E-17	4.45E-17	4.20E-17
7.40E-17	6.85E-17	6.90E-17	5.38E-17	5.75E-17	5.65E-17	5.76E-17	6.72E-17	5.76E-17	3.75E-17	4.30E-17	5.36E-17	5.00E-17	4.23E-17
7.01E-17	6.54E-17	6.39E-17	4.59E-17	5.53E-17	5.94E-17	5.84E-17	6.19E-17	5.75E-17	3.31E-17	4.85E-17	5.84E-17	5.75E-17	4.51E-17
6.63E-17	5.98E-17	5.66E-17	6.04E-17	5.42E-17	5.64E-17	5.84E-17	5.60E-17	4.88E-17	3.32E-17	4.93E-17	5.42E-17	5.87E-17	4.31E-17
6.38E-17	6.10E-17	5.60E-17	5.97E-17	5.29E-17	5.89E-17	6.33E-17	5.69E-17	4.80E-17	3.48E-17	4.80E-17	5.21E-17	5.61E-17	4.07E-17
6.56E-17	5.88E-17	5.88E-17	6.40E-17	5.35E-17	6.10E-17	6.10E-17	5.63E-17	5.08E-17	3.66E-17	4.44E-17	5.18E-17	5.64E-17	4.59E-17
6.59E-17	6.11E-17	6.06E-17	6.28E-17	5.50E-17	6.68E-17	6.01E-17	4.82E-17	4.77E-17	4.07E-17	4.85E-17	4.97E-17	5.38E-17	5.10E-17
5.38E-17	6.01E-17	6.23E-17	6.05E-17	5.68E-17	6.41E-17	6.11E-17	4.53E-17	4.51E-17	3.94E-17	4.73E-17	4.64E-17	4.94E-17	4.87E-17
4.69E-17	5.52E-17	5.82E-17	6.56E-17	6.23E-17	5.63E-17	5.54E-17	4.68E-17	5.01E-17	4.38E-17	4.60E-17	4.10E-17	4.26E-17	4.63E-17
3.93E-17	5.36E-17	5.12E-17	6.15E-17	6.08E-17	5.47E-17	5.24E-17	5.04E-17	5.84E-17	5.47E-17	5.15E-17	4.48E-17	3.92E-17	4.28E-17
3.56E-17	5.70E-17	4.91E-17	5.68E-17	5.72E-17	5.61E-17	5.34E-17	5.56E-17	6.35E-17	5.61E-17	5.54E-17	4.70E-17	4.18E-17	4.40E-17
3.74E-17	5.41E-17	5.13E-17	5.45E-17	5.22E-17	5.55E-17	5.43E-17	4.77E-17	6.16E-17	5.17E-17	5.20E-17	4.95E-17	4.51E-17	4.75E-17
3.14E-17	4.77E-17	5.58E-17	5.76E-17	4.71E-17	4.80E-17	5.33E-17	3.62E-17	5.93E-17	4.80E-17	5.27E-17	5.34E-17	4.81E-17	4.64E-17
3.57E-17	4.91E-17	5.68E-17	5.66E-17	4.47E-17	4.14E-17	4.63E-17	9.71E-18	5.10E-17	4.29E-17	5.11E-17	5.21E-17	4.64E-17	4.61E-17
4.16E-17	4.99E-17	5.87E-17	5.51E-17	4.56E-17	3.31E-17	3.63E-17	7.57E-19	4.79E-17	3.86E-17	4.86E-17	5.20E-17	4.45E-17	4.50E-17
4.82E-17	4.74E-17	5.15E-17	5.08E-17	3.99E-17	4.16E-17	2.85E-17	2.82E-18	4.46E-17	3.80E-17	4.88E-17	5.14E-17	4.01E-17	4.15E-17
4.56E-17	4.23E-17	4.20E-17	4.28E-17	3.87E-17	4.71E-17	3.91E-17	4.10E-17	4.58E-17	3.78E-17	5.11E-17	4.79E-17	4.02E-17	3.87E-17
4.22E-17	4.00E-17	4.21E-17	4.29E-17	3.98E-17	4.61E-17	4.27E-17	4.42E-17	4.58E-17	3.48E-17	4.85E-17	4.47E-17	3.88E-17	3.62E-17
4.10E-17	4.22E-17	4.45E-17	4.52E-17	4.35E-17	4.33E-17	4.32E-17	4.42E-17	4.67E-17	3.73E-17	5.02E-17	3.72E-17	3.59E-17	3.59E-17
4.08E-17	4.22E-17	5.21E-17	5.32E-17	5.30E-17	4.57E-17	4.95E-17	4.41E-17	4.50E-17	4.40E-17	4.62E-17	2.72E-17	3.19E-17	3.57E-17
3.67E-17	4.01E-17	5.08E-17	5.40E-17	5.17E-17	4.84E-17	4.78E-17	4.03E-17	4.32E-17	3.93E-17	3.82E-17	2.88E-17	3.48E-17	3.75E-17
3.32E-17	4.18E-17	4.79E-17	5.09E-17	4.77E-17	4.43E-17	4.11E-17	3.76E-17	3.77E-17	3.05E-17	2.99E-17	3.00E-17	4.09E-17	4.03E-17
3.27E-17	4.57E-17	4.54E-17	5.22E-17	4.48E-17	3.85E-17	3.63E-17	3.78E-17	3.04E-17	2.91E-17	2.87E-17	3.33E-17	4.13E-17	4.55E-17
3.44E-17	4.19E-17	4.66E-17	4.59E-17	4.30E-17	3.58E-17	3.72E-17	3.74E-17	2.65E-17	3.40E-17	2.77E-17	2.95E-17	3.74E-17	4.58E-17
4.32E-17	3.53E-17	4.43E-17	3.65E-17	3.76E-17	2.92E-17	3.06E-17	3.67E-17	2.55E-17	3.79E-17	2.52E-17	3.45E-17	3.54E-17	3.83E-17
4.38E-17	2.86E-17	3.88E-17	2.95E-17	3.41E-17	2.57E-17	2.74E-17	3.70E-17	3.14E-17	4.03E-17	2.45E-17	3.25E-17	2.96E-17	3.27E-17
3.68E-17	2.48E-17	3.84E-17	3.07E-17	3.92E-17	3.32E-17	2.74E-17	3.66E-17	2.83E-17	3.94E-17	2.81E-17	3.19E-17	3.02E-17	3.01E-17
3.58E-17	2.91E-17	3.99E-17	3.60E-17	4.41E-17	3.51E-17	3.10E-17	3.59E-17	2.35E-17	3.71E-17	2.72E-17	3.12E-17	3.69E-17	3.17E-17
3.56E-17	3.31E-17	4.33E-17	4.11E-17	3.96E-17	3.53E-17	2.99E-17	3.18E-17	2.40E-17	3.42E-17	2.39E-17	2.96E-17	3.76E-17	2.95E-17
3.59E-17	3.91E-17	4.57E-17	4.38E-17	3.55E-17	3.29E-17	2.60E-17	3.09E-17	2.96E-17	3.04E-17	2.52E-17	2.67E-17	3.43E-17	2.52E-17
3.51E-17	3.51E-17	4.20E-17	4.02E-17	3.59E-17	3.19E-17	1.96E-17	3.01E-17	2.62E-17	2.49E-17	2.71E-17	2.32E-17	3.09E-17	2.12E-17
3.55E-17	3.32E-17	3.62E-17	3.74E-17	3.87E-17	2.73E-17	1.50E-17	2.27E-17	1.95E-17	2.16E-17	2.25E-17	1.67E-17	2.93E-17	1.95E-17
2.93E-17	3.45E-17	3.56E-17	3.51E-17	3.64E-17	2.29E-17	1.17E-17	1.76E-17	1.38E-17	1.94E-17	1.81E-17	1.45E-17	2.70E-17	2.43E-17
2.44E-17	2.48E-17	3.15E-17	3.09E-17	2.94E-17	1.52E-17	1.03E-17	1.81E-17	1.05E-17	1.78E-17	1.59E-17	1.51E-17	2.38E-17	2.07E-17
2.32E-17	1.64E-17	2.62E-17	2.19E-17	2.34E-17	1.42E-17	1.01E-17	2.06E-17	1.54E-17	1.90E-17	1.66E-17	1.55E-17	1.78E-17	1.65E-17
2.20E-17	1.72E-17	2.18E-17	1.58E-17	2.01E-17	1.40E-17	1.45E-17	2.01E-17	1.78E-17	2.03E-17	1.75E-17	1.73E-17	1.63E-17	1.65E-17
1.72E-17	1.61E-17	2.01E-17	1.58E-17	1.49E-17	1.09E-17	1.46E-17	1.95E-17	1.56E-17	1.92E-17	1.93E-17	1.54E-17	1.50E-17	1.74E-17
1.31E-17	1.35E-17	2.10E-17	1.37E-17	1.29E-17	1.17E-17	1.41E-17	1.62E-17	1.45E-17	1.62E-17	1.73E-17	1.45E-17	1.50E-17	1.63E-17
1.12E-17	1.74E-17	2.18E-17	1.29E-17	1.23E-17	1.32E-17	1.41E-17	1.32E-17	1.17E-17	1.71E-17	1.66E-17	1.45E-17	1.67E-17	1.61E-17
1.26E-17	1.81E-17	2.07E-17	1.61E-17	1.47E-17	1.61E-17	1.80E-17	1.08E-17	1.09E-17	1.66E-17	1.62E-17	1.33E-17	1.93E-17	1.53E-17
1.27E-17	1.60E-17	1.66E-17	1.51E-17	1.35E-17	1.60E-17	1.72E-17	9.62E-18	1.26E-17	1.35E-17	1.21E-17	9.68E-18	1.86E-17	1.35E-17
1.17E-17	1.02E-17	1.04E-17	1.09E-17	1.25E-17	1.51E-17	1.40E-17	1.01E-17	1.26E-17	1.17E-17	9.35E-18	6.74E-18	1.38E-17	1.01E-17
8.19E-18	7.23E-18	7.59E-18	9.14E-18	1.32E-17	1.23E-17	1.14E-17	1.04E-17	1.17E-17	1.13E-17	7.05E-18	4.04E-18	1.07E-17	8.85E-18
3.38E-18	4.37E-18	8.26E-18	8.19E-18	1.10E-17	9.16E-18	8.62E-18	1.15E-17	1.09E-17	1.04E-17	5.30E-18	3.72E-18	7.91E-18	8.66E-18
1.38E-18	3.99E-18	7.83E-18	8.28E-18	8.36E-18	6.10E-18	7.95E-18	8.79E-18	9.70E-18	8.77E-18	3.06E-18	4.54E-18	7.15E-18	7.95E-18
4.34E-19	3.15E-18	7.39E-18	6.86E-18	5.66E-18	3.89E-18	5.61E-18	6.31E-18	9.43E-18	6.47E-18	2.36E-18	6.42E-18	5.82E-18	7.11E-18
4.09E-21	2.54E-18	6.65E-18	5.68E-18	4.22E-18	2.78E-18	5.58E-18	5.30E-18	7.14E-18	4.58E-18	4.10E-18	6.58E-18	3.63E-18	5.81E-18
9.54E-20	1.59E-18	5.12E-18	4.23E-18	3.48E-18	2.11E-18	7.29E-18	4.85E-18	4.61E-18	3.43E-18	4.36E-18	3.90E-18	1.55E-18	4.31E-18
1.61E-20	8.52E-19	3.24E-18	1.85E-18	3.22E-18	1.75E-18	6.37E-18	3.74E-18	2.17E-18	2.03E-18	3.00E-18	2.30E-18	5.78E-19	2.13E-18
2.26E-19	2.98E-19	1.16E-18	8.74E-19	1.16E-18	1.73E-18	3.22E-18	2.19E-18	1.36E-18	9.92E-19	2.15E-18	1.63E-18	2.08E-19	3.40E-19
5.28E-20	1.17E-20	6.49E-19	7.06E-19	2.03E-19	5.81E-19	5.42E-19	5.11E-19	6.58E-19	5.53E-19	1.81E-18	1.37E-18	4.04E-19	3.32E-19
3.39E-19	1.30E-19	2.30E-19	1.14E-19	2.54E-19	2.01E-20	1.01E-21	2.09E-20	6.20E-22	1.00E-19	1.23E-18	5.99E-19	4.90E-19	4.78E-19
6.66E-19	5.04E-19	7.90E-22	2.84E-20	1.10E-18	4.07E-19	5.28E-19	6.92E-19	7.71E-19	1.92E-19	3.06E-19	3.32E-19	1.72E-19	4.16E-20
1.13E-18	1.39E-18	4.53E-19	3.04E-19	1.44E-18	2.54E-19	2.00E-18	1.89E-18	1.68E-18	6.94E-19	1.28E-20	2.37E-19	1.05E-20	8.60E-22
1.42E-18	3.24E-18	1.83E-18	1.10E-18	8.87E-19	4.40E-19	4.64E-19	1.64E-18	1.88E-18	5.22E-19	3.67E-19	1.32E-20	1.04E-23	2.85E-19
1.39E-18	2.81E-18	4.00E-18	2.18E-18	1.17E-18	3.78E-19	3.10E-19	2.04E-18	1.25E-18	2.27E-21	6.79E-19	3.87E-19	3.77E-19	8.64E-19
1.61E-18	3.73E-18	8.06E-18	2.95E-18	2.37E-18	1.12E-18	6.23E-19	3.81E-18	1.49E-18	4.73E-24	7.11E-19	2.11E-18	1.26E-18	2.08E-18
2.71E-18	6.14E-18	1.20E-17	2.81E-18	2.92E-18	2.09E-18	1.27E-18	5.86E-18	1.46E-18	9.33E-19	2.07E-18	4.89E-18	3.66E-18	4.60E-18
5.30E-18	8.04E-18	1.10E-17	4.31E-18	4.83E-18	3.95E-18	2.20E-18	5.83E-18	3.99E-18	3.46E-18	2.43E-18	5.44E-18	6.24E-18	5.64E-18
7.19E-18	1.48E-17	1.39E-17	6.77E-18	1.31E-17	7.84E-18	6.44E-18	7.91E-18	1.05E-17	6.58E-18	3.22E-18	4.02E-18	6.88E-18	4.27E-18
9.90E-18	1.47E-17	1.29E-17	9.76E-18	1.79E-17	8.99E-18	1.17E-17	9.59E-18	1.41E-17	9.92E-18	4.48E-18	7.08E-18	6.95E-18	5.22E-18
1.46E-17	1.41E-17	1.66E-17	1.29E-17	1.66E-17	1.40E-17	1.66E-17	1.07E-17	1.42E-17	1.16E-17	8.26E-18	1.31E-17	8.45E-18	8.19E-18
2.02E-17	1.83E-17	2.12E-17	1.78E-17	2.06E-17	2.01E-17	2.22E-17	1.47E-17	1.51E-17	1.47E-17	1.13E-17	2.38E-17	1.25E-17	1.20E-17
3.14E-17	2.48E-17	2.82E-17	2.60E-17	2.85E-17	2.32E-17	2.67E-17	1.87E-17	1.93E-17	1.78E-17	1.67E-17	2.69E-17	1.56E-17	1.27E-17
4.24E-17	3.73E-17	3.80E-17	3.03E-17	3.53E-17	2.80E-17	2.88E-17	2.28E-17	2.62E-17	2.58E-17	2.59E-17	2.99E-17	2.45E-17	1.84E-17
5.29E-17	4.71E-17	4.86E-17	3.93E-17	3.94E-17	3.61E-17	3.64E-17	2.40E-17	3.43E-17	3.28E-17	3.56E-17	4.47E-17	3.26E-17	2.64E-17
6.40E-17	5.55E-17	6.01E-17	4.92E-17	4.71E-17	4.43E-17	4.10E-17	3.32E-17	3.89E-17	4.05E-17	4.77E-17	5.36E-17	3.75E-17	3.45E-17
7.11E-17	6.30E-17	6.89E-17	5.29E-17	5.60E-17	5.43E-17	5.02E-17	4.45E-17	5.65E-17	4.99E-17	5.00E-17	4.52E-17	4.12E-17	5.04E-17
8.47E-17	7.31E-17	8.25E-17	5.65E-17	5.82E-17	6.20E-17	6.12E-17	5.39E-17	6.87E-17	5.78E-17	5.62E-17	4.97E-17	4.77E-17	6.11E-17
1.07E-16	8.51E-17	8.17E-17	7.12E-17	7.23E-17	7.05E-17	7.11E-17	6.54E-17	7.63E-17	6.10E-17	6.87E-17	5.77E-17	5.57E-17	6.41E-17
1.18E-16	9.58E-17	8.82E-17	8.81E-17	8.32E-17	8.34E-17	7.67E-17	8.00E-17	8.32E-17	7.47E-17	8.13E-17	6.89E-17	6.46E-17	7.27E-17
1.28E-16	1.08E-16	1.01E-16	1.04E-16	9.58E-17	9.54E-17	9.30E-17	9.69E-17	9.53E-17	8.42E-17	1.01E-16	8.41E-17	7.90E-17	8.55E-17
1.39E-16	1.27E-16	1.14E-16	1.24E-16	1.12E-16	1.09E-16	1.13E-16	1.25E-16	1.13E-16	1.00E-16	1.19E-16	1.00E-16	1.00E-16	1.08E-16
1.63E-16	1.60E-16	1.40E-16	1.53E-16	1.27E-16	1.25E-16	1.30E-16	1.48E-16	1.32E-16	1.20E-16	1.32E-16	1.18E-16	1.22E-16	1.32E-16
1.75E-16	1.86E-16	1.68E-16	1.75E-16	1.46E-16	1.55E-16	1.52E-16	1.53E-16	1.47E-16	1.45E-16	1.44E-16	1.38E-16	1.48E-16	1.46E-16
1.74E-16	1.96E-16	1.86E-16	1.97E-16	1.74E-16	1.84E-16	1.74E-16	1.68E-16	1.78E-16	1.78E-16	1.64E-16	1.69E-16	1.75E-16	1.67E-16
1.95E-16	2.16E-16	2.05E-16	2.17E-16	2.03E-16	2.15E-16	1.95E-16	1.80E-16	2.04E-16	2.02E-16	1.81E-16	1.98E-16	1.96E-16	1.84E-16
2.23E-16	2.38E-16	2.32E-16	2.40E-16	2.31E-16	2.32E-16	2.24E-16	2.01E-16	2.25E-16	2.10E-16	2.09E-16	2.20E-16	2.19E-16	2.07E-16
2.61E-16	2.55E-16	2.69E-16	2.73E-16	2.48E-16	2.46E-16	2.55E-16	2.37E-16	2.40E-16	2.30E-16	2.49E-16	2.31E-16	2.31E-16	2.37E-16
2.96E-16	2.77E-16	3.00E-16	3.02E-16	2.68E-16	2.63E-16	2.91E-16	2.60E-16	2.59E-16	2.70E-16	2.83E-16	2.52E-16	2.57E-16	2.66E-16
3.34E-16	3.14E-16	3.13E-16	3.11E-16	3.02E-16	2.89E-16	3.19E-16	2.92E-16	2.88E-16	2.90E-16	2.98E-16	2.89E-16	2.89E-16	3.00E-16

3.60E-16	3.39E-16	3.39E-16	3.27E-16	3.35E-16	3.28E-16	3.58E-16	3.22E-16	3.23E-16	3.20E-16	3.27E-16	3.28E-16	3.31E-16	3.37E-16
3.79E-16	3.69E-16	3.67E-16	3.74E-16	3.76E-16	3.73E-16	3.80E-16	3.57E-16	3.59E-16	3.57E-16	3.64E-16	3.70E-16	3.76E-16	3.59E-16
4.02E-16	3.99E-16	4.10E-16	4.26E-16	4.15E-16	4.09E-16	4.11E-16	3.99E-16	3.91E-16	3.88E-16	4.01E-16	4.05E-16	3.99E-16	3.82E-16
4.44E-16	4.40E-16	4.64E-16	4.90E-16	4.54E-16	4.49E-16	4.45E-16	4.21E-16	4.23E-16	4.28E-16	4.36E-16	4.27E-16	4.23E-16	4.17E-16
4.95E-16	4.92E-16	5.07E-16	5.36E-16	5.09E-16	5.00E-16	4.91E-16	4.52E-16	4.71E-16	4.79E-16	4.82E-16	4.60E-16	4.65E-16	4.65E-16
5.40E-16	5.52E-16	5.69E-16	5.60E-16	5.63E-16	5.49E-16	5.21E-16	4.96E-16	5.13E-16	5.37E-16	5.19E-16	4.87E-16	5.15E-16	5.22E-16
5.80E-16	6.05E-16	6.13E-16	6.18E-16	6.13E-16	5.94E-16	5.55E-16	5.34E-16	5.48E-16	5.85E-16	5.63E-16	5.27E-16	5.78E-16	5.81E-16
6.57E-16	6.63E-16	6.61E-16	6.74E-16	6.82E-16	6.46E-16	5.99E-16	5.84E-16	5.95E-16	6.36E-16	6.06E-16	5.91E-16	6.42E-16	6.28E-16
7.34E-16	7.13E-16	7.24E-16	7.43E-16	7.66E-16	7.10E-16	6.66E-16	6.50E-16	6.50E-16	7.03E-16	6.58E-16	6.56E-16	7.16E-16	6.58E-16
7.93E-16	7.75E-16	7.67E-16	7.80E-16	8.09E-16	7.66E-16	7.59E-16	7.27E-16	7.18E-16	7.65E-16	7.25E-16	7.35E-16	7.84E-16	7.07E-16
8.55E-16	8.32E-16	8.09E-16	8.44E-16	8.70E-16	8.22E-16	8.61E-16	8.09E-16	7.97E-16	8.25E-16	7.86E-16	7.97E-16	8.24E-16	7.71E-16
1.25E-14	1.24E-14	1.25E-14	1.25E-14	1.24E-14	1.18E-14	1.15E-14	1.08E-14	1.09E-14	1.08E-14	1.07E-14	1.04E-14	1.06E-14	1.04E-14

-2.57E-09	-2.20E-09	-5.63E-10	1.74E-09	3.36E-09	7.11E-09	1.18E-08	1.43E-08	1.09E-08	8.37E-09	8.53E-09	4.23E-09	6.26E-09	7.61E-09	8.20E-09
-2.15E-09	-2.28E-09	-4.44E-10	1.95E-09	3.98E-09	8.35E-09	1.37E-08	1.67E-08	1.31E-08	1.07E-08	1.21E-08	5.63E-09	7.67E-09	9.54E-09	1.01E-08
-2.38E-09	-1.82E-09	-5.57E-10	1.36E-09	3.97E-09	6.90E-09	1.22E-08	1.51E-08	1.39E-08	1.15E-08	1.43E-08	7.44E-09	8.47E-09	1.04E-08	1.07E-08
-2.73E-09	-1.68E-09	-8.52E-10	8.78E-10	3.82E-09	5.81E-09	1.02E-08	1.29E-08	1.20E-08	1.01E-08	8.76E-09	8.95E-09	8.54E-09	1.14E-08	1.18E-08
-2.82E-09	-1.34E-09	-6.28E-10	1.00E-09	2.51E-09	4.71E-09	7.40E-09	8.61E-09	8.34E-09	7.06E-09	4.67E-09	5.62E-09	5.82E-09	9.41E-09	1.09E-08
-2.51E-09	-1.36E-09	1.05E-10	1.25E-09	1.63E-09	2.64E-09	4.21E-09	3.56E-09	4.59E-09	4.97E-09	2.13E-09	2.96E-09	3.22E-09	5.39E-09	9.76E-09
-2.27E-09	-1.08E-09	3.23E-10	1.19E-09	8.16E-10	2.84E-10	1.74E-09	1.05E-09	1.98E-09	2.82E-09	9.01E-10	2.11E-09	2.76E-09	3.50E-09	8.27E-09
-2.40E-09	-1.13E-09	1.84E-10	3.65E-10	2.56E-10	-4.57E-10	4.54E-10	2.99E-10	9.19E-10	4.93E-10	4.83E-10	2.09E-09	2.66E-09	2.64E-09	5.92E-09
-2.32E-09	-1.74E-09	-6.56E-10	-1.25E-09	-2.33E-10	-4.52E-10	-4.35E-10	-2.70E-10	3.42E-10	-1.67E-10	-2.83E-10	1.12E-09	1.33E-09	1.26E-09	3.54E-09
-2.30E-09	-2.55E-09	-1.84E-09	-1.93E-09	-1.45E-09	-8.46E-10	-9.58E-10	-4.85E-10	4.61E-10	-8.05E-10	-7.70E-10	3.37E-10	6.71E-10	1.08E-09	2.76E-09
-2.45E-09	-3.22E-09	-2.23E-09	-1.22E-09	-2.23E-09	-1.91E-09	-1.53E-09	-4.61E-10	5.34E-10	-1.15E-09	-6.75E-10	6.42E-10	8.73E-10	1.82E-09	3.51E-09
-2.82E-09	-3.57E-09	-2.04E-09	-1.62E-09	-2.25E-09	-2.60E-09	-2.24E-09	-8.42E-10	8.14E-10	-9.34E-10	-1.78E-10	1.23E-09	1.60E-09	2.95E-09	4.28E-09
-3.51E-09	-3.83E-09	-1.98E-09	-2.87E-09	-2.27E-09	-2.99E-09	-2.13E-09	-7.86E-10	1.51E-09	-9.66E-10	4.25E-10	1.46E-09	2.47E-09	3.79E-09	4.58E-09
-3.65E-09	-3.37E-09	-2.15E-09	-3.45E-09	-2.35E-09	-2.91E-09	-1.76E-09	-8.25E-10	1.26E-09	-4.78E-10	1.49E-09	2.57E-09	3.43E-09	4.51E-09	5.29E-09
-3.47E-09	-2.82E-09	-3.11E-09	-2.88E-09	-1.97E-09	-2.80E-09	-1.78E-09	-9.88E-10	4.78E-10	1.28E-09	3.26E-09	3.59E-09	4.53E-09	4.43E-09	4.55E-09
-3.66E-09	-2.96E-09	-3.23E-09	-2.67E-09	-1.60E-09	-2.69E-09	-1.57E-09	-5.88E-10	-1.92E-10	1.44E-09	2.94E-09	2.92E-09	3.63E-09	3.59E-09	2.95E-09
-3.86E-09	-3.90E-09	-3.60E-09	-2.46E-09	-1.29E-09	-2.02E-09	-1.38E-09	-4.95E-10	-1.26E-10	8.52E-10	1.48E-09	1.61E-09	2.34E-09	2.27E-09	1.54E-09
-4.13E-09	-4.49E-09	-3.52E-09	-1.83E-09	-4.57E-10	-9.29E-10	-1.54E-09	-8.00E-10	6.55E-11	4.47E-10	-7.31E-11	6.34E-10	4.99E-10	5.56E-10	-3.46E-10
-3.91E-09	-4.05E-09	-3.14E-09	-2.12E-09	-1.88E-10	1.12E-10	-8.62E-10	-2.52E-10	6.52E-10	1.04E-09	-1.08E-09	-3.46E-10	-1.15E-09	-9.96E-10	-2.13E-09
-4.42E-09	-3.83E-09	-2.37E-09	-2.31E-09	-1.31E-09	4.23E-10	-2.72E-10	3.04E-10	4.00E-10	7.37E-10	-1.29E-09	-1.40E-09	-2.30E-09	-2.06E-09	-3.37E-09
-4.74E-09	-3.61E-09	-2.37E-09	-2.65E-09	-3.58E-09	-1.11E-09	-1.34E-09	-9.11E-10	-2.84E-10	-1.30E-09	-2.71E-09	-2.85E-09	-3.64E-09	-3.50E-09	-4.02E-09
-5.21E-09	-4.19E-09	-2.89E-09	-3.17E-09	-4.71E-09	-2.42E-09	-3.15E-09	-3.11E-09	-2.21E-09	-3.15E-09	-4.04E-09	-3.36E-09	-4.27E-09	-4.32E-09	-4.59E-09
-5.84E-09	-4.80E-09	-4.06E-09	-4.05E-09	-4.99E-09	-4.01E-09	-4.40E-09	-5.16E-09	-4.16E-09	-4.27E-09	-4.71E-09	-3.39E-09	-4.74E-09	-4.97E-09	-5.26E-09
-6.48E-09	-5.54E-09	-5.22E-09	-4.34E-09	-5.35E-09	-4.70E-09	-4.95E-09	-5.79E-09	-5.47E-09	-5.26E-09	-5.24E-09	-3.45E-09	-5.27E-09	-5.99E-09	-6.17E-09
-7.25E-09	-5.98E-09	-6.00E-09	-4.86E-09	-6.11E-09	-5.06E-09	-5.09E-09	-5.72E-09	-5.57E-09	-5.59E-09	-5.55E-09	-5.75E-09	-5.79E-09	-6.59E-09	-6.84E-09
-8.20E-09	-6.35E-09	-5.94E-09	-5.33E-09	-6.46E-09	-5.29E-09	-5.11E-09	-5.79E-09	-5.64E-09	-5.86E-09	-5.69E-09	-6.22E-09	-6.00E-09	-6.41E-09	-6.58E-09
-7.67E-09	-6.25E-09	-6.15E-09	-5.73E-09	-6.34E-09	-5.35E-09	-5.62E-09	-6.23E-09	-5.99E-09	-6.20E-09	-6.32E-09	-6.05E-09	-5.39E-09	-6.11E-09	-6.70E-09
-5.88E-09	-5.96E-09	-6.37E-09	-5.73E-09	-6.45E-09	-6.35E-09	-6.35E-09	-6.72E-09	-6.41E-09	-6.82E-09	-6.66E-09	-5.74E-09	-5.36E-09	-5.70E-09	-6.54E-09
-6.31E-09	-6.10E-09	-6.55E-09	-5.78E-09	-6.40E-09	-7.09E-09	-6.55E-09	-6.75E-09	-6.86E-09	-7.13E-09	-6.69E-09	-6.09E-09	-5.97E-09	-6.38E-09	-7.08E-09
-6.20E-09	-6.42E-09	-6.76E-09	-5.78E-09	-6.19E-09	-6.89E-09	-6.93E-09	-7.29E-09	-7.45E-09	-6.93E-09	-7.26E-09	-6.79E-09	-7.41E-09	-7.73E-09	-7.86E-09
-6.26E-09	-6.38E-09	-6.82E-09	-5.78E-09	-6.18E-09	-6.77E-09	-6.93E-09	-7.57E-09	-7.98E-09	-7.05E-09	-7.19E-09	-6.87E-09	-8.11E-09	-8.21E-09	-8.38E-09
-6.66E-09	-6.28E-09	-6.72E-09	-5.68E-09	-6.18E-09	-6.61E-09	-6.93E-09	-7.54E-09	-7.72E-09	-7.36E-09	-7.13E-09	-6.92E-09	-8.17E-09	-8.00E-09	-8.48E-09
-6.66E-09	-6.56E-09	-7.03E-09	-5.81E-09	-6.37E-09	-6.05E-09	-6.89E-09	-7.61E-09	-7.71E-09	-7.75E-09	-7.35E-09	-7.58E-09	-8.24E-09	-8.00E-09	-8.49E-09
-6.62E-09	-6.64E-09	-7.24E-09	-6.47E-09	-6.77E-09	-6.28E-09	-7.21E-09	-7.62E-09	-7.95E-09	-8.52E-09	-7.83E-09	-8.10E-09	-8.30E-09	-8.44E-09	-8.53E-09
-6.89E-09	-6.41E-09	-7.52E-09	-6.78E-09	-6.75E-09	-6.65E-09	-7.26E-09	-7.08E-09	-8.09E-09	-8.73E-09	-7.73E-09	-8.67E-09	-8.66E-09	-8.70E-09	-8.62E-09
-6.77E-09	-6.19E-09	-7.28E-09	-6.93E-09	-6.60E-09	-6.90E-09	-7.43E-09	-6.84E-09	-8.05E-09	-8.76E-09	-7.76E-09	-8.80E-09	-8.88E-09	-8.76E-09	-8.43E-09
-6.60E-09	-6.35E-09	-7.01E-09	-6.71E-09	-6.61E-09	-7.54E-09	-8.14E-09	-7.31E-09	-8.29E-09	-8.97E-09	-8.11E-09	-8.88E-09	-8.81E-09	-9.11E-09	-8.55E-09
-6.58E-09	-6.19E-09	-7.05E-09	-7.27E-09	-7.00E-09	-7.71E-09	-9.10E-09	-7.69E-09	-8.57E-09	-8.70E-09	-8.53E-09	-8.96E-09	-8.75E-09	-9.01E-09	-8.54E-09
-6.86E-09	-6.12E-09	-7.28E-09	-7.54E-09	-7.02E-09	-7.68E-09	-8.78E-09	-7.79E-09	-8.25E-09	-8.15E-09	-8.74E-09	-9.40E-09	-8.63E-09	-9.09E-09	-8.75E-09
-6.78E-09	-6.40E-09	-7.58E-09	-7.43E-09	-6.94E-09	-7.39E-09	-8.72E-09	-7.86E-09	-7.87E-09	-7.84E-09	-8.76E-09	-9.35E-09	-8.63E-09	-8.85E-09	-8.70E-09
-6.62E-09	-6.91E-09	-7.77E-09	-7.41E-09	-7.11E-09	-7.29E-09	-8.90E-09	-7.77E-09	-7.71E-09	-7.56E-09	-8.82E-09	-9.08E-09	-8.44E-09	-8.37E-09	-8.78E-09
-6.49E-09	-7.00E-09	-7.16E-09	-7.72E-09	-7.57E-09	-7.10E-09	-8.53E-09	-7.46E-09	-7.81E-09	-7.81E-09	-8.66E-09	-8.55E-09	-8.00E-09	-8.20E-09	-7.93E-09
-6.50E-09	-6.80E-09	-6.84E-09	-7.69E-09	-7.66E-09	-7.50E-09	-8.01E-09	-7.55E-09	-7.58E-09	-7.98E-09	-8.58E-09	-8.38E-09	-8.08E-09	-8.20E-09	-7.30E-09
-6.72E-09	-6.78E-09	-7.09E-09	-7.82E-09	-7.66E-09	-7.72E-09	-7.86E-09	-8.12E-09	-8.16E-09	-8.34E-09	-8.47E-09	-8.39E-09	-8.13E-09	-7.92E-09	-6.51E-09
-6.79E-09	-6.99E-09	-7.08E-09	-8.12E-09	-7.26E-09	-7.87E-09	-8.33E-09	-8.31E-09	-8.27E-09	-8.31E-09	-8.42E-09	-8.69E-09	-8.06E-09	-7.45E-09	-7.06E-09
-6.87E-09	-7.17E-09	-7.21E-09	-8.01E-09	-7.32E-09	-7.90E-09	-8.40E-09	-8.27E-09	-8.22E-09	-8.07E-09	-8.29E-09	-8.38E-09	-8.34E-09	-7.85E-09	-7.81E-09
-6.92E-09	-7.20E-09	-7.48E-09	-8.10E-09	-7.36E-09	-7.93E-09	-7.84E-09	-7.75E-09	-7.83E-09	-7.61E-09	-8.16E-09	-8.26E-09	-8.30E-09	-8.30E-09	-8.58E-09
-6.91E-09	-7.31E-09	-7.50E-09	-7.69E-09	-7.36E-09	-7.92E-09	-7.11E-09	-7.07E-09	-7.86E-09	-7.26E-09	-8.43E-09	-8.44E-09	-8.60E-09	-8.48E-09	-9.14E-09
-6.68E-09	-7.58E-09	-7.56E-09	-7.29E-09	-7.06E-09	-7.66E-09	-6.71E-09	-6.91E-09	-7.73E-09	-7.11E-09	-8.62E-09	-8.49E-09	-8.58E-09	-8.89E-09	-8.97E-09
-6.63E-09	-7.32E-09	-7.24E-09	-6.96E-09	-7.05E-09	-7.69E-09	-6.66E-09	-6.67E-09	-7.68E-09	-7.20E-09	-9.14E-09	-8.89E-09	-8.06E-09	-8.38E-09	-8.57E-09
-6.51E-09	-7.04E-09	-7.15E-09	-6.51E-09	-7.67E-09	-7.80E-09	-7.07E-09	-6.99E-09	-7.51E-09	-7.15E-09	-9.15E-09	-9.70E-09	-7.67E-09	-7.20E-09	-7.51E-09
-6.62E-09	-6.89E-09	-7.03E-09	-6.54E-09	-7.54E-09	-7.92E-09	-7.69E-09	-7.35E-09	-7.53E-09	-7.18E-09	-8.59E-09	-9.25E-09	-8.22E-09	-6.17E-09	-5.83E-09
-6.73E-09	-7.09E-09	-7.01E-09	-6.32E-09	-7.52E-09	-7.62E-09	-7.85E-09	-7.83E-09	-8.01E-09	-7.34E-09	-8.39E-09	-8.94E-09	-8.40E-09	-4.63E-09	-2.57E-09
-7.00E-09	-7.20E-09	-7.01E-09	-6.82E-09	-7.68E-09	-7.72E-09	-8.04E-09	-7.92E-09	-8.51E-09	-7.84E-09	-8.40E-09	-8.69E-09	-8.64E-09	-1.40E-09	3.03E-09
-7.25E-09	-7.55E-09	-7.36E-09	-7.39E-09	-8.03E-09	-7.86E-09	-8.43E-09	-8.12E-09	-8.51E-09	-8.28E-09	-8.48E-09	-8.60E-09	-8.76E-09	1.54E-09	8.94E-09
-7.07E-09	-7.42E-09	-7.27E-09	-7.69E-09	-8.06E-09	-7.84E-09	-8.50E-09	-8.07E-09	-8.13E-09	-8.43E-09	-8.30E-09	-8.33E-09	-8.67E-09	-7.87E-09	-2.41E-09
-6.67E-09	-7.42E-09	-7.21E-09	-7.91E-09	-7.65E-09	-7.84E-09	-8.62E-09	-8.09E-09	-7.98E-09	-8.05E-09	-7.82E-09	-8.06E-09	-8.17E-09	-8.67E-09	-8.41E-09
-6.34E-09	-7.45E-09	-7.07E-09	-7.57E-09	-7.35E-09	-7.79E-09	-8.27E-09	-8.17E-09	-7.75E-09	-7.68E-09	-7.72E-09	-7.70E-09	-7.99E-09	-8.18E-09	-7.73E-09
-6.40E-09	-6.90E-09	-6.28E-09	-7.13E-09	-7.28E-09	-7.81E-09	-7.75E-09	-7.83E-09	-7.43E-09	-7.24E-09	-7.88E-09	-7.30E-09	-8.21E-09	-8.28E-09	-7.82E-09
-6.24E-09	-6.37E-09	-5.20E-09	-6.61E-09	-6.83E-09	-7.65E-09	-7.53E-09	-7.25E-09	-7.33E-09	-6.93E-09	-7.63E-09	-7.42E-09	-8.18E-09	-8.73E-09	-8.06E-09
-5.84E-09	-6.12E-09	-4.62E-09	-6.23E-09	-6.56E-09	-7.35E-09	-7.53E-09	-6.62E-09	-7.36E-09	-7.11E-09	-7.11E-09	-7.61E-09	-8.20E-09	-8.72E-09	-8.25E-09
-5.50E-09	-6.07E-09	-5.52E-09	-6.32E-09	-6.16E-09	-7.16E-09	-7.01E-09	-6.23E-09	-7.45E-09	-7.60E-09	-7.21E-09	-7.37E-09	-7.75E-09	-8.61E-09	-8.10E-09
-5.63E-09	-6.08E-09	-6.15E-09	-6.56E-09	-6.34E-09	-6.85E-09	-6.70E-09	-6.81E-09	-7.50E-09	-7.43E-09	-7.33E-09	-6.76E-09	-7.05E-09	-7.92E-09	-7.62E-09
-5.56E-09	-5.72E-09	-6.26E-09	-6.44E-09	-6.42E-09	-6.21E-09	-6.29E-09	-7.15E-09	-7.20E-09	-7.32E-09	-7.00E-09	-6.35E-09	-6.49E-09	-7.28E-09	-7.54E-09
-5.58E-09	-5.46E-09	-6.10E-09	-6.03E-09	-6.38E-09	-5.93E-09	-6.18E-09	-7.24E-09	-6.65E-09	-6.80E-09	-6.40E-09	-6.06E-09	-6.22E-09	-6.54E-09	-6.80E-09
-5.11E-09	-4.71E-09	-6.13E-09	-5.51E-09	-6.13E-09	-5.50E-09	-5.93E-09	-7.22E-09	-6.28E-09	-6.49E-09	-5.80E-09	-5.62E-09	-5.64E-09	-5.43E-09	-6.24E-09
-5.00E-09	-4.37E-09	-5.37E-09	-4.63E-09	-5.68E-09	-5.27E-09	-5.91E-09	-7.11E-09	-6.40E-09	-6.83E-09	-5.45E-09	-5.15E-09	-4.81E-09	-4.63E-09	-5.78E-09
-5.05E-09	-4.79E-09	-5.41E-09	-4.40E-09	-4.95E-09	-5.53E-09	-5.96E-09	-6.60E-09	-6.28E-09	-7.09E-09	-5.94E-09	-5.06E-09	-4.45E-09	-4.48E-09	-5.46E-09
-5.34E-09	-5.12E-09	-5.59E-09	-5.05E-09	-4.82E-09	-5.54E-09	-6.13E-09	-6.08E-09	-6.15E-09	-6.83E-09	-5.51E-09	-5.22E-09	-4.54E-09	-4.51E-09	-5.28E-09
-5.39E-09	-5.71E-09	-5.46E-09	-5.08E-09	-5.03E-09	-5.39E-09	-5.82E-09	-5.52E-09	-5.94E-09	-6.08E-09	-4.71E-09	-4.81E-09	-4.39E-09	-4.05E-09	-4.53E-09
-5.10E-09	-5.66E-09	-5.00E-09	-4.86E-09	-4.55E-09	-5.29E-09	-5.45E-09	-4.95E-09	-5.47E-09	-5.39E-09	-4.69E-09	-5.05E-09	-3.93E-09	-2.73E-09	-3.31E-09
-4.62E-09	-4.91E-09	-4.39E-09	-4.89E-09	-4.40E-09	-5.06E-09	-5.32E-09	-4.16E-09	-4.98E-09	-5.35E-09	-4.80E-09	-4.98E-09	-3.67E-09	-1.62E-09	-2.83E-09
-4.05E-09	-4.19E-09	-3.90E-09	-4.58E-09	-4.10E-09	-5.29E-09	-5.13E-09	-4.40E-09	-5.15E-09	-5.38E-09	-5.00E-09	-5.03E-09	-4.35E-09	-2.51E-09	-3.19E-09
-3.63E-09	-3.67E-09	-3.67E-09	-3.90E-09	-3.57E-09	-5.08E-09	-4.61E-09	-4.95E-09	-5.21E-09	-5.27E-09	-4.75E-09	-5.04E-09	-4.98E-09	-3.93E-09	-3.33E-09
-3.66E-09	-3.36E-09	-3.67E-09	-4.02E-09	-4.24E-09	-4.96E-09	-4.23E-09	-4.52E-09	-5.12E-09	-5.30E-09	-4.80E-09	-4.86E-09	-5.28E-09	-4.64E-09	-4.21E-09
-4.00E-09	-3.54E-09	-3.50E-09	-4.04E-09	-4.32E-09	-4.77E-09	-4.23E-09	-4.25E-09	-4.88E-09	-5.48E-09	-4.65E-09	-4.41E-09	-5.11E-09	-4.86E-09	-4.12E-09
-3.89E-09	-3.88E-09	-3.41E-09	-3.80E-09	-4.14E-09	-4.45E-09	-4.02E-09	-4.15E-09	-4.75E-09	-4.95E-09	-4.29E-09	-4.07E-09	-4.55E-09	-4.63E-09	-4.08E-09
-3.13E-09	-3.50E-09	-3.35E-09	-3.59E-09	-3.51E-09	-3.85E-09	-4.00E-09	-3.90E-09	-4.42E-09	-4.35E-09	-3.55E-09	-3.63E-09	-4.20E-09	-4.00E-09	-3.60E-09

-2.86E-09	-2.96E-09	-3.38E-09	-3.73E-09	-3.33E-09	-3.66E-09	-3.70E-09	-3.51E-09	-4.00E-09	-4.07E-09	-2.74E-09	-3.56E-09	-3.73E-09	-3.70E-09	-2.68E-09
-2.95E-09	-2.72E-09	-3.16E-09	-3.54E-09	-3.44E-09	-3.88E-09	-3.27E-09	-3.41E-09	-3.77E-09	-4.08E-09	-2.52E-09	-3.37E-09	-3.43E-09	-3.64E-09	-2.35E-09
-2.63E-09	-2.19E-09	-2.80E-09	-3.18E-09	-3.08E-09	-3.80E-09	-3.31E-09	-3.30E-09	-3.81E-09	-4.08E-09	-3.12E-09	-3.30E-09	-3.59E-09	-3.19E-09	-2.45E-09
-2.42E-09	-2.06E-09	-2.41E-09	-2.71E-09	-2.86E-09	-3.40E-09	-3.21E-09	-3.15E-09	-3.71E-09	-3.72E-09	-2.64E-09	-3.20E-09	-3.25E-09	-3.20E-09	-2.49E-09
-2.43E-09	-2.22E-09	-2.21E-09	-2.51E-09	-2.71E-09	-3.11E-09	-3.28E-09	-2.63E-09	-3.34E-09	-3.15E-09	-2.46E-09	-2.84E-09	-3.09E-09	-3.46E-09	-2.55E-09
-2.02E-09	-2.04E-09	-2.19E-09	-2.56E-09	-2.47E-09	-2.67E-09	-2.88E-09	-2.90E-09	-3.01E-09	-2.72E-09	-2.20E-09	-2.49E-09	-2.78E-09	-3.24E-09	-2.88E-09
-1.50E-09	-2.03E-09	-2.27E-09	-2.59E-09	-2.04E-09	-2.01E-09	-2.18E-09	-2.29E-09	-2.42E-09	-2.19E-09	-2.03E-09	-1.89E-09	-2.43E-09	-3.15E-09	-3.07E-09
-8.68E-10	-1.89E-09	-2.02E-09	-2.43E-09	-1.73E-09	-1.44E-09	-1.65E-09	-1.45E-09	-1.90E-09	-1.91E-09	-1.92E-09	-1.55E-09	-2.34E-09	-2.98E-09	-2.58E-09
-4.44E-10	-1.23E-09	-1.39E-09	-2.24E-09	-1.30E-09	-1.59E-09	-8.66E-10	-1.46E-09	-1.86E-09	-1.70E-09	-1.90E-09	-1.48E-09	-2.46E-09	-2.99E-09	-2.42E-09
9.28E-11	-5.96E-10	-3.04E-10	-1.81E-09	-1.05E-09	-1.41E-09	-7.02E-10	-1.96E-09	-1.75E-09	-1.64E-09	-1.72E-09	-1.54E-09	-2.14E-09	-2.89E-09	-2.32E-09
1.27E-09	2.44E-10	-8.87E-11	-9.66E-10	-6.39E-10	-9.11E-10	-7.41E-10	-1.98E-09	-1.43E-09	-1.81E-09	-1.59E-09	-1.40E-09	-1.80E-09	-2.61E-09	-2.49E-09
1.48E-09	5.41E-10	-1.52E-10	-6.45E-10	-7.87E-10	-9.32E-10	-1.00E-09	-1.98E-09	-9.66E-10	-1.87E-09	-1.70E-09	-1.15E-09	-1.41E-09	-2.45E-09	-2.38E-09
1.05E-09	7.56E-10	1.56E-10	-2.32E-10	-5.15E-10	-9.64E-10	-5.23E-10	-1.52E-09	-1.05E-09	-1.16E-09	-1.37E-09	-4.48E-10	-6.08E-10	-2.13E-09	-2.36E-09
1.32E-09	1.17E-09	7.99E-10	2.88E-10	-7.68E-11	-6.49E-10	2.50E-10	-6.97E-10	-9.54E-10	-1.88E-10	-3.09E-10	4.94E-10	3.13E-10	-1.47E-09	-1.85E-09
1.25E-09	1.33E-09	1.23E-09	9.46E-10	1.00E-09	-2.44E-10	9.87E-10	-6.16E-11	-1.59E-10	4.37E-10	7.28E-10	9.23E-10	9.38E-10	-9.37E-10	-1.42E-09
1.79E-09	1.26E-09	1.30E-09	1.59E-09	2.36E-09	3.49E-10	6.58E-11	1.37E-10	5.03E-10	1.03E-09	9.79E-10	7.62E-10	2.77E-10	-3.40E-10	-1.05E-09
2.23E-09	1.69E-09	1.59E-09	2.24E-09	3.61E-09	9.43E-10	5.75E-10	2.81E-10	5.81E-10	1.86E-09	1.44E-09	8.31E-10	4.69E-10	5.35E-11	-8.08E-10
2.18E-09	1.95E-09	1.94E-09	2.03E-09	1.93E-09	1.73E-09	9.56E-10	7.39E-10	1.23E-09	2.51E-09	2.00E-09	1.31E-09	1.03E-09	1.81E-10	-4.13E-10
2.69E-09	2.36E-09	2.77E-09	2.20E-09	2.32E-09	2.41E-09	1.44E-09	1.11E-09	1.78E-09	3.40E-09	2.43E-09	1.63E-09	9.18E-10	5.96E-10	-4.62E-10
2.94E-09	2.78E-09	3.19E-09	2.31E-09	2.68E-09	3.31E-09	1.84E-09	1.61E-09	1.71E-09	5.31E-09	2.96E-09	2.02E-09	1.15E-09	1.42E-09	-1.15E-10
3.64E-09	3.69E-09	4.00E-09	3.18E-09	3.33E-09	3.73E-09	2.76E-09	2.52E-09	2.20E-09	4.10E-09	3.09E-09	2.62E-09	2.29E-09	2.03E-09	6.46E-10
4.36E-09	4.48E-09	4.71E-09	4.28E-09	4.57E-09	4.87E-09	4.22E-09	3.50E-09	2.73E-09	3.63E-09	3.52E-09	3.46E-09	3.42E-09	2.56E-09	1.52E-09
5.12E-09	5.21E-09	4.98E-09	4.95E-09	5.15E-09	5.77E-09	5.16E-09	4.20E-09	3.50E-09	3.71E-09	4.38E-09	4.24E-09	4.11E-09	2.86E-09	2.56E-09
5.48E-09	5.83E-09	5.32E-09	5.19E-09	5.54E-09	5.66E-09	5.64E-09	4.26E-09	4.11E-09	3.89E-09	5.00E-09	4.58E-09	4.84E-09	3.46E-09	2.97E-09
5.68E-09	6.35E-09	5.80E-09	5.64E-09	5.75E-09	5.81E-09	5.54E-09	4.39E-09	4.86E-09	4.19E-09	5.31E-09	4.83E-09	4.99E-09	4.32E-09	3.51E-09
6.40E-09	6.72E-09	6.26E-09	6.35E-09	5.76E-09	6.25E-09	5.97E-09	4.94E-09	5.77E-09	4.69E-09	5.71E-09	5.32E-09	5.35E-09	5.06E-09	4.41E-09
7.21E-09	7.52E-09	6.76E-09	7.17E-09	6.08E-09	6.92E-09	6.59E-09	5.26E-09	6.54E-09	5.42E-09	6.24E-09	5.81E-09	5.58E-09	5.08E-09	5.02E-09
7.67E-09	7.90E-09	7.57E-09	7.63E-09	6.75E-09	7.21E-09	7.01E-09	6.49E-09	7.45E-09	6.47E-09	6.61E-09	6.49E-09	6.13E-09	5.44E-09	5.34E-09
8.27E-09	8.62E-09	8.48E-09	8.25E-09	7.45E-09	8.21E-09	7.83E-09	7.49E-09	7.93E-09	7.37E-09	7.26E-09	7.50E-09	6.93E-09	5.94E-09	5.68E-09
9.08E-09	9.37E-09	9.45E-09	9.21E-09	8.76E-09	9.17E-09	8.48E-09	8.14E-09	8.29E-09	8.03E-09	7.88E-09	8.15E-09	7.52E-09	6.69E-09	6.56E-09
1.04E-08	9.95E-09	9.83E-09	9.98E-09	1.03E-08	9.92E-09	9.18E-09	8.89E-09	9.14E-09	8.45E-09	8.56E-09	8.39E-09	7.85E-09	7.79E-09	7.35E-09
1.14E-08	1.09E-08	1.04E-08	1.10E-08	1.12E-08	1.04E-08	1.02E-08	1.01E-08	9.83E-09	9.29E-09	9.74E-09	9.01E-09	8.84E-09	8.70E-09	8.28E-09
1.20E-08	1.22E-08	1.15E-08	1.16E-08	1.13E-08	1.10E-08	1.11E-08	1.12E-08	1.05E-08	9.94E-09	1.06E-08	9.99E-09	9.96E-09	9.59E-09	9.04E-09
1.25E-08	1.28E-08	1.25E-08	1.26E-08	1.13E-08	1.19E-08	1.20E-08	1.26E-08	1.15E-08	1.09E-08	1.16E-08	1.09E-08	1.10E-08	1.04E-08	9.67E-09
1.32E-08	1.33E-08	1.29E-08	1.32E-08	1.17E-08	1.26E-08	1.25E-08	1.32E-08	1.23E-08	1.19E-08	1.24E-08	1.15E-08	1.20E-08	1.14E-08	1.04E-08
1.39E-08	1.40E-08	1.34E-08	1.40E-08	1.23E-08	1.36E-08	1.31E-08	1.37E-08	1.31E-08	1.33E-08	1.29E-08	1.25E-08	1.27E-08	1.24E-08	1.11E-08
1.50E-08	1.43E-08	1.42E-08	1.46E-08	1.37E-08	1.48E-08	1.41E-08	1.44E-08	1.38E-08	1.48E-08	1.40E-08	1.38E-08	1.36E-08	1.34E-08	1.19E-08
1.58E-08	1.51E-08	1.52E-08	1.57E-08	1.53E-08	1.57E-08	1.52E-08	1.44E-08	1.43E-08	1.60E-08	1.55E-08	1.49E-08	1.49E-08	1.41E-08	1.33E-08
1.68E-08	1.64E-08	1.63E-08	1.61E-08	1.65E-08	1.66E-08	1.59E-08	1.51E-08	1.49E-08	1.66E-08	1.65E-08	1.60E-08	1.63E-08	1.52E-08	1.46E-08
1.80E-08	1.73E-08	1.69E-08	1.70E-08	1.73E-08	1.77E-08	1.68E-08	1.62E-08	1.62E-08	1.71E-08	1.71E-08	1.67E-08	1.76E-08	1.61E-08	1.57E-08
1.87E-08	1.85E-08	1.80E-08	1.83E-08	1.87E-08	1.91E-08	1.82E-08	1.77E-08	1.79E-08	1.82E-08	1.79E-08	1.76E-08	1.81E-08	1.73E-08	1.65E-08
1.92E-08	1.96E-08	1.93E-08	1.88E-08	1.97E-08	1.96E-08	1.94E-08	1.91E-08	1.89E-08	1.89E-08	1.85E-08	1.84E-08	1.88E-08	1.86E-08	1.76E-08
2.01E-08	2.06E-08	2.03E-08	1.95E-08	2.01E-08	1.98E-08	2.00E-08	2.03E-08	1.99E-08	1.97E-08	1.97E-08	1.94E-08	1.97E-08	1.98E-08	1.89E-08
2.14E-08	2.15E-08	2.15E-08	2.08E-08	2.11E-08	2.07E-08	2.09E-08	2.15E-08	2.11E-08	2.08E-08	2.09E-08	2.05E-08	2.08E-08	2.07E-08	1.97E-08
2.24E-08	2.26E-08	2.24E-08	2.22E-08	2.23E-08	2.20E-08	2.21E-08	2.23E-08	2.20E-08	2.20E-08	2.18E-08	2.17E-08	2.19E-08	2.16E-08	2.09E-08
2.39E-08	2.37E-08	2.37E-08	2.37E-08	2.35E-08	2.34E-08	2.31E-08	2.30E-08	2.29E-08	2.32E-08	2.29E-08	2.30E-08	2.30E-08	2.24E-08	2.21E-08
2.51E-08	2.51E-08	2.49E-08	2.50E-08	2.46E-08	2.48E-08	2.42E-08	2.34E-08	2.34E-08	2.41E-08	2.38E-08	2.40E-08	2.38E-08	2.38E-08	2.32E-08
2.59E-08	2.60E-08	2.57E-08	2.61E-08	2.56E-08	2.59E-08	2.51E-08	2.45E-08	2.45E-08	2.53E-08	2.51E-08	2.52E-08	2.50E-08	2.52E-08	2.41E-08
2.70E-08	2.75E-08	2.67E-08	2.75E-08	2.71E-08	2.69E-08	2.62E-08	2.60E-08	2.59E-08	2.63E-08	2.66E-08	2.63E-08	2.62E-08	2.62E-08	2.53E-08
2.83E-08	2.88E-08	2.79E-08	2.87E-08	2.83E-08	2.79E-08	2.72E-08	2.77E-08	2.73E-08	2.79E-08	2.84E-08	2.78E-08	2.74E-08	2.71E-08	2.70E-08

6.59E-18	4.84E-18	3.17E-19	3.04E-18	1.13E-17	5.05E-17	1.40E-16	2.04E-16	1.19E-16	7.00E-17	7.28E-17	1.79E-17	3.92E-17	5.79E-17	6.73E-17
4.64E-18	5.18E-18	1.97E-19	3.80E-18	1.58E-17	6.97E-17	1.88E-16	2.78E-16	1.71E-16	1.15E-16	1.47E-16	3.17E-17	5.88E-17	9.10E-17	1.01E-16
5.68E-18	3.30E-18	3.11E-19	1.84E-18	1.58E-17	4.76E-17	1.48E-16	2.27E-16	1.93E-16	1.32E-16	2.05E-16	5.53E-17	7.17E-17	1.09E-16	1.14E-16
7.44E-18	2.81E-18	7.27E-19	7.71E-19	1.46E-17	3.37E-17	1.04E-16	1.65E-16	1.43E-16	1.01E-16	7.68E-17	8.01E-17	7.29E-17	1.30E-16	1.38E-16
7.96E-18	1.79E-18	3.94E-19	1.00E-18	6.30E-18	2.22E-17	5.48E-17	7.41E-17	6.95E-17	4.99E-17	2.18E-17	3.16E-17	3.39E-17	8.86E-17	1.20E-16
6.33E-18	1.85E-18	1.11E-20	1.55E-18	2.66E-18	6.95E-18	1.78E-17	1.27E-17	2.11E-17	2.47E-17	4.53E-18	8.74E-18	1.04E-17	2.91E-17	9.53E-17
5.14E-18	1.16E-18	1.05E-19	1.41E-18	6.66E-19	8.07E-20	3.03E-18	1.10E-18	3.90E-18	7.94E-18	8.11E-19	4.44E-18	7.60E-18	1.22E-17	6.85E-17
5.78E-18	1.28E-18	3.38E-20	1.33E-19	6.53E-20	2.09E-19	2.06E-19	8.96E-20	8.44E-19	2.43E-19	2.34E-19	4.35E-18	7.06E-18	6.96E-18	3.51E-17
5.40E-18	3.04E-18	4.31E-19	1.56E-18	5.42E-20	2.04E-19	1.89E-19	7.30E-20	1.17E-19	2.79E-20	8.03E-20	1.26E-18	1.78E-18	1.60E-18	1.25E-17
5.31E-18	6.51E-18	3.39E-18	3.71E-18	2.10E-18	7.15E-19	9.18E-19	2.35E-19	2.13E-19	6.49E-19	5.92E-19	1.14E-19	4.51E-19	1.18E-18	7.61E-18
6.01E-18	1.04E-17	4.98E-18	1.49E-18	4.99E-18	3.65E-18	2.35E-18	2.13E-19	2.85E-19	1.33E-18	4.55E-19	4.12E-19	7.61E-19	3.30E-18	1.23E-17
7.96E-18	1.28E-17	4.17E-18	2.62E-18	5.08E-18	6.76E-18	5.02E-18	7.09E-19	6.63E-19	8.73E-19	3.18E-20	1.52E-18	2.56E-18	8.70E-18	1.83E-17
1.23E-17	1.47E-17	3.92E-18	8.23E-18	5.17E-18	8.97E-18	4.52E-18	6.18E-19	2.28E-18	9.34E-19	1.80E-19	2.14E-18	6.10E-18	1.44E-17	2.10E-17
1.33E-17	1.13E-17	4.63E-18	1.19E-17	5.51E-18	8.44E-18	3.10E-18	6.81E-19	1.59E-18	2.29E-19	2.23E-18	6.60E-18	1.18E-17	2.03E-17	2.80E-17
1.20E-17	7.97E-18	9.68E-18	8.32E-18	3.87E-18	7.82E-18	3.15E-18	9.76E-19	2.29E-19	1.65E-18	1.06E-17	1.29E-17	2.05E-17	1.96E-17	2.07E-17
1.34E-17	8.73E-18	1.04E-17	7.13E-18	2.56E-18	7.25E-18	2.46E-18	3.46E-19	3.69E-20	2.07E-18	8.66E-18	8.53E-18	1.32E-17	1.29E-17	8.69E-18
1.49E-17	1.52E-17	1.30E-17	6.05E-18	1.65E-18	4.10E-18	1.91E-18	2.45E-19	1.58E-20	7.25E-19	2.19E-18	2.59E-18	5.48E-18	5.13E-18	2.38E-18
1.71E-17	2.01E-17	1.24E-17	3.33E-18	2.09E-19	8.63E-19	2.36E-18	6.41E-19	4.30E-21	2.00E-19	5.35E-21	4.02E-19	2.49E-19	3.10E-19	1.20E-19
1.53E-17	1.64E-17	9.88E-18	4.51E-18	3.52E-20	1.25E-20	7.43E-19	6.33E-20	4.25E-19	1.08E-18	1.16E-18	1.20E-19	1.32E-18	9.92E-19	4.54E-18
1.95E-17	1.46E-17	5.63E-18	5.36E-18	1.71E-18	1.79E-19	7.40E-20	9.24E-20	1.60E-19	5.44E-19	1.66E-18	1.96E-18	5.30E-18	4.26E-18	1.13E-17
2.25E-17	1.30E-17	5.63E-18	7.04E-18	1.28E-17	1.24E-18	1.79E-18	8.29E-19	8.04E-20	1.68E-18	7.36E-18	8.13E-18	1.32E-17	1.23E-17	1.62E-17
2.71E-17	1.75E-17	8.37E-18	1.00E-17	2.21E-17	5.88E-18	9.94E-18	9.70E-18	4.90E-18	9.93E-18	1.63E-17	1.13E-17	1.82E-17	1.86E-17	2.10E-17
3.41E-17	2.31E-17	1.65E-17	1.64E-17	2.49E-17	1.61E-17	1.94E-17	2.66E-17	1.73E-17	1.83E-17	2.22E-17	1.15E-17	2.25E-17	2.47E-17	2.77E-17
4.20E-17	3.07E-17	2.72E-17	1.89E-17	2.86E-17	2.20E-17	2.45E-17	3.35E-17	2.99E-17	2.76E-17	2.74E-17	1.19E-17	2.78E-17	3.58E-17	3.81E-17
5.26E-17	3.58E-17	3.59E-17	2.36E-17	3.73E-17	2.56E-17	2.59E-17	3.28E-17	3.10E-17	3.13E-17	3.08E-17	3.30E-17	3.35E-17	4.34E-17	4.68E-17
6.73E-17	4.04E-17	3.53E-17	2.84E-17	4.18E-17	2.80E-17	2.61E-17	3.35E-17	3.18E-17	3.43E-17	3.24E-17	3.87E-17	3.60E-17	4.11E-17	4.33E-17
5.88E-17	3.91E-17	3.79E-17	3.28E-17	4.02E-17	2.86E-17	3.15E-17	3.88E-17	3.59E-17	3.84E-17	4.00E-17	3.66E-17	2.91E-17	3.73E-17	4.49E-17
3.46E-17	3.55E-17	4.05E-17	3.28E-17	4.15E-17	4.04E-17	4.04E-17	4.51E-17	4.11E-17	4.65E-17	4.43E-17	3.30E-17	2.87E-17	3.25E-17	4.28E-17
3.99E-17	3.72E-17	4.30E-17	3.34E-17	4.09E-17	5.02E-17	4.29E-17	4.56E-17	4.71E-17	5.08E-17	4.47E-17	3.71E-17	3.57E-17	4.07E-17	5.01E-17
3.85E-17	4.13E-17	4.57E-17	3.34E-17	3.83E-17	4.74E-17	4.81E-17	5.32E-17	5.55E-17	4.81E-17	5.27E-17	4.61E-17	5.49E-17	5.97E-17	6.18E-17
3.92E-17	4.08E-17	4.66E-17	3.34E-17	3.82E-17	4.58E-17	4.80E-17	5.73E-17	6.36E-17	4.97E-17	5.17E-17	4.72E-17	6.58E-17	6.74E-17	7.02E-17

4.43E-17	3.95E-17	4.51E-17	3.22E-17	3.82E-17	4.37E-17	4.81E-17	5.69E-17	5.96E-17	5.42E-17	5.09E-17	4.78E-17	6.67E-17	6.40E-17	7.19E-17
4.44E-17	4.30E-17	4.95E-17	3.38E-17	4.06E-17	3.66E-17	4.75E-17	5.79E-17	5.94E-17	6.01E-17	5.41E-17	5.75E-17	6.78E-17	6.40E-17	7.21E-17
4.39E-17	4.41E-17	5.25E-17	4.18E-17	4.58E-17	3.94E-17	5.20E-17	5.81E-17	6.33E-17	7.26E-17	6.13E-17	6.56E-17	6.89E-17	7.12E-17	7.28E-17
4.75E-17	4.11E-17	5.66E-17	4.60E-17	4.55E-17	4.42E-17	5.27E-17	5.01E-17	6.55E-17	7.63E-17	5.98E-17	7.51E-17	7.50E-17	7.57E-17	7.43E-17
4.59E-17	3.83E-17	5.30E-17	4.80E-17	4.35E-17	4.76E-17	5.52E-17	4.68E-17	6.48E-17	7.67E-17	6.02E-17	7.75E-17	7.89E-17	7.67E-17	7.10E-17
4.36E-17	4.04E-17	4.91E-17	4.50E-17	4.36E-17	5.68E-17	6.63E-17	5.35E-17	6.87E-17	8.05E-17	6.57E-17	7.89E-17	7.75E-17	8.29E-17	7.31E-17
4.33E-17	3.83E-17	4.97E-17	5.28E-17	4.89E-17	5.94E-17	8.28E-17	5.92E-17	7.34E-17	7.58E-17	7.28E-17	8.03E-17	7.65E-17	8.12E-17	7.30E-17
4.71E-17	3.75E-17	5.29E-17	5.68E-17	4.93E-17	5.89E-17	7.71E-17	6.07E-17	6.81E-17	6.64E-17	7.65E-17	8.84E-17	7.45E-17	8.26E-17	7.65E-17
4.60E-17	4.09E-17	5.74E-17	5.51E-17	4.81E-17	5.47E-17	7.61E-17	6.18E-17	6.20E-17	6.15E-17	7.68E-17	8.74E-17	7.46E-17	7.84E-17	7.57E-17
4.38E-17	4.78E-17	6.04E-17	5.48E-17	5.06E-17	5.32E-17	7.92E-17	6.04E-17	5.95E-17	5.71E-17	7.78E-17	8.24E-17	7.12E-17	7.01E-17	7.71E-17
4.21E-17	4.91E-17	5.13E-17	5.96E-17	5.73E-17	5.04E-17	7.28E-17	5.56E-17	6.10E-17	6.10E-17	7.49E-17	7.30E-17	6.40E-17	6.72E-17	6.29E-17
4.22E-17	4.62E-17	4.68E-17	5.92E-17	5.86E-17	5.62E-17	6.41E-17	5.70E-17	5.75E-17	6.37E-17	7.37E-17	7.03E-17	6.52E-17	6.73E-17	5.33E-17
4.51E-17	4.60E-17	5.03E-17	6.12E-17	5.87E-17	5.96E-17	6.18E-17	6.59E-17	6.65E-17	6.96E-17	7.18E-17	7.05E-17	6.60E-17	6.27E-17	4.24E-17
4.61E-17	4.88E-17	5.01E-17	6.59E-17	5.27E-17	6.20E-17	6.95E-17	6.90E-17	6.85E-17	6.91E-17	7.09E-17	7.55E-17	6.50E-17	5.55E-17	4.98E-17
4.72E-17	5.15E-17	5.20E-17	6.42E-17	5.35E-17	6.23E-17	7.06E-17	6.84E-17	6.76E-17	6.52E-17	6.88E-17	7.02E-17	6.96E-17	6.16E-17	6.09E-17
4.79E-17	5.19E-17	5.59E-17	6.56E-17	5.42E-17	6.29E-17	6.15E-17	6.01E-17	6.13E-17	5.79E-17	6.66E-17	6.82E-17	6.89E-17	6.89E-17	7.36E-17
4.77E-17	5.34E-17	5.63E-17	5.91E-17	5.42E-17	6.27E-17	5.05E-17	4.99E-17	6.19E-17	5.28E-17	7.11E-17	7.13E-17	7.40E-17	7.20E-17	8.35E-17
4.46E-17	5.74E-17	5.72E-17	5.32E-17	4.99E-17	5.86E-17	4.50E-17	4.78E-17	5.97E-17	5.05E-17	7.43E-17	7.22E-17	7.36E-17	7.91E-17	8.05E-17
4.40E-17	5.36E-17	5.25E-17	4.85E-17	4.97E-17	5.92E-17	4.43E-17	4.45E-17	5.89E-17	5.19E-17	8.36E-17	7.90E-17	6.49E-17	7.02E-17	7.35E-17
4.24E-17	4.96E-17	5.11E-17	4.24E-17	5.88E-17	6.08E-17	5.00E-17	4.88E-17	5.63E-17	5.11E-17	8.37E-17	9.40E-17	5.89E-17	5.18E-17	5.64E-17
4.39E-17	4.75E-17	4.95E-17	4.28E-17	5.69E-17	6.27E-17	5.91E-17	5.40E-17	5.67E-17	5.15E-17	7.38E-17	8.56E-17	6.76E-17	3.80E-17	3.40E-17
4.52E-17	5.03E-17	4.91E-17	4.00E-17	5.65E-17	5.80E-17	6.16E-17	6.13E-17	6.42E-17	5.38E-17	7.03E-17	8.00E-17	7.05E-17	2.14E-17	6.59E-18
4.90E-17	5.18E-17	4.91E-17	4.65E-17	5.90E-17	5.96E-17	6.47E-17	6.27E-17	7.23E-17	6.15E-17	7.06E-17	7.55E-17	7.47E-17	1.95E-18	9.18E-18
5.25E-17	5.71E-17	5.41E-17	5.47E-17	6.45E-17	6.18E-17	7.11E-17	6.59E-17	7.24E-17	6.85E-17	7.19E-17	7.40E-17	7.67E-17	2.38E-18	8.00E-17
5.00E-17	5.50E-17	5.28E-17	5.92E-17	6.49E-17	6.15E-17	7.23E-17	6.51E-17	6.61E-17	7.10E-17	6.90E-17	6.94E-17	7.52E-17	6.20E-17	5.80E-18
4.45E-17	5.50E-17	5.20E-17	6.26E-17	5.85E-17	6.15E-17	7.43E-17	6.54E-17	6.37E-17	6.48E-17	6.12E-17	6.50E-17	6.67E-17	7.51E-17	7.07E-17
4.03E-17	5.55E-17	5.00E-17	5.72E-17	5.41E-17	6.07E-17	6.83E-17	6.67E-17	6.01E-17	5.89E-17	5.97E-17	5.94E-17	6.38E-17	6.69E-17	5.98E-17
4.10E-17	4.76E-17	3.94E-17	5.08E-17	5.30E-17	6.10E-17	6.01E-17	6.14E-17	5.52E-17	5.24E-17	6.20E-17	5.33E-17	6.74E-17	6.86E-17	6.11E-17
3.89E-17	4.06E-17	2.71E-17	4.37E-17	4.66E-17	5.86E-17	5.67E-17	5.26E-17	5.37E-17	4.81E-17	5.83E-17	5.51E-17	6.70E-17	7.62E-17	6.50E-17
3.41E-17	3.75E-17	2.13E-17	3.88E-17	4.30E-17	5.40E-17	5.67E-17	4.38E-17	5.41E-17	5.05E-17	5.06E-17	5.79E-17	6.72E-17	7.60E-17	6.81E-17
3.02E-17	3.69E-17	3.05E-17	3.99E-17	3.79E-17	5.12E-17	4.91E-17	3.89E-17	5.54E-17	5.77E-17	5.20E-17	5.44E-17	6.00E-17	7.41E-17	6.57E-17
3.17E-17	3.70E-17	3.79E-17	4.30E-17	4.02E-17	4.70E-17	4.48E-17	4.64E-17	5.62E-17	5.52E-17	5.38E-17	4.58E-17	4.96E-17	6.27E-17	5.81E-17
3.09E-17	3.28E-17	3.92E-17	4.15E-17	4.12E-17	3.86E-17	3.95E-17	5.11E-17	5.19E-17	5.36E-17	4.89E-17	4.04E-17	4.21E-17	5.30E-17	5.69E-17
3.11E-17	2.98E-17	3.72E-17	3.63E-17	4.07E-17	3.52E-17	3.82E-17	5.24E-17	4.42E-17	4.63E-17	4.09E-17	3.68E-17	3.87E-17	4.27E-17	4.62E-17
2.62E-17	2.22E-17	3.76E-17	3.03E-17	3.76E-17	3.03E-17	3.52E-17	5.21E-17	3.95E-17	4.21E-17	3.36E-17	3.16E-17	3.18E-17	2.95E-17	3.89E-17
2.50E-17	1.91E-17	2.88E-17	2.14E-17	3.22E-17	2.78E-17	3.49E-17	5.05E-17	4.09E-17	4.67E-17	2.97E-17	2.65E-17	2.31E-17	2.15E-17	3.34E-17
2.55E-17	2.30E-17	2.93E-17	1.94E-17	2.45E-17	3.06E-17	3.55E-17	4.35E-17	3.95E-17	5.03E-17	3.53E-17	2.56E-17	1.98E-17	2.01E-17	2.98E-17
2.85E-17	2.62E-17	3.13E-17	2.55E-17	2.32E-17	3.07E-17	3.76E-17	3.70E-17	3.78E-17	4.66E-17	3.04E-17	2.72E-17	2.06E-17	2.04E-17	2.79E-17
2.91E-17	3.26E-17	2.98E-17	2.58E-17	2.53E-17	2.90E-17	3.38E-17	3.04E-17	3.53E-17	3.70E-17	2.22E-17	2.31E-17	1.92E-17	1.64E-17	2.05E-17
2.60E-17	3.21E-17	2.50E-17	2.36E-17	2.07E-17	2.80E-17	2.97E-17	2.45E-17	2.99E-17	2.91E-17	2.20E-17	2.55E-17	1.54E-17	7.45E-18	1.09E-17
2.13E-17	2.41E-17	1.93E-17	2.39E-17	1.94E-17	2.57E-17	2.83E-17	1.73E-17	2.48E-17	2.87E-17	2.31E-17	2.48E-17	1.34E-17	2.61E-18	8.01E-18
1.64E-17	1.75E-17	1.52E-17	2.10E-17	1.68E-17	2.80E-17	2.63E-17	1.93E-17	2.66E-17	2.90E-17	2.50E-17	2.53E-17	1.89E-17	6.31E-18	1.02E-17
1.32E-17	1.35E-17	1.34E-17	1.52E-17	1.27E-17	2.58E-17	2.13E-17	2.45E-17	2.72E-17	2.77E-17	2.26E-17	2.54E-17	2.48E-17	1.54E-17	1.11E-17
1.34E-17	1.13E-17	1.35E-17	1.62E-17	1.80E-17	2.46E-17	1.79E-17	2.05E-17	2.63E-17	2.81E-17	2.31E-17	2.36E-17	2.79E-17	2.15E-17	1.77E-17
1.60E-17	1.25E-17	1.22E-17	1.63E-17	1.87E-17	2.28E-17	1.79E-17	1.81E-17	2.38E-17	3.00E-17	2.16E-17	1.94E-17	2.61E-17	2.36E-17	1.69E-17
1.52E-17	1.50E-17	1.17E-17	1.44E-17	1.71E-17	1.98E-17	1.62E-17	1.73E-17	2.26E-17	2.45E-17	1.84E-17	1.66E-17	2.07E-17	2.15E-17	1.66E-17
9.80E-18	1.22E-17	1.12E-17	1.29E-17	1.23E-17	1.48E-17	1.60E-17	1.52E-17	1.95E-17	1.89E-17	1.26E-17	1.32E-17	1.77E-17	1.60E-17	1.29E-17
8.19E-18	8.77E-18	1.14E-17	1.39E-17	1.11E-17	1.34E-17	1.37E-17	1.23E-17	1.60E-17	1.66E-17	7.53E-18	1.27E-17	1.39E-17	1.37E-17	7.21E-18
8.71E-18	7.41E-18	1.00E-17	1.25E-17	1.18E-17	1.50E-17	1.07E-17	1.16E-17	1.42E-17	1.66E-17	6.35E-18	1.14E-17	1.18E-17	1.33E-17	5.51E-18
6.89E-18	4.79E-18	7.84E-18	1.01E-17	9.48E-18	1.44E-17	1.10E-17	1.09E-17	1.45E-17	1.67E-17	9.74E-18	1.09E-17	1.29E-17	1.02E-17	6.02E-18
5.87E-18	4.26E-18	5.82E-18	7.34E-18	8.19E-18	1.16E-17	1.03E-17	9.95E-18	1.38E-17	1.38E-17	6.97E-18	1.02E-17	1.06E-17	1.03E-17	6.20E-18
5.89E-18	4.93E-18	4.89E-18	6.28E-18	7.35E-18	9.69E-18	1.07E-17	6.90E-18	1.11E-17	9.91E-18	6.07E-18	8.09E-18	9.54E-18	1.20E-17	6.51E-18
4.08E-18	4.14E-18	4.82E-18	6.56E-18	6.09E-18	7.14E-18	8.31E-18	8.42E-18	9.09E-18	7.37E-18	4.84E-18	6.21E-18	7.74E-18	1.05E-17	8.28E-18
2.26E-18	4.11E-18	5.14E-18	6.71E-18	4.17E-18	4.03E-18	4.77E-18	5.23E-18	5.83E-18	4.79E-18	4.13E-18	3.58E-18	5.89E-18	9.90E-18	9.44E-18
7.54E-19	3.57E-18	4.10E-18	5.88E-18	3.01E-18	2.07E-18	2.73E-18	2.11E-18	3.60E-18	3.66E-18	3.70E-18	2.39E-18	5.49E-18	8.90E-18	6.67E-18
1.97E-19	1.51E-18	1.94E-18	5.01E-18	1.69E-18	2.54E-18	7.50E-19	2.14E-18	3.46E-18	2.88E-18	3.61E-18	2.18E-18	6.04E-18	8.97E-18	5.87E-18
8.61E-21	3.55E-19	9.26E-20	3.27E-18	1.11E-18	2.00E-18	4.92E-19	3.84E-18	3.05E-18	2.69E-18	2.95E-18	2.36E-18	4.56E-18	8.34E-18	5.38E-18
1.62E-18	5.94E-20	7.88E-21	9.32E-19	4.09E-19	8.29E-19	5.48E-19	3.92E-18	2.04E-18	3.28E-18	2.51E-18	1.95E-18	3.24E-18	6.81E-18	6.20E-18
2.19E-18	2.93E-19	2.33E-20	4.16E-19	6.20E-19	8.69E-19	1.01E-18	3.92E-18	9.32E-19	3.50E-18	2.88E-18	1.33E-18	1.99E-18	5.99E-18	5.67E-18
1.11E-18	5.71E-19	2.44E-20	5.38E-20	2.65E-19	9.30E-19	2.74E-19	2.31E-18	1.11E-18	1.34E-18	1.88E-18	2.00E-19	3.70E-19	4.55E-18	5.55E-18
1.75E-18	1.37E-18	6.38E-19	8.32E-20	5.89E-21	4.22E-19	6.26E-20	4.86E-19	9.10E-19	3.55E-20	9.54E-20	2.44E-19	9.77E-20	2.15E-18	3.44E-18
1.56E-18	1.78E-18	1.52E-18	8.95E-19	1.01E-18	5.95E-20	9.73E-19	3.80E-21	2.53E-20	1.91E-19	5.29E-19	8.52E-19	8.80E-19	8.79E-19	2.02E-18
3.22E-18	1.58E-18	1.68E-18	2.54E-18	5.55E-18	1.22E-19	4.33E-21	1.89E-20	2.53E-19	1.06E-18	9.59E-19	5.81E-19	7.68E-20	1.15E-19	1.11E-18
4.98E-18	2.85E-18	2.53E-18	5.00E-18	1.30E-17	8.89E-19	3.30E-19	7.88E-20	3.38E-19	3.46E-18	2.06E-18	6.91E-19	2.20E-19	2.87E-21	6.53E-19
4.75E-18	3.80E-18	3.76E-18	4.12E-18	3.73E-18	3.00E-18	9.14E-19	5.46E-19	1.52E-18	6.31E-18	4.00E-18	1.71E-18	1.05E-18	3.27E-20	1.71E-19
7.23E-18	5.56E-18	7.69E-18	4.84E-18	5.37E-18	5.83E-18	2.06E-18	1.24E-18	3.18E-18	1.15E-17	5.92E-18	2.65E-18	8.42E-19	3.56E-19	2.14E-19
8.65E-18	7.76E-18	1.02E-17	5.35E-18	7.20E-18	1.10E-17	3.37E-18	2.58E-18	2.91E-18	2.82E-17	8.75E-18	4.08E-18	1.32E-18	2.02E-18	1.32E-20
1.32E-17	1.36E-17	1.60E-17	1.01E-17	1.11E-17	1.39E-17	7.63E-18	6.33E-18	4.83E-18	1.68E-17	9.54E-18	6.88E-18	5.23E-18	4.14E-18	4.17E-19
1.90E-17	2.01E-17	2.22E-17	1.83E-17	2.09E-17	2.37E-17	1.78E-17	1.23E-17	7.46E-18	1.32E-17	1.24E-17	1.20E-17	1.17E-17	6.58E-18	2.32E-18
2.63E-17	2.71E-17	2.48E-17	2.45E-17	2.65E-17	3.32E-17	2.66E-17	1.76E-17	1.23E-17	1.38E-17	1.92E-17	1.80E-17	1.69E-17	8.17E-18	6.54E-18
3.01E-17	3.40E-17	2.83E-17	2.69E-17	3.07E-17	3.21E-17	3.18E-17	1.82E-17	1.69E-17	1.51E-17	2.50E-17	2.10E-17	2.34E-17	1.20E-17	8.79E-18
3.23E-17	4.03E-17	3.37E-17	3.18E-17	3.30E-17	3.37E-17	3.07E-17	1.93E-17	2.36E-17	1.76E-17	2.82E-17	2.33E-17	2.49E-17	1.87E-17	1.23E-17
4.09E-17	4.52E-17	3.92E-17	4.03E-17	3.32E-17	3.91E-17	3.56E-17	2.44E-17	3.33E-17	2.20E-17	3.25E-17	2.83E-17	2.86E-17	2.56E-17	1.94E-17
5.19E-17	5.65E-17	4.56E-17	5.13E-17	3.70E-17	4.79E-17	4.34E-17	2.77E-17	4.28E-17	2.94E-17	3.89E-17	3.38E-17	3.12E-17	2.58E-17	2.52E-17
5.88E-17	6.25E-17	5.73E-17	5.82E-17	4.56E-17	5.19E-17	4.92E-17	4.21E-17	5.55E-17	4.19E-17	4.36E-17	4.21E-17	3.76E-17	2.96E-17	2.85E-17
6.83E-17	7.44E-17	7.19E-17	6.80E-17	5.55E-17	6.74E-17	6.13E-17	5.61E-17	6.29E-17	5.44E-17	5.27E-17	5.62E-17	4.81E-17	3.53E-17	3.23E-17
8.25E-17	8.79E-17	8.93E-17	8.49E-17	7.67E-17	8.41E-17	7.19E-17	6.62E-17	6.88E-17	6.45E-17	6.21E-17	6.65E-17	5.66E-17	4.48E-17	4.30E-17
1.08E-16	9.91E-17	9.66E-17	9.95E-17	1.06E-16	9.83E-17	8.42E-17	7.91E-17	8.35E-17	7.14E-17	7.32E-17	7.05E-17	6.15E-17	6.07E-17	5.40E-17
1.30E-16	1.19E-16	1.09E-16	1.20E-16	1.24E-16	1.07E-16	1.05E-16	1.02E-16	9.65E-17	8.63E-17	9.48E-17	8.12E-17	7.81E-17	7.57E-17	6.86E-17
1.44E-16	1.49E-16	1.32E-16	1.35E-16	1.27E-16	1.21E-16	1.23E-16	1.26E-16	1.09E-16	9.87E-17	1.12E-16	9.98E-17	9.92E-17	9.19E-17	8.18E-17
1.57E-16	1.64E-16	1.56E-16	1.58E-16	1.27E-16	1.42E-16	1.45E-16	1.58E-16	1.31E-16	1.18E-16	1.35E-16	1.20E-16	1.20E-16	1.08E-16	9.34E-17
1.73E-16	1.77E-16	1.65E-16	1.75E-16	1.37E-16	1.59E-16	1.57E-16	1.74E-16	1.51E-16	1.42E-16	1.55E-16	1.31E-16	1.44E-16	1.29E-16	1.09E-16
1.94E-16	1.95E-16	1.79E-16	1.96E-16	1.51E-16	1.84E-16	1.71E-16	1.88E-16	1.72E-16	1.76E-16	1.66E-16	1.55E-16	1.60E-16	1.53E-16	1.22E-16
2.26E-16	2.06E-16	2.01E-16	2.13E-16	1.88E-16	2.18E-16	2.00E-16	2.07E-16	1.90E-16	2.20E-16	1.96E-16	1.91E-16	1.84E-16	1.80E-16	1.43E-16
2.51E-16	2.29E-16	2.31E-16	2.45E-16	2.34E-16	2.46E-16	2.31E-16	2.08E-16	2.06E-16	2.56E-16	2.41E-16	2.21E-16	2.22E-16	2.00E-16	1.77E-16
2.83E-16	2.70E-16	2.65E-16	2.58E-16	2.71E-16	2.75E-16	2.52E-16	2.27E-16	2.23E-16	2.75E-16	2.74E-16	2.55E-16	2.66E-16	2.32E-16	2.13E-16

3.24E-16	3.01E-16	2.87E-16	2.90E-16	3.01E-16	3.12E-16	2.82E-16	2.63E-16	2.64E-16	2.92E-16	2.92E-16	2.80E-16	3.11E-16	2.60E-16	2.45E-16
3.51E-16	3.43E-16	3.23E-16	3.33E-16	3.49E-16	3.66E-16	3.32E-16	3.12E-16	3.19E-16	3.33E-16	3.20E-16	3.09E-16	3.28E-16	3.00E-16	2.72E-16
3.67E-16	3.84E-16	3.71E-16	3.55E-16	3.87E-16	3.84E-16	3.77E-16	3.64E-16	3.59E-16	3.59E-16	3.44E-16	3.39E-16	3.52E-16	3.47E-16	3.10E-16
4.02E-16	4.23E-16	4.13E-16	3.81E-16	4.06E-16	3.92E-16	3.99E-16	4.13E-16	3.95E-16	3.89E-16	3.87E-16	3.76E-16	3.87E-16	3.92E-16	3.56E-16
4.60E-16	4.61E-16	4.63E-16	4.32E-16	4.46E-16	4.27E-16	4.35E-16	4.61E-16	4.43E-16	4.31E-16	4.35E-16	4.19E-16	4.34E-16	4.28E-16	3.90E-16
5.04E-16	5.12E-16	5.04E-16	4.94E-16	4.96E-16	4.84E-16	4.88E-16	4.99E-16	4.82E-16	4.86E-16	4.76E-16	4.69E-16	4.80E-16	4.65E-16	4.37E-16
5.72E-16	5.60E-16	5.63E-16	5.64E-16	5.51E-16	5.48E-16	5.32E-16	5.28E-16	5.24E-16	5.39E-16	5.23E-16	5.28E-16	5.29E-16	5.01E-16	4.88E-16
6.30E-16	6.28E-16	6.18E-16	6.25E-16	6.06E-16	6.13E-16	5.84E-16	5.46E-16	5.48E-16	5.83E-16	5.67E-16	5.75E-16	5.67E-16	5.66E-16	5.38E-16
6.69E-16	6.77E-16	6.61E-16	6.81E-16	6.53E-16	6.69E-16	6.30E-16	6.00E-16	5.98E-16	6.40E-16	6.30E-16	6.33E-16	6.23E-16	6.35E-16	5.83E-16
7.30E-16	7.58E-16	7.14E-16	7.57E-16	7.34E-16	7.24E-16	6.86E-16	6.78E-16	6.70E-16	6.92E-16	7.06E-16	6.91E-16	6.88E-16	6.84E-16	6.39E-16
7.99E-16	8.28E-16	7.80E-16	8.22E-16	8.01E-16	7.77E-16	7.42E-16	7.68E-16	7.43E-16	7.78E-16	8.07E-16	7.72E-16	7.49E-16	7.35E-16	7.31E-16
1.04E-14	1.03E-14	1.01E-14	1.02E-14	1.01E-14	1.06E-14	1.08E-14	1.09E-14	1.07E-14	1.08E-14	1.08E-14	1.03E-14	1.05E-14	1.03E-14	9.99E-15

4.97E-09	3.44E-09	2.58E-09	2.11E-09	2.67E-10	-6.57E-11	1.43E-09	1.97E-09	-9.68E-10	-2.78E-09	-7.62E-10	1.16E-09	2.91E-09	3.14E-09	2.30E-09
5.98E-09	4.30E-09	3.90E-09	2.79E-09	1.15E-09	9.39E-10	1.66E-09	1.27E-09	-1.11E-09	-2.21E-09	-6.81E-10	3.53E-10	2.12E-09	3.00E-09	1.59E-09
7.99E-09	5.79E-09	4.61E-09	2.95E-09	1.80E-09	2.03E-09	1.74E-09	-5.32E-10	-1.10E-09	-2.02E-09	-1.64E-10	-5.28E-11	8.43E-10	1.85E-09	-1.24E-11
9.90E-09	7.14E-09	5.38E-09	2.91E-09	1.30E-09	1.77E-09	1.69E-09	-1.42E-09	-1.11E-09	-1.68E-09	-5.18E-10	-9.71E-10	-1.08E-09	-6.42E-10	-2.13E-09
1.14E-08	7.89E-09	5.66E-09	2.95E-09	4.54E-10	1.19E-09	1.94E-09	-1.11E-09	-1.62E-09	-1.38E-09	-2.19E-09	-2.42E-09	-2.82E-09	-3.04E-09	-3.38E-09
1.22E-08	8.76E-09	5.78E-09	2.36E-09	4.44E-10	1.06E-09	1.27E-09	-1.06E-09	-2.34E-09	-1.25E-09	-3.08E-09	-3.67E-09	-3.73E-09	-3.98E-09	-4.11E-09
1.17E-08	9.76E-09	6.40E-09	2.78E-09	1.34E-09	8.09E-10	-1.10E-10	-1.80E-09	-2.69E-09	-1.38E-09	-2.82E-09	-4.31E-09	-3.91E-09	-3.95E-09	-4.19E-09
9.36E-09	9.61E-09	7.19E-09	3.57E-09	1.26E-09	3.33E-11	-1.32E-09	-2.47E-09	-2.99E-09	-2.23E-09	-2.94E-09	-4.24E-09	-3.41E-09	-4.38E-09	-4.38E-09
6.54E-09	8.20E-09	7.10E-09	3.48E-09	1.01E-09	6.14E-10	-1.33E-09	-2.38E-09	-2.60E-09	-2.40E-09	-3.43E-09	-4.12E-09	-3.29E-09	-4.15E-09	-4.48E-09
4.56E-09	7.08E-09	7.66E-09	4.13E-09	1.83E-09	2.38E-09	9.71E-11	-1.42E-09	-1.40E-09	-1.66E-09	-3.39E-09	-4.01E-09	-3.40E-09	-4.10E-09	-4.31E-09
4.25E-09	7.03E-09	8.33E-09	5.24E-09	3.55E-09	4.43E-09	1.73E-09	5.19E-13	-5.47E-10	-1.24E-09	-2.91E-09	-3.54E-09	-3.45E-09	-4.45E-09	-4.31E-09
4.71E-09	6.72E-09	7.49E-09	6.37E-09	4.76E-09	4.49E-09	2.64E-09	7.19E-10	-6.98E-10	-9.85E-10	-2.50E-09	-3.08E-09	-3.23E-09	-4.42E-09	-4.50E-09
5.02E-09	5.79E-09	5.21E-09	5.48E-09	4.61E-09	3.84E-09	2.24E-09	1.53E-09	1.03E-10	-3.11E-10	-2.16E-09	-2.84E-09	-3.19E-09	-4.15E-09	-4.09E-09
5.18E-09	4.86E-09	3.79E-09	4.01E-09	3.62E-09	2.98E-09	2.60E-09	2.19E-09	8.96E-10	4.04E-10	-1.62E-09	-2.48E-09	-3.02E-09	-3.67E-09	-3.58E-09
5.30E-09	4.69E-09	3.37E-09	3.85E-09	2.84E-09	3.02E-09	2.64E-09	2.47E-09	1.53E-09	9.08E-10	-1.30E-09	-1.97E-09	-2.27E-09	-3.18E-09	-3.41E-09
4.68E-09	3.65E-09	2.94E-09	2.49E-09	1.78E-09	2.29E-09	2.29E-09	1.85E-09	1.31E-09	1.05E-09	-9.16E-10	-1.22E-09	-2.36E-09	-2.94E-09	-3.64E-09
3.13E-09	1.84E-09	1.63E-09	8.60E-10	7.60E-10	1.18E-09	7.28E-10	4.21E-10	3.83E-10	8.44E-11	-1.47E-09	-1.52E-09	-3.17E-09	-3.37E-09	-4.33E-09
1.18E-09	2.83E-10	-3.63E-10	-7.40E-10	-4.68E-10	-8.44E-10	-8.47E-10	-1.13E-09	-1.18E-09	-1.59E-09	-2.63E-09	-2.08E-09	-3.44E-09	-3.82E-09	-4.79E-09
-1.92E-10	-6.23E-10	-1.98E-09	-1.78E-09	-1.88E-09	-2.03E-09	-1.92E-09	-2.41E-09	-2.51E-09	-3.27E-09	-3.78E-09	-2.76E-09	-4.12E-09	-4.51E-09	-5.37E-09
-1.97E-09	-1.91E-09	-3.40E-09	-2.78E-09	-2.76E-09	-2.85E-09	-3.24E-09	-3.64E-09	-3.98E-09	-4.52E-09	-4.75E-09	-3.42E-09	-4.95E-09	-5.24E-09	-6.01E-09
-3.37E-09	-2.92E-09	-4.29E-09	-3.26E-09	-2.90E-09	-3.34E-09	-4.47E-09	-4.95E-09	-5.36E-09	-5.71E-09	-5.59E-09	-4.58E-09	-6.02E-09	-5.86E-09	-6.39E-09
-4.10E-09	-4.10E-09	-5.07E-09	-3.97E-09	-3.64E-09	-4.14E-09	-5.43E-09	-5.65E-09	-6.34E-09	-6.33E-09	-6.71E-09	-5.92E-09	-6.66E-09	-6.23E-09	-6.67E-09
-4.62E-09	-5.43E-09	-5.85E-09	-4.85E-09	-5.35E-09	-5.51E-09	-6.28E-09	-6.26E-09	-6.94E-09	-6.94E-09	-7.61E-09	-6.92E-09	-6.78E-09	-6.71E-09	-6.82E-09
-5.30E-09	-6.00E-09	-6.29E-09	-6.27E-09	-6.30E-09	-6.94E-09	-6.99E-09	-6.86E-09	-7.31E-09	-7.38E-09	-8.13E-09	-7.42E-09	-6.90E-09	-7.14E-09	-7.05E-09
-5.81E-09	-6.31E-09	-6.74E-09	-7.14E-09	-7.07E-09	-7.47E-09	-7.29E-09	-7.05E-09	-7.49E-09	-7.66E-09	-8.26E-09	-7.98E-09	-7.29E-09	-7.54E-09	-7.38E-09
-6.29E-09	-6.98E-09	-6.70E-09	-7.62E-09	-7.49E-09	-7.34E-09	-7.29E-09	-7.23E-09	-7.92E-09	-7.87E-09	-8.09E-09	-8.13E-09	-7.54E-09	-7.98E-09	-7.34E-09
-6.68E-09	-7.27E-09	-6.45E-09	-7.66E-09	-7.84E-09	-7.28E-09	-7.20E-09	-7.17E-09	-8.42E-09	-8.34E-09	-8.11E-09	-8.44E-09	-7.97E-09	-8.30E-09	-7.97E-09
-6.84E-09	-7.46E-09	-6.94E-09	-7.72E-09	-8.25E-09	-7.42E-09	-7.46E-09	-7.56E-09	-8.67E-09	-8.53E-09	-8.17E-09	-8.71E-09	-8.71E-09	-8.82E-09	-8.82E-09
-7.31E-09	-7.33E-09	-7.39E-09	-7.39E-09	-8.10E-09	-7.74E-09	-8.30E-09	-8.51E-09	-8.67E-09	-8.88E-09	-7.99E-09	-8.54E-09	-8.89E-09	-9.01E-09	-9.16E-09
-7.55E-09	-7.60E-09	-7.61E-09	-7.97E-09	-8.37E-09	-8.69E-09	-8.77E-09	-8.92E-09	-9.00E-09	-8.64E-09	-7.98E-09	-8.56E-09	-8.51E-09	-8.86E-09	-8.84E-09
-7.93E-09	-7.99E-09	-7.66E-09	-8.39E-09	-8.59E-09	-9.49E-09	-9.21E-09	-9.39E-09	-9.26E-09	-8.27E-09	-7.73E-09	-8.56E-09	-8.01E-09	-8.29E-09	-8.85E-09
-8.46E-09	-8.36E-09	-7.68E-09	-8.39E-09	-9.09E-09	-9.59E-09	-9.45E-09	-1.00E-08	-9.35E-09	-8.25E-09	-8.27E-09	-8.84E-09	-7.12E-09	-7.69E-09	-8.65E-09
-8.85E-09	-8.47E-09	-7.68E-09	-8.86E-09	-9.38E-09	-9.22E-09	-9.59E-09	-1.03E-08	-9.42E-09	-8.67E-09	-8.13E-09	-8.37E-09	-6.52E-09	-7.54E-09	-8.93E-09
-8.97E-09	-8.20E-09	-7.90E-09	-9.09E-09	-9.44E-09	-9.34E-09	-9.63E-09	-9.74E-09	-9.30E-09	-8.50E-09	-7.32E-09	-8.17E-09	-6.95E-09	-8.18E-09	-9.25E-09
-9.42E-09	-8.95E-09	-8.33E-09	-9.04E-09	-8.80E-09	-8.75E-09	-9.68E-09	-9.03E-09	-8.96E-09	-8.30E-09	-6.59E-09	-8.51E-09	-8.52E-09	-9.25E-09	-9.41E-09
-9.31E-09	-9.56E-09	-9.12E-09	-9.10E-09	-8.80E-09	-7.17E-09	-9.28E-09	-8.81E-09	-8.60E-09	-8.35E-09	-7.58E-09	-8.86E-09	-9.39E-09	-9.27E-09	-9.14E-09
-8.73E-09	-9.54E-09	-9.60E-09	-9.52E-09	-9.34E-09	-4.97E-09	-8.75E-09	-8.89E-09	-8.94E-09	-9.72E-09	-8.41E-09	-8.87E-09	-9.20E-09	-9.32E-09	-8.78E-09
-8.87E-09	-9.54E-09	-9.79E-09	-1.00E-08	-9.97E-09	-2.30E-09	-8.52E-09	-8.92E-09	-9.39E-09	-9.86E-09	-8.92E-09	-8.99E-09	-8.80E-09	-9.51E-09	-9.03E-09
-9.75E-09	-9.90E-09	-9.56E-09	-9.66E-09	-9.61E-09	-6.90E-09	-9.13E-09	-9.52E-09	-9.52E-09	-9.75E-09	-8.95E-09	-8.77E-09	-8.99E-09	-9.61E-09	-9.25E-09
-9.72E-09	-9.64E-09	-9.48E-09	-9.93E-09	-9.97E-09	-9.36E-09	-9.08E-09	-9.17E-09	-9.75E-09	-1.03E-08	-9.35E-09	-9.44E-09	-9.16E-09	-9.77E-09	-8.95E-09
-9.40E-09	-9.02E-09	-8.91E-09	-9.86E-09	-1.01E-08	-9.86E-09	-9.81E-09	-9.12E-09	-9.64E-09	-1.02E-08	-8.84E-09	-9.37E-09	-9.22E-09	-1.00E-08	-9.35E-09
-8.94E-09	-8.46E-09	-8.30E-09	-9.74E-09	-1.02E-08	-9.96E-09	-9.85E-09	-8.69E-09	-9.22E-09	-9.94E-09	-8.96E-09	-9.39E-09	-9.90E-09	-1.00E-08	-9.20E-09
-8.15E-09	-7.86E-09	-8.20E-09	-9.34E-09	-1.00E-08	-9.81E-09	-9.36E-09	-8.70E-09	-9.27E-09	-9.76E-09	-8.85E-09	-8.97E-09	-9.39E-09	-9.52E-09	-9.28E-09
-7.14E-09	-7.33E-09	-8.41E-09	-9.09E-09	-1.00E-08	-9.60E-09	-9.00E-09	-8.85E-09	-9.55E-09	-9.57E-09	-8.83E-09	-8.93E-09	-8.83E-09	-9.09E-09	-9.43E-09
-7.32E-09	-7.44E-09	-8.51E-09	-9.29E-09	-9.89E-09	-9.33E-09	-8.93E-09	-8.94E-09	-9.58E-09	-9.36E-09	-8.72E-09	-8.89E-09	-8.42E-09	-8.74E-09	-9.07E-09
-7.76E-09	-8.35E-09	-9.04E-09	-9.11E-09	-9.72E-09	-8.97E-09	-8.58E-09	-8.81E-09	-9.48E-09	-9.02E-09	-8.56E-09	-8.97E-09	-8.30E-09	-8.42E-09	-8.68E-09
-8.16E-09	-8.20E-09	-9.40E-09	-8.96E-09	-9.26E-09	-8.41E-09	-8.33E-09	-9.06E-09	-9.25E-09	-8.69E-09	-8.31E-09	-9.16E-09	-8.67E-09	-8.56E-09	-8.39E-09
-8.67E-09	-8.77E-09	-9.35E-09	-8.54E-09	-8.93E-09	-8.54E-09	-8.59E-09	-9.45E-09	-8.72E-09	-8.93E-09	-8.28E-09	-9.10E-09	-8.87E-09	-8.83E-09	-8.51E-09
-8.85E-09	-8.99E-09	-9.50E-09	-8.48E-09	-8.70E-09	-8.49E-09	-8.77E-09	-9.12E-09	-8.25E-09	-8.89E-09	-8.34E-09	-8.73E-09	-8.87E-09	-8.94E-09	-8.78E-09
-8.77E-09	-8.94E-09	-9.39E-09	-8.44E-09	-8.69E-09	-8.29E-09	-8.54E-09	-8.32E-09	-7.41E-09	-8.28E-09	-8.27E-09	-8.40E-09	-8.68E-09	-9.06E-09	-8.47E-09
-8.53E-09	-9.13E-09	-9.08E-09	-8.30E-09	-8.65E-09	-8.19E-09	-7.99E-09	-7.40E-09	-6.21E-09	-7.11E-09	-7.89E-09	-8.15E-09	-8.08E-09	-8.93E-09	-8.02E-09
-8.31E-09	-9.17E-09	-8.76E-09	-8.45E-09	-8.58E-09	-8.00E-09	-7.57E-09	-7.17E-09	-5.97E-09	-6.84E-09	-7.71E-09	-7.77E-09	-8.22E-09	-8.90E-09	-8.18E-09
-8.11E-09	-9.07E-09	-9.05E-09	-8.58E-09	-8.31E-09	-7.66E-09	-6.88E-09	-6.27E-09	-5.62E-09	-7.01E-09	-7.61E-09	-7.70E-09	-8.38E-09	-8.98E-09	-8.33E-09
-7.62E-09	-8.97E-09	-9.06E-09	-8.77E-09	-8.39E-09	-7.11E-09	-5.90E-09	-5.00E-09	-5.18E-09	-7.09E-09	-7.96E-09	-7.76E-09	-8.56E-09	-9.04E-09	-8.76E-09
-6.83E-09	-8.57E-09	-8.74E-09	-8.71E-09	-7.90E-09	-7.03E-09	-6.28E-09	-5.34E-09	-5.33E-09	-7.24E-09	-8.68E-09	-8.27E-09	-8.51E-09	-8.85E-09	-8.79E-09
-6.97E-09	-8.22E-09	-8.35E-09	-7.97E-09	-7.05E-09	-7.04E-09	-7.76E-09	-7.31E-09	-6.67E-09	-7.47E-09	-8.32E-09	-8.06E-09	-8.16E-09	-8.79E-09	-8.49E-09
-8.42E-09	-8.01E-09	-7.94E-09	-7.18E-09	-6.03E-09	-6.63E-09	-7.47E-09	-7.94E-09	-7.36E-09	-7.59E-09	-7.71E-09	-7.13E-09	-7.85E-09	-8.29E-09	-8.10E-09
-7.72E-09	-7.62E-09	-7.62E-09	-6.14E-09	-4.33E-09	-5.34E-09	-6.15E-09	-7.59E-09	-7.19E-09	-7.54E-09	-7.36E-09	-5.95E-09	-7.39E-09	-7.83E-09	-7.51E-09
-7.27E-09	-6.72E-09	-7.07E-09	-5.79E-09	-3.49E-09	-4.12E-09	-4.16E-09	-6.12E-09	-6.20E-09	-6.84E-09	-6.76E-09	-6.30E-09	-7.83E-09	-7.12E-09	-6.57E-09
-6.74E-09	-5.53E-09	-6.14E-09	-5.76E-09	-4.79E-09	-4.17E-09	-2.88E-09	-4.56E-09	-5.37E-09	-6.44E-09	-6.86E-09	-7.33E-09	-8.03E-09	-7.09E-09	-6.89E-09
-6.15E-09	-5.29E-09	-5.56E-09	-5.22E-09	-5.33E-09	-5.29E-09	-3.25E-09	-4.36E-09	-6.29E-09	-6.71E-09	-7.46E-09	-7.77E-09	-7.89E-09	-7.54E-09	-7.73E-09
-5.65E-09	-5.18E-09	-5.27E-09	-4.89E-09	-4.54E-09	-6.58E-09	-4.71E-09	-4.75E-09	-7.04E-09	-7.15E-09	-7.83E-09	-7.41E-09	-7.62E-09	-7.19E-09	-8.14E-09
-5.28E-09	-5.38E-09	-5.53E-09	-5.68E-09	-4.77E-09	-7.37E-09	-6.77E-09	-5.66E-09	-7.38E-09	-7.53E-09	-8.06E-09	-7.39E-09	-7.05E-09	-7.07E-09	-7.97E-09
-5.47E-09	-5.38E-09	-5.68E-09	-6.32E-09	-5.62E-09	-7.46E-09	-7.38E-09	-7.05E-09	-7.60E-09	-7.79E-09	-8.01E-09	-7.54E-09	-7.34E-09	-7.42E-09	-7.78E-09
-5.84E-09	-4.93E-09	-5.15E-09	-6.65E-09	-6.10E-09	-7.26E-09	-7.15E-09	-7.41E-09	-7.98E-09	-7.62E-09	-7.77E-09	-7.60E-09	-7.33E-09	-7.15E-09	-7.05E-09
-6.16E-09	-4.66E-09	-5.34E-09	-6.95E-09	-6.46E-09	-7.11E-09	-7.25E-09	-7.47E-09	-7.91E-09	-7.54E-09	-7.55E-09	-7.25E-09	-6.58E-09	-6.57E-09	-6.23E-09
-6.15E-09	-5.84E-09	-5.62E-09	-6.80E-09	-6.55E-09	-7.21E-09	-7.20E-09	-7.47E-09	-7.49E-09	-6.96E-09	-7.13E-09	-6.63E-09	-6.30E-09	-6.65E-09	-6.29E-09
-6.67E-09	-6.59E-09	-5.36E-09	-6.37E-09	-6.33E-09	-6.46E-09	-6.97E-09	-6.98E-09	-6.84E-09	-6.53E-09	-6.37E-09	-6.15E-09	-6.21E-09	-6.39E-09	-6.37E-09
-6.26E-09	-5.97E-09	-4.49E-09	-5.10E-09	-5.60E-09	-5.80E-09	-6.68E-09	-6.31E-09	-6.50E-09	-5.93E-09	-5.58E-09	-5.55E-09	-6.42E-09	-6.29E-09	-5.97E-09
-5.58E-09	-5.24E-09	-3.07E-09	-3.37E-09	-4.61E-09	-4.81E-09	-6.11E-09	-5.25E-09	-5.98E-09	-5.12E-09	-4.96E-09	-5.05E-09	-6.02E-09	-6.23E-09	-5.54E-09
-4.63E-09	-4.06E-09	-1.50E-09	-1.71E-09	-3.42E-09	-4.59E-09	-4.83E-09	-3.66E-09	-4.67E-09	-3.73E-09	-4.24E-09	-4.98E-09	-4.85E-09	-5.72E-09	-4.96E-09
-4.30E-09	-3.39E-09	-1.30E-09	-5.89E-10	-1.90E-09	-4.12E-09	-3.87E-09	-3.21E-09	-3.74E-09	-2.85E-09	-3.49E-09	-4.60E-09	-3.88E-09	-4.64E-09	-4.68E-09
-4.35E-09	-4.04E-09	-1.41E-09	-1.78E-10	-1.11E-09	-2.80E-09	-3.48E-09	-3.60E-09	-3.11E-09	-2.41E-09	-2.63E-09	-4.04E-09	-2.94E-09	-4.10E-09	-4.83E-09
-4.29E-09	-4.08E-09	-1.47E-09	-5.65E-10	-9.13E-10	-7.17E-10	-1.81E-09	-2.03E-09	-4.37E-10	-1.07E-09	-2.05E-09	-3.83E-09	-3.14E-09	-3.86E-09	-4.83E-09
-4.16E-09	-3.57E-09	-1.67E-09	-9.48E-10	-6.67E-10	1.78E-09	-4.87E-12	2.01E-11	2.36E-09	2.24E-09	-6.28E-10	-3.53E-09	-2.72E-09	-3.57E-09	-4.57E-09
-3.95E-09	-2.98E-09	-8.48E-10	-7.47E-11	-1.67E-10	1.14E-09	8.57E-10	1.99E-09	6.60E-09	6.54E-09	1.32E-09	-2.69E-09	-2.18E-09	-3.39E-09	-4.01E-09
-3.50E-09	-2.23E-09	5.21E-10	1.89E-09	-5.26E-11	-1.93E-10	9.00E-10	2.95E-09	9.36E-09	9.00E-09	1.94E-09	-2.39E-09	-2.28E-09	-3.62E-09	-4.04E-09
-2.66E-09	-1.59E-09	2.76E-09	4.06E-09	2.91E-10	-5.80E-10	6.16E-10	2.98E-09	8.11E-09	5.95E-09	9.65E-10	-2.84E-09	-3.39E-09	-3.89E-09	-3.82E-09

-1.29E-09	-7.85E-10	2.63E-09	3.19E-09	-8.41E-11	-1.18E-09	6.77E-11	2.38E-09	4.64E-09	2.02E-09	-3.77E-10	-2.91E-09	-3.92E-09	-3.78E-09	-3.22E-09
-5.86E-10	-1.44E-10	-2.34E-10	1.61E-09	-4.08E-10	-1.79E-09	-7.78E-10	1.09E-09	2.11E-09	-3.98E-10	-1.46E-09	-2.90E-09	-3.57E-09	-3.76E-09	-3.10E-09
-4.61E-10	-3.50E-10	-2.07E-09	-3.15E-10	-3.88E-10	-1.90E-09	-1.50E-09	-3.21E-10	2.82E-10	-1.16E-09	-1.98E-09	-2.76E-09	-3.15E-09	-3.75E-09	-3.06E-09
-2.05E-09	-1.81E-09	-2.88E-09	-1.79E-09	-1.33E-09	-1.59E-09	-1.37E-09	-1.48E-09	-6.03E-10	-1.10E-09	-2.03E-09	-2.36E-09	-3.14E-09	-3.34E-09	-2.89E-09
-2.92E-09	-2.99E-09	-3.45E-09	-1.99E-09	-1.38E-09	-8.61E-10	-1.00E-09	-1.53E-09	-1.92E-09	-1.13E-09	-1.98E-09	-1.87E-09	-2.82E-09	-2.73E-09	-2.59E-09
-2.75E-09	-3.20E-09	-3.53E-09	-1.80E-09	-2.14E-10	4.99E-11	-8.64E-10	-1.17E-09	-1.94E-09	-9.11E-10	-1.65E-09	-1.24E-09	-2.14E-09	-1.84E-09	-1.97E-09
-2.31E-09	-2.45E-09	-3.34E-09	-1.92E-09	1.69E-10	-2.60E-10	-7.80E-10	-8.82E-10	-1.63E-09	-1.70E-10	-1.22E-09	-2.76E-10	-1.53E-09	-7.52E-10	-1.45E-09
-1.90E-09	-2.06E-09	-2.91E-09	-2.34E-09	-2.45E-10	-8.54E-10	-6.75E-10	-7.95E-10	-1.71E-09	3.32E-10	-8.81E-10	6.75E-10	-4.28E-10	-1.26E-10	-7.88E-10
-1.40E-09	-1.75E-09	-2.63E-09	-2.32E-09	-3.50E-10	-5.73E-10	-2.33E-10	-6.89E-10	-1.42E-09	1.27E-09	1.01E-10	2.09E-09	1.49E-09	9.74E-10	-2.04E-10
-1.19E-09	-1.41E-09	-2.15E-09	-2.35E-09	-9.43E-10	2.62E-10	9.20E-10	-3.68E-10	-8.39E-10	8.86E-10	8.09E-10	3.22E-09	3.76E-09	1.36E-09	4.22E-10
-1.77E-09	-1.71E-09	-2.09E-09	-2.83E-09	-1.23E-09	-2.93E-10	1.21E-09	-3.85E-10	-5.24E-10	4.28E-10	4.47E-10	3.18E-09	3.64E-09	1.45E-09	3.81E-10
-2.16E-09	-2.22E-09	-2.08E-09	-2.55E-09	-1.33E-09	-1.18E-09	8.15E-10	-1.81E-10	-8.44E-10	4.38E-10	-4.51E-11	1.09E-09	3.83E-10	3.51E-10	-1.27E-10
-2.19E-09	-2.42E-09	-1.80E-09	-2.18E-09	-1.07E-09	-1.17E-09	-3.08E-12	-7.28E-10	-1.34E-09	1.17E-11	-1.91E-10	-6.31E-10	-1.24E-09	-1.74E-10	-1.05E-10
-2.00E-09	-2.19E-09	-1.81E-09	-2.08E-09	-8.72E-10	-1.12E-09	-1.19E-09	-7.68E-10	-1.57E-09	-1.06E-09	-6.52E-10	-1.03E-09	-1.10E-09	2.50E-10	4.44E-10
-1.59E-09	-1.53E-09	-1.60E-09	-1.74E-09	-6.58E-10	-6.85E-10	-1.15E-09	-3.66E-10	-1.49E-09	-1.07E-09	-1.13E-09	-9.90E-10	-7.11E-10	-1.92E-10	2.53E-10
-1.32E-09	-1.38E-09	-1.20E-09	-1.03E-09	-2.94E-10	-4.68E-10	-6.06E-10	1.80E-10	-8.61E-10	-4.23E-10	-7.48E-10	-9.18E-10	-2.22E-10	2.88E-11	5.20E-10
-7.19E-10	-8.34E-10	-4.61E-10	-6.33E-10	3.39E-11	-1.88E-10	2.14E-10	4.69E-10	2.90E-11	-2.89E-10	-9.44E-10	-6.07E-10	3.27E-10	9.55E-11	8.85E-10
-3.15E-10	-3.98E-10	1.78E-10	-2.67E-10	3.96E-10	2.24E-10	1.13E-09	1.42E-09	4.61E-10	1.39E-10	-1.33E-10	1.82E-10	8.69E-10	9.17E-11	1.28E-09
7.79E-11	-1.23E-10	6.26E-10	5.84E-10	8.88E-10	6.86E-10	1.65E-09	1.67E-09	8.31E-10	5.01E-10	6.27E-10	1.18E-09	1.68E-09	9.88E-10	1.53E-09
9.83E-10	5.55E-10	1.10E-09	6.58E-10	1.49E-09	1.21E-09	1.93E-09	1.61E-09	1.13E-09	1.13E-09	1.04E-09	2.08E-09	2.57E-09	1.77E-09	1.76E-09
1.46E-09	1.22E-09	1.56E-09	9.96E-10	1.66E-09	1.55E-09	1.88E-09	1.89E-09	1.41E-09	1.30E-09	1.60E-09	1.84E-09	2.63E-09	2.33E-09	2.02E-09
2.02E-09	1.99E-09	2.11E-09	2.14E-09	2.40E-09	2.19E-09	1.67E-09	1.94E-09	1.90E-09	1.95E-09	2.40E-09	2.35E-09	2.96E-09	3.00E-09	2.75E-09
2.77E-09	2.85E-09	2.74E-09	3.23E-09	3.17E-09	2.76E-09	2.06E-09	2.49E-09	2.62E-09	3.15E-09	3.74E-09	2.90E-09	3.19E-09	3.61E-09	3.44E-09
3.33E-09	3.71E-09	3.00E-09	4.19E-09	3.99E-09	2.87E-09	2.85E-09	3.36E-09	3.02E-09	4.09E-09	4.69E-09	3.34E-09	3.86E-09	3.87E-09	4.17E-09
3.73E-09	5.45E-09	3.59E-09	4.90E-09	4.28E-09	3.11E-09	3.38E-09	3.97E-09	3.68E-09	3.28E-09	4.55E-09	3.98E-09	4.64E-09	4.54E-09	5.05E-09
3.83E-09	6.63E-09	4.39E-09	4.98E-09	4.34E-09	3.68E-09	3.59E-09	4.47E-09	4.63E-09	4.27E-09	5.09E-09	4.92E-09	5.32E-09	5.82E-09	5.79E-09
4.21E-09	6.67E-09	4.74E-09	5.31E-09	4.49E-09	4.50E-09	4.35E-09	5.19E-09	5.61E-09	4.89E-09	5.55E-09	5.74E-09	6.15E-09	6.69E-09	6.78E-09
4.97E-09	7.83E-09	5.78E-09	5.83E-09	5.06E-09	5.11E-09	5.06E-09	5.57E-09	5.91E-09	5.36E-09	5.90E-09	6.20E-09	6.75E-09	7.29E-09	7.24E-09
5.87E-09	6.81E-09	6.62E-09	6.50E-09	6.02E-09	6.00E-09	5.87E-09	5.95E-09	5.88E-09	6.02E-09	6.47E-09	6.69E-09	7.75E-09	7.91E-09	7.62E-09
6.52E-09	6.63E-09	7.15E-09	7.67E-09	7.20E-09	7.54E-09	6.80E-09	6.65E-09	6.35E-09	6.57E-09	7.51E-09	7.40E-09	8.25E-09	8.96E-09	8.31E-09
7.24E-09	7.43E-09	7.63E-09	8.56E-09	8.26E-09	8.90E-09	7.84E-09	7.55E-09	7.19E-09	7.26E-09	8.71E-09	8.35E-09	8.95E-09	9.70E-09	8.75E-09
8.30E-09	8.19E-09	8.01E-09	8.86E-09	9.09E-09	9.25E-09	8.94E-09	8.53E-09	8.03E-09	7.97E-09	9.84E-09	9.73E-09	9.98E-09	1.10E-08	9.81E-09
9.10E-09	9.62E-09	8.82E-09	9.50E-09	9.66E-09	9.84E-09	1.01E-08	9.15E-09	9.05E-09	9.08E-09	1.12E-08	1.12E-08	1.12E-08	1.18E-08	1.11E-08
9.83E-09	1.03E-08	9.76E-09	1.04E-08	1.01E-08	1.06E-08	1.09E-08	9.82E-09	1.01E-08	1.01E-08	1.20E-08	1.30E-08	1.21E-08	1.21E-08	1.23E-08
1.07E-08	1.12E-08	1.06E-08	1.13E-08	1.11E-08	1.09E-08	1.12E-08	1.06E-08	1.13E-08	1.08E-08	1.24E-08	1.45E-08	1.26E-08	1.28E-08	1.27E-08
1.14E-08	1.16E-08	1.13E-08	1.20E-08	1.20E-08	1.14E-08	1.21E-08	1.11E-08	1.27E-08	1.23E-08	1.34E-08	1.57E-08	1.28E-08	1.39E-08	1.39E-08
1.22E-08	1.23E-08	1.24E-08	1.25E-08	1.27E-08	1.22E-08	1.25E-08	1.21E-08	1.33E-08	1.35E-08	1.46E-08	1.51E-08	1.39E-08	1.50E-08	1.50E-08
1.33E-08	1.34E-08	1.40E-08	1.32E-08	1.39E-08	1.35E-08	1.31E-08	1.31E-08	1.39E-08	1.47E-08	1.60E-08	1.52E-08	1.53E-08	1.60E-08	1.65E-08
1.49E-08	1.48E-08	1.60E-08	1.41E-08	1.48E-08	1.46E-08	1.40E-08	1.41E-08	1.54E-08	1.57E-08	1.69E-08	1.64E-08	1.63E-08	1.70E-08	1.73E-08
1.62E-08	1.56E-08	1.74E-08	1.54E-08	1.59E-08	1.56E-08	1.57E-08	1.56E-08	1.64E-08	1.66E-08	1.79E-08	1.73E-08	1.72E-08	1.77E-08	1.85E-08
1.73E-08	1.70E-08	1.71E-08	1.66E-08	1.71E-08	1.72E-08	1.76E-08	1.72E-08	1.72E-08	1.75E-08	1.83E-08	1.84E-08	1.82E-08	1.89E-08	1.97E-08
1.82E-08	1.85E-08	1.77E-08	1.81E-08	1.81E-08	1.89E-08	1.94E-08	1.86E-08	1.83E-08	1.84E-08	1.90E-08	1.99E-08	1.96E-08	2.05E-08	2.07E-08
1.94E-08	1.97E-08	1.94E-08	1.96E-08	1.91E-08	2.01E-08	2.01E-08	1.99E-08	1.94E-08	1.95E-08	1.97E-08	2.10E-08	2.12E-08	2.21E-08	2.19E-08
2.01E-08	2.07E-08	2.12E-08	2.09E-08	1.99E-08	2.09E-08	2.08E-08	2.09E-08	2.06E-08	2.09E-08	2.07E-08	2.20E-08	2.25E-08	2.35E-08	2.32E-08
2.13E-08	2.13E-08	2.18E-08	2.19E-08	2.12E-08	2.20E-08	2.20E-08	2.20E-08	2.21E-08	2.24E-08	2.19E-08	2.28E-08	2.36E-08	2.51E-08	2.45E-08
2.27E-08	2.24E-08	2.27E-08	2.27E-08	2.23E-08	2.29E-08	2.29E-08	2.32E-08	2.31E-08	2.36E-08	2.34E-08	2.39E-08	2.49E-08	2.61E-08	2.56E-08
2.39E-08	2.34E-08	2.31E-08	2.37E-08	2.34E-08	2.33E-08	2.40E-08	2.43E-08	2.43E-08	2.52E-08	2.54E-08	2.56E-08	2.66E-08	2.70E-08	2.73E-08
2.49E-08	2.44E-08	2.42E-08	2.46E-08	2.47E-08	2.48E-08	2.57E-08	2.60E-08	2.58E-08	2.67E-08	2.72E-08	2.75E-08	2.79E-08	2.85E-08	2.93E-08
2.59E-08	2.53E-08	2.52E-08	2.58E-08	2.62E-08	2.64E-08	2.70E-08	2.74E-08	2.75E-08	2.80E-08	2.87E-08	2.92E-08	2.99E-08	3.05E-08	3.12E-08
2.71E-08	2.67E-08	2.67E-08	2.72E-08	2.77E-08	2.80E-08	2.83E-08	2.80E-08	2.93E-08	2.90E-08	3.02E-08	3.03E-08	3.13E-08	3.20E-08	3.29E-08

2.47E-17	1.19E-17	6.67E-18	4.47E-18	7.13E-20	4.32E-21	2.04E-18	3.89E-18	9.36E-19	7.72E-18	5.81E-19	1.35E-18	8.45E-18	9.85E-18	5.31E-18
3.57E-17	1.85E-17	1.52E-17	7.80E-18	1.31E-18	8.82E-19	2.75E-18	1.61E-18	1.23E-18	4.90E-18	4.64E-19	1.25E-19	4.48E-18	9.00E-18	2.53E-18
6.39E-17	3.36E-17	2.13E-17	8.68E-18	3.25E-18	4.11E-18	3.02E-18	2.83E-19	1.20E-18	4.10E-18	2.69E-20	2.78E-21	7.11E-19	3.41E-18	1.53E-22
9.79E-17	5.10E-17	2.90E-17	8.44E-18	1.68E-18	3.13E-18	2.86E-18	2.02E-18	1.24E-18	2.81E-18	2.68E-19	9.42E-19	1.16E-18	4.12E-19	4.55E-18
1.30E-16	6.22E-17	3.20E-17	8.71E-18	2.06E-19	1.41E-18	3.77E-18	1.22E-18	2.64E-18	1.89E-18	4.78E-18	5.85E-18	7.94E-18	9.27E-18	1.14E-17
1.48E-16	7.68E-17	3.34E-17	5.56E-18	1.97E-19	1.13E-18	1.61E-18	1.12E-18	5.46E-18	1.55E-18	9.52E-18	1.35E-17	1.39E-17	1.59E-17	1.69E-17
1.38E-16	9.53E-17	4.09E-17	7.75E-18	1.81E-18	6.55E-19	1.21E-20	3.23E-18	7.24E-18	1.91E-18	7.96E-18	1.86E-17	1.53E-17	1.56E-17	1.76E-17
8.76E-17	9.23E-17	5.17E-17	1.27E-17	1.59E-18	1.11E-21	1.75E-18	6.09E-18	8.96E-18	4.97E-18	8.66E-18	1.80E-17	1.16E-17	1.92E-17	1.91E-17
4.27E-17	6.72E-17	5.04E-17	1.21E-17	1.02E-18	3.77E-19	1.77E-18	5.68E-18	6.76E-18	5.77E-18	1.18E-17	1.69E-17	1.08E-17	1.72E-17	2.01E-17
2.08E-17	5.02E-17	5.86E-17	1.71E-17	3.37E-18	5.65E-18	9.42E-21	2.01E-18	1.95E-18	2.75E-18	1.15E-17	1.61E-17	1.16E-17	1.68E-17	1.86E-17
1.80E-17	4.94E-17	6.93E-17	2.74E-17	1.26E-17	1.96E-17	2.99E-18	2.69E-25	3.00E-19	1.53E-18	8.50E-18	1.25E-17	1.19E-17	1.98E-17	1.85E-17
2.22E-17	4.51E-17	5.61E-17	4.06E-17	2.26E-17	2.02E-17	6.98E-18	5.17E-19	4.88E-19	9.71E-19	6.25E-18	9.46E-18	1.04E-17	1.95E-17	2.02E-17
2.52E-17	3.36E-17	2.72E-17	3.00E-17	2.13E-17	1.47E-17	5.00E-18	2.34E-18	1.06E-20	9.66E-20	4.66E-18	8.06E-18	1.02E-17	1.72E-17	1.67E-17
2.68E-17	2.36E-17	1.43E-17	1.61E-17	1.31E-17	8.87E-18	6.78E-18	4.81E-18	8.03E-19	1.63E-19	2.61E-18	6.16E-18	9.13E-18	1.35E-17	1.28E-17
2.81E-17	2.20E-17	1.14E-17	1.48E-17	8.04E-18	9.14E-18	6.98E-18	6.10E-18	2.34E-18	8.25E-19	1.68E-18	3.87E-18	5.17E-18	1.01E-17	1.16E-17
2.19E-17	1.33E-17	8.67E-18	6.18E-18	3.16E-18	5.25E-18	5.24E-18	3.43E-18	1.72E-18	1.11E-18	8.40E-19	1.50E-18	5.56E-18	8.66E-18	1.32E-17
9.80E-18	3.37E-18	2.66E-18	7.40E-19	5.78E-19	1.40E-18	5.30E-19	1.78E-19	1.47E-19	7.12E-21	2.15E-18	2.30E-18	1.01E-17	1.14E-17	1.88E-17
1.40E-18	8.01E-20	1.31E-19	5.47E-19	2.19E-19	7.12E-19	7.17E-19	1.28E-18	1.39E-18	2.54E-18	6.89E-18	4.31E-18	1.18E-17	1.46E-17	2.29E-17
3.68E-20	3.88E-19	3.94E-18	3.18E-18	3.53E-18	4.13E-18	3.70E-18	5.80E-18	6.31E-18	1.07E-17	1.43E-17	7.62E-18	1.70E-17	2.03E-17	2.88E-17
3.89E-18	3.66E-18	1.15E-17	7.75E-18	7.60E-18	8.13E-18	1.05E-17	1.32E-17	1.59E-17	2.04E-17	2.25E-17	1.17E-17	2.45E-17	2.75E-17	3.61E-17
1.13E-17	8.55E-18	1.84E-17	1.06E-17	8.39E-18	1.12E-17	2.00E-17	2.45E-17	2.87E-17	3.26E-17	3.12E-17	2.10E-17	3.63E-17	3.43E-17	4.08E-17
1.68E-17	1.68E-17	2.57E-17	1.58E-17	1.33E-17	1.72E-17	2.94E-17	3.19E-17	4.01E-17	4.01E-17	4.50E-17	3.50E-17	4.43E-17	3.88E-17	4.45E-17
2.13E-17	2.95E-17	3.42E-17	2.35E-17	2.86E-17	3.04E-17	3.95E-17	3.92E-17	4.81E-17	4.82E-17	5.79E-17	4.79E-17	4.59E-17	4.50E-17	4.65E-17
2.81E-17	3.61E-17	3.96E-17	3.93E-17	3.97E-17	4.82E-17	4.89E-17	4.70E-17	5.35E-17	5.44E-17	6.61E-17	5.51E-17	4.76E-17	5.09E-17	4.97E-17
3.37E-17	3.98E-17	4.55E-17	5.10E-17	5.00E-17	5.59E-17	5.31E-17	4.97E-17	5.61E-17	5.87E-17	6.82E-17	6.36E-17	5.32E-17	5.69E-17	5.45E-17
3.96E-17	4.87E-17	4.50E-17	5.81E-17	5.61E-17	5.39E-17	5.32E-17	5.23E-17	6.28E-17	6.19E-17	6.54E-17	6.61E-17	5.68E-17	6.36E-17	5.39E-17
4.46E-17	5.29E-17	4.16E-17	5.86E-17	6.14E-17	5.30E-17	5.19E-17	5.14E-17	7.08E-17	6.96E-17	6.58E-17	7.13E-17	6.34E-17	6.90E-17	6.36E-17
4.68E-17	5.57E-17	4.82E-17	5.96E-17	6.81E-17	5.50E-17	5.57E-17	5.72E-17	7.51E-17	7.28E-17	6.67E-17	7.59E-17	7.59E-17	7.79E-17	7.78E-17
5.34E-17	5.37E-17	5.46E-17	5.46E-17	6.56E-17	5.99E-17	6.90E-17	7.24E-17	7.52E-17	7.88E-17	6.39E-17	7.29E-17	7.90E-17	8.13E-17	8.39E-17
5.69E-17	5.78E-17	5.80E-17	6.35E-17	7.01E-17	7.56E-17	7.69E-17	7.95E-17	8.09E-17	7.46E-17	6.36E-17	7.33E-17	7.25E-17	7.86E-17	7.81E-17
6.28E-17	6.39E-17	5.87E-17	7.04E-17	7.39E-17	9.01E-17	8.48E-17	8.81E-17	8.57E-17	6.85E-17	5.97E-17	7.32E-17	6.42E-17	6.87E-17	7.83E-17

7.15E-17	7.00E-17	5.90E-17	7.05E-17	8.26E-17	9.19E-17	8.93E-17	1.00E-16	8.74E-17	6.80E-17	6.84E-17	7.82E-17	5.06E-17	5.91E-17	7.48E-17
7.84E-17	7.18E-17	5.89E-17	7.86E-17	8.80E-17	8.51E-17	9.19E-17	1.05E-16	8.88E-17	7.51E-17	6.62E-17	7.01E-17	4.25E-17	5.69E-17	7.98E-17
8.04E-17	6.72E-17	6.25E-17	8.27E-17	8.91E-17	8.72E-17	9.27E-17	9.48E-17	8.64E-17	7.22E-17	5.36E-17	6.68E-17	4.83E-17	6.70E-17	8.56E-17
8.87E-17	8.01E-17	6.93E-17	8.17E-17	7.74E-17	7.65E-17	9.36E-17	8.15E-17	8.02E-17	6.89E-17	4.34E-17	7.25E-17	7.26E-17	8.56E-17	8.85E-17
8.67E-17	9.14E-17	8.32E-17	8.27E-17	7.74E-17	5.15E-17	8.61E-17	7.76E-17	7.40E-17	6.96E-17	5.74E-17	7.85E-17	8.82E-17	8.60E-17	8.36E-17
7.62E-17	9.10E-17	9.22E-17	9.05E-17	8.73E-17	2.47E-17	7.65E-17	7.90E-17	8.00E-17	9.44E-17	7.08E-17	7.87E-17	8.47E-17	8.69E-17	7.70E-17
7.87E-17	9.10E-17	9.58E-17	1.00E-16	9.94E-17	5.29E-18	7.26E-17	7.96E-17	8.82E-17	9.72E-17	7.95E-17	8.08E-17	7.74E-17	9.05E-17	8.15E-17
9.52E-17	9.80E-17	9.14E-17	9.33E-17	9.24E-17	4.77E-17	8.34E-17	9.06E-17	9.06E-17	9.50E-17	8.02E-17	7.70E-17	8.09E-17	9.24E-17	8.56E-17
9.45E-17	9.29E-17	8.99E-17	9.86E-17	9.93E-17	8.76E-17	8.24E-17	8.40E-17	9.50E-17	1.07E-16	8.75E-17	8.92E-17	8.39E-17	9.54E-17	8.01E-17
8.83E-17	8.13E-17	7.93E-17	9.71E-17	1.03E-16	9.72E-17	9.63E-17	8.33E-17	9.30E-17	1.04E-16	7.82E-17	8.78E-17	8.50E-17	1.00E-16	8.74E-17
7.99E-17	7.16E-17	6.90E-17	9.49E-17	1.04E-16	9.92E-17	9.71E-17	7.55E-17	8.50E-17	9.87E-17	8.02E-17	8.81E-17	9.79E-17	1.01E-16	8.46E-17
6.65E-17	6.17E-17	6.73E-17	8.72E-17	1.00E-16	9.63E-17	8.75E-17	7.58E-17	8.59E-17	9.52E-17	7.82E-17	8.05E-17	8.82E-17	9.07E-17	8.61E-17
5.09E-17	5.38E-17	7.07E-17	8.25E-17	1.00E-16	9.21E-17	8.10E-17	7.84E-17	9.12E-17	9.15E-17	7.80E-17	7.98E-17	7.79E-17	8.27E-17	8.89E-17
5.36E-17	5.53E-17	7.25E-17	8.63E-17	9.77E-17	8.71E-17	7.97E-17	7.99E-17	9.17E-17	8.76E-17	7.61E-17	7.91E-17	7.09E-17	7.64E-17	8.23E-17
6.03E-17	6.97E-17	8.18E-17	8.31E-17	9.44E-17	8.04E-17	7.36E-17	7.76E-17	8.98E-17	8.14E-17	7.33E-17	8.04E-17	6.88E-17	7.10E-17	7.53E-17
6.65E-17	6.72E-17	8.83E-17	8.03E-17	8.57E-17	7.08E-17	6.94E-17	8.21E-17	8.55E-17	7.56E-17	6.90E-17	8.39E-17	7.51E-17	7.33E-17	7.03E-17
7.51E-17	7.69E-17	8.75E-17	7.29E-17	7.97E-17	7.30E-17	7.38E-17	8.93E-17	7.61E-17	7.97E-17	6.86E-17	8.29E-17	7.87E-17	7.79E-17	7.24E-17
7.83E-17	8.07E-17	9.02E-17	7.19E-17	7.57E-17	7.21E-17	7.69E-17	8.32E-17	6.80E-17	7.89E-17	6.96E-17	7.63E-17	7.86E-17	8.00E-17	7.72E-17
7.69E-17	8.00E-17	8.81E-17	7.12E-17	7.55E-17	6.87E-17	7.29E-17	6.91E-17	5.50E-17	6.85E-17	6.84E-17	7.06E-17	7.54E-17	8.21E-17	7.17E-17
7.28E-17	8.34E-17	8.25E-17	6.89E-17	7.48E-17	6.71E-17	6.39E-17	5.47E-17	3.86E-17	5.05E-17	6.22E-17	6.65E-17	6.54E-17	7.97E-17	6.43E-17
6.91E-17	8.42E-17	7.67E-17	7.14E-17	7.36E-17	6.40E-17	5.73E-17	5.15E-17	3.57E-17	4.68E-17	5.95E-17	6.04E-17	6.76E-17	7.93E-17	6.70E-17
6.57E-17	8.23E-17	8.19E-17	7.36E-17	6.91E-17	5.86E-17	4.73E-17	3.93E-17	3.15E-17	4.91E-17	5.80E-17	5.92E-17	7.03E-17	8.07E-17	6.94E-17
5.80E-17	8.05E-17	8.20E-17	7.70E-17	7.04E-17	5.05E-17	3.48E-17	2.50E-17	2.68E-17	5.02E-17	6.33E-17	6.03E-17	7.33E-17	8.17E-17	7.68E-17
4.67E-17	7.35E-17	7.64E-17	7.58E-17	6.25E-17	4.94E-17	3.94E-17	2.85E-17	2.84E-17	5.24E-17	7.54E-17	6.84E-17	7.23E-17	7.82E-17	7.73E-17
4.85E-17	6.76E-17	6.97E-17	6.34E-17	4.98E-17	4.96E-17	6.03E-17	5.34E-17	4.44E-17	5.58E-17	6.92E-17	6.49E-17	6.65E-17	7.73E-17	7.22E-17
7.09E-17	6.41E-17	6.30E-17	5.15E-17	3.64E-17	4.40E-17	5.58E-17	6.31E-17	5.41E-17	5.75E-17	5.94E-17	5.09E-17	6.16E-17	6.87E-17	6.56E-17
5.96E-17	5.80E-17	5.80E-17	3.77E-17	1.88E-17	2.86E-17	3.79E-17	5.76E-17	5.17E-17	5.69E-17	5.42E-17	3.54E-17	5.46E-17	6.12E-17	5.64E-17
5.28E-17	4.52E-17	5.00E-17	3.36E-17	1.22E-17	1.69E-17	1.73E-17	3.75E-17	3.84E-17	4.68E-17	4.57E-17	3.96E-17	6.14E-17	5.06E-17	4.32E-17
4.55E-17	3.05E-17	3.76E-17	3.32E-17	2.29E-17	1.74E-17	8.32E-18	2.08E-17	2.88E-17	4.15E-17	4.71E-17	5.37E-17	6.45E-17	5.03E-17	4.75E-17
3.78E-17	2.80E-17	3.09E-17	2.73E-17	2.84E-17	2.80E-17	1.06E-17	1.90E-17	3.96E-17	4.50E-17	5.56E-17	6.04E-17	6.22E-17	5.69E-17	5.98E-17
3.19E-17	2.69E-17	2.78E-17	2.39E-17	2.06E-17	4.33E-17	2.22E-17	2.26E-17	4.96E-17	5.11E-17	6.14E-17	5.49E-17	5.81E-17	5.17E-17	6.63E-17
2.78E-17	2.90E-17	3.06E-17	3.23E-17	2.28E-17	5.43E-17	4.58E-17	3.20E-17	5.44E-17	5.68E-17	6.49E-17	5.46E-17	4.97E-17	4.99E-17	6.35E-17
3.00E-17	2.90E-17	3.23E-17	3.99E-17	3.16E-17	5.56E-17	5.45E-17	4.97E-17	5.77E-17	6.08E-17	6.42E-17	5.68E-17	5.38E-17	5.51E-17	6.06E-17
3.41E-17	2.43E-17	2.65E-17	4.43E-17	3.72E-17	5.27E-17	5.11E-17	5.49E-17	6.37E-17	5.81E-17	6.04E-17	5.78E-17	5.37E-17	5.11E-17	4.97E-17
3.80E-17	2.17E-17	2.85E-17	4.83E-17	4.17E-17	5.06E-17	5.25E-17	5.58E-17	6.25E-17	5.68E-17	5.70E-17	5.25E-17	4.33E-17	4.31E-17	3.88E-17
3.79E-17	3.41E-17	3.16E-17	4.63E-17	4.30E-17	5.20E-17	5.18E-17	5.58E-17	5.60E-17	4.85E-17	5.08E-17	4.40E-17	3.97E-17	4.42E-17	3.95E-17
4.45E-17	4.34E-17	2.87E-17	4.05E-17	4.01E-17	4.17E-17	4.85E-17	4.87E-17	4.68E-17	4.26E-17	4.05E-17	3.78E-17	3.86E-17	4.08E-17	4.05E-17
3.91E-17	3.57E-17	2.02E-17	2.60E-17	3.14E-17	3.37E-17	4.46E-17	3.99E-17	4.22E-17	3.52E-17	3.12E-17	3.08E-17	4.13E-17	3.96E-17	3.56E-17
3.11E-17	2.75E-17	9.40E-18	1.14E-17	2.12E-17	2.31E-17	3.73E-17	2.76E-17	3.58E-17	2.62E-17	2.46E-17	2.55E-17	3.62E-17	3.89E-17	3.06E-17
2.15E-17	1.65E-17	2.25E-18	2.93E-18	1.17E-17	2.10E-17	2.33E-17	1.34E-17	2.18E-17	1.39E-17	1.80E-17	2.48E-17	2.35E-17	3.27E-17	2.46E-17
1.85E-17	1.15E-17	1.68E-18	3.47E-19	3.60E-18	1.70E-17	1.50E-17	1.03E-17	1.40E-17	8.13E-18	1.22E-17	2.12E-17	1.51E-17	2.16E-17	2.19E-17
1.89E-17	1.64E-17	1.98E-18	3.17E-20	1.22E-18	7.82E-18	1.21E-17	1.30E-17	9.68E-18	5.80E-18	6.91E-18	1.63E-17	8.62E-18	1.68E-17	2.33E-17
1.84E-17	1.67E-17	2.17E-18	3.19E-19	8.34E-19	5.14E-19	3.28E-18	4.12E-18	1.91E-19	1.15E-18	4.21E-18	1.47E-17	9.85E-18	1.49E-17	2.33E-17
1.73E-17	1.28E-17	2.79E-18	8.99E-19	4.45E-19	3.16E-18	2.38E-23	4.04E-22	5.57E-18	5.01E-18	3.94E-19	1.25E-17	7.43E-18	1.27E-17	2.09E-17
1.56E-17	8.87E-18	7.20E-19	5.58E-21	2.77E-20	1.29E-18	7.34E-19	3.95E-18	4.35E-17	4.28E-17	1.74E-18	7.21E-18	4.76E-18	1.15E-17	1.61E-17
1.22E-17	4.99E-18	2.72E-19	3.56E-18	2.76E-21	3.72E-20	8.11E-19	8.68E-18	8.76E-17	8.11E-17	3.75E-18	5.73E-18	5.22E-18	1.31E-17	1.63E-17
7.07E-18	2.52E-18	7.63E-18	1.64E-17	8.49E-20	3.37E-19	3.79E-19	8.89E-18	6.58E-17	3.54E-17	9.32E-19	8.09E-18	1.15E-17	1.51E-17	1.46E-17
1.67E-18	6.17E-19	6.89E-18	1.02E-17	7.07E-21	1.39E-18	4.59E-21	5.68E-18	2.15E-17	4.07E-18	1.42E-19	8.44E-18	1.54E-17	1.43E-17	1.04E-17
3.43E-19	2.08E-20	5.48E-20	2.58E-18	1.66E-19	3.20E-18	6.06E-19	1.20E-18	4.43E-18	1.59E-19	2.14E-18	8.39E-18	1.28E-17	1.41E-17	9.61E-18
2.13E-19	1.23E-19	4.30E-18	9.90E-20	1.51E-19	3.62E-18	2.25E-18	1.03E-19	7.94E-20	1.34E-18	3.92E-18	7.64E-18	9.89E-18	1.40E-17	9.36E-18
4.20E-18	3.28E-18	8.28E-18	3.20E-18	1.77E-18	2.53E-18	1.88E-18	2.20E-18	3.64E-19	1.22E-18	4.12E-18	5.56E-18	9.83E-18	1.12E-17	8.35E-18
8.54E-18	8.93E-18	1.19E-17	3.95E-18	1.90E-18	7.41E-19	1.01E-18	2.33E-18	3.68E-18	1.27E-18	3.90E-18	3.51E-18	7.97E-18	7.47E-18	6.70E-18
7.56E-18	1.02E-17	1.25E-17	3.25E-18	4.58E-20	2.49E-21	7.46E-19	1.36E-18	3.78E-18	8.30E-19	2.73E-18	1.53E-18	4.59E-18	3.40E-18	3.88E-18
5.34E-18	6.02E-18	1.11E-17	3.67E-18	2.84E-20	6.79E-20	6.09E-19	7.78E-19	2.67E-18	2.89E-20	1.48E-18	7.60E-20	2.33E-18	5.66E-19	2.11E-18
3.62E-18	4.25E-18	8.47E-18	5.46E-18	6.02E-20	7.29E-19	4.56E-19	6.33E-19	2.91E-18	1.10E-19	7.77E-19	4.56E-19	1.83E-19	1.58E-20	6.21E-19
1.97E-18	3.08E-18	6.91E-18	5.37E-18	1.22E-19	3.28E-19	5.41E-20	4.75E-19	2.01E-18	1.62E-18	1.03E-20	4.36E-18	2.23E-18	9.49E-19	4.14E-20
1.41E-18	1.99E-18	4.60E-18	5.50E-18	8.90E-19	6.87E-20	8.47E-19	1.36E-19	7.03E-19	7.84E-19	6.54E-19	1.04E-17	1.42E-17	1.86E-18	1.78E-19
3.12E-18	2.93E-18	4.35E-18	8.01E-18	1.52E-18	8.58E-20	1.46E-18	1.49E-19	2.75E-19	1.84E-19	2.00E-19	1.01E-17	1.33E-17	2.10E-18	1.46E-19
4.68E-18	4.94E-18	4.33E-18	6.52E-18	1.76E-18	1.40E-18	6.64E-19	3.26E-20	7.12E-19	1.92E-19	2.03E-21	1.20E-18	1.46E-19	1.23E-19	1.62E-20
4.79E-18	5.86E-18	3.24E-18	4.74E-18	1.14E-18	1.37E-18	9.51E-24	5.31E-19	1.79E-18	1.36E-22	3.66E-20	3.98E-19	1.53E-18	3.03E-20	1.11E-20
3.98E-18	4.78E-18	3.26E-18	4.33E-18	7.60E-19	1.25E-18	1.42E-18	5.90E-19	2.47E-18	1.12E-18	4.25E-19	1.06E-18	1.22E-18	6.27E-20	1.97E-19
2.51E-18	2.33E-18	2.55E-18	3.02E-18	4.33E-19	4.69E-19	1.32E-18	1.34E-19	2.23E-18	1.15E-18	1.28E-18	9.80E-19	5.06E-19	3.69E-20	6.43E-20
1.73E-18	1.89E-18	1.44E-18	1.07E-18	8.65E-20	2.19E-19	3.67E-19	3.25E-20	7.42E-19	1.79E-19	5.59E-19	8.43E-19	4.91E-20	8.28E-22	2.70E-19
5.17E-19	6.96E-19	2.13E-19	4.00E-19	1.15E-21	3.54E-20	4.60E-20	2.20E-19	8.42E-22	8.33E-20	8.92E-19	3.69E-19	1.07E-19	9.11E-21	7.83E-19
9.95E-20	1.59E-19	3.17E-20	7.13E-20	1.57E-19	5.00E-20	1.28E-18	2.02E-18	2.12E-19	1.92E-20	1.78E-20	3.33E-20	7.56E-19	8.41E-21	1.65E-18
6.06E-21	1.51E-20	3.92E-19	3.41E-19	7.89E-19	4.70E-19	2.72E-18	2.80E-18	6.90E-19	2.51E-19	3.94E-19	1.39E-18	2.83E-18	9.77E-19	2.34E-18
9.67E-19	3.08E-19	1.20E-18	4.33E-19	2.23E-18	1.46E-18	3.72E-18	2.59E-18	1.29E-18	1.28E-18	1.08E-18	4.31E-18	6.62E-18	3.14E-18	3.10E-18
2.13E-18	1.49E-18	2.44E-18	9.92E-19	2.75E-18	2.41E-18	3.54E-18	3.57E-18	1.98E-18	1.68E-18	2.55E-18	3.37E-18	6.91E-18	5.43E-18	4.08E-18
4.09E-18	3.98E-18	4.47E-18	4.59E-18	5.75E-18	4.81E-18	2.79E-18	3.77E-18	3.60E-18	3.80E-18	5.77E-18	5.53E-18	8.79E-18	9.03E-18	7.55E-18
7.70E-18	8.10E-18	7.53E-18	1.04E-17	1.00E-17	7.62E-18	4.23E-18	6.18E-18	6.89E-18	9.93E-18	1.40E-17	8.39E-18	1.02E-17	1.30E-17	1.18E-17
1.11E-17	1.38E-17	9.00E-18	1.75E-17	1.59E-17	8.22E-18	8.15E-18	1.13E-17	9.15E-18	1.68E-17	2.20E-17	1.12E-17	1.49E-17	1.50E-17	1.74E-17
1.39E-17	2.97E-17	1.29E-17	2.40E-17	1.83E-17	9.66E-18	1.14E-17	1.58E-17	1.35E-17	1.07E-17	2.07E-17	1.58E-17	2.16E-17	2.06E-17	2.55E-17
1.47E-17	4.39E-17	1.93E-17	2.48E-17	1.89E-17	1.35E-17	1.29E-17	2.00E-17	2.14E-17	1.82E-17	2.59E-17	2.42E-17	2.83E-17	3.38E-17	3.36E-17
1.78E-17	4.45E-17	2.24E-17	2.82E-17	2.01E-17	2.02E-17	1.89E-17	2.70E-17	3.15E-17	2.39E-17	3.08E-17	3.30E-17	3.78E-17	4.48E-17	4.60E-17
2.47E-17	6.14E-17	3.34E-17	3.40E-17	2.56E-17	2.61E-17	2.56E-17	3.11E-17	3.49E-17	2.87E-17	3.48E-17	3.84E-17	4.56E-17	5.31E-17	5.25E-17
3.45E-17	4.64E-17	4.39E-17	4.23E-17	3.63E-17	3.60E-17	3.45E-17	3.54E-17	3.46E-17	3.62E-17	4.19E-17	4.47E-17	6.01E-17	6.26E-17	5.81E-17
4.25E-17	4.39E-17	5.11E-17	5.88E-17	5.19E-17	5.68E-17	4.63E-17	4.42E-17	4.04E-17	4.31E-17	5.64E-17	5.48E-17	6.81E-17	8.02E-17	6.90E-17
5.24E-17	5.53E-17	5.83E-17	7.32E-17	6.82E-17	7.92E-17	6.14E-17	5.71E-17	5.16E-17	5.27E-17	7.58E-17	6.98E-17	8.01E-17	9.41E-17	7.66E-17
6.89E-17	6.71E-17	6.41E-17	7.85E-17	8.25E-17	8.56E-17	7.99E-17	7.28E-17	6.44E-17	6.35E-17	9.69E-17	9.48E-17	9.95E-17	1.21E-16	9.62E-17
8.28E-17	9.26E-17	7.77E-17	9.03E-17	9.32E-17	9.68E-17	1.01E-16	8.37E-17	8.20E-17	8.24E-17	1.25E-16	1.25E-16	1.26E-16	1.40E-16	1.24E-16
9.67E-17	1.07E-16	9.53E-17	1.08E-16	1.03E-16	1.13E-16	1.19E-16	9.65E-17	1.02E-16	1.02E-16	1.44E-16	1.70E-16	1.47E-16	1.47E-16	1.51E-16
1.14E-16	1.25E-16	1.12E-16	1.28E-16	1.23E-16	1.19E-16	1.26E-16	1.13E-16	1.28E-16	1.17E-16	1.55E-16	2.10E-16	1.59E-16	1.65E-16	1.62E-16
1.30E-16	1.34E-16	1.28E-16	1.44E-16	1.45E-16	1.31E-16	1.47E-16	1.23E-16	1.62E-16	1.51E-16	1.80E-16	2.48E-16	1.65E-16	1.93E-16	1.92E-16
1.48E-16	1.52E-16	1.54E-16	1.55E-16	1.62E-16	1.50E-16	1.56E-16	1.47E-16	1.77E-16	1.84E-16	2.12E-16	2.27E-16	1.95E-16	2.24E-16	2.26E-16
1.77E-16	1.80E-16	1.97E-16	1.74E-16	1.92E-16	1.82E-16	1.71E-16	1.72E-16	1.94E-16	2.16E-16	2.57E-16	2.32E-16	2.34E-16	2.56E-16	2.71E-16
2.23E-16	2.18E-16	2.57E-16	2.00E-16	2.20E-16	2.12E-16	1.96E-16	1.97E-16	2.36E-16	2.48E-16	2.87E-16	2.69E-16	2.65E-16	2.88E-16	2.99E-16

2.61E-16	2.42E-16	3.02E-16	2.37E-16	2.53E-16	2.43E-16	2.46E-16	2.43E-16	2.69E-16	2.77E-16	3.19E-16	2.99E-16	2.97E-16	3.14E-16	3.42E-16
2.98E-16	2.91E-16	2.92E-16	2.77E-16	2.91E-16	2.97E-16	3.10E-16	2.96E-16	2.95E-16	3.08E-16	3.36E-16	3.39E-16	3.32E-16	3.56E-16	3.90E-16
3.31E-16	3.41E-16	3.12E-16	3.28E-16	3.28E-16	3.56E-16	3.77E-16	3.45E-16	3.34E-16	3.40E-16	3.59E-16	3.96E-16	3.83E-16	4.19E-16	4.27E-16
3.76E-16	3.89E-16	3.77E-16	3.86E-16	3.66E-16	4.04E-16	4.02E-16	3.98E-16	3.75E-16	3.81E-16	3.87E-16	4.41E-16	4.49E-16	4.89E-16	4.79E-16
4.05E-16	4.27E-16	4.50E-16	4.35E-16	3.95E-16	4.38E-16	4.31E-16	4.38E-16	4.25E-16	4.37E-16	4.27E-16	4.82E-16	5.06E-16	5.54E-16	5.39E-16
4.52E-16	4.52E-16	4.77E-16	4.78E-16	4.48E-16	4.86E-16	4.83E-16	4.83E-16	4.87E-16	5.01E-16	4.82E-16	5.20E-16	5.55E-16	6.28E-16	6.01E-16
5.14E-16	5.03E-16	5.15E-16	5.16E-16	4.99E-16	5.23E-16	5.22E-16	5.39E-16	5.34E-16	5.58E-16	5.50E-16	5.69E-16	6.19E-16	6.79E-16	6.56E-16
5.71E-16	5.48E-16	5.35E-16	5.63E-16	5.48E-16	5.45E-16	5.76E-16	5.89E-16	5.92E-16	6.34E-16	6.43E-16	6.56E-16	7.07E-16	7.29E-16	7.43E-16
6.20E-16	5.94E-16	5.84E-16	6.07E-16	6.09E-16	6.13E-16	6.58E-16	6.76E-16	6.68E-16	7.11E-16	7.42E-16	7.59E-16	7.80E-16	8.11E-16	8.56E-16
6.72E-16	6.41E-16	6.37E-16	6.67E-16	6.88E-16	6.96E-16	7.30E-16	7.50E-16	7.57E-16	7.84E-16	8.23E-16	8.55E-16	8.92E-16	9.28E-16	9.74E-16
7.36E-16	7.12E-16	7.11E-16	7.39E-16	7.65E-16	7.84E-16	8.00E-16	7.86E-16	8.61E-16	8.40E-16	9.15E-16	9.18E-16	9.82E-16	1.03E-15	1.08E-15
1.04E-14	1.04E-14	1.01E-14	1.00E-14	9.78E-15	9.74E-15	1.00E-14	9.97E-15	1.05E-14	1.07E-14	1.11E-14	1.16E-14	1.19E-14	1.28E-14	1.29E-14

7.27E-10	2.42E-10	-1.80E-09	-2.17E-09	-3.78E-09	-4.94E-09	-5.02E-09	-5.01E-09	-4.66E-09	-3.57E-09	-4.33E-09	-4.86E-09	-5.39E-09	-4.67E-09
6.18E-10	3.60E-10	-2.51E-09	-2.21E-09	-4.07E-09	-4.73E-09	-5.33E-09	-5.20E-09	-4.70E-09	-4.57E-09	-4.98E-09	-5.03E-09	-5.25E-09	-4.95E-09
-7.27E-10	-9.40E-10	-2.67E-09	-2.19E-09	-4.31E-09	-4.83E-09	-5.81E-09	-5.40E-09	-5.08E-09	-5.13E-09	-5.54E-09	-5.25E-09	-4.96E-09	-5.19E-09
-2.21E-09	-1.60E-09	-3.11E-09	-2.76E-09	-4.45E-09	-5.11E-09	-5.92E-09	-5.42E-09	-5.41E-09	-5.09E-09	-5.88E-09	-5.78E-09	-5.19E-09	-5.35E-09
-3.22E-09	-2.85E-09	-4.29E-09	-4.34E-09	-4.49E-09	-5.03E-09	-5.67E-09	-5.60E-09	-5.16E-09	-4.69E-09	-5.82E-09	-6.01E-09	-6.31E-09	-5.87E-09
-4.69E-09	-4.06E-09	-5.11E-09	-4.97E-09	-4.64E-09	-4.81E-09	-5.40E-09	-5.14E-09	-5.02E-09	-4.41E-09	-5.46E-09	-6.07E-09	-6.94E-09	-6.67E-09
-4.81E-09	-4.31E-09	-5.23E-09	-4.97E-09	-4.45E-09	-4.49E-09	-5.39E-09	-5.47E-09	-5.85E-09	-5.14E-09	-5.97E-09	-6.08E-09	-7.18E-09	-6.79E-09
-4.25E-09	-4.44E-09	-5.21E-09	-5.02E-09	-4.51E-09	-4.70E-09	-5.89E-09	-5.95E-09	-6.75E-09	-6.32E-09	-6.43E-09	-6.45E-09	-7.60E-09	-6.70E-09
-4.41E-09	-4.35E-09	-5.28E-09	-5.33E-09	-5.35E-09	-5.20E-09	-6.72E-09	-6.14E-09	-7.26E-09	-6.43E-09	-6.05E-09	-6.67E-09	-7.43E-09	-6.49E-09
-4.28E-09	-4.68E-09	-5.22E-09	-5.43E-09	-6.24E-09	-5.89E-09	-7.15E-09	-6.36E-09	-7.39E-09	-6.58E-09	-5.52E-09	-6.66E-09	-7.23E-09	-6.47E-09
-3.94E-09	-4.48E-09	-5.44E-09	-5.71E-09	-6.77E-09	-5.98E-09	-6.92E-09	-6.96E-09	-7.18E-09	-6.28E-09	-5.86E-09	-6.85E-09	-7.13E-09	-6.58E-09
-4.01E-09	-4.67E-09	-5.65E-09	-5.73E-09	-6.92E-09	-6.01E-09	-6.63E-09	-6.99E-09	-6.66E-09	-6.35E-09	-6.76E-09	-7.02E-09	-7.36E-09	-7.07E-09
-3.95E-09	-4.47E-09	-5.06E-09	-5.52E-09	-6.68E-09	-6.21E-09	-6.53E-09	-6.87E-09	-6.33E-09	-6.95E-09	-7.18E-09	-6.92E-09	-7.68E-09	-7.34E-09
-3.76E-09	-4.01E-09	-4.78E-09	-5.23E-09	-6.49E-09	-6.64E-09	-6.67E-09	-7.68E-09	-6.71E-09	-7.32E-09	-7.56E-09	-7.26E-09	-8.42E-09	-8.08E-09
-3.56E-09	-3.71E-09	-4.91E-09	-5.00E-09	-6.60E-09	-6.43E-09	-7.50E-09	-8.08E-09	-7.65E-09	-7.82E-09	-7.83E-09	-7.66E-09	-8.43E-09	-8.32E-09
-4.04E-09	-4.24E-09	-5.16E-09	-5.07E-09	-6.71E-09	-6.46E-09	-7.67E-09	-7.91E-09	-8.21E-09	-8.05E-09	-8.15E-09	-8.19E-09	-8.31E-09	-8.33E-09
-4.87E-09	-4.87E-09	-5.50E-09	-5.23E-09	-7.01E-09	-6.58E-09	-7.87E-09	-8.17E-09	-8.54E-09	-8.41E-09	-8.04E-09	-8.33E-09	-8.30E-09	-8.88E-09
-5.70E-09	-5.48E-09	-5.75E-09	-6.16E-09	-7.17E-09	-6.91E-09	-8.04E-09	-8.54E-09	-8.57E-09	-8.57E-09	-7.97E-09	-8.43E-09	-8.59E-09	-9.17E-09
-6.02E-09	-5.71E-09	-5.94E-09	-6.93E-09	-7.02E-09	-7.27E-09	-8.17E-09	-8.60E-09	-8.87E-09	-8.61E-09	-7.59E-09	-8.10E-09	-8.99E-09	-9.38E-09
-6.09E-09	-5.91E-09	-6.01E-09	-7.06E-09	-7.24E-09	-7.23E-09	-8.18E-09	-8.39E-09	-8.34E-09	-8.56E-09	-8.11E-09	-8.09E-09	-8.91E-09	-9.70E-09
-6.19E-09	-6.40E-09	-6.53E-09	-6.83E-09	-7.30E-09	-6.69E-09	-8.12E-09	-8.75E-09	-8.46E-09	-8.81E-09	-9.06E-09	-8.30E-09	-8.98E-09	-9.99E-09
-6.06E-09	-6.95E-09	-6.65E-09	-6.63E-09	-7.10E-09	-6.92E-09	-8.27E-09	-8.94E-09	-8.38E-09	-8.90E-09	-9.43E-09	-8.53E-09	-9.32E-09	-1.03E-08
-6.01E-09	-7.57E-09	-6.77E-09	-6.54E-09	-7.33E-09	-7.91E-09	-8.84E-09	-9.31E-09	-8.17E-09	-8.90E-09	-9.55E-09	-8.86E-09	-9.73E-09	-1.05E-08
-7.29E-09	-7.93E-09	-7.30E-09	-7.00E-09	-7.79E-09	-8.54E-09	-8.91E-09	-9.16E-09	-8.43E-09	-8.62E-09	-9.44E-09	-9.20E-09	-1.01E-08	-1.06E-08
-8.00E-09	-8.30E-09	-7.86E-09	-7.66E-09	-8.30E-09	-8.96E-09	-8.59E-09	-8.91E-09	-8.71E-09	-8.86E-09	-9.47E-09	-9.52E-09	-1.04E-08	-1.09E-08
-8.24E-09	-8.68E-09	-8.27E-09	-8.64E-09	-8.19E-09	-9.24E-09	-8.90E-09	-8.85E-09	-9.22E-09	-9.51E-09	-9.75E-09	-9.52E-09	-1.08E-08	-1.09E-08
-8.47E-09	-8.61E-09	-8.52E-09	-9.00E-09	-8.53E-09	-9.50E-09	-9.12E-09	-8.88E-09	-9.77E-09	-9.90E-09	-9.99E-09	-9.67E-09	-1.08E-08	-1.06E-08
-9.05E-09	-8.89E-09	-8.55E-09	-9.09E-09	-8.84E-09	-9.68E-09	-9.14E-09	-9.08E-09	-9.81E-09	-9.62E-09	-9.99E-09	-9.67E-09	-1.06E-08	-1.07E-08
-9.14E-09	-9.26E-09	-8.50E-09	-9.11E-09	-8.95E-09	-9.69E-09	-9.26E-09	-9.35E-09	-9.76E-09	-9.93E-09	-1.05E-08	-1.05E-08	-1.02E-08	-1.11E-08
-9.24E-09	-9.47E-09	-8.27E-09	-8.90E-09	-8.89E-09	-9.83E-09	-9.46E-09	-9.65E-09	-9.73E-09	-1.06E-08	-1.11E-08	-1.14E-08	-1.04E-08	-1.15E-08
-8.97E-09	-9.17E-09	-7.95E-09	-8.64E-09	-9.54E-09	-9.91E-09	-9.80E-09	-9.81E-09	-9.58E-09	-1.04E-08	-1.11E-08	-1.15E-08	-1.06E-08	-1.14E-08
-9.06E-09	-9.46E-09	-8.09E-09	-8.65E-09	-9.48E-09	-1.03E-08	-1.03E-08	-1.02E-08	-1.01E-08	-1.08E-08	-1.09E-08	-1.13E-08	-1.14E-08	-1.12E-08
-9.20E-09	-9.48E-09	-8.22E-09	-8.67E-09	-9.41E-09	-1.06E-08	-1.06E-08	-1.01E-08	-9.82E-09	-1.04E-08	-1.06E-08	-1.13E-08	-1.18E-08	-1.14E-08
-9.17E-09	-9.24E-09	-8.56E-09	-9.22E-09	-9.59E-09	-1.05E-08	-1.05E-08	-1.00E-08	-9.45E-09	-1.03E-08	-1.07E-08	-1.08E-08	-1.14E-08	-1.14E-08
-9.41E-09	-9.39E-09	-8.81E-09	-9.57E-09	-9.67E-09	-1.03E-08	-9.93E-09	-9.35E-09	-9.39E-09	-1.02E-08	-1.07E-08	-1.05E-08	-1.13E-08	-1.18E-08
-9.36E-09	-9.29E-09	-8.56E-09	-9.65E-09	-9.38E-09	-1.02E-08	-9.78E-09	-8.75E-09	-9.59E-09	-1.01E-08	-1.06E-08	-1.05E-08	-1.08E-08	-1.16E-08
-9.34E-09	-9.10E-09	-8.65E-09	-9.41E-09	-9.32E-09	-1.02E-08	-1.01E-08	-8.92E-09	-9.38E-09	-9.88E-09	-1.03E-08	-1.08E-08	-1.05E-08	-1.11E-08
-9.47E-09	-9.10E-09	-8.68E-09	-9.30E-09	-1.02E-08	-1.02E-08	-9.80E-09	-9.09E-09	-9.68E-09	-1.02E-08	-1.04E-08	-1.08E-08	-9.95E-09	-1.09E-08
-9.52E-09	-9.29E-09	-9.09E-09	-8.43E-09	-9.84E-09	-1.01E-08	-9.56E-09	-9.32E-09	-9.62E-09	-1.02E-08	-1.04E-08	-1.05E-08	-9.98E-09	-1.09E-08
-9.39E-09	-9.57E-09	-9.37E-09	-8.75E-09	-9.31E-09	-9.54E-09	-9.33E-09	-9.46E-09	-9.90E-09	-1.01E-08	-1.03E-08	-1.07E-08	-1.03E-08	-1.08E-08
-9.09E-09	-9.43E-09	-9.26E-09	-8.86E-09	-9.02E-09	-9.25E-09	-9.21E-09	-9.41E-09	-9.61E-09	-9.96E-09	-1.01E-08	-1.05E-08	-1.06E-08	-1.05E-08
-8.79E-09	-8.97E-09	-9.70E-09	-9.56E-09	-8.89E-09	-9.48E-09	-9.34E-09	-9.09E-09	-8.84E-09	-9.58E-09	-9.44E-09	-1.05E-08	-1.02E-08	-1.08E-08
-8.83E-09	-8.91E-09	-9.83E-09	-9.41E-09	-8.56E-09	-9.26E-09	-9.65E-09	-9.02E-09	-8.50E-09	-9.33E-09	-9.15E-09	-9.86E-09	-1.01E-08	-1.10E-08
-8.73E-09	-8.48E-09	-8.92E-09	-9.13E-09	-8.47E-09	-9.10E-09	-9.30E-09	-8.59E-09	-8.42E-09	-9.05E-09	-8.99E-09	-9.38E-09	-9.47E-09	-1.08E-08
-8.47E-09	-7.69E-09	-8.35E-09	-8.86E-09	-8.12E-09	-8.56E-09	-8.72E-09	-8.22E-09	-7.91E-09	-8.10E-09	-8.22E-09	-8.02E-09	-8.50E-09	-9.90E-09
-8.51E-09	-7.44E-09	-8.12E-09	-8.63E-09	-8.28E-09	-7.90E-09	-7.67E-09	-7.09E-09	-6.22E-09	-6.09E-09	-6.79E-09	-6.33E-09	-7.59E-09	-9.19E-09
-8.83E-09	-8.07E-09	-8.22E-09	-8.43E-09	-8.18E-09	-7.07E-09	-6.35E-09	-5.59E-09	-4.72E-09	-4.53E-09	-6.32E-09	-5.52E-09	-6.61E-09	-8.17E-09
-8.96E-09	-7.99E-09	-8.05E-09	-8.34E-09	-8.47E-09	-7.17E-09	-6.52E-09	-4.85E-09	-4.66E-09	-4.83E-09	-6.47E-09	-5.91E-09	-6.46E-09	-7.38E-09
-9.20E-09	-8.23E-09	-7.85E-09	-8.38E-09	-8.52E-09	-7.57E-09	-7.39E-09	-5.60E-09	-5.54E-09	-5.72E-09	-6.18E-09	-6.07E-09	-5.78E-09	-6.60E-09
-9.11E-09	-8.32E-09	-8.14E-09	-8.56E-09	-8.69E-09	-8.12E-09	-8.05E-09	-7.48E-09	-6.07E-09	-5.63E-09	-5.30E-09	-5.89E-09	-5.60E-09	-6.43E-09
-8.91E-09	-8.69E-09	-9.19E-09	-8.85E-09	-8.67E-09	-8.08E-09	-8.51E-09	-7.76E-09	-6.19E-09	-5.04E-09	-4.31E-09	-4.78E-09	-5.73E-09	-7.50E-09
-8.97E-09	-9.07E-09	-9.82E-09	-8.93E-09	-8.83E-09	-8.25E-09	-8.94E-09	-8.28E-09	-6.56E-09	-4.44E-09	-3.67E-09	-4.27E-09	-6.47E-09	-8.24E-09
-8.87E-09	-9.13E-09	-9.61E-09	-8.71E-09	-9.10E-09	-8.65E-09	-8.84E-09	-8.24E-09	-6.95E-09	-4.73E-09	-4.13E-09	-4.65E-09	-6.55E-09	-8.78E-09
-9.02E-09	-9.28E-09	-9.38E-09	-8.97E-09	-9.20E-09	-8.32E-09	-8.42E-09	-8.02E-09	-7.11E-09	-5.68E-09	-6.15E-09	-6.24E-09	-7.22E-09	-8.94E-09
-8.82E-09	-9.27E-09	-9.44E-09	-9.23E-09	-9.23E-09	-7.83E-09	-7.22E-09	-7.95E-09	-7.47E-09	-6.52E-09	-6.88E-09	-8.15E-09	-8.45E-09	-8.84E-09
-8.62E-09	-9.12E-09	-9.17E-09	-9.22E-09	-9.28E-09	-8.23E-09	-6.48E-09	-8.20E-09	-7.62E-09	-7.69E-09	-7.52E-09	-9.36E-09	-9.16E-09	-8.98E-09
-8.27E-09	-8.81E-09	-8.52E-09	-9.08E-09	-9.41E-09	-8.60E-09	-7.53E-09	-8.86E-09	-7.59E-09	-8.82E-09	-8.77E-09	-9.46E-09	-9.53E-09	-9.23E-09
-7.63E-09	-8.25E-09	-8.84E-09	-9.20E-09	-9.38E-09	-8.80E-09	-8.84E-09	-9.96E-09	-8.89E-09	-9.27E-09	-9.16E-09	-9.80E-09	-9.65E-09	-9.46E-09
-7.20E-09	-8.39E-09	-8.81E-09	-9.06E-09	-9.46E-09	-9.14E-09	-9.24E-09	-1.00E-08	-9.51E-09	-9.21E-09	-8.98E-09	-9.90E-09	-9.40E-09	-9.44E-09
-6.97E-09	-7.87E-09	-7.83E-09	-8.48E-09	-9.50E-09	-9.55E-09	-9.02E-09	-9.02E-09	-9.14E-09	-9.01E-09	-8.85E-09	-9.61E-09	-9.14E-09	-9.28E-09
-7.55E-09	-7.68E-09	-7.23E-09	-7.97E-09	-9.05E-09	-9.24E-09	-7.93E-09	-7.71E-09	-7.74E-09	-8.24E-09	-8.43E-09	-9.33E-09	-9.17E-09	-9.55E-09
-7.85E-09	-7.51E-09	-7.44E-09	-8.00E-09	-8.36E-09	-8.47E-09	-6.35E-09	-5.99E-09	-6.14E-09	-6.94E-09	-7.60E-09	-9.16E-09	-9.47E-09	-9.41E-09
-7.85E-09	-7.63E-09	-7.74E-09	-7.18E-09	-7.07E-09	-7.35E-09	-4.54E-09	-3.70E-09	-4.52E-09	-6.01E-09	-6.99E-09	-8.81E-09	-9.42E-09	-9.23E-09
-7.31E-09	-7.59E-09	-7.81E-09	-7.25E-09	-6.39E-09	-6.78E-09	-3.81E-09	-2.06E-09	-2.72E-09	-5.57E-09	-6.92E-09	-8.24E-09	-9.22E-09	-9.39E-09
-6.87E-09	-7.38E-09	-7.83E-09	-7.83E-09	-6.86E-09	-7.12E-09	-5.15E-09	-2.77E-09	-2.77E-09	-5.67E-09	-6.63E-09	-7.85E-09	-9.13E-09	-9.27E-09
-6.68E-09	-7.27E-09	-7.74E-09	-8.32E-09	-7.43E-09	-7.90E-09	-7.22E-09	-5.75E-09	-5.18E-09	-7.03E-09	-6.82E-09	-7.43E-09	-9.32E-09	-9.15E-09
-6.82E-09	-7.43E-09	-7.83E-09	-8.08E-09	-7.71E-09	-8.14E-09	-8.29E-09	-7.81E-09	-6.99E-09	-8.24E-09	-7.81E-09	-7.34E-09	-9.21E-09	-8.93E-09
-7.27E-09	-7.44E-09	-7.73E-09	-7.41E-09	-7.27E-09	-7.75E-09	-8.16E-09	-8.29E-09	-7.45E-09	-8.55E-09	-8.64E-09	-8.32E-09	-9.18E-09	-8.31E-09
-7.25E-09	-7.00E-09	-7.05E-09	-6.45E-09	-6.84E-09	-7.49E-09	-7.44E-09	-7.58E-09	-7.28E-09	-8.27E-09	-8.66E-09	-8.54E-09	-8.86E-09	-7.80E-09
-6.68E-09	-6.08E-09	-6.13E-09	-5.71E-09	-6.62E-09	-7.30E-09	-7.26E-09	-6.84E-09	-7.09E-09	-8.01E-09	-8.37E-09	-8.37E-09	-8.36E-09	-7.72E-09
-5.96E-09	-5.37E-09	-5.40E-09	-5.38E-09	-6.38E-09	-7.18E-09	-7.09E-09	-6.79E-09	-7.39E-09	-8.04E-09	-8.43E-09	-8.48E-09	-8.61E-09	-7.73E-09
-5.43E-09	-5.75E-09	-5.55E-09	-5.54E-09	-5.94E-09	-6.29E-09	-5.95E-09	-6.09E-09	-7.33E-09	-7.92E-09	-8.79E-09	-8.63E-09	-8.70E-09	-7.38E-09
-5.55E-09	-6.03E-09	-5.69E-09	-5.85E-09	-5.74E-09	-4.96E-09	-4.28E-09	-4.76E-09	-6.36E-09	-7.49E-09	-8.69E-09	-8.65E-09	-8.46E-09	-6.90E-09
-5.23E-09	-5.38E-09	-4.98E-09	-5.60E-09	-4.95E-09	-4.00E-09	-2.83E-09	-4.02E-09	-6.14E-09	-7.31E-09	-8.87E-09	-8.19E-09	-7.93E-09	-6.84E-09
-4.40E-09	-4.82E-09	-4.25E-09	-5.02E-09	-4.27E-09	-2.96E-09	-2.29E-09	-3.57E-09	-6.32E-09	-7.07E-09	-8.59E-09	-7.88E-09	-7.51E-09	-6.62E-09
-3.42E-09	-4.10E-09	-3.52E-09	-4.92E-09	-3.98E-09	-2.88E-09	-2.53E-09	-3.39E-09	-6.16E-09	-6.85E-09	-8.38E-09	-7.45E-09	-7.09E-09	-6.53E-09
-2.62E-09	-3.21E-09	-2.97E-09	-4.78E-09	-4.12E-09	-3.88E-09	-4.01E-09	-4.21E-09	-6.27E-09	-7.06E-09	-7.91E-09	-7.25E-09	-6.72E-09	-6.93E-09
-2.31E-09	-3.49E-09	-3.46E-09	-4.82E-09	-5.11E-09	-4.99E-09	-4.96E-09	-4.87E-09	-6.03E-09	-6.80E-09	-7.31E-09	-7.30E-09	-6.73E-09	-7.05E-09

-2.49E-09	-3.64E-09	-4.36E-09	-4.94E-09	-5.13E-09	-5.02E-09	-4.33E-09	-4.02E-09	-5.32E-09	-6.59E-09	-7.15E-09	-7.08E-09	-6.80E-09	-6.69E-09
-2.78E-09	-3.49E-09	-4.63E-09	-5.05E-09	-4.58E-09	-5.02E-09	-3.81E-09	-3.54E-09	-5.10E-09	-5.89E-09	-6.73E-09	-6.72E-09	-6.61E-09	-6.21E-09
-2.53E-09	-3.03E-09	-4.28E-09	-4.86E-09	-3.95E-09	-4.27E-09	-3.83E-09	-3.65E-09	-4.93E-09	-5.33E-09	-5.79E-09	-5.77E-09	-6.02E-09	-6.08E-09
-2.06E-09	-3.09E-09	-3.86E-09	-4.53E-09	-3.43E-09	-3.53E-09	-3.65E-09	-3.64E-09	-5.03E-09	-4.87E-09	-4.73E-09	-4.19E-09	-4.93E-09	-5.60E-09
-2.23E-09	-3.15E-09	-3.41E-09	-3.93E-09	-2.66E-09	-3.20E-09	-3.04E-09	-3.77E-09	-5.21E-09	-5.38E-09	-4.62E-09	-3.42E-09	-4.21E-09	-5.00E-09
-2.01E-09	-2.70E-09	-3.12E-09	-3.48E-09	-2.69E-09	-3.44E-09	-3.69E-09	-4.94E-09	-5.78E-09	-5.49E-09	-5.05E-09	-4.06E-09	-4.30E-09	-3.70E-09
-2.11E-09	-2.26E-09	-3.14E-09	-3.39E-09	-3.40E-09	-3.36E-09	-3.83E-09	-5.62E-09	-6.01E-09	-5.36E-09	-4.90E-09	-4.90E-09	-4.42E-09	-2.07E-09
-2.23E-09	-1.97E-09	-2.72E-09	-2.82E-09	-4.04E-09	-2.96E-09	-3.24E-09	-5.14E-09	-6.01E-09	-5.03E-09	-4.68E-09	-4.76E-09	-4.28E-09	-2.76E-09
-1.78E-09	-1.48E-09	-2.37E-09	-2.58E-09	-3.49E-09	-2.65E-09	-3.35E-09	-4.69E-09	-5.46E-09	-4.40E-09	-4.01E-09	-4.27E-09	-4.07E-09	-3.42E-09
-8.24E-10	-1.22E-09	-2.48E-09	-2.84E-09	-2.96E-09	-2.16E-09	-3.59E-09	-4.12E-09	-4.52E-09	-3.62E-09	-3.43E-09	-3.60E-09	-3.86E-09	-3.47E-09
-5.44E-10	-5.83E-10	-2.24E-09	-2.05E-09	-1.78E-09	-2.35E-09	-3.01E-09	-3.68E-09	-4.18E-09	-3.25E-09	-2.81E-09	-3.34E-09	-3.43E-09	-3.47E-09
-4.58E-10	-4.74E-10	-1.52E-09	-9.61E-10	-5.08E-10	-1.38E-09	-2.03E-09	-3.17E-09	-3.69E-09	-2.77E-09	-2.63E-09	-2.76E-09	-3.07E-09	-3.19E-09
-3.34E-11	-1.45E-10	-6.18E-10	4.88E-10	2.95E-10	-1.92E-10	-6.16E-10	-1.79E-09	-3.14E-09	-2.27E-09	-2.50E-09	-2.24E-09	-2.66E-09	-2.40E-09
5.20E-11	3.61E-10	3.04E-10	1.35E-09	7.70E-10	6.49E-10	4.71E-10	-5.46E-10	-1.94E-09	-1.92E-09	-1.91E-09	-1.58E-09	-2.00E-09	-1.56E-09
-3.33E-10	2.00E-10	-1.01E-10	1.59E-09	7.33E-10	6.68E-10	7.74E-10	4.41E-10	-4.73E-10	-9.83E-10	-1.18E-09	-9.94E-10	-1.32E-09	-9.69E-10
-4.52E-10	-7.23E-12	-5.26E-10	1.16E-09	5.62E-10	3.43E-10	1.08E-09	1.30E-09	1.04E-09	-1.41E-10	-3.97E-10	-4.00E-10	-8.02E-10	-4.30E-10
-8.56E-11	6.02E-10	-3.18E-11	9.46E-10	3.80E-10	-1.46E-10	8.56E-10	2.21E-09	1.02E-09	6.66E-10	1.17E-09	5.44E-10	-2.96E-10	-8.81E-11
5.41E-10	1.53E-09	9.00E-10	1.51E-09	8.22E-10	-4.24E-11	1.18E-09	8.37E-10	8.47E-10	1.11E-09	2.18E-09	1.10E-09	4.22E-10	2.86E-10
1.15E-09	1.91E-09	1.54E-09	1.83E-09	9.38E-10	8.32E-10	1.50E-09	5.64E-10	9.62E-10	1.45E-09	1.97E-09	1.15E-09	9.96E-10	1.01E-09
1.50E-09	1.41E-09	2.29E-09	2.64E-09	1.62E-09	1.54E-09	1.66E-09	1.43E-09	1.50E-09	1.83E-09	1.80E-09	1.25E-09	1.78E-09	1.77E-09
2.07E-09	2.04E-09	3.02E-09	4.02E-09	2.25E-09	1.91E-09	2.26E-09	2.02E-09	2.14E-09	2.88E-09	2.22E-09	2.10E-09	2.84E-09	2.22E-09
2.94E-09	2.94E-09	3.54E-09	4.94E-09	2.88E-09	2.63E-09	3.31E-09	2.39E-09	2.78E-09	2.74E-09	2.85E-09	3.00E-09	3.72E-09	2.78E-09
3.60E-09	3.62E-09	3.92E-09	2.96E-09	3.40E-09	3.52E-09	3.84E-09	3.10E-09	3.80E-09	3.48E-09	3.67E-09	3.59E-09	4.28E-09	3.60E-09
4.12E-09	3.86E-09	4.50E-09	3.67E-09	4.40E-09	4.45E-09	4.69E-09	4.19E-09	5.34E-09	4.21E-09	4.28E-09	4.29E-09	5.06E-09	4.61E-09
5.15E-09	4.46E-09	5.22E-09	4.84E-09	5.56E-09	5.54E-09	5.22E-09	5.36E-09	6.04E-09	5.05E-09	5.10E-09	5.27E-09	5.95E-09	5.87E-09
5.71E-09	5.40E-09	6.11E-09	5.75E-09	6.53E-09	6.24E-09	5.70E-09	6.72E-09	7.26E-09	6.54E-09	6.32E-09	6.22E-09	6.63E-09	7.60E-09
6.69E-09	6.04E-09	6.55E-09	6.37E-09	7.22E-09	7.12E-09	6.38E-09	7.55E-09	8.24E-09	7.60E-09	7.14E-09	7.33E-09	8.00E-09	9.05E-09
7.79E-09	7.28E-09	7.59E-09	7.51E-09	7.80E-09	7.70E-09	7.23E-09	7.46E-09	8.90E-09	8.36E-09	7.67E-09	8.63E-09	9.68E-09	9.98E-09
8.49E-09	8.29E-09	8.81E-09	8.23E-09	8.45E-09	8.17E-09	7.79E-09	7.87E-09	9.13E-09	8.84E-09	8.89E-09	1.03E-08	1.09E-08	1.13E-08
9.09E-09	8.83E-09	9.81E-09	9.22E-09	9.22E-09	8.79E-09	8.83E-09	9.07E-09	1.00E-08	9.85E-09	1.03E-08	1.16E-08	1.18E-08	1.21E-08
1.00E-08	9.80E-09	1.02E-08	1.02E-08	1.04E-08	9.88E-09	1.03E-08	1.07E-08	1.10E-08	1.07E-08	1.16E-08	1.28E-08	1.30E-08	1.34E-08
1.11E-08	1.10E-08	1.12E-08	1.12E-08	1.15E-08	1.10E-08	1.11E-08	1.18E-08	1.21E-08	1.17E-08	1.25E-08	1.33E-08	1.36E-08	1.43E-08
1.16E-08	1.21E-08	1.24E-08	1.19E-08	1.31E-08	1.21E-08	1.20E-08	1.25E-08	1.33E-08	1.25E-08	1.34E-08	1.38E-08	1.42E-08	1.49E-08
1.27E-08	1.31E-08	1.30E-08	1.28E-08	1.45E-08	1.35E-08	1.31E-08	1.36E-08	1.41E-08	1.37E-08	1.40E-08	1.50E-08	1.50E-08	1.56E-08
1.32E-08	1.28E-08	1.36E-08	1.36E-08	1.48E-08	1.45E-08	1.46E-08	1.47E-08	1.47E-08	1.48E-08	1.55E-08	1.55E-08	1.62E-08	1.64E-08
1.40E-08	1.37E-08	1.45E-08	1.46E-08	1.56E-08	1.55E-08	1.58E-08	1.65E-08	1.58E-08	1.61E-08	1.71E-08	1.66E-08	1.76E-08	1.74E-08
1.53E-08	1.51E-08	1.56E-08	1.58E-08	1.68E-08	1.69E-08	1.71E-08	1.80E-08	1.67E-08	1.75E-08	1.81E-08	1.78E-08	1.92E-08	1.87E-08
1.69E-08	1.65E-08	1.66E-08	1.65E-08	1.78E-08	1.81E-08	1.83E-08	1.93E-08	1.80E-08	1.91E-08	1.90E-08	1.96E-08	2.04E-08	1.96E-08
1.85E-08	1.83E-08	1.79E-08	1.77E-08	1.87E-08	1.90E-08	1.87E-08	1.88E-08	1.96E-08	2.08E-08	2.02E-08	2.12E-08	2.14E-08	2.06E-08
1.99E-08	1.98E-08	1.92E-08	1.92E-08	2.01E-08	2.03E-08	1.93E-08	1.99E-08	2.04E-08	2.16E-08	2.11E-08	2.18E-08	2.20E-08	2.16E-08
2.09E-08	2.06E-08	2.03E-08	2.08E-08	2.15E-08	2.15E-08	2.07E-08	2.13E-08	2.10E-08	2.19E-08	2.22E-08	2.19E-08	2.26E-08	2.29E-08
2.16E-08	2.20E-08	2.17E-08	2.28E-08	2.26E-08	2.28E-08	2.19E-08	2.28E-08	2.22E-08	2.28E-08	2.37E-08	2.32E-08	2.41E-08	2.44E-08
2.27E-08	2.34E-08	2.34E-08	2.41E-08	2.36E-08	2.40E-08	2.33E-08	2.34E-08	2.33E-08	2.45E-08	2.53E-08	2.50E-08	2.53E-08	2.59E-08
2.35E-08	2.46E-08	2.45E-08	2.48E-08	2.45E-08	2.54E-08	2.43E-08	2.42E-08	2.42E-08	2.59E-08	2.68E-08	2.66E-08	2.66E-08	2.74E-08
2.45E-08	2.57E-08	2.61E-08	2.64E-08	2.58E-08	2.67E-08	2.58E-08	2.55E-08	2.60E-08	2.75E-08	2.80E-08	2.80E-08	2.83E-08	2.84E-08
2.63E-08	2.69E-08	2.77E-08	2.83E-08	2.73E-08	2.80E-08	2.74E-08	2.69E-08	2.77E-08	2.88E-08	2.95E-08	2.95E-08	2.97E-08	2.97E-08
2.81E-08	2.85E-08	2.92E-08	2.96E-08	2.88E-08	2.92E-08	2.88E-08	2.82E-08	2.90E-08	3.00E-08	3.07E-08	3.06E-08	3.06E-08	3.07E-08
2.96E-08	3.03E-08	3.12E-08	3.07E-08	2.98E-08	2.99E-08	2.99E-08	2.95E-08	3.03E-08	3.12E-08	3.20E-08	3.20E-08	3.25E-08	3.23E-08
3.18E-08	3.28E-08	3.21E-08	3.17E-08	3.09E-08	3.11E-08	3.13E-08	3.11E-08	3.14E-08	3.22E-08	3.29E-08	3.32E-08	3.43E-08	3.42E-08
3.35E-08	3.41E-08	3.26E-08	3.28E-08	3.24E-08	3.33E-08	3.33E-08	3.26E-08	3.26E-08	3.34E-08	3.39E-08	3.45E-08	3.62E-08	3.62E-08

5.28E-19	5.86E-20	3.22E-18	4.70E-18	1.43E-17	2.44E-17	2.52E-17	2.51E-17	2.17E-17	1.27E-17	1.88E-17	2.36E-17	2.91E-17	2.18E-17
3.82E-19	1.29E-19	6.30E-18	4.88E-18	1.66E-17	2.24E-17	2.84E-17	2.71E-17	2.21E-17	2.09E-17	2.48E-17	2.53E-17	2.75E-17	2.45E-17
5.28E-19	8.84E-19	7.15E-18	4.81E-18	1.86E-17	2.33E-17	3.38E-17	2.91E-17	2.58E-17	2.63E-17	3.07E-17	2.75E-17	2.46E-17	2.69E-17
4.87E-18	2.57E-18	9.67E-18	7.64E-18	1.98E-17	2.61E-17	3.50E-17	2.94E-17	2.92E-17	2.59E-17	3.45E-17	3.34E-17	2.69E-17	2.87E-17
1.03E-17	8.13E-18	1.84E-17	1.88E-17	2.02E-17	2.53E-17	3.21E-17	3.14E-17	2.66E-17	2.20E-17	3.39E-17	3.62E-17	3.98E-17	3.45E-17
2.20E-17	1.65E-17	2.61E-17	2.47E-17	2.15E-17	2.31E-17	2.92E-17	2.64E-17	2.52E-17	1.94E-17	2.98E-17	3.69E-17	4.82E-17	4.45E-17
2.32E-17	1.86E-17	2.73E-17	2.47E-17	1.98E-17	2.01E-17	2.91E-17	2.99E-17	3.42E-17	2.64E-17	3.57E-17	3.69E-17	5.16E-17	4.61E-17
1.81E-17	1.98E-17	2.71E-17	2.52E-17	2.03E-17	2.21E-17	3.47E-17	3.55E-17	4.55E-17	4.00E-17	4.14E-17	4.16E-17	5.78E-17	4.48E-17
1.94E-17	1.89E-17	2.78E-17	2.84E-17	2.87E-17	2.70E-17	4.52E-17	3.76E-17	5.28E-17	4.14E-17	3.66E-17	4.44E-17	5.51E-17	4.21E-17
1.83E-17	2.19E-17	2.73E-17	2.94E-17	3.90E-17	3.47E-17	5.11E-17	4.04E-17	5.45E-17	4.33E-17	3.05E-17	4.43E-17	5.22E-17	4.19E-17
1.56E-17	2.01E-17	2.96E-17	3.26E-17	4.59E-17	3.57E-17	4.79E-17	4.84E-17	5.16E-17	3.95E-17	3.44E-17	4.69E-17	5.08E-17	4.34E-17
1.61E-17	2.18E-17	3.20E-17	3.28E-17	4.79E-17	3.61E-17	4.39E-17	4.89E-17	4.43E-17	4.03E-17	4.56E-17	4.93E-17	5.42E-17	4.99E-17
1.56E-17	2.00E-17	2.56E-17	3.04E-17	4.47E-17	3.86E-17	4.27E-17	4.72E-17	4.01E-17	4.84E-17	5.15E-17	4.79E-17	5.90E-17	5.39E-17
1.41E-17	1.61E-17	2.28E-17	2.73E-17	4.21E-17	4.41E-17	4.45E-17	5.90E-17	4.50E-17	5.36E-17	5.71E-17	5.27E-17	7.10E-17	6.53E-17
1.27E-17	1.38E-17	2.41E-17	2.50E-17	4.35E-17	4.14E-17	5.63E-17	6.53E-17	5.85E-17	6.11E-17	6.14E-17	5.87E-17	7.10E-17	6.93E-17
1.63E-17	1.80E-17	2.66E-17	2.57E-17	4.51E-17	4.18E-17	5.89E-17	6.26E-17	6.75E-17	6.48E-17	6.64E-17	6.71E-17	6.90E-17	6.94E-17
2.37E-17	2.37E-17	3.02E-17	2.73E-17	4.91E-17	4.32E-17	6.19E-17	6.68E-17	7.28E-17	7.08E-17	6.47E-17	6.94E-17	6.90E-17	7.88E-17
3.25E-17	3.01E-17	3.31E-17	3.80E-17	5.14E-17	4.77E-17	6.47E-17	7.29E-17	7.35E-17	7.34E-17	6.35E-17	7.11E-17	7.37E-17	8.42E-17
3.62E-17	3.27E-17	3.53E-17	4.81E-17	4.93E-17	5.29E-17	6.67E-17	7.40E-17	7.86E-17	7.41E-17	5.76E-17	6.57E-17	8.08E-17	8.80E-17
3.71E-17	3.49E-17	3.62E-17	4.98E-17	5.25E-17	5.23E-17	6.69E-17	7.04E-17	6.96E-17	7.33E-17	6.57E-17	6.55E-17	7.94E-17	9.41E-17
3.84E-17	4.09E-17	4.26E-17	4.67E-17	5.34E-17	4.48E-17	6.59E-17	7.66E-17	7.16E-17	7.76E-17	8.21E-17	6.90E-17	8.07E-17	9.98E-17
3.67E-17	4.82E-17	4.42E-17	4.39E-17	5.05E-17	4.79E-17	6.84E-17	7.99E-17	7.03E-17	7.92E-17	8.89E-17	7.27E-17	8.70E-17	1.06E-16
3.61E-17	5.73E-17	4.59E-17	4.28E-17	5.38E-17	6.26E-17	7.82E-17	8.67E-17	6.67E-17	7.92E-17	9.12E-17	7.85E-17	9.46E-17	1.09E-16
5.31E-17	6.29E-17	5.33E-17	4.90E-17	6.07E-17	7.29E-17	7.95E-17	8.39E-17	7.11E-17	7.42E-17	8.91E-17	8.47E-17	1.03E-16	1.12E-16
6.40E-17	6.89E-17	6.19E-17	5.86E-17	6.90E-17	8.03E-17	7.38E-17	7.95E-17	7.58E-17	7.84E-17	8.96E-17	9.07E-17	1.09E-16	1.18E-16
6.78E-17	7.54E-17	6.84E-17	7.46E-17	6.71E-17	8.54E-17	7.92E-17	7.83E-17	8.49E-17	9.05E-17	9.51E-17	9.06E-17	1.17E-16	1.19E-16
7.17E-17	7.41E-17	7.26E-17	8.11E-17	7.28E-17	9.02E-17	8.31E-17	7.88E-17	9.55E-17	9.80E-17	9.98E-17	9.36E-17	1.17E-16	1.12E-16
8.18E-17	7.90E-17	7.30E-17	8.26E-17	7.82E-17	9.37E-17	8.36E-17	8.24E-17	9.62E-17	9.25E-17	9.98E-17	9.35E-17	1.13E-16	1.14E-16
8.35E-17	8.58E-17	7.22E-17	8.30E-17	8.00E-17	9.38E-17	8.57E-17	8.75E-17	9.53E-17	9.85E-17	1.10E-16	1.10E-16	1.04E-16	1.24E-16
8.54E-17	8.96E-17	6.83E-17	7.93E-17	7.90E-17	9.67E-17	8.94E-17	9.31E-17	9.46E-17	1.12E-16	1.23E-16	1.29E-16	1.07E-16	1.31E-16
8.05E-17	8.42E-17	6.32E-17	7.46E-17	9.10E-17	9.83E-17	9.61E-17	9.62E-17	9.18E-17	1.07E-16	1.24E-16	1.31E-16	1.13E-16	1.30E-16

8.22E-17	8.96E-17	6.55E-17	7.49E-17	8.99E-17	1.06E-16	1.07E-16	1.05E-16	1.02E-16	1.16E-16	1.18E-16	1.29E-16	1.29E-16	1.25E-16
8.47E-17	8.98E-17	6.75E-17	7.52E-17	8.85E-17	1.12E-16	1.13E-16	1.02E-16	9.64E-17	1.07E-16	1.12E-16	1.28E-16	1.39E-16	1.31E-16
8.41E-17	8.54E-17	7.32E-17	8.49E-17	9.21E-17	1.11E-16	1.11E-16	1.01E-16	8.92E-17	1.05E-16	1.14E-16	1.17E-16	1.30E-16	1.30E-16
8.85E-17	8.82E-17	7.76E-17	9.16E-17	9.36E-17	1.06E-16	9.86E-17	8.74E-17	8.82E-17	1.04E-16	1.14E-16	1.10E-16	1.28E-16	1.40E-16
8.76E-17	8.63E-17	7.32E-17	9.31E-17	8.81E-17	1.03E-16	9.56E-17	7.66E-17	9.19E-17	1.02E-16	1.12E-16	1.10E-16	1.18E-16	1.34E-16
8.71E-17	8.29E-17	7.49E-17	8.86E-17	8.69E-17	1.04E-16	1.01E-16	7.96E-17	8.79E-17	9.77E-17	1.06E-16	1.17E-16	1.10E-16	1.23E-16
8.97E-17	8.29E-17	7.53E-17	8.66E-17	1.03E-16	1.04E-16	9.59E-17	8.26E-17	9.36E-17	1.04E-16	1.08E-16	1.16E-16	9.90E-17	1.18E-16
9.07E-17	8.63E-17	8.26E-17	7.11E-17	9.67E-17	1.01E-16	9.14E-17	8.68E-17	9.25E-17	1.04E-16	1.09E-16	1.10E-16	9.97E-17	1.19E-16
8.82E-17	9.15E-17	8.78E-17	7.65E-17	8.66E-17	9.11E-17	8.70E-17	8.96E-17	9.81E-17	1.02E-16	1.06E-16	1.14E-16	1.06E-16	1.18E-16
8.26E-17	8.90E-17	8.57E-17	7.86E-17	8.13E-17	8.55E-17	8.48E-17	8.86E-17	9.23E-17	9.91E-17	1.02E-16	1.11E-16	1.12E-16	1.11E-16
7.73E-17	8.04E-17	9.41E-17	9.13E-17	7.91E-17	9.00E-17	8.73E-17	8.26E-17	7.82E-17	9.17E-17	8.92E-17	1.10E-16	1.05E-16	1.16E-16
7.79E-17	7.95E-17	9.66E-17	8.85E-17	7.32E-17	8.57E-17	9.31E-17	8.14E-17	7.23E-17	8.71E-17	8.38E-17	9.72E-17	1.02E-16	1.21E-16
7.63E-17	7.20E-17	7.96E-17	8.34E-17	7.17E-17	8.28E-17	8.65E-17	7.38E-17	7.09E-17	8.19E-17	8.07E-17	8.79E-17	8.97E-17	1.16E-16
7.17E-17	5.92E-17	6.97E-17	7.86E-17	6.59E-17	7.33E-17	7.60E-17	6.75E-17	6.26E-17	6.56E-17	6.75E-17	6.44E-17	7.23E-17	9.80E-17
7.24E-17	5.53E-17	6.59E-17	7.45E-17	6.86E-17	6.25E-17	5.88E-17	5.03E-17	3.87E-17	3.71E-17	4.60E-17	4.00E-17	5.76E-17	8.45E-17
7.80E-17	6.51E-17	6.75E-17	7.10E-17	6.69E-17	5.00E-17	4.03E-17	3.12E-17	2.23E-17	2.05E-17	4.00E-17	3.04E-17	4.37E-17	6.68E-17
8.03E-17	6.38E-17	6.47E-17	6.96E-17	7.17E-17	5.14E-17	4.25E-17	2.35E-17	2.17E-17	2.33E-17	4.18E-17	3.50E-17	4.17E-17	5.45E-17
8.47E-17	6.77E-17	6.16E-17	7.02E-17	7.26E-17	5.73E-17	5.46E-17	3.13E-17	3.07E-17	3.27E-17	3.82E-17	3.69E-17	3.34E-17	4.36E-17
8.29E-17	6.92E-17	6.63E-17	7.33E-17	7.55E-17	6.60E-17	6.47E-17	5.60E-17	3.69E-17	3.17E-17	2.81E-17	3.47E-17	3.14E-17	4.13E-17
7.94E-17	7.56E-17	8.44E-17	7.83E-17	7.51E-17	6.53E-17	7.25E-17	6.03E-17	3.83E-17	2.54E-17	1.86E-17	2.28E-17	3.29E-17	5.63E-17
8.05E-17	8.23E-17	9.64E-17	7.97E-17	7.80E-17	6.81E-17	7.99E-17	6.85E-17	4.30E-17	1.97E-17	1.35E-17	1.82E-17	4.18E-17	6.79E-17
7.87E-17	8.33E-17	9.24E-17	7.59E-17	8.29E-17	7.48E-17	7.82E-17	6.79E-17	4.82E-17	2.24E-17	1.70E-17	2.16E-17	4.29E-17	7.71E-17
8.14E-17	8.60E-17	8.81E-17	8.05E-17	8.46E-17	6.93E-17	7.09E-17	6.43E-17	5.06E-17	3.22E-17	3.78E-17	3.89E-17	5.22E-17	7.99E-17
7.78E-17	8.60E-17	8.91E-17	8.51E-17	8.52E-17	6.13E-17	5.22E-17	6.32E-17	5.57E-17	4.25E-17	4.74E-17	6.64E-17	7.15E-17	7.81E-17
7.44E-17	8.31E-17	8.40E-17	8.51E-17	8.61E-17	6.78E-17	4.21E-17	6.73E-17	5.80E-17	5.92E-17	5.66E-17	8.77E-17	8.39E-17	8.07E-17
6.84E-17	7.75E-17	7.26E-17	8.24E-17	8.85E-17	7.39E-17	5.66E-17	7.85E-17	5.76E-17	7.79E-17	7.69E-17	8.95E-17	9.09E-17	8.52E-17
5.83E-17	6.81E-17	7.81E-17	8.47E-17	8.80E-17	7.74E-17	7.82E-17	9.91E-17	7.90E-17	8.59E-17	8.39E-17	9.61E-17	9.31E-17	8.95E-17
5.19E-17	7.04E-17	7.76E-17	8.22E-17	8.95E-17	8.36E-17	8.53E-17	1.00E-16	9.04E-17	8.48E-17	8.06E-17	9.80E-17	8.83E-17	8.90E-17
4.85E-17	6.19E-17	6.13E-17	7.19E-17	9.02E-17	9.13E-17	8.14E-17	8.14E-17	8.35E-17	8.12E-17	7.83E-17	9.24E-17	8.35E-17	8.60E-17
5.70E-17	5.90E-17	5.23E-17	6.35E-17	8.20E-17	8.53E-17	6.29E-17	5.95E-17	6.00E-17	6.78E-17	7.11E-17	8.71E-17	8.41E-17	9.12E-17
6.16E-17	5.64E-17	5.53E-17	6.40E-17	6.99E-17	7.17E-17	4.03E-17	3.59E-17	3.77E-17	4.81E-17	5.78E-17	8.40E-17	8.97E-17	8.86E-17
6.16E-17	5.82E-17	5.99E-17	5.15E-17	4.99E-17	5.41E-17	2.06E-17	1.37E-17	2.04E-17	3.61E-17	4.89E-17	7.77E-17	8.86E-17	8.51E-17
5.34E-17	5.76E-17	6.10E-17	5.25E-17	4.09E-17	4.59E-17	1.45E-17	4.24E-18	7.39E-18	3.10E-17	4.79E-17	6.80E-17	8.49E-17	8.81E-17
4.72E-17	5.45E-17	6.13E-17	6.13E-17	4.71E-17	5.07E-17	2.66E-17	7.67E-18	7.69E-18	3.22E-17	4.40E-17	6.16E-17	8.34E-17	8.59E-17
4.46E-17	5.29E-17	5.99E-17	6.93E-17	5.53E-17	6.24E-17	5.22E-17	3.31E-17	2.69E-17	4.94E-17	4.65E-17	5.52E-17	8.68E-17	8.38E-17
4.66E-17	5.52E-17	6.12E-17	6.53E-17	5.95E-17	6.62E-17	6.88E-17	6.11E-17	4.89E-17	6.78E-17	6.10E-17	5.39E-17	8.48E-17	7.97E-17
5.29E-17	5.54E-17	5.98E-17	5.49E-17	5.29E-17	6.01E-17	6.66E-17	6.88E-17	5.55E-17	7.31E-17	7.47E-17	6.93E-17	8.43E-17	6.90E-17
5.26E-17	4.91E-17	4.97E-17	4.16E-17	4.68E-17	5.62E-17	5.54E-17	5.75E-17	5.30E-17	6.84E-17	7.49E-17	7.29E-17	7.84E-17	6.09E-17
4.46E-17	3.70E-17	3.76E-17	3.26E-17	4.38E-17	5.33E-17	5.27E-17	4.67E-17	5.03E-17	6.41E-17	7.00E-17	7.00E-17	6.99E-17	5.95E-17
3.55E-17	2.88E-17	2.92E-17	2.89E-17	4.07E-17	5.15E-17	5.03E-17	4.61E-17	5.46E-17	6.46E-17	7.11E-17	7.19E-17	7.41E-17	5.97E-17
2.95E-17	3.30E-17	3.08E-17	3.07E-17	3.53E-17	3.96E-17	3.54E-17	3.70E-17	5.38E-17	6.27E-17	7.74E-17	7.45E-17	7.56E-17	5.45E-17
3.08E-17	3.64E-17	3.24E-17	3.42E-17	3.30E-17	2.46E-17	1.83E-17	2.27E-17	4.04E-17	5.61E-17	7.55E-17	7.48E-17	7.16E-17	4.76E-17
2.73E-17	2.90E-17	2.48E-17	3.13E-17	2.45E-17	1.60E-17	8.03E-18	1.61E-17	3.77E-17	5.34E-17	7.87E-17	6.71E-17	6.28E-17	4.67E-17
1.94E-17	2.32E-17	1.81E-17	2.52E-17	1.82E-17	8.75E-18	5.25E-18	1.28E-17	4.00E-17	4.99E-17	7.38E-17	6.21E-17	5.64E-17	4.38E-17
1.17E-17	1.68E-17	1.24E-17	2.42E-17	1.59E-17	8.30E-18	6.38E-18	1.15E-17	3.79E-17	4.69E-17	7.02E-17	5.56E-17	5.02E-17	4.26E-17
6.85E-18	1.03E-17	8.83E-18	2.29E-17	1.70E-17	1.51E-17	1.61E-17	1.77E-17	3.94E-17	4.98E-17	6.25E-17	5.26E-17	4.52E-17	4.80E-17
5.36E-18	1.22E-17	1.20E-17	2.32E-17	2.62E-17	2.49E-17	2.46E-17	2.38E-17	3.63E-17	4.62E-17	5.35E-17	5.32E-17	4.53E-17	4.96E-17
6.18E-18	1.32E-17	1.90E-17	2.44E-17	2.63E-17	2.52E-17	1.87E-17	1.62E-17	2.83E-17	4.34E-17	5.11E-17	5.02E-17	4.63E-17	4.47E-17
7.75E-18	1.22E-17	2.14E-17	2.55E-17	2.10E-17	2.52E-17	1.45E-17	1.25E-17	2.60E-17	3.47E-17	4.53E-17	4.51E-17	4.37E-17	3.85E-17
6.42E-18	9.18E-18	1.83E-17	2.36E-17	1.56E-17	1.83E-17	1.47E-17	1.33E-17	2.43E-17	2.85E-17	3.36E-17	3.33E-17	3.62E-17	3.69E-17
4.24E-18	9.53E-18	1.49E-17	2.05E-17	1.18E-17	1.25E-17	1.33E-17	1.33E-17	2.53E-17	2.37E-17	2.24E-17	1.76E-17	2.43E-17	3.14E-17
4.98E-18	9.93E-18	1.17E-17	1.55E-17	7.05E-18	1.02E-17	9.23E-18	1.42E-17	2.71E-17	2.90E-17	2.14E-17	1.17E-17	1.77E-17	2.50E-17
4.03E-18	7.27E-18	9.75E-18	1.21E-17	7.24E-18	1.18E-17	1.36E-17	2.44E-17	3.35E-17	3.01E-17	2.55E-17	1.65E-17	1.85E-17	1.37E-17
4.47E-18	5.11E-18	9.88E-18	1.15E-17	1.16E-17	1.13E-17	1.46E-17	3.16E-17	3.61E-17	2.87E-17	2.40E-17	2.40E-17	1.95E-17	4.29E-18
4.97E-18	3.87E-18	7.40E-18	7.98E-18	1.63E-17	8.75E-18	1.05E-17	2.64E-17	3.61E-17	2.53E-17	2.19E-17	2.26E-17	1.83E-17	7.62E-18
3.18E-18	2.19E-18	5.60E-18	6.66E-18	1.22E-17	7.01E-18	1.12E-17	2.20E-17	2.98E-17	1.94E-17	1.61E-17	1.82E-17	1.66E-17	1.17E-17
6.79E-19	1.49E-18	6.14E-18	8.04E-18	8.75E-18	4.65E-18	1.29E-17	1.69E-17	2.04E-17	1.31E-17	1.18E-17	1.29E-17	1.49E-17	1.20E-17
2.96E-19	3.40E-19	5.01E-18	4.18E-18	3.18E-18	5.51E-18	9.05E-18	1.36E-17	1.74E-17	1.06E-17	7.89E-18	1.11E-17	1.18E-17	1.21E-17
2.10E-19	2.24E-19	2.30E-18	9.24E-19	2.58E-19	1.89E-18	4.14E-18	1.00E-17	1.36E-17	7.68E-18	6.92E-18	7.60E-18	9.43E-18	1.02E-17
1.11E-21	2.09E-20	3.82E-19	2.39E-19	8.68E-20	3.68E-20	3.79E-19	3.22E-18	9.86E-18	5.16E-18	6.23E-18	5.04E-18	7.10E-18	5.76E-18
2.70E-21	1.31E-19	9.24E-20	1.82E-18	5.94E-19	4.22E-19	2.21E-19	2.99E-19	3.75E-18	3.70E-18	3.66E-18	2.49E-18	4.01E-18	2.43E-18
1.11E-19	3.98E-20	1.02E-20	2.53E-18	5.37E-19	4.46E-19	5.99E-19	1.95E-19	2.24E-19	9.67E-19	1.38E-18	9.88E-19	1.74E-18	9.39E-19
2.04E-19	5.23E-23	2.76E-19	1.36E-18	3.16E-19	1.17E-19	1.17E-18	1.68E-18	1.07E-18	1.98E-20	1.58E-19	1.60E-19	6.44E-19	1.85E-19
7.33E-21	3.63E-19	1.01E-21	8.96E-19	1.44E-19	2.12E-20	7.32E-19	4.89E-18	1.04E-18	4.44E-19	1.37E-18	2.96E-19	8.79E-20	7.77E-21
2.93E-19	2.35E-18	8.10E-19	2.28E-18	6.76E-19	1.80E-21	1.39E-18	7.01E-19	7.17E-19	1.24E-18	4.73E-18	1.20E-18	1.78E-19	8.16E-20
1.32E-18	3.64E-18	2.36E-18	3.33E-18	8.79E-19	6.93E-19	2.24E-18	3.18E-19	9.25E-19	2.11E-18	3.90E-18	1.32E-18	9.92E-19	1.03E-18
2.26E-18	2.00E-18	5.27E-18	6.96E-18	2.63E-18	2.36E-18	2.76E-18	2.05E-18	2.24E-18	3.34E-18	3.25E-18	1.57E-18	3.18E-18	3.15E-18
4.28E-18	4.16E-18	9.12E-18	1.62E-17	5.06E-18	3.64E-18	5.11E-18	4.10E-18	4.57E-18	8.27E-18	4.93E-18	4.42E-18	8.07E-18	4.95E-18
8.62E-18	8.66E-18	1.25E-17	2.44E-17	8.28E-18	6.93E-18	1.10E-17	5.72E-18	7.70E-18	7.53E-18	8.10E-18	9.03E-18	1.38E-17	7.71E-18
1.30E-17	1.31E-17	1.53E-17	8.77E-18	1.16E-17	1.24E-17	1.48E-17	9.61E-18	1.44E-17	1.21E-17	1.35E-17	1.29E-17	1.83E-17	1.30E-17
1.70E-17	1.49E-17	2.02E-17	1.35E-17	1.94E-17	1.98E-17	2.20E-17	1.75E-17	2.85E-17	1.77E-17	1.83E-17	1.84E-17	2.56E-17	2.12E-17
2.65E-17	1.99E-17	2.72E-17	2.34E-17	3.09E-17	3.07E-17	2.73E-17	2.87E-17	3.64E-17	2.55E-17	2.61E-17	2.78E-17	3.55E-17	3.44E-17
3.26E-17	2.91E-17	3.73E-17	3.30E-17	4.27E-17	3.90E-17	3.25E-17	4.52E-17	5.26E-17	4.28E-17	4.00E-17	3.87E-17	4.40E-17	5.77E-17
4.48E-17	3.64E-17	4.30E-17	4.06E-17	5.21E-17	5.06E-17	4.07E-17	5.70E-17	6.79E-17	5.78E-17	5.10E-17	5.38E-17	6.39E-17	8.19E-17
6.07E-17	5.30E-17	5.77E-17	5.63E-17	6.09E-17	5.92E-17	5.23E-17	5.57E-17	7.93E-17	6.98E-17	5.88E-17	7.45E-17	9.37E-17	9.95E-17
7.20E-17	6.87E-17	7.77E-17	6.78E-17	7.14E-17	6.68E-17	6.07E-17	6.19E-17	8.34E-17	7.81E-17	7.91E-17	1.07E-16	1.19E-16	1.27E-16
8.27E-17	7.80E-17	9.62E-17	8.50E-17	8.50E-17	7.74E-17	7.79E-17	8.22E-17	1.01E-16	9.71E-17	1.06E-16	1.34E-16	1.39E-16	1.47E-16
1.01E-16	9.59E-17	1.03E-16	1.04E-16	1.07E-16	9.76E-17	1.06E-16	1.14E-16	1.21E-16	1.14E-16	1.35E-16	1.65E-16	1.70E-16	1.79E-16
1.23E-16	1.21E-16	1.24E-16	1.26E-16	1.33E-16	1.21E-16	1.24E-16	1.39E-16	1.46E-16	1.36E-16	1.56E-16	1.76E-16	1.85E-16	2.06E-16
1.35E-16	1.47E-16	1.53E-16	1.42E-16	1.71E-16	1.46E-16	1.44E-16	1.57E-16	1.76E-16	1.57E-16	1.80E-16	1.90E-16	2.03E-16	2.21E-16
1.60E-16	1.71E-16	1.68E-16	1.64E-16	2.12E-16	1.82E-16	1.72E-16	1.85E-16	2.00E-16	1.87E-16	1.96E-16	2.24E-16	2.26E-16	2.43E-16
1.73E-16	1.64E-16	1.84E-16	1.86E-16	2.20E-16	2.09E-16	2.13E-16	2.17E-16	2.17E-16	2.20E-16	2.40E-16	2.40E-16	2.62E-16	2.68E-16
1.96E-16	1.89E-16	2.11E-16	2.14E-16	2.45E-16	2.39E-16	2.49E-16	2.73E-16	2.50E-16	2.58E-16	2.93E-16	2.76E-16	3.09E-16	3.04E-16
2.35E-16	2.29E-16	2.42E-16	2.50E-16	2.82E-16	2.85E-16	2.94E-16	3.25E-16	2.81E-16	3.07E-16	3.27E-16	3.17E-16	3.68E-16	3.51E-16
2.85E-16	2.74E-16	2.76E-16	2.73E-16	3.17E-16	3.29E-16	3.37E-16	3.71E-16	3.22E-16	3.66E-16	3.61E-16	3.84E-16	4.15E-16	3.84E-16
3.44E-16	3.34E-16	3.20E-16	3.12E-16	3.48E-16	3.62E-16	3.49E-16	3.55E-16	3.83E-16	4.32E-16	4.07E-16	4.47E-16	4.57E-16	4.24E-16

3.96E-16	3.91E-16	3.67E-16	3.67E-16	4.02E-16	4.11E-16	3.73E-16	3.98E-16	4.15E-16	4.66E-16	4.46E-16	4.76E-16	4.83E-16	4.67E-16
4.37E-16	4.26E-16	4.12E-16	4.33E-16	4.64E-16	4.63E-16	4.27E-16	4.55E-16	4.41E-16	4.80E-16	4.94E-16	4.81E-16	5.09E-16	5.24E-16
4.69E-16	4.83E-16	4.71E-16	5.18E-16	5.11E-16	5.21E-16	4.78E-16	5.20E-16	4.92E-16	5.18E-16	5.60E-16	5.36E-16	5.81E-16	5.94E-16
5.15E-16	5.50E-16	5.47E-16	5.79E-16	5.58E-16	5.77E-16	5.43E-16	5.49E-16	5.41E-16	6.00E-16	6.40E-16	6.26E-16	6.41E-16	6.73E-16
5.54E-16	6.04E-16	6.02E-16	6.15E-16	6.03E-16	6.47E-16	5.93E-16	5.84E-16	5.84E-16	6.72E-16	7.16E-16	7.07E-16	7.08E-16	7.49E-16
6.01E-16	6.59E-16	6.83E-16	6.98E-16	6.65E-16	7.11E-16	6.63E-16	6.49E-16	6.75E-16	7.54E-16	7.83E-16	7.84E-16	7.99E-16	8.08E-16
6.90E-16	7.26E-16	7.65E-16	8.00E-16	7.43E-16	7.84E-16	7.48E-16	7.21E-16	7.66E-16	8.32E-16	8.70E-16	8.69E-16	8.85E-16	8.84E-16
7.92E-16	8.12E-16	8.51E-16	8.79E-16	8.28E-16	8.51E-16	8.29E-16	7.94E-16	8.40E-16	9.02E-16	9.45E-16	9.38E-16	9.37E-16	9.43E-16
8.75E-16	9.17E-16	9.72E-16	9.45E-16	8.90E-16	8.96E-16	8.96E-16	8.72E-16	9.18E-16	9.72E-16	1.02E-15	1.02E-15	1.06E-15	1.04E-15
1.01E-15	1.07E-15	1.03E-15	1.01E-15	9.56E-16	9.68E-16	9.83E-16	9.66E-16	9.85E-16	1.04E-15	1.08E-15	1.10E-15	1.18E-15	1.17E-15
1.12E-15	1.16E-15	1.06E-15	1.07E-15	1.05E-15	1.11E-15	1.11E-15	1.06E-15	1.06E-15	1.12E-15	1.15E-15	1.19E-15	1.31E-15	1.31E-15
1.36E-14	1.40E-14	1.42E-14	1.46E-14	1.48E-14	1.51E-14	1.48E-14	1.49E-14	1.52E-14	1.61E-14	1.70E-14	1.74E-14	1.85E-14	1.89E-14

-4.81E-09	-5.55E-09	-5.55E-09	-5.57E-09	-6.73E-09	-6.63E-09	-6.22E-09	-6.54E-09	-6.38E-09	-6.86E-09	-7.44E-09	-6.84E-09	-7.40E-09	-6.75E-09
-4.35E-09	-4.68E-09	-5.07E-09	-5.36E-09	-6.89E-09	-6.75E-09	-6.41E-09	-6.30E-09	-6.69E-09	-7.28E-09	-7.68E-09	-7.39E-09	-7.96E-09	-7.37E-09
-4.10E-09	-4.31E-09	-4.93E-09	-5.43E-09	-7.05E-09	-7.23E-09	-7.29E-09	-5.89E-09	-7.08E-09	-7.73E-09	-7.56E-09	-8.08E-09	-8.71E-09	-8.56E-09
-5.07E-09	-4.58E-09	-4.96E-09	-5.97E-09	-7.18E-09	-7.94E-09	-7.99E-09	-6.50E-09	-7.54E-09	-7.61E-09	-7.19E-09	-8.32E-09	-9.00E-09	-9.17E-09
-6.26E-09	-4.75E-09	-5.55E-09	-6.20E-09	-7.46E-09	-8.05E-09	-8.46E-09	-7.89E-09	-7.89E-09	-7.58E-09	-7.59E-09	-8.28E-09	-9.03E-09	-9.70E-09
-6.97E-09	-5.40E-09	-6.00E-09	-6.74E-09	-7.63E-09	-8.03E-09	-8.45E-09	-8.00E-09	-7.97E-09	-8.37E-09	-8.49E-09	-8.51E-09	-9.26E-09	-1.03E-08
-7.54E-09	-5.92E-09	-6.61E-09	-7.30E-09	-7.99E-09	-7.56E-09	-8.49E-09	-8.41E-09	-8.25E-09	-8.96E-09	-8.90E-09	-8.93E-09	-9.51E-09	-1.04E-08
-7.44E-09	-6.43E-09	-7.11E-09	-7.58E-09	-8.10E-09	-7.26E-09	-8.13E-09	-8.54E-09	-8.67E-09	-9.52E-09	-9.07E-09	-9.36E-09	-9.56E-09	-1.02E-08
-7.40E-09	-6.85E-09	-7.51E-09	-7.70E-09	-8.19E-09	-7.63E-09	-8.10E-09	-8.67E-09	-8.86E-09	-9.46E-09	-9.56E-09	-9.75E-09	-9.78E-09	-9.82E-09
-7.44E-09	-6.90E-09	-7.72E-09	-8.09E-09	-8.47E-09	-8.26E-09	-8.34E-09	-8.62E-09	-8.74E-09	-9.45E-09	-9.54E-09	-9.40E-09	-1.00E-08	-1.01E-08
-7.24E-09	-6.98E-09	-7.62E-09	-8.07E-09	-8.81E-09	-8.51E-09	-8.69E-09	-8.75E-09	-8.80E-09	-9.86E-09	-9.38E-09	-9.44E-09	-1.02E-08	-1.07E-08
-7.40E-09	-7.73E-09	-7.51E-09	-8.11E-09	-8.76E-09	-8.77E-09	-8.73E-09	-8.88E-09	-9.24E-09	-1.02E-08	-9.53E-09	-9.90E-09	-1.03E-08	-1.12E-08
-7.96E-09	-8.25E-09	-8.35E-09	-8.38E-09	-8.68E-09	-8.87E-09	-9.45E-09	-9.31E-09	-9.63E-09	-1.01E-08	-9.92E-09	-9.87E-09	-1.03E-08	-1.17E-08
-8.99E-09	-8.44E-09	-8.40E-09	-9.06E-09	-8.72E-09	-9.36E-09	-1.00E-08	-9.88E-09	-9.75E-09	-9.93E-09	-1.00E-08	-1.02E-08	-1.06E-08	-1.19E-08
-8.97E-09	-8.00E-09	-8.50E-09	-9.71E-09	-8.74E-09	-9.55E-09	-1.03E-08	-1.06E-08	-9.87E-09	-9.97E-09	-1.01E-08	-1.09E-08	-1.07E-08	-1.18E-08
-8.75E-09	-8.27E-09	-9.16E-09	-9.65E-09	-9.16E-09	-1.01E-08	-1.05E-08	-1.08E-08	-1.02E-08	-9.75E-09	-1.01E-08	-1.14E-08	-1.09E-08	-1.20E-08
-8.97E-09	-8.43E-09	-9.82E-09	-9.86E-09	-1.04E-08	-1.07E-08	-1.07E-08	-1.09E-08	-1.05E-08	-1.01E-08	-1.07E-08	-1.20E-08	-1.15E-08	-1.22E-08
-9.16E-09	-8.73E-09	-1.00E-08	-9.95E-09	-1.03E-08	-1.15E-08	-1.09E-08	-1.10E-08	-1.07E-08	-1.03E-08	-1.07E-08	-1.20E-08	-1.15E-08	-1.25E-08
-9.71E-09	-9.19E-09	-1.00E-08	-9.98E-09	-1.00E-08	-1.18E-08	-1.09E-08	-1.11E-08	-1.10E-08	-1.10E-08	-1.08E-08	-1.24E-08	-1.22E-08	-1.30E-08
-9.89E-09	-9.69E-09	-9.98E-09	-9.89E-09	-1.00E-08	-1.15E-08	-1.11E-08	-1.12E-08	-1.14E-08	-1.12E-08	-1.14E-08	-1.28E-08	-1.31E-08	-1.32E-08
-9.86E-09	-1.01E-08	-9.79E-09	-1.03E-08	-1.03E-08	-1.11E-08	-1.10E-08	-1.13E-08	-1.16E-08	-1.12E-08	-1.18E-08	-1.29E-08	-1.31E-08	-1.33E-08
-9.86E-09	-1.01E-08	-9.97E-09	-1.14E-08	-1.06E-08	-1.10E-08	-1.09E-08	-1.15E-08	-1.15E-08	-1.13E-08	-1.21E-08	-1.27E-08	-1.30E-08	-1.34E-08
-9.78E-09	-1.00E-08	-9.64E-09	-1.12E-08	-1.08E-08	-1.10E-08	-1.07E-08	-1.17E-08	-1.16E-08	-1.18E-08	-1.24E-08	-1.29E-08	-1.34E-08	-1.38E-08
-9.33E-09	-9.18E-09	-9.94E-09	-1.13E-08	-1.09E-08	-1.11E-08	-1.12E-08	-1.19E-08	-1.21E-08	-1.23E-08	-1.28E-08	-1.35E-08	-1.37E-08	-1.40E-08
-9.47E-09	-9.16E-09	-1.03E-08	-1.17E-08	-1.06E-08	-1.15E-08	-1.17E-08	-1.21E-08	-1.26E-08	-1.26E-08	-1.29E-08	-1.34E-08	-1.37E-08	-1.41E-08
-9.28E-09	-9.77E-09	-1.03E-08	-1.20E-08	-9.59E-09	-1.21E-08	-1.20E-08	-1.17E-08	-1.29E-08	-1.27E-08	-1.31E-08	-1.33E-08	-1.37E-08	-1.44E-08
-9.52E-09	-1.00E-08	-9.80E-09	-1.18E-08	-1.03E-08	-1.21E-08	-1.16E-08	-1.15E-08	-1.31E-08	-1.31E-08	-1.31E-08	-1.31E-08	-1.39E-08	-1.44E-08
-9.29E-09	-1.05E-08	-1.01E-08	-1.19E-08	-1.25E-08	-1.23E-08	-1.15E-08	-1.15E-08	-1.33E-08	-1.31E-08	-1.30E-08	-1.32E-08	-1.39E-08	-1.46E-08
-9.71E-09	-1.12E-08	-1.05E-08	-1.19E-08	-1.25E-08	-1.23E-08	-1.19E-08	-1.18E-08	-1.37E-08	-1.32E-08	-1.27E-08	-1.29E-08	-1.35E-08	-1.41E-08
-1.00E-08	-1.12E-08	-1.10E-08	-1.20E-08	-1.22E-08	-1.20E-08	-1.25E-08	-1.29E-08	-1.40E-08	-1.31E-08	-1.25E-08	-1.30E-08	-1.36E-08	-1.41E-08
-1.08E-08	-1.15E-08	-1.13E-08	-1.16E-08	-1.14E-08	-1.22E-08	-1.29E-08	-1.30E-08	-1.31E-08	-1.28E-08	-1.30E-08	-1.35E-08	-1.41E-08	-1.48E-08
-1.08E-08	-1.19E-08	-1.13E-08	-1.13E-08	-1.10E-08	-1.21E-08	-1.27E-08	-1.28E-08	-1.24E-08	-1.29E-08	-1.35E-08	-1.33E-08	-1.40E-08	-1.53E-08
-1.10E-08	-1.18E-08	-1.16E-08	-1.14E-08	-1.11E-08	-1.24E-08	-1.22E-08	-1.23E-08	-1.27E-08	-1.34E-08	-1.42E-08	-1.35E-08	-1.40E-08	-1.54E-08
-1.09E-08	-1.19E-08	-1.19E-08	-1.18E-08	-1.21E-08	-1.23E-08	-1.18E-08	-1.21E-08	-1.27E-08	-1.35E-08	-1.41E-08	-1.40E-08	-1.45E-08	-1.51E-08
-1.10E-08	-1.19E-08	-1.14E-08	-1.14E-08	-1.25E-08	-1.26E-08	-1.25E-08	-1.29E-08	-1.34E-08	-1.35E-08	-1.43E-08	-1.38E-08	-1.41E-08	-1.48E-08
-1.11E-08	-1.22E-08	-1.16E-08	-1.17E-08	-1.27E-08	-1.27E-08	-1.28E-08	-1.29E-08	-1.35E-08	-1.40E-08	-1.48E-08	-1.44E-08	-1.48E-08	-1.50E-08
-1.15E-08	-1.22E-08	-1.19E-08	-1.16E-08	-1.28E-08	-1.29E-08	-1.31E-08	-1.29E-08	-1.37E-08	-1.42E-08	-1.46E-08	-1.41E-08	-1.53E-08	-1.57E-08
-1.13E-08	-1.16E-08	-1.12E-08	-1.15E-08	-1.25E-08	-1.30E-08	-1.37E-08	-1.29E-08	-1.35E-08	-1.42E-08	-1.45E-08	-1.39E-08	-1.56E-08	-1.57E-08
-1.11E-08	-1.14E-08	-1.05E-08	-1.10E-08	-1.21E-08	-1.29E-08	-1.38E-08	-1.32E-08	-1.33E-08	-1.42E-08	-1.45E-08	-1.39E-08	-1.57E-08	-1.56E-08
-1.09E-08	-1.14E-08	-1.04E-08	-1.13E-08	-1.18E-08	-1.32E-08	-1.35E-08	-1.34E-08	-1.32E-08	-1.43E-08	-1.46E-08	-1.44E-08	-1.59E-08	-1.59E-08
-1.09E-08	-1.16E-08	-1.11E-08	-1.18E-08	-1.20E-08	-1.31E-08	-1.31E-08	-1.34E-08	-1.35E-08	-1.42E-08	-1.45E-08	-1.42E-08	-1.57E-08	-1.60E-08
-1.10E-08	-1.16E-08	-1.16E-08	-1.22E-08	-1.22E-08	-1.28E-08	-1.24E-08	-1.33E-08	-1.35E-08	-1.41E-08	-1.43E-08	-1.44E-08	-1.54E-08	-1.56E-08
-1.14E-08	-1.13E-08	-1.16E-08	-1.25E-08	-1.22E-08	-1.20E-08	-1.11E-08	-1.25E-08	-1.31E-08	-1.41E-08	-1.41E-08	-1.47E-08	-1.53E-08	-1.49E-08
-1.11E-08	-1.09E-08	-1.13E-08	-1.20E-08	-1.20E-08	-1.16E-08	-1.02E-08	-1.24E-08	-1.26E-08	-1.38E-08	-1.37E-08	-1.46E-08	-1.51E-08	-1.45E-08
-1.07E-08	-1.07E-08	-1.12E-08	-1.17E-08	-1.16E-08	-1.17E-08	-1.04E-08	-1.28E-08	-1.29E-08	-1.33E-08	-1.31E-08	-1.46E-08	-1.48E-08	-1.48E-08
-1.08E-08	-1.00E-08	-1.01E-08	-1.11E-08	-1.14E-08	-1.22E-08	-1.20E-08	-1.31E-08	-1.29E-08	-1.33E-08	-1.28E-08	-1.45E-08	-1.47E-08	-1.51E-08
-1.01E-08	-1.02E-08	-1.03E-08	-1.11E-08	-1.15E-08	-1.19E-08	-1.26E-08	-1.29E-08	-1.26E-08	-1.37E-08	-1.38E-08	-1.45E-08	-1.45E-08	-1.50E-08
-9.29E-09	-1.02E-08	-1.05E-08	-1.05E-08	-1.09E-08	-1.15E-08	-1.28E-08	-1.29E-08	-1.26E-08	-1.38E-08	-1.41E-08	-1.41E-08	-1.38E-08	-1.48E-08
-8.44E-09	-9.41E-09	-9.89E-09	-9.45E-09	-9.65E-09	-1.10E-08	-1.22E-08	-1.29E-08	-1.29E-08	-1.40E-08	-1.38E-08	-1.44E-08	-1.39E-08	-1.47E-08
-7.35E-09	-8.92E-09	-9.10E-09	-7.96E-09	-8.65E-09	-1.02E-08	-1.19E-08	-1.33E-08	-1.27E-08	-1.42E-08	-1.35E-08	-1.43E-08	-1.39E-08	-1.46E-08
-7.17E-09	-8.92E-09	-8.56E-09	-7.37E-09	-8.98E-09	-1.03E-08	-1.23E-08	-1.34E-08	-1.27E-08	-1.39E-08	-1.34E-08	-1.42E-08	-1.43E-08	-1.44E-08
-7.73E-09	-9.50E-09	-8.91E-09	-8.10E-09	-1.03E-08	-1.13E-08	-1.27E-08	-1.33E-08	-1.27E-08	-1.35E-08	-1.34E-08	-1.42E-08	-1.47E-08	-1.44E-08
-9.24E-09	-1.01E-08	-1.01E-08	-9.61E-09	-1.10E-08	-1.21E-08	-1.26E-08	-1.28E-08	-1.21E-08	-1.27E-08	-1.25E-08	-1.40E-08	-1.52E-08	-1.50E-08
-9.74E-09	-1.05E-08	-1.04E-08	-9.82E-09	-1.02E-08	-1.22E-08	-1.21E-08	-1.23E-08	-1.17E-08	-1.23E-08	-1.13E-08	-1.40E-08	-1.49E-08	-1.48E-08
-9.65E-09	-1.03E-08	-1.00E-08	-9.64E-09	-1.01E-08	-1.18E-08	-1.15E-08	-1.19E-08	-1.11E-08	-1.17E-08	-1.10E-08	-1.40E-08	-1.45E-08	-1.44E-08
-9.77E-09	-1.02E-08	-9.71E-09	-9.76E-09	-1.05E-08	-1.15E-08	-1.11E-08	-1.16E-08	-1.11E-08	-1.14E-08	-1.23E-08	-1.37E-08	-1.40E-08	-1.38E-08
-9.97E-09	-1.03E-08	-1.02E-08	-1.07E-08	-1.11E-08	-1.16E-08	-1.08E-08	-1.11E-08	-1.09E-08	-1.10E-08	-1.19E-08	-1.33E-08	-1.31E-08	-1.31E-08
-1.02E-08	-1.06E-08	-1.08E-08	-1.11E-08	-1.08E-08	-1.11E-08	-1.05E-08	-1.08E-08	-1.10E-08	-1.07E-08	-1.16E-08	-1.27E-08	-1.22E-08	-1.24E-08
-1.00E-08	-1.04E-08	-1.07E-08	-1.11E-08	-1.03E-08	-1.06E-08	-1.07E-08	-1.06E-08	-1.11E-08	-1.13E-08	-1.16E-08	-1.24E-08	-1.12E-08	-1.12E-08
-1.00E-08	-1.01E-08	-1.07E-08	-1.08E-08	-1.05E-08	-1.05E-08	-1.02E-08	-9.96E-09	-1.10E-08	-1.18E-08	-1.14E-08	-1.13E-08	-9.57E-09	-9.61E-09
-1.02E-08	-9.72E-09	-1.07E-08	-1.07E-08	-1.07E-08	-1.07E-08	-9.95E-09	-9.65E-09	-1.07E-08	-1.17E-08	-1.12E-08	-1.02E-08	-7.82E-09	-8.61E-09
-1.01E-08	-9.43E-09	-1.00E-08	-1.01E-08	-1.00E-08	-1.06E-08	-1.01E-08	-9.35E-09	-1.03E-08	-1.10E-08	-1.11E-08	-9.42E-09	-7.51E-09	-8.64E-09
-9.77E-09	-8.97E-09	-9.38E-09	-9.98E-09	-1.00E-08	-1.07E-08	-9.71E-09	-9.38E-09	-9.84E-09	-1.01E-08	-1.09E-08	-8.58E-09	-7.88E-09	-8.06E-09
-9.89E-09	-9.14E-09	-9.36E-09	-1.01E-08	-1.02E-08	-1.02E-08	-9.84E-09	-9.71E-09	-9.56E-09	-9.61E-09	-9.85E-09	-7.59E-09	-6.50E-09	-6.20E-09
-9.92E-09	-9.38E-09	-9.65E-09	-1.03E-08	-9.85E-09	-9.77E-09	-1.00E-08	-1.00E-08	-9.65E-09	-9.37E-09	-9.16E-09	-6.71E-09	-4.52E-09	-3.29E-09
-9.72E-09	-9.72E-09	-1.02E-08	-1.04E-08	-9.26E-09	-9.73E-09	-9.86E-09	-9.80E-09	-9.93E-09	-9.49E-09	-9.13E-09	-6.42E-09	-3.29E-09	-1.57E-09
-9.48E-09	-9.69E-09	-1.04E-08	-1.02E-08	-9.13E-09	-9.67E-09	-9.53E-09	-9.19E-09	-1.00E-08	-9.40E-09	-9.17E-09	-6.84E-09	-4.02E-09	-2.34E-09
-9.06E-09	-9.32E-09	-1.02E-08	-9.74E-09	-8.86E-09	-9.66E-09	-9.47E-09	-8.83E-09	-9.80E-09	-9.04E-09	-9.15E-09	-6.76E-09	-4.70E-09	-3.76E-09
-8.48E-09	-9.23E-09	-9.71E-09	-9.73E-09	-8.64E-09	-9.06E-09	-9.25E-09	-8.84E-09	-9.43E-09	-8.58E-09	-9.11E-09	-6.70E-09	-5.39E-09	-4.49E-09
-7.89E-09	-8.98E-09	-8.94E-09	-9.64E-09	-8.34E-09	-8.21E-09	-8.79E-09	-8.72E-09	-9.30E-09	-8.66E-09	-8.79E-09	-6.99E-09	-6.29E-09	-5.72E-09
-7.56E-09	-8.37E-09	-8.57E-09	-8.97E-09	-8.03E-09	-7.29E-09	-8.19E-09	-8.56E-09	-8.77E-09	-8.02E-09	-8.41E-09	-7.54E-09	-7.51E-09	-6.91E-09
-7.04E-09	-8.43E-09	-8.26E-09	-8.62E-09	-7.59E-09	-6.87E-09	-7.73E-09	-8.65E-09	-8.57E-09	-7.50E-09	-8.12E-09	-7.70E-09	-7.89E-09	-7.15E-09
-7.31E-09	-7.87E-09	-7.42E-09	-8.46E-09	-7.76E-09	-7.12E-09	-7.82E-09	-8.74E-09	-8.06E-09	-6.83E-09	-7.37E-09	-7.59E-09	-7.46E-09	-6.90E-09
-7.25E-09	-6.82E-09	-6.39E-09	-7.94E-09	-8.39E-09	-7.90E-09	-8.41E-09	-8.53E-09	-7.56E-09	-6.19E-09	-6.39E-09	-6.94E-09	-6.40E-09	-7.02E-09
-6.90E-09	-5.78E-09	-5.42E-09	-7.17E-09	-8.75E-09	-8.70E-09	-8.61E-09	-8.52E-09	-7.42E-09	-6.11E-09	-4.94E-09	-5.72E-09	-5.04E-09	-6.87E-09
-6.10E-09	-5.37E-09	-5.27E-09	-7.06E-09	-8.48E-09	-8.81E-09	-8.48E-09	-8.14E-09	-7.13E-09	-5.86E-09	-3.95E-09	-4.68E-09	-4.20E-09	-6.42E-09
-6.38E-09	-6.23E-09	-5.92E-09	-7.27E-09	-7.97E-09	-8.36E-09	-8.05E-09	-7.65E-09	-6.87E-09	-5.51E-09	-4.67E-09	-4.53E-09	-4.98E-09	-6.26E-09
-6.60E-09	-6.89E-09	-6.27E-09	-7.20E-09	-7.42E-09	-7.76E-09	-7.58E-09	-7.00E-09	-6.62E-09	-6.08E-09	-5.93E-09	-5.80E-09	-5.55E-09	-6.03E-09

-6.33E-09	-6.97E-09	-6.64E-09	-7.11E-09	-7.05E-09	-7.54E-09	-7.30E-09	-6.83E-09	-6.40E-09	-6.37E-09	-6.47E-09	-6.10E-09	-6.04E-09	-5.90E-09
-6.31E-09	-7.45E-09	-6.92E-09	-6.98E-09	-6.31E-09	-7.32E-09	-6.77E-09	-6.81E-09	-5.99E-09	-6.13E-09	-6.05E-09	-6.45E-09	-6.45E-09	-6.13E-09
-6.15E-09	-7.43E-09	-6.56E-09	-6.57E-09	-5.83E-09	-6.81E-09	-6.24E-09	-6.31E-09	-4.63E-09	-5.59E-09	-5.99E-09	-6.53E-09	-6.55E-09	-6.42E-09
-5.41E-09	-6.68E-09	-6.20E-09	-6.23E-09	-6.42E-09	-6.17E-09	-5.39E-09	-5.27E-09	-4.38E-09	-5.39E-09	-6.42E-09	-6.36E-09	-6.68E-09	-6.37E-09
-5.53E-09	-5.53E-09	-5.61E-09	-5.79E-09	-6.45E-09	-5.74E-09	-4.34E-09	-4.24E-09	-5.21E-09	-5.41E-09	-6.36E-09	-6.34E-09	-6.64E-09	-6.26E-09
-5.05E-09	-4.15E-09	-4.87E-09	-5.31E-09	-6.14E-09	-4.91E-09	-3.81E-09	-3.75E-09	-4.70E-09	-4.85E-09	-5.72E-09	-6.30E-09	-6.44E-09	-5.89E-09
-3.59E-09	-2.54E-09	-4.01E-09	-4.81E-09	-5.85E-09	-4.87E-09	-3.52E-09	-3.45E-09	-4.40E-09	-4.14E-09	-4.80E-09	-5.63E-09	-6.06E-09	-5.22E-09
-2.93E-09	-2.01E-09	-3.51E-09	-4.40E-09	-5.17E-09	-4.42E-09	-2.77E-09	-3.15E-09	-3.84E-09	-3.53E-09	-4.30E-09	-4.86E-09	-5.18E-09	-4.96E-09
-2.76E-09	-2.49E-09	-3.25E-09	-3.93E-09	-3.89E-09	-3.69E-09	-2.33E-09	-1.93E-09	-3.17E-09	-3.47E-09	-4.09E-09	-4.19E-09	-4.36E-09	-4.68E-09
-3.55E-09	-2.90E-09	-3.59E-09	-3.50E-09	-3.57E-09	-3.16E-09	-1.96E-09	-1.53E-09	-2.31E-09	-3.54E-09	-3.43E-09	-3.74E-09	-3.80E-09	-4.05E-09
-3.35E-09	-2.52E-09	-3.09E-09	-3.08E-09	-2.63E-09	-2.75E-09	-1.75E-09	-1.06E-09	-1.99E-09	-3.39E-09	-3.12E-09	-3.40E-09	-3.54E-09	-3.23E-09
-3.18E-09	-2.01E-09	-2.40E-09	-2.83E-09	-2.18E-09	-2.26E-09	-1.73E-09	-7.87E-10	-1.57E-09	-2.93E-09	-2.91E-09	-3.27E-09	-2.82E-09	-2.34E-09
-2.66E-09	-1.52E-09	-2.27E-09	-2.07E-09	-1.90E-09	-1.90E-09	-1.67E-09	-3.05E-10	-1.12E-09	-2.53E-09	-2.50E-09	-2.57E-09	-1.73E-09	-1.54E-09
-2.30E-09	-1.03E-09	-1.75E-09	-1.34E-09	-1.30E-09	-1.63E-09	-1.49E-09	-2.80E-10	-1.44E-10	-1.97E-09	-1.95E-09	-1.76E-09	-1.53E-09	-1.14E-09
-1.56E-09	-5.82E-10	-6.90E-10	-6.29E-10	-8.77E-10	-9.09E-10	-1.06E-09	-6.70E-10	1.85E-10	-1.32E-09	-1.42E-09	-9.60E-10	-6.54E-10	-5.54E-10
-1.18E-09	-1.82E-10	-2.63E-10	8.11E-11	-4.37E-11	-2.88E-10	-1.27E-10	-1.65E-11	1.51E-10	-5.48E-10	-5.54E-10	-3.00E-10	3.86E-10	-2.04E-10
1.41E-11	6.23E-10	2.01E-10	3.55E-10	5.28E-10	1.61E-10	1.19E-09	1.17E-09	5.88E-10	1.27E-10	2.10E-10	-1.55E-11	6.18E-10	4.00E-10
1.16E-09	1.21E-09	1.10E-09	6.88E-10	9.84E-10	1.03E-09	1.92E-09	2.21E-09	1.23E-09	9.57E-10	1.05E-09	9.29E-10	1.00E-09	7.58E-10
1.93E-09	1.47E-09	1.53E-09	1.24E-09	1.49E-09	2.00E-09	2.57E-09	2.36E-09	1.66E-09	1.68E-09	1.86E-09	2.26E-09	1.84E-09	1.50E-09
2.06E-09	1.97E-09	2.20E-09	2.32E-09	2.42E-09	2.88E-09	3.91E-09	3.46E-09	2.47E-09	3.07E-09	2.81E-09	3.46E-09	2.87E-09	2.77E-09
2.75E-09	2.90E-09	2.78E-09	3.31E-09	3.55E-09	4.68E-09	4.78E-09	4.74E-09	3.71E-09	4.57E-09	3.83E-09	4.30E-09	3.94E-09	4.07E-09
3.44E-09	4.18E-09	3.68E-09	4.26E-09	4.77E-09	6.44E-09	5.47E-09	5.69E-09	5.06E-09	6.00E-09	4.89E-09	4.99E-09	5.11E-09	5.42E-09
4.11E-09	5.14E-09	4.63E-09	5.17E-09	5.57E-09	7.53E-09	6.10E-09	5.60E-09	5.60E-09	6.56E-09	6.02E-09	5.71E-09	6.17E-09	6.50E-09
5.24E-09	6.28E-09	6.18E-09	6.52E-09	6.58E-09	8.14E-09	6.60E-09	6.23E-09	5.93E-09	7.24E-09	7.46E-09	6.57E-09	6.89E-09	7.52E-09
6.42E-09	7.46E-09	7.31E-09	7.22E-09	8.14E-09	7.57E-09	6.90E-09	7.25E-09	7.06E-09	8.02E-09	9.38E-09	7.49E-09	8.06E-09	8.84E-09
7.92E-09	8.74E-09	8.32E-09	7.79E-09	8.70E-09	7.38E-09	7.38E-09	8.13E-09	8.39E-09	9.03E-09	1.01E-08	8.43E-09	9.04E-09	9.54E-09
9.39E-09	9.11E-09	9.04E-09	8.24E-09	9.27E-09	8.32E-09	8.84E-09	9.01E-09	9.43E-09	1.00E-08	1.07E-08	9.48E-09	9.82E-09	1.03E-08
1.05E-08	9.39E-09	9.24E-09	9.27E-09	1.02E-08	9.97E-09	1.03E-08	9.94E-09	1.03E-08	1.06E-08	1.14E-08	1.04E-08	1.10E-08	1.14E-08
1.18E-08	1.00E-08	9.72E-09	1.02E-08	1.09E-08	1.20E-08	1.16E-08	1.10E-08	1.10E-08	1.15E-08	1.22E-08	1.17E-08	1.17E-08	1.24E-08
1.24E-08	1.14E-08	1.13E-08	1.19E-08	1.23E-08	1.34E-08	1.29E-08	1.23E-08	1.17E-08	1.30E-08	1.32E-08	1.29E-08	1.28E-08	1.33E-08
1.30E-08	1.29E-08	1.34E-08	1.42E-08	1.36E-08	1.47E-08	1.42E-08	1.37E-08	1.29E-08	1.41E-08	1.43E-08	1.43E-08	1.35E-08	1.42E-08
1.40E-08	1.38E-08	1.56E-08	1.63E-08	1.47E-08	1.51E-08	1.51E-08	1.55E-08	1.47E-08	1.55E-08	1.53E-08	1.54E-08	1.45E-08	1.53E-08
1.52E-08	1.51E-08	1.66E-08	1.73E-08	1.52E-08	1.56E-08	1.57E-08	1.64E-08	1.63E-08	1.75E-08	1.64E-08	1.63E-08	1.57E-08	1.64E-08
1.61E-08	1.62E-08	1.65E-08	1.84E-08	1.62E-08	1.65E-08	1.67E-08	1.72E-08	1.81E-08	1.91E-08	1.81E-08	1.74E-08	1.69E-08	1.75E-08
1.78E-08	1.71E-08	1.67E-08	1.90E-08	1.72E-08	1.78E-08	1.83E-08	1.86E-08	1.98E-08	2.07E-08	1.98E-08	1.88E-08	1.79E-08	1.89E-08
1.87E-08	1.78E-08	1.74E-08	1.87E-08	1.80E-08	1.92E-08	2.00E-08	1.99E-08	2.11E-08	2.13E-08	2.08E-08	2.01E-08	1.93E-08	2.03E-08
1.95E-08	1.87E-08	1.91E-08	1.93E-08	1.93E-08	2.11E-08	2.10E-08	2.07E-08	2.20E-08	2.19E-08	2.19E-08	2.17E-08	2.06E-08	2.19E-08
1.99E-08	1.94E-08	2.06E-08	2.08E-08	2.08E-08	2.31E-08	2.21E-08	2.20E-08	2.29E-08	2.31E-08	2.28E-08	2.26E-08	2.19E-08	2.33E-08
2.10E-08	2.07E-08	2.17E-08	2.22E-08	2.25E-08	2.44E-08	2.30E-08	2.33E-08	2.42E-08	2.40E-08	2.37E-08	2.39E-08	2.36E-08	2.49E-08
2.26E-08	2.21E-08	2.30E-08	2.34E-08	2.38E-08	2.53E-08	2.42E-08	2.44E-08	2.50E-08	2.52E-08	2.46E-08	2.53E-08	2.51E-08	2.61E-08
2.39E-08	2.36E-08	2.46E-08	2.48E-08	2.52E-08	2.59E-08	2.51E-08	2.54E-08	2.57E-08	2.58E-08	2.60E-08	2.64E-08	2.63E-08	2.70E-08
2.48E-08	2.50E-08	2.59E-08	2.66E-08	2.67E-08	2.70E-08	2.64E-08	2.68E-08	2.74E-08	2.68E-08	2.75E-08	2.80E-08	2.80E-08	2.80E-08
2.58E-08	2.66E-08	2.70E-08	2.77E-08	2.85E-08	2.89E-08	2.78E-08	2.84E-08	2.95E-08	2.83E-08	2.95E-08	2.95E-08	3.00E-08	2.96E-08
2.70E-08	2.79E-08	2.82E-08	2.90E-08	3.03E-08	3.01E-08	2.92E-08	2.96E-08	3.10E-08	2.97E-08	3.08E-08	3.06E-08	3.15E-08	3.15E-08
2.83E-08	2.91E-08	2.92E-08	3.10E-08	3.16E-08	3.08E-08	3.06E-08	3.12E-08	3.21E-08	3.10E-08	3.19E-08	3.23E-08	3.34E-08	3.34E-08
2.95E-08	3.05E-08	3.07E-08	3.23E-08	3.24E-08	3.20E-08	3.25E-08	3.32E-08	3.30E-08	3.29E-08	3.36E-08	3.38E-08	3.47E-08	3.47E-08
3.08E-08	3.18E-08	3.21E-08	3.35E-08	3.35E-08	3.33E-08	3.45E-08	3.44E-08	3.44E-08	3.46E-08	3.53E-08	3.55E-08	3.60E-08	3.61E-08
3.22E-08	3.30E-08	3.35E-08	3.48E-08	3.54E-08	3.50E-08	3.64E-08	3.61E-08	3.57E-08	3.63E-08	3.67E-08	3.72E-08	3.72E-08	3.72E-08
3.37E-08	3.45E-08	3.50E-08	3.61E-08	3.68E-08	3.70E-08	3.76E-08	3.76E-08	3.75E-08	3.82E-08	3.84E-08	3.92E-08	3.90E-08	3.88E-08
3.61E-08	3.66E-08	3.67E-08	3.74E-08	3.79E-08	3.87E-08	3.86E-08	3.90E-08	3.91E-08	3.99E-08	3.99E-08	4.07E-08	4.04E-08	4.07E-08

2.31E-17	3.08E-17	3.08E-17	3.10E-17	4.53E-17	4.39E-17	3.87E-17	4.28E-17	4.07E-17	4.70E-17	5.53E-17	4.67E-17	5.48E-17	4.56E-17
1.89E-17	2.19E-17	2.57E-17	2.87E-17	4.74E-17	4.55E-17	4.11E-17	3.96E-17	4.48E-17	5.30E-17	5.90E-17	5.46E-17	6.33E-17	5.43E-17
1.68E-17	1.85E-17	2.43E-17	2.94E-17	4.97E-17	5.22E-17	5.31E-17	3.46E-17	5.01E-17	5.97E-17	5.72E-17	6.53E-17	7.59E-17	7.33E-17
2.57E-17	2.10E-17	2.46E-17	3.56E-17	5.16E-17	6.30E-17	6.39E-17	4.23E-17	5.69E-17	5.80E-17	5.17E-17	6.91E-17	8.10E-17	8.41E-17
3.92E-17	2.26E-17	3.08E-17	3.85E-17	5.56E-17	6.49E-17	7.16E-17	6.23E-17	6.22E-17	5.75E-17	5.75E-17	6.86E-17	8.16E-17	9.41E-17
4.86E-17	2.91E-17	3.61E-17	4.54E-17	5.83E-17	6.44E-17	7.15E-17	6.39E-17	6.35E-17	7.01E-17	7.20E-17	7.25E-17	8.58E-17	1.06E-16
5.69E-17	3.50E-17	4.37E-17	5.33E-17	6.38E-17	5.71E-17	7.21E-17	7.07E-17	6.81E-17	8.04E-17	7.92E-17	7.98E-17	9.04E-17	1.08E-16
5.53E-17	4.14E-17	5.05E-17	5.74E-17	6.56E-17	5.28E-17	6.62E-17	7.30E-17	7.53E-17	9.07E-17	8.22E-17	8.75E-17	9.13E-17	1.05E-16
5.47E-17	4.69E-17	5.65E-17	5.92E-17	6.72E-17	5.82E-17	6.55E-17	7.51E-17	7.85E-17	8.95E-17	9.14E-17	9.51E-17	9.57E-17	9.64E-17
5.53E-17	4.76E-17	5.97E-17	6.55E-17	7.17E-17	6.83E-17	6.96E-17	7.44E-17	7.63E-17	8.93E-17	9.09E-17	8.84E-17	1.00E-16	1.02E-16
5.24E-17	4.87E-17	5.80E-17	6.51E-17	7.75E-17	7.23E-17	7.55E-17	7.66E-17	7.74E-17	9.71E-17	8.79E-17	8.90E-17	1.05E-16	1.15E-16
5.48E-17	5.97E-17	5.64E-17	6.58E-17	7.68E-17	7.69E-17	7.62E-17	7.89E-17	8.55E-17	1.03E-16	9.07E-17	9.80E-17	1.06E-16	1.25E-16
6.34E-17	6.81E-17	6.97E-17	7.03E-17	7.53E-17	7.87E-17	8.94E-17	8.67E-17	9.27E-17	1.02E-16	9.85E-17	9.75E-17	1.06E-16	1.37E-16
8.08E-17	7.12E-17	7.06E-17	8.21E-17	7.60E-17	8.76E-17	1.01E-16	9.76E-17	9.50E-17	9.86E-17	1.01E-16	1.03E-16	1.13E-16	1.41E-16
8.05E-17	6.40E-17	7.23E-17	9.43E-17	7.64E-17	9.12E-17	1.06E-16	1.12E-16	9.74E-17	9.93E-17	1.01E-16	1.18E-16	1.14E-16	1.40E-16
7.65E-17	6.84E-17	8.40E-17	9.31E-17	8.38E-17	1.02E-16	1.10E-16	1.16E-16	1.04E-16	9.50E-17	1.02E-16	1.30E-16	1.18E-16	1.44E-16
8.05E-17	7.11E-17	9.65E-17	9.72E-17	1.08E-16	1.14E-16	1.13E-16	1.18E-16	1.11E-16	1.01E-16	1.14E-16	1.44E-16	1.33E-16	1.50E-16
8.38E-17	7.62E-17	1.00E-16	9.91E-17	1.05E-16	1.32E-16	1.18E-16	1.21E-16	1.14E-16	1.06E-16	1.14E-16	1.45E-16	1.32E-16	1.56E-16
9.43E-17	8.44E-17	1.00E-16	9.97E-17	1.00E-16	1.39E-16	1.19E-16	1.24E-16	1.22E-16	1.20E-16	1.17E-16	1.53E-16	1.49E-16	1.69E-16
9.79E-17	9.38E-17	9.97E-17	9.79E-17	1.00E-16	1.31E-16	1.23E-16	1.26E-16	1.31E-16	1.26E-16	1.30E-16	1.63E-16	1.72E-16	1.75E-16
9.72E-17	1.01E-16	9.58E-17	1.05E-16	1.06E-16	1.22E-16	1.21E-16	1.28E-16	1.34E-16	1.26E-16	1.40E-16	1.67E-16	1.72E-16	1.77E-16
9.73E-17	1.03E-16	9.95E-17	1.30E-16	1.12E-16	1.20E-16	1.18E-16	1.33E-16	1.33E-16	1.28E-16	1.48E-16	1.62E-16	1.68E-16	1.80E-16
9.57E-17	9.99E-17	9.29E-17	1.26E-16	1.17E-16	1.21E-16	1.15E-16	1.38E-16	1.35E-16	1.39E-16	1.54E-16	1.66E-16	1.80E-16	1.92E-16
8.71E-17	8.43E-17	9.89E-17	1.27E-16	1.19E-16	1.23E-16	1.25E-16	1.42E-16	1.46E-16	1.52E-16	1.64E-16	1.81E-16	1.88E-16	1.97E-16
8.98E-17	8.39E-17	1.05E-16	1.37E-16	1.12E-16	1.33E-16	1.37E-16	1.46E-16	1.59E-16	1.58E-16	1.65E-16	1.79E-16	1.88E-16	2.00E-16
8.61E-17	9.55E-17	1.07E-16	1.44E-16	9.20E-17	1.45E-16	1.44E-16	1.37E-16	1.65E-16	1.63E-16	1.71E-16	1.77E-16	1.87E-16	2.08E-16
9.06E-17	1.00E-16	9.60E-17	1.38E-16	1.06E-16	1.47E-16	1.35E-16	1.32E-16	1.71E-16	1.72E-16	1.71E-16	1.72E-16	1.92E-16	2.07E-16
8.64E-17	1.10E-16	1.01E-16	1.42E-16	1.55E-16	1.52E-16	1.32E-16	1.32E-16	1.78E-16	1.72E-16	1.68E-16	1.74E-16	1.92E-16	2.12E-16
9.43E-17	1.25E-16	1.10E-16	1.43E-16	1.55E-16	1.52E-16	1.41E-16	1.39E-16	1.87E-16	1.76E-16	1.61E-16	1.66E-16	1.82E-16	1.98E-16
1.00E-16	1.25E-16	1.21E-16	1.43E-16	1.48E-16	1.44E-16	1.57E-16	1.67E-16	1.96E-16	1.71E-16	1.55E-16	1.69E-16	1.85E-16	1.98E-16
1.17E-16	1.32E-16	1.27E-16	1.34E-16	1.30E-16	1.49E-16	1.65E-16	1.69E-16	1.73E-16	1.63E-16	1.70E-16	1.81E-16	2.00E-16	2.19E-16

1.17E-16	1.42E-16	1.27E-16	1.27E-16	1.21E-16	1.47E-16	1.62E-16	1.64E-16	1.53E-16	1.66E-16	1.82E-16	1.78E-16	1.96E-16	2.35E-16
1.21E-16	1.40E-16	1.35E-16	1.30E-16	1.23E-16	1.54E-16	1.48E-16	1.51E-16	1.60E-16	1.80E-16	2.01E-16	1.83E-16	1.96E-16	2.38E-16
1.19E-16	1.41E-16	1.42E-16	1.40E-16	1.47E-16	1.51E-16	1.40E-16	1.47E-16	1.61E-16	1.81E-16	2.00E-16	1.97E-16	2.10E-16	2.27E-16
1.21E-16	1.41E-16	1.31E-16	1.30E-16	1.56E-16	1.59E-16	1.56E-16	1.66E-16	1.79E-16	1.83E-16	2.04E-16	1.91E-16	1.99E-16	2.20E-16
1.23E-16	1.50E-16	1.35E-16	1.38E-16	1.61E-16	1.61E-16	1.64E-16	1.67E-16	1.81E-16	1.97E-16	2.18E-16	2.09E-16	2.18E-16	2.24E-16
1.32E-16	1.49E-16	1.41E-16	1.35E-16	1.63E-16	1.66E-16	1.72E-16	1.67E-16	1.88E-16	2.02E-16	2.12E-16	1.99E-16	2.33E-16	2.46E-16
1.27E-16	1.34E-16	1.26E-16	1.31E-16	1.57E-16	1.68E-16	1.87E-16	1.65E-16	1.82E-16	2.02E-16	2.09E-16	1.94E-16	2.43E-16	2.46E-16
1.24E-16	1.29E-16	1.10E-16	1.22E-16	1.46E-16	1.67E-16	1.89E-16	1.73E-16	1.77E-16	2.01E-16	2.10E-16	1.93E-16	2.45E-16	2.44E-16
1.19E-16	1.29E-16	1.09E-16	1.28E-16	1.40E-16	1.73E-16	1.83E-16	1.79E-16	1.75E-16	2.06E-16	2.14E-16	2.07E-16	2.54E-16	2.54E-16
1.19E-16	1.35E-16	1.23E-16	1.39E-16	1.43E-16	1.72E-16	1.73E-16	1.80E-16	1.83E-16	2.02E-16	2.10E-16	2.01E-16	2.48E-16	2.56E-16
1.21E-16	1.34E-16	1.35E-16	1.49E-16	1.48E-16	1.64E-16	1.54E-16	1.77E-16	1.83E-16	1.98E-16	2.06E-16	2.06E-16	2.36E-16	2.44E-16
1.31E-16	1.27E-16	1.34E-16	1.55E-16	1.48E-16	1.44E-16	1.24E-16	1.57E-16	1.71E-16	2.00E-16	1.98E-16	2.15E-16	2.35E-16	2.23E-16
1.23E-16	1.18E-16	1.28E-16	1.43E-16	1.45E-16	1.34E-16	1.03E-16	1.54E-16	1.59E-16	1.92E-16	1.89E-16	2.13E-16	2.27E-16	2.09E-16
1.15E-16	1.15E-16	1.24E-16	1.36E-16	1.34E-16	1.37E-16	1.09E-16	1.65E-16	1.66E-16	1.78E-16	1.72E-16	2.12E-16	2.18E-16	2.19E-16
1.16E-16	1.00E-16	1.03E-16	1.24E-16	1.31E-16	1.49E-16	1.43E-16	1.71E-16	1.66E-16	1.76E-16	1.63E-16	2.10E-16	2.17E-16	2.29E-16
1.02E-16	1.03E-16	1.07E-16	1.23E-16	1.31E-16	1.41E-16	1.60E-16	1.67E-16	1.58E-16	1.87E-16	1.92E-16	2.09E-16	2.11E-16	2.24E-16
8.63E-17	1.03E-16	1.09E-16	1.11E-16	1.19E-16	1.33E-16	1.65E-16	1.67E-16	1.59E-16	1.91E-16	1.98E-16	2.00E-16	1.92E-16	2.18E-16
7.12E-17	8.86E-17	9.78E-17	8.94E-17	9.31E-17	1.21E-16	1.49E-16	1.67E-16	1.68E-16	1.96E-16	1.90E-16	2.06E-16	1.92E-16	2.17E-16
5.40E-17	7.95E-17	8.29E-17	6.34E-17	7.48E-17	1.04E-16	1.41E-16	1.78E-16	1.62E-16	2.02E-16	1.84E-16	2.04E-16	1.93E-16	2.15E-16
5.14E-17	7.96E-17	7.33E-17	5.44E-17	8.06E-17	1.06E-16	1.51E-16	1.81E-16	1.62E-16	1.92E-16	1.81E-16	2.03E-16	2.05E-16	2.09E-16
5.98E-17	9.03E-17	7.93E-17	6.56E-17	1.07E-16	1.28E-16	1.61E-16	1.77E-16	1.61E-16	1.81E-16	1.79E-16	2.03E-16	2.16E-16	2.08E-16
8.55E-17	1.02E-16	1.02E-16	9.24E-17	1.21E-16	1.47E-16	1.58E-16	1.64E-16	1.47E-16	1.61E-16	1.57E-16	1.96E-16	2.30E-16	2.25E-16
9.48E-17	1.09E-16	1.09E-16	9.64E-17	1.03E-16	1.50E-16	1.46E-16	1.51E-16	1.36E-16	1.51E-16	1.27E-16	1.95E-16	2.23E-16	2.19E-16
9.32E-17	1.07E-16	1.00E-16	9.29E-17	1.02E-16	1.40E-16	1.33E-16	1.41E-16	1.24E-16	1.37E-16	1.20E-16	1.95E-16	2.11E-16	2.08E-16
9.55E-17	1.04E-16	9.43E-17	9.53E-17	1.10E-16	1.31E-16	1.23E-16	1.34E-16	1.22E-16	1.30E-16	1.52E-16	1.88E-16	1.95E-16	1.91E-16
9.95E-17	1.06E-16	1.04E-16	1.14E-16	1.23E-16	1.35E-16	1.16E-16	1.23E-16	1.19E-16	1.21E-16	1.41E-16	1.77E-16	1.72E-16	1.73E-16
1.03E-16	1.13E-16	1.16E-16	1.24E-16	1.17E-16	1.24E-16	1.10E-16	1.17E-16	1.21E-16	1.15E-16	1.34E-16	1.62E-16	1.49E-16	1.55E-16
1.01E-16	1.08E-16	1.15E-16	1.23E-16	1.06E-16	1.13E-16	1.14E-16	1.12E-16	1.24E-16	1.29E-16	1.34E-16	1.53E-16	1.26E-16	1.26E-16
1.00E-16	1.01E-16	1.14E-16	1.16E-16	1.11E-16	1.10E-16	1.05E-16	9.92E-17	1.22E-16	1.40E-16	1.29E-16	1.27E-16	9.15E-17	9.24E-17
1.03E-16	9.45E-17	1.15E-16	1.14E-16	1.14E-16	1.15E-16	9.91E-17	9.32E-17	1.14E-16	1.36E-16	1.25E-16	1.04E-16	6.11E-17	7.42E-17
1.02E-16	8.89E-17	1.00E-16	1.02E-16	1.01E-16	1.13E-16	1.02E-16	8.75E-17	1.06E-16	1.21E-16	1.24E-16	8.88E-17	5.64E-17	7.47E-17
9.54E-17	8.05E-17	8.79E-17	9.97E-17	1.01E-16	1.14E-16	9.44E-17	8.79E-17	9.69E-17	1.02E-16	1.19E-16	7.37E-17	6.21E-17	6.50E-17
9.79E-17	8.35E-17	8.76E-17	1.02E-16	1.04E-16	1.05E-16	9.68E-17	9.44E-17	9.14E-17	9.23E-17	9.70E-17	5.77E-17	4.23E-17	3.84E-17
9.85E-17	8.81E-17	9.31E-17	1.06E-16	9.69E-17	9.55E-17	1.01E-16	1.00E-16	9.30E-17	8.79E-17	8.38E-17	4.51E-17	2.05E-17	1.08E-17
9.44E-17	9.45E-17	1.04E-16	1.07E-16	8.58E-17	9.47E-17	9.71E-17	9.61E-17	9.86E-17	9.01E-17	8.34E-17	4.13E-17	1.08E-17	2.48E-18
8.98E-17	9.39E-17	1.08E-16	1.04E-16	8.33E-17	9.35E-17	9.08E-17	8.44E-17	1.00E-16	8.83E-17	8.41E-17	4.68E-17	1.62E-17	5.47E-18
8.21E-17	8.69E-17	1.05E-16	9.48E-17	7.85E-17	9.33E-17	8.96E-17	7.80E-17	9.60E-17	8.17E-17	8.38E-17	4.57E-17	2.21E-17	1.41E-17
7.19E-17	8.53E-17	9.42E-17	9.47E-17	7.47E-17	8.22E-17	8.55E-17	7.81E-17	8.90E-17	7.35E-17	8.30E-17	4.48E-17	2.91E-17	2.02E-17
6.22E-17	8.06E-17	8.00E-17	9.30E-17	6.95E-17	6.73E-17	7.72E-17	7.60E-17	8.64E-17	7.49E-17	7.72E-17	4.89E-17	3.95E-17	3.27E-17
5.71E-17	7.01E-17	7.34E-17	8.05E-17	6.44E-17	5.31E-17	6.70E-17	7.33E-17	7.69E-17	6.44E-17	7.07E-17	5.68E-17	5.64E-17	4.77E-17
4.96E-17	7.11E-17	6.82E-17	7.43E-17	5.76E-17	4.71E-17	5.98E-17	7.49E-17	7.34E-17	5.62E-17	6.59E-17	5.94E-17	6.22E-17	5.12E-17
5.35E-17	6.20E-17	5.50E-17	7.16E-17	6.01E-17	5.06E-17	6.12E-17	7.63E-17	6.49E-17	4.66E-17	5.43E-17	5.76E-17	5.57E-17	4.76E-17
5.26E-17	4.66E-17	4.08E-17	6.30E-17	7.04E-17	6.24E-17	7.07E-17	7.28E-17	5.71E-17	3.84E-17	4.09E-17	4.82E-17	4.10E-17	4.92E-17
4.77E-17	3.35E-17	2.94E-17	5.14E-17	7.65E-17	7.57E-17	7.41E-17	7.26E-17	5.50E-17	3.74E-17	2.45E-17	3.28E-17	2.54E-17	4.72E-17
3.72E-17	2.88E-17	2.78E-17	4.98E-17	7.19E-17	7.76E-17	7.19E-17	6.62E-17	5.08E-17	3.43E-17	1.56E-17	2.19E-17	1.76E-17	4.13E-17
4.06E-17	3.88E-17	3.50E-17	5.28E-17	6.35E-17	6.99E-17	6.48E-17	5.85E-17	4.73E-17	3.04E-17	2.18E-17	2.06E-17	2.48E-17	3.92E-17
4.36E-17	4.74E-17	3.93E-17	5.19E-17	5.51E-17	6.02E-17	5.75E-17	4.90E-17	4.38E-17	3.70E-17	3.52E-17	3.37E-17	3.08E-17	3.64E-17
4.01E-17	4.86E-17	4.40E-17	5.06E-17	4.97E-17	5.68E-17	5.33E-17	4.67E-17	4.09E-17	4.05E-17	4.19E-17	3.72E-17	3.65E-17	3.48E-17
3.98E-17	5.55E-17	4.79E-17	4.87E-17	3.98E-17	5.35E-17	4.58E-17	4.64E-17	3.59E-17	3.76E-17	3.66E-17	4.16E-17	4.15E-17	3.76E-17
3.78E-17	5.53E-17	4.30E-17	4.32E-17	3.40E-17	4.63E-17	3.89E-17	3.98E-17	2.14E-17	3.13E-17	3.59E-17	4.26E-17	4.29E-17	4.12E-17
2.93E-17	4.47E-17	3.84E-17	3.88E-17	4.13E-17	3.81E-17	2.90E-17	2.78E-17	1.92E-17	2.90E-17	4.13E-17	4.04E-17	4.46E-17	4.06E-17
3.06E-17	3.06E-17	3.15E-17	3.35E-17	4.16E-17	3.29E-17	1.88E-17	1.80E-17	2.72E-17	2.93E-17	4.05E-17	4.02E-17	4.41E-17	3.91E-17
2.55E-17	1.72E-17	2.37E-17	2.82E-17	3.77E-17	2.41E-17	1.45E-17	1.40E-17	2.21E-17	2.35E-17	3.27E-17	3.97E-17	4.14E-17	3.47E-17
1.29E-17	6.46E-18	1.61E-17	2.31E-17	3.42E-17	2.37E-17	1.24E-17	1.19E-17	1.93E-17	1.71E-17	2.30E-17	3.17E-17	3.67E-17	2.72E-17
8.58E-18	4.04E-18	1.23E-17	1.93E-17	2.67E-17	1.95E-17	7.69E-18	9.95E-18	1.47E-17	1.25E-17	1.85E-17	2.37E-17	2.69E-17	2.46E-17
7.62E-18	6.22E-18	1.06E-17	1.55E-17	1.51E-17	1.36E-17	5.45E-18	3.71E-18	1.01E-17	1.21E-17	1.67E-17	1.75E-17	1.90E-17	2.19E-17
1.26E-17	8.42E-18	1.29E-17	1.23E-17	1.27E-17	9.96E-18	3.86E-18	2.34E-18	5.35E-18	1.25E-17	1.18E-17	1.40E-17	1.44E-17	1.64E-17
1.12E-17	6.36E-18	9.56E-18	9.49E-18	6.89E-18	7.58E-18	3.07E-18	1.13E-18	3.95E-18	1.15E-17	9.71E-18	1.15E-17	1.25E-17	1.04E-17
1.01E-17	4.03E-18	5.78E-18	8.01E-18	4.77E-18	5.11E-18	3.01E-18	6.20E-19	2.48E-18	8.60E-18	8.48E-18	1.07E-17	7.94E-18	5.45E-18
7.07E-18	2.30E-18	5.17E-18	4.28E-18	3.61E-18	3.60E-18	2.79E-18	9.31E-20	1.26E-18	6.42E-18	6.25E-18	6.61E-18	2.99E-18	2.38E-18
5.28E-18	1.06E-18	3.06E-18	1.80E-18	1.70E-18	2.65E-18	2.22E-18	7.85E-20	2.07E-20	3.88E-18	3.81E-18	3.10E-18	2.35E-18	1.31E-18
2.42E-18	3.38E-19	4.77E-19	3.96E-19	7.68E-19	8.27E-19	1.13E-18	4.49E-19	3.43E-20	1.75E-18	2.00E-18	9.22E-19	4.28E-19	3.06E-19
1.39E-18	3.32E-20	6.89E-20	6.57E-21	1.91E-21	8.31E-20	1.60E-20	2.73E-22	2.29E-20	3.00E-19	3.07E-19	9.03E-20	1.49E-19	4.18E-20
1.98E-22	3.88E-19	4.05E-20	1.26E-19	2.79E-19	2.60E-20	1.42E-18	1.37E-18	3.46E-19	1.62E-20	4.40E-20	2.41E-22	3.81E-19	1.60E-19
1.34E-18	1.46E-18	1.21E-18	4.73E-19	9.67E-19	1.06E-18	3.70E-18	4.90E-18	1.50E-18	9.15E-19	1.10E-18	8.63E-19	1.01E-18	5.75E-19
3.71E-18	2.17E-18	2.35E-18	1.53E-18	2.23E-18	3.99E-18	6.58E-18	5.58E-18	2.75E-18	2.83E-18	3.45E-18	5.09E-18	3.38E-18	2.26E-18
4.23E-18	3.86E-18	4.85E-18	5.37E-18	5.85E-18	8.29E-18	1.53E-17	1.20E-17	6.09E-18	9.43E-18	7.91E-18	1.20E-17	8.22E-18	7.68E-18
7.55E-18	8.44E-18	7.76E-18	1.10E-17	1.26E-17	2.19E-17	2.28E-17	2.24E-17	1.38E-17	2.09E-17	1.47E-17	1.85E-17	1.55E-17	1.66E-17
1.18E-17	1.74E-17	1.35E-17	1.82E-17	2.27E-17	4.15E-17	2.99E-17	3.24E-17	2.56E-17	3.61E-17	2.39E-17	2.49E-17	2.61E-17	2.93E-17
1.69E-17	2.64E-17	2.15E-17	2.67E-17	3.10E-17	5.66E-17	3.73E-17	3.14E-17	3.14E-17	4.30E-17	3.62E-17	3.26E-17	3.81E-17	4.23E-17
2.75E-17	3.94E-17	3.82E-17	4.25E-17	4.33E-17	6.62E-17	4.36E-17	3.88E-17	3.52E-17	5.25E-17	5.56E-17	4.32E-17	4.75E-17	5.65E-17
4.12E-17	5.56E-17	5.34E-17	5.22E-17	6.62E-17	5.73E-17	4.77E-17	5.25E-17	4.98E-17	6.44E-17	8.81E-17	5.62E-17	6.49E-17	7.82E-17
6.28E-17	7.64E-17	6.91E-17	6.06E-17	7.57E-17	5.45E-17	5.45E-17	6.60E-17	7.04E-17	8.15E-17	1.02E-16	7.10E-17	8.18E-17	9.10E-17
8.82E-17	8.31E-17	8.16E-17	6.79E-17	8.60E-17	6.92E-17	7.81E-17	8.12E-17	8.90E-17	1.00E-16	1.14E-16	8.98E-17	9.64E-17	1.07E-16
1.09E-16	8.81E-17	8.53E-17	8.60E-17	1.03E-16	9.95E-17	1.07E-16	9.88E-17	1.05E-16	1.13E-16	1.29E-16	1.07E-16	1.21E-16	1.29E-16
1.39E-16	1.01E-16	9.44E-17	1.04E-16	1.19E-16	1.43E-16	1.34E-16	1.21E-16	1.21E-16	1.33E-16	1.48E-16	1.37E-16	1.36E-16	1.54E-16
1.54E-16	1.31E-16	1.28E-16	1.41E-16	1.51E-16	1.80E-16	1.65E-16	1.50E-16	1.36E-16	1.68E-16	1.75E-16	1.66E-16	1.63E-16	1.77E-16
1.70E-16	1.65E-16	1.79E-16	2.01E-16	1.84E-16	2.17E-16	2.03E-16	1.88E-16	1.66E-16	1.99E-16	2.06E-16	2.03E-16	1.83E-16	2.03E-16
1.95E-16	1.91E-16	2.43E-16	2.65E-16	2.17E-16	2.27E-16	2.28E-16	2.39E-16	2.16E-16	2.39E-16	2.33E-16	2.38E-16	2.10E-16	2.35E-16
2.31E-16	2.28E-16	2.75E-16	2.99E-16	2.33E-16	2.45E-16	2.45E-16	2.69E-16	2.67E-16	3.05E-16	2.68E-16	2.67E-16	2.47E-16	2.69E-16
2.60E-16	2.61E-16	2.73E-16	3.39E-16	2.62E-16	2.73E-16	2.80E-16	2.96E-16	3.29E-16	3.65E-16	3.29E-16	3.04E-16	2.84E-16	3.07E-16
3.18E-16	2.93E-16	2.79E-16	3.59E-16	2.94E-16	3.17E-16	3.34E-16	3.47E-16	3.91E-16	4.27E-16	3.91E-16	3.54E-16	3.20E-16	3.58E-16
3.50E-16	3.15E-16	3.04E-16	3.51E-16	3.26E-16	3.69E-16	3.99E-16	3.95E-16	4.44E-16	4.53E-16	4.32E-16	4.04E-16	3.71E-16	4.11E-16
3.80E-16	3.50E-16	3.64E-16	3.72E-16	3.72E-16	4.46E-16	4.42E-16	4.30E-16	4.83E-16	4.80E-16	4.81E-16	4.70E-16	4.26E-16	4.79E-16
3.94E-16	3.76E-16	4.26E-16	4.31E-16	4.33E-16	5.35E-16	4.90E-16	4.84E-16	5.25E-16	5.34E-16	5.19E-16	5.11E-16	4.81E-16	5.44E-16
4.42E-16	4.29E-16	4.70E-16	4.95E-16	5.07E-16	5.94E-16	5.27E-16	5.43E-16	5.86E-16	5.77E-16	5.60E-16	5.70E-16	5.57E-16	6.21E-16

5.10E-16	4.87E-16	5.29E-16	5.49E-16	5.66E-16	6.41E-16	5.88E-16	5.94E-16	6.27E-16	6.33E-16	6.06E-16	6.39E-16	6.29E-16	6.79E-16
5.69E-16	5.55E-16	6.08E-16	6.16E-16	6.34E-16	6.68E-16	6.32E-16	6.47E-16	6.62E-16	6.66E-16	6.76E-16	6.96E-16	6.90E-16	7.27E-16
6.15E-16	6.26E-16	6.72E-16	7.06E-16	7.13E-16	7.32E-16	6.97E-16	7.19E-16	7.53E-16	7.20E-16	7.59E-16	7.86E-16	7.86E-16	7.86E-16
6.66E-16	7.08E-16	7.30E-16	7.66E-16	8.13E-16	8.37E-16	7.73E-16	8.04E-16	8.68E-16	8.00E-16	8.70E-16	8.71E-16	9.03E-16	8.74E-16
7.27E-16	7.76E-16	7.93E-16	8.40E-16	9.18E-16	9.03E-16	8.51E-16	8.78E-16	9.63E-16	8.80E-16	9.46E-16	9.37E-16	9.95E-16	9.95E-16
8.02E-16	8.47E-16	8.55E-16	9.60E-16	9.97E-16	9.49E-16	9.36E-16	9.73E-16	1.03E-15	9.62E-16	1.02E-15	1.04E-15	1.12E-15	1.12E-15
8.73E-16	9.31E-16	9.40E-16	1.04E-15	1.05E-15	1.02E-15	1.05E-15	1.10E-15	1.09E-15	1.08E-15	1.13E-15	1.14E-15	1.20E-15	1.21E-15
9.48E-16	1.01E-15	1.03E-15	1.12E-15	1.12E-15	1.11E-15	1.19E-15	1.18E-15	1.18E-15	1.20E-15	1.25E-15	1.26E-15	1.29E-15	1.30E-15
1.04E-15	1.09E-15	1.12E-15	1.21E-15	1.25E-15	1.22E-15	1.32E-15	1.30E-15	1.28E-15	1.32E-15	1.35E-15	1.38E-15	1.38E-15	1.39E-15
1.13E-15	1.19E-15	1.22E-15	1.30E-15	1.35E-15	1.37E-15	1.41E-15	1.41E-15	1.41E-15	1.46E-15	1.47E-15	1.54E-15	1.52E-15	1.51E-15
1.30E-15	1.34E-15	1.34E-15	1.40E-15	1.44E-15	1.50E-15	1.49E-15	1.52E-15	1.53E-15	1.59E-15	1.59E-15	1.65E-15	1.63E-15	1.65E-15
1.94E-14	1.99E-14	2.06E-14	2.23E-14	2.26E-14	2.40E-14	2.39E-14	2.44E-14	2.51E-14	2.58E-14	2.63E-14	2.68E-14	2.72E-14	2.83E-14

-7.68E-09	-8.08E-09	-8.36E-09	-8.45E-09	-8.99E-09	-8.87E-09	-8.81E-09	-8.10E-09	-8.13E-09	-9.13E-09	-8.82E-09	-8.95E-09	-8.23E-09	-7.79E-09
-8.06E-09	-8.54E-09	-8.66E-09	-8.97E-09	-9.34E-09	-9.04E-09	-9.01E-09	-8.39E-09	-8.22E-09	-9.23E-09	-9.47E-09	-9.90E-09	-8.64E-09	-8.44E-09
-8.47E-09	-9.20E-09	-9.21E-09	-9.60E-09	-9.94E-09	-9.47E-09	-8.99E-09	-8.02E-09	-8.25E-09	-9.31E-09	-9.80E-09	-1.02E-08	-8.26E-09	-9.02E-09
-8.71E-09	-9.99E-09	-9.49E-09	-9.89E-09	-1.02E-08	-9.59E-09	-8.45E-09	-7.93E-09	-8.37E-09	-9.62E-09	-9.92E-09	-1.02E-08	-7.62E-09	-9.48E-09
-9.31E-09	-1.04E-08	-9.87E-09	-9.68E-09	-1.00E-08	-9.80E-09	-8.32E-09	-7.96E-09	-9.34E-09	-9.72E-09	-9.88E-09	-9.83E-09	-9.52E-09	-1.03E-08
-9.96E-09	-1.04E-08	-1.04E-08	-1.00E-08	-9.66E-09	-1.00E-08	-8.54E-09	-8.62E-09	-9.63E-09	-1.01E-08	-9.76E-09	-9.84E-09	-1.12E-08	-1.07E-08
-9.99E-09	-1.05E-08	-1.10E-08	-1.06E-08	-9.70E-09	-1.05E-08	-9.77E-09	-9.34E-09	-9.83E-09	-1.03E-08	-1.02E-08	-1.03E-08	-1.14E-08	-1.09E-08
-1.02E-08	-1.07E-08	-1.09E-08	-1.10E-08	-1.05E-08	-1.05E-08	-1.04E-08	-1.03E-08	-1.08E-08	-1.01E-08	-1.10E-08	-1.08E-08	-1.16E-08	-1.09E-08
-1.02E-08	-1.05E-08	-1.12E-08	-1.11E-08	-1.11E-08	-1.04E-08	-1.05E-08	-1.08E-08	-1.14E-08	-1.03E-08	-1.12E-08	-1.13E-08	-1.19E-08	-1.10E-08
-1.03E-08	-1.04E-08	-1.12E-08	-1.14E-08	-1.10E-08	-1.01E-08	-1.04E-08	-1.08E-08	-1.16E-08	-1.06E-08	-1.13E-08	-1.11E-08	-1.20E-08	-1.11E-08
-1.06E-08	-1.13E-08	-1.16E-08	-1.17E-08	-1.11E-08	-1.03E-08	-1.04E-08	-1.13E-08	-1.14E-08	-1.12E-08	-1.11E-08	-1.07E-08	-1.20E-08	-1.12E-08
-1.14E-08	-1.21E-08	-1.19E-08	-1.17E-08	-1.14E-08	-1.11E-08	-1.12E-08	-1.18E-08	-1.15E-08	-1.13E-08	-1.11E-08	-1.17E-08	-1.23E-08	-1.19E-08
-1.18E-08	-1.22E-08	-1.23E-08	-1.15E-08	-1.17E-08	-1.21E-08	-1.21E-08	-1.20E-08	-1.19E-08	-1.10E-08	-1.14E-08	-1.21E-08	-1.28E-08	-1.22E-08
-1.19E-08	-1.21E-08	-1.24E-08	-1.17E-08	-1.19E-08	-1.26E-08	-1.26E-08	-1.24E-08	-1.23E-08	-1.12E-08	-1.22E-08	-1.25E-08	-1.28E-08	-1.23E-08
-1.17E-08	-1.23E-08	-1.24E-08	-1.19E-08	-1.19E-08	-1.28E-08	-1.26E-08	-1.30E-08	-1.20E-08	-1.18E-08	-1.30E-08	-1.28E-08	-1.29E-08	-1.28E-08
-1.16E-08	-1.26E-08	-1.24E-08	-1.21E-08	-1.22E-08	-1.28E-08	-1.31E-08	-1.34E-08	-1.28E-08	-1.26E-08	-1.39E-08	-1.31E-08	-1.31E-08	-1.33E-08
-1.14E-08	-1.28E-08	-1.23E-08	-1.21E-08	-1.24E-08	-1.28E-08	-1.30E-08	-1.37E-08	-1.34E-08	-1.32E-08	-1.42E-08	-1.34E-08	-1.34E-08	-1.37E-08
-1.16E-08	-1.29E-08	-1.21E-08	-1.23E-08	-1.26E-08	-1.28E-08	-1.28E-08	-1.37E-08	-1.37E-08	-1.38E-08	-1.43E-08	-1.40E-08	-1.40E-08	-1.46E-08
-1.23E-08	-1.37E-08	-1.24E-08	-1.28E-08	-1.29E-08	-1.33E-08	-1.29E-08	-1.36E-08	-1.34E-08	-1.41E-08	-1.45E-08	-1.43E-08	-1.48E-08	-1.48E-08
-1.30E-08	-1.41E-08	-1.26E-08	-1.29E-08	-1.33E-08	-1.37E-08	-1.30E-08	-1.37E-08	-1.33E-08	-1.45E-08	-1.45E-08	-1.49E-08	-1.52E-08	-1.47E-08
-1.33E-08	-1.43E-08	-1.29E-08	-1.32E-08	-1.34E-08	-1.39E-08	-1.36E-08	-1.42E-08	-1.38E-08	-1.49E-08	-1.48E-08	-1.56E-08	-1.56E-08	-1.49E-08
-1.36E-08	-1.42E-08	-1.32E-08	-1.37E-08	-1.39E-08	-1.43E-08	-1.45E-08	-1.52E-08	-1.46E-08	-1.53E-08	-1.51E-08	-1.60E-08	-1.57E-08	-1.49E-08
-1.38E-08	-1.40E-08	-1.36E-08	-1.43E-08	-1.43E-08	-1.47E-08	-1.47E-08	-1.55E-08	-1.50E-08	-1.51E-08	-1.53E-08	-1.62E-08	-1.57E-08	-1.47E-08
-1.41E-08	-1.36E-08	-1.39E-08	-1.47E-08	-1.48E-08	-1.50E-08	-1.50E-08	-1.56E-08	-1.51E-08	-1.49E-08	-1.52E-08	-1.61E-08	-1.59E-08	-1.53E-08
-1.45E-08	-1.37E-08	-1.39E-08	-1.49E-08	-1.52E-08	-1.52E-08	-1.52E-08	-1.57E-08	-1.54E-08	-1.50E-08	-1.54E-08	-1.59E-08	-1.62E-08	-1.60E-08
-1.49E-08	-1.40E-08	-1.39E-08	-1.50E-08	-1.55E-08	-1.49E-08	-1.58E-08	-1.63E-08	-1.57E-08	-1.55E-08	-1.58E-08	-1.58E-08	-1.62E-08	-1.63E-08
-1.50E-08	-1.44E-08	-1.38E-08	-1.50E-08	-1.53E-08	-1.46E-08	-1.62E-08	-1.64E-08	-1.62E-08	-1.61E-08	-1.62E-08	-1.60E-08	-1.62E-08	-1.66E-08
-1.49E-08	-1.46E-08	-1.39E-08	-1.53E-08	-1.55E-08	-1.51E-08	-1.62E-08	-1.65E-08	-1.62E-08	-1.64E-08	-1.61E-08	-1.64E-08	-1.65E-08	-1.66E-08
-1.45E-08	-1.52E-08	-1.42E-08	-1.53E-08	-1.56E-08	-1.56E-08	-1.58E-08	-1.62E-08	-1.57E-08	-1.65E-08	-1.63E-08	-1.63E-08	-1.65E-08	-1.68E-08
-1.40E-08	-1.54E-08	-1.44E-08	-1.48E-08	-1.54E-08	-1.57E-08	-1.58E-08	-1.59E-08	-1.56E-08	-1.66E-08	-1.63E-08	-1.67E-08	-1.70E-08	-1.74E-08
-1.45E-08	-1.54E-08	-1.52E-08	-1.48E-08	-1.54E-08	-1.55E-08	-1.55E-08	-1.60E-08	-1.67E-08	-1.66E-08	-1.63E-08	-1.70E-08	-1.75E-08	-1.77E-08
-1.47E-08	-1.51E-08	-1.52E-08	-1.51E-08	-1.54E-08	-1.51E-08	-1.55E-08	-1.65E-08	-1.66E-08	-1.68E-08	-1.69E-08	-1.74E-08	-1.77E-08	-1.81E-08
-1.46E-08	-1.49E-08	-1.52E-08	-1.51E-08	-1.54E-08	-1.53E-08	-1.57E-08	-1.70E-08	-1.64E-08	-1.74E-08	-1.74E-08	-1.76E-08	-1.78E-08	-1.81E-08
-1.49E-08	-1.46E-08	-1.53E-08	-1.53E-08	-1.54E-08	-1.55E-08	-1.59E-08	-1.68E-08	-1.59E-08	-1.76E-08	-1.77E-08	-1.80E-08	-1.80E-08	-1.76E-08
-1.50E-08	-1.50E-08	-1.54E-08	-1.55E-08	-1.52E-08	-1.57E-08	-1.61E-08	-1.64E-08	-1.64E-08	-1.76E-08	-1.76E-08	-1.78E-08	-1.81E-08	-1.73E-08
-1.51E-08	-1.46E-08	-1.54E-08	-1.58E-08	-1.48E-08	-1.60E-08	-1.61E-08	-1.64E-08	-1.69E-08	-1.76E-08	-1.75E-08	-1.75E-08	-1.82E-08	-1.73E-08
-1.58E-08	-1.50E-08	-1.57E-08	-1.61E-08	-1.51E-08	-1.58E-08	-1.59E-08	-1.63E-08	-1.70E-08	-1.76E-08	-1.75E-08	-1.79E-08	-1.81E-08	-1.74E-08
-1.55E-08	-1.50E-08	-1.60E-08	-1.62E-08	-1.55E-08	-1.65E-08	-1.67E-08	-1.63E-08	-1.67E-08	-1.76E-08	-1.76E-08	-1.78E-08	-1.79E-08	-1.72E-08
-1.49E-08	-1.57E-08	-1.64E-08	-1.61E-08	-1.56E-08	-1.64E-08	-1.68E-08	-1.67E-08	-1.77E-08	-1.81E-08	-1.81E-08	-1.81E-08	-1.80E-08	-1.77E-08
-1.44E-08	-1.53E-08	-1.62E-08	-1.60E-08	-1.62E-08	-1.63E-08	-1.62E-08	-1.65E-08	-1.79E-08	-1.79E-08	-1.80E-08	-1.81E-08	-1.77E-08	-1.84E-08
-1.44E-08	-1.52E-08	-1.59E-08	-1.61E-08	-1.66E-08	-1.63E-08	-1.57E-08	-1.62E-08	-1.78E-08	-1.80E-08	-1.82E-08	-1.80E-08	-1.75E-08	-1.86E-08
-1.50E-08	-1.55E-08	-1.59E-08	-1.58E-08	-1.68E-08	-1.60E-08	-1.61E-08	-1.66E-08	-1.75E-08	-1.76E-08	-1.79E-08	-1.79E-08	-1.78E-08	-1.89E-08
-1.48E-08	-1.57E-08	-1.61E-08	-1.64E-08	-1.64E-08	-1.59E-08	-1.66E-08	-1.69E-08	-1.72E-08	-1.76E-08	-1.79E-08	-1.79E-08	-1.76E-08	-1.85E-08
-1.43E-08	-1.59E-08	-1.57E-08	-1.68E-08	-1.67E-08	-1.63E-08	-1.66E-08	-1.70E-08	-1.73E-08	-1.76E-08	-1.77E-08	-1.75E-08	-1.78E-08	-1.84E-08
-1.42E-08	-1.61E-08	-1.58E-08	-1.63E-08	-1.66E-08	-1.62E-08	-1.68E-08	-1.71E-08	-1.69E-08	-1.73E-08	-1.76E-08	-1.77E-08	-1.82E-08	-1.83E-08
-1.52E-08	-1.60E-08	-1.59E-08	-1.59E-08	-1.64E-08	-1.63E-08	-1.67E-08	-1.68E-08	-1.68E-08	-1.67E-08	-1.75E-08	-1.75E-08	-1.79E-08	-1.84E-08
-1.53E-08	-1.55E-08	-1.54E-08	-1.52E-08	-1.60E-08	-1.60E-08	-1.64E-08	-1.65E-08	-1.66E-08	-1.65E-08	-1.74E-08	-1.71E-08	-1.73E-08	-1.81E-08
-1.54E-08	-1.55E-08	-1.49E-08	-1.45E-08	-1.56E-08	-1.54E-08	-1.61E-08	-1.65E-08	-1.64E-08	-1.64E-08	-1.72E-08	-1.66E-08	-1.69E-08	-1.81E-08
-1.55E-08	-1.55E-08	-1.55E-08	-1.46E-08	-1.52E-08	-1.48E-08	-1.57E-08	-1.61E-08	-1.65E-08	-1.67E-08	-1.69E-08	-1.62E-08	-1.67E-08	-1.78E-08
-1.57E-08	-1.57E-08	-1.61E-08	-1.51E-08	-1.50E-08	-1.48E-08	-1.54E-08	-1.55E-08	-1.63E-08	-1.70E-08	-1.69E-08	-1.65E-08	-1.70E-08	-1.75E-08
-1.56E-08	-1.54E-08	-1.60E-08	-1.50E-08	-1.51E-08	-1.52E-08	-1.58E-08	-1.60E-08	-1.61E-08	-1.67E-08	-1.66E-08	-1.71E-08	-1.71E-08	-1.75E-08
-1.55E-08	-1.50E-08	-1.55E-08	-1.50E-08	-1.49E-08	-1.55E-08	-1.59E-08	-1.63E-08	-1.59E-08	-1.66E-08	-1.67E-08	-1.75E-08	-1.73E-08	-1.77E-08
-1.50E-08	-1.45E-08	-1.48E-08	-1.46E-08	-1.47E-08	-1.54E-08	-1.58E-08	-1.64E-08	-1.56E-08	-1.60E-08	-1.60E-08	-1.73E-08	-1.73E-08	-1.77E-08
-1.46E-08	-1.35E-08	-1.41E-08	-1.42E-08	-1.46E-08	-1.50E-08	-1.60E-08	-1.63E-08	-1.55E-08	-1.57E-08	-1.56E-08	-1.71E-08	-1.74E-08	-1.78E-08
-1.41E-08	-1.25E-08	-1.42E-08	-1.37E-08	-1.46E-08	-1.49E-08	-1.55E-08	-1.57E-08	-1.53E-08	-1.55E-08	-1.61E-08	-1.72E-08	-1.70E-08	-1.73E-08
-1.37E-08	-1.11E-08	-1.50E-08	-1.26E-08	-1.38E-08	-1.45E-08	-1.49E-08	-1.51E-08	-1.51E-08	-1.56E-08	-1.65E-08	-1.69E-08	-1.60E-08	-1.66E-08
-1.29E-08	-1.03E-08	-1.49E-08	-1.14E-08	-1.37E-08	-1.47E-08	-1.48E-08	-1.47E-08	-1.54E-08	-1.56E-08	-1.61E-08	-1.62E-08	-1.53E-08	-1.58E-08
-1.28E-08	-1.22E-08	-1.48E-08	-1.10E-08	-1.33E-08	-1.46E-08	-1.47E-08	-1.50E-08	-1.57E-08	-1.59E-08	-1.58E-08	-1.61E-08	-1.48E-08	-1.52E-08
-1.28E-08	-1.33E-08	-1.42E-08	-1.35E-08	-1.37E-08	-1.44E-08	-1.46E-08	-1.50E-08	-1.54E-08	-1.58E-08	-1.55E-08	-1.60E-08	-1.48E-08	-1.57E-08
-1.20E-08	-1.29E-08	-1.36E-08	-1.34E-08	-1.34E-08	-1.41E-08	-1.47E-08	-1.46E-08	-1.50E-08	-1.56E-08	-1.49E-08	-1.56E-08	-1.51E-08	-1.55E-08
-1.09E-08	-1.18E-08	-1.26E-08	-1.34E-08	-1.30E-08	-1.37E-08	-1.45E-08	-1.39E-08	-1.42E-08	-1.51E-08	-1.43E-08	-1.47E-08	-1.52E-08	-1.50E-08
-9.78E-09	-1.09E-08	-1.17E-08	-1.31E-08	-1.26E-08	-1.32E-08	-1.42E-08	-1.36E-08	-1.35E-08	-1.43E-08	-1.32E-08	-1.36E-08	-1.48E-08	-1.51E-08
-8.95E-09	-9.67E-09	-1.10E-08	-1.24E-08	-1.20E-08	-1.27E-08	-1.37E-08	-1.34E-08	-1.34E-08	-1.33E-08	-1.25E-08	-1.31E-08	-1.42E-08	-1.51E-08
-7.72E-09	-8.31E-09	-1.00E-08	-1.13E-08	-1.14E-08	-1.22E-08	-1.35E-08	-1.36E-08	-1.34E-08	-1.28E-08	-1.27E-08	-1.36E-08	-1.39E-08	-1.45E-08
-5.04E-09	-7.29E-09	-8.87E-09	-1.04E-08	-1.10E-08	-1.17E-08	-1.28E-08	-1.35E-08	-1.33E-08	-1.29E-08	-1.33E-08	-1.35E-08	-1.37E-08	-1.39E-08
-3.10E-09	-5.92E-09	-7.45E-09	-9.61E-09	-1.09E-08	-1.16E-08	-1.26E-08	-1.35E-08	-1.28E-08	-1.29E-08	-1.38E-08	-1.38E-08	-1.34E-08	-1.36E-08
-3.07E-09	-4.60E-09	-5.93E-09	-8.12E-09	-1.00E-08	-1.15E-08	-1.22E-08	-1.26E-08	-1.19E-08	-1.22E-08	-1.34E-08	-1.32E-08	-1.27E-08	-1.30E-08
-3.85E-09	-3.52E-09	-3.65E-09	-6.15E-09	-9.00E-09	-1.10E-08	-1.14E-08	-1.20E-08	-1.16E-08	-1.17E-08	-1.29E-08	-1.23E-08	-1.19E-08	-1.26E-08
-3.91E-09	-2.88E-09	-1.87E-09	-4.65E-09	-7.84E-09	-1.02E-08	-1.07E-08	-1.13E-08	-1.14E-08	-1.17E-08	-1.19E-08	-1.17E-08	-1.07E-08	-1.20E-08
-4.95E-09	-3.90E-09	-1.66E-09	-3.28E-09	-6.63E-09	-8.97E-09	-9.88E-09	-1.01E-08	-1.02E-08	-1.04E-08	-9.81E-09	-9.95E-09	-9.55E-09	-1.27E-08
-6.01E-09	-4.29E-09	-1.91E-09	-2.34E-09	-5.87E-09	-7.33E-09	-8.73E-09	-8.76E-09	-8.75E-09	-8.29E-09	-6.78E-09	-7.27E-09	-8.49E-09	-1.22E-08
-6.73E-09	-4.83E-09	-3.18E-09	-3.02E-09	-5.30E-09	-6.20E-09	-7.83E-09	-7.53E-09	-7.11E-09	-5.83E-09	-3.78E-09	-4.93E-09	-7.63E-09	-6.34E-09
-7.34E-09	-5.69E-09	-4.35E-09	-4.51E-09	-6.04E-09	-5.94E-09	-6.94E-09	-6.52E-09	-6.26E-09	-4.68E-09	-3.05E-09	-3.77E-09	-5.00E-09	-1.78E-09
-7.64E-09	-6.03E-09	-5.38E-09	-5.10E-09	-6.91E-09	-5.73E-09	-6.50E-09	-6.45E-09	-6.61E-09	-5.72E-09	-4.72E-09	-5.44E-09	-6.72E-09	-7.51E-09
-7.40E-09	-6.41E-09	-6.22E-09	-6.03E-09	-7.36E-09	-6.01E-09	-6.67E-09	-6.64E-09	-7.18E-09	-6.46E-09	-6.82E-09	-7.03E-09	-7.77E-09	-8.22E-09
-7.05E-09	-5.73E-09	-6.49E-09	-6.62E-09	-7.27E-09	-6.39E-09	-7.08E-09	-6.77E-09	-7.40E-09	-7.02E-09	-7.48E-09	-7.54E-09	-8.21E-09	-8.49E-09
-6.64E-09	-5.06E-09	-6.59E-09	-6.83E-09	-7.25E-09	-6.47E-09	-7.37E-09	-7.10E-09	-7.36E-09	-6.80E-09	-7.12E-09	-7.27E-09	-7.71E-09	-8.58E-09
-6.40E-09	-5.25E-09	-7.01E-09	-7.31E-09	-7.16E-09	-6.23E-09	-7.18E-09	-7.00E-09	-7.41E-09	-6.57E-09	-6.65E-09	-6.67E-09	-7.08E-09	-7.97E-09

-6.02E-09	-6.27E-09	-7.41E-09	-7.31E-09	-7.22E-09	-6.47E-09	-6.75E-09	-7.05E-09	-7.27E-09	-6.17E-09	-6.33E-09	-6.50E-09	-6.80E-09	-7.50E-09
-6.32E-09	-6.88E-09	-7.46E-09	-7.28E-09	-7.28E-09	-6.79E-09	-5.92E-09	-6.58E-09	-6.86E-09	-6.14E-09	-6.47E-09	-6.69E-09	-6.94E-09	-7.03E-09
-6.35E-09	-6.75E-09	-6.99E-09	-6.95E-09	-6.71E-09	-6.90E-09	-5.71E-09	-6.18E-09	-6.51E-09	-6.49E-09	-6.68E-09	-6.72E-09	-6.86E-09	-6.64E-09
-6.16E-09	-6.39E-09	-6.52E-09	-6.17E-09	-5.83E-09	-6.60E-09	-5.85E-09	-5.69E-09	-6.39E-09	-6.66E-09	-6.58E-09	-6.92E-09	-6.62E-09	-6.19E-09
-6.11E-09	-6.42E-09	-6.08E-09	-5.47E-09	-5.27E-09	-6.42E-09	-5.77E-09	-5.47E-09	-5.98E-09	-6.23E-09	-6.18E-09	-6.66E-09	-6.40E-09	-5.72E-09
-5.36E-09	-5.86E-09	-5.73E-09	-5.30E-09	-4.92E-09	-5.97E-09	-5.42E-09	-4.82E-09	-5.54E-09	-5.95E-09	-5.85E-09	-6.24E-09	-6.02E-09	-4.79E-09
-5.10E-09	-5.21E-09	-5.52E-09	-5.48E-09	-4.56E-09	-5.24E-09	-4.98E-09	-4.62E-09	-5.26E-09	-5.96E-09	-5.56E-09	-5.55E-09	-5.19E-09	-4.09E-09
-4.91E-09	-4.66E-09	-5.00E-09	-4.86E-09	-4.28E-09	-4.60E-09	-4.61E-09	-4.83E-09	-4.95E-09	-5.55E-09	-4.92E-09	-4.84E-09	-4.63E-09	-3.90E-09
-4.58E-09	-4.09E-09	-4.46E-09	-4.28E-09	-3.48E-09	-3.82E-09	-4.16E-09	-4.24E-09	-4.44E-09	-4.74E-09	-4.44E-09	-4.05E-09	-3.99E-09	-3.55E-09
-4.19E-09	-3.47E-09	-3.93E-09	-3.59E-09	-3.16E-09	-2.97E-09	-3.44E-09	-3.19E-09	-3.89E-09	-3.56E-09	-3.99E-09	-3.38E-09	-3.25E-09	-2.73E-09
-3.69E-09	-3.26E-09	-3.67E-09	-3.34E-09	-3.01E-09	-2.31E-09	-2.30E-09	-2.75E-09	-3.08E-09	-2.49E-09	-2.98E-09	-2.56E-09	-2.72E-09	-1.76E-09
-2.91E-09	-2.90E-09	-3.24E-09	-2.97E-09	-2.86E-09	-1.56E-09	-1.71E-09	-2.61E-09	-2.01E-09	-1.39E-09	-1.62E-09	-1.35E-09	-2.12E-09	-9.59E-10
-2.27E-09	-2.13E-09	-2.29E-09	-2.15E-09	-2.18E-09	-1.18E-09	-1.39E-09	-1.92E-09	-1.07E-09	-3.07E-10	-3.07E-10	-6.12E-10	-1.31E-09	-5.07E-10
-1.32E-09	-1.21E-09	-1.15E-09	-1.23E-09	-1.74E-09	-8.84E-10	-5.63E-10	-9.91E-10	-2.26E-10	6.00E-10	8.23E-10	3.27E-10	-7.34E-10	-3.75E-10
-9.92E-10	-5.41E-10	-5.04E-10	-8.41E-10	-1.05E-09	1.72E-10	1.41E-10	-2.03E-10	-5.52E-11	4.58E-10	1.59E-09	1.10E-09	3.72E-10	3.12E-10
-8.61E-10	-2.37E-10	1.53E-10	-3.76E-10	4.26E-11	1.30E-09	1.10E-09	7.20E-10	9.00E-10	1.23E-09	1.76E-09	1.58E-09	1.42E-09	1.58E-09
-2.04E-10	4.81E-10	1.26E-09	7.44E-11	1.14E-09	1.79E-09	1.95E-09	1.90E-09	1.91E-09	2.51E-09	1.83E-09	1.88E-09	1.93E-09	3.13E-09
1.17E-09	1.58E-09	2.31E-09	1.15E-09	1.94E-09	2.24E-09	2.89E-09	2.88E-09	3.23E-09	3.94E-09	2.67E-09	2.81E-09	3.32E-09	4.34E-09
2.32E-09	2.52E-09	3.21E-09	2.27E-09	3.26E-09	3.55E-09	3.74E-09	3.46E-09	4.25E-09	4.90E-09	3.44E-09	3.81E-09	4.65E-09	5.20E-09
3.34E-09	3.65E-09	4.00E-09	3.38E-09	4.34E-09	4.37E-09	4.43E-09	4.18E-09	4.44E-09	4.61E-09	4.31E-09	5.04E-09	5.75E-09	5.45E-09
4.05E-09	4.42E-09	4.98E-09	4.72E-09	5.69E-09	5.14E-09	5.03E-09	5.24E-09	5.14E-09	5.50E-09	5.52E-09	6.07E-09	6.94E-09	6.34E-09
4.96E-09	5.46E-09	5.82E-09	5.78E-09	6.88E-09	6.13E-09	6.28E-09	6.87E-09	6.62E-09	6.59E-09	7.03E-09	7.50E-09	8.38E-09	7.84E-09
5.72E-09	6.63E-09	6.84E-09	6.77E-09	7.37E-09	7.76E-09	7.87E-09	8.20E-09	7.86E-09	7.38E-09	8.33E-09	8.55E-09	8.96E-09	9.28E-09
6.79E-09	7.37E-09	7.71E-09	8.10E-09	8.29E-09	9.14E-09	8.98E-09	9.18E-09	9.04E-09	8.60E-09	9.73E-09	9.44E-09	9.81E-09	1.07E-08
7.97E-09	8.18E-09	8.60E-09	9.92E-09	9.46E-09	9.90E-09	9.92E-09	9.90E-09	9.75E-09	9.70E-09	1.11E-08	1.05E-08	1.07E-08	1.16E-08
9.84E-09	9.46E-09	1.01E-08	1.13E-08	1.04E-08	1.03E-08	1.07E-08	1.07E-08	1.08E-08	1.10E-08	1.21E-08	1.14E-08	1.17E-08	1.27E-08
1.14E-08	1.08E-08	1.14E-08	1.18E-08	1.10E-08	1.14E-08	1.18E-08	1.20E-08	1.24E-08	1.21E-08	1.28E-08	1.21E-08	1.29E-08	1.36E-08
1.23E-08	1.20E-08	1.30E-08	1.26E-08	1.21E-08	1.23E-08	1.27E-08	1.28E-08	1.39E-08	1.30E-08	1.34E-08	1.29E-08	1.39E-08	1.47E-08
1.33E-08	1.33E-08	1.42E-08	1.38E-08	1.33E-08	1.35E-08	1.39E-08	1.38E-08	1.47E-08	1.45E-08	1.47E-08	1.42E-08	1.49E-08	1.54E-08
1.37E-08	1.43E-08	1.52E-08	1.48E-08	1.50E-08	1.49E-08	1.51E-08	1.48E-08	1.58E-08	1.62E-08	1.62E-08	1.56E-08	1.61E-08	1.66E-08
1.40E-08	1.49E-08	1.58E-08	1.55E-08	1.62E-08	1.61E-08	1.68E-08	1.66E-08	1.69E-08	1.77E-08	1.75E-08	1.71E-08	1.73E-08	1.78E-08
1.52E-08	1.61E-08	1.69E-08	1.68E-08	1.71E-08	1.74E-08	1.76E-08	1.79E-08	1.81E-08	1.88E-08	1.85E-08	1.87E-08	1.86E-08	1.89E-08
1.68E-08	1.75E-08	1.78E-08	1.80E-08	1.85E-08	1.89E-08	1.87E-08	1.94E-08	1.93E-08	1.97E-08	1.98E-08	2.03E-08	1.99E-08	2.00E-08
1.82E-08	1.88E-08	1.87E-08	1.93E-08	2.01E-08	2.05E-08	2.01E-08	2.06E-08	2.03E-08	2.09E-08	2.10E-08	2.13E-08	2.13E-08	2.11E-08
1.94E-08	1.95E-08	1.99E-08	2.07E-08	2.14E-08	2.19E-08	2.16E-08	2.20E-08	2.17E-08	2.23E-08	2.18E-08	2.23E-08	2.27E-08	2.27E-08
2.06E-08	2.05E-08	2.12E-08	2.20E-08	2.27E-08	2.30E-08	2.26E-08	2.33E-08	2.28E-08	2.26E-08	2.30E-08	2.36E-08	2.44E-08	2.47E-08
2.22E-08	2.16E-08	2.26E-08	2.27E-08	2.41E-08	2.39E-08	2.40E-08	2.45E-08	2.42E-08	2.44E-08	2.45E-08	2.52E-08	2.62E-08	2.64E-08
2.39E-08	2.32E-08	2.39E-08	2.40E-08	2.51E-08	2.48E-08	2.57E-08	2.57E-08	2.56E-08	2.58E-08	2.61E-08	2.72E-08	2.79E-08	2.74E-08
2.57E-08	2.46E-08	2.52E-08	2.53E-08	2.63E-08	2.61E-08	2.72E-08	2.72E-08	2.73E-08	2.74E-08	2.78E-08	2.88E-08	2.93E-08	2.84E-08
2.69E-08	2.60E-08	2.66E-08	2.67E-08	2.80E-08	2.75E-08	2.88E-08	2.91E-08	2.93E-08	2.87E-08	2.96E-08	3.04E-08	3.02E-08	3.02E-08
2.83E-08	2.75E-08	2.79E-08	2.80E-08	2.94E-08	2.89E-08	3.03E-08	3.05E-08	3.07E-08	3.01E-08	3.11E-08	3.19E-08	3.14E-08	3.14E-08
2.93E-08	2.88E-08	2.92E-08	2.94E-08	3.10E-08	3.07E-08	3.17E-08	3.18E-08	3.18E-08	3.18E-08	3.26E-08	3.32E-08	3.29E-08	3.27E-08
3.05E-08	3.03E-08	3.05E-08	3.11E-08	3.27E-08	3.30E-08	3.31E-08	3.34E-08	3.28E-08	3.35E-08	3.33E-08	3.42E-08	3.41E-08	3.38E-08
3.21E-08	3.16E-08	3.19E-08	3.29E-08	3.45E-08	3.42E-08	3.47E-08	3.51E-08	3.44E-08	3.50E-08	3.48E-08	3.59E-08	3.54E-08	3.56E-08
3.39E-08	3.37E-08	3.36E-08	3.44E-08	3.53E-08	3.52E-08	3.59E-08	3.66E-08	3.64E-08	3.69E-08	3.65E-08	3.79E-08	3.72E-08	3.72E-08
3.57E-08	3.55E-08	3.57E-08	3.57E-08	3.66E-08	3.68E-08	3.74E-08	3.83E-08	3.85E-08	3.92E-08	3.85E-08	4.00E-08	3.90E-08	3.87E-08
3.68E-08	3.65E-08	3.73E-08	3.73E-08	3.80E-08	3.86E-08	3.94E-08	4.01E-08	4.02E-08	4.16E-08	4.08E-08	4.21E-08	4.11E-08	4.06E-08
3.76E-08	3.79E-08	3.86E-08	3.88E-08	3.95E-08	4.03E-08	4.12E-08	4.23E-08	4.23E-08	4.32E-08	4.29E-08	4.33E-08	4.31E-08	4.26E-08
3.92E-08	3.94E-08	4.02E-08	4.03E-08	4.12E-08	4.21E-08	4.28E-08	4.35E-08	4.42E-08	4.44E-08	4.40E-08	4.43E-08	4.46E-08	4.44E-08
4.09E-08	4.14E-08	4.17E-08	4.17E-08	4.28E-08	4.39E-08	4.44E-08	4.47E-08	4.59E-08	4.53E-08	4.53E-08	4.58E-08	4.61E-08	4.61E-08

5.90E-17	6.53E-17	6.99E-17	7.14E-17	8.07E-17	7.86E-17	7.76E-17	6.57E-17	6.61E-17	8.34E-17	7.79E-17	8.01E-17	6.77E-17	6.06E-17
6.50E-17	7.29E-17	7.49E-17	8.05E-17	8.72E-17	8.17E-17	8.13E-17	7.04E-17	6.76E-17	8.53E-17	8.96E-17	9.80E-17	7.46E-17	7.12E-17
7.18E-17	8.47E-17	8.48E-17	9.22E-17	9.89E-17	8.97E-17	8.08E-17	6.43E-17	6.81E-17	8.66E-17	9.61E-17	1.05E-16	6.83E-17	8.14E-17
7.59E-17	9.97E-17	9.01E-17	9.78E-17	1.05E-16	9.20E-17	7.15E-17	6.28E-17	7.00E-17	9.25E-17	9.84E-17	1.04E-16	5.81E-17	8.98E-17
8.67E-17	1.09E-16	9.73E-17	9.36E-17	1.00E-16	9.61E-17	6.92E-17	6.33E-17	8.72E-17	9.45E-17	9.75E-17	9.67E-17	9.07E-17	1.05E-16
9.91E-17	1.08E-16	1.08E-16	1.00E-16	9.33E-17	1.00E-16	7.29E-17	7.43E-17	9.27E-17	1.02E-16	9.53E-17	9.69E-17	1.26E-16	1.15E-16
9.99E-17	1.11E-16	1.20E-16	1.11E-16	9.41E-17	1.10E-16	9.54E-17	8.73E-17	9.66E-17	1.06E-16	1.03E-16	1.06E-16	1.29E-16	1.19E-16
1.03E-16	1.14E-16	1.18E-16	1.22E-16	1.11E-16	1.10E-16	1.09E-16	1.07E-16	1.16E-16	1.03E-16	1.21E-16	1.16E-16	1.34E-16	1.20E-16
1.04E-16	1.10E-16	1.25E-16	1.23E-16	1.23E-16	1.07E-16	1.11E-16	1.18E-16	1.30E-16	1.05E-16	1.26E-16	1.27E-16	1.41E-16	1.21E-16
1.06E-16	1.09E-16	1.26E-16	1.29E-16	1.20E-16	1.02E-16	1.08E-16	1.17E-16	1.34E-16	1.12E-16	1.27E-16	1.24E-16	1.44E-16	1.22E-16
1.12E-16	1.27E-16	1.34E-16	1.36E-16	1.22E-16	1.06E-16	1.09E-16	1.28E-16	1.31E-16	1.26E-16	1.23E-16	1.14E-16	1.44E-16	1.26E-16
1.31E-16	1.47E-16	1.41E-16	1.36E-16	1.29E-16	1.24E-16	1.26E-16	1.40E-16	1.31E-16	1.27E-16	1.23E-16	1.36E-16	1.52E-16	1.42E-16
1.40E-16	1.49E-16	1.52E-16	1.33E-16	1.37E-16	1.47E-16	1.46E-16	1.45E-16	1.42E-16	1.20E-16	1.30E-16	1.45E-16	1.64E-16	1.49E-16
1.41E-16	1.48E-16	1.54E-16	1.38E-16	1.42E-16	1.59E-16	1.58E-16	1.53E-16	1.51E-16	1.26E-16	1.49E-16	1.57E-16	1.65E-16	1.52E-16
1.37E-16	1.51E-16	1.55E-16	1.42E-16	1.42E-16	1.63E-16	1.58E-16	1.68E-16	1.44E-16	1.40E-16	1.68E-16	1.64E-16	1.67E-16	1.65E-16
1.35E-16	1.60E-16	1.54E-16	1.45E-16	1.49E-16	1.64E-16	1.72E-16	1.79E-16	1.63E-16	1.60E-16	1.94E-16	1.71E-16	1.72E-16	1.77E-16
1.30E-16	1.63E-16	1.51E-16	1.46E-16	1.54E-16	1.63E-16	1.68E-16	1.89E-16	1.81E-16	1.74E-16	2.01E-16	1.80E-16	1.79E-16	1.88E-16
1.34E-16	1.68E-16	1.47E-16	1.51E-16	1.58E-16	1.64E-16	1.65E-16	1.89E-16	1.88E-16	1.90E-16	2.06E-16	1.95E-16	1.95E-16	2.14E-16
1.52E-16	1.88E-16	1.53E-16	1.63E-16	1.67E-16	1.77E-16	1.65E-16	1.86E-16	1.81E-16	1.98E-16	2.09E-16	2.05E-16	2.18E-16	2.18E-16
1.68E-16	1.98E-16	1.59E-16	1.66E-16	1.77E-16	1.88E-16	1.68E-16	1.88E-16	1.78E-16	2.10E-16	2.11E-16	2.21E-16	2.32E-16	2.16E-16
1.76E-16	2.05E-16	1.67E-16	1.75E-16	1.81E-16	1.92E-16	1.86E-16	2.00E-16	1.89E-16	2.22E-16	2.19E-16	2.44E-16	2.45E-16	2.21E-16
1.85E-16	2.01E-16	1.76E-16	1.88E-16	1.94E-16	2.03E-16	2.11E-16	2.31E-16	2.14E-16	2.34E-16	2.27E-16	2.57E-16	2.46E-16	2.21E-16
1.90E-16	1.97E-16	1.84E-16	2.04E-16	2.04E-16	2.17E-16	2.17E-16	2.39E-16	2.24E-16	2.29E-16	2.34E-16	2.62E-16	2.47E-16	2.15E-16
2.00E-16	1.84E-16	1.93E-16	2.17E-16	2.19E-16	2.26E-16	2.25E-16	2.42E-16	2.27E-16	2.22E-16	2.30E-16	2.59E-16	2.53E-16	2.35E-16
2.11E-16	1.87E-16	1.94E-16	2.23E-16	2.32E-16	2.31E-16	2.31E-16	2.45E-16	2.38E-16	2.26E-16	2.37E-16	2.53E-16	2.63E-16	2.56E-16
2.22E-16	1.95E-16	1.94E-16	2.24E-16	2.39E-16	2.23E-16	2.50E-16	2.67E-16	2.47E-16	2.41E-16	2.51E-16	2.50E-16	2.61E-16	2.65E-16
2.26E-16	2.07E-16	1.91E-16	2.24E-16	2.34E-16	2.13E-16	2.63E-16	2.68E-16	2.62E-16	2.60E-16	2.63E-16	2.57E-16	2.64E-16	2.74E-16
2.21E-16	2.14E-16	1.92E-16	2.35E-16	2.40E-16	2.29E-16	2.61E-16	2.72E-16	2.64E-16	2.70E-16	2.59E-16	2.68E-16	2.71E-16	2.75E-16
2.09E-16	2.30E-16	2.02E-16	2.34E-16	2.42E-16	2.45E-16	2.51E-16	2.63E-16	2.47E-16	2.73E-16	2.64E-16	2.67E-16	2.73E-16	2.82E-16
1.97E-16	2.38E-16	2.08E-16	2.19E-16	2.38E-16	2.46E-16	2.50E-16	2.54E-16	2.44E-16	2.76E-16	2.67E-16	2.80E-16	2.90E-16	3.02E-16
2.09E-16	2.36E-16	2.31E-16	2.20E-16	2.38E-16	2.40E-16	2.40E-16	2.55E-16	2.77E-16	2.75E-16	2.66E-16	2.88E-16	3.06E-16	3.12E-16

2.15E-16	2.28E-16	2.30E-16	2.28E-16	2.37E-16	2.27E-16	2.40E-16	2.71E-16	2.77E-16	2.82E-16	2.84E-16	3.01E-16	3.14E-16	3.27E-16
2.13E-16	2.22E-16	2.33E-16	2.29E-16	2.36E-16	2.34E-16	2.45E-16	2.89E-16	2.70E-16	3.02E-16	3.02E-16	3.10E-16	3.16E-16	3.26E-16
2.21E-16	2.12E-16	2.34E-16	2.34E-16	2.37E-16	2.41E-16	2.52E-16	2.83E-16	2.54E-16	3.10E-16	3.13E-16	3.24E-16	3.23E-16	3.10E-16
2.24E-16	2.24E-16	2.37E-16	2.40E-16	2.31E-16	2.46E-16	2.60E-16	2.71E-16	2.69E-16	3.10E-16	3.09E-16	3.18E-16	3.26E-16	2.99E-16
2.28E-16	2.12E-16	2.36E-16	2.48E-16	2.18E-16	2.55E-16	2.59E-16	2.68E-16	2.87E-16	3.08E-16	3.05E-16	3.07E-16	3.30E-16	2.98E-16
2.50E-16	2.24E-16	2.47E-16	2.59E-16	2.27E-16	2.49E-16	2.52E-16	2.64E-16	2.90E-16	3.08E-16	3.07E-16	3.19E-16	3.29E-16	3.03E-16
2.39E-16	2.25E-16	2.55E-16	2.62E-16	2.41E-16	2.71E-16	2.78E-16	2.67E-16	2.80E-16	3.11E-16	3.09E-16	3.16E-16	3.21E-16	2.96E-16
2.21E-16	2.45E-16	2.70E-16	2.60E-16	2.44E-16	2.68E-16	2.83E-16	2.79E-16	3.14E-16	3.28E-16	3.27E-16	3.27E-16	3.23E-16	3.14E-16
2.07E-16	2.34E-16	2.63E-16	2.57E-16	2.63E-16	2.65E-16	2.63E-16	2.71E-16	3.21E-16	3.21E-16	3.25E-16	3.28E-16	3.13E-16	3.39E-16
2.07E-16	2.31E-16	2.54E-16	2.58E-16	2.77E-16	2.67E-16	2.48E-16	2.62E-16	3.19E-16	3.23E-16	3.31E-16	3.22E-16	3.07E-16	3.47E-16
2.26E-16	2.41E-16	2.54E-16	2.50E-16	2.81E-16	2.54E-16	2.61E-16	2.77E-16	3.06E-16	3.11E-16	3.22E-16	3.20E-16	3.16E-16	3.56E-16
2.19E-16	2.48E-16	2.59E-16	2.67E-16	2.70E-16	2.54E-16	2.74E-16	2.86E-16	2.97E-16	3.08E-16	3.21E-16	3.21E-16	3.11E-16	3.41E-16
2.03E-16	2.52E-16	2.47E-16	2.83E-16	2.78E-16	2.66E-16	2.76E-16	2.90E-16	3.00E-16	3.10E-16	3.12E-16	3.08E-16	3.18E-16	3.39E-16
2.01E-16	2.60E-16	2.48E-16	2.66E-16	2.74E-16	2.61E-16	2.81E-16	2.94E-16	2.85E-16	2.98E-16	3.11E-16	3.12E-16	3.32E-16	3.36E-16
2.31E-16	2.55E-16	2.54E-16	2.54E-16	2.68E-16	2.65E-16	2.77E-16	2.84E-16	2.83E-16	2.79E-16	3.06E-16	3.05E-16	3.19E-16	3.38E-16
2.36E-16	2.41E-16	2.37E-16	2.31E-16	2.56E-16	2.57E-16	2.68E-16	2.73E-16	2.74E-16	2.73E-16	3.02E-16	2.92E-16	2.99E-16	3.28E-16
2.38E-16	2.41E-16	2.22E-16	2.11E-16	2.43E-16	2.36E-16	2.59E-16	2.74E-16	2.68E-16	2.69E-16	2.95E-16	2.76E-16	2.85E-16	3.28E-16
2.39E-16	2.40E-16	2.40E-16	2.14E-16	2.30E-16	2.20E-16	2.47E-16	2.60E-16	2.73E-16	2.78E-16	2.86E-16	2.63E-16	2.79E-16	3.16E-16
2.48E-16	2.46E-16	2.60E-16	2.27E-16	2.24E-16	2.20E-16	2.38E-16	2.41E-16	2.65E-16	2.88E-16	2.86E-16	2.72E-16	2.90E-16	3.07E-16
2.43E-16	2.36E-16	2.57E-16	2.25E-16	2.29E-16	2.32E-16	2.51E-16	2.57E-16	2.60E-16	2.78E-16	2.75E-16	2.91E-16	2.92E-16	3.07E-16
2.41E-16	2.24E-16	2.40E-16	2.24E-16	2.22E-16	2.39E-16	2.54E-16	2.67E-16	2.54E-16	2.76E-16	2.78E-16	3.07E-16	2.99E-16	3.14E-16
2.25E-16	2.10E-16	2.18E-16	2.12E-16	2.16E-16	2.36E-16	2.50E-16	2.68E-16	2.44E-16	2.58E-16	2.56E-16	3.00E-16	2.98E-16	3.14E-16
2.13E-16	1.83E-16	1.99E-16	2.01E-16	2.13E-16	2.24E-16	2.55E-16	2.66E-16	2.41E-16	2.48E-16	2.45E-16	2.92E-16	3.02E-16	3.17E-16
1.99E-16	1.57E-16	2.02E-16	1.88E-16	2.12E-16	2.22E-16	2.40E-16	2.45E-16	2.35E-16	2.40E-16	2.60E-16	2.96E-16	2.88E-16	3.00E-16
1.88E-16	1.24E-16	2.24E-16	1.60E-16	1.91E-16	2.11E-16	2.21E-16	2.27E-16	2.29E-16	2.42E-16	2.72E-16	2.84E-16	2.56E-16	2.76E-16
1.68E-16	1.06E-16	2.23E-16	1.30E-16	1.88E-16	2.17E-16	2.19E-16	2.17E-16	2.36E-16	2.44E-16	2.58E-16	2.61E-16	2.34E-16	2.50E-16
1.64E-16	1.49E-16	2.19E-16	1.22E-16	1.77E-16	2.12E-16	2.16E-16	2.25E-16	2.45E-16	2.54E-16	2.48E-16	2.59E-16	2.18E-16	2.32E-16
1.63E-16	1.78E-16	2.01E-16	1.82E-16	1.89E-16	2.07E-16	2.14E-16	2.26E-16	2.37E-16	2.50E-16	2.40E-16	2.56E-16	2.20E-16	2.46E-16
1.44E-16	1.67E-16	1.85E-16	1.80E-16	1.79E-16	1.98E-16	2.18E-16	2.12E-16	2.24E-16	2.43E-16	2.22E-16	2.42E-16	2.29E-16	2.40E-16
1.18E-16	1.40E-16	1.58E-16	1.80E-16	1.69E-16	1.88E-16	2.10E-16	1.94E-16	2.02E-16	2.29E-16	2.04E-16	2.15E-16	2.30E-16	2.24E-16
9.56E-17	1.19E-16	1.36E-16	1.71E-16	1.58E-16	1.74E-16	2.02E-16	1.84E-16	1.82E-16	2.04E-16	1.74E-16	1.84E-16	2.19E-16	2.27E-16
8.01E-17	9.35E-17	1.20E-16	1.54E-16	1.43E-16	1.61E-16	1.88E-16	1.79E-16	1.78E-16	1.78E-16	1.56E-16	1.71E-16	2.02E-16	2.29E-16
5.95E-17	6.91E-17	1.00E-16	1.28E-16	1.31E-16	1.49E-16	1.83E-16	1.86E-16	1.81E-16	1.63E-16	1.60E-16	1.85E-16	1.94E-16	2.10E-16
2.54E-17	5.31E-17	7.86E-17	1.07E-16	1.21E-16	1.38E-16	1.65E-16	1.84E-16	1.78E-16	1.67E-16	1.76E-16	1.82E-16	1.87E-16	1.93E-16
9.61E-18	3.51E-17	5.56E-17	9.23E-17	1.19E-16	1.35E-16	1.58E-16	1.83E-16	1.64E-16	1.65E-16	1.89E-16	1.90E-16	1.81E-16	1.85E-16
9.43E-18	2.11E-17	3.51E-17	6.59E-17	1.01E-16	1.32E-16	1.48E-16	1.60E-16	1.43E-16	1.50E-16	1.79E-16	1.73E-16	1.61E-16	1.68E-16
1.48E-17	1.24E-17	1.33E-17	3.78E-17	8.10E-17	1.21E-16	1.30E-16	1.44E-16	1.35E-16	1.38E-16	1.65E-16	1.52E-16	1.41E-16	1.58E-16
1.53E-17	8.32E-18	3.49E-18	2.17E-17	6.15E-17	1.04E-16	1.14E-16	1.27E-16	1.29E-16	1.36E-16	1.41E-16	1.36E-16	1.15E-16	1.45E-16
2.45E-17	1.52E-17	2.76E-18	1.07E-17	4.40E-17	8.05E-17	9.76E-17	1.01E-16	1.05E-16	1.08E-16	9.62E-17	9.91E-17	9.11E-17	1.60E-16
3.62E-17	1.84E-17	3.66E-18	5.45E-18	3.44E-17	5.38E-17	7.62E-17	7.68E-17	7.66E-17	6.87E-17	4.60E-17	5.28E-17	7.21E-17	1.49E-16
4.52E-17	2.33E-17	1.01E-17	9.15E-18	2.81E-17	3.85E-17	6.13E-17	5.67E-17	5.06E-17	3.40E-17	1.43E-17	2.43E-17	5.82E-17	4.02E-17
5.39E-17	3.24E-17	1.89E-17	2.03E-17	3.65E-17	3.53E-17	4.82E-17	4.24E-17	3.92E-17	2.19E-17	9.31E-18	1.42E-17	2.50E-17	3.18E-18
5.83E-17	3.64E-17	2.90E-17	2.60E-17	4.78E-17	3.29E-17	4.23E-17	4.16E-17	4.37E-17	3.27E-17	2.23E-17	2.96E-17	4.51E-17	5.63E-17
5.47E-17	4.11E-17	3.87E-17	3.64E-17	5.42E-17	3.61E-17	4.45E-17	4.41E-17	5.15E-17	4.17E-17	4.66E-17	4.95E-17	6.04E-17	6.75E-17
4.97E-17	3.28E-17	4.21E-17	4.38E-17	5.28E-17	4.08E-17	5.01E-17	4.59E-17	5.47E-17	4.93E-17	5.59E-17	5.69E-17	6.74E-17	7.21E-17
4.42E-17	2.56E-17	4.34E-17	4.66E-17	5.26E-17	4.18E-17	5.42E-17	5.04E-17	5.42E-17	4.63E-17	5.07E-17	5.28E-17	5.95E-17	7.36E-17
4.09E-17	2.76E-17	4.92E-17	5.35E-17	5.13E-17	3.88E-17	5.16E-17	4.90E-17	5.49E-17	4.31E-17	4.43E-17	4.45E-17	5.01E-17	6.36E-17
3.63E-17	3.93E-17	5.49E-17	5.34E-17	5.22E-17	4.19E-17	4.55E-17	4.97E-17	5.29E-17	3.81E-17	4.01E-17	4.22E-17	4.63E-17	5.63E-17
3.99E-17	4.73E-17	5.57E-17	5.29E-17	5.30E-17	4.61E-17	3.50E-17	4.33E-17	4.71E-17	3.77E-17	4.19E-17	4.48E-17	4.82E-17	4.95E-17
4.03E-17	4.55E-17	4.89E-17	4.83E-17	4.51E-17	4.76E-17	3.26E-17	3.82E-17	4.24E-17	4.21E-17	4.46E-17	4.51E-17	4.70E-17	4.40E-17
3.79E-17	4.08E-17	4.25E-17	3.81E-17	3.40E-17	4.35E-17	3.43E-17	3.24E-17	4.08E-17	4.44E-17	4.33E-17	4.78E-17	4.38E-17	3.84E-17
3.73E-17	4.12E-17	3.70E-17	2.99E-17	2.78E-17	4.12E-17	3.32E-17	3.00E-17	3.58E-17	3.88E-17	3.82E-17	4.44E-17	4.09E-17	3.27E-17
2.88E-17	3.44E-17	3.28E-17	2.81E-17	2.42E-17	3.56E-17	2.94E-17	2.33E-17	3.07E-17	3.53E-17	3.43E-17	3.89E-17	3.62E-17	2.29E-17
2.61E-17	2.71E-17	3.04E-17	3.00E-17	2.08E-17	2.75E-17	2.48E-17	2.13E-17	2.77E-17	3.55E-17	3.09E-17	3.08E-17	2.69E-17	1.68E-17
2.41E-17	2.17E-17	2.50E-17	2.37E-17	1.84E-17	2.12E-17	2.12E-17	2.34E-17	2.45E-17	3.08E-17	2.42E-17	2.34E-17	2.15E-17	1.52E-17
2.10E-17	1.67E-17	1.99E-17	1.83E-17	1.21E-17	1.46E-17	1.73E-17	1.80E-17	1.97E-17	2.24E-17	1.97E-17	1.64E-17	1.59E-17	1.26E-17
1.76E-17	1.20E-17	1.54E-17	1.29E-17	9.97E-18	8.80E-18	1.18E-17	1.02E-17	1.51E-17	1.27E-17	1.59E-17	1.14E-17	1.06E-17	7.48E-18
1.36E-17	1.06E-17	1.35E-17	1.12E-17	9.09E-18	5.34E-18	5.28E-18	7.56E-18	9.48E-18	6.20E-18	8.86E-18	6.57E-18	7.40E-18	3.11E-18
8.47E-18	8.39E-18	1.05E-17	8.84E-18	8.17E-18	2.42E-18	2.92E-18	6.83E-18	4.04E-18	1.94E-18	2.62E-18	1.83E-18	4.48E-18	9.20E-19
5.14E-18	4.54E-18	5.22E-18	4.64E-18	4.74E-18	1.40E-18	1.94E-18	3.67E-18	1.14E-18	9.45E-20	9.45E-20	3.75E-19	1.70E-18	2.57E-19
1.74E-18	1.46E-18	1.32E-18	1.50E-18	3.03E-18	7.82E-19	3.17E-19	9.82E-19	5.09E-20	3.60E-19	6.77E-19	1.07E-19	5.39E-19	1.41E-19
9.85E-19	2.93E-19	2.54E-19	7.07E-19	1.09E-18	2.96E-20	2.00E-20	4.11E-20	3.04E-21	2.10E-19	2.54E-18	1.21E-18	1.38E-19	9.74E-20
7.42E-19	5.62E-20	2.34E-20	1.41E-19	1.82E-21	1.68E-18	1.21E-18	5.18E-19	8.10E-19	1.51E-18	3.10E-18	2.51E-18	2.01E-18	2.49E-18
4.15E-20	2.31E-19	1.58E-18	5.53E-21	1.29E-18	3.21E-18	3.80E-18	3.63E-18	3.64E-18	6.32E-18	3.36E-18	3.55E-18	3.72E-18	9.78E-18
1.36E-18	2.50E-18	5.31E-18	1.32E-18	3.76E-18	5.03E-18	8.35E-18	8.27E-18	1.05E-17	1.56E-17	7.12E-18	7.89E-18	1.10E-17	1.88E-17
5.36E-18	6.35E-18	1.03E-17	5.15E-18	1.06E-17	1.26E-17	1.40E-17	1.20E-17	1.81E-17	2.40E-17	1.18E-17	1.45E-17	2.16E-17	2.71E-17
1.11E-17	1.33E-17	1.60E-17	1.14E-17	1.88E-17	1.91E-17	1.96E-17	1.75E-17	1.97E-17	2.12E-17	1.86E-17	2.54E-17	3.30E-17	2.97E-17
1.64E-17	1.95E-17	2.48E-17	2.22E-17	3.24E-17	2.64E-17	2.53E-17	2.74E-17	2.64E-17	3.02E-17	3.05E-17	3.68E-17	4.81E-17	4.02E-17
2.46E-17	2.98E-17	3.38E-17	3.34E-17	4.73E-17	3.75E-17	3.95E-17	4.72E-17	4.39E-17	4.35E-17	4.94E-17	5.63E-17	7.01E-17	6.14E-17
3.27E-17	4.39E-17	4.68E-17	4.58E-17	5.44E-17	6.02E-17	6.19E-17	6.72E-17	6.17E-17	5.44E-17	6.94E-17	7.31E-17	8.03E-17	8.61E-17
4.61E-17	5.43E-17	5.95E-17	6.56E-17	6.87E-17	8.36E-17	8.07E-17	8.43E-17	8.16E-17	7.39E-17	9.46E-17	8.91E-17	9.63E-17	1.15E-16
6.36E-17	6.69E-17	7.40E-17	9.85E-17	8.95E-17	9.79E-17	9.84E-17	9.79E-17	9.51E-17	9.40E-17	1.23E-16	1.09E-16	1.14E-16	1.34E-16
9.69E-17	8.96E-17	1.02E-16	1.27E-16	1.08E-16	1.06E-16	1.14E-16	1.15E-16	1.17E-16	1.20E-16	1.47E-16	1.30E-16	1.37E-16	1.61E-16
1.30E-16	1.17E-16	1.29E-16	1.40E-16	1.20E-16	1.29E-16	1.39E-16	1.44E-16	1.55E-16	1.46E-16	1.63E-16	1.46E-16	1.67E-16	1.86E-16
1.51E-16	1.44E-16	1.68E-16	1.58E-16	1.45E-16	1.52E-16	1.63E-16	1.64E-16	1.92E-16	1.69E-16	1.80E-16	1.66E-16	1.94E-16	2.15E-16
1.76E-16	1.77E-16	2.01E-16	1.90E-16	1.78E-16	1.84E-16	1.94E-16	1.90E-16	2.17E-16	2.10E-16	2.17E-16	2.01E-16	2.22E-16	2.36E-16
1.87E-16	2.06E-16	2.31E-16	2.19E-16	2.26E-16	2.23E-16	2.27E-16	2.19E-16	2.48E-16	2.62E-16	2.62E-16	2.42E-16	2.60E-16	2.76E-16
1.95E-16	2.22E-16	2.49E-16	2.40E-16	2.61E-16	2.60E-16	2.83E-16	2.74E-16	2.86E-16	3.12E-16	3.08E-16	2.92E-16	3.01E-16	3.16E-16
2.32E-16	2.59E-16	2.85E-16	2.82E-16	2.94E-16	3.04E-16	3.09E-16	3.20E-16	3.27E-16	3.53E-16	3.43E-16	3.49E-16	3.45E-16	3.58E-16
2.82E-16	3.05E-16	3.17E-16	3.23E-16	3.41E-16	3.56E-16	3.49E-16	3.75E-16	3.72E-16	3.88E-16	3.91E-16	4.10E-16	3.95E-16	3.99E-16
3.30E-16	3.53E-16	3.51E-16	3.72E-16	4.03E-16	4.22E-16	4.05E-16	4.25E-16	4.13E-16	4.36E-16	4.40E-16	4.54E-16	4.55E-16	4.47E-16
3.75E-16	3.81E-16	3.98E-16	4.30E-16	4.59E-16	4.80E-16	4.69E-16	4.85E-16	4.72E-16	4.95E-16	4.77E-16	4.97E-16	5.14E-16	5.17E-16
4.25E-16	4.21E-16	4.48E-16	4.83E-16	5.16E-16	5.28E-16	5.12E-16	5.45E-16	5.22E-16	5.12E-16	5.29E-16	5.59E-16	5.97E-16	6.10E-16
4.93E-16	4.66E-16	5.10E-16	5.15E-16	5.82E-16	5.71E-16	5.75E-16	6.03E-16	5.85E-16	5.94E-16	6.00E-16	6.33E-16	6.87E-16	6.99E-16
5.72E-16	5.39E-16	5.69E-16	5.78E-16	6.31E-16	6.13E-16	6.62E-16	6.58E-16	6.54E-16	6.66E-16	6.80E-16	7.40E-16	7.81E-16	7.51E-16
6.60E-16	6.04E-16	6.34E-16	6.42E-16	6.93E-16	6.82E-16	7.43E-16	7.41E-16	7.46E-16	7.49E-16	7.71E-16	8.29E-16	8.61E-16	8.09E-16

7.25E-16	6.75E-16	7.08E-16	7.11E-16	7.84E-16	7.59E-16	8.28E-16	8.47E-16	8.56E-16	8.25E-16	8.76E-16	9.24E-16	9.10E-16	9.10E-16
8.03E-16	7.56E-16	7.81E-16	7.86E-16	8.65E-16	8.36E-16	9.20E-16	9.29E-16	9.45E-16	9.05E-16	9.69E-16	1.02E-15	9.87E-16	9.88E-16
8.58E-16	8.28E-16	8.55E-16	8.66E-16	9.59E-16	9.45E-16	1.01E-15	1.01E-15	1.01E-15	1.01E-15	1.06E-15	1.10E-15	1.08E-15	1.07E-15
9.28E-16	9.15E-16	9.31E-16	9.70E-16	1.07E-15	1.09E-15	1.09E-15	1.11E-15	1.07E-15	1.12E-15	1.11E-15	1.17E-15	1.16E-15	1.14E-15
1.03E-15	9.98E-16	1.02E-15	1.08E-15	1.19E-15	1.17E-15	1.20E-15	1.23E-15	1.18E-15	1.22E-15	1.21E-15	1.29E-15	1.25E-15	1.27E-15
1.15E-15	1.13E-15	1.13E-15	1.18E-15	1.24E-15	1.24E-15	1.29E-15	1.34E-15	1.33E-15	1.36E-15	1.33E-15	1.44E-15	1.38E-15	1.38E-15
1.27E-15	1.26E-15	1.27E-15	1.27E-15	1.34E-15	1.35E-15	1.40E-15	1.47E-15	1.48E-15	1.54E-15	1.48E-15	1.60E-15	1.52E-15	1.50E-15
1.35E-15	1.33E-15	1.39E-15	1.39E-15	1.45E-15	1.49E-15	1.55E-15	1.61E-15	1.61E-15	1.73E-15	1.66E-15	1.77E-15	1.69E-15	1.65E-15
1.41E-15	1.43E-15	1.49E-15	1.50E-15	1.56E-15	1.63E-15	1.70E-15	1.79E-15	1.79E-15	1.87E-15	1.84E-15	1.87E-15	1.86E-15	1.81E-15
1.54E-15	1.55E-15	1.62E-15	1.62E-15	1.70E-15	1.77E-15	1.83E-15	1.89E-15	1.95E-15	1.97E-15	1.94E-15	1.97E-15	1.99E-15	1.98E-15
1.67E-15	1.71E-15	1.74E-15	1.74E-15	1.83E-15	1.93E-15	1.97E-15	2.00E-15	2.11E-15	2.05E-15	2.05E-15	2.10E-15	2.12E-15	2.12E-15
2.91E-14	2.94E-14	3.06E-14	3.10E-14	3.28E-14	3.35E-14	3.49E-14	3.60E-14	3.64E-14	3.72E-14	3.76E-14	3.89E-14	3.92E-14	3.96E-14

-8.00E-09	-8.37E-09	-8.64E-09	-9.40E-09	-8.82E-09	-9.89E-09	-8.97E-09	-8.98E-09	-9.47E-09	-9.80E-09	-9.34E-09	-9.51E-09	-9.37E-09	-8.55E-09
-8.55E-09	-9.19E-09	-8.94E-09	-1.02E-08	-9.87E-09	-9.95E-09	-9.05E-09	-9.23E-09	-1.01E-08	-1.02E-08	-9.65E-09	-1.03E-08	-9.58E-09	-9.29E-09
-9.64E-09	-9.99E-09	-9.86E-09	-1.07E-08	-1.05E-08	-9.95E-09	-9.08E-09	-9.87E-09	-1.06E-08	-1.02E-08	-9.83E-09	-1.06E-08	-9.42E-09	-9.72E-09
-1.01E-08	-1.05E-08	-1.06E-08	-1.08E-08	-1.08E-08	-1.05E-08	-9.88E-09	-1.06E-08	-1.07E-08	-1.03E-08	-9.91E-09	-1.07E-08	-9.37E-09	-9.37E-09
-1.01E-08	-1.07E-08	-1.07E-08	-1.10E-08	-1.08E-08	-1.14E-08	-1.08E-08	-1.10E-08	-1.06E-08	-1.08E-08	-1.02E-08	-1.10E-08	-1.00E-08	-9.84E-09
-1.05E-08	-1.09E-08	-1.10E-08	-1.10E-08	-1.11E-08	-1.19E-08	-1.13E-08	-1.13E-08	-1.10E-08	-1.16E-08	-1.12E-08	-1.17E-08	-1.06E-08	-1.10E-08
-1.08E-08	-1.12E-08	-1.11E-08	-1.15E-08	-1.11E-08	-1.21E-08	-1.17E-08	-1.14E-08	-1.12E-08	-1.17E-08	-1.19E-08	-1.21E-08	-1.15E-08	-1.20E-08
-1.10E-08	-1.13E-08	-1.15E-08	-1.20E-08	-1.14E-08	-1.23E-08	-1.20E-08	-1.15E-08	-1.10E-08	-1.19E-08	-1.24E-08	-1.24E-08	-1.20E-08	-1.27E-08
-1.11E-08	-1.18E-08	-1.27E-08	-1.28E-08	-1.16E-08	-1.23E-08	-1.25E-08	-1.16E-08	-1.13E-08	-1.21E-08	-1.25E-08	-1.21E-08	-1.21E-08	-1.30E-08
-1.10E-08	-1.19E-08	-1.33E-08	-1.29E-08	-1.19E-08	-1.25E-08	-1.31E-08	-1.22E-08	-1.19E-08	-1.25E-08	-1.26E-08	-1.21E-08	-1.25E-08	-1.30E-08
-1.16E-08	-1.20E-08	-1.27E-08	-1.26E-08	-1.24E-08	-1.28E-08	-1.34E-08	-1.30E-08	-1.21E-08	-1.28E-08	-1.28E-08	-1.25E-08	-1.28E-08	-1.32E-08
-1.22E-08	-1.21E-08	-1.25E-08	-1.26E-08	-1.30E-08	-1.33E-08	-1.37E-08	-1.34E-08	-1.26E-08	-1.32E-08	-1.30E-08	-1.32E-08	-1.33E-08	-1.36E-08
-1.24E-08	-1.22E-08	-1.27E-08	-1.28E-08	-1.32E-08	-1.32E-08	-1.39E-08	-1.39E-08	-1.33E-08	-1.35E-08	-1.32E-08	-1.34E-08	-1.35E-08	-1.40E-08
-1.27E-08	-1.27E-08	-1.33E-08	-1.33E-08	-1.33E-08	-1.34E-08	-1.41E-08	-1.41E-08	-1.38E-08	-1.38E-08	-1.35E-08	-1.37E-08	-1.37E-08	-1.43E-08
-1.29E-08	-1.34E-08	-1.38E-08	-1.39E-08	-1.36E-08	-1.35E-08	-1.43E-08	-1.44E-08	-1.40E-08	-1.44E-08	-1.39E-08	-1.40E-08	-1.42E-08	-1.46E-08
-1.37E-08	-1.43E-08	-1.43E-08	-1.43E-08	-1.40E-08	-1.34E-08	-1.43E-08	-1.45E-08	-1.44E-08	-1.49E-08	-1.39E-08	-1.44E-08	-1.46E-08	-1.49E-08
-1.45E-08	-1.52E-08	-1.45E-08	-1.47E-08	-1.44E-08	-1.39E-08	-1.45E-08	-1.50E-08	-1.45E-08	-1.50E-08	-1.41E-08	-1.49E-08	-1.48E-08	-1.51E-08
-1.50E-08	-1.55E-08	-1.47E-08	-1.48E-08	-1.43E-08	-1.46E-08	-1.49E-08	-1.54E-08	-1.49E-08	-1.49E-08	-1.45E-08	-1.49E-08	-1.50E-08	-1.52E-08
-1.46E-08	-1.58E-08	-1.48E-08	-1.51E-08	-1.50E-08	-1.51E-08	-1.53E-08	-1.58E-08	-1.54E-08	-1.48E-08	-1.48E-08	-1.54E-08	-1.54E-08	-1.52E-08
-1.44E-08	-1.59E-08	-1.49E-08	-1.50E-08	-1.53E-08	-1.54E-08	-1.56E-08	-1.61E-08	-1.58E-08	-1.48E-08	-1.51E-08	-1.59E-08	-1.55E-08	-1.53E-08
-1.48E-08	-1.54E-08	-1.50E-08	-1.50E-08	-1.58E-08	-1.59E-08	-1.61E-08	-1.62E-08	-1.57E-08	-1.51E-08	-1.55E-08	-1.61E-08	-1.61E-08	-1.58E-08
-1.51E-08	-1.49E-08	-1.47E-08	-1.54E-08	-1.58E-08	-1.57E-08	-1.62E-08	-1.62E-08	-1.58E-08	-1.59E-08	-1.57E-08	-1.64E-08	-1.65E-08	-1.63E-08
-1.49E-08	-1.48E-08	-1.50E-08	-1.53E-08	-1.64E-08	-1.57E-08	-1.64E-08	-1.66E-08	-1.60E-08	-1.65E-08	-1.61E-08	-1.65E-08	-1.68E-08	-1.63E-08
-1.50E-08	-1.53E-08	-1.59E-08	-1.59E-08	-1.68E-08	-1.59E-08	-1.65E-08	-1.69E-08	-1.66E-08	-1.71E-08	-1.67E-08	-1.67E-08	-1.73E-08	-1.62E-08
-1.59E-08	-1.61E-08	-1.65E-08	-1.57E-08	-1.67E-08	-1.60E-08	-1.66E-08	-1.68E-08	-1.66E-08	-1.70E-08	-1.69E-08	-1.70E-08	-1.78E-08	-1.64E-08
-1.69E-08	-1.70E-08	-1.69E-08	-1.57E-08	-1.68E-08	-1.60E-08	-1.71E-08	-1.65E-08	-1.61E-08	-1.69E-08	-1.67E-08	-1.70E-08	-1.83E-08	-1.71E-08
-1.73E-08	-1.73E-08	-1.71E-08	-1.66E-08	-1.71E-08	-1.62E-08	-1.80E-08	-1.63E-08	-1.59E-08	-1.71E-08	-1.70E-08	-1.71E-08	-1.84E-08	-1.78E-08
-1.71E-08	-1.74E-08	-1.70E-08	-1.66E-08	-1.72E-08	-1.67E-08	-1.83E-08	-1.67E-08	-1.65E-08	-1.72E-08	-1.72E-08	-1.72E-08	-1.82E-08	-1.85E-08
-1.69E-08	-1.75E-08	-1.71E-08	-1.76E-08	-1.77E-08	-1.71E-08	-1.85E-08	-1.72E-08	-1.74E-08	-1.74E-08	-1.76E-08	-1.76E-08	-1.86E-08	-1.91E-08
-1.74E-08	-1.77E-08	-1.77E-08	-1.84E-08	-1.78E-08	-1.73E-08	-1.85E-08	-1.83E-08	-1.88E-08	-1.82E-08	-1.86E-08	-1.83E-08	-1.96E-08	-1.93E-08
-1.81E-08	-1.78E-08	-1.80E-08	-1.86E-08	-1.79E-08	-1.75E-08	-1.85E-08	-1.85E-08	-1.96E-08	-1.89E-08	-1.95E-08	-1.86E-08	-1.99E-08	-1.93E-08
-1.87E-08	-1.77E-08	-1.82E-08	-1.89E-08	-1.83E-08	-1.80E-08	-1.83E-08	-1.86E-08	-1.96E-08	-1.93E-08	-2.00E-08	-1.90E-08	-2.01E-08	-1.94E-08
-1.88E-08	-1.76E-08	-1.82E-08	-1.90E-08	-1.85E-08	-1.85E-08	-1.83E-08	-1.89E-08	-1.99E-08	-1.90E-08	-2.00E-08	-1.95E-08	-2.02E-08	-1.95E-08
-1.85E-08	-1.71E-08	-1.83E-08	-1.90E-08	-1.85E-08	-1.83E-08	-1.84E-08	-1.91E-08	-2.00E-08	-1.91E-08	-1.96E-08	-1.95E-08	-2.02E-08	-1.94E-08
-1.80E-08	-1.72E-08	-1.83E-08	-1.86E-08	-1.87E-08	-1.79E-08	-1.86E-08	-1.88E-08	-1.98E-08	-1.91E-08	-1.91E-08	-1.94E-08	-1.98E-08	-1.93E-08
-1.80E-08	-1.74E-08	-1.85E-08	-1.85E-08	-1.83E-08	-1.77E-08	-1.86E-08	-1.85E-08	-1.96E-08	-1.95E-08	-1.88E-08	-1.92E-08	-1.95E-08	-1.91E-08
-1.75E-08	-1.75E-08	-1.85E-08	-1.84E-08	-1.84E-08	-1.80E-08	-1.80E-08	-1.87E-08	-1.95E-08	-1.97E-08	-1.91E-08	-1.96E-08	-1.97E-08	-1.93E-08
-1.74E-08	-1.77E-08	-1.83E-08	-1.80E-08	-1.83E-08	-1.81E-08	-1.78E-08	-1.91E-08	-1.99E-08	-2.01E-08	-2.01E-08	-2.09E-08	-2.08E-08	-2.03E-08
-1.73E-08	-1.80E-08	-1.84E-08	-1.79E-08	-1.80E-08	-1.84E-08	-1.83E-08	-1.93E-08	-2.00E-08	-2.02E-08	-1.97E-08	-2.12E-08	-2.10E-08	-2.16E-08
-1.78E-08	-1.84E-08	-1.89E-08	-1.82E-08	-1.83E-08	-1.87E-08	-1.89E-08	-1.92E-08	-1.97E-08	-2.02E-08	-1.90E-08	-2.09E-08	-2.07E-08	-2.16E-08
-1.77E-08	-1.87E-08	-1.94E-08	-1.89E-08	-1.92E-08	-1.92E-08	-1.94E-08	-1.91E-08	-1.94E-08	-2.03E-08	-2.00E-08	-2.06E-08	-2.03E-08	-2.11E-08
-1.82E-08	-1.90E-08	-1.92E-08	-1.92E-08	-1.90E-08	-1.91E-08	-1.94E-08	-1.94E-08	-1.98E-08	-2.08E-08	-2.06E-08	-2.00E-08	-2.01E-08	-2.09E-08
-1.80E-08	-1.90E-08	-1.86E-08	-1.91E-08	-1.89E-08	-1.94E-08	-1.93E-08	-1.91E-08	-1.92E-08	-2.04E-08	-2.01E-08	-2.00E-08	-2.04E-08	-2.13E-08
-1.77E-08	-1.88E-08	-1.83E-08	-1.88E-08	-1.83E-08	-1.98E-08	-1.94E-08	-1.91E-08	-1.95E-08	-2.04E-08	-2.02E-08	-2.02E-08	-2.05E-08	-2.10E-08
-1.79E-08	-1.86E-08	-1.90E-08	-1.87E-08	-1.81E-08	-1.99E-08	-1.94E-08	-1.94E-08	-1.98E-08	-2.03E-08	-2.01E-08	-2.02E-08	-2.03E-08	-2.09E-08
-1.81E-08	-1.85E-08	-1.88E-08	-1.88E-08	-1.83E-08	-1.96E-08	-1.88E-08	-1.92E-08	-1.98E-08	-1.99E-08	-2.03E-08	-2.07E-08	-2.07E-08	-2.10E-08
-1.78E-08	-1.79E-08	-1.86E-08	-1.87E-08	-1.89E-08	-1.96E-08	-1.86E-08	-1.90E-08	-1.96E-08	-1.99E-08	-2.05E-08	-2.10E-08	-2.11E-08	-2.13E-08
-1.75E-08	-1.74E-08	-1.87E-08	-1.82E-08	-1.89E-08	-1.99E-08	-1.89E-08	-1.92E-08	-1.97E-08	-2.00E-08	-2.08E-08	-2.10E-08	-2.12E-08	-2.14E-08
-1.74E-08	-1.75E-08	-1.87E-08	-1.80E-08	-1.89E-08	-2.03E-08	-1.97E-08	-1.98E-08	-1.99E-08	-2.03E-08	-2.09E-08	-2.11E-08	-2.10E-08	-2.12E-08
-1.73E-08	-1.82E-08	-1.86E-08	-1.79E-08	-1.88E-08	-2.00E-08	-1.99E-08	-2.00E-08	-2.03E-08	-2.05E-08	-2.08E-08	-2.10E-08	-2.06E-08	-2.07E-08
-1.72E-08	-1.81E-08	-1.85E-08	-1.79E-08	-1.83E-08	-1.92E-08	-1.97E-08	-2.01E-08	-2.02E-08	-2.07E-08	-2.06E-08	-2.04E-08	-2.10E-08	-2.07E-08
-1.74E-08	-1.78E-08	-1.83E-08	-1.81E-08	-1.81E-08	-1.86E-08	-1.96E-08	-2.03E-08	-1.99E-08	-2.06E-08	-2.03E-08	-2.03E-08	-2.16E-08	-2.09E-08
-1.79E-08	-1.81E-08	-1.83E-08	-1.80E-08	-1.76E-08	-1.84E-08	-1.90E-08	-1.98E-08	-1.96E-08	-2.05E-08	-2.04E-08	-2.00E-08	-2.06E-08	-2.04E-08
-1.76E-08	-1.78E-08	-1.79E-08	-1.77E-08	-1.75E-08	-1.83E-08	-1.85E-08	-1.94E-08	-1.93E-08	-2.02E-08	-2.03E-08	-1.99E-08	-1.93E-08	-2.01E-08
-1.68E-08	-1.70E-08	-1.75E-08	-1.77E-08	-1.78E-08	-1.84E-08	-1.82E-08	-1.96E-08	-1.94E-08	-2.03E-08	-2.05E-08	-2.00E-08	-1.92E-08	-1.97E-08
-1.61E-08	-1.68E-08	-1.72E-08	-1.78E-08	-1.80E-08	-1.81E-08	-1.82E-08	-1.94E-08	-1.93E-08	-2.00E-08	-2.03E-08	-2.02E-08	-1.96E-08	-2.00E-08
-1.53E-08	-1.67E-08	-1.69E-08	-1.79E-08	-1.80E-08	-1.75E-08	-1.81E-08	-1.90E-08	-1.90E-08	-1.92E-08	-2.03E-08	-2.02E-08	-2.00E-08	-1.99E-08
-1.49E-08	-1.61E-08	-1.68E-08	-1.77E-08	-1.77E-08	-1.74E-08	-1.78E-08	-1.84E-08	-1.85E-08	-1.84E-08	-2.00E-08	-1.97E-08	-1.94E-08	-1.96E-08
-1.48E-08	-1.61E-08	-1.65E-08	-1.73E-08	-1.71E-08	-1.72E-08	-1.78E-08	-1.81E-08	-1.83E-08	-1.78E-08	-1.91E-08	-1.89E-08	-1.90E-08	-1.91E-08
-1.52E-08	-1.60E-08	-1.60E-08	-1.68E-08	-1.70E-08	-1.77E-08	-1.78E-08	-1.81E-08	-1.81E-08	-1.77E-08	-1.87E-08	-1.82E-08	-1.88E-08	-1.93E-08
-1.55E-08	-1.56E-08	-1.57E-08	-1.62E-08	-1.70E-08	-1.73E-08	-1.74E-08	-1.80E-08	-1.77E-08	-1.75E-08	-1.82E-08	-1.76E-08	-1.86E-08	-1.94E-08
-1.54E-08	-1.52E-08	-1.53E-08	-1.57E-08	-1.67E-08	-1.68E-08	-1.68E-08	-1.76E-08	-1.68E-08	-1.67E-08	-1.76E-08	-1.73E-08	-1.85E-08	-1.93E-08
-1.51E-08	-1.48E-08	-1.46E-08	-1.51E-08	-1.63E-08	-1.62E-08	-1.63E-08	-1.74E-08	-1.65E-08	-1.59E-08	-1.68E-08	-1.71E-08	-1.79E-08	-1.87E-08
-1.44E-08	-1.43E-08	-1.43E-08	-1.47E-08	-1.56E-08	-1.56E-08	-1.61E-08	-1.67E-08	-1.61E-08	-1.54E-08	-1.69E-08	-1.73E-08	-1.74E-08	-1.80E-08
-1.38E-08	-1.47E-08	-1.41E-08	-1.43E-08	-1.50E-08	-1.46E-08	-1.53E-08	-1.59E-08	-1.58E-08	-1.50E-08	-1.68E-08	-1.68E-08	-1.66E-08	-1.71E-08
-1.36E-08	-1.54E-08	-1.37E-08	-1.36E-08	-1.44E-08	-1.34E-08	-1.42E-08	-1.46E-08	-1.54E-08	-1.49E-08	-1.64E-08	-1.59E-08	-1.58E-08	-1.63E-08
-1.35E-08	-1.69E-08	-1.28E-08	-1.27E-08	-1.40E-08	-1.30E-08	-1.35E-08	-1.35E-08	-1.47E-08	-1.46E-08	-1.55E-08	-1.55E-08	-1.55E-08	-1.58E-08
-1.38E-08	-1.73E-08	-1.13E-08	-1.17E-08	-1.30E-08	-1.32E-08	-1.34E-08	-1.25E-08	-1.37E-08	-1.39E-08	-1.47E-08	-1.51E-08	-1.51E-08	-1.48E-08
-1.45E-08	-1.51E-08	-8.56E-09	-1.03E-08	-1.28E-08	-1.29E-08	-1.28E-08	-1.30E-08	-1.33E-08	-1.35E-08	-1.41E-08	-1.46E-08	-1.44E-08	-1.42E-08
-1.35E-08	-8.44E-09	-3.61E-09	-8.35E-09	-1.25E-08	-1.24E-08	-1.25E-08	-1.25E-08	-1.31E-08	-1.31E-08	-1.38E-08	-1.43E-08	-1.40E-08	-1.44E-08
-8.77E-09	-1.11E-09	1.80E-09	-7.10E-09	-1.22E-08	-1.20E-08	-1.23E-08	-1.21E-08	-1.28E-08	-1.24E-08	-1.32E-08	-1.39E-08	-1.37E-08	-1.44E-08
-1.21E-09	-1.01E-09	-4.20E-09	-1.06E-08	-1.19E-08	-1.14E-08	-1.19E-08	-1.17E-08	-1.19E-08	-1.18E-08	-1.26E-08	-1.31E-08	-1.30E-08	-1.39E-08
-3.58E-09	-8.37E-09	-1.05E-08	-1.02E-08	-1.13E-08	-1.11E-08	-1.15E-08	-1.13E-08	-1.13E-08	-1.12E-08	-1.21E-08	-1.25E-08	-1.23E-08	-1.31E-08
-7.93E-09	-8.32E-09	-9.50E-09	-9.13E-09	-1.04E-08	-1.07E-08	-1.12E-08	-1.06E-08	-1.11E-08	-1.09E-08	-1.15E-08	-1.19E-08	-1.16E-08	-1.26E-08
-8.07E-09	-8.00E-09	-9.28E-09	-9.12E-09	-9.45E-09	-1.03E-08	-1.03E-08	-9.82E-09	-1.06E-08	-1.05E-08	-1.03E-08	-1.10E-08	-1.07E-08	-1.21E-08
-8.85E-09	-8.80E-09	-9.68E-09	-9.08E-09	-8.74E-09	-1.01E-08	-9.77E-09	-9.57E-09	-1.02E-08	-9.90E-09	-9.57E-09	-1.02E-08	-1.03E-08	-1.15E-08
-9.17E-09	-9.08E-09	-9.74E-09	-9.01E-09	-8.60E-09	-9.64E-09	-9.61E-09	-9.38E-09	-9.73E-09	-9.70E-09	-9.32E-09	-9.67E-09	-9.79E-09	-1.10E-08
-8.62E-09	-8.90E-09	-9.57E-09	-8.89E-09	-8.56E-09	-8.99E-09	-9.16E-09	-8.76E-09	-9.21E-09	-9.43E-09	-9.08E-09	-9.45E-09	-9.21E-09	-1.03E-08

-8.35E-09	-8.24E-09	-9.20E-09	-8.51E-09	-7.90E-09	-8.11E-09	-8.83E-09	-8.69E-09	-8.89E-09	-9.11E-09	-8.89E-09	-9.08E-09	-8.70E-09	-9.33E-09
-8.07E-09	-7.49E-09	-8.45E-09	-7.94E-09	-7.26E-09	-7.36E-09	-8.01E-09	-8.10E-09	-8.40E-09	-8.23E-09	-8.45E-09	-8.52E-09	-8.21E-09	-8.52E-09
-7.69E-09	-6.97E-09	-7.74E-09	-7.20E-09	-6.77E-09	-6.93E-09	-6.88E-09	-7.42E-09	-7.94E-09	-7.58E-09	-7.61E-09	-7.98E-09	-7.63E-09	-8.02E-09
-6.65E-09	-6.28E-09	-7.03E-09	-6.53E-09	-6.43E-09	-6.65E-09	-6.27E-09	-6.74E-09	-7.32E-09	-6.87E-09	-6.88E-09	-7.58E-09	-7.31E-09	-7.58E-09
-5.39E-09	-5.94E-09	-6.82E-09	-5.78E-09	-5.55E-09	-6.16E-09	-5.81E-09	-6.10E-09	-6.54E-09	-6.45E-09	-6.45E-09	-7.07E-09	-6.36E-09	-6.90E-09
-4.65E-09	-5.54E-09	-6.01E-09	-5.47E-09	-5.12E-09	-5.54E-09	-5.21E-09	-5.05E-09	-5.85E-09	-5.97E-09	-5.82E-09	-5.92E-09	-5.14E-09	-5.73E-09
-4.30E-09	-4.84E-09	-5.00E-09	-5.22E-09	-4.53E-09	-4.86E-09	-4.59E-09	-4.19E-09	-4.93E-09	-5.34E-09	-5.05E-09	-4.47E-09	-4.10E-09	-4.56E-09
-4.41E-09	-4.36E-09	-3.83E-09	-4.74E-09	-3.76E-09	-4.23E-09	-3.98E-09	-3.44E-09	-3.99E-09	-4.38E-09	-4.49E-09	-4.15E-09	-3.83E-09	-3.79E-09
-3.90E-09	-3.59E-09	-3.35E-09	-4.03E-09	-3.03E-09	-3.28E-09	-2.98E-09	-2.41E-09	-2.71E-09	-3.35E-09	-4.01E-09	-3.97E-09	-3.55E-09	-3.34E-09
-3.10E-09	-3.20E-09	-3.18E-09	-3.44E-09	-2.58E-09	-2.15E-09	-1.28E-09	-1.49E-09	-1.49E-09	-2.32E-09	-3.12E-09	-3.46E-09	-2.55E-09	-2.74E-09
-2.34E-09	-2.27E-09	-2.39E-09	-2.53E-09	-2.12E-09	-1.53E-09	-4.00E-10	-9.22E-10	-3.47E-10	-1.55E-09	-2.33E-09	-2.36E-09	-1.83E-09	-1.78E-09
-1.45E-09	-1.84E-09	-1.58E-09	-1.72E-09	-1.21E-09	-1.00E-09	-3.70E-10	-2.35E-11	8.33E-10	-4.01E-10	-1.12E-09	-1.43E-09	-5.71E-10	-9.91E-10
-4.24E-10	-1.08E-09	-1.13E-09	-9.16E-10	1.97E-10	-2.49E-10	1.23E-10	1.81E-10	1.46E-09	8.99E-10	8.00E-11	-3.03E-10	5.68E-10	1.67E-11
1.37E-10	-3.64E-10	-3.05E-10	2.18E-10	1.07E-09	5.52E-10	7.16E-10	5.99E-10	1.17E-09	1.24E-09	9.90E-10	1.01E-09	1.33E-09	1.06E-09
7.38E-10	1.28E-10	7.45E-10	8.33E-10	1.24E-09	1.32E-09	1.68E-09	8.62E-10	1.57E-09	1.52E-09	1.66E-09	1.70E-09	2.22E-09	2.16E-09
1.80E-09	8.92E-10	1.62E-09	1.54E-09	1.51E-09	1.85E-09	2.39E-09	1.62E-09	2.65E-09	2.23E-09	2.04E-09	2.67E-09	3.31E-09	3.11E-09
2.78E-09	2.23E-09	2.61E-09	2.67E-09	2.61E-09	2.74E-09	3.41E-09	2.86E-09	3.86E-09	3.33E-09	3.12E-09	3.55E-09	3.91E-09	4.02E-09
4.22E-09	3.86E-09	3.96E-09	3.77E-09	3.88E-09	4.08E-09	4.33E-09	4.28E-09	4.59E-09	4.63E-09	4.72E-09	4.66E-09	4.80E-09	5.11E-09
5.32E-09	4.65E-09	5.11E-09	4.88E-09	4.76E-09	5.13E-09	5.58E-09	5.39E-09	5.24E-09	5.89E-09	6.02E-09	5.83E-09	5.81E-09	5.98E-09
5.52E-09	5.55E-09	6.01E-09	6.35E-09	5.80E-09	6.22E-09	6.79E-09	6.52E-09	6.50E-09	7.05E-09	7.09E-09	6.66E-09	6.99E-09	7.13E-09
6.21E-09	6.43E-09	7.19E-09	7.59E-09	7.12E-09	7.27E-09	7.93E-09	7.41E-09	7.55E-09	7.95E-09	7.72E-09	7.61E-09	7.67E-09	8.40E-09
7.51E-09	7.62E-09	8.42E-09	7.90E-09	8.12E-09	8.64E-09	8.65E-09	8.30E-09	8.67E-09	8.79E-09	8.73E-09	9.13E-09	9.05E-09	9.85E-09
8.75E-09	8.72E-09	9.67E-09	8.83E-09	9.08E-09	9.56E-09	9.50E-09	9.37E-09	9.74E-09	9.89E-09	9.65E-09	1.04E-08	1.03E-08	1.10E-08
1.01E-08	1.03E-08	1.08E-08	9.86E-09	1.00E-08	1.07E-08	1.04E-08	1.04E-08	1.08E-08	1.10E-08	1.10E-08	1.13E-08	1.12E-08	1.15E-08
1.16E-08	1.20E-08	1.23E-08	1.09E-08	1.12E-08	1.22E-08	1.15E-08	1.15E-08	1.20E-08	1.24E-08	1.23E-08	1.23E-08	1.24E-08	1.22E-08
1.27E-08	1.30E-08	1.32E-08	1.21E-08	1.25E-08	1.38E-08	1.25E-08	1.22E-08	1.27E-08	1.35E-08	1.37E-08	1.41E-08	1.36E-08	1.34E-08
1.35E-08	1.39E-08	1.39E-08	1.33E-08	1.41E-08	1.47E-08	1.38E-08	1.34E-08	1.39E-08	1.50E-08	1.50E-08	1.53E-08	1.46E-08	1.50E-08
1.45E-08	1.58E-08	1.51E-08	1.52E-08	1.55E-08	1.57E-08	1.53E-08	1.52E-08	1.58E-08	1.66E-08	1.67E-08	1.63E-08	1.59E-08	1.61E-08
1.60E-08	1.73E-08	1.63E-08	1.69E-08	1.69E-08	1.72E-08	1.66E-08	1.71E-08	1.76E-08	1.85E-08	1.83E-08	1.81E-08	1.72E-08	1.75E-08
1.76E-08	1.85E-08	1.76E-08	1.81E-08	1.78E-08	1.83E-08	1.78E-08	1.91E-08	1.90E-08	1.96E-08	1.93E-08	1.94E-08	1.85E-08	1.92E-08
1.81E-08	1.91E-08	1.88E-08	1.87E-08	1.90E-08	1.95E-08	1.91E-08	2.04E-08	1.95E-08	2.04E-08	2.08E-08	2.09E-08	1.98E-08	2.05E-08
1.89E-08	1.96E-08	2.01E-08	1.97E-08	2.02E-08	2.04E-08	2.02E-08	2.11E-08	2.02E-08	2.09E-08	2.17E-08	2.14E-08	2.09E-08	2.17E-08
2.02E-08	2.07E-08	2.14E-08	2.10E-08	2.13E-08	2.14E-08	2.14E-08	2.14E-08	2.14E-08	2.21E-08	2.27E-08	2.25E-08	2.27E-08	2.34E-08
2.15E-08	2.23E-08	2.29E-08	2.28E-08	2.29E-08	2.27E-08	2.26E-08	2.22E-08	2.25E-08	2.35E-08	2.37E-08	2.40E-08	2.47E-08	2.53E-08
2.35E-08	2.38E-08	2.43E-08	2.47E-08	2.50E-08	2.41E-08	2.40E-08	2.37E-08	2.41E-08	2.48E-08	2.49E-08	2.53E-08	2.59E-08	2.71E-08
2.56E-08	2.55E-08	2.55E-08	2.61E-08	2.65E-08	2.53E-08	2.55E-08	2.56E-08	2.58E-08	2.60E-08	2.63E-08	2.70E-08	2.73E-08	2.85E-08
2.72E-08	2.64E-08	2.64E-08	2.73E-08	2.76E-08	2.69E-08	2.69E-08	2.74E-08	2.73E-08	2.74E-08	2.79E-08	2.89E-08	2.89E-08	2.97E-08
2.82E-08	2.75E-08	2.79E-08	2.83E-08	2.87E-08	2.83E-08	2.82E-08	2.89E-08	2.86E-08	2.91E-08	2.96E-08	3.06E-08	3.06E-08	3.08E-08
2.89E-08	2.92E-08	2.94E-08	2.96E-08	2.96E-08	3.00E-08	2.99E-08	3.05E-08	3.03E-08	3.08E-08	3.16E-08	3.18E-08	3.22E-08	3.24E-08
2.98E-08	3.09E-08	3.07E-08	3.14E-08	3.11E-08	3.13E-08	3.16E-08	3.23E-08	3.22E-08	3.23E-08	3.29E-08	3.30E-08	3.37E-08	3.33E-08
3.16E-08	3.26E-08	3.17E-08	3.30E-08	3.31E-08	3.31E-08	3.35E-08	3.42E-08	3.40E-08	3.35E-08	3.41E-08	3.44E-08	3.50E-08	3.41E-08
3.31E-08	3.41E-08	3.35E-08	3.43E-08	3.48E-08	3.48E-08	3.56E-08	3.62E-08	3.56E-08	3.53E-08	3.57E-08	3.56E-08	3.62E-08	3.54E-08
3.44E-08	3.51E-08	3.50E-08	3.58E-08	3.61E-08	3.64E-08	3.73E-08	3.78E-08	3.73E-08	3.72E-08	3.74E-08	3.65E-08	3.75E-08	3.68E-08
3.60E-08	3.63E-08	3.63E-08	3.75E-08	3.77E-08	3.81E-08	3.88E-08	3.86E-08	3.85E-08	3.85E-08	3.86E-08	3.77E-08	3.83E-08	3.77E-08
3.76E-08	3.78E-08	3.75E-08	3.95E-08	3.95E-08	3.95E-08	4.02E-08	3.98E-08	3.96E-08	3.97E-08	4.01E-08	3.93E-08	3.95E-08	3.88E-08
3.91E-08	3.94E-08	3.95E-08	4.18E-08	4.13E-08	4.09E-08	4.19E-08	4.13E-08	4.10E-08	4.10E-08	4.19E-08	4.13E-08	4.11E-08	4.06E-08
4.09E-08	4.14E-08	4.16E-08	4.39E-08	4.31E-08	4.25E-08	4.36E-08	4.27E-08	4.29E-08	4.26E-08	4.35E-08	4.29E-08	4.31E-08	4.24E-08
4.29E-08	4.34E-08	4.32E-08	4.46E-08	4.44E-08	4.41E-08	4.50E-08	4.44E-08	4.49E-08	4.44E-08	4.47E-08	4.44E-08	4.47E-08	4.41E-08
4.44E-08	4.52E-08	4.48E-08	4.53E-08	4.56E-08	4.54E-08	4.62E-08	4.63E-08	4.64E-08	4.59E-08	4.64E-08	4.58E-08	4.64E-08	4.56E-08
4.59E-08	4.74E-08	4.67E-08	4.65E-08	4.70E-08	4.69E-08	4.75E-08	4.76E-08	4.75E-08	4.77E-08	4.81E-08	4.74E-08	4.81E-08	4.75E-08

6.40E-17	7.01E-17	7.47E-17	8.85E-17	7.77E-17	9.78E-17	8.04E-17	8.07E-17	8.97E-17	9.60E-17	8.73E-17	9.05E-17	8.78E-17	7.32E-17
7.31E-17	8.45E-17	8.00E-17	1.04E-16	9.74E-17	9.90E-17	8.19E-17	8.51E-17	1.02E-16	1.04E-16	9.30E-17	1.06E-16	9.17E-17	8.62E-17
9.29E-17	9.98E-17	9.71E-17	1.14E-16	1.10E-16	9.90E-17	8.25E-17	9.74E-17	1.12E-16	1.05E-16	9.66E-17	1.13E-16	8.87E-17	9.45E-17
1.03E-16	1.10E-16	1.12E-16	1.18E-16	1.16E-16	1.11E-16	9.76E-17	1.13E-16	1.14E-16	1.05E-16	9.83E-17	1.14E-16	8.79E-17	8.79E-17
1.01E-16	1.16E-16	1.15E-16	1.20E-16	1.17E-16	1.30E-16	1.16E-16	1.22E-16	1.12E-16	1.17E-16	1.04E-16	1.21E-16	1.00E-16	9.67E-17
1.10E-16	1.19E-16	1.20E-16	1.20E-16	1.23E-16	1.41E-16	1.27E-16	1.29E-16	1.21E-16	1.34E-16	1.26E-16	1.36E-16	1.12E-16	1.21E-16
1.18E-16	1.25E-16	1.24E-16	1.31E-16	1.24E-16	1.45E-16	1.37E-16	1.29E-16	1.25E-16	1.37E-16	1.42E-16	1.46E-16	1.32E-16	1.44E-16
1.21E-16	1.27E-16	1.32E-16	1.45E-16	1.30E-16	1.51E-16	1.44E-16	1.32E-16	1.22E-16	1.41E-16	1.53E-16	1.53E-16	1.43E-16	1.62E-16
1.24E-16	1.39E-16	1.61E-16	1.63E-16	1.36E-16	1.51E-16	1.55E-16	1.35E-16	1.27E-16	1.48E-16	1.57E-16	1.46E-16	1.47E-16	1.70E-16
1.21E-16	1.42E-16	1.76E-16	1.66E-16	1.41E-16	1.56E-16	1.72E-16	1.48E-16	1.41E-16	1.55E-16	1.59E-16	1.46E-16	1.55E-16	1.68E-16
1.35E-16	1.45E-16	1.62E-16	1.58E-16	1.55E-16	1.65E-16	1.78E-16	1.69E-16	1.48E-16	1.65E-16	1.64E-16	1.57E-16	1.64E-16	1.74E-16
1.48E-16	1.45E-16	1.56E-16	1.59E-16	1.69E-16	1.77E-16	1.87E-16	1.81E-16	1.59E-16	1.74E-16	1.70E-16	1.74E-16	1.76E-16	1.86E-16
1.55E-16	1.50E-16	1.62E-16	1.63E-16	1.75E-16	1.73E-16	1.93E-16	1.94E-16	1.76E-16	1.82E-16	1.73E-16	1.79E-16	1.82E-16	1.95E-16
1.62E-16	1.61E-16	1.77E-16	1.77E-16	1.76E-16	1.78E-16	1.98E-16	2.00E-16	1.89E-16	1.89E-16	1.82E-16	1.88E-16	1.87E-16	2.05E-16
1.67E-16	1.79E-16	1.90E-16	1.92E-16	1.84E-16	1.82E-16	2.03E-16	2.06E-16	1.96E-16	2.08E-16	1.93E-16	1.97E-16	2.02E-16	2.13E-16
1.89E-16	2.04E-16	2.05E-16	2.06E-16	1.97E-16	1.81E-16	2.04E-16	2.10E-16	2.07E-16	2.22E-16	1.92E-16	2.09E-16	2.14E-16	2.23E-16
2.10E-16	2.30E-16	2.09E-16	2.15E-16	2.07E-16	1.93E-16	2.11E-16	2.26E-16	2.10E-16	2.26E-16	1.99E-16	2.21E-16	2.18E-16	2.29E-16
2.25E-16	2.42E-16	2.17E-16	2.18E-16	2.05E-16	2.12E-16	2.23E-16	2.36E-16	2.22E-16	2.22E-16	2.10E-16	2.21E-16	2.26E-16	2.32E-16
2.14E-16	2.50E-16	2.20E-16	2.27E-16	2.25E-16	2.27E-16	2.34E-16	2.48E-16	2.36E-16	2.20E-16	2.19E-16	2.36E-16	2.38E-16	2.30E-16
2.06E-16	2.52E-16	2.22E-16	2.26E-16	2.35E-16	2.38E-16	2.44E-16	2.58E-16	2.51E-16	2.19E-16	2.28E-16	2.52E-16	2.39E-16	2.35E-16
2.19E-16	2.38E-16	2.26E-16	2.25E-16	2.50E-16	2.52E-16	2.58E-16	2.62E-16	2.48E-16	2.29E-16	2.41E-16	2.61E-16	2.59E-16	2.51E-16
2.27E-16	2.23E-16	2.15E-16	2.37E-16	2.48E-16	2.47E-16	2.63E-16	2.64E-16	2.48E-16	2.53E-16	2.45E-16	2.69E-16	2.73E-16	2.66E-16
2.22E-16	2.18E-16	2.25E-16	2.35E-16	2.70E-16	2.45E-16	2.69E-16	2.75E-16	2.56E-16	2.73E-16	2.58E-16	2.71E-16	2.83E-16	2.65E-16
2.24E-16	2.34E-16	2.52E-16	2.53E-16	2.81E-16	2.53E-16	2.73E-16	2.86E-16	2.75E-16	2.92E-16	2.79E-16	2.79E-16	3.00E-16	2.62E-16
2.54E-16	2.61E-16	2.71E-16	2.46E-16	2.80E-16	2.56E-16	2.76E-16	2.83E-16	2.76E-16	2.90E-16	2.84E-16	2.88E-16	3.17E-16	2.70E-16
2.85E-16	2.88E-16	2.87E-16	2.47E-16	2.82E-16	2.56E-16	2.92E-16	2.71E-16	2.59E-16	2.85E-16	2.80E-16	2.90E-16	3.33E-16	2.94E-16
2.98E-16	2.99E-16	2.92E-16	2.74E-16	2.92E-16	2.63E-16	3.23E-16	2.65E-16	2.54E-16	2.92E-16	2.88E-16	2.94E-16	3.37E-16	3.18E-16
2.93E-16	3.01E-16	2.88E-16	2.76E-16	2.97E-16	2.78E-16	3.34E-16	2.79E-16	2.72E-16	2.95E-16	2.95E-16	2.97E-16	3.29E-16	3.41E-16
2.86E-16	3.06E-16	2.91E-16	3.11E-16	3.12E-16	2.92E-16	3.42E-16	2.96E-16	3.01E-16	3.02E-16	3.10E-16	3.09E-16	3.47E-16	3.64E-16
3.04E-16	3.12E-16	3.13E-16	3.39E-16	3.16E-16	3.00E-16	3.42E-16	3.36E-16	3.55E-16	3.32E-16	3.46E-16	3.36E-16	3.83E-16	3.71E-16
3.29E-16	3.18E-16	3.25E-16	3.47E-16	3.19E-16	3.07E-16	3.41E-16	3.42E-16	3.83E-16	3.56E-16	3.79E-16	3.45E-16	3.94E-16	3.71E-16

3.48E-16	3.15E-16	3.30E-16	3.56E-16	3.34E-16	3.25E-16	3.33E-16	3.47E-16	3.84E-16	3.72E-16	4.00E-16	3.59E-16	4.06E-16	3.77E-16
3.55E-16	3.09E-16	3.30E-16	3.59E-16	3.40E-16	3.42E-16	3.36E-16	3.57E-16	3.97E-16	3.63E-16	4.00E-16	3.80E-16	4.07E-16	3.79E-16
3.41E-16	2.93E-16	3.35E-16	3.59E-16	3.43E-16	3.33E-16	3.37E-16	3.65E-16	3.99E-16	3.65E-16	3.83E-16	3.79E-16	4.08E-16	3.76E-16
3.26E-16	2.97E-16	3.36E-16	3.46E-16	3.51E-16	3.20E-16	3.45E-16	3.53E-16	3.93E-16	3.63E-16	3.66E-16	3.75E-16	3.90E-16	3.72E-16
3.22E-16	3.03E-16	3.42E-16	3.42E-16	3.36E-16	3.13E-16	3.44E-16	3.44E-16	3.85E-16	3.80E-16	3.55E-16	3.69E-16	3.82E-16	3.64E-16
3.07E-16	3.07E-16	3.44E-16	3.40E-16	3.39E-16	3.24E-16	3.23E-16	3.51E-16	3.82E-16	3.88E-16	3.65E-16	3.85E-16	3.86E-16	3.72E-16
3.03E-16	3.14E-16	3.36E-16	3.24E-16	3.35E-16	3.29E-16	3.16E-16	3.64E-16	3.98E-16	4.05E-16	4.04E-16	4.35E-16	4.32E-16	4.12E-16
3.00E-16	3.23E-16	3.40E-16	3.22E-16	3.23E-16	3.38E-16	3.35E-16	3.74E-16	4.00E-16	4.10E-16	3.88E-16	4.47E-16	4.41E-16	4.64E-16
3.15E-16	3.38E-16	3.58E-16	3.30E-16	3.33E-16	3.51E-16	3.56E-16	3.69E-16	3.86E-16	4.10E-16	3.60E-16	4.35E-16	4.28E-16	4.68E-16
3.14E-16	3.48E-16	3.77E-16	3.57E-16	3.68E-16	3.68E-16	3.77E-16	3.66E-16	3.76E-16	4.12E-16	4.00E-16	4.25E-16	4.12E-16	4.44E-16
3.30E-16	3.61E-16	3.68E-16	3.67E-16	3.62E-16	3.65E-16	3.77E-16	3.74E-16	3.92E-16	4.31E-16	4.24E-16	4.00E-16	4.04E-16	4.36E-16
3.23E-16	3.59E-16	3.44E-16	3.63E-16	3.56E-16	3.75E-16	3.73E-16	3.63E-16	3.68E-16	4.18E-16	4.04E-16	3.99E-16	4.15E-16	4.52E-16
3.13E-16	3.52E-16	3.36E-16	3.55E-16	3.35E-16	3.92E-16	3.77E-16	3.65E-16	3.82E-16	4.18E-16	4.08E-16	4.08E-16	4.21E-16	4.42E-16
3.22E-16	3.45E-16	3.61E-16	3.48E-16	3.27E-16	3.94E-16	3.75E-16	3.77E-16	3.91E-16	4.14E-16	4.04E-16	4.07E-16	4.12E-16	4.37E-16
3.27E-16	3.41E-16	3.55E-16	3.54E-16	3.34E-16	3.85E-16	3.54E-16	3.69E-16	3.92E-16	3.96E-16	4.11E-16	4.28E-16	4.28E-16	4.39E-16
3.17E-16	3.22E-16	3.45E-16	3.48E-16	3.57E-16	3.83E-16	3.46E-16	3.60E-16	3.84E-16	3.98E-16	4.22E-16	4.41E-16	4.46E-16	4.54E-16
3.05E-16	3.03E-16	3.50E-16	3.31E-16	3.56E-16	3.98E-16	3.59E-16	3.70E-16	3.89E-16	4.00E-16	4.32E-16	4.43E-16	4.51E-16	4.59E-16
3.02E-16	3.06E-16	3.50E-16	3.26E-16	3.56E-16	4.10E-16	3.89E-16	3.91E-16	3.96E-16	4.14E-16	4.35E-16	4.47E-16	4.43E-16	4.49E-16
2.99E-16	3.30E-16	3.47E-16	3.20E-16	3.52E-16	3.99E-16	3.94E-16	4.01E-16	4.12E-16	4.18E-16	4.34E-16	4.41E-16	4.26E-16	4.29E-16
2.97E-16	3.26E-16	3.44E-16	3.22E-16	3.34E-16	3.67E-16	3.88E-16	4.04E-16	4.08E-16	4.27E-16	4.24E-16	4.14E-16	4.41E-16	4.27E-16
3.02E-16	3.17E-16	3.36E-16	3.28E-16	3.27E-16	3.45E-16	3.84E-16	4.11E-16	3.97E-16	4.24E-16	4.12E-16	4.12E-16	4.66E-16	4.35E-16
3.21E-16	3.29E-16	3.36E-16	3.23E-16	3.10E-16	3.40E-16	3.60E-16	3.94E-16	3.82E-16	4.22E-16	4.15E-16	4.00E-16	4.23E-16	4.17E-16
3.10E-16	3.17E-16	3.21E-16	3.13E-16	3.08E-16	3.37E-16	3.41E-16	3.77E-16	3.71E-16	4.10E-16	4.12E-16	3.96E-16	3.71E-16	4.05E-16
2.82E-16	2.89E-16	3.06E-16	3.12E-16	3.17E-16	3.37E-16	3.30E-16	3.85E-16	3.76E-16	4.12E-16	4.18E-16	4.00E-16	3.70E-16	3.88E-16
2.58E-16	2.82E-16	2.94E-16	3.16E-16	3.23E-16	3.28E-16	3.33E-16	3.75E-16	3.72E-16	3.98E-16	4.13E-16	4.07E-16	3.86E-16	4.02E-16
2.34E-16	2.78E-16	2.85E-16	3.19E-16	3.24E-16	3.07E-16	3.27E-16	3.61E-16	3.61E-16	3.68E-16	4.13E-16	4.07E-16	4.00E-16	3.95E-16
2.21E-16	2.59E-16	2.82E-16	3.14E-16	3.13E-16	3.02E-16	3.15E-16	3.39E-16	3.41E-16	3.39E-16	3.99E-16	3.89E-16	3.78E-16	3.84E-16
2.19E-16	2.59E-16	2.74E-16	2.99E-16	2.92E-16	2.95E-16	3.16E-16	3.29E-16	3.36E-16	3.19E-16	3.64E-16	3.58E-16	3.60E-16	3.66E-16
2.32E-16	2.57E-16	2.56E-16	2.82E-16	2.89E-16	3.13E-16	3.17E-16	3.28E-16	3.27E-16	3.13E-16	3.51E-16	3.32E-16	3.52E-16	3.73E-16
2.41E-16	2.44E-16	2.48E-16	2.62E-16	2.90E-16	2.99E-16	3.04E-16	3.23E-16	3.14E-16	3.05E-16	3.32E-16	3.10E-16	3.46E-16	3.78E-16
2.36E-16	2.32E-16	2.35E-16	2.47E-16	2.79E-16	2.83E-16	2.83E-16	3.10E-16	2.84E-16	2.80E-16	3.10E-16	3.01E-16	3.41E-16	3.72E-16
2.27E-16	2.19E-16	2.13E-16	2.27E-16	2.66E-16	2.63E-16	2.64E-16	3.04E-16	2.72E-16	2.53E-16	2.82E-16	2.92E-16	3.20E-16	3.49E-16
2.08E-16	2.04E-16	2.03E-16	2.15E-16	2.42E-16	2.43E-16	2.61E-16	2.78E-16	2.60E-16	2.36E-16	2.84E-16	3.01E-16	3.01E-16	3.25E-16
1.91E-16	2.17E-16	1.98E-16	2.04E-16	2.24E-16	2.13E-16	2.34E-16	2.51E-16	2.48E-16	2.26E-16	2.84E-16	2.82E-16	2.74E-16	2.93E-16
1.85E-16	2.39E-16	1.87E-16	1.85E-16	2.06E-16	1.78E-16	2.02E-16	2.14E-16	2.38E-16	2.22E-16	2.69E-16	2.53E-16	2.49E-16	2.66E-16
1.81E-16	2.86E-16	1.64E-16	1.61E-16	1.96E-16	1.69E-16	1.82E-16	1.81E-16	2.17E-16	2.12E-16	2.41E-16	2.39E-16	2.40E-16	2.50E-16
1.91E-16	3.00E-16	1.27E-16	1.38E-16	1.70E-16	1.73E-16	1.79E-16	1.56E-16	1.89E-16	1.94E-16	2.15E-16	2.29E-16	2.27E-16	2.18E-16
2.10E-16	2.28E-16	7.33E-17	1.06E-16	1.63E-16	1.68E-16	1.65E-16	1.69E-16	1.76E-16	1.82E-16	2.00E-16	2.13E-16	2.08E-16	2.00E-16
1.82E-16	7.13E-17	1.30E-17	6.98E-17	1.56E-16	1.54E-16	1.57E-16	1.56E-16	1.72E-16	1.72E-16	1.90E-16	2.04E-16	1.97E-16	2.06E-16
7.68E-17	1.22E-18	3.23E-18	5.04E-17	1.50E-16	1.45E-16	1.52E-16	1.46E-16	1.64E-16	1.55E-16	1.75E-16	1.94E-16	1.88E-16	2.07E-16
1.46E-18	1.02E-18	1.77E-17	1.12E-16	1.42E-16	1.31E-16	1.42E-16	1.37E-16	1.42E-16	1.40E-16	1.59E-16	1.72E-16	1.68E-16	1.94E-16
1.28E-17	7.00E-17	1.09E-16	1.03E-16	1.28E-16	1.23E-16	1.33E-16	1.27E-16	1.28E-16	1.26E-16	1.46E-16	1.56E-16	1.51E-16	1.73E-16
6.30E-17	6.92E-17	9.03E-17	8.34E-17	1.08E-16	1.15E-16	1.25E-16	1.12E-16	1.23E-16	1.20E-16	1.33E-16	1.41E-16	1.35E-16	1.58E-16
6.52E-17	6.40E-17	8.62E-17	8.33E-17	8.94E-17	1.05E-16	1.06E-16	9.64E-17	1.12E-16	1.09E-16	1.07E-16	1.21E-16	1.15E-16	1.47E-16
7.83E-17	7.75E-17	9.37E-17	8.25E-17	7.65E-17	1.01E-16	9.55E-17	9.15E-17	1.03E-16	9.81E-17	9.15E-17	1.03E-16	1.06E-16	1.33E-16
8.41E-17	8.25E-17	9.48E-17	8.12E-17	7.40E-17	9.28E-17	9.23E-17	8.80E-17	9.47E-17	9.41E-17	8.69E-17	9.36E-17	9.59E-17	1.21E-16
7.43E-17	7.92E-17	9.15E-17	7.91E-17	7.32E-17	8.08E-17	8.38E-17	7.67E-17	8.49E-17	8.90E-17	8.24E-17	8.93E-17	8.49E-17	1.05E-16
6.97E-17	6.79E-17	8.46E-17	7.25E-17	6.24E-17	6.58E-17	7.79E-17	7.55E-17	7.90E-17	8.29E-17	7.90E-17	8.25E-17	7.58E-17	8.70E-17
6.51E-17	5.61E-17	7.14E-17	6.30E-17	5.28E-17	5.42E-17	6.41E-17	6.56E-17	7.05E-17	6.77E-17	7.14E-17	7.27E-17	6.74E-17	7.26E-17
5.91E-17	4.86E-17	5.99E-17	5.19E-17	4.58E-17	4.81E-17	4.73E-17	5.50E-17	6.31E-17	5.74E-17	5.78E-17	6.37E-17	5.82E-17	6.43E-17
4.42E-17	3.94E-17	4.94E-17	4.26E-17	4.13E-17	4.42E-17	3.93E-17	4.55E-17	5.36E-17	4.73E-17	4.73E-17	5.75E-17	5.34E-17	5.75E-17
2.90E-17	3.52E-17	4.65E-17	3.34E-17	3.08E-17	3.80E-17	3.37E-17	3.72E-17	4.28E-17	4.17E-17	4.16E-17	5.00E-17	4.05E-17	4.77E-17
2.17E-17	3.07E-17	3.61E-17	2.99E-17	2.63E-17	3.07E-17	2.72E-17	2.55E-17	3.43E-17	3.57E-17	3.39E-17	3.51E-17	2.64E-17	3.29E-17
1.85E-17	2.35E-17	2.50E-17	2.73E-17	2.05E-17	2.37E-17	2.11E-17	1.76E-17	2.43E-17	2.86E-17	2.55E-17	1.99E-17	1.68E-17	2.08E-17
1.95E-17	1.90E-17	1.47E-17	2.25E-17	1.42E-17	1.79E-17	1.59E-17	1.18E-17	1.59E-17	1.92E-17	2.01E-17	1.72E-17	1.47E-17	1.44E-17
1.52E-17	1.29E-17	1.12E-17	1.62E-17	9.21E-18	1.08E-17	8.89E-18	5.81E-18	7.34E-18	1.13E-17	1.61E-17	1.57E-17	1.26E-17	1.11E-17
9.59E-18	1.03E-17	1.01E-17	1.18E-17	6.66E-18	4.60E-18	1.65E-18	2.23E-18	2.22E-18	5.37E-18	9.71E-18	1.20E-17	6.50E-18	7.51E-18
5.48E-18	5.13E-18	5.70E-18	6.42E-18	4.49E-18	2.33E-18	1.60E-19	8.51E-19	1.20E-19	2.40E-18	5.43E-18	5.59E-18	3.35E-18	3.16E-18
2.09E-18	3.37E-18	2.50E-18	2.97E-18	1.46E-18	1.01E-18	1.37E-19	5.52E-22	6.94E-19	1.61E-19	1.26E-18	2.05E-18	3.26E-19	9.81E-19
1.80E-19	1.17E-18	1.28E-18	8.39E-19	3.87E-20	6.21E-20	1.50E-20	3.28E-20	2.12E-18	8.09E-19	6.40E-21	9.17E-20	3.23E-19	2.79E-22
1.87E-20	1.33E-19	9.32E-20	4.74E-20	1.15E-18	3.04E-19	5.13E-19	3.58E-19	1.38E-18	1.55E-18	9.81E-19	1.01E-18	1.77E-18	1.12E-18
5.44E-19	1.64E-20	5.55E-19	6.94E-19	1.53E-18	1.73E-18	2.83E-18	7.43E-19	2.46E-18	2.32E-18	2.75E-18	2.88E-18	4.94E-18	4.64E-18
3.24E-18	7.96E-19	2.64E-18	2.36E-18	2.27E-18	3.43E-18	5.70E-18	2.61E-18	7.02E-18	4.96E-18	4.15E-18	7.13E-18	1.10E-17	9.68E-18
7.73E-18	4.98E-18	6.80E-18	7.13E-18	6.82E-18	7.51E-18	1.16E-17	8.20E-18	1.49E-17	1.11E-17	9.75E-18	1.26E-17	1.53E-17	1.61E-17
1.78E-17	1.49E-17	1.57E-17	1.42E-17	1.50E-17	1.66E-17	1.88E-17	1.83E-17	2.11E-17	2.15E-17	2.23E-17	2.17E-17	2.30E-17	2.61E-17
2.83E-17	2.17E-17	2.61E-17	2.38E-17	2.26E-17	2.63E-17	3.11E-17	2.91E-17	2.75E-17	3.47E-17	3.62E-17	3.40E-17	3.38E-17	3.57E-17
3.05E-17	3.08E-17	3.61E-17	4.03E-17	3.36E-17	3.87E-17	4.61E-17	4.25E-17	4.22E-17	4.98E-17	5.02E-17	4.43E-17	4.89E-17	5.08E-17
3.86E-17	4.13E-17	5.16E-17	5.77E-17	5.07E-17	5.29E-17	6.29E-17	5.50E-17	5.70E-17	6.32E-17	5.96E-17	5.80E-17	5.88E-17	7.06E-17
5.64E-17	5.81E-17	7.09E-17	6.24E-17	6.59E-17	7.46E-17	7.48E-17	6.89E-17	7.52E-17	7.73E-17	7.62E-17	8.33E-17	8.20E-17	9.70E-17
7.65E-17	7.60E-17	9.34E-17	7.80E-17	8.25E-17	9.13E-17	9.02E-17	8.78E-17	9.49E-17	9.77E-17	9.32E-17	1.07E-16	1.05E-16	1.21E-16
1.03E-16	1.07E-16	1.16E-16	9.72E-17	1.00E-16	1.14E-16	1.07E-16	1.09E-16	1.16E-16	1.21E-16	1.21E-16	1.27E-16	1.25E-16	1.33E-16
1.34E-16	1.44E-16	1.51E-16	1.19E-16	1.25E-16	1.49E-16	1.32E-16	1.33E-16	1.45E-16	1.54E-16	1.52E-16	1.52E-16	1.53E-16	1.50E-16
1.60E-16	1.68E-16	1.75E-16	1.47E-16	1.55E-16	1.91E-16	1.55E-16	1.49E-16	1.62E-16	1.82E-16	1.89E-16	1.99E-16	1.85E-16	1.80E-16
1.82E-16	1.93E-16	1.92E-16	1.78E-16	1.97E-16	2.15E-16	1.90E-16	1.79E-16	1.92E-16	2.24E-16	2.26E-16	2.33E-16	2.14E-16	2.24E-16
2.10E-16	2.50E-16	2.27E-16	2.30E-16	2.39E-16	2.47E-16	2.33E-16	2.30E-16	2.49E-16	2.77E-16	2.79E-16	2.67E-16	2.53E-16	2.58E-16
2.57E-16	2.98E-16	2.67E-16	2.87E-16	2.86E-16	2.95E-16	2.74E-16	2.92E-16	3.09E-16	3.43E-16	3.33E-16	3.27E-16	2.97E-16	3.08E-16
3.11E-16	3.42E-16	3.09E-16	3.27E-16	3.19E-16	3.36E-16	3.17E-16	3.65E-16	3.60E-16	3.86E-16	3.71E-16	3.78E-16	3.42E-16	3.68E-16
3.26E-16	3.64E-16	3.52E-16	3.51E-16	3.61E-16	3.80E-16	3.63E-16	4.15E-16	3.79E-16	4.17E-16	4.33E-16	4.35E-16	3.91E-16	4.19E-16
3.58E-16	3.82E-16	4.04E-16	3.87E-16	4.07E-16	4.16E-16	4.08E-16	4.43E-16	4.07E-16	4.36E-16	4.72E-16	4.58E-16	4.37E-16	4.71E-16
4.08E-16	4.28E-16	4.59E-16	4.43E-16	4.52E-16	4.59E-16	4.59E-16	4.60E-16	4.59E-16	4.88E-16	5.17E-16	5.05E-16	5.16E-16	5.47E-16
4.62E-16	4.96E-16	5.24E-16	5.22E-16	5.25E-16	5.14E-16	5.09E-16	4.93E-16	5.08E-16	5.54E-16	5.61E-16	5.74E-16	6.12E-16	6.39E-16
5.51E-16	5.68E-16	5.91E-16	6.10E-16	6.26E-16	5.80E-16	5.75E-16	5.60E-16	5.80E-16	6.14E-16	6.20E-16	6.42E-16	6.73E-16	7.33E-16
6.54E-16	6.49E-16	6.51E-16	6.79E-16	7.03E-16	6.42E-16	6.48E-16	6.58E-16	6.67E-16	6.76E-16	6.89E-16	7.29E-16	7.45E-16	8.14E-16
7.38E-16	6.99E-16	6.99E-16	7.44E-16	7.61E-16	7.22E-16	7.24E-16	7.49E-16	7.45E-16	7.52E-16	7.78E-16	8.35E-16	8.36E-16	8.79E-16
7.96E-16	7.56E-16	7.76E-16	8.03E-16	8.24E-16	8.01E-16	7.98E-16	8.33E-16	8.19E-16	8.47E-16	8.78E-16	9.37E-16	9.36E-16	9.46E-16
8.36E-16	8.52E-16	8.64E-16	8.77E-16	8.77E-16	8.98E-16	8.94E-16	9.32E-16	9.16E-16	9.50E-16	9.97E-16	1.01E-15	1.04E-15	1.05E-15

8.89E-16	9.52E-16	9.41E-16	9.87E-16	9.66E-16	9.83E-16	9.97E-16	1.04E-15	1.03E-15	1.05E-15	1.08E-15	1.09E-15	1.14E-15	1.11E-15
9.95E-16	1.06E-15	1.01E-15	1.09E-15	1.10E-15	1.09E-15	1.12E-15	1.17E-15	1.16E-15	1.12E-15	1.16E-15	1.18E-15	1.23E-15	1.16E-15
1.09E-15	1.16E-15	1.12E-15	1.17E-15	1.21E-15	1.21E-15	1.27E-15	1.31E-15	1.27E-15	1.25E-15	1.28E-15	1.26E-15	1.31E-15	1.26E-15
1.18E-15	1.23E-15	1.22E-15	1.28E-15	1.31E-15	1.33E-15	1.39E-15	1.43E-15	1.39E-15	1.38E-15	1.40E-15	1.33E-15	1.40E-15	1.35E-15
1.29E-15	1.32E-15	1.32E-15	1.40E-15	1.42E-15	1.45E-15	1.51E-15	1.49E-15	1.48E-15	1.48E-15	1.49E-15	1.42E-15	1.47E-15	1.42E-15
1.41E-15	1.43E-15	1.40E-15	1.56E-15	1.56E-15	1.56E-15	1.62E-15	1.59E-15	1.57E-15	1.57E-15	1.61E-15	1.54E-15	1.56E-15	1.51E-15
1.53E-15	1.55E-15	1.56E-15	1.74E-15	1.70E-15	1.67E-15	1.76E-15	1.71E-15	1.68E-15	1.68E-15	1.75E-15	1.70E-15	1.69E-15	1.65E-15
1.67E-15	1.71E-15	1.73E-15	1.93E-15	1.85E-15	1.81E-15	1.90E-15	1.82E-15	1.84E-15	1.82E-15	1.89E-15	1.84E-15	1.85E-15	1.80E-15
1.84E-15	1.88E-15	1.87E-15	1.99E-15	1.97E-15	1.94E-15	2.03E-15	1.97E-15	2.02E-15	1.97E-15	2.00E-15	1.97E-15	2.00E-15	1.94E-15
1.97E-15	2.05E-15	2.00E-15	2.05E-15	2.08E-15	2.06E-15	2.14E-15	2.14E-15	2.15E-15	2.11E-15	2.15E-15	2.10E-15	2.15E-15	2.08E-15
2.11E-15	2.24E-15	2.18E-15	2.16E-15	2.21E-15	2.20E-15	2.26E-15	2.27E-15	2.26E-15	2.28E-15	2.32E-15	2.25E-15	2.31E-15	2.26E-15
4.01E-14	4.17E-14	4.18E-14	4.32E-14	4.39E-14	4.42E-14	4.54E-14	4.59E-14	4.63E-14	4.70E-14	4.81E-14	4.83E-14	4.90E-14	4.93E-14

SUM OF SQUARED QUADRA		2.85E-12
TOTAL NUMBER VALUES IN		16,384
AVERAGE SQUARED		1.74E-16
RMS	1.31999E-08	

-9.07E-09	-8.30E-09	-8.98E-09	-8.81E-09	-9.66E-09	-9.83E-09
-9.85E-09	-9.15E-09	-9.60E-09	-1.01E-08	-1.04E-08	-9.01E-09
-1.01E-08	-9.84E-09	-1.02E-08	-1.06E-08	-1.08E-08	-7.97E-09
-1.01E-08	-1.03E-08	-1.07E-08	-1.09E-08	-1.15E-08	-9.36E-09
-1.00E-08	-1.07E-08	-1.09E-08	-1.18E-08	-1.24E-08	-1.24E-08
-1.05E-08	-1.12E-08	-1.17E-08	-1.23E-08	-1.28E-08	-1.34E-08
-1.13E-08	-1.17E-08	-1.27E-08	-1.31E-08	-1.36E-08	-1.48E-08
-1.17E-08	-1.26E-08	-1.31E-08	-1.37E-08	-1.42E-08	-1.57E-08
-1.26E-08	-1.26E-08	-1.35E-08	-1.43E-08	-1.50E-08	-1.62E-08
-1.33E-08	-1.32E-08	-1.38E-08	-1.46E-08	-1.55E-08	-1.67E-08
-1.37E-08	-1.38E-08	-1.43E-08	-1.51E-08	-1.58E-08	-1.69E-08
-1.40E-08	-1.46E-08	-1.48E-08	-1.52E-08	-1.60E-08	-1.72E-08
-1.43E-08	-1.50E-08	-1.51E-08	-1.51E-08	-1.61E-08	-1.74E-08
-1.48E-08	-1.53E-08	-1.55E-08	-1.52E-08	-1.61E-08	-1.79E-08
-1.54E-08	-1.56E-08	-1.59E-08	-1.55E-08	-1.63E-08	-1.82E-08
-1.58E-08	-1.59E-08	-1.65E-08	-1.61E-08	-1.64E-08	-1.86E-08
-1.60E-08	-1.62E-08	-1.67E-08	-1.63E-08	-1.66E-08	-1.91E-08
-1.62E-08	-1.65E-08	-1.71E-08	-1.64E-08	-1.72E-08	-1.94E-08
-1.62E-08	-1.69E-08	-1.73E-08	-1.72E-08	-1.81E-08	-1.99E-08
-1.62E-08	-1.72E-08	-1.73E-08	-1.78E-08	-1.90E-08	-2.03E-08
-1.63E-08	-1.72E-08	-1.78E-08	-1.83E-08	-1.96E-08	-2.02E-08
-1.71E-08	-1.74E-08	-1.81E-08	-1.85E-08	-1.99E-08	-2.03E-08
-1.75E-08	-1.76E-08	-1.84E-08	-1.88E-08	-1.98E-08	-2.05E-08
-1.77E-08	-1.82E-08	-1.85E-08	-1.92E-08	-1.96E-08	-2.08E-08
-1.78E-08	-1.86E-08	-1.92E-08	-1.96E-08	-1.98E-08	-2.14E-08
-1.82E-08	-1.95E-08	-1.96E-08	-1.97E-08	-2.04E-08	-2.22E-08
-1.89E-08	-2.00E-08	-1.99E-08	-2.00E-08	-2.13E-08	-2.31E-08
-1.96E-08	-2.04E-08	-2.04E-08	-2.02E-08	-2.20E-08	-2.38E-08
-1.99E-08	-2.05E-08	-2.06E-08	-2.07E-08	-2.23E-08	-2.47E-08
-1.98E-08	-2.06E-08	-2.11E-08	-2.14E-08	-2.25E-08	-2.53E-08
-1.96E-08	-2.03E-08	-2.13E-08	-2.19E-08	-2.30E-08	-2.58E-08
-1.92E-08	-2.00E-08	-2.13E-08	-2.21E-08	-2.34E-08	-2.64E-08
-1.94E-08	-2.03E-08	-2.14E-08	-2.23E-08	-2.41E-08	-2.71E-08
-1.97E-08	-2.08E-08	-2.14E-08	-2.27E-08	-2.45E-08	-2.74E-08
-1.98E-08	-2.13E-08	-2.17E-08	-2.30E-08	-2.51E-08	-2.77E-08
-2.03E-08	-2.13E-08	-2.21E-08	-2.32E-08	-2.53E-08	-2.80E-08
-2.10E-08	-2.16E-08	-2.29E-08	-2.36E-08	-2.53E-08	-2.85E-08
-2.12E-08	-2.17E-08	-2.37E-08	-2.41E-08	-2.54E-08	-2.90E-08
-2.13E-08	-2.24E-08	-2.42E-08	-2.46E-08	-2.58E-08	-2.94E-08
-2.17E-08	-2.26E-08	-2.42E-08	-2.44E-08	-2.60E-08	-2.97E-08
-2.17E-08	-2.25E-08	-2.36E-08	-2.41E-08	-2.62E-08	-2.97E-08
-2.15E-08	-2.26E-08	-2.31E-08	-2.44E-08	-2.58E-08	-2.97E-08
-2.21E-08	-2.23E-08	-2.31E-08	-2.49E-08	-2.61E-08	-3.00E-08
-2.20E-08	-2.18E-08	-2.31E-08	-2.55E-08	-2.64E-08	-3.03E-08
-2.20E-08	-2.19E-08	-2.34E-08	-2.50E-08	-2.61E-08	-3.00E-08
-2.19E-08	-2.21E-08	-2.35E-08	-2.48E-08	-2.59E-08	-2.95E-08
-2.22E-08	-2.27E-08	-2.38E-08	-2.53E-08	-2.60E-08	-2.93E-08
-2.27E-08	-2.30E-08	-2.38E-08	-2.52E-08	-2.60E-08	-2.91E-08
-2.26E-08	-2.31E-08	-2.37E-08	-2.48E-08	-2.62E-08	-2.91E-08
-2.19E-08	-2.28E-08	-2.37E-08	-2.46E-08	-2.62E-08	-2.92E-08
-2.15E-08	-2.26E-08	-2.35E-08	-2.47E-08	-2.63E-08	-2.92E-08
-2.15E-08	-2.25E-08	-2.32E-08	-2.47E-08	-2.61E-08	-2.89E-08
-2.12E-08	-2.25E-08	-2.33E-08	-2.44E-08	-2.62E-08	-2.87E-08
-2.13E-08	-2.21E-08	-2.33E-08	-2.42E-08	-2.59E-08	-2.83E-08
-2.09E-08	-2.18E-08	-2.31E-08	-2.40E-08	-2.54E-08	-2.78E-08
-2.08E-08	-2.20E-08	-2.29E-08	-2.35E-08	-2.49E-08	-2.77E-08
-2.08E-08	-2.20E-08	-2.25E-08	-2.29E-08	-2.48E-08	-2.78E-08
-2.02E-08	-2.14E-08	-2.21E-08	-2.25E-08	-2.48E-08	-2.78E-08
-2.01E-08	-2.11E-08	-2.16E-08	-2.25E-08	-2.47E-08	-2.73E-08
-2.02E-08	-2.09E-08	-2.12E-08	-2.23E-08	-2.39E-08	-2.66E-08
-1.97E-08	-2.04E-08	-2.08E-08	-2.16E-08	-2.30E-08	-2.59E-08
-1.91E-08	-1.99E-08	-2.03E-08	-2.08E-08	-2.22E-08	-2.51E-08
-1.84E-08	-1.92E-08	-2.01E-08	-2.01E-08	-2.18E-08	-2.39E-08
-1.78E-08	-1.90E-08	-1.96E-08	-1.94E-08	-2.11E-08	-2.31E-08
-1.76E-08	-1.89E-08	-1.89E-08	-1.89E-08	-2.04E-08	-2.27E-08
-1.74E-08	-1.82E-08	-1.76E-08	-1.83E-08	-2.02E-08	-2.20E-08
-1.68E-08	-1.72E-08	-1.64E-08	-1.82E-08	-1.99E-08	-2.14E-08
-1.56E-08	-1.65E-08	-1.71E-08	-1.80E-08	-1.95E-08	-2.11E-08
-1.48E-08	-1.57E-08	-1.68E-08	-1.72E-08	-1.88E-08	-2.07E-08
-1.42E-08	-1.50E-08	-1.59E-08	-1.69E-08	-1.82E-08	-2.00E-08
-1.41E-08	-1.50E-08	-1.54E-08	-1.61E-08	-1.75E-08	-1.93E-08
-1.31E-08	-1.45E-08	-1.47E-08	-1.54E-08	-1.70E-08	-1.85E-08
-1.25E-08	-1.40E-08	-1.41E-08	-1.47E-08	-1.64E-08	-1.81E-08
-1.24E-08	-1.35E-08	-1.36E-08	-1.44E-08	-1.54E-08	-1.72E-08
-1.18E-08	-1.31E-08	-1.29E-08	-1.37E-08	-1.44E-08	-1.61E-08
-1.13E-08	-1.24E-08	-1.21E-08	-1.27E-08	-1.38E-08	-1.50E-08
-1.10E-08	-1.16E-08	-1.11E-08	-1.20E-08	-1.31E-08	-1.45E-08
-1.03E-08	-1.07E-08	-1.04E-08	-1.13E-08	-1.23E-08	-1.38E-08

-9.57E-09	-9.60E-09	-9.56E-09	-1.03E-08	-1.13E-08	-1.29E-08
-9.13E-09	-8.82E-09	-8.46E-09	-9.45E-09	-1.02E-08	-1.17E-08
-8.71E-09	-8.84E-09	-8.11E-09	-8.77E-09	-9.02E-09	-1.06E-08
-8.23E-09	-8.50E-09	-8.21E-09	-8.07E-09	-8.38E-09	-9.63E-09
-7.08E-09	-7.59E-09	-7.26E-09	-7.32E-09	-8.06E-09	-8.74E-09
-6.01E-09	-6.40E-09	-6.30E-09	-6.80E-09	-7.35E-09	-8.01E-09
-4.68E-09	-5.10E-09	-5.07E-09	-5.41E-09	-6.04E-09	-7.05E-09
-3.80E-09	-3.90E-09	-3.97E-09	-3.97E-09	-4.90E-09	-5.97E-09
-3.31E-09	-3.25E-09	-2.97E-09	-2.71E-09	-3.87E-09	-4.82E-09
-3.11E-09	-2.68E-09	-1.95E-09	-2.14E-09	-3.21E-09	-3.57E-09
-2.20E-09	-1.80E-09	-1.30E-09	-1.77E-09	-2.60E-09	-2.29E-09
-1.44E-09	-9.90E-10	-7.63E-10	-1.21E-09	-1.40E-09	-1.11E-09
-5.07E-10	-4.22E-10	4.87E-10	1.65E-10	-1.99E-12	6.68E-11
6.73E-10	9.50E-10	1.21E-09	1.44E-09	1.38E-09	1.29E-09
1.75E-09	2.17E-09	2.00E-09	3.16E-09	2.77E-09	2.39E-09
2.50E-09	2.62E-09	3.10E-09	4.63E-09	4.18E-09	3.79E-09
3.58E-09	3.62E-09	4.34E-09	4.99E-09	4.89E-09	5.06E-09
4.61E-09	5.29E-09	5.18E-09	6.06E-09	5.73E-09	6.40E-09
5.85E-09	6.58E-09	6.63E-09	7.49E-09	6.75E-09	7.43E-09
6.78E-09	7.47E-09	7.73E-09	8.42E-09	8.03E-09	8.69E-09
7.82E-09	8.46E-09	8.74E-09	9.17E-09	9.45E-09	1.03E-08
9.16E-09	9.58E-09	1.00E-08	1.05E-08	1.11E-08	1.22E-08
1.07E-08	1.11E-08	1.16E-08	1.23E-08	1.27E-08	1.39E-08
1.21E-08	1.26E-08	1.27E-08	1.35E-08	1.36E-08	1.52E-08
1.30E-08	1.38E-08	1.42E-08	1.45E-08	1.48E-08	1.65E-08
1.38E-08	1.48E-08	1.56E-08	1.56E-08	1.62E-08	1.80E-08
1.54E-08	1.63E-08	1.66E-08	1.70E-08	1.76E-08	1.99E-08
1.75E-08	1.78E-08	1.76E-08	1.83E-08	1.95E-08	2.14E-08
1.89E-08	1.89E-08	1.92E-08	1.93E-08	2.12E-08	2.32E-08
2.07E-08	2.00E-08	2.10E-08	2.11E-08	2.22E-08	2.43E-08
2.26E-08	2.21E-08	2.28E-08	2.29E-08	2.32E-08	2.62E-08
2.39E-08	2.38E-08	2.45E-08	2.45E-08	2.50E-08	2.83E-08
2.51E-08	2.50E-08	2.59E-08	2.62E-08	2.72E-08	3.04E-08
2.59E-08	2.61E-08	2.72E-08	2.77E-08	2.93E-08	3.20E-08
2.68E-08	2.73E-08	2.79E-08	2.87E-08	3.10E-08	3.38E-08
2.81E-08	2.85E-08	2.86E-08	3.04E-08	3.27E-08	3.58E-08
2.93E-08	3.00E-08	3.03E-08	3.18E-08	3.41E-08	3.78E-08
3.09E-08	3.19E-08	3.23E-08	3.35E-08	3.57E-08	3.92E-08
3.24E-08	3.37E-08	3.42E-08	3.49E-08	3.72E-08	4.08E-08
3.37E-08	3.57E-08	3.57E-08	3.66E-08	3.86E-08	4.26E-08
3.51E-08	3.71E-08	3.69E-08	3.81E-08	4.03E-08	4.47E-08
3.67E-08	3.85E-08	3.81E-08	3.95E-08	4.21E-08	4.71E-08
3.81E-08	3.97E-08	3.95E-08	4.07E-08	4.40E-08	4.89E-08
3.94E-08	4.07E-08	4.08E-08	4.26E-08	4.62E-08	5.05E-08
4.07E-08	4.17E-08	4.24E-08	4.42E-08	4.78E-08	5.24E-08
4.22E-08	4.31E-08	4.44E-08	4.55E-08	4.92E-08	5.43E-08
4.37E-08	4.49E-08	4.65E-08	4.69E-08	5.10E-08	5.64E-08
4.54E-08	4.68E-08	4.90E-08	4.88E-08	5.27E-08	5.81E-08
4.68E-08	4.84E-08	5.08E-08	5.07E-08	5.44E-08	6.00E-08
4.82E-08	4.99E-08	5.16E-08	5.24E-08	5.58E-08	6.21E-08

8.23E-17	6.90E-17	8.07E-17	7.76E-17	9.34E-17	9.65E-17
9.69E-17	8.37E-17	9.22E-17	1.01E-16	1.08E-16	8.12E-17
1.03E-16	9.68E-17	1.05E-16	1.12E-16	1.17E-16	6.35E-17
1.02E-16	1.05E-16	1.15E-16	1.18E-16	1.33E-16	8.75E-17
1.00E-16	1.14E-16	1.19E-16	1.39E-16	1.54E-16	1.54E-16
1.11E-16	1.25E-16	1.38E-16	1.52E-16	1.64E-16	1.81E-16
1.29E-16	1.38E-16	1.61E-16	1.73E-16	1.86E-16	2.20E-16
1.36E-16	1.58E-16	1.72E-16	1.88E-16	2.03E-16	2.45E-16
1.58E-16	1.58E-16	1.83E-16	2.06E-16	2.25E-16	2.63E-16
1.76E-16	1.73E-16	1.91E-16	2.12E-16	2.41E-16	2.80E-16
1.86E-16	1.91E-16	2.06E-16	2.28E-16	2.50E-16	2.85E-16
1.95E-16	2.13E-16	2.20E-16	2.31E-16	2.55E-16	2.97E-16
2.05E-16	2.25E-16	2.27E-16	2.29E-16	2.58E-16	3.04E-16
2.18E-16	2.33E-16	2.39E-16	2.30E-16	2.59E-16	3.19E-16
2.37E-16	2.42E-16	2.54E-16	2.41E-16	2.64E-16	3.32E-16
2.49E-16	2.54E-16	2.71E-16	2.58E-16	2.67E-16	3.47E-16
2.56E-16	2.61E-16	2.80E-16	2.64E-16	2.76E-16	3.67E-16
2.63E-16	2.74E-16	2.91E-16	2.70E-16	2.97E-16	3.77E-16
2.62E-16	2.85E-16	3.00E-16	2.95E-16	3.29E-16	3.94E-16
2.64E-16	2.94E-16	2.99E-16	3.19E-16	3.60E-16	4.11E-16
2.64E-16	2.94E-16	3.17E-16	3.34E-16	3.85E-16	4.08E-16
2.92E-16	3.01E-16	3.29E-16	3.41E-16	3.96E-16	4.13E-16
3.07E-16	3.10E-16	3.39E-16	3.53E-16	3.93E-16	4.19E-16
3.13E-16	3.32E-16	3.44E-16	3.68E-16	3.84E-16	4.34E-16
3.16E-16	3.44E-16	3.67E-16	3.83E-16	3.92E-16	4.58E-16
3.31E-16	3.80E-16	3.86E-16	3.90E-16	4.15E-16	4.93E-16
3.57E-16	3.99E-16	3.98E-16	4.01E-16	4.56E-16	5.32E-16
3.86E-16	4.16E-16	4.14E-16	4.08E-16	4.82E-16	5.65E-16
3.97E-16	4.20E-16	4.23E-16	4.29E-16	4.98E-16	6.11E-16
3.94E-16	4.25E-16	4.45E-16	4.57E-16	5.07E-16	6.39E-16
3.84E-16	4.12E-16	4.55E-16	4.80E-16	5.31E-16	6.66E-16

3.70E-16	4.00E-16	4.54E-16	4.90E-16	5.47E-16	6.98E-16
3.76E-16	4.13E-16	4.58E-16	4.99E-16	5.83E-16	7.34E-16
3.87E-16	4.33E-16	4.56E-16	5.17E-16	6.01E-16	7.51E-16
3.92E-16	4.55E-16	4.71E-16	5.29E-16	6.28E-16	7.68E-16
4.13E-16	4.56E-16	4.88E-16	5.36E-16	6.43E-16	7.82E-16
4.39E-16	4.67E-16	5.26E-16	5.56E-16	6.43E-16	8.11E-16
4.49E-16	4.73E-16	5.63E-16	5.79E-16	6.45E-16	8.41E-16
4.55E-16	5.02E-16	5.85E-16	6.05E-16	6.68E-16	8.67E-16
4.73E-16	5.11E-16	5.84E-16	5.97E-16	6.75E-16	8.82E-16
4.71E-16	5.08E-16	5.57E-16	5.79E-16	6.84E-16	8.80E-16
4.64E-16	5.10E-16	5.34E-16	5.94E-16	6.68E-16	8.80E-16
4.88E-16	4.99E-16	5.32E-16	6.21E-16	6.79E-16	9.02E-16
4.83E-16	4.75E-16	5.35E-16	6.48E-16	6.96E-16	9.19E-16
4.84E-16	4.81E-16	5.48E-16	6.27E-16	6.83E-16	8.99E-16
4.81E-16	4.88E-16	5.54E-16	6.16E-16	6.73E-16	8.69E-16
4.93E-16	5.17E-16	5.65E-16	6.39E-16	6.76E-16	8.60E-16
5.14E-16	5.28E-16	5.66E-16	6.36E-16	6.77E-16	8.49E-16
5.10E-16	5.32E-16	5.62E-16	6.17E-16	6.87E-16	8.44E-16
4.82E-16	5.18E-16	5.60E-16	6.04E-16	6.88E-16	8.55E-16
4.63E-16	5.09E-16	5.51E-16	6.10E-16	6.89E-16	8.52E-16
4.60E-16	5.07E-16	5.37E-16	6.10E-16	6.79E-16	8.33E-16
4.48E-16	5.07E-16	5.42E-16	5.98E-16	6.85E-16	8.24E-16
4.53E-16	4.88E-16	5.41E-16	5.87E-16	6.73E-16	8.02E-16
4.35E-16	4.74E-16	5.34E-16	5.75E-16	6.46E-16	7.75E-16
4.32E-16	4.86E-16	5.26E-16	5.52E-16	6.19E-16	7.66E-16
4.31E-16	4.83E-16	5.05E-16	5.25E-16	6.17E-16	7.73E-16
4.06E-16	4.60E-16	4.89E-16	5.08E-16	6.16E-16	7.71E-16
4.03E-16	4.47E-16	4.68E-16	5.08E-16	6.10E-16	7.47E-16
4.09E-16	4.36E-16	4.48E-16	4.96E-16	5.69E-16	7.05E-16
3.89E-16	4.18E-16	4.32E-16	4.68E-16	5.31E-16	6.69E-16
3.67E-16	3.97E-16	4.13E-16	4.32E-16	4.94E-16	6.28E-16
3.38E-16	3.69E-16	4.03E-16	4.05E-16	4.76E-16	5.73E-16
3.17E-16	3.62E-16	3.83E-16	3.76E-16	4.46E-16	5.31E-16
3.08E-16	3.55E-16	3.57E-16	3.58E-16	4.17E-16	5.15E-16
3.03E-16	3.31E-16	3.10E-16	3.35E-16	4.07E-16	4.84E-16
2.84E-16	2.96E-16	2.68E-16	3.30E-16	3.96E-16	4.56E-16
2.43E-16	2.73E-16	2.92E-16	3.23E-16	3.80E-16	4.46E-16
2.19E-16	2.48E-16	2.81E-16	2.97E-16	3.52E-16	4.27E-16
2.01E-16	2.26E-16	2.52E-16	2.85E-16	3.31E-16	4.01E-16
1.98E-16	2.26E-16	2.38E-16	2.61E-16	3.08E-16	3.74E-16
1.71E-16	2.11E-16	2.17E-16	2.38E-16	2.89E-16	3.42E-16
1.57E-16	1.97E-16	2.00E-16	2.15E-16	2.69E-16	3.29E-16
1.54E-16	1.82E-16	1.85E-16	2.08E-16	2.37E-16	2.96E-16
1.39E-16	1.72E-16	1.67E-16	1.87E-16	2.08E-16	2.58E-16
1.28E-16	1.54E-16	1.45E-16	1.60E-16	1.90E-16	2.26E-16
1.20E-16	1.35E-16	1.23E-16	1.44E-16	1.72E-16	2.10E-16
1.06E-16	1.14E-16	1.08E-16	1.27E-16	1.50E-16	1.90E-16
9.17E-17	9.21E-17	9.14E-17	1.07E-16	1.29E-16	1.67E-16
8.33E-17	7.78E-17	7.16E-17	8.94E-17	1.05E-16	1.36E-16
7.58E-17	7.81E-17	6.58E-17	7.68E-17	8.14E-17	1.13E-16
6.78E-17	7.23E-17	6.74E-17	6.51E-17	7.02E-17	9.26E-17
5.01E-17	5.75E-17	5.27E-17	5.35E-17	6.50E-17	7.65E-17
3.61E-17	4.09E-17	3.96E-17	4.63E-17	5.40E-17	6.42E-17
2.19E-17	2.60E-17	2.57E-17	2.93E-17	3.65E-17	4.97E-17
1.44E-17	1.52E-17	1.57E-17	1.58E-17	2.40E-17	3.57E-17
1.10E-17	1.06E-17	8.85E-18	7.36E-18	1.50E-17	2.33E-17
9.70E-18	7.20E-18	3.80E-18	4.59E-18	1.03E-17	1.27E-17
4.83E-18	3.22E-18	1.69E-18	3.12E-18	6.75E-18	5.24E-18
2.08E-18	9.80E-19	5.83E-19	1.45E-18	1.97E-18	1.23E-18
2.57E-19	1.78E-19	2.37E-19	2.74E-20	3.96E-24	4.47E-21
4.53E-19	9.02E-19	1.47E-18	2.07E-18	1.90E-18	1.67E-18
3.07E-18	4.70E-18	3.99E-18	1.00E-17	7.66E-18	5.70E-18
6.24E-18	6.89E-18	9.59E-18	2.14E-17	1.75E-17	1.44E-17
1.28E-17	1.31E-17	1.89E-17	2.49E-17	2.39E-17	2.56E-17
2.13E-17	2.80E-17	2.68E-17	3.67E-17	3.28E-17	4.10E-17
3.43E-17	4.33E-17	4.39E-17	5.61E-17	4.55E-17	5.52E-17
4.60E-17	5.58E-17	5.98E-17	7.09E-17	6.44E-17	7.55E-17
6.11E-17	7.16E-17	7.64E-17	8.40E-17	8.93E-17	1.05E-16
8.39E-17	9.18E-17	1.01E-16	1.10E-16	1.24E-16	1.50E-16
1.15E-16	1.24E-16	1.35E-16	1.51E-16	1.60E-16	1.93E-16
1.46E-16	1.60E-16	1.62E-16	1.81E-16	1.86E-16	2.32E-16
1.69E-16	1.91E-16	2.03E-16	2.09E-16	2.19E-16	2.72E-16
1.90E-16	2.19E-16	2.43E-16	2.44E-16	2.63E-16	3.25E-16
2.37E-16	2.67E-16	2.77E-16	2.90E-16	3.10E-16	3.96E-16
3.05E-16	3.16E-16	3.11E-16	3.35E-16	3.79E-16	4.59E-16
3.56E-16	3.56E-16	3.70E-16	3.74E-16	4.50E-16	5.37E-16
4.28E-16	4.01E-16	4.39E-16	4.45E-16	4.92E-16	5.90E-16
5.09E-16	4.87E-16	5.21E-16	5.25E-16	5.40E-16	6.87E-16
5.69E-16	5.68E-16	6.00E-16	6.01E-16	6.27E-16	8.01E-16
6.28E-16	6.24E-16	6.71E-16	6.88E-16	7.40E-16	9.26E-16
6.72E-16	6.79E-16	7.42E-16	7.70E-16	8.59E-16	1.03E-15
7.17E-16	7.46E-16	7.76E-16	8.26E-16	9.61E-16	1.14E-15
7.91E-16	8.10E-16	8.17E-16	9.22E-16	1.07E-15	1.28E-15
8.61E-16	9.01E-16	9.21E-16	1.01E-15	1.16E-15	1.43E-15
9.56E-16	1.02E-15	1.04E-15	1.13E-15	1.27E-15	1.54E-15
1.05E-15	1.13E-15	1.17E-15	1.22E-15	1.38E-15	1.67E-15

1.14E-15	1.28E-15	1.28E-15	1.34E-15	1.49E-15	1.81E-15	
1.23E-15	1.38E-15	1.36E-15	1.45E-15	1.62E-15	2.00E-15	
1.35E-15	1.48E-15	1.45E-15	1.56E-15	1.77E-15	2.22E-15	
1.45E-15	1.57E-15	1.56E-15	1.66E-15	1.94E-15	2.39E-15	
1.55E-15	1.65E-15	1.67E-15	1.81E-15	2.13E-15	2.55E-15	
1.65E-15	1.74E-15	1.80E-15	1.95E-15	2.28E-15	2.74E-15	
1.78E-15	1.86E-15	1.97E-15	2.07E-15	2.42E-15	2.95E-15	
1.91E-15	2.02E-15	2.16E-15	2.20E-15	2.60E-15	3.18E-15	
2.06E-15	2.19E-15	2.40E-15	2.38E-15	2.78E-15	3.37E-15	
2.19E-15	2.34E-15	2.58E-15	2.57E-15	2.95E-15	3.60E-15	
2.32E-15	2.49E-15	2.67E-15	2.74E-15	3.11E-15	3.86E-15	
5.25E-14	5.62E-14	5.92E-14	6.28E-14	7.14E-14	8.73E-14	2.85E-12

Primary Data cut into 4 quadrants

AFM of PLA + Van Height Retrace (Image 0013)

Quadrant 1 Data (16384 values):

-9.26E-09	-8.41E-09	2.04E-09	-1.62E-09	-9.16E-09	-7.48E-09	-5.44E-09	-8.63E-09	-7.36E-09	-5.48E-09	-5.96E-09	-4.85E-09	-6.00E-09	-3.18E-09	-2.45E-09	-4.88E-09	-3.20E-09	-3.44E-09	-2.74E-09	-3.01E-09	-6.56E-09
-1.04E-08	-8.24E-09	-4.75E-09	-7.71E-09	-9.35E-09	-5.97E-09	-4.94E-09	-8.68E-09	-6.06E-09	-4.90E-09	-6.45E-09	-4.92E-09	-5.94E-09	-2.80E-09	-1.47E-09	-5.03E-09	-3.38E-09	-2.54E-09	-3.66E-09	-2.89E-09	-6.27E-09
-9.43E-09	-7.30E-09	-6.04E-09	-7.95E-09	-7.97E-09	-3.39E-09	-6.08E-09	-8.27E-09	-6.14E-09	-4.27E-09	-6.69E-09	-5.11E-09	-4.76E-09	-3.67E-09	-2.41E-10	-3.90E-09	-3.98E-09	-2.38E-09	-3.70E-09	-4.15E-09	-6.32E-09
-8.21E-09	-9.45E-09	-6.24E-09	-7.24E-09	-7.80E-09	-3.56E-09	-6.66E-09	-7.97E-09	-7.71E-09	-6.04E-09	-5.80E-09	-4.41E-09	-3.37E-09	-2.74E-09	-1.03E-09	-3.66E-09	-4.36E-09	-3.07E-09	-3.15E-09	-3.57E-09	-6.41E-09
-7.36E-09	-1.10E-08	-7.03E-09	-6.95E-09	-8.78E-09	-6.20E-09	-8.06E-09	-7.99E-09	-7.69E-09	-5.83E-09	-5.28E-09	-3.12E-09	-2.24E-09	-1.90E-09	-3.48E-09	-4.07E-09	-3.92E-09	-3.61E-09	-2.87E-09	-2.27E-09	-7.05E-09
-6.42E-09	-1.15E-08	-8.50E-09	-6.48E-09	-6.49E-09	-5.36E-09	-7.98E-09	-7.38E-09	-6.21E-09	-4.71E-09	-5.95E-09	-2.76E-09	-6.09E-10	8.70E-12	-3.47E-09	-3.17E-09	-2.84E-09	-2.94E-09	-1.82E-09	-2.74E-09	-7.30E-09
-6.04E-09	-1.00E-08	-7.85E-09	-5.09E-09	-5.16E-09	-5.54E-09	-7.58E-09	-6.06E-09	-6.27E-09	-4.72E-09	-6.02E-09	-2.29E-09	2.74E-09	1.77E-09	-3.62E-09	-1.27E-09	-2.47E-09	-1.98E-09	-8.76E-10	-3.79E-09	-7.37E-09
-7.41E-09	-8.34E-09	-6.38E-09	-3.74E-09	-4.47E-09	-6.98E-09	-7.90E-09	-4.80E-09	-7.62E-09	-5.96E-09	-4.75E-09	-1.61E-09	6.72E-09	4.46E-09	-2.71E-09	-1.03E-09	-1.34E-09	-1.05E-09	6.01E-10	-3.65E-09	-6.51E-09
-6.94E-09	-7.23E-09	-5.66E-09	-3.04E-09	-2.16E-09	-6.55E-09	-7.79E-09	-4.21E-09	-7.39E-09	-5.55E-09	-4.09E-09	-1.73E-09	5.83E-09	4.69E-09	-1.39E-09	-2.55E-09	-5.04E-10	5.59E-10	2.20E-09	-2.72E-09	-5.57E-09
-6.23E-09	-6.74E-09	-4.42E-09	-2.94E-09	-1.72E-09	-5.95E-09	-6.82E-09	-4.75E-09	-7.28E-09	-5.73E-09	-4.60E-09	-3.85E-09	1.10E-09	9.04E-10	-9.96E-10	-2.44E-09	-2.81E-10	3.54E-09	2.17E-09	-2.04E-09	-4.54E-09
-7.05E-09	-6.31E-09	-3.29E-09	-4.46E-09	-5.48E-09	-6.66E-09	-6.19E-09	-4.27E-09	-7.05E-09	-6.52E-09	-4.40E-09	-4.24E-09	-8.02E-10	-1.47E-09	-8.38E-10	-2.02E-09	-2.56E-12	7.03E-09	2.74E-09	-1.02E-09	-4.47E-09
-7.10E-09	-5.75E-09	-4.08E-09	-4.94E-09	-7.84E-09	-5.93E-09	-5.80E-09	-6.30E-09	-7.56E-09	-5.24E-09	-2.34E-09	-3.42E-09	-9.55E-12	-9.41E-10	4.79E-10	-2.16E-09	2.84E-10	6.33E-09	2.03E-09	-6.08E-10	-5.39E-09
-6.40E-09	-5.58E-09	-5.23E-09	-4.91E-09	-8.03E-09	-4.98E-09	-5.10E-09	-6.75E-09	-6.71E-09	-3.65E-09	-1.05E-09	-2.18E-09	-1.58E-09	-1.75E-09	1.99E-09	-2.14E-09	-7.42E-10	1.46E-09	9.79E-10	-7.90E-10	-5.55E-09
-5.39E-09	-6.21E-09	-5.49E-09	-4.62E-09	-7.54E-09	-5.11E-09	-4.24E-09	-5.38E-09	-4.94E-09	-3.11E-09	-2.25E-09	-1.26E-09	-2.56E-09	-2.79E-09	2.18E-09	-1.66E-09	-1.33E-09	2.73E-10	1.00E-09	-7.12E-10	-5.50E-09
-5.34E-09	-5.66E-09	-5.54E-09	-3.71E-09	-6.45E-09	-4.71E-09	-2.67E-09	-5.55E-09	-2.64E-09	-4.20E-09	-3.73E-09	-3.74E-10	-1.33E-09	-3.12E-09	1.09E-09	-9.97E-10	-4.06E-10	1.51E-09	3.06E-10	3.03E-10	-5.36E-09
-5.70E-09	-4.37E-09	-4.74E-09	-2.33E-09	-5.33E-09	-4.78E-09	-2.32E-10	-5.33E-09	-8.49E-10	-3.43E-09	-2.56E-09	-7.12E-10	-2.32E-09	-1.57E-09	1.95E-09	-1.19E-09	1.06E-09	3.03E-09	-8.59E-10	1.11E-09	-5.04E-09
-5.82E-09	-4.85E-09	-4.08E-09	-2.18E-09	-4.50E-09	-4.04E-09	1.02E-09	-4.00E-09	-1.69E-09	-1.75E-09	-6.79E-10	3.39E-10	-3.27E-09	1.70E-10	2.71E-09	-1.02E-09	4.65E-10	2.47E-09	-2.71E-10	1.85E-09	-5.04E-09
-5.69E-09	-6.50E-09	-4.80E-09	-3.09E-09	-3.57E-09	-2.61E-09	1.37E-09	-3.06E-09	-2.21E-09	-7.69E-10	4.33E-10	1.08E-09	-3.39E-09	9.11E-10	2.70E-09	9.59E-11	-3.50E-10	2.47E-09	4.14E-10	2.35E-10	-4.83E-09
-5.14E-09	-6.50E-09	-4.25E-09	-2.48E-09	-2.03E-09	-8.24E-10	2.39E-09	-2.28E-09	-1.70E-09	-1.45E-09	-7.77E-11	-4.39E-10	-2.63E-09	1.23E-09	3.96E-09	1.19E-09	7.16E-10	3.54E-09	1.30E-10	-1.80E-09	-4.80E-09
-3.63E-09	-5.44E-09	-3.91E-09	-1.45E-09	-5.19E-10	7.49E-11	3.48E-09	-6.65E-10	-4.60E-10	-7.03E-10	6.39E-10	-1.84E-10	-1.93E-09	9.93E-10	4.59E-09	2.89E-09	2.70E-09	2.29E-09	-1.30E-10	-2.16E-09	-4.00E-09
-3.14E-09	-5.18E-09	-4.71E-09	-1.09E-09	-1.65E-10	6.66E-10	4.10E-09	1.45E-10	1.86E-10	1.72E-09	1.60E-09	5.56E-10	-2.05E-09	9.43E-10	6.34E-09	5.90E-09	5.09E-09	1.87E-09	-5.05E-11	-3.43E-09	-3.34E-09
-4.99E-09	-5.27E-09	-4.60E-09	-1.61E-09	-3.97E-10	3.47E-10	2.84E-09	2.30E-10	1.07E-09	4.51E-09	-3.76E-11	-6.38E-10	-2.37E-09	2.07E-10	7.87E-09	1.04E-08	9.49E-09	3.36E-09	2.54E-11	-3.75E-09	-5.08E-09
-5.80E-09	-4.49E-09	-3.93E-09	-2.07E-09	-5.43E-10	4.11E-10	2.14E-09	9.03E-10	2.15E-09	5.40E-09	-3.59E-10	-1.53E-09	-1.51E-09	-1.84E-09	3.81E-09	1.22E-08	1.40E-08	5.77E-09	-5.04E-11	-2.95E-09	-7.48E-09
-5.37E-09	-3.49E-09	-4.11E-09	-2.08E-09	-1.23E-09	3.56E-10	2.63E-09	2.33E-09	2.98E-09	3.92E-09	3.07E-10	-5.14E-10	1.31E-10	-3.40E-09	-2.23E-10	6.77E-09	1.22E-08	7.74E-09	2.54E-11	-2.48E-09	-8.07E-09
-5.42E-09	-3.71E-09	-4.92E-09	-1.56E-09	-1.43E-09	-3.29E-10	3.23E-09	4.07E-09	3.64E-09	4.84E-09	1.19E-09	1.76E-09	7.81E-10	-3.01E-09	-1.21E-09	1.15E-10	5.70E-09	6.98E-09	4.41E-11	-2.96E-09	-8.11E-09
-5.25E-09	-4.35E-09	-4.64E-09	-1.32E-09	-8.56E-10	-2.19E-10	3.32E-09	4.05E-09	4.27E-09	4.54E-09	2.08E-09	1.26E-09	1.55E-10	-2.57E-09	-1.46E-09	-9.98E-10	-6.83E-10	2.62E-09	1.17E-10	-2.66E-09	-7.56E-09
-4.72E-09	-4.50E-09	-3.87E-09	-2.39E-09	1.70E-10	5.93E-10	3.26E-09	3.44E-09	5.11E-09	3.37E-09	1.75E-09	-3.42E-10	-1.72E-09	-3.00E-09	-1.21E-09	-8.19E-10	-1.06E-09	5.37E-10	4.68E-11	-1.91E-09	-7.28E-09
-3.92E-09	-4.61E-09	-3.25E-09	-2.09E-09	1.60E-09	2.21E-09	3.81E-09	3.28E-09	4.34E-09	1.09E-09	6.11E-10	-1.99E-09	-2.18E-09	-3.68E-09	-1.23E-09	-1.49E-10	-6.38E-10	6.09E-10	-9.17E-10	-1.55E-09	-7.24E-09
-2.81E-09	-4.11E-09	-2.96E-09	-3.84E-10	4.07E-09	3.41E-09	3.97E-09	3.08E-09	3.05E-09	-7.51E-10	-1.03E-09	-3.20E-09	-1.98E-09	-2.99E-09	-1.47E-09	4.31E-10	-1.21E-09	-5.39E-10	-1.90E-09	-2.30E-09	-6.67E-09
-2.38E-09	-3.87E-09	-1.58E-09	-7.78E-11	6.34E-09	3.77E-09	2.87E-09	2.14E-09	2.62E-09	-1.38E-09	-1.09E-09	-3.81E-09	-3.57E-09	-2.04E-09	-1.57E-09	-2.12E-10	-1.50E-09	-5.91E-10	-1.86E-09	-3.43E-09	-5.48E-09
-2.00E-09	-3.81E-09	2.27E-11	-8.61E-12	6.96E-09	3.31E-09	1.07E-09	1.84E-09	2.03E-09	-1.59E-09	-1.81E-09	-3.64E-09	-3.44E-09	-2.44E-09	-2.21E-09	-1.18E-09	-1.88E-09	-7.32E-10	-1.36E-09	-2.94E-09	-5.20E-09
-1.43E-09	-3.18E-09	3.67E-10	-1.28E-10	4.53E-09	1.13E-09	-5.61E-10	2.62E-09	8.74E-10	-1.66E-09	-1.99E-09	-2.83E-09	-2.05E-09	-3.22E-09	-3.90E-09	-1.46E-09	-2.21E-09	-1.32E-09	-6.84E-10	-9.73E-10	-5.08E-09
-2.43E-09	-3.05E-09	-3.11E-10	3.55E-11	2.34E-09	-4.66E-10	-1.78E-09	2.77E-09	-1.40E-10	-2.67E-09	-2.56E-09	-2.78E-09	-1.38E-09	-1.87E-09	-5.05E-09	-1.26E-09	-3.01E-09	-1.76E-09	-8.23E-10	-4.22E-10	-4.71E-09
-2.19E-09	-3.06E-09	-3.61E-10	-5.80E-10	1.22E-10	-1.76E-09	-2.69E-09	-6.43E-11	-2.15E-09	-4.11E-09	-2.37E-09	-1.68E-09	-8.65E-10	-6.93E-10	-4.62E-09	-1.04E-09	-2.69E-09	-8.27E-10	-2.23E-10	-8.68E-10	-4.71E-09
-1.23E-09	-3.02E-09	2.90E-10	-1.86E-09	-1.63E-09	-1.90E-09	-3.37E-09	-3.47E-09	-4.14E-09	-6.75E-09	-2.95E-09	-8.95E-10	-2.22E-10	-4.60E-10	-4.88E-09	-1.34E-09	-1.06E-09	9.18E-10	3.50E-10	-1.64E-09	-5.48E-09
-5.93E-10	-2.59E-09	-4.59E-10	-4.04E-09	-2.64E-09	-1.50E-09	-4.32E-09	-4.30E-09	-5.85E-09	-8.03E-09	-2.51E-09	-1.59E-09	-2.58E-11	-6.59E-10	-4.36E-09	-1.16E-09	1.32E-10	1.30E-09	8.34E-10	-1.81E-09	-4.95E-09
-2.79E-10	-1.42E-09	-6.87E-10	-4.55E-09	-3.31E-09	-3.64E-10	-4.85E-09	-3.89E-09	-7.24E-09	-8.12E-09	-1.50E-09	-2.30E-09	-1.86E-09	-1.25E-09	-3.68E-09	-4.97E-10	3.68E-10	7.96E-10	1.26E-09	-2.44E-09	-5.19E-09
-7.85E-10	-4.63E-10	9.73E-10	-3.14E-09	-3.39E-09	1.19E-09	-4.54E-09	-5.35E-09	-6.39E-09	-6.86E-09	-2.19E-09	-2.40E-09	-2.90E-09	-8.28E-10	-2.48E-09	-4.98E-10	1.63E-09	3.80E-10	1.44E-09	-2.86E-09	-5.37E-09
-1.04E-09	-8.04E-12	2.12E-09	-2.12E-09	-3.45E-09	-3.78E-10	-4.63E-09	-5.38E-09	-5.10E-09	-5.66E-09	-3.02E-09	-3.03E-09	-3.21E-09	-1.57E-10	-8.56E-10	-2.38E-10	3.62E-09	1.77E-10	1.76E-09	-1.86E-09	-5.59E-09
-8.70E-10	-5.38E-10	1.78E-09	-2.25E-09	-2.32E-09	-3.03E-09	-4.85E-09	-5.00E-09	-4.15E-09	-3.95E-09	-4.16E-09	-4.17E-09	-1.57E-09	1.81E-10	1.05E-09	5.27E-10	5.58E-09	-2.28E-11	1.88E-09	-1.71E-09	-5.52E-09
-1.34E-09	-1.84E-10	1.37E-09	-1.98E-09	-1.86E-09	-4.74E-09	-4.49E-09	-4.36E-09	-3.21E-09	-3.65E-09	-4.97E-09	-3.54E-09	-1.06E-09	-2.80E-10	1.87E-09	1.45E-09	4.67E-09	-5.44E-10	3.14E-09	-2.11E-09	-4.99E-09
-4.08E-09	-2.66E-10	5.89E-10	-1.39E-09	-1.60E-09	-4.81E-09	-3.69E-09	-4.74E-09	-1.91E-09	-3.67E-09	-5.30E-09	-2.11E-09	-1.93E-09	-7.78E-10	1.51E-09	1.51E-09	3.64E-09	-2.66E-10	3.50E-09	-1.69E-09	-3.73E-09
-5.27E-09	-8.43E-10	-8.09E-10	-1.73E-09	-1.99E-09	-4.60E-09	-3.60E-09	-4.87E-09	-1.47E-09	-3.07E-09	-4.05E-09	-2.14E-09	-3.15E-09	-9.38E-10	1.64E-10	1.59E-09	2.89E-09	1.49E-10	3.12E-09	-1.35E-09	-2.27E-09
-4.70E-09	-1.92E-09	-1.98E-09	-2.08E-09	-2.14E-09	-3.47E-09	-4.20E-09	-3.69E-09	-7.00E-11	-2.44E-09	-2.42E-09	-2.52E-09	-3.00E-09	-8.26E-10	6.32E-10	1.42E-09	1.99E-09	3.04E-10	6.15E-10	-1.14E-09	4.52E-10
-3.54E-09	-1.69E-09	-1.81E-09	-1.70E-09	-2.46E-09	-1.95E-09	-3.32E-09	-2.22E-09	4.13E-09	-8.09E-10	-1.80E-09	-1.94E-09	-2.08E-09	-1.17E-09	6.13E-10	8.51E-10	1.80E-09	-4.47E-11	-3.35E-10	-1.76E-10	5.21E-09
-3.09E-09	-1.00E-09	-1.03E-09	-7.07E-10	-1.85E-09	-1.48E-09	-2.46E-09	-5.74E-10	1.03E-08	2.68E-09	-1.14E-09	-1.53E-09	-8.12E-10	-1.09E-09	5.59E-10	8.47E-10	7.61E-10	-5.98E-10	-2.10E-10	1.61E-09	1.28E-08
-2.63E-09	-1.51E-09	-1.01E-09	3.08E-10	-1.91E-09	-1.47E-09	-2.51E-09	-8.77E-11	1.49E-08	5.85E-09	4.20E-10	-9.68E-10	-9.11E-10	1.10E-10	5.88E-10	8.06E-10	-6.66E-11	-3.18E-10	5.76E-10	5.47E-09	2.08E-08
-1.65E-09	-2.29E-09	-2.12E-09	6.43E-10	-1.38E-09	-7.80E-10	-2.37E-09	-7.75E-10	9.37E-09	3.08E-09	1.71E-09	-1.06E-09	-6.26E-10	2.70E-10	8.51E-10	-1.78E-12	-2.72E-10	1.08E-09	1.64E-09	1.07E-08	2.81E-08
-1.24E-09	-3.55E-09	-2.79E-09	-2.33E-10	-3.63E-10	-3.36E-10	-1.66E-09	-9.19E-10	1.99E-09	5.61E-10	1.48E-09	-8.84E-10	-5.48E-10	-6.99E-10	1.43E-09	-8.64E-10	6.23E-10	1.59E-09	2.50E-09	1.52E-08	3.35E-08
-1.36E-09	-3.99E-09	-1.45E-09	-5.92E-10	2.29E-10	-5.51E-10	-1.01E-09	-6.08E-10	-1.05E-09	7.07E-10	7.89E-10	-5.22E-10	-1.11E-09	-8.79E-10	1.16E-09	-8.73E-10	1.28E-09	1.68E-09	3.06E-09	1.71E-08	3.62E-08
-4.45E-10	-2.41E-09	-9.45E-10	-2.26E-10	1.86E-10	-1.26E-09	-9.14E-10	-5.20E-10	-2.13E-09	9.70E-11	7.27E-10	-7.45E-10	-2.03E-09	-5.99E-10	4.20E-10	-3.51E-10	8.45E-10	1.32E-09	3.07E-09	1.72E-08	3.68E-08
-1.06E-09	-1.52E-09	-1.74E-09	-2.41E-10	1.90E-10	-1.47E-09	-2.01E-09	-1.10E-09	-2.90E-09	-1.09E-09	-8.32E-11	-1.11E-09	-2.08E-09	-4.55E-10	5.80E-10	-9.59E-10	4.67E-11	3.35E-10	2.59E-09	1.67E-08	3.51E-08
-1.45E-09	-5.80E-10	-1.69E-09	2.30E-10	3.95E-10	-1.20E-09	-2.82E-09	-2.09E-09	-3.20E-09	-1.39E-09	-1.31E-09	-1.64E-09	-1.89E-09	-2.35E-10	-3.43E-11	-1.97E-09	-6.68E-10	-9.39E-10	1.76E-09	1.45E-08	3.07E-08
-3.00E-10	-4.52E-10	-1.39E-09	6.02E-11	8.76E-10	-1.53E-09	-3.15E-09	-1.94E-09	-3.85E-09	-1.17E-09	-2.44E-09	-1.66E-09	-1.26E-09	-2.56E-10	-7.54E-10	-1.95E-09	-1.05E-09	-1.50E-09	6.82E-10	1.02E-08	2.48E-08
9.57E-10	5.39E-10	-8.57E-10	-4.08E-10	9.91E-10	-1.40E-09	-3.00E-09	-2.76E-09	-3.93E-09	-1.88E-09	-1.96E-09	-1.26E-09	-7.68E-10	-6.88E-10	-1.33E-09	-1.81E-09	-1.70E-09	-2.19E-09	-4.91E-10	5.52E-09	1.83E-08

1.97E-10	1.59E-09	-8.10E-10	-1.76E-09	-9.08E-10	-7.05E-10	-3.89E-09	-3.73E-09	-3.83E-09	-2.96E-09	-8.20E-10	-3.88E-10	-7.47E-10	-4.23E-10	-1.76E-09	-2.45E-09	-2.47E-09	-2.84E-09	-1.29E-09	1.92E-09	1.17E-08
6.58E-10	2.74E-09	-2.14E-09	-2.02E-09	-1.05E-09	5.68E-10	-4.76E-09	-2.03E-09	-3.56E-09	-2.66E-09	4.52E-10	-1.55E-10	-7.80E-10	-3.84E-10	-1.61E-09	-3.20E-09	-2.63E-09	-2.97E-09	-1.05E-09	-2.22E-10	5.98E-09
2.14E-09	4.42E-09	-1.84E-09	-1.46E-09	-5.66E-10	-2.06E-10	-4.71E-09	5.71E-10	-2.89E-09	-2.11E-09	7.74E-10	3.17E-10	2.24E-10	-6.24E-10	-1.27E-09	-3.08E-09	-1.94E-09	-2.73E-09	-4.51E-10	-8.97E-10	1.37E-09
1.72E-09	3.90E-09	-9.87E-10	-8.61E-10	-3.62E-10	-2.50E-09	-3.58E-09	9.03E-10	-2.53E-09	-1.99E-09	-6.92E-10	1.02E-09	1.83E-09	-4.05E-10	-4.82E-10	-2.19E-09	-1.87E-09	-2.46E-09	7.60E-11	-2.62E-10	-2.56E-09
4.58E-10	9.80E-10	-1.55E-09	-9.39E-10	-8.33E-10	-2.40E-09	-2.57E-09	-1.92E-09	-3.97E-09	-2.31E-09	-1.62E-09	6.86E-10	2.41E-09	-9.11E-10	-1.15E-09	-1.79E-09	-2.16E-09	-2.69E-09	-1.10E-10	1.07E-10	-4.80E-09
1.75E-09	9.27E-10	-1.90E-09	-6.39E-10	-1.29E-09	-2.55E-09	-2.64E-09	-2.38E-09	-4.68E-09	-2.27E-09	-1.94E-09	-8.09E-10	1.91E-10	-1.18E-09	-2.07E-09	-1.23E-09	-2.19E-09	-2.82E-09	-9.77E-10	7.60E-11	-5.05E-09
3.30E-09	2.18E-09	-2.46E-09	-4.18E-10	-1.10E-09	-3.15E-09	-2.87E-09	-2.67E-10	-4.13E-09	-1.57E-09	-2.44E-09	-1.23E-09	-9.41E-10	-1.01E-09	-1.66E-09	-5.45E-10	-2.35E-09	-2.19E-09	-1.40E-09	6.49E-10	-4.83E-09
5.64E-09	2.99E-09	-2.03E-09	-4.77E-10	-7.59E-10	-2.71E-09	-2.29E-09	1.45E-09	-2.81E-09	-1.79E-09	-3.63E-09	-6.71E-10	-1.07E-09	-1.30E-09	-1.15E-09	-8.03E-10	-2.53E-09	-1.57E-09	-1.29E-09	1.66E-09	-4.97E-09
6.42E-09	3.93E-09	-1.25E-09	-1.01E-09	-6.62E-10	-2.03E-09	-4.94E-10	2.43E-09	-1.58E-09	-1.55E-09	-3.65E-09	-7.86E-10	-9.49E-10	-2.18E-09	-2.14E-09	-1.63E-09	-3.19E-09	-1.49E-09	-1.17E-09	1.62E-10	-5.05E-09
4.31E-09	4.02E-09	-2.81E-10	-1.47E-09	-1.50E-10	-6.59E-10	-6.55E-10	1.25E-09	-2.11E-09	-6.36E-10	-3.11E-09	-8.80E-10	-1.41E-09	-2.58E-09	-3.03E-09	-1.66E-09	-3.71E-09	-2.05E-09	-2.14E-09	-1.91E-09	-4.80E-09
2.96E-09	3.47E-09	4.00E-10	-9.60E-10	4.58E-10	-2.63E-10	-1.37E-09	4.74E-10	-1.33E-09	2.23E-10	-1.92E-09	2.72E-10	-7.36E-10	-1.93E-09	-2.69E-09	-2.18E-09	-3.87E-09	-2.03E-09	-3.37E-09	-3.06E-09	-5.04E-09
2.84E-09	4.01E-09	6.36E-10	-2.98E-10	8.56E-10	-4.72E-10	-1.81E-09	6.62E-10	-2.02E-10	9.45E-10	-1.10E-09	1.74E-09	-4.42E-10	-3.14E-10	-2.27E-09	-2.69E-09	-3.52E-09	-2.38E-09	-3.03E-09	-3.57E-09	-5.64E-09
1.99E-09	5.35E-09	1.02E-09	-1.18E-10	5.93E-10	-5.82E-10	-3.83E-10	1.45E-09	-1.86E-10	1.05E-09	-4.15E-10	2.15E-09	-2.00E-10	5.25E-10	-2.44E-09	-1.91E-09	-3.50E-09	-2.75E-09	-1.77E-09	-3.38E-09	-6.03E-09
2.31E-09	7.03E-09	2.17E-09	-5.13E-10	2.72E-10	-2.03E-10	4.73E-10	1.20E-09	3.23E-10	5.60E-10	-5.02E-10	1.06E-09	-1.38E-10	4.71E-10	-2.77E-09	-1.11E-09	-3.59E-09	-2.26E-09	-1.56E-09	-3.25E-09	-6.15E-09
2.45E-09	5.60E-09	2.44E-09	-8.72E-10	3.16E-10	6.17E-10	-7.10E-10	8.54E-10	8.18E-10	1.00E-09	-6.34E-10	4.04E-10	-7.74E-10	1.14E-10	-2.52E-09	-1.28E-09	-3.09E-09	-1.42E-09	-2.03E-09	-3.16E-09	-6.16E-09
3.02E-09	2.68E-09	2.36E-09	1.68E-10	6.93E-10	-6.84E-11	-4.91E-10	1.54E-09	1.46E-09	1.06E-09	-3.21E-10	3.19E-10	-9.21E-10	5.48E-10	-2.16E-09	-1.41E-09	-2.43E-09	-9.66E-10	-2.00E-09	-2.89E-09	-6.13E-09
4.59E-09	2.60E-09	3.05E-09	4.00E-10	5.12E-10	-7.06E-10	4.71E-10	2.54E-09	2.43E-09	1.57E-09	2.77E-10	1.03E-09	-4.89E-10	3.35E-10	-1.77E-09	-1.66E-09	-1.50E-09	-7.38E-10	-1.16E-09	-2.44E-09	-6.28E-09
6.15E-09	4.24E-09	3.78E-09	-5.36E-10	3.85E-10	2.34E-10	1.52E-09	2.18E-09	2.59E-09	2.44E-09	5.09E-10	7.09E-10	2.44E-12	2.25E-10	-1.10E-09	-1.07E-09	-1.19E-09	-7.09E-10	-9.78E-10	-2.49E-09	-6.29E-09
9.03E-09	6.64E-09	4.59E-09	-1.10E-09	8.13E-11	9.00E-10	2.25E-09	2.00E-09	2.47E-09	2.93E-09	1.01E-09	9.43E-10	2.48E-10	-1.54E-09	-3.55E-10	-7.77E-10	-1.67E-09	-7.11E-10	-1.25E-09	-3.10E-09	-5.96E-09
1.26E-08	7.95E-09	2.80E-09	-1.32E-09	1.48E-10	8.31E-10	2.09E-09	2.00E-09	3.33E-09	4.14E-09	1.64E-09	1.94E-09	4.93E-10	-1.07E-09	8.08E-10	-1.53E-10	-1.91E-09	-5.31E-10	-1.44E-09	-3.06E-09	-5.53E-09
1.84E-08	9.22E-09	2.01E-10	-4.08E-10	7.90E-10	6.17E-10	2.85E-09	1.91E-09	4.27E-09	5.51E-09	2.52E-09	2.62E-09	7.06E-10	-6.28E-10	2.29E-09	9.89E-10	-1.68E-09	-9.41E-10	-8.74E-10	-2.83E-09	-5.18E-09
2.14E-08	1.16E-08	9.33E-10	1.14E-10	1.15E-09	9.49E-10	2.89E-09	1.85E-09	4.65E-09	4.64E-09	4.88E-09	3.91E-09	9.98E-10	-4.55E-10	2.50E-09	1.39E-09	-1.09E-09	-1.75E-09	-8.02E-11	-3.11E-09	-4.76E-09
1.78E-08	1.00E-08	1.11E-09	-2.49E-10	7.56E-10	9.24E-10	2.92E-09	2.16E-09	5.40E-09	5.51E-09	7.78E-09	4.92E-09	1.34E-09	-8.56E-10	7.56E-10	9.08E-11	-7.83E-10	-1.32E-09	1.52E-09	-8.75E-10	-3.78E-09
1.03E-08	4.88E-09	-2.68E-10	3.21E-10	1.25E-09	1.07E-09	3.18E-09	2.93E-09	7.29E-09	9.27E-09	1.01E-08	6.26E-09	2.32E-09	-2.69E-10	3.64E-10	3.84E-10	-1.07E-09	-1.43E-10	4.27E-09	2.87E-09	-1.78E-09
3.69E-09	2.81E-10	1.73E-10	1.59E-09	1.36E-09	8.66E-10	3.12E-09	2.84E-09	8.09E-09	1.17E-08	1.21E-08	8.14E-09	3.20E-09	7.74E-10	2.03E-10	1.07E-09	-5.50E-10	1.12E-09	8.19E-09	7.44E-09	8.41E-10
7.04E-10	-6.99E-10	4.92E-10	2.32E-09	1.04E-09	1.15E-09	3.01E-09	1.77E-09	7.46E-09	1.06E-08	1.25E-08	9.09E-09	4.32E-09	1.39E-09	2.62E-10	1.38E-09	-1.61E-11	2.81E-09	1.37E-08	1.21E-08	4.06E-09
1.38E-09	-2.93E-10	8.13E-10	1.38E-09	1.50E-09	1.72E-09	3.25E-09	1.35E-09	5.89E-09	7.78E-09	8.87E-09	7.98E-09	4.91E-09	1.55E-09	1.53E-09	7.97E-10	4.79E-10	4.90E-09	1.62E-08	1.27E-08	4.29E-09
9.71E-10	1.08E-09	1.31E-09	1.36E-10	1.49E-09	1.34E-09	2.81E-09	6.09E-10	4.90E-09	5.27E-09	5.16E-09	5.97E-09	5.23E-09	2.33E-09	2.90E-09	1.12E-09	7.52E-10	4.06E-09	1.10E-08	7.37E-09	-7.48E-10
4.79E-10	2.69E-09	3.67E-09	8.33E-10	7.13E-10	1.06E-09	2.28E-09	-3.37E-10	4.29E-09	3.17E-09	2.06E-09	4.16E-09	5.48E-09	3.40E-09	3.23E-09	2.92E-09	1.99E-09	2.16E-09	3.04E-09	-3.43E-10	-3.22E-09
5.14E-10	4.31E-09	5.23E-09	2.48E-09	8.93E-10	1.10E-09	9.45E-10	-1.36E-09	4.05E-09	1.60E-09	1.22E-09	3.51E-09	4.50E-09	3.58E-09	2.44E-09	4.33E-09	2.81E-09	3.31E-09	1.39E-09	-2.93E-09	-2.33E-09
9.04E-10	4.53E-09	3.88E-09	3.15E-09	1.93E-09	1.15E-09	-3.63E-10	-1.26E-09	3.20E-09	4.72E-10	1.11E-09	2.11E-09	3.02E-09	2.86E-09	2.01E-09	3.90E-09	1.78E-09	4.67E-09	3.86E-09	-2.38E-09	-3.10E-09
1.41E-09	3.78E-09	3.18E-09	3.11E-09	3.40E-09	1.86E-09	3.87E-10	-7.13E-10	3.54E-09	1.33E-09	9.55E-10	5.46E-10	2.31E-09	1.62E-09	1.90E-09	1.35E-09	1.13E-09	5.45E-09	4.02E-09	-9.56E-10	-4.44E-09
1.48E-09	3.43E-09	4.47E-09	3.23E-09	5.09E-09	3.02E-09	1.10E-09	-1.15E-10	2.16E-09	1.13E-09	5.15E-10	1.11E-09	2.68E-09	6.66E-10	2.18E-09	-2.93E-10	1.04E-09	2.91E-09	1.81E-10	4.09E-10	-3.61E-09
1.80E-09	3.13E-09	6.28E-09	4.88E-09	4.88E-09	4.09E-09	1.75E-09	1.15E-09	1.53E-09	5.18E-10	1.98E-12	1.06E-09	2.66E-09	1.04E-09	1.88E-09	-7.54E-11	1.62E-09	3.24E-10	-8.42E-10	7.24E-10	-2.87E-09
1.76E-09	3.05E-09	6.35E-09	7.25E-09	4.28E-09	3.91E-09	3.41E-09	3.17E-09	1.57E-09	1.69E-11	5.91E-10	4.61E-10	1.85E-09	9.05E-10	1.03E-09	7.54E-10	1.18E-09	-2.24E-10	3.41E-10	1.14E-10	-1.50E-09
2.38E-09	1.86E-09	3.69E-09	8.73E-09	4.78E-09	4.53E-09	5.51E-09	5.15E-09	1.23E-09	-5.72E-12	1.37E-09	2.80E-10	1.25E-09	5.97E-10	7.06E-10	7.70E-10	3.67E-10	-1.31E-12	6.12E-10	2.30E-10	-1.04E-09
3.43E-09	2.61E-09	3.73E-09	9.66E-09	6.02E-09	5.56E-09	8.69E-09	7.16E-09	1.15E-09	4.52E-10	1.30E-09	4.43E-10	1.53E-09	9.56E-10	9.19E-10	1.03E-11	-4.23E-10	-9.31E-10	-6.65E-10	8.36E-10	-9.50E-10
4.13E-09	3.08E-09	4.24E-09	9.93E-09	6.81E-09	7.67E-09	1.19E-08	1.03E-08	1.33E-09	2.24E-10	1.16E-09	-2.03E-10	1.23E-09	1.27E-09	2.12E-09	-3.98E-10	-1.32E-09	-1.64E-09	-1.46E-09	4.56E-10	-6.32E-11
4.01E-09	2.88E-09	3.74E-09	6.02E-09	5.93E-09	1.07E-08	1.70E-08	1.60E-08	2.15E-09	-1.80E-10	1.74E-09	-5.01E-10	1.08E-09	1.29E-09	2.65E-09	6.61E-10	-1.87E-09	-1.79E-09	-1.34E-09	-1.92E-09	-2.01E-09
2.34E-09	2.95E-09	3.87E-09	4.02E-09	6.26E-09	1.44E-08	2.36E-08	2.21E-08	2.39E-09	5.01E-11	1.32E-09	-4.27E-10	1.52E-09	1.07E-09	2.41E-09	1.59E-09	-1.44E-09	-1.23E-09	-9.72E-10	-2.70E-09	-3.24E-09
1.86E-09	3.68E-09	3.78E-09	3.48E-09	6.91E-09	1.61E-08	2.49E-08	2.18E-08	2.11E-09	8.67E-10	9.09E-10	-4.79E-10	1.21E-09	6.85E-10	2.62E-09	1.11E-09	-1.57E-09	-4.90E-10	-3.56E-10	-2.20E-09	-3.55E-09
2.35E-09	4.66E-09	4.12E-09	3.37E-09	5.16E-09	1.03E-08	1.16E-08	9.05E-09	1.56E-09	1.67E-09	1.30E-09	-9.09E-10	5.19E-10	6.70E-10	2.84E-09	1.01E-09	-1.33E-09	-4.78E-10	-1.26E-10	-2.73E-09	-4.74E-09
2.44E-09	5.46E-09	4.24E-09	3.21E-09	2.25E-09	2.21E-09	2.93E-09	2.80E-09	1.54E-09	1.48E-09	1.88E-09	-6.43E-10	1.56E-10	1.17E-09	3.21E-09	1.92E-09	-8.41E-10	-1.03E-09	-6.62E-10	-4.01E-09	-5.27E-09
1.90E-09	4.84E-09	3.88E-09	3.57E-09	1.81E-09	8.43E-10	2.50E-09	2.78E-09	2.41E-09	2.14E-09	1.67E-09	-4.22E-10	-5.02E-10	1.57E-09	2.54E-09	1.76E-09	-7.56E-10	-5.72E-10	-4.17E-10	-4.50E-09	-6.42E-09
2.07E-09	4.15E-09	3.47E-09	3.69E-09	2.08E-09	1.28E-09	3.65E-09	3.74E-09	3.59E-09	4.42E-09	1.96E-09	-4.02E-10	-1.33E-09	1.63E-09	3.48E-10	1.01E-09	-9.55E-10	-3.96E-10	-2.80E-10	-3.77E-09	-7.36E-09
2.40E-09	3.95E-09	3.25E-09	4.34E-09	2.81E-09	1.13E-09	1.18E-09	5.79E-09	2.82E-09	6.82E-09	2.19E-09	1.23E-10	-1.84E-09	1.15E-09	-2.63E-10	5.08E-10	-3.28E-10	-1.73E-10	-5.40E-11	-3.15E-09	-7.36E-09
2.09E-09	3.93E-09	3.23E-09	5.32E-09	2.79E-09	1.82E-09	4.45E-10	6.48E-09	3.95E-09	8.83E-09	2.56E-09	5.38E-10	-1.74E-09	1.14E-09	-3.91E-10	1.54E-09	2.53E-10	1.17E-10	-7.52E-10	-4.36E-09	-6.29E-09
1.41E-09	5.15E-09	3.40E-09	5.59E-09	3.15E-09	2.84E-09	8.44E-10	3.75E-09	5.45E-09	7.41E-09	3.29E-09	-4.77E-10	-1.30E-09	1.16E-09	-2.79E-10	1.31E-09	2.45E-10	2.44E-10	-1.02E-09	-4.35E-09	-4.45E-09
1.58E-09	6.04E-09	3.90E-09	4.35E-09	3.66E-09	2.47E-09	1.06E-09	2.70E-09	6.96E-09	8.37E-09	4.30E-09	1.09E-10	-9.70E-10	2.89E-09	-7.17E-10	5.47E-10	-1.02E-09	9.50E-10	1.93E-12	-2.63E-09	-1.33E-09
2.45E-09	5.85E-09	4.32E-09	3.14E-09	4.82E-09	2.97E-09	1.84E-09	2.73E-09	9.41E-09	1.10E-08	4.98E-09	5.33E-10	-1.21E-09	3.35E-09	1.80E-10	7.73E-10	-4.98E-10	1.79E-09	6.19E-10	-3.94E-10	4.42E-09
3.90E-09	5.51E-09	4.57E-09	2.81E-09	4.87E-09	3.68E-09	2.60E-09	3.05E-09	9.95E-09	1.14E-08	5.79E-09	1.54E-09	-7.41E-10	1.99E-10	1.33E-09	1.24E-09	-6.23E-10	1.44E-09	1.64E-10	1.98E-09	1.44E-08
4.57E-09	6.12E-09	4.97E-09	2.34E-09	4.54E-09	4.11E-09	1.84E-09	3.21E-09	4.99E-09	7.30E-09	7.48E-09	2.60E-09	-9.26E-11	-8.04E-10	2.42E-09	9.04E-10	-7.88E-10	1.01E-09	-2.45E-11	5.36E-09	2.71E-08
3.76E-09	5.51E-09	5.60E-09	2.62E-09	3.69E-09	4.12E-09	1.14E-09	2.30E-09	1.12E-09	5.62E-09	1.17E-08	4.37E-09	1.61E-09	5.35E-10	2.84E-09	6.14E-10	-1.60E-09	2.26E-10	1.23E-09	9.83E-09	3.85E-08
3.96E-09	4.13E-09	6.13E-09	3.15E-09	3.19E-09	3.55E-09	1.11E-09	2.52E-09	1.67E-09	7.29E-09	1.63E-08	6.63E-09	3.22E-09	1.56E-09	3.06E-09	2.54E-10	-2.61E-09	-7.87E-11	2.23E-09	1.37E-08	4.57E-08
4.22E-09	3.30E-09	6.20E-09	3.55E-09	3.97E-09	4.70E-09	1.68E-09	3.95E-09	2.34E-09	7.60E-09	1.68E-08	8.93E-09	4.45E-09	1.98E-09	2.83E-09	9.70E-10	-2.51E-09	-7.46E-10	3.20E-09	1.70E-08	5.01E-08
3.65E-09	4.39E-09	5.65E-09	4.49E-09	4.41E-09	5.26E-09	2.66E-09	3.96E-09	1.66E-09	4.84E-09	1.17E-08	1.18E-08	6.66E-09	2.50E-09	1.50E-09	1.50E-09	-2.24E-09	-1.27E-09	4.44E-09	1.71E-08	5.10E-08
4.48E-09	4.83E-09	6.11E-09	5.18E-09	5.15E-09	3.82E-09	2.77E-09	2.46E-09	1.19E-09	3.17E-09	1.12E-08	1.80E-08	8.02E-09	3.88E-09	2.39E-09	1.49E-09	-1.82E-09	-1.71E-09	3.94E-09	1.48E-08	4.78E-08
4.92E-09	3.84E-09	6.30E-09	5.99E-09	5.89E-09	3.49E-09	3.01E-09	3.02E-09	1.32E-09	4.40E-09	1.58E-08	2.11E-08	9.62E-09	5.97E-09	3.18E-09	8.35E-10	-9.60E-10	-1.99E-09	1.90E-09	1.14E-08	4.11E-08
3.80E-09	4.35E-09	5.71E-09	5.64E-09	6.27E-09	4.08E-09	4.48E-09	3.69E-09	1.08E-09	4.56E-09	1.85E-08	1.54E-08	7.17E-09	8.06E-09	3.57E-09	1.06E-09	7.51E-10	-1.73E-09	7.43E-10	8.15E-09	3.27E-08
3.66E-09	4.87E-09	6.05E-09	4.24E-09	5.42E-09	5.19E-09	5.38E-09	3.92E-09	9.66E-10	2.36E-09	1.29E-08	2.36E-08	8.32E-09	1.16E-08	3.62E-09	1.28E-09	1.89E-09	-1.28E-09	4.92E-10	5.02E-09	2.38E-08
4.42E-09	4.48E-09	6.79E-09	3.93E-09	5.42E-09	5.39E-09	4.64E-09	3.87E-09	1.88E-09	1.93E-09	3.32E-09	2.04E-09	1.20E-08	1.57E-08	4.79E-09	2.07E-09	3.19E-09	-9.87E-10	3.77E-10	1.92E-09	1.38E-08
4.07E-09	4.04E-09	7.09E-09	4.10E-09	5.66E-09	5.84E-09	3.23E-09	5.01E-09	3.09E-09	2.78E-09	2.04E-09	1.82E-09	1.18E-08	1.54E-08	5.63E-09	2.98E-09	5.92E-09	-1.96E-10	-5.87E-11	9.62E-11	4.74E-09
4.30E-09	4.18E-09	6.13E-09	3.99E-09	4.51E-09	4.58E-09	4.17E-09	4.77E-09	4.16E-09	3.75E-09	2.35E-09	2.17E-09	6.54E-09	8.31E-09	3.83E-09	2.95E-09	9.50E-09	1.64E-09	-4.08E-10	-1.68E-10	-1.01E-09

4.46E-09	4.28E-09	4.78E-09	4.27E-09	3.94E-09	3.71E-09	4.52E-09	4.26E-09	4.50E-09	4.18E-09	2.77E-09	2.58E-09	3.84E-09	4.33E-09	4.16E-09	5.73E-09	1.38E-08	3.15E-09	1.02E-10	-2.31E-10	-2.85E-09
3.72E-09	4.32E-09	4.68E-09	4.15E-09	3.40E-09	3.43E-09	3.57E-09	2.33E-09	5.40E-09	6.02E-09	2.67E-09	2.43E-09	3.33E-09	5.29E-09	4.84E-09	1.00E-08	1.89E-08	3.59E-09	7.67E-10	-3.75E-10	-3.51E-09
2.79E-09	4.77E-09	4.60E-09	3.99E-09	3.53E-09	3.22E-09	3.84E-09	2.18E-09	6.16E-09	6.37E-09	1.39E-09	1.58E-09	3.17E-09	7.15E-09	5.26E-09	1.52E-08	2.21E-08	3.55E-09	7.46E-10	-1.37E-10	-3.19E-09
3.43E-09	4.81E-09	5.02E-09	4.10E-09	3.52E-09	3.73E-09	4.92E-09	2.39E-09	4.68E-09	2.67E-09	1.30E-09	1.93E-09	3.90E-09	6.95E-09	3.75E-09	2.21E-08	2.58E-08	4.78E-09	7.30E-10	1.07E-09	-3.44E-09
3.86E-09	4.75E-09	4.20E-09	4.80E-09	3.03E-09	4.24E-09	4.56E-09	2.83E-09	3.28E-09	1.60E-09	1.23E-09	3.43E-09	4.15E-09	5.00E-09	3.32E-09	2.21E-08	1.96E-08	2.49E-09	1.65E-09	3.30E-09	-4.11E-09
3.05E-09	4.09E-09	3.07E-09	5.31E-09	2.96E-09	4.37E-09	3.31E-09	3.01E-09	2.04E-09	2.99E-09	1.05E-09	5.46E-09	4.03E-09	2.75E-09	2.99E-09	1.05E-08	4.82E-09	-4.74E-10	4.99E-09	5.72E-09	-4.28E-09
2.30E-09	3.58E-09	2.67E-09	4.32E-09	3.66E-09	4.42E-09	2.93E-09	3.15E-09	2.08E-09	3.61E-09	1.65E-09	6.25E-09	4.74E-09	2.96E-09	3.11E-09	1.75E-09	-6.81E-10	-3.28E-10	8.15E-09	7.28E-09	-3.69E-09
1.61E-09	4.10E-09	3.14E-09	3.49E-09	4.07E-09	4.26E-09	3.27E-09	3.18E-09	3.30E-09	2.03E-09	2.20E-09	6.41E-09	7.91E-09	3.39E-09	2.76E-09	1.58E-09	9.00E-11	7.41E-10	5.02E-09	4.63E-09	-4.06E-09
1.61E-09	3.89E-09	2.99E-09	2.95E-09	3.07E-09	3.49E-09	3.23E-09	2.62E-09	4.73E-09	1.49E-09	1.86E-09	8.13E-09	1.03E-08	1.89E-09	3.55E-09	1.97E-09	1.51E-09	2.26E-09	-6.89E-10	9.63E-10	-3.22E-09
2.01E-09	4.03E-09	2.85E-09	2.90E-09	2.31E-09	2.88E-09	2.66E-09	1.67E-09	4.78E-09	1.75E-09	3.85E-10	6.34E-09	7.80E-09	5.74E-10	4.06E-09	2.82E-09	2.43E-09	2.43E-09	-4.97E-10	1.92E-09	-9.07E-10

Quadrant 1 Data Squared (16384 values):

8.58E-17	7.08E-17	4.17E-18	2.62E-18	8.39E-17	5.59E-17	2.96E-17	7.44E-17	5.42E-17	3.01E-17	3.56E-17	2.36E-17	3.60E-17	1.01E-17	6.02E-18	2.38E-17	1.03E-17	1.18E-17	7.49E-18	9.05E-18	4.30E-17
1.07E-16	6.79E-17	2.26E-17	5.95E-17	8.75E-17	3.56E-17	2.44E-17	7.54E-17	3.67E-17	2.40E-17	4.16E-17	2.42E-17	3.53E-17	7.83E-18	2.16E-18	2.54E-17	1.15E-17	6.43E-18	1.34E-17	8.35E-18	3.93E-17
8.89E-17	5.34E-17	3.64E-17	6.33E-17	6.35E-17	1.15E-17	3.70E-17	6.84E-17	3.77E-17	1.82E-17	4.47E-17	2.62E-17	2.27E-17	1.34E-17	5.79E-20	1.52E-17	1.58E-17	5.65E-18	1.37E-17	1.72E-17	4.00E-17
6.74E-17	8.94E-17	3.90E-17	5.24E-17	6.09E-17	1.27E-17	4.44E-17	6.36E-17	5.95E-17	3.65E-17	3.37E-17	1.95E-17	1.13E-17	7.51E-18	1.06E-18	1.34E-17	1.90E-17	9.43E-18	9.93E-18	1.28E-17	4.10E-17
5.42E-17	1.22E-16	4.95E-17	4.84E-17	7.70E-17	3.84E-17	6.50E-17	6.39E-17	5.91E-17	3.40E-17	2.79E-17	9.74E-18	5.03E-18	3.60E-18	1.21E-17	1.66E-17	1.54E-17	1.31E-17	8.24E-18	5.14E-18	4.97E-17
4.12E-17	1.31E-16	7.22E-17	4.20E-17	4.22E-17	2.88E-17	6.38E-17	5.45E-17	3.86E-17	2.22E-17	3.55E-17	7.61E-18	3.70E-19	7.56E-23	1.20E-17	1.01E-17	8.06E-18	8.64E-18	3.31E-18	7.50E-18	5.33E-17
3.64E-17	1.00E-16	6.17E-17	2.59E-17	2.66E-17	3.07E-17	5.75E-17	3.67E-17	3.93E-17	2.23E-17	3.63E-17	5.23E-18	7.52E-18	3.13E-18	1.31E-17	1.61E-18	6.12E-18	3.94E-18	7.67E-19	1.44E-17	5.43E-17
5.49E-17	6.96E-17	4.07E-17	1.40E-17	2.00E-17	4.87E-17	6.25E-17	2.31E-17	5.80E-17	3.56E-17	2.25E-17	2.61E-18	4.52E-17	1.99E-17	7.33E-18	1.06E-18	1.79E-18	1.11E-18	3.61E-19	1.33E-17	4.24E-17
4.81E-17	5.22E-17	3.20E-17	9.23E-18	4.68E-18	4.29E-17	6.07E-17	1.77E-17	5.46E-17	3.08E-17	1.68E-17	2.98E-18	3.40E-17	2.20E-17	1.93E-18	6.50E-18	2.54E-19	3.13E-19	4.84E-18	7.42E-18	3.10E-17
3.88E-17	4.55E-17	1.95E-17	8.64E-18	2.94E-18	3.54E-17	4.65E-17	2.25E-17	5.30E-17	3.29E-17	2.12E-17	1.48E-17	1.21E-18	8.18E-19	9.92E-19	5.96E-18	7.90E-20	1.25E-17	4.72E-18	4.16E-18	2.06E-17
4.98E-17	3.98E-17	1.08E-17	1.98E-17	3.00E-17	4.44E-17	3.83E-17	1.83E-17	4.97E-17	4.26E-17	1.94E-17	1.80E-17	6.43E-19	2.17E-18	7.02E-19	4.10E-18	6.54E-24	4.94E-17	7.50E-18	1.05E-18	2.00E-17
5.04E-17	3.30E-17	1.67E-17	2.44E-17	6.15E-17	3.51E-17	3.36E-17	3.97E-17	5.71E-17	2.75E-17	5.47E-18	1.17E-17	9.12E-23	8.85E-19	2.29E-19	4.66E-18	8.06E-20	4.01E-17	4.13E-18	3.70E-19	2.91E-17
4.09E-17	3.12E-17	2.73E-17	2.41E-17	6.45E-17	2.48E-17	2.60E-17	4.56E-17	4.50E-17	1.33E-17	1.11E-18	4.73E-18	2.49E-18	3.07E-18	3.96E-18	4.59E-18	5.51E-19	2.14E-18	9.57E-19	6.24E-19	3.08E-17
2.91E-17	3.85E-17	3.02E-17	2.13E-17	5.69E-17	2.62E-17	1.80E-17	2.89E-17	2.45E-17	9.70E-18	5.07E-18	1.59E-18	6.55E-18	7.80E-18	4.75E-18	2.74E-18	1.76E-18	7.45E-20	1.00E-18	5.07E-19	3.03E-17
2.85E-17	3.20E-17	3.06E-17	1.37E-17	4.16E-17	2.22E-17	7.12E-18	3.08E-17	6.96E-18	1.76E-17	1.39E-17	1.40E-19	1.76E-18	9.74E-18	1.18E-18	9.93E-19	1.65E-19	2.28E-18	9.34E-20	9.17E-20	2.88E-17
3.25E-17	1.91E-17	2.25E-17	5.44E-18	2.84E-17	2.29E-17	5.38E-20	2.84E-17	7.21E-19	1.18E-17	6.56E-18	5.07E-19	5.36E-18	2.48E-18	3.81E-18	1.41E-18	1.13E-18	9.21E-18	7.38E-19	1.23E-18	2.54E-17
3.39E-17	2.36E-17	1.67E-17	4.74E-18	2.03E-17	1.63E-17	1.05E-18	1.60E-17	2.84E-18	3.05E-18	4.61E-19	1.15E-19	1.07E-17	2.88E-20	7.37E-18	1.04E-18	2.16E-19	6.10E-18	7.37E-20	3.44E-18	2.54E-17
3.23E-17	4.23E-17	2.31E-17	9.56E-18	1.28E-17	6.82E-18	1.87E-18	9.39E-18	4.90E-18	5.91E-19	1.87E-19	1.17E-18	1.15E-17	8.30E-19	7.27E-18	9.19E-21	1.22E-19	6.10E-18	1.71E-19	5.52E-20	2.33E-17
2.65E-17	4.22E-17	1.80E-17	6.15E-18	4.10E-18	6.78E-19	5.74E-18	5.18E-18	2.89E-18	2.10E-18	6.03E-21	1.93E-19	6.89E-18	1.51E-18	1.57E-17	1.41E-18	5.13E-19	1.25E-17	1.70E-20	3.23E-18	2.31E-17
1.32E-17	2.96E-17	1.53E-17	2.10E-18	2.69E-19	5.61E-21	1.21E-17	4.43E-19	2.12E-19	4.95E-19	4.09E-19	3.39E-20	3.72E-18	9.86E-19	2.10E-17	8.33E-18	7.31E-18	5.23E-18	1.70E-20	4.67E-18	1.60E-17
9.87E-18	2.69E-17	2.22E-17	1.18E-18	2.73E-20	4.44E-19	1.68E-17	2.11E-20	3.45E-20	2.95E-18	2.57E-18	3.09E-19	4.21E-18	8.89E-19	4.02E-17	3.48E-17	2.59E-17	3.50E-18	2.55E-21	1.17E-17	1.12E-17
2.49E-17	2.77E-17	2.11E-17	2.60E-18	1.57E-19	1.20E-19	8.05E-18	5.28E-20	1.15E-18	2.03E-17	1.41E-21	4.07E-19	5.60E-18	4.28E-20	6.20E-17	1.08E-16	9.01E-17	1.13E-17	6.43E-22	1.41E-17	2.58E-17
3.36E-17	2.02E-17	1.54E-17	4.29E-18	2.95E-19	1.69E-19	4.56E-18	8.16E-19	4.62E-18	2.91E-17	1.29E-19	2.34E-18	2.29E-18	3.37E-18	1.45E-17	1.49E-16	1.95E-16	3.33E-17	2.55E-21	8.69E-18	5.59E-17
2.88E-17	1.22E-17	1.69E-17	4.31E-18	1.51E-18	1.27E-19	6.93E-18	5.45E-18	8.87E-18	1.54E-17	9.43E-20	2.64E-19	1.72E-20	1.16E-17	4.96E-20	4.58E-17	1.48E-16	6.00E-17	6.46E-22	6.13E-18	6.51E-17
2.94E-17	1.37E-17	2.42E-17	2.43E-18	2.03E-18	1.08E-19	1.04E-17	1.65E-17	1.33E-17	2.34E-17	1.41E-18	3.11E-18	6.10E-19	9.04E-18	1.46E-18	1.33E-20	3.24E-17	4.87E-17	1.94E-21	8.74E-18	6.59E-17
2.75E-17	1.89E-17	2.15E-17	1.74E-18	7.33E-19	4.80E-20	1.10E-17	1.64E-17	1.82E-17	2.06E-17	4.31E-18	1.59E-18	2.42E-20	6.58E-18	2.12E-18	9.96E-19	4.66E-19	6.89E-18	1.37E-20	7.07E-18	5.72E-17
2.23E-17	2.03E-17	1.50E-17	5.70E-18	2.90E-20	3.52E-19	1.06E-17	1.18E-17	2.62E-17	1.13E-17	3.05E-18	1.17E-19	2.95E-18	8.97E-18	1.47E-18	6.70E-19	1.13E-18	2.88E-19	2.19E-21	3.66E-18	5.30E-17
1.54E-17	2.12E-17	1.06E-17	4.39E-18	2.58E-18	4.89E-18	1.45E-17	1.08E-17	1.88E-17	1.19E-18	3.73E-19	3.94E-18	4.75E-18	1.36E-17	1.51E-18	2.21E-20	4.07E-19	3.70E-19	8.42E-19	2.39E-18	5.24E-17
7.88E-18	1.69E-17	8.75E-18	1.47E-19	1.65E-17	1.16E-17	1.58E-17	9.46E-18	9.30E-18	5.64E-19	1.06E-18	1.02E-17	3.93E-18	8.97E-18	2.17E-18	1.86E-19	1.46E-18	2.90E-19	3.63E-18	5.30E-18	4.45E-17
5.68E-18	1.50E-17	2.50E-18	6.06E-21	4.02E-17	1.42E-17	8.24E-18	4.58E-18	6.87E-18	1.91E-18	1.20E-18	1.45E-17	1.27E-17	4.18E-18	2.46E-18	4.48E-20	2.24E-18	3.49E-19	3.47E-18	1.17E-17	3.01E-17
4.02E-18	1.45E-17	5.13E-22	7.42E-23	4.85E-17	1.10E-17	1.14E-18	3.40E-18	4.10E-18	2.53E-18	3.29E-18	1.33E-17	1.19E-17	5.98E-18	4.87E-18	1.40E-18	3.52E-18	5.35E-19	1.85E-18	8.62E-18	2.70E-17
2.04E-18	1.01E-17	1.35E-19	1.64E-20	2.05E-17	1.28E-18	3.15E-19	6.89E-18	7.64E-19	2.74E-18	3.97E-18	7.99E-18	4.21E-18	1.04E-17	1.52E-17	2.13E-18	4.90E-18	1.75E-18	4.68E-19	9.46E-19	2.58E-17
5.92E-18	9.33E-18	9.65E-20	1.26E-21	5.47E-18	2.17E-19	3.16E-18	7.66E-18	1.96E-20	7.10E-18	6.56E-18	7.73E-18	1.89E-18	3.51E-18	2.55E-17	1.58E-18	9.08E-18	3.11E-18	6.77E-19	1.78E-19	2.22E-17
4.80E-18	9.33E-18	1.30E-19	3.37E-19	1.50E-20	3.09E-18	7.21E-18	4.14E-21	4.63E-18	1.69E-17	5.60E-18	2.82E-18	7.48E-19	4.80E-19	2.13E-17	1.08E-18	7.23E-18	6.84E-19	4.97E-20	7.53E-19	2.22E-17
1.52E-18	9.11E-18	8.39E-20	3.44E-18	2.67E-18	3.63E-18	1.14E-17	1.20E-17	1.71E-17	4.55E-17	8.70E-18	8.00E-19	4.93E-20	2.11E-19	2.38E-17	1.80E-18	1.13E-18	8.43E-19	1.23E-19	2.70E-18	3.00E-17
3.52E-19	6.72E-18	2.10E-19	1.63E-17	6.95E-18	2.25E-18	1.87E-17	1.85E-17	3.43E-17	6.44E-17	6.32E-18	2.52E-18	6.66E-22	4.34E-19	1.90E-17	1.35E-18	1.74E-20	1.70E-18	6.96E-19	3.27E-18	2.45E-17
7.78E-20	2.02E-18	4.72E-19	2.07E-17	1.09E-17	1.32E-19	2.35E-17	1.51E-17	5.24E-17	6.60E-17	2.25E-18	5.28E-18	3.47E-18	1.56E-18	1.36E-17	2.47E-19	1.35E-19	6.34E-19	1.58E-18	5.94E-18	2.69E-17
6.16E-19	2.15E-19	9.46E-19	9.87E-18	1.15E-17	1.42E-18	2.06E-17	2.87E-17	4.08E-17	4.71E-17	4.82E-18	5.76E-18	8.41E-18	6.85E-19	6.14E-18	2.48E-19	2.67E-18	1.44E-19	2.07E-18	8.19E-18	2.88E-17
1.08E-18	6.47E-23	4.48E-18	4.50E-18	1.19E-17	1.43E-19	2.15E-17	2.89E-17	2.60E-17	3.20E-17	9.12E-18	9.19E-18	1.03E-17	2.47E-20	7.32E-19	5.65E-20	1.31E-17	3.12E-20	3.11E-18	3.47E-18	3.12E-17
7.57E-19	2.90E-19	3.16E-18	5.05E-18	5.37E-18	9.16E-18	2.36E-17	2.50E-17	1.72E-17	1.56E-17	1.73E-17	1.74E-17	2.45E-18	3.28E-20	1.10E-18	2.77E-19	3.11E-17	5.21E-22	3.55E-18	2.92E-18	3.04E-17
1.80E-18	3.39E-20	1.88E-18	3.91E-18	3.45E-18	2.25E-17	2.02E-17	1.90E-17	1.03E-17	1.33E-17	2.47E-17	1.26E-17	1.11E-18	7.83E-20	3.50E-18	2.10E-18	2.18E-17	2.96E-19	9.86E-18	4.46E-18	2.49E-17
1.67E-17	7.09E-20	3.48E-19	1.92E-18	2.55E-18	2.32E-17	1.36E-17	2.25E-17	3.64E-18	1.35E-17	2.81E-17	4.47E-18	3.73E-18	6.05E-19	2.28E-18	2.27E-18	1.32E-17	7.07E-20	1.23E-17	2.84E-18	1.39E-17
2.78E-17	7.11E-19	6.55E-19	3.00E-18	3.95E-18	2.11E-17	1.30E-17	2.37E-17	2.16E-18	9.45E-18	1.64E-17	4.59E-18	9.93E-18	8.81E-19	2.67E-20	2.52E-18	8.37E-18	2.22E-20	9.71E-18	1.84E-18	5.17E-18
2.21E-17	3.69E-18	3.91E-18	4.32E-18	4.57E-18	1.21E-17	1.76E-17	1.37E-17	4.90E-21	5.97E-18	5.86E-18	6.36E-18	8.98E-18	6.83E-19	3.99E-19	2.03E-18	3.96E-18	9.21E-20	3.79E-19	1.31E-18	2.05E-19
1.25E-17	2.87E-18	3.28E-18	2.88E-18	6.04E-18	3.81E-18	1.10E-17	4.94E-18	1.70E-17	6.54E-19	3.23E-18	3.78E-18	4.31E-18	1.36E-18	3.76E-19	7.25E-19	3.24E-18	2.00E-21	1.13E-19	3.11E-20	2.71E-17
9.57E-18	1.01E-18	1.06E-18	4.99E-19	3.41E-18	2.20E-18	6.03E-18	3.29E-19	1.07E-16	7.16E-18	1.29E-18	2.34E-18	6.60E-19	1.19E-18	3.12E-19	7.18E-19	5.80E-19	3.57E-19	4.43E-20	2.61E-18	1.65E-16
6.93E-18	2.29E-18	1.02E-18	9.50E-20	3.64E-18	2.17E-18	6.28E-18	7.69E-21	2.21E-16	3.42E-17	1.77E-19	9.38E-19	8.30E-19	1.21E-20	3.46E-19	6.49E-19	4.43E-21	1.01E-19	3.31E-19	2.99E-17	4.33E-16
2.71E-18	5.24E-18	4.50E-18	4.14E-19	1.91E-18	6.08E-19	5.63E-18	6.00E-19	8.78E-17	9.47E-18	2.93E-18	1.12E-18	3.92E-19	7.30E-20	7.23E-19	3.16E-24	7.41E-20	1.16E-18	2.69E-18	1.14E-16	7.88E-16

1.53E-18	1.26E-17	7.76E-18	5.42E-20	1.32E-19	1.13E-19	2.77E-18	8.44E-19	3.96E-18	3.15E-19	2.19E-18	7.81E-19	3.00E-19	4.88E-19	2.04E-18	7.46E-19	3.89E-19	2.53E-18	6.25E-18	2.30E-16	1.12E-15
1.84E-18	1.59E-17	2.11E-18	3.50E-19	5.24E-20	3.03E-19	1.01E-18	3.70E-19	1.11E-18	5.00E-19	6.23E-19	2.73E-19	1.24E-18	7.73E-19	1.34E-18	7.63E-19	1.63E-18	2.81E-18	9.36E-18	2.93E-16	1.31E-15
1.98E-19	5.81E-18	8.94E-19	5.11E-20	3.47E-20	1.59E-18	8.36E-19	2.70E-19	4.54E-18	9.40E-21	5.28E-19	5.55E-19	4.12E-18	3.59E-19	1.77E-19	1.23E-19	7.15E-19	1.73E-18	9.40E-18	2.95E-16	1.35E-15
1.11E-18	2.31E-18	3.04E-18	5.79E-20	3.61E-20	2.17E-18	4.06E-18	1.20E-18	8.41E-18	1.20E-18	6.93E-21	1.23E-18	4.32E-18	2.07E-19	3.36E-19	9.20E-19	2.18E-21	1.12E-19	6.70E-18	2.80E-16	1.23E-15
2.10E-18	3.37E-19	2.87E-18	5.31E-20	1.56E-19	1.45E-18	7.93E-18	4.37E-18	1.02E-17	1.94E-18	1.70E-18	2.68E-18	3.57E-18	5.54E-20	1.18E-21	3.87E-18	4.46E-19	8.81E-19	3.11E-18	2.10E-16	9.40E-16
8.99E-20	2.04E-19	1.93E-18	3.63E-21	7.67E-19	2.34E-18	9.90E-18	3.76E-18	1.48E-17	1.36E-18	5.95E-18	2.74E-18	1.59E-18	6.56E-20	5.68E-19	3.82E-18	1.10E-18	2.26E-18	4.65E-19	1.04E-16	6.17E-16
9.17E-19	2.91E-19	7.34E-19	1.66E-19	9.83E-19	1.95E-18	8.98E-18	7.60E-18	1.55E-17	3.52E-18	3.85E-18	1.58E-18	5.90E-19	4.73E-19	1.78E-18	3.28E-18	2.89E-19	4.80E-18	2.41E-19	3.05E-17	3.35E-16
3.89E-20	2.53E-18	6.56E-19	3.11E-18	8.24E-19	4.97E-19	1.51E-17	1.39E-17	1.47E-17	8.77E-18	6.72E-19	1.51E-19	5.58E-19	1.79E-19	3.11E-18	5.99E-18	6.12E-18	8.07E-18	1.65E-18	3.67E-18	1.37E-16
4.33E-19	7.53E-18	4.58E-18	4.08E-18	1.10E-18	3.23E-19	2.26E-17	4.13E-18	1.27E-17	7.06E-18	2.04E-19	2.39E-20	6.08E-19	1.47E-19	2.60E-18	1.02E-17	6.91E-18	8.85E-18	1.10E-18	4.92E-20	3.58E-17
4.60E-18	1.96E-17	3.39E-18	2.14E-18	3.21E-19	4.24E-20	2.22E-17	3.26E-19	8.36E-18	4.43E-18	5.99E-19	1.01E-19	5.00E-20	3.89E-19	1.61E-18	9.51E-18	3.78E-18	7.43E-18	2.03E-19	8.05E-19	1.89E-18
2.95E-18	1.52E-17	9.74E-19	7.41E-19	1.31E-19	6.26E-18	1.28E-17	8.15E-19	6.41E-18	3.95E-18	4.79E-19	1.04E-18	3.35E-18	1.64E-19	2.32E-19	4.78E-18	3.50E-18	6.05E-18	5.77E-21	6.85E-20	6.53E-18
2.09E-19	9.60E-19	2.40E-18	8.82E-19	6.93E-19	5.74E-18	6.62E-18	3.69E-18	1.58E-17	5.32E-18	2.64E-18	4.71E-19	5.81E-18	8.31E-19	1.31E-18	3.20E-18	4.68E-18	7.25E-18	1.21E-20	1.14E-20	2.30E-17
3.07E-18	8.59E-19	3.63E-18	4.08E-19	1.67E-18	6.49E-18	6.99E-18	5.67E-18	2.19E-17	5.16E-18	3.76E-18	6.54E-19	3.66E-20	1.38E-18	4.29E-18	1.51E-18	4.79E-18	7.97E-18	9.55E-19	5.77E-21	2.55E-17
1.09E-17	4.75E-18	6.06E-18	1.74E-19	1.20E-18	9.89E-18	8.23E-18	7.11E-20	1.71E-17	2.47E-18	5.97E-18	1.50E-18	8.85E-19	1.03E-18	2.77E-18	2.97E-19	5.54E-18	4.81E-18	1.97E-18	4.21E-19	2.33E-17
3.19E-17	8.93E-18	4.13E-18	2.28E-19	5.76E-19	7.35E-18	5.26E-18	2.12E-18	7.91E-18	3.21E-18	1.32E-17	4.50E-19	1.15E-18	1.69E-18	1.32E-18	6.45E-19	6.42E-18	2.47E-18	1.66E-18	2.76E-18	2.47E-17
4.13E-17	1.55E-17	1.56E-18	1.02E-18	4.38E-19	4.13E-18	2.44E-19	5.92E-18	2.50E-18	2.41E-18	1.34E-17	6.17E-19	9.00E-19	4.74E-18	4.60E-18	2.66E-18	1.02E-17	2.23E-18	1.37E-18	2.64E-20	2.55E-17
1.86E-17	1.62E-17	7.88E-20	2.16E-18	2.25E-20	4.34E-19	4.28E-19	1.55E-18	4.44E-18	4.04E-19	9.69E-18	7.75E-19	1.98E-18	6.66E-18	9.16E-18	2.76E-18	1.37E-17	4.20E-18	4.58E-18	3.67E-18	2.30E-17
8.74E-18	1.20E-17	1.60E-19	9.21E-19	2.09E-19	6.91E-20	1.88E-18	2.25E-19	1.78E-18	4.98E-20	3.68E-18	7.42E-20	5.41E-19	3.71E-18	7.25E-18	4.76E-18	1.50E-17	4.12E-18	1.13E-17	9.37E-18	2.54E-17
8.05E-18	1.61E-17	4.05E-19	8.85E-20	7.33E-19	2.23E-19	3.28E-18	4.38E-19	4.08E-20	8.94E-19	1.20E-18	3.04E-18	1.96E-19	9.83E-20	5.17E-18	7.24E-18	1.24E-17	5.65E-18	9.16E-18	1.27E-17	3.18E-17
3.96E-18	2.86E-17	1.03E-18	1.39E-20	3.52E-19	3.38E-19	1.47E-19	2.11E-18	3.44E-20	1.10E-18	1.72E-19	4.63E-18	3.98E-20	2.75E-19	5.94E-18	3.65E-18	1.22E-17	7.56E-18	3.12E-18	1.14E-17	3.63E-17
5.35E-18	4.94E-17	4.73E-18	2.63E-19	7.41E-20	4.13E-20	2.24E-19	1.44E-18	1.04E-19	3.13E-19	2.52E-19	1.12E-18	1.89E-20	2.22E-19	7.68E-18	1.24E-18	1.29E-17	5.09E-18	2.44E-18	1.05E-17	3.78E-17
6.02E-18	3.14E-17	5.94E-18	7.60E-19	9.98E-20	3.81E-19	5.04E-19	7.30E-19	6.70E-19	1.00E-18	4.02E-19	1.63E-19	5.99E-19	1.29E-20	6.34E-18	1.65E-18	9.56E-18	2.03E-18	4.13E-18	1.00E-17	3.79E-17
9.14E-18	7.20E-18	5.55E-18	2.83E-20	4.81E-19	4.68E-21	2.41E-19	2.38E-18	2.14E-18	1.13E-18	1.03E-19	1.02E-19	8.48E-19	3.00E-19	4.65E-18	2.00E-18	5.92E-18	9.32E-19	3.99E-18	8.36E-18	3.76E-17
2.11E-17	6.78E-18	9.32E-18	1.60E-19	2.63E-19	4.98E-19	2.22E-19	6.43E-18	5.89E-18	2.46E-18	7.69E-20	1.05E-18	2.39E-19	1.12E-19	3.14E-18	2.77E-18	2.24E-18	5.45E-19	1.35E-18	5.95E-18	3.94E-17
3.78E-17	1.80E-17	1.43E-17	2.88E-19	1.48E-19	5.47E-20	2.30E-18	4.76E-18	6.69E-18	5.94E-18	2.59E-19	5.03E-19	5.94E-24	5.08E-20	1.21E-18	1.15E-18	1.43E-18	5.02E-19	9.57E-19	6.18E-18	3.96E-17
8.16E-17	4.40E-17	2.11E-17	1.22E-18	6.60E-21	8.09E-19	5.06E-18	4.00E-18	6.10E-18	8.59E-18	1.02E-18	8.90E-19	6.15E-20	2.37E-18	1.26E-19	6.03E-19	2.78E-18	5.06E-19	1.57E-18	9.62E-18	3.56E-17
1.58E-16	6.32E-17	7.84E-18	1.73E-18	2.18E-20	6.91E-19	4.38E-18	4.02E-18	1.11E-17	1.71E-17	2.69E-18	3.75E-18	2.43E-19	1.15E-18	6.54E-19	2.35E-20	3.64E-18	2.82E-19	2.07E-18	9.35E-18	3.06E-17
3.39E-16	8.50E-17	4.04E-20	1.67E-19	6.24E-19	3.80E-19	8.12E-18	3.64E-18	1.82E-17	3.04E-17	6.36E-18	6.87E-18	4.98E-19	3.95E-19	5.23E-18	9.78E-19	2.83E-18	8.85E-19	7.64E-19	8.00E-18	2.68E-17
4.59E-16	1.34E-16	8.70E-19	1.31E-20	1.33E-18	9.00E-19	8.38E-18	3.44E-18	2.16E-17	2.16E-17	2.38E-17	1.53E-17	9.95E-19	2.07E-19	6.23E-18	1.93E-18	1.20E-18	3.07E-18	6.44E-21	9.68E-18	2.26E-17
3.17E-16	1.01E-16	1.22E-18	6.18E-20	5.72E-19	8.55E-19	8.50E-18	4.67E-18	2.92E-17	3.04E-17	6.05E-17	2.42E-17	1.79E-18	7.33E-19	5.71E-19	8.24E-21	6.12E-19	1.73E-18	2.30E-18	7.66E-19	1.43E-17
1.06E-16	2.38E-17	7.16E-20	1.03E-19	1.56E-18	1.14E-18	1.01E-17	8.59E-18	5.32E-17	8.60E-17	1.02E-16	3.91E-17	5.37E-18	7.24E-20	1.32E-19	1.48E-19	1.14E-18	2.03E-20	1.83E-17	8.23E-18	3.16E-18
1.36E-17	7.89E-20	2.99E-20	2.53E-18	1.85E-18	7.51E-19	9.72E-18	8.08E-18	6.55E-17	1.37E-16	1.47E-16	6.63E-17	1.03E-17	5.99E-19	4.11E-20	1.15E-18	3.02E-19	1.26E-18	6.71E-17	5.53E-17	7.08E-19
4.95E-19	4.88E-19	2.42E-19	5.40E-18	1.08E-18	1.33E-18	9.07E-18	3.14E-18	5.56E-17	1.12E-16	1.56E-16	8.26E-17	1.86E-17	1.93E-18	6.84E-20	1.89E-18	2.59E-22	7.90E-18	1.87E-16	1.47E-16	1.65E-17
1.89E-18	8.58E-20	6.61E-19	1.90E-18	2.26E-18	2.97E-18	1.06E-17	1.82E-18	3.47E-17	6.05E-17	7.86E-17	6.37E-17	2.41E-17	2.42E-18	2.34E-18	6.36E-19	2.30E-19	2.41E-17	2.64E-16	1.61E-16	1.84E-17
9.42E-19	1.16E-18	1.72E-18	1.86E-20	2.23E-18	1.79E-18	7.92E-18	3.71E-19	2.40E-17	2.78E-17	2.66E-17	3.56E-17	2.74E-17	5.45E-18	8.41E-18	1.26E-18	5.66E-19	1.65E-17	1.20E-16	5.43E-17	5.60E-19
2.30E-19	7.23E-18	1.35E-17	6.94E-19	5.08E-19	1.12E-18	5.22E-18	1.14E-19	1.84E-17	1.00E-17	4.26E-18	1.73E-17	3.00E-17	1.15E-17	1.04E-17	8.51E-18	3.97E-18	4.67E-18	9.22E-18	1.18E-19	1.04E-17
2.64E-19	1.86E-17	2.74E-17	6.16E-18	7.98E-19	1.20E-18	8.94E-19	1.86E-18	1.64E-17	2.58E-18	1.49E-18	1.23E-17	2.02E-17	1.28E-17	5.96E-18	1.88E-17	7.91E-18	1.09E-17	1.93E-18	8.57E-18	5.43E-18
8.18E-19	2.05E-17	1.50E-17	9.92E-18	3.71E-18	1.33E-18	1.32E-19	1.59E-18	1.02E-17	2.23E-19	1.24E-18	4.44E-18	9.14E-18	8.16E-18	4.03E-18	1.52E-17	3.16E-18	2.18E-17	1.49E-17	5.67E-18	9.61E-18
1.99E-18	1.43E-17	1.01E-17	9.67E-18	1.15E-17	3.45E-18	1.50E-19	5.09E-19	1.25E-17	1.76E-18	9.13E-19	2.98E-19	5.35E-18	2.64E-18	3.59E-18	1.83E-18	1.27E-18	2.97E-17	1.62E-17	9.15E-19	1.97E-17
2.19E-18	1.18E-17	1.99E-17	1.05E-17	2.59E-17	9.14E-18	1.20E-18	1.33E-20	4.66E-18	1.27E-18	2.65E-19	1.23E-18	7.18E-18	4.44E-19	4.77E-18	8.60E-20	1.08E-18	8.49E-18	3.27E-20	1.68E-19	1.30E-17
3.24E-18	9.77E-18	3.95E-17	2.38E-17	2.38E-17	1.67E-17	3.08E-18	1.31E-18	2.34E-18	2.69E-19	3.90E-24	1.12E-18	7.09E-18	1.09E-18	3.53E-18	5.69E-21	2.61E-18	1.05E-19	7.09E-19	5.25E-19	8.26E-18
3.09E-18	9.27E-18	4.03E-17	5.25E-17	1.83E-17	1.53E-17	1.16E-17	1.00E-17	2.47E-18	2.85E-22	3.50E-19	2.13E-19	3.42E-18	8.20E-19	1.05E-18	5.69E-19	1.38E-18	5.00E-20	1.16E-19	1.30E-20	2.25E-18
5.65E-18	3.44E-18	1.37E-17	7.63E-17	2.29E-17	2.05E-17	3.04E-17	2.65E-17	1.50E-18	3.27E-23	1.87E-18	7.85E-20	1.56E-18	3.57E-19	4.98E-19	5.93E-19	1.35E-19	1.72E-24	3.74E-19	5.28E-20	1.07E-18
1.18E-17	6.83E-18	1.39E-17	9.34E-17	3.62E-17	3.09E-17	7.55E-17	5.13E-17	1.33E-18	2.04E-19	1.69E-18	1.97E-19	2.34E-18	9.13E-19	8.44E-19	1.06E-22	1.79E-19	8.66E-19	4.42E-19	6.99E-19	9.03E-19
1.71E-17	9.48E-18	1.80E-17	9.86E-17	4.63E-17	5.88E-17	1.43E-16	1.06E-16	1.78E-18	5.03E-20	1.36E-18	4.13E-20	1.51E-18	1.62E-18	4.50E-18	1.59E-19	1.75E-18	2.68E-18	2.14E-18	2.08E-19	3.99E-21
1.61E-17	8.29E-18	1.40E-17	3.63E-17	3.52E-17	1.15E-16	2.90E-16	2.57E-16	4.64E-18	3.24E-20	3.03E-18	2.51E-19	1.16E-18	1.66E-18	7.04E-18	4.38E-19	3.51E-18	3.20E-18	1.80E-18	3.67E-18	4.02E-18
5.47E-18	8.69E-18	1.50E-17	1.61E-17	3.92E-17	2.08E-16	5.55E-16	4.90E-16	5.70E-18	2.51E-21	1.74E-18	1.82E-19	2.32E-18	1.14E-18	5.79E-18	2.54E-18	2.08E-18	1.51E-18	9.45E-19	7.30E-18	1.05E-17
3.44E-18	1.35E-17	1.43E-17	1.21E-17	4.78E-17	2.60E-16	6.20E-16	4.76E-16	4.47E-18	7.51E-19	8.27E-19	2.30E-19	1.47E-18	4.69E-19	6.87E-18	1.22E-18	2.47E-18	2.40E-19	1.27E-19	4.85E-18	1.26E-17
5.55E-18	2.18E-17	1.70E-17	1.14E-17	2.66E-17	1.07E-16	1.34E-16	8.19E-17	2.42E-18	2.80E-18	1.70E-18	8.27E-19	2.70E-19	4.48E-19	8.09E-18	1.03E-18	1.77E-18	2.28E-19	1.59E-20	7.45E-18	2.25E-17
5.95E-18	2.98E-17	1.80E-17	1.03E-17	5.08E-18	4.87E-18	8.60E-18	7.85E-18	2.38E-18	2.19E-18	3.55E-18	4.14E-19	2.43E-20	1.37E-18	1.03E-17	3.68E-18	7.08E-19	1.07E-18	4.38E-19	1.61E-17	2.77E-17
3.61E-18	2.34E-17	1.50E-17	1.28E-17	3.29E-18	7.10E-19	6.24E-18	7.72E-18	5.80E-18	4.58E-18	2.80E-18	1.78E-19	2.52E-19	2.46E-18	6.45E-18	3.09E-18	5.71E-19	3.27E-19	1.74E-19	2.02E-17	4.12E-17
4.29E-18	1.72E-17	1.20E-17	1.36E-17	4.32E-18	1.63E-18	1.33E-17	1.40E-17	1.29E-17	1.96E-17	3.83E-18	1.62E-19	1.76E-18	2.67E-18	1.21E-19	1.02E-18	9.11E-19	1.57E-19	7.85E-20	1.42E-17	5.41E-17
5.78E-18	1.56E-17	1.06E-17	1.89E-17	7.92E-18	1.28E-18	1.39E-18	3.35E-17	7.94E-18	4.65E-17	4.78E-18	1.51E-20	3.39E-18	1.31E-18	6.93E-20	2.58E-19	1.07E-19	2.99E-20	2.92E-21	9.92E-18	5.41E-17
4.36E-18	1.55E-17	1.04E-17	2.83E-17	7.80E-18	3.32E-18	1.98E-19	4.20E-17	1.56E-17	7.79E-17	6.56E-18	2.89E-19	3.01E-18	1.31E-18	1.53E-19	2.36E-18	6.39E-20	1.37E-20	5.66E-19	1.90E-17	3.95E-17
2.00E-18	2.66E-17	1.15E-17	3.12E-17	9.91E-18	8.06E-18	7.13E-19	1.41E-17	2.97E-17	5.48E-17	1.08E-17	2.28E-19	1.69E-18	1.35E-18	7.77E-20	1.73E-18	6.03E-20	5.94E-20	1.04E-18	1.89E-17	1.98E-17
2.49E-18	3.64E-17	1.52E-17	1.89E-17	1.34E-17	6.12E-18	1.13E-18	7.28E-18	4.84E-17	7.01E-17	1.85E-17	1.19E-20	9.41E-19	8.33E-18	5.15E-19	3.00E-19	1.05E-18	9.02E-19	3.74E-24	6.89E-18	1.76E-18
6.00E-18	3.43E-17	1.87E-17	9.88E-18	2.32E-17	8.83E-18	3.37E-18	7.44E-18	8.85E-17	1.22E-16	2.48E-17	2.84E-19	1.47E-18	1.12E-17	3.25E-20	5.98E-19	2.48E-19	3.19E-18	3.83E-19	1.55E-19	1.95E-17
1.52E-17	3.03E-17	2.09E-17	7.88E-18	2.37E-17	1.35E-17	6.74E-18	9.32E-18	9.90E-17	1.29E-16	3.35E-17	2.37E-18	5.49E-19	3.97E-20	1.76E-18	1.54E-18	3.88E-19	2.08E-18	2.70E-20	3.91E-18	2.07E-16
2.09E-17	3.74E-17	2.47E-17	5.46E-18	2.06E-17	1.69E-17	3.38E-18	1.03E-17	2.49E-17	5.32E-17	5.59E-17	6.78E-18	8.58E-21	6.47E-19	5.88E-18	8.18E-19	6.21E-19	1.03E-18	6.03E-22	2.87E-17	7.34E-16
1.41E-17	3.04E-17	3.14E-17	6.89E-18	1.36E-17	1.69E-17	1.30E-18	5.29E-18	1.25E-18	3.16E-17	1.37E-16	1.91E-17	2.58E-18	2.87E-19	8.07E-18	3.77E-19	2.55E-18	5.10E-20	1.52E-18	9.66E-17	1.48E-15
1.57E-17	1.70E-17	3.76E-17	9.92E-18	1.02E-17	1.26E-17	1.24E-18	6.34E-18	2.78E-18	5.31E-17	2.66E-16	4.40E-17	1.04E-17	2.43E-18	9.36E-18	6.43E-20	6.79E-18	6.19E-21	5.00E-18	1.87E-16	2.09E-15
1.78E-17	1.09E-17	3.85E-17	1.26E-17	1.58E-17	2.21E-17	2.83E-18	1.56E-17	5.47E-18	5.78E-17	2.81E-16	7.98E-17	1.98E-17	3.92E-18	7.99E-18	9.41E-19	6.29E-18	5.57E-19	1.02E-17	2.88E-16	2.51E-15
1.33E-17	1.92E-17	3.19E-17	2.02E-17	1.95E-17	2.76E-17	7.06E-18	1.57E-17	2.77E-18	2.35E-17	1.37E-16	1.38E-16	4.43E-17	6.24E-18	2.26E-18	2.26E-18	5.00E-18	1.61E-18	1.97E-17	2.91E-16	2.61E-15

2.00E-17	2.33E-17	3.74E-17	2.68E-17	2.66E-17	1.46E-17	7.65E-18	6.03E-18	1.42E-18	1.01E-17	1.24E-16	3.23E-16	6.43E-17	1.50E-17	5.69E-18	2.23E-18	3.30E-18	2.91E-18	1.55E-17	2.18E-16	2.28E-15
2.42E-17	1.47E-17	3.97E-17	3.58E-17	3.46E-17	1.22E-17	9.05E-18	9.14E-18	1.73E-18	1.94E-17	2.50E-16	4.44E-16	9.26E-17	3.56E-17	1.01E-17	6.97E-19	9.22E-19	3.97E-18	3.59E-18	1.30E-16	1.69E-15
1.45E-17	1.89E-17	3.26E-17	3.18E-17	3.93E-17	1.66E-17	2.01E-17	1.36E-17	1.17E-18	2.08E-17	3.44E-16	2.38E-16	5.14E-17	6.50E-17	1.27E-17	1.13E-18	5.63E-19	2.99E-18	5.52E-19	6.64E-17	1.07E-15
1.34E-17	2.37E-17	3.66E-17	1.80E-17	2.94E-17	2.69E-17	2.89E-17	1.54E-17	9.33E-19	5.59E-18	1.67E-16	5.56E-18	6.92E-17	1.34E-16	1.31E-17	1.63E-18	3.57E-18	1.64E-18	2.42E-19	2.52E-17	5.65E-16
1.95E-17	2.01E-17	4.61E-17	1.54E-17	2.94E-17	2.91E-17	2.15E-17	1.49E-17	3.52E-18	3.71E-18	1.10E-17	4.15E-18	1.45E-16	2.47E-16	2.29E-17	4.30E-18	1.02E-17	9.75E-19	1.42E-19	3.67E-18	1.91E-16
1.66E-17	1.63E-17	5.02E-17	1.68E-17	3.21E-17	3.41E-17	1.05E-17	2.51E-17	9.52E-18	7.75E-18	4.15E-18	3.33E-18	1.39E-16	2.36E-16	3.17E-17	8.89E-18	3.50E-17	3.84E-20	3.45E-21	9.26E-21	2.25E-17
1.85E-17	1.75E-17	3.76E-17	1.60E-17	2.04E-17	2.10E-17	1.74E-17	2.28E-17	1.73E-17	1.41E-17	5.52E-18	4.71E-18	4.28E-17	6.90E-17	1.47E-17	8.71E-18	9.03E-17	2.70E-18	1.67E-19	2.84E-20	1.02E-18
1.99E-17	1.83E-17	2.29E-17	1.82E-17	1.55E-17	1.38E-17	2.05E-17	1.81E-17	2.03E-17	1.74E-17	7.66E-18	6.68E-18	1.47E-17	1.87E-17	1.73E-17	3.28E-17	1.91E-16	9.92E-18	1.05E-20	5.33E-20	8.11E-18
1.38E-17	1.86E-17	2.19E-17	1.72E-17	1.16E-17	1.18E-17	1.28E-17	5.42E-18	2.92E-17	3.63E-17	7.13E-18	5.92E-18	1.11E-17	2.80E-17	2.34E-17	1.00E-16	3.56E-16	1.29E-17	5.89E-19	1.40E-19	1.23E-17
7.78E-18	2.28E-17	2.12E-17	1.59E-17	1.25E-17	1.03E-17	1.47E-17	4.77E-18	3.80E-17	4.06E-17	1.93E-18	2.50E-18	1.01E-17	5.11E-17	2.76E-17	2.32E-16	4.89E-16	1.26E-17	5.57E-19	1.87E-20	1.02E-17
1.17E-17	2.31E-17	2.52E-17	1.68E-17	1.24E-17	1.39E-17	2.42E-17	5.70E-18	2.19E-17	7.11E-18	1.70E-18	3.72E-18	1.52E-17	4.83E-17	1.41E-17	4.87E-16	6.68E-16	2.28E-17	5.33E-19	1.14E-18	1.18E-17
1.49E-17	2.26E-17	1.76E-17	2.30E-17	9.15E-18	1.80E-17	2.08E-17	8.02E-18	1.08E-17	2.54E-18	1.52E-18	1.18E-17	1.72E-17	2.50E-17	1.10E-17	4.89E-16	3.84E-16	6.20E-18	2.72E-18	1.09E-17	1.69E-17
9.29E-18	1.67E-17	9.40E-18	2.82E-17	8.77E-18	1.91E-17	1.09E-17	9.07E-18	4.16E-18	8.93E-18	1.11E-18	2.98E-17	1.62E-17	7.59E-18	8.97E-18	1.10E-16	2.33E-17	2.25E-19	2.49E-17	3.27E-17	1.83E-17
5.30E-18	1.28E-17	7.13E-18	1.86E-17	1.34E-17	1.95E-17	8.59E-18	9.95E-18	4.32E-18	1.31E-17	2.73E-18	3.90E-17	2.25E-17	8.78E-18	9.65E-18	3.06E-18	4.63E-19	1.08E-19	6.65E-17	5.30E-17	1.36E-17
2.58E-18	1.68E-17	9.85E-18	1.22E-17	1.66E-17	1.82E-17	1.07E-17	1.01E-17	1.09E-17	4.14E-18	4.85E-18	4.10E-17	6.26E-17	1.15E-17	7.63E-18	2.51E-18	8.11E-21	5.50E-19	2.52E-17	2.14E-17	1.65E-17
2.60E-18	1.52E-17	8.94E-18	8.73E-18	9.43E-18	1.22E-17	1.04E-17	6.84E-18	2.23E-17	2.23E-18	3.46E-18	6.60E-17	1.06E-16	3.57E-18	1.26E-17	3.88E-18	2.29E-18	5.11E-18	4.74E-19	9.28E-19	1.04E-17
4.03E-18	1.63E-17	8.10E-18	8.40E-18	5.34E-18	8.31E-18	7.10E-18	2.79E-18	2.29E-17	3.08E-18	1.48E-19	4.02E-17	6.09E-17	3.30E-19	1.65E-17	7.94E-18	5.89E-18	5.90E-18	2.47E-19	3.69E-18	8.22E-19
3.41E-15	2.98E-15	1.90E-15	1.59E-15	1.87E-15	2.05E-15	3.38E-15	3.03E-15	2.60E-15	2.51E-15	3.15E-15	2.28E-15	1.60E-15	1.35E-15	8.72E-16	2.16E-15	3.20E-15	7.27E-16	1.12E-15	3.99E-15	2.65E-14

Total Values:

RMS Quadrant 1	RMS Quadrant 2	RMS Quadrant 3	RMS Quadrant 4
5.0954E-08	4.0949E-08	2.04982E-08	4.0549E-08

**RMS AVG**	3.824E-08
**STDEV**	1.277E-08

-1.30E-08	-1.75E-08	-2.27E-08	-3.27E-08	-3.56E-08	-4.19E-08	-4.97E-08	-5.59E-08	-6.43E-08	-7.14E-08	-7.51E-08	-7.51E-08	-8.17E-08	-7.68E-08	-7.63E-08	-7.73E-08	-7.83E-08	-7.41E-08	-7.10E-08	-6.48E-08	-5.84E-08
-1.38E-08	-1.76E-08	-2.30E-08	-3.29E-08	-3.48E-08	-4.13E-08	-4.93E-08	-5.67E-08	-6.42E-08	-7.08E-08	-7.53E-08	-7.57E-08	-8.21E-08	-7.83E-08	-7.59E-08	-7.54E-08	-7.69E-08	-7.28E-08	-7.08E-08	-6.47E-08	-5.71E-08
-1.33E-08	-1.77E-08	-2.24E-08	-3.25E-08	-3.49E-08	-4.18E-08	-4.95E-08	-5.73E-08	-6.39E-08	-7.02E-08	-7.68E-08	-7.82E-08	-8.16E-08	-7.74E-08	-7.38E-08	-7.17E-08	-7.53E-08	-7.16E-08	-7.06E-08	-6.52E-08	-5.73E-08
-1.36E-08	-1.67E-08	-2.28E-08	-3.17E-08	-3.49E-08	-4.07E-08	-4.91E-08	-5.77E-08	-6.39E-08	-6.96E-08	-7.73E-08	-7.84E-08	-8.13E-08	-7.58E-08	-7.18E-08	-6.74E-08	-7.30E-08	-7.10E-08	-6.97E-08	-6.66E-08	-5.73E-08
-1.45E-08	-1.52E-08	-2.28E-08	-3.10E-08	-3.54E-08	-3.93E-08	-4.85E-08	-5.82E-08	-6.60E-08	-7.03E-08	-7.66E-08	-7.80E-08	-8.19E-08	-7.75E-08	-6.91E-08	-6.12E-08	-6.92E-08	-7.08E-08	-6.87E-08	-6.68E-08	-6.39E-08
-1.44E-08	-1.55E-08	-2.31E-08	-3.06E-08	-3.62E-08	-3.98E-08	-4.81E-08	-5.83E-08	-6.72E-08	-6.91E-08	-7.59E-08	-8.13E-08	-8.19E-08	-7.78E-08	-6.64E-08	-6.21E-08	-6.94E-08	-7.17E-08	-6.91E-08	-6.66E-08	-6.43E-08
-1.37E-08	-1.72E-08	-2.38E-08	-3.05E-08	-3.57E-08	-4.08E-08	-4.87E-08	-5.83E-08	-6.67E-08	-6.92E-08	-7.50E-08	-8.18E-08	-8.07E-08	-7.78E-08	-6.76E-08	-6.60E-08	-7.44E-08	-7.28E-08	-6.98E-08	-6.65E-08	-6.43E-08
-1.26E-08	-1.58E-08	-2.26E-08	-2.99E-08	-3.48E-08	-4.03E-08	-4.87E-08	-5.72E-08	-6.56E-08	-7.19E-08	-7.34E-08	-8.17E-08	-8.03E-08	-7.79E-08	-7.27E-08	-7.49E-08	-7.87E-08	-7.30E-08	-6.88E-08	-6.62E-08	-6.48E-08
-1.14E-08	-1.41E-08	-2.08E-08	-2.87E-08	-3.53E-08	-3.94E-08	-4.76E-08	-5.45E-08	-6.27E-08	-7.20E-08	-7.36E-08	-8.05E-08	-8.06E-08	-7.81E-08	-7.53E-08	-7.65E-08	-7.70E-08	-7.30E-08	-6.81E-08	-6.53E-08	-6.41E-08
-1.09E-08	-1.51E-08	-1.89E-08	-2.75E-08	-3.58E-08	-3.92E-08	-4.69E-08	-5.29E-08	-5.98E-08	-7.10E-08	-7.26E-08	-8.03E-08	-8.21E-08	-7.78E-08	-7.65E-08	-7.67E-08	-7.59E-08	-7.23E-08	-6.75E-08	-6.40E-08	-6.30E-08
-1.05E-08	-1.48E-08	-1.70E-08	-2.71E-08	-3.44E-08	-3.96E-08	-4.61E-08	-5.23E-08	-6.13E-08	-6.96E-08	-6.96E-08	-8.16E-08	-8.12E-08	-7.69E-08	-7.61E-08	-7.71E-08	-7.64E-08	-7.11E-08	-6.73E-08	-6.38E-08	-6.20E-08
-1.11E-08	-1.33E-08	-1.66E-08	-2.73E-08	-3.28E-08	-4.02E-08	-4.51E-08	-5.36E-08	-6.25E-08	-6.97E-08	-6.99E-08	-8.09E-08	-8.01E-08	-7.72E-08	-7.46E-08	-7.65E-08	-7.76E-08	-6.99E-08	-6.80E-08	-6.52E-08	-6.09E-08
-1.09E-08	-1.15E-08	-1.84E-08	-2.69E-08	-3.13E-08	-3.92E-08	-4.50E-08	-5.44E-08	-6.21E-08	-6.93E-08	-7.06E-08	-7.90E-08	-7.90E-08	-7.68E-08	-7.44E-08	-7.69E-08	-7.68E-08	-6.87E-08	-6.73E-08	-6.49E-08	-6.03E-08
-9.71E-09	-1.16E-08	-1.94E-08	-2.62E-08	-3.10E-08	-3.90E-08	-4.46E-08	-5.22E-08	-6.17E-08	-6.76E-08	-6.99E-08	-7.76E-08	-7.84E-08	-7.57E-08	-7.50E-08	-7.65E-08	-7.46E-08	-6.87E-08	-6.66E-08	-6.34E-08	-5.99E-08
-8.51E-09	-1.49E-08	-2.07E-08	-2.65E-08	-3.21E-08	-4.02E-08	-4.36E-08	-4.98E-08	-6.40E-08	-6.53E-08	-6.71E-08	-7.85E-08	-7.89E-08	-7.44E-08	-7.53E-08	-7.65E-08	-7.29E-08	-6.93E-08	-6.66E-08	-6.25E-08	-6.04E-08
-8.47E-09	-1.60E-08	-2.01E-08	-2.67E-08	-3.22E-08	-4.05E-08	-4.43E-08	-5.37E-08	-6.43E-08	-6.64E-08	-6.91E-08	-7.90E-08	-7.82E-08	-7.50E-08	-7.52E-08	-7.56E-08	-7.33E-08	-6.90E-08	-6.67E-08	-6.27E-08	-6.12E-08
-9.50E-09	-1.47E-08	-1.87E-08	-2.58E-08	-3.26E-08	-4.00E-08	-4.63E-08	-5.73E-08	-6.45E-08	-6.72E-08	-7.12E-08	-7.76E-08	-7.98E-08	-7.64E-08	-7.48E-08	-7.56E-08	-7.47E-08	-7.08E-08	-6.71E-08	-6.37E-08	-6.19E-08
-9.25E-09	-1.35E-08	-1.88E-08	-2.47E-08	-3.19E-08	-3.90E-08	-4.58E-08	-5.66E-08	-6.38E-08	-6.62E-08	-7.08E-08	-7.74E-08	-8.19E-08	-7.72E-08	-7.55E-08	-7.47E-08	-7.48E-08	-7.06E-08	-6.75E-08	-6.45E-08	-6.26E-08
-8.49E-09	-1.41E-08	-1.95E-08	-2.41E-08	-3.29E-08	-3.95E-08	-4.40E-08	-5.54E-08	-6.25E-08	-6.55E-08	-7.07E-08	-7.74E-08	-8.21E-08	-7.66E-08	-7.55E-08	-7.53E-08	-7.50E-08	-7.08E-08	-6.86E-08	-6.52E-08	-6.16E-08
-8.54E-09	-1.41E-08	-2.00E-08	-2.43E-08	-3.38E-08	-4.02E-08	-4.40E-08	-5.55E-08	-6.12E-08	-6.58E-08	-7.21E-08	-7.93E-08	-8.08E-08	-7.58E-08	-7.52E-08	-7.53E-08	-7.50E-08	-7.09E-08	-7.02E-08	-6.65E-08	-6.09E-08
-8.53E-09	-1.45E-08	-2.02E-08	-2.67E-08	-3.53E-08	-4.20E-08	-4.58E-08	-5.50E-08	-6.06E-08	-6.64E-08	-7.18E-08	-7.80E-08	-8.01E-08	-7.60E-08	-7.56E-08	-7.62E-08	-7.55E-08	-7.00E-08	-7.01E-08	-6.67E-08	-6.00E-08
-7.47E-09	-1.43E-08	-2.12E-08	-2.76E-08	-3.54E-08	-4.23E-08	-4.56E-08	-5.48E-08	-6.16E-08	-6.69E-08	-7.21E-08	-7.71E-08	-8.09E-08	-7.63E-08	-7.63E-08	-7.69E-08	-7.59E-08	-7.00E-08	-6.95E-08	-6.66E-08	-5.76E-08
-6.01E-09	-1.31E-08	-2.30E-08	-2.78E-08	-3.50E-08	-4.22E-08	-4.55E-08	-5.50E-08	-6.24E-08	-6.59E-08	-7.29E-08	-7.74E-08	-8.01E-08	-7.52E-08	-7.66E-08	-7.54E-08	-7.54E-08	-7.21E-08	-6.96E-08	-6.60E-08	-5.56E-08
-5.03E-09	-1.24E-08	-2.39E-08	-2.83E-08	-3.49E-08	-4.21E-08	-4.71E-08	-5.54E-08	-6.24E-08	-6.71E-08	-7.24E-08	-7.82E-08	-7.90E-08	-7.47E-08	-7.65E-08	-7.46E-08	-7.50E-08	-7.30E-08	-6.99E-08	-6.60E-08	-5.93E-08
-7.39E-09	-1.44E-08	-2.43E-08	-2.89E-08	-3.58E-08	-4.17E-08	-4.76E-08	-5.27E-08	-6.18E-08	-6.95E-08	-6.95E-08	-7.64E-08	-7.86E-08	-7.46E-08	-7.52E-08	-7.51E-08	-7.50E-08	-7.18E-08	-7.05E-08	-6.61E-08	-6.03E-08
-1.22E-08	-1.79E-08	-2.46E-08	-2.94E-08	-3.75E-08	-4.23E-08	-4.60E-08	-4.74E-08	-6.19E-08	-6.86E-08	-6.45E-08	-7.38E-08	-7.93E-08	-7.67E-08	-7.46E-08	-7.50E-08	-7.43E-08	-7.11E-08	-7.07E-08	-6.61E-08	-5.87E-08
-1.34E-08	-1.81E-08	-2.47E-08	-2.99E-08	-3.78E-08	-4.19E-08	-4.31E-08	-3.98E-08	-6.19E-08	-6.75E-08	-5.96E-08	-7.13E-08	-7.96E-08	-7.71E-08	-7.50E-08	-7.46E-08	-7.54E-08	-7.17E-08	-7.06E-08	-6.57E-08	-5.87E-08
-1.24E-08	-1.87E-08	-2.45E-08	-2.94E-08	-3.74E-08	-4.01E-08	-3.99E-08	-3.36E-08	-6.16E-08	-6.68E-08	-6.00E-08	-7.19E-08	-7.93E-08	-7.63E-08	-7.51E-08	-7.43E-08	-7.53E-08	-7.12E-08	-7.06E-08	-6.62E-08	-6.03E-08
-1.05E-08	-1.71E-08	-2.57E-08	-2.79E-08	-3.66E-08	-3.89E-08	-3.95E-08	-4.62E-08	-6.17E-08	-6.54E-08	-7.00E-08	-7.47E-08	-7.72E-08	-7.66E-08	-7.53E-08	-7.40E-08	-7.44E-08	-7.08E-08	-6.98E-08	-6.62E-08	-6.12E-08
-7.46E-09	-1.39E-08	-2.64E-08	-2.64E-08	-3.57E-08	-4.03E-08	-4.39E-08	-4.43E-08	-5.98E-08	-6.51E-08	-6.94E-08	-7.38E-08	-7.51E-08	-7.62E-08	-7.50E-08	-7.35E-08	-7.39E-08	-7.00E-08	-6.86E-08	-6.57E-08	-6.17E-08
-5.77E-09	-1.34E-08	-2.53E-08	-2.46E-08	-3.50E-08	-4.15E-08	-4.75E-08	-4.27E-08	-5.81E-08	-6.63E-08	-6.85E-08	-7.28E-08	-7.62E-08	-7.52E-08	-7.38E-08	-7.43E-08	-7.42E-08	-6.93E-08	-6.85E-08	-6.59E-08	-6.23E-08
-9.53E-09	-1.59E-08	-2.36E-08	-2.28E-08	-3.40E-08	-4.19E-08	-4.78E-08	-4.44E-08	-5.97E-08	-6.78E-08	-6.70E-08	-7.08E-08	-7.75E-08	-7.34E-08	-7.29E-08	-7.40E-08	-7.37E-08	-6.98E-08	-6.96E-08	-6.57E-08	-6.23E-08
-1.10E-08	-1.57E-08	-2.26E-08	-2.30E-08	-3.34E-08	-4.12E-08	-4.65E-08	-5.98E-08	-6.29E-08	-6.81E-08	-6.62E-08	-6.86E-08	-7.74E-08	-7.29E-08	-7.23E-08	-7.10E-08	-7.04E-08	-6.83E-08	-6.75E-08	-6.38E-08	-6.01E-08
-1.06E-08	-1.52E-08	-2.21E-08	-2.70E-08	-3.43E-08	-4.10E-08	-4.54E-08	-5.77E-08	-6.17E-08	-6.63E-08	-6.62E-08	-6.96E-08	-7.43E-08	-7.06E-08	-6.96E-08	-6.54E-08	-6.45E-08	-6.41E-08	-6.32E-08	-5.93E-08	-5.39E-08
-1.04E-08	-1.48E-08	-2.20E-08	-2.88E-08	-3.41E-08	-4.01E-08	-4.55E-08	-5.43E-08	-5.76E-08	-6.17E-08	-6.59E-08	-6.62E-08	-6.87E-08	-6.49E-08	-6.32E-08	-5.73E-08	-5.72E-08	-5.61E-08	-5.48E-08	-5.18E-08	-4.57E-08
-1.03E-08	-1.28E-08	-2.14E-08	-2.82E-08	-3.33E-08	-3.90E-08	-4.52E-08	-5.12E-08	-5.47E-08	-5.46E-08	-5.83E-08	-5.75E-08	-5.88E-08	-5.44E-08	-5.09E-08	-4.40E-08	-4.58E-08	-4.31E-08	-4.12E-08	-3.87E-08	-3.36E-08
-1.02E-08	-1.10E-08	-2.00E-08	-2.71E-08	-3.13E-08	-3.49E-08	-4.01E-08	-4.77E-08	-4.60E-08	-4.51E-08	-4.48E-08	-4.22E-08	-4.23E-08	-3.83E-08	-3.40E-08	-2.45E-08	-2.89E-08	-2.50E-08	-2.35E-08	-2.20E-08	-1.85E-08
-9.83E-09	-1.11E-08	-2.02E-08	-2.62E-08	-2.74E-08	-2.84E-08	-3.02E-08	-3.69E-08	-3.04E-08	-2.84E-08	-2.70E-08	-2.35E-08	-2.27E-08	-1.95E-08	-1.53E-08	-7.41E-09	-8.93E-09	-5.02E-09	-4.64E-09	-4.09E-09	-4.66E-09
-9.01E-09	-1.10E-08	-1.96E-08	-2.28E-08	-2.10E-08	-1.75E-08	-1.58E-08	-2.13E-08	-1.18E-08	-9.24E-09	-8.02E-09	-3.66E-09	-2.46E-09	9.75E-10	5.69E-09	1.40E-08	1.20E-08	1.52E-08	1.42E-08	1.48E-08	1.33E-08
-8.14E-09	-8.87E-09	-1.59E-08	-1.50E-08	-9.66E-09	-2.47E-09	8.98E-10	-3.29E-09	6.51E-09	1.10E-08	1.24E-08	1.67E-08	1.90E-08	2.25E-08	2.79E-08	3.29E-08	3.33E-08	3.55E-08	3.30E-08	3.34E-08	3.23E-08
-7.49E-09	-7.86E-09	-9.43E-09	-2.82E-09	4.92E-09	1.37E-08	1.80E-08	1.53E-08	2.58E-08	3.15E-08	3.36E-08	3.77E-08	4.13E-08	4.43E-08	5.02E-08	5.38E-08	5.44E-08	5.64E-08	5.24E-08	5.20E-08	4.81E-08
-4.58E-09	-3.16E-09	1.32E-09	1.01E-08	1.96E-08	3.01E-08	3.61E-08	3.50E-08	4.62E-08	5.24E-08	5.55E-08	6.01E-08	6.38E-08	6.72E-08	7.19E-08	7.52E-08	7.65E-08	7.78E-08	7.26E-08	7.06E-08	6.64E-08
1.42E-09	6.47E-09	1.37E-08	2.31E-08	3.52E-08	4.67E-08	5.51E-08	5.54E-08	6.71E-08	7.44E-08	7.74E-08	8.39E-08	8.69E-08	9.11E-08	9.41E-08	9.75E-08	9.98E-08	9.95E-08	9.38E-08	8.96E-08	8.43E-08
8.91E-09	1.78E-08	2.63E-08	3.66E-08	5.08E-08	6.31E-08	7.39E-08	7.59E-08	8.86E-08	9.69E-08	9.96E-08	1.07E-07	1.10E-07	1.14E-07	1.17E-07	1.20E-07	1.23E-07	1.22E-07	1.15E-07	1.08E-07	1.02E-07
1.83E-08	2.90E-08	3.85E-08	4.95E-08	6.47E-08	7.86E-08	9.17E-08	9.60E-08	1.10E-07	1.18E-07	1.20E-07	1.28E-07	1.32E-07	1.35E-07	1.40E-07	1.43E-07	1.44E-07	1.44E-07	1.35E-07	1.26E-07	1.19E-07
2.86E-08	3.97E-08	4.96E-08	6.12E-08	7.71E-08	9.21E-08	1.07E-07	1.13E-07	1.28E-07	1.36E-07	1.37E-07	1.46E-07	1.51E-07	1.54E-07	1.61E-07	1.64E-07	1.63E-07	1.63E-07	1.53E-07	1.43E-07	1.34E-07
3.88E-08	4.95E-08	5.99E-08	7.20E-08	8.73E-08	1.02E-07	1.18E-07	1.25E-07	1.41E-07	1.50E-07	1.50E-07	1.60E-07	1.66E-07	1.71E-07	1.79E-07	1.82E-07	1.79E-07	1.77E-07	1.68E-07	1.57E-07	1.44E-07
4.79E-08	5.85E-08	6.99E-08	8.14E-08	9.52E-08	1.10E-07	1.25E-07	1.32E-07	1.49E-07	1.61E-07	1.62E-07	1.73E-07	1.80E-07	1.86E-07	1.93E-07	1.96E-07	1.92E-07	1.90E-07	1.81E-07	1.70E-07	1.55E-07
5.55E-08	6.65E-08	7.79E-08	8.88E-08	1.02E-07	1.14E-07	1.28E-07	1.37E-07	1.57E-07	1.72E-07	1.73E-07	1.84E-07	1.94E-07	1.99E-07	2.04E-07	2.04E-07	2.02E-07	2.01E-07	1.93E-07	1.81E-07	1.65E-07
6.07E-08	7.25E-08	8.30E-08	9.38E-08	1.07E-07	1.18E-07	1.31E-07	1.41E-07	1.63E-07	1.80E-07	1.81E-07	1.94E-07	2.07E-07	2.10E-07	2.14E-07	2.10E-07	2.09E-07	2.10E-07	2.03E-07	1.92E-07	1.73E-07
6.14E-08	7.45E-08	8.56E-08	9.69E-08	1.11E-07	1.20E-07	1.33E-07	1.43E-07	1.64E-07	1.83E-07	1.85E-07	2.01E-07	2.16E-07	2.19E-07	2.22E-07	2.15E-07	2.15E-07	2.17E-07	2.11E-07	2.01E-07	1.82E-07
5.70E-08	7.19E-08	8.52E-08	9.83E-08	1.12E-07	1.20E-07	1.33E-07	1.43E-07	1.62E-07	1.77E-07	1.85E-07	2.02E-07	2.21E-07	2.22E-07	2.23E-07	2.19E-07	2.18E-07	2.21E-07	2.18E-07	2.08E-07	1.91E-07
5.11E-08	6.62E-08	8.17E-08	9.68E-08	1.10E-07	1.17E-07	1.30E-07	1.42E-07	1.58E-07	1.73E-07	1.83E-07	2.00E-07	2.22E-07	2.20E-07	2.20E-07	2.18E-07	2.19E-07	2.22E-07	2.22E-07	2.14E-07	1.97E-07
4.47E-08	5.90E-08	7.56E-08	9.12E-08	1.04E-07	1.11E-07	1.26E-07	1.40E-07	1.54E-07	1.67E-07	1.80E-07	1.96E-07	2.17E-07	2.13E-07	2.16E-07	2.16E-07	2.17E-07	2.21E-07	2.23E-07	2.16E-07	2.02E-07
3.73E-08	5.11E-08	6.78E-08	8.27E-08	9.57E-08	1.05E-07	1.22E-07	1.40E-07	1.50E-07	1.62E-07	1.75E-07	1.91E-07	2.07E-07	2.07E-07	2.09E-07	2.10E-07	2.13E-07	2.16E-07	2.17E-07	2.14E-07	2.03E-07

2.91E-08	4.28E-08	5.86E-08	7.28E-08	8.63E-08	9.89E-08	1.17E-07	1.41E-07	1.47E-07	1.57E-07	1.71E-07	1.87E-07	2.04E-07	2.03E-07	2.00E-07	2.03E-07	2.06E-07	2.08E-07	2.10E-07	2.10E-07	2.04E-07
2.06E-08	3.41E-08	4.93E-08	6.21E-08	7.57E-08	9.03E-08	1.06E-07	1.38E-07	1.43E-07	1.52E-07	1.66E-07	1.83E-07	2.02E-07	1.97E-07	1.90E-07	1.93E-07	1.96E-07	1.98E-07	2.02E-07	2.04E-07	2.03E-07
1.18E-08	2.45E-08	3.92E-08	5.14E-08	6.37E-08	7.58E-08	8.90E-08	1.22E-07	1.33E-07	1.45E-07	1.59E-07	1.76E-07	1.90E-07	1.85E-07	1.77E-07	1.81E-07	1.85E-07	1.87E-07	1.95E-07	1.98E-07	1.99E-07
3.50E-09	1.42E-08	2.80E-08	3.94E-08	5.00E-08	5.81E-08	6.89E-08	1.02E-07	1.17E-07	1.28E-07	1.44E-07	1.58E-07	1.75E-07	1.70E-07	1.59E-07	1.67E-07	1.74E-07	1.75E-07	1.86E-07	1.91E-07	1.93E-07
-3.51E-09	4.53E-09	1.56E-08	2.60E-08	3.55E-08	4.22E-08	5.06E-08	7.61E-08	9.95E-08	1.08E-07	1.24E-07	1.41E-07	1.57E-07	1.49E-07	1.38E-07	1.51E-07	1.63E-07	1.60E-07	1.76E-07	1.82E-07	1.85E-07
-8.46E-09	-4.56E-09	4.31E-09	1.26E-08	2.16E-08	2.79E-08	3.39E-08	5.48E-08	7.86E-08	8.74E-08	1.00E-07	1.12E-07	1.35E-07	1.24E-07	1.16E-07	1.37E-07	1.54E-07	1.43E-07	1.67E-07	1.71E-07	1.77E-07
-1.11E-08	-1.23E-08	-5.02E-09	9.52E-10	8.17E-09	1.46E-08	1.84E-08	3.45E-08	5.74E-08	6.41E-08	7.63E-08	8.53E-08	9.36E-08	1.10E-07	9.54E-08	1.28E-07	1.48E-07	1.25E-07	1.58E-07	1.58E-07	1.68E-07
-1.20E-08	-1.66E-08	-1.28E-08	-9.93E-09	-4.30E-09	1.76E-09	4.48E-09	1.87E-08	3.55E-08	4.36E-08	5.61E-08	6.72E-08	6.60E-08	9.77E-08	7.61E-08	1.18E-07	1.43E-07	1.05E-07	1.46E-07	1.44E-07	1.53E-07
-1.23E-08	-1.74E-08	-1.81E-08	-1.92E-08	-1.57E-08	-1.05E-08	-9.16E-09	6.76E-09	1.86E-08	2.63E-08	3.74E-08	4.73E-08	4.87E-08	6.09E-08	5.89E-08	8.71E-08	1.18E-07	7.98E-08	1.08E-07	1.26E-07	1.11E-07
-1.19E-08	-1.72E-08	-2.03E-08	-2.53E-08	-2.50E-08	-2.16E-08	-2.13E-08	-4.78E-09	5.92E-09	7.74E-09	1.89E-08	2.74E-08	3.37E-08	1.67E-08	3.96E-08	4.09E-08	6.44E-08	5.06E-08	5.89E-08	8.77E-08	7.30E-08
-1.12E-08	-1.76E-08	-2.02E-08	-2.72E-08	-3.09E-08	-3.04E-08	-3.20E-08	-1.58E-08	-1.00E-08	-9.77E-09	3.52E-10	7.78E-09	1.24E-08	-3.54E-09	1.53E-08	7.98E-09	1.69E-08	2.36E-08	2.76E-08	4.33E-08	4.10E-08
-1.12E-08	-1.82E-08	-2.05E-08	-2.74E-08	-3.31E-08	-3.50E-08	-3.92E-08	-2.47E-08	-1.96E-08	-1.90E-08	-1.51E-08	-1.06E-08	-1.15E-08	-2.01E-08	-1.11E-08	-1.28E-08	-8.56E-09	-1.97E-09	3.52E-09	1.28E-08	1.56E-08
-1.19E-08	-1.80E-08	-2.10E-08	-2.75E-08	-3.33E-08	-3.60E-08	-4.18E-08	-3.10E-08	-3.19E-08	-2.37E-08	-2.63E-08	-2.44E-08	-2.59E-08	-3.62E-08	-3.12E-08	-3.16E-08	-2.76E-08	-2.21E-08	-1.73E-08	-9.94E-09	-6.74E-09
-1.27E-08	-1.82E-08	-2.15E-08	-2.71E-08	-3.37E-08	-3.64E-08	-4.19E-08	-5.03E-08	-5.73E-08	-4.98E-08	-3.76E-08	-3.55E-08	-3.80E-08	-5.06E-08	-4.64E-08	-4.72E-08	-4.31E-08	-3.91E-08	-3.46E-08	-2.68E-08	-2.46E-08
-1.30E-08	-1.82E-08	-2.19E-08	-2.71E-08	-3.41E-08	-3.74E-08	-4.28E-08	-5.06E-08	-5.68E-08	-5.97E-08	-6.16E-08	-6.15E-08	-4.74E-08	-5.85E-08	-5.66E-08	-5.75E-08	-5.41E-08	-5.15E-08	-4.77E-08	-4.28E-08	-3.84E-08
-1.24E-08	-1.78E-08	-2.15E-08	-2.77E-08	-3.44E-08	-3.81E-08	-4.43E-08	-5.02E-08	-5.65E-08	-5.88E-08	-6.19E-08	-6.55E-08	-6.27E-08	-6.08E-08	-6.18E-08	-6.23E-08	-5.98E-08	-5.85E-08	-5.64E-08	-5.04E-08	-4.76E-08
-1.16E-08	-1.74E-08	-2.06E-08	-2.78E-08	-3.38E-08	-3.80E-08	-4.43E-08	-4.92E-08	-5.59E-08	-5.88E-08	-6.18E-08	-6.51E-08	-6.32E-08	-6.06E-08	-6.21E-08	-6.34E-08	-6.07E-08	-6.03E-08	-5.97E-08	-5.44E-08	-5.08E-08
-1.11E-08	-1.69E-08	-2.09E-08	-2.73E-08	-3.29E-08	-3.77E-08	-4.31E-08	-4.76E-08	-5.57E-08	-5.89E-08	-6.09E-08	-6.55E-08	-6.40E-08	-6.00E-08	-6.14E-08	-6.38E-08	-6.04E-08	-6.05E-08	-5.92E-08	-5.48E-08	-5.28E-08
-1.06E-08	-1.64E-08	-2.11E-08	-2.72E-08	-3.31E-08	-3.78E-08	-4.25E-08	-4.63E-08	-5.54E-08	-5.83E-08	-5.99E-08	-6.56E-08	-6.33E-08	-5.96E-08	-6.14E-08	-6.46E-08	-6.04E-08	-6.05E-08	-5.86E-08	-5.44E-08	-5.31E-08
-1.01E-08	-1.58E-08	-2.09E-08	-2.81E-08	-3.33E-08	-3.72E-08	-4.31E-08	-4.72E-08	-5.49E-08	-5.72E-08	-5.94E-08	-6.43E-08	-6.32E-08	-6.17E-08	-6.14E-08	-6.47E-08	-6.07E-08	-5.94E-08	-5.90E-08	-5.64E-08	-5.27E-08
-9.52E-09	-1.57E-08	-2.07E-08	-2.84E-08	-3.27E-08	-3.62E-08	-4.27E-08	-4.77E-08	-5.52E-08	-5.65E-08	-6.00E-08	-6.34E-08	-6.41E-08	-6.29E-08	-6.10E-08	-6.43E-08	-6.05E-08	-5.83E-08	-5.89E-08	-5.69E-08	-5.05E-08
-9.80E-09	-1.57E-08	-2.09E-08	-2.77E-08	-3.23E-08	-3.58E-08	-4.18E-08	-4.76E-08	-5.58E-08	-5.76E-08	-6.14E-08	-6.47E-08	-6.45E-08	-6.33E-08	-6.10E-08	-6.45E-08	-6.00E-08	-5.80E-08	-5.82E-08	-5.67E-08	-4.81E-08
-9.12E-09	-1.57E-08	-2.19E-08	-2.76E-08	-3.31E-08	-3.55E-08	-4.14E-08	-4.55E-08	-5.54E-08	-5.76E-08	-6.21E-08	-6.54E-08	-6.46E-08	-6.26E-08	-6.09E-08	-6.51E-08	-6.02E-08	-5.85E-08	-5.84E-08	-5.51E-08	-4.70E-08
-8.14E-09	-1.54E-08	-2.14E-08	-2.77E-08	-3.36E-08	-3.51E-08	-4.13E-08	-4.32E-08	-5.56E-08	-5.71E-08	-6.16E-08	-6.37E-08	-6.37E-08	-6.29E-08	-6.11E-08	-6.48E-08	-6.02E-08	-5.76E-08	-5.82E-08	-5.30E-08	-4.51E-08
-8.56E-09	-1.57E-08	-1.97E-08	-2.69E-08	-3.28E-08	-3.43E-08	-4.05E-08	-4.16E-08	-5.41E-08	-5.71E-08	-6.09E-08	-6.26E-08	-6.31E-08	-6.23E-08	-6.11E-08	-6.37E-08	-6.01E-08	-5.68E-08	-5.83E-08	-5.13E-08	-4.86E-08
-8.49E-09	-1.51E-08	-1.82E-08	-2.55E-08	-3.19E-08	-3.40E-08	-4.00E-08	-4.71E-08	-5.41E-08	-5.79E-08	-6.08E-08	-6.29E-08	-6.35E-08	-6.17E-08	-6.01E-08	-6.26E-08	-6.14E-08	-5.79E-08	-5.83E-08	-5.03E-08	-4.93E-08
-8.14E-09	-1.48E-08	-1.89E-08	-2.52E-08	-3.18E-08	-3.48E-08	-4.03E-08	-4.77E-08	-5.33E-08	-5.67E-08	-6.22E-08	-6.44E-08	-6.34E-08	-6.14E-08	-5.99E-08	-6.32E-08	-6.24E-08	-5.77E-08	-5.80E-08	-5.10E-08	-5.02E-08
-9.44E-09	-1.48E-08	-1.92E-08	-2.50E-08	-3.17E-08	-3.54E-08	-4.09E-08	-4.80E-08	-5.30E-08	-5.60E-08	-6.24E-08	-6.48E-08	-6.38E-08	-6.17E-08	-6.17E-08	-6.32E-08	-6.23E-08	-5.64E-08	-5.74E-08	-5.47E-08	-4.98E-08
-9.17E-09	-1.44E-08	-2.06E-08	-2.45E-08	-3.06E-08	-3.53E-08	-4.13E-08	-4.77E-08	-5.22E-08	-5.68E-08	-6.14E-08	-6.44E-08	-6.42E-08	-6.12E-08	-6.18E-08	-6.17E-08	-6.21E-08	-5.69E-08	-5.79E-08	-5.49E-08	-4.89E-08
-7.48E-09	-1.39E-08	-2.05E-08	-2.34E-08	-3.01E-08	-3.60E-08	-4.11E-08	-4.67E-08	-5.08E-08	-5.79E-08	-6.18E-08	-6.38E-08	-6.30E-08	-6.25E-08	-6.16E-08	-6.05E-08	-6.13E-08	-5.64E-08	-5.79E-08	-5.48E-08	-4.52E-08
-7.00E-09	-1.29E-08	-2.01E-08	-2.21E-08	-3.08E-08	-3.67E-08	-4.02E-08	-4.63E-08	-4.85E-08	-5.75E-08	-6.25E-08	-6.25E-08	-6.29E-08	-6.28E-08	-6.07E-08	-6.13E-08	-6.03E-08	-5.46E-08	-5.76E-08	-5.50E-08	-3.96E-08
-7.90E-09	-1.25E-08	-1.99E-08	-2.19E-08	-3.07E-08	-3.64E-08	-4.01E-08	-4.40E-08	-4.55E-08	-5.67E-08	-6.29E-08	-6.06E-08	-6.15E-08	-6.21E-08	-6.10E-08	-6.14E-08	-5.94E-08	-5.44E-08	-5.71E-08	-5.46E-08	-3.61E-08
-8.44E-09	-1.27E-08	-1.97E-08	-2.56E-08	-3.20E-08	-3.56E-08	-4.02E-08	-4.13E-08	-4.64E-08	-5.74E-08	-6.17E-08	-6.05E-08	-6.11E-08	-6.08E-08	-6.08E-08	-6.07E-08	-5.90E-08	-5.42E-08	-5.68E-08	-5.41E-08	-3.52E-08
-7.80E-09	-1.33E-08	-1.89E-08	-2.70E-08	-3.22E-08	-3.48E-08	-4.11E-08	-4.19E-08	-5.46E-08	-5.75E-08	-6.04E-08	-6.31E-08	-6.06E-08	-6.04E-08	-5.98E-08	-6.01E-08	-5.87E-08	-5.38E-08	-5.61E-08	-5.23E-08	-4.59E-08
-6.64E-09	-1.30E-08	-1.84E-08	-2.65E-08	-3.13E-08	-3.48E-08	-4.09E-08	-4.97E-08	-5.51E-08	-5.69E-08	-5.95E-08	-6.27E-08	-5.92E-08	-6.11E-08	-5.79E-08	-5.78E-08	-6.03E-08	-5.56E-08	-5.55E-08	-5.18E-08	-5.23E-08
-6.96E-09	-1.30E-08	-1.99E-08	-2.61E-08	-3.11E-08	-3.44E-08	-4.11E-08	-4.91E-08	-5.45E-08	-5.68E-08	-5.93E-08	-6.16E-08	-5.81E-08	-6.10E-08	-5.76E-08	-5.80E-08	-6.00E-08	-5.60E-08	-5.48E-08	-5.36E-08	-4.94E-08
-8.00E-09	-1.40E-08	-2.17E-08	-2.62E-08	-3.24E-08	-3.43E-08	-4.16E-08	-4.93E-08	-5.36E-08	-5.58E-08	-5.87E-08	-6.12E-08	-5.46E-08	-5.90E-08	-5.92E-08	-5.92E-08	-5.99E-08	-5.64E-08	-5.36E-08	-5.27E-08	-4.77E-08
-8.44E-09	-1.45E-08	-2.18E-08	-2.67E-08	-3.33E-08	-3.43E-08	-4.16E-08	-4.93E-08	-5.32E-08	-5.45E-08	-5.69E-08	-6.03E-08	-5.20E-08	-5.73E-08	-6.05E-08	-5.89E-08	-5.74E-08	-5.60E-08	-5.30E-08	-5.20E-08	-4.73E-08
-9.28E-09	-1.44E-08	-2.15E-08	-2.70E-08	-3.33E-08	-3.42E-08	-4.02E-08	-4.91E-08	-5.21E-08	-5.38E-08	-5.52E-08	-5.93E-08	-5.21E-08	-5.90E-08	-5.98E-08	-5.78E-08	-5.56E-08	-5.53E-08	-5.43E-08	-5.38E-08	-4.83E-08
-9.76E-09	-1.43E-08	-2.17E-08	-2.72E-08	-3.33E-08	-3.44E-08	-3.85E-08	-4.84E-08	-4.91E-08	-5.11E-08	-5.28E-08	-5.65E-08	-5.80E-08	-5.71E-08	-5.70E-08	-5.62E-08	-5.63E-08	-5.43E-08	-5.41E-08	-5.38E-08	-4.80E-08
-1.00E-08	-1.52E-08	-2.18E-08	-2.72E-08	-3.36E-08	-3.44E-08	-3.69E-08	-4.60E-08	-4.36E-08	-4.48E-08	-4.68E-08	-4.93E-08	-5.27E-08	-5.22E-08	-5.19E-08	-5.22E-08	-5.45E-08	-5.13E-08	-5.19E-08	-5.27E-08	-4.59E-08
-1.04E-08	-1.58E-08	-2.21E-08	-2.71E-08	-3.33E-08	-3.29E-08	-3.35E-08	-4.19E-08	-3.48E-08	-3.51E-08	-3.60E-08	-3.78E-08	-4.25E-08	-4.25E-08	-4.33E-08	-4.40E-08	-4.69E-08	-4.60E-08	-4.71E-08	-4.89E-08	-4.46E-08
-1.10E-08	-1.61E-08	-2.19E-08	-2.57E-08	-3.10E-08	-2.84E-08	-2.75E-08	-3.30E-08	-2.10E-08	-1.93E-08	-1.96E-08	-2.00E-08	-2.53E-08	-2.64E-08	-2.89E-08	-3.09E-08	-3.45E-08	-3.60E-08	-3.87E-08	-4.34E-08	-4.22E-08
-1.06E-08	-1.51E-08	-1.98E-08	-2.23E-08	-2.55E-08	-2.03E-08	-1.65E-08	-1.78E-08	-1.22E-09	3.33E-09	4.63E-09	6.55E-09	1.12E-09	-1.31E-09	-5.05E-09	-9.76E-09	-1.54E-08	-1.92E-08	-2.51E-08	-3.27E-08	-3.56E-08
-9.93E-09	-1.27E-08	-1.55E-08	-1.65E-08	-1.52E-08	-6.37E-09	2.64E-09	6.87E-09	2.83E-08	3.54E-08	3.75E-08	3.89E-08	3.34E-08	3.11E-08	2.67E-08	2.04E-08	1.18E-08	5.53E-09	-3.90E-09	-1.53E-08	-2.28E-08
-8.86E-09	-8.68E-09	-8.53E-09	-5.73E-09	2.18E-09	1.66E-08	3.10E-08	3.96E-08	6.24E-08	6.98E-08	7.20E-08	7.23E-08	6.61E-08	6.41E-08	5.89E-08	5.24E-08	4.24E-08	3.40E-08	2.26E-08	8.34E-09	-3.50E-09
-6.22E-09	-2.33E-09	3.16E-09	1.29E-08	2.88E-08	4.80E-08	6.42E-08	7.32E-08	9.66E-08	1.05E-07	1.07E-07	1.06E-07	9.89E-08	9.59E-08	8.96E-08	8.18E-08	7.09E-08	6.04E-08	4.80E-08	3.32E-08	1.86E-08
-8.61E-10	8.73E-09	2.19E-08	4.00E-08	6.08E-08	8.01E-08	9.60E-08	1.05E-07	1.29E-07	1.39E-07	1.42E-07	1.38E-07	1.30E-07	1.25E-07	1.19E-07	1.09E-07	9.59E-08	8.47E-08	7.08E-08	5.46E-08	3.92E-08
7.96E-09	2.55E-08	4.76E-08	7.02E-08	9.17E-08	1.09E-07	1.25E-07	1.34E-07	1.60E-07	1.72E-07	1.76E-07	1.70E-07	1.59E-07	1.53E-07	1.45E-07	1.32E-07	1.17E-07	1.06E-07	9.04E-08	7.22E-08	5.69E-08
2.13E-08	4.75E-08	7.52E-08	9.84E-08	1.20E-07	1.36E-07	1.51E-07	1.60E-07	1.88E-07	2.02E-07	2.07E-07	1.97E-07	1.85E-07	1.76E-07	1.67E-07	1.52E-07	1.36E-07	1.23E-07	1.06E-07	8.62E-08	7.14E-08
3.97E-08	6.97E-08	9.97E-08	1.24E-07	1.45E-07	1.60E-07	1.72E-07	1.81E-07	2.10E-07	2.25E-07	2.29E-07	2.17E-07	2.06E-07	1.95E-07	1.84E-07	1.68E-07	1.52E-07	1.36E-07	1.19E-07	9.79E-08	8.33E-08
5.78E-08	8.85E-08	1.19E-07	1.44E-07	1.66E-07	1.80E-07	1.89E-07	1.97E-07	2.22E-07	2.36E-07	2.37E-07	2.27E-07	2.19E-07	2.06E-07	1.95E-07	1.79E-07	1.63E-07	1.47E-07	1.30E-07	1.06E-07	9.17E-08
7.06E-08	1.03E-07	1.33E-07	1.59E-07	1.82E-07	1.93E-07	2.02E-07	2.11E-07	2.27E-07	2.41E-07	2.37E-07	2.30E-07	2.23E-07	2.10E-07	2.01E-07	1.85E-07	1.69E-07	1.54E-07	1.36E-07	1.12E-07	9.71E-08
7.78E-08	1.11E-07	1.42E-07	1.67E-07	1.90E-07	2.00E-07	2.10E-07	2.18E-07	2.28E-07	2.37E-07	2.35E-07	2.30E-07	2.26E-07	2.12E-07	2.02E-07	1.87E-07	1.72E-07	1.56E-07	1.38E-07	1.16E-07	1.00E-07
8.16E-08	1.14E-07	1.43E-07	1.68E-07	1.89E-07	2.00E-07	2.12E-07	2.17E-07	2.26E-07	2.29E-07	2.29E-07	2.28E-07	2.25E-07	2.12E-07	2.01E-07	1.86E-07	1.71E-07	1.55E-07	1.38E-07	1.16E-07	1.02E-07
8.20E-08	1.13E-07	1.40E-07	1.63E-07	1.85E-07	1.96E-07	2.10E-07	2.13E-07	2.21E-07	2.21E-07	2.21E-07	2.23E-07	2.21E-07	2.08E-07	1.97E-07	1.82E-07	1.66E-07	1.52E-07	1.36E-07	1.15E-07	1.01E-07
7.80E-08	1.07E-07	1.34E-07	1.56E-07	1.78E-07	1.90E-07	2.04E-07	2.08E-07	2.14E-07	2.13E-07	2.12E-07	2.16E-07	2.14E-07	1.99E-07	1.87E-07	1.72E-07	1.58E-07	1.45E-07	1.29E-07	1.10E-07	9.53E-08
7.03E-08	9.83E-08	1.25E-07	1.46E-07	1.68E-07	1.80E-07	1.94E-07	2.01E-07	2.06E-07	2.03E-07	2.01E-07	2.05E-07	1.99E-07	1.83E-07	1.73E-07	1.60E-07	1.47E-07	1.34E-07	1.19E-07	1.01E-07	8.62E-08
5.98E-08	8.61E-08	1.12E-07	1.33E-07	1.55E-07	1.65E-07	1.80E-07	1.90E-07	1.97E-07	1.94E-07	1.89E-07	1.90E-07	1.80E-07	1.64E-07	1.58E-07	1.46E-07	1.33E-07	1.21E-07	1.06E-07	8.89E-08	7.43E-08
4.74E-08	7.07E-08	9.50E-08	1.15E-07	1.36E-07	1.45E-07	1.61E-07	1.74E-07	1.88E-07	1.82E-07	1.74E-07	1.74E-07	1.58E-07	1.47E-07	1.40E-07	1.29E-07	1.17E-07	1.05E-07	9.11E-08	7.33E-08	6.05E-08
3.30E-08	5.31E-08	7.49E-08	9.29E-08	1.13E-07	1.22E-07	1.37E-07	1.56E-07	1.72E-07	1.66E-07	1.58E-07	1.59E-07	1.39E-07	1.27E-07	1.19E-07	1.09E-07	9.79E-08	8.79E-08	7.38E-08	5.76E-08	4.53E-08
1.84E-08	3.45E-08	5.34E-08	6.92E-08	8.83E-08	9.75E-08	1.11E-07	1.29E-07	1.40E-07	1.48E-07	1.41E-07	1.45E-07	1.18E-07	1.06E-07	9.70E-08	8.83E-08	7.79E-08	6.74E-08	5.45E-08	4.09E-08	2.87E-08
5.85E-09	1.61E-08	3.15E-08	4.61E-08	6.22E-08	7.20E-08	8.52E-08	1.04E-07	1.16E-07	1.28E-07	1.23E-07	1.28E-07	1.00E-07	8.61E-08	7.41E-08	6.59E-08	5.56E-08	4.53E-08	3.37E-08	2.39E-08	1.27E-08

-2.44E-09	5.90E-10	1.18E-08	2.39E-08	3.70E-08	4.57E-08	5.84E-08	7.83E-08	8.97E-08	1.05E-07	9.01E-08	1.07E-07	8.19E-08	6.74E-08	5.05E-08	4.23E-08	3.27E-08	2.32E-08	1.22E-08	6.91E-09	5.03E-10
-4.86E-09	-8.64E-09	-4.22E-09	3.50E-09	1.42E-08	2.10E-08	2.99E-08	5.33E-08	5.45E-08	6.35E-08	6.07E-08	8.20E-08	6.12E-08	4.87E-08	2.67E-08	1.76E-08	1.01E-08	1.32E-09	-8.40E-09	-5.76E-09	-7.58E-09
-5.80E-09	-1.12E-08	-1.29E-08	-1.18E-08	-4.37E-09	9.39E-12	5.53E-09	2.57E-08	1.77E-08	1.84E-08	3.18E-08	5.04E-08	3.28E-08	2.73E-08	4.00E-09	-5.70E-09	-9.87E-09	-1.93E-08	-2.60E-08	-1.36E-08	-1.55E-08
-5.51E-09	-1.14E-08	-1.53E-08	-1.84E-08	-1.69E-08	-1.54E-08	-1.26E-08	-3.42E-10	-1.20E-08	-1.04E-08	-4.39E-09	1.25E-08	-7.14E-11	-4.14E-09	-1.61E-08	-2.38E-08	-2.54E-08	-3.49E-08	-3.76E-08	-2.20E-08	-3.99E-08
-4.79E-09	-1.06E-08	-1.61E-08	-1.90E-08	-2.23E-08	-2.37E-08	-2.43E-08	-1.65E-08	-3.03E-08	-3.05E-08	-3.42E-08	-1.83E-08	-2.45E-08	-2.49E-08	-3.54E-08	-3.72E-08	-3.83E-08	-4.20E-08	-4.19E-08	-4.08E-08	-4.12E-08
-3.78E-09	-9.15E-09	-1.54E-08	-1.89E-08	-2.29E-08	-2.58E-08	-2.89E-08	-3.36E-08	-3.69E-08	-3.99E-08	-4.44E-08	-3.91E-08	-4.16E-08	-4.33E-08	-4.49E-08	-4.53E-08	-4.40E-08	-4.33E-08	-4.24E-08	-3.97E-08	-3.89E-08
-3.62E-09	-7.53E-09	-1.48E-08	-1.91E-08	-2.23E-08	-2.54E-08	-3.00E-08	-3.39E-08	-3.77E-08	-4.07E-08	-4.50E-08	-4.54E-08	-4.52E-08	-4.41E-08	-4.45E-08	-4.49E-08	-4.38E-08	-4.37E-08	-4.25E-08	-3.99E-08	-3.83E-08
-4.68E-09	-7.20E-09	-1.34E-08	-1.91E-08	-2.17E-08	-2.44E-08	-2.93E-08	-3.27E-08	-3.77E-08	-3.98E-08	-4.44E-08	-4.52E-08	-4.59E-08	-4.47E-08	-4.37E-08	-4.43E-08	-4.32E-08	-4.35E-08	-4.23E-08	-4.10E-08	-3.72E-08
-3.98E-09	-7.41E-09	-1.19E-08	-1.76E-08	-2.12E-08	-2.36E-08	-2.82E-08	-3.06E-08	-3.76E-08	-3.91E-08	-4.31E-08	-4.42E-08	-4.54E-08	-4.45E-08	-4.28E-08	-4.40E-08	-4.22E-08	-4.23E-08	-4.25E-08	-4.00E-08	-3.77E-08
-2.62E-09	-8.02E-09	-1.12E-08	-1.60E-08	-2.05E-08	-2.30E-08	-2.75E-08	-3.04E-08	-3.62E-08	-3.93E-08	-4.25E-08	-4.38E-08	-4.52E-08	-4.34E-08	-4.25E-08	-4.37E-08	-4.07E-08	-4.05E-08	-4.28E-08	-3.90E-08	-3.81E-08

1.70E-16	3.06E-16	5.14E-16	1.07E-15	1.27E-15	1.76E-15	2.47E-15	3.12E-15	4.14E-15	5.10E-15	5.64E-15	5.64E-15	6.67E-15	5.90E-15	5.82E-15	5.97E-15	6.13E-15	5.48E-15	5.05E-15	4.20E-15	3.41E-15
1.90E-16	3.11E-16	5.30E-16	1.08E-15	1.21E-15	1.71E-15	2.43E-15	3.22E-15	4.13E-15	5.01E-15	5.66E-15	5.74E-15	6.75E-15	6.13E-15	5.75E-15	5.68E-15	5.92E-15	5.29E-15	5.01E-15	4.18E-15	3.26E-15
1.76E-16	3.12E-16	5.03E-16	1.06E-15	1.22E-15	1.75E-15	2.45E-15	3.29E-15	4.09E-15	4.93E-15	5.90E-15	6.11E-15	6.66E-15	5.99E-15	5.44E-15	5.15E-15	5.67E-15	5.12E-15	4.98E-15	4.26E-15	3.28E-15
1.85E-16	2.80E-16	5.22E-16	1.01E-15	1.22E-15	1.66E-15	2.41E-15	3.33E-15	4.09E-15	4.85E-15	5.97E-15	6.14E-15	6.62E-15	5.74E-15	5.16E-15	4.54E-15	5.33E-15	5.04E-15	4.85E-15	4.43E-15	3.28E-15
2.11E-16	2.31E-16	5.20E-16	9.59E-16	1.25E-15	1.54E-15	2.35E-15	3.39E-15	4.36E-15	4.95E-15	5.87E-15	6.09E-15	6.70E-15	6.01E-15	4.77E-15	3.74E-15	4.79E-15	5.01E-15	4.72E-15	4.46E-15	4.09E-15
2.09E-16	2.41E-16	5.35E-16	9.39E-16	1.31E-15	1.58E-15	2.31E-15	3.40E-15	4.51E-15	4.78E-15	5.76E-15	6.61E-15	6.70E-15	6.05E-15	4.41E-15	3.86E-15	4.81E-15	5.14E-15	4.77E-15	4.43E-15	4.14E-15
1.88E-16	2.95E-16	5.66E-16	9.31E-16	1.28E-15	1.66E-15	2.38E-15	3.40E-15	4.45E-15	4.78E-15	5.62E-15	6.70E-15	6.51E-15	6.06E-15	4.57E-15	4.36E-15	5.54E-15	5.29E-15	4.88E-15	4.42E-15	4.13E-15
1.58E-16	2.49E-16	5.09E-16	8.92E-16	1.21E-15	1.62E-15	2.37E-15	3.28E-15	4.31E-15	5.18E-15	5.39E-15	6.68E-15	6.44E-15	6.06E-15	5.28E-15	5.61E-15	6.20E-15	5.33E-15	4.73E-15	4.38E-15	4.19E-15
1.30E-16	1.98E-16	4.33E-16	8.22E-16	1.24E-15	1.55E-15	2.26E-15	2.97E-15	3.93E-15	5.19E-15	5.41E-15	6.48E-15	6.50E-15	6.10E-15	5.67E-15	5.85E-15	5.93E-15	5.33E-15	4.64E-15	4.26E-15	4.11E-15
1.19E-16	2.29E-16	3.56E-16	7.56E-16	1.28E-15	1.54E-15	2.20E-15	2.80E-15	3.57E-15	5.04E-15	5.28E-15	6.45E-15	6.74E-15	6.06E-15	5.85E-15	5.89E-15	5.76E-15	5.23E-15	4.56E-15	4.09E-15	3.96E-15
1.11E-16	2.18E-16	2.90E-16	7.36E-16	1.18E-15	1.57E-15	2.12E-15	2.74E-15	3.76E-15	4.85E-15	4.85E-15	6.66E-15	6.59E-15	5.92E-15	5.79E-15	5.94E-15	5.84E-15	5.05E-15	4.52E-15	4.08E-15	3.84E-15
1.22E-16	1.76E-16	2.76E-16	7.46E-16	1.07E-15	1.61E-15	2.04E-15	2.87E-15	3.91E-15	4.85E-15	4.89E-15	6.54E-15	6.42E-15	5.97E-15	5.57E-15	5.85E-15	6.02E-15	4.88E-15	4.63E-15	4.25E-15	3.71E-15
1.20E-16	1.31E-16	3.39E-16	7.23E-16	9.82E-16	1.53E-15	2.02E-15	2.96E-15	3.86E-15	4.81E-15	4.98E-15	6.24E-15	6.24E-15	5.90E-15	5.54E-15	5.92E-15	5.91E-15	4.72E-15	4.52E-15	4.22E-15	3.64E-15
9.43E-17	1.34E-16	3.75E-16	6.84E-16	9.61E-16	1.52E-15	1.99E-15	2.73E-15	3.81E-15	4.57E-15	4.88E-15	6.02E-15	6.15E-15	5.74E-15	5.62E-15	5.85E-15	5.57E-15	4.71E-15	4.44E-15	4.02E-15	3.59E-15
7.24E-17	2.23E-16	4.27E-16	7.01E-16	1.03E-15	1.62E-15	1.90E-15	2.48E-15	4.09E-15	4.26E-15	4.51E-15	6.16E-15	6.23E-15	5.53E-15	5.67E-15	5.86E-15	5.32E-15	4.80E-15	4.44E-15	3.91E-15	3.64E-15
7.17E-17	2.55E-16	4.05E-16	7.13E-16	1.04E-15	1.64E-15	1.96E-15	2.89E-15	4.14E-15	4.41E-15	4.77E-15	6.24E-15	6.12E-15	5.63E-15	5.66E-15	5.72E-15	5.38E-15	4.76E-15	4.45E-15	3.93E-15	3.75E-15
9.02E-17	2.17E-16	3.50E-16	6.63E-16	1.06E-15	1.60E-15	2.15E-15	3.29E-15	4.16E-15	4.52E-15	5.07E-15	6.02E-15	6.37E-15	5.84E-15	5.59E-15	5.72E-15	5.58E-15	5.01E-15	4.50E-15	4.06E-15	3.84E-15
8.55E-17	1.82E-16	3.54E-16	6.12E-16	1.01E-15	1.52E-15	2.10E-15	3.21E-15	4.07E-15	4.39E-15	5.01E-15	6.00E-15	6.71E-15	5.97E-15	5.70E-15	5.58E-15	5.59E-15	4.98E-15	4.55E-15	4.16E-15	3.91E-15
7.21E-17	2.00E-16	3.81E-16	5.79E-16	1.08E-15	1.56E-15	1.94E-15	3.07E-15	3.91E-15	4.29E-15	4.99E-15	5.99E-15	6.74E-15	5.86E-15	5.70E-15	5.67E-15	5.63E-15	5.01E-15	4.71E-15	4.26E-15	3.79E-15
7.29E-17	2.00E-16	4.02E-16	5.93E-16	1.14E-15	1.61E-15	1.93E-15	3.09E-15	3.75E-15	4.33E-15	5.20E-15	6.29E-15	6.52E-15	5.74E-15	5.66E-15	5.68E-15	5.63E-15	5.03E-15	4.92E-15	4.42E-15	3.71E-15
7.27E-17	2.10E-16	4.07E-16	7.15E-16	1.25E-15	1.76E-15	2.09E-15	3.03E-15	3.68E-15	4.41E-15	5.16E-15	6.09E-15	6.42E-15	5.77E-15	5.72E-15	5.81E-15	5.69E-15	4.90E-15	4.91E-15	4.45E-15	3.60E-15
5.58E-17	2.04E-16	4.49E-16	7.61E-16	1.25E-15	1.79E-15	2.08E-15	3.00E-15	3.80E-15	4.48E-15	5.20E-15	5.95E-15	6.55E-15	5.82E-15	5.81E-15	5.91E-15	5.76E-15	4.89E-15	4.83E-15	4.43E-15	3.32E-15
3.61E-17	1.73E-16	5.31E-16	7.75E-16	1.23E-15	1.78E-15	2.07E-15	3.02E-15	3.90E-15	4.34E-15	5.32E-15	5.99E-15	6.42E-15	5.65E-15	5.87E-15	5.68E-15	5.68E-15	5.20E-15	4.84E-15	4.36E-15	3.09E-15
2.53E-17	1.53E-16	5.72E-16	7.98E-16	1.22E-15	1.77E-15	2.22E-15	3.07E-15	3.89E-15	4.50E-15	5.24E-15	6.12E-15	6.24E-15	5.58E-15	5.85E-15	5.57E-15	5.63E-15	5.32E-15	4.89E-15	4.35E-15	3.52E-15
5.46E-17	2.08E-16	5.89E-16	8.35E-16	1.28E-15	1.74E-15	2.26E-15	2.78E-15	3.82E-15	4.83E-15	4.83E-15	5.84E-15	6.17E-15	5.57E-15	5.66E-15	5.65E-15	5.62E-15	5.16E-15	4.97E-15	4.37E-15	3.63E-15
1.48E-16	3.20E-16	6.04E-16	8.67E-16	1.40E-15	1.79E-15	2.12E-15	2.25E-15	3.84E-15	4.71E-15	4.16E-15	5.44E-15	6.29E-15	5.88E-15	5.57E-15	5.62E-15	5.53E-15	5.06E-15	5.00E-15	4.37E-15	3.45E-15
1.80E-16	3.27E-16	6.09E-16	8.93E-16	1.43E-15	1.76E-15	1.86E-15	1.58E-15	3.83E-15	4.56E-15	3.55E-15	5.08E-15	6.33E-15	5.95E-15	5.63E-15	5.56E-15	5.69E-15	5.15E-15	4.98E-15	4.31E-15	3.44E-15
1.53E-16	3.50E-16	6.01E-16	8.66E-16	1.40E-15	1.61E-15	1.59E-15	1.13E-15	3.79E-15	4.46E-15	3.60E-15	5.17E-15	6.29E-15	5.83E-15	5.65E-15	5.51E-15	5.67E-15	5.08E-15	4.98E-15	4.38E-15	3.64E-15
1.11E-16	2.93E-16	6.60E-16	7.78E-16	1.34E-15	1.51E-15	1.56E-15	2.14E-15	3.80E-15	4.28E-15	4.90E-15	5.58E-15	5.96E-15	5.87E-15	5.67E-15	5.48E-15	5.54E-15	5.01E-15	4.87E-15	4.38E-15	3.75E-15
5.57E-17	1.94E-16	6.95E-16	6.95E-16	1.27E-15	1.62E-15	1.92E-15	1.96E-15	3.57E-15	4.24E-15	4.82E-15	5.45E-15	5.63E-15	5.81E-15	5.62E-15	5.40E-15	5.46E-15	4.91E-15	4.71E-15	4.32E-15	3.80E-15
3.33E-17	1.79E-16	6.41E-16	6.03E-16	1.22E-15	1.73E-15	2.25E-15	1.83E-15	3.38E-15	4.39E-15	4.69E-15	5.31E-15	5.81E-15	5.65E-15	5.45E-15	5.52E-15	5.51E-15	4.80E-15	4.70E-15	4.34E-15	3.89E-15
9.07E-17	2.54E-16	5.59E-16	5.18E-16	1.15E-15	1.75E-15	2.28E-15	1.97E-15	3.57E-15	4.60E-15	4.49E-15	5.01E-15	6.00E-15	5.39E-15	5.32E-15	5.47E-15	5.43E-15	4.87E-15	4.85E-15	4.32E-15	3.88E-15
1.21E-16	2.45E-16	5.12E-16	5.31E-16	1.11E-15	1.70E-15	2.17E-15	3.58E-15	3.96E-15	4.64E-15	4.38E-15	4.70E-15	6.00E-15	5.32E-15	5.23E-15	5.05E-15	4.95E-15	4.67E-15	4.56E-15	4.07E-15	3.61E-15
1.12E-16	2.32E-16	4.90E-16	7.31E-16	1.18E-15	1.68E-15	2.06E-15	3.33E-15	3.80E-15	4.40E-15	4.39E-15	4.85E-15	5.52E-15	4.99E-15	4.84E-15	4.28E-15	4.16E-15	4.10E-15	4.00E-15	3.52E-15	2.91E-15
1.07E-16	2.20E-16	4.86E-16	8.30E-16	1.17E-15	1.61E-15	2.07E-15	2.95E-15	3.32E-15	3.81E-15	4.34E-15	4.39E-15	4.72E-15	4.21E-15	4.00E-15	3.28E-15	3.27E-15	3.15E-15	3.00E-15	2.69E-15	2.09E-15
1.07E-16	1.65E-16	4.58E-16	7.96E-16	1.11E-15	1.52E-15	2.04E-15	2.62E-15	2.99E-15	2.99E-15	3.40E-15	3.31E-15	3.46E-15	2.96E-15	2.59E-15	1.94E-15	2.09E-15	1.86E-15	1.70E-15	1.50E-15	1.13E-15
1.03E-16	1.22E-16	4.00E-16	7.34E-16	9.79E-16	1.22E-15	1.60E-15	2.27E-15	2.11E-15	2.04E-15	2.00E-15	1.78E-15	1.79E-15	1.46E-15	1.15E-15	5.99E-16	8.33E-16	6.26E-16	5.51E-16	4.86E-16	3.41E-16
9.66E-17	1.23E-16	4.09E-16	6.85E-16	7.52E-16	8.09E-16	9.12E-16	1.36E-15	9.27E-16	8.08E-16	7.29E-16	5.51E-16	5.17E-16	3.79E-16	2.36E-16	5.49E-17	7.97E-17	2.52E-17	2.15E-17	1.68E-17	2.17E-17
8.12E-17	1.20E-16	3.83E-16	5.22E-16	4.42E-16	3.05E-16	2.49E-16	4.53E-16	1.38E-16	8.53E-17	6.44E-17	1.34E-17	6.05E-18	9.51E-19	3.24E-17	1.97E-16	1.43E-16	2.31E-16	2.01E-16	2.18E-16	1.78E-16
6.63E-17	7.87E-17	2.53E-16	2.26E-16	9.32E-17	6.12E-18	8.06E-19	1.08E-17	4.24E-17	1.21E-16	1.53E-16	2.78E-16	3.63E-16	5.04E-16	7.78E-16	1.08E-15	1.11E-15	1.26E-15	1.09E-15	1.12E-15	1.04E-15
5.61E-17	6.18E-17	8.89E-17	7.96E-18	2.42E-17	1.87E-16	3.25E-16	2.35E-16	6.65E-16	9.95E-16	1.13E-15	1.42E-15	1.71E-15	1.96E-15	2.52E-15	2.89E-15	2.96E-15	3.19E-15	2.75E-15	2.70E-15	2.31E-15
2.10E-17	1.00E-17	1.73E-18	1.02E-16	3.84E-16	9.04E-16	1.31E-15	1.23E-15	2.14E-15	2.75E-15	3.07E-15	3.62E-15	4.07E-15	4.52E-15	5.16E-15	5.66E-15	5.86E-15	6.06E-15	5.27E-15	4.98E-15	4.41E-15
2.01E-18	4.19E-17	1.88E-16	5.32E-16	1.24E-15	2.18E-15	3.03E-15	3.07E-15	4.51E-15	5.54E-15	6.00E-15	7.05E-15	7.56E-15	8.30E-15	8.85E-15	9.51E-15	9.97E-15	9.91E-15	8.80E-15	8.02E-15	7.10E-15
7.94E-17	3.17E-16	6.90E-16	1.34E-15	2.58E-15	3.99E-15	5.47E-15	5.76E-15	7.85E-15	9.40E-15	9.91E-15	1.15E-14	1.22E-14	1.29E-14	1.37E-14	1.44E-14	1.51E-14	1.49E-14	1.33E-14	1.17E-14	1.05E-14
3.33E-16	8.42E-16	1.48E-15	2.45E-15	4.19E-15	6.18E-15	8.40E-15	9.21E-15	1.21E-14	1.39E-14	1.44E-14	1.64E-14	1.75E-14	1.81E-14	1.96E-14	2.04E-14	2.09E-14	2.08E-14	1.83E-14	1.58E-14	1.42E-14
8.20E-16	1.57E-15	2.46E-15	3.75E-15	5.94E-15	8.49E-15	1.14E-14	1.28E-14	1.64E-14	1.84E-14	1.87E-14	2.12E-14	2.27E-14	2.36E-14	2.60E-14	2.68E-14	2.67E-14	2.65E-14	2.33E-14	2.04E-14	1.80E-14
1.50E-15	2.45E-15	3.59E-15	5.19E-15	7.62E-15	1.04E-14	1.38E-14	1.57E-14	1.98E-14	2.24E-14	2.25E-14	2.56E-14	2.74E-14	2.92E-14	3.20E-14	3.31E-14	3.19E-14	3.14E-14	2.81E-14	2.48E-14	2.07E-14
2.29E-15	3.42E-15	4.89E-15	6.63E-15	9.07E-15	1.20E-14	1.55E-14	1.75E-14	2.23E-14	2.60E-14	2.63E-14	2.98E-14	3.23E-14	3.45E-14	3.72E-14	3.84E-14	3.67E-14	3.60E-14	3.27E-14	2.89E-14	2.40E-14

3.08E-15	4.43E-15	6.07E-15	7.89E-15	1.03E-14	1.31E-14	1.65E-14	1.87E-14	2.47E-14	2.94E-14	3.01E-14	3.40E-14	3.77E-14	3.94E-14	4.18E-14	4.16E-14	4.09E-14	4.06E-14	3.72E-14	3.29E-14	2.71E-14
3.69E-15	5.25E-15	6.89E-15	8.81E-15	1.15E-14	1.39E-14	1.71E-14	1.98E-14	2.65E-14	3.24E-14	3.29E-14	3.77E-14	4.27E-14	4.42E-14	4.59E-14	4.41E-14	4.36E-14	4.42E-14	4.10E-14	3.68E-14	3.01E-14
3.77E-15	5.54E-15	7.33E-15	9.40E-15	1.22E-14	1.43E-14	1.76E-14	2.05E-14	2.70E-14	3.36E-14	3.43E-14	4.03E-14	4.66E-14	4.79E-14	4.91E-14	4.64E-14	4.61E-14	4.70E-14	4.44E-14	4.04E-14	3.32E-14
3.25E-15	5.16E-15	7.27E-15	9.67E-15	1.25E-14	1.43E-14	1.76E-14	2.06E-14	2.62E-14	3.15E-14	3.44E-14	4.08E-14	4.90E-14	4.94E-14	4.96E-14	4.80E-14	4.76E-14	4.86E-14	4.73E-14	4.34E-14	3.64E-14
2.62E-15	4.38E-15	6.67E-15	9.38E-15	1.21E-14	1.37E-14	1.70E-14	2.02E-14	2.50E-14	2.98E-14	3.36E-14	4.01E-14	4.91E-14	4.86E-14	4.86E-14	4.77E-14	4.78E-14	4.92E-14	4.94E-14	4.57E-14	3.90E-14
2.00E-15	3.49E-15	5.72E-15	8.32E-15	1.08E-14	1.24E-14	1.59E-14	1.95E-14	2.37E-14	2.80E-14	3.23E-14	3.84E-14	4.69E-14	4.54E-14	4.65E-14	4.65E-14	4.73E-14	4.89E-14	4.96E-14	4.68E-14	4.08E-14
1.39E-15	2.61E-15	4.60E-15	6.83E-15	9.17E-15	1.10E-14	1.48E-14	1.96E-14	2.26E-14	2.63E-14	3.06E-14	3.65E-14	4.27E-14	4.28E-14	4.35E-14	4.42E-14	4.53E-14	4.68E-14	4.73E-14	4.60E-14	4.14E-14
8.47E-16	1.83E-15	3.43E-15	5.30E-15	7.44E-15	9.79E-15	1.36E-14	2.00E-14	2.16E-14	2.47E-14	2.91E-14	3.51E-14	4.17E-14	4.13E-14	3.99E-14	4.11E-14	4.22E-14	4.33E-14	4.40E-14	4.39E-14	4.14E-14
4.24E-16	1.16E-15	2.43E-15	3.85E-15	5.74E-15	8.15E-15	1.12E-14	1.89E-14	2.04E-14	2.31E-14	2.74E-14	3.35E-14	4.10E-14	3.86E-14	3.62E-14	3.72E-14	3.84E-14	3.93E-14	4.10E-14	4.16E-14	4.11E-14
1.40E-16	6.01E-16	1.54E-15	2.64E-15	4.06E-15	5.75E-15	7.92E-15	1.48E-14	1.78E-14	2.10E-14	2.53E-14	3.09E-14	3.62E-14	3.41E-14	3.14E-14	3.26E-14	3.43E-14	3.50E-14	3.79E-14	3.92E-14	3.98E-14
1.23E-17	2.01E-16	7.82E-16	1.55E-15	2.50E-15	3.38E-15	4.74E-15	1.04E-14	1.36E-14	1.65E-14	2.08E-14	2.49E-14	3.05E-14	2.91E-14	2.53E-14	2.79E-14	3.01E-14	3.05E-14	3.46E-14	3.66E-14	3.72E-14
1.23E-17	2.05E-17	2.44E-16	6.78E-16	1.26E-15	1.78E-15	2.56E-15	5.79E-15	9.89E-15	1.17E-14	1.54E-14	2.00E-14	2.46E-14	2.22E-14	1.91E-14	2.29E-14	2.66E-14	2.56E-14	3.11E-14	3.31E-14	3.41E-14
7.16E-17	2.08E-17	1.86E-17	1.59E-16	4.65E-16	7.79E-16	1.15E-15	3.01E-15	6.17E-15	7.64E-15	1.01E-14	1.26E-14	1.84E-14	1.53E-14	1.35E-14	1.88E-14	2.38E-14	2.05E-14	2.79E-14	2.92E-14	3.12E-14
1.22E-16	1.52E-16	2.52E-17	9.07E-19	6.68E-17	2.12E-16	3.39E-16	1.19E-15	3.29E-15	4.11E-15	5.83E-15	7.27E-15	8.77E-15	1.21E-14	9.11E-15	1.63E-14	2.20E-14	1.57E-14	2.49E-14	2.50E-14	2.83E-14
1.43E-16	2.75E-16	1.63E-16	9.86E-17	1.85E-17	3.11E-18	2.00E-17	3.51E-16	1.26E-15	1.90E-15	3.14E-15	4.52E-15	4.36E-15	9.54E-15	5.79E-15	1.39E-14	2.03E-14	1.10E-14	2.12E-14	2.08E-14	2.34E-14
1.52E-16	3.03E-16	3.28E-16	3.70E-16	2.46E-16	1.11E-16	8.39E-17	4.57E-17	3.46E-16	6.93E-16	1.40E-15	2.24E-15	2.37E-15	3.71E-15	3.47E-15	7.58E-15	1.40E-14	6.37E-15	1.17E-14	1.60E-14	1.23E-14
1.41E-16	2.94E-16	4.12E-16	6.38E-16	6.24E-16	4.68E-16	4.55E-16	2.29E-17	3.50E-17	6.00E-17	3.57E-16	7.52E-16	1.13E-15	2.80E-16	1.57E-15	1.67E-15	4.14E-15	2.56E-15	3.47E-15	7.69E-15	5.33E-15
1.25E-16	3.08E-16	4.10E-16	7.42E-16	9.52E-16	9.26E-16	1.03E-15	2.48E-16	1.00E-16	9.54E-17	1.24E-19	6.05E-17	1.54E-16	1.25E-17	2.34E-16	6.38E-17	2.85E-16	5.59E-16	7.62E-16	1.87E-15	1.68E-15
1.25E-16	3.31E-16	4.21E-16	7.49E-16	1.09E-15	1.22E-15	1.54E-15	6.11E-16	3.85E-16	3.62E-16	2.27E-16	1.12E-16	1.33E-16	4.05E-16	1.24E-16	1.63E-16	7.33E-17	3.90E-18	1.24E-17	1.64E-16	2.42E-16
1.42E-16	3.24E-16	4.41E-16	7.55E-16	1.11E-15	1.30E-15	1.74E-15	9.61E-16	1.02E-15	5.62E-16	6.92E-16	5.95E-16	6.69E-16	1.31E-15	9.72E-16	9.96E-16	7.61E-16	4.89E-16	3.01E-16	9.88E-17	4.54E-17
1.62E-16	3.33E-16	4.63E-16	7.37E-16	1.14E-15	1.33E-15	1.75E-15	2.53E-15	3.28E-15	2.48E-15	1.41E-15	1.26E-15	1.44E-15	2.56E-15	2.15E-15	2.23E-15	1.86E-15	1.53E-15	1.20E-15	7.21E-16	6.03E-16
1.69E-16	3.29E-16	4.79E-16	7.36E-16	1.16E-15	1.40E-15	1.83E-15	2.56E-15	3.23E-15	3.57E-15	3.80E-15	3.78E-15	2.25E-15	3.42E-15	3.20E-15	3.31E-15	2.93E-15	2.65E-15	2.28E-15	1.83E-15	1.48E-15
1.53E-16	3.16E-16	4.60E-16	7.66E-16	1.18E-15	1.45E-15	1.96E-15	2.52E-15	3.19E-15	3.45E-15	3.83E-15	4.29E-15	3.94E-15	3.69E-15	3.82E-15	3.89E-15	3.58E-15	3.42E-15	3.18E-15	2.54E-15	2.27E-15
1.34E-16	3.03E-16	4.24E-16	7.71E-16	1.14E-15	1.44E-15	1.96E-15	2.42E-15	3.13E-15	3.46E-15	3.82E-15	4.24E-15	4.00E-15	3.67E-15	3.86E-15	4.02E-15	3.69E-15	3.64E-15	3.56E-15	2.95E-15	2.58E-15
1.22E-16	2.84E-16	4.36E-16	7.43E-16	1.08E-15	1.42E-15	1.86E-15	2.26E-15	3.10E-15	3.47E-15	3.70E-15	4.29E-15	4.09E-15	3.60E-15	3.77E-15	4.08E-15	3.64E-15	3.66E-15	3.50E-15	3.01E-15	2.79E-15
1.13E-16	2.70E-16	4.47E-16	7.38E-16	1.09E-15	1.43E-15	1.81E-15	2.14E-15	3.07E-15	3.39E-15	3.59E-15	4.31E-15	4.00E-15	3.56E-15	3.77E-15	4.17E-15	3.64E-15	3.66E-15	3.44E-15	2.96E-15	2.82E-15
1.02E-16	2.49E-16	4.38E-16	7.91E-16	1.11E-15	1.39E-15	1.85E-15	2.23E-15	3.02E-15	3.27E-15	3.53E-15	4.14E-15	4.00E-15	3.81E-15	3.77E-15	4.18E-15	3.68E-15	3.53E-15	3.48E-15	3.18E-15	2.77E-15
9.07E-17	2.48E-16	4.28E-16	8.05E-16	1.07E-15	1.31E-15	1.82E-15	2.27E-15	3.05E-15	3.19E-15	3.60E-15	4.02E-15	4.10E-15	3.95E-15	3.72E-15	4.13E-15	3.65E-15	3.39E-15	3.47E-15	3.24E-15	2.55E-15
9.61E-17	2.47E-16	4.37E-16	7.68E-16	1.04E-15	1.28E-15	1.74E-15	2.27E-15	3.11E-15	3.32E-15	3.77E-15	4.18E-15	4.16E-15	4.01E-15	3.72E-15	4.15E-15	3.60E-15	3.37E-15	3.39E-15	3.21E-15	2.31E-15
8.32E-17	2.48E-16	4.82E-16	7.62E-16	1.10E-15	1.26E-15	1.71E-15	2.07E-15	3.07E-15	3.32E-15	3.85E-15	4.27E-15	4.18E-15	3.92E-15	3.71E-15	4.24E-15	3.62E-15	3.42E-15	3.41E-15	3.04E-15	2.21E-15
6.63E-17	2.39E-16	4.58E-16	7.65E-16	1.13E-15	1.23E-15	1.71E-15	1.86E-15	3.09E-15	3.27E-15	3.80E-15	4.05E-15	4.06E-15	3.95E-15	3.74E-15	4.19E-15	3.63E-15	3.32E-15	3.39E-15	2.81E-15	2.04E-15
7.33E-17	2.47E-16	3.87E-16	7.24E-16	1.08E-15	1.18E-15	1.64E-15	1.73E-15	2.93E-15	3.26E-15	3.71E-15	3.92E-15	3.98E-15	3.88E-15	3.73E-15	4.06E-15	3.61E-15	3.23E-15	3.40E-15	2.64E-15	2.36E-15
7.20E-17	2.29E-16	3.31E-16	6.51E-16	1.02E-15	1.16E-15	1.60E-15	2.22E-15	2.92E-15	3.35E-15	3.70E-15	3.96E-15	4.04E-15	3.81E-15	3.61E-15	3.92E-15	3.77E-15	3.35E-15	3.40E-15	2.53E-15	2.43E-15
6.62E-17	2.20E-16	3.55E-16	6.37E-16	1.01E-15	1.21E-15	1.62E-15	2.28E-15	2.84E-15	3.22E-15	3.87E-15	4.15E-15	4.02E-15	3.77E-15	3.58E-15	4.00E-15	3.89E-15	3.33E-15	3.36E-15	2.60E-15	2.52E-15
8.92E-17	2.18E-16	3.69E-16	6.26E-16	1.00E-15	1.25E-15	1.68E-15	2.30E-15	2.81E-15	3.13E-15	3.90E-15	4.20E-15	4.08E-15	3.80E-15	3.81E-15	3.99E-15	3.88E-15	3.18E-15	3.30E-15	2.99E-15	2.48E-15
8.41E-17	2.08E-16	4.26E-16	6.00E-16	9.37E-16	1.25E-15	1.70E-15	2.28E-15	2.73E-15	3.23E-15	3.77E-15	4.14E-15	4.12E-15	3.75E-15	3.82E-15	3.81E-15	3.86E-15	3.24E-15	3.35E-15	3.01E-15	2.39E-15
5.59E-17	1.93E-16	4.21E-16	5.49E-16	9.09E-16	1.29E-15	1.69E-15	2.18E-15	2.58E-15	3.35E-15	3.82E-15	4.07E-15	3.97E-15	3.91E-15	3.79E-15	3.66E-15	3.76E-15	3.18E-15	3.36E-15	3.00E-15	2.04E-15
4.90E-17	1.68E-16	4.03E-16	4.87E-16	9.48E-16	1.35E-15	1.62E-15	2.14E-15	2.36E-15	3.31E-15	3.90E-15	3.90E-15	3.95E-15	3.94E-15	3.69E-15	3.75E-15	3.64E-15	2.98E-15	3.32E-15	3.02E-15	1.57E-15
6.24E-17	1.56E-16	3.97E-16	4.81E-16	9.45E-16	1.33E-15	1.60E-15	1.94E-15	2.07E-15	3.22E-15	3.95E-15	3.67E-15	3.78E-15	3.86E-15	3.72E-15	3.77E-15	3.53E-15	2.96E-15	3.26E-15	2.98E-15	1.30E-15
7.13E-17	1.62E-16	3.88E-16	6.54E-16	1.02E-15	1.26E-15	1.62E-15	1.71E-15	2.15E-15	3.30E-15	3.81E-15	3.66E-15	3.73E-15	3.70E-15	3.69E-15	3.68E-15	3.48E-15	2.94E-15	3.23E-15	2.93E-15	1.24E-15
6.08E-17	1.77E-16	3.57E-16	7.27E-16	1.04E-15	1.21E-15	1.69E-15	1.76E-15	2.98E-15	3.31E-15	3.64E-15	3.98E-15	3.67E-15	3.65E-15	3.57E-15	3.61E-15	3.45E-15	2.90E-15	3.15E-15	2.74E-15	2.11E-15
4.40E-17	1.69E-16	3.38E-16	7.04E-16	9.79E-16	1.21E-15	1.67E-15	2.47E-15	3.03E-15	3.24E-15	3.55E-15	3.93E-15	3.50E-15	3.73E-15	3.36E-15	3.34E-15	3.64E-15	3.09E-15	3.08E-15	2.68E-15	2.74E-15
4.84E-17	1.69E-16	3.96E-16	6.81E-16	9.69E-16	1.18E-15	1.69E-15	2.41E-15	2.97E-15	3.22E-15	3.51E-15	3.79E-15	3.37E-15	3.72E-15	3.31E-15	3.36E-15	3.60E-15	3.13E-15	3.01E-15	2.88E-15	2.44E-15
6.39E-17	1.96E-16	4.72E-16	6.86E-16	1.05E-15	1.17E-15	1.73E-15	2.43E-15	2.87E-15	3.11E-15	3.45E-15	3.74E-15	2.98E-15	3.48E-15	3.50E-15	3.50E-15	3.58E-15	3.18E-15	2.87E-15	2.78E-15	2.28E-15
7.12E-17	2.10E-16	4.75E-16	7.14E-16	1.11E-15	1.18E-15	1.73E-15	2.43E-15	2.83E-15	2.97E-15	3.24E-15	3.64E-15	2.71E-15	3.28E-15	3.67E-15	3.47E-15	3.30E-15	3.14E-15	2.81E-15	2.70E-15	2.24E-15
8.62E-17	2.08E-16	4.63E-16	7.30E-16	1.11E-15	1.17E-15	1.62E-15	2.41E-15	2.72E-15	2.90E-15	3.05E-15	3.52E-15	2.71E-15	3.48E-15	3.58E-15	3.34E-15	3.09E-15	3.06E-15	2.95E-15	2.90E-15	2.33E-15
9.52E-17	2.06E-16	4.72E-16	7.38E-16	1.11E-15	1.18E-15	1.48E-15	2.34E-15	2.41E-15	2.61E-15	2.78E-15	3.19E-15	3.37E-15	3.26E-15	3.25E-15	3.16E-15	3.16E-15	2.95E-15	2.92E-15	2.90E-15	2.30E-15
1.00E-16	2.30E-16	4.74E-16	7.39E-16	1.13E-15	1.18E-15	1.36E-15	2.12E-15	1.90E-15	2.01E-15	2.19E-15	2.43E-15	2.77E-15	2.72E-15	2.70E-15	2.72E-15	2.97E-15	2.64E-15	2.69E-15	2.77E-15	2.10E-15
1.07E-16	2.51E-16	4.87E-16	7.34E-16	1.11E-15	1.09E-15	1.12E-15	1.76E-15	1.21E-15	1.23E-15	1.29E-15	1.43E-15	1.81E-15	1.81E-15	1.88E-15	1.94E-15	2.20E-15	2.11E-15	2.22E-15	2.39E-15	1.99E-15
1.21E-16	2.58E-16	4.78E-16	6.59E-16	9.64E-16	8.06E-16	7.58E-16	1.09E-15	4.43E-16	3.72E-16	3.83E-16	3.98E-16	6.42E-16	6.97E-16	8.32E-16	9.54E-16	1.19E-15	1.30E-15	1.50E-15	1.88E-15	1.78E-15
1.12E-16	2.27E-16	3.90E-16	4.99E-16	6.51E-16	4.12E-16	2.73E-16	3.16E-16	1.48E-18	1.11E-17	2.14E-17	4.29E-17	1.25E-18	1.73E-18	2.55E-17	9.53E-17	2.36E-16	3.70E-16	6.32E-16	1.07E-15	1.27E-15
9.86E-17	1.60E-16	2.41E-16	2.73E-16	2.30E-16	4.05E-17	6.99E-18	4.72E-17	8.03E-16	1.25E-15	1.41E-15	1.52E-15	1.12E-15	9.69E-16	7.15E-16	4.16E-16	1.40E-16	3.06E-17	1.52E-17	2.36E-16	5.19E-16
7.85E-17	7.54E-17	7.28E-17	3.28E-17	4.75E-18	2.76E-16	9.59E-16	1.57E-15	3.89E-15	4.88E-15	5.18E-15	5.23E-15	4.37E-15	4.11E-15	3.47E-15	2.74E-15	1.79E-15	1.16E-15	5.12E-16	6.95E-17	1.22E-17
3.86E-17	5.41E-18	9.98E-18	1.66E-16	8.29E-16	2.30E-15	4.12E-15	5.36E-15	9.33E-15	1.10E-14	1.15E-14	1.11E-14	9.77E-15	9.19E-15	8.03E-15	6.70E-15	5.03E-15	3.65E-15	2.30E-15	1.10E-15	3.46E-16
7.41E-19	7.62E-17	4.79E-16	1.60E-15	3.70E-15	6.41E-15	9.21E-15	1.11E-14	1.67E-14	1.93E-14	2.03E-14	1.92E-14	1.70E-14	1.57E-14	1.41E-14	1.18E-14	9.19E-15	7.17E-15	5.01E-15	2.98E-15	1.54E-15
6.34E-17	6.52E-16	2.27E-15	4.92E-15	8.40E-15	1.20E-14	1.56E-14	1.80E-14	2.56E-14	2.96E-14	3.11E-14	2.89E-14	2.54E-14	2.33E-14	2.11E-14	1.75E-14	1.38E-14	1.12E-14	8.18E-15	5.21E-15	3.24E-15
4.54E-16	2.25E-15	5.66E-15	9.68E-15	1.45E-14	1.86E-14	2.27E-14	2.55E-14	3.53E-14	4.10E-14	4.29E-14	3.89E-14	3.43E-14	3.11E-14	2.80E-14	2.32E-14	1.85E-14	1.51E-14	1.13E-14	7.44E-15	5.10E-15
1.57E-15	4.86E-15	9.95E-15	1.53E-14	2.11E-14	2.56E-14	2.96E-14	3.26E-14	4.40E-14	5.06E-14	5.24E-14	4.71E-14	4.25E-14	3.81E-14	3.37E-14	2.83E-14	2.30E-14	1.86E-14	1.43E-14	9.58E-15	6.93E-15
3.34E-15	7.84E-15	1.43E-14	2.08E-14	2.77E-14	3.23E-14	3.59E-14	3.87E-14	4.91E-14	5.59E-14	5.61E-14	5.15E-14	4.78E-14	4.24E-14	3.81E-14	3.21E-14	2.65E-14	2.17E-14	1.68E-14	1.13E-14	8.41E-15
4.98E-15	1.06E-14	1.78E-14	2.54E-14	3.30E-14	3.74E-14	4.09E-14	4.43E-14	5.16E-14	5.80E-14	5.61E-14	5.27E-14	4.99E-14	4.41E-14	4.03E-14	3.43E-14	2.86E-14	2.37E-14	1.84E-14	1.27E-14	9.44E-15
6.06E-15	1.23E-14	2.01E-14	2.79E-14	3.61E-14	4.01E-14	4.40E-14	4.75E-14	5.21E-14	5.60E-14	5.53E-14	5.29E-14	5.09E-14	4.48E-14	4.09E-14	3.50E-14	2.96E-14	2.43E-14	1.91E-14	1.34E-14	1.00E-14
6.67E-15	1.30E-14	2.06E-14	2.81E-14	3.59E-14	4.01E-14	4.51E-14	4.72E-14	5.11E-14	5.25E-14	5.24E-14	5.20E-14	5.07E-14	4.48E-14	4.05E-14	3.47E-14	2.94E-14	2.40E-14	1.91E-14	1.36E-14	1.04E-14
6.73E-15	1.27E-14	1.97E-14	2.65E-14	3.41E-14	3.85E-14	4.40E-14	4.53E-14	4.87E-14	4.88E-14	4.87E-14	4.98E-14	4.90E-14	4.34E-14	3.87E-14	3.29E-14	2.77E-14	2.31E-14	1.84E-14	1.31E-14	1.01E-14

6.09E-15	1.15E-14	1.80E-14	2.43E-14	3.18E-14	3.62E-14	4.15E-14	4.33E-14	4.59E-14	4.55E-14	4.49E-14	4.65E-14	4.56E-14	3.96E-14	3.49E-14	2.96E-14	2.51E-14	2.11E-14	1.67E-14	1.20E-14	9.08E-15
4.94E-15	9.66E-15	1.56E-14	2.13E-14	2.84E-14	3.25E-14	3.77E-14	4.05E-14	4.24E-14	4.14E-14	4.05E-14	4.20E-14	3.97E-14	3.33E-14	3.00E-14	2.55E-14	2.17E-14	1.80E-14	1.42E-14	1.02E-14	7.43E-15
3.57E-15	7.41E-15	1.26E-14	1.77E-14	2.39E-14	2.73E-14	3.24E-14	3.62E-14	3.90E-14	3.76E-14	3.56E-14	3.62E-14	3.26E-14	2.71E-14	2.49E-14	2.12E-14	1.78E-14	1.45E-14	1.13E-14	7.91E-15	5.52E-15
2.25E-15	5.00E-15	9.03E-15	1.32E-14	1.85E-14	2.10E-14	2.58E-14	3.03E-14	3.53E-14	3.30E-14	3.03E-14	3.02E-14	2.50E-14	2.15E-14	1.95E-14	1.66E-14	1.36E-14	1.11E-14	8.30E-15	5.37E-15	3.66E-15
1.09E-15	2.82E-15	5.61E-15	8.63E-15	1.28E-14	1.49E-14	1.88E-14	2.44E-14	2.97E-14	2.76E-14	2.49E-14	2.54E-14	1.94E-14	1.60E-14	1.41E-14	1.19E-14	9.59E-15	7.73E-15	5.45E-15	3.31E-15	2.05E-15
3.38E-16	1.19E-15	2.85E-15	4.79E-15	7.79E-15	9.51E-15	1.24E-14	1.67E-14	1.95E-14	2.19E-14	1.99E-14	2.09E-14	1.40E-14	1.12E-14	9.41E-15	7.79E-15	6.07E-15	4.55E-15	2.97E-15	1.67E-15	8.22E-16
3.42E-17	2.58E-16	9.95E-16	2.12E-15	3.87E-15	5.18E-15	7.25E-15	1.08E-14	1.34E-14	1.64E-14	1.52E-14	1.63E-14	1.00E-14	7.41E-15	5.49E-15	4.34E-15	3.09E-15	2.05E-15	1.14E-15	5.72E-16	1.61E-16
5.93E-18	3.48E-19	1.38E-16	5.70E-16	1.37E-15	2.09E-15	3.41E-15	6.13E-15	8.04E-15	1.11E-14	8.11E-15	1.15E-14	6.72E-15	4.54E-15	2.55E-15	1.79E-15	1.07E-15	5.38E-16	1.49E-16	4.78E-17	2.53E-19
2.37E-17	7.47E-17	1.78E-17	1.23E-17	2.02E-16	4.39E-16	8.96E-16	2.84E-15	2.97E-15	4.03E-15	3.69E-15	6.73E-15	3.75E-15	2.37E-15	7.15E-16	3.09E-16	1.02E-16	1.75E-18	7.05E-17	3.32E-17	5.74E-17
3.36E-17	1.26E-16	1.66E-16	1.40E-16	1.91E-17	8.82E-23	3.06E-17	6.63E-16	3.13E-16	3.39E-16	1.01E-15	2.54E-15	1.07E-15	7.45E-16	1.60E-17	3.25E-17	9.74E-17	3.71E-16	6.78E-16	1.84E-16	2.41E-16
3.04E-17	1.30E-16	2.33E-16	3.37E-16	2.86E-16	2.36E-16	1.58E-16	1.17E-19	1.45E-16	1.07E-16	1.93E-17	1.57E-16	5.10E-21	1.71E-17	2.60E-16	5.64E-16	6.45E-16	1.22E-15	1.41E-15	4.84E-16	1.60E-15
2.29E-17	1.11E-16	2.59E-16	3.59E-16	4.95E-16	5.60E-16	5.89E-16	2.73E-16	9.20E-16	9.29E-16	1.17E-15	3.33E-16	5.99E-16	6.21E-16	1.25E-15	1.39E-15	1.47E-15	1.76E-15	1.76E-15	1.67E-15	1.70E-15
1.43E-17	8.37E-17	2.38E-16	3.56E-16	5.25E-16	6.64E-16	8.35E-16	1.13E-15	1.36E-15	1.59E-15	1.97E-15	1.53E-15	1.73E-15	1.87E-15	2.02E-15	2.05E-15	1.94E-15	1.88E-15	1.80E-15	1.57E-15	1.51E-15
1.31E-17	5.67E-17	2.18E-16	3.66E-16	4.96E-16	6.46E-16	9.03E-16	1.15E-15	1.42E-15	1.66E-15	2.03E-15	2.07E-15	2.04E-15	1.94E-15	1.98E-15	2.02E-15	1.92E-15	1.91E-15	1.81E-15	1.59E-15	1.47E-15
2.19E-17	5.18E-17	1.79E-16	3.66E-16	4.70E-16	5.96E-16	8.58E-16	1.07E-15	1.42E-15	1.59E-15	1.97E-15	2.04E-15	2.10E-15	2.00E-15	1.91E-15	1.96E-15	1.87E-15	1.89E-15	1.79E-15	1.68E-15	1.38E-15
1.58E-17	5.49E-17	1.42E-16	3.10E-16	4.49E-16	5.57E-16	7.96E-16	9.38E-16	1.41E-15	1.53E-15	1.86E-15	1.96E-15	2.07E-15	1.98E-15	1.84E-15	1.94E-15	1.78E-15	1.79E-15	1.81E-15	1.60E-15	1.42E-15
6.88E-18	6.43E-17	1.25E-16	2.57E-16	4.21E-16	5.28E-16	7.54E-16	9.24E-16	1.31E-15	1.55E-15	1.81E-15	1.92E-15	2.04E-15	1.88E-15	1.81E-15	1.91E-15	1.66E-15	1.64E-15	1.83E-15	1.52E-15	1.45E-15
8.32E-14	1.64E-13	2.78E-13	4.06E-13	5.60E-13	6.77E-13	8.29E-13	9.86E-13	1.21E-12	1.36E-12	1.42E-12	1.54E-12	1.57E-12	1.49E-12	1.42E-12	1.38E-12	1.35E-12	1.24E-12	1.19E-12	1.08E-12	9.47E-13

-5.42E-08	-4.66E-08	-4.05E-08	-4.44E-08	-3.33E-08	-2.50E-08	-2.23E-08	-5.41E-09	6.62E-09	1.07E-09	1.43E-08	1.66E-08	2.37E-08	1.71E-08	1.99E-08	1.63E-08	2.01E-08	1.99E-08	1.98E-08	2.28E-08	2.16E-08	1.88E-08
-5.54E-08	-4.72E-08	-3.77E-08	-4.31E-08	-3.19E-08	-1.89E-08	-2.14E-08	-5.37E-09	-3.37E-09	1.71E-09	1.07E-08	1.66E-08	2.41E-08	1.79E-08	2.16E-08	1.74E-08	1.82E-08	1.99E-08	2.05E-08	2.26E-08	2.13E-08	1.88E-08
-5.52E-08	-4.90E-08	-4.63E-08	-4.16E-08	-3.14E-08	-1.53E-08	-2.13E-08	-3.58E-09	-2.13E-09	-4.80E-09	8.13E-09	1.81E-08	1.91E-08	2.01E-08	2.39E-08	1.82E-08	1.95E-08	1.96E-08	2.05E-08	2.12E-08	2.06E-08	1.83E-08
-5.40E-08	-4.87E-08	-4.89E-08	-4.17E-08	-3.20E-08	-2.99E-08	-2.13E-08	-1.54E-09	-1.22E-08	-2.55E-09	1.10E-08	1.35E-08	1.46E-08	2.26E-08	2.62E-08	1.72E-08	2.06E-08	1.91E-08	1.93E-08	2.06E-08	1.95E-08	1.73E-08
-5.38E-08	-4.82E-08	-4.87E-08	-4.11E-08	-3.41E-08	-3.21E-08	-1.78E-08	-1.61E-08	-1.14E-08	-1.24E-09	6.32E-09	1.55E-08	1.25E-08	2.49E-08	2.31E-08	1.59E-08	2.11E-08	1.96E-08	1.87E-08	2.07E-08	1.92E-08	1.67E-08
-5.40E-08	-4.90E-08	-4.95E-08	-4.04E-08	-3.46E-08	-3.09E-08	-1.48E-08	-1.36E-08	-9.67E-09	-3.42E-09	4.08E-09	1.65E-08	1.39E-08	2.63E-08	1.76E-08	1.66E-08	2.31E-08	1.89E-08	1.88E-08	2.04E-08	2.00E-08	1.77E-08
-5.31E-08	-5.00E-08	-4.97E-08	-3.95E-08	-3.54E-08	-2.85E-08	-1.68E-08	-9.95E-09	-6.87E-09	-5.67E-09	3.49E-09	1.32E-08	1.56E-08	1.58E-08	1.62E-08	1.69E-08	2.04E-08	1.80E-08	1.81E-08	1.89E-08	1.96E-08	1.90E-08
-4.89E-08	-4.99E-08	-4.88E-08	-3.90E-08	-3.53E-08	-2.44E-08	-2.50E-08	-4.85E-09	-2.89E-09	-3.58E-09	6.17E-09	1.04E-08	2.03E-08	1.52E-08	1.60E-08	1.50E-08	1.95E-08	1.83E-08	1.78E-08	1.81E-08	1.91E-08	2.02E-08
-4.42E-08	-4.92E-08	-4.65E-08	-3.88E-08	-3.44E-08	-1.79E-08	-2.49E-08	-3.47E-09	-5.99E-09	-1.60E-09	9.74E-09	1.42E-08	2.21E-08	1.54E-08	1.53E-08	1.54E-08	1.67E-08	1.83E-08	1.73E-08	1.84E-08	1.92E-08	2.07E-08
-4.21E-08	-4.95E-08	-4.30E-08	-3.49E-08	-3.41E-08	-1.04E-08	-2.48E-08	-1.42E-08	-8.56E-09	2.78E-09	1.25E-08	1.85E-08	2.18E-08	1.43E-08	1.52E-08	1.68E-08	1.67E-08	1.78E-08	1.64E-08	1.85E-08	2.01E-08	1.92E-08
-5.32E-08	-4.99E-08	-3.86E-08	-2.90E-08	-3.46E-08	-1.42E-08	-2.27E-08	-1.32E-08	-6.77E-09	8.33E-09	1.35E-08	2.05E-08	1.77E-08	1.42E-08	1.55E-08	1.77E-08	1.67E-08	1.77E-08	1.73E-08	1.79E-08	2.03E-08	1.84E-08
-5.47E-08	-5.01E-08	-3.42E-08	-2.48E-08	-3.49E-08	-2.13E-08	-2.07E-08	-1.22E-08	-3.26E-09	5.87E-09	5.67E-09	2.00E-08	1.67E-08	1.38E-08	1.51E-08	1.74E-08	1.66E-08	1.80E-08	1.77E-08	1.71E-08	1.95E-08	1.80E-08
-5.47E-08	-4.99E-08	-3.46E-08	-4.15E-08	-3.57E-08	-2.15E-08	-1.70E-08	-9.91E-09	-5.63E-11	-4.72E-09	4.49E-09	1.49E-08	1.32E-08	1.37E-08	1.56E-08	1.77E-08	1.61E-08	1.80E-08	1.87E-08	1.73E-08	1.90E-08	1.77E-08
-5.40E-08	-4.98E-08	-4.83E-08	-4.41E-08	-3.57E-08	-3.26E-08	-1.24E-08	-1.80E-08	1.38E-09	-4.54E-09	2.35E-09	8.49E-09	1.61E-08	1.35E-08	1.62E-08	1.64E-08	1.62E-08	1.90E-08	1.95E-08	1.75E-08	1.93E-08	1.79E-08
-5.42E-08	-5.00E-08	-4.78E-08	-4.39E-08	-3.63E-08	-3.14E-08	-1.23E-08	-1.66E-08	-1.26E-08	-2.46E-09	5.61E-09	8.39E-09	1.59E-08	1.47E-08	1.56E-08	1.59E-08	1.58E-08	1.93E-08	1.87E-08	1.69E-08	1.92E-08	1.79E-08
-5.49E-08	-5.02E-08	-4.67E-08	-4.37E-08	-3.57E-08	-2.77E-08	-2.34E-08	-1.74E-08	-1.05E-08	-2.50E-10	7.27E-09	9.54E-09	1.43E-08	1.56E-08	1.55E-08	1.64E-08	1.58E-08	1.84E-08	1.68E-08	1.65E-08	1.92E-08	1.79E-08
-5.69E-08	-5.12E-08	-4.89E-08	-4.58E-08	-3.50E-08	-2.43E-08	-2.08E-08	-1.65E-08	-7.67E-09	6.10E-09	-2.23E-09	1.02E-08	1.04E-08	1.53E-08	1.52E-08	1.79E-08	1.61E-08	1.63E-08	1.60E-08	1.65E-08	1.80E-08	1.80E-08
-5.81E-08	-5.13E-08	-4.91E-08	-4.62E-08	-3.56E-08	-2.12E-08	-1.65E-08	-1.38E-08	-4.67E-09	1.31E-08	-3.14E-09	1.16E-08	1.16E-08	1.49E-08	1.40E-08	1.69E-08	1.54E-08	1.48E-08	1.61E-08	1.60E-08	1.63E-08	1.79E-08
-5.76E-08	-5.08E-08	-4.99E-08	-4.52E-08	-3.69E-08	-2.41E-08	-1.36E-08	-1.07E-08	-6.22E-09	1.31E-08	-2.03E-09	1.12E-08	1.38E-08	1.36E-08	1.32E-08	1.72E-08	1.55E-08	1.42E-08	1.60E-08	1.46E-08	1.47E-08	1.76E-08
-5.69E-08	-5.00E-08	-4.90E-08	-4.32E-08	-3.69E-08	-2.44E-08	-2.36E-08	-1.35E-08	-6.14E-09	-5.33E-09	-6.23E-10	9.87E-09	1.18E-08	1.27E-08	1.17E-08	1.89E-08	1.57E-08	1.37E-08	1.58E-08	1.42E-08	1.33E-08	1.67E-08
-5.68E-08	-4.95E-08	-4.96E-08	-4.27E-08	-3.64E-08	-1.81E-08	-2.25E-08	-9.04E-09	-1.45E-08	-5.16E-09	3.58E-09	9.08E-09	1.26E-08	1.38E-08	1.09E-08	1.86E-08	1.44E-08	1.27E-08	1.45E-08	1.39E-08	1.36E-08	1.64E-08
-5.62E-08	-4.97E-08	-4.93E-08	-4.40E-08	-3.67E-08	-1.34E-08	-2.02E-08	-5.29E-09	-1.40E-08	-3.80E-09	6.76E-09	2.86E-09	1.10E-08	1.24E-08	1.05E-08	1.38E-08	1.42E-08	1.09E-08	1.34E-08	1.33E-08	1.42E-08	1.64E-08
-5.59E-08	-5.16E-08	-4.91E-08	-4.49E-08	-3.79E-08	-2.56E-08	-1.57E-08	-9.75E-09	-1.16E-08	-1.24E-09	1.21E-08	3.76E-09	1.11E-08	1.09E-08	1.06E-08	1.12E-08	1.31E-08	1.01E-08	1.32E-08	1.28E-08	1.40E-08	1.60E-08
-5.58E-08	-5.19E-08	-4.95E-08	-4.58E-08	-3.85E-08	-2.84E-08	-9.47E-09	-7.16E-09	-7.86E-09	-3.27E-09	1.35E-08	6.79E-09	9.54E-09	1.23E-08	1.15E-08	1.16E-08	1.17E-08	9.90E-09	1.35E-08	1.27E-08	1.34E-08	1.52E-08
-5.61E-08	-5.20E-08	-5.14E-08	-4.52E-08	-3.79E-08	-2.48E-08	-5.66E-11	-1.13E-08	-4.20E-09	-1.37E-09	1.31E-08	6.42E-09	8.12E-09	1.27E-08	1.18E-08	1.04E-08	1.21E-08	1.07E-08	1.42E-08	1.28E-08	1.22E-08	1.43E-08
-5.68E-08	-5.25E-08	-5.07E-08	-4.35E-08	-3.77E-08	-1.91E-08	-3.98E-09	-8.66E-09	-6.64E-09	3.22E-09	1.33E-08	7.68E-09	5.89E-09	1.23E-08	1.24E-08	8.88E-09	1.28E-08	1.13E-08	1.31E-08	1.16E-08	1.26E-08	1.41E-08
-5.69E-08	-5.33E-08	-4.97E-08	-4.16E-08	-3.75E-08	-1.41E-08	-2.68E-08	-4.59E-09	-9.12E-09	7.59E-09	9.06E-09	5.32E-09	5.08E-09	1.21E-08	1.15E-08	7.62E-09	1.42E-08	1.11E-08	1.29E-08	1.09E-08	1.23E-08	1.21E-08
-5.68E-08	-5.27E-08	-4.86E-08	-4.04E-08	-3.72E-08	-2.57E-08	-2.67E-08	-1.80E-08	-4.12E-09	-1.93E-09	1.83E-08	6.26E-09	7.23E-09	1.24E-08	9.18E-09	7.31E-09	1.11E-08	1.09E-08	1.33E-08	1.12E-08	1.23E-08	1.11E-08
-5.67E-08	-5.14E-08	-4.92E-08	-4.08E-08	-3.53E-08	-3.10E-08	-2.62E-08	-2.08E-08	3.56E-09	5.73E-10	1.49E-08	5.42E-09	1.13E-08	7.93E-09	7.23E-09	7.85E-09	1.03E-08	1.11E-08	1.41E-08	1.18E-08	1.12E-08	9.66E-09
-5.71E-08	-5.11E-08	-4.86E-08	-4.20E-08	-3.41E-08	-2.71E-08	-2.50E-08	-1.96E-08	2.14E-09	6.02E-09	1.00E-08	7.71E-09	9.91E-09	7.18E-09	5.80E-09	9.56E-09	1.12E-08	1.10E-08	1.65E-08	1.25E-08	9.47E-09	8.67E-09
-5.85E-08	-5.18E-08	-4.61E-08	-3.94E-08	-3.43E-08	-2.45E-08	-2.11E-08	-1.88E-08	4.42E-10	8.98E-09	4.71E-09	2.51E-09	9.77E-09	7.44E-09	6.51E-09	8.63E-09	1.09E-08	1.01E-08	1.77E-08	1.28E-08	9.30E-09	8.34E-09
-5.88E-08	-5.30E-08	-4.17E-08	-3.64E-08	-3.57E-08	-3.37E-08	-1.32E-08	-1.86E-08	1.03E-09	2.97E-10	5.35E-09	5.85E-09	9.70E-09	7.32E-09	7.60E-09	8.28E-09	1.05E-08	1.02E-08	1.46E-08	1.08E-08	8.81E-09	7.75E-09
-5.82E-08	-5.27E-08	-3.82E-08	-3.47E-08	-3.69E-08	-3.38E-08	-5.69E-09	-1.68E-08	-5.48E-09	3.57E-09	-1.41E-09	1.04E-09	5.41E-09	7.60E-09	8.12E-09	8.61E-09	1.08E-08	1.07E-08	9.17E-09	8.80E-09	8.54E-09	7.16E-09
-5.44E-08	-5.04E-08	-3.93E-08	-4.09E-08	-3.64E-08	-3.39E-08	-1.24E-08	-1.44E-08	2.11E-09	2.83E-09	-2.55E-09	5.62E-09	3.43E-09	7.68E-09	8.19E-09	8.69E-09	1.15E-08	8.86E-09	8.55E-09	8.03E-09	8.55E-09	6.68E-09
-4.78E-08	-4.56E-08	-4.57E-08	-3.91E-08	-3.36E-08	-3.35E-08	-2.83E-08	-1.05E-08	2.82E-09	-5.91E-10	-1.41E-10	7.04E-09	4.64E-09	7.37E-09	8.62E-09	7.94E-09	9.88E-09	8.31E-09	8.91E-09	7.12E-09	8.76E-09	6.24E-09
-3.74E-08	-3.88E-08	-3.93E-08	-3.44E-08	-3.00E-08	-3.28E-08	-2.68E-08	-5.94E-09	1.34E-09	4.66E-09	2.26E-09	2.34E-09	4.39E-09	7.51E-09	8.18E-09	9.60E-09	8.62E-09	9.65E-09	9.40E-09	7.41E-09	9.29E-09	6.49E-09
-2.32E-08	-2.77E-08	-2.85E-08	-2.68E-08	-2.45E-08	-2.87E-08	-2.30E-08	-8.53E-09	-4.77E-09	6.63E-09	2.25E-09	2.82E-09	7.23E-09	7.13E-09	7.57E-09	1.16E-08	8.28E-09	1.07E-08	9.89E-09	7.26E-09	8.54E-09	7.09E-09
-1.07E-08	-1.15E-08	-1.24E-08	-1.56E-08	-1.51E-08	-2.13E-08	-2.05E-08	-7.15E-09	-5.49E-10	8.28E-09	3.24E-09	4.19E-09	3.67E-09	6.51E-09	6.87E-09	1.38E-08	8.08E-09	9.91E-09	9.06E-09	6.95E-09	8.45E-09	7.76E-09
7.58E-09	6.59E-09	2.72E-09	-6.88E-09	-4.38E-09	-1.01E-08	-1.67E-08	-9.88E-09	6.10E-09	9.84E-10	6.89E-09	4.78E-09	5.38E-09	6.79E-09	7.07E-09	1.46E-08	7.94E-09	9.36E-09	8.66E-09	6.92E-09	9.16E-09	8.43E-09
2.84E-08	2.45E-08	1.94E-08	2.75E-09	-3.56E-09	-2.85E-09	-7.06E-09	-9.14E-09	3.81E-09	-1.03E-08	2.95E-09	6.46E-09	6.19E-09	7.01E-09	7.59E-09	9.43E-09	6.93E-09	1.01E-08	9.16E-09	7.13E-09	9.33E-09	8.39E-09
4.63E-08	4.19E-08	3.59E-08	1.78E-08	5.81E-09	-1.06E-08	1.82E-09	-2.06E-09	-2.67E-09	-7.06E-09	5.77E-09	5.70E-09	8.37E-09	7.10E-09	7.41E-09	7.68E-09	5.42E-09	1.06E-08	8.96E-09	7.80E-09	8.79E-09	7.40E-09
5.97E-08	5.87E-08	5.15E-08	3.25E-08	2.05E-08	3.43E-09	-6.18E-09	5.68E-09	-2.32E-09	-3.87E-09	-8.17E-10	-4.47E-10	1.09E-08	7.39E-09	8.33E-09	9.73E-09	5.61E-09	9.10E-09	8.36E-09	8.26E-09	8.68E-09	7.02E-09
7.50E-08	7.39E-08	6.86E-08	4.60E-08	3.41E-08	1.70E-08	4.49E-09	-4.04E-09	5.91E-09	3.73E-09	-1.49E-09	3.09E-09	5.18E-09	8.66E-09	1.03E-08	1.14E-08	5.44E-09	8.17E-09	7.56E-09	8.27E-09	8.30E-09	6.64E-09
9.10E-08	8.77E-08	8.54E-08	5.91E-08	4.68E-08	3.06E-08	1.62E-08	5.74E-09	1.76E-09	1.40E-08	1.93E-09	4.36E-09	6.65E-09	1.12E-08	1.31E-08	1.23E-08	5.43E-09	8.01E-09	8.02E-09	8.17E-09	7.87E-09	5.63E-09
1.07E-07	1.01E-07	9.93E-08	7.19E-08	5.99E-08	4.36E-08	2.84E-08	1.73E-08	6.98E-09	1.08E-08	9.55E-09	2.27E-09	9.70E-09	1.13E-08	1.22E-08	8.76E-09	4.79E-09	8.24E-09	8.02E-09	8.22E-09	7.44E-09	5.21E-09
1.21E-07	1.15E-07	1.12E-07	8.44E-08	7.28E-08	5.40E-08	4.07E-08	2.86E-08	1.86E-08	9.00E-09	3.10E-09	-1.80E-10	6.99E-09	7.10E-09	6.99E-09	4.64E-09	4.16E-09	8.27E-09	8.13E-09	9.64E-09	7.18E-09	4.73E-09
1.35E-07	1.27E-07	1.24E-07	9.60E-08	8.20E-08	6.34E-08	5.21E-08	3.95E-08	2.86E-08	1.83E-08	8.37E-11	3.34E-09	5.18E-09	3.42E-09	4.05E-09	2.48E-09	3.27E-09	7.79E-09	9.36E-09	1.13E-08	6.93E-09	4.23E-09
1.47E-07	1.38E-07	1.36E-07	1.08E-07	9.37E-08	7.42E-08	6.22E-08	4.91E-08	3.68E-08	2.55E-08	7.73E-09	-2.95E-09	1.22E-08	4.16E-09	4.43E-09	1.47E-09	2.94E-09	7.27E-09	9.65E-09	1.03E-08	6.19E-09	3.49E-09
1.57E-07	1.49E-07	1.45E-07	1.20E-07	1.05E-07	8.46E-08	7.28E-08	5.81E-08	4.49E-08	3.20E-08	1.45E-08	1.39E-09	1.55E-08	5.94E-09	5.52E-09	3.02E-09	2.45E-09	7.28E-09	6.08E-09	5.34E-09	4.22E-09	2.61E-09
1.65E-07	1.59E-07	1.54E-07	1.31E-07	1.16E-07	9.57E-08	8.37E-08	6.79E-08	5.29E-08	3.89E-08	1.98E-08	7.16E-09	1.27E-08	7.42E-09	4.58E-09	4.97E-09	2.73E-09	6.28E-09	4.53E-09	3.20E-09	3.05E-09	1.56E-09
1.73E-07	1.68E-07	1.61E-07	1.43E-07	1.28E-07	1.08E-07	9.46E-08	7.77E-08	6.11E-08	4.60E-08	2.48E-08	1.22E-08	1.07E-09	7.60E-09	4.07E-09	4.83E-09	3.75E-09	5.18E-09	4.11E-09	2.90E-09	2.53E-09	1.24E-09
1.81E-07	1.74E-07	1.65E-07	1.55E-07	1.41E-07	1.21E-07	1.06E-07	8.81E-08	6.94E-08	5.23E-08	3.07E-08	1.65E-08	5.24E-10	6.86E-09	3.13E-09	4.79E-09	4.65E-09	5.66E-09	4.20E-09	2.65E-09	2.39E-09	1.10E-09
1.87E-07	1.79E-07	1.71E-07	1.68E-07	1.53E-07	1.34E-07	1.18E-07	9.84E-08	7.77E-08	5.83E-08	3.73E-08	2.03E-08	2.56E-09	9.31E-09	3.03E-09	4.73E-09	2.93E-09	4.49E-09	4.04E-09	1.65E-09	1.98E-09	5.68E-10
1.91E-07	1.81E-07	1.76E-07	1.77E-07	1.64E-07	1.46E-07	1.31E-07	1.09E-07	8.51E-08	6.41E-08	4.17E-08	2.41E-08	5.74E-09	1.09E-08	2.90E-09	1.45E-09	1.11E-09	2.80E-09	3.98E-09	1.05E-09	9.97E-10	-2.35E-10
1.92E-07	1.81E-07	1.81E-07	1.82E-07	1.72E-07	1.57E-07	1.43E-07	1.19E-07	9.08E-08	6.88E-08	4.44E-08	2.72E-08	8.88E-09	1.10E-08	2.45E-09	2.14E-09	9.96E-10	1.73E-09	3.42E-09	1.30E-09	4.90E-10	-8.67E-10

1.93E-07	1.78E-07	1.83E-07	1.82E-07	1.77E-07	1.63E-07	1.51E-07	1.25E-07	9.40E-08	7.17E-08	4.60E-08	2.85E-08	1.02E-08	9.40E-09	2.92E-09	2.41E-09	2.00E-09	1.66E-09	2.06E-09	1.04E-09	6.62E-10	-9.69E-10
1.93E-07	1.73E-07	1.82E-07	1.79E-07	1.78E-07	1.64E-07	1.53E-07	1.28E-07	9.41E-08	7.24E-08	4.60E-08	2.75E-08	8.68E-09	9.49E-09	2.38E-09	3.53E-09	2.32E-09	1.85E-09	9.53E-10	-1.47E-10	6.02E-10	-9.89E-10
1.92E-07	1.71E-07	1.78E-07	1.77E-07	1.80E-07	1.66E-07	1.57E-07	1.28E-07	9.10E-08	7.05E-08	4.43E-08	2.60E-08	6.37E-09	7.65E-09	5.57E-10	5.95E-09	1.02E-09	7.78E-10	5.22E-10	-1.27E-09	-5.90E-11	-1.73E-09
1.85E-07	1.72E-07	1.76E-07	1.76E-07	1.83E-07	1.72E-07	1.58E-07	1.25E-07	8.71E-08	6.68E-08	4.13E-08	2.36E-08	4.22E-09	6.11E-09	-1.30E-09	4.11E-09	-9.90E-10	-7.30E-11	9.86E-11	-1.68E-09	-8.67E-10	-2.60E-09
1.77E-07	1.71E-07	1.78E-07	1.76E-07	1.72E-07	1.75E-07	1.55E-07	1.20E-07	8.10E-08	6.12E-08	3.80E-08	1.88E-08	1.87E-09	5.27E-09	-1.69E-09	-6.54E-10	-9.72E-10	-3.76E-10	-7.94E-10	-2.20E-09	-2.03E-09	-2.80E-09
1.70E-07	1.63E-07	1.80E-07	1.76E-07	1.69E-07	1.74E-07	1.40E-07	1.11E-07	7.04E-08	5.22E-08	3.42E-08	1.16E-08	-5.56E-10	3.15E-09	-1.34E-09	1.34E-11	5.59E-10	5.25E-12	-1.53E-09	-2.70E-09	-2.60E-09	-3.02E-09
1.61E-07	1.50E-07	1.73E-07	1.69E-07	1.54E-07	1.58E-07	1.20E-07	9.10E-08	5.55E-08	4.04E-08	2.51E-08	4.95E-09	-2.05E-09	-2.73E-09	-1.19E-09	1.08E-09	1.06E-09	7.30E-10	-1.64E-09	-2.72E-09	-2.83E-09	-3.54E-09
1.47E-07	1.35E-07	1.48E-07	1.41E-07	1.30E-07	1.11E-07	8.89E-08	6.69E-08	3.91E-08	2.88E-08	1.03E-08	6.22E-10	-1.85E-09	-3.79E-09	-8.18E-10	1.62E-09	1.24E-09	4.49E-11	-2.53E-09	-3.12E-09	-2.97E-09	-4.22E-09
1.07E-07	1.21E-07	1.10E-07	9.80E-08	9.73E-08	8.03E-08	6.41E-08	4.41E-08	2.47E-08	2.10E-08	1.38E-09	-2.36E-09	2.54E-10	-3.60E-09	-7.45E-10	1.31E-09	9.61E-10	-1.93E-09	-3.70E-09	-3.87E-09	-3.57E-09	-4.25E-09
6.36E-08	9.07E-08	8.28E-08	7.23E-08	6.79E-08	5.62E-08	4.31E-08	2.64E-08	1.30E-08	1.52E-08	-3.08E-09	-2.97E-09	-1.58E-11	-3.95E-09	-1.96E-09	4.60E-10	-2.93E-10	-2.26E-09	-4.70E-09	-4.27E-09	-4.56E-09	-4.13E-09
3.81E-08	5.54E-08	6.15E-08	4.93E-08	4.37E-08	3.46E-08	2.57E-08	7.47E-09	4.48E-09	5.92E-09	-5.33E-09	-1.01E-09	-1.46E-09	-3.85E-09	-3.59E-09	-1.86E-09	-4.13E-09	-2.71E-09	-4.56E-09	-4.02E-09	-4.95E-09	-4.69E-09
1.41E-08	3.28E-08	3.73E-08	2.63E-08	2.32E-08	1.61E-08	8.86E-09	-6.29E-09	-2.60E-09	-7.91E-09	-7.45E-09	-2.74E-09	1.82E-09	-3.90E-09	-5.58E-09	-3.52E-09	-6.14E-09	-4.26E-09	-4.37E-09	-4.17E-09	-4.86E-09	-5.15E-09
-8.61E-09	1.20E-08	2.00E-08	5.26E-09	4.56E-09	-3.22E-10	-3.95E-09	-1.48E-08	-9.16E-09	-1.25E-08	-8.58E-09	3.36E-09	-1.19E-09	-4.92E-09	-6.33E-09	-4.53E-09	-7.02E-09	-5.47E-09	-4.66E-09	-4.13E-09	-4.98E-09	-5.43E-09
-2.53E-08	-6.57E-09	4.75E-09	-7.26E-09	-8.24E-09	-7.46E-09	-1.21E-08	-2.03E-08	-1.12E-08	-2.02E-08	-7.96E-09	3.46E-09	-2.79E-09	-6.22E-09	-7.12E-09	-5.32E-09	-6.89E-09	-5.69E-09	-5.06E-09	-3.91E-09	-5.41E-09	-5.86E-09
-3.78E-08	-1.79E-08	-5.81E-09	-1.41E-08	-2.43E-08	-1.44E-08	-1.87E-08	-2.43E-08	-1.58E-08	-1.86E-08	-2.77E-09	6.70E-10	-4.35E-09	-6.86E-09	-7.81E-09	-5.86E-09	-6.79E-09	-6.28E-09	-4.98E-09	-3.66E-09	-5.72E-09	-6.95E-09
-4.61E-08	-3.21E-08	-1.75E-08	-2.42E-08	-3.16E-08	-2.73E-08	-3.02E-08	-2.18E-08	-1.06E-08	-1.74E-08	2.91E-10	5.42E-09	-2.95E-09	-6.88E-09	-7.77E-09	-7.16E-09	-7.62E-09	-6.86E-09	-4.83E-09	-2.85E-09	-5.84E-09	-7.75E-09
-5.02E-08	-4.96E-08	-4.42E-08	-4.17E-08	-3.50E-08	-3.51E-08	-2.62E-08	-1.63E-08	-7.97E-09	-1.90E-08	-4.10E-09	-1.96E-09	-3.13E-09	-7.96E-09	-7.66E-09	-6.98E-09	-8.00E-09	-7.62E-09	-5.09E-09	-2.47E-09	-5.77E-09	-7.53E-09
-5.09E-08	-5.04E-08	-4.58E-08	-4.19E-08	-3.58E-08	-3.51E-08	-2.14E-08	-1.17E-08	-2.63E-08	-2.09E-08	-1.09E-08	-8.90E-09	-5.58E-09	-8.81E-09	-7.40E-09	-7.15E-09	-7.71E-09	-7.55E-09	-5.85E-09	-4.53E-09	-6.59E-09	-7.52E-09
-5.00E-08	-5.02E-08	-4.66E-08	-4.05E-08	-3.49E-08	-3.41E-08	-1.74E-08	-2.12E-08	-2.64E-08	-2.10E-08	-1.63E-08	-8.09E-09	-7.49E-09	-9.52E-09	-7.65E-09	-7.79E-09	-8.51E-09	-7.24E-09	-6.32E-09	-6.15E-09	-7.87E-09	-7.52E-09
-4.92E-08	-4.87E-08	-4.56E-08	-3.84E-08	-3.39E-08	-3.24E-08	-1.39E-08	-2.85E-08	-2.62E-08	-1.98E-08	-1.89E-08	-6.15E-09	-8.40E-09	-9.17E-09	-8.52E-09	-8.63E-09	-9.03E-09	-7.06E-09	-5.30E-09	-5.64E-09	-8.37E-09	-7.84E-09
-4.77E-08	-4.79E-08	-4.62E-08	-3.77E-08	-3.39E-08	-3.04E-08	-2.57E-08	-2.70E-08	-2.67E-08	-1.71E-08	-1.99E-08	-6.35E-09	-8.42E-09	-8.93E-09	-8.84E-09	-8.71E-09	-9.45E-09	-7.13E-09	-4.41E-09	-4.34E-09	-8.20E-09	-8.43E-09
-4.60E-08	-4.74E-08	-4.72E-08	-3.85E-08	-3.40E-08	-2.79E-08	-2.52E-08	-2.25E-08	-2.49E-08	-1.34E-08	-2.05E-08	-1.22E-08	-7.21E-09	-9.36E-09	-9.02E-09	-8.89E-09	-9.63E-09	-7.68E-09	-2.89E-09	-3.36E-09	-7.57E-09	-8.34E-09
-4.44E-08	-4.91E-08	-4.67E-08	-3.81E-08	-3.47E-08	-2.45E-08	-1.80E-08	-1.81E-08	-1.98E-08	-1.19E-08	-1.96E-08	-1.01E-08	-5.80E-09	-9.70E-09	-8.70E-09	-8.12E-09	-1.01E-08	-7.76E-09	-2.68E-09	-4.13E-09	-8.21E-09	-8.20E-09
-4.26E-08	-4.97E-08	-4.61E-08	-3.56E-08	-3.43E-08	-2.71E-08	-1.11E-08	-1.77E-08	-1.49E-08	-1.24E-08	-1.70E-08	-5.37E-09	-4.75E-09	-9.77E-09	-8.50E-09	-7.52E-09	-9.86E-09	-6.85E-09	-5.00E-09	-6.78E-09	-9.51E-09	-8.36E-09
-4.40E-08	-4.83E-08	-4.65E-08	-3.00E-08	-3.31E-08	-3.40E-08	-1.90E-08	-2.67E-08	-1.20E-08	-1.26E-08	-1.37E-08	-4.07E-09	-2.33E-09	-1.03E-08	-7.62E-09	-6.65E-09	-9.72E-09	-6.94E-09	-4.81E-09	-7.10E-09	-9.84E-09	-9.70E-09
-4.23E-08	-4.70E-08	-4.52E-08	-2.33E-08	-3.27E-08	-3.20E-08	-3.40E-08	-2.34E-08	-2.24E-08	-1.90E-08	-9.46E-09	-3.57E-09	-1.42E-09	-1.05E-08	-7.92E-09	-5.25E-09	-9.37E-09	-7.04E-09	-5.00E-09	-7.00E-09	-9.40E-09	-1.06E-08
-4.16E-08	-4.43E-08	-4.38E-08	-1.87E-08	-3.09E-08	-2.92E-08	-3.23E-08	-1.99E-08	-1.91E-08	-2.33E-08	-7.03E-09	-1.14E-08	-1.14E-08	-9.95E-09	-9.55E-09	-6.94E-09	-1.02E-08	-7.60E-09	-7.19E-09	-7.69E-09	-9.20E-09	-1.05E-08
-3.93E-08	-4.01E-08	-4.50E-08	-3.55E-08	-2.71E-08	-2.33E-08	-3.06E-08	-1.64E-08	-1.34E-08	-2.26E-08	-2.89E-09	-1.20E-08	-1.26E-08	-1.01E-08	-1.06E-08	-9.90E-09	-1.12E-08	-8.45E-09	-8.94E-09	-8.19E-09	-9.25E-09	-1.07E-08
-3.53E-08	-3.46E-08	-4.73E-08	-3.75E-08	-2.43E-08	-1.65E-08	-2.96E-08	-2.25E-08	-1.45E-08	-2.02E-08	-1.52E-08	-1.70E-08	-1.09E-08	-1.10E-08	-9.72E-09	-1.03E-08	-1.15E-08	-9.00E-09	-9.07E-09	-7.51E-09	-8.70E-09	-1.08E-08
-3.54E-08	-3.04E-08	-4.75E-08	-3.63E-08	-3.48E-08	-8.57E-09	-2.92E-08	-2.15E-08	-2.73E-08	-1.89E-08	-2.08E-08	-1.41E-08	-9.47E-09	-1.13E-08	-9.57E-09	-9.69E-09	-1.21E-08	-1.05E-08	-9.69E-09	-6.79E-09	-7.09E-09	-1.08E-08
-2.96E-08	-2.36E-08	-4.63E-08	-3.49E-08	-3.24E-08	-9.55E-09	-2.69E-08	-1.98E-08	-2.80E-08	-1.75E-08	-1.88E-08	-1.43E-08	-1.13E-08	-1.08E-08	-9.21E-09	-1.04E-08	-1.14E-08	-1.10E-08	-1.09E-08	-8.11E-09	-7.65E-09	-1.07E-08
-2.87E-08	-1.82E-08	-4.51E-08	-3.15E-08	-2.68E-08	-1.28E-08	-2.48E-08	-2.01E-08	-2.54E-08	-1.20E-08	-1.53E-08	-1.74E-08	-1.21E-08	-1.09E-08	-8.80E-09	-1.09E-08	-1.10E-08	-1.18E-08	-1.20E-08	-1.03E-08	-1.11E-08	-1.09E-08
-3.11E-08	-2.30E-08	-4.53E-08	-2.52E-08	-1.90E-08	-1.35E-08	-1.82E-08	-2.57E-08	-2.15E-08	-1.20E-08	-1.90E-08	-1.71E-08	-1.51E-08	-1.07E-08	-8.73E-09	-1.27E-08	-1.05E-08	-1.24E-08	-1.26E-08	-1.08E-08	-1.32E-08	-1.17E-08
-3.61E-08	-4.52E-08	-4.60E-08	-1.86E-08	-1.43E-08	-1.41E-08	-1.24E-08	-2.54E-08	-1.77E-08	-9.23E-09	-1.67E-08	-1.22E-08	-1.34E-08	-9.06E-09	-9.91E-09	-1.40E-08	-9.45E-09	-1.27E-08	-1.26E-08	-1.08E-08	-1.42E-08	-1.21E-08
-4.44E-08	-4.56E-08	-4.53E-08	-1.50E-08	-1.42E-08	-1.30E-08	-2.59E-08	-2.36E-08	-1.53E-08	-1.90E-08	-1.69E-08	-6.21E-09	-7.62E-09	-7.38E-09	-9.59E-09	-1.36E-08	-7.46E-09	-1.26E-08	-1.27E-08	-1.15E-08	-1.44E-08	-1.23E-08
-4.29E-08	-4.49E-08	-4.39E-08	-2.50E-08	-2.24E-08	-2.53E-08	-2.88E-08	-2.26E-08	-1.39E-08	-1.53E-08	-8.06E-09	-9.37E-09	-4.22E-09	-7.73E-09	-9.22E-09	-1.23E-08	-4.24E-09	-1.27E-08	-1.34E-08	-1.25E-08	-1.41E-08	-1.21E-08
-3.98E-08	-4.25E-08	-4.37E-08	-2.34E-08	-3.14E-08	-3.28E-08	-3.04E-08	-1.92E-08	-1.11E-08	-9.65E-09	-1.85E-09	-8.21E-09	-4.07E-09	-1.14E-08	-9.96E-09	-1.12E-08	-6.11E-09	-1.25E-08	-1.47E-08	-1.30E-08	-1.36E-08	-1.17E-08
-3.61E-08	-3.96E-08	-4.35E-08	-1.80E-08	-2.77E-08	-3.13E-08	-3.20E-08	-1.52E-08	-4.70E-09	-4.99E-09	-7.50E-09	-9.24E-09	-8.02E-09	-1.12E-08	-1.07E-08	-1.11E-08	-1.26E-08	-1.24E-08	-1.45E-08	-1.37E-08	-1.42E-08	-1.18E-08
-3.28E-08	-3.70E-08	-4.23E-08	-2.68E-08	-2.33E-08	-2.95E-08	-3.11E-08	-1.31E-08	-5.24E-09	-1.21E-08	-1.53E-08	-1.04E-08	-9.24E-09	-1.11E-08	-1.14E-08	-1.41E-08	-1.23E-08	-1.18E-08	-1.43E-08	-1.46E-08	-1.50E-08	-1.30E-08
-3.20E-08	-3.80E-08	-4.21E-08	-3.27E-08	-1.77E-08	-2.79E-08	-3.04E-08	-1.10E-08	-1.05E-08	-6.82E-09	-1.08E-08	-7.82E-09	-1.37E-08	-1.11E-08	-1.10E-08	-1.42E-08	-1.08E-08	-1.16E-08	-1.40E-08	-1.46E-08	-1.50E-08	-1.33E-08
-3.45E-08	-4.03E-08	-4.23E-08	-3.40E-08	-1.72E-08	-2.35E-08	-2.92E-08	-2.00E-08	-1.56E-08	-2.93E-09	-8.63E-09	-7.12E-09	-1.38E-08	-1.05E-08	-1.08E-08	-1.40E-08	-1.08E-08	-1.23E-08	-1.41E-08	-1.45E-08	-1.43E-08	-1.19E-08
-4.36E-08	-3.90E-08	-4.22E-08	-3.50E-08	-1.96E-08	-1.80E-08	-2.71E-08	-2.27E-08	-1.44E-08	-5.37E-09	-1.28E-08	-6.35E-09	-1.19E-08	-9.71E-09	-1.13E-08	-1.43E-08	-1.27E-08	-1.17E-08	-1.42E-08	-1.42E-08	-1.33E-08	-1.09E-08
-4.05E-08	-3.70E-08	-4.06E-08	-3.59E-08	-2.47E-08	-2.34E-08	-2.40E-08	-1.75E-08	-7.65E-09	-4.66E-09	-6.91E-09	-1.50E-08	-1.59E-08	-1.09E-08	-1.14E-08	-1.41E-08	-1.42E-08	-1.23E-08	-1.36E-08	-1.38E-08	-1.32E-08	-1.33E-08
-3.56E-08	-3.75E-08	-3.78E-08	-3.44E-08	-2.35E-08	-3.28E-08	-1.79E-08	-1.28E-08	-1.12E-09	-2.67E-09	-2.96E-09	-1.52E-08	-1.48E-08	-1.16E-08	-1.11E-08	-1.46E-08	-1.31E-08	-1.23E-08	-1.19E-08	-1.29E-08	-1.34E-08	-1.49E-08
-2.68E-08	-3.18E-08	-3.31E-08	-3.13E-08	-1.87E-08	-3.24E-08	-1.22E-08	-1.61E-08	3.35E-09	-1.53E-09	3.56E-11	-1.66E-08	-1.37E-08	-1.21E-08	-1.04E-08	-1.33E-08	-1.22E-08	-1.12E-08	-1.10E-08	-1.23E-08	-1.29E-08	-1.53E-08
-1.11E-08	-2.24E-08	-2.58E-08	-2.51E-08	-1.71E-08	-3.12E-08	-1.18E-08	-1.93E-08	-7.66E-09	-6.37E-10	-1.66E-09	-1.41E-08	-1.30E-08	-1.16E-08	-1.00E-08	-1.25E-08	-1.18E-08	-1.07E-08	-1.24E-08	-1.14E-08	-1.20E-08	-1.62E-08
6.22E-09	-6.79E-09	-1.43E-08	-1.71E-08	-2.25E-08	-2.93E-08	-1.41E-08	-1.41E-08	-1.18E-08	-6.07E-11	-2.71E-09	-1.26E-08	-1.46E-08	-1.05E-08	-8.08E-09	-1.21E-08	-1.08E-08	-1.04E-08	-1.18E-08	-1.10E-08	-1.24E-08	-1.70E-08
2.35E-08	1.08E-08	-8.88E-11	-6.71E-09	-2.05E-08	-2.52E-08	-1.23E-08	-1.01E-08	-5.72E-09	-2.84E-09	-1.02E-08	-9.52E-09	-1.28E-08	-9.70E-09	-6.36E-09	-1.25E-08	-9.61E-09	-8.87E-09	-9.26E-09	-1.18E-08	-1.40E-08	-1.72E-08
4.04E-08	2.73E-08	1.35E-08	-2.59E-09	-1.49E-08	-1.90E-08	-2.00E-08	-6.22E-09	-2.75E-09	-4.10E-10	-1.53E-08	-1.34E-08	-6.53E-09	-8.99E-09	-4.96E-09	-1.24E-08	-1.17E-08	-6.19E-09	-7.13E-09	-1.26E-08	-1.46E-08	-1.68E-08
5.48E-08	4.12E-08	2.74E-08	7.29E-09	-8.55E-09	-1.03E-08	-1.51E-08	-4.35E-09	-1.10E-08	-1.85E-08	-1.46E-08	-1.18E-08	-1.16E-08	-9.42E-09	-2.88E-09	-1.19E-08	-1.20E-08	-2.99E-09	-5.26E-09	-1.35E-08	-1.46E-08	-1.64E-08
6.68E-08	5.31E-08	3.87E-08	1.83E-08	7.06E-10	-4.74E-09	-5.30E-09	-1.35E-08	-1.01E-08	-1.80E-08	-1.10E-08	-1.35E-08	-9.46E-09	-9.68E-09	-1.95E-09	-1.21E-08	-1.21E-08	-1.16E-09	-5.74E-09	-1.42E-08	-1.45E-08	-1.70E-08
7.58E-08	6.48E-08	4.77E-08	2.69E-08	7.80E-09	-1.27E-08	1.54E-09	-1.93E-08	-1.35E-08	-1.68E-08	-1.29E-08	-1.35E-08	-1.38E-08	-1.10E-08	-5.70E-10	-1.24E-08	-1.21E-08	-5.20E-09	-1.12E-08	-1.47E-08	-1.48E-08	-1.73E-08
8.18E-08	7.22E-08	5.62E-08	3.23E-08	1.25E-08	-1.35E-08	2.68E-09	-1.56E-08	-1.67E-08	-1.42E-08	-1.28E-08	-8.21E-09	-1.25E-08	-1.20E-08	-7.09E-09	-1.21E-08	-1.11E-08	-1.37E-08	-1.34E-08	-1.46E-08	-1.53E-08	-1.74E-08
8.56E-08	7.62E-08	6.16E-08	3.57E-08	1.57E-08	-1.21E-08	8.01E-09	-1.34E-08	-1.96E-08	-1.97E-08	-1.12E-08	-6.82E-09	-1.74E-08	-1.39E-08	-1.28E-08	-1.21E-08	-1.03E-08	-1.32E-08	-1.32E-08	-1.41E-08	-1.50E-08	-1.76E-08
8.64E-08	7.82E-08	6.36E-08	3.75E-08	1.68E-08	-1.09E-08	1.64E-08	-2.56E-08	-1.91E-08	-1.85E-08	-1.04E-08	-2.33E-09	-1.67E-08	-1.41E-08	-1.17E-08	-1.05E-08	-1.01E-08	-1.30E-08	-1.32E-08	-1.42E-08	-1.48E-08	-1.77E-08
8.42E-08	7.67E-08	6.43E-08	3.59E-08	1.62E-08	-9.20E-09	1.53E-08	-2.36E-08	-1.52E-08	-1.76E-08	-8.45E-09	-1.14E-08	-1.78E-08	-1.37E-08	-1.11E-08	-9.93E-09	-9.83E-09	-1.43E-08	-1.43E-08	-1.39E-08	-1.43E-08	-1.70E-08
7.83E-08	7.21E-08	6.15E-08	3.22E-08	1.33E-08	-7.09E-09	-1.07E-09	-2.44E-08	-1.21E-08	-1.40E-08	-1.14E-08	-8.41E-09	-1.73E-08	-1.28E-08	-9.89E-09	-9.65E-09	-8.47E-09	-1.35E-08	-1.41E-08	-1.41E-08	-1.43E-08	-1.62E-08
6.98E-08	6.46E-08	5.54E-08	2.73E-08	9.02E-09	-6.32E-09	-7.90E-09	-2.29E-08	-1.56E-08	-9.83E-09	-9.74E-09	-8.79E-09	-1.44E-08	-1.20E-08	-1.07E-08	-1.01E-08	-1.10E-08	-1.36E-08	-1.40E-08	-1.36E-08	-1.42E-08	-1.64E-08
5.86E-08	5.68E-08	4.70E-08	2.18E-08	3.26E-09	-1.11E-08	-6.21E-09	-1.86E-08	-1.37E-08	-1.31E-08	-4.58E-09	-3.94E-09	-1.17E-08	-1.26E-08	-1.04E-08	-1.16E-08	-1.58E-08	-1.43E-08	-1.45E-08	-1.34E-08	-1.46E-08	-1.69E-08
4.59E-08	4.88E-08	3.30E-08	1.46E-08	-2.51E-09	-1.19E-08	-2.49E-08	-1.21E-08	-1.16E-08	-1.13E-08	-3.06E-09	-6.83E-09	-5.85E-09	-1.31E-08	-1.05E-08	-1.36E-08	-1.56E-08	-1.41E-08	-1.50E-08	-1.36E-08	-1.46E-08	-1.66E-08
3.08E-08	3.65E-08	2.25E-08	7.46E-09	-6.47E-09	-1.46E-08	-2.72E-08	-6.53E-09	-7.42E-09	-7.63E-09	-1.13E-08	-1.28E-08	-4.19E-09	-1.21E-08	-1.09E-08	-1.56E-08	-1.45E-08	-1.39E-08	-1.49E-08	-1.33E-08	-1.49E-08	-1.60E-08
1.53E-08	2.87E-08	1.31E-08	3.07E-09	-1.54E-08	-2.49E-08	-2.68E-08	-4.23E-09	-7.01E-09	-3.87E-09	-1.11E-08	-1.07E-08	-1.45E-08	-1.23E-08	-1.17E-08	-1.67E-08	-1.35E-08	-1.40E-08	-1.46E-08	-1.17E-08	-1.46E-08	-1.61E-08
1.38E-09	1.99E-08	5.45E-09	-5.25E-09	-2.77E-08	-2.04E-08	-2.35E-08	-7.73E-10	-9.58E-09	-6.94E-10	-6.58E-09	-1.59E-08	-1.73E-08	-1.19E-08	-9.65E-09	-1.58E-08	-1.20E-08	-1.42E-08	-1.44E-08	-1.12E-08	-1.45E-08	-1.64E-08

-7.74E-09	6.47E-09	-1.52E-08	-2.72E-08	-2.50E-08	-1.55E-08	-2.13E-08	2.90E-09	-3.32E-09	-1.12E-08	-1.50E-09	-1.86E-08	-1.75E-08	-1.03E-08	-8.36E-09	-1.47E-08	-1.09E-08	-1.41E-08	-1.43E-08	-1.37E-08	-1.49E-08	-1.50E-08
-1.31E-08	-2.46E-08	-3.55E-08	-2.65E-08	-2.17E-08	-1.58E-08	-1.71E-08	-1.15E-08	-3.24E-10	-1.63E-08	-6.87E-09	-1.45E-08	-1.97E-08	-8.41E-09	-1.31E-08	-1.39E-08	-9.09E-09	-1.46E-08	-1.38E-08	-1.42E-08	-1.48E-08	-1.27E-08
-2.68E-08	-3.69E-08	-3.51E-08	-2.57E-08	-1.67E-08	-9.40E-09	-1.39E-08	-4.17E-09	-1.07E-08	-1.17E-08	-1.16E-08	-9.77E-09	-2.06E-08	-7.22E-09	-1.37E-08	-1.42E-08	-1.14E-08	-1.50E-08	-1.36E-08	-1.38E-08	-1.55E-08	-9.88E-09
-3.88E-08	-3.82E-08	-3.39E-08	-2.73E-08	-1.07E-08	-6.52E-09	-1.33E-08	4.68E-10	-1.34E-08	-6.16E-09	-1.35E-08	-9.89E-09	-1.90E-08	-1.07E-08	-1.24E-08	-1.41E-08	-1.44E-08	-1.47E-08	-1.45E-08	-1.43E-08	-1.58E-08	-6.46E-09
-3.74E-08	-3.90E-08	-3.35E-08	-2.87E-08	-9.71E-09	-2.23E-08	-1.89E-08	-2.93E-09	-1.29E-08	-9.27E-09	-9.38E-09	-1.03E-08	-1.59E-08	-1.37E-08	-1.17E-08	-1.25E-08	-1.26E-08	-1.37E-08	-1.47E-08	-1.42E-08	-1.48E-08	-4.84E-09
-3.46E-08	-3.76E-08	-3.38E-08	-2.68E-08	-1.73E-08	-1.84E-08	-1.64E-08	-1.87E-08	-1.54E-08	-9.34E-09	-1.12E-08	-1.10E-08	-1.13E-08	-1.38E-08	-1.11E-08	-1.07E-08	-9.79E-09	-1.26E-08	-1.35E-08	-1.39E-08	-1.39E-08	-7.33E-09
-3.10E-08	-3.68E-08	-3.35E-08	-2.61E-08	-2.11E-08	-1.12E-08	-1.32E-08	-1.38E-08	-1.44E-08	-1.14E-08	-1.11E-08	-7.54E-09	-1.05E-08	-1.40E-08	-1.10E-08	-9.16E-09	-7.10E-09	-1.25E-08	-1.22E-08	-1.35E-08	-1.42E-08	-1.11E-08
-2.87E-08	-3.54E-08	-3.24E-08	-2.65E-08	-2.81E-08	-6.29E-09	-1.03E-08	-8.88E-09	-9.98E-09	-1.93E-08	-8.54E-09	-4.15E-09	-1.21E-08	-1.38E-08	-1.10E-08	-7.83E-09	-6.47E-09	-1.37E-08	-1.30E-08	-1.33E-08	-1.39E-08	-1.25E-08
-3.21E-08	-3.40E-08	-3.27E-08	-2.76E-08	-2.69E-08	-2.37E-08	-7.92E-09	-1.47E-08	-7.24E-09	-1.85E-08	-1.24E-08	-7.10E-09	-9.36E-09	-1.36E-08	-1.20E-08	-6.60E-09	-4.29E-09	-1.28E-08	-1.35E-08	-1.30E-08	-1.23E-08	-1.18E-08
-3.18E-08	-3.34E-08	-3.24E-08	-2.50E-08	-2.73E-08	-2.55E-08	-1.57E-08	-1.75E-08	-1.86E-08	-1.75E-08	-7.26E-09	-1.00E-08	-5.59E-09	-1.31E-08	-1.18E-08	-5.71E-09	-3.17E-09	-1.29E-08	-1.33E-08	-1.33E-08	-1.05E-08	-1.15E-08

2.93E-15	2.18E-15	1.64E-15	1.97E-15	1.11E-15	6.26E-16	4.97E-16	2.92E-17	4.38E-17	1.15E-18	2.05E-16	2.76E-16	5.63E-16	2.94E-16	3.95E-16	2.67E-16	4.04E-16	3.95E-16	3.92E-16	5.20E-16	4.66E-16	3.53E-16
3.06E-15	2.23E-15	1.42E-15	1.86E-15	1.02E-15	3.57E-16	4.60E-16	2.88E-17	1.14E-17	2.92E-18	1.13E-16	2.76E-16	5.83E-16	3.22E-16	4.66E-16	3.02E-16	3.33E-16	3.95E-16	4.19E-16	5.13E-16	4.54E-16	3.52E-16
3.05E-15	2.40E-15	2.14E-15	1.73E-15	9.86E-16	2.35E-16	4.54E-16	1.28E-17	4.56E-18	2.30E-17	6.61E-17	3.29E-16	3.65E-16	4.03E-16	5.71E-16	3.30E-16	3.82E-16	3.86E-16	4.20E-16	4.50E-16	4.24E-16	3.34E-16
2.92E-15	2.37E-15	2.39E-15	1.74E-15	1.02E-15	8.92E-16	4.55E-16	2.38E-16	1.50E-16	6.52E-18	1.21E-16	1.82E-16	2.13E-16	5.09E-16	6.87E-16	2.95E-16	4.23E-16	3.64E-16	3.72E-16	4.25E-16	3.81E-16	3.00E-16
2.90E-15	2.32E-15	2.37E-15	1.69E-15	1.16E-15	1.03E-15	3.17E-16	2.58E-16	1.30E-16	1.53E-18	4.00E-17	2.40E-16	1.57E-16	6.19E-16	5.34E-16	2.53E-16	4.45E-16	3.83E-16	3.51E-16	4.28E-16	3.69E-16	2.79E-16
2.92E-15	2.41E-15	2.45E-15	1.64E-15	1.20E-15	9.54E-16	2.19E-16	1.85E-16	9.36E-17	1.17E-17	1.66E-17	2.73E-16	1.94E-16	6.92E-16	3.08E-16	2.77E-16	5.35E-16	3.58E-16	3.52E-16	4.16E-16	3.99E-16	3.14E-16
2.81E-15	2.50E-15	2.47E-15	1.56E-15	1.25E-15	8.13E-16	2.83E-16	9.90E-17	4.72E-17	3.21E-17	1.22E-17	1.75E-16	2.44E-16	2.50E-16	2.62E-16	2.87E-16	4.17E-16	3.23E-16	3.29E-16	3.58E-16	3.86E-16	3.62E-16
2.39E-15	2.49E-15	2.38E-15	1.52E-15	1.25E-15	5.93E-16	6.26E-16	2.35E-17	8.33E-18	1.28E-17	3.80E-17	1.08E-16	4.11E-16	2.30E-16	2.54E-16	2.25E-16	3.79E-16	3.36E-16	3.18E-16	3.26E-16	3.63E-16	4.08E-16
1.96E-15	2.42E-15	2.16E-15	1.50E-15	1.18E-15	3.21E-16	6.22E-16	1.21E-17	3.59E-17	2.55E-18	9.48E-17	2.01E-16	4.88E-16	2.37E-16	2.34E-16	2.39E-16	2.79E-16	3.35E-16	2.98E-16	3.37E-16	3.69E-16	4.27E-16
1.78E-15	2.45E-15	1.85E-15	1.22E-15	1.16E-15	1.07E-16	6.16E-16	2.01E-16	7.33E-17	7.73E-18	1.56E-16	3.42E-16	4.75E-16	2.05E-16	2.32E-16	2.83E-16	2.79E-16	3.16E-16	2.70E-16	3.43E-16	4.03E-16	3.68E-16
2.83E-15	2.49E-15	1.49E-15	8.42E-16	1.20E-15	2.02E-16	5.15E-16	1.74E-16	4.58E-17	6.93E-17	1.81E-16	4.20E-16	3.13E-16	2.02E-16	2.39E-16	3.14E-16	2.77E-16	3.15E-16	2.99E-16	3.21E-16	4.14E-16	3.39E-16
2.99E-15	2.51E-15	1.17E-15	6.13E-16	1.22E-15	4.52E-16	4.28E-16	1.49E-16	1.06E-17	3.44E-17	3.21E-17	3.98E-16	2.80E-16	1.90E-16	2.29E-16	3.04E-16	2.74E-16	3.24E-16	3.12E-16	2.93E-16	3.80E-16	3.25E-16
2.99E-15	2.49E-15	1.20E-15	1.72E-15	1.27E-15	4.63E-16	2.89E-16	9.81E-17	3.17E-21	2.23E-17	2.02E-17	2.21E-16	1.73E-16	1.89E-16	2.44E-16	3.12E-16	2.60E-16	3.26E-16	3.49E-16	2.98E-16	3.61E-16	3.15E-16
2.92E-15	2.48E-15	2.33E-15	1.95E-15	1.27E-15	1.06E-15	1.53E-16	3.25E-16	1.92E-18	2.06E-17	5.51E-18	7.21E-17	2.60E-16	1.81E-16	2.61E-16	2.69E-16	2.61E-16	3.61E-16	3.79E-16	3.07E-16	3.73E-16	3.20E-16
2.94E-15	2.50E-15	2.29E-15	1.92E-15	1.32E-15	9.83E-16	1.50E-16	2.75E-16	1.60E-16	6.08E-18	3.15E-17	7.04E-17	2.53E-16	2.17E-16	2.44E-16	2.52E-16	2.49E-16	3.72E-16	3.49E-16	2.85E-16	3.69E-16	3.22E-16
3.01E-15	2.52E-15	2.18E-15	1.91E-15	1.27E-15	7.67E-16	5.46E-16	3.04E-16	1.10E-16	6.27E-20	5.29E-17	9.10E-17	2.05E-16	2.45E-16	2.41E-16	2.68E-16	2.50E-16	3.38E-16	2.82E-16	2.71E-16	3.70E-16	3.20E-16
3.24E-15	2.62E-15	2.39E-15	2.10E-15	1.23E-15	5.89E-16	4.32E-16	2.72E-16	5.88E-17	3.72E-17	4.99E-18	1.03E-16	1.08E-16	2.33E-16	2.32E-16	3.19E-16	2.59E-16	2.64E-16	2.55E-16	2.71E-16	3.23E-16	3.23E-16
3.38E-15	2.63E-15	2.41E-15	2.13E-15	1.27E-15	4.52E-16	2.74E-16	1.91E-16	2.18E-17	1.73E-16	9.84E-18	1.34E-16	1.34E-16	2.23E-16	1.97E-16	2.87E-16	2.37E-16	2.19E-16	2.61E-16	2.56E-16	2.65E-16	3.21E-16
3.32E-15	2.58E-15	2.49E-15	2.04E-15	1.36E-15	5.83E-16	1.85E-16	1.16E-16	3.87E-17	1.72E-16	4.10E-18	1.25E-16	1.89E-16	1.85E-16	1.73E-16	2.96E-16	2.41E-16	2.00E-16	2.57E-16	2.12E-16	2.17E-16	3.09E-16
3.24E-15	2.50E-15	2.40E-15	1.87E-15	1.37E-15	5.97E-16	5.56E-16	1.82E-16	3.78E-17	2.84E-17	3.88E-19	9.75E-17	1.40E-16	1.62E-16	1.38E-16	3.56E-16	2.46E-16	1.87E-16	2.49E-16	2.00E-16	1.78E-16	2.78E-16
3.23E-15	2.45E-15	2.46E-15	1.83E-15	1.33E-15	3.28E-16	5.06E-16	8.17E-17	2.10E-16	2.66E-17	1.28E-17	8.25E-17	1.58E-16	1.90E-16	1.18E-16	3.46E-16	2.08E-16	1.60E-16	2.11E-16	1.93E-16	1.84E-16	2.68E-16
3.16E-15	2.47E-15	2.43E-15	1.94E-15	1.35E-15	1.80E-16	4.10E-16	2.80E-17	1.97E-16	1.45E-17	4.57E-17	8.18E-18	1.22E-16	1.55E-16	1.09E-16	1.90E-16	2.02E-16	1.18E-16	1.81E-16	1.77E-16	2.02E-16	2.70E-16
3.13E-15	2.66E-15	2.41E-15	2.01E-15	1.44E-15	6.53E-16	2.47E-16	9.50E-17	1.35E-16	1.54E-18	1.46E-16	1.41E-17	1.22E-16	1.19E-16	1.12E-16	1.26E-16	1.71E-16	1.02E-16	1.75E-16	1.63E-16	1.96E-16	2.55E-16
3.12E-15	2.70E-15	2.45E-15	2.09E-15	1.48E-15	8.07E-16	8.98E-17	5.13E-17	6.17E-17	1.07E-17	1.82E-16	4.61E-17	9.09E-17	1.52E-16	1.32E-16	1.35E-16	1.38E-16	9.80E-17	1.82E-16	1.61E-16	1.78E-16	2.32E-16
3.15E-15	2.71E-15	2.64E-15	2.04E-15	1.44E-15	6.16E-16	3.21E-21	1.28E-16	1.76E-17	1.87E-18	1.72E-16	4.12E-17	6.59E-17	1.61E-16	1.39E-16	1.09E-16	1.46E-16	1.14E-16	2.02E-16	1.63E-16	1.49E-16	2.06E-16
3.22E-15	2.76E-15	2.57E-15	1.89E-15	1.42E-15	3.64E-16	1.58E-17	7.49E-17	4.41E-17	1.04E-17	1.76E-16	5.90E-17	3.47E-17	1.51E-16	1.53E-16	7.88E-17	1.63E-16	1.27E-16	1.70E-16	1.34E-16	1.58E-16	1.98E-16
3.24E-15	2.84E-15	2.47E-15	1.73E-15	1.41E-15	1.99E-16	7.17E-16	2.10E-17	8.32E-17	5.77E-17	8.21E-17	2.83E-17	2.58E-17	1.47E-16	1.32E-16	5.80E-17	2.02E-16	1.23E-16	1.67E-16	1.19E-16	1.52E-16	1.45E-16
3.23E-15	2.78E-15	2.36E-15	1.63E-15	1.38E-15	6.61E-16	7.14E-16	3.25E-16	1.69E-17	3.72E-18	3.34E-16	3.92E-17	5.22E-17	1.53E-16	8.42E-17	5.34E-17	1.24E-16	1.19E-16	1.77E-16	1.25E-16	1.50E-16	1.24E-16
3.22E-15	2.64E-15	2.42E-15	1.66E-15	1.25E-15	9.63E-16	6.86E-16	4.33E-16	1.27E-17	3.29E-19	2.21E-16	2.93E-17	1.28E-16	6.28E-17	5.22E-17	6.17E-17	1.07E-16	1.23E-16	2.00E-16	1.39E-16	1.26E-16	9.33E-17
3.26E-15	2.61E-15	2.36E-15	1.76E-15	1.17E-15	7.35E-16	6.27E-16	3.84E-16	4.59E-18	3.62E-17	1.00E-16	5.94E-17	9.83E-17	5.16E-17	3.36E-17	9.14E-17	1.25E-16	1.21E-16	2.74E-16	1.57E-16	8.96E-17	7.52E-17
3.42E-15	2.68E-15	2.12E-15	1.56E-15	1.18E-15	6.00E-16	4.46E-16	3.54E-16	1.96E-19	8.07E-17	2.22E-17	6.30E-18	9.55E-17	5.53E-17	4.24E-17	7.45E-17	1.18E-16	1.02E-16	3.12E-16	1.64E-16	8.65E-17	6.95E-17
3.46E-15	2.81E-15	1.74E-15	1.32E-15	1.28E-15	1.14E-15	1.74E-16	3.45E-16	1.06E-18	8.83E-20	2.86E-17	3.42E-17	9.42E-17	5.36E-17	5.78E-17	6.86E-17	1.10E-16	1.05E-16	2.14E-16	1.16E-16	7.76E-17	6.01E-17
3.39E-15	2.78E-15	1.46E-15	1.20E-15	1.36E-15	1.14E-15	3.24E-17	2.82E-16	3.01E-17	1.27E-17	1.97E-18	1.08E-18	2.92E-17	5.77E-17	6.59E-17	7.41E-17	1.16E-16	1.14E-16	8.41E-17	7.75E-17	7.30E-17	5.13E-17
2.96E-15	2.54E-15	1.55E-15	1.68E-15	1.33E-15	1.15E-15	1.54E-16	2.06E-16	4.45E-18	8.02E-18	6.50E-18	3.16E-17	1.18E-17	5.90E-17	6.71E-17	7.56E-17	1.32E-16	7.85E-17	7.31E-17	6.46E-17	7.31E-17	4.46E-17
2.28E-15	2.08E-15	2.09E-15	1.53E-15	1.13E-15	1.12E-15	7.99E-16	1.10E-16	7.95E-18	3.49E-19	1.99E-20	4.96E-17	2.15E-17	5.43E-17	7.43E-17	6.31E-17	9.77E-17	6.90E-17	7.94E-17	5.07E-17	7.67E-17	3.89E-17
1.40E-15	1.50E-15	1.55E-15	1.18E-15	9.02E-16	1.08E-15	7.18E-16	3.52E-17	1.79E-18	2.17E-17	5.12E-18	5.49E-18	1.93E-17	5.65E-17	6.70E-17	9.22E-17	7.43E-17	9.31E-17	8.84E-17	5.49E-17	8.63E-17	4.22E-17
5.36E-16	7.65E-16	8.14E-16	7.19E-16	6.01E-16	8.22E-16	5.29E-16	7.28E-17	2.28E-17	4.40E-17	5.04E-18	7.93E-18	5.22E-17	5.08E-17	5.74E-17	1.35E-16	6.86E-17	1.14E-16	9.78E-17	5.27E-17	7.29E-17	5.03E-17
1.14E-16	1.32E-16	1.55E-16	2.45E-16	2.29E-16	4.52E-16	4.20E-16	5.11E-17	3.01E-19	6.86E-17	1.05E-17	1.75E-17	1.35E-17	4.24E-17	4.71E-17	1.91E-16	6.53E-17	9.83E-17	8.20E-17	4.82E-17	7.14E-17	6.02E-17
5.75E-17	4.35E-17	7.38E-18	4.74E-17	1.92E-17	1.03E-16	2.79E-16	9.75E-17	3.72E-17	9.69E-19	4.75E-17	2.29E-17	2.89E-17	4.61E-17	5.00E-17	2.13E-16	6.30E-17	8.76E-17	7.50E-17	4.79E-17	8.39E-17	7.11E-17
8.07E-16	6.01E-16	3.76E-16	7.55E-18	1.27E-17	8.15E-18	4.98E-17	8.36E-17	1.45E-17	1.07E-16	8.69E-18	4.17E-17	3.83E-17	4.91E-17	5.76E-17	8.89E-17	4.80E-17	1.01E-16	8.39E-17	5.08E-17	8.71E-17	7.05E-17
2.14E-15	1.76E-15	1.29E-15	3.17E-16	3.38E-17	1.12E-16	3.33E-18	4.23E-18	7.11E-18	4.99E-17	3.33E-17	3.24E-17	7.01E-17	5.04E-17	5.49E-17	5.90E-17	2.94E-17	1.11E-16	8.03E-17	6.09E-17	7.73E-17	5.47E-17
3.56E-15	3.45E-15	2.65E-15	1.05E-15	4.18E-16	1.18E-17	3.81E-17	3.23E-17	5.40E-18	1.49E-17	6.68E-19	1.99E-19	1.19E-16	5.46E-17	6.95E-17	9.47E-17	3.14E-17	8.29E-17	6.99E-17	6.82E-17	7.53E-17	4.93E-17
5.62E-15	5.46E-15	4.71E-15	2.12E-15	1.16E-15	2.89E-16	2.02E-17	1.63E-17	3.49E-17	1.39E-17	2.23E-18	9.55E-18	2.68E-17	7.51E-17	1.06E-16	1.29E-16	2.96E-17	6.68E-17	5.72E-17	6.84E-17	6.89E-17	4.40E-17
8.28E-15	7.70E-15	7.29E-15	3.49E-15	2.19E-15	9.39E-16	2.64E-16	3.30E-17	3.09E-18	1.95E-16	3.74E-18	1.90E-17	4.42E-17	1.26E-16	1.72E-16	1.51E-16	2.94E-17	6.41E-17	6.44E-17	6.68E-17	6.19E-17	3.17E-17
1.14E-14	1.02E-14	9.87E-15	5.17E-15	3.59E-15	1.90E-15	8.08E-16	3.00E-16	4.87E-17	1.17E-16	9.12E-17	5.15E-18	9.41E-17	1.28E-16	1.48E-16	7.68E-17	2.29E-17	6.80E-17	6.43E-17	6.75E-17	5.53E-17	2.71E-17
1.48E-14	1.31E-14	1.26E-14	7.13E-15	5.30E-15	2.92E-15	1.65E-15	8.21E-16	3.45E-16	8.10E-17	9.62E-18	3.25E-20	4.89E-17	5.04E-17	4.88E-17	2.15E-17	1.73E-17	6.84E-17	6.62E-17	9.29E-17	5.15E-17	2.24E-17
1.82E-14	1.61E-14	1.54E-14	9.21E-15	6.72E-15	4.01E-15	2.71E-15	1.56E-15	8.17E-16	3.34E-16	7.00E-21	1.11E-17	2.68E-17	1.17E-17	1.64E-17	6.13E-18	1.07E-17	6.07E-17	8.76E-17	1.27E-16	4.80E-17	1.79E-17
2.17E-14	1.90E-14	1.85E-14	1.16E-14	8.78E-15	5.50E-15	3.87E-15	2.41E-15	1.36E-15	6.50E-16	5.98E-17	8.72E-18	1.49E-16	1.73E-17	1.96E-17	2.17E-18	8.63E-18	5.29E-17	9.32E-17	1.07E-16	3.83E-17	1.22E-17

2.47E-14	2.22E-14	2.11E-14	1.43E-14	1.09E-14	7.16E-15	5.31E-15	3.37E-15	2.01E-15	1.03E-15	2.12E-16	1.93E-18	2.40E-16	3.53E-17	3.04E-17	9.10E-18	6.01E-18	5.30E-17	3.70E-17	2.85E-17	1.78E-17	6.80E-18
2.72E-14	2.54E-14	2.38E-14	1.71E-14	1.35E-14	9.15E-15	7.01E-15	4.61E-15	2.80E-15	1.51E-15	3.90E-16	5.13E-17	1.60E-16	5.50E-17	2.10E-17	2.47E-17	7.44E-18	3.94E-17	2.05E-17	1.02E-17	9.31E-18	2.42E-18
2.99E-14	2.81E-14	2.59E-14	2.04E-14	1.65E-14	1.17E-14	8.94E-15	6.04E-15	3.73E-15	2.11E-15	6.14E-16	1.49E-16	1.15E-18	5.77E-17	1.66E-17	2.33E-17	1.41E-17	2.68E-17	1.69E-17	8.44E-18	6.38E-18	1.54E-18
3.26E-14	3.02E-14	2.71E-14	2.41E-14	1.99E-14	1.46E-14	1.12E-14	7.75E-15	4.81E-15	2.73E-15	9.42E-16	2.71E-16	2.74E-19	4.70E-17	9.83E-18	2.30E-17	2.16E-17	3.20E-17	1.77E-17	7.03E-18	5.73E-18	1.21E-18
3.50E-14	3.19E-14	2.92E-14	2.82E-14	2.35E-14	1.79E-14	1.40E-14	9.69E-15	6.04E-15	3.40E-15	1.39E-15	4.13E-16	6.55E-18	8.66E-17	9.20E-18	2.24E-17	8.59E-18	2.02E-17	1.63E-17	2.73E-18	3.92E-18	3.23E-19
3.64E-14	3.29E-14	3.11E-14	3.12E-14	2.68E-14	2.14E-14	1.72E-14	1.19E-14	7.24E-15	4.10E-15	1.74E-15	5.79E-16	3.30E-17	1.20E-16	8.40E-18	2.10E-18	1.22E-18	7.85E-18	1.59E-17	1.11E-18	9.93E-19	5.50E-20
3.69E-14	3.29E-14	3.29E-14	3.31E-14	2.95E-14	2.45E-14	2.04E-14	1.41E-14	8.24E-15	4.73E-15	1.97E-15	7.38E-16	7.88E-17	1.20E-16	5.98E-18	4.60E-18	9.92E-19	3.01E-18	1.17E-17	1.69E-18	2.40E-19	7.52E-19
3.71E-14	3.16E-14	3.36E-14	3.32E-14	3.13E-14	2.65E-14	2.27E-14	1.57E-14	8.84E-15	5.13E-15	2.11E-15	8.10E-16	1.03E-16	8.83E-17	8.53E-18	5.81E-18	3.98E-18	2.76E-18	4.26E-18	1.08E-18	4.39E-19	9.38E-19
3.73E-14	2.99E-14	3.31E-14	3.21E-14	3.17E-14	2.68E-14	2.35E-14	1.65E-14	8.85E-15	5.25E-15	2.11E-15	7.58E-16	7.53E-17	9.00E-17	5.68E-18	1.24E-17	5.40E-18	3.41E-18	9.07E-19	2.15E-20	3.62E-19	9.79E-19
3.68E-14	2.92E-14	3.15E-14	3.12E-14	3.25E-14	2.77E-14	2.45E-14	1.63E-14	8.28E-15	4.97E-15	1.96E-15	6.75E-16	4.05E-17	5.86E-17	3.10E-19	3.54E-17	1.04E-18	6.06E-19	2.72E-19	1.62E-18	3.48E-21	3.00E-18
3.44E-14	2.98E-14	3.10E-14	3.09E-14	3.34E-14	2.95E-14	2.49E-14	1.56E-14	7.58E-15	4.46E-15	1.71E-15	5.55E-16	1.78E-17	3.73E-17	1.69E-18	1.69E-17	9.79E-19	5.33E-21	9.72E-21	2.83E-18	7.52E-19	6.75E-18
3.13E-14	2.93E-14	3.16E-14	3.09E-14	2.96E-14	3.07E-14	2.39E-14	1.44E-14	6.55E-15	3.74E-15	1.44E-15	3.55E-16	3.49E-18	2.78E-17	2.87E-18	4.28E-19	9.46E-19	1.42E-19	6.30E-19	4.84E-18	4.13E-18	7.82E-18
2.88E-14	2.67E-14	3.23E-14	3.09E-14	2.87E-14	3.04E-14	1.96E-14	1.23E-14	4.95E-15	2.72E-15	1.17E-15	1.35E-16	3.09E-19	9.92E-18	1.79E-18	1.80E-22	3.13E-19	2.76E-23	2.34E-18	7.30E-18	6.73E-18	9.13E-18
2.58E-14	2.25E-14	3.00E-14	2.86E-14	2.39E-14	2.49E-14	1.43E-14	8.28E-15	3.08E-15	1.63E-15	6.32E-16	2.45E-17	4.19E-18	7.46E-18	1.42E-18	1.17E-18	1.12E-18	5.33E-19	2.70E-18	7.38E-18	8.02E-18	1.25E-17
2.15E-14	1.82E-14	2.18E-14	2.00E-14	1.68E-14	1.23E-14	7.89E-15	4.48E-15	1.53E-15	8.29E-16	1.05E-16	3.86E-19	3.42E-18	1.44E-17	6.69E-19	2.62E-18	1.53E-18	2.02E-21	6.43E-18	9.76E-18	8.83E-18	1.78E-17
1.15E-14	1.45E-14	1.21E-14	9.60E-15	9.47E-15	6.44E-15	4.11E-15	1.95E-15	6.11E-16	4.42E-16	1.90E-18	5.59E-18	6.44E-20	1.30E-17	5.55E-19	1.71E-18	9.23E-19	3.71E-18	1.37E-17	1.50E-17	1.28E-17	1.81E-17
4.04E-15	8.23E-15	6.86E-15	5.23E-15	4.60E-15	3.15E-15	1.85E-15	6.96E-16	1.70E-16	2.30E-16	9.46E-18	8.81E-18	2.50E-22	1.56E-17	3.84E-18	2.12E-19	8.60E-20	5.10E-18	2.21E-17	1.82E-17	2.08E-17	1.71E-17
1.45E-15	3.07E-15	3.79E-15	2.43E-15	1.91E-15	1.20E-15	6.61E-16	5.57E-17	2.01E-17	3.51E-17	2.84E-17	1.03E-18	2.12E-18	1.48E-17	1.29E-17	3.45E-18	1.70E-17	7.32E-18	2.08E-17	1.62E-17	2.45E-17	2.20E-17
1.99E-16	1.08E-15	1.39E-15	6.90E-16	5.40E-16	2.58E-16	7.85E-17	3.95E-17	6.75E-18	6.26E-17	5.55E-17	7.50E-18	3.32E-18	1.52E-17	3.12E-17	1.24E-17	3.77E-17	1.82E-17	1.91E-17	1.74E-17	2.36E-17	2.66E-17
7.41E-17	1.44E-16	4.00E-16	2.77E-17	2.08E-17	1.04E-19	1.56E-17	2.18E-16	8.39E-17	1.56E-16	7.36E-17	1.13E-17	1.40E-18	2.42E-17	4.00E-17	2.05E-17	4.93E-17	3.00E-17	2.17E-17	1.71E-17	2.48E-17	2.95E-17
6.38E-16	4.32E-17	2.26E-17	5.27E-17	6.79E-17	5.57E-17	1.46E-16	4.12E-16	1.25E-16	4.10E-16	6.34E-17	1.20E-17	7.78E-18	3.87E-17	5.07E-17	2.83E-17	4.75E-17	3.24E-17	2.56E-17	1.53E-17	2.93E-17	3.43E-17
1.43E-15	3.19E-16	3.38E-17	1.98E-16	5.92E-16	2.08E-16	3.51E-16	5.89E-16	2.49E-16	3.47E-16	7.65E-18	4.49E-19	1.89E-17	4.71E-17	6.10E-17	3.43E-17	4.61E-17	3.94E-17	2.48E-17	1.34E-17	3.27E-17	4.83E-17
2.12E-15	1.03E-15	3.05E-16	5.85E-16	9.96E-16	7.45E-16	9.12E-16	4.77E-16	1.12E-16	3.03E-16	8.49E-20	2.94E-17	8.72E-18	4.73E-17	6.04E-17	5.12E-17	5.81E-17	4.70E-17	2.33E-17	8.11E-18	3.41E-17	6.01E-17
2.52E-15	2.46E-15	1.96E-15	1.74E-15	1.22E-15	1.23E-15	6.88E-16	2.66E-16	6.35E-17	3.62E-16	1.68E-17	3.86E-18	9.81E-18	6.34E-17	5.87E-17	4.87E-17	6.40E-17	5.80E-17	2.59E-17	6.11E-18	3.33E-17	5.67E-17
2.59E-15	2.54E-15	2.10E-15	1.75E-15	1.28E-15	1.23E-15	4.58E-16	1.37E-16	6.90E-16	4.36E-16	1.18E-16	7.93E-17	3.11E-17	7.76E-17	5.48E-17	5.11E-17	5.95E-17	5.70E-17	3.42E-17	2.05E-17	4.35E-17	5.65E-17
2.50E-15	2.52E-15	2.17E-15	1.64E-15	1.22E-15	1.16E-15	3.02E-16	4.48E-16	6.96E-16	4.42E-16	2.67E-16	6.55E-17	5.60E-17	9.07E-17	5.85E-17	6.06E-17	7.25E-17	5.24E-17	3.99E-17	3.78E-17	6.19E-17	5.65E-17
2.43E-15	2.37E-15	2.08E-15	1.48E-15	1.15E-15	1.05E-15	1.93E-16	8.12E-16	6.89E-16	3.91E-16	3.56E-16	3.79E-17	7.06E-17	8.41E-17	7.27E-17	7.44E-17	8.16E-17	4.99E-17	2.81E-17	3.18E-17	7.01E-17	6.14E-17
2.27E-15	2.29E-15	2.14E-15	1.42E-15	1.15E-15	9.21E-16	6.59E-16	7.27E-16	7.11E-16	2.91E-16	3.96E-16	4.03E-17	7.08E-17	7.97E-17	7.81E-17	7.58E-17	8.94E-17	5.08E-17	1.95E-17	1.88E-17	6.73E-17	7.11E-17
2.12E-15	2.25E-15	2.23E-15	1.48E-15	1.15E-15	7.79E-16	6.37E-16	5.07E-16	6.18E-16	1.79E-16	4.19E-16	1.49E-16	5.19E-17	8.76E-17	8.14E-17	7.90E-17	9.27E-17	5.91E-17	8.36E-18	1.13E-17	5.73E-17	6.95E-17
1.97E-15	2.41E-15	2.18E-15	1.45E-15	1.20E-15	5.98E-16	3.24E-16	3.28E-16	3.93E-16	1.41E-16	3.84E-16	1.02E-16	3.36E-17	9.41E-17	7.57E-17	6.59E-17	1.03E-16	6.03E-17	7.19E-18	1.70E-17	6.75E-17	6.72E-17
1.81E-15	2.47E-15	2.12E-15	1.26E-15	1.18E-15	7.34E-16	1.23E-16	3.12E-16	2.21E-16	1.54E-16	2.88E-16	2.88E-17	2.26E-17	9.54E-17	7.22E-17	5.65E-17	9.72E-17	4.69E-17	2.50E-17	4.59E-17	9.04E-17	6.99E-17
1.93E-15	2.33E-15	2.16E-15	9.03E-16	1.10E-15	1.16E-15	3.62E-16	7.11E-16	1.44E-16	1.58E-16	1.87E-16	1.65E-17	5.43E-18	1.06E-16	5.81E-17	4.42E-17	9.44E-17	4.81E-17	2.32E-17	5.04E-17	9.68E-17	9.41E-17
1.79E-15	2.21E-15	2.05E-15	5.44E-16	1.07E-15	1.02E-15	1.16E-15	5.48E-16	5.03E-16	3.62E-16	8.96E-17	1.27E-17	2.02E-18	1.11E-16	6.27E-17	2.76E-17	8.77E-17	4.95E-17	2.50E-17	4.90E-17	8.83E-17	1.12E-16
1.73E-15	1.96E-15	1.91E-15	3.50E-16	9.55E-16	8.51E-16	1.04E-15	3.98E-16	3.65E-16	5.42E-16	4.95E-17	1.29E-16	1.31E-16	9.90E-17	9.13E-17	4.82E-17	1.04E-16	5.78E-17	5.17E-17	5.92E-17	8.46E-17	1.10E-16
1.54E-15	1.61E-15	2.03E-15	1.26E-15	7.35E-16	5.42E-16	9.39E-16	2.69E-16	1.79E-16	5.11E-16	8.36E-18	1.43E-16	1.59E-16	1.02E-16	1.13E-16	9.79E-17	1.25E-16	7.15E-17	8.00E-17	6.70E-17	8.55E-17	1.14E-16
1.25E-15	1.20E-15	2.23E-15	1.41E-15	5.91E-16	2.72E-16	8.79E-16	5.08E-16	2.10E-16	4.09E-16	2.30E-16	2.89E-16	1.20E-16	1.21E-16	9.45E-17	1.06E-16	1.33E-16	8.10E-17	8.22E-17	5.63E-17	7.56E-17	1.16E-16
1.25E-15	9.23E-16	2.26E-15	1.32E-15	1.21E-15	7.35E-17	8.53E-16	4.61E-16	7.45E-16	3.56E-16	4.34E-16	2.00E-16	8.98E-17	1.29E-16	9.15E-17	9.40E-17	1.47E-16	1.09E-16	9.39E-17	4.61E-17	5.03E-17	1.17E-16
8.74E-16	5.59E-16	2.14E-15	1.22E-15	1.05E-15	9.12E-17	7.22E-16	3.92E-16	7.85E-16	3.05E-16	3.52E-16	2.05E-16	1.27E-16	1.16E-16	8.49E-17	1.08E-16	1.29E-16	1.20E-16	1.20E-16	6.57E-17	5.85E-17	1.14E-16
8.23E-16	3.32E-16	2.04E-15	9.92E-16	7.16E-16	1.63E-16	6.15E-16	4.04E-16	6.44E-16	1.44E-16	2.35E-16	3.03E-16	1.46E-16	1.19E-16	7.74E-17	1.19E-16	1.21E-16	1.39E-16	1.45E-16	1.05E-16	1.23E-16	1.19E-16
9.68E-16	5.30E-16	2.06E-15	6.37E-16	3.62E-16	1.83E-16	3.33E-16	6.61E-16	4.61E-16	1.45E-16	3.60E-16	2.92E-16	2.28E-16	1.15E-16	7.63E-17	1.62E-16	1.11E-16	1.53E-16	1.59E-16	1.17E-16	1.75E-16	1.36E-16
1.31E-15	2.04E-15	2.11E-15	3.45E-16	2.04E-16	2.00E-16	1.54E-16	6.45E-16	3.12E-16	8.53E-17	2.80E-16	1.48E-16	1.79E-16	8.22E-17	9.82E-17	1.97E-16	8.93E-17	1.62E-16	1.60E-16	1.16E-16	2.02E-16	1.47E-16
1.97E-15	2.08E-15	2.06E-15	2.26E-15	2.01E-16	1.68E-16	6.73E-16	5.56E-16	2.35E-16	3.61E-16	2.84E-16	3.85E-17	5.81E-17	5.45E-17	9.19E-17	1.84E-16	5.56E-17	1.58E-16	1.60E-16	1.32E-16	2.06E-16	1.52E-16
1.84E-15	2.02E-15	1.93E-15	6.28E-16	5.04E-16	6.39E-16	8.27E-16	5.10E-16	1.95E-16	2.35E-16	6.49E-17	8.77E-17	1.78E-17	5.97E-17	8.51E-17	1.51E-16	1.80E-17	1.62E-16	1.80E-16	1.55E-16	1.99E-16	1.47E-16
1.58E-15	1.81E-15	1.91E-15	5.47E-16	9.86E-16	1.07E-15	9.25E-16	3.70E-16	1.23E-16	9.32E-17	3.44E-18	6.73E-17	1.65E-17	1.30E-16	9.92E-17	1.25E-16	3.74E-17	1.57E-16	2.16E-16	1.70E-16	1.86E-16	1.36E-16
1.30E-15	1.57E-15	1.89E-15	3.23E-16	7.66E-16	9.78E-16	1.02E-15	2.32E-16	2.21E-17	2.49E-17	5.63E-17	8.54E-17	6.44E-17	1.26E-16	1.14E-16	1.24E-16	1.59E-16	1.54E-16	2.10E-16	1.89E-16	2.01E-16	1.40E-16
1.08E-15	1.37E-15	1.79E-15	7.17E-16	5.44E-16	8.68E-16	9.70E-16	1.71E-16	2.74E-17	1.45E-16	2.35E-16	1.07E-16	8.53E-17	1.23E-16	1.29E-16	2.00E-16	1.52E-16	1.40E-16	2.04E-16	2.15E-16	2.25E-16	1.69E-16
1.02E-15	1.44E-15	1.77E-15	1.07E-15	3.12E-16	7.76E-16	9.22E-16	1.21E-16	1.10E-16	4.66E-17	1.17E-16	6.11E-17	1.89E-16	1.22E-16	1.20E-16	2.01E-16	1.17E-16	1.35E-16	1.95E-16	2.12E-16	2.25E-16	1.77E-16
1.19E-15	1.63E-15	1.79E-15	1.16E-15	2.96E-16	5.53E-16	8.52E-16	4.01E-16	2.44E-16	8.60E-18	7.44E-17	5.06E-17	1.90E-16	1.10E-16	1.17E-16	1.96E-16	1.17E-16	1.52E-16	1.99E-16	2.11E-16	2.04E-16	1.42E-16
1.90E-15	1.52E-15	1.78E-15	1.23E-15	3.83E-16	3.24E-16	7.34E-16	5.14E-16	2.06E-16	2.88E-17	1.64E-16	4.03E-17	1.41E-16	9.42E-17	1.27E-16	2.04E-16	1.62E-16	1.37E-16	2.00E-16	2.02E-16	1.77E-16	1.18E-16
1.64E-15	1.37E-15	1.64E-15	1.29E-15	6.08E-16	5.46E-16	5.75E-16	3.05E-16	5.86E-17	2.17E-17	4.77E-17	2.26E-16	2.53E-16	1.18E-16	1.29E-16	1.98E-16	2.01E-16	1.51E-16	1.85E-16	1.90E-16	1.74E-16	1.76E-16
1.27E-15	1.40E-15	1.43E-15	1.18E-15	5.54E-16	1.08E-15	3.20E-16	1.64E-16	1.25E-18	7.14E-18	8.79E-18	2.30E-16	2.19E-16	1.34E-16	1.22E-16	2.12E-16	1.73E-16	1.52E-16	1.42E-16	1.67E-16	1.80E-16	2.23E-16
7.16E-16	1.01E-15	1.09E-15	9.82E-16	3.49E-16	1.05E-15	1.49E-16	2.59E-16	1.12E-17	2.33E-18	1.27E-21	2.76E-16	1.88E-16	1.48E-16	1.08E-16	1.77E-16	1.49E-16	1.26E-16	1.22E-16	1.51E-16	1.66E-16	2.35E-16
1.22E-16	5.04E-16	6.65E-16	6.30E-16	2.91E-16	9.75E-16	1.39E-16	3.74E-16	5.86E-17	4.05E-19	2.77E-18	1.99E-16	1.69E-16	1.36E-16	1.01E-16	1.56E-16	1.39E-16	1.14E-16	1.54E-16	1.31E-16	1.45E-16	2.64E-16
3.87E-17	4.61E-17	2.05E-16	2.92E-16	5.07E-16	8.61E-16	1.99E-16	2.00E-16	1.40E-16	3.68E-21	7.34E-18	1.60E-16	2.14E-16	1.11E-16	6.54E-17	1.46E-16	1.17E-16	1.09E-16	1.38E-16	1.22E-16	1.53E-16	2.87E-16
5.50E-16	1.16E-16	7.89E-21	4.50E-17	4.19E-16	6.35E-16	1.51E-16	1.03E-16	3.27E-17	8.09E-18	1.05E-16	9.07E-17	1.64E-16	9.40E-17	4.04E-17	1.57E-16	9.23E-17	7.87E-17	8.58E-17	1.40E-16	1.97E-16	2.95E-16
1.63E-15	7.47E-16	1.83E-16	6.70E-18	2.21E-16	3.63E-16	3.99E-16	3.87E-17	7.57E-18	1.68E-19	2.36E-16	1.79E-16	4.27E-17	8.09E-17	2.46E-17	1.53E-16	1.37E-16	3.83E-17	5.09E-17	1.58E-16	2.14E-16	2.81E-16
3.00E-15	1.70E-15	7.50E-16	5.32E-17	7.30E-17	1.05E-16	2.29E-16	1.89E-17	1.20E-16	3.43E-16	2.14E-16	1.40E-16	1.35E-18	8.87E-17	8.28E-18	1.41E-16	1.45E-16	8.92E-18	2.77E-17	1.82E-16	2.14E-16	2.70E-16
4.46E-15	2.82E-15	1.50E-15	3.35E-16	4.98E-19	2.24E-17	2.81E-17	1.83E-16	1.02E-16	3.23E-16	1.22E-16	1.81E-16	8.95E-17	9.38E-17	3.80E-18	1.48E-16	1.45E-16	1.34E-18	3.29E-17	2.01E-16	2.11E-16	2.88E-16
5.74E-15	4.20E-15	2.28E-15	7.23E-16	6.08E-17	1.62E-16	2.38E-18	3.72E-16	1.84E-16	2.81E-16	1.66E-16	1.83E-16	1.90E-16	1.20E-16	3.25E-19	1.53E-16	1.47E-16	2.70E-17	1.25E-16	2.17E-16	2.20E-16	2.98E-16
6.70E-15	5.21E-15	3.16E-15	1.04E-15	1.57E-16	1.83E-16	7.20E-18	2.42E-16	2.78E-16	2.02E-16	1.64E-16	6.73E-17	1.56E-16	1.44E-16	5.02E-17	1.46E-16	1.22E-16	1.87E-16	1.79E-16	2.13E-16	2.34E-16	3.01E-16
7.32E-15	5.80E-15	3.80E-15	1.27E-15	2.47E-16	1.47E-16	6.42E-17	1.79E-16	3.86E-16	3.88E-16	1.25E-16	4.65E-17	3.02E-16	1.93E-16	1.63E-16	1.47E-16	1.07E-16	1.74E-16	1.74E-16	1.99E-16	2.26E-16	3.09E-16
7.46E-15	6.11E-15	4.05E-15	1.41E-15	2.83E-16	1.18E-16	2.69E-16	6.55E-16	3.65E-16	3.42E-16	1.08E-16	5.42E-18	2.80E-16	1.99E-16	1.36E-16	1.11E-16	1.02E-16	1.68E-16	1.74E-16	2.01E-16	2.19E-16	3.12E-16
7.09E-15	5.88E-15	4.14E-15	1.29E-15	2.63E-16	8.47E-17	2.34E-16	5.57E-16	2.32E-16	3.09E-16	7.14E-17	1.29E-16	3.16E-16	1.87E-16	1.24E-16	9.86E-17	9.66E-17	2.04E-16	2.06E-16	1.92E-16	2.04E-16	2.90E-16

6.14E-15	5.20E-15	3.78E-15	1.03E-15	1.77E-16	5.03E-17	1.15E-18	5.95E-16	1.46E-16	1.97E-16	1.30E-16	7.07E-17	3.00E-16	1.64E-16	9.78E-17	9.31E-17	7.17E-17	1.83E-16	1.99E-16	2.00E-16	2.05E-16	2.61E-16
4.87E-15	4.18E-15	3.07E-15	7.48E-16	8.14E-17	3.99E-17	6.24E-17	5.25E-16	2.44E-16	9.67E-17	9.49E-17	7.72E-17	2.08E-16	1.45E-16	1.14E-16	1.03E-16	1.21E-16	1.86E-16	1.96E-16	1.84E-16	2.02E-16	2.71E-16
3.43E-15	3.23E-15	2.21E-15	4.74E-16	1.06E-17	1.23E-16	3.85E-17	3.44E-16	1.87E-16	1.72E-16	2.10E-17	1.56E-17	1.37E-16	1.59E-16	1.08E-16	1.33E-16	2.48E-16	2.04E-16	2.11E-16	1.80E-16	2.13E-16	2.87E-16
2.10E-15	2.38E-15	1.09E-15	2.14E-16	6.32E-18	1.40E-16	6.20E-16	1.47E-16	1.35E-16	1.27E-16	9.36E-18	4.67E-17	3.42E-17	1.72E-16	1.11E-16	1.85E-16	2.43E-16	1.98E-16	2.25E-16	1.84E-16	2.14E-16	2.75E-16
9.46E-16	1.33E-15	5.05E-16	5.57E-17	4.18E-17	2.14E-16	7.42E-16	4.27E-17	5.51E-17	5.82E-17	1.27E-16	1.64E-16	1.76E-17	1.47E-16	1.18E-16	2.42E-16	2.09E-16	1.93E-16	2.22E-16	1.76E-16	2.21E-16	2.55E-16
2.34E-16	8.25E-16	1.71E-16	9.44E-18	2.36E-16	6.21E-16	7.17E-16	1.79E-17	4.91E-17	1.50E-17	1.24E-16	1.15E-16	2.11E-16	1.51E-16	1.37E-16	2.78E-16	1.82E-16	1.95E-16	2.12E-16	1.37E-16	2.14E-16	2.60E-16
1.90E-18	3.95E-16	2.97E-17	2.75E-17	7.66E-16	4.18E-16	5.54E-16	5.97E-19	9.17E-17	4.82E-19	4.34E-17	2.52E-16	3.00E-16	1.42E-16	9.32E-17	2.51E-16	1.44E-16	2.01E-16	2.08E-16	1.25E-16	2.11E-16	2.70E-16
5.98E-17	4.19E-17	2.30E-16	7.42E-16	6.26E-16	2.40E-16	4.55E-16	8.40E-18	1.11E-17	1.25E-16	2.24E-18	3.46E-16	3.08E-16	1.06E-16	6.99E-17	2.16E-16	1.19E-16	1.99E-16	2.04E-16	1.88E-16	2.22E-16	2.24E-16
1.72E-16	6.08E-16	1.26E-15	7.04E-16	4.69E-16	2.49E-16	2.92E-16	1.32E-16	1.05E-19	2.64E-16	4.72E-17	2.11E-16	3.90E-16	7.07E-17	1.71E-16	1.94E-16	8.27E-17	2.14E-16	1.90E-16	2.01E-16	2.19E-16	1.62E-16
7.18E-16	1.36E-15	1.24E-15	6.61E-16	2.79E-16	8.84E-17	1.93E-16	1.74E-17	1.15E-16	1.36E-16	1.34E-16	9.54E-17	4.26E-16	5.22E-17	1.87E-16	2.00E-16	1.30E-16	2.24E-16	1.86E-16	1.90E-16	2.40E-16	9.76E-17
1.51E-15	1.46E-15	1.15E-15	7.43E-16	1.15E-16	4.25E-17	1.76E-16	2.19E-19	1.80E-16	3.80E-17	1.83E-16	9.78E-17	3.62E-16	1.15E-16	1.54E-16	1.98E-16	2.06E-16	2.17E-16	2.09E-16	2.03E-16	2.49E-16	4.17E-17
1.40E-15	1.52E-15	1.12E-15	8.25E-16	9.43E-17	4.96E-16	3.56E-16	8.59E-18	1.67E-16	8.59E-17	8.79E-17	1.07E-16	2.52E-16	1.88E-16	1.37E-16	1.57E-16	1.58E-16	1.87E-16	2.15E-16	2.02E-16	2.20E-16	2.34E-17
1.20E-15	1.42E-15	1.14E-15	7.19E-16	2.98E-16	3.37E-16	2.68E-16	3.50E-16	2.36E-16	8.72E-17	1.25E-16	1.21E-16	1.27E-16	1.90E-16	1.23E-16	1.14E-16	9.58E-17	1.59E-16	1.83E-16	1.92E-16	1.94E-16	5.37E-17
9.58E-16	1.35E-15	1.12E-15	6.79E-16	4.46E-16	1.26E-16	1.76E-16	1.91E-16	2.07E-16	1.29E-16	1.23E-16	5.69E-17	1.09E-16	1.95E-16	1.20E-16	8.39E-17	5.04E-17	1.57E-16	1.49E-16	1.81E-16	2.02E-16	1.23E-16
8.21E-16	1.25E-15	1.05E-15	7.01E-16	7.90E-16	3.96E-17	1.05E-16	7.88E-17	9.96E-17	3.72E-16	7.29E-17	1.72E-17	1.47E-16	1.90E-16	1.21E-16	6.13E-17	4.19E-17	1.89E-16	1.68E-16	1.76E-16	1.94E-16	1.56E-16
1.03E-15	1.16E-15	1.07E-15	7.59E-16	7.23E-16	5.63E-16	6.27E-17	2.15E-16	5.24E-17	3.43E-16	1.54E-16	5.04E-17	8.76E-17	1.85E-16	1.44E-16	4.36E-17	1.84E-17	1.64E-16	1.81E-16	1.70E-16	1.50E-16	1.39E-16
1.01E-15	1.12E-15	1.05E-15	6.24E-16	7.46E-16	6.48E-16	2.48E-16	3.07E-16	3.44E-16	3.07E-16	5.28E-17	1.01E-16	3.12E-17	1.73E-16	1.39E-16	3.26E-17	1.00E-17	1.67E-16	1.77E-16	1.78E-16	1.11E-16	1.32E-16
8.09E-13	7.30E-13	7.10E-13	5.74E-13	4.92E-13	3.97E-13	3.04E-13	1.95E-13	1.04E-13	6.41E-14	3.01E-14	1.73E-14	1.68E-14	1.62E-14	1.43E-14	1.66E-14	1.60E-14	1.70E-14	1.84E-14	1.79E-14	1.98E-14	1.94E-14

1.95E-08	2.14E-08	2.13E-08	2.11E-08	2.42E-08	2.23E-08	2.53E-08	2.20E-08	2.48E-08	2.44E-08	2.78E-08	2.19E-08	2.08E-08	2.30E-08	2.01E-08	2.52E-08	2.96E-08	2.76E-08	2.96E-08	2.80E-08	2.70E-08	2.69E-08
1.88E-08	2.06E-08	2.02E-08	1.93E-08	2.35E-08	2.29E-08	2.43E-08	2.26E-08	2.89E-08	2.48E-08	2.58E-08	2.17E-08	2.10E-08	2.19E-08	2.04E-08	2.52E-08	2.79E-08	2.66E-08	3.09E-08	2.71E-08	2.67E-08	2.69E-08
1.81E-08	2.04E-08	1.89E-08	1.90E-08	2.29E-08	2.28E-08	2.41E-08	2.15E-08	3.15E-08	2.42E-08	2.46E-08	2.16E-08	2.07E-08	2.19E-08	1.95E-08	2.53E-08	2.74E-08	2.68E-08	3.15E-08	2.62E-08	2.62E-08	2.68E-08
1.83E-08	2.03E-08	1.89E-08	1.84E-08	2.19E-08	2.30E-08	2.37E-08	2.13E-08	3.02E-08	2.32E-08	2.40E-08	2.05E-08	1.94E-08	2.22E-08	1.95E-08	2.54E-08	2.73E-08	2.62E-08	2.94E-08	2.53E-08	2.68E-08	2.70E-08
1.86E-08	2.02E-08	1.90E-08	1.83E-08	2.15E-08	2.45E-08	2.26E-08	2.20E-08	1.88E-08	2.37E-08	2.31E-08	2.00E-08	1.86E-08	2.08E-08	2.01E-08	2.38E-08	2.74E-08	2.51E-08	2.84E-08	2.48E-08	2.82E-08	2.61E-08
1.95E-08	2.05E-08	1.88E-08	1.91E-08	2.23E-08	2.52E-08	2.19E-08	2.18E-08	2.02E-08	2.49E-08	2.21E-08	2.05E-08	1.76E-08	1.98E-08	2.08E-08	2.22E-08	2.58E-08	2.49E-08	2.69E-08	2.44E-08	2.93E-08	2.64E-08
2.06E-08	2.04E-08	1.81E-08	1.99E-08	2.15E-08	2.20E-08	2.23E-08	2.14E-08	2.12E-08	2.59E-08	2.18E-08	2.08E-08	1.75E-08	2.02E-08	2.01E-08	2.13E-08	2.58E-08	2.61E-08	2.59E-08	2.45E-08	2.50E-08	2.69E-08
2.20E-08	2.01E-08	1.78E-08	1.99E-08	2.01E-08	2.30E-08	2.25E-08	1.94E-08	2.13E-08	2.57E-08	2.25E-08	2.00E-08	1.66E-08	2.08E-08	1.90E-08	2.15E-08	2.62E-08	2.69E-08	2.39E-08	2.45E-08	2.38E-08	2.73E-08
2.29E-08	2.04E-08	1.82E-08	1.97E-08	1.97E-08	2.38E-08	2.12E-08	1.92E-08	2.28E-08	2.49E-08	2.16E-08	2.04E-08	1.66E-08	2.15E-08	1.84E-08	2.08E-08	2.57E-08	2.64E-08	2.42E-08	2.50E-08	2.43E-08	2.60E-08
2.05E-08	2.06E-08	1.89E-08	2.02E-08	2.18E-08	2.38E-08	1.96E-08	2.12E-08	2.10E-08	2.02E-08	2.00E-08	2.16E-08	1.85E-08	2.30E-08	1.85E-08	1.98E-08	2.49E-08	2.42E-08	2.54E-08	2.41E-08	2.49E-08	2.56E-08
1.86E-08	2.01E-08	1.88E-08	2.13E-08	2.43E-08	2.50E-08	2.03E-08	2.22E-08	1.99E-08	2.03E-08	2.02E-08	2.30E-08	2.00E-08	2.40E-08	2.00E-08	1.95E-08	2.43E-08	2.28E-08	2.63E-08	2.34E-08	2.47E-08	2.45E-08
1.84E-08	1.90E-08	1.83E-08	2.24E-08	2.61E-08	2.60E-08	2.09E-08	2.19E-08	2.01E-08	2.08E-08	1.99E-08	2.46E-08	2.34E-08	2.44E-08	2.03E-08	2.00E-08	2.48E-08	2.35E-08	2.68E-08	2.41E-08	2.40E-08	2.38E-08
1.83E-08	1.89E-08	1.88E-08	2.23E-08	2.57E-08	2.61E-08	2.04E-08	2.23E-08	2.03E-08	1.98E-08	1.89E-08	2.69E-08	2.94E-08	2.86E-08	2.12E-08	2.00E-08	2.37E-08	2.45E-08	2.46E-08	2.47E-08	2.27E-08	2.46E-08
1.79E-08	1.94E-08	1.93E-08	1.90E-08	2.26E-08	2.26E-08	1.98E-08	2.32E-08	2.04E-08	1.97E-08	1.75E-08	2.99E-08	3.68E-08	3.49E-08	2.42E-08	1.90E-08	2.30E-08	2.37E-08	2.41E-08	2.42E-08	2.18E-08	2.56E-08
1.71E-08	1.95E-08	1.90E-08	1.74E-08	1.99E-08	1.91E-08	1.83E-08	2.48E-08	1.90E-08	1.84E-08	1.73E-08	3.07E-08	3.83E-08	3.83E-08	2.67E-08	1.91E-08	2.17E-08	2.16E-08	2.48E-08	2.38E-08	2.20E-08	2.69E-08
1.65E-08	1.89E-08	1.83E-08	1.79E-08	1.87E-08	1.73E-08	1.83E-08	2.23E-08	1.73E-08	1.82E-08	1.72E-08	2.58E-08	3.29E-08	3.31E-08	2.65E-08	1.97E-08	2.10E-08	2.20E-08	2.48E-08	2.40E-08	2.27E-08	2.71E-08
1.67E-08	1.80E-08	1.79E-08	1.65E-08	1.81E-08	1.74E-08	1.86E-08	2.15E-08	1.70E-08	1.86E-08	1.69E-08	1.95E-08	2.56E-08	2.40E-08	2.35E-08	2.02E-08	2.02E-08	2.22E-08	2.51E-08	2.35E-08	2.27E-08	2.60E-08
1.68E-08	1.62E-08	1.77E-08	1.55E-08	1.80E-08	1.57E-08	1.85E-08	2.04E-08	1.84E-08	1.91E-08	1.67E-08	1.66E-08	1.95E-08	1.80E-08	1.71E-08	2.07E-08	2.05E-08	2.19E-08	2.33E-08	2.29E-08	2.29E-08	2.49E-08
1.63E-08	1.50E-08	1.71E-08	1.54E-08	1.76E-08	1.46E-08	1.79E-08	2.03E-08	1.99E-08	1.92E-08	1.66E-08	1.64E-08	1.75E-08	1.72E-08	1.79E-08	2.11E-08	1.97E-08	2.12E-08	2.25E-08	2.29E-08	2.23E-08	2.31E-08
1.58E-08	1.47E-08	1.62E-08	1.58E-08	1.70E-08	1.44E-08	1.82E-08	2.11E-08	2.11E-08	1.91E-08	1.66E-08	1.55E-08	1.71E-08	1.73E-08	1.82E-08	2.20E-08	1.96E-08	2.12E-08	2.18E-08	2.25E-08	2.03E-08	2.19E-08
1.52E-08	1.42E-08	1.52E-08	1.52E-08	1.62E-08	1.42E-08	1.99E-08	2.34E-08	2.27E-08	1.93E-08	1.61E-08	1.45E-08	1.65E-08	1.68E-08	1.75E-08	2.19E-08	1.91E-08	2.15E-08	2.19E-08	2.04E-08	2.00E-08	1.94E-08
1.45E-08	1.39E-08	1.51E-08	1.38E-08	1.57E-08	1.39E-08	2.29E-08	2.92E-08	2.85E-08	2.01E-08	1.62E-08	1.46E-08	1.75E-08	1.58E-08	1.71E-08	2.08E-08	1.93E-08	2.27E-08	2.23E-08	1.94E-08	2.02E-08	1.91E-08
1.38E-08	1.35E-08	1.51E-08	1.26E-08	1.53E-08	1.35E-08	2.77E-08	3.62E-08	3.72E-08	2.17E-08	1.68E-08	1.52E-08	1.56E-08	1.44E-08	1.74E-08	2.00E-08	1.79E-08	2.20E-08	2.02E-08	2.00E-08	2.04E-08	1.94E-08
1.28E-08	1.30E-08	1.35E-08	1.24E-08	1.43E-08	1.35E-08	3.07E-08	3.72E-08	4.35E-08	2.08E-08	1.72E-08	1.53E-08	1.29E-08	1.41E-08	1.81E-08	2.02E-08	1.62E-08	2.15E-08	1.81E-08	2.04E-08	1.96E-08	1.95E-08
1.26E-08	1.26E-08	1.18E-08	1.26E-08	1.47E-08	1.36E-08	2.90E-08	3.04E-08	4.20E-08	1.89E-08	1.70E-08	1.53E-08	1.27E-08	1.37E-08	1.74E-08	1.92E-08	1.59E-08	2.16E-08	1.73E-08	1.91E-08	1.89E-08	1.98E-08
1.24E-08	1.22E-08	1.20E-08	1.17E-08	1.41E-08	1.32E-08	2.35E-08	2.36E-08	3.19E-08	1.55E-08	1.59E-08	1.48E-08	1.35E-08	1.45E-08	1.63E-08	1.82E-08	1.58E-08	2.19E-08	1.67E-08	1.83E-08	1.73E-08	1.95E-08
1.18E-08	1.20E-08	1.13E-08	1.05E-08	1.31E-08	1.25E-08	1.16E-08	1.36E-08	1.31E-08	1.57E-08	1.53E-08	1.44E-08	1.47E-08	1.40E-08	1.63E-08	1.83E-08	1.60E-08	2.13E-08	1.65E-08	1.87E-08	1.73E-08	1.93E-08
1.09E-08	1.09E-08	1.06E-08	9.45E-09	1.12E-08	1.19E-08	1.22E-08	1.14E-08	1.35E-08	1.39E-08	1.56E-08	1.46E-08	1.46E-08	1.43E-08	1.69E-08	1.89E-08	1.55E-08	2.13E-08	1.58E-08	1.80E-08	1.85E-08	1.76E-08
1.07E-08	1.04E-08	1.06E-08	9.14E-09	1.05E-08	1.23E-08	1.14E-08	1.06E-08	1.23E-08	1.33E-08	1.46E-08	1.43E-08	1.31E-08	1.37E-08	1.78E-08	1.86E-08	1.53E-08	2.02E-08	1.57E-08	1.66E-08	1.77E-08	1.76E-08
1.09E-08	1.07E-08	1.01E-08	8.76E-09	1.01E-08	1.20E-08	1.14E-08	1.00E-08	1.08E-08	1.34E-08	1.26E-08	1.36E-08	1.28E-08	1.46E-08	1.69E-08	1.76E-08	1.55E-08	1.83E-08	1.53E-08	1.68E-08	1.60E-08	1.78E-08
1.08E-08	1.05E-08	9.44E-09	8.58E-09	9.58E-09	1.18E-08	1.20E-08	1.03E-08	1.13E-08	1.42E-08	1.25E-08	1.36E-08	1.42E-08	1.44E-08	1.48E-08	1.74E-08	1.64E-08	1.60E-08	1.49E-08	1.65E-08	1.63E-08	1.77E-08
1.02E-08	1.00E-08	9.51E-09	9.29E-09	9.84E-09	1.22E-08	1.24E-08	1.08E-08	1.25E-08	1.37E-08	1.38E-08	1.42E-08	1.40E-08	1.26E-08	1.30E-08	1.79E-08	1.73E-08	1.46E-08	1.47E-08	1.52E-08	1.74E-08	1.77E-08
9.45E-09	1.02E-08	9.48E-09	9.87E-09	1.07E-08	1.32E-08	1.18E-08	9.43E-09	1.26E-08	1.23E-08	1.32E-08	1.41E-08	1.22E-08	1.07E-08	1.25E-08	1.75E-08	1.63E-08	1.49E-08	1.35E-08	1.61E-08	1.55E-08	1.78E-08
8.72E-09	9.88E-09	1.00E-08	9.87E-09	1.08E-08	1.33E-08	1.24E-08	7.82E-09	9.53E-09	1.19E-08	1.16E-08	1.42E-08	1.19E-08	1.08E-08	1.21E-08	1.70E-08	1.58E-08	1.50E-08	1.14E-08	1.67E-08	1.37E-08	1.73E-08
7.85E-09	8.83E-09	8.85E-09	8.52E-09	1.02E-08	1.16E-08	1.25E-08	7.60E-09	8.92E-09	1.19E-08	1.07E-08	1.30E-08	1.17E-08	1.19E-08	1.29E-08	1.58E-08	1.38E-08	1.46E-08	1.09E-08	1.55E-08	1.33E-08	1.57E-08
7.81E-09	9.25E-09	9.03E-09	8.86E-09	9.86E-09	1.13E-08	1.20E-08	7.80E-09	9.23E-09	1.20E-08	1.13E-08	1.20E-08	1.03E-08	1.39E-08	1.40E-08	1.50E-08	1.27E-08	1.42E-08	1.07E-08	1.27E-08	1.37E-08	1.37E-08
8.24E-09	9.85E-09	9.27E-09	9.68E-09	9.66E-09	1.11E-08	1.19E-08	8.86E-09	8.30E-09	1.19E-08	1.14E-08	1.20E-08	1.08E-08	1.62E-08	1.58E-08	1.56E-08	1.22E-08	1.38E-08	1.01E-08	1.14E-08	1.31E-08	1.32E-08
8.22E-09	9.98E-09	9.33E-09	9.47E-09	9.22E-09	1.05E-08	1.29E-08	9.51E-09	8.99E-09	1.12E-08	1.06E-08	1.05E-08	9.73E-09	1.62E-08	1.50E-08	1.57E-08	1.13E-08	1.40E-08	9.73E-09	1.12E-08	1.07E-08	1.34E-08
7.10E-09	9.37E-09	8.46E-09	8.02E-09	8.28E-09	1.07E-08	1.37E-08	9.78E-09	9.02E-09	1.12E-08	1.05E-08	9.20E-09	8.23E-09	1.23E-08	1.32E-08	1.54E-08	1.17E-08	1.29E-08	1.11E-08	1.09E-08	9.48E-09	1.29E-08
6.62E-09	8.79E-09	7.31E-09	6.24E-09	7.39E-09	1.00E-08	1.30E-08	1.04E-08	8.92E-09	1.18E-08	1.16E-08	9.23E-09	9.00E-09	1.25E-08	1.47E-08	1.77E-08	1.24E-08	9.95E-09	1.23E-08	1.08E-08	1.01E-08	1.18E-08
6.66E-09	7.53E-09	7.53E-09	7.22E-09	6.73E-09	8.61E-09	1.08E-08	1.02E-08	7.70E-09	1.16E-08	1.18E-08	8.83E-09	1.02E-08	1.40E-08	2.13E-08	2.35E-08	1.43E-08	8.60E-09	1.33E-08	1.13E-08	1.04E-08	1.16E-08
6.63E-09	7.68E-09	8.36E-09	7.97E-09	5.75E-09	7.18E-09	1.12E-08	1.02E-08	7.42E-09	1.27E-08	1.13E-08	8.52E-09	1.33E-08	1.91E-08	2.98E-08	3.05E-08	1.67E-08	8.14E-09	1.17E-08	1.06E-08	9.37E-09	1.30E-08
5.90E-09	7.52E-09	7.65E-09	6.96E-09	5.10E-09	6.97E-09	9.77E-09	8.93E-09	9.20E-09	1.36E-08	1.06E-08	8.43E-09	1.81E-08	2.52E-08	3.57E-08	3.15E-08	1.51E-08	8.73E-09	1.19E-08	9.68E-09	8.32E-09	1.16E-08
4.91E-09	7.42E-09	6.84E-09	5.39E-09	4.61E-09	6.59E-09	6.54E-09	8.70E-09	1.04E-08	1.37E-08	9.61E-09	7.17E-09	2.22E-08	2.53E-08	3.20E-08	2.48E-08	1.20E-08	8.47E-09	1.07E-08	9.62E-09	8.09E-09	9.60E-09
4.27E-09	6.69E-09	6.29E-09	5.00E-09	3.92E-09	5.83E-09	5.39E-09	8.76E-09	9.23E-09	1.07E-08	8.06E-09	6.34E-09	1.05E-08	1.70E-08	2.31E-08	1.96E-08	1.13E-08	8.29E-09	1.06E-08	9.70E-09	7.83E-09	8.56E-09
3.51E-09	5.25E-09	5.84E-09	5.92E-09	4.01E-09	5.82E-09	6.97E-09	5.38E-09	5.68E-09	6.66E-09	6.04E-09	6.53E-09	6.45E-09	1.26E-08	1.76E-08	1.70E-08	1.32E-08	8.45E-09	1.19E-08	9.46E-09	8.09E-09	7.87E-09
3.27E-09	4.27E-09	5.87E-09	6.82E-09	5.01E-09	5.63E-09	8.36E-09	5.11E-09	3.06E-09	4.49E-09	5.06E-09	7.06E-09	6.47E-09	1.11E-08	1.50E-08	1.72E-08	1.47E-08	9.16E-09	1.07E-08	1.08E-08	9.37E-09	7.59E-09
2.70E-09	3.82E-09	5.56E-09	6.40E-09	3.88E-09	4.65E-09	6.99E-09	4.66E-09	3.81E-09	4.73E-09	5.67E-09	7.19E-09	6.53E-09	1.20E-08	1.45E-08	1.63E-08	1.46E-08	8.68E-09	9.45E-09	1.49E-08	1.11E-08	7.66E-09
1.95E-09	2.99E-09	4.33E-09	4.42E-09	2.09E-09	2.43E-09	3.77E-09	5.02E-09	4.92E-09	4.18E-09	6.16E-09	6.44E-09	5.63E-09	1.08E-08	1.33E-08	1.40E-08	1.21E-08	7.04E-09	8.98E-09	1.98E-08	1.23E-08	8.34E-09
9.80E-10	1.99E-09	2.91E-09	2.91E-09	1.58E-09	4.13E-10	4.44E-09	5.40E-09	4.50E-09	3.52E-09	5.25E-09	5.42E-09	4.05E-09	9.15E-09	1.21E-08	1.13E-08	7.90E-09	6.14E-09	8.91E-09	2.16E-08	1.20E-08	8.11E-09
5.64E-10	1.53E-09	2.56E-09	2.55E-09	2.04E-09	6.10E-10	4.81E-09	4.21E-09	2.25E-09	2.47E-09	3.51E-09	4.45E-09	3.50E-09	7.32E-09	1.06E-08	9.19E-09	5.78E-09	5.19E-09	7.30E-09	1.32E-08	9.06E-09	6.28E-09
-1.73E-10	1.11E-09	2.36E-09	3.04E-09	2.56E-09	1.90E-09	4.25E-09	5.00E-09	2.50E-09	1.46E-09	3.46E-09	4.49E-09	3.95E-09	7.76E-09	9.28E-09	9.11E-09	4.13E-09	3.68E-09	6.39E-09	6.45E-09	7.86E-09	6.00E-09
-9.21E-10	5.12E-10	2.15E-09	2.85E-09	1.92E-09	1.81E-09	2.82E-09	3.25E-09	2.84E-09	1.59E-09	4.07E-09	4.72E-09	4.24E-09	7.88E-09	9.01E-09	9.64E-09	4.22E-09	3.43E-09	5.67E-09	5.83E-09	7.47E-09	4.82E-09
-1.66E-09	-2.93E-10	1.20E-09	2.20E-09	2.08E-10	-5.86E-10	1.60E-09	2.54E-09	3.53E-09	1.32E-09	3.50E-09	4.40E-09	4.65E-09	5.77E-09	9.26E-09	9.02E-09	4.71E-09	3.68E-09	4.95E-09	4.85E-09	6.20E-09	4.39E-09
-1.86E-09	-1.14E-09	3.75E-10	6.14E-10	-1.08E-09	-1.77E-09	-2.01E-10	-1.47E-09	1.44E-09	6.48E-10	2.74E-09	3.79E-09	2.55E-09	5.89E-09	9.01E-09	6.94E-09	4.86E-09	3.38E-09	5.27E-09	4.50E-09	4.38E-09	4.61E-09

-2.13E-09	-1.58E-09	2.63E-10	-7.07E-11	-1.83E-09	-2.56E-09	-1.75E-09	-1.58E-09	-8.53E-10	-2.06E-10	2.46E-09	3.06E-09	2.31E-09	4.76E-09	8.01E-09	6.95E-09	5.61E-09	3.46E-09	4.82E-09	4.27E-09	4.08E-09	3.88E-09
-2.66E-09	-1.85E-09	-5.17E-10	2.24E-10	-1.97E-09	-2.66E-09	-2.14E-09	-1.94E-09	-5.30E-10	-4.08E-10	2.21E-09	2.70E-09	3.85E-09	5.22E-09	7.95E-09	6.55E-09	4.70E-09	2.96E-09	4.87E-09	4.40E-09	4.40E-09	1.19E-09
-3.01E-09	-2.08E-09	-9.05E-10	2.07E-10	-1.41E-09	-3.03E-09	-2.27E-09	-2.88E-09	-1.08E-09	-4.00E-10	1.42E-09	2.57E-09	4.83E-09	5.39E-09	9.21E-09	6.21E-09	3.04E-09	2.63E-09	3.05E-09	5.69E-09	4.46E-09	-1.01E-10
-3.00E-09	-2.12E-09	-9.41E-10	3.05E-10	-7.78E-10	-3.94E-09	-2.35E-09	-2.39E-09	-3.35E-09	-1.10E-09	1.13E-09	2.48E-09	4.18E-09	6.13E-09	1.05E-08	5.15E-09	3.26E-09	3.44E-09	2.25E-09	5.14E-09	3.63E-09	2.63E-10
-3.13E-09	-2.69E-09	-1.87E-09	-1.19E-10	-6.75E-10	-3.44E-09	-1.06E-09	-1.60E-09	-2.97E-09	-3.12E-09	1.47E-09	2.53E-09	4.08E-09	6.32E-09	9.06E-09	4.12E-09	2.73E-09	2.58E-09	1.36E-09	2.79E-09	2.47E-09	3.76E-10
-2.93E-09	-3.45E-09	-2.54E-09	1.02E-09	1.11E-09	-1.40E-09	-6.49E-10	-7.48E-10	-2.57E-09	-3.24E-09	1.84E-09	2.39E-09	4.81E-09	4.79E-09	1.03E-08	4.58E-09	1.55E-09	9.99E-10	1.07E-09	2.49E-09	2.10E-09	4.29E-10
-2.82E-09	-3.34E-09	-2.11E-09	3.13E-09	4.31E-09	1.03E-09	4.01E-11	-2.42E-09	-2.33E-09	-2.26E-09	1.37E-09	2.84E-09	6.05E-09	5.24E-09	1.15E-08	5.45E-09	1.53E-09	5.16E-10	6.38E-10	1.36E-09	1.54E-09	4.51E-10
-3.61E-09	-2.78E-09	-8.24E-10	5.61E-09	9.22E-09	4.55E-09	8.61E-10	-2.99E-09	-2.75E-09	-2.63E-09	7.22E-10	3.22E-09	6.84E-09	5.71E-09	1.01E-08	5.08E-09	2.04E-09	9.86E-10	1.66E-10	9.33E-10	2.40E-09	-7.85E-10
-4.10E-09	-2.94E-09	5.77E-10	8.77E-09	1.42E-08	8.25E-09	1.66E-09	-3.88E-09	-4.06E-09	-3.98E-09	9.38E-10	3.87E-09	7.06E-09	6.19E-09	9.39E-09	3.88E-09	2.17E-09	1.45E-09	-3.38E-10	-1.47E-10	3.09E-09	-8.03E-10
-3.84E-09	-2.81E-09	1.87E-09	1.01E-08	1.42E-08	9.67E-09	1.93E-09	-3.60E-09	-3.79E-09	-5.28E-09	6.28E-10	4.49E-09	7.09E-09	5.29E-09	9.31E-09	3.05E-09	-1.14E-09	2.31E-09	-5.04E-10	-1.33E-09	1.83E-09	-1.81E-09
-4.06E-09	-2.28E-09	1.41E-09	6.93E-09	8.93E-09	6.00E-09	2.31E-09	-4.11E-09	-3.19E-09	-5.23E-09	-8.02E-11	5.32E-09	8.38E-09	5.56E-09	1.06E-08	3.17E-09	-3.30E-09	9.47E-10	-2.40E-09	-2.05E-09	1.11E-09	-3.65E-09
-4.62E-09	-1.63E-09	8.76E-10	2.52E-09	1.64E-09	9.13E-10	2.29E-09	-4.77E-09	-4.12E-09	-5.05E-09	-1.17E-09	6.37E-09	1.03E-08	7.18E-09	1.08E-08	2.69E-09	-3.73E-09	-3.10E-11	-2.66E-09	-2.77E-09	-1.32E-09	-4.22E-09
-4.41E-09	-1.45E-09	2.47E-09	1.14E-09	-3.57E-09	-1.83E-09	-4.25E-09	-6.30E-09	-5.69E-09	-5.95E-09	-2.10E-09	7.84E-09	1.15E-08	9.24E-09	1.07E-08	2.30E-09	-2.58E-09	-5.28E-10	-2.54E-09	-3.14E-09	-2.16E-09	-4.69E-09
-3.74E-09	-2.89E-09	1.11E-09	3.44E-09	-4.91E-09	-1.39E-09	-2.62E-09	-7.10E-09	-5.72E-09	-6.50E-09	-2.26E-09	9.33E-09	1.25E-08	1.02E-08	1.35E-08	3.35E-09	-2.72E-09	-6.27E-10	-2.87E-09	-3.32E-09	-3.74E-09	-4.95E-09
-4.60E-09	-5.68E-09	-1.79E-09	6.62E-09	-4.13E-09	1.77E-09	5.58E-10	-5.03E-09	-5.45E-09	-6.72E-09	-1.69E-09	1.07E-08	1.68E-08	1.34E-08	1.66E-08	5.70E-09	-4.48E-09	-3.43E-09	-3.42E-09	-3.66E-09	-4.33E-09	-6.31E-09
-5.89E-09	-6.54E-09	-3.37E-09	4.95E-09	-3.33E-09	6.28E-09	1.92E-09	-3.02E-09	-4.60E-09	-6.95E-09	-8.92E-10	1.47E-08	2.46E-08	2.03E-08	2.21E-08	7.81E-09	-4.78E-09	-4.72E-09	-5.32E-09	-3.45E-09	-6.90E-09	-6.83E-09
-5.79E-09	-7.52E-09	-7.18E-09	-5.96E-09	-3.95E-09	8.00E-09	-6.26E-09	-5.18E-10	-5.63E-09	-7.09E-09	4.57E-10	2.07E-08	3.19E-08	2.88E-08	2.85E-08	1.07E-08	-4.67E-09	-4.92E-09	-7.48E-09	-3.79E-09	-7.93E-09	-7.74E-09
-5.89E-09	-8.30E-09	-7.94E-09	-9.30E-09	-6.73E-09	2.18E-09	-7.54E-09	2.26E-09	-7.28E-09	-7.06E-09	1.84E-09	2.37E-08	3.26E-08	3.04E-08	3.06E-08	1.23E-08	-5.29E-09	-5.46E-09	-7.82E-09	-5.78E-09	-7.05E-09	-8.36E-09
-7.06E-09	-9.35E-09	-8.15E-09	-9.86E-09	-7.63E-09	-5.37E-09	-7.79E-09	4.73E-09	-8.76E-09	-7.24E-09	1.52E-09	1.86E-08	2.66E-08	2.57E-08	2.41E-08	7.79E-09	-6.41E-09	-6.79E-09	-8.60E-09	-6.12E-09	-6.62E-09	-8.89E-09
-7.85E-09	-1.02E-08	-9.25E-09	-1.08E-08	-8.00E-09	-4.49E-09	-8.11E-09	-8.95E-09	-7.84E-09	-7.98E-09	-2.03E-09	7.42E-09	1.59E-08	1.55E-08	1.12E-08	-1.88E-09	-6.86E-09	-8.56E-09	-9.79E-09	-6.37E-09	-6.62E-09	-8.42E-09
-8.07E-09	-1.06E-08	-9.59E-09	-1.15E-08	-7.57E-09	-2.12E-09	-9.60E-09	-9.82E-09	-8.07E-09	-8.53E-09	-6.42E-09	-3.15E-09	1.83E-09	9.37E-10	-2.09E-09	-9.13E-09	-7.09E-09	-9.21E-09	-1.00E-08	-6.10E-09	-6.32E-09	-6.97E-09
-8.39E-09	-1.10E-08	-1.03E-08	-1.18E-08	-7.76E-09	-2.25E-09	-9.80E-09	-8.76E-09	-9.46E-09	-8.31E-09	-1.01E-08	-1.03E-08	-8.89E-09	-1.02E-08	-9.90E-09	-1.13E-08	-7.07E-09	-9.66E-09	-9.88E-09	-5.48E-09	-4.54E-09	-4.27E-09
-9.72E-09	-1.18E-08	-1.09E-08	-1.24E-08	-9.14E-09	-7.91E-09	-1.07E-08	-9.93E-09	-9.81E-09	-7.76E-09	-9.67E-09	-1.06E-08	-1.28E-08	-1.40E-08	-1.38E-08	-1.05E-08	-7.61E-09	-1.10E-08	-9.20E-09	-4.03E-09	4.37E-10	-9.71E-10
-1.08E-08	-1.21E-08	-1.16E-08	-1.33E-08	-1.04E-08	-8.21E-09	-9.94E-09	-1.07E-08	-9.84E-09	-7.60E-09	-7.97E-09	-1.01E-08	-1.34E-08	-1.50E-08	-1.61E-08	-1.26E-08	-9.20E-09	-1.27E-08	-8.84E-09	-2.06E-09	7.03E-09	4.41E-09
-1.15E-08	-1.26E-08	-1.26E-08	-1.43E-08	-1.14E-08	-9.08E-09	-1.04E-08	-1.12E-08	-9.15E-09	-9.14E-09	-6.59E-09	-9.53E-09	-1.52E-08	-1.63E-08	-1.78E-08	-1.55E-08	-1.25E-08	-1.41E-08	-9.02E-09	-1.92E-09	9.84E-09	8.54E-09
-1.27E-08	-1.35E-08	-1.38E-08	-1.46E-08	-1.22E-08	-1.02E-08	-1.16E-08	-1.11E-08	-8.80E-09	-9.41E-09	-5.93E-09	-8.43E-09	-1.55E-08	-1.72E-08	-1.88E-08	-1.59E-08	-1.40E-08	-1.51E-08	-1.11E-08	-8.80E-09	-2.05E-09	3.84E-09
-1.41E-08	-1.45E-08	-1.41E-08	-1.47E-08	-1.26E-08	-1.04E-08	-1.06E-08	-1.02E-08	-1.01E-08	-8.04E-09	-6.26E-09	-9.36E-09	-1.55E-08	-1.84E-08	-1.85E-08	-1.53E-08	-1.69E-08	-1.67E-08	-1.20E-08	-1.27E-08	-1.52E-08	-1.30E-08
-1.46E-08	-1.55E-08	-1.40E-08	-1.47E-08	-1.35E-08	-1.13E-08	-1.02E-08	-1.14E-08	-1.29E-08	-5.40E-09	-5.95E-09	-9.21E-09	-1.57E-08	-1.86E-08	-1.91E-08	-1.60E-08	-1.73E-08	-1.63E-08	-1.20E-08	-1.39E-08	-1.58E-08	-1.42E-08
-1.43E-08	-1.64E-08	-1.44E-08	-1.52E-08	-1.50E-08	-1.19E-08	-1.18E-08	-1.41E-08	-1.34E-08	-4.85E-09	-3.19E-09	-5.27E-09	-1.23E-08	-1.69E-08	-2.24E-08	-1.76E-08	-1.37E-08	-1.44E-08	-1.15E-08	-1.54E-08	-1.66E-08	-1.48E-08
-1.45E-08	-1.74E-08	-1.64E-08	-1.61E-08	-1.64E-08	-1.31E-08	-1.35E-08	-1.37E-08	-1.24E-08	-3.37E-09	1.30E-09	1.83E-09	-7.15E-09	-1.40E-08	-2.33E-08	-1.77E-08	-1.07E-08	-1.39E-08	-1.18E-08	-1.54E-08	-1.67E-08	-1.49E-08
-1.48E-08	-1.75E-08	-1.76E-08	-1.77E-08	-1.67E-08	-1.43E-08	-1.46E-08	-1.24E-08	-1.02E-08	2.58E-09	7.08E-09	7.61E-09	-6.99E-10	-1.19E-08	-2.37E-08	-1.78E-08	-1.06E-08	-1.35E-08	-1.39E-08	-1.46E-08	-1.64E-08	-1.52E-08
-1.52E-08	-1.71E-08	-1.76E-08	-1.87E-08	-1.67E-08	-1.39E-08	-1.38E-08	-1.15E-08	-8.71E-09	6.18E-09	9.21E-09	7.74E-09	1.47E-09	-9.47E-09	-2.27E-08	-1.78E-08	-1.24E-08	-1.50E-08	-1.44E-08	-1.33E-08	-1.59E-08	-1.44E-08
-1.44E-08	-1.65E-08	-1.69E-08	-1.84E-08	-1.70E-08	-1.37E-08	-1.42E-08	-1.30E-08	-8.50E-09	4.06E-09	6.91E-09	2.87E-09	-1.42E-09	-1.08E-08	-2.27E-08	-1.81E-08	-1.26E-08	-1.52E-08	-1.50E-08	-1.47E-08	-1.56E-08	-1.39E-08
-1.33E-08	-1.63E-08	-1.68E-08	-1.81E-08	-1.66E-08	-1.30E-08	-1.49E-08	-1.32E-08	-9.03E-09	-2.76E-09	7.54E-10	-4.98E-09	-7.35E-09	-1.57E-08	-2.35E-08	-1.86E-08	-1.35E-08	-1.55E-08	-1.46E-08	-1.50E-08	-1.35E-08	-1.31E-08
-1.30E-08	-1.62E-08	-1.68E-08	-1.86E-08	-1.59E-08	-1.38E-08	-1.49E-08	-1.26E-08	-1.17E-08	-9.63E-09	-6.62E-09	-1.27E-08	-1.39E-08	-1.97E-08	-2.36E-08	-1.96E-08	-1.44E-08	-1.50E-08	-1.39E-08	-1.33E-08	-1.21E-08	-1.28E-08
-1.40E-08	-1.62E-08	-1.64E-08	-1.91E-08	-1.64E-08	-1.48E-08	-1.41E-08	-1.25E-08	-1.56E-08	-1.38E-08	-1.18E-08	-1.73E-08	-2.02E-08	-2.10E-08	-2.30E-08	-2.05E-08	-1.45E-08	-1.45E-08	-1.35E-08	-1.32E-08	-1.26E-08	-1.33E-08
-1.49E-08	-1.64E-08	-1.62E-08	-1.92E-08	-1.74E-08	-1.56E-08	-1.34E-08	-1.19E-08	-1.49E-08	-1.34E-08	-1.37E-08	-1.79E-08	-1.97E-08	-2.16E-08	-2.23E-08	-2.00E-08	-1.36E-08	-1.32E-08	-1.48E-08	-1.40E-08	-1.40E-08	-1.37E-08
-1.43E-08	-1.62E-08	-1.70E-08	-1.86E-08	-1.77E-08	-1.52E-08	-1.51E-08	-1.22E-08	-1.35E-08	-1.21E-08	-1.33E-08	-1.82E-08	-1.93E-08	-2.04E-08	-2.18E-08	-1.97E-08	-1.29E-08	-1.41E-08	-1.55E-08	-1.49E-08	-1.52E-08	-1.29E-08
-1.35E-08	-1.64E-08	-1.94E-08	-1.80E-08	-1.68E-08	-1.47E-08	-1.58E-08	-1.34E-08	-1.32E-08	-1.08E-08	-1.28E-08	-1.77E-08	-1.92E-08	-1.97E-08	-2.27E-08	-2.02E-08	-1.36E-08	-1.58E-08	-1.61E-08	-1.50E-08	-1.67E-08	-1.23E-08
-1.40E-08	-1.77E-08	-2.06E-08	-1.80E-08	-1.64E-08	-1.49E-08	-1.56E-08	-1.29E-08	-1.39E-08	-8.14E-09	-1.20E-08	-1.76E-08	-1.88E-08	-2.04E-08	-2.37E-08	-2.10E-08	-1.51E-08	-1.64E-08	-1.58E-08	-1.60E-08	-1.92E-08	-1.35E-08
-1.45E-08	-1.80E-08	-2.07E-08	-1.78E-08	-1.70E-08	-1.75E-08	-1.35E-08	-1.17E-08	-1.26E-08	-5.81E-09	-1.14E-08	-1.96E-08	-1.98E-08	-2.21E-08	-2.43E-08	-2.22E-08	-1.57E-08	-1.77E-08	-1.67E-08	-1.76E-08	-2.00E-08	-1.53E-08
-1.54E-08	-1.75E-08	-2.03E-08	-1.70E-08	-1.71E-08	-1.69E-08	-1.27E-08	-1.37E-08	-1.14E-08	-5.35E-09	-1.28E-08	-2.18E-08	-2.17E-08	-2.44E-08	-2.34E-08	-2.25E-08	-1.56E-08	-1.85E-08	-1.77E-08	-1.95E-08	-2.05E-08	-1.69E-08
-1.64E-08	-1.66E-08	-2.00E-08	-1.63E-08	-1.70E-08	-1.63E-08	-1.21E-08	-1.28E-08	-1.25E-08	-6.32E-09	-1.56E-08	-2.20E-08	-2.29E-08	-2.43E-08	-2.28E-08	-2.33E-08	-1.72E-08	-2.04E-08	-1.83E-08	-2.01E-08	-1.98E-08	-1.77E-08
-1.69E-08	-1.63E-08	-2.00E-08	-1.60E-08	-1.70E-08	-1.65E-08	-1.14E-08	-1.22E-08	-1.59E-08	-1.18E-08	-1.75E-08	-2.14E-08	-2.23E-08	-2.42E-08	-2.36E-08	-2.41E-08	-1.87E-08	-2.06E-08	-1.72E-08	-2.01E-08	-1.87E-08	-1.65E-08
-1.86E-08	-1.64E-08	-2.00E-08	-1.67E-08	-1.77E-08	-1.83E-08	-1.11E-08	-1.08E-08	-1.79E-08	-1.36E-08	-1.62E-08	-2.14E-08	-2.21E-08	-2.46E-08	-2.47E-08	-2.47E-08	-1.86E-08	-2.05E-08	-1.57E-08	-1.99E-08	-1.82E-08	-1.86E-08
-1.97E-08	-1.78E-08	-1.91E-08	-1.64E-08	-1.82E-08	-2.04E-08	-1.79E-08	-1.01E-08	-1.67E-08	-1.23E-08	-1.58E-08	-2.12E-08	-2.21E-08	-2.52E-08	-2.46E-08	-2.46E-08	-1.88E-08	-1.93E-08	-1.57E-08	-1.90E-08	-1.83E-08	-1.86E-08
-1.95E-08	-1.80E-08	-1.76E-08	-1.58E-08	-1.96E-08	-2.23E-08	-1.92E-08	-1.39E-08	-1.64E-08	-1.54E-08	-1.68E-08	-2.00E-08	-2.11E-08	-2.59E-08	-2.43E-08	-2.42E-08	-1.82E-08	-1.91E-08	-1.62E-08	-1.87E-08	-1.80E-08	-1.86E-08
-1.90E-08	-1.83E-08	-1.85E-08	-1.58E-08	-2.03E-08	-2.32E-08	-2.03E-08	-1.56E-08	-1.42E-08	-1.64E-08	-1.67E-08	-1.76E-08	-1.86E-08	-2.52E-08	-2.37E-08	-2.33E-08	-1.81E-08	-1.99E-08	-1.73E-08	-1.88E-08	-1.68E-08	-1.84E-08
-1.91E-08	-1.84E-08	-1.98E-08	-1.65E-08	-2.08E-08	-2.28E-08	-2.06E-08	-1.59E-08	-1.34E-08	-1.69E-08	-1.45E-08	-1.56E-08	-1.49E-08	-2.53E-08	-2.34E-08	-2.29E-08	-1.88E-08	-1.93E-08	-1.91E-08	-1.91E-08	-1.65E-08	-1.74E-08
-1.93E-08	-1.87E-08	-1.96E-08	-1.81E-08	-2.16E-08	-2.24E-08	-1.92E-08	-1.48E-08	-1.59E-08	-1.85E-08	-1.53E-08	-1.76E-08	-1.38E-08	-2.55E-08	-2.30E-08	-2.26E-08	-2.04E-08	-1.88E-08	-2.10E-08	-2.01E-08	-1.77E-08	-1.74E-08
-1.91E-08	-1.85E-08	-1.82E-08	-2.04E-08	-2.27E-08	-2.16E-08	-1.87E-08	-1.84E-08	-1.87E-08	-1.87E-08	-2.01E-08	-2.06E-08	-1.82E-08	-2.47E-08	-2.30E-08	-2.18E-08	-2.05E-08	-1.90E-08	-2.14E-08	-2.09E-08	-1.76E-08	-1.82E-08
-1.82E-08	-1.78E-08	-1.77E-08	-2.20E-08	-2.20E-08	-2.05E-08	-1.90E-08	-1.69E-08	-1.99E-08	-1.88E-08	-1.97E-08	-2.05E-08	-2.28E-08	-2.60E-08	-2.42E-08	-2.17E-08	-2.12E-08	-2.04E-08	-2.20E-08	-2.04E-08	-1.88E-08	-1.97E-08
-1.77E-08	-1.74E-08	-1.77E-08	-2.21E-08	-2.08E-08	-2.04E-08	-1.85E-08	-1.75E-08	-2.02E-08	-1.99E-08	-1.97E-08	-2.14E-08	-2.29E-08	-2.63E-08	-2.44E-08	-2.25E-08	-2.10E-08	-2.15E-08	-2.15E-08	-1.90E-08	-1.87E-08	-2.00E-08
-1.68E-08	-1.69E-08	-1.81E-08	-2.15E-08	-2.02E-08	-2.08E-08	-1.76E-08	-2.02E-08	-2.03E-08	-2.01E-08	-1.99E-08	-2.32E-08	-2.50E-08	-2.74E-08	-2.43E-08	-2.29E-08	-2.01E-08	-2.18E-08	-2.15E-08	-2.08E-08	-1.84E-08	-2.01E-08
-1.62E-08	-1.61E-08	-1.85E-08	-2.15E-08	-2.07E-08	-1.97E-08	-2.01E-08	-2.11E-08	-2.21E-08	-2.03E-08	-2.05E-08	-2.36E-08	-2.58E-08	-2.71E-08	-2.53E-08	-2.25E-08	-1.95E-08	-2.02E-08	-2.21E-08	-2.21E-08	-1.83E-08	-2.17E-08
-1.57E-08	-1.53E-08	-1.81E-08	-2.21E-08	-2.07E-08	-1.85E-08	-2.00E-08	-2.14E-08	-2.25E-08	-1.92E-08	-2.03E-08	-2.36E-08	-2.67E-08	-2.77E-08	-2.60E-08	-2.30E-08	-1.91E-08	-1.88E-08	-2.15E-08	-2.22E-08	-1.92E-08	-2.28E-08
-1.51E-08	-1.47E-08	-1.68E-08	-2.20E-08	-2.00E-08	-1.99E-08	-1.93E-08	-2.01E-08	-2.18E-08	-1.77E-08	-2.02E-08	-2.43E-08	-2.67E-08	-2.84E-08	-2.72E-08	-2.40E-08	-1.92E-08	-1.80E-08	-2.02E-08	-2.10E-08	-2.22E-08	-2.41E-08
-1.47E-08	-1.41E-08	-1.58E-08	-2.18E-08	-2.00E-08	-2.13E-08	-1.95E-08	-2.06E-08	-2.25E-08	-1.58E-08	-2.02E-08	-2.36E-08	-2.56E-08	-2.85E-08	-2.75E-08	-2.43E-08	-2.07E-08	-1.75E-08	-1.94E-08	-2.15E-08	-2.30E-08	-2.39E-08
-1.51E-08	-1.46E-08	-1.78E-08	-2.18E-08	-2.11E-08	-2.09E-08	-2.09E-08	-2.06E-08	-2.26E-08	-1.79E-08	-2.05E-08	-2.28E-08	-2.47E-08	-2.81E-08	-2.72E-08	-2.52E-08	-2.18E-08	-1.55E-08	-1.83E-08	-2.40E-08	-2.25E-08	-2.33E-08
-1.51E-08	-1.59E-08	-1.92E-08	-2.13E-08	-2.02E-08	-1.97E-08	-2.12E-08	-2.18E-08	-2.21E-08	-1.86E-08	-2.14E-08	-2.36E-08	-2.59E-08	-2.70E-08	-2.60E-08	-2.58E-08	-2.16E-08	-1.21E-08	-1.48E-08	-2.60E-08	-2.30E-08	-2.28E-08
-1.50E-08	-1.64E-08	-1.89E-08	-1.96E-08	-1.78E-08	-1.92E-08	-2.20E-08	-2.14E-08	-2.14E-08	-1.78E-08	-2.19E-08	-2.51E-08	-2.65E-08	-2.66E-08	-2.54E-08	-2.63E-08	-2.23E-08	-1.10E-08	-1.04E-08	-2.55E-08	-2.34E-08	-2.33E-08
-1.49E-08	-1.63E-08	-1.85E-08	-1.86E-08	-1.70E-08	-1.90E-08	-2.11E-08	-2.01E-08	-2.03E-08	-1.77E-08	-2.17E-08	-2.49E-08	-2.69E-08	-2.68E-08	-2.65E-08	-2.65E-08	-2.39E-08	-1.64E-08	-9.47E-09	-2.43E-08	-2.30E-08	-2.33E-08
-1.50E-08	-1.54E-08	-1.84E-08	-2.04E-08	-1.89E-08	-1.75E-08	-1.98E-08	-1.83E-08	-1.86E-08	-2.11E-08	-2.19E-08	-2.47E-08	-2.64E-08	-2.69E-08	-2.79E-08	-2.61E-08	-2.40E-08	-2.13E-08	-1.69E-08	-2.44E-08	-2.15E-08	-2.40E-08

-1.39E-08	-1.45E-08	-1.89E-08	-2.13E-08	-1.98E-08	-1.55E-08	-1.84E-08	-1.69E-08	-1.81E-08	-2.04E-08	-2.21E-08	-2.49E-08	-2.56E-08	-2.64E-08	-2.77E-08	-2.57E-08	-2.23E-08	-2.10E-08	-2.30E-08	-2.42E-08	-2.17E-08	-2.47E-08
-1.20E-08	-1.31E-08	-1.78E-08	-2.07E-08	-1.91E-08	-1.33E-08	-1.71E-08	-1.72E-08	-1.80E-08	-2.01E-08	-2.17E-08	-2.56E-08	-2.52E-08	-2.54E-08	-2.73E-08	-2.64E-08	-2.06E-08	-2.01E-08	-2.27E-08	-2.40E-08	-2.34E-08	-2.70E-08
-9.60E-09	-1.12E-08	-1.76E-08	-2.00E-08	-1.89E-08	-1.17E-08	-1.75E-08	-1.78E-08	-1.81E-08	-2.08E-08	-2.14E-08	-2.59E-08	-2.47E-08	-2.49E-08	-2.87E-08	-2.70E-08	-2.01E-08	-1.92E-08	-2.25E-08	-2.41E-08	-2.37E-08	-2.69E-08
-5.93E-09	-9.40E-09	-1.74E-08	-1.89E-08	-1.80E-08	-1.29E-08	-1.88E-08	-1.86E-08	-1.76E-08	-2.13E-08	-2.06E-08	-2.60E-08	-2.38E-08	-2.36E-08	-2.95E-08	-2.76E-08	-2.08E-08	-1.88E-08	-2.22E-08	-2.40E-08	-2.34E-08	-2.59E-08
-4.59E-09	-9.52E-09	-1.72E-08	-1.69E-08	-1.68E-08	-1.76E-08	-1.88E-08	-1.81E-08	-1.94E-08	-2.07E-08	-2.01E-08	-2.57E-08	-2.32E-08	-2.36E-08	-2.89E-08	-2.76E-08	-2.26E-08	-1.99E-08	-2.33E-08	-2.46E-08	-2.31E-08	-2.59E-08
-6.86E-09	-1.14E-08	-1.69E-08	-1.55E-08	-1.64E-08	-1.74E-08	-1.86E-08	-1.80E-08	-1.80E-08	-1.89E-08	-1.98E-08	-2.47E-08	-2.34E-08	-2.32E-08	-2.73E-08	-2.79E-08	-2.23E-08	-2.13E-08	-2.36E-08	-2.40E-08	-2.16E-08	-2.63E-08
-1.10E-08	-1.38E-08	-1.66E-08	-1.60E-08	-1.70E-08	-1.93E-08	-1.85E-08	-1.89E-08	-1.69E-08	-1.82E-08	-1.95E-08	-2.39E-08	-2.27E-08	-2.05E-08	-2.60E-08	-2.74E-08	-2.20E-08	-2.01E-08	-2.35E-08	-2.39E-08	-2.26E-08	-2.60E-08
-1.50E-08	-1.60E-08	-1.73E-08	-1.91E-08	-2.00E-08	-1.98E-08	-1.93E-08	-1.98E-08	-1.63E-08	-1.96E-08	-1.92E-08	-2.43E-08	-2.24E-08	-2.02E-08	-2.44E-08	-2.58E-08	-2.12E-08	-2.03E-08	-2.37E-08	-2.39E-08	-2.36E-08	-2.62E-08
-1.58E-08	-1.71E-08	-1.82E-08	-2.00E-08	-1.98E-08	-1.86E-08	-2.02E-08	-1.89E-08	-1.86E-08	-1.91E-08	-1.98E-08	-2.48E-08	-2.39E-08	-2.20E-08	-2.31E-08	-2.39E-08	-2.07E-08	-2.00E-08	-2.41E-08	-2.52E-08	-2.30E-08	-2.59E-08
-1.56E-08	-1.75E-08	-1.81E-08	-1.91E-08	-1.92E-08	-1.80E-08	-1.90E-08	-1.89E-08	-1.72E-08	-1.92E-08	-2.07E-08	-2.41E-08	-2.47E-08	-2.02E-08	-2.14E-08	-2.25E-08	-2.07E-08	-1.90E-08	-2.39E-08	-2.54E-08	-2.38E-08	-2.53E-08

3.80E-16	4.59E-16	4.53E-16	4.44E-16	5.86E-16	4.99E-16	6.38E-16	4.86E-16	6.13E-16	5.96E-16	7.75E-16	4.80E-16	4.34E-16	5.31E-16	4.03E-16	6.35E-16	8.77E-16	7.63E-16	8.74E-16	7.83E-16	7.31E-16	7.23E-16
3.54E-16	4.26E-16	4.09E-16	3.73E-16	5.53E-16	5.24E-16	5.89E-16	5.12E-16	8.34E-16	6.16E-16	6.68E-16	4.70E-16	4.40E-16	4.78E-16	4.15E-16	6.36E-16	7.79E-16	7.07E-16	9.52E-16	7.34E-16	7.14E-16	7.22E-16
3.28E-16	4.17E-16	3.59E-16	3.60E-16	5.26E-16	5.19E-16	5.82E-16	4.60E-16	9.92E-16	5.87E-16	6.05E-16	4.67E-16	4.27E-16	4.81E-16	3.80E-16	6.42E-16	7.49E-16	7.17E-16	9.94E-16	6.86E-16	6.87E-16	7.20E-16
3.35E-16	4.12E-16	3.58E-16	3.37E-16	4.80E-16	5.27E-16	5.60E-16	4.54E-16	9.14E-16	5.39E-16	5.77E-16	4.22E-16	3.76E-16	4.91E-16	3.81E-16	6.44E-16	7.45E-16	6.85E-16	8.64E-16	6.38E-16	7.20E-16	7.29E-16
3.46E-16	4.10E-16	3.62E-16	3.35E-16	4.64E-16	6.00E-16	5.10E-16	4.82E-16	3.54E-16	5.60E-16	5.32E-16	3.99E-16	3.46E-16	4.32E-16	4.06E-16	5.66E-16	7.50E-16	6.30E-16	8.09E-16	6.14E-16	7.98E-16	6.80E-16
3.81E-16	4.18E-16	3.54E-16	3.66E-16	4.95E-16	6.37E-16	4.79E-16	4.75E-16	4.10E-16	6.19E-16	4.88E-16	4.22E-16	3.08E-16	3.90E-16	4.34E-16	4.93E-16	6.67E-16	6.22E-16	7.22E-16	5.93E-16	8.60E-16	6.99E-16
4.24E-16	4.17E-16	3.27E-16	3.95E-16	4.63E-16	4.86E-16	4.95E-16	4.58E-16	4.49E-16	6.71E-16	4.77E-16	4.34E-16	3.05E-16	4.06E-16	4.04E-16	4.56E-16	6.68E-16	6.81E-16	6.69E-16	5.98E-16	6.23E-16	7.26E-16
4.86E-16	4.02E-16	3.15E-16	3.94E-16	4.04E-16	5.27E-16	5.08E-16	3.78E-16	4.55E-16	6.60E-16	5.06E-16	4.01E-16	2.77E-16	4.34E-16	3.61E-16	4.63E-16	6.85E-16	7.26E-16	5.72E-16	6.00E-16	5.66E-16	7.45E-16
5.25E-16	4.15E-16	3.32E-16	3.89E-16	3.88E-16	5.64E-16	4.49E-16	3.70E-16	5.19E-16	6.18E-16	4.67E-16	4.17E-16	2.76E-16	4.64E-16	3.40E-16	4.32E-16	6.59E-16	6.98E-16	5.86E-16	6.25E-16	5.90E-16	6.75E-16
4.22E-16	4.26E-16	3.59E-16	4.07E-16	4.75E-16	5.66E-16	3.83E-16	4.48E-16	4.40E-16	4.07E-16	4.00E-16	4.65E-16	3.43E-16	5.27E-16	3.41E-16	3.91E-16	6.19E-16	5.86E-16	6.44E-16	5.79E-16	6.19E-16	6.57E-16
3.45E-16	4.06E-16	3.53E-16	4.54E-16	5.91E-16	6.24E-16	4.14E-16	4.94E-16	3.96E-16	4.11E-16	4.08E-16	5.30E-16	4.01E-16	5.74E-16	4.00E-16	3.80E-16	5.89E-16	5.21E-16	6.91E-16	5.49E-16	6.09E-16	6.00E-16
3.39E-16	3.60E-16	3.35E-16	5.00E-16	6.79E-16	6.78E-16	4.39E-16	4.78E-16	4.04E-16	4.34E-16	3.98E-16	6.07E-16	5.48E-16	5.94E-16	4.11E-16	3.98E-16	6.14E-16	5.50E-16	7.17E-16	5.82E-16	5.74E-16	5.67E-16
3.35E-16	3.55E-16	3.54E-16	4.96E-16	6.58E-16	6.79E-16	4.17E-16	4.97E-16	4.11E-16	3.93E-16	3.56E-16	7.25E-16	8.66E-16	8.15E-16	4.50E-16	3.98E-16	5.64E-16	5.99E-16	6.07E-16	6.09E-16	5.14E-16	6.05E-16
3.20E-16	3.76E-16	3.72E-16	3.63E-16	5.09E-16	5.11E-16	3.92E-16	5.36E-16	4.17E-16	3.87E-16	3.05E-16	8.95E-16	1.35E-15	1.22E-15	5.86E-16	3.60E-16	5.31E-16	5.60E-16	5.80E-16	5.87E-16	4.75E-16	6.55E-16
2.93E-16	3.80E-16	3.59E-16	3.04E-16	3.95E-16	3.65E-16	3.34E-16	6.13E-16	3.62E-16	3.37E-16	3.01E-16	9.44E-16	1.47E-15	1.46E-15	7.14E-16	3.65E-16	4.72E-16	4.67E-16	6.13E-16	5.66E-16	4.83E-16	7.25E-16
2.73E-16	3.57E-16	3.33E-16	3.22E-16	3.50E-16	2.98E-16	3.34E-16	4.96E-16	2.99E-16	3.30E-16	2.97E-16	6.64E-16	1.09E-15	1.10E-15	7.00E-16	3.89E-16	4.40E-16	4.83E-16	6.13E-16	5.75E-16	5.17E-16	7.33E-16
2.79E-16	3.26E-16	3.20E-16	2.74E-16	3.26E-16	3.03E-16	3.47E-16	4.62E-16	2.89E-16	3.46E-16	2.85E-16	3.80E-16	6.53E-16	5.75E-16	5.53E-16	4.08E-16	4.10E-16	4.94E-16	6.32E-16	5.54E-16	5.14E-16	6.76E-16
2.83E-16	2.64E-16	3.13E-16	2.41E-16	3.24E-16	2.47E-16	3.43E-16	4.15E-16	3.37E-16	3.66E-16	2.78E-16	2.75E-16	3.82E-16	3.24E-16	2.91E-16	4.29E-16	4.19E-16	4.80E-16	5.43E-16	5.24E-16	5.24E-16	6.21E-16
2.65E-16	2.24E-16	2.92E-16	2.37E-16	3.09E-16	2.13E-16	3.19E-16	4.14E-16	3.97E-16	3.69E-16	2.74E-16	2.70E-16	3.05E-16	2.95E-16	3.20E-16	4.47E-16	3.90E-16	4.51E-16	5.08E-16	5.26E-16	4.97E-16	5.32E-16
2.50E-16	2.15E-16	2.62E-16	2.51E-16	2.88E-16	2.09E-16	3.31E-16	4.47E-16	4.44E-16	3.63E-16	2.75E-16	2.42E-16	2.92E-16	2.98E-16	3.31E-16	4.85E-16	3.82E-16	4.49E-16	4.77E-16	5.06E-16	4.12E-16	4.81E-16
2.31E-16	2.01E-16	2.31E-16	2.31E-16	2.64E-16	2.02E-16	3.97E-16	5.49E-16	5.17E-16	3.74E-16	2.59E-16	2.09E-16	2.73E-16	2.81E-16	3.06E-16	4.79E-16	3.64E-16	4.63E-16	4.81E-16	4.18E-16	4.00E-16	3.77E-16
2.11E-16	1.93E-16	2.28E-16	1.91E-16	2.47E-16	1.94E-16	5.24E-16	8.51E-16	8.15E-16	4.02E-16	2.61E-16	2.13E-16	3.05E-16	2.49E-16	2.93E-16	4.32E-16	3.72E-16	5.16E-16	4.95E-16	3.75E-16	4.09E-16	3.66E-16
1.90E-16	1.82E-16	2.28E-16	1.59E-16	2.33E-16	1.81E-16	7.66E-16	1.31E-15	1.38E-15	4.72E-16	2.83E-16	2.31E-16	2.44E-16	2.08E-16	3.02E-16	4.00E-16	3.22E-16	4.82E-16	4.10E-16	3.99E-16	4.15E-16	3.75E-16
1.65E-16	1.68E-16	1.82E-16	1.55E-16	2.03E-16	1.83E-16	9.42E-16	1.39E-15	1.89E-15	4.35E-16	2.97E-16	2.35E-16	1.67E-16	2.00E-16	3.27E-16	4.08E-16	2.64E-16	4.64E-16	3.26E-16	4.15E-16	3.84E-16	3.79E-16
1.59E-16	1.58E-16	1.40E-16	1.58E-16	2.17E-16	1.84E-16	8.39E-16	9.24E-16	1.76E-15	3.56E-16	2.89E-16	2.33E-16	1.63E-16	1.88E-16	3.03E-16	3.70E-16	2.53E-16	4.67E-16	2.99E-16	3.65E-16	3.56E-16	3.90E-16
1.53E-16	1.48E-16	1.43E-16	1.37E-16	1.99E-16	1.73E-16	5.54E-16	5.59E-16	1.02E-15	2.39E-16	2.53E-16	2.20E-16	1.82E-16	2.09E-16	2.67E-16	3.30E-16	2.51E-16	4.78E-16	2.79E-16	3.35E-16	2.98E-16	3.81E-16
1.39E-16	1.44E-16	1.28E-16	1.10E-16	1.73E-16	1.56E-16	1.35E-16	1.86E-16	1.71E-16	2.47E-16	2.33E-16	2.07E-16	2.15E-16	1.96E-16	2.67E-16	3.34E-16	2.57E-16	4.55E-16	2.71E-16	3.50E-16	2.99E-16	3.73E-16
1.19E-16	1.19E-16	1.13E-16	8.93E-17	1.25E-16	1.41E-16	1.49E-16	1.29E-16	1.82E-16	1.93E-16	2.43E-16	2.14E-16	2.14E-16	2.04E-16	2.87E-16	3.56E-16	2.40E-16	4.56E-16	2.50E-16	3.22E-16	3.44E-16	3.11E-16
1.14E-16	1.09E-16	1.12E-16	8.34E-17	1.11E-16	1.52E-16	1.31E-16	1.13E-16	1.52E-16	1.78E-16	2.12E-16	2.03E-16	1.71E-16	1.88E-16	3.17E-16	3.44E-16	2.35E-16	4.10E-16	2.45E-16	2.76E-16	3.12E-16	3.11E-16
1.18E-16	1.14E-16	1.02E-16	7.67E-17	1.01E-16	1.44E-16	1.29E-16	1.00E-16	1.17E-16	1.80E-16	1.60E-16	1.86E-16	1.64E-16	2.14E-16	2.85E-16	3.09E-16	2.40E-16	3.34E-16	2.33E-16	2.82E-16	2.55E-16	3.16E-16
1.17E-16	1.11E-16	8.92E-17	7.36E-17	9.18E-17	1.40E-16	1.43E-16	1.06E-16	1.27E-16	2.01E-16	1.56E-16	1.85E-16	2.02E-16	2.07E-16	2.18E-16	3.03E-16	2.70E-16	2.56E-16	2.23E-16	2.73E-16	2.65E-16	3.12E-16
1.03E-16	1.00E-16	9.05E-17	8.64E-17	9.68E-17	1.49E-16	1.55E-16	1.17E-16	1.56E-16	1.88E-16	1.91E-16	2.01E-16	1.96E-16	1.59E-16	1.70E-16	3.19E-16	2.98E-16	2.14E-16	2.15E-16	2.32E-16	3.02E-16	3.14E-16
8.92E-17	1.04E-16	8.99E-17	9.74E-17	1.16E-16	1.73E-16	1.39E-16	8.90E-17	1.59E-16	1.52E-16	1.74E-16	2.00E-16	1.50E-16	1.15E-16	1.57E-16	3.06E-16	2.67E-16	2.23E-16	1.82E-16	2.59E-16	2.40E-16	3.18E-16
7.60E-17	9.77E-17	1.01E-16	9.73E-17	1.16E-16	1.78E-16	1.53E-16	6.11E-17	9.08E-17	1.41E-16	1.34E-16	2.02E-16	1.42E-16	1.17E-16	1.47E-16	2.88E-16	2.48E-16	2.24E-16	1.30E-16	2.79E-16	1.87E-16	2.98E-16
6.17E-17	7.79E-17	7.83E-17	7.26E-17	1.03E-16	1.35E-16	1.55E-16	5.78E-17	7.96E-17	1.42E-16	1.15E-16	1.69E-16	1.36E-16	1.41E-16	1.66E-16	2.49E-16	1.89E-16	2.13E-16	1.18E-16	2.40E-16	1.78E-16	2.46E-16
6.10E-17	8.56E-17	8.16E-17	7.84E-17	9.73E-17	1.28E-16	1.44E-16	6.09E-17	8.51E-17	1.44E-16	1.27E-16	1.45E-16	1.07E-16	1.93E-16	1.97E-16	2.24E-16	1.62E-16	2.01E-16	1.15E-16	1.60E-16	1.86E-16	1.88E-16
6.78E-17	9.71E-17	8.60E-17	9.36E-17	9.33E-17	1.24E-16	1.42E-16	7.85E-17	6.89E-17	1.41E-16	1.30E-16	1.44E-16	1.16E-16	2.61E-16	2.49E-16	2.45E-16	1.48E-16	1.90E-16	1.01E-16	1.31E-16	1.72E-16	1.75E-16
6.76E-17	9.96E-17	8.70E-17	8.96E-17	8.50E-17	1.11E-16	1.67E-16	9.05E-17	8.08E-17	1.26E-16	1.13E-16	1.10E-16	9.48E-17	2.61E-16	2.26E-16	2.46E-16	1.27E-16	1.96E-16	9.47E-17	1.25E-16	1.15E-16	1.80E-16
5.04E-17	8.78E-17	7.16E-17	6.43E-17	6.85E-17	1.13E-16	1.88E-16	9.57E-17	8.13E-17	1.26E-16	1.10E-16	8.46E-17	6.78E-17	1.51E-16	1.75E-16	2.36E-16	1.36E-16	1.66E-16	1.22E-16	1.19E-16	8.98E-17	1.67E-16
4.39E-17	7.73E-17	5.34E-17	3.89E-17	5.46E-17	1.00E-16	1.70E-16	1.07E-16	7.96E-17	1.39E-16	1.35E-16	8.51E-17	8.10E-17	1.56E-16	2.17E-16	3.12E-16	1.55E-16	9.89E-17	1.52E-16	1.17E-16	1.02E-16	1.39E-16
4.44E-17	5.67E-17	5.67E-17	5.22E-17	4.53E-17	7.41E-17	1.16E-16	1.05E-16	5.93E-17	1.34E-16	1.40E-16	7.79E-17	1.05E-16	1.97E-16	4.56E-16	5.51E-16	2.04E-16	7.39E-17	1.78E-16	1.27E-16	1.08E-16	1.34E-16
4.39E-17	5.90E-17	6.98E-17	6.34E-17	3.31E-17	5.16E-17	1.26E-16	1.04E-16	5.50E-17	1.61E-16	1.27E-16	7.26E-17	1.77E-16	3.65E-16	8.88E-16	9.33E-16	2.78E-16	6.62E-17	1.37E-16	1.11E-16	8.78E-17	1.69E-16
3.48E-17	5.65E-17	5.85E-17	4.84E-17	2.60E-17	4.86E-17	9.55E-17	7.97E-17	8.46E-17	1.85E-16	1.13E-16	7.11E-17	3.28E-16	6.36E-16	1.28E-15	9.95E-16	2.27E-16	7.61E-17	1.43E-16	9.36E-17	6.92E-17	1.35E-16
2.41E-17	5.51E-17	4.68E-17	2.91E-17	2.13E-17	4.34E-17	4.28E-17	7.56E-17	1.08E-16	1.89E-16	9.24E-17	5.15E-17	4.94E-16	6.39E-16	1.02E-15	6.15E-16	1.43E-16	7.18E-17	1.14E-16	9.25E-17	6.55E-17	9.22E-17
1.82E-17	4.47E-17	3.95E-17	2.50E-17	1.54E-17	3.40E-17	2.91E-17	7.67E-17	8.52E-17	1.14E-16	6.50E-17	4.02E-17	1.11E-16	2.88E-16	5.34E-16	3.84E-16	1.27E-16	6.87E-17	1.13E-16	9.41E-17	6.13E-17	7.33E-17
1.23E-17	2.75E-17	3.42E-17	3.50E-17	1.60E-17	3.39E-17	4.86E-17	2.89E-17	3.23E-17	4.44E-17	3.65E-17	4.27E-17	4.16E-17	1.58E-16	3.08E-16	2.88E-16	1.73E-16	7.15E-17	1.41E-16	8.95E-17	6.55E-17	6.19E-17
1.07E-17	1.82E-17	3.44E-17	4.65E-17	2.51E-17	3.17E-17	6.99E-17	2.61E-17	9.36E-18	2.02E-17	2.56E-17	4.98E-17	4.19E-17	1.23E-16	2.24E-16	2.96E-16	2.15E-16	8.40E-17	1.14E-16	1.16E-16	8.78E-17	5.75E-17
7.31E-18	1.46E-17	3.09E-17	4.10E-17	1.51E-17	2.16E-17	4.89E-17	2.17E-17	1.45E-17	2.24E-17	3.22E-17	5.17E-17	4.27E-17	1.44E-16	2.09E-16	2.65E-16	2.14E-16	7.53E-17	8.93E-17	2.23E-16	1.24E-16	5.86E-17

3.82E-18	8.97E-18	1.87E-17	1.96E-17	4.36E-18	5.90E-18	1.42E-17	2.52E-17	2.42E-17	1.75E-17	3.79E-17	4.14E-17	3.17E-17	1.16E-16	1.78E-16	1.95E-16	1.47E-16	4.96E-17	8.07E-17	3.93E-16	1.51E-16	6.95E-17
9.61E-19	3.97E-18	8.47E-18	8.45E-18	2.48E-18	1.71E-19	1.97E-17	2.92E-17	2.02E-17	1.24E-17	2.76E-17	2.93E-17	1.64E-17	8.37E-17	1.47E-16	1.28E-16	6.24E-17	3.77E-17	7.95E-17	4.65E-16	1.43E-16	6.58E-17
3.18E-19	2.34E-18	6.55E-18	6.49E-18	4.16E-18	3.72E-19	2.32E-17	1.78E-17	5.08E-18	6.10E-18	1.23E-17	1.98E-17	1.23E-17	5.36E-17	1.12E-16	8.44E-17	3.34E-17	2.69E-17	5.33E-17	1.73E-16	8.21E-17	3.94E-17
3.01E-20	1.23E-18	5.59E-18	9.21E-18	6.54E-18	3.62E-18	1.81E-17	2.50E-17	6.23E-18	2.12E-18	1.20E-17	2.02E-17	1.56E-17	6.02E-17	8.62E-17	8.30E-17	1.71E-17	1.36E-17	4.08E-17	4.16E-17	6.17E-17	3.60E-17
8.47E-19	2.62E-19	4.61E-18	8.14E-18	3.67E-18	3.29E-18	7.94E-18	1.06E-17	8.06E-18	2.53E-18	1.66E-17	2.23E-17	1.80E-17	6.21E-17	8.12E-17	9.30E-17	1.78E-17	1.18E-17	3.22E-17	3.40E-17	5.57E-17	2.33E-17
2.75E-18	8.60E-20	1.44E-18	4.83E-18	4.32E-20	3.43E-19	2.56E-18	6.46E-18	1.25E-17	1.73E-18	1.23E-17	1.93E-17	2.17E-17	3.33E-17	8.58E-17	8.14E-17	2.22E-17	1.35E-17	2.45E-17	2.35E-17	3.84E-17	1.93E-17
3.46E-18	1.30E-18	1.41E-19	3.77E-19	1.17E-18	3.13E-18	4.04E-20	2.16E-18	2.07E-18	4.20E-19	7.53E-18	1.44E-17	6.48E-18	3.47E-17	8.12E-17	4.82E-17	2.36E-17	1.14E-17	2.78E-17	2.03E-17	1.92E-17	2.12E-17
4.53E-18	2.50E-18	6.92E-20	4.99E-21	3.36E-18	6.55E-18	3.08E-18	2.48E-18	7.27E-19	4.24E-20	6.07E-18	9.34E-18	5.34E-18	2.27E-17	6.41E-17	4.83E-17	3.15E-17	1.20E-17	2.32E-17	1.83E-17	1.66E-17	1.51E-17
7.10E-18	3.41E-18	2.67E-19	5.01E-20	3.87E-18	7.05E-18	4.60E-18	3.77E-18	2.81E-19	1.66E-19	4.88E-18	7.30E-18	1.49E-17	2.72E-17	6.32E-17	4.28E-17	2.21E-17	8.77E-18	2.37E-17	1.94E-17	1.93E-17	1.41E-18
9.08E-18	4.34E-18	8.19E-19	4.29E-20	1.98E-18	9.16E-18	5.14E-18	8.27E-18	1.16E-18	1.60E-19	2.01E-18	6.59E-18	2.33E-17	2.91E-17	8.48E-17	3.86E-17	9.24E-18	6.91E-18	9.33E-18	3.23E-17	1.99E-17	1.02E-20
9.03E-18	4.47E-18	8.86E-19	9.28E-20	6.06E-19	1.55E-17	5.50E-18	5.72E-18	1.12E-17	1.20E-18	1.27E-18	6.16E-18	1.75E-17	3.76E-17	1.09E-16	2.65E-17	1.06E-17	1.18E-17	5.08E-18	2.64E-17	1.32E-17	6.89E-20
9.81E-18	7.22E-18	3.50E-18	1.42E-20	4.56E-19	1.18E-17	1.13E-18	2.55E-18	8.84E-18	9.74E-18	2.16E-18	6.41E-18	1.66E-17	3.99E-17	8.20E-17	1.70E-17	7.47E-18	6.66E-18	1.84E-18	7.76E-18	6.10E-18	1.42E-19
8.60E-18	1.19E-17	6.44E-18	1.04E-18	1.23E-18	1.97E-18	4.22E-19	5.59E-19	6.63E-18	1.05E-17	3.39E-18	5.72E-18	2.31E-17	2.29E-17	1.05E-16	2.10E-17	2.39E-18	9.99E-19	1.15E-18	6.20E-18	4.43E-18	1.84E-19
7.97E-18	1.11E-17	4.46E-18	9.80E-18	1.86E-17	1.05E-18	1.61E-21	5.85E-18	5.45E-18	5.12E-18	1.88E-18	8.06E-18	3.66E-17	2.75E-17	1.33E-16	2.96E-17	2.34E-18	2.66E-19	4.07E-19	1.86E-18	2.38E-18	2.03E-19
1.30E-17	7.75E-18	6.79E-19	3.15E-17	8.50E-17	2.07E-17	7.42E-19	8.94E-18	7.57E-18	6.92E-18	5.21E-19	1.04E-17	4.68E-17	3.26E-17	1.02E-16	2.58E-17	4.18E-18	9.73E-19	2.75E-20	8.70E-19	5.77E-18	6.17E-19
1.68E-17	8.63E-18	3.33E-19	7.70E-17	2.00E-16	6.81E-17	2.76E-18	1.51E-17	1.65E-17	1.58E-17	8.79E-19	1.50E-17	4.99E-17	3.83E-17	8.81E-17	1.51E-17	4.71E-18	2.11E-18	1.14E-19	2.15E-20	9.54E-18	6.46E-19
1.47E-17	7.92E-18	3.49E-18	1.01E-16	2.03E-16	9.35E-17	3.71E-18	1.30E-17	1.44E-17	2.79E-17	3.95E-19	2.01E-17	5.03E-17	2.80E-17	8.67E-17	9.27E-18	1.31E-18	5.35E-18	2.54E-19	1.76E-18	3.34E-18	3.26E-18
1.65E-17	5.21E-18	1.98E-18	4.80E-17	7.97E-17	3.60E-17	5.33E-18	1.69E-17	1.01E-17	2.73E-17	6.43E-21	2.83E-17	7.02E-17	3.09E-17	1.12E-16	1.00E-17	1.09E-17	8.97E-19	5.78E-18	4.20E-18	1.24E-18	1.33E-17
2.13E-17	2.67E-18	7.67E-19	6.33E-18	2.68E-18	8.33E-19	5.25E-18	2.28E-17	1.70E-17	2.55E-17	1.37E-18	4.06E-17	1.05E-16	5.15E-17	1.16E-16	7.25E-18	1.39E-17	9.62E-22	7.08E-18	7.69E-18	1.75E-18	1.78E-17
1.94E-17	2.12E-18	6.08E-18	1.30E-18	1.28E-17	3.36E-18	1.81E-17	3.96E-17	3.24E-17	3.55E-17	4.42E-18	6.15E-17	1.33E-16	8.53E-17	1.15E-16	5.31E-18	6.63E-18	2.78E-19	6.44E-18	9.83E-18	4.66E-18	2.20E-17
1.40E-17	8.38E-18	1.22E-18	1.18E-17	2.41E-17	1.92E-18	6.86E-18	5.04E-17	3.27E-17	4.23E-17	5.11E-18	8.70E-17	1.57E-16	1.04E-16	1.82E-16	1.12E-17	7.37E-18	3.93E-19	8.22E-18	1.10E-17	1.40E-17	2.45E-17
2.12E-17	3.23E-17	3.22E-18	4.38E-17	1.71E-17	3.15E-18	3.12E-19	2.53E-17	2.97E-17	4.52E-17	2.85E-18	1.15E-16	2.82E-16	1.78E-16	2.77E-16	3.25E-17	2.01E-17	1.18E-17	1.17E-17	1.34E-17	1.87E-17	3.99E-17
3.46E-17	4.28E-17	1.14E-17	2.46E-17	1.11E-17	3.95E-17	3.68E-18	9.11E-18	2.12E-17	4.82E-17	7.95E-19	2.15E-16	6.04E-16	4.11E-16	4.89E-16	6.10E-17	2.28E-17	2.23E-17	2.83E-17	1.19E-17	4.76E-17	4.67E-17
3.36E-17	5.65E-17	5.15E-17	3.55E-17	1.56E-17	6.40E-17	3.92E-17	2.69E-19	3.17E-17	5.03E-17	2.09E-19	4.29E-16	1.02E-15	8.31E-16	8.11E-16	1.15E-16	2.18E-17	2.42E-17	5.60E-17	1.44E-17	6.28E-17	5.99E-17
3.47E-17	6.89E-17	6.30E-17	8.65E-17	4.53E-17	4.76E-18	5.68E-17	5.12E-18	5.30E-17	4.98E-17	3.37E-18	5.60E-16	1.07E-15	9.27E-16	9.33E-16	1.52E-16	2.80E-17	2.98E-17	6.11E-17	3.34E-17	4.97E-17	7.00E-17
4.98E-17	8.75E-17	6.64E-17	9.72E-17	5.81E-17	2.89E-17	6.07E-17	2.24E-17	7.67E-17	5.24E-17	2.32E-18	3.45E-16	7.10E-16	6.59E-16	5.83E-16	6.07E-17	4.11E-17	4.61E-17	7.40E-17	3.75E-17	4.38E-17	7.90E-17
6.17E-17	1.05E-16	8.56E-17	1.17E-16	6.40E-17	2.02E-17	6.57E-17	8.01E-17	6.15E-17	6.37E-17	4.14E-18	5.51E-17	2.53E-16	2.39E-16	1.26E-16	3.52E-18	4.71E-17	7.33E-17	9.59E-17	4.05E-17	4.39E-17	7.09E-17
6.51E-17	1.12E-16	9.20E-17	1.32E-16	5.72E-17	4.49E-18	9.22E-17	9.65E-17	6.51E-17	7.27E-17	4.13E-17	9.95E-18	3.35E-18	8.79E-19	4.37E-18	8.33E-17	5.03E-17	8.48E-17	1.01E-16	3.72E-17	4.00E-17	4.86E-17
7.04E-17	1.21E-16	1.06E-16	1.39E-16	6.02E-17	5.06E-18	9.61E-17	7.67E-17	8.96E-17	6.91E-17	1.02E-16	1.06E-16	7.90E-17	1.03E-16	9.80E-17	1.28E-16	4.99E-17	9.34E-17	9.75E-17	3.01E-17	2.06E-17	1.82E-17
9.46E-17	1.39E-16	1.20E-16	1.53E-16	8.35E-17	6.25E-17	1.15E-16	9.86E-17	9.62E-17	6.02E-17	9.35E-17	1.12E-16	1.64E-16	1.97E-16	1.90E-16	1.09E-16	5.80E-17	1.22E-16	8.47E-17	1.62E-17	1.91E-19	9.43E-19
1.16E-16	1.47E-16	1.34E-16	1.77E-16	1.09E-16	6.74E-17	9.87E-17	1.15E-16	9.68E-17	5.77E-17	6.35E-17	1.02E-16	1.80E-16	2.25E-16	2.58E-16	1.59E-16	8.46E-17	1.61E-16	7.81E-17	4.24E-18	4.94E-17	1.94E-17
1.33E-16	1.60E-16	1.60E-16	2.03E-16	1.31E-16	8.24E-17	1.09E-16	1.25E-16	8.37E-17	8.36E-17	4.34E-17	9.08E-17	2.31E-16	2.67E-16	3.16E-16	2.41E-16	1.56E-16	1.99E-16	8.14E-17	3.69E-18	9.69E-17	7.30E-17
1.61E-16	1.83E-16	1.91E-16	2.14E-16	1.49E-16	1.04E-16	1.34E-16	1.23E-16	7.74E-17	8.85E-17	3.51E-17	7.10E-17	2.40E-16	2.95E-16	3.54E-16	2.54E-16	1.97E-16	2.28E-16	1.23E-16	7.75E-17	4.19E-18	1.48E-17
1.99E-16	2.11E-16	1.98E-16	2.16E-16	1.59E-16	1.09E-16	1.11E-16	1.03E-16	1.02E-16	6.47E-17	3.92E-17	8.76E-17	2.41E-16	3.39E-16	3.42E-16	2.35E-16	2.84E-16	2.79E-16	1.44E-16	1.61E-16	2.30E-16	1.70E-16
2.13E-16	2.40E-16	1.96E-16	2.15E-16	1.81E-16	1.27E-16	1.03E-16	1.29E-16	1.66E-16	2.91E-17	3.54E-17	8.48E-17	2.46E-16	3.45E-16	3.67E-16	2.55E-16	3.01E-16	2.66E-16	1.43E-16	1.94E-16	2.50E-16	2.01E-16
2.05E-16	2.69E-16	2.07E-16	2.31E-16	2.26E-16	1.42E-16	1.39E-16	1.99E-16	1.80E-16	2.35E-17	1.02E-17	2.77E-17	1.52E-16	2.85E-16	5.03E-16	3.10E-16	1.87E-16	2.07E-16	1.32E-16	2.36E-16	2.74E-16	2.18E-16
2.09E-16	3.02E-16	2.68E-16	2.61E-16	2.69E-16	1.72E-16	1.81E-16	1.88E-16	1.55E-16	1.13E-17	1.69E-18	3.34E-18	5.11E-17	1.95E-16	5.44E-16	3.14E-16	1.15E-16	1.92E-16	1.40E-16	2.39E-16	2.80E-16	2.21E-16
2.20E-16	3.08E-16	3.08E-16	3.14E-16	2.79E-16	2.04E-16	2.13E-16	1.54E-16	1.04E-16	6.64E-18	5.01E-17	5.79E-17	4.89E-19	1.42E-16	5.60E-16	3.15E-16	1.13E-16	1.82E-16	1.94E-16	2.12E-16	2.71E-16	2.30E-16
2.30E-16	2.91E-16	3.08E-16	3.50E-16	2.80E-16	1.93E-16	1.92E-16	1.33E-16	7.58E-17	3.82E-17	8.49E-17	5.99E-17	2.16E-18	8.98E-17	5.18E-16	3.15E-16	1.55E-16	2.24E-16	2.09E-16	1.77E-16	2.53E-16	2.07E-16
2.08E-16	2.73E-16	2.85E-16	3.38E-16	2.89E-16	1.86E-16	2.02E-16	1.70E-16	7.23E-17	1.65E-17	4.77E-17	8.23E-18	2.02E-18	1.16E-16	5.17E-16	3.26E-16	1.60E-16	2.30E-16	2.26E-16	2.15E-16	2.42E-16	1.92E-16
1.77E-16	2.66E-16	2.83E-16	3.28E-16	2.75E-16	1.70E-16	2.21E-16	1.74E-16	8.16E-17	7.61E-18	5.68E-19	2.48E-17	5.40E-17	2.47E-16	5.52E-16	3.47E-16	1.82E-16	2.39E-16	2.12E-16	2.26E-16	1.83E-16	1.73E-16
1.70E-16	2.63E-16	2.84E-16	3.45E-16	2.53E-16	1.90E-16	2.21E-16	1.58E-16	1.37E-16	9.27E-17	4.38E-17	1.62E-16	1.92E-16	3.88E-16	5.56E-16	3.84E-16	2.07E-16	2.24E-16	1.92E-16	1.78E-16	1.47E-16	1.65E-16
1.95E-16	2.63E-16	2.70E-16	3.65E-16	2.68E-16	2.20E-16	1.98E-16	1.57E-16	2.44E-16	1.89E-16	1.40E-16	3.00E-16	4.09E-16	4.42E-16	5.30E-16	4.20E-16	2.09E-16	2.10E-16	1.82E-16	1.74E-16	1.59E-16	1.77E-16
2.21E-16	2.70E-16	2.61E-16	3.69E-16	3.04E-16	2.42E-16	1.80E-16	1.41E-16	2.21E-16	1.80E-16	1.88E-16	3.20E-16	3.88E-16	4.68E-16	4.95E-16	4.01E-16	1.86E-16	1.74E-16	2.19E-16	1.96E-16	1.95E-16	1.87E-16
2.05E-16	2.63E-16	2.89E-16	3.46E-16	3.14E-16	2.31E-16	2.28E-16	1.49E-16	1.82E-16	1.47E-16	1.78E-16	3.30E-16	3.73E-16	4.17E-16	4.75E-16	3.88E-16	1.67E-16	2.00E-16	2.40E-16	2.21E-16	2.30E-16	1.67E-16
1.82E-16	2.68E-16	3.77E-16	3.24E-16	2.82E-16	2.15E-16	2.51E-16	1.80E-16	1.74E-16	1.17E-16	1.64E-16	3.12E-16	3.68E-16	3.90E-16	5.14E-16	4.07E-16	1.85E-16	2.49E-16	2.59E-16	2.25E-16	2.78E-16	1.51E-16
1.96E-16	3.12E-16	4.26E-16	3.23E-16	2.69E-16	2.22E-16	2.43E-16	1.66E-16	1.93E-16	6.63E-17	1.45E-16	3.09E-16	3.54E-16	4.16E-16	5.61E-16	4.41E-16	2.27E-16	2.69E-16	2.49E-16	2.57E-16	3.68E-16	1.83E-16
2.09E-16	3.22E-16	4.28E-16	3.16E-16	2.89E-16	3.05E-16	1.81E-16	1.37E-16	1.59E-16	3.37E-17	1.31E-16	3.84E-16	3.92E-16	4.90E-16	5.91E-16	4.92E-16	2.48E-16	3.13E-16	2.81E-16	3.09E-16	4.00E-16	2.33E-16
2.36E-16	3.06E-16	4.12E-16	2.90E-16	2.92E-16	2.87E-16	1.61E-16	1.87E-16	1.30E-16	2.86E-17	1.65E-16	4.77E-16	4.72E-16	5.95E-16	5.46E-16	5.07E-16	2.43E-16	3.43E-16	3.14E-16	3.80E-16	4.19E-16	2.87E-16
2.70E-16	2.76E-16	4.01E-16	2.66E-16	2.90E-16	2.67E-16	1.47E-16	1.64E-16	1.56E-16	3.99E-17	2.42E-16	4.85E-16	5.22E-16	5.89E-16	5.20E-16	5.42E-16	2.94E-16	4.15E-16	3.34E-16	4.03E-16	3.93E-16	3.14E-16
2.87E-16	2.65E-16	3.99E-16	2.57E-16	2.90E-16	2.71E-16	1.30E-16	1.48E-16	2.52E-16	1.38E-16	3.05E-16	4.57E-16	4.98E-16	5.85E-16	5.58E-16	5.81E-16	3.48E-16	4.25E-16	2.96E-16	4.04E-16	3.49E-16	2.71E-16
3.46E-16	2.70E-16	4.02E-16	2.78E-16	3.12E-16	3.35E-16	1.24E-16	1.16E-16	3.22E-16	1.86E-16	2.64E-16	4.58E-16	4.88E-16	6.05E-16	6.12E-16	6.12E-16	3.47E-16	4.18E-16	2.47E-16	3.94E-16	3.31E-16	3.46E-16
3.90E-16	3.18E-16	3.66E-16	2.68E-16	3.30E-16	4.17E-16	3.20E-16	1.03E-16	2.80E-16	1.52E-16	2.50E-16	4.51E-16	4.87E-16	6.35E-16	6.07E-16	6.05E-16	3.55E-16	3.74E-16	2.45E-16	3.60E-16	3.35E-16	3.46E-16
3.82E-16	3.25E-16	3.10E-16	2.49E-16	3.84E-16	4.99E-16	3.67E-16	1.92E-16	2.68E-16	2.38E-16	2.84E-16	4.02E-16	4.47E-16	6.72E-16	5.89E-16	5.86E-16	3.30E-16	3.65E-16	2.63E-16	3.50E-16	3.24E-16	3.47E-16
3.61E-16	3.34E-16	3.43E-16	2.48E-16	4.14E-16	5.36E-16	4.12E-16	2.42E-16	2.02E-16	2.68E-16	2.78E-16	3.12E-16	3.47E-16	6.34E-16	5.60E-16	5.45E-16	3.29E-16	3.97E-16	3.00E-16	3.55E-16	2.83E-16	3.39E-16
3.66E-16	3.38E-16	3.91E-16	2.71E-16	4.33E-16	5.18E-16	4.24E-16	2.53E-16	1.81E-16	2.86E-16	2.11E-16	2.44E-16	2.22E-16	6.39E-16	5.50E-16	5.24E-16	3.54E-16	3.72E-16	3.67E-16	3.63E-16	2.72E-16	3.04E-16
3.74E-16	3.50E-16	3.83E-16	3.29E-16	4.68E-16	5.01E-16	3.68E-16	2.19E-16	2.52E-16	3.41E-16	2.33E-16	3.10E-16	1.91E-16	6.48E-16	5.27E-16	5.09E-16	4.17E-16	3.52E-16	4.40E-16	4.03E-16	3.14E-16	3.04E-16
3.64E-16	3.42E-16	3.32E-16	4.17E-16	5.15E-16	4.65E-16	3.50E-16	3.40E-16	3.50E-16	3.50E-16	4.05E-16	4.23E-16	3.30E-16	6.12E-16	5.31E-16	4.73E-16	4.19E-16	3.63E-16	4.58E-16	4.38E-16	3.09E-16	3.31E-16
3.33E-16	3.18E-16	3.14E-16	4.85E-16	4.85E-16	4.21E-16	3.59E-16	2.85E-16	3.94E-16	3.53E-16	3.86E-16	4.21E-16	5.19E-16	6.74E-16	5.85E-16	4.72E-16	4.51E-16	4.16E-16	4.82E-16	4.14E-16	3.54E-16	3.88E-16
3.14E-16	3.02E-16	3.13E-16	4.87E-16	4.34E-16	4.15E-16	3.43E-16	3.06E-16	4.10E-16	3.96E-16	3.87E-16	4.57E-16	5.23E-16	6.92E-16	5.93E-16	5.06E-16	4.40E-16	4.61E-16	4.62E-16	3.63E-16	3.49E-16	3.98E-16
2.83E-16	2.85E-16	3.27E-16	4.63E-16	4.09E-16	4.34E-16	3.10E-16	4.08E-16	4.14E-16	4.05E-16	3.95E-16	5.37E-16	6.24E-16	7.49E-16	5.89E-16	5.23E-16	4.04E-16	4.73E-16	4.61E-16	4.33E-16	3.38E-16	4.04E-16
2.62E-16	2.58E-16	3.42E-16	4.64E-16	4.30E-16	3.88E-16	4.03E-16	4.45E-16	4.88E-16	4.12E-16	4.21E-16	5.56E-16	6.65E-16	7.34E-16	6.41E-16	5.08E-16	3.82E-16	4.10E-16	4.87E-16	4.88E-16	3.34E-16	4.71E-16
2.47E-16	2.34E-16	3.27E-16	4.88E-16	4.29E-16	3.42E-16	4.00E-16	4.57E-16	5.08E-16	3.70E-16	4.14E-16	5.59E-16	7.11E-16	7.69E-16	6.76E-16	5.27E-16	3.67E-16	3.54E-16	4.63E-16	4.91E-16	3.70E-16	5.22E-16

2.27E-16	2.16E-16	2.83E-16	4.85E-16	3.98E-16	3.95E-16	3.71E-16	4.04E-16	4.76E-16	3.13E-16	4.09E-16	5.89E-16	7.12E-16	8.06E-16	7.37E-16	5.75E-16	3.69E-16	3.25E-16	4.07E-16	4.40E-16	4.94E-16	5.79E-16
2.16E-16	1.99E-16	2.51E-16	4.76E-16	4.00E-16	4.55E-16	3.82E-16	4.23E-16	5.05E-16	2.51E-16	4.10E-16	5.59E-16	6.58E-16	8.11E-16	7.57E-16	5.92E-16	4.29E-16	3.08E-16	3.75E-16	4.64E-16	5.27E-16	5.71E-16
2.28E-16	2.14E-16	3.17E-16	4.76E-16	4.45E-16	4.38E-16	4.36E-16	4.26E-16	5.12E-16	3.21E-16	4.20E-16	5.20E-16	6.11E-16	7.90E-16	7.38E-16	6.36E-16	4.74E-16	2.39E-16	3.35E-16	5.74E-16	5.08E-16	5.44E-16
2.29E-16	2.53E-16	3.69E-16	4.54E-16	4.09E-16	3.88E-16	4.51E-16	4.76E-16	4.89E-16	3.47E-16	4.57E-16	5.55E-16	6.70E-16	7.26E-16	6.74E-16	6.64E-16	4.66E-16	1.47E-16	2.19E-16	6.78E-16	5.29E-16	5.21E-16
2.24E-16	2.69E-16	3.58E-16	3.86E-16	3.17E-16	3.67E-16	4.82E-16	4.57E-16	4.56E-16	3.18E-16	4.79E-16	6.29E-16	7.04E-16	7.06E-16	6.46E-16	6.90E-16	4.98E-16	1.20E-16	1.08E-16	6.49E-16	5.50E-16	5.41E-16
2.22E-16	2.66E-16	3.42E-16	3.45E-16	2.90E-16	3.62E-16	4.47E-16	4.04E-16	4.14E-16	3.13E-16	4.70E-16	6.20E-16	7.22E-16	7.21E-16	7.01E-16	7.04E-16	5.71E-16	2.70E-16	8.97E-17	5.88E-16	5.28E-16	5.41E-16
2.26E-16	2.38E-16	3.39E-16	4.16E-16	3.59E-16	3.05E-16	3.93E-16	3.35E-16	3.47E-16	4.43E-16	4.78E-16	6.09E-16	6.99E-16	7.22E-16	7.76E-16	6.79E-16	5.76E-16	4.55E-16	2.84E-16	5.96E-16	4.62E-16	5.78E-16
1.93E-16	2.10E-16	3.58E-16	4.54E-16	3.91E-16	2.42E-16	3.37E-16	2.87E-16	3.27E-16	4.15E-16	4.87E-16	6.22E-16	6.55E-16	6.96E-16	7.68E-16	6.60E-16	4.99E-16	4.43E-16	5.27E-16	5.84E-16	4.71E-16	6.12E-16
1.45E-16	1.72E-16	3.17E-16	4.28E-16	3.65E-16	1.78E-16	2.93E-16	2.96E-16	3.23E-16	4.06E-16	4.69E-16	6.55E-16	6.34E-16	6.47E-16	7.46E-16	6.98E-16	4.24E-16	4.04E-16	5.17E-16	5.76E-16	5.47E-16	7.31E-16
9.22E-17	1.26E-16	3.08E-16	4.01E-16	3.58E-16	1.38E-16	3.06E-16	3.16E-16	3.29E-16	4.34E-16	4.58E-16	6.72E-16	6.11E-16	6.19E-16	8.23E-16	7.27E-16	4.04E-16	3.70E-16	5.06E-16	5.80E-16	5.61E-16	7.26E-16
3.52E-17	8.84E-17	3.02E-16	3.59E-16	3.24E-16	1.66E-16	3.54E-16	3.45E-16	3.10E-16	4.52E-16	4.26E-16	6.77E-16	5.66E-16	5.57E-16	8.73E-16	7.62E-16	4.32E-16	3.55E-16	4.94E-16	5.76E-16	5.49E-16	6.69E-16
2.11E-17	9.06E-17	2.97E-16	2.86E-16	2.82E-16	3.09E-16	3.52E-16	3.26E-16	3.74E-16	4.30E-16	4.05E-16	6.59E-16	5.37E-16	5.55E-16	8.35E-16	7.60E-16	5.13E-16	3.97E-16	5.43E-16	6.03E-16	5.32E-16	6.73E-16
4.70E-17	1.31E-16	2.84E-16	2.41E-16	2.69E-16	3.02E-16	3.47E-16	3.24E-16	3.23E-16	3.56E-16	3.93E-16	6.08E-16	5.47E-16	5.40E-16	7.45E-16	7.80E-16	4.97E-16	4.52E-16	5.55E-16	5.76E-16	4.67E-16	6.93E-16
1.21E-16	1.91E-16	2.76E-16	2.56E-16	2.88E-16	3.71E-16	3.42E-16	3.57E-16	2.84E-16	3.32E-16	3.81E-16	5.70E-16	5.15E-16	4.20E-16	6.77E-16	7.49E-16	4.82E-16	4.03E-16	5.54E-16	5.69E-16	5.13E-16	6.78E-16
2.24E-16	2.57E-16	3.00E-16	3.65E-16	3.99E-16	3.93E-16	3.74E-16	3.93E-16	2.67E-16	3.82E-16	3.70E-16	5.91E-16	5.01E-16	4.08E-16	5.95E-16	6.67E-16	4.50E-16	4.12E-16	5.64E-16	5.71E-16	5.55E-16	6.84E-16
2.49E-16	2.92E-16	3.32E-16	4.01E-16	3.92E-16	3.45E-16	4.07E-16	3.56E-16	3.45E-16	3.65E-16	3.91E-16	6.14E-16	5.73E-16	4.83E-16	5.35E-16	5.69E-16	4.28E-16	3.99E-16	5.80E-16	6.36E-16	5.29E-16	6.69E-16
2.43E-16	3.06E-16	3.26E-16	3.63E-16	3.68E-16	3.24E-16	3.60E-16	3.58E-16	2.95E-16	3.68E-16	4.27E-16	5.78E-16	6.10E-16	4.07E-16	4.59E-16	5.08E-16	4.30E-16	3.61E-16	5.70E-16	6.44E-16	5.65E-16	6.40E-16
2.14E-14	2.38E-14	2.60E-14	2.80E-14	2.96E-14	2.83E-14	3.05E-14	3.04E-14	3.35E-14	2.73E-14	2.70E-14	3.61E-14	4.21E-14	4.89E-14	5.35E-14	4.74E-14	3.60E-14	3.55E-14	3.66E-14	3.92E-14	3.69E-14	3.98E-14

2.97E-08	2.62E-08	2.74E-08	2.45E-08	2.32E-08	2.04E-08	2.07E-08	1.48E-08	1.26E-08	6.36E-09	3.82E-09	7.53E-10	-3.88E-09	-2.70E-09	-2.27E-09	-3.01E-09	-3.83E-09	2.60E-11	1.37E-09	6.72E-09	6.82E-09	1.34E-09
2.69E-08	2.60E-08	3.00E-08	2.65E-08	2.37E-08	2.05E-08	1.89E-08	1.53E-08	1.11E-08	6.24E-09	2.73E-09	8.94E-10	-3.93E-09	-4.23E-09	-2.46E-09	-2.58E-09	-3.27E-09	-7.26E-10	1.76E-09	3.47E-09	4.55E-09	2.03E-09
2.65E-08	2.70E-08	3.02E-08	2.89E-08	2.41E-08	2.12E-08	1.98E-08	1.59E-08	1.09E-08	5.11E-09	2.54E-09	1.99E-10	-4.09E-09	-4.01E-09	-5.31E-09	-2.51E-09	-3.44E-09	-6.58E-10	-1.06E-09	-5.15E-11	1.98E-09	2.56E-09
2.64E-08	2.76E-08	3.37E-08	3.31E-08	2.42E-08	2.09E-08	2.27E-08	1.68E-08	1.13E-08	3.53E-09	1.05E-09	-1.41E-09	-5.05E-09	-4.53E-09	-6.39E-09	-2.64E-09	-4.70E-09	-1.00E-09	-4.00E-09	-2.12E-09	1.50E-09	2.03E-09
2.62E-08	2.87E-08	3.98E-08	3.94E-08	2.35E-08	2.08E-08	2.45E-08	1.60E-08	1.03E-08	3.93E-09	-8.07E-10	-2.52E-09	-5.09E-09	-5.66E-09	-6.60E-09	-3.74E-09	-5.33E-09	-2.14E-09	-4.20E-09	-3.22E-09	2.55E-09	1.62E-09
2.66E-08	2.96E-08	4.56E-08	4.46E-08	2.31E-08	2.02E-08	2.55E-08	1.55E-08	8.53E-09	3.92E-09	-2.17E-11	-2.91E-09	-4.94E-09	-7.67E-09	-8.54E-09	-3.84E-09	-5.53E-09	-3.37E-09	-5.35E-09	-3.42E-09	2.64E-09	2.18E-09
2.66E-08	2.83E-08	4.59E-08	4.40E-08	2.38E-08	2.03E-08	1.95E-08	1.27E-08	7.72E-09	3.05E-09	4.95E-10	-3.15E-09	-4.94E-09	-8.37E-09	-9.94E-09	-3.58E-09	-6.10E-09	-4.01E-09	-5.36E-09	-3.73E-09	5.31E-10	3.46E-09
2.74E-08	2.49E-08	3.34E-08	2.88E-08	2.35E-08	2.06E-08	1.51E-08	1.11E-08	7.48E-09	1.92E-09	1.43E-09	-3.27E-09	-5.50E-09	-8.90E-09	-9.34E-09	-4.38E-09	-7.44E-09	-4.52E-09	-5.46E-09	-4.61E-09	-1.41E-09	5.49E-09
2.76E-08	2.43E-08	2.19E-08	2.32E-08	2.25E-08	2.08E-08	1.41E-08	1.23E-08	7.57E-09	2.16E-09	1.64E-09	-3.64E-09	-6.85E-09	-1.00E-08	-8.43E-09	-7.76E-09	-9.63E-09	-5.22E-09	-7.14E-09	-5.39E-09	-2.23E-09	8.21E-09
2.80E-08	2.50E-08	2.19E-08	2.20E-08	2.07E-08	2.06E-08	1.31E-08	1.27E-08	7.41E-09	3.07E-09	1.40E-09	-3.71E-09	-7.66E-09	-1.05E-08	-9.24E-09	-8.95E-09	-9.97E-09	-5.65E-09	-7.49E-09	-5.01E-09	-2.69E-09	1.11E-08
2.82E-08	2.55E-08	2.17E-08	2.19E-08	2.07E-08	1.99E-08	1.30E-08	1.19E-08	7.45E-09	3.69E-09	2.89E-09	-3.59E-09	-7.57E-09	-1.11E-08	-9.39E-09	-8.33E-09	-1.02E-08	-6.21E-09	-8.52E-09	-4.75E-09	-3.79E-09	9.04E-09
2.83E-08	2.59E-08	2.06E-08	2.28E-08	2.11E-08	1.99E-08	1.26E-08	1.26E-08	7.65E-09	4.67E-09	6.41E-09	-4.20E-09	-7.16E-09	-1.10E-08	-8.68E-09	-8.12E-09	-1.12E-08	-6.28E-09	-1.02E-08	-7.63E-09	-4.79E-09	1.51E-09
2.69E-08	2.52E-08	1.98E-08	2.19E-08	2.04E-08	1.95E-08	1.27E-08	1.30E-08	7.18E-09	5.95E-09	1.05E-08	-6.19E-09	-6.92E-09	-1.12E-08	-9.07E-09	-9.40E-09	-1.18E-08	-6.64E-09	-1.01E-08	-8.81E-09	-6.77E-09	4.66E-11
2.67E-08	2.62E-08	1.98E-08	2.10E-08	1.83E-08	1.89E-08	1.28E-08	1.13E-08	7.57E-09	5.46E-09	9.12E-09	-7.35E-09	-7.14E-09	-1.09E-08	-9.05E-09	-1.01E-08	-1.19E-08	-7.08E-09	-9.27E-09	-8.38E-09	-8.72E-09	-6.37E-10
2.55E-08	2.67E-08	2.06E-08	2.06E-08	1.74E-08	1.83E-08	1.24E-08	9.90E-09	8.24E-09	2.90E-09	-4.83E-09	-7.14E-09	-6.63E-09	-1.13E-08	-1.06E-08	-1.09E-08	-1.06E-08	-6.26E-09	-9.63E-09	-7.21E-09	-1.01E-08	-4.12E-09
2.50E-08	2.40E-08	2.09E-08	2.12E-08	1.69E-08	1.79E-08	1.29E-08	1.01E-08	7.81E-09	1.58E-09	-5.02E-09	-6.29E-09	-8.44E-09	-1.12E-08	-1.22E-08	-1.08E-08	-7.78E-09	-3.06E-09	-8.06E-09	-8.32E-09	-9.77E-09	-7.25E-09
2.44E-08	2.28E-08	2.04E-08	1.96E-08	1.69E-08	1.83E-08	1.35E-08	1.08E-08	6.66E-09	1.39E-09	-4.84E-09	-6.50E-09	-9.89E-09	-1.14E-08	-1.21E-08	-9.69E-09	-3.82E-09	1.91E-09	-5.85E-09	-9.40E-09	-9.66E-09	-7.51E-09
2.39E-08	2.31E-08	1.95E-08	1.88E-08	1.68E-08	1.82E-08	1.50E-08	1.02E-08	4.80E-09	9.67E-10	-4.94E-09	-8.65E-09	-9.78E-09	-1.13E-08	-1.18E-08	-7.66E-09	1.18E-09	7.29E-09	-3.36E-09	-9.78E-09	-1.14E-08	-6.71E-09
2.35E-08	2.33E-08	1.94E-08	1.82E-08	1.67E-08	1.88E-08	1.74E-08	1.04E-08	5.17E-09	1.99E-10	-5.12E-09	-9.47E-09	-9.62E-09	-1.01E-08	-1.14E-08	-6.45E-09	2.74E-09	8.68E-09	-3.63E-09	-9.68E-09	-1.21E-08	-7.09E-09
2.35E-08	2.23E-08	1.90E-08	1.82E-08	1.68E-08	1.85E-08	2.18E-08	1.14E-08	5.73E-09	1.01E-09	-5.52E-09	-9.68E-09	-1.11E-08	-9.34E-09	-1.09E-08	-8.99E-09	-1.20E-09	4.64E-09	-7.37E-09	-9.07E-09	-1.18E-08	-8.63E-09
2.36E-08	1.98E-08	1.84E-08	1.78E-08	1.70E-08	1.70E-08	2.42E-08	1.08E-08	4.03E-09	-2.67E-11	-6.19E-09	-1.03E-08	-1.15E-08	-9.45E-09	-1.06E-08	-1.30E-08	-7.66E-09	-2.11E-09	-1.16E-08	-9.99E-09	-1.07E-08	-9.84E-09
2.29E-08	1.94E-08	1.76E-08	1.75E-08	1.64E-08	1.59E-08	1.56E-08	7.40E-09	3.86E-09	-1.12E-09	-5.45E-09	-1.12E-08	-1.08E-08	-1.17E-08	-1.28E-08	-1.46E-08	-1.43E-08	-1.60E-08	-1.34E-08	-1.18E-08	-9.68E-09	-9.37E-09
2.02E-08	1.95E-08	1.73E-08	1.67E-08	1.42E-08	1.34E-08	1.05E-08	6.34E-09	4.16E-09	-1.21E-09	-5.27E-09	-1.13E-08	-9.64E-09	-1.31E-08	-1.26E-08	-1.27E-08	-1.74E-08	-1.74E-08	-1.37E-08	-1.14E-08	-9.17E-09	-1.02E-08
1.93E-08	1.87E-08	1.66E-08	1.69E-08	1.38E-08	1.28E-08	1.02E-08	7.17E-09	4.08E-09	-9.28E-10	-5.58E-09	-9.77E-09	-1.01E-08	-1.37E-08	-1.08E-08	-1.15E-08	-1.61E-08	-1.75E-08	-1.40E-08	-9.98E-09	-1.03E-08	-1.11E-08
1.87E-08	1.71E-08	1.58E-08	1.91E-08	1.47E-08	1.33E-08	9.70E-09	7.90E-09	3.78E-09	-9.26E-10	-6.98E-09	-9.04E-09	-9.36E-09	-1.43E-08	-1.10E-08	-1.28E-08	-1.50E-08	-1.86E-08	-1.41E-08	-1.00E-08	-1.11E-08	-1.15E-08
1.79E-08	1.65E-08	1.53E-08	2.18E-08	1.54E-08	1.10E-08	9.48E-09	6.65E-09	5.16E-09	-2.84E-09	-6.68E-09	-8.36E-09	-9.08E-09	-1.36E-08	-1.36E-08	-1.44E-08	-1.55E-08	-1.87E-08	-1.47E-08	-1.16E-08	-1.16E-08	-1.12E-08
1.72E-08	1.56E-08	1.54E-08	2.37E-08	1.51E-08	9.75E-09	1.02E-08	6.82E-09	6.01E-09	-1.39E-09	-5.28E-09	-7.99E-09	-1.13E-08	-1.31E-08	-1.51E-08	-1.47E-08	-1.62E-08	-1.88E-08	-1.58E-08	-1.29E-08	-1.25E-08	-1.06E-08
1.79E-08	1.49E-08	1.60E-08	1.95E-08	1.42E-08	9.63E-09	9.52E-09	8.16E-09	5.69E-09	-2.43E-11	-3.04E-09	-7.52E-09	-1.25E-08	-1.44E-08	-1.53E-08	-1.67E-08	-1.76E-08	-2.01E-08	-1.73E-08	-1.30E-08	-1.34E-08	-1.09E-08
1.85E-08	1.42E-08	1.61E-08	1.31E-08	1.41E-08	9.35E-09	8.59E-09	9.41E-09	3.60E-09	-4.09E-10	-1.96E-09	-9.35E-09	-1.29E-08	-1.45E-08	-1.51E-08	-1.90E-08	-1.78E-08	-2.17E-08	-1.67E-08	-1.32E-08	-1.39E-08	-1.20E-08
1.83E-08	1.40E-08	1.57E-08	1.35E-08	1.22E-08	8.55E-09	8.11E-09	9.49E-09	2.39E-09	-1.22E-09	-4.37E-09	-1.20E-08	-1.46E-08	-1.50E-08	-1.55E-08	-1.87E-08	-1.76E-08	-2.29E-08	-1.70E-08	-1.37E-08	-1.40E-08	-1.37E-08
1.81E-08	1.39E-08	1.76E-08	1.53E-08	1.16E-08	7.97E-09	8.30E-09	1.05E-08	1.39E-09	-3.97E-09	-8.25E-09	-1.33E-08	-1.60E-08	-1.70E-08	-1.83E-08	-1.84E-08	-1.81E-08	-2.28E-08	-1.75E-08	-1.34E-08	-1.56E-08	-1.41E-08
1.82E-08	1.49E-08	1.96E-08	1.64E-08	1.23E-08	7.88E-09	7.63E-09	1.17E-08	5.18E-12	-6.08E-09	-9.56E-09	-1.30E-08	-1.79E-08	-1.92E-08	-2.07E-08	-1.76E-08	-1.70E-08	-2.32E-08	-1.77E-08	-1.52E-08	-1.62E-08	-1.56E-08
1.86E-08	1.59E-08	1.86E-08	1.47E-08	1.22E-08	8.77E-09	6.19E-09	7.70E-09	-9.31E-10	-7.09E-09	-1.02E-08	-1.28E-08	-1.87E-08	-2.02E-08	-2.22E-08	-1.66E-08	-1.49E-08	-2.38E-08	-1.85E-08	-1.69E-08	-1.58E-08	-1.67E-08
1.73E-08	1.53E-08	1.57E-08	1.11E-08	1.09E-08	9.08E-09	5.38E-09	2.81E-09	-9.56E-10	-7.98E-09	-1.24E-08	-1.43E-08	-1.96E-08	-2.03E-08	-2.23E-08	-1.42E-08	-1.39E-08	-2.51E-08	-1.96E-08	-1.65E-08	-1.69E-08	-1.65E-08
1.61E-08	1.40E-08	1.28E-08	8.62E-09	9.76E-09	8.38E-09	5.05E-09	1.97E-09	-1.66E-09	-8.98E-09	-1.43E-08	-1.46E-08	-2.15E-08	-2.15E-08	-2.18E-08	-1.08E-08	-1.99E-08	-2.56E-08	-2.13E-08	-1.59E-08	-1.77E-08	-1.48E-08
1.44E-08	1.23E-08	1.15E-08	8.03E-09	8.43E-09	6.32E-09	5.16E-09	7.85E-10	-2.79E-09	-9.29E-09	-1.56E-08	-1.49E-08	-2.23E-08	-2.26E-08	-2.15E-08	-1.39E-08	-2.22E-08	-2.57E-08	-2.18E-08	-1.78E-08	-1.84E-08	-1.43E-08
1.29E-08	1.25E-08	1.10E-08	8.25E-09	8.74E-09	5.57E-09	5.63E-09	-1.09E-09	-3.97E-09	-1.00E-08	-1.66E-08	-1.56E-08	-2.31E-08	-2.31E-08	-2.29E-08	-2.51E-08	-2.23E-08	-2.50E-08	-2.10E-08	-1.98E-08	-1.76E-08	-1.55E-08
1.15E-08	1.27E-08	1.12E-08	8.15E-09	9.61E-09	4.52E-09	6.26E-09	-8.05E-10	-4.24E-09	-9.78E-09	-1.66E-08	-1.52E-08	-2.38E-08	-2.34E-08	-2.53E-08	-2.71E-08	-2.30E-08	-2.52E-08	-2.14E-08	-2.10E-08	-1.71E-08	-1.67E-08
1.17E-08	1.16E-08	1.06E-08	7.43E-09	8.47E-09	2.96E-09	5.04E-09	-6.16E-10	-3.82E-09	-1.13E-08	-1.58E-08	-1.52E-08	-2.35E-08	-2.37E-08	-2.50E-08	-2.63E-08	-2.35E-08	-2.64E-08	-2.25E-08	-2.08E-08	-1.83E-08	-1.80E-08
1.14E-08	1.09E-08	9.90E-09	7.21E-09	5.77E-09	2.02E-09	2.80E-09	-3.52E-10	-4.67E-09	-1.22E-08	-1.53E-08	-1.55E-08	-2.35E-08	-2.43E-08	-2.48E-08	-2.63E-08	-2.44E-08	-2.59E-08	-2.27E-08	-2.10E-08	-1.85E-08	-1.86E-08
1.08E-08	1.04E-08	8.38E-09	6.25E-09	4.55E-09	1.54E-09	1.22E-09	-1.02E-09	-6.47E-09	-1.17E-08	-1.40E-08	-1.58E-08	-2.37E-08	-2.47E-08	-2.51E-08	-2.72E-08	-2.42E-08	-2.47E-08	-2.25E-08	-2.26E-08	-1.74E-08	-1.82E-08
9.31E-09	9.99E-09	8.13E-09	5.00E-09	4.77E-09	2.18E-09	5.69E-10	-2.67E-09	-8.71E-09	-1.04E-08	-1.09E-08	-1.61E-08	-2.46E-08	-2.59E-08	-2.45E-08	-2.74E-08	-2.43E-08	-2.40E-08	-2.34E-08	-2.40E-08	-1.76E-08	-1.97E-08
9.07E-09	9.94E-09	7.63E-09	3.31E-09	6.16E-09	2.13E-09	-5.04E-10	-2.45E-09	-9.35E-09	-1.01E-08	-6.94E-09	-1.49E-08	-2.45E-08	-2.64E-08	-2.62E-08	-2.93E-08	-2.50E-08	-2.58E-08	-2.43E-08	-2.42E-08	-2.08E-08	-2.14E-08
8.75E-09	1.06E-08	7.50E-09	3.98E-09	7.42E-09	2.31E-09	-1.24E-09	-2.19E-09	-9.78E-09	-1.09E-08	-7.91E-09	-1.71E-08	-2.43E-08	-2.76E-08	-2.95E-08	-2.95E-08	-2.60E-08	-2.77E-08	-2.42E-08	-2.40E-08	-2.24E-08	-2.26E-08
8.49E-09	1.10E-08	6.98E-09	4.05E-09	7.43E-09	3.11E-09	-1.27E-09	-3.07E-09	-1.04E-08	-1.33E-08	-1.25E-08	-2.19E-08	-2.48E-08	-2.83E-08	-2.97E-08	-2.94E-08	-2.72E-08	-2.80E-08	-2.39E-08	-2.38E-08	-2.34E-08	-2.37E-08
7.87E-09	1.13E-08	5.64E-09	3.19E-09	5.75E-09	2.64E-09	-1.40E-09	-3.23E-09	-1.11E-08	-1.42E-08	-1.92E-08	-2.50E-08	-2.60E-08	-2.88E-08	-2.96E-08	-3.05E-08	-2.78E-08	-2.81E-08	-2.40E-08	-2.45E-08	-2.36E-08	-2.50E-08
7.81E-09	9.03E-09	5.34E-09	2.53E-09	5.70E-09	2.15E-09	-1.34E-09	-3.06E-09	-1.12E-08	-1.67E-08	-2.02E-08	-2.64E-08	-2.65E-08	-2.89E-08	-3.01E-08	-3.14E-08	-2.74E-08	-2.91E-08	-2.54E-08	-2.57E-08	-2.34E-08	-2.55E-08
7.00E-09	8.51E-09	3.92E-09	2.09E-09	4.11E-09	3.54E-10	-1.36E-09	-4.28E-09	-1.10E-08	-1.85E-08	-2.15E-08	-2.76E-08	-2.77E-08	-2.97E-08	-3.10E-08	-3.24E-08	-2.85E-08	-3.14E-08	-2.63E-08	-2.63E-08	-2.19E-08	-2.50E-08
5.68E-09	7.58E-09	2.34E-09	2.35E-09	7.78E-10	-1.07E-09	-2.69E-09	-4.92E-09	-1.20E-08	-1.93E-08	-2.15E-08	-2.79E-08	-2.88E-08	-3.10E-08	-3.19E-08	-3.29E-08	-2.95E-08	-3.16E-08	-2.80E-08	-2.68E-08	-2.38E-08	-2.42E-08
4.59E-09	5.96E-09	1.94E-09	1.69E-09	1.99E-10	-1.32E-09	-4.83E-09	-6.88E-09	-1.24E-08	-1.84E-08	-2.03E-08	-2.76E-08	-3.02E-08	-3.16E-08	-3.29E-08	-3.36E-08	-3.01E-08	-3.21E-08	-2.81E-08	-2.77E-08	-2.47E-08	-2.37E-08
5.06E-09	5.26E-09	1.17E-09	3.81E-10	-5.81E-10	-2.33E-09	-5.01E-09	-8.29E-09	-1.23E-08	-1.83E-08	-2.04E-08	-2.72E-08	-3.10E-08	-3.13E-08	-3.39E-08	-3.44E-08	-3.10E-08	-3.28E-08	-2.94E-08	-2.87E-08	-2.57E-08	-2.38E-08
5.40E-09	3.18E-09	5.36E-10	1.50E-09	-9.83E-10	-1.96E-09	-4.91E-09	-1.05E-08	-1.17E-08	-1.90E-08	-2.05E-08	-2.74E-08	-3.01E-08	-3.15E-08	-3.36E-08	-3.40E-08	-3.22E-08	-3.35E-08	-3.08E-08	-2.85E-08	-2.62E-08	-2.33E-08
3.68E-09	1.66E-09	4.33E-10	1.86E-09	-7.60E-10	-2.86E-09	-6.16E-09	-1.17E-08	-1.31E-08	-1.89E-08	-2.06E-08	-2.64E-08	-3.04E-08	-3.31E-08	-3.38E-08	-3.38E-08	-3.23E-08	-3.39E-08	-3.26E-08	-2.83E-08	-2.65E-08	-2.39E-08
2.28E-09	1.33E-09	4.94E-10	1.24E-09	-7.10E-10	-3.84E-09	-7.49E-09	-1.21E-08	-1.31E-08	-1.84E-08	-2.13E-08	-2.51E-08	-3.04E-08	-3.41E-08	-3.42E-08	-3.44E-08	-3.43E-08	-3.46E-08	-3.36E-08	-2.97E-08	-2.83E-08	-2.61E-08
2.46E-09	1.04E-09	5.62E-10	3.67E-09	-5.98E-12	-4.41E-09	-8.64E-09	-1.21E-08	-1.30E-08	-1.76E-08	-2.06E-08	-2.36E-08	-3.15E-08	-3.43E-08	-3.51E-08	-3.45E-08	-3.54E-08	-3.51E-08	-3.41E-08	-3.26E-08	-3.10E-08	-2.78E-08

2.99E-09	8.72E-11	-1.23E-10	4.84E-09	5.95E-10	-4.60E-09	-9.06E-09	-1.29E-08	-1.23E-08	-1.55E-08	-1.82E-08	-2.12E-08	-3.29E-08	-3.43E-08	-3.62E-08	-3.42E-08	-3.58E-08	-3.52E-08	-3.52E-08	-3.34E-08	-3.31E-08	-2.93E-08
3.76E-09	4.10E-11	-1.13E-09	4.35E-09	7.81E-11	-4.13E-09	-9.66E-09	-1.30E-08	-1.20E-08	-1.17E-08	-1.43E-08	-1.65E-08	-3.40E-08	-3.52E-08	-3.69E-08	-3.49E-08	-3.62E-08	-3.59E-08	-3.60E-08	-3.31E-08	-3.51E-08	-2.92E-08
2.93E-09	-3.07E-10	-2.86E-09	-3.77E-10	-1.71E-09	-4.91E-09	-9.25E-09	-1.36E-08	-1.12E-08	-7.18E-09	-1.30E-08	-1.11E-08	-3.51E-08	-3.61E-08	-3.71E-08	-3.58E-08	-3.63E-08	-3.68E-08	-3.61E-08	-3.37E-08	-3.60E-08	-3.04E-08
1.63E-09	-2.30E-09	-3.39E-09	-1.89E-09	-2.08E-09	-5.23E-09	-9.52E-09	-1.31E-08	-1.51E-08	-8.00E-09	-1.94E-08	-1.56E-08	-3.54E-08	-3.70E-08	-3.72E-08	-3.67E-08	-3.64E-08	-3.79E-08	-3.79E-08	-3.49E-08	-3.59E-08	-3.20E-08
5.72E-10	-3.42E-09	-3.34E-09	-2.57E-09	-2.48E-09	-7.02E-09	-1.06E-08	-1.41E-08	-1.95E-08	-2.08E-08	-2.61E-08	-3.28E-08	-3.59E-08	-3.81E-08	-3.77E-08	-3.68E-08	-3.62E-08	-3.86E-08	-3.82E-08	-3.67E-08	-3.54E-08	-3.16E-08
-1.38E-09	-3.13E-09	-2.60E-09	-5.45E-09	-1.38E-09	-7.61E-09	-1.30E-08	-1.62E-08	-1.99E-08	-2.44E-08	-2.81E-08	-3.35E-08	-3.67E-08	-3.96E-08	-3.91E-08	-3.79E-08	-3.49E-08	-3.94E-08	-3.78E-08	-3.65E-08	-3.54E-08	-3.13E-08
-2.35E-09	-2.33E-09	-2.39E-09	-6.14E-09	-2.39E-09	-6.86E-09	-1.34E-08	-1.67E-08	-2.08E-08	-2.65E-08	-2.99E-08	-3.34E-08	-3.85E-08	-4.13E-08	-4.05E-08	-3.97E-08	-3.43E-08	-4.02E-08	-3.60E-08	-3.67E-08	-3.56E-08	-3.27E-08
-2.74E-09	-2.81E-09	-1.87E-09	-6.25E-09	-4.56E-09	-7.27E-09	-1.24E-08	-1.64E-08	-2.32E-08	-2.67E-08	-3.18E-08	-3.38E-08	-3.99E-08	-4.13E-08	-4.10E-08	-4.14E-08	-4.21E-08	-4.10E-08	-3.53E-08	-3.86E-08	-3.63E-08	-3.51E-08
-2.92E-09	-3.34E-09	-2.93E-09	-6.22E-09	-7.76E-09	-8.04E-09	-1.20E-08	-1.59E-08	-2.36E-08	-2.65E-08	-3.17E-08	-3.52E-08	-4.07E-08	-4.14E-08	-4.15E-08	-4.36E-08	-4.22E-08	-4.03E-08	-3.78E-08	-3.89E-08	-3.73E-08	-3.51E-08
-1.98E-09	-5.26E-09	-3.13E-09	-7.73E-09	-7.80E-09	-7.74E-09	-1.15E-08	-1.77E-08	-2.31E-08	-2.62E-08	-3.19E-08	-3.68E-08	-4.08E-08	-4.10E-08	-4.13E-08	-4.44E-08	-4.31E-08	-3.94E-08	-3.80E-08	-3.81E-08	-3.78E-08	-3.65E-08
-1.49E-09	-4.87E-09	-4.17E-09	-8.23E-09	-8.37E-09	-9.37E-09	-1.23E-08	-1.79E-08	-2.26E-08	-2.64E-08	-3.26E-08	-3.73E-08	-4.06E-08	-4.16E-08	-4.07E-08	-4.56E-08	-4.27E-08	-3.88E-08	-3.83E-08	-3.85E-08	-3.87E-08	-3.84E-08
-1.69E-09	-5.03E-09	-5.82E-09	-8.52E-09	-9.91E-09	-1.14E-08	-1.24E-08	-1.74E-08	-2.24E-08	-2.79E-08	-3.45E-08	-3.68E-08	-4.05E-08	-4.08E-08	-4.09E-08	-4.51E-08	-4.20E-08	-3.84E-08	-4.12E-08	-3.80E-08	-3.94E-08	-3.79E-08
-2.13E-09	-5.56E-09	-6.01E-09	-9.00E-09	-1.00E-08	-1.14E-08	-1.27E-08	-1.76E-08	-2.26E-08	-2.78E-08	-3.43E-08	-3.56E-08	-3.88E-08	-3.88E-08	-4.02E-08	-4.29E-08	-4.12E-08	-3.91E-08	-4.01E-08	-3.65E-08	-3.89E-08	-3.63E-08
-3.78E-09	-5.07E-09	-4.63E-09	-9.34E-09	-9.58E-09	-1.24E-08	-1.41E-08	-1.79E-08	-2.23E-08	-2.71E-08	-3.41E-08	-3.45E-08	-3.51E-08	-3.46E-08	-3.80E-08	-3.93E-08	-3.89E-08	-3.62E-08	-3.62E-08	-3.45E-08	-3.54E-08	-3.53E-08
-4.38E-09	-4.93E-09	-6.33E-09	-1.09E-08	-1.04E-08	-1.20E-08	-1.58E-08	-1.75E-08	-2.26E-08	-2.46E-08	-3.16E-08	-3.06E-08	-2.81E-08	-2.97E-08	-3.15E-08	-3.25E-08	-3.26E-08	-2.98E-08	-2.91E-08	-2.89E-08	-3.01E-08	-3.40E-08
-4.19E-09	-3.87E-09	-6.90E-09	-1.15E-08	-1.12E-08	-1.15E-08	-1.62E-08	-1.80E-08	-2.15E-08	-2.03E-08	-2.47E-08	-2.21E-08	-2.04E-08	-1.91E-08	-1.86E-08	-2.04E-08	-2.11E-08	-1.78E-08	-1.81E-08	-1.98E-08	-2.28E-08	-2.82E-08
-3.57E-09	-1.18E-09	-5.62E-09	-1.12E-08	-1.14E-08	-1.10E-08	-1.53E-08	-1.58E-08	-1.69E-08	-1.41E-08	-1.28E-08	-7.42E-09	-5.27E-09	-1.79E-09	-5.07E-10	-2.97E-09	-4.61E-09	-3.69E-10	-1.51E-09	-4.48E-09	-1.13E-08	-1.81E-08
-1.49E-09	3.96E-09	-3.27E-09	-9.94E-09	-1.02E-08	-7.75E-09	-1.22E-08	-1.00E-08	-8.65E-09	-2.41E-09	6.31E-09	1.35E-08	1.58E-08	1.92E-08	1.93E-08	1.73E-08	1.48E-08	1.91E-08	1.76E-08	1.35E-08	5.70E-09	-1.72E-09
1.05E-09	1.03E-08	-2.93E-09	-7.61E-09	-6.74E-09	-3.40E-09	-6.16E-09	-1.51E-09	5.20E-09	1.96E-08	3.25E-08	3.86E-08	4.02E-08	4.24E-08	4.12E-08	3.89E-08	3.55E-08	3.87E-08	3.71E-08	3.18E-08	2.38E-08	1.56E-08
2.72E-09	1.72E-08	-1.86E-09	-4.19E-09	-1.54E-09	3.22E-09	4.04E-09	1.13E-08	2.64E-08	4.91E-08	6.45E-08	6.77E-08	6.75E-08	6.81E-08	6.57E-08	6.16E-08	5.75E-08	5.92E-08	5.73E-08	5.07E-08	4.30E-08	3.38E-08
-1.43E-09	1.47E-08	-3.81E-09	1.64E-09	6.26E-09	1.66E-08	2.12E-08	3.37E-08	5.58E-08	8.30E-08	9.86E-08	9.90E-08	9.62E-08	9.56E-08	9.18E-08	8.56E-08	8.05E-08	8.18E-08	7.86E-08	7.13E-08	6.37E-08	5.38E-08
-5.36E-09	5.33E-09	-1.60E-09	9.69E-09	1.83E-08	3.26E-08	4.41E-08	6.29E-08	8.92E-08	1.17E-07	1.30E-07	1.29E-07	1.25E-07	1.24E-07	1.18E-07	1.11E-07	1.05E-07	1.05E-07	1.01E-07	9.31E-08	8.50E-08	7.52E-08
-5.77E-09	-6.06E-09	3.68E-09	1.85E-08	3.11E-08	4.89E-08	6.91E-08	9.41E-08	1.20E-07	1.46E-07	1.52E-07	1.54E-07	1.52E-07	1.52E-07	1.46E-07	1.38E-07	1.29E-07	1.29E-07	1.24E-07	1.16E-07	1.07E-07	9.77E-08
-8.05E-09	-4.94E-09	9.79E-09	2.69E-08	4.19E-08	6.30E-08	8.71E-08	1.16E-07	1.40E-07	1.61E-07	1.68E-07	1.76E-07	1.79E-07	1.80E-07	1.75E-07	1.64E-07	1.53E-07	1.52E-07	1.48E-07	1.40E-07	1.30E-07	1.21E-07
-9.81E-09	-1.68E-09	1.50E-08	3.36E-08	4.94E-08	7.32E-08	9.62E-08	1.21E-07	1.45E-07	1.67E-07	1.87E-07	2.00E-07	2.08E-07	2.11E-07	2.04E-07	1.89E-07	1.78E-07	1.75E-07	1.70E-07	1.63E-07	1.55E-07	1.47E-07
-1.04E-08	9.22E-10	1.87E-08	3.80E-08	5.48E-08	7.96E-08	1.02E-07	1.26E-07	1.52E-07	1.77E-07	2.06E-07	2.26E-07	2.39E-07	2.43E-07	2.33E-07	2.14E-07	2.01E-07	1.98E-07	1.93E-07	1.86E-07	1.80E-07	1.75E-07
-1.12E-08	2.18E-09	2.11E-08	4.06E-08	5.85E-08	8.23E-08	1.04E-07	1.30E-07	1.57E-07	1.86E-07	2.21E-07	2.52E-07	2.69E-07	2.74E-07	2.61E-07	2.38E-07	2.22E-07	2.19E-07	2.14E-07	2.07E-07	2.03E-07	2.04E-07
-1.02E-08	2.58E-09	2.15E-08	4.15E-08	5.99E-08	8.28E-08	1.05E-07	1.32E-07	1.59E-07	1.90E-07	2.29E-07	2.69E-07	2.89E-07	2.96E-07	2.82E-07	2.58E-07	2.41E-07	2.38E-07	2.31E-07	2.24E-07	2.22E-07	2.30E-07
-9.45E-09	2.48E-09	2.04E-08	4.01E-08	5.86E-08	8.16E-08	1.03E-07	1.30E-07	1.58E-07	1.89E-07	2.27E-07	2.67E-07	2.88E-07	2.99E-07	2.91E-07	2.74E-07	2.58E-07	2.54E-07	2.44E-07	2.37E-07	2.35E-07	2.47E-07
-9.72E-09	1.19E-09	1.78E-08	3.63E-08	5.51E-08	7.79E-08	9.85E-08	1.25E-07	1.53E-07	1.82E-07	2.18E-07	2.55E-07	2.81E-07	2.93E-07	2.94E-07	2.88E-07	2.74E-07	2.69E-07	2.54E-07	2.44E-07	2.38E-07	2.48E-07
-1.04E-08	-1.80E-09	1.38E-08	3.02E-08	4.92E-08	7.09E-08	9.19E-08	1.17E-07	1.44E-07	1.71E-07	2.06E-07	2.45E-07	2.75E-07	2.86E-07	2.94E-07	2.98E-07	2.89E-07	2.81E-07	2.61E-07	2.47E-07	2.38E-07	2.37E-07
-1.12E-08	-5.55E-09	9.11E-09	2.30E-08	4.20E-08	6.15E-08	8.31E-08	1.06E-07	1.33E-07	1.59E-07	1.93E-07	2.35E-07	2.69E-07	2.77E-07	2.93E-07	3.05E-07	3.02E-07	2.91E-07	2.62E-07	2.47E-07	2.38E-07	2.27E-07
-1.15E-08	-9.10E-09	5.19E-09	1.45E-08	3.39E-08	5.00E-08	7.21E-08	9.47E-08	1.21E-07	1.46E-07	1.79E-07	2.21E-07	2.63E-07	2.70E-07	2.90E-07	2.98E-07	3.02E-07	2.89E-07	2.60E-07	2.43E-07	2.35E-07	2.19E-07
-1.19E-08	-1.17E-08	1.39E-09	5.36E-09	2.39E-08	3.79E-08	6.06E-08	8.22E-08	1.07E-07	1.33E-07	1.64E-07	2.06E-07	2.53E-07	2.59E-07	2.71E-07	2.78E-07	2.89E-07	2.69E-07	2.55E-07	2.34E-07	2.27E-07	2.06E-07
-1.29E-08	-1.31E-08	-6.18E-09	-3.53E-09	9.40E-09	2.44E-08	4.75E-08	6.93E-08	9.18E-08	1.18E-07	1.45E-07	1.88E-07	2.27E-07	2.29E-07	2.37E-07	2.54E-07	2.59E-07	2.39E-07	2.45E-07	2.21E-07	2.13E-07	1.87E-07
-1.40E-08	-1.43E-08	-1.47E-08	-1.17E-08	-1.90E-09	9.32E-09	2.94E-08	5.46E-08	7.54E-08	1.03E-07	1.22E-07	1.47E-07	1.77E-07	1.80E-07	2.00E-07	2.18E-07	2.20E-07	2.11E-07	2.09E-07	2.03E-07	1.89E-07	1.63E-07
-1.59E-08	-1.57E-08	-1.62E-08	-1.31E-08	-1.02E-08	-5.38E-09	1.07E-08	3.75E-08	5.89E-08	8.73E-08	9.57E-08	1.07E-07	1.30E-07	1.46E-07	1.71E-07	1.66E-07	1.79E-07	1.86E-07	1.74E-07	1.73E-07	1.65E-07	1.42E-07
-1.63E-08	-1.54E-08	-1.74E-08	-1.39E-08	-1.21E-08	-1.35E-08	-4.14E-09	1.72E-08	4.14E-08	6.90E-08	7.43E-08	8.28E-08	1.01E-07	1.21E-07	1.38E-07	1.30E-07	1.42E-07	1.54E-07	1.45E-07	1.44E-07	1.35E-07	1.24E-07
-1.55E-08	-1.54E-08	-1.78E-08	-1.51E-08	-1.31E-08	-1.60E-08	-1.49E-08	-3.10E-09	1.75E-08	4.64E-08	5.31E-08	6.32E-08	8.31E-08	9.65E-08	1.08E-07	1.02E-07	1.20E-07	1.27E-07	1.22E-07	1.21E-07	1.12E-07	1.09E-07
-1.50E-08	-1.61E-08	-1.71E-08	-1.56E-08	-1.52E-08	-1.78E-08	-1.90E-08	-1.39E-08	-3.20E-09	2.21E-08	3.50E-08	4.42E-08	6.37E-08	7.13E-08	7.82E-08	7.57E-08	9.67E-08	1.01E-07	1.02E-07	1.03E-07	9.63E-08	9.56E-08
-1.63E-08	-1.62E-08	-1.73E-08	-1.74E-08	-1.67E-08	-1.92E-08	-1.95E-08	-1.98E-08	-1.66E-08	3.16E-09	1.93E-08	2.56E-08	4.27E-08	4.91E-08	5.55E-08	5.43E-08	7.15E-08	7.82E-08	8.48E-08	8.56E-08	8.41E-08	8.48E-08
-1.72E-08	-1.73E-08	-1.82E-08	-2.03E-08	-1.86E-08	-2.20E-08	-2.19E-08	-2.22E-08	-2.56E-08	-1.46E-08	-3.10E-10	7.70E-09	2.30E-08	2.97E-08	3.65E-08	3.61E-08	5.09E-08	5.82E-08	6.63E-08	7.02E-08	7.20E-08	7.45E-08
-1.87E-08	-1.99E-08	-1.88E-08	-2.19E-08	-1.85E-08	-2.43E-08	-2.50E-08	-2.50E-08	-2.84E-08	-2.76E-08	-1.89E-08	-1.14E-08	6.46E-09	1.19E-08	1.73E-08	1.97E-08	3.22E-08	4.11E-08	5.10E-08	5.65E-08	6.04E-08	6.48E-08
-1.92E-08	-2.02E-08	-1.92E-08	-2.26E-08	-1.86E-08	-2.51E-08	-2.75E-08	-2.72E-08	-3.06E-08	-3.48E-08	-3.33E-08	-2.45E-08	-1.15E-08	-5.01E-09	-4.59E-10	3.26E-09	1.58E-08	2.61E-08	4.04E-08	4.57E-08	4.95E-08	5.39E-08
-1.88E-08	-1.99E-08	-1.84E-08	-2.39E-08	-2.06E-08	-2.60E-08	-2.83E-08	-2.78E-08	-3.29E-08	-3.70E-08	-3.46E-08	-3.96E-08	-3.00E-08	-1.71E-08	-1.54E-08	-1.22E-08	7.34E-10	1.38E-08	2.70E-08	3.17E-08	3.67E-08	4.09E-08
-1.87E-08	-2.00E-08	-2.02E-08	-2.42E-08	-2.42E-08	-2.50E-08	-2.86E-08	-2.87E-08	-3.41E-08	-3.83E-08	-3.67E-08	-4.26E-08	-4.41E-08	-2.68E-08	-2.81E-08	-2.29E-08	-1.34E-08	1.47E-09	1.27E-08	1.80E-08	2.20E-08	2.58E-08
-1.95E-08	-2.02E-08	-2.24E-08	-2.52E-08	-2.46E-08	-2.61E-08	-2.95E-08	-3.04E-08	-3.56E-08	-3.86E-08	-3.94E-08	-4.50E-08	-4.49E-08	-3.71E-08	-3.77E-08	-2.78E-08	-2.47E-08	-1.12E-08	-3.30E-09	-1.61E-10	6.57E-09	9.10E-09
-2.13E-08	-2.04E-08	-2.36E-08	-2.56E-08	-2.54E-08	-2.71E-08	-3.10E-08	-3.33E-08	-3.63E-08	-3.82E-08	-4.21E-08	-4.55E-08	-4.57E-08	-4.68E-08	-4.64E-08	-3.36E-08	-2.99E-08	-3.13E-08	-2.39E-08	-1.33E-08	-8.05E-09	-6.39E-09
-2.24E-08	-2.16E-08	-2.40E-08	-2.67E-08	-2.74E-08	-2.78E-08	-3.17E-08	-3.38E-08	-3.62E-08	-3.86E-08	-4.25E-08	-4.61E-08	-4.75E-08	-4.70E-08	-4.78E-08	-4.72E-08	-3.81E-08	-4.33E-08	-3.70E-08	-2.16E-08	-2.15E-08	-2.12E-08
-2.29E-08	-2.16E-08	-2.43E-08	-2.79E-08	-2.78E-08	-2.90E-08	-3.21E-08	-3.29E-08	-3.59E-08	-3.90E-08	-4.28E-08	-4.68E-08	-4.79E-08	-4.68E-08	-4.87E-08	-4.78E-08	-4.95E-08	-5.10E-08	-4.80E-08	-3.13E-08	-3.67E-08	-3.42E-08
-2.31E-08	-2.17E-08	-2.33E-08	-2.92E-08	-2.68E-08	-2.95E-08	-3.24E-08	-3.21E-08	-3.50E-08	-3.85E-08	-4.25E-08	-4.65E-08	-4.85E-08	-4.61E-08	-4.85E-08	-4.69E-08	-4.95E-08	-5.08E-08	-5.08E-08	-4.89E-08	-4.40E-08	-4.36E-08
-2.26E-08	-2.13E-08	-2.27E-08	-2.79E-08	-2.65E-08	-3.04E-08	-3.30E-08	-3.31E-08	-3.48E-08	-3.87E-08	-4.26E-08	-4.62E-08	-4.93E-08	-4.68E-08	-4.80E-08	-4.73E-08	-4.92E-08	-5.18E-08	-5.01E-08	-4.92E-08	-4.45E-08	-4.88E-08
-2.20E-08	-2.09E-08	-2.37E-08	-2.63E-08	-2.60E-08	-3.00E-08	-3.31E-08	-3.51E-08	-3.44E-08	-3.97E-08	-4.22E-08	-4.51E-08	-4.95E-08	-4.70E-08	-4.80E-08	-4.82E-08	-4.90E-08	-5.24E-08	-4.93E-08	-4.84E-08	-4.32E-08	-4.88E-08
-2.11E-08	-2.09E-08	-2.48E-08	-2.49E-08	-2.72E-08	-2.81E-08	-3.36E-08	-3.46E-08	-3.43E-08	-3.98E-08	-4.19E-08	-4.46E-08	-4.90E-08	-4.56E-08	-4.72E-08	-4.80E-08	-4.90E-08	-5.09E-08	-5.05E-08	-4.80E-08	-4.25E-08	-4.93E-08
-1.95E-08	-2.12E-08	-2.54E-08	-2.42E-08	-2.70E-08	-2.84E-08	-3.30E-08	-3.47E-08	-3.41E-08	-3.93E-08	-4.19E-08	-4.42E-08	-4.82E-08	-4.56E-08	-4.58E-08	-4.73E-08	-4.90E-08	-5.03E-08	-5.23E-08	-4.88E-08	-3.85E-08	-4.95E-08
-1.73E-08	-2.20E-08	-2.45E-08	-2.53E-08	-2.72E-08	-2.96E-08	-3.37E-08	-3.51E-08	-3.42E-08	-3.96E-08	-4.14E-08	-4.52E-08	-4.75E-08	-4.64E-08	-4.57E-08	-4.67E-08	-4.80E-08	-5.12E-08	-5.27E-08	-4.91E-08	-3.51E-08	-5.09E-08
-1.62E-08	-2.40E-08	-2.39E-08	-2.59E-08	-2.79E-08	-3.04E-08	-3.36E-08	-3.58E-08	-3.42E-08	-4.01E-08	-4.08E-08	-4.52E-08	-4.71E-08	-4.82E-08	-4.82E-08	-4.87E-08	-4.79E-08	-5.29E-08	-5.21E-08	-4.85E-08	-4.62E-08	-5.14E-08
-1.75E-08	-2.53E-08	-2.49E-08	-2.62E-08	-2.83E-08	-3.05E-08	-3.30E-08	-3.60E-08	-3.50E-08	-3.97E-08	-4.20E-08	-4.43E-08	-4.62E-08	-4.95E-08	-4.92E-08	-4.89E-08	-4.87E-08	-5.30E-08	-5.09E-08	-4.95E-08	-4.83E-08	-4.96E-08
-2.21E-08	-2.58E-08	-2.69E-08	-2.68E-08	-2.91E-08	-3.13E-08	-3.27E-08	-3.64E-08	-3.59E-08	-3.88E-08	-4.33E-08	-4.48E-08	-4.64E-08	-4.95E-08	-4.86E-08	-4.81E-08	-4.93E-08	-5.27E-08	-5.01E-08	-4.97E-08	-4.40E-08	-4.75E-08
-2.27E-08	-2.59E-08	-2.72E-08	-2.88E-08	-3.01E-08	-3.23E-08	-3.30E-08	-3.72E-08	-3.59E-08	-3.95E-08	-4.29E-08	-4.50E-08	-4.66E-08	-5.01E-08	-4.81E-08	-4.77E-08	-5.04E-08	-5.28E-08	-5.06E-08	-4.90E-08	-3.99E-08	-4.66E-08
-2.22E-08	-2.55E-08	-2.80E-08	-3.06E-08	-2.92E-08	-3.28E-08	-3.28E-08	-3.77E-08	-3.50E-08	-4.16E-08	-4.23E-08	-4.69E-08	-4.72E-08	-5.14E-08	-4.93E-08	-4.91E-08	-5.06E-08	-5.21E-08	-5.06E-08	-4.75E-08	-4.39E-08	-4.77E-08
-2.29E-08	-2.56E-08	-2.73E-08	-3.09E-08	-2.94E-08	-3.24E-08	-3.21E-08	-3.70E-08	-3.44E-08	-4.18E-08	-4.28E-08	-4.74E-08	-4.73E-08	-5.11E-08	-4.97E-08	-4.98E-08	-5.13E-08	-5.11E-08	-5.08E-08	-4.79E-08	-5.10E-08	-4.72E-08
-2.27E-08	-2.58E-08	-2.96E-08	-3.06E-08	-3.04E-08	-3.23E-08	-3.26E-08	-3.65E-08	-3.37E-08	-4.17E-08	-4.51E-08	-4.74E-08	-4.68E-08	-5.03E-08	-4.87E-08	-4.99E-08	-5.18E-08	-5.11E-08	-5.16E-08	-4.97E-08	-5.26E-08	-4.77E-08

-2.23E-08	-2.70E-08	-2.98E-08	-2.95E-08	-3.13E-08	-3.21E-08	-3.29E-08	-3.57E-08	-3.24E-08	-4.13E-08	-4.61E-08	-4.73E-08	-4.68E-08	-5.00E-08	-5.02E-08	-4.90E-08	-5.19E-08	-5.15E-08	-5.20E-08	-5.21E-08	-5.32E-08	-4.81E-08
-2.31E-08	-2.67E-08	-2.87E-08	-2.96E-08	-3.17E-08	-3.13E-08	-3.26E-08	-3.54E-08	-3.59E-08	-4.09E-08	-4.53E-08	-4.73E-08	-4.69E-08	-5.03E-08	-5.01E-08	-4.95E-08	-5.32E-08	-5.19E-08	-5.16E-08	-5.32E-08	-5.34E-08	-4.77E-08
-2.36E-08	-2.57E-08	-2.74E-08	-2.94E-08	-3.20E-08	-3.10E-08	-3.24E-08	-3.42E-08	-3.76E-08	-4.03E-08	-4.44E-08	-4.76E-08	-4.77E-08	-5.12E-08	-5.09E-08	-4.96E-08	-5.44E-08	-5.16E-08	-5.02E-08	-5.20E-08	-5.27E-08	-4.59E-08
-2.51E-08	-2.47E-08	-2.70E-08	-2.91E-08	-3.19E-08	-3.18E-08	-3.23E-08	-3.40E-08	-3.69E-08	-4.00E-08	-4.55E-08	-4.76E-08	-4.73E-08	-5.11E-08	-5.14E-08	-4.93E-08	-5.36E-08	-5.05E-08	-4.93E-08	-5.06E-08	-5.18E-08	-4.76E-08
-2.72E-08	-2.43E-08	-2.77E-08	-2.85E-08	-3.12E-08	-3.30E-08	-3.15E-08	-3.43E-08	-3.74E-08	-3.82E-08	-4.62E-08	-4.73E-08	-4.76E-08	-4.94E-08	-5.27E-08	-5.05E-08	-5.31E-08	-5.11E-08	-4.94E-08	-5.01E-08	-4.95E-08	-5.11E-08
-2.73E-08	-2.41E-08	-2.64E-08	-2.75E-08	-3.02E-08	-3.28E-08	-3.05E-08	-3.53E-08	-3.71E-08	-3.47E-08	-4.47E-08	-4.88E-08	-4.88E-08	-4.79E-08	-5.21E-08	-4.94E-08	-5.25E-08	-5.23E-08	-4.91E-08	-5.06E-08	-4.86E-08	-5.06E-08
-2.76E-08	-2.48E-08	-2.57E-08	-2.76E-08	-3.01E-08	-3.21E-08	-3.15E-08	-3.56E-08	-3.61E-08	-3.36E-08	-4.38E-08	-4.95E-08	-4.84E-08	-4.82E-08	-5.09E-08	-4.79E-08	-5.14E-08	-5.23E-08	-4.92E-08	-5.17E-08	-4.93E-08	-4.96E-08
-2.83E-08	-2.51E-08	-2.61E-08	-2.92E-08	-2.96E-08	-3.15E-08	-3.13E-08	-3.57E-08	-3.54E-08	-3.74E-08	-4.49E-08	-4.89E-08	-4.66E-08	-4.85E-08	-5.00E-08	-4.80E-08	-5.14E-08	-5.19E-08	-5.02E-08	-5.19E-08	-5.04E-08	-4.85E-08
-2.90E-08	-2.57E-08	-2.78E-08	-2.99E-08	-2.96E-08	-3.08E-08	-3.07E-08	-3.55E-08	-3.50E-08	-4.04E-08	-4.65E-08	-4.83E-08	-4.62E-08	-4.83E-08	-5.02E-08	-4.82E-08	-5.18E-08	-5.22E-08	-4.92E-08	-5.12E-08	-4.94E-08	-4.94E-08
-2.90E-08	-2.67E-08	-2.92E-08	-3.02E-08	-3.08E-08	-2.97E-08	-3.00E-08	-3.48E-08	-3.65E-08	-3.97E-08	-4.62E-08	-4.77E-08	-4.78E-08	-4.84E-08	-4.98E-08	-4.91E-08	-5.15E-08	-5.18E-08	-4.91E-08	-5.07E-08	-4.85E-08	-5.19E-08

8.80E-16	6.85E-16	7.53E-16	6.02E-16	5.38E-16	4.15E-16	4.27E-16	2.18E-16	1.59E-16	4.04E-17	1.46E-17	5.68E-19	1.50E-17	7.29E-18	5.14E-18	9.06E-18	1.46E-17	6.78E-22	1.88E-18	4.51E-17	4.66E-17	1.80E-18
7.26E-16	6.76E-16	9.01E-16	7.04E-16	5.62E-16	4.21E-16	3.58E-16	2.35E-16	1.23E-16	3.90E-17	7.44E-18	7.99E-19	1.55E-17	1.79E-17	6.06E-18	6.64E-18	1.07E-17	5.27E-19	3.10E-18	1.20E-17	2.07E-17	4.11E-18
7.04E-16	7.31E-16	9.13E-16	8.33E-16	5.79E-16	4.48E-16	3.91E-16	2.53E-16	1.19E-16	2.61E-17	6.47E-18	3.96E-20	1.68E-17	1.61E-17	2.82E-17	6.29E-18	1.18E-17	4.33E-19	1.11E-18	2.65E-21	3.93E-18	6.57E-18
6.95E-16	7.62E-16	1.14E-15	1.09E-15	5.84E-16	4.38E-16	5.15E-16	2.83E-16	1.28E-16	1.25E-17	1.10E-18	2.00E-18	2.55E-17	2.06E-17	4.08E-17	6.99E-18	2.21E-17	1.01E-18	1.60E-17	4.51E-18	2.24E-18	4.13E-18
6.88E-16	8.23E-16	1.59E-15	1.56E-15	5.54E-16	4.33E-16	5.99E-16	2.55E-16	1.06E-16	1.54E-17	6.51E-19	6.35E-18	2.59E-17	3.20E-17	4.35E-17	1.40E-17	2.84E-17	4.60E-18	1.76E-17	1.04E-17	6.51E-18	2.61E-18
7.10E-16	8.78E-16	2.08E-15	1.99E-15	5.34E-16	4.06E-16	6.53E-16	2.41E-16	7.27E-17	1.54E-17	4.69E-22	8.45E-18	2.45E-17	5.89E-17	7.30E-17	1.47E-17	3.05E-17	1.13E-17	2.87E-17	1.17E-17	6.95E-18	4.73E-18
7.08E-16	8.01E-16	2.11E-15	1.94E-15	5.66E-16	4.14E-16	3.79E-16	1.60E-16	5.96E-17	9.32E-18	2.45E-19	9.90E-18	2.44E-17	7.01E-17	9.89E-17	1.28E-17	3.72E-17	1.61E-17	2.87E-17	1.39E-17	2.82E-19	1.19E-17
7.51E-16	6.19E-16	1.12E-15	8.30E-16	5.53E-16	4.26E-16	2.28E-16	1.24E-16	5.60E-17	3.70E-18	2.05E-18	1.07E-17	3.02E-17	7.92E-17	8.73E-17	1.92E-17	5.54E-17	2.04E-17	2.98E-17	2.12E-17	1.98E-18	3.02E-17
7.64E-16	5.89E-16	4.79E-16	5.37E-16	5.08E-16	4.32E-16	1.98E-16	1.52E-16	5.72E-17	4.68E-18	2.70E-18	1.32E-17	4.69E-17	1.01E-16	7.10E-17	6.02E-17	9.28E-17	2.73E-17	5.10E-17	2.90E-17	4.99E-18	6.74E-17
7.83E-16	6.24E-16	4.81E-16	4.85E-16	4.29E-16	4.24E-16	1.71E-16	1.62E-16	5.49E-17	9.44E-18	1.95E-18	1.38E-17	5.87E-17	1.11E-16	8.54E-17	8.01E-17	9.93E-17	3.19E-17	5.62E-17	2.51E-17	7.25E-18	1.22E-16
7.94E-16	6.52E-16	4.71E-16	4.81E-16	4.27E-16	3.96E-16	1.70E-16	1.42E-16	5.55E-17	1.36E-17	8.36E-18	1.29E-17	5.74E-17	1.22E-16	8.81E-17	6.93E-17	1.05E-16	3.85E-17	7.26E-17	2.25E-17	1.44E-17	8.17E-17
7.99E-16	6.70E-16	4.25E-16	5.18E-16	4.46E-16	3.95E-16	1.59E-16	1.58E-16	5.86E-17	2.19E-17	4.11E-17	1.76E-17	5.13E-17	1.20E-16	7.53E-17	6.60E-17	1.26E-16	3.94E-17	1.03E-16	5.82E-17	2.30E-17	2.27E-18
7.26E-16	6.34E-16	3.92E-16	4.81E-16	4.14E-16	3.79E-16	1.60E-16	1.68E-16	5.15E-17	3.54E-17	1.11E-16	3.83E-17	4.79E-17	1.26E-16	8.23E-17	8.84E-17	1.39E-16	4.41E-17	1.03E-16	7.77E-17	4.59E-17	2.17E-21
7.14E-16	6.88E-16	3.91E-16	4.40E-16	3.33E-16	3.58E-16	1.64E-16	1.27E-16	5.74E-17	2.98E-17	8.31E-17	5.40E-17	5.09E-17	1.18E-16	8.18E-17	1.03E-16	1.41E-16	5.01E-17	8.58E-17	7.02E-17	7.61E-17	4.06E-19
6.51E-16	7.12E-16	4.25E-16	4.25E-16	3.04E-16	3.34E-16	1.54E-16	9.81E-17	6.79E-17	8.43E-18	2.34E-17	5.10E-17	4.40E-17	1.28E-16	1.12E-16	1.19E-16	1.13E-16	3.92E-17	9.28E-17	5.19E-17	1.03E-16	1.70E-17
6.25E-16	5.78E-16	4.35E-16	4.50E-16	2.87E-16	3.21E-16	1.67E-16	1.02E-16	6.09E-17	2.48E-18	2.52E-17	3.95E-17	7.12E-17	1.25E-16	1.48E-16	1.16E-16	6.06E-17	9.35E-18	6.50E-17	6.92E-17	9.54E-17	5.25E-17
5.98E-16	5.18E-16	4.16E-16	3.85E-16	2.86E-16	3.36E-16	1.83E-16	1.16E-16	4.44E-17	1.94E-18	2.34E-17	4.23E-17	9.79E-17	1.30E-16	1.46E-16	9.39E-17	1.46E-17	3.66E-18	3.43E-17	8.85E-17	9.34E-17	5.64E-17
5.71E-16	5.33E-16	3.81E-16	3.52E-16	2.84E-16	3.30E-16	2.25E-16	1.03E-16	2.31E-17	9.35E-19	2.44E-17	7.49E-17	9.56E-17	1.29E-16	1.39E-16	5.87E-17	1.39E-18	5.31E-17	1.13E-17	9.57E-17	1.30E-16	4.50E-17
5.54E-16	5.44E-16	3.78E-16	3.31E-16	2.80E-16	3.54E-16	3.02E-16	1.08E-16	2.68E-17	3.98E-20	2.62E-17	8.97E-17	9.25E-17	1.02E-16	1.30E-16	4.16E-17	7.51E-18	7.53E-17	1.31E-17	9.37E-17	1.47E-16	5.03E-17
5.51E-16	4.96E-16	3.61E-16	3.31E-16	2.81E-16	3.42E-16	4.77E-16	1.31E-16	3.28E-17	1.03E-18	3.05E-17	9.36E-17	1.23E-16	8.72E-17	1.19E-16	8.09E-17	1.44E-18	2.15E-17	5.44E-17	8.22E-17	1.38E-16	7.45E-17
5.58E-16	3.91E-16	3.39E-16	3.16E-16	2.89E-16	2.90E-16	5.85E-16	1.17E-16	1.62E-17	7.11E-22	3.84E-17	1.05E-16	1.33E-16	8.93E-17	1.13E-16	1.68E-16	5.86E-17	4.44E-18	1.34E-16	9.97E-17	1.15E-16	9.68E-17
5.26E-16	3.77E-16	3.09E-16	3.07E-16	2.68E-16	2.51E-16	2.45E-16	5.48E-17	1.49E-17	1.26E-18	2.97E-17	1.25E-16	1.16E-16	1.38E-16	1.64E-16	2.12E-16	2.06E-16	2.58E-16	1.79E-16	1.40E-16	9.38E-17	8.79E-17
4.07E-16	3.81E-16	2.98E-16	2.79E-16	2.02E-16	1.79E-16	1.11E-16	4.02E-17	1.73E-17	1.46E-18	2.78E-17	1.27E-16	9.29E-17	1.71E-16	1.60E-16	1.60E-16	3.03E-16	3.02E-16	1.89E-16	1.29E-16	8.40E-17	1.04E-16
3.73E-16	3.49E-16	2.75E-16	2.87E-16	1.90E-16	1.64E-16	1.05E-16	5.14E-17	1.67E-17	8.62E-19	3.12E-17	9.54E-17	1.02E-16	1.88E-16	1.16E-16	1.33E-16	2.60E-16	3.06E-16	1.96E-16	9.95E-17	1.05E-16	1.23E-16
3.50E-16	2.91E-16	2.48E-16	3.64E-16	2.15E-16	1.77E-16	9.41E-17	6.25E-17	1.43E-17	8.57E-19	4.87E-17	8.17E-17	8.75E-17	2.04E-16	1.20E-16	1.65E-16	2.25E-16	3.44E-16	2.00E-16	1.00E-16	1.23E-16	1.32E-16
3.21E-16	2.71E-16	2.33E-16	4.76E-16	2.37E-16	1.21E-16	9.00E-17	4.42E-17	2.66E-17	8.07E-18	4.47E-17	7.00E-17	8.25E-17	1.86E-16	1.84E-16	2.06E-16	2.39E-16	3.49E-16	2.16E-16	1.36E-16	1.34E-16	1.25E-16
2.96E-16	2.43E-16	2.37E-16	5.60E-16	2.29E-16	9.51E-17	1.04E-16	4.66E-17	3.61E-17	1.92E-18	2.79E-17	6.39E-17	1.28E-16	1.72E-16	2.27E-16	2.18E-16	2.63E-16	3.54E-16	2.51E-16	1.65E-16	1.56E-16	1.13E-16
3.19E-16	2.22E-16	2.57E-16	3.79E-16	2.01E-16	9.27E-17	9.06E-17	6.67E-17	3.24E-17	5.89E-22	9.25E-18	5.66E-17	1.56E-16	2.06E-16	2.33E-16	2.80E-16	3.11E-16	4.05E-16	2.98E-16	1.70E-16	1.81E-16	1.19E-16
3.41E-16	2.03E-16	2.58E-16	1.72E-16	2.00E-16	8.73E-17	7.39E-17	8.85E-17	1.30E-17	1.67E-19	3.85E-18	8.73E-17	1.67E-16	2.09E-16	2.27E-16	3.61E-16	3.18E-16	4.72E-16	2.79E-16	1.75E-16	1.92E-16	1.45E-16
3.34E-16	1.96E-16	2.46E-16	1.84E-16	1.48E-16	7.31E-17	6.58E-17	9.00E-17	5.70E-18	1.48E-18	1.91E-17	1.44E-16	2.13E-16	2.24E-16	2.42E-16	3.51E-16	3.08E-16	5.23E-16	2.88E-16	1.88E-16	1.97E-16	1.88E-16
3.28E-16	1.93E-16	3.09E-16	2.33E-16	1.35E-16	6.35E-17	6.88E-17	1.10E-16	1.93E-18	1.58E-17	6.81E-17	1.77E-16	2.57E-16	2.88E-16	3.36E-16	3.39E-16	3.26E-16	5.22E-16	3.07E-16	1.80E-16	2.44E-16	1.99E-16
3.33E-16	2.23E-16	3.85E-16	2.69E-16	1.52E-16	6.21E-17	5.83E-17	1.37E-16	2.69E-23	3.70E-17	9.13E-17	1.68E-16	3.19E-16	3.67E-16	4.29E-16	3.10E-16	2.88E-16	5.40E-16	3.14E-16	2.30E-16	2.64E-16	2.44E-16
3.47E-16	2.52E-16	3.47E-16	2.15E-16	1.49E-16	7.69E-17	3.83E-17	5.92E-17	8.66E-19	5.02E-17	1.03E-16	1.63E-16	3.51E-16	4.08E-16	4.94E-16	2.74E-16	2.23E-16	5.68E-16	3.42E-16	2.86E-16	2.49E-16	2.77E-16
3.00E-16	2.34E-16	2.47E-16	1.23E-16	1.19E-16	8.24E-17	2.90E-17	7.89E-18	9.13E-19	6.36E-17	1.54E-16	2.05E-16	3.85E-16	4.14E-16	4.97E-16	2.02E-16	1.93E-16	6.30E-16	3.83E-16	2.74E-16	2.85E-16	2.71E-16
2.59E-16	1.95E-16	1.64E-16	7.43E-17	9.53E-17	7.02E-17	2.55E-17	3.89E-18	2.74E-18	8.07E-17	2.06E-16	2.14E-16	4.62E-16	4.64E-16	4.74E-16	1.16E-16	3.98E-16	6.55E-16	4.55E-16	2.51E-16	3.13E-16	2.18E-16
2.07E-16	1.50E-16	1.33E-16	6.45E-17	7.10E-17	4.00E-17	2.67E-17	6.16E-19	7.80E-18	8.62E-17	2.44E-16	2.21E-16	4.98E-16	5.10E-16	4.64E-16	1.93E-16	4.93E-16	6.61E-16	4.73E-16	3.16E-16	3.38E-16	2.03E-16
1.68E-16	1.55E-16	1.22E-16	6.81E-17	7.63E-17	3.10E-17	3.17E-17	1.18E-18	1.58E-17	1.00E-16	2.77E-16	2.44E-16	5.35E-16	5.36E-16	5.23E-16	6.28E-16	4.99E-16	6.25E-16	4.39E-16	3.94E-16	3.11E-16	2.40E-16
1.32E-16	1.62E-16	1.26E-16	6.64E-17	9.24E-17	2.04E-17	3.92E-17	6.48E-19	1.80E-17	9.56E-17	2.75E-16	2.30E-16	5.66E-16	5.48E-16	6.40E-16	7.37E-16	5.29E-16	6.34E-16	4.57E-16	4.42E-16	2.91E-16	2.79E-16
1.37E-16	1.35E-16	1.13E-16	5.52E-17	7.17E-17	8.77E-18	2.54E-17	3.80E-19	1.46E-17	1.27E-16	2.49E-16	2.30E-16	5.53E-16	5.62E-16	6.23E-16	6.94E-16	5.50E-16	6.99E-16	5.08E-16	4.34E-16	3.36E-16	3.26E-16
1.31E-16	1.18E-16	9.80E-17	5.20E-17	3.33E-17	4.07E-18	7.84E-18	1.24E-19	2.18E-17	1.48E-16	2.34E-16	2.40E-16	5.53E-16	5.90E-16	6.15E-16	6.93E-16	5.93E-16	6.72E-16	5.14E-16	4.42E-16	3.41E-16	3.44E-16
1.16E-16	1.09E-16	7.02E-17	3.90E-17	2.07E-17	2.37E-18	1.48E-18	1.04E-18	4.19E-17	1.38E-16	1.97E-16	2.51E-16	5.62E-16	6.11E-16	6.29E-16	7.38E-16	5.86E-16	6.09E-16	5.05E-16	5.09E-16	3.02E-16	3.31E-16
8.66E-17	9.98E-17	6.61E-17	2.50E-17	2.28E-17	4.75E-18	3.23E-19	7.12E-18	7.59E-17	1.09E-16	1.18E-16	2.59E-16	6.06E-16	6.68E-16	6.02E-16	7.48E-16	5.89E-16	5.75E-16	5.49E-16	5.75E-16	3.09E-16	3.86E-16
8.23E-17	9.88E-17	5.82E-17	1.09E-17	3.79E-17	4.54E-18	2.54E-19	6.01E-18	8.75E-17	1.01E-16	4.81E-17	2.23E-16	6.02E-16	6.97E-16	6.89E-16	8.57E-16	6.25E-16	6.67E-16	5.90E-16	5.85E-16	4.33E-16	4.60E-16
7.65E-17	1.13E-16	5.63E-17	1.59E-17	5.50E-17	5.35E-18	1.54E-18	4.81E-18	9.57E-17	1.19E-16	6.26E-17	2.92E-16	5.92E-16	7.61E-16	8.71E-16	8.71E-16	6.78E-16	7.69E-16	5.85E-16	5.75E-16	5.01E-16	5.12E-16
7.21E-17	1.21E-16	4.87E-17	1.64E-17	5.51E-17	9.65E-18	1.62E-18	9.42E-18	1.08E-16	1.76E-16	1.57E-16	4.80E-16	6.16E-16	8.02E-16	8.84E-16	8.66E-16	7.42E-16	7.82E-16	5.71E-16	5.68E-16	5.47E-16	5.62E-16
6.19E-17	1.28E-16	3.18E-17	1.02E-17	3.30E-17	6.95E-18	1.97E-18	1.05E-17	1.23E-16	2.03E-16	3.70E-16	6.24E-16	6.77E-16	8.27E-16	8.77E-16	9.31E-16	7.72E-16	7.89E-16	5.74E-16	5.98E-16	5.57E-16	6.23E-16
6.10E-17	8.15E-17	2.86E-17	6.41E-18	3.25E-17	4.64E-18	1.80E-18	9.39E-18	1.24E-16	2.79E-16	4.08E-16	6.95E-16	7.01E-16	8.33E-16	9.04E-16	9.85E-16	7.49E-16	8.49E-16	6.43E-16	6.60E-16	5.49E-16	6.49E-16
4.90E-17	7.24E-17	1.54E-17	4.36E-18	1.69E-17	1.25E-19	1.86E-18	1.83E-17	1.20E-16	3.42E-16	4.63E-16	7.62E-16	7.68E-16	8.82E-16	9.63E-16	1.05E-15	8.11E-16	9.86E-16	6.89E-16	6.92E-16	4.81E-16	6.24E-16

3.22E-17	5.75E-17	5.47E-18	5.54E-18	6.05E-19	1.15E-18	7.25E-18	2.42E-17	1.45E-16	3.74E-16	4.62E-16	7.77E-16	8.28E-16	9.61E-16	1.02E-15	1.08E-15	8.70E-16	9.96E-16	7.83E-16	7.17E-16	5.67E-16	5.87E-16
2.11E-17	3.56E-17	3.77E-18	2.86E-18	3.94E-20	1.74E-18	2.33E-17	4.74E-17	1.54E-16	3.40E-16	4.13E-16	7.62E-16	9.12E-16	9.98E-16	1.08E-15	1.13E-15	9.07E-16	1.03E-15	7.91E-16	7.69E-16	6.10E-16	5.61E-16
2.56E-17	2.77E-17	1.36E-18	1.45E-19	3.38E-19	5.41E-18	2.51E-17	6.88E-17	1.51E-16	3.34E-16	4.18E-16	7.40E-16	9.61E-16	9.81E-16	1.15E-15	1.18E-15	9.60E-16	1.07E-15	8.67E-16	8.22E-16	6.62E-16	5.66E-16
2.92E-17	1.01E-17	2.87E-19	2.26E-18	9.67E-19	3.84E-18	2.41E-17	1.11E-16	1.37E-16	3.61E-16	4.19E-16	7.51E-16	9.04E-16	9.91E-16	1.13E-15	1.16E-15	1.04E-15	1.12E-15	9.51E-16	8.11E-16	6.87E-16	5.41E-16
1.35E-17	2.74E-18	1.87E-19	3.44E-18	5.78E-19	8.18E-18	3.80E-17	1.36E-16	1.71E-16	3.56E-16	4.25E-16	6.95E-16	9.23E-16	1.09E-15	1.14E-15	1.14E-15	1.04E-15	1.15E-15	1.06E-15	7.99E-16	7.04E-16	5.71E-16
5.21E-18	1.78E-18	2.44E-19	1.54E-18	5.05E-19	1.47E-17	5.61E-17	1.46E-16	1.71E-16	3.39E-16	4.55E-16	6.32E-16	9.27E-16	1.16E-15	1.17E-15	1.18E-15	1.17E-15	1.20E-15	1.13E-15	8.81E-16	8.00E-16	6.80E-16
6.06E-18	1.09E-18	3.15E-19	1.35E-17	3.58E-23	1.95E-17	7.46E-17	1.45E-16	1.70E-16	3.09E-16	4.24E-16	5.56E-16	9.95E-16	1.17E-15	1.23E-15	1.19E-15	1.26E-15	1.23E-15	1.17E-15	1.06E-15	9.60E-16	7.70E-16
8.96E-18	7.60E-21	1.53E-20	2.34E-17	3.54E-19	2.12E-17	8.21E-17	1.66E-16	1.52E-16	2.42E-16	3.30E-16	4.50E-16	1.08E-15	1.17E-15	1.31E-15	1.17E-15	1.28E-15	1.24E-15	1.24E-15	1.12E-15	1.09E-15	8.60E-16
1.41E-17	1.68E-21	1.28E-18	1.89E-17	6.11E-21	1.71E-17	9.33E-17	1.68E-16	1.43E-16	1.37E-16	2.05E-16	2.72E-16	1.15E-15	1.24E-15	1.36E-15	1.22E-15	1.31E-15	1.29E-15	1.30E-15	1.10E-15	1.23E-15	8.51E-16
8.61E-18	9.43E-20	8.16E-18	1.42E-19	2.93E-18	2.41E-17	8.56E-17	1.86E-16	1.24E-16	5.16E-17	1.70E-16	1.24E-16	1.23E-15	1.30E-15	1.38E-15	1.28E-15	1.32E-15	1.35E-15	1.30E-15	1.13E-15	1.29E-15	9.22E-16
2.65E-18	5.28E-18	1.15E-17	3.58E-18	4.32E-18	2.73E-17	9.07E-17	1.71E-16	2.29E-16	6.39E-17	3.77E-16	2.44E-16	1.26E-15	1.37E-15	1.38E-15	1.35E-15	1.33E-15	1.44E-15	1.44E-15	1.22E-15	1.29E-15	1.02E-15
3.27E-19	1.17E-17	1.12E-17	6.59E-18	6.16E-18	4.93E-17	1.12E-16	1.98E-16	3.80E-16	4.32E-16	6.83E-16	1.08E-15	1.29E-15	1.45E-15	1.42E-15	1.35E-15	1.31E-15	1.49E-15	1.46E-15	1.35E-15	1.26E-15	1.00E-15
1.90E-18	9.80E-18	6.78E-18	2.97E-17	1.89E-18	5.79E-17	1.70E-16	2.63E-16	3.97E-16	5.96E-16	7.87E-16	1.12E-15	1.35E-15	1.57E-15	1.53E-15	1.43E-15	1.22E-15	1.55E-15	1.43E-15	1.33E-15	1.25E-15	9.78E-16
5.53E-18	5.45E-18	5.72E-18	3.77E-17	5.71E-18	4.70E-17	1.79E-16	2.78E-16	4.31E-16	7.05E-16	8.96E-16	1.11E-15	1.48E-15	1.71E-15	1.64E-15	1.58E-15	1.18E-15	1.62E-15	1.30E-15	1.35E-15	1.27E-15	1.07E-15
7.52E-18	7.92E-18	3.49E-18	3.91E-17	2.08E-17	5.29E-17	1.53E-16	2.70E-16	5.37E-16	7.14E-16	1.01E-15	1.14E-15	1.59E-15	1.70E-15	1.68E-15	1.71E-15	1.77E-15	1.68E-15	1.24E-15	1.49E-15	1.32E-15	1.23E-15
8.51E-18	1.12E-17	8.59E-18	3.86E-17	6.02E-17	6.46E-17	1.44E-16	2.52E-16	5.56E-16	7.01E-16	1.00E-15	1.24E-15	1.65E-15	1.71E-15	1.73E-15	1.90E-15	1.78E-15	1.62E-15	1.43E-15	1.51E-15	1.39E-15	1.23E-15
3.92E-18	2.77E-17	9.78E-18	5.98E-17	6.09E-17	5.99E-17	1.32E-16	3.15E-16	5.32E-16	6.86E-16	1.02E-15	1.35E-15	1.67E-15	1.68E-15	1.71E-15	1.97E-15	1.86E-15	1.55E-15	1.45E-15	1.45E-15	1.43E-15	1.33E-15
2.23E-18	2.38E-17	1.74E-17	6.78E-17	7.00E-17	8.78E-17	1.50E-16	3.19E-16	5.11E-16	6.96E-16	1.07E-15	1.39E-15	1.65E-15	1.73E-15	1.66E-15	2.08E-15	1.82E-15	1.51E-15	1.47E-15	1.48E-15	1.50E-15	1.48E-15
2.84E-18	2.53E-17	3.38E-17	7.25E-17	9.82E-17	1.29E-16	1.55E-16	3.03E-16	5.01E-16	7.80E-16	1.19E-15	1.35E-15	1.64E-15	1.66E-15	1.67E-15	2.04E-15	1.77E-15	1.47E-15	1.70E-15	1.44E-15	1.55E-15	1.44E-15
4.55E-18	3.09E-17	3.62E-17	8.09E-17	1.00E-16	1.31E-16	1.62E-16	3.11E-16	5.13E-16	7.75E-16	1.18E-15	1.27E-15	1.51E-15	1.50E-15	1.61E-15	1.84E-15	1.70E-15	1.53E-15	1.61E-15	1.33E-15	1.51E-15	1.32E-15
1.43E-17	2.57E-17	2.14E-17	8.72E-17	9.18E-17	1.53E-16	1.99E-16	3.19E-16	4.95E-16	7.34E-16	1.16E-15	1.19E-15	1.23E-15	1.20E-15	1.45E-15	1.55E-15	1.51E-15	1.31E-15	1.31E-15	1.19E-15	1.26E-15	1.24E-15
1.92E-17	2.43E-17	4.01E-17	1.20E-16	1.09E-16	1.43E-16	2.49E-16	3.08E-16	5.13E-16	6.07E-16	9.98E-16	9.39E-16	7.90E-16	8.85E-16	9.95E-16	1.06E-15	1.06E-15	8.87E-16	8.44E-16	8.36E-16	9.04E-16	1.16E-15
1.76E-17	1.50E-17	4.76E-17	1.31E-16	1.26E-16	1.31E-16	2.61E-16	3.26E-16	4.62E-16	4.11E-16	6.09E-16	4.89E-16	4.18E-16	3.64E-16	3.47E-16	4.17E-16	4.46E-16	3.17E-16	3.26E-16	3.92E-16	5.19E-16	7.97E-16
1.28E-17	1.39E-18	3.16E-17	1.25E-16	1.30E-16	1.21E-16	2.33E-16	2.49E-16	2.87E-16	1.98E-16	1.63E-16	5.51E-17	2.78E-17	3.21E-18	2.57E-19	8.82E-18	2.12E-17	1.36E-19	2.29E-18	2.01E-17	1.28E-16	3.29E-16
2.22E-18	1.57E-17	1.07E-17	9.87E-17	1.04E-16	6.01E-17	1.48E-16	1.01E-16	7.47E-17	5.82E-18	3.98E-17	1.83E-16	2.50E-16	3.68E-16	3.73E-16	2.98E-16	2.19E-16	3.65E-16	3.11E-16	1.81E-16	3.25E-17	2.95E-18
1.11E-18	1.07E-16	8.61E-18	5.79E-17	4.54E-17	1.15E-17	3.80E-17	2.27E-18	2.70E-17	3.84E-16	1.06E-15	1.49E-15	1.61E-15	1.80E-15	1.70E-15	1.52E-15	1.26E-15	1.50E-15	1.38E-15	1.01E-15	5.66E-16	2.44E-16
7.38E-18	2.97E-16	3.45E-18	1.75E-17	2.36E-18	1.04E-17	1.64E-17	1.27E-16	6.98E-16	2.41E-15	4.17E-15	4.58E-15	4.55E-15	4.63E-15	4.32E-15	3.80E-15	3.31E-15	3.51E-15	3.29E-15	2.57E-15	1.85E-15	1.15E-15
2.04E-18	2.15E-16	1.45E-17	2.70E-18	3.92E-17	2.77E-16	4.49E-16	1.13E-15	3.12E-15	6.89E-15	9.72E-15	9.81E-15	9.25E-15	9.14E-15	8.43E-15	7.32E-15	6.47E-15	6.68E-15	6.18E-15	5.08E-15	4.06E-15	2.90E-15
2.88E-17	2.84E-17	2.55E-18	9.39E-17	3.36E-16	1.07E-15	1.95E-15	3.95E-15	7.96E-15	1.37E-14	1.70E-14	1.66E-14	1.56E-14	1.54E-14	1.40E-14	1.24E-14	1.09E-14	1.11E-14	1.03E-14	8.67E-15	7.23E-15	5.66E-15
3.33E-17	3.67E-17	1.36E-17	3.43E-16	9.69E-16	2.39E-15	4.78E-15	8.85E-15	1.45E-14	2.14E-14	2.32E-14	2.36E-14	2.31E-14	2.32E-14	2.12E-14	1.90E-14	1.67E-14	1.66E-14	1.55E-14	1.35E-14	1.14E-14	9.55E-15
6.48E-17	2.44E-17	9.58E-17	7.25E-16	1.76E-15	3.97E-15	7.58E-15	1.34E-14	1.95E-14	2.58E-14	2.83E-14	3.11E-14	3.20E-14	3.26E-14	3.05E-14	2.69E-14	2.35E-14	2.31E-14	2.18E-14	1.96E-14	1.70E-14	1.47E-14
9.62E-17	2.82E-18	2.24E-16	1.13E-15	2.44E-15	5.35E-15	9.26E-15	1.47E-14	2.10E-14	2.77E-14	3.48E-14	4.01E-14	4.32E-14	4.43E-14	4.16E-14	3.59E-14	3.15E-14	3.07E-14	2.90E-14	2.67E-14	2.40E-14	2.15E-14
1.08E-16	8.51E-19	3.49E-16	1.44E-15	3.00E-15	6.34E-15	1.04E-14	1.60E-14	2.30E-14	3.13E-14	4.23E-14	5.12E-14	5.71E-14	5.91E-14	5.45E-14	4.59E-14	4.02E-14	3.91E-14	3.73E-14	3.47E-14	3.22E-14	3.06E-14
1.25E-16	4.74E-18	4.44E-16	1.65E-15	3.42E-15	6.77E-15	1.09E-14	1.70E-14	2.47E-14	3.47E-14	4.90E-14	6.35E-14	7.23E-14	7.51E-14	6.80E-14	5.65E-14	4.94E-14	4.78E-14	4.56E-14	4.30E-14	4.11E-14	4.17E-14
1.03E-16	6.67E-18	4.63E-16	1.72E-15	3.59E-15	6.86E-15	1.10E-14	1.74E-14	2.54E-14	3.62E-14	5.22E-14	7.23E-14	8.37E-14	8.74E-14	7.93E-14	6.66E-14	5.82E-14	5.66E-14	5.34E-14	5.04E-14	4.93E-14	5.27E-14
8.93E-17	6.17E-18	4.16E-16	1.61E-15	3.43E-15	6.65E-15	1.06E-14	1.70E-14	2.49E-14	3.56E-14	5.16E-14	7.12E-14	8.32E-14	8.91E-14	8.45E-14	7.52E-14	6.65E-14	6.46E-14	5.97E-14	5.59E-14	5.50E-14	6.09E-14
9.45E-17	1.41E-18	3.16E-16	1.32E-15	3.03E-15	6.07E-15	9.71E-15	1.56E-14	2.33E-14	3.31E-14	4.76E-14	6.52E-14	7.92E-14	8.60E-14	8.62E-14	8.31E-14	7.49E-14	7.22E-14	6.46E-14	5.95E-14	5.67E-14	6.15E-14
1.09E-16	3.23E-18	1.90E-16	9.15E-16	2.42E-15	5.03E-15	8.44E-15	1.36E-14	2.08E-14	2.94E-14	4.23E-14	6.03E-14	7.56E-14	8.18E-14	8.62E-14	8.86E-14	8.37E-14	7.91E-14	6.81E-14	6.12E-14	5.68E-14	5.62E-14
1.25E-16	3.08E-17	8.29E-17	5.30E-16	1.76E-15	3.78E-15	6.90E-15	1.13E-14	1.78E-14	2.53E-14	3.73E-14	5.52E-14	7.23E-14	7.68E-14	8.56E-14	9.28E-14	9.11E-14	8.44E-14	6.89E-14	6.10E-14	5.67E-14	5.17E-14
1.33E-16	8.29E-17	2.70E-17	2.12E-16	1.15E-15	2.50E-15	5.20E-15	8.96E-15	1.46E-14	2.14E-14	3.20E-14	4.90E-14	6.90E-14	7.31E-14	8.44E-14	8.87E-14	9.12E-14	8.34E-14	6.76E-14	5.89E-14	5.54E-14	4.82E-14
1.43E-16	1.36E-16	1.94E-18	2.87E-17	5.73E-16	1.44E-15	3.67E-15	6.76E-15	1.14E-14	1.76E-14	2.67E-14	4.25E-14	6.38E-14	6.69E-14	7.35E-14	7.74E-14	8.33E-14	7.25E-14	6.53E-14	5.48E-14	5.17E-14	4.25E-14
1.67E-16	1.71E-16	3.82E-17	1.25E-17	8.84E-17	5.95E-16	2.25E-15	4.80E-15	8.43E-15	1.40E-14	2.10E-14	3.52E-14	5.16E-14	5.26E-14	5.61E-14	6.43E-14	6.70E-14	5.70E-14	6.02E-14	4.88E-14	4.54E-14	3.49E-14
1.97E-16	2.06E-16	2.16E-16	1.38E-16	3.63E-18	8.68E-17	8.66E-16	2.99E-15	5.68E-15	1.06E-14	1.48E-14	2.17E-14	3.14E-14	3.25E-14	4.02E-14	4.74E-14	4.86E-14	4.46E-14	4.36E-14	4.10E-14	3.56E-14	2.65E-14
2.52E-16	2.46E-16	2.62E-16	1.71E-16	1.04E-16	2.89E-17	1.14E-16	1.41E-15	3.47E-15	7.62E-15	9.15E-15	1.14E-14	1.68E-14	2.14E-14	2.93E-14	2.76E-14	3.22E-14	3.44E-14	3.01E-14	2.99E-14	2.72E-14	2.03E-14
2.65E-16	2.39E-16	3.01E-16	1.94E-16	1.45E-16	1.83E-16	1.72E-17	2.97E-16	1.71E-15	4.77E-15	5.52E-15	6.86E-15	1.02E-14	1.46E-14	1.90E-14	1.69E-14	2.01E-14	2.36E-14	2.11E-14	2.07E-14	1.81E-14	1.53E-14
2.42E-16	2.38E-16	3.17E-16	2.27E-16	1.70E-16	2.58E-16	2.21E-16	9.63E-18	3.08E-16	2.15E-15	2.82E-15	4.00E-15	6.91E-15	9.31E-15	1.16E-14	1.05E-14	1.44E-14	1.61E-14	1.48E-14	1.45E-14	1.26E-14	1.18E-14
2.26E-16	2.60E-16	2.93E-16	2.42E-16	2.30E-16	3.18E-16	3.61E-16	1.92E-16	1.02E-17	4.88E-16	1.23E-15	1.95E-15	4.06E-15	5.09E-15	6.12E-15	5.73E-15	9.35E-15	1.01E-14	1.05E-14	1.06E-14	9.28E-15	9.14E-15
2.66E-16	2.62E-16	2.99E-16	3.04E-16	2.78E-16	3.68E-16	3.81E-16	3.91E-16	2.74E-16	9.96E-18	3.74E-16	6.54E-16	1.82E-15	2.41E-15	3.08E-15	2.95E-15	5.11E-15	6.12E-15	7.19E-15	7.33E-15	7.07E-15	7.19E-15
2.96E-16	2.98E-16	3.32E-16	4.11E-16	3.47E-16	4.84E-16	4.79E-16	4.94E-16	6.54E-16	2.12E-16	9.62E-20	5.94E-17	5.30E-16	8.82E-16	1.33E-15	1.30E-15	2.59E-15	3.38E-15	4.40E-15	4.93E-15	5.18E-15	5.55E-15
3.49E-16	3.96E-16	3.54E-16	4.81E-16	3.43E-16	5.90E-16	6.27E-16	6.25E-16	8.06E-16	7.62E-16	3.57E-16	1.30E-16	4.17E-17	1.41E-16	3.00E-16	3.88E-16	1.04E-15	1.69E-15	2.60E-15	3.19E-15	3.64E-15	4.20E-15
3.69E-16	4.06E-16	3.69E-16	5.11E-16	3.47E-16	6.30E-16	7.59E-16	7.37E-16	9.35E-16	1.21E-15	1.11E-15	6.01E-16	1.32E-16	2.51E-17	2.10E-19	1.06E-17	2.50E-16	6.84E-16	1.63E-15	2.09E-15	2.45E-15	2.91E-15
3.54E-16	3.95E-16	3.39E-16	5.73E-16	4.23E-16	6.74E-16	8.03E-16	7.75E-16	1.08E-15	1.37E-15	1.20E-15	1.57E-15	9.02E-16	2.94E-16	2.38E-16	1.49E-16	5.38E-19	1.90E-16	7.30E-16	1.00E-15	1.35E-15	1.67E-15
3.48E-16	4.01E-16	4.10E-16	5.85E-16	5.85E-16	6.26E-16	8.16E-16	8.22E-16	1.16E-15	1.47E-15	1.35E-15	1.82E-15	1.95E-15	7.18E-16	7.87E-16	5.26E-16	1.80E-16	2.16E-18	1.61E-16	3.23E-16	4.86E-16	6.64E-16
3.80E-16	4.06E-16	5.00E-16	6.35E-16	6.03E-16	6.81E-16	8.72E-16	9.25E-16	1.27E-15	1.49E-15	1.56E-15	2.03E-15	2.01E-15	1.38E-15	1.42E-15	7.72E-16	6.12E-16	1.26E-16	1.09E-17	2.58E-20	4.32E-17	8.28E-17
4.54E-16	4.16E-16	5.58E-16	6.55E-16	6.44E-16	7.32E-16	9.64E-16	1.11E-15	1.32E-15	1.46E-15	1.77E-15	2.07E-15	2.09E-15	2.19E-15	2.15E-15	1.13E-15	8.91E-16	9.81E-16	5.71E-16	1.77E-16	6.48E-17	4.08E-17
5.00E-16	4.68E-16	5.76E-16	7.12E-16	7.50E-16	7.71E-16	1.00E-15	1.14E-15	1.31E-15	1.49E-15	1.80E-15	2.13E-15	2.26E-15	2.21E-15	2.28E-15	2.23E-15	1.45E-15	1.87E-15	1.37E-15	4.67E-16	4.63E-16	4.50E-16
5.23E-16	4.67E-16	5.91E-16	7.79E-16	7.73E-16	8.39E-16	1.03E-15	1.08E-15	1.29E-15	1.52E-15	1.84E-15	2.19E-15	2.29E-15	2.19E-15	2.38E-15	2.29E-15	2.45E-15	2.60E-15	2.31E-15	9.81E-16	1.35E-15	1.17E-15
5.34E-16	4.71E-16	5.43E-16	8.55E-16	7.21E-16	8.72E-16	1.05E-15	1.03E-15	1.22E-15	1.48E-15	1.81E-15	2.16E-15	2.35E-15	2.13E-15	2.35E-15	2.20E-15	2.45E-15	2.58E-15	2.58E-15	2.39E-15	1.94E-15	1.91E-15
5.09E-16	4.53E-16	5.17E-16	7.80E-16	7.02E-16	9.22E-16	1.09E-15	1.09E-15	1.21E-15	1.50E-15	1.81E-15	2.14E-15	2.43E-15	2.19E-15	2.31E-15	2.24E-15	2.42E-15	2.69E-15	2.51E-15	2.42E-15	1.98E-15	2.38E-15
4.86E-16	4.36E-16	5.63E-16	6.92E-16	6.77E-16	8.98E-16	1.10E-15	1.23E-15	1.19E-15	1.57E-15	1.78E-15	2.04E-15	2.45E-15	2.21E-15	2.30E-15	2.33E-15	2.40E-15	2.74E-15	2.43E-15	2.35E-15	1.87E-15	2.38E-15
4.43E-16	4.36E-16	6.14E-16	6.20E-16	7.39E-16	7.92E-16	1.13E-15	1.20E-15	1.18E-15	1.59E-15	1.75E-15	1.99E-15	2.40E-15	2.08E-15	2.23E-15	2.31E-15	2.40E-15	2.59E-15	2.55E-15	2.30E-15	1.81E-15	2.43E-15
3.79E-16	4.51E-16	6.47E-16	5.85E-16	7.30E-16	8.06E-16	1.09E-15	1.20E-15	1.16E-15	1.55E-15	1.76E-15	1.96E-15	2.32E-15	2.08E-15	2.10E-15	2.24E-15	2.40E-15	2.53E-15	2.74E-15	2.38E-15	1.48E-15	2.45E-15
2.99E-16	4.84E-16	5.98E-16	6.38E-16	7.38E-16	8.79E-16	1.14E-15	1.23E-15	1.17E-15	1.57E-15	1.71E-15	2.05E-15	2.26E-15	2.16E-15	2.08E-15	2.19E-15	2.31E-15	2.62E-15	2.78E-15	2.41E-15	1.23E-15	2.59E-15

2.63E-16	5.76E-16	5.71E-16	6.70E-16	7.77E-16	9.27E-16	1.13E-15	1.28E-15	1.17E-15	1.61E-15	1.66E-15	2.04E-15	2.22E-15	2.32E-15	2.32E-15	2.37E-15	2.29E-15	2.80E-15	2.71E-15	2.36E-15	2.14E-15	2.64E-15
3.08E-16	6.39E-16	6.21E-16	6.87E-16	8.03E-16	9.28E-16	1.09E-15	1.29E-15	1.23E-15	1.57E-15	1.77E-15	1.96E-15	2.14E-15	2.45E-15	2.42E-15	2.39E-15	2.37E-15	2.81E-15	2.59E-15	2.45E-15	2.34E-15	2.46E-15
4.90E-16	6.66E-16	7.21E-16	7.19E-16	8.49E-16	9.78E-16	1.07E-15	1.32E-15	1.29E-15	1.51E-15	1.88E-15	2.00E-15	2.15E-15	2.45E-15	2.36E-15	2.31E-15	2.43E-15	2.77E-15	2.51E-15	2.47E-15	1.93E-15	2.25E-15
5.16E-16	6.72E-16	7.41E-16	8.32E-16	9.07E-16	1.04E-15	1.09E-15	1.39E-15	1.29E-15	1.56E-15	1.84E-15	2.03E-15	2.17E-15	2.51E-15	2.32E-15	2.27E-15	2.54E-15	2.78E-15	2.56E-15	2.41E-15	1.60E-15	2.17E-15
4.91E-16	6.51E-16	7.82E-16	9.35E-16	8.52E-16	1.08E-15	1.07E-15	1.42E-15	1.22E-15	1.73E-15	1.79E-15	2.20E-15	2.23E-15	2.65E-15	2.43E-15	2.41E-15	2.56E-15	2.71E-15	2.56E-15	2.26E-15	1.93E-15	2.27E-15
5.26E-16	6.56E-16	7.47E-16	9.57E-16	8.66E-16	1.05E-15	1.03E-15	1.37E-15	1.19E-15	1.75E-15	1.84E-15	2.25E-15	2.24E-15	2.61E-15	2.47E-15	2.48E-15	2.63E-15	2.61E-15	2.58E-15	2.30E-15	2.60E-15	2.23E-15
5.16E-16	6.66E-16	8.76E-16	9.37E-16	9.25E-16	1.04E-15	1.06E-15	1.33E-15	1.13E-15	1.74E-15	2.03E-15	2.25E-15	2.19E-15	2.53E-15	2.37E-15	2.49E-15	2.69E-15	2.61E-15	2.66E-15	2.47E-15	2.77E-15	2.27E-15
4.99E-16	7.29E-16	8.90E-16	8.68E-16	9.78E-16	1.03E-15	1.08E-15	1.28E-15	1.05E-15	1.71E-15	2.12E-15	2.23E-15	2.19E-15	2.50E-15	2.52E-15	2.41E-15	2.69E-15	2.65E-15	2.71E-15	2.72E-15	2.83E-15	2.31E-15
5.34E-16	7.13E-16	8.24E-16	8.74E-16	1.01E-15	9.79E-16	1.06E-15	1.25E-15	1.29E-15	1.67E-15	2.05E-15	2.24E-15	2.20E-15	2.53E-15	2.51E-15	2.45E-15	2.84E-15	2.70E-15	2.67E-15	2.83E-15	2.85E-15	2.27E-15
5.55E-16	6.62E-16	7.48E-16	8.66E-16	1.02E-15	9.61E-16	1.05E-15	1.17E-15	1.42E-15	1.63E-15	1.97E-15	2.26E-15	2.27E-15	2.63E-15	2.59E-15	2.46E-15	2.96E-15	2.66E-15	2.52E-15	2.71E-15	2.78E-15	2.11E-15
6.32E-16	6.10E-16	7.32E-16	8.50E-16	1.02E-15	1.01E-15	1.05E-15	1.16E-15	1.37E-15	1.60E-15	2.07E-15	2.27E-15	2.24E-15	2.61E-15	2.64E-15	2.43E-15	2.87E-15	2.55E-15	2.43E-15	2.56E-15	2.69E-15	2.27E-15
7.40E-16	5.88E-16	7.65E-16	8.12E-16	9.76E-16	1.09E-15	9.89E-16	1.18E-15	1.40E-15	1.46E-15	2.14E-15	2.24E-15	2.26E-15	2.44E-15	2.77E-15	2.55E-15	2.82E-15	2.61E-15	2.44E-15	2.51E-15	2.45E-15	2.61E-15
7.43E-16	5.82E-16	6.96E-16	7.57E-16	9.10E-16	1.08E-15	9.29E-16	1.25E-15	1.38E-15	1.20E-15	2.00E-15	2.38E-15	2.38E-15	2.29E-15	2.71E-15	2.44E-15	2.76E-15	2.73E-15	2.41E-15	2.56E-15	2.36E-15	2.56E-15
7.59E-16	6.15E-16	6.59E-16	7.63E-16	9.05E-16	1.03E-15	9.92E-16	1.27E-15	1.30E-15	1.13E-15	1.92E-15	2.45E-15	2.35E-15	2.32E-15	2.59E-15	2.29E-15	2.65E-15	2.73E-15	2.42E-15	2.67E-15	2.43E-15	2.46E-15
8.03E-16	6.28E-16	6.81E-16	8.50E-16	8.78E-16	9.94E-16	9.82E-16	1.27E-15	1.26E-15	1.40E-15	2.02E-15	2.39E-15	2.17E-15	2.35E-15	2.50E-15	2.30E-15	2.64E-15	2.70E-15	2.52E-15	2.69E-15	2.54E-15	2.35E-15
8.41E-16	6.61E-16	7.75E-16	8.95E-16	8.76E-16	9.51E-16	9.45E-16	1.26E-15	1.22E-15	1.63E-15	2.16E-15	2.34E-15	2.13E-15	2.33E-15	2.52E-15	2.33E-15	2.69E-15	2.73E-15	2.42E-15	2.63E-15	2.44E-15	2.44E-15
8.39E-16	7.12E-16	8.51E-16	9.11E-16	9.47E-16	8.84E-16	8.99E-16	1.21E-15	1.33E-15	1.58E-15	2.14E-15	2.28E-15	2.29E-15	2.34E-15	2.48E-15	2.41E-15	2.65E-15	2.68E-15	2.42E-15	2.58E-15	2.35E-15	2.69E-15
4.02E-14	3.92E-14	4.56E-14	5.73E-14	6.68E-14	9.99E-14	1.48E-13	2.22E-13	3.22E-13	4.63E-13	6.30E-13	8.29E-13	1.01E-12	1.07E-12	1.10E-12	1.07E-12	1.04E-12	1.00E-12	9.18E-13	8.36E-13	7.77E-13	7.29E-13

2.61E-09	5.97E-09	8.53E-09	1.63E-08	2.12E-08	2.67E-08	3.18E-08	3.21E-08	3.44E-08	3.89E-08	3.65E-08	3.34E-08	2.72E-08	3.41E-08	3.61E-08	3.46E-08	4.23E-08	4.23E-08	3.92E-08	4.67E-08
9.58E-10	5.96E-09	1.02E-08	1.64E-08	2.01E-08	2.56E-08	3.07E-08	3.14E-08	3.45E-08	3.93E-08	3.76E-08	3.40E-08	2.63E-08	3.36E-08	3.54E-08	3.39E-08	3.37E-08	3.42E-08	3.88E-08	4.62E-08
1.37E-09	5.06E-09	1.12E-08	1.65E-08	1.99E-08	2.51E-08	3.00E-08	3.08E-08	3.54E-08	3.92E-08	3.82E-08	3.50E-08	2.61E-08	3.29E-08	3.69E-08	3.42E-08	3.37E-08	3.45E-08	3.87E-08	4.30E-08
2.85E-09	3.87E-09	1.24E-08	1.60E-08	1.93E-08	2.58E-08	2.95E-08	3.01E-08	3.56E-08	3.89E-08	3.71E-08	3.41E-08	2.62E-08	3.21E-08	3.68E-08	3.47E-08	3.36E-08	3.48E-08	3.97E-08	4.18E-08
4.31E-09	4.28E-09	1.55E-08	1.41E-08	1.91E-08	2.77E-08	2.96E-08	2.92E-08	3.29E-08	3.81E-08	3.54E-08	3.22E-08	2.63E-08	3.12E-08	3.38E-08	3.42E-08	3.53E-08	3.75E-08	4.04E-08	4.05E-08
3.90E-09	3.91E-09	9.35E-09	1.37E-08	1.84E-08	2.83E-08	2.85E-08	2.77E-08	2.99E-08	3.59E-08	3.41E-08	3.10E-08	2.61E-08	3.07E-08	3.33E-08	3.29E-08	3.74E-08	3.84E-08	3.97E-08	4.00E-08
3.75E-09	3.55E-09	4.58E-09	1.38E-08	1.67E-08	2.43E-08	2.71E-08	2.78E-08	2.84E-08	3.54E-08	3.23E-08	3.00E-08	2.52E-08	3.01E-08	3.31E-08	3.28E-08	3.90E-08	3.45E-08	3.99E-08	3.92E-08
5.90E-09	2.47E-09	4.45E-09	1.51E-08	1.56E-08	2.17E-08	2.67E-08	2.89E-08	2.88E-08	3.47E-08	3.21E-08	2.85E-08	2.56E-08	3.00E-08	3.32E-08	3.22E-08	3.29E-08	3.44E-08	4.19E-08	3.73E-08
1.05E-08	2.02E-09	3.53E-09	1.51E-08	1.48E-08	2.12E-08	2.66E-08	2.93E-08	2.89E-08	3.40E-08	3.05E-08	2.72E-08	2.48E-08	2.96E-08	3.09E-08	3.21E-08	3.16E-08	3.55E-08	4.12E-08	3.51E-08
1.53E-08	1.62E-09	3.80E-09	1.24E-08	1.47E-08	2.05E-08	2.66E-08	2.93E-08	2.95E-08	3.23E-08	2.95E-08	2.66E-08	2.48E-08	2.85E-08	2.99E-08	3.20E-08	3.15E-08	3.83E-08	3.68E-08	3.47E-08
9.75E-09	2.90E-09	3.30E-09	1.28E-08	1.52E-08	2.01E-08	2.81E-08	3.10E-08	3.08E-08	3.24E-08	2.95E-08	2.64E-08	2.53E-08	2.87E-08	3.01E-08	3.10E-08	3.19E-08	3.15E-08	3.60E-08	3.38E-08
3.91E-09	3.71E-09	3.50E-09	1.41E-08	1.48E-08	2.04E-08	3.14E-08	3.58E-08	3.42E-08	3.30E-08	2.92E-08	2.55E-08	2.53E-08	2.94E-08	2.98E-08	3.11E-08	3.25E-08	3.37E-08	3.69E-08	3.31E-08
3.84E-09	3.46E-09	4.43E-09	1.00E-08	1.46E-08	2.34E-08	3.67E-08	4.36E-08	3.84E-08	3.28E-08	2.79E-08	2.34E-08	2.28E-08	2.92E-08	2.93E-08	3.01E-08	2.84E-08	3.72E-08	3.56E-08	3.35E-08
3.38E-09	1.13E-09	5.87E-09	1.06E-08	1.44E-08	2.71E-08	4.40E-08	5.26E-08	4.45E-08	3.26E-08	2.58E-08	2.21E-08	2.17E-08	2.88E-08	2.86E-08	2.82E-08	3.00E-08	3.58E-08	3.49E-08	3.39E-08
7.25E-10	-3.91E-10	5.42E-09	1.02E-08	1.23E-08	2.91E-08	4.58E-08	5.43E-08	4.72E-08	3.20E-08	2.55E-08	2.16E-08	2.27E-08	2.76E-08	2.91E-08	2.68E-08	2.83E-08	2.58E-08	3.34E-08	3.43E-08
-2.86E-09	-1.35E-09	7.42E-09	9.01E-09	1.16E-08	2.70E-08	4.00E-08	4.74E-08	4.29E-08	2.96E-08	2.55E-08	2.11E-08	2.19E-08	2.66E-08	2.92E-08	2.63E-08	2.80E-08	2.69E-08	3.38E-08	3.44E-08
-3.64E-09	-3.81E-10	1.01E-08	8.12E-09	1.24E-08	2.21E-08	3.01E-08	3.71E-08	3.62E-08	2.58E-08	2.56E-08	2.09E-08	2.27E-08	2.48E-08	2.71E-08	2.59E-08	2.97E-08	2.81E-08	3.32E-08	3.43E-08
-3.20E-09	-4.92E-10	1.07E-08	7.51E-09	1.23E-08	1.36E-08	2.02E-08	2.62E-08	2.53E-08	2.16E-08	2.60E-08	2.14E-08	2.40E-08	2.45E-08	2.65E-08	2.60E-08	2.99E-08	3.15E-08	3.35E-08	3.47E-08
-3.14E-09	-2.00E-09	1.12E-10	6.89E-09	1.09E-08	1.36E-08	1.68E-08	1.82E-08	1.68E-08	2.05E-08	2.62E-08	2.07E-08	2.39E-08	2.43E-08	2.68E-08	2.60E-08	3.10E-08	3.73E-08	3.41E-08	3.45E-08
-4.63E-09	-3.52E-09	-2.97E-11	4.44E-09	1.02E-08	1.37E-08	1.81E-08	1.74E-08	1.77E-08	2.21E-08	2.52E-08	2.01E-08	2.45E-08	2.37E-08	2.64E-08	2.56E-08	3.32E-08	3.71E-08	3.59E-08	3.68E-08
-6.04E-09	-3.66E-09	-1.64E-09	4.68E-09	1.04E-08	1.58E-08	2.07E-08	1.82E-08	1.83E-08	2.44E-08	2.42E-08	1.96E-08	2.38E-08	2.34E-08	2.45E-08	2.65E-08	3.19E-08	3.66E-08	3.56E-08	3.60E-08
-6.63E-09	-3.82E-09	-3.28E-09	3.91E-09	9.30E-09	1.87E-08	2.48E-08	1.85E-08	1.84E-08	2.69E-08	2.24E-08	2.03E-08	2.34E-08	2.28E-08	2.47E-08	2.79E-08	2.58E-08	3.32E-08	3.04E-08	2.90E-08
-7.21E-09	-6.74E-09	-3.80E-09	4.75E-09	7.75E-09	2.17E-08	2.97E-08	1.78E-08	1.88E-08	2.78E-08	2.19E-08	2.06E-08	2.35E-08	2.30E-08	2.58E-08	2.97E-08	2.58E-08	2.76E-08	2.68E-08	2.82E-08
-8.11E-09	-8.88E-09	-4.22E-09	4.37E-09	7.49E-09	2.11E-08	2.95E-08	1.53E-08	1.85E-08	2.17E-08	2.20E-08	1.98E-08	2.26E-08	2.23E-08	2.55E-08	2.81E-08	2.74E-08	2.85E-08	2.73E-08	2.79E-08
-8.39E-09	-8.31E-09	-4.31E-09	2.46E-09	7.23E-09	1.35E-08	1.77E-08	1.44E-08	1.65E-08	2.13E-08	2.28E-08	1.86E-08	2.14E-08	2.21E-08	2.28E-08	2.46E-08	2.68E-08	2.66E-08	2.58E-08	2.68E-08
-8.86E-09	-7.66E-09	-4.42E-09	2.38E-09	6.36E-09	8.15E-09	1.03E-08	1.52E-08	1.66E-08	2.20E-08	2.37E-08	1.72E-08	2.05E-08	2.14E-08	2.17E-08	2.36E-08	2.71E-08	2.64E-08	2.41E-08	2.79E-08
-9.78E-09	-7.78E-09	-4.61E-09	3.04E-09	5.19E-09	7.57E-09	9.93E-09	1.56E-08	1.64E-08	2.16E-08	2.38E-08	1.54E-08	1.95E-08	2.15E-08	1.98E-08	2.26E-08	2.38E-08	3.04E-08	2.35E-08	2.85E-08
-9.83E-09	-9.54E-09	-5.63E-09	1.81E-09	4.46E-09	6.54E-09	9.76E-09	1.45E-08	1.60E-08	1.83E-08	2.19E-08	1.56E-08	1.92E-08	2.04E-08	1.91E-08	1.93E-08	2.04E-08	2.57E-08	2.31E-08	2.62E-08
-9.97E-09	-9.35E-09	-4.49E-09	-2.93E-10	4.12E-09	7.03E-09	8.89E-09	1.24E-08	1.81E-08	1.90E-08	2.10E-08	1.58E-08	1.87E-08	1.73E-08	1.85E-08	1.83E-08	2.22E-08	2.54E-08	2.32E-08	2.26E-08
-1.12E-08	-7.34E-09	-4.58E-09	-9.53E-10	3.78E-09	8.30E-09	8.36E-09	1.07E-08	2.23E-08	1.95E-08	1.86E-08	1.60E-08	1.84E-08	1.67E-08	1.80E-08	1.74E-08	2.40E-08	2.51E-08	2.39E-08	2.16E-08
-1.20E-08	-5.72E-09	-3.67E-09	-1.01E-09	3.85E-09	9.33E-09	9.19E-09	1.06E-08	2.67E-08	1.93E-08	1.71E-08	1.64E-08	1.77E-08	1.65E-08	1.55E-08	1.73E-08	2.48E-08	2.52E-08	2.35E-08	2.10E-08
-1.32E-08	-7.27E-09	-2.57E-09	-3.44E-10	3.93E-09	7.26E-09	8.79E-09	1.10E-08	2.43E-08	1.68E-08	1.68E-08	1.73E-08	1.71E-08	1.65E-08	1.44E-08	1.79E-08	2.49E-08	2.83E-08	2.44E-08	2.00E-08
-1.39E-08	-1.01E-08	-4.18E-09	6.69E-10	3.15E-09	4.01E-09	7.06E-09	1.11E-08	1.10E-08	1.43E-08	1.71E-08	1.60E-08	1.72E-08	1.74E-08	1.46E-08	1.72E-08	1.75E-08	2.59E-08	2.63E-08	2.02E-08
-1.43E-08	-1.18E-08	-9.78E-09	1.23E-09	1.93E-09	3.80E-09	6.23E-09	1.16E-08	1.05E-08	1.40E-08	1.68E-08	1.45E-08	1.65E-08	1.67E-08	1.50E-08	1.53E-08	1.92E-08	2.86E-08	2.71E-08	2.08E-08
-1.38E-08	-1.17E-08	-9.30E-09	2.81E-09	3.36E-10	3.52E-09	5.88E-09	1.12E-08	9.63E-09	1.24E-08	1.57E-08	1.59E-08	1.69E-08	1.68E-08	1.45E-08	1.46E-08	2.03E-08	2.29E-08	2.26E-08	2.06E-08
-1.41E-08	-1.26E-08	-1.04E-08	5.66E-09	-5.52E-10	2.58E-09	5.47E-09	1.00E-08	9.93E-09	1.17E-08	1.35E-08	1.40E-08	1.69E-08	1.61E-08	1.27E-08	1.37E-08	1.94E-08	1.91E-08	1.91E-08	1.90E-08
-1.57E-08	-1.31E-08	-9.66E-09	7.38E-09	-2.18E-09	1.61E-09	5.05E-09	1.01E-08	1.11E-08	1.10E-08	1.19E-08	1.19E-08	1.58E-08	1.51E-08	1.13E-08	1.17E-08	1.73E-08	1.23E-08	1.64E-08	1.76E-08
-1.62E-08	-1.25E-08	-9.73E-09	-2.86E-09	-3.41E-09	1.41E-09	3.70E-09	1.02E-08	1.38E-08	1.20E-08	1.09E-08	1.11E-08	1.45E-08	1.27E-08	1.12E-08	1.09E-08	2.10E-08	1.10E-08	1.59E-08	1.80E-08
-1.63E-08	-1.26E-08	-9.00E-09	-1.67E-09	-3.92E-09	9.23E-10	2.38E-09	8.37E-09	1.73E-08	1.29E-08	9.93E-09	1.00E-08	1.35E-08	1.19E-08	1.04E-08	1.04E-08	2.59E-08	1.03E-08	1.44E-08	1.79E-08
-1.65E-08	-1.43E-08	-7.14E-09	-1.07E-09	-4.27E-09	-2.80E-10	2.49E-09	7.77E-09	2.30E-08	1.47E-08	9.94E-09	9.23E-09	1.21E-08	1.11E-08	9.94E-09	9.62E-09	2.57E-08	1.10E-08	1.31E-08	1.65E-08
-1.66E-08	-1.43E-08	-5.45E-09	-6.59E-09	-4.49E-09	-1.53E-09	2.61E-09	6.70E-09	2.11E-08	1.81E-08	9.87E-09	7.61E-09	1.14E-08	1.07E-08	9.27E-09	8.95E-09	1.39E-08	1.03E-08	1.24E-08	1.48E-08
-1.75E-08	-1.37E-08	-2.17E-09	-8.43E-09	-4.10E-09	-2.03E-09	2.51E-09	6.65E-09	1.97E-08	1.99E-08	8.38E-09	8.52E-09	1.14E-08	1.06E-08	7.92E-09	9.46E-09	1.43E-08	1.06E-08	1.19E-08	1.47E-08
-1.86E-08	-1.50E-08	-6.43E-09	-8.95E-09	-5.01E-09	-1.49E-09	2.00E-09	5.72E-09	1.19E-08	1.09E-08	7.90E-09	9.06E-09	1.22E-08	9.45E-09	8.41E-09	1.08E-08	1.27E-08	1.04E-08	1.23E-08	1.44E-08
-1.82E-08	-1.61E-08	-1.06E-08	-9.75E-09	-5.14E-09	-1.72E-09	1.59E-09	4.98E-09	3.81E-09	9.60E-09	7.17E-09	5.72E-09	1.25E-08	8.19E-09	8.35E-09	1.08E-08	1.18E-08	1.04E-08	1.15E-08	1.41E-08
-1.82E-08	-1.72E-08	-9.62E-09	-8.93E-09	-5.20E-09	-2.31E-09	1.11E-09	4.02E-09	3.40E-09	1.14E-08	6.41E-09	3.54E-09	1.19E-08	7.56E-09	7.87E-09	1.23E-08	1.08E-08	9.22E-09	1.05E-08	1.38E-08
-1.95E-08	-1.63E-08	-7.21E-09	-9.02E-09	-5.00E-09	-2.98E-09	1.92E-10	3.40E-09	4.02E-09	1.36E-08	6.66E-09	4.26E-09	6.89E-09	4.96E-09	7.78E-09	1.65E-08	1.17E-08	9.43E-09	1.02E-08	1.37E-08
-2.07E-08	-1.69E-08	-1.10E-08	-9.32E-09	-5.59E-09	-2.90E-09	4.08E-10	2.84E-09	5.96E-09	7.11E-09	6.31E-09	5.52E-09	5.89E-09	2.55E-09	9.15E-09	2.20E-08	1.39E-08	1.27E-08	1.09E-08	1.18E-08
-2.16E-08	-2.02E-08	-1.57E-08	-9.07E-09	-6.75E-09	-3.19E-09	-8.40E-11	2.30E-09	6.08E-09	5.04E-09	5.88E-09	4.33E-09	5.95E-09	2.24E-09	7.84E-09	2.40E-08	1.57E-08	9.63E-09	1.08E-08	1.01E-08
-2.38E-08	-2.07E-08	-1.39E-08	-9.10E-09	-7.14E-09	-4.06E-09	-7.13E-10	1.87E-09	1.89E-09	3.94E-09	4.72E-09	3.85E-09	5.72E-09	2.74E-09	3.78E-09	2.13E-08	1.50E-08	4.85E-09	1.01E-08	9.64E-09
-2.49E-08	-2.06E-08	-1.33E-08	-1.01E-08	-7.36E-09	-5.13E-09	-2.29E-09	2.57E-10	2.14E-09	4.61E-09	4.13E-09	4.30E-09	5.25E-09	2.34E-09	2.24E-09	1.06E-08	7.49E-09	6.58E-09	1.20E-08	9.80E-09
-2.59E-08	-2.09E-08	-1.46E-08	-1.08E-08	-7.16E-09	-6.15E-09	-3.65E-09	1.33E-10	2.38E-09	4.44E-09	3.81E-09	2.40E-09	5.76E-09	1.63E-09	2.57E-09	1.42E-08	7.39E-09	1.15E-08	1.43E-08	8.45E-09
-2.53E-08	-2.17E-08	-1.39E-08	-1.13E-08	-7.32E-09	-5.14E-09	-3.31E-09	-6.76E-10	6.85E-10	5.61E-09	1.93E-09	2.45E-09	5.51E-09	1.35E-09	2.59E-09	1.59E-08	7.98E-09	1.17E-08	7.98E-09	8.09E-09
-2.49E-08	-2.22E-08	-1.49E-08	-1.16E-08	-7.40E-09	-4.44E-09	-3.33E-09	-1.31E-09	-5.10E-10	6.26E-09	1.56E-09	1.79E-09	4.80E-09	1.73E-09	1.82E-09	1.05E-08	6.54E-09	4.80E-09	8.89E-09	8.86E-09
-2.55E-08	-2.18E-08	-1.18E-08	-1.30E-08	-7.45E-09	-4.49E-09	-3.56E-09	-2.07E-09	-1.21E-10	6.43E-09	1.65E-09	4.13E-10	3.13E-09	1.38E-09	1.54E-09	4.10E-11	5.43E-09	6.10E-09	8.84E-09	9.88E-09
-2.71E-08	-2.49E-08	-1.67E-08	-1.29E-08	-7.36E-09	-4.31E-09	-2.77E-09	-2.47E-09	4.96E-11	2.24E-09	1.10E-09	-2.98E-11	3.26E-09	1.11E-09	3.38E-10	-2.65E-10	4.32E-09	4.16E-09	9.22E-09	1.21E-08

-2.84E-08	-2.66E-08	-2.08E-08	-1.35E-08	-6.48E-09	-3.31E-09	-1.47E-09	-3.16E-09	5.00E-10	2.50E-09	-2.44E-10	-1.85E-10	3.46E-09	1.61E-09	-3.64E-10	-7.82E-10	2.14E-09	1.35E-09	1.01E-08	1.42E-08
-2.80E-08	-2.72E-08	-2.18E-08	-1.31E-08	-4.90E-09	-2.71E-09	-6.43E-10	-3.86E-09	1.11E-09	1.30E-09	-1.22E-09	3.71E-10	3.44E-09	1.94E-09	1.01E-10	-1.82E-09	-1.35E-09	1.70E-09	1.01E-08	1.59E-08
-2.98E-08	-2.68E-08	-2.20E-08	-1.26E-08	-4.65E-09	-2.82E-09	-6.30E-10	-3.46E-09	8.24E-10	-6.45E-10	-9.51E-10	1.63E-09	2.82E-09	2.61E-09	1.61E-09	-1.84E-09	1.78E-10	4.10E-09	7.55E-09	1.21E-08
-3.23E-08	-2.75E-08	-2.03E-08	-1.41E-08	-7.72E-09	-5.27E-09	-3.07E-09	-3.60E-09	-1.61E-11	-2.59E-09	-3.42E-10	6.93E-10	2.32E-09	2.12E-09	2.72E-09	-1.84E-09	-1.07E-10	6.32E-09	7.46E-09	4.34E-09
-3.40E-08	-2.86E-08	-1.79E-08	-1.72E-08	-1.12E-08	-8.18E-09	-5.52E-09	-5.62E-09	-6.05E-10	-2.43E-09	5.63E-10	-1.06E-10	3.38E-09	3.18E-09	1.73E-09	-1.10E-09	1.38E-09	4.09E-09	4.24E-09	3.84E-09
-3.38E-08	-2.87E-08	-1.49E-08	-1.93E-08	-1.32E-08	-9.45E-09	-5.66E-09	-6.18E-09	1.16E-09	-1.97E-09	1.61E-09	2.73E-10	4.77E-09	5.06E-09	2.92E-09	2.57E-10	2.89E-09	-1.39E-09	4.51E-09	4.06E-09
-3.28E-08	-2.73E-08	-1.59E-08	-2.01E-08	-1.48E-08	-1.08E-08	-5.37E-09	-6.92E-09	-2.63E-09	-4.15E-10	4.52E-09	4.29E-09	7.62E-09	8.44E-09	6.06E-09	2.00E-09	4.21E-09	1.81E-09	4.96E-09	4.45E-09
-3.33E-08	-2.84E-08	-2.60E-08	-2.12E-08	-1.69E-08	-1.19E-08	-6.00E-09	-5.98E-09	-3.65E-09	1.66E-09	8.32E-09	1.09E-08	1.43E-08	1.43E-08	1.15E-08	6.22E-09	-7.86E-11	3.64E-09	4.89E-09	3.58E-09
-3.57E-08	-3.13E-08	-2.73E-08	-2.30E-08	-1.77E-08	-1.21E-08	-7.18E-09	-5.58E-09	-7.20E-11	6.00E-09	1.55E-08	2.18E-08	2.56E-08	2.57E-08	2.06E-08	1.33E-08	2.93E-09	8.41E-09	3.94E-09	2.12E-09
-3.55E-08	-3.35E-08	-2.89E-08	-2.33E-08	-1.86E-08	-1.32E-08	-7.47E-09	-6.83E-09	5.08E-09	1.44E-08	2.94E-08	3.70E-08	4.05E-08	4.18E-08	3.53E-08	2.42E-08	8.86E-09	1.37E-08	2.86E-09	7.95E-10
-3.50E-08	-3.44E-08	-2.98E-08	-2.27E-08	-1.97E-08	-1.45E-08	-9.50E-09	-5.71E-09	7.02E-09	2.55E-08	4.34E-08	5.07E-08	5.30E-08	5.50E-08	4.92E-08	3.71E-08	1.75E-08	1.77E-08	3.85E-09	5.70E-10
-3.53E-08	-3.48E-08	-2.96E-08	-2.27E-08	-2.12E-08	-1.57E-08	-1.10E-08	-2.00E-09	1.43E-08	3.48E-08	5.19E-08	5.98E-08	6.21E-08	6.28E-08	5.78E-08	4.70E-08	2.61E-08	4.22E-09	6.79E-09	-4.38E-10
-3.52E-08	-3.61E-08	-3.11E-08	-2.28E-08	-2.19E-08	-1.49E-08	-1.08E-08	2.50E-09	2.02E-08	3.96E-08	5.62E-08	6.56E-08	6.89E-08	6.70E-08	6.19E-08	5.31E-08	3.18E-08	8.04E-09	1.25E-08	-1.44E-09
-3.53E-08	-3.48E-08	-3.05E-08	-2.28E-08	-2.29E-08	-1.58E-08	-1.11E-08	6.21E-09	2.28E-08	4.19E-08	5.90E-08	6.81E-08	7.06E-08	6.83E-08	6.29E-08	5.38E-08	3.45E-08	9.75E-09	1.35E-08	-3.54E-09
-3.31E-08	-3.24E-08	-2.85E-08	-2.32E-08	-2.33E-08	-1.77E-08	-1.24E-08	8.18E-09	2.26E-08	4.04E-08	6.05E-08	6.81E-08	6.91E-08	6.72E-08	6.18E-08	5.09E-08	3.38E-08	9.96E-09	5.30E-09	-4.40E-09
-2.92E-08	-2.90E-08	-2.86E-08	-2.38E-08	-2.27E-08	-1.86E-08	-1.39E-08	1.01E-08	2.03E-08	3.58E-08	6.09E-08	6.62E-08	6.50E-08	6.51E-08	6.06E-08	4.77E-08	3.07E-08	9.47E-09	-2.53E-09	-4.09E-09
-2.19E-08	-2.35E-08	-2.64E-08	-2.24E-08	-2.15E-08	-1.92E-08	-1.53E-08	1.07E-08	1.83E-08	2.96E-08	5.85E-08	6.02E-08	5.72E-08	6.11E-08	5.85E-08	4.43E-08	2.79E-08	9.21E-09	-5.35E-09	-5.37E-09
-9.73E-09	-1.40E-08	-1.87E-08	-1.81E-08	-1.95E-08	-1.92E-08	-1.62E-08	-1.28E-09	1.64E-08	2.13E-08	4.66E-08	4.63E-08	4.51E-08	4.84E-08	4.97E-08	3.72E-08	1.92E-08	8.63E-09	-6.59E-09	-6.71E-09
6.79E-09	1.03E-09	-8.81E-09	-1.09E-08	-1.54E-08	-1.92E-08	-1.70E-08	-1.49E-08	1.23E-08	1.02E-08	2.55E-08	2.78E-08	2.74E-08	2.61E-08	3.20E-08	2.53E-08	1.06E-08	-1.05E-08	-8.20E-09	-6.76E-09
2.43E-08	1.72E-08	3.29E-09	-8.26E-10	-7.98E-09	-1.67E-08	-1.66E-08	-1.51E-08	-1.10E-08	2.09E-09	8.17E-09	1.65E-08	1.46E-08	1.53E-08	1.53E-08	-2.47E-09	-9.72E-09	-1.16E-08	-8.51E-09	-6.68E-09
4.34E-08	3.32E-08	1.70E-08	1.23E-08	2.23E-09	-1.10E-08	-1.58E-08	-1.51E-08	-1.35E-08	-1.18E-08	-8.42E-09	3.23E-09	-1.37E-09	-9.76E-09	-1.24E-08	-1.29E-08	-1.00E-08	-1.09E-08	-7.94E-09	-7.98E-09
6.43E-08	5.14E-08	3.41E-08	2.63E-08	1.47E-08	-2.87E-09	-1.33E-08	-1.48E-08	-1.46E-08	-1.22E-08	-1.13E-08	-8.17E-09	-1.19E-08	-1.37E-08	-1.27E-08	-1.35E-08	-9.89E-09	-9.28E-09	-7.82E-09	-9.23E-09
8.57E-08	7.25E-08	5.32E-08	4.13E-08	2.89E-08	7.26E-09	-9.34E-09	-1.44E-08	-1.60E-08	-1.23E-08	-1.17E-08	-8.34E-09	-1.20E-08	-1.43E-08	-1.25E-08	-1.27E-08	-8.85E-09	-5.54E-09	-7.37E-09	-9.12E-09
1.09E-07	9.56E-08	7.32E-08	5.74E-08	4.40E-08	1.99E-08	-4.05E-09	-1.28E-08	-1.60E-08	-1.19E-08	-1.17E-08	-8.54E-09	-1.16E-08	-1.47E-08	-1.34E-08	-1.22E-08	-7.34E-09	-3.74E-09	-7.62E-09	-9.54E-09
1.36E-07	1.21E-07	9.48E-08	7.47E-08	6.22E-08	3.63E-08	5.18E-09	-9.31E-09	-1.38E-08	-1.13E-08	-1.16E-08	-9.49E-09	-1.06E-08	-1.51E-08	-1.44E-08	-1.28E-08	-4.68E-09	-7.30E-09	-1.16E-08	-9.91E-09
1.66E-07	1.51E-07	1.20E-07	9.22E-08	8.39E-08	5.48E-08	1.73E-08	-3.52E-09	-1.26E-08	-1.16E-08	-1.24E-08	-1.11E-08	-1.18E-08	-1.57E-08	-1.41E-08	-1.39E-08	-5.86E-09	-7.52E-09	-1.20E-08	-1.03E-08
2.00E-07	1.85E-07	1.48E-07	1.09E-07	1.08E-07	7.32E-08	2.83E-08	3.55E-09	-9.45E-09	-1.31E-08	-1.33E-08	-1.28E-08	-1.41E-08	-1.57E-08	-1.38E-08	-1.36E-08	-1.51E-08	-1.39E-08	-1.21E-08	-1.04E-08
2.32E-07	2.20E-07	1.75E-07	1.23E-07	1.25E-07	8.09E-08	3.62E-08	1.10E-08	-1.03E-08	-1.63E-08	-1.35E-08	-1.37E-08	-1.50E-08	-1.66E-08	-1.52E-08	-1.20E-08	-1.52E-08	-1.22E-08	-1.21E-08	-1.12E-08
2.55E-07	2.43E-07	1.94E-07	1.31E-07	1.23E-07	7.48E-08	4.11E-08	1.59E-08	-8.92E-09	-1.75E-08	-1.36E-08	-1.39E-08	-1.52E-08	-1.78E-08	-1.65E-08	-1.26E-08	-1.53E-08	-1.78E-08	-1.29E-08	-1.19E-08
2.52E-07	2.39E-07	1.97E-07	1.33E-07	1.03E-07	7.17E-08	4.42E-08	1.75E-08	-8.76E-09	-1.89E-08	-1.61E-08	-1.42E-08	-1.53E-08	-1.80E-08	-1.78E-08	-1.62E-08	-1.54E-08	-1.71E-08	-1.40E-08	-1.23E-08
2.35E-07	2.23E-07	1.83E-07	1.29E-07	1.02E-07	7.21E-08	4.50E-08	1.72E-08	-6.47E-09	-2.25E-08	-1.81E-08	-1.47E-08	-1.55E-08	-1.70E-08	-1.78E-08	-1.67E-08	-1.42E-08	-1.83E-08	-1.48E-08	-1.24E-08
2.17E-07	2.01E-07	1.55E-07	1.23E-07	9.86E-08	7.14E-08	4.56E-08	1.69E-08	-5.78E-09	-2.34E-08	-1.87E-08	-1.52E-08	-1.53E-08	-1.75E-08	-1.76E-08	-1.73E-08	-1.22E-08	-1.67E-08	-1.54E-08	-1.40E-08
1.99E-07	1.72E-07	1.39E-07	1.15E-07	9.41E-08	7.00E-08	4.60E-08	1.80E-08	-7.41E-09	-2.31E-08	-1.95E-08	-1.62E-08	-1.58E-08	-1.92E-08	-1.82E-08	-1.67E-08	-1.05E-08	-1.39E-08	-1.51E-08	-1.52E-08
1.83E-07	1.53E-07	1.29E-07	1.07E-07	9.04E-08	6.88E-08	4.66E-08	2.07E-08	-1.30E-08	-2.25E-08	-2.02E-08	-1.64E-08	-1.67E-08	-2.00E-08	-1.97E-08	-1.52E-08	-9.72E-09	-1.09E-08	-1.52E-08	-1.50E-08
1.65E-07	1.40E-07	1.21E-07	9.96E-08	8.60E-08	6.78E-08	4.75E-08	2.42E-08	-8.94E-09	-2.21E-08	-2.10E-08	-1.75E-08	-1.84E-08	-1.98E-08	-1.99E-08	-1.54E-08	-1.92E-08	-1.79E-08	-1.61E-08	-1.56E-08
1.46E-07	1.29E-07	1.14E-07	9.23E-08	8.07E-08	6.62E-08	4.84E-08	2.73E-08	-6.77E-09	-2.12E-08	-2.22E-08	-2.02E-08	-2.02E-08	-2.05E-08	-2.07E-08	-1.97E-08	-2.16E-08	-1.81E-08	-1.61E-08	-1.75E-08
1.30E-07	1.19E-07	1.06E-07	8.57E-08	7.66E-08	6.35E-08	4.80E-08	2.78E-08	-4.82E-09	-2.03E-08	-2.28E-08	-2.15E-08	-2.27E-08	-2.18E-08	-2.19E-08	-2.07E-08	-2.36E-08	-2.12E-08	-1.61E-08	-1.71E-08
1.15E-07	1.09E-07	9.63E-08	8.10E-08	7.33E-08	5.99E-08	4.47E-08	2.68E-08	-1.70E-09	-2.00E-08	-2.26E-08	-2.24E-08	-2.37E-08	-2.41E-08	-2.29E-08	-2.21E-08	-2.35E-08	-1.81E-08	-1.81E-08	-1.79E-08
1.03E-07	1.00E-07	8.93E-08	7.72E-08	7.00E-08	5.69E-08	4.17E-08	2.60E-08	1.13E-09	-1.93E-08	-2.19E-08	-2.28E-08	-2.45E-08	-2.57E-08	-2.36E-08	-2.32E-08	-2.33E-08	-1.62E-08	-1.82E-08	-1.88E-08
9.33E-08	9.26E-08	8.37E-08	7.35E-08	6.62E-08	5.40E-08	4.02E-08	2.56E-08	1.51E-09	-1.89E-08	-2.02E-08	-2.38E-08	-2.53E-08	-2.50E-08	-2.47E-08	-2.44E-08	-2.26E-08	-1.42E-08	-1.83E-08	-1.93E-08
8.53E-08	8.59E-08	8.02E-08	6.88E-08	6.22E-08	5.16E-08	3.88E-08	2.48E-08	1.40E-09	-1.75E-08	-1.86E-08	-2.38E-08	-2.54E-08	-2.58E-08	-2.53E-08	-2.44E-08	-2.22E-08	-8.89E-09	-2.00E-08	-2.02E-08
7.81E-08	8.08E-08	7.66E-08	6.50E-08	5.91E-08	4.97E-08	3.72E-08	2.43E-08	1.77E-09	-1.52E-08	-1.93E-08	-2.33E-08	-2.56E-08	-2.75E-08	-2.46E-08	-2.51E-08	-2.06E-08	-1.17E-08	-2.15E-08	-2.14E-08
7.16E-08	7.71E-08	7.23E-08	6.11E-08	5.59E-08	4.78E-08	3.62E-08	2.48E-08	3.81E-09	-1.35E-08	-1.96E-08	-2.27E-08	-2.57E-08	-2.82E-08	-2.38E-08	-2.60E-08	-1.85E-08	-2.13E-08	-2.19E-08	-2.30E-08
6.41E-08	6.99E-08	6.53E-08	5.59E-08	5.00E-08	4.37E-08	3.42E-08	2.52E-08	7.86E-09	-1.08E-08	-1.90E-08	-2.32E-08	-2.68E-08	-2.72E-08	-2.48E-08	-2.77E-08	-2.12E-08	-1.99E-08	-2.16E-08	-2.44E-08
5.22E-08	6.01E-08	5.34E-08	4.86E-08	4.03E-08	3.57E-08	2.80E-08	2.37E-08	1.32E-08	-5.14E-09	-1.76E-08	-2.44E-08	-2.60E-08	-2.85E-08	-2.61E-08	-2.75E-08	-2.85E-08	-1.58E-08	-2.56E-08	-2.59E-08
3.47E-08	4.82E-08	4.03E-08	3.77E-08	2.83E-08	2.47E-08	1.83E-08	1.92E-08	1.68E-08	-1.15E-09	-1.65E-08	-2.32E-08	-2.46E-08	-2.98E-08	-2.76E-08	-2.83E-08	-2.81E-08	-1.78E-08	-2.54E-08	-2.64E-08
1.85E-08	2.88E-08	2.42E-08	2.47E-08	1.55E-08	1.29E-08	7.37E-09	1.36E-08	1.50E-08	-2.69E-09	-1.99E-08	-2.22E-08	-2.38E-08	-3.08E-08	-2.82E-08	-2.94E-08	-2.93E-08	-2.26E-08	-2.55E-08	-2.68E-08
1.92E-09	6.03E-09	4.71E-09	1.27E-08	1.67E-09	3.45E-10	-4.39E-09	5.87E-09	7.41E-09	-9.29E-09	-2.48E-08	-2.47E-08	-2.62E-08	-3.09E-08	-2.99E-08	-2.91E-08	-2.99E-08	-2.14E-08	-2.59E-08	-2.75E-08
-1.41E-08	-1.37E-08	-1.03E-08	2.00E-09	-1.19E-08	-1.25E-08	-1.63E-08	-6.11E-09	-7.93E-09	-1.87E-08	-2.98E-08	-2.75E-08	-2.91E-08	-2.99E-08	-3.03E-08	-2.87E-08	-2.95E-08	-2.34E-08	-2.58E-08	-2.76E-08
-3.21E-08	-2.80E-08	-2.01E-08	-1.34E-08	-2.30E-08	-2.41E-08	-2.64E-08	-2.13E-08	-1.51E-08	-2.68E-08	-3.18E-08	-2.84E-08	-3.00E-08	-2.94E-08	-3.02E-08	-2.97E-08	-2.92E-08	-1.95E-08	-2.56E-08	-2.76E-08
-4.21E-08	-3.77E-08	-2.35E-08	-2.72E-08	-3.15E-08	-3.27E-08	-3.42E-08	-2.84E-08	-2.29E-08	-3.02E-08	-3.18E-08	-2.74E-08	-3.01E-08	-3.02E-08	-3.00E-08	-3.06E-08	-2.92E-08	-2.03E-08	-2.63E-08	-2.78E-08
-4.66E-08	-4.43E-08	-2.66E-08	-3.30E-08	-3.68E-08	-3.71E-08	-3.73E-08	-3.47E-08	-2.68E-08	-3.09E-08	-3.25E-08	-2.66E-08	-3.07E-08	-3.20E-08	-3.17E-08	-2.94E-08	-2.75E-08	-2.76E-08	-2.71E-08	-2.79E-08
-4.64E-08	-4.63E-08	-3.50E-08	-3.96E-08	-3.85E-08	-3.69E-08	-3.65E-08	-3.35E-08	-3.29E-08	-3.08E-08	-3.25E-08	-2.61E-08	-3.10E-08	-3.19E-08	-3.27E-08	-2.92E-08	-2.68E-08	-3.01E-08	-2.87E-08	-2.84E-08
-4.65E-08	-4.64E-08	-3.07E-08	-3.97E-08	-3.88E-08	-3.66E-08	-3.55E-08	-3.17E-08	-3.13E-08	-3.25E-08	-3.24E-08	-2.78E-08	-3.08E-08	-3.30E-08	-3.36E-08	-3.18E-08	-3.06E-08	-2.84E-08	-2.93E-08	-2.81E-08
-4.77E-08	-4.59E-08	-3.01E-08	-3.98E-08	-3.80E-08	-3.69E-08	-3.54E-08	-2.99E-08	-3.14E-08	-3.28E-08	-3.39E-08	-3.02E-08	-3.19E-08	-3.35E-08	-3.45E-08	-3.52E-08	-3.05E-08	-3.01E-08	-2.99E-08	-2.77E-08
-4.88E-08	-4.56E-08	-3.82E-08	-3.81E-08	-3.77E-08	-3.71E-08	-3.60E-08	-2.78E-08	-2.95E-08	-3.10E-08	-3.50E-08	-3.08E-08	-3.26E-08	-3.34E-08	-3.46E-08	-3.51E-08	-3.07E-08	-2.92E-08	-2.98E-08	-2.75E-08
-4.97E-08	-4.57E-08	-3.47E-08	-3.62E-08	-3.83E-08	-3.67E-08	-3.59E-08	-2.62E-08	-2.55E-08	-2.96E-08	-3.60E-08	-3.10E-08	-3.21E-08	-3.33E-08	-3.56E-08	-3.43E-08	-2.98E-08	-2.92E-08	-3.06E-08	-2.88E-08
-5.03E-08	-4.50E-08	-3.24E-08	-3.94E-08	-3.99E-08	-3.74E-08	-3.58E-08	-3.19E-08	-2.87E-08	-3.10E-08	-3.58E-08	-3.20E-08	-3.31E-08	-3.37E-08	-3.62E-08	-3.51E-08	-3.42E-08	-3.30E-08	-3.17E-08	-3.09E-08
-5.01E-08	-4.35E-08	-4.29E-08	-4.22E-08	-4.01E-08	-3.85E-08	-3.68E-08	-3.60E-08	-3.09E-08	-2.91E-08	-3.61E-08	-3.31E-08	-3.47E-08	-3.36E-08	-3.63E-08	-3.54E-08	-3.29E-08	-3.26E-08	-3.14E-08	-3.09E-08
-5.02E-08	-4.03E-08	-4.54E-08	-4.27E-08	-4.01E-08	-3.93E-08	-3.75E-08	-3.61E-08	-3.18E-08	-3.10E-08	-3.68E-08	-3.29E-08	-3.51E-08	-3.43E-08	-3.64E-08	-3.48E-08	-3.27E-08	-3.45E-08	-3.14E-08	-3.09E-08
-4.88E-08	-3.61E-08	-4.54E-08	-4.32E-08	-4.12E-08	-3.97E-08	-3.69E-08	-3.60E-08	-3.26E-08	-3.50E-08	-3.79E-08	-3.33E-08	-3.51E-08	-3.41E-08	-3.68E-08	-3.54E-08	-3.27E-08	-3.35E-08	-3.15E-08	-3.11E-08
-4.79E-08	-3.35E-08	-4.63E-08	-4.35E-08	-4.23E-08	-4.07E-08	-3.71E-08	-3.66E-08	-3.01E-08	-3.37E-08	-3.71E-08	-3.58E-08	-3.60E-08	-3.44E-08	-3.77E-08	-3.71E-08	-3.19E-08	-3.12E-08	-3.22E-08	-3.18E-08
-4.81E-08	-4.73E-08	-4.65E-08	-4.41E-08	-4.30E-08	-4.13E-08	-3.70E-08	-3.66E-08	-2.78E-08	-3.29E-08	-3.77E-08	-3.68E-08	-3.63E-08	-3.51E-08	-3.84E-08	-3.87E-08	-2.91E-08	-3.13E-08	-3.35E-08	-3.19E-08

-4.82E-08	-4.74E-08	-4.56E-08	-4.34E-08	-4.28E-08	-4.14E-08	-3.72E-08	-3.69E-08	-3.67E-08	-3.08E-08	-3.83E-08	-3.68E-08	-3.63E-08	-3.52E-08	-3.86E-08	-3.94E-08	-2.73E-08	-3.21E-08	-3.30E-08	-3.15E-08
-4.73E-08	-4.61E-08	-4.40E-08	-4.35E-08	-4.29E-08	-4.20E-08	-3.84E-08	-3.70E-08	-3.92E-08	-2.94E-08	-3.90E-08	-3.64E-08	-3.71E-08	-3.62E-08	-3.95E-08	-3.93E-08	-2.47E-08	-3.03E-08	-3.25E-08	-3.18E-08
-4.59E-08	-4.56E-08	-4.41E-08	-4.40E-08	-4.37E-08	-4.19E-08	-3.84E-08	-3.77E-08	-3.93E-08	-3.22E-08	-3.83E-08	-3.57E-08	-3.85E-08	-3.70E-08	-3.91E-08	-3.91E-08	-2.71E-08	-3.48E-08	-3.34E-08	-3.16E-08
-4.68E-08	-4.65E-08	-4.57E-08	-4.46E-08	-4.34E-08	-4.15E-08	-3.90E-08	-3.79E-08	-4.03E-08	-3.42E-08	-3.82E-08	-3.54E-08	-4.00E-08	-3.84E-08	-3.90E-08	-3.89E-08	-2.98E-08	-3.80E-08	-3.41E-08	-3.11E-08
-5.02E-08	-4.58E-08	-4.66E-08	-4.25E-08	-4.20E-08	-4.16E-08	-4.03E-08	-3.82E-08	-3.97E-08	-3.47E-08	-3.89E-08	-3.44E-08	-4.14E-08	-3.90E-08	-4.02E-08	-3.92E-08	-3.75E-08	-3.72E-08	-3.30E-08	-3.00E-08
-4.99E-08	-4.45E-08	-4.74E-08	-4.12E-08	-4.21E-08	-4.22E-08	-4.04E-08	-3.90E-08	-3.79E-08	-3.23E-08	-3.84E-08	-3.46E-08	-4.29E-08	-4.17E-08	-4.10E-08	-3.91E-08	-3.82E-08	-3.62E-08	-3.17E-08	-2.97E-08
-5.03E-08	-4.22E-08	-4.72E-08	-4.10E-08	-4.29E-08	-4.19E-08	-4.04E-08	-3.90E-08	-3.59E-08	-3.04E-08	-3.66E-08	-3.43E-08	-4.46E-08	-4.40E-08	-4.03E-08	-3.86E-08	-3.91E-08	-3.42E-08	-3.20E-08	-3.18E-08
-5.01E-08	-3.91E-08	-4.82E-08	-4.16E-08	-4.19E-08	-4.17E-08	-4.01E-08	-3.89E-08	-3.52E-08	-3.20E-08	-3.53E-08	-3.41E-08	-4.24E-08	-4.24E-08	-3.88E-08	-3.86E-08	-3.68E-08	-3.15E-08	-3.36E-08	-3.39E-08
-4.96E-08	-4.13E-08	-4.79E-08	-3.91E-08	-3.98E-08	-4.17E-08	-3.97E-08	-3.82E-08	-3.17E-08	-3.33E-08	-3.44E-08	-3.30E-08	-3.84E-08	-3.88E-08	-3.84E-08	-3.83E-08	-3.41E-08	-3.07E-08	-3.48E-08	-3.40E-08
-5.07E-08	-4.91E-08	-4.69E-08	-3.70E-08	-3.89E-08	-4.21E-08	-4.00E-08	-3.60E-08	-3.01E-08	-3.31E-08	-3.37E-08	-3.26E-08	-3.56E-08	-3.75E-08	-3.83E-08	-3.74E-08	-3.24E-08	-3.68E-08	-3.47E-08	-3.13E-08

6.83E-18	3.56E-17	7.27E-17	2.65E-16	4.50E-16	7.11E-16	1.01E-15	1.03E-15	1.18E-15	1.51E-15	1.33E-15	1.12E-15	7.40E-16	1.16E-15	1.31E-15	1.20E-15	1.79E-15	1.79E-15	1.54E-15	2.18E-15
9.17E-19	3.55E-17	1.04E-16	2.70E-16	4.06E-16	6.56E-16	9.44E-16	9.86E-16	1.19E-15	1.54E-15	1.41E-15	1.16E-15	6.90E-16	1.13E-15	1.25E-15	1.15E-15	1.13E-15	1.17E-15	1.51E-15	2.14E-15
1.88E-18	2.56E-17	1.26E-16	2.71E-16	3.97E-16	6.28E-16	9.00E-16	9.47E-16	1.25E-15	1.53E-15	1.46E-15	1.22E-15	6.80E-16	1.08E-15	1.36E-15	1.17E-15	1.14E-15	1.19E-15	1.50E-15	1.85E-15
8.13E-18	1.50E-17	1.53E-16	2.55E-16	3.74E-16	6.63E-16	8.71E-16	9.08E-16	1.27E-15	1.51E-15	1.38E-15	1.16E-15	6.89E-16	1.03E-15	1.36E-15	1.20E-15	1.13E-15	1.21E-15	1.58E-15	1.75E-15
1.86E-17	1.83E-17	2.40E-16	2.00E-16	3.66E-16	7.69E-16	8.76E-16	8.51E-16	1.08E-15	1.45E-15	1.25E-15	1.04E-15	6.93E-16	9.74E-16	1.14E-15	1.17E-15	1.25E-15	1.41E-15	1.63E-15	1.64E-15
1.52E-17	1.53E-17	8.73E-17	1.89E-16	3.40E-16	7.99E-16	8.15E-16	7.70E-16	8.97E-16	1.29E-15	1.16E-15	9.61E-16	6.81E-16	9.44E-16	1.11E-15	1.08E-15	1.40E-15	1.47E-15	1.57E-15	1.60E-15
1.40E-17	1.26E-17	2.09E-17	1.89E-16	2.78E-16	5.88E-16	7.33E-16	7.74E-16	8.08E-16	1.25E-15	1.04E-15	9.02E-16	6.36E-16	9.06E-16	1.10E-15	1.08E-15	1.52E-15	1.19E-15	1.59E-15	1.54E-15
3.48E-17	6.11E-18	1.98E-17	2.30E-16	2.43E-16	4.69E-16	7.14E-16	8.36E-16	8.28E-16	1.20E-15	1.03E-15	8.13E-16	6.53E-16	8.98E-16	1.10E-15	1.03E-15	1.08E-15	1.18E-15	1.75E-15	1.39E-15
1.10E-16	4.09E-18	1.24E-17	2.29E-16	2.19E-16	4.50E-16	7.06E-16	8.60E-16	8.38E-16	1.16E-15	9.30E-16	7.40E-16	6.18E-16	8.77E-16	9.55E-16	1.03E-15	9.99E-16	1.26E-15	1.70E-15	1.23E-15
2.34E-16	2.62E-18	1.44E-17	1.53E-16	2.15E-16	4.19E-16	7.10E-16	8.56E-16	8.70E-16	1.04E-15	8.73E-16	7.07E-16	6.17E-16	8.10E-16	8.95E-16	1.02E-15	9.89E-16	1.47E-15	1.35E-15	1.20E-15
9.50E-17	8.39E-18	1.09E-17	1.63E-16	2.30E-16	4.04E-16	7.90E-16	9.64E-16	9.48E-16	1.05E-15	8.72E-16	6.99E-16	6.42E-16	8.22E-16	9.05E-16	9.58E-16	1.02E-15	9.95E-16	1.30E-15	1.14E-15
1.53E-17	1.37E-17	1.22E-17	2.00E-16	2.18E-16	4.18E-16	9.84E-16	1.28E-15	1.17E-15	1.09E-15	8.53E-16	6.51E-16	6.39E-16	8.63E-16	8.87E-16	9.66E-16	1.05E-15	1.14E-15	1.36E-15	1.10E-15
1.47E-17	1.19E-17	1.96E-17	1.00E-16	2.12E-16	5.49E-16	1.35E-15	1.90E-15	1.48E-15	1.08E-15	7.80E-16	5.50E-16	5.18E-16	8.54E-16	8.60E-16	9.05E-16	8.05E-16	1.39E-15	1.27E-15	1.12E-15
1.14E-17	1.27E-18	3.44E-17	1.12E-16	2.07E-16	7.36E-16	1.93E-15	2.77E-15	1.98E-15	1.07E-15	6.66E-16	4.89E-16	4.72E-16	8.28E-16	8.20E-16	7.94E-16	8.99E-16	1.28E-15	1.22E-15	1.15E-15
5.26E-19	1.53E-19	2.93E-17	1.04E-16	1.51E-16	8.47E-16	2.09E-15	2.95E-15	2.23E-15	1.03E-15	6.48E-16	4.65E-16	5.14E-16	7.61E-16	8.45E-16	7.18E-16	8.02E-16	6.65E-16	1.11E-15	1.18E-15
8.16E-18	1.83E-18	5.50E-17	8.12E-17	1.35E-16	7.27E-16	1.60E-15	2.25E-15	1.84E-15	8.78E-16	6.49E-16	4.45E-16	4.79E-16	7.10E-16	8.51E-16	6.93E-16	7.87E-16	7.25E-16	1.14E-15	1.18E-15
1.32E-17	1.45E-19	1.02E-16	6.60E-17	1.53E-16	4.90E-16	9.07E-16	1.38E-15	1.31E-15	6.68E-16	6.57E-16	4.37E-16	5.15E-16	6.15E-16	7.33E-16	6.70E-16	8.82E-16	7.90E-16	1.10E-15	1.17E-15
1.03E-17	2.42E-19	1.14E-16	5.64E-17	1.52E-16	1.84E-16	4.10E-16	6.86E-16	6.40E-16	4.65E-16	6.76E-16	4.58E-16	5.77E-16	5.99E-16	7.03E-16	6.76E-16	8.91E-16	9.93E-16	1.12E-15	1.20E-15
9.87E-18	4.00E-18	1.25E-20	4.75E-17	1.18E-16	1.84E-16	2.83E-16	3.30E-16	2.82E-16	4.22E-16	6.84E-16	4.29E-16	5.71E-16	5.90E-16	7.17E-16	6.76E-16	9.62E-16	1.39E-15	1.16E-15	1.19E-15
2.15E-17	1.24E-17	8.83E-22	1.97E-17	1.05E-16	1.87E-16	3.29E-16	3.04E-16	3.13E-16	4.86E-16	6.35E-16	4.04E-16	6.02E-16	5.61E-16	6.96E-16	6.53E-16	1.10E-15	1.38E-15	1.29E-15	1.35E-15
3.65E-17	1.34E-17	2.70E-18	2.19E-17	1.07E-16	2.49E-16	4.30E-16	3.30E-16	3.36E-16	5.98E-16	5.84E-16	3.82E-16	5.69E-16	5.49E-16	6.01E-16	7.01E-16	1.02E-15	1.34E-15	1.27E-15	1.29E-15
4.40E-17	1.46E-17	1.08E-17	1.53E-17	8.64E-17	3.49E-16	6.15E-16	3.42E-16	3.39E-16	7.24E-16	5.02E-16	4.12E-16	5.49E-16	5.18E-16	6.11E-16	7.79E-16	6.64E-16	1.11E-15	9.23E-16	8.43E-16
5.20E-17	4.55E-17	1.45E-17	2.26E-17	6.01E-17	4.69E-16	8.85E-16	3.17E-16	3.55E-16	7.71E-16	4.78E-16	4.23E-16	5.54E-16	5.27E-16	6.67E-16	8.80E-16	6.64E-16	7.59E-16	7.20E-16	7.95E-16
6.57E-17	7.88E-17	1.78E-17	1.91E-17	5.61E-17	4.44E-16	8.69E-16	2.36E-16	3.43E-16	4.73E-16	4.84E-16	3.90E-16	5.09E-16	4.99E-16	6.50E-16	7.92E-16	7.53E-16	8.14E-16	7.46E-16	7.78E-16
7.04E-17	6.90E-17	1.86E-17	6.04E-18	5.23E-17	1.83E-16	3.14E-16	2.09E-16	2.73E-16	4.55E-16	5.19E-16	3.47E-16	4.58E-16	4.86E-16	5.18E-16	6.05E-16	7.19E-16	7.08E-16	6.66E-16	7.21E-16
7.86E-17	5.86E-17	1.95E-17	5.68E-18	4.04E-17	6.65E-17	1.05E-16	2.32E-16	2.75E-16	4.86E-16	5.63E-16	2.97E-16	4.21E-16	4.59E-16	4.69E-16	5.57E-16	7.33E-16	6.99E-16	5.83E-16	7.77E-16
9.56E-17	6.06E-17	2.12E-17	9.26E-18	2.70E-17	5.74E-17	9.87E-17	2.43E-16	2.68E-16	4.68E-16	5.65E-16	2.38E-16	3.78E-16	4.61E-16	3.94E-16	5.11E-16	5.64E-16	9.26E-16	5.51E-16	8.10E-16
9.66E-17	9.11E-17	3.17E-17	3.29E-18	1.99E-17	4.28E-17	9.52E-17	2.11E-16	2.58E-16	3.35E-16	4.81E-16	2.42E-16	3.69E-16	4.17E-16	3.66E-16	3.71E-16	4.15E-16	6.60E-16	5.33E-16	6.85E-16
9.94E-17	8.75E-17	2.02E-17	8.56E-20	1.69E-17	4.94E-17	7.90E-17	1.54E-16	3.27E-16	3.61E-16	4.40E-16	2.49E-16	3.49E-16	2.99E-16	3.42E-16	3.35E-16	4.95E-16	6.45E-16	5.39E-16	5.09E-16
1.26E-16	5.39E-17	2.10E-17	9.09E-19	1.43E-17	6.89E-17	7.00E-17	1.15E-16	4.97E-16	3.81E-16	3.47E-16	2.55E-16	3.39E-16	2.80E-16	3.24E-16	3.03E-16	5.74E-16	6.32E-16	5.69E-16	4.67E-16
1.45E-16	3.28E-17	1.35E-17	1.02E-18	1.48E-17	8.71E-17	8.45E-17	1.13E-16	7.15E-16	3.74E-16	2.92E-16	2.70E-16	3.14E-16	2.71E-16	2.40E-16	3.00E-16	6.16E-16	6.34E-16	5.51E-16	4.42E-16
1.75E-16	5.29E-17	6.61E-18	1.18E-19	1.54E-17	5.27E-17	7.73E-17	1.20E-16	5.89E-16	2.81E-16	2.82E-16	2.99E-16	2.94E-16	2.74E-16	2.08E-16	3.20E-16	6.18E-16	8.00E-16	5.94E-16	4.00E-16
1.92E-16	1.02E-16	1.75E-17	4.48E-19	9.90E-18	1.61E-17	4.99E-17	1.24E-16	1.21E-16	2.04E-16	2.93E-16	2.55E-16	2.95E-16	3.02E-16	2.14E-16	2.94E-16	3.08E-16	6.72E-16	6.92E-16	4.07E-16
2.04E-16	1.40E-16	9.56E-17	1.52E-18	3.71E-18	1.44E-17	3.89E-17	1.36E-16	1.10E-16	1.96E-16	2.82E-16	2.11E-16	2.72E-16	2.80E-16	2.25E-16	2.33E-16	3.69E-16	8.18E-16	7.35E-16	4.32E-16
1.89E-16	1.38E-16	8.65E-17	7.88E-18	1.13E-19	1.24E-17	3.46E-17	1.25E-16	9.27E-17	1.54E-16	2.45E-16	2.53E-16	2.86E-16	2.81E-16	2.09E-16	2.13E-16	4.11E-16	5.23E-16	5.09E-16	4.24E-16
1.98E-16	1.59E-16	1.08E-16	3.20E-17	3.04E-19	6.64E-18	2.99E-17	1.00E-16	9.87E-17	1.37E-16	1.82E-16	1.97E-16	2.85E-16	2.59E-16	1.60E-16	1.89E-16	3.77E-16	3.64E-16	3.64E-16	3.62E-16
2.46E-16	1.73E-16	9.33E-17	5.44E-17	4.75E-18	2.60E-18	2.55E-17	1.02E-16	1.23E-16	1.20E-16	1.41E-16	1.41E-16	2.50E-16	2.28E-16	1.28E-16	1.37E-16	2.99E-16	1.51E-16	2.70E-16	3.10E-16
2.62E-16	1.56E-16	9.48E-17	8.17E-18	1.17E-17	1.99E-18	1.37E-17	1.04E-16	1.90E-16	1.43E-16	1.18E-16	1.23E-16	2.11E-16	1.62E-16	1.27E-16	1.18E-16	4.43E-16	1.22E-16	2.53E-16	3.24E-16
2.64E-16	1.59E-16	8.11E-17	2.79E-18	1.53E-17	8.52E-19	5.66E-18	7.01E-17	3.00E-16	1.66E-16	9.86E-17	1.00E-16	1.81E-16	1.41E-16	1.09E-16	1.07E-16	6.68E-16	1.07E-16	2.08E-16	3.19E-16
2.72E-16	2.04E-16	5.10E-17	1.15E-18	1.82E-17	7.84E-20	6.19E-18	6.04E-17	5.31E-16	2.16E-16	9.88E-17	8.52E-17	1.46E-16	1.23E-16	9.87E-17	9.26E-17	6.59E-16	1.20E-16	1.72E-16	2.73E-16
2.74E-16	2.03E-16	2.97E-17	4.34E-17	2.02E-17	2.35E-18	6.81E-18	4.49E-17	4.47E-16	3.29E-16	9.74E-17	5.79E-17	1.29E-16	1.14E-16	8.60E-17	8.00E-17	1.92E-16	1.07E-16	1.53E-16	2.20E-16
3.06E-16	1.87E-16	4.70E-18	7.11E-17	1.68E-17	4.11E-18	6.28E-18	4.42E-17	3.89E-16	3.97E-16	7.02E-17	7.25E-17	1.29E-16	1.12E-16	6.27E-17	8.95E-17	2.05E-16	1.12E-16	1.42E-16	2.17E-16
3.46E-16	2.26E-16	4.14E-17	8.01E-17	2.51E-17	2.23E-18	3.98E-18	3.27E-17	1.42E-16	1.19E-16	6.24E-17	8.21E-17	1.48E-16	8.92E-17	7.08E-17	1.17E-16	1.62E-16	1.09E-16	1.50E-16	2.08E-16
3.31E-16	2.58E-16	1.13E-16	9.50E-17	2.64E-17	2.96E-18	2.52E-18	2.48E-17	1.45E-17	9.21E-17	5.14E-17	3.27E-17	1.56E-16	6.71E-17	6.97E-17	1.18E-16	1.40E-16	1.08E-16	1.33E-16	1.98E-16
3.30E-16	2.95E-16	9.26E-17	7.98E-17	2.71E-17	5.35E-18	1.24E-18	1.61E-17	1.16E-17	1.30E-16	4.11E-17	1.25E-17	1.43E-16	5.71E-17	6.20E-17	1.52E-16	1.16E-16	8.50E-17	1.11E-16	1.90E-16
3.80E-16	2.67E-16	5.19E-17	8.13E-17	2.50E-17	8.90E-18	3.68E-20	1.16E-17	1.62E-17	1.86E-16	4.43E-17	1.82E-17	4.75E-17	2.46E-17	6.05E-17	2.72E-16	1.37E-16	8.88E-17	1.04E-16	1.87E-16
4.28E-16	2.84E-16	1.20E-16	8.69E-17	3.12E-17	8.42E-18	1.66E-19	8.08E-18	3.56E-17	5.06E-17	3.98E-17	3.05E-17	3.47E-17	6.49E-18	8.37E-17	4.85E-16	1.93E-16	1.62E-16	1.20E-16	1.39E-16
4.65E-16	4.08E-16	2.45E-16	8.23E-17	4.56E-17	1.02E-17	7.06E-21	5.27E-18	3.69E-17	2.54E-17	3.46E-17	1.87E-17	3.54E-17	5.02E-18	6.15E-17	5.74E-16	2.48E-16	9.28E-17	1.17E-16	1.01E-16

5.65E-16	4.27E-16	1.94E-16	8.28E-17	5.09E-17	1.65E-17	5.08E-19	3.51E-18	3.56E-18	1.55E-17	2.23E-17	1.48E-17	3.28E-17	7.49E-18	1.43E-17	4.52E-16	2.25E-16	2.35E-17	1.01E-16	9.28E-17
6.19E-16	4.22E-16	1.78E-16	1.02E-16	5.42E-17	2.63E-17	5.26E-18	6.61E-20	4.59E-18	2.13E-17	1.70E-17	1.85E-17	2.75E-17	5.49E-18	5.00E-18	1.11E-16	5.61E-17	4.33E-17	1.44E-16	9.60E-17
6.70E-16	4.36E-16	2.12E-16	1.17E-16	5.13E-17	3.79E-17	1.33E-17	1.78E-20	5.68E-18	1.97E-17	1.45E-17	5.74E-18	3.32E-17	2.64E-18	6.61E-18	2.01E-16	5.46E-17	1.32E-16	2.05E-16	7.15E-17
6.41E-16	4.72E-16	1.92E-16	1.28E-16	5.36E-17	2.64E-17	1.10E-17	4.57E-19	4.69E-19	3.14E-17	3.72E-18	6.02E-18	3.04E-17	1.83E-18	6.71E-18	2.53E-16	6.36E-17	1.36E-16	6.36E-17	6.55E-17
6.19E-16	4.91E-16	2.23E-16	1.35E-16	5.47E-17	1.97E-17	1.11E-17	1.72E-18	2.60E-19	3.92E-17	2.43E-18	3.19E-18	2.31E-17	3.00E-18	3.30E-18	1.09E-16	4.27E-17	2.31E-17	7.91E-17	7.84E-17
6.49E-16	4.77E-16	1.40E-16	1.69E-16	5.56E-17	2.01E-17	1.27E-17	4.30E-18	1.47E-20	4.14E-17	2.71E-18	1.70E-19	9.78E-18	1.90E-18	2.39E-18	1.68E-21	2.95E-17	3.72E-17	7.81E-17	9.77E-17
7.34E-16	6.20E-16	2.78E-16	1.67E-16	5.42E-17	1.85E-17	7.65E-18	6.10E-18	2.46E-21	5.00E-18	1.21E-18	8.91E-22	1.06E-17	1.22E-18	1.14E-19	7.01E-20	1.87E-17	1.73E-17	8.50E-17	1.47E-16
8.04E-16	7.09E-16	4.32E-16	1.81E-16	4.20E-17	1.10E-17	2.16E-18	9.97E-18	2.50E-19	6.24E-18	5.94E-20	3.43E-20	1.20E-17	2.58E-18	1.33E-19	6.12E-19	4.56E-18	1.83E-18	1.02E-16	2.03E-16
7.86E-16	7.42E-16	4.74E-16	1.72E-16	2.40E-17	7.34E-18	4.13E-19	1.49E-17	1.23E-18	1.70E-18	1.48E-18	1.38E-19	1.18E-17	3.78E-18	1.02E-20	3.30E-18	1.82E-18	2.89E-18	1.02E-16	2.54E-16
8.85E-16	7.19E-16	4.84E-16	1.58E-16	2.16E-17	7.95E-18	3.97E-19	1.20E-17	6.79E-19	4.17E-19	9.04E-19	2.67E-18	7.92E-18	6.80E-18	2.60E-18	3.39E-18	3.18E-20	1.68E-17	5.69E-17	1.47E-16
1.04E-15	7.59E-16	4.12E-16	1.99E-16	5.96E-17	2.77E-17	9.44E-18	1.29E-17	2.61E-22	6.73E-18	1.17E-19	4.80E-19	5.37E-18	4.49E-18	7.42E-18	3.39E-18	1.15E-20	3.99E-17	5.57E-17	1.88E-17
1.16E-15	8.16E-16	3.20E-16	2.97E-16	1.26E-16	6.69E-17	3.05E-17	3.16E-17	3.66E-19	5.89E-18	3.17E-19	1.12E-20	1.14E-17	1.01E-17	3.01E-18	1.20E-18	1.89E-18	1.67E-17	1.80E-17	1.47E-17
1.14E-15	8.21E-16	2.23E-16	3.73E-16	1.75E-16	8.94E-17	3.21E-17	3.81E-17	1.33E-18	3.86E-18	2.59E-18	7.46E-20	2.27E-17	2.56E-17	8.53E-18	6.61E-20	8.36E-18	1.92E-18	2.04E-17	1.64E-17
1.08E-15	7.47E-16	2.52E-16	4.05E-16	2.18E-16	1.16E-16	2.88E-17	4.79E-17	6.89E-18	1.72E-19	2.04E-17	1.84E-17	5.81E-17	7.12E-17	3.68E-17	4.01E-18	1.78E-17	3.29E-18	2.46E-17	1.98E-17
1.11E-15	8.04E-16	6.75E-16	4.50E-16	2.87E-16	1.42E-16	3.60E-17	3.57E-17	1.33E-17	2.77E-18	6.93E-17	1.19E-16	2.04E-16	2.06E-16	1.33E-16	3.87E-17	6.18E-21	1.33E-17	2.39E-17	1.28E-17
1.28E-15	9.79E-16	7.44E-16	5.27E-16	3.12E-16	1.47E-16	5.16E-17	3.12E-17	5.19E-21	3.60E-17	2.41E-16	4.74E-16	6.53E-16	6.60E-16	4.25E-16	1.76E-16	8.60E-18	7.07E-17	1.55E-17	4.50E-18
1.26E-15	1.12E-15	8.37E-16	5.41E-16	3.45E-16	1.73E-16	5.59E-17	4.67E-17	2.58E-17	2.07E-16	8.66E-16	1.37E-15	1.64E-15	1.75E-15	1.24E-15	5.84E-16	7.85E-17	1.88E-16	8.16E-18	6.33E-19
1.22E-15	1.19E-15	8.87E-16	5.17E-16	3.88E-16	2.11E-16	9.03E-17	3.25E-17	4.93E-17	6.52E-16	1.89E-15	2.57E-15	2.80E-15	3.02E-15	2.42E-15	1.38E-15	3.08E-16	3.15E-16	1.48E-17	3.25E-19
1.24E-15	1.21E-15	8.79E-16	5.16E-16	4.48E-16	2.45E-16	1.22E-16	4.01E-18	2.06E-16	1.21E-15	2.69E-15	3.57E-15	3.86E-15	3.95E-15	3.34E-15	2.21E-15	6.80E-16	1.78E-17	4.61E-17	1.92E-19
1.24E-15	1.30E-15	9.66E-16	5.19E-16	4.80E-16	2.23E-16	1.17E-16	6.25E-18	4.08E-16	1.57E-15	3.16E-15	4.31E-15	4.74E-15	4.49E-15	3.83E-15	2.81E-15	1.01E-15	6.46E-17	1.55E-16	2.09E-18
1.25E-15	1.21E-15	9.30E-16	5.21E-16	5.25E-16	2.48E-16	1.23E-16	3.86E-17	5.19E-16	1.75E-15	3.48E-15	4.64E-15	4.99E-15	4.67E-15	3.95E-15	2.90E-15	1.19E-15	9.50E-17	1.82E-16	1.25E-17
1.09E-15	1.05E-15	8.14E-16	5.37E-16	5.44E-16	3.13E-16	1.53E-16	6.68E-17	5.11E-16	1.63E-15	3.66E-15	4.64E-15	4.77E-15	4.51E-15	3.82E-15	2.59E-15	1.14E-15	9.92E-17	2.81E-17	1.94E-17
8.55E-16	8.41E-16	8.18E-16	5.64E-16	5.14E-16	3.47E-16	1.92E-16	1.01E-16	4.10E-16	1.28E-15	3.70E-15	4.38E-15	4.22E-15	4.23E-15	3.68E-15	2.27E-15	9.44E-16	8.96E-17	6.40E-18	1.67E-17
4.82E-16	5.50E-16	6.96E-16	5.02E-16	4.60E-16	3.67E-16	2.35E-16	1.15E-16	3.35E-16	8.77E-16	3.43E-15	3.63E-15	3.27E-15	3.74E-15	3.42E-15	1.96E-15	7.79E-16	8.48E-17	2.86E-17	2.88E-17
9.47E-17	1.95E-16	3.51E-16	3.28E-16	3.81E-16	3.70E-16	2.63E-16	1.63E-18	2.68E-16	4.55E-16	2.17E-15	2.14E-15	2.04E-15	2.34E-15	2.47E-15	1.38E-15	3.69E-16	7.46E-17	4.34E-17	4.50E-17
4.61E-17	1.07E-18	7.76E-17	1.18E-16	2.37E-16	3.70E-16	2.89E-16	2.23E-16	1.52E-16	1.04E-16	6.52E-16	7.73E-16	7.48E-16	6.81E-16	1.02E-15	6.40E-16	1.13E-16	1.11E-16	6.73E-17	4.57E-17
5.90E-16	2.96E-16	1.08E-17	6.82E-19	6.37E-17	2.79E-16	2.76E-16	2.28E-16	1.21E-16	4.35E-18	6.67E-17	2.73E-16	2.12E-16	2.34E-16	2.33E-16	6.11E-18	9.45E-17	1.35E-16	7.23E-17	4.46E-17
1.88E-15	1.10E-15	2.88E-16	1.52E-16	4.96E-18	1.22E-16	2.49E-16	2.28E-16	1.82E-16	1.40E-16	7.10E-17	1.04E-17	1.87E-18	9.52E-17	1.54E-16	1.66E-16	1.00E-16	1.18E-16	6.30E-17	6.37E-17
4.13E-15	2.65E-15	1.16E-15	6.93E-16	2.16E-16	8.23E-18	1.76E-16	2.20E-16	2.14E-16	1.49E-16	1.28E-16	6.67E-17	1.43E-16	1.89E-16	1.62E-16	1.81E-16	9.78E-17	8.61E-17	6.12E-17	8.52E-17
7.34E-15	5.25E-15	2.83E-15	1.70E-15	8.36E-16	5.27E-17	8.72E-17	2.07E-16	2.56E-16	1.51E-16	1.36E-16	6.96E-17	1.44E-16	2.05E-16	1.57E-16	1.62E-16	7.84E-17	3.07E-17	5.43E-17	8.31E-17
1.19E-14	9.14E-15	5.35E-15	3.29E-15	1.93E-15	3.96E-16	1.64E-17	1.63E-16	2.56E-16	1.41E-16	1.38E-16	7.29E-17	1.35E-16	2.16E-16	1.80E-16	1.50E-16	5.38E-17	1.40E-17	5.81E-17	9.11E-17
1.84E-14	1.46E-14	8.99E-15	5.58E-15	3.87E-15	1.32E-15	2.68E-17	8.67E-17	1.91E-16	1.27E-16	1.35E-16	9.01E-17	1.13E-16	2.27E-16	2.08E-16	1.64E-16	2.19E-17	5.33E-17	1.34E-16	9.83E-17
2.77E-14	2.28E-14	1.45E-14	8.49E-15	7.04E-15	3.01E-15	2.99E-16	1.24E-17	1.59E-16	1.35E-16	1.55E-16	1.24E-16	1.40E-16	2.46E-16	1.99E-16	1.94E-16	3.43E-17	5.65E-17	1.44E-16	1.07E-16
3.99E-14	3.44E-14	2.19E-14	1.19E-14	1.16E-14	5.36E-15	7.98E-16	1.26E-17	8.93E-17	1.73E-16	1.76E-16	1.64E-16	2.00E-16	2.47E-16	1.90E-16	1.85E-16	2.27E-16	1.93E-16	1.47E-16	1.09E-16
5.38E-14	4.83E-14	3.07E-14	1.51E-14	1.56E-14	6.55E-15	1.31E-15	1.20E-16	1.07E-16	2.65E-16	1.84E-16	1.88E-16	2.24E-16	2.76E-16	2.31E-16	1.44E-16	2.30E-16	1.49E-16	1.46E-16	1.25E-16
6.48E-14	5.92E-14	3.76E-14	1.71E-14	1.51E-14	5.60E-15	1.69E-15	2.52E-16	7.95E-17	3.05E-16	1.84E-16	1.93E-16	2.31E-16	3.15E-16	2.72E-16	1.60E-16	2.33E-16	3.15E-16	1.66E-16	1.42E-16
6.33E-14	5.71E-14	3.88E-14	1.76E-14	1.06E-14	5.14E-15	1.95E-15	3.06E-16	7.68E-17	3.55E-16	2.59E-16	2.02E-16	2.33E-16	3.25E-16	3.17E-16	2.64E-16	2.36E-16	2.91E-16	1.97E-16	1.51E-16
5.54E-14	4.95E-14	3.35E-14	1.67E-14	1.04E-14	5.20E-15	2.03E-15	2.97E-16	4.19E-17	5.05E-16	3.26E-16	2.16E-16	2.40E-16	2.88E-16	3.19E-16	2.78E-16	2.01E-16	3.36E-16	2.19E-16	1.54E-16
4.72E-14	4.04E-14	2.39E-14	1.52E-14	9.72E-15	5.10E-15	2.08E-15	2.86E-16	3.34E-17	5.46E-16	3.49E-16	2.31E-16	2.35E-16	3.06E-16	3.10E-16	3.00E-16	1.49E-16	2.79E-16	2.37E-16	1.97E-16
3.96E-14	2.95E-14	1.93E-14	1.32E-14	8.86E-15	4.91E-15	2.11E-15	3.25E-16	5.49E-17	5.34E-16	3.80E-16	2.63E-16	2.50E-16	3.68E-16	3.32E-16	2.79E-16	1.10E-16	1.93E-16	2.29E-16	2.32E-16
3.33E-14	2.34E-14	1.66E-14	1.14E-14	8.16E-15	4.74E-15	2.17E-15	4.29E-16	1.69E-16	5.06E-16	4.08E-16	2.69E-16	2.78E-16	3.99E-16	3.88E-16	2.31E-16	9.45E-17	1.19E-16	2.30E-16	2.25E-16
2.72E-14	1.96E-14	1.45E-14	9.92E-15	7.39E-15	4.60E-15	2.26E-15	5.84E-16	8.00E-17	4.88E-16	4.43E-16	3.08E-16	3.37E-16	3.94E-16	3.98E-16	2.36E-16	3.67E-16	3.22E-16	2.58E-16	2.44E-16
2.12E-14	1.65E-14	1.29E-14	8.51E-15	6.50E-15	4.38E-15	2.34E-15	7.48E-16	4.58E-17	4.50E-16	4.92E-16	4.06E-16	4.07E-16	4.21E-16	4.27E-16	3.89E-16	4.68E-16	3.28E-16	2.59E-16	3.08E-16
1.68E-14	1.41E-14	1.12E-14	7.34E-15	5.87E-15	4.04E-15	2.30E-15	7.73E-16	2.33E-17	4.13E-16	5.21E-16	4.64E-16	5.17E-16	4.75E-16	4.80E-16	4.30E-16	5.56E-16	4.51E-16	2.59E-16	2.92E-16
1.33E-14	1.19E-14	9.27E-15	6.56E-15	5.37E-15	3.59E-15	2.00E-15	7.20E-16	2.87E-18	3.98E-16	5.12E-16	5.01E-16	5.61E-16	5.80E-16	5.25E-16	4.88E-16	5.54E-16	3.26E-16	3.29E-16	3.21E-16
1.06E-14	1.01E-14	7.97E-15	5.96E-15	4.89E-15	3.23E-15	1.74E-15	6.78E-16	1.29E-18	3.71E-16	4.78E-16	5.19E-16	6.00E-16	6.61E-16	5.55E-16	5.40E-16	5.44E-16	2.61E-16	3.32E-16	3.55E-16
8.70E-15	8.58E-15	7.00E-15	5.41E-15	4.38E-15	2.92E-15	1.62E-15	6.54E-16	2.27E-18	3.59E-16	4.08E-16	5.66E-16	6.39E-16	6.27E-16	6.08E-16	5.95E-16	5.12E-16	2.03E-16	3.34E-16	3.74E-16
7.28E-15	7.38E-15	6.43E-15	4.74E-15	3.87E-15	2.66E-15	1.51E-15	6.17E-16	1.95E-18	3.05E-16	3.47E-16	5.68E-16	6.43E-16	6.64E-16	6.40E-16	5.95E-16	4.92E-16	7.91E-17	4.00E-16	4.08E-16
6.10E-15	6.53E-15	5.87E-15	4.23E-15	3.49E-15	2.47E-15	1.39E-15	5.90E-16	3.12E-18	2.32E-16	3.72E-16	5.43E-16	6.56E-16	7.58E-16	6.03E-16	6.32E-16	4.26E-16	1.37E-16	4.62E-16	4.58E-16
5.13E-15	5.94E-15	5.23E-15	3.74E-15	3.12E-15	2.28E-15	1.31E-15	6.14E-16	1.45E-17	1.82E-16	3.85E-16	5.17E-16	6.63E-16	7.95E-16	5.65E-16	6.73E-16	3.44E-16	4.54E-16	4.82E-16	5.29E-16
4.11E-15	4.89E-15	4.27E-15	3.13E-15	2.50E-15	1.91E-15	1.17E-15	6.36E-16	6.18E-17	1.17E-16	3.61E-16	5.36E-16	7.21E-16	7.37E-16	6.13E-16	7.65E-16	4.49E-16	3.95E-16	4.68E-16	5.94E-16
2.72E-15	3.61E-15	2.85E-15	2.37E-15	1.63E-15	1.27E-15	7.82E-16	5.62E-16	1.74E-16	2.64E-17	3.11E-16	5.96E-16	6.75E-16	8.14E-16	6.79E-16	7.59E-16	8.11E-16	2.51E-16	6.53E-16	6.68E-16
1.21E-15	2.32E-15	1.62E-15	1.42E-15	8.01E-16	6.10E-16	3.35E-16	3.69E-16	2.83E-16	1.32E-18	2.73E-16	5.36E-16	6.03E-16	8.88E-16	7.60E-16	8.03E-16	7.89E-16	3.16E-16	6.47E-16	6.98E-16
3.41E-16	8.27E-16	5.88E-16	6.12E-16	2.41E-16	1.65E-16	5.44E-17	1.84E-16	2.26E-16	7.26E-18	3.97E-16	4.94E-16	5.64E-16	9.50E-16	7.94E-16	8.65E-16	8.60E-16	5.09E-16	6.49E-16	7.17E-16
3.68E-18	3.64E-17	2.22E-17	1.62E-16	2.80E-18	1.19E-19	1.92E-17	3.45E-17	5.49E-17	8.63E-17	6.15E-16	6.09E-16	6.89E-16	9.56E-16	8.94E-16	8.49E-16	8.96E-16	4.59E-16	6.70E-16	7.56E-16
1.99E-16	1.87E-16	1.06E-16	4.01E-18	1.42E-16	1.56E-16	2.66E-16	3.74E-17	6.29E-17	3.49E-16	8.91E-16	7.57E-16	8.50E-16	8.96E-16	9.17E-16	8.23E-16	8.68E-16	5.46E-16	6.68E-16	7.62E-16
1.03E-15	7.85E-16	4.04E-16	1.79E-16	5.27E-16	5.79E-16	6.98E-16	4.55E-16	2.29E-16	7.16E-16	1.01E-15	8.07E-16	8.99E-16	8.63E-16	9.13E-16	8.80E-16	8.55E-16	3.80E-16	6.57E-16	7.63E-16
1.78E-15	1.42E-15	5.53E-16	7.42E-16	9.94E-16	1.07E-15	1.17E-15	8.08E-16	5.24E-16	9.09E-16	1.01E-15	7.51E-16	9.03E-16	9.14E-16	9.01E-16	9.35E-16	8.54E-16	4.14E-16	6.91E-16	7.75E-16
2.17E-15	1.96E-15	7.06E-16	1.09E-15	1.36E-15	1.37E-15	1.39E-15	1.20E-15	7.21E-16	9.53E-16	1.06E-15	7.06E-16	9.42E-16	1.02E-15	1.00E-15	8.63E-16	7.59E-16	7.64E-16	7.33E-16	7.78E-16
2.15E-15	2.14E-15	1.23E-15	1.57E-15	1.49E-15	1.36E-15	1.34E-15	1.12E-15	1.08E-15	9.46E-16	1.06E-15	6.82E-16	9.62E-16	1.02E-15	1.07E-15	8.54E-16	7.17E-16	9.07E-16	8.23E-16	8.04E-16
2.16E-15	2.15E-15	9.42E-16	1.58E-15	1.51E-15	1.34E-15	1.26E-15	1.01E-15	9.82E-16	1.06E-15	1.05E-15	7.72E-16	9.46E-16	1.09E-15	1.13E-15	1.01E-15	9.36E-16	8.05E-16	8.60E-16	7.92E-16
2.27E-15	2.11E-15	9.05E-16	1.58E-15	1.45E-15	1.36E-15	1.26E-15	8.97E-16	9.84E-16	1.08E-15	1.15E-15	9.12E-16	1.02E-15	1.12E-15	1.19E-15	1.24E-15	9.29E-16	9.08E-16	8.92E-16	7.66E-16
2.39E-15	2.08E-15	1.46E-15	1.45E-15	1.42E-15	1.38E-15	1.30E-15	7.71E-16	8.68E-16	9.61E-16	1.23E-15	9.51E-16	1.06E-15	1.11E-15	1.20E-15	1.23E-15	9.40E-16	8.53E-16	8.89E-16	7.55E-16

2.47E-15	2.09E-15	1.21E-15	1.31E-15	1.46E-15	1.35E-15	1.29E-15	6.86E-16	6.50E-16	8.75E-16	1.29E-15	9.60E-16	1.03E-15	1.11E-15	1.27E-15	1.18E-15	8.87E-16	8.51E-16	9.34E-16	8.31E-16	
2.53E-15	2.02E-15	1.05E-15	1.56E-15	1.59E-15	1.40E-15	1.28E-15	1.02E-15	8.21E-16	9.60E-16	1.28E-15	1.02E-15	1.09E-15	1.13E-15	1.31E-15	1.23E-15	1.17E-15	1.09E-15	1.01E-15	9.54E-16	
2.51E-15	1.89E-15	1.84E-15	1.78E-15	1.61E-15	1.48E-15	1.36E-15	1.30E-15	9.56E-16	8.47E-16	1.30E-15	1.10E-15	1.21E-15	1.13E-15	1.32E-15	1.25E-15	1.08E-15	1.06E-15	9.88E-16	9.55E-16	
2.52E-15	1.63E-15	2.06E-15	1.82E-15	1.61E-15	1.54E-15	1.40E-15	1.30E-15	1.01E-15	9.63E-16	1.36E-15	1.08E-15	1.23E-15	1.17E-15	1.33E-15	1.21E-15	1.07E-15	1.19E-15	9.85E-16	9.52E-16	
2.38E-15	1.30E-15	2.06E-15	1.87E-15	1.70E-15	1.58E-15	1.36E-15	1.29E-15	1.06E-15	1.23E-15	1.44E-15	1.11E-15	1.23E-15	1.16E-15	1.35E-15	1.25E-15	1.07E-15	1.12E-15	9.92E-16	9.68E-16	
2.29E-15	1.12E-15	2.14E-15	1.89E-15	1.79E-15	1.66E-15	1.37E-15	1.34E-15	9.08E-16	1.13E-15	1.38E-15	1.28E-15	1.29E-15	1.19E-15	1.42E-15	1.38E-15	1.02E-15	9.71E-16	1.04E-15	1.01E-15	
2.31E-15	2.24E-15	2.17E-15	1.95E-15	1.85E-15	1.70E-15	1.37E-15	1.34E-15	7.71E-16	1.08E-15	1.42E-15	1.35E-15	1.32E-15	1.23E-15	1.48E-15	1.50E-15	8.47E-16	9.82E-16	1.12E-15	1.02E-15	
2.32E-15	2.25E-15	2.08E-15	1.88E-15	1.84E-15	1.71E-15	1.38E-15	1.36E-15	1.35E-15	9.52E-16	1.47E-15	1.36E-15	1.32E-15	1.24E-15	1.49E-15	1.55E-15	7.43E-16	1.03E-15	1.09E-15	9.93E-16	
2.24E-15	2.12E-15	1.94E-15	1.89E-15	1.84E-15	1.76E-15	1.48E-15	1.37E-15	1.53E-15	8.62E-16	1.52E-15	1.32E-15	1.38E-15	1.31E-15	1.56E-15	1.55E-15	6.10E-16	9.19E-16	1.06E-15	1.01E-15	
2.11E-15	2.08E-15	1.95E-15	1.94E-15	1.91E-15	1.76E-15	1.47E-15	1.42E-15	1.54E-15	1.03E-15	1.47E-15	1.28E-15	1.48E-15	1.37E-15	1.53E-15	1.53E-15	7.37E-16	1.21E-15	1.12E-15	9.99E-16	
2.19E-15	2.17E-15	2.09E-15	1.99E-15	1.88E-15	1.73E-15	1.52E-15	1.44E-15	1.62E-15	1.17E-15	1.46E-15	1.25E-15	1.60E-15	1.47E-15	1.52E-15	1.51E-15	8.87E-16	1.44E-15	1.17E-15	9.70E-16	
2.52E-15	2.10E-15	2.17E-15	1.81E-15	1.76E-15	1.73E-15	1.62E-15	1.46E-15	1.58E-15	1.20E-15	1.51E-15	1.18E-15	1.71E-15	1.52E-15	1.62E-15	1.54E-15	1.41E-15	1.38E-15	1.09E-15	9.02E-16	
2.49E-15	1.98E-15	2.24E-15	1.70E-15	1.78E-15	1.78E-15	1.63E-15	1.52E-15	1.43E-15	1.04E-15	1.47E-15	1.20E-15	1.84E-15	1.74E-15	1.68E-15	1.53E-15	1.46E-15	1.31E-15	1.00E-15	8.80E-16	
2.53E-15	1.78E-15	2.23E-15	1.68E-15	1.84E-15	1.76E-15	1.63E-15	1.52E-15	1.29E-15	9.26E-16	1.34E-15	1.17E-15	1.99E-15	1.94E-15	1.63E-15	1.49E-15	1.53E-15	1.17E-15	1.03E-15	1.01E-15	
2.51E-15	1.53E-15	2.32E-15	1.73E-15	1.75E-15	1.74E-15	1.60E-15	1.51E-15	1.24E-15	1.03E-15	1.25E-15	1.17E-15	1.80E-15	1.80E-15	1.51E-15	1.49E-15	1.36E-15	9.93E-16	1.13E-15	1.15E-15	
2.46E-15	1.71E-15	2.29E-15	1.53E-15	1.59E-15	1.74E-15	1.57E-15	1.46E-15	1.01E-15	1.11E-15	1.18E-15	1.09E-15	1.47E-15	1.51E-15	1.48E-15	1.47E-15	1.16E-15	9.43E-16	1.21E-15	1.16E-15	
2.57E-15	2.41E-15	2.20E-15	1.37E-15	1.51E-15	1.77E-15	1.60E-15	1.30E-15	9.03E-16	1.10E-15	1.14E-15	1.07E-15	1.26E-15	1.41E-15	1.47E-15	1.40E-15	1.05E-15	1.36E-15	1.21E-15	9.78E-16	
6.78E-13	5.79E-13	4.01E-13	2.52E-13	2.04E-13	1.35E-13	9.25E-14	6.80E-14	6.07E-14	7.20E-14	9.33E-14	8.90E-14	9.72E-14	1.04E-13	1.02E-13	9.20E-14	7.65E-14	7.07E-14	7.48E-14	7.42E-14	4.25E-11

SUM OF SQUARED QUADR		4.25E-11
TOTAL NUMBER VALUES IN		16,384
AVERAGE SQUARED		2.60E-15
RMS	5.0954E-08	

Quadrant 2 Data (16384 values):

5.00E-08	3.72E-08	3.98E-08	4.04E-08	3.88E-08	3.73E-08	4.13E-08	3.89E-08	4.23E-08	5.16E-08	5.38E-08	5.03E-08	5.47E-08	5.20E-08	4.37E-08	4.29E-08	4.32E-08	4.39E-08
4.36E-08	3.39E-08	3.83E-08	3.78E-08	3.88E-08	3.63E-08	4.10E-08	3.86E-08	4.16E-08	5.16E-08	5.62E-08	5.24E-08	5.54E-08	5.41E-08	4.32E-08	4.20E-08	4.33E-08	4.26E-08
4.12E-08	2.99E-08	3.61E-08	3.57E-08	3.83E-08	3.52E-08	3.60E-08	3.59E-08	4.12E-08	5.06E-08	5.66E-08	5.22E-08	5.41E-08	5.36E-08	4.33E-08	4.16E-08	4.25E-08	4.18E-08
3.93E-08	2.94E-08	3.54E-08	3.48E-08	3.88E-08	3.63E-08	3.43E-08	3.47E-08	4.00E-08	4.87E-08	5.40E-08	5.07E-08	5.01E-08	5.04E-08	4.39E-08	4.17E-08	4.15E-08	4.04E-08
3.77E-08	2.85E-08	3.49E-08	3.32E-08	3.91E-08	3.64E-08	3.33E-08	3.50E-08	3.91E-08	4.56E-08	4.80E-08	4.90E-08	4.67E-08	4.89E-08	4.47E-08	4.34E-08	4.00E-08	3.95E-08
3.63E-08	2.75E-08	3.39E-08	3.19E-08	3.64E-08	3.37E-08	3.28E-08	3.51E-08	3.81E-08	4.29E-08	4.32E-08	4.73E-08	4.45E-08	4.75E-08	4.56E-08	4.51E-08	3.85E-08	3.82E-08
3.37E-08	2.68E-08	3.32E-08	3.17E-08	3.36E-08	3.20E-08	3.25E-08	3.38E-08	3.69E-08	4.01E-08	4.11E-08	4.33E-08	4.28E-08	4.57E-08	4.72E-08	4.35E-08	3.86E-08	3.79E-08
3.20E-08	2.63E-08	3.29E-08	3.18E-08	3.28E-08	3.20E-08	3.18E-08	3.23E-08	3.56E-08	3.77E-08	3.86E-08	4.06E-08	4.14E-08	4.29E-08	4.71E-08	4.34E-08	3.92E-08	3.75E-08
3.11E-08	2.62E-08	3.30E-08	3.14E-08	3.25E-08	3.15E-08	3.04E-08	3.14E-08	3.43E-08	3.62E-08	3.62E-08	3.85E-08	4.02E-08	4.06E-08	4.63E-08	4.50E-08	4.03E-08	3.80E-08
3.10E-08	2.63E-08	3.31E-08	3.08E-08	3.26E-08	3.06E-08	2.95E-08	3.10E-08	3.33E-08	3.47E-08	3.55E-08	3.76E-08	3.91E-08	3.97E-08	4.55E-08	4.59E-08	4.28E-08	3.94E-08
3.09E-08	2.68E-08	3.29E-08	3.10E-08	3.20E-08	3.00E-08	2.93E-08	2.97E-08	3.18E-08	3.20E-08	3.65E-08	3.80E-08	3.79E-08	3.82E-08	4.27E-08	4.41E-08	4.41E-08	4.03E-08
3.00E-08	2.66E-08	3.16E-08	3.16E-08	3.09E-08	2.98E-08	2.90E-08	2.81E-08	3.04E-08	3.00E-08	3.83E-08	3.99E-08	3.75E-08	3.63E-08	3.80E-08	4.00E-08	4.31E-08	4.00E-08
2.87E-08	2.56E-08	2.98E-08	3.02E-08	3.00E-08	2.99E-08	2.85E-08	2.75E-08	2.92E-08	2.90E-08	3.99E-08	4.08E-08	3.64E-08	3.29E-08	3.47E-08	3.65E-08	3.93E-08	4.00E-08
2.85E-08	2.50E-08	2.88E-08	2.78E-08	2.99E-08	2.95E-08	2.80E-08	2.77E-08	2.75E-08	2.89E-08	4.12E-08	4.14E-08	3.38E-08	2.98E-08	3.27E-08	3.40E-08	3.51E-08	4.08E-08
2.87E-08	2.45E-08	2.79E-08	2.69E-08	2.92E-08	2.87E-08	2.77E-08	2.78E-08	2.58E-08	2.83E-08	3.94E-08	4.00E-08	3.28E-08	2.87E-08	3.12E-08	3.19E-08	3.37E-08	4.07E-08
2.81E-08	2.37E-08	2.72E-08	2.71E-08	2.86E-08	2.87E-08	2.76E-08	2.84E-08	2.53E-08	2.64E-08	3.20E-08	3.51E-08	3.24E-08	2.86E-08	3.08E-08	2.92E-08	3.17E-08	3.90E-08
2.76E-08	2.25E-08	2.65E-08	2.73E-08	2.88E-08	2.98E-08	2.74E-08	2.96E-08	2.56E-08	2.56E-08	2.61E-08	2.92E-08	3.17E-08	2.79E-08	2.92E-08	2.76E-08	2.84E-08	3.55E-08
2.81E-08	2.12E-08	2.59E-08	2.61E-08	2.97E-08	3.12E-08	2.70E-08	3.07E-08	2.52E-08	2.58E-08	2.59E-08	2.77E-08	3.10E-08	2.74E-08	2.78E-08	2.84E-08	2.86E-08	3.43E-08
2.91E-08	1.97E-08	2.53E-08	2.54E-08	2.95E-08	3.13E-08	2.65E-08	3.02E-08	2.31E-08	2.61E-08	2.60E-08	2.72E-08	3.07E-08	2.71E-08	2.75E-08	2.84E-08	3.05E-08	3.65E-08
2.99E-08	1.91E-08	2.54E-08	2.47E-08	2.74E-08	2.99E-08	2.69E-08	2.94E-08	2.33E-08	2.63E-08	2.57E-08	2.69E-08	3.13E-08	2.75E-08	2.67E-08	2.71E-08	2.97E-08	3.69E-08
2.98E-08	2.15E-08	2.63E-08	2.47E-08	2.43E-08	2.64E-08	2.57E-08	2.55E-08	2.44E-08	2.66E-08	2.49E-08	2.53E-08	3.09E-08	2.73E-08	2.68E-08	2.77E-08	2.59E-08	2.98E-08
3.06E-08	2.48E-08	2.65E-08	2.39E-08	2.37E-08	2.51E-08	2.61E-08	2.42E-08	2.44E-08	2.70E-08	2.43E-08	2.39E-08	3.01E-08	2.76E-08	2.79E-08	3.13E-08	2.51E-08	2.42E-08
3.10E-08	2.78E-08	2.65E-08	2.32E-08	2.35E-08	2.33E-08	2.66E-08	2.50E-08	2.30E-08	2.82E-08	2.44E-08	2.40E-08	2.91E-08	2.88E-08	2.87E-08	3.66E-08	2.77E-08	2.31E-08
2.98E-08	2.95E-08	2.65E-08	2.34E-08	2.34E-08	2.18E-08	2.50E-08	2.60E-08	2.28E-08	2.89E-08	2.18E-08	2.37E-08	2.91E-08	2.94E-08	2.87E-08	3.88E-08	2.95E-08	2.34E-08
2.89E-08	3.21E-08	2.86E-08	2.44E-08	2.36E-08	2.16E-08	2.54E-08	2.75E-08	2.35E-08	2.99E-08	2.01E-08	2.38E-08	2.78E-08	2.63E-08	2.52E-08	3.38E-08	2.72E-08	2.52E-08
2.82E-08	3.46E-08	2.93E-08	2.59E-08	2.37E-08	2.20E-08	2.88E-08	3.00E-08	2.36E-08	2.93E-08	1.96E-08	2.39E-08	2.62E-08	2.41E-08	2.13E-08	2.66E-08	2.02E-08	2.61E-08
2.86E-08	2.87E-08	2.90E-08	2.80E-08	2.48E-08	2.23E-08	3.41E-08	3.41E-08	2.09E-08	2.40E-08	1.97E-08	2.34E-08	2.52E-08	2.46E-08	2.04E-08	2.44E-08	2.09E-08	2.65E-08
2.75E-08	2.68E-08	2.78E-08	2.92E-08	2.67E-08	2.28E-08	3.74E-08	3.65E-08	1.99E-08	2.19E-08	2.03E-08	2.12E-08	2.38E-08	2.49E-08	2.07E-08	2.53E-08	2.18E-08	2.78E-08
2.56E-08	2.82E-08	2.72E-08	3.02E-08	2.83E-08	2.38E-08	3.01E-08	2.86E-08	1.90E-08	2.16E-08	2.08E-08	1.97E-08	2.33E-08	2.37E-08	2.09E-08	2.55E-08	2.31E-08	3.18E-08
2.53E-08	3.15E-08	2.65E-08	3.07E-08	2.79E-08	2.53E-08	2.23E-08	2.17E-08	1.89E-08	2.18E-08	2.03E-08	1.85E-08	2.33E-08	2.23E-08	2.10E-08	2.67E-08	2.61E-08	3.48E-08
2.64E-08	3.11E-08	2.67E-08	3.13E-08	2.76E-08	2.58E-08	2.21E-08	2.17E-08	1.89E-08	2.17E-08	1.90E-08	1.74E-08	2.29E-08	2.28E-08	2.04E-08	2.70E-08	2.57E-08	3.37E-08
2.75E-08	3.60E-08	2.79E-08	3.13E-08	2.56E-08	2.37E-08	2.35E-08	2.17E-08	1.83E-08	2.17E-08	1.89E-08	1.73E-08	2.21E-08	2.23E-08	1.82E-08	2.45E-08	2.49E-08	2.92E-08
2.73E-08	3.35E-08	2.70E-08	2.70E-08	2.44E-08	2.33E-08	2.37E-08	2.13E-08	1.89E-08	2.12E-08	1.88E-08	1.75E-08	2.08E-08	2.06E-08	1.73E-08	2.12E-08	2.31E-08	2.47E-08
2.52E-08	2.73E-08	2.45E-08	2.51E-08	2.38E-08	2.32E-08	2.23E-08	2.16E-08	2.07E-08	2.06E-08	1.88E-08	1.76E-08	1.94E-08	1.90E-08	1.69E-08	1.74E-08	1.91E-08	2.21E-08
2.26E-08	2.41E-08	2.32E-08	2.32E-08	2.33E-08	2.24E-08	2.16E-08	2.22E-08	2.17E-08	2.00E-08	1.97E-08	1.68E-08	1.93E-08	1.79E-08	1.61E-08	1.56E-08	1.62E-08	2.08E-08
2.10E-08	2.01E-08	2.35E-08	2.24E-08	2.35E-08	2.13E-08	2.17E-08	2.21E-08	2.12E-08	1.97E-08	2.09E-08	1.76E-08	1.92E-08	1.79E-08	1.54E-08	1.55E-08	1.58E-08	1.77E-08
2.06E-08	1.92E-08	2.36E-08	2.38E-08	2.37E-08	2.15E-08	2.18E-08	2.16E-08	2.11E-08	2.02E-08	2.15E-08	1.90E-08	1.94E-08	1.82E-08	1.61E-08	1.61E-08	1.71E-08	1.42E-08
1.97E-08	2.02E-08	2.35E-08	2.58E-08	2.40E-08	2.09E-08	2.10E-08	2.24E-08	2.22E-08	2.04E-08	2.13E-08	2.06E-08	1.97E-08	1.87E-08	1.77E-08	1.68E-08	1.86E-08	1.27E-08
1.94E-08	1.91E-08	2.31E-08	2.51E-08	2.36E-08	1.93E-08	2.04E-08	2.41E-08	2.32E-08	1.97E-08	2.02E-08	2.16E-08	2.07E-08	2.07E-08	2.03E-08	1.75E-08	2.07E-08	1.35E-08
1.85E-08	1.81E-08	2.26E-08	2.05E-08	2.04E-08	1.77E-08	1.92E-08	2.39E-08	2.20E-08	1.88E-08	1.95E-08	2.14E-08	2.04E-08	2.25E-08	2.27E-08	1.91E-08	2.33E-08	1.55E-08
1.78E-08	1.66E-08	1.92E-08	1.62E-08	1.75E-08	1.75E-08	1.72E-08	2.17E-08	1.93E-08	1.77E-08	1.84E-08	1.98E-08	1.90E-08	2.35E-08	2.35E-08	1.91E-08	2.32E-08	1.74E-08
1.70E-08	1.60E-08	1.84E-08	1.47E-08	1.70E-08	1.87E-08	1.64E-08	2.04E-08	1.84E-08	1.69E-08	1.81E-08	1.70E-08	1.82E-08	2.52E-08	2.56E-08	1.83E-08	2.19E-08	1.80E-08
1.61E-08	1.68E-08	1.80E-08	1.49E-08	1.73E-08	1.75E-08	1.54E-08	2.01E-08	1.89E-08	1.66E-08	1.68E-08	1.66E-08	1.84E-08	3.03E-08	2.98E-08	1.66E-08	2.18E-08	1.73E-08
1.55E-08	1.85E-08	1.80E-08	1.57E-08	1.69E-08	1.59E-08	1.49E-08	1.94E-08	1.79E-08	1.55E-08	1.47E-08	1.64E-08	1.87E-08	3.25E-08	3.31E-08	1.58E-08	2.21E-08	1.61E-08
1.59E-08	2.07E-08	1.66E-08	1.70E-08	1.73E-08	1.51E-08	1.50E-08	1.84E-08	1.65E-08	1.48E-08	1.37E-08	1.70E-08	1.88E-08	2.91E-08	2.54E-08	1.62E-08	2.12E-08	1.58E-08
1.54E-08	2.11E-08	1.51E-08	1.62E-08	1.77E-08	1.60E-08	1.59E-08	1.78E-08	1.54E-08	1.41E-08	1.38E-08	1.73E-08	1.68E-08	1.85E-08	1.41E-08	1.66E-08	1.96E-08	1.53E-08
1.33E-08	1.32E-08	1.50E-08	1.52E-08	1.53E-08	1.62E-08	1.68E-08	1.78E-08	1.50E-08	1.36E-08	1.38E-08	1.59E-08	1.50E-08	1.74E-08	1.28E-08	1.65E-08	1.75E-08	1.54E-08
1.14E-08	1.25E-08	1.53E-08	1.37E-08	1.47E-08	1.60E-08	1.69E-08	1.69E-08	1.49E-08	1.31E-08	1.30E-08	1.49E-08	1.26E-08	1.37E-08	1.20E-08	1.45E-08	1.57E-08	1.47E-08
1.10E-08	1.41E-08	1.39E-08	1.38E-08	1.51E-08	1.35E-08	1.62E-08	1.52E-08	1.38E-08	1.25E-08	1.22E-08	1.47E-08	1.14E-08	1.20E-08	1.22E-08	1.27E-08	1.42E-08	1.29E-08
1.07E-08	1.59E-08	1.39E-08	1.41E-08	1.53E-08	1.31E-08	1.54E-08	1.52E-08	1.34E-08	1.19E-08	1.11E-08	1.28E-08	1.09E-08	1.09E-08	1.13E-08	1.15E-08	1.27E-08	1.20E-08
1.09E-08	1.78E-08	1.38E-08	1.32E-08	1.48E-08	1.29E-08	1.42E-08	1.59E-08	1.39E-08	1.25E-08	9.53E-09	9.67E-09	1.04E-08	9.20E-09	1.02E-08	1.09E-08	1.31E-08	1.17E-08
1.03E-08	1.18E-08	1.24E-08	1.24E-08	1.37E-08	1.26E-08	1.40E-08	1.51E-08	1.45E-08	1.50E-08	9.41E-09	9.24E-09	1.04E-08	8.63E-09	9.35E-09	1.04E-08	1.28E-08	1.18E-08
9.28E-09	1.11E-08	1.17E-08	1.14E-08	1.24E-08	1.28E-08	1.47E-08	1.41E-08	1.45E-08	1.73E-08	9.62E-09	8.60E-09	9.37E-09	9.13E-09	9.89E-09	1.02E-08	1.25E-08	1.15E-08
9.50E-09	1.43E-08	1.08E-08	1.09E-08	1.21E-08	1.33E-08	1.59E-08	1.40E-08	1.36E-08	1.56E-08	9.05E-09	7.27E-09	8.43E-09	9.14E-09	1.16E-08	1.03E-08	1.35E-08	1.03E-08
9.69E-09	1.37E-08	9.33E-09	1.11E-08	1.17E-08	1.33E-08	1.58E-08	1.36E-08	1.12E-08	1.13E-08	7.72E-09	6.52E-09	7.92E-09	8.65E-09	1.22E-08	1.05E-08	1.45E-08	9.30E-09

8.40E-09	1.27E-08	9.01E-09	1.08E-08	1.07E-08	1.36E-08	1.53E-08	1.12E-08	9.81E-09	9.82E-09	7.76E-09	6.48E-09	7.31E-09	7.65E-09	1.02E-08	1.05E-08	1.24E-08	9.44E-09
7.37E-09	6.31E-09	8.34E-09	1.01E-08	1.02E-08	1.18E-08	1.18E-08	9.13E-09	1.01E-08	9.69E-09	7.84E-09	7.16E-09	6.64E-09	6.89E-09	8.27E-09	9.81E-09	1.01E-08	8.41E-09
6.25E-09	7.39E-09	7.45E-09	8.30E-09	8.71E-09	8.67E-09	8.78E-09	9.66E-09	1.06E-08	9.18E-09	7.70E-09	8.39E-09	5.35E-09	6.30E-09	6.87E-09	9.26E-09	8.74E-09	6.87E-09
5.89E-09	8.48E-09	6.49E-09	5.10E-09	7.74E-09	7.95E-09	9.09E-09	1.09E-08	1.02E-08	8.57E-09	8.08E-09	8.22E-09	4.30E-09	5.19E-09	6.91E-09	8.48E-09	7.83E-09	5.47E-09
5.04E-09	3.49E-09	6.11E-09	5.40E-09	6.02E-09	7.77E-09	8.91E-09	1.09E-08	9.05E-09	8.20E-09	7.63E-09	8.03E-09	3.82E-09	5.13E-09	6.69E-09	8.22E-09	7.14E-09	4.26E-09
4.08E-09	4.49E-09	4.33E-09	5.51E-09	5.60E-09	7.66E-09	7.28E-09	8.74E-09	8.01E-09	8.01E-09	6.39E-09	6.18E-09	3.09E-09	4.37E-09	6.50E-09	8.06E-09	6.11E-09	4.00E-09
4.14E-09	4.98E-09	3.49E-09	5.05E-09	4.91E-09	6.13E-09	6.43E-09	7.74E-09	7.20E-09	7.63E-09	5.05E-09	5.98E-09	2.71E-09	2.54E-09	5.71E-09	7.63E-09	5.56E-09	2.65E-09
4.57E-09	5.44E-09	2.10E-09	4.04E-09	4.18E-09	4.14E-09	5.97E-09	7.10E-09	6.57E-09	7.35E-09	4.20E-09	6.30E-09	2.43E-09	1.33E-09	4.00E-09	4.99E-09	4.97E-09	1.67E-09
3.65E-09	1.37E-09	7.25E-10	2.25E-09	2.54E-09	3.80E-09	5.10E-09	5.34E-09	6.40E-09	6.61E-09	2.65E-09	5.59E-09	1.58E-09	7.45E-10	3.20E-09	2.33E-09	4.17E-09	1.55E-09
2.43E-09	2.92E-09	2.58E-10	6.24E-10	2.19E-09	4.28E-09	3.91E-09	3.86E-09	5.70E-09	5.54E-09	1.33E-09	2.11E-09	1.39E-09	1.92E-10	3.94E-09	2.39E-09	4.69E-09	1.99E-09
2.32E-09	3.50E-09	1.33E-10	5.88E-10	2.28E-09	2.68E-09	3.21E-09	3.83E-09	4.73E-09	4.63E-09	4.38E-10	-4.48E-10	2.00E-09	-8.70E-10	3.23E-09	3.19E-09	3.87E-09	1.31E-09
1.39E-09	5.29E-09	-5.20E-10	3.08E-10	1.48E-09	6.60E-10	2.44E-09	4.81E-09	5.23E-09	5.02E-09	-2.98E-10	-1.95E-09	-3.09E-10	-2.03E-09	1.57E-09	1.90E-09	-6.74E-10	-9.58E-10
-3.05E-10	2.33E-09	3.18E-10	6.34E-10	2.19E-10	8.03E-10	2.21E-09	5.76E-09	6.74E-09	5.60E-09	-1.30E-09	-2.48E-09	-6.78E-10	-2.41E-09	1.83E-09	-3.57E-10	-1.36E-09	-9.62E-10
-1.54E-09	5.25E-09	4.08E-10	-2.85E-10	-1.34E-09	-1.73E-10	1.80E-09	5.81E-09	7.23E-09	4.64E-09	-2.63E-09	-2.61E-09	-1.91E-09	-2.51E-09	2.03E-09	-1.08E-09	-1.54E-09	5.43E-10
-2.13E-09	3.08E-09	7.82E-10	-1.19E-09	-2.31E-09	-3.54E-10	1.61E-09	4.30E-09	4.59E-09	1.25E-09	-3.90E-09	-2.13E-09	-2.58E-09	-2.66E-09	1.15E-09	-1.21E-09	-6.59E-10	1.49E-09
-2.73E-09	1.03E-09	-3.62E-10	-3.19E-09	-3.77E-09	-1.61E-10	2.10E-09	2.56E-09	1.83E-09	-1.01E-09	-4.77E-09	-2.39E-09	-1.93E-09	-2.95E-09	-1.37E-09	-1.33E-09	-4.83E-10	1.42E-09
-3.71E-09	1.08E-09	-2.30E-09	-3.47E-09	-3.62E-09	-3.78E-10	1.70E-09	2.75E-09	1.17E-09	-1.87E-09	-5.54E-09	-4.76E-09	-1.84E-09	-3.82E-09	-3.53E-09	-2.87E-09	-1.13E-09	5.67E-10
-4.77E-09	2.46E-09	-2.67E-09	-4.29E-09	-3.19E-09	-2.42E-09	4.65E-10	3.51E-09	2.13E-09	-1.95E-09	-5.52E-09	-4.76E-09	-3.09E-09	-5.04E-09	-5.22E-09	-3.71E-09	-2.68E-09	-3.29E-10
-5.38E-09	2.99E-09	-3.64E-09	-5.17E-09	-2.80E-09	-3.19E-09	1.11E-09	4.47E-09	3.13E-09	-1.44E-09	-4.95E-09	-4.42E-09	-3.74E-09	-4.74E-09	-5.94E-09	-3.74E-09	-3.38E-09	-2.47E-09
-6.76E-09	-1.18E-09	-4.97E-09	-5.79E-09	-3.23E-09	-1.66E-09	3.30E-09	7.38E-09	5.32E-09	-4.50E-10	-5.30E-09	-5.62E-09	-3.78E-09	-6.14E-09	-6.15E-09	-5.62E-09	-5.22E-09	-3.78E-09
-8.81E-09	-1.10E-09	-6.83E-09	-5.02E-09	-2.72E-09	1.88E-09	8.27E-09	1.31E-08	9.24E-09	2.82E-09	-5.35E-09	-6.60E-09	-4.34E-09	-7.83E-09	-7.05E-09	-6.33E-09	-6.51E-09	-5.76E-09
-9.17E-09	-3.67E-09	-6.41E-09	-4.06E-09	8.19E-10	7.72E-09	1.70E-08	2.14E-08	1.64E-08	8.59E-09	-3.74E-09	-7.94E-09	-6.08E-09	-7.99E-09	-7.20E-09	-6.85E-09	-7.39E-09	-8.54E-09
-9.02E-09	-3.48E-09	-6.80E-09	-3.70E-09	6.62E-09	1.69E-08	2.71E-08	2.94E-08	2.40E-08	1.54E-08	-2.44E-10	-8.05E-09	-8.01E-09	-8.41E-09	-6.37E-09	-7.30E-09	-7.60E-09	-9.03E-09
-9.30E-09	-5.28E-09	-9.15E-09	-1.90E-09	1.20E-08	2.45E-08	3.30E-08	3.30E-08	2.77E-08	1.95E-08	3.55E-09	-7.14E-09	-8.66E-09	-8.75E-09	-6.08E-09	-9.17E-09	-9.37E-09	-9.12E-09
-9.85E-09	-8.12E-09	-9.68E-09	-1.29E-09	1.21E-08	2.44E-08	3.30E-08	3.10E-08	2.60E-08	1.84E-08	4.85E-09	-5.96E-09	-8.44E-09	-9.94E-09	-8.81E-09	-9.36E-09	-1.03E-08	-8.97E-09
-1.03E-08	-7.59E-09	-1.00E-08	-2.56E-09	7.17E-09	1.75E-08	2.64E-08	2.44E-08	2.18E-08	1.41E-08	2.95E-09	-4.47E-09	-8.96E-09	-1.11E-08	-1.06E-08	-1.09E-08	-1.10E-08	-9.23E-09
-1.10E-08	-7.17E-09	-1.10E-08	-6.38E-09	-5.96E-10	7.64E-09	1.53E-08	1.31E-08	1.44E-08	7.50E-09	-9.77E-10	-4.89E-09	-1.03E-08	-1.07E-08	-9.15E-09	-1.08E-08	-1.09E-08	-9.15E-09
-1.21E-08	-1.18E-08	-1.27E-08	-9.90E-09	-7.45E-09	-1.63E-09	2.08E-09	9.40E-10	4.44E-09	-1.01E-09	-5.17E-09	-7.91E-09	-1.15E-08	-1.13E-08	-9.36E-09	-1.08E-08	-9.19E-09	-8.78E-09
-1.25E-08	-1.24E-08	-1.19E-08	-1.14E-08	-1.27E-08	-7.86E-09	-8.62E-09	-8.85E-09	-5.80E-09	-8.50E-09	-6.85E-09	-8.03E-09	-1.18E-08	-1.21E-08	-1.05E-08	-1.03E-08	-8.31E-09	-9.39E-09
-1.25E-08	-1.39E-08	-1.03E-08	-1.05E-08	-1.38E-08	-1.35E-08	-1.62E-08	-1.68E-08	-1.49E-08	-1.18E-08	-4.06E-09	-6.22E-09	-1.28E-08	-1.39E-08	-1.25E-08	-1.17E-08	-8.80E-09	-9.32E-09
-1.28E-08	-1.27E-08	-9.86E-09	-1.05E-08	-1.40E-08	-1.45E-08	-1.82E-08	-2.08E-08	-1.81E-08	-1.20E-08	4.14E-10	-3.18E-09	-1.55E-08	-1.62E-08	-1.25E-08	-1.21E-08	-1.02E-08	-9.78E-09
-1.38E-08	-1.34E-08	-1.01E-08	-1.02E-08	-1.40E-08	-1.50E-08	-1.76E-08	-2.18E-08	-1.90E-08	-1.33E-08	-3.76E-10	-1.89E-09	-1.66E-08	-1.64E-08	-1.23E-08	-1.17E-08	-1.05E-08	-9.96E-09
-1.41E-08	-1.31E-08	-8.15E-09	-8.62E-09	-1.35E-08	-1.57E-08	-1.71E-08	-2.19E-08	-1.91E-08	-1.65E-08	-5.83E-09	-1.03E-08	-1.65E-08	-1.64E-08	-1.10E-08	-1.16E-08	-1.20E-08	-1.05E-08
-1.47E-08	-9.68E-09	-5.69E-09	-7.37E-09	-1.52E-08	-1.57E-08	-1.87E-08	-2.34E-08	-1.98E-08	-1.85E-08	-7.83E-09	-1.11E-08	-1.56E-08	-1.58E-08	-1.26E-08	-1.23E-08	-1.32E-08	-1.29E-08
-1.62E-08	-7.18E-09	-2.74E-09	-6.58E-09	-1.61E-08	-1.61E-08	-2.00E-08	-2.43E-08	-2.09E-08	-1.75E-08	-1.57E-09	-8.98E-09	-1.59E-08	-1.50E-08	-1.39E-08	-1.39E-08	-1.34E-08	-1.47E-08
-1.82E-08	-9.99E-09	2.08E-09	-4.95E-09	-1.66E-08	-1.72E-08	-2.06E-08	-2.51E-08	-2.29E-08	-1.78E-08	-3.30E-10	-9.14E-09	-1.71E-08	-1.47E-08	-1.59E-08	-1.50E-08	-1.40E-08	-1.56E-08
-1.74E-08	-6.19E-09	3.13E-09	-1.01E-08	-1.71E-08	-1.87E-08	-2.09E-08	-2.58E-08	-2.32E-08	-2.08E-08	-4.92E-09	-1.41E-08	-1.79E-08	-1.52E-08	-1.86E-08	-1.59E-08	-1.60E-08	-1.58E-08
-1.69E-08	-6.60E-09	-4.34E-09	-1.51E-08	-1.78E-08	-1.93E-08	-2.19E-08	-2.62E-08	-2.26E-08	-2.26E-08	-1.83E-08	-1.78E-08	-1.87E-08	-1.88E-08	-1.93E-08	-1.71E-08	-1.79E-08	-1.63E-08
-1.66E-08	-1.61E-08	-2.08E-08	-1.90E-08	-1.96E-08	-2.04E-08	-2.31E-08	-2.74E-08	-2.28E-08	-2.29E-08	-2.15E-08	-1.89E-08	-1.93E-08	-1.98E-08	-2.11E-08	-1.85E-08	-1.86E-08	-1.66E-08
-1.67E-08	-1.34E-08	-2.12E-08	-2.02E-08	-2.08E-08	-2.11E-08	-2.44E-08	-2.84E-08	-2.40E-08	-2.30E-08	-2.14E-08	-1.92E-08	-2.06E-08	-2.03E-08	-2.21E-08	-2.07E-08	-1.87E-08	-1.81E-08
-1.69E-08	-1.60E-08	-2.10E-08	-2.07E-08	-2.08E-08	-2.14E-08	-2.44E-08	-2.87E-08	-2.52E-08	-2.39E-08	-2.29E-08	-2.04E-08	-2.17E-08	-1.98E-08	-2.17E-08	-2.11E-08	-1.85E-08	-1.78E-08
-1.81E-08	-2.21E-08	-2.16E-08	-2.10E-08	-2.06E-08	-2.13E-08	-2.44E-08	-2.94E-08	-2.54E-08	-2.50E-08	-2.48E-08	-2.13E-08	-2.21E-08	-2.16E-08	-1.98E-08	-2.07E-08	-1.76E-08	-1.77E-08
-2.03E-08	-2.15E-08	-2.27E-08	-2.16E-08	-2.29E-08	-2.23E-08	-2.49E-08	-2.97E-08	-2.49E-08	-2.56E-08	-2.56E-08	-2.18E-08	-2.24E-08	-2.17E-08	-1.77E-08	-2.01E-08	-1.89E-08	-1.80E-08
-2.15E-08	-2.43E-08	-2.26E-08	-2.15E-08	-2.44E-08	-2.45E-08	-2.59E-08	-2.87E-08	-2.48E-08	-2.54E-08	-2.56E-08	-2.23E-08	-2.25E-08	-2.01E-08	-2.24E-08	-2.18E-08	-1.98E-08	-1.82E-08
-2.28E-08	-2.26E-08	-2.29E-08	-2.19E-08	-2.53E-08	-2.41E-08	-2.57E-08	-2.78E-08	-2.45E-08	-2.55E-08	-2.57E-08	-2.41E-08	-2.32E-08	-1.90E-08	-2.32E-08	-2.18E-08	-2.17E-08	-1.95E-08
-2.32E-08	-2.23E-08	-2.47E-08	-2.32E-08	-2.68E-08	-2.54E-08	-2.52E-08	-2.91E-08	-2.48E-08	-2.65E-08	-2.65E-08	-2.42E-08	-2.39E-08	-2.18E-08	-2.48E-08	-2.26E-08	-2.34E-08	-2.33E-08
-2.34E-08	-2.31E-08	-2.54E-08	-2.44E-08	-2.65E-08	-2.71E-08	-2.51E-08	-3.02E-08	-2.56E-08	-2.70E-08	-2.71E-08	-2.40E-08	-2.50E-08	-2.46E-08	-2.71E-08	-2.45E-08	-2.52E-08	-2.46E-08
-2.40E-08	-2.31E-08	-2.58E-08	-2.45E-08	-2.67E-08	-2.85E-08	-2.65E-08	-3.09E-08	-2.59E-08	-2.71E-08	-2.70E-08	-2.48E-08	-2.53E-08	-2.51E-08	-2.93E-08	-2.58E-08	-2.64E-08	-2.58E-08
-2.39E-08	-2.18E-08	-2.58E-08	-2.47E-08	-2.77E-08	-2.84E-08	-2.83E-08	-3.09E-08	-2.59E-08	-2.74E-08	-2.71E-08	-2.44E-08	-2.64E-08	-2.54E-08	-3.01E-08	-2.55E-08	-2.61E-08	-2.70E-08
-2.35E-08	-2.24E-08	-2.62E-08	-2.41E-08	-2.78E-08	-2.74E-08	-2.96E-08	-3.06E-08	-2.64E-08	-2.78E-08	-2.83E-08	-2.60E-08	-2.68E-08	-2.72E-08	-3.01E-08	-2.52E-08	-2.60E-08	-2.79E-08
-2.37E-08	-2.69E-08	-2.66E-08	-2.56E-08	-2.89E-08	-2.83E-08	-3.02E-08	-3.04E-08	-2.75E-08	-2.88E-08	-2.92E-08	-2.77E-08	-2.73E-08	-2.68E-08	-2.91E-08	-2.55E-08	-2.66E-08	-2.81E-08
-2.56E-08	-2.62E-08	-2.73E-08	-2.75E-08	-3.06E-08	-3.05E-08	-3.09E-08	-3.28E-08	-2.80E-08	-3.01E-08	-3.01E-08	-2.96E-08	-2.76E-08	-2.63E-08	-2.76E-08	-2.75E-08	-2.81E-08	-2.77E-08
-2.67E-08	-2.33E-08	-2.81E-08	-2.82E-08	-3.08E-08	-3.01E-08	-3.06E-08	-3.34E-08	-2.72E-08	-3.11E-08	-3.06E-08	-3.00E-08	-2.89E-08	-2.78E-08	-2.76E-08	-2.86E-08	-2.91E-08	-2.79E-08
-2.71E-08	-2.15E-08	-2.86E-08	-2.80E-08	-3.12E-08	-2.94E-08	-3.07E-08	-3.40E-08	-2.80E-08	-3.17E-08	-3.13E-08	-2.94E-08	-2.90E-08	-2.91E-08	-2.95E-08	-2.81E-08	-3.05E-08	-2.92E-08
-2.76E-08	-2.43E-08	-2.92E-08	-2.81E-08	-3.03E-08	-3.03E-08	-3.18E-08	-3.31E-08	-2.83E-08	-3.24E-08	-3.10E-08	-2.92E-08	-2.97E-08	-2.95E-08	-2.98E-08	-2.76E-08	-3.10E-08	-2.94E-08
-2.84E-08	-2.70E-08	-3.02E-08	-2.80E-08	-3.03E-08	-3.14E-08	-3.38E-08	-3.34E-08	-2.88E-08	-3.28E-08	-3.07E-08	-3.03E-08	-3.01E-08	-3.01E-08	-3.01E-08	-2.93E-08	-3.16E-08	-3.02E-08
-2.93E-08	-2.79E-08	-3.05E-08	-2.90E-08	-3.08E-08	-3.11E-08	-3.42E-08	-3.28E-08	-2.98E-08	-3.33E-08	-3.15E-08	-3.20E-08	-3.07E-08	-2.97E-08	-2.90E-08	-3.00E-08	-3.17E-08	-3.10E-08
-3.02E-08	-3.13E-08	-3.10E-08	-2.90E-08	-3.08E-08	-3.06E-08	-3.44E-08	-3.45E-08	-3.05E-08	-3.40E-08	-3.21E-08	-3.29E-08	-3.21E-08	-2.93E-08	-2.89E-08	-2.94E-08	-3.22E-08	-3.28E-08
-3.07E-08	-2.89E-08	-3.13E-08	-2.87E-08	-3.09E-08	-3.06E-08	-3.42E-08	-3.37E-08	-3.22E-08	-3.43E-08	-3.05E-08	-3.31E-08	-3.19E-08	-2.84E-08	-2.78E-08	-2.98E-08	-3.34E-08	-3.35E-08
-3.13E-08	-2.78E-08	-3.13E-08	-2.94E-08	-3.23E-08	-3.20E-08	-3.48E-08	-3.71E-08	-3.30E-08	-3.40E-08	-3.04E-08	-3.21E-08	-3.25E-08	-2.67E-08	-2.55E-08	-3.14E-08	-3.28E-08	-3.44E-08
-3.11E-08	-3.00E-08	-3.16E-08	-3.01E-08	-3.33E-08	-3.18E-08	-3.55E-08	-3.75E-08	-3.38E-08	-3.34E-08	-3.15E-08	-3.14E-08	-3.31E-08	-2.59E-08	-2.39E-08	-3.22E-08	-3.21E-08	-3.34E-08
-3.00E-08	-3.25E-08	-3.14E-08	-3.15E-08	-3.33E-08	-3.11E-08	-3.54E-08	-3.81E-08	-3.49E-08	-3.43E-08	-3.19E-08	-3.21E-08	-3.28E-08	-3.52E-08	-3.00E-08	-3.27E-08	-3.07E-08	-3.21E-08
-2.98E-08	-3.21E-08	-3.28E-08	-3.21E-08	-3.32E-08	-3.00E-08	-3.54E-08	-3.96E-08	-3.60E-08	-3.55E-08	-3.16E-08	-3.20E-08	-3.27E-08	-3.57E-08	-3.36E-08	-3.20E-08	-3.10E-08	-3.17E-08

-3.00E-08	-3.01E-08	-3.36E-08	-3.29E-08	-3.38E-08	-2.98E-08	-3.47E-08	-3.99E-08	-3.62E-08	-3.47E-08	-3.19E-08	-3.16E-08	-3.27E-08	-3.47E-08	-3.33E-08	-3.16E-08	-3.17E-08	-3.29E-08
-2.97E-08	-3.28E-08	-3.34E-08	-3.33E-08	-3.46E-08	-2.94E-08	-3.50E-08	-3.88E-08	-3.67E-08	-3.42E-08	-3.36E-08	-3.15E-08	-3.34E-08	-3.27E-08	-3.40E-08	-3.29E-08	-3.18E-08	-3.22E-08
-2.93E-08	-3.45E-08	-3.37E-08	-3.36E-08	-3.47E-08	-2.84E-08	-3.60E-08	-3.78E-08	-3.70E-08	-3.64E-08	-3.46E-08	-3.27E-08	-3.47E-08	-3.20E-08	-3.62E-08	-3.43E-08	-3.10E-08	-3.11E-08
-3.08E-08	-3.35E-08	-3.36E-08	-3.32E-08	-3.44E-08	-2.95E-08	-3.63E-08	-3.75E-08	-3.69E-08	-3.79E-08	-3.58E-08	-3.36E-08	-3.52E-08	-3.20E-08	-3.60E-08	-3.38E-08	-3.23E-08	-3.12E-08
-3.11E-08	-3.24E-08	-3.32E-08	-3.27E-08	-3.56E-08	-3.55E-08	-3.61E-08	-3.88E-08	-3.69E-08	-3.81E-08	-3.64E-08	-3.35E-08	-3.51E-08	-3.47E-08	-3.63E-08	-3.34E-08	-3.42E-08	-3.22E-08
-3.09E-08	-2.96E-08	-3.25E-08	-3.24E-08	-3.58E-08	-3.65E-08	-3.78E-08	-3.81E-08	-3.66E-08	-3.81E-08	-3.69E-08	-3.54E-08	-3.60E-08	-3.47E-08	-3.63E-08	-3.44E-08	-3.42E-08	-3.28E-08
-3.22E-08	-2.91E-08	-3.37E-08	-3.20E-08	-3.52E-08	-3.67E-08	-3.86E-08	-3.63E-08	-3.70E-08	-3.93E-08	-3.65E-08	-3.60E-08	-3.70E-08	-3.56E-08	-3.63E-08	-3.54E-08	-3.49E-08	-3.33E-08
-3.38E-08	-3.14E-08	-3.34E-08	-3.02E-08	-3.42E-08	-3.55E-08	-3.92E-08	-3.48E-08	-3.67E-08	-4.07E-08	-3.73E-08	-3.52E-08	-3.81E-08	-3.67E-08	-3.75E-08	-3.63E-08	-3.58E-08	-3.38E-08
-3.36E-08	-3.01E-08	-3.25E-08	-3.12E-08	-3.33E-08	-3.57E-08	-3.87E-08	-3.37E-08	-3.66E-08	-4.13E-08	-3.86E-08	-3.49E-08	-3.81E-08	-3.75E-08	-3.97E-08	-3.85E-08	-3.70E-08	-3.52E-08
-3.18E-08	-2.53E-08	-3.08E-08	-3.08E-08	-3.35E-08	-3.73E-08	-3.89E-08	-3.51E-08	-3.66E-08	-4.09E-08	-3.94E-08	-3.55E-08	-3.84E-08	-3.71E-08	-3.98E-08	-3.99E-08	-3.79E-08	-3.72E-08

Quadrant 2 Data Squared (16384 values):

2.50E-15	1.38E-15	1.58E-15	1.63E-15	1.51E-15	1.39E-15	1.70E-15	1.51E-15	1.79E-15	2.66E-15	2.89E-15	2.53E-15	2.99E-15	2.70E-15	1.91E-15	1.84E-15	1.87E-15	1.92E-15
1.90E-15	1.15E-15	1.47E-15	1.43E-15	1.51E-15	1.32E-15	1.68E-15	1.49E-15	1.73E-15	2.66E-15	3.16E-15	2.74E-15	3.07E-15	2.93E-15	1.87E-15	1.77E-15	1.88E-15	1.81E-15
1.70E-15	8.96E-16	1.31E-15	1.27E-15	1.47E-15	1.24E-15	1.30E-15	1.29E-15	1.70E-15	2.56E-15	3.20E-15	2.72E-15	2.92E-15	2.88E-15	1.88E-15	1.73E-15	1.81E-15	1.75E-15
1.55E-15	8.63E-16	1.25E-15	1.21E-15	1.50E-15	1.32E-15	1.17E-15	1.20E-15	1.60E-15	2.37E-15	2.92E-15	2.57E-15	2.51E-15	2.54E-15	1.93E-15	1.74E-15	1.72E-15	1.63E-15
1.43E-15	8.09E-16	1.22E-15	1.10E-15	1.53E-15	1.32E-15	1.11E-15	1.23E-15	1.53E-15	2.08E-15	2.31E-15	2.40E-15	2.18E-15	2.39E-15	2.00E-15	1.88E-15	1.60E-15	1.56E-15
1.32E-15	7.57E-16	1.15E-15	1.02E-15	1.33E-15	1.14E-15	1.08E-15	1.23E-15	1.45E-15	1.84E-15	1.87E-15	2.23E-15	1.98E-15	2.25E-15	2.08E-15	2.03E-15	1.48E-15	1.46E-15
1.14E-15	7.18E-16	1.10E-15	1.01E-15	1.13E-15	1.02E-15	1.06E-15	1.15E-15	1.36E-15	1.61E-15	1.69E-15	1.88E-15	1.84E-15	2.09E-15	2.22E-15	1.90E-15	1.49E-15	1.44E-15
1.02E-15	6.92E-16	1.08E-15	1.01E-15	1.07E-15	1.03E-15	1.01E-15	1.04E-15	1.27E-15	1.42E-15	1.49E-15	1.65E-15	1.71E-15	1.84E-15	2.22E-15	1.88E-15	1.54E-15	1.40E-15
9.67E-16	6.87E-16	1.09E-15	9.87E-16	1.06E-15	9.95E-16	9.24E-16	9.88E-16	1.18E-15	1.31E-15	1.31E-15	1.48E-15	1.61E-15	1.65E-15	2.14E-15	2.02E-15	1.63E-15	1.45E-15
9.62E-16	6.93E-16	1.09E-15	9.51E-16	1.07E-15	9.37E-16	8.71E-16	9.63E-16	1.11E-15	1.20E-15	1.26E-15	1.41E-15	1.53E-15	1.58E-15	2.07E-15	2.11E-15	1.83E-15	1.55E-15
9.56E-16	7.18E-16	1.08E-15	9.58E-16	1.03E-15	8.99E-16	8.59E-16	8.85E-16	1.01E-15	1.02E-15	1.33E-15	1.44E-15	1.43E-15	1.46E-15	1.82E-15	1.94E-15	1.94E-15	1.63E-15
8.98E-16	7.08E-16	9.98E-16	9.96E-16	9.54E-16	8.88E-16	8.40E-16	7.90E-16	9.26E-16	8.98E-16	1.47E-15	1.60E-15	1.41E-15	1.32E-15	1.44E-15	1.60E-15	1.86E-15	1.60E-15
8.24E-16	6.56E-16	8.88E-16	9.14E-16	9.01E-16	8.94E-16	8.12E-16	7.58E-16	8.54E-16	8.41E-16	1.59E-15	1.67E-15	1.33E-15	1.08E-15	1.20E-15	1.33E-15	1.54E-15	1.60E-15
8.12E-16	6.27E-16	8.31E-16	7.71E-16	8.92E-16	8.70E-16	7.85E-16	7.67E-16	7.54E-16	8.34E-16	1.70E-15	1.72E-15	1.14E-15	8.86E-16	1.07E-15	1.15E-15	1.23E-15	1.66E-15
8.21E-16	6.02E-16	7.79E-16	7.25E-16	8.55E-16	8.25E-16	7.69E-16	7.73E-16	6.66E-16	7.98E-16	1.55E-15	1.60E-15	1.07E-15	8.26E-16	9.71E-16	1.02E-15	1.14E-15	1.66E-15
7.89E-16	5.60E-16	7.38E-16	7.36E-16	8.18E-16	8.22E-16	7.64E-16	8.04E-16	6.38E-16	6.99E-16	1.02E-15	1.23E-15	1.05E-15	8.17E-16	9.46E-16	8.55E-16	1.01E-15	1.52E-15
7.60E-16	5.08E-16	7.04E-16	7.47E-16	8.30E-16	8.87E-16	7.50E-16	8.76E-16	6.54E-16	6.56E-16	6.81E-16	8.51E-16	1.01E-15	7.81E-16	8.53E-16	7.62E-16	8.09E-16	1.26E-15
7.88E-16	4.48E-16	6.72E-16	6.82E-16	8.79E-16	9.70E-16	7.27E-16	9.41E-16	6.33E-16	6.67E-16	6.71E-16	7.66E-16	9.59E-16	7.51E-16	7.76E-16	8.04E-16	8.18E-16	1.18E-15
8.48E-16	3.88E-16	6.39E-16	6.46E-16	8.71E-16	9.82E-16	7.01E-16	9.12E-16	5.35E-16	6.82E-16	6.76E-16	7.41E-16	9.45E-16	7.36E-16	7.57E-16	8.05E-16	9.28E-16	1.33E-15
8.97E-16	3.64E-16	6.47E-16	6.08E-16	7.48E-16	8.97E-16	7.22E-16	8.65E-16	5.42E-16	6.92E-16	6.63E-16	7.23E-16	9.79E-16	7.54E-16	7.15E-16	7.34E-16	8.81E-16	1.36E-15
8.88E-16	4.60E-16	6.93E-16	6.08E-16	5.90E-16	6.97E-16	6.63E-16	6.50E-16	5.96E-16	7.09E-16	6.19E-16	6.42E-16	9.52E-16	7.48E-16	7.16E-16	7.68E-16	6.69E-16	8.89E-16
9.36E-16	6.16E-16	7.01E-16	5.72E-16	5.63E-16	6.30E-16	6.81E-16	5.84E-16	5.95E-16	7.30E-16	5.91E-16	5.70E-16	9.03E-16	7.61E-16	7.76E-16	9.77E-16	6.29E-16	5.84E-16
9.61E-16	7.73E-16	7.01E-16	5.40E-16	5.52E-16	5.43E-16	7.09E-16	6.24E-16	5.29E-16	7.93E-16	5.94E-16	5.78E-16	8.44E-16	8.31E-16	8.21E-16	1.34E-15	7.70E-16	5.35E-16
8.88E-16	8.68E-16	7.00E-16	5.47E-16	5.46E-16	4.76E-16	6.27E-16	6.78E-16	5.18E-16	8.33E-16	4.75E-16	5.62E-16	8.46E-16	8.66E-16	8.23E-16	1.51E-15	8.73E-16	5.48E-16
8.35E-16	1.03E-15	8.20E-16	5.96E-16	5.59E-16	4.66E-16	6.44E-16	7.57E-16	5.52E-16	8.94E-16	4.05E-16	5.65E-16	7.71E-16	6.93E-16	6.37E-16	1.14E-15	7.41E-16	6.34E-16
7.93E-16	1.20E-15	8.61E-16	6.70E-16	5.63E-16	4.83E-16	8.30E-16	8.99E-16	5.59E-16	8.60E-16	3.84E-16	5.70E-16	6.87E-16	5.83E-16	4.55E-16	7.06E-16	4.06E-16	6.80E-16
8.19E-16	8.24E-16	8.41E-16	7.83E-16	6.16E-16	4.99E-16	1.16E-15	1.17E-15	4.35E-16	5.74E-16	3.87E-16	5.47E-16	6.33E-16	6.08E-16	4.17E-16	5.97E-16	4.35E-16	7.04E-16
7.56E-16	7.19E-16	7.74E-16	8.56E-16	7.10E-16	5.18E-16	1.40E-15	1.33E-15	3.95E-16	4.79E-16	4.12E-16	4.48E-16	5.64E-16	6.21E-16	4.29E-16	6.41E-16	4.75E-16	7.74E-16
6.56E-16	7.93E-16	7.42E-16	9.12E-16	7.99E-16	5.67E-16	9.04E-16	8.17E-16	3.60E-16	4.68E-16	4.32E-16	3.88E-16	5.44E-16	5.60E-16	4.37E-16	6.50E-16	5.34E-16	1.01E-15
6.38E-16	9.92E-16	7.04E-16	9.43E-16	7.77E-16	6.39E-16	4.99E-16	4.71E-16	3.59E-16	4.74E-16	4.14E-16	3.41E-16	5.42E-16	4.99E-16	4.40E-16	7.11E-16	6.83E-16	1.21E-15
6.95E-16	9.69E-16	7.12E-16	9.81E-16	7.64E-16	6.66E-16	4.90E-16	4.70E-16	3.58E-16	4.69E-16	3.61E-16	3.02E-16	5.22E-16	5.19E-16	4.16E-16	7.29E-16	6.58E-16	1.14E-15
7.58E-16	1.29E-15	7.79E-16	9.83E-16	6.53E-16	5.60E-16	5.54E-16	4.73E-16	3.37E-16	4.73E-16	3.58E-16	3.00E-16	4.89E-16	4.96E-16	3.33E-16	5.99E-16	6.18E-16	8.53E-16
7.44E-16	1.12E-15	7.28E-16	7.28E-16	5.94E-16	5.44E-16	5.63E-16	4.55E-16	3.56E-16	4.48E-16	3.52E-16	3.05E-16	4.33E-16	4.23E-16	2.98E-16	4.49E-16	5.34E-16	6.09E-16
6.37E-16	7.47E-16	5.98E-16	6.32E-16	5.66E-16	5.38E-16	4.96E-16	4.67E-16	4.27E-16	4.25E-16	3.55E-16	3.10E-16	3.78E-16	3.63E-16	2.85E-16	3.04E-16	3.66E-16	4.87E-16
5.09E-16	5.83E-16	5.40E-16	5.40E-16	5.45E-16	5.02E-16	4.67E-16	4.91E-16	4.69E-16	3.99E-16	3.87E-16	2.82E-16	3.71E-16	3.22E-16	2.58E-16	2.45E-16	2.63E-16	4.32E-16
4.42E-16	4.04E-16	5.50E-16	5.03E-16	5.53E-16	4.53E-16	4.71E-16	4.89E-16	4.50E-16	3.89E-16	4.37E-16	3.09E-16	3.70E-16	3.20E-16	2.37E-16	2.42E-16	2.51E-16	3.12E-16
4.24E-16	3.70E-16	5.57E-16	5.65E-16	5.61E-16	4.62E-16	4.75E-16	4.66E-16	4.46E-16	4.07E-16	4.64E-16	3.63E-16	3.77E-16	3.31E-16	2.59E-16	2.58E-16	2.93E-16	2.03E-16
3.89E-16	4.07E-16	5.51E-16	6.64E-16	5.74E-16	4.38E-16	4.42E-16	5.03E-16	4.95E-16	4.17E-16	4.52E-16	4.24E-16	3.86E-16	3.50E-16	3.12E-16	2.81E-16	3.47E-16	1.60E-16
3.76E-16	3.65E-16	5.33E-16	6.29E-16	5.58E-16	3.73E-16	4.17E-16	5.80E-16	5.39E-16	3.87E-16	4.07E-16	4.65E-16	4.30E-16	4.27E-16	4.13E-16	3.07E-16	4.30E-16	1.82E-16
3.41E-16	3.26E-16	5.09E-16	4.22E-16	4.16E-16	3.14E-16	3.67E-16	5.70E-16	4.83E-16	3.52E-16	3.79E-16	4.57E-16	4.15E-16	5.04E-16	5.15E-16	3.64E-16	5.42E-16	2.39E-16
3.16E-16	2.76E-16	3.68E-16	2.64E-16	3.05E-16	3.07E-16	2.96E-16	4.71E-16	3.73E-16	3.14E-16	3.40E-16	3.92E-16	3.62E-16	5.53E-16	5.52E-16	3.65E-16	5.38E-16	3.02E-16
2.90E-16	2.57E-16	3.40E-16	2.17E-16	2.90E-16	3.49E-16	2.69E-16	4.16E-16	3.40E-16	2.87E-16	3.26E-16	2.88E-16	3.33E-16	6.36E-16	6.57E-16	3.36E-16	4.81E-16	3.26E-16
2.60E-16	2.81E-16	3.22E-16	2.22E-16	2.99E-16	3.08E-16	2.39E-16	4.04E-16	3.55E-16	2.76E-16	2.82E-16	2.76E-16	3.37E-16	9.18E-16	8.88E-16	2.77E-16	4.76E-16	3.00E-16
2.39E-16	3.41E-16	3.24E-16	2.45E-16	2.87E-16	2.54E-16	2.21E-16	3.78E-16	3.22E-16	2.42E-16	2.17E-16	2.69E-16	3.51E-16	1.06E-15	1.10E-15	2.50E-16	4.89E-16	2.59E-16
2.52E-16	4.29E-16	2.75E-16	2.88E-16	3.01E-16	2.29E-16	2.25E-16	3.38E-16	2.71E-16	2.18E-16	1.86E-16	2.87E-16	3.54E-16	8.46E-16	6.45E-16	2.63E-16	4.48E-16	2.49E-16
2.36E-16	4.47E-16	2.29E-16	2.64E-16	3.14E-16	2.57E-16	2.54E-16	3.15E-16	2.38E-16	1.98E-16	1.91E-16	3.00E-16	2.83E-16	3.41E-16	2.00E-16	2.75E-16	3.84E-16	2.36E-16
1.78E-16	1.75E-16	2.24E-16	2.30E-16	2.33E-16	2.61E-16	2.83E-16	3.18E-16	2.26E-16	1.86E-16	1.89E-16	2.52E-16	2.25E-16	3.02E-16	1.64E-16	2.72E-16	3.07E-16	2.36E-16
1.30E-16	1.56E-16	2.35E-16	1.89E-16	2.16E-16	2.55E-16	2.85E-16	2.87E-16	2.23E-16	1.72E-16	1.68E-16	2.22E-16	1.59E-16	1.88E-16	1.45E-16	2.11E-16	2.47E-16	2.15E-16

1.22E-16	1.98E-16	1.93E-16	1.92E-16	2.28E-16	1.84E-16	2.61E-16	2.31E-16	1.91E-16	1.56E-16	1.49E-16	2.15E-16	1.31E-16	1.43E-16	1.49E-16	1.62E-16	2.01E-16	1.66E-16
1.14E-16	2.52E-16	1.93E-16	2.00E-16	2.35E-16	1.72E-16	2.38E-16	2.32E-16	1.78E-16	1.41E-16	1.24E-16	1.63E-16	1.19E-16	1.20E-16	1.28E-16	1.33E-16	1.62E-16	1.45E-16
1.19E-16	3.18E-16	1.89E-16	1.75E-16	2.18E-16	1.65E-16	2.00E-16	2.54E-16	1.94E-16	1.55E-16	9.07E-17	9.35E-17	1.07E-16	8.46E-17	1.04E-16	1.19E-16	1.73E-16	1.38E-16
1.07E-16	1.40E-16	1.55E-16	1.54E-16	1.86E-16	1.60E-16	1.96E-16	2.27E-16	2.12E-16	2.25E-16	8.86E-17	8.54E-17	1.08E-16	7.45E-17	8.73E-17	1.08E-16	1.63E-16	1.40E-16
8.62E-17	1.23E-16	1.36E-16	1.31E-16	1.55E-16	1.64E-16	2.17E-16	1.99E-16	2.10E-16	2.99E-16	9.26E-17	7.40E-17	8.78E-17	8.33E-17	9.78E-17	1.05E-16	1.55E-16	1.32E-16
9.02E-17	2.04E-16	1.17E-16	1.18E-16	1.46E-16	1.76E-16	2.51E-16	1.95E-16	1.86E-16	2.45E-16	8.19E-17	5.29E-17	7.11E-17	8.35E-17	1.34E-16	1.06E-16	1.82E-16	1.05E-16
9.39E-17	1.88E-16	8.70E-17	1.23E-16	1.36E-16	1.77E-16	2.50E-16	1.84E-16	1.26E-16	1.28E-16	5.96E-17	4.25E-17	6.28E-17	7.49E-17	1.48E-16	1.11E-16	2.11E-16	8.65E-17
7.05E-17	1.61E-16	8.12E-17	1.17E-16	1.15E-16	1.85E-16	2.35E-16	1.27E-16	9.63E-17	9.64E-17	6.02E-17	4.20E-17	5.35E-17	5.85E-17	1.05E-16	1.10E-16	1.53E-16	8.92E-17
5.43E-17	3.98E-17	6.96E-17	1.03E-16	1.05E-16	1.40E-16	1.38E-16	8.33E-17	1.02E-16	9.40E-17	6.15E-17	5.12E-17	4.41E-17	4.75E-17	6.83E-17	9.62E-17	1.01E-16	7.06E-17
3.91E-17	5.46E-17	5.55E-17	6.88E-17	7.59E-17	7.51E-17	7.72E-17	9.34E-17	1.11E-16	8.43E-17	5.92E-17	7.05E-17	2.86E-17	3.97E-17	4.72E-17	8.57E-17	7.64E-17	4.72E-17
3.47E-17	7.20E-17	4.22E-17	2.60E-17	6.00E-17	6.32E-17	8.27E-17	1.18E-16	1.05E-16	7.34E-17	6.54E-17	6.76E-17	1.85E-17	2.69E-17	4.78E-17	7.19E-17	6.14E-17	2.99E-17
2.54E-17	1.22E-17	3.73E-17	2.91E-17	3.63E-17	6.03E-17	7.94E-17	1.18E-16	8.19E-17	6.72E-17	5.83E-17	6.44E-17	1.46E-17	2.63E-17	4.47E-17	6.75E-17	5.10E-17	1.82E-17
1.67E-17	2.01E-17	1.88E-17	3.03E-17	3.13E-17	5.87E-17	5.30E-17	7.64E-17	6.42E-17	6.42E-17	4.09E-17	3.82E-17	9.57E-18	1.91E-17	4.23E-17	6.49E-17	3.73E-17	1.60E-17
1.71E-17	2.48E-17	1.21E-17	2.55E-17	2.41E-17	3.75E-17	4.13E-17	5.99E-17	5.18E-17	5.82E-17	2.55E-17	3.58E-17	7.32E-18	6.48E-18	3.26E-17	5.82E-17	3.09E-17	7.03E-18
2.09E-17	2.96E-17	4.40E-18	1.63E-17	1.74E-17	1.72E-17	3.56E-17	5.04E-17	4.31E-17	5.40E-17	1.77E-17	3.97E-17	5.91E-18	1.76E-18	1.60E-17	2.49E-17	2.47E-17	2.79E-18
1.33E-17	1.87E-18	5.26E-19	5.07E-18	6.43E-18	1.44E-17	2.60E-17	2.85E-17	4.10E-17	4.37E-17	7.01E-18	3.12E-17	2.50E-18	5.54E-19	1.03E-17	5.43E-18	1.74E-17	2.40E-18
5.92E-18	8.50E-18	6.65E-20	3.89E-19	4.81E-18	1.83E-17	1.53E-17	1.49E-17	3.25E-17	3.07E-17	1.77E-18	4.45E-18	1.92E-18	3.69E-20	1.56E-17	5.72E-18	2.20E-17	3.98E-18
5.38E-18	1.23E-17	1.78E-20	3.46E-19	5.21E-18	7.18E-18	1.03E-17	1.47E-17	2.24E-17	2.14E-17	1.92E-19	2.01E-19	4.01E-18	7.56E-19	1.05E-17	1.02E-17	1.49E-17	1.72E-18
1.94E-18	2.80E-17	2.71E-19	9.47E-20	2.18E-18	4.36E-19	5.97E-18	2.31E-17	2.74E-17	2.52E-17	8.87E-20	3.80E-18	9.52E-20	4.11E-18	2.48E-18	3.59E-18	4.55E-19	9.18E-19
9.28E-20	5.41E-18	1.01E-19	4.02E-19	4.78E-20	6.45E-19	4.87E-18	3.32E-17	4.54E-17	3.13E-17	1.70E-18	6.13E-18	4.59E-19	5.82E-18	3.35E-18	1.28E-19	1.85E-18	9.25E-19
2.36E-18	2.75E-17	1.66E-19	8.12E-20	1.80E-18	2.99E-20	3.23E-18	3.37E-17	5.22E-17	2.16E-17	6.90E-18	6.81E-18	3.65E-18	6.29E-18	4.11E-18	1.17E-18	2.37E-18	2.94E-19
4.55E-18	9.51E-18	6.12E-19	1.41E-18	5.31E-18	1.25E-19	2.60E-18	1.85E-17	2.10E-17	1.57E-18	1.52E-17	4.53E-18	6.64E-18	7.06E-18	1.32E-18	1.45E-18	4.34E-19	2.23E-18
7.48E-18	1.06E-18	1.31E-19	1.02E-17	1.42E-17	2.58E-20	4.42E-18	6.55E-18	3.35E-18	1.01E-18	2.27E-17	5.69E-18	3.74E-18	8.71E-18	1.89E-18	1.76E-18	2.34E-19	2.02E-18
1.38E-17	1.16E-18	5.29E-18	1.20E-17	1.31E-17	1.43E-19	2.90E-18	7.54E-18	1.36E-18	3.50E-18	3.07E-17	2.27E-17	3.37E-18	1.46E-17	1.25E-17	8.25E-18	1.28E-18	3.22E-19
2.28E-17	6.06E-18	7.13E-18	1.84E-17	1.02E-17	5.88E-18	2.16E-19	1.23E-17	4.55E-18	3.81E-18	3.05E-17	2.27E-17	9.57E-18	2.54E-17	2.73E-17	1.38E-17	7.18E-18	1.08E-19
2.89E-17	8.92E-18	1.32E-17	2.67E-17	7.82E-18	1.02E-17	1.22E-18	2.00E-17	9.81E-18	2.06E-18	2.45E-17	1.95E-17	1.40E-17	2.25E-17	3.53E-17	1.40E-17	1.14E-17	6.10E-18
4.56E-17	1.39E-18	2.47E-17	3.35E-17	1.04E-17	2.74E-18	1.09E-17	5.45E-17	2.83E-17	2.02E-19	2.81E-17	3.15E-17	1.43E-17	3.77E-17	3.78E-17	3.16E-17	2.73E-17	1.43E-17
7.76E-17	1.22E-18	4.67E-17	2.52E-17	7.42E-18	3.54E-18	6.84E-17	1.71E-16	8.54E-17	7.96E-18	2.87E-17	4.36E-17	1.88E-17	6.13E-17	4.98E-17	4.01E-17	4.23E-17	3.31E-17
8.41E-17	1.35E-17	4.11E-17	1.65E-17	6.71E-19	5.96E-17	2.88E-16	4.58E-16	2.69E-16	7.37E-17	1.40E-17	6.30E-17	3.69E-17	6.38E-17	5.18E-17	4.69E-17	5.47E-17	7.29E-17
8.13E-17	1.21E-17	4.62E-17	1.37E-17	4.38E-17	2.86E-16	7.34E-16	8.63E-16	5.77E-16	2.37E-16	5.95E-20	6.48E-17	6.42E-17	7.07E-17	4.06E-17	5.33E-17	5.77E-17	8.16E-17
8.65E-17	2.79E-17	8.37E-17	3.59E-18	1.43E-16	6.03E-16	1.09E-15	1.09E-15	7.68E-16	3.79E-16	1.26E-17	5.10E-17	7.50E-17	7.66E-17	3.70E-17	8.41E-17	8.79E-17	8.31E-17
9.70E-17	6.59E-17	9.36E-17	1.67E-18	1.47E-16	5.96E-16	1.09E-15	9.61E-16	6.77E-16	3.37E-16	2.35E-17	3.56E-17	7.12E-17	9.88E-17	7.77E-17	8.77E-17	1.06E-16	8.05E-17
1.06E-16	5.76E-17	1.00E-16	6.57E-18	5.14E-17	3.08E-16	6.96E-16	5.95E-16	4.76E-16	1.98E-16	8.70E-18	2.00E-17	8.03E-17	1.24E-16	1.13E-16	1.19E-16	1.22E-16	8.53E-17
1.21E-16	5.14E-17	1.21E-16	4.06E-17	3.55E-19	5.83E-17	2.35E-16	1.71E-16	2.06E-16	5.63E-17	9.54E-19	2.39E-17	1.06E-16	1.13E-16	8.38E-17	1.16E-16	1.18E-16	8.37E-17
1.47E-16	1.40E-16	1.61E-16	9.79E-17	5.56E-17	2.66E-18	4.31E-18	8.84E-19	1.97E-17	1.03E-18	2.67E-17	6.25E-17	1.31E-16	1.27E-16	8.77E-17	1.17E-16	8.44E-17	7.70E-17
1.56E-16	1.53E-16	1.43E-16	1.31E-16	1.61E-16	6.19E-17	7.42E-17	7.84E-17	3.36E-17	7.22E-17	4.70E-17	6.45E-17	1.40E-16	1.47E-16	1.10E-16	1.07E-16	6.91E-17	8.81E-17
1.57E-16	1.94E-16	1.06E-16	1.10E-16	1.91E-16	1.82E-16	2.62E-16	2.84E-16	2.21E-16	1.39E-16	1.65E-17	3.87E-17	1.65E-16	1.92E-16	1.57E-16	1.36E-16	7.74E-17	8.68E-17
1.64E-16	1.62E-16	9.73E-17	1.10E-16	1.95E-16	2.11E-16	3.32E-16	4.31E-16	3.28E-16	1.44E-16	1.72E-19	1.01E-17	2.40E-16	2.62E-16	1.57E-16	1.47E-16	1.04E-16	9.57E-17
1.91E-16	1.80E-16	1.02E-16	1.05E-16	1.96E-16	2.25E-16	3.11E-16	4.74E-16	3.60E-16	1.76E-16	1.42E-19	3.58E-18	2.75E-16	2.69E-16	1.51E-16	1.37E-16	1.11E-16	9.92E-17
2.00E-16	1.72E-16	6.65E-17	7.44E-17	1.83E-16	2.46E-16	2.94E-16	4.79E-16	3.64E-16	2.73E-16	3.40E-17	1.05E-16	2.72E-16	2.70E-16	1.22E-16	1.35E-16	1.44E-16	1.10E-16
2.15E-16	9.37E-17	3.24E-17	5.43E-17	2.32E-16	2.46E-16	3.51E-16	5.48E-16	3.94E-16	3.42E-16	6.14E-17	1.24E-16	2.43E-16	2.49E-16	1.59E-16	1.51E-16	1.74E-16	1.67E-16
2.62E-16	5.16E-17	7.51E-18	4.33E-17	2.59E-16	2.58E-16	3.99E-16	5.90E-16	4.39E-16	3.05E-16	2.46E-18	8.06E-17	2.54E-16	2.26E-16	1.93E-16	1.93E-16	1.81E-16	2.15E-16
3.30E-16	9.98E-17	4.33E-18	2.45E-17	2.76E-16	2.95E-16	4.23E-16	6.28E-16	5.24E-16	3.16E-16	1.09E-19	8.35E-17	2.92E-16	2.17E-16	2.53E-16	2.26E-16	1.96E-16	2.45E-16
3.04E-16	3.83E-17	9.81E-18	1.02E-16	2.92E-16	3.51E-16	4.35E-16	6.68E-16	5.40E-16	4.31E-16	2.42E-17	2.00E-16	3.22E-16	2.31E-16	3.47E-16	2.52E-16	2.55E-16	2.51E-16
2.84E-16	4.36E-17	1.88E-17	2.27E-16	3.17E-16	3.71E-16	4.79E-16	6.84E-16	5.10E-16	5.10E-16	3.36E-16	3.17E-16	3.48E-16	3.53E-16	3.73E-16	2.92E-16	3.20E-16	2.64E-16
2.74E-16	2.58E-16	4.33E-16	3.63E-16	3.84E-16	4.15E-16	5.34E-16	7.52E-16	5.22E-16	5.24E-16	4.61E-16	3.57E-16	3.74E-16	3.94E-16	4.44E-16	3.41E-16	3.47E-16	2.75E-16
2.79E-16	1.79E-16	4.47E-16	4.09E-16	4.34E-16	4.46E-16	5.96E-16	8.04E-16	5.78E-16	5.27E-16	4.59E-16	3.70E-16	4.23E-16	4.10E-16	4.87E-16	4.27E-16	3.51E-16	3.27E-16
2.86E-16	2.56E-16	4.40E-16	4.30E-16	4.34E-16	4.57E-16	5.95E-16	8.25E-16	6.36E-16	5.71E-16	5.23E-16	4.17E-16	4.70E-16	3.93E-16	4.72E-16	4.45E-16	3.41E-16	3.18E-16
3.26E-16	4.89E-16	4.68E-16	4.40E-16	4.23E-16	4.54E-16	5.95E-16	8.64E-16	6.45E-16	6.24E-16	6.13E-16	4.52E-16	4.90E-16	4.66E-16	3.92E-16	4.29E-16	3.11E-16	3.14E-16
4.11E-16	4.64E-16	5.16E-16	4.67E-16	5.25E-16	4.97E-16	6.21E-16	8.79E-16	6.19E-16	6.57E-16	6.56E-16	4.75E-16	5.01E-16	4.70E-16	3.14E-16	4.04E-16	3.58E-16	3.24E-16
4.62E-16	5.93E-16	5.11E-16	4.61E-16	5.95E-16	6.01E-16	6.72E-16	8.25E-16	6.15E-16	6.47E-16	6.55E-16	4.97E-16	5.05E-16	4.04E-16	5.00E-16	4.74E-16	3.93E-16	3.30E-16
5.18E-16	5.11E-16	5.26E-16	4.79E-16	6.41E-16	5.79E-16	6.62E-16	7.74E-16	6.01E-16	6.53E-16	6.61E-16	5.81E-16	5.38E-16	3.63E-16	5.38E-16	4.77E-16	4.73E-16	3.80E-16
5.37E-16	4.96E-16	6.10E-16	5.39E-16	7.17E-16	6.44E-16	6.35E-16	8.44E-16	6.14E-16	7.04E-16	7.02E-16	5.88E-16	5.73E-16	4.74E-16	6.13E-16	5.09E-16	5.46E-16	5.41E-16
5.48E-16	5.36E-16	6.45E-16	5.93E-16	7.02E-16	7.36E-16	6.32E-16	9.12E-16	6.53E-16	7.31E-16	7.33E-16	5.78E-16	6.27E-16	6.04E-16	7.37E-16	6.01E-16	6.36E-16	6.04E-16
5.74E-16	5.35E-16	6.66E-16	6.00E-16	7.12E-16	8.14E-16	7.00E-16	9.54E-16	6.69E-16	7.35E-16	7.29E-16	6.15E-16	6.42E-16	6.28E-16	8.56E-16	6.64E-16	6.97E-16	6.67E-16
5.72E-16	4.76E-16	6.66E-16	6.10E-16	7.65E-16	8.05E-16	8.02E-16	9.58E-16	6.69E-16	7.51E-16	7.37E-16	5.95E-16	6.95E-16	6.48E-16	9.08E-16	6.48E-16	6.81E-16	7.28E-16
5.52E-16	5.00E-16	6.84E-16	5.83E-16	7.72E-16	7.53E-16	8.74E-16	9.38E-16	6.95E-16	7.71E-16	8.02E-16	6.78E-16	7.19E-16	7.41E-16	9.04E-16	6.35E-16	6.74E-16	7.81E-16
5.61E-16	7.22E-16	7.10E-16	6.53E-16	8.36E-16	8.01E-16	9.11E-16	9.24E-16	7.56E-16	8.29E-16	8.54E-16	7.66E-16	7.47E-16	7.17E-16	8.44E-16	6.53E-16	7.08E-16	7.91E-16
6.56E-16	6.87E-16	7.46E-16	7.59E-16	9.34E-16	9.30E-16	9.57E-16	1.08E-15	7.82E-16	9.09E-16	9.08E-16	8.77E-16	7.64E-16	6.93E-16	7.63E-16	7.54E-16	7.89E-16	7.65E-16
7.12E-16	5.43E-16	7.91E-16	7.93E-16	9.47E-16	9.03E-16	9.38E-16	1.12E-15	7.39E-16	9.69E-16	9.37E-16	8.99E-16	8.37E-16	7.76E-16	7.62E-16	8.20E-16	8.45E-16	7.79E-16
7.35E-16	4.62E-16	8.16E-16	7.85E-16	9.75E-16	8.66E-16	9.42E-16	1.16E-15	7.84E-16	1.01E-15	9.82E-16	8.67E-16	8.39E-16	8.49E-16	8.68E-16	7.92E-16	9.30E-16	8.55E-16
7.62E-16	5.88E-16	8.55E-16	7.90E-16	9.20E-16	9.16E-16	1.01E-15	1.09E-15	8.01E-16	1.05E-15	9.61E-16	8.55E-16	8.80E-16	8.72E-16	8.87E-16	7.64E-16	9.61E-16	8.64E-16
8.06E-16	7.29E-16	9.11E-16	7.85E-16	9.16E-16	9.85E-16	1.14E-15	1.11E-15	8.27E-16	1.08E-15	9.45E-16	9.17E-16	9.07E-16	9.04E-16	9.05E-16	8.57E-16	9.99E-16	9.14E-16

8.57E-16	7.76E-16	9.31E-16	8.40E-16	9.48E-16	9.65E-16	1.17E-15	1.08E-15	8.88E-16	1.11E-15	9.90E-16	1.02E-15	9.41E-16	8.80E-16	8.42E-16	8.99E-16	1.00E-15	9.62E-16
9.14E-16	9.79E-16	9.64E-16	8.43E-16	9.49E-16	9.39E-16	1.18E-15	1.19E-15	9.31E-16	1.15E-15	1.03E-15	1.09E-15	1.03E-15	8.61E-16	8.38E-16	8.66E-16	1.03E-15	1.08E-15
9.45E-16	8.35E-16	9.79E-16	8.26E-16	9.55E-16	9.36E-16	1.17E-15	1.13E-15	1.03E-15	1.18E-15	9.32E-16	1.10E-15	1.02E-15	8.08E-16	7.73E-16	8.88E-16	1.12E-15	1.12E-15
9.80E-16	7.75E-16	9.77E-16	8.64E-16	1.04E-15	1.02E-15	1.21E-15	1.37E-15	1.09E-15	1.15E-15	9.26E-16	1.03E-15	1.05E-15	7.13E-16	6.52E-16	9.85E-16	1.08E-15	1.18E-15
9.68E-16	8.98E-16	9.99E-16	9.08E-16	1.11E-15	1.01E-15	1.26E-15	1.40E-15	1.14E-15	1.12E-15	9.89E-16	9.86E-16	1.09E-15	6.70E-16	5.71E-16	1.04E-15	1.03E-15	1.11E-15
9.02E-16	1.06E-15	9.89E-16	9.89E-16	1.11E-15	9.70E-16	1.25E-15	1.45E-15	1.22E-15	1.17E-15	1.02E-15	1.03E-15	1.07E-15	1.24E-15	9.03E-16	1.07E-15	9.43E-16	1.03E-15
8.87E-16	1.03E-15	1.08E-15	1.03E-15	1.10E-15	9.03E-16	1.25E-15	1.57E-15	1.30E-15	1.26E-15	1.00E-15	1.03E-15	1.07E-15	1.27E-15	1.13E-15	1.02E-15	9.62E-16	1.00E-15
8.97E-16	9.09E-16	1.13E-15	1.08E-15	1.14E-15	8.87E-16	1.20E-15	1.59E-15	1.31E-15	1.20E-15	1.02E-15	1.00E-15	1.07E-15	1.20E-15	1.11E-15	9.98E-16	1.00E-15	1.08E-15
8.80E-16	1.07E-15	1.11E-15	1.11E-15	1.20E-15	8.66E-16	1.23E-15	1.51E-15	1.35E-15	1.17E-15	1.13E-15	9.94E-16	1.11E-15	1.07E-15	1.15E-15	1.08E-15	1.01E-15	1.03E-15
8.61E-16	1.19E-15	1.14E-15	1.13E-15	1.20E-15	8.07E-16	1.30E-15	1.43E-15	1.37E-15	1.33E-15	1.20E-15	1.07E-15	1.21E-15	1.03E-15	1.31E-15	1.18E-15	9.60E-16	9.69E-16
9.51E-16	1.12E-15	1.13E-15	1.10E-15	1.18E-15	8.68E-16	1.32E-15	1.41E-15	1.36E-15	1.44E-15	1.28E-15	1.13E-15	1.24E-15	1.02E-15	1.29E-15	1.14E-15	1.04E-15	9.71E-16
9.66E-16	1.05E-15	1.10E-15	1.07E-15	1.27E-15	1.26E-15	1.30E-15	1.51E-15	1.36E-15	1.45E-15	1.33E-15	1.12E-15	1.23E-15	1.21E-15	1.31E-15	1.11E-15	1.17E-15	1.04E-15
9.57E-16	8.78E-16	1.06E-15	1.05E-15	1.28E-15	1.33E-15	1.43E-15	1.45E-15	1.34E-15	1.45E-15	1.36E-15	1.25E-15	1.29E-15	1.20E-15	1.32E-15	1.18E-15	1.17E-15	1.08E-15
1.04E-15	8.45E-16	1.13E-15	1.02E-15	1.24E-15	1.35E-15	1.49E-15	1.32E-15	1.37E-15	1.55E-15	1.34E-15	1.29E-15	1.37E-15	1.27E-15	1.32E-15	1.26E-15	1.22E-15	1.11E-15
1.14E-15	9.87E-16	1.11E-15	9.12E-16	1.17E-15	1.26E-15	1.54E-15	1.21E-15	1.35E-15	1.66E-15	1.39E-15	1.24E-15	1.45E-15	1.34E-15	1.40E-15	1.32E-15	1.28E-15	1.14E-15
1.13E-15	9.07E-16	1.06E-15	9.73E-16	1.11E-15	1.27E-15	1.50E-15	1.13E-15	1.34E-15	1.70E-15	1.49E-15	1.22E-15	1.45E-15	1.41E-15	1.57E-15	1.48E-15	1.37E-15	1.24E-15
1.01E-15	6.40E-16	9.46E-16	9.47E-16	1.12E-15	1.39E-15	1.52E-15	1.23E-15	1.34E-15	1.67E-15	1.55E-15	1.26E-15	1.48E-15	1.37E-15	1.58E-15	1.59E-15	1.43E-15	1.38E-15
6.83E-14	5.99E-14	6.82E-14	6.50E-14	7.26E-14	7.01E-14	8.07E-14	8.78E-14	7.59E-14	8.30E-14	7.92E-14	7.79E-14	8.33E-14	8.15E-14	7.98E-14	7.88E-14	7.69E-14	7.76E-14

4.66E-08	5.14E-08	4.98E-08	5.06E-08	4.91E-08	5.03E-08	4.88E-08	5.05E-08	5.01E-08	5.38E-08	5.35E-08	5.04E-08	4.95E-08	5.11E-08	5.25E-08	5.08E-08	4.53E-08	4.41E-08	5.16E-08	5.29E-08	4.48E-08	2.43E-08	2.08E-08
4.35E-08	4.88E-08	4.90E-08	5.05E-08	4.87E-08	4.90E-08	4.89E-08	5.09E-08	5.05E-08	5.23E-08	5.09E-08	5.01E-08	4.85E-08	5.03E-08	5.25E-08	4.94E-08	4.33E-08	4.54E-08	5.89E-08	6.28E-08	5.20E-08	2.90E-08	2.14E-08
4.18E-08	4.59E-08	4.79E-08	4.97E-08	4.78E-08	4.75E-08	4.73E-08	4.89E-08	4.94E-08	5.02E-08	5.04E-08	4.94E-08	4.88E-08	4.96E-08	5.01E-08	4.82E-08	4.21E-08	4.60E-08	6.06E-08	6.48E-08	5.40E-08	3.23E-08	2.17E-08
4.11E-08	4.27E-08	4.46E-08	4.80E-08	4.72E-08	4.62E-08	4.52E-08	4.75E-08	4.62E-08	4.89E-08	5.07E-08	4.85E-08	4.88E-08	4.89E-08	4.89E-08	4.79E-08	4.26E-08	4.25E-08	5.33E-08	5.68E-08	4.79E-08	2.85E-08	2.07E-08
3.99E-08	4.19E-08	4.10E-08	4.63E-08	4.59E-08	4.53E-08	4.47E-08	4.69E-08	4.52E-08	4.87E-08	5.11E-08	4.76E-08	4.79E-08	4.82E-08	4.89E-08	4.74E-08	4.26E-08	3.76E-08	4.16E-08	4.33E-08	3.70E-08	2.14E-08	1.81E-08
3.89E-08	4.15E-08	3.79E-08	4.56E-08	4.52E-08	4.54E-08	4.41E-08	4.68E-08	4.48E-08	4.89E-08	5.13E-08	4.71E-08	4.70E-08	4.79E-08	4.97E-08	4.69E-08	4.15E-08	3.33E-08	3.07E-08	3.10E-08	2.50E-08	1.63E-08	1.74E-08
3.87E-08	4.07E-08	3.75E-08	4.48E-08	4.42E-08	4.41E-08	4.26E-08	4.45E-08	4.45E-08	4.66E-08	4.86E-08	4.70E-08	4.65E-08	4.80E-08	4.94E-08	4.63E-08	4.02E-08	3.22E-08	2.59E-08	2.38E-08	1.98E-08	1.55E-08	1.89E-08
3.83E-08	4.04E-08	3.71E-08	4.13E-08	4.30E-08	4.29E-08	3.99E-08	4.41E-08	4.39E-08	4.43E-08	4.58E-08	4.68E-08	4.67E-08	4.78E-08	4.72E-08	4.56E-08	4.02E-08	3.17E-08	2.55E-08	2.33E-08	1.97E-08	1.69E-08	2.15E-08
3.89E-08	4.07E-08	3.62E-08	3.88E-08	4.12E-08	4.15E-08	3.86E-08	4.32E-08	4.38E-08	4.31E-08	4.48E-08	4.63E-08	4.64E-08	4.72E-08	4.64E-08	4.54E-08	4.11E-08	3.14E-08	2.50E-08	2.31E-08	2.03E-08	2.01E-08	2.45E-08
4.04E-08	4.19E-08	3.63E-08	3.76E-08	3.97E-08	3.96E-08	3.80E-08	4.09E-08	4.25E-08	4.22E-08	4.37E-08	4.53E-08	4.58E-08	4.66E-08	4.60E-08	4.59E-08	4.10E-08	3.13E-08	2.51E-08	2.33E-08	2.15E-08	2.42E-08	2.77E-08
4.16E-08	4.31E-08	3.79E-08	3.80E-08	3.77E-08	3.67E-08	3.57E-08	3.77E-08	3.96E-08	4.03E-08	4.23E-08	4.37E-08	4.44E-08	4.58E-08	4.49E-08	4.60E-08	4.03E-08	3.12E-08	2.60E-08	2.49E-08	2.34E-08	2.66E-08	3.14E-08
4.28E-08	4.60E-08	3.93E-08	3.75E-08	3.63E-08	3.50E-08	3.28E-08	3.53E-08	3.66E-08	3.79E-08	3.99E-08	4.26E-08	4.28E-08	4.55E-08	4.39E-08	4.54E-08	3.97E-08	3.33E-08	2.89E-08	2.84E-08	2.79E-08	3.12E-08	3.86E-08
4.49E-08	5.09E-08	3.97E-08	3.58E-08	3.44E-08	3.40E-08	3.08E-08	3.33E-08	3.43E-08	3.54E-08	3.73E-08	3.99E-08	4.10E-08	4.48E-08	4.21E-08	4.44E-08	4.15E-08	3.75E-08	3.53E-08	3.62E-08	3.93E-08	4.42E-08	5.62E-08
4.70E-08	5.44E-08	3.97E-08	3.44E-08	3.38E-08	3.25E-08	3.02E-08	3.14E-08	3.28E-08	3.37E-08	3.50E-08	3.60E-08	3.86E-08	4.30E-08	4.10E-08	4.27E-08	4.42E-08	4.32E-08	4.72E-08	5.13E-08	5.92E-08	6.96E-08	8.75E-08
4.45E-08	4.84E-08	3.72E-08	3.33E-08	3.26E-08	3.25E-08	3.06E-08	2.83E-08	3.09E-08	3.34E-08	3.42E-08	3.37E-08	3.52E-08	3.91E-08	3.94E-08	4.21E-08	4.90E-08	5.53E-08	6.82E-08	7.66E-08	8.90E-08	1.05E-07	1.24E-07
3.85E-08	3.83E-08	3.28E-08	3.21E-08	3.16E-08	3.24E-08	3.07E-08	2.69E-08	3.00E-08	3.22E-08	3.39E-08	3.14E-08	3.31E-08	3.60E-08	3.61E-08	4.11E-08	5.83E-08	7.71E-08	9.82E-08	1.11E-07	1.25E-07	1.41E-07	1.55E-07
3.37E-08	3.36E-08	3.05E-08	3.09E-08	3.13E-08	3.14E-08	2.99E-08	2.65E-08	2.95E-08	3.06E-08	3.27E-08	3.04E-08	3.18E-08	3.51E-08	3.28E-08	3.92E-08	7.37E-08	1.07E-07	1.31E-07	1.43E-07	1.58E-07	1.69E-07	1.79E-07
3.18E-08	3.15E-08	2.86E-08	2.95E-08	3.09E-08	3.17E-08	2.99E-08	2.68E-08	2.84E-08	2.94E-08	3.14E-08	2.97E-08	3.06E-08	3.46E-08	3.17E-08	3.90E-08	9.24E-08	1.35E-07	1.52E-07	1.60E-07	1.80E-07	1.95E-07	2.04E-07
3.25E-08	2.95E-08	2.74E-08	2.75E-08	3.05E-08	3.23E-08	3.04E-08	2.75E-08	2.78E-08	2.93E-08	3.11E-08	2.81E-08	2.94E-08	3.33E-08	3.09E-08	4.11E-08	9.69E-08	1.43E-07	1.61E-07	1.70E-07	1.97E-07	2.18E-07	2.27E-07
3.15E-08	2.84E-08	2.65E-08	2.54E-08	2.95E-08	3.12E-08	3.19E-08	2.88E-08	2.77E-08	2.95E-08	3.11E-08	2.61E-08	2.81E-08	3.18E-08	2.92E-08	3.92E-08	8.55E-08	1.38E-07	1.61E-07	1.77E-07	2.08E-07	2.30E-07	2.40E-07
2.82E-08	2.88E-08	2.59E-08	2.45E-08	2.83E-08	3.02E-08	3.46E-08	3.12E-08	2.75E-08	2.91E-08	3.07E-08	2.50E-08	2.62E-08	3.12E-08	2.78E-08	3.39E-08	6.95E-08	1.28E-07	1.55E-07	1.78E-07	2.08E-07	2.28E-07	2.40E-07
2.77E-08	2.93E-08	2.52E-08	2.41E-08	2.84E-08	2.97E-08	3.76E-08	3.32E-08	2.78E-08	2.90E-08	2.73E-08	2.46E-08	2.49E-08	3.02E-08	2.64E-08	3.10E-08	5.06E-08	1.08E-07	1.38E-07	1.66E-07	1.89E-07	2.05E-07	2.17E-07
2.83E-08	2.85E-08	2.36E-08	2.34E-08	2.94E-08	3.05E-08	3.60E-08	3.06E-08	2.82E-08	2.95E-08	2.67E-08	2.41E-08	2.45E-08	2.90E-08	2.58E-08	2.98E-08	3.31E-08	7.72E-08	9.68E-08	1.23E-07	1.45E-07	1.59E-07	1.66E-07
2.76E-08	2.76E-08	2.39E-08	2.33E-08	2.97E-08	2.93E-08	3.03E-08	2.62E-08	2.85E-08	2.89E-08	2.60E-08	2.38E-08	2.42E-08	2.67E-08	2.65E-08	2.88E-08	2.39E-08	3.47E-08	4.91E-08	6.97E-08	9.67E-08	1.04E-07	1.11E-07
2.72E-08	2.91E-08	2.64E-08	2.53E-08	2.75E-08	2.70E-08	2.78E-08	2.59E-08	2.73E-08	2.70E-08	2.46E-08	2.42E-08	2.39E-08	2.61E-08	2.84E-08	2.86E-08	2.35E-08	1.97E-08	1.93E-08	2.71E-08	4.93E-08	5.23E-08	5.97E-08
2.85E-08	3.12E-08	2.77E-08	2.81E-08	2.60E-08	2.80E-08	2.85E-08	2.46E-08	2.68E-08	2.53E-08	2.33E-08	2.54E-08	2.39E-08	2.54E-08	2.81E-08	2.72E-08	2.32E-08	1.86E-08	9.70E-09	8.81E-09	1.19E-08	1.60E-08	2.21E-08
3.04E-08	3.30E-08	2.88E-08	2.89E-08	2.61E-08	2.93E-08	2.88E-08	2.34E-08	2.75E-08	2.28E-08	2.24E-08	2.65E-08	2.52E-08	2.40E-08	2.37E-08	2.38E-08	2.06E-08	1.78E-08	1.05E-08	7.28E-09	5.29E-09	3.99E-09	2.34E-09
3.41E-08	3.61E-08	3.10E-08	2.69E-08	2.54E-08	2.78E-08	2.77E-08	2.26E-08	2.71E-08	2.22E-08	2.30E-08	2.80E-08	2.68E-08	2.44E-08	2.21E-08	2.23E-08	1.96E-08	1.73E-08	1.09E-08	6.83E-09	5.76E-09	3.16E-09	-1.19E-09
3.94E-08	4.11E-08	3.42E-08	2.50E-08	2.54E-08	2.41E-08	2.49E-08	2.20E-08	2.34E-08	2.17E-08	2.20E-08	2.93E-08	2.73E-08	2.45E-08	2.29E-08	2.12E-08	2.02E-08	1.69E-08	1.09E-08	6.22E-09	5.51E-09	7.55E-10	-2.00E-09
4.26E-08	4.40E-08	3.55E-08	2.59E-08	2.60E-08	2.36E-08	2.37E-08	2.22E-08	2.26E-08	2.15E-08	2.05E-08	2.49E-08	2.41E-08	2.43E-08	2.31E-08	2.03E-08	2.03E-08	1.68E-08	1.25E-08	5.95E-09	3.48E-09	1.16E-09	-1.40E-09
3.91E-08	3.98E-08	3.23E-08	2.64E-08	2.69E-08	2.52E-08	2.45E-08	2.27E-08	2.33E-08	2.23E-08	1.99E-08	2.16E-08	2.31E-08	2.32E-08	2.22E-08	2.08E-08	1.88E-08	1.48E-08	1.56E-08	8.06E-09	2.87E-09	3.33E-09	-5.33E-10
3.22E-08	3.12E-08	2.63E-08	2.61E-08	2.71E-08	2.58E-08	2.57E-08	2.37E-08	2.47E-08	2.39E-08	2.11E-08	2.08E-08	2.34E-08	2.27E-08	2.16E-08	2.14E-08	1.74E-08	1.42E-08	1.96E-08	9.25E-09	3.04E-09	5.39E-09	6.49E-10
2.51E-08	2.29E-08	2.25E-08	2.53E-08	2.57E-08	2.47E-08	2.56E-08	2.46E-08	2.59E-08	2.51E-08	2.29E-08	2.22E-08	2.29E-08	2.18E-08	2.15E-08	2.21E-08	1.80E-08	1.52E-08	1.92E-08	9.27E-09	3.48E-09	2.38E-09	8.20E-10
2.19E-08	1.91E-08	2.18E-08	2.48E-08	2.45E-08	2.35E-08	2.42E-08	2.46E-08	2.56E-08	2.50E-08	2.34E-08	2.49E-08	2.36E-08	2.17E-08	2.20E-08	2.31E-08	1.91E-08	1.29E-08	1.26E-08	7.48E-09	3.39E-09	-1.71E-09	1.27E-09
2.03E-08	1.81E-08	2.19E-08	2.51E-08	2.37E-08	2.24E-08	2.37E-08	2.39E-08	2.45E-08	2.47E-08	2.23E-08	2.67E-08	2.57E-08	2.19E-08	2.35E-08	2.53E-08	2.00E-08	1.26E-08	9.58E-09	9.04E-09	3.54E-09	-1.12E-09	1.29E-09
1.89E-08	1.78E-08	2.20E-08	2.52E-08	2.32E-08	2.22E-08	2.31E-08	2.33E-08	2.45E-08	2.38E-08	2.20E-08	2.71E-08	2.73E-08	2.18E-08	2.37E-08	2.43E-08	1.99E-08	1.38E-08	9.88E-09	9.73E-09	4.52E-09	1.36E-09	7.38E-10
1.73E-08	1.69E-08	2.05E-08	2.30E-08	2.16E-08	2.18E-08	2.26E-08	2.25E-08	2.45E-08	2.25E-08	2.25E-08	2.54E-08	2.69E-08	2.12E-08	2.18E-08	2.18E-08	1.88E-08	1.43E-08	9.34E-09	7.77E-09	4.53E-09	2.73E-09	3.18E-10
1.47E-08	1.53E-08	1.85E-08	2.07E-08	2.12E-08	2.09E-08	2.22E-08	2.25E-08	2.30E-08	2.20E-08	2.25E-08	2.31E-08	2.53E-08	2.14E-08	2.10E-08	2.00E-08	1.87E-08	1.51E-08	8.75E-09	6.63E-09	4.13E-09	2.90E-09	-1.03E-10
1.41E-08	1.52E-08	1.80E-08	2.00E-08	2.14E-08	1.99E-08	2.11E-08	2.19E-08	2.04E-08	2.18E-08	2.12E-08	2.18E-08	2.48E-08	2.20E-08	2.14E-08	1.94E-08	1.83E-08	1.44E-08	8.97E-09	6.57E-09	3.68E-09	2.27E-09	-2.86E-10
1.46E-08	1.55E-08	1.80E-08	1.94E-08	2.04E-08	1.91E-08	1.94E-08	2.08E-08	1.90E-08	2.16E-08	2.02E-08	2.15E-08	2.33E-08	2.10E-08	2.13E-08	1.89E-08	1.68E-08	1.15E-08	8.93E-09	6.99E-09	2.98E-09	1.66E-09	-1.49E-09
1.61E-08	1.54E-08	1.77E-08	1.85E-08	1.96E-08	1.89E-08	1.89E-08	2.03E-08	1.85E-08	2.02E-08	1.98E-08	2.13E-08	2.10E-08	1.92E-08	2.02E-08	1.89E-08	1.60E-08	1.02E-08	9.00E-09	7.40E-09	3.15E-09	2.21E-09	-1.69E-09
1.73E-08	1.53E-08	1.75E-08	1.81E-08	1.95E-08	1.82E-08	1.87E-08	2.03E-08	1.74E-08	1.89E-08	1.89E-08	2.06E-08	2.00E-08	1.75E-08	1.88E-08	1.84E-08	1.55E-08	1.02E-08	8.44E-09	7.19E-09	3.74E-09	2.15E-09	-8.21E-10
1.73E-08	1.45E-08	1.80E-08	1.80E-08	1.87E-08	1.78E-08	1.85E-08	1.96E-08	1.58E-08	1.76E-08	1.78E-08	2.08E-08	1.94E-08	1.66E-08	1.87E-08	1.69E-08	1.47E-08	9.36E-09	7.06E-09	5.71E-09	2.64E-09	3.10E-10	-4.76E-10
1.72E-08	1.42E-08	1.76E-08	1.76E-08	1.75E-08	1.77E-08	1.75E-08	1.76E-08	1.50E-08	1.66E-08	1.63E-08	2.12E-08	1.89E-08	1.70E-08	1.90E-08	1.58E-08	1.38E-08	8.10E-09	4.61E-09	3.76E-09	1.03E-09	-7.10E-10	-2.19E-09
1.66E-08	1.45E-08	1.75E-08	1.80E-08	1.71E-08	1.72E-08	1.61E-08	1.58E-08	1.50E-08	1.52E-08	1.52E-08	2.12E-08	1.89E-08	1.68E-08	1.82E-08	1.58E-08	1.32E-08	7.39E-09	3.71E-09	3.20E-09	4.04E-10	-1.09E-09	-1.67E-09
1.56E-08	1.60E-08	2.02E-08	1.85E-08	1.69E-08	1.66E-08	1.48E-08	1.44E-08	1.45E-08	1.47E-08	1.44E-08	2.07E-08	1.80E-08	1.54E-08	1.60E-08	1.50E-08	1.26E-08	7.12E-09	2.63E-09	2.23E-09	1.39E-10	-1.23E-09	-5.78E-10
1.44E-08	1.81E-08	2.47E-08	1.73E-08	1.60E-08	1.55E-08	1.38E-08	1.28E-08	1.30E-08	1.40E-08	1.33E-08	1.74E-08	1.63E-08	1.40E-08	1.49E-08	1.35E-08	1.19E-08	6.95E-09	1.97E-09	6.47E-10	-1.47E-09	2.27E-10	-1.95E-09
1.38E-08	1.97E-08	2.82E-08	1.53E-08	1.44E-08	1.45E-08	1.29E-08	1.28E-08	1.24E-08	1.33E-08	1.27E-08	1.51E-08	1.50E-08	1.35E-08	1.42E-08	1.24E-08	1.09E-08	6.47E-09	1.61E-09	-9.16E-10	-2.83E-09	3.15E-09	-4.51E-09
1.27E-08	1.79E-08	2.42E-08	1.53E-08	1.26E-08	1.34E-08	1.24E-08	1.27E-08	1.16E-08	1.28E-08	1.20E-08	1.49E-08	1.44E-08	1.29E-08	1.39E-08	1.23E-08	1.08E-08	6.14E-09	1.21E-09	-1.77E-09	-3.76E-09	4.55E-09	-5.39E-09
1.20E-08	1.37E-08	1.49E-08	1.35E-08	1.21E-08	1.32E-08	1.22E-08	1.12E-08	1.05E-08	1.21E-08	1.15E-08	1.48E-08	1.41E-08	1.18E-08	1.33E-08	1.22E-08	1.11E-08	5.92E-09	8.96E-10	-1.57E-09	-3.87E-09	1.21E-09	-6.30E-09
1.15E-08	1.27E-08	1.27E-08	1.38E-08	1.14E-08	1.29E-08	1.10E-08	1.03E-08	1.01E-08	1.10E-08	1.09E-08	1.37E-08	1.35E-08	1.12E-08	1.19E-08	1.15E-08	1.13E-08	6.07E-09	1.65E-10	-8.74E-10	-3.99E-09	-6.82E-09	-6.26E-09
1.10E-08	1.17E-08	1.27E-08	1.29E-08	9.87E-09	1.09E-08	1.01E-08	1.05E-08	1.02E-08	9.56E-09	1.00E-08	1.24E-08	1.31E-08	1.10E-08	1.07E-08	9.53E-09	9.98E-09	5.61E-09	-2.18E-10	-6.12E-10	-5.15E-09	-6.63E-09	-6.15E-09
1.01E-08	1.12E-08	1.30E-08	1.10E-08	9.99E-09	1.04E-08	9.54E-09	1.03E-08	1.04E-08	8.69E-09	8.33E-09	1.22E-08	1.26E-08	1.13E-08	9.72E-09	7.71E-09	8.88E-09	4.63E-09	-5.57E-10	-6.35E-10	-5.41E-09	-6.61E-09	-6.87E-09
8.83E-09	1.00E-08	1.19E-08	1.00E-08	1.01E-08	9.81E-09	9.30E-09	9.78E-09	1.06E-08	8.62E-09	7.51E-09	1.21E-08	1.23E-08	1.18E-08	9.12E-09	6.66E-09	7.61E-09	4.12E-09	-9.92E-10	-1.80E-09	-5.98E-09	-7.46E-09	-8.10E-09
7.64E-09	7.75E-09	8.10E-09	9.07E-09	9.15E-09	9.89E-09	8.95E-09	8.06E-09	1.10E-08	9.19E-09	6.91E-09	1.09E-08	1.11E-08	1.23E-08	7.65E-09	6.26E-09	6.66E-09	3.74E-09	-1.82E-09	-3.39E-09	-7.05E-09	-7.49E-09	-1.08E-08

7.43E-09	5.58E-09	6.63E-09	8.85E-09	8.05E-09	9.45E-09	9.20E-09	8.31E-09	1.14E-08	1.06E-08	6.79E-09	9.62E-09	1.04E-08	1.25E-08	6.98E-09	5.78E-09	5.54E-09	2.94E-09	-2.77E-09	-4.77E-09	-7.64E-09	-8.27E-09	-1.22E-08
5.32E-09	4.02E-09	6.67E-09	8.25E-09	6.99E-09	8.56E-09	1.03E-08	9.07E-09	1.13E-08	1.16E-08	7.32E-09	8.95E-09	9.47E-09	1.03E-08	7.57E-09	5.61E-09	3.88E-09	1.39E-09	-3.36E-09	-6.31E-09	-8.09E-09	-1.02E-08	-1.45E-08
3.95E-09	3.35E-09	6.08E-09	7.26E-09	6.84E-09	8.21E-09	1.10E-08	9.75E-09	9.92E-09	9.87E-09	6.65E-09	8.62E-09	7.98E-09	8.80E-09	9.41E-09	6.07E-09	2.76E-09	-8.86E-11	-4.11E-09	-7.66E-09	-8.03E-09	-1.06E-08	-1.62E-08
3.08E-09	2.68E-09	5.17E-09	7.13E-09	6.25E-09	7.80E-09	1.06E-08	9.54E-09	8.73E-09	8.35E-09	5.32E-09	6.88E-09	5.92E-09	7.88E-09	1.16E-08	7.31E-09	1.91E-09	-1.26E-09	-4.04E-09	-7.72E-09	-7.92E-09	-1.03E-08	-1.69E-08
2.98E-09	1.72E-09	4.82E-09	7.09E-09	5.32E-09	6.61E-09	7.76E-09	7.46E-09	8.22E-09	7.22E-09	4.64E-09	5.44E-09	5.94E-09	8.28E-09	1.29E-08	8.41E-09	1.40E-09	-2.20E-09	-4.06E-09	-7.90E-09	-8.24E-09	-1.05E-08	-1.66E-08
1.89E-09	8.38E-10	3.84E-09	5.34E-09	3.96E-09	5.03E-09	6.00E-09	7.31E-09	8.14E-09	5.99E-09	4.54E-09	4.87E-09	6.00E-09	8.19E-09	1.08E-08	6.88E-09	8.61E-10	-2.78E-09	-5.12E-09	-8.60E-09	-8.80E-09	-1.04E-08	-1.68E-08
7.48E-10	-1.69E-10	2.46E-09	4.11E-09	2.99E-09	4.15E-09	4.33E-09	4.73E-09	6.76E-09	4.90E-09	4.10E-09	4.15E-09	3.63E-09	5.79E-09	5.69E-09	3.91E-09	3.79E-10	-3.25E-09	-7.03E-09	-9.83E-09	-9.13E-09	-1.01E-08	-1.75E-08
-8.53E-10	-1.50E-09	1.97E-09	4.00E-09	2.75E-09	3.36E-09	3.35E-09	3.86E-09	4.95E-09	4.84E-09	3.75E-09	3.44E-09	2.52E-09	4.12E-09	3.88E-09	3.16E-09	-1.08E-09	-3.93E-09	-6.96E-09	-1.04E-08	-9.73E-09	-1.11E-08	-1.76E-08
-9.72E-10	-1.76E-09	2.06E-09	4.17E-09	2.60E-09	2.04E-09	2.83E-09	3.81E-09	4.46E-09	4.89E-09	2.90E-09	2.85E-09	2.89E-09	4.49E-09	3.70E-09	2.03E-09	-2.13E-09	-5.21E-09	-7.08E-09	-1.05E-08	-1.15E-08	-1.31E-08	-1.91E-08
-1.45E-09	-2.09E-09	2.22E-09	2.87E-09	1.65E-09	5.35E-10	2.43E-09	3.48E-09	3.78E-09	4.15E-09	2.94E-09	3.16E-09	2.97E-09	5.54E-09	3.80E-09	1.69E-09	-1.90E-09	-6.40E-09	-8.20E-09	-1.15E-08	-1.30E-08	-1.41E-08	-2.09E-08
-2.50E-09	-2.67E-09	1.84E-09	1.99E-09	1.93E-09	-6.53E-10	1.45E-09	3.55E-09	3.50E-09	3.89E-09	3.98E-09	3.51E-09	3.04E-09	4.71E-09	3.36E-09	1.75E-09	-1.03E-09	-6.80E-09	-8.89E-09	-1.22E-08	-1.39E-08	-1.48E-08	-2.16E-08
-3.40E-09	-2.92E-09	1.58E-09	1.84E-09	2.31E-09	-2.56E-10	9.16E-10	3.95E-09	3.33E-09	3.94E-09	4.52E-09	3.13E-09	2.67E-09	3.00E-09	3.01E-09	1.74E-09	-1.20E-09	-7.22E-09	-8.98E-09	-1.24E-08	-1.38E-08	-1.58E-08	-2.01E-08
-3.78E-09	-3.18E-09	1.52E-09	1.91E-09	2.52E-09	-3.85E-10	1.60E-09	2.77E-09	3.14E-09	3.86E-09	3.46E-09	2.22E-09	1.92E-09	1.81E-09	3.04E-09	1.15E-09	-2.75E-09	-8.08E-09	-9.05E-09	-1.23E-08	-1.32E-08	-1.59E-08	-1.91E-08
-2.84E-09	-2.98E-09	7.77E-10	1.80E-09	2.71E-09	-1.63E-09	9.85E-10	1.80E-09	3.39E-09	3.07E-09	1.78E-09	1.32E-09	7.92E-10	1.44E-09	2.21E-09	-1.80E-10	-4.62E-09	-9.67E-09	-9.30E-09	-1.20E-08	-1.35E-08	-1.56E-08	-2.03E-08
-1.97E-09	-3.02E-09	-8.35E-10	1.46E-09	3.01E-09	-3.02E-09	-9.98E-10	4.29E-10	3.17E-09	2.35E-09	1.31E-09	3.67E-10	1.06E-10	7.88E-10	7.39E-10	-1.38E-09	-5.71E-09	-1.04E-08	-1.12E-08	-1.35E-08	-1.50E-08	-1.63E-08	-2.08E-08
-1.52E-09	-3.88E-09	-1.54E-09	-1.12E-09	3.54E-09	-3.18E-09	-2.69E-09	-7.51E-10	2.03E-09	1.80E-09	9.30E-10	-6.95E-10	-9.66E-10	-9.52E-10	-5.57E-10	-2.31E-09	-6.76E-09	-1.09E-08	-1.32E-08	-1.53E-08	-1.59E-08	-1.73E-08	-2.05E-08
-1.58E-09	-4.69E-09	-2.55E-09	-3.06E-09	4.81E-10	-2.79E-09	-2.38E-09	-1.34E-09	5.10E-10	1.09E-09	1.30E-10	-1.56E-09	-2.03E-09	-2.39E-09	-1.74E-09	-2.90E-09	-7.87E-09	-1.14E-08	-1.35E-08	-1.64E-08	-1.71E-08	-1.84E-08	-2.00E-08
-1.78E-09	-6.03E-09	-4.38E-09	-5.11E-09	-1.67E-09	-2.04E-09	-1.98E-09	-1.84E-09	2.55E-10	1.34E-10	-5.16E-10	-1.91E-09	-2.79E-09	-2.93E-09	-3.63E-09	-3.75E-09	-8.31E-09	-1.16E-08	-1.33E-08	-1.68E-08	-1.84E-08	-1.96E-08	-2.12E-08
-3.48E-09	-7.89E-09	-6.11E-09	-5.24E-09	-1.05E-09	-2.53E-09	-2.41E-09	-2.51E-09	1.17E-10	-3.87E-10	-1.23E-09	-2.21E-09	-3.28E-09	-3.58E-09	-5.79E-09	-4.95E-09	-8.37E-09	-1.17E-08	-1.37E-08	-1.73E-08	-1.97E-08	-2.07E-08	-2.27E-08
-5.66E-09	-8.68E-09	-6.44E-09	-5.82E-09	-1.66E-09	-3.55E-09	-3.08E-09	-1.59E-09	-1.03E-09	-1.13E-09	-1.74E-09	-3.76E-09	-4.41E-09	-5.64E-09	-7.13E-09	-6.05E-09	-9.09E-09	-1.13E-08	-1.46E-08	-1.82E-08	-2.09E-08	-2.23E-08	-2.43E-08
-7.01E-09	-8.72E-09	-6.83E-09	-6.45E-09	-3.51E-09	-4.01E-09	-3.45E-09	-5.44E-10	-1.87E-09	-2.71E-09	-2.80E-09	-5.51E-09	-5.85E-09	-7.31E-09	-7.59E-09	-6.59E-09	-9.82E-09	-1.05E-08	-1.51E-08	-1.89E-08	-2.14E-08	-2.43E-08	-2.59E-08
-8.69E-09	-9.10E-09	-6.83E-09	-6.43E-09	-4.85E-09	-4.53E-09	-3.66E-09	-1.47E-09	-4.06E-09	-4.92E-09	-4.22E-09	-6.83E-09	-6.77E-09	-8.20E-09	-8.21E-09	-7.54E-09	-1.08E-08	-1.05E-08	-1.62E-08	-1.93E-08	-2.28E-08	-2.49E-08	-2.67E-08
-1.06E-08	-1.07E-08	-6.57E-09	-6.54E-09	-5.81E-09	-5.28E-09	-4.39E-09	-3.60E-09	-4.54E-09	-6.79E-09	-4.08E-09	-7.45E-09	-7.32E-09	-8.73E-09	-9.78E-09	-9.65E-09	-1.24E-08	-1.16E-08	-1.75E-08	-2.05E-08	-2.43E-08	-2.52E-08	-2.73E-08
-1.19E-08	-1.20E-08	-6.58E-09	-7.34E-09	-6.31E-09	-6.61E-09	-5.85E-09	-5.53E-09	-6.11E-09	-7.12E-09	-3.14E-09	-8.11E-09	-7.50E-09	-1.05E-08	-1.10E-08	-1.14E-08	-1.37E-08	-1.41E-08	-1.85E-08	-2.20E-08	-2.52E-08	-2.63E-08	-2.74E-08
-1.16E-08	-1.29E-08	-6.78E-09	-7.13E-09	-6.70E-09	-8.14E-09	-7.56E-09	-6.61E-09	-6.92E-09	-7.32E-09	-1.41E-09	-8.45E-09	-8.52E-09	-1.17E-08	-1.17E-08	-1.18E-08	-1.44E-08	-1.64E-08	-1.91E-08	-2.27E-08	-2.56E-08	-2.73E-08	-2.79E-08
-1.11E-08	-1.35E-08	-8.17E-09	-7.11E-09	-7.14E-09	-8.77E-09	-8.64E-09	-7.16E-09	-7.79E-09	-8.51E-09	1.30E-10	-8.61E-09	-1.01E-08	-1.26E-08	-1.18E-08	-1.17E-08	-1.43E-08	-1.62E-08	-1.99E-08	-2.27E-08	-2.61E-08	-2.69E-08	-2.76E-08
-1.14E-08	-1.42E-08	-9.91E-09	-8.26E-09	-7.33E-09	-8.56E-09	-8.82E-09	-8.52E-09	-8.88E-09	-1.12E-08	-4.09E-09	-1.13E-08	-1.12E-08	-1.37E-08	-1.29E-08	-1.23E-08	-1.45E-08	-1.59E-08	-2.04E-08	-2.30E-08	-2.68E-08	-2.67E-08	-2.79E-08
-1.25E-08	-1.45E-08	-1.12E-08	-1.03E-08	-8.02E-09	-8.19E-09	-9.72E-09	-9.19E-09	-9.44E-09	-1.30E-08	-1.09E-08	-1.43E-08	-1.18E-08	-1.53E-08	-1.56E-08	-1.38E-08	-1.55E-08	-1.69E-08	-2.09E-08	-2.34E-08	-2.73E-08	-2.74E-08	-2.79E-08
-1.31E-08	-1.44E-08	-1.18E-08	-1.17E-08	-9.09E-09	-9.53E-09	-1.07E-08	-1.02E-08	-9.73E-09	-1.26E-08	-1.41E-08	-1.53E-08	-1.26E-08	-1.69E-08	-1.71E-08	-1.48E-08	-1.58E-08	-1.82E-08	-2.06E-08	-2.26E-08	-2.68E-08	-2.76E-08	-2.77E-08
-1.27E-08	-1.43E-08	-1.17E-08	-1.23E-08	-1.03E-08	-1.09E-08	-1.12E-08	-8.97E-09	-1.01E-08	-1.25E-08	-1.44E-08	-1.56E-08	-1.32E-08	-1.68E-08	-1.63E-08	-1.44E-08	-1.57E-08	-1.88E-08	-2.05E-08	-2.14E-08	-2.51E-08	-2.63E-08	-2.69E-08
-1.29E-08	-1.33E-08	-9.72E-09	-1.21E-08	-1.10E-08	-1.15E-08	-1.04E-08	-6.46E-09	-1.09E-08	-1.34E-08	-1.53E-08	-1.63E-08	-1.45E-08	-1.63E-08	-1.52E-08	-1.44E-08	-1.65E-08	-1.91E-08	-2.09E-08	-2.06E-08	-2.44E-08	-2.66E-08	-2.61E-08
-1.20E-08	-1.11E-08	-8.06E-09	-1.21E-08	-1.30E-08	-1.21E-08	-8.99E-09	-3.31E-09	-1.10E-08	-1.43E-08	-1.56E-08	-1.71E-08	-1.56E-08	-1.67E-08	-1.55E-08	-1.58E-08	-1.82E-08	-1.92E-08	-2.16E-08	-2.13E-08	-2.58E-08	-2.70E-08	-2.55E-08
-1.08E-08	-8.95E-09	-7.22E-09	-1.09E-08	-1.40E-08	-1.31E-08	-1.01E-08	-3.49E-09	-1.15E-08	-1.44E-08	-1.58E-08	-1.79E-08	-1.59E-08	-1.70E-08	-1.60E-08	-1.68E-08	-1.96E-08	-2.03E-08	-2.26E-08	-2.37E-08	-2.72E-08	-2.76E-08	-2.57E-08
-1.04E-08	-8.07E-09	-5.69E-09	-9.79E-09	-1.46E-08	-1.44E-08	-1.44E-08	-1.03E-08	-1.41E-08	-1.50E-08	-1.65E-08	-1.84E-08	-1.61E-08	-1.75E-08	-1.66E-08	-1.75E-08	-2.06E-08	-2.24E-08	-2.39E-08	-2.46E-08	-2.75E-08	-2.83E-08	-2.68E-08
-1.11E-08	-9.54E-09	-6.09E-09	-1.06E-08	-1.57E-08	-1.59E-08	-1.65E-08	-1.71E-08	-1.66E-08	-1.66E-08	-1.83E-08	-1.91E-08	-1.65E-08	-1.76E-08	-1.76E-08	-1.84E-08	-2.10E-08	-2.43E-08	-2.56E-08	-2.52E-08	-2.81E-08	-2.95E-08	-2.75E-08
-1.30E-08	-1.25E-08	-1.00E-08	-1.30E-08	-1.75E-08	-1.79E-08	-1.88E-08	-1.83E-08	-1.82E-08	-1.86E-08	-1.99E-08	-2.03E-08	-1.82E-08	-1.81E-08	-1.86E-08	-1.93E-08	-2.12E-08	-2.52E-08	-2.71E-08	-2.67E-08	-2.88E-08	-3.04E-08	-2.92E-08
-1.52E-08	-1.59E-08	-1.51E-08	-1.58E-08	-1.89E-08	-1.99E-08	-2.05E-08	-1.96E-08	-1.94E-08	-2.00E-08	-2.09E-08	-2.11E-08	-2.01E-08	-1.97E-08	-1.97E-08	-1.98E-08	-2.24E-08	-2.70E-08	-2.91E-08	-2.83E-08	-2.92E-08	-3.15E-08	-3.12E-08
-1.64E-08	-1.78E-08	-1.80E-08	-1.94E-08	-2.05E-08	-2.05E-08	-2.15E-08	-2.23E-08	-2.07E-08	-2.04E-08	-2.13E-08	-2.15E-08	-2.10E-08	-2.13E-08	-2.09E-08	-2.07E-08	-2.39E-08	-2.90E-08	-3.06E-08	-3.01E-08	-2.95E-08	-3.27E-08	-3.31E-08
-1.69E-08	-1.96E-08	-1.86E-08	-2.09E-08	-2.09E-08	-2.10E-08	-2.25E-08	-2.36E-08	-2.19E-08	-2.14E-08	-2.20E-08	-2.21E-08	-2.16E-08	-2.16E-08	-2.22E-08	-2.25E-08	-2.51E-08	-3.04E-08	-3.14E-08	-3.19E-08	-3.01E-08	-3.25E-08	-3.22E-08
-1.89E-08	-2.13E-08	-1.96E-08	-2.18E-08	-2.21E-08	-2.17E-08	-2.27E-08	-2.42E-08	-2.30E-08	-2.22E-08	-2.21E-08	-2.27E-08	-2.22E-08	-2.19E-08	-2.31E-08	-2.39E-08	-2.56E-08	-3.14E-08	-3.29E-08	-3.29E-08	-3.08E-08	-3.26E-08	-3.14E-08
-2.05E-08	-2.25E-08	-2.04E-08	-2.26E-08	-2.28E-08	-2.19E-08	-2.20E-08	-2.55E-08	-2.32E-08	-2.16E-08	-2.15E-08	-2.28E-08	-2.22E-08	-2.21E-08	-2.38E-08	-2.43E-08	-2.57E-08	-3.18E-08	-3.40E-08	-3.32E-08	-3.13E-08	-3.33E-08	-3.27E-08
-2.02E-08	-2.31E-08	-2.13E-08	-2.31E-08	-2.32E-08	-2.16E-08	-2.21E-08	-2.65E-08	-2.34E-08	-2.14E-08	-2.17E-08	-2.19E-08	-2.18E-08	-2.22E-08	-2.39E-08	-2.44E-08	-2.54E-08	-3.19E-08	-3.42E-08	-3.39E-08	-3.22E-08	-3.48E-08	-3.38E-08
-2.07E-08	-2.38E-08	-2.20E-08	-2.35E-08	-2.32E-08	-2.11E-08	-2.23E-08	-2.61E-08	-2.33E-08	-2.20E-08	-2.20E-08	-2.10E-08	-2.20E-08	-2.25E-08	-2.39E-08	-2.47E-08	-2.50E-08	-3.18E-08	-3.44E-08	-3.49E-08	-3.35E-08	-3.56E-08	-3.41E-08
-2.04E-08	-2.39E-08	-2.30E-08	-2.46E-08	-2.33E-08	-2.15E-08	-2.19E-08	-2.54E-08	-2.35E-08	-2.24E-08	-2.21E-08	-2.07E-08	-2.24E-08	-2.28E-08	-2.46E-08	-2.55E-08	-2.51E-08	-3.20E-08	-3.61E-08	-3.51E-08	-3.46E-08	-3.54E-08	-3.42E-08
-2.15E-08	-2.42E-08	-2.34E-08	-2.47E-08	-2.32E-08	-2.18E-08	-2.13E-08	-2.52E-08	-2.40E-08	-2.19E-08	-2.22E-08	-2.09E-08	-2.34E-08	-2.38E-08	-2.55E-08	-2.62E-08	-2.64E-08	-3.23E-08	-3.76E-08	-3.51E-08	-3.56E-08	-3.61E-08	-3.45E-08
-2.35E-08	-2.49E-08	-2.35E-08	-2.51E-08	-2.38E-08	-2.24E-08	-2.20E-08	-2.63E-08	-2.44E-08	-2.24E-08	-2.31E-08	-2.19E-08	-2.41E-08	-2.48E-08	-2.57E-08	-2.67E-08	-2.77E-08	-3.19E-08	-3.70E-08	-3.58E-08	-3.66E-08	-3.74E-08	-3.53E-08
-2.48E-08	-2.51E-08	-2.54E-08	-2.63E-08	-2.47E-08	-2.32E-08	-2.34E-08	-2.68E-08	-2.51E-08	-2.42E-08	-2.42E-08	-2.30E-08	-2.46E-08	-2.54E-08	-2.53E-08	-2.71E-08	-2.80E-08	-3.14E-08	-3.47E-08	-3.63E-08	-3.70E-08	-3.72E-08	-3.57E-08
-2.60E-08	-2.51E-08	-2.70E-08	-2.67E-08	-2.51E-08	-2.39E-08	-2.35E-08	-2.66E-08	-2.70E-08	-2.63E-08	-2.55E-08	-2.41E-08	-2.49E-08	-2.57E-08	-2.56E-08	-2.74E-08	-2.78E-08	-3.13E-08	-3.35E-08	-3.61E-08	-3.71E-08	-3.67E-08	-3.53E-08
-2.79E-08	-2.57E-08	-2.81E-08	-2.68E-08	-2.57E-08	-2.52E-08	-2.41E-08	-2.68E-08	-2.76E-08	-2.70E-08	-2.66E-08	-2.48E-08	-2.49E-08	-2.58E-08	-2.67E-08	-2.79E-08	-2.79E-08	-3.18E-08	-3.42E-08	-3.54E-08	-3.74E-08	-3.74E-08	-3.44E-08
-2.89E-08	-2.70E-08	-2.90E-08	-2.71E-08	-2.66E-08	-2.53E-08	-2.50E-08	-2.71E-08	-2.78E-08	-2.66E-08	-2.74E-08	-2.56E-08	-2.53E-08	-2.60E-08	-2.73E-08	-2.79E-08	-2.86E-08	-3.20E-08	-3.47E-08	-3.61E-08	-3.84E-08	-3.79E-08	-3.38E-08
-2.81E-08	-2.81E-08	-2.99E-08	-2.92E-08	-2.88E-08	-2.66E-08	-2.60E-08	-2.72E-08	-2.82E-08	-2.72E-08	-2.89E-08	-2.71E-08	-2.60E-08	-2.61E-08	-2.67E-08	-2.76E-08	-2.89E-08	-3.17E-08	-3.37E-08	-3.66E-08	-3.95E-08	-3.71E-08	-3.42E-08
-2.72E-08	-2.75E-08	-2.99E-08	-3.00E-08	-2.92E-08	-2.80E-08	-2.63E-08	-2.67E-08	-2.86E-08	-2.84E-08	-2.99E-08	-2.88E-08	-2.79E-08	-2.69E-08	-2.69E-08	-2.87E-08	-2.88E-08	-3.11E-08	-3.30E-08	-3.56E-08	-3.89E-08	-3.65E-08	-3.48E-08
-2.78E-08	-2.70E-08	-2.95E-08	-2.97E-08	-2.85E-08	-2.82E-08	-2.60E-08	-2.70E-08	-2.86E-08	-2.87E-08	-2.99E-08	-2.98E-08	-3.00E-08	-2.84E-08	-2.84E-08	-3.04E-08	-2.98E-08	-3.21E-08	-3.38E-08	-3.51E-08	-3.85E-08	-3.70E-08	-3.49E-08
-2.91E-08	-2.85E-08	-2.96E-08	-3.00E-08	-2.89E-08	-2.86E-08	-2.62E-08	-2.70E-08	-2.81E-08	-2.95E-08	-3.07E-08	-3.07E-08	-3.08E-08	-2.97E-08	-2.96E-08	-3.14E-08	-3.16E-08	-3.41E-08	-3.51E-08	-3.61E-08	-4.01E-08	-3.83E-08	-3.54E-08
-3.02E-08	-3.00E-08	-3.05E-08	-3.15E-08	-3.10E-08	-2.93E-08	-2.72E-08	-2.71E-08	-2.78E-08	-3.06E-08	-3.24E-08	-3.20E-08	-3.05E-08	-3.01E-08	-2.99E-08	-3.17E-08	-3.29E-08	-3.50E-08	-3.52E-08	-3.75E-08	-4.28E-08	-3.96E-08	-3.66E-08
-3.09E-08	-3.14E-08	-3.21E-08	-3.30E-08	-3.23E-08	-3.04E-08	-2.89E-08	-2.81E-08	-2.85E-08	-3.18E-08	-3.31E-08	-3.28E-08	-3.01E-08	-3.00E-08	-3.00E-08	-3.23E-08	-3.34E-08	-3.48E-08	-3.45E-08	-3.85E-08	-4.41E-08	-4.11E-08	-3.81E-08
-3.24E-08	-3.18E-08	-3.27E-08	-3.29E-08	-3.21E-08	-3.04E-08	-3.04E-08	-2.94E-08	-2.96E-08	-3.32E-08	-3.37E-08	-3.27E-08	-3.15E-08	-3.03E-08	-3.03E-08	-3.31E-08	-3.43E-08	-3.50E-08	-3.45E-08	-3.92E-08	-4.39E-08	-4.11E-08	-3.90E-08
-3.41E-08	-3.25E-08	-3.36E-08	-3.27E-08	-3.23E-08	-3.04E-08	-3.04E-08	-3.06E-08	-3.00E-08	-3.40E-08	-3.45E-08	-3.38E-08	-3.36E-08	-3.14E-08	-3.12E-08	-3.35E-08	-3.59E-08	-3.64E-08	-3.57E-08	-4.00E-08	-4.34E-08	-3.99E-08	-3.97E-08
-3.40E-08	-3.41E-08	-3.46E-08	-3.27E-08	-3.34E-08	-2.95E-08	-2.98E-08	-3.17E-08	-3.04E-08	-3.40E-08	-3.48E-08	-3.49E-08	-3.44E-08	-3.23E-08	-3.25E-08	-3.34E-08	-3.66E-08	-3.80E-08	-3.71E-08	-4.03E-08	-4.33E-08	-3.95E-08	-4.10E-08
-3.49E-08	-3.43E-08	-3.53E-08	-3.39E-08	-3.52E-08	-2.77E-08	-2.98E-08	-3.34E-08	-3.19E-08	-3.41E-08	-3.49E-08	-3.45E-08	-3.45E-08	-3.39E-08	-3.36E-08	-3.37E-08	-3.60E-08	-3.90E-08	-3.78E-08	-4.10E-08	-4.31E-08	-4.04E-08	-4.22E-08
-3.43E-08	-3.37E-08	-3.56E-08	-3.49E-08	-3.49E-08	-2.82E-08	-3.35E-08	-3.46E-08	-3.30E-08	-3.42E-08	-3.48E-08	-3.38E-08	-3.45E-08	-3.59E-08	-3.47E-08	-3.43E-08	-3.63E-08	-3.94E-08	-3.84E-08	-4.02E-08	-4.18E-08	-4.05E-08	-4.32E-08
-3.43E-08	-3.39E-08	-3.56E-08	-3.43E-08	-3.50E-08	-3.35E-08	-3.46E-08	-3.49E-08	-3.31E-08	-3.44E-08	-3.52E-08	-3.43E-08	-3.53E-08	-3.75E-08	-3.57E-08	-3.48E-08	-3.69E-08	-4.01E-08	-3.97E-08	-3.94E-08	-4.09E-08	-3.94E-08	-4.48E-08
-3.57E-08	-3.50E-08	-3.47E-08	-3.34E-08	-3.55E-08	-3.38E-08	-3.43E-08	-3.46E-08	-3.34E-08	-3.47E-08	-3.48E-08	-3.50E-08	-3.69E-08	-3.74E-08	-3.59E-08	-3.50E-08	-3.74E-08	-4.05E-08	-4.01E-08	-3.94E-08	-4.16E-08	-3.92E-08	-4.54E-08

-3.54E-08	-3.58E-08	-3.43E-08	-3.32E-08	-3.53E-08	-3.32E-08	-3.43E-08	-3.54E-08	-3.40E-08	-3.49E-08	-3.37E-08	-3.49E-08	-3.78E-08	-3.69E-08	-3.59E-08	-3.49E-08	-3.76E-08	-4.05E-08	-3.95E-08	-4.02E-08	-4.28E-08	-4.05E-08	-4.59E-08
-3.49E-08	-3.62E-08	-3.55E-08	-3.35E-08	-3.50E-08	-3.25E-08	-3.46E-08	-3.59E-08	-3.47E-08	-3.51E-08	-3.30E-08	-3.47E-08	-3.71E-08	-3.76E-08	-3.61E-08	-3.50E-08	-3.78E-08	-4.04E-08	-3.90E-08	-4.05E-08	-4.32E-08	-4.20E-08	-4.58E-08
-3.51E-08	-3.58E-08	-3.70E-08	-3.41E-08	-3.57E-08	-3.25E-08	-3.47E-08	-3.57E-08	-3.56E-08	-3.53E-08	-3.46E-08	-3.53E-08	-3.60E-08	-3.79E-08	-3.73E-08	-3.67E-08	-3.88E-08	-4.05E-08	-3.89E-08	-4.02E-08	-4.30E-08	-4.26E-08	-4.51E-08
-3.61E-08	-3.57E-08	-3.67E-08	-3.43E-08	-3.59E-08	-3.32E-08	-3.46E-08	-3.52E-08	-3.59E-08	-3.63E-08	-3.58E-08	-3.55E-08	-3.65E-08	-3.78E-08	-3.85E-08	-3.81E-08	-3.93E-08	-4.05E-08	-3.92E-08	-4.11E-08	-4.30E-08	-4.25E-08	-4.48E-08
-3.52E-08	-3.70E-08	-3.63E-08	-3.40E-08	-3.57E-08	-3.39E-08	-3.40E-08	-3.48E-08	-3.62E-08	-3.75E-08	-3.65E-08	-3.51E-08	-3.71E-08	-3.81E-08	-3.82E-08	-3.73E-08	-3.77E-08	-3.93E-08	-3.97E-08	-4.28E-08	-4.35E-08	-4.23E-08	-4.50E-08
-3.39E-08	-3.72E-08	-3.63E-08	-3.35E-08	-3.63E-08	-3.44E-08	-3.36E-08	-3.48E-08	-3.62E-08	-3.73E-08	-3.72E-08	-3.53E-08	-3.71E-08	-3.87E-08	-3.74E-08	-3.70E-08	-3.77E-08	-3.73E-08	-3.98E-08	-4.37E-08	-4.38E-08	-4.25E-08	-4.52E-08
-3.36E-08	-3.65E-08	-3.61E-08	-3.42E-08	-3.69E-08	-3.46E-08	-3.37E-08	-3.51E-08	-3.66E-08	-3.66E-08	-3.71E-08	-3.60E-08	-3.69E-08	-3.97E-08	-3.79E-08	-3.73E-08	-3.96E-08	-3.78E-08	-4.03E-08	-4.32E-08	-4.37E-08	-4.24E-08	-4.54E-08
-3.43E-08	-3.74E-08	-3.65E-08	-3.49E-08	-3.71E-08	-3.46E-08	-3.51E-08	-3.61E-08	-3.73E-08	-3.70E-08	-3.71E-08	-3.64E-08	-3.79E-08	-4.05E-08	-3.83E-08	-3.80E-08	-4.07E-08	-4.00E-08	-4.14E-08	-4.24E-08	-4.38E-08	-4.24E-08	-4.57E-08
-3.54E-08	-3.93E-08	-3.74E-08	-3.50E-08	-3.75E-08	-3.52E-08	-3.68E-08	-3.76E-08	-3.78E-08	-3.77E-08	-3.77E-08	-3.74E-08	-3.97E-08	-4.07E-08	-3.84E-08	-3.83E-08	-3.97E-08	-4.10E-08	-4.15E-08	-4.25E-08	-4.51E-08	-4.37E-08	-4.68E-08
-3.67E-08	-3.98E-08	-3.79E-08	-3.51E-08	-3.80E-08	-3.61E-08	-3.69E-08	-3.82E-08	-3.88E-08	-3.86E-08	-3.85E-08	-3.82E-08	-4.07E-08	-4.12E-08	-3.93E-08	-3.85E-08	-3.99E-08	-4.07E-08	-4.18E-08	-4.35E-08	-4.54E-08	-4.58E-08	-4.81E-08

2.17E-15	2.64E-15	2.48E-15	2.56E-15	2.41E-15	2.53E-15	2.38E-15	2.55E-15	2.51E-15	2.89E-15	2.86E-15	2.54E-15	2.45E-15	2.61E-15	2.76E-15	2.58E-15	2.05E-15	1.95E-15	2.66E-15	2.80E-15	2.00E-15	5.91E-16	4.34E-16
1.89E-15	2.38E-15	2.40E-15	2.55E-15	2.38E-15	2.40E-15	2.40E-15	2.59E-15	2.55E-15	2.73E-15	2.59E-15	2.51E-15	2.36E-15	2.53E-15	2.76E-15	2.44E-15	1.87E-15	2.06E-15	3.47E-15	3.95E-15	2.71E-15	8.41E-16	4.59E-16
1.75E-15	2.11E-15	2.29E-15	2.47E-15	2.28E-15	2.25E-15	2.24E-15	2.39E-15	2.44E-15	2.52E-15	2.54E-15	2.44E-15	2.38E-15	2.46E-15	2.51E-15	2.32E-15	1.77E-15	2.11E-15	3.67E-15	4.20E-15	2.91E-15	1.04E-15	4.70E-16
1.69E-15	1.83E-15	1.99E-15	2.31E-15	2.23E-15	2.13E-15	2.05E-15	2.26E-15	2.14E-15	2.40E-15	2.57E-15	2.35E-15	2.38E-15	2.39E-15	2.39E-15	2.30E-15	1.81E-15	1.81E-15	2.84E-15	3.22E-15	2.29E-15	8.12E-16	4.28E-16
1.59E-15	1.76E-15	1.68E-15	2.14E-15	2.11E-15	2.05E-15	1.99E-15	2.20E-15	2.04E-15	2.37E-15	2.61E-15	2.26E-15	2.30E-15	2.33E-15	2.39E-15	2.24E-15	1.81E-15	1.42E-15	1.73E-15	1.87E-15	1.37E-15	4.59E-16	3.27E-16
1.51E-15	1.73E-15	1.44E-15	2.08E-15	2.04E-15	2.07E-15	1.95E-15	2.19E-15	2.01E-15	2.39E-15	2.64E-15	2.22E-15	2.21E-15	2.29E-15	2.47E-15	2.20E-15	1.72E-15	1.11E-15	9.40E-16	9.64E-16	6.27E-16	2.66E-16	3.02E-16
1.49E-15	1.66E-15	1.41E-15	2.01E-15	1.95E-15	1.94E-15	1.81E-15	1.98E-15	1.98E-15	2.17E-15	2.36E-15	2.21E-15	2.16E-15	2.31E-15	2.45E-15	2.15E-15	1.62E-15	1.03E-15	6.70E-16	5.69E-16	3.93E-16	2.39E-16	3.58E-16
1.47E-15	1.63E-15	1.38E-15	1.70E-15	1.84E-15	1.84E-15	1.59E-15	1.94E-15	1.93E-15	1.96E-15	2.10E-15	2.19E-15	2.18E-15	2.29E-15	2.23E-15	2.08E-15	1.62E-15	1.01E-15	6.51E-16	5.41E-16	3.87E-16	2.87E-16	4.63E-16
1.52E-15	1.66E-15	1.31E-15	1.50E-15	1.70E-15	1.72E-15	1.49E-15	1.86E-15	1.92E-15	1.86E-15	2.00E-15	2.14E-15	2.15E-15	2.23E-15	2.16E-15	2.06E-15	1.69E-15	9.89E-16	6.25E-16	5.36E-16	4.12E-16	4.05E-16	5.98E-16
1.63E-15	1.75E-15	1.31E-15	1.41E-15	1.57E-15	1.57E-15	1.44E-15	1.67E-15	1.80E-15	1.78E-15	1.91E-15	2.05E-15	2.09E-15	2.17E-15	2.12E-15	2.11E-15	1.68E-15	9.80E-16	6.29E-16	5.43E-16	4.64E-16	5.84E-16	7.67E-16
1.73E-15	1.86E-15	1.43E-15	1.44E-15	1.42E-15	1.35E-15	1.27E-15	1.42E-15	1.57E-15	1.62E-15	1.79E-15	1.91E-15	1.97E-15	2.09E-15	2.02E-15	2.12E-15	1.62E-15	9.75E-16	6.74E-16	6.21E-16	5.45E-16	7.06E-16	9.88E-16
1.83E-15	2.11E-15	1.54E-15	1.40E-15	1.32E-15	1.23E-15	1.08E-15	1.25E-15	1.34E-15	1.43E-15	1.59E-15	1.82E-15	1.83E-15	2.07E-15	1.93E-15	2.06E-15	1.58E-15	1.11E-15	8.38E-16	8.08E-16	7.76E-16	9.75E-16	1.49E-15
2.02E-15	2.59E-15	1.58E-15	1.28E-15	1.18E-15	1.16E-15	9.47E-16	1.11E-15	1.18E-15	1.25E-15	1.39E-15	1.59E-15	1.68E-15	2.00E-15	1.77E-15	1.97E-15	1.72E-15	1.41E-15	1.25E-15	1.31E-15	1.54E-15	1.95E-15	3.16E-15
2.21E-15	2.96E-15	1.58E-15	1.18E-15	1.14E-15	1.06E-15	9.12E-16	9.88E-16	1.08E-15	1.14E-15	1.23E-15	1.30E-15	1.49E-15	1.85E-15	1.68E-15	1.83E-15	1.95E-15	1.86E-15	2.23E-15	2.63E-15	3.51E-15	4.84E-15	7.66E-15
1.98E-15	2.35E-15	1.38E-15	1.11E-15	1.06E-15	1.06E-15	9.39E-16	8.04E-16	9.55E-16	1.12E-15	1.17E-15	1.14E-15	1.24E-15	1.53E-15	1.55E-15	1.77E-15	2.40E-15	3.06E-15	4.65E-15	5.87E-15	7.93E-15	1.10E-14	1.54E-14
[truncated: 1,057,237 more chars]
