# Supplementary material for: Delayed delivery of antibiotics by ultrasound-mediated rupture of polylactic acid pockets: In vitro and in vivo studies
Source: PLoS One. 2025 Dec 11;20(12):e0337717. doi: 10.1371/journal.pone.0337717 (PMC12698016; doi:10.1371/journal.pone.0337717)
Supplement: Attachment — Submitted filename: Response to reviewers.docx [file pone.0337717.s004.docx]

August 12, 2025

Dola Sundeep

Academic Editor

PLOS ONE

**Re: Delayed delivery of antibiotics by ultrasound-mediated rupture of polylactic acid pockets: in vitro and in vivo studies; Manuscript no: PONE-D-25-14645**

Dear Dr. Sundeep

Thank you for your email of April 28^th^, 2025 and for the reviewer’s insightful comments on our manuscript referenced above. Our responses to the questions raised can be found below. All line references refer to the revised and marked version of the manuscript. Finally, we discovered a few trivial typographical and linguistic errors in the original manuscript and those have also been corrected.

Journal requirements:

When submitting your revision, we need you to address these additional requirements.

1.  Please ensure that your manuscript meets PLOS ONE's style requirements, including those for file naming. The PLOS ONE style templates can be found at

[https://journals.plos.org/plosone/s/file?id=wjVg/PLOSOne_formatting_sample_main_body.pdf](https://nam12.safelinks.protection.outlook.com/?url=https%3A%2F%2Ftrack.editorialmanager.com%2FCL0%2Fhttps%3A%252F%252Fjournals.plos.org%252Fplosone%252Fs%252Ffile%253Fid%3DwjVg%252FPLOSOne_formatting_sample_main_body.pdf%2F1%2F010f01967bb2b3c1-471e11e3-4d64-404b-bc9d-028014f9508a-000000%2FzFJaBvaTh6wePR-9n0bnJrB5DJU5fTX0Rrgog2esoLc%3D208&data=05%7C02%7CSelin.Isguven%40students.jefferson.edu%7Cf73d912cc85b48eca8ca08dd96fad314%7Ced4cf9769b924010a0b7a7222ba6cdef%7C0%7C0%7C638832726978219171%7CUnknown%7CTWFpbGZsb3d8eyJFbXB0eU1hcGkiOnRydWUsIlYiOiIwLjAuMDAwMCIsIlAiOiJXaW4zMiIsIkFOIjoiTWFpbCIsIldUIjoyfQ%3D%3D%7C0%7C%7C%7C&sdata=tGJNsfVYeWmdAqk2g5n6TaJu1Y607g8RM4pyaifOnQM%3D&reserved=0)  and

[https://journals.plos.org/plosone/s/file?id=ba62/PLOSOne_formatting_sample_title_authors_affiliations.pdf](https://nam12.safelinks.protection.outlook.com/?url=https%3A%2F%2Ftrack.editorialmanager.com%2FCL0%2Fhttps%3A%252F%252Fjournals.plos.org%252Fplosone%252Fs%252Ffile%253Fid%3Dba62%252FPLOSOne_formatting_sample_title_authors_affiliations.pdf%2F1%2F010f01967bb2b3c1-471e11e3-4d64-404b-bc9d-028014f9508a-000000%2FTKhc16XqjmmoG-3s8prdIxMZUGvTOOCbSgyNlw0aBD8%3D208&data=05%7C02%7CSelin.Isguven%40students.jefferson.edu%7Cf73d912cc85b48eca8ca08dd96fad314%7Ced4cf9769b924010a0b7a7222ba6cdef%7C0%7C0%7C638832726978234278%7CUnknown%7CTWFpbGZsb3d8eyJFbXB0eU1hcGkiOnRydWUsIlYiOiIwLjAuMDAwMCIsIlAiOiJXaW4zMiIsIkFOIjoiTWFpbCIsIldUIjoyfQ%3D%3D%7C0%7C%7C%7C&sdata=OlvuDTh0BVWRtc9sxKpq2M%2FaVUGAASkWbGPnZHazaCI%3D&reserved=0)

We have carefully gone through the entire manuscript and made sure it conforms to the journal guidelines.

2. To comply with PLOS ONE submissions requirements, in your Methods section, please provide additional information regarding the experiments involving animals and ensure you have included details on (1) methods of sacrifice, (2) methods of anesthesia and/or analgesia, and (3) efforts to alleviate suffering.

As requested we have expanded the information regarding our animal experiments, which now reads (in part):

“*Animal experiments were performed according to a protocol (Protocol Number: 22-11-606) approved by the Institutional Animal Care & Use Committee (IACUC) of Thomas Jefferson University and in accordance with the National Research Council's “Guide for the Care and Use of Laboratory Animals.” All surgery was performed under 1-4% isoflurane anesthesia, and all efforts were made to minimize suffering.*

*Rabbits were pre-medicated with ketamine 30-40 mg/kg, xylazine 2-5 mg/kg, and acepromazine 0.25-1.00 mg/kg. Anesthesia was induced with 4-5% isoflurane and maintained with 1-4% isoflurane during the entire procedure. ...*

*Following implantation, the rabbits were allowed unrestricted ambulation and observed for activity and recovery. Wounds were inspected daily for drainage, erythema, warmth, and swelling, as well as MeB release … for a total of 6 days, at which time all animals were euthanized with a barbiturate overdose in accordance with AVMA recommendations*.” (lines 247 - 268)

3. Thank you for stating the following financial disclosure:

“NIH R01 AR069119

The Mullin Fund for Spinal Research at Thomas Jefferson University”

Please state what role the funders took in the study.  If the funders had no role, please state: "The funders had no role in study design, data collection and analysis, decision to publish, or preparation of the manuscript."

If this statement is not correct you must amend it as needed.

Please include this amended Role of Funder statement in your cover letter; we will change the online submission form on your behalf.

Thank you for your guidance. The cover letter now includes the statement:

“*NIH R01 AR069119*

*The Mullin Fund for Spinal Research at Thomas Jefferson University*

*The funders had no role in study design, data collection and analysis, decision to publish, or preparation of the manuscript*.”

4. Thank you for stating the following in the Acknowledgments Section of your manuscript:

“We thank Hebah Falatah for Definity droplet preparation and Catherine Gurr for help with data presentation. Research reported in this manuscript was supported by the National Institute of Arthritis and Musculoskeletal Diseases of the National Institutes of Health under award number R01AR069119 (Hickok, Forsberg, Isguven) and by the Mullin Fund for Spinal Research at Thomas Jefferson University. The content is solely the responsibility of the authors and does not necessarily represent the official views of the National Institutes of Health. “

We note that you have provided funding information that is currently declared in your Funding Statement. However, funding information should not appear in the Acknowledgments section or other areas of your manuscript. We will only publish funding information present in the Funding Statement section of the online submission form.

Please remove any funding-related text from the manuscript and let us know how you would like to update your Funding Statement. Currently, your Funding Statement reads as follows:

“NIH R01 AR069119

The Mullin Fund for Spinal Research at Thomas Jefferson University”

Please include your amended statements within your cover letter; we will change the online submission form on your behalf.

Thank you for your guidance. The cover letter now includes the statement:

“*NIH R01 AR069119*

*The Mullin Fund for Spinal Research at Thomas Jefferson University*

*The funders had no role in study design, data collection and analysis, decision to publish, or preparation of the manuscript*.”

Moreover, we have removed the funding information from the Acknowledgement section, which now reads”

“*We thank Hebah Falatah for Definity droplet preparation and Catherine Gurr for help with data presentation*.” (lines 561 - 562)

5. Thank you for stating the following in the Competing Interests section:

“I have read the journal's policy and the authors of this manuscript have the following competing interests: Selin Isguven Billmyer, Noreen Hickok, Flemming Forsberg reports financial support was provided by National Institutes of Health. Flemming Forsberg reports a relationship with GE HealthCare that includes: consulting or advisory. Flemming Forsberg reports a relationship with Lantheus Medical Imaging Inc that includes: consulting or advisory. Selin Isguven Billmyert, Noreen Hickok, Flemming Forsberg has patent PCT Application No. PCT/US2023/077854 pending to Thomas Jefferson University. The other authors, declare that they have no known competing financial interests or personal relationships that could have appeared to influence the work reported in this paper.”

Please confirm that this does not alter your adherence to all PLOS ONE policies on sharing data and materials, by including the following statement: "This does not alter our adherence to  PLOSONE policies on sharing data and materials.” (as detailed online in our guide for authors [http://journals.plos.org/plosone/s/competing-interests](https://nam12.safelinks.protection.outlook.com/?url=http%3A%2F%2Fjournals.plos.org%2Fplosone%2Fs%2Fcompeting-interests&data=05%7C02%7CSelin.Isguven%40students.jefferson.edu%7Cf73d912cc85b48eca8ca08dd96fad314%7Ced4cf9769b924010a0b7a7222ba6cdef%7C0%7C0%7C638832726978249148%7CUnknown%7CTWFpbGZsb3d8eyJFbXB0eU1hcGkiOnRydWUsIlYiOiIwLjAuMDAwMCIsIlAiOiJXaW4zMiIsIkFOIjoiTWFpbCIsIldUIjoyfQ%3D%3D%7C0%7C%7C%7C&sdata=kx2OwBG0kbax0bOovxyh5epEY1ziTJX3cgEJwXvN0Io%3D&reserved=0)).  If there are restrictions on sharing of data and/or materials, please state these. Please note that we cannot proceed with consideration of your article until this information has been declared.

Please include your updated Competing Interests statement in your cover letter; we will change the online submission form on your behalf.

Thank you for your guidance. The cover letter now includes the statement:

“*I have read the journal's policy and the authors of this manuscript have the following competing interests: Selin Isguven Billmyer, Noreen Hickok, Flemming Forsberg report financial support was provided by National Institutes of Health. Flemming Forsberg reports a relationship with GE HealthCare that includes: consulting or advisory. Flemming Forsberg reports a relationship with Lantheus Medical Imaging Inc that includes: consulting or advisory. Selin Isguven Billmyer, Noreen Hickok, Flemming Forsberg have patent PCT Application No. PCT/US2023/077854 pending to Thomas Jefferson University. The other authors declare that they have no known competing financial interests or personal relationships that could have appeared to influence the work reported in this paper. This does not alter our adherence to PLOS One policies on sharing data and materials.”*

6. In this instance it seems there may be acceptable restrictions in place that prevent the public sharing of your minimal data. However, in line with our goal of ensuring long-term data availability to all interested researchers, PLOS’ Data Policy states that authors cannot be the sole named individuals responsible for ensuring data access ([http://journals.plos.org/plosone/s/data-availability#loc-acceptable-data-sharing-methods](https://nam12.safelinks.protection.outlook.com/?url=http%3A%2F%2Fjournals.plos.org%2Fplosone%2Fs%2Fdata-availability%23loc-acceptable-data-sharing-methods&data=05%7C02%7CSelin.Isguven%40students.jefferson.edu%7Cf73d912cc85b48eca8ca08dd96fad314%7Ced4cf9769b924010a0b7a7222ba6cdef%7C0%7C0%7C638832726978263016%7CUnknown%7CTWFpbGZsb3d8eyJFbXB0eU1hcGkiOnRydWUsIlYiOiIwLjAuMDAwMCIsIlAiOiJXaW4zMiIsIkFOIjoiTWFpbCIsIldUIjoyfQ%3D%3D%7C0%7C%7C%7C&sdata=3a4Zegd1hNoCQtggKjs7SJMqx82bmsV6zrQryNY1A4E%3D&reserved=0)).

Data requests to a non-author institutional point of contact, such as a data access or ethics committee, helps guarantee long term stability and availability of data. Providing interested researchers with a durable point of contact ensures data will be accessible even if an author changes email addresses, institutions, or becomes unavailable to answer requests.

Before we proceed with your manuscript, please also provide non-author contact information (phone/email/hyperlink) for a data access committee, ethics committee, or other institutional body to which data requests may be sent. If no institutional body is available to respond to requests for your minimal data, please consider if there any institutional representatives who did not collaborate in the study, and are not listed as authors on the manuscript, who would be able to hold the data and respond to external requests for data access? If so, please provide their contact information (i.e., email address). Please also provide details on how you will ensure persistent or long-term data storage and availability.

Our institution does not have a data access committee or anything similar. We will provide a complete copy of all the data to Dr. John Eisenbrey (Professor of Radiology) who will maintain long term availability of the data from this study and who will handle any requests regarding access. His email is: [john.eisenbrey@jefferson.edu](mailto:john.eisenbrey@jefferson.edu)

7. Please review your reference list to ensure that it is complete and correct. If you have cited papers that have been retracted, please include the rationale for doing so in the manuscript text, or remove these references and replace them with relevant current references. Any changes to the reference list should be mentioned in the rebuttal letter that accompanies your revised manuscript. If you need to cite a retracted article, indicate the article’s retracted status in the References list and also include a citation and full reference for the retraction notice.

We are not aware of any retractions among our reference list have updated the list to reflect the PLOS One style, as requested. Moreover, we replaced the abstract cited as (26) with the peer-reviewed paper of this study. Finally, three new references were added to comply with the expanded discussion requested by the reviewers (32, 33, 50).

Additional Editor Comments:

Subject: Decision on Your Manuscript PONE-D-25-14645

Dear Dr. Forsberg,

We have now received the required number of reviewer reports for your manuscript titled "Delayed delivery of antibiotics by ultrasound-mediated rupture of polylactic acid pockets: in vitro and in vivo studies" (Manuscript Number: PONE-D-25-14645).

Based on the reviewers' comments, the decision on your manuscript is Minor Revision.

We look forward to receiving your revised submission.

Thank you for your continued interest in our journal.

Best regards,
Dr. Dola Sundeep

Reviewers' comments:

Reviewer's Responses to Questions

**Comments to the Author**

1. Is the manuscript technically sound, and do the data support the conclusions?

The manuscript must describe a technically sound piece of scientific research with data that supports the conclusions. Experiments must have been conducted rigorously, with appropriate controls, replication, and sample sizes. The conclusions must be drawn appropriately based on the data presented.

Reviewer #1: Yes

Reviewer #2: Yes

2. Has the statistical analysis been performed appropriately and rigorously?

Reviewer #1: Yes

Reviewer #2: Yes

3. Have the authors made all data underlying the findings in their manuscript fully available?

The [PLOS Data policy](https://nam12.safelinks.protection.outlook.com/?url=http%3A%2F%2Ftrack.editorialmanager.com%2FCL0%2Fhttp%3A%252F%252Fwww.plosone.org%252Fstatic%252Fpolicies.action%2523sharing%2F1%2F010f01967bb2b3c1-471e11e3-4d64-404b-bc9d-028014f9508a-000000%2FSR6cDI_WWA7cnrjRrBN7yEgAXyAmPCd88j33hIqu2Qw%3D208&data=05%7C02%7CSelin.Isguven%40students.jefferson.edu%7Cf73d912cc85b48eca8ca08dd96fad314%7Ced4cf9769b924010a0b7a7222ba6cdef%7C0%7C0%7C638832726978276547%7CUnknown%7CTWFpbGZsb3d8eyJFbXB0eU1hcGkiOnRydWUsIlYiOiIwLjAuMDAwMCIsIlAiOiJXaW4zMiIsIkFOIjoiTWFpbCIsIldUIjoyfQ%3D%3D%7C0%7C%7C%7C&sdata=kH8bCx2AGOaogK4tRo0iEo8xW9%2BXPJXamiy7AYYkWVc%3D&reserved=0) requires authors to make all data underlying the findings described in their manuscript fully available without restriction, with rare exception (please refer to the Data Availability Statement in the manuscript PDF file). The data should be provided as part of the manuscript or its supporting information, or deposited to a public repository. For example, in addition to summary statistics, the data points behind means, medians and variance measures should be available. If there are restrictions on publicly sharing data—e.g. participant privacy or use of data from a third party—those must be specified.

Reviewer #1: Yes

Reviewer #2: Yes

4. Is the manuscript presented in an intelligible fashion and written in standard English?

PLOS ONE does not copyedit accepted manuscripts, so the language in submitted articles must be clear, correct, and unambiguous. Any typographical or grammatical errors should be corrected at revision, so please note any specific errors here.

Reviewer #1: Yes

Reviewer #2: Yes

5. Review Comments to the Author

Please use the space provided to explain your answers to the questions above. You may also include additional comments for the author, including concerns about dual publication, research ethics, or publication ethics. (Please upload your review as an attachment if it exceeds 20,000 characters)

Reviewer #1: This study introduces a promising and innovative ultrasound-triggered antibiotic delivery system for spinal surgery infections. The approach is highly relevant and potentially impactful, offering a non-invasive method for localized infection control. However, to strengthen the manuscript, several areas need improvement:

Statistical Analysis – Key outcomes like rupture rates and antibiotic release should include significance testing.

We tested the rupture rates for neat PLA and PLA-VAN pockets and found no statistically significant difference (p > 0.99). We also clarified the issue with the one excluded pocket and the text now reads:

“*Importantly, all 5 of the intact pockets (3 neat PLA and 2 PLA-VAN) that were allocated for insonation ruptured following US (100%; p > 0.99). For the neat PLA pockets, 5 out of 9 were intact on day 3, while 4 out of 6 PLA-VAN pockets survived until day 3 (p > 0.99). An additional pocket from Rabbit #5 was excluded from analysis, due to the emptying of the pocket contents occurring without staining, making it impossible to determine the timeline of leakage*.” (lines 433 - 438).

The antibiotic release rates were converted to % (as requested by Reviewer 2) and testing showed no increase by day 15 (p = 0.19) but after 27 days the release was statistically significantly higher (p = 0.0436). Both p-values have now been added to the text, which was rewritten to:

” *For a first trial, the percent VAN release from the film in the first day and up to 15 days (Fig. 11-A) are shown. Total release values are close to 2.5 mg or 2,500 µg (n = 3; p = 0.19).*

*Despite the variability in each sample, superficial VAN dissolved in the first couple of hours, in keeping with many passive elution systems. We next investigated if VAN release was still present at 27 days. Again, we measured immediate VAN release expressed as percent of the total loading (n = 3) on the first day and up to 27 days, which was statistically significantly higher (p = 0.0436; Fig. 11-B). Most of the VAN dissolved early in the incubation period, although some additional release was measured after the first day, which we speculate is due to some PLA degradation which releases additional VAN*.“ (lines 403 - 416).

Long-Term Efficacy – Additional data on stability and prolonged effectiveness are needed.

The premise of our local drug delivery system is that all pockets must be activated within 6 days to reduce the possibility of bacterial infections taking hold (i.e., for prophylaxis). There is therefore no need for long-term efficacy studies, and we have not made any changes to the manuscript in this regard.

Comparative Controls – Comparing with other delivery systems (e.g., beads, hydrogels) would add context.

While we do agree with the reviewer, we feel that such experiments are outside the scope of this manuscript. We do however allude to hydrogels in the revised Discussion section. Specifically:

“*Similar to hydrogels, PLA constructs can be fine-tuned for the needs of drug delivery applications with alterations in mechanical strength and degradation kinetics (33).*” (lines 473 - 475).

Tissue Compatibility – More information on inflammatory responses or biocompatibility is recommended.

We have expanded the Discussion markedly to cover these aspects (including 2 new references (32, 33)) and the text now states:

“*To achieve triggered release, we focused on PLA as it is the leading biocompatible and biodegradable polymer in clinical application design, including orthopaedics (31–35). Examples include biodegradable sutures, orthopaedic biodegradable screws and plates, and after spinal surgeries including laminectomy (31,32). Similar to hydrogels, PLA constructs can be fine-tuned for the needs of drug delivery applications with alterations in mechanical strength and degradation kinetics (33). Upon degradation into smaller monomers and oligomers, PLA is assumed to be excreted from the body via urination or exhalation. As such, additional surgeries to remove the PLA device are not necessary, improving patient recovery and optimizing health system costs (31,32).*” (lines 469 - 478).

Ultrasound Parameters – Clearer details (e.g., frequency, intensity, duration) would aid reproducibility.

We have expanded our description of the ultrasound parameters used in these experiments (both in vitro and in vivo). The revised text now reads:

“*For clinical US, an S50 scanner (SonoScape, Shenzhen, China) with a curvilinear C1-6 probe was used for 20 min of Power Doppler imaging (2.2 MHz, highest line density, 100% power or 703.6 kPa peak to peak pressure) followed by 10 min of flash replenishment imaging (3.0 MHz harmonic imaging at 100% power or 1.08 MPa peak to peak pressure every 4 seconds). These parameters were measured with a calibrated 0.5 mm needle hydrophone (Precision Acoustics, Dorchester, UK) or selected based on prior experiments (11). HIFU involved 20 min of insonation (2.0 MHz at 4 V equivalent to 5.5 MPa peak negative pressure with 50% duty cycle) using an SU-101 probe (Sonic Concepts, Bothell, WA, USA) run by an 8116A pulse generator (Hewlett Packard, Palo Alto, CA. USA) with 50 dB amplification*.“ (lines 202 - 211)

and

*“…their pockets insonified for 30 minutes in Power Doppler imaging mode using an S50 clinical US scanner with a curvilinear C1-6 probe and the same settings as the in vitro studies*.” (lines 264 - 266).

Limitations & Future Work – The authors should discuss the small sample size and outline next steps.

As requested, we have expanded our paragraph on limitations and future directions (including a new reference (50)). The text now reads:

“*There were limitations to our studies, where additional experiments could discern differences in outcomes with regards to type of US or cavitation nuclei, and allow us to better characterize drug release profile.* *Another limitation to this study was the small sample size for the in vivo evaluations. In accordance with the 3 Rs of responsible in vivo research, we performed this pilot study using rabbits based on the results of our previous studies (26). Most importantly, the stability and drug release of the in vitro experiments did not reflect the outcome achieved in the in vivo experiments. These differences could be due to the insertion environment (proteins, ions, pH), or the mechanical constraint at the insertion site* *combined with the pocket design. Future iterations of pocket designs would benefit from earlier and larger in vivo studies, where the design parameters could be adjusted* *(e.g., not cone shaped and/or altering the hydrophilicity of the thin film PLA by replacing it with hydrophobic PLGA) according to in vivo outcomes.* *Finally, a crucial next step will be to evaluate the efficacy of this drug delivery concept against common bacteria such as Staphylococcus aureus* *similar to our recent work (50).*” (lines 536 - 548)

Overall, this is a well-designed, novel study with strong potential, but a few enhancements would improve its clarity, reproducibility, and clinical applicability.

Reviewer #2: PONE-D-25-14645 Review

I would recommend publishing however, authors should explain the points below and consider the following revisions.

1) Authors should explain why the cone shaped geometry was selected for this application.

The PLA films were folded into a cone for ease of assembly and this is now stated explicitly in the revised manuscript (line 113). We also included alternative pocket design as a next step in this project (given the *in vivo* results) and this is mentioned in the Discussion section:

“*the design parameters could be adjusted (e.g., not cone shaped…*” (lines 544 - 545)

2) Authors should consider and report MeB and VAN release data as % release / surface area of the pocket

We agree with this suggestion. The MeB release rates are already in % (cf., Fig 10) and we have now converted the VAN release data to % as well and replotted Fig 11 (please note these two figures were numbered 9 and 10 in the original submission). For your convenience the new figure is included below.

3) How many pockets will be needed to deliver the typical bolus dose of VAN post-surgery ?

Surgeons typically place 1-2 g of VAN (or another antibiotic) in the spine wound site, but site volumes vary (maximum about 5 mL) and with drains very often placed the actual VAN concentration is difficult to estimate. However, our pockets can contain up to 4.5 mL, and 1 g of VAN is soluble in 3.0 mL of water, which means that a single pocket can replicate the initial VAN dosage. The wide range of concentrations possible in one pocket emphasizes the ability of our device to be tuned to the requirements of the individual patient. We have rewritten the Discussion to include the following:

“*PLA films were used to construct a drug-loaded (in these experiments VAN) pocket containing up to 4.5 mL. Since 1 g of VAN is soluble in 3.0 mL of water a single pocket can replicate a wide range of concentrations including that of the initial intraoperative antibiotics. The ability of our device to be tuned to the requirements of the individual patient is an important feature.*” (lines 453 - 457)

***Fig. 11:*** *Relative VAN release from VAN-embedded 0.50 g PLA film pieces; Trial 1, high concentration VAN (A), and Trial 2, low concentration VAN (B), over time.*

4) Have the authors altering the hydrophilicity of PLA , by replacing it with hydrophobic PLGA in the thin film ?

We have not tried this, but it is now mentioned in the Discussion section as a possible next step. See response to Reviewer 1 above (and lines 545 - 546).

5) Detailed surface characterization using AFM and visualization using SEM, might help explain the difference between invitro and invivo disconnect that the authors observed.

Thank you for that suggestion. We have now conducted an extensive AFM investigation and added two new sub-sections (one each to Methods and Results) as well as a new Fig. 8 with representative results (all subsequent figures have been re-numbered). The Methods sub-section reads:

“***Atomic Force Microscopy (AFM) Analysis of PLA Films***

*To further evaluate film characteristics, we performed atomic force microscopy (AFM) of neat PLA and VAN-embedded PLA films to observe surface topography and visualize VAN inclusions. Film samples were prepared by* *excising 10 x 3 cm sections from a 19 cm diameter sheet and imaged using an MFP-3D Infinity BIO AFM integrated with a Nikon inverted fluorescence microscope (Asylum Research, Santa Barbara, CA, USA). Images were acquired in AC mode using a triangular gold coated silicon nitride pyramid tip probe (15 nm tip radius, force constant 0.32 N/m, PNP-TR-Au cantilever 1, Asylum Research) over a 20 x 20 μm area with 256 lines/256 points at a scan rate of 0.95 Hz. All images obtained were processed using AR software (Igor Pro, Wavemetrics, Portland, OR, USA) integrated with the MFP-3D-BIO AFM. Root mean square (RMS) roughness was quantified using four 10 x 10 μm area regions within each scan from the Z-sensor (height) channel after flattening.*” (lines 164 - 175)

while the Results now include:

“***PLA-VAN films have increased roughness***

*The AFM neat PLA micrographs showed local irregularities due to miniscule air pockets and dust particles (Fig. 8-A). These inclusions were apparent on the 3D maps although their effect on overall smoothness was small. The PLA-VAN film (Fig. 8-B) showed an overall smooth surface interrupted by inclusions of various sizes and distribution. In the largest inclusions, granulated material consistent with the presence of VAN were observed. The topographic map of the PLA-VAN was irregular where the frequency of the deviations from the surface increased suggesting a film that was affected by the VAN.* *The surface topography of the neat PLA film had an RMS roughness of 16.00 ± 5.75 nm, while the PLA-VAN film had an RMS roughness of 38.24 ± 12.77 nm (p = 0.0192). These data suggest that rupture can be achieved more easily with VAN-PLA films, which is beneficial for drug release. Additionally, this finding is consistent with data from the previously discussed mechanical testing.*” (lines 353 - 364)

The new figure has been included below for your convenience,

Finally, given the work involved in the AFM studies we have added two new co-authors (A. H. Harris and E. McLaughlin). All the original authors agree with this.

***Fig. 8****: Amplitude retrace topography images and 3D surface mapping of PLA films captured at a 20 x 20 µm area ScanSize. (A) Surface characteristics of neat PLA film exhibiting consistent, smooth surface topography with minimal irregularities due to air pockets or dust particles. (B) PLA-VAN film with topographic map revealing distinct VAN granulations consistently across the film’s surface with varying concentration and distribution.*
